# Supplementary material for: High resolution DNA barcode library for European butterflies reveals continental patterns of mitochondrial genetic diversity
Source: Commun Biol. 2021 Mar 9;4:315. doi: 10.1038/s42003-021-01834-7 (PMC7943782; doi:10.1038/s42003-021-01834-7)
Supplement: Supplementary file 2 — Supplementary Information [file 42003_2021_1834_MOESM2_ESM.pdf]

COMMUNICATIONS BIOLOGY

SUPPLEMENTARY INFORMATION

High resolution DNA barcode library for European butterflies reveals continental patterns of mitochondrial genetic diversity

Vlad Dincă, Leonardo Dapporto, Panu Somervuo, Raluca Vodă, Sylvain Cuvelier, Martin Gascoigne-Pees, Peter Huemer, Marko Mutanen, Paul D. N. Hebert, Roger Vila

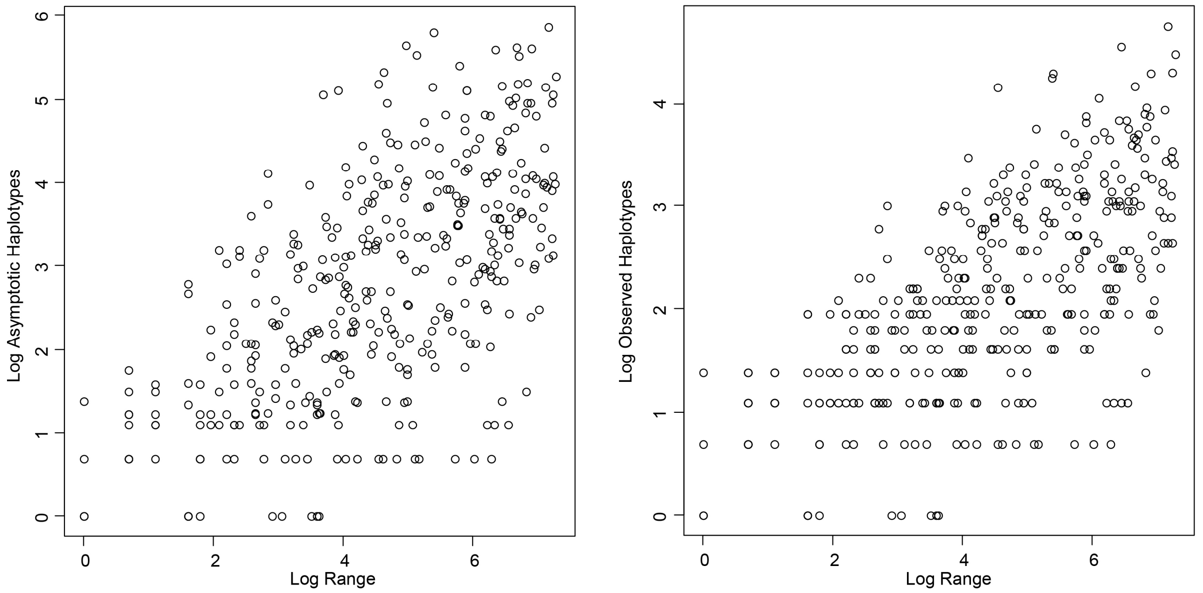

Supplementary Fig. 1 Relationship between the number of *COI* haplotypes (left - estimated, right - observed) and species range size (n = 385 species).

Supplementary Methods

Specimens identified to genus level only

Although many species of European butterflies can be readily identified based on external and/or internal morphology, a number of species are extremely difficult to determine using this methodology. This can be the case in both sexes, but is mostly confined to one gender. Sometimes, such instances involve species with a questionable taxonomic status where further research is required. Therefore, 232 specimens in our dataset (Supplementary Data 1) were identified only to genus level.

The neighbor-joining tree provided in Supplementary Data 14 facilitates the visual inspection of *COI* patterns detected within European butterflies.

Colias (family Pieridae): Nine specimens were not assigned to a particular species because they are suspected to represent hybrids.

Pieris (family Pieridae): Twenty-five specimens with the morphology of *Pieris napi* or *Pieris balcana* were not assigned to either of the two taxa. The taxonomic status, relationship and distribution of *balcana*, with respect to *napi*, are not fully understood and require further studies. Because putative morphological differences are extremely subtle (e.g.[1,2]), we did not assign any of the specimens sampled from the Balkans to any one species, because the distribution of *balcana* supposedly encompasses parts of this region.

Lysandra (family Lycaenidae): Three specimens were not assigned to a particular species because they are suspected to represent hybrids.

Polyommatus (subgenus *Agrodiaetus*) (family Lycaenidae): Several European taxa of the subgenus *Agrodiaetus* of the genus *Polyommatus* have extremely similar morphology and their taxonomy is often based on karyotypes. The situation is particularly complex in the Balkans, where the distribution and status of several morphologically similar taxa may require further research. For these reasons, in our dataset we only designated to species level specimens included in the most recent revision of Balkan *Agrodiaetus*[3].

Boloria (family Nymphalidae): Seven specimens with the morphology of *Boloria pales* or *Boloria napaea* formed a clade differentiated from all other specimens of *B. pales* and *B. napaea* available. Given that these two species are very difficult to identify, especially in the

case of males, we preferred not to name the seven specimens until further research is undertaken.

Hipparchia (family Nymphalidae): Sixty-one specimens were not assigned to a particular species. Some of the European *Hipparchia* are virtually indistinguishable based on wing morphology and sometimes display only subtle differences in genitalia[4]. The taxonomic status of some taxa is also not fully elucidated (e.g.[5,6]).

To name specimens present in our dataset, we relied on genitalia examination and/or distribution patterns, the latter criterion being only applied in the case of allopatric species. As a result, 61 specimens, mostly from areas where several taxa could theoretically occur in sympatry and for which genitalia were not examined (or yielded inconclusive results), were named only to genus level.

Melitaea (family Nymphalidae): Seventy-four specimens with the morphology of *Melitaea phoebe* or *Melitaea ornata* were not identified to species level. *Melitaea phoebe* and *M. ornata* are virtually indistinguishable based on external morphology of the adults and male genitalia structures show only subtle differences[7,8]. It has been shown that eastern European specimens (the Balkans) of *M. ornata* can be reliably separated based on COI[9]. However, the distribution of *M. ornata* may extend further into western Europe[9,10] and it appears that only nuclear markers can reliably distinguish such specimens. Consequently, specimens with external morphology of *M. phoebe* or *M. ornata* originating from areas in central and western Europe (Supplementary Data 1) where *M. ornata* could potentially occur, were not assigned to either of the two species, given the lack of nuclear DNA.

The two species appear to be separable based on larval morphology ([9] and references therein) and we identified a few such barcoded specimens to species level because they have been bred, even if they originated from outside the Balkans.

Pyrgus (family Hesperiidae): Five specimens with the external morphology of *Pyrgus alveus* or *Pyrgus foulquieri* were not assigned to either of the two taxa. Both taxa have extremely similar external morphology, but differences in the genitalia and in the number of chromosomes have been documented[11]. Five specimens have not been identified to species level because the genitalia were not available for examination and they originate from areas where both taxa could theoretically occur (Supplementary Data 1).

Analyses of DNA sequences

For the dataset, we retained only *COI* sequences of at least 600 bp.

Most DNA barcodes used in our dataset were generated at the Biodiversity Institute of Ontario, Canada following standard protocols for Lepidoptera[12]. A 658-bp fragment of *COI* was targeted for amplification using the primers LepF1 (5′-ATTCAACCAATCATAAAGATATTGG-3′) and LepR1 (5′-TAAACTTCTGGATGTCCAAAAAATCA-3′). Samples that did not produce a PCR product with the primers LepF1 and LepR1 were amplified with the primers LepF1 and Enh_LepR1 (5′-CTCCWCCAGCAGGATCAAAA-3′), which amplify a 609-bp fragment of *COI*.

A number of sequences were generated in the Butterfly Diversity and Evolution Lab at the Institute of Evolutionary Biology (CSIC-UPF), Barcelona, Spain. In this case, total genomic DNA was extracted using Chelex 100 resin, 100–200 mesh, sodium form (Biorad), under the following protocol: one leg was removed and introduced into 100 µl of Chelex 10% and 5 µl of Proteinase K (20 mg/ml) were added. The samples were incubated overnight at 55ºC and were subsequently incubated at 100ºC for 15 minutes. Samples were then centrifuged for 10 s at 3000 rpm. A 658-bp fragment near the 5′ end of COI was amplified by polymerase chain reaction using the primers LepF1 and LepR1. Double-stranded DNA was amplified in 25-µL volume reactions containing: 14.4 µl autoclaved Milli-Q water, 5 µl 5x buffer, 2 µl 25 mM $MgCl_2$, 0.5 µl 10 mM dNTPs, 0.5 µl of each primer (10 µM), 0.1 µl Taq DNA Polymerase (Promega, 5U/ µl) and 2 µl of extracted DNA. The typical thermal cycling profile followed this protocol: first denaturation at 92ºC for 60 s, followed by five cycles of 92ºC for 15 s, 48ºC for 45 s and 62ºC for 150 s, and then by 35 cycles of 92ºC for 15 s, 52ºC for 45 s and 62ºC for 150 s and a final extension at 62ºC for 420 s. PCR products were purified and sequenced by Macrogen Inc.

To facilitate the visualisation of genetic distances, a neighbour-joining tree of the 22,306 *COI* sequences was built using BOLD, based on uncorrected p distances[13,14]. Other distance-based analyses (e.g. distances to nearest neighbour) were also run using BOLD.

All sequences used in this study have been submitted to GenBank and, together with associated information, are publicly available in the dataset DS-EUGENMAP on BOLD at www.barcodinglife.org.

Supplementary references

1. Lafranchis, T. *Butterflies of Europe*. (Diatheo, 2004).
2. Tshikolovets, V.V. *Butterflies of Europe and the Mediterranean area.* (Tshikolovets Publications, 2011).
3. Vishnevskaya M.S., Saifitdinova A.F. & Lukhtanov A.V. Karyosystematics and molecular taxonomy of the anomalous blue butterflies (Lepidoptera, Lycaenidae) from the Balkan Peninsula. *Comparative Cytogenetics* **10,** 1–85. doi: 10.3897/CompCytogen.v10i5.10944 (2016).
4. Kudrna, O. *A revision of the genus* Hipparchia *Fabricius*. (E. W. Classey Ltd., 1977).
5. Wakeham-Dawson, A., Coutsis, J. G., Dennis, R. L. H. & Holloway, J. D. Observations on the integrity of Balkan *Parahipparchia* Kudrna, 1977 (Lepidoptera: Nymphalidae, Satyrinae) taxa based on male genitalia. *Entomologist's Gazette* **54**, 71–103 (2003).
6. Wakeham-Dawson, A., Jakšić, P., Holloway, J. D. & Dennis, R. Multivariate analysis of male genital structures in the *Hipparchia semele-muelleri-delattini* complex (Nymphalidae, Satyrinae) from the Balkans: how many taxa? *Nota lepid.* **27,** 103–124 (2004).
7. Tóth, J.P. & Varga, Z. Inter- and intraspecific variation in the genitalia of the '*Melitaea phoebe* group' (Lepidoptera, Nymphalidae). *Zoologischer Anzeiger – A Journal of Comparative Zoology* **250,** 258–268 (2011).
8. Tóth, J.P., Bereczki, J., Varga, Z., Rota, J., Sramkó, G. & Wahlberg, N. Relationships within the *Melitaea phoebe* species group (Lepidoptera: Nymphalidae): new insights from molecular and morphometric information. *Systematic Entomology* **39,** 749–757 (2014).
9. Tóth, J. P., Varga, Z., Verovnik, R., Wahlberg, N., Váradi, A. & Bereczki, J. Mito-nuclear discordance helps to reveal the phylogeographic patterns of *Melitaea ornata* (Lepidoptera: Nymphalidae). *Biological Journal of the Linnean Society* **121,** 267–281 (2017).
10. Mesa, L. S. & Muñoz Sariot, M. G. *Melitaea ornata* (Cristoph, 1893), nueva especie para la Península Ibérica. Primeros datos de su morfología, biología y ecología comparada con los de *Melitaea phoebe* (Denis & Schiffermüller, 1775). (Lepidoptera: Nymphalidae). *Arquivos Entomolóxicos* **18,** 313–324 (2017).
11. Hernández-Roldán, J. L. & Munguira, M. L. Multivariate analysis techniques in the study of the male genitalia of *Pyrgus bellieri* (Oberthür 1910) and *P. alveus* (Hübner

1803) (Lepidoptera : Hesperiidae): species discrimination and distribution in the Iberian Peninsula. *Ann. Soc. Entomol. Fr.* **44,** 145–155 (2008).

12. deWaard, J. R., Ivanova, N. V., Hajibabaei, M. & Hebert, P. D. N. Assembling DNA barcodes: analytical protocols in *Methods in molecular biology: environmental genetics* (ed. Martin, C.) 275–293 (Humana Press, 2008).
13. Collins, R. A., Boykin, L. M., Cruickshank, R. H. & Armstrong, C. F. Barcoding´s next top model: an evaluation of nucleotide substitution models for specimen identification. *Methods Ecol. Evol.* **3,** 457–465 (2012)
14. Srivathsan, A. & Meier, R. On the inappropriate use of Kimura-2-parameter (K2P) divergences in the DNA-barcoding literature. *Cladistics* **28,** 190–194 (2012).
