# Supplementary material for: High resolution DNA barcode library for European butterflies reveals continental patterns of mitochondrial genetic diversity
Source: Commun Biol. 2021 Mar 9;4:315. doi: 10.1038/s42003-021-01834-7 (PMC7943782; doi:10.1038/s42003-021-01834-7)
Supplement: Supplementary file 3 — Description of Additional Supplementary Files [file 42003_2021_1834_MOESM3_ESM.pdf]

Description of Additional Supplementary Files

File name: Supplementary Data 1.
Description: Specimens used in the study.

File name: Supplementary Data 2.
Description: Species of European butterflies included in this study. The taxonomy and nomenclature follow Wiemers et al. (2018).

File name: Supplementary Data 3.
Description: Observed and estimated genetic diversity for 404 species of European butterflies represented by at least six COI sequences in the dataset.

File name: Supplementary Data 4.
Description: Percentage of haplotypes belonging to different octaves of frequency (log2) for the 404 species examined.

File name: Supplementary Data 5.
Description: The number of haplotypes retrieved in latitude belts in the full dataset (Haplotypes_full) and the homogeneous subset (Haplotypes_subset). The number of species included in each belt and the mean distance among specimens of the same species are also reported.

File name: Supplementary Data 6.
Description: The number of haplotypes retrieved in longitude belts in the full dataset (Haplotypes_full) and the homogeneous subset (Haplotypes_subset). The number of species included in each belt and the mean distance among specimens of the same species are also reported.

File name: Supplementary Data 7.
Description: The significant effects of latitude and longitude in explaining haplotype diversity (full datasets and subsets) in General Additive Mixed Models comparing the effects of smoothed latitude and longitude and geographic distances over haplotype richness.

File name: Supplementary Data 8.
Description: The non-significant effects of distances between specimens in explaining haplotype diversity (full datasets and subsets) in General Additive Mixed Models comparing the effects of smoothed latitude and longitude and geographic distances.

File name: Supplementary Data 9.
Description: PROTAX probabilities of correct specimen identification. Eight of the 459 species in the dataset were represented by singletons, hence their probability of assignment to the correct species was zero; these species are not included in the table (Azanus jesous, Polyommatus aroaniensis, Tongeia

fischeri, Lycaena dimorpha, Hipparchia blachieri, Hipparchia pellucida, Hipparchia autonoe, Erebia cyclopius).

File name: Supplementary Data 10.
Description: PROTAX average probability of correct identification for each species and distances to nearest neighbour for 451 species of European butterflies. PROTAX probabilities could not be inferred for 8 species represented by singletons (Azanus jesous, Polyommatus aroaniensis, Tongeia fischeri, Lycaena dimorpha, Hipparchia blachieri, Hipparchia pellucida, Hipparchia autonoe, Erebia cyclopius).

File name: Supplementary Data 11.
Description: Barcode index numbers (BINs) that are concordant with the taxonomy used in this paper.

File name: Supplementary Data 12.
Description: Barcode index numbers (BINs) represented by a single specimen from the dataset.

File name: Supplementary Data 13.
Description: Barcode index numbers (BINs) that each include more than one species recognized in this study. 168 species (36.6% of the species in the dataset) share BIN with at least another species.

File name: Supplementary Data 14.
Description: Neighbor-joining tree of 22,306 COI sequences used in the study.

File name: Supplementary Data 15.
Description: Summary of distances to nearest neighbour (NN). Cases of barcode sharing are highlighted in orange. Cases that do not share barcodes, but had PROTAX probabilities of correct taxonomic assignment lower than 0.999, are highlighted in light blue. When the NN distance was higher than 0.61%, PROTAX probabilities or correct assignment to species were always at least 0.999 (99.9%).

File name: Supplementary Data 16.
Description: Taxa displaying at least 2.5% maximum intraspecific divergence.
