# Supplementary material for: High resolution DNA barcode library for European butterflies reveals continental patterns of mitochondrial genetic diversity
Source: Commun Biol. 2021 Mar 9;4:315. doi: 10.1038/s42003-021-01834-7 (PMC7943782; doi:10.1038/s42003-021-01834-7)
Supplement: Supplementary file 17 — Supplementary Data 14 [file 42003_2021_1834_MOESM17_ESM.pdf]

BOLD TaxonID Tree

Title	:	Tree Result - DS-EUGENMAP
Date	:	23-Mar-2019
Data Type	:	Nucleotide
Distance Model	:	Pairwise Distance
Marker	:	COI-5P
Label	:	Sample ID
Label	:	Process ID
Label	:	Taxon
Label	:	Extra Info
Label	:	Country
Label	:	Sequence Length
Sequence Count	:	22306
Species count	:	459
Genus count	:	107
Family count	:	6
Unidentified	:	232
BIN Count	:	441

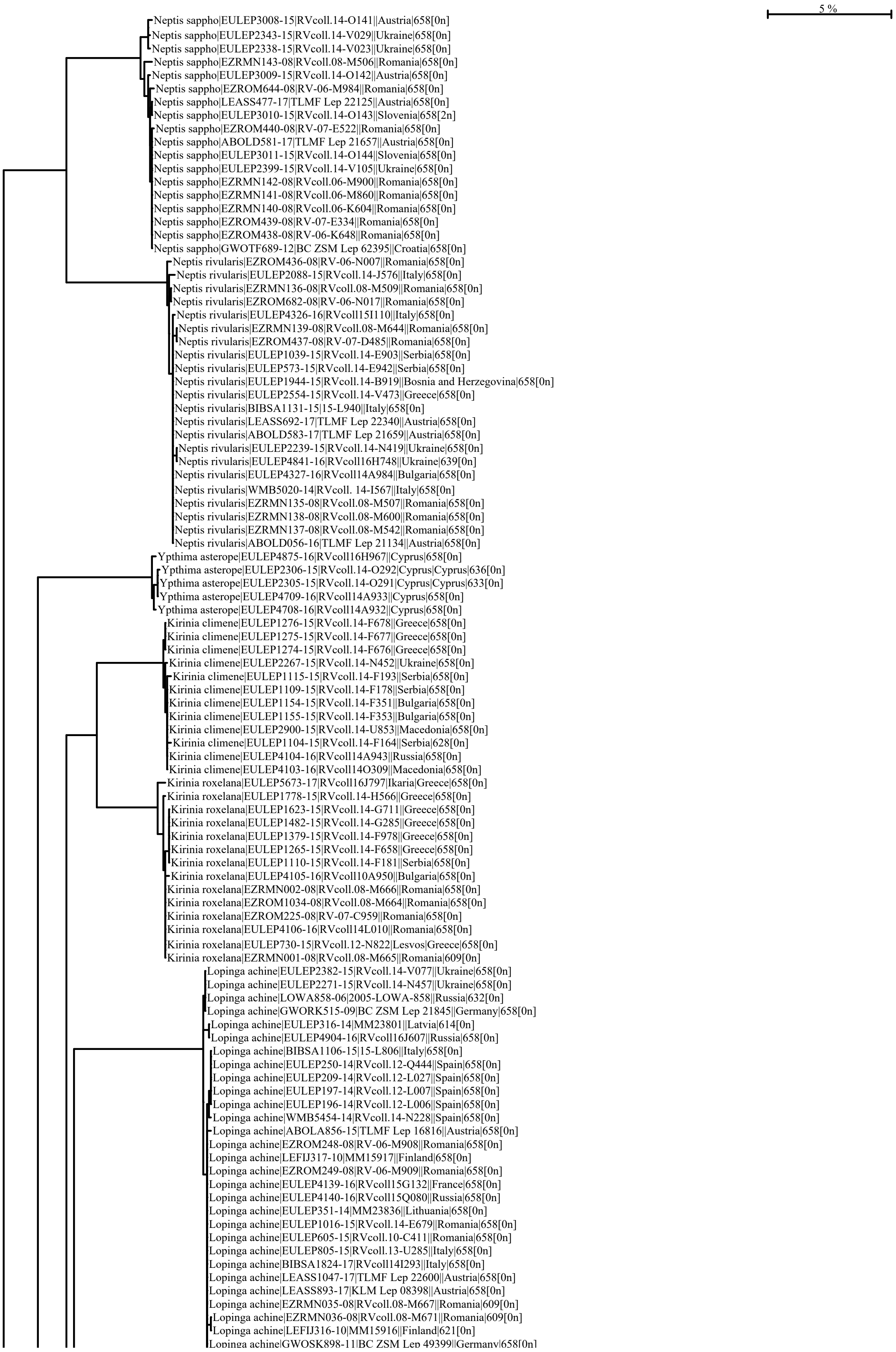
5 %
Neptis sappho|EULEP3008-15|RVcoll.14-O141||Austria|658[0n]
Neptis sappho|EULEP2343-15|RVcoll.14-V029||Ukraine|658[0n]
Neptis sappho|EULEP2338-15|RVcoll.14-V023||Ukraine|658[0n]
Neptis sappho|EZRMN143-08|RVcoll.08-M506||Romania|658[0n]
Neptis sappho|EULEP3009-15|RVcoll.14-O142||Austria|658[0n]
Neptis sappho|EZROM644-08|RV-06-M984||Romania|658[0n]
Neptis sappho|LEASS477-17|TLMF Lep 22125||Austria|658[0n]
Neptis sappho|EULEP3010-15|RVcoll.14-O143||Slovenia|658[2n]
Neptis sappho|EZROM440-08|RV-07-E522||Romania|658[0n]
Neptis sappho|ABOLD581-17|TLMF Lep 21657||Austria|658[0n]
Neptis sappho|EULEP3011-15|RVcoll.14-O144||Slovenia|658[0n]
Neptis sappho|EULEP2399-15|RVcoll.14-V105||Ukraine|658[0n]
Neptis sappho|EZRMN142-08|RVcoll.06-M900||Romania|658[0n]
Neptis sappho|EZRMN141-08|RVcoll.06-M860||Romania|658[0n]
Neptis sappho|EZRMN140-08|RVcoll.06-K604||Romania|658[0n]
Neptis sappho|EZROM439-08|RV-07-E334||Romania|658[0n]
Neptis sappho|EZROM438-08|RV-06-K648||Romania|658[0n]
Neptis sappho|GWOTF689-12|BC ZSM Lep 62395||Croatia|658[0n]
Neptis rivularis|EZROM436-08|RV-06-N007||Romania|658[0n]
Neptis rivularis|EULEP2088-15|RVcoll.14-J576||Italy|658[0n]
Neptis rivularis|EZRMN136-08|RVcoll.08-M509||Romania|658[0n]
Neptis rivularis|EZROM682-08|RV-06-N017||Romania|658[0n]
Neptis rivularis|EULEP4326-16|RVcoll15I110||Italy|658[0n]
Neptis rivularis|EZRMN139-08|RVcoll.08-M644||Romania|658[0n]
Neptis rivularis|EZROM437-08|RV-07-D485||Romania|658[0n]
Neptis rivularis|EULEP1039-15|RVcoll.14-E903||Serbia|658[0n]
Neptis rivularis|EULEP573-15|RVcoll.14-E942||Serbia|658[0n]
Neptis rivularis|EULEP1944-15|RVcoll.14-B919||Bosnia and Herzegovina|658[0n]
Neptis rivularis|EULEP2554-15|RVcoll.14-V473||Greece|658[0n]
Neptis rivularis|BIBSA1131-15|15-L940||Italy|658[0n]
Neptis rivularis|LEASS692-17|TLMF Lep 22340||Austria|658[0n]
Neptis rivularis|ABOLD583-17|TLMF Lep 21659||Austria|658[0n]
Neptis rivularis|EULEP2239-15|RVcoll.14-N419||Ukraine|658[0n]
Neptis rivularis|EULEP4841-16|RVcoll16H748||Ukraine|639[0n]
Neptis rivularis|EULEP4327-16|RVcoll14A984||Bulgaria|658[0n]
Neptis rivularis|WMB5020-14|RVcoll. 14-I567||Italy|658[0n]
Neptis rivularis|EZRMN135-08|RVcoll.08-M507||Romania|658[0n]
Neptis rivularis|EZRMN138-08|RVcoll.08-M600||Romania|658[0n]
Neptis rivularis|EZRMN137-08|RVcoll.08-M542||Romania|658[0n]
Neptis rivularis|ABOLD056-16|TLMF Lep 21134||Austria|658[0n]
Ypthima asterope|EULEP4875-16|RVcoll16H967||Cyprus|658[0n]
Ypthima asterope|EULEP2306-15|RVcoll.14-O292|Cyprus|Cyprus|636[0n]
Ypthima asterope|EULEP2305-15|RVcoll.14-O291|Cyprus|Cyprus|633[0n]
Ypthima asterope|EULEP4709-16|RVcoll14A933||Cyprus|658[0n]
Ypthima asterope|EULEP4708-16|RVcoll14A932||Cyprus|658[0n]
Kirinia climene|EULEP1276-15|RVcoll.14-F678||Greece|658[0n]
Kirinia climene|EULEP1275-15|RVcoll.14-F677||Greece|658[0n]
Kirinia climene|EULEP1274-15|RVcoll.14-F676||Greece|658[0n]
Kirinia climene|EULEP2267-15|RVcoll.14-N452||Ukraine|658[0n]
Kirinia climene|EULEP1115-15|RVcoll.14-F193||Serbia|658[0n]
Kirinia climene|EULEP1109-15|RVcoll.14-F178||Serbia|658[0n]
Kirinia climene|EULEP1154-15|RVcoll.14-F351||Bulgaria|658[0n]
Kirinia climene|EULEP1155-15|RVcoll.14-F353||Bulgaria|658[0n]
Kirinia climene|EULEP2900-15|RVcoll.14-U853||Macedonia|658[0n]
Kirinia climene|EULEP1104-15|RVcoll.14-F164||Serbia|628[0n]
Kirinia climene|EULEP4104-16|RVcoll14A943||Russia|658[0n]
Kirinia climene|EULEP4103-16|RVcoll14O309||Macedonia|658[0n]
Kirinia roxelana|EULEP5673-17|RVcoll16J797|Ikaria|Greece|658[0n]
Kirinia roxelana|EULEP1778-15|RVcoll.14-H566||Greece|658[0n]
Kirinia roxelana|EULEP1623-15|RVcoll.14-G711||Greece|658[0n]
Kirinia roxelana|EULEP1482-15|RVcoll.14-G285||Greece|658[0n]
Kirinia roxelana|EULEP1379-15|RVcoll.14-F978||Greece|658[0n]
Kirinia roxelana|EULEP1265-15|RVcoll.14-F658||Greece|658[0n]
Kirinia roxelana|EULEP1110-15|RVcoll.14-F181||Serbia|658[0n]
Kirinia roxelana|EULEP4105-16|RVcoll10A950||Bulgaria|658[0n]
Kirinia roxelana|EZRMN002-08|RVcoll.08-M666||Romania|658[0n]
Kirinia roxelana|EZROM1034-08|RVcoll.08-M664||Romania|658[0n]
Kirinia roxelana|EZROM225-08|RV-07-C959||Romania|658[0n]
Kirinia roxelana|EULEP4106-16|RVcoll14L010||Romania|658[0n]
Kirinia roxelana|EULEP730-15|RVcoll.12-N822|Lesvos|Greece|658[0n]
Kirinia roxelana|EZRMN001-08|RVcoll.08-M665||Romania|609[0n]
Lopinga achine|EULEP2382-15|RVcoll.14-V077||Ukraine|658[0n]
Lopinga achine|EULEP2271-15|RVcoll.14-N457||Ukraine|658[0n]
Lopinga achine|LOWA858-06|2005-LOWA-858||Russia|632[0n]
Lopinga achine|GWORK515-09|BC ZSM Lep 21845||Germany|658[0n]
Lopinga achine|EULEP316-14|MM23801||Latvia|614[0n]
Lopinga achine|EULEP4904-16|RVcoll16J607||Russia|658[0n]
Lopinga achine|BIBSA1106-15|15-L806||Italy|658[0n]
Lopinga achine|EULEP250-14|RVcoll.12-Q444||Spain|658[0n]
Lopinga achine|EULEP209-14|RVcoll.12-L027||Spain|658[0n]
Lopinga achine|EULEP197-14|RVcoll.12-L007||Spain|658[0n]
Lopinga achine|EULEP196-14|RVcoll.12-L006||Spain|658[0n]
Lopinga achine|WMB5454-14|RVcoll.14-N228||Spain|658[0n]
Lopinga achine|ABOLA856-15|TLMF Lep 16816||Austria|658[0n]
Lopinga achine|EZROM248-08|RV-06-M908||Romania|658[0n]
Lopinga achine|LEFIJ317-10|MM15917||Finland|658[0n]
Lopinga achine|EZROM249-08|RV-06-M909||Romania|658[0n]
Lopinga achine|EULEP4139-16|RVcoll15G132||France|658[0n]
Lopinga achine|EULEP4140-16|RVcoll15Q080||Russia|658[0n]
Lopinga achine|EULEP351-14|MM23836||Lithuania|658[0n]
Lopinga achine|EULEP1016-15|RVcoll.14-E679||Romania|658[0n]
Lopinga achine|EULEP605-15|RVcoll.10-C411||Romania|658[0n]
Lopinga achine|EULEP805-15|RVcoll.13-U285||Italy|658[0n]
Lopinga achine|BIBSA1824-17|RVcoll14I293||Italy|658[0n]
Lopinga achine|LEASS1047-17|TLMF Lep 22600||Austria|658[0n]
Lopinga achine|LEASS893-17|KLM Lep 08398||Austria|658[0n]
Lopinga achine|EZRMN035-08|RVcoll.08-M667||Romania|609[0n]
Lopinga achine|EZRMN036-08|RVcoll.08-M671||Romania|609[0n]
Lopinga achine|LEFIJ316-10|MM15916||Finland|621[0n]
Lopinga achine|GWOSK898-11|BC ZSM Lep 49399||Germany|658[0n]

Lopinga achine|EZRMN036-08|RVcoll.08-M671||Romania|609[0n]
Lopinga achine|LEFIJ316-10|MM15916||Finland|621[0n]
Lopinga achine|GWOSK898-11|BC ZSM Lep 49399||Germany|658[0n]
Lopinga achine|GWORT459-10|BC ZSM Lep 32145||Germany|658[0n]
Lopinga achine|GWOTF679-12|BC ZSM Lep 62385||Germany|658[0n]
Lopinga achine|ABOLD041-16|TLMF Lep 21119||Austria|658[0n]
Lopinga achine|ABOLA857-15|TLMF Lep 16817||Austria|658[0n]
Lopinga achine|LASTS332-14|TLMF Lep 14784||Austria|658[0n]
Lasiommata maera|EZSPM779-12|Rvcoll. 070111GN29||Spain|658[0n]
Lasiommata maera|WMB6130-18|RVcoll11I674||France|658[0n]
Lasiommata maera|EZSPM406-09|Rvcoll.09-V818||Spain|658[0n]
Lasiommata maera|WMB1651-13|RVcoll.12-O995||France|658[0n]
Lasiommata maera|WMB010-11|RVcoll.11-E019||Spain|658[0n]
Lasiommata maera|EZSPC462-09|RVcoll.08-M926||Spain|658[0n]
Lasiommata maera|WMB3191-14|RVcoll.08-J048.1||Spain|658[0n]
Lasiommata maera|WMB5509-14|RVcoll.14-M305||France|658[0n]
Lasiommata maera|EZSPN961-09|RVcoll.08-L966||Spain|656[0n]
Lasiommata maera|WMB3640-14|RVcoll.11-I503||Spain|658[0n]
Lasiommata maera|OXB1544-16|OXB-TGS-1262||France|658[0n]
Lasiommata maera|WMB6156-18|RVcoll12P101||France|658[0n]
Lasiommata maera|WMB3584-14|RVcoll.11-E068||Spain|658[0n]
Lasiommata maera|EZSPC1238-10|RVcoll.09-V746||Spain|658[0n]
Lasiommata maera|EZSPC1234-10|RVcoll.09-V705||Spain|658[0n]
Lasiommata maera|EZSPC1384-10|RVcoll.09-X531||Spain|658[0n]
Lasiommata maera|EZSPM264-09|RVcoll.08-R479||Spain|658[0n]
Lasiommata maera|EZSPM145-09|RVcoll.08-P658||Spain|658[0n]
Lasiommata maera|EZSPN974-09|RVcoll.08-L999||Spain|658[0n]
Lasiommata maera|EZSPN972-09|RVcoll.08-L997||Spain|658[0n]
Lasiommata maera|EZSPN966-09|RVcoll.08-L980||Spain|658[0n]
Lasiommata maera|EZSPM367-09|RVcoll.08-J832||Spain|658[0n]
Lasiommata maera|EZSPM766-12|Rvcoll. 040611HZ35||Spain|658[0n]
Lasiommata maera|EZSPC463-09|RVcoll.08-R110||Spain|658[0n]
Lasiommata maera|EZSPC461-09|RVcoll.08-M907||Spain|658[0n]
Lasiommata maera|WMB009-11|RVcoll.11-D967||Spain|658[0n]
Lasiommata maera|WMB6165-18|RVcoll12R249||Greece|658[0n]
Lasiommata maera|EULEP2912-15|RVcoll.14-U865||Greece|603[1n]
Lasiommata maera|WMB6149-18|RVcoll12N800|Lesvos|Greece|658[0n]
Lasiommata maera|WMB6106-18|RVcoll11D560||Russia|658[0n]
Lasiommata maera|EZROM228-08|RV-06-K626|Genit. examined|Romania|658[0n]
Lasiommata maera|EULEP895-15|RVcoll.14-C695||Greece|658[0n]
Lasiommata maera|EULEP2001-15|RVcoll.14-I778||Slovakia|658[0n]
Lasiommata maera|EULEP5028-16|RVcoll16I967||Slovakia|613[0n]
Lasiommata maera|EULEP5059-16|RVcoll16J110||Czech Republic|658[0n]
Lasiommata maera|WMB6177-18|RVcoll12Z333||Sweden|658[0n]
Lasiommata maera|WMB6176-18|RVcoll12Z332||Sweden|658[0n]
Lasiommata maera|GWOSK897-11|BC ZSM Lep 49398||Germany|658[0n]
Lasiommata maera|GWORT460-10|BC ZSM Lep 32146||Germany|658[0n]
Lasiommata maera|BIBSA1058-15|11-H390||Italy|658[0n]
Lasiommata maera|EULEP1101-15|RVcoll.14-F150||Serbia|658[0n]
Lasiommata maera|EULEP1038-15|RVcoll.14-E901||Serbia|614[0n]
Lasiommata maera|EULEP5664-17|RVcoll15P000||Romania|658[0n]
Lasiommata maera|EZROM1056-09|RVcoll.07-E341||Romania|658[0n]
Lasiommata maera|EZROM232-08|RV-07-D219||Romania|655[0n]
Lasiommata maera|EZROM230-08|RV-07-D092||Romania|658[0n]
Lasiommata maera|GWOTD802-12|BC ZSM Lep 64693||Croatia|658[0n]
Lasiommata maera|EZROM229-08|RV-06-M844||Romania|658[0n]
Lasiommata maera|EULEP4124-16|RVcoll15Q196||Ukraine|658[0n]
Lasiommata maera|EZROM1043-09|RVcoll.07-D062||Romania|658[0n]
Lasiommata maera|EULEP903-15|RVcoll.14-C741||Bulgaria|658[0n]
Lasiommata maera|WMB6193-18|RVcoll14C839||Bulgaria|658[1n]
Lasiommata maera|ABOLD015-16|TLMF Lep 21093||Austria|658[0n]
Lasiommata maera|LEFIJ563-10|MM17188||Finland|658[0n]
Lasiommata maera|LEFID593-10|MM06603||Finland|658[0n]
Lasiommata maera|EZRMN004-08|RVcoll.08-M494||Romania|658[0n]
Lasiommata maera|EZRMN005-08|RVcoll.08-M594||Romania|658[0n]
Lasiommata maera|EZROM1052-09|RVcoll.07-D495||Romania|658[0n]
Lasiommata maera|EZRMN003-08|RVcoll.06-M986||Romania|658[0n]
Lasiommata maera|EZROM231-08|RV-07-D153||Romania|658[0n]
Lasiommata maera|EZROM1048-09|RVcoll.07-D183||Romania|658[0n]
Lasiommata maera|EULEP1921-15|RVcoll.14-B845||Bosnia and Herzegovina|658[0n]
Lasiommata maera|EULEP2008-15|RVcoll.14-I797||Poland|658[0n]
Lasiommata maera|EULEP1599-15|RVcoll.14-G652||Greece|616[0n]
Lasiommata maera|EULEP1375-15|RVcoll.14-F970||Greece|658[0n]
Lasiommata maera|WMB6109-18|RVcoll11D563||Russia|658[0n]
Lasiommata maera|LEFIJ562-10|MM17187||Finland|615[0n]
Lasiommata maera|ABOLD050-16|TLMF Lep 21128||Austria|658[0n]
Lasiommata maera|BIBSA399-15|RVcoll.14-I067||Italy|658[0n]
Lasiommata maera|EULEP4120-16|RVcoll15H308||Switzerland|658[0n]
Lasiommata maera|EULEP4118-16|RVcoll15G971||Switzerland|658[0n]
Lasiommata maera|WMB4248-14|RVcoll.13-U052||Italy|658[0n]
Lasiommata maera|WMB1966-13|RVcoll.11-I146||Italy|658[0n]
Lasiommata maera|WMB1960-13|RVcoll.11-I088|Sicily|Italy|655[0n]
Lasiommata maera|BIBSA1556-16|11-H309||Italy|658[0n]
Lasiommata maera|BIBSA1545-16|LEP-SS-00379||Italy|658[0n]
Lasiommata maera|BIBSA504-15|LEP-SS-00124||Italy|658[0n]
Lasiommata maera|OXB1084-15|15-A617||Italy|658[0n]
Lasiommata maera|WMB4894-14|RVcoll.14-I441||Italy|645[0n]
Lasiommata maera|WMB6258-18|RVcollLD2927|Sicily|Italy|658[0n]
Lasiommata maera|WMB6217-18|RVcollLD1505|Sicily|Italy|658[0n]
Lasiommata maera|WMB6216-18|RVcollLD1495|Sicily|Italy|658[0n]
Lasiommata maera|WMB6105-18|RVcoll10C508||Italy|658[0n]
Lasiommata maera|WMB6163-18|RVcoll12R172|Sicily|Italy|658[0n]
Lasiommata maera|WMB1961-13|RVcoll.11-I089|Sicily|Italy|658[0n]
Lasiommata maera|WMB1923-13|RVcoll.11-H783|Sicily|Italy|658[0n]
Lasiommata maera|WMB6264-18|RVcollLD3151||Italy|658[0n]
Lasiommata maera|BIBSA1759-16|12-Q855||Italy|658[0n]
Lasiommata maera|WMB4335-14|RVcoll.14-A720||Italy|658[0n]
Lasiommata maera|WMB4860-14|RVcoll.14-I407||Italy|658[0n]
Lasiommata maera|EULEP4119-16|RVcoll15H228||Switzerland|658[0n]
Lasiommata maera|WMB822-13|RVcoll.07-E186||Italy|636[0n]
Lasiommata maera|LEASS748-17|TLMF Lep 22396||Austria|658[0n]
Lasiommata maera|OXB961-15|15-A911||Italy|658[0n]
[illegible]

Lasiommata maera|WMB822-13|RVcoll.07-E186||Italy|658[0n]
Lasiommata maera|LEASS748-17|TLMF Lep 22396||Austria|658[0n]
Lasiommata maera|OXB961-15|15-A911||Italy|658[0n]
Lasiommata maera|WMB4969-14|RVcoll. 14-I516||Italy|658[0n]
Lasiommata maera|WMB6241-18|RVcollLD2342||Italy|658[0n]
Lasiommata maera|WMB6131-18|RVcoll11J142||Switzerland|658[0n]
Lasiommata maera|WMB6101-18|RVcoll10C330||Switzerland|658[0n]
Lasiommata maera|WMB6100-18|RVcoll10C071||France|658[0n]
Lasiommata maera|LEATG511-14|TLMF Lep 14298||Italy|658[0n]
Lasiommata maera|LEATG074-14|TLMF Lep 13861||Austria|658[0n]
Lasiommata maera|LEATD156-13|TLMF Lep 12803||Italy|658[0n]
Lasiommata maera|GWORZ034-10|BC ZSM Lep 30390||Italy|658[0n]
Lasiommata maera|FBLMU437-09|BC ZSM Lep 27087||Germany|658[0n]
Lasiommata maera|GWORK564-09|BC ZSM Lep 21894||Germany|658[0n]
Lasiommata maera|WMB3492-14|RVcoll.10-B687||France|658[0n]
Lasiommata maera|WMB1604-13|RVcoll.12-O665||France|658[0n]
Lasiommata maera|WMB4159-14|RVcoll.13-S716||Italy|658[0n]
Lasiommata maera|WMB6187-18|RVcoll13T328||Italy|658[0n]
Lasiommata maera|WMB6190-18|RVcoll13U425||Italy|658[0n]
Lasiommata maera|WMB6252-18|RVcollLD2801||Italy|658[0n]
Lasiommata maera|WMB6253-18|RVcollLD2857||Italy|658[0n]
Lasiommata maera|WMB6262-18|RVcollLD3144||Italy|658[0n]
Lasiommata maera|WMB6263-18|RVcollLD3145||Italy|658[0n]
Lasiommata maera|WMB5175-14|RVcoll.13-S522||Italy|658[0n]
Lasiommata maera|EULEP4121-16|RVcoll15I043||Italy|658[0n]
Lasiommata maera|EULEP4122-16|RVcoll15I379||Austria|658[0n]
Lasiommata maera|EULEP4123-16|RVcoll15I640||Austria|658[0n]
Lasiommata maera|BIBSA1167-15|15-M188||Italy|658[0n]
Lasiommata maera|BIBSA1045-15|15-C276||Italy|658[0n]
Lasiommata maera|BIBSA105-15|RVcoll.14-D529||Italy|658[0n]
Lasiommata maera|BIBSA119-15|RVcoll.14-D554||Italy|658[0n]
Lasiommata maera|BIBSA1362-15|15-M884||Italy|658[0n]
Lasiommata maera|BIBSA673-15|RVcoll. 11-I277||Italy|658[0n]
Lasiommata maera|BIBSA674-15|RVcoll. 11-I238||Italy|658[0n]
Lasiommata maera|BIBSA505-15|LEP-SS-00125||Italy|658[0n]
Lasiommata maera|BIBSA227-15|RVcoll.14-E018||Italy|658[0n]
Lasiommata maera|LEASS747-17|TLMF Lep 22395||Austria|658[0n]
Lasiommata maera|LEASS983-17|TLMF Lep 22536||Austria|658[0n]
Lasiommata maera|OXB585-15|14-N974||Italy|658[0n]
Lasiommata maera|OXB702-15|13-T875||Italy|658[0n]
Lasiommata maera|OXB907-15|15-A551||Italy|658[0n]
Lasiommata maera|OXB1567-16|OXB-TGS-1285||France|637[0n]
Lasiommata maera|OXB1099-15|15-A632||Italy|658[0n]
Lasiommata maera|OXB608-15|14-N997||Italy|658[0n]
Lasiommata maera|WMB6259-18|RVcollLD3136||Italy|658[0n]
Lasiommata maera|WMB2464-13|RVcoll.11-H353||Italy|658[0n]
Lasiommata maera|LEATH759-14|TLMF Lep 15971||Italy|634[0n]
Lasiommata maera|WMB6189-18|RVcoll13U266||Italy|658[0n]
Lasiommata maera|WMB6186-18|RVcoll13T326||Italy|658[0n]
Lasiommata maera|LEATA410-13|TLMF Lep 10017||Austria|658[0n]
Lasiommata megera|OLEP057-15|RVcoll.14-V561|Lampedusa|Italy|658[0n]
Lasiommata megera|WMB5918-17|RVcoll11D126|Pantelleria. Genit. examined|Italy|658[0n]
Lasiommata megera|WMB5917-17|RVcoll11D125|Pantelleria. Genit. examined|Italy|658[0n]
Lasiommata megera|WMB5916-17|RVcoll11D124|Pantelleria. Genit. examined|Italy|658[0n]
Lasiommata megera|WMB2329-13|RVcoll.11-H674|Pantelleria|Italy|658[0n]
Lasiommata megera|WMB2328-13|RVcoll.11-H673|Pantelleria|Italy|658[0n]
Lasiommata megera|WMB2327-13|RVcoll.11-H672|Pantelleria|Italy|658[0n]
Lasiommata megera|WMB132-11|RVcoll.11-D134|Pantelleria. Genit. examined|Italy|658[0n]
Lasiommata paramegaera|WMB2712-13|RVcoll.10-C451|Capraia|Italy|658[0n]
Lasiommata paramegaera|WMB6314-18|RVcollLD0442|Capraia|Italy|658[0n]
Lasiommata paramegaera|WMB6315-18|RVcollLD0443|Capraia|Italy|658[0n]
Lasiommata paramegaera|WMB2711-13|RVcoll.10-C450|Capraia|Italy|632[0n]
Lasiommata paramegaera|WMB6254-18|RVcollLD2867|Asinara|Italy|658[0n]
Lasiommata paramegaera|WMB1161-13|RVcoll.11-E620|Santa Maria|Italy|658[0n]
Lasiommata paramegaera|WMB1168-13|RVcoll.11-E717|Corsica|France|658[0n]
Lasiommata paramegaera|WMB847-13|RVcoll.08-H750|Sardinia|Italy|658[0n]
Lasiommata paramegaera|WMB1547-13|RVcoll.12-O314|La Maddalena|Italy|658[0n]
Lasiommata paramegaera|WMB2311-13|RVcoll.11-E161|Montecristo. Genit. examined|Italy|658[0n]
Lasiommata paramegaera|WMB2312-13|RVcoll.11-E162|Montecristo. Genit. examined|Italy|658[0n]
Lasiommata paramegaera|WMB2629-13|RVcoll.12-O181|Corsica|France|658[0n]
Lasiommata paramegaera|WMB2630-13|RVcoll.12-O209|Corsica|France|658[0n]
Lasiommata paramegaera|WMB6152-18|RVcoll12O315|La Maddalena|Italy|658[0n]
Lasiommata paramegaera|WMB6153-18|RVcoll12O323|La Maddalena|Italy|658[0n]
Lasiommata paramegaera|WMB6154-18|RVcoll12O324|Caprera|Italy|658[0n]
Lasiommata paramegaera|WMB6155-18|RVcoll12O336|Caprera|Italy|658[0n]
Lasiommata paramegaera|WMB6255-18|RVcollLD2868|Asinara|Italy|658[0n]
Lasiommata paramegaera|WMB6256-18|RVcollLD2869|Asinara|Italy|658[0n]
Lasiommata paramegaera|WMB6257-18|RVcollLD2870|Asinara|Italy|658[0n]
Lasiommata paramegaera|WMB6295-18|RVcoll09T506|Sardinia|Italy|658[0n]
Lasiommata paramegaera|WMB6296-18|RVcoll09T507|Sardinia|Italy|658[0n]
Lasiommata paramegaera|WMB6297-18|RVcoll11E159|Montecristo. Genit. examined|Italy|658[0n]
Lasiommata paramegaera|WMB6298-18|RVcoll11E160|Montecristo. Genit. examined|Italy|658[0n]
Lasiommata paramegaera|WMB6299-18|RVcoll11E910|Corsica. Genit. examined|France|658[0n]
Lasiommata paramegaera|WMB6300-18|RVcoll11E912|Corsica. Genit. examined|France|658[0n]
Lasiommata paramegaera|WMB6301-18|RVcoll11E913|Corsica. Genit. examined|France|658[0n]
Lasiommata paramegaera|WMB6302-18|RVcoll11E964|Corsica. Genit. examined|France|658[0n]
Lasiommata paramegaera|WMB6303-18|RVcoll11E965|Corsica. Genit. examined|France|658[0n]
Lasiommata paramegaera|WMB6304-18|RVcoll11E966|Corsica. Genit. examined|France|658[0n]
Lasiommata paramegaera|WMB6305-18|RVcoll12O088|Corsica|France|658[0n]
Lasiommata paramegaera|WMB6306-18|RVcoll12O179|Corsica|France|658[0n]
Lasiommata paramegaera|WMB6307-18|RVcoll12O266|Corsica|France|658[0n]
Lasiommata paramegaera|WMB6308-18|RVcoll12O346|Sardinia|Italy|658[0n]
Lasiommata paramegaera|WMB6309-18|RVcoll12O534|Sardinia|Italy|658[0n]
Lasiommata paramegaera|WMB6310-18|RVcoll12Q474|Sardinia|Italy|658[0n]
Lasiommata paramegaera|WMB6311-18|RVcollLD0390|Corsica|France|658[0n]
Lasiommata paramegaera|WMB6312-18|RVcollLD0391|Corsica|France|658[0n]
Lasiommata paramegaera|WMB6313-18|RVcollLD0440|Capraia|Italy|658[0n]
Lasiommata paramegaera|WMB6316-18|RVcollLD0502|Sardinia|Italy|658[0n]
Lasiommata paramegaera|WMB6317-18|RVcollLD2217|Sardinia|Italy|658[0n]
Lasiommata paramegaera|WMB6318-18|RVcollLD2218|Sardinia|Italy|658[0n]
Lasiommata paramegaera|WMB6319-18|RVcollLD2341|Montecristo|Italy|658[0n]
Lasiommata paramegaera|BIBSA1268-15|08-H775|Corsica|France|658[0n]
Lasiommata paramegaera|BIBSA1316-15|12-O379|Sardinia|Italy|658[0n]

Lasiommata paramegaera|WMB6319-18|RVcollLD2341|Montecristo|Italy|658[0n]
Lasiommata paramegaera|BIBSA1268-15|08-H775|Corsica|France|658[0n]
Lasiommata paramegaera|BIBSA1316-15|12-O379|Sardinia|Italy|658[0n]
Lasiommata paramegaera|WMB1151-13|RVcoll.11-E305|Sardinia|Italy|615[0n]
Lasiommata paramegaera|WMB1149-13|RVcoll.11-E158|Montecristo. Genit. examined|Italy|601[0n]
Lasiommata paramegaera|WMB1148-13|RVcoll.11-E157|Montecristo. Genit. examined|Italy|658[0n]
Lasiommata paramegaera|WMB3002-14|RVcoll.12-O432|Sant Antioco|Italy|658[0n]
Lasiommata paramegaera|WMB2852-14|RVcoll.08-H749|Sardinia|Italy|658[0n]
Lasiommata megera|WMB6240-18|RVcollLD2339||Ukraine|658[0n]
Lasiommata megera|EULEP1323-15|RVcoll.14-F792||Greece|658[0n]
Lasiommata megera|WMB6151-18|RVcoll12N808|Lesvos|Greece|654[0n]
Lasiommata megera|WMB6205-18|RVcoll17A734||Croatia|658[0n]
Lasiommata megera|WMB6206-18|RVcoll17A770||Croatia|658[0n]
Lasiommata megera|WMB6207-18|RVcoll17B031|Pag|Croatia|658[0n]
Lasiommata megera|WMB6208-18|RVcoll17B033|Pag|Croatia|658[0n]
Lasiommata megera|WMB6247-18|RVcollLD2376||Russia|658[0n]
Lasiommata megera|WMB6248-18|RVcollLD2377||Russia|658[0n]
Lasiommata megera|WMB6265-18|RVcollLD3156||Greece|658[0n]
Lasiommata megera|EULEP1213-15|RVcoll.14-F509||Greece|658[0n]
Lasiommata megera|EULEP1294-15|RVcoll.14-F724||Greece|658[0n]
Lasiommata megera|EULEP1431-15|RVcoll.14-G114||Greece|658[0n]
Lasiommata megera|EULEP1439-15|RVcoll.14-G131||Greece|658[0n]
Lasiommata megera|EULEP1911-15|RVcoll.14-B808||Bosnia and Herzegovina|658[0n]
Lasiommata megera|WMB6204-18|RVcoll17A707||Croatia|658[0n]
Lasiommata megera|WMB6203-18|RVcoll17A671||Croatia|658[0n]
Lasiommata megera|WMB6202-18|RVcoll17A636||Croatia|658[0n]
Lasiommata megera|WMB6201-18|RVcoll17A427||Croatia|658[0n]
Lasiommata megera|WMB6200-18|RVcoll17A403||Croatia|658[0n]
Lasiommata megera|WMB6199-18|RVcoll17A401||Croatia|658[0n]
Lasiommata megera|WMB6198-18|RVcoll14F490||Bulgaria|658[0n]
Lasiommata megera|WMB6142-18|RVcoll12M168|Crete|Greece|658[0n]
Lasiommata megera|WMB6141-18|RVcoll12M136|Crete|Greece|658[0n]
Lasiommata megera|WMB6140-18|RVcoll12M135|Crete|Greece|658[0n]
Lasiommata megera|WMB6135-18|RVcoll11J517|Karpathos|Greece|658[0n]
Lasiommata megera|WMB6134-18|RVcoll11J509|Karpathos|Greece|658[0n]
Lasiommata megera|WMB6099-18|RVcoll10B454|Genit. examined|Croatia|658[0n]
Lasiommata megera|WMB6097-18|RVcoll09X799||Greece|658[0n]
Lasiommata megera|WMB6096-18|RVcoll09X798||Macedonia|658[0n]
Lasiommata megera|WMB6175-18|RVcoll12R914||Greece|658[0n]
Lasiommata megera|WMB6174-18|RVcoll12R913||Greece|658[0n]
Lasiommata megera|WMB6173-18|RVcoll12R912||Greece|658[0n]
Lasiommata megera|WMB6167-18|RVcoll12R251||Greece|658[0n]
Lasiommata megera|WMB6166-18|RVcoll12R250||Greece|658[0n]
Lasiommata megera|WMB6164-18|RVcoll12R244||Greece|658[0n]
Lasiommata megera|WMB6150-18|RVcoll12N802|Lesvos|Greece|658[0n]
Lasiommata megera|WMB6147-18|RVcoll12M248|Crete|Greece|658[0n]
Lasiommata megera|WMB6146-18|RVcoll12M203|Crete|Greece|658[0n]
Lasiommata megera|WMB6145-18|RVcoll12M202|Crete|Greece|658[0n]
Lasiommata megera|WMB6144-18|RVcoll12M201|Crete|Greece|658[0n]
Lasiommata megera|WMB6143-18|RVcoll12M181|Crete|Greece|658[0n]
Lasiommata megera|LEFIL142-10|MM19142|Crete|Greece|658[0n]
Lasiommata megera|WMB6148-18|RVcoll12N642|Menorca|Spain|658[0n]
Lasiommata megera|EZSPM433-09|Rvcoll.09-X118||Spain|658[0n]
Lasiommata megera|EZSPM1000-12|Rvcoll.12-L886||Spain|658[0n]
Lasiommata megera|EZSPM041-09|RVcoll.08-P249||Spain|658[0n]
Lasiommata megera|WMB2302-13|RVcoll.11-E126|Gozo. Genit. examined|Malta|658[0n]
Lasiommata megera|WMB1139-13|RVcoll.11-E129|Gozo. Genit. examined|Malta|658[0n]
Lasiommata megera|WMB5100-14|RVcoll. 14-L231||Italy|658[0n]
Lasiommata megera|WMB6220-18|RVcollLD1577||Italy|658[0n]
Lasiommata megera|WMB4974-14|RVcoll. 14-I521||Italy|658[0n]
Lasiommata megera|WMB5945-17|RVcoll12M392|Sicily|Italy|658[0n]
Lasiommata megera|WMB5932-17|RVcoll11H720|Levanzo|Italy|658[0n]
Lasiommata megera|BIBSA1021-15|15-C164||Italy|658[0n]
Lasiommata megera|WMB4902-14|RVcoll.14-I449||Italy|658[0n]
Lasiommata megera|WMB6237-18|RVcollLD2185|Elba|Italy|658[0n]
Lasiommata megera|WMB6225-18|RVcollLD1634||Italy|658[0n]
Lasiommata megera|WMB5979-17|RVcollLD1608|Lipari|Italy|658[0n]
Lasiommata megera|WMB5941-17|RVcoll11H915|Salina. Genit. examined|Italy|658[0n]
Lasiommata megera|WMB5934-17|RVcoll11H853|Vulcano|Italy|658[0n]
Lasiommata megera|WMB5990-17|RVcollLD2052|Ustica|Italy|658[0n]
Lasiommata megera|WMB5921-17|RVcoll11D193|Marettimo. Genit. examined|Italy|658[0n]
Lasiommata megera|WMB5933-17|RVcoll11H852|Vulcano|Italy|658[0n]
Lasiommata megera|WMB5935-17|RVcoll11H909|Salina|Italy|658[0n]
Lasiommata megera|WMB5936-17|RVcoll11H910|Salina. Genit. examined|Italy|658[0n]
Lasiommata megera|WMB5937-17|RVcoll11H911|Salina. Genit. examined|Italy|658[0n]
Lasiommata megera|WMB5938-17|RVcoll11H912|Salina. Genit. examined|Italy|658[0n]
Lasiommata megera|WMB5939-17|RVcoll11H913|Salina. Genit. examined|Italy|658[0n]
Lasiommata megera|WMB5940-17|RVcoll11H914|Salina. Genit. examined|Italy|658[0n]
Lasiommata megera|WMB5944-17|RVcoll12M391|Sicily|Italy|658[0n]
Lasiommata megera|WMB5958-17|RVcoll14A784||Italy|658[0n]
Lasiommata megera|WMB5978-17|RVcollLD0204|Sicily|Italy|658[0n]
Lasiommata megera|WMB5980-17|RVcollLD1611|Lipari|Italy|658[0n]
Lasiommata megera|WMB5981-17|RVcollLD1670|Stromboli|Italy|658[0n]
Lasiommata megera|WMB5982-17|RVcollLD1673|Stromboli|Italy|658[0n]
Lasiommata megera|WMB5983-17|RVcollLD1884||Italy|658[0n]
Lasiommata megera|WMB5984-17|RVcollLD1885||Italy|658[0n]
Lasiommata megera|WMB5985-17|RVcollLD1988|Malta|Malta|658[0n]
Lasiommata megera|WMB5986-17|RVcollLD1989|Malta|Malta|658[0n]
Lasiommata megera|WMB5987-17|RVcollLD1990|Malta|Malta|658[0n]
Lasiommata megera|WMB5988-17|RVcollLD2050|Ustica|Italy|658[0n]
Lasiommata megera|WMB5989-17|RVcollLD2051|Ustica|Italy|658[0n]
Lasiommata megera|WMB5991-17|RVcollLD2081|Sicily|Italy|658[0n]
Lasiommata megera|WMB4297-14|RVcoll.14-A393||Italy|658[0n]
Lasiommata megera|WMB6178-18|RVcoll12Z339|Ponza|Italy|658[0n]
Lasiommata megera|WMB6179-18|RVcoll13S541|San Domino|Italy|658[0n]
Lasiommata megera|WMB6180-18|RVcoll13S543|San Domino|Italy|658[0n]
Lasiommata megera|WMB6181-18|RVcoll13S544|San Domino|Italy|658[0n]
Lasiommata megera|WMB6182-18|RVcoll13S671||Italy|658[0n]
Lasiommata megera|WMB6183-18|RVcoll13S672||Italy|658[0n]
Lasiommata megera|WMB6188-18|RVcoll13T821||Italy|658[0n]
Lasiommata megera|WMB6191-18|RVcoll14A380||Italy|658[0n]
Lasiommata megera|WMB6192-18|RVcoll14A581||Italy|658[0n]
Lasiommata megera|WMB6194-18|RVcoll14D502||Italy|658[0n]

[illegible]
Lasiommata megera|WMB6191-18|RVcoll14A380||Italy|658[0n]
Lasiommata megera|WMB6192-18|RVcoll14A581||Italy|658[0n]
Lasiommata megera|WMB6194-18|RVcoll14D502||Italy|658[0n]
Lasiommata megera|WMB4735-14|RVcoll.LD-3284|Ischia|Italy|658[0n]
Lasiommata megera|WMB6103-18|RVcoll10C436|Elba|Italy|658[0n]
Lasiommata megera|WMB6104-18|RVcoll10C503||Italy|658[0n]
Lasiommata megera|WMB6124-18|RVcoll11I316|Pianosa|Italy|658[0n]
Lasiommata megera|WMB6125-18|RVcoll11I317|Pianosa|Italy|658[0n]
Lasiommata megera|WMB6126-18|RVcoll11I318|Pianosa|Italy|658[0n]
Lasiommata megera|WMB6127-18|RVcoll11I319|Pianosa|Italy|658[0n]
Lasiommata megera|WMB6195-18|RVcoll14D504||Italy|658[0n]
Lasiommata megera|WMB6196-18|RVcoll14D505||Italy|658[0n]
Lasiommata megera|WMB6197-18|RVcoll14D506||Italy|658[0n]
Lasiommata megera|WMB6213-18|RVcollLD1084|Ponza|Italy|658[0n]
Lasiommata megera|WMB6214-18|RVcollLD1085|Ponza|Italy|658[0n]
Lasiommata megera|WMB6215-18|RVcollLD1086|Ponza|Italy|658[0n]
Lasiommata megera|WMB6218-18|RVcollLD1575|Argentario|Italy|658[0n]
Lasiommata megera|WMB6219-18|RVcollLD1576||Italy|658[0n]
Lasiommata megera|WMB6221-18|RVcollLD1578||Italy|658[0n]
Lasiommata megera|WMB6222-18|RVcollLD1594|Capri|Italy|658[0n]
Lasiommata megera|WMB6223-18|RVcollLD1595|Capri|Italy|658[0n]
Lasiommata megera|WMB6224-18|RVcollLD1596|Capri|Italy|658[0n]
Lasiommata megera|WMB6226-18|RVcollLD1776|Giglio|Italy|658[0n]
Lasiommata megera|WMB6227-18|RVcollLD1784|Giglio|Italy|658[0n]
Lasiommata megera|WMB6228-18|RVcollLD1787||Italy|658[0n]
Lasiommata megera|WMB6230-18|RVcollLD1888|Ventotene|Italy|658[0n]
Lasiommata megera|WMB6231-18|RVcollLD1900|Ischia|Italy|658[0n]
Lasiommata megera|WMB6232-18|RVcollLD1901|Ischia|Italy|658[0n]
Lasiommata megera|WMB6236-18|RVcollLD2183|Elba|Italy|658[0n]
Lasiommata megera|WMB6242-18|RVcollLD2347|Gorgona|Italy|658[0n]
Lasiommata megera|WMB6243-18|RVcollLD2348|Gorgona|Italy|658[0n]
Lasiommata megera|WMB6244-18|RVcollLD2349|Gorgona|Italy|658[0n]
Lasiommata megera|WMB6245-18|RVcollLD2350|Gorgona|Italy|658[0n]
Lasiommata megera|WMB5069-14|RVcoll. 14-L200||Italy|658[0n]
Lasiommata megera|WMB5070-14|RVcoll. 14-L201||Italy|658[0n]
Lasiommata megera|WMB4763-14|RVcoll. 14-I310||Italy|658[0n]
Lasiommata megera|WMB4774-14|RVcoll. 14-I321||Italy|658[0n]
Lasiommata megera|WMB4917-14|RVcoll.14-I464||Italy|658[0n]
Lasiommata megera|WMB5056-14|RVcoll. 14-L187||Italy|658[0n]
Lasiommata megera|WMB5059-14|RVcoll. 14-L190||Italy|658[0n]
Lasiommata megera|WMB5065-14|RVcoll. 14-L196||Italy|658[0n]
Lasiommata megera|WMB5068-14|RVcoll. 14-L199||Italy|658[0n]
Lasiommata megera|WMB5096-14|RVcoll. 14-L227||Italy|658[0n]
Lasiommata megera|WMB5168-14|RVcoll.13-S509||Italy|658[0n]
Lasiommata megera|WMB5203-14|RVcoll.13-T932||Italy|658[0n]
Lasiommata megera|WMB5480-14|RVcoll.14-N705|Alicudi|Italy|658[0n]
Lasiommata megera|BIBSA1611-16|15-C372||Italy|658[0n]
Lasiommata megera|BIBSA1032-15|15-C322||Italy|658[0n]
Lasiommata megera|BIBSA1782-16|16-A513||Italy|658[0n]
Lasiommata megera|BIBSA576-15|LEP-SS-00196||Italy|658[0n]
Lasiommata megera|BIBSA1388-15|15-M984||Italy|658[0n]
Lasiommata megera|BIBSA1397-15|15-N001||Italy|658[0n]
Lasiommata megera|BIBSA712-15|RVcoll. 14-A704||Italy|658[0n]
Lasiommata megera|BIBSA1547-16|11-D356||Italy|658[0n]
Lasiommata megera|BIBSA502-15|LEP-SS-00122||Italy|658[0n]
Lasiommata megera|BIBSA503-15|LEP-SS-00123||Italy|658[0n]
Lasiommata megera|BIBSA1858-17|RVcoll16C743||Italy|658[0n]
Lasiommata megera|BIBSA336-15|RVcoll.14-I004||Italy|658[0n]
Lasiommata megera|BIBSA1652-16|15-C651||Italy|658[0n]
Lasiommata megera|OXB865-15|15-A509||Italy|658[0n]
Lasiommata megera|OXB872-15|15-A514||Italy|658[0n]
Lasiommata megera|OXB1126-15|15-A659||Italy|658[0n]
Lasiommata megera|OXB681-15|12-M339|Sicily E|Italy|658[0n]
Lasiommata megera|OXB918-15|15-A562||Italy|658[0n]
Lasiommata megera|OXB838-15|14-U801||Italy|658[0n]
Lasiommata megera|OXB997-15|15-A947||Italy|658[0n]
Lasiommata megera|OXB1032-15|LD-2133||Italy|658[0n]
Lasiommata megera|OXB1011-15|15-A961||Italy|658[0n]
Lasiommata megera|OXB1064-15|14-A842||Italy|658[0n]
Lasiommata megera|OXB1172-15|Rvcoll_16-A059||Italy|658[0n]
Lasiommata megera|OXB1071-15|15-A604||Italy|643[2n]
Lasiommata megera|OXB676-15|11-D355|Sicily E|Italy|620[0n]
Lasiommata megera|WMB5931-17|RVcoll11H719|Levanzo|Italy|641[0n]
Lasiommata megera|WMB5930-17|RVcoll11H718|Levanzo|Italy|658[0n]
Lasiommata megera|WMB5924-17|RVcoll11D196|Marettimo. Genit. examined|Italy|658[0n]
Lasiommata megera|WMB5923-17|RVcoll11D195|Marettimo. Genit. examined|Italy|658[0n]
Lasiommata megera|WMB5922-17|RVcoll11D194|Marettimo. Genit. examined|Italy|658[0n]
Lasiommata megera|WMB5920-17|RVcoll11D192|Marettimo. Genit. examined|Italy|658[0n]
Lasiommata megera|WMB5919-17|RVcoll11D191|Marettimo. Genit. examined|Italy|658[0n]
Lasiommata megera|WMB5915-17|RVcoll11D031|Sicily|Italy|658[0n]
Lasiommata megera|WMB5914-17|RVcoll09T556|Lipari|Italy|658[0n]
Lasiommata megera|WMB2407-13|RVcoll.10-C438|Elba|Italy|658[0n]
Lasiommata megera|WMB2346-13|RVcoll.12-M494||Italy|658[0n]
Lasiommata megera|WMB2372-13|RVcoll.LD-1987||Malta|658[0n]
Lasiommata megera|WMB1138-13|RVcoll.11-E128|Gozo. Genit. examined|Malta|658[0n]
Lasiommata megera|WMB1137-13|RVcoll.11-E127|Gozo. Genit. examined|Malta|658[0n]
Lasiommata megera|WMB2792-13|RVcoll.11-J628||Italy|658[0n]
Lasiommata megera|WMB2973-14|RVcoll.12-O065|Argentario|Italy|658[0n]
Lasiommata megera|WMB2972-14|RVcoll.12-O064|Argentario|Italy|658[0n]
Lasiommata megera|WMB2971-14|RVcoll.12-O063|Argentario|Italy|658[0n]
Lasiommata megera|WMB2882-14|RVcoll.10-C772|Giglio|Italy|658[0n]
Lasiommata megera|WMB040-11|RVcoll.LD-1636||Italy|658[0n]
Lasiommata megera|WMB182-11|RVcoll.11-D482||Italy|658[0n]
Lasiommata megera|WMB177-11|RVcoll.11-D446|Sicily|Italy|658[0n]
Lasiommata megera|WMB159-11|RVcoll.11-D321|Sicily|Italy|658[0n]
Lasiommata megera|WMB146-11|RVcoll.11-D197|Marettimo|Italy|658[0n]
Lasiommata megera|GWORZ033-10|BC ZSM Lep 30389||Italy|658[0n]
Lasiommata megera|GBLAD216-14|BC ZSM Lep 78787||Germany|658[0n]
Lasiommata megera|EZSPN610-09|RVcoll.08-J330|Genit. examined|Spain|658[0n]
Lasiommata megera|WMB6235-18|RVcollLD1952|Ibiza|Spain|658[0n]
Lasiommata megera|LON969-12|NHMO Lep2011.029||Norway|658[0n]
Lasiommata megera|LON958-12|NHMO Lep2011.018||Norway|658[0n]

Lasiommata megera|WMB6235-18|RVcollLD1952|Ibiza|Spain|658[0n]
Lasiommata megera|LON969-12|NHMO Lep2011.029||Norway|658[0n]
Lasiommata megera|LON958-12|NHMO Lep2011.018||Norway|658[0n]
Lasiommata megera|LON186-08|NHMO-06187||Norway|657[0n]
Lasiommata megera|WMB6261-18|RVcollLD3143||Italy|658[0n]
Lasiommata megera|WMB6159-18|RVcoll12P558||France|658[0n]
Lasiommata megera|WMB3547-14|RVcoll.11-D872||Spain|658[0n]
Lasiommata megera|EZSPN1122-11|Rvcoll.10-C352||Spain|658[0n]
Lasiommata megera|EZROM729-08|RV-07-C005||Spain|658[0n]
Lasiommata megera|OXB578-15|14-N967||Italy|658[0n]
Lasiommata megera|BIBSA1136-15|15-L949||Italy|658[0n]
Lasiommata megera|WMB675-12|RVcoll.10-C139||France|658[0n]
Lasiommata megera|LEASS903-17|KLM Lep 08408||Austria|658[0n]
Lasiommata megera|WMB5003-14|RVcoll. 14-I550||Italy|658[0n]
Lasiommata megera|EZSPN368-09|RVcoll.08-H393||Spain|658[0n]
Lasiommata megera|EZROM633-08|RV-07-D159||Romania|658[0n]
Lasiommata megera|WMB6120-18|RVcoll11H222||Germany|658[0n]
Lasiommata megera|EZSPM208-09|RVcoll.08-R326||Spain|658[0n]
Lasiommata megera|WMB5297-14|RVcoll.14-I597||France|658[0n]
Lasiommata megera|EZROM235-08|RV-07-D216||Romania|658[2n]
Lasiommata megera|OXB513-15|OXB-TGS-743||United Kingdom|658[0n]
Lasiommata megera|ABOLD064-16|TLMF Lep 21142||Austria|658[0n]
Lasiommata megera|LEATI046-15|TLMF Lep 17431||Austria|658[0n]
Lasiommata megera|LEFIL143-10|MM19143||Hungary|658[0n]
Lasiommata megera|FBLMT903-09|BC ZSM Lep 25463||Germany|658[0n]
Lasiommata megera|EZRMN006-08|RVcoll.07-D067|Genit. examined|Romania|658[0n]
Lasiommata megera|EZRMN007-08|RVcoll.08-M403|Genit. examined|Romania|658[0n]
Lasiommata megera|EZSPC466-09|RVcoll.08-J343|Genit. examined|Spain|658[0n]
Lasiommata megera|EZSPN714-09|RVcoll.08-L080||Spain|658[0n]
Lasiommata megera|EZROM233-08|RV-06-K633|Genit. examined|Romania|658[0n]
Lasiommata megera|EZROM234-08|RV-06-V655|Genit. examined|Romania|658[0n]
Lasiommata megera|EZROM236-08|RV-07-C960||Romania|658[0n]
Lasiommata megera|EZSPN085-09|RVcoll.06-A004|Genit. examined|Spain|658[0n]
Lasiommata megera|EZSPC464-09|RVcoll.08-J366|Genit. examined|Spain|658[0n]
Lasiommata megera|EZSPC467-09|RVcoll.08-L427|Genit. examined|Spain|658[0n]
Lasiommata megera|EZSPN816-09|RVcoll.08-L621||Spain|658[0n]
Lasiommata megera|EZSPN353-09|RVcoll.08-H324||Spain|658[0n]
Lasiommata megera|EZSPM1019-12|Rvcoll.12-M768||Spain|658[0n]
Lasiommata megera|EZSPN499-09|RVcoll.08-H948||Spain|658[0n]
Lasiommata megera|EZSPN600-09|RVcoll.08-J191||Portugal|658[0n]
Lasiommata megera|EZSPM036-09|RVcoll.08-P239||Spain|658[0n]
Lasiommata megera|EZSPM065-09|RVcoll.08-P298||Spain|658[0n]
Lasiommata megera|EZSPM211-09|RVcoll.08-R332||Spain|658[0n]
Lasiommata megera|EZSPM925-12|Rvcoll.12-M648||Spain|658[0n]
Lasiommata megera|EZSPM926-12|Rvcoll.12-M649||Spain|658[0n]
Lasiommata megera|WMB2867-14|RVcoll.10-C552|Ibiza|Spain|658[0n]
Lasiommata megera|WMB098-11|RVcoll.10-B605||France|658[0n]
Lasiommata megera|WMB3388-14|RVcoll.09-V477||Spain|658[0n]
Lasiommata megera|WMB3396-14|RVcoll.09-V581|Genit. examined|Spain|658[0n]
Lasiommata megera|WMB2938-14|RVcoll.12-L528|Ibiza|Spain|658[0n]
Lasiommata megera|WMB3022-14|RVcoll.13-S338|Mallorca|Spain|658[0n]
Lasiommata megera|WMB3166-14|RVcoll.08-H476|Genit. examined|Spain|658[0n]
Lasiommata megera|WMB3225-14|RVcoll.08-J764||Spain|658[0n]
Lasiommata megera|WMB3232-14|RVcoll.08-J881||Spain|658[0n]
Lasiommata megera|WMB3555-14|RVcoll.11-D898||Spain|658[0n]
Lasiommata megera|WMB3565-14|RVcoll.11-D968||Spain|658[0n]
Lasiommata megera|WMB3573-14|RVcoll.11-D991||Spain|658[0n]
Lasiommata megera|WMB3796-14|RVcoll.12-O608||France|658[0n]
Lasiommata megera|WMB878-13|RVcoll.09-X280||France|658[0n]
Lasiommata megera|WMB1758-13|RVcoll.12-P985|Levant|France|658[0n]
Lasiommata megera|WMB1759-13|RVcoll.12-P986|Levant|France|658[0n]
Lasiommata megera|WMB4369-14|RVcoll.14-B116||Portugal|658[0n]
Lasiommata megera|WMB6157-18|RVcoll12P316||France|658[0n]
Lasiommata megera|WMB6158-18|RVcoll12P390||France|658[0n]
Lasiommata megera|WMB6160-18|RVcoll12P751||France|658[0n]
Lasiommata megera|WMB6161-18|RVcoll12P752|Levant|France|658[0n]
Lasiommata megera|WMB6162-18|RVcoll12P753||France|658[0n]
Lasiommata megera|WMB6184-18|RVcoll13T323||Italy|658[0n]
Lasiommata megera|WMB6098-18|RVcoll10A940||Bulgaria|658[0n]
Lasiommata megera|WMB6102-18|RVcoll10C360||Spain|658[0n]
Lasiommata megera|WMB6111-18|RVcoll11D570||Sweden|658[0n]
Lasiommata megera|WMB6112-18|RVcoll11D614|Genit. examined|Spain|658[0n]
Lasiommata megera|WMB6121-18|RVcoll11H235||Germany|658[0n]
Lasiommata megera|WMB6129-18|RVcoll11I505|Genit. examined|Spain|658[0n]
Lasiommata megera|WMB6132-18|RVcoll11J143||Switzerland|658[0n]
Lasiommata megera|WMB6133-18|RVcoll11J144||Switzerland|658[0n]
Lasiommata megera|WMB6136-18|RVcoll11J782||Sweden|658[0n]
Lasiommata megera|WMB6209-18|RVcollLD0652||Italy|658[0n]
Lasiommata megera|WMB6210-18|RVcollLD0655||Italy|658[0n]
Lasiommata megera|WMB6233-18|RVcollLD1936|Ibiza|Spain|658[0n]
Lasiommata megera|WMB6234-18|RVcollLD1938|Ibiza|Spain|658[0n]
Lasiommata megera|WMB6260-18|RVcollLD3142||Italy|658[0n]
Lasiommata megera|WMB674-12|RVcoll.10-A656||France|658[0n]
Lasiommata megera|WMB5358-14|RVcoll.14-J701||France|658[0n]
Lasiommata megera|WMB5000-14|RVcoll. 14-I547||Italy|658[0n]
Lasiommata megera|WMB5011-14|RVcoll. 14-I558||Italy|658[0n]
Lasiommata megera|EULEP4125-16|RVcoll15J139||Switzerland|658[0n]
Lasiommata megera|EULEP5024-16|RVcoll16I952||Slovakia|658[0n]
Lasiommata megera|EULEP5002-16|RVcoll16I800||Germany|658[0n]
Lasiommata megera|EULEP181-14|RVcoll.11-J413||Romania|658[0n]
Lasiommata megera|EULEP5187-17|RVcoll16I438||Poland|658[0n]
Lasiommata megera|EULEP2147-15|RVcoll.14-K027||Switzerland|658[0n]
Lasiommata megera|EULEP2413-15|RVcoll.14-V188||Belgium|658[0n]
Lasiommata megera|BIBSA1133-15|15-L944||Italy|658[0n]
Lasiommata megera|BIBSA1173-15|15-M202||Italy|658[0n]
Lasiommata megera|BIBSA1248-15|15-F827||France|658[0n]
Lasiommata megera|GBLAA018-14|BC ZSM Lep 80679||Germany|658[0n]
Lasiommata megera|GBLAB148-13|BC ZSM Lep 75774||Germany|658[0n]
Lasiommata megera|GBLAA1339-15|BC ZSM Lep 87035||Germany|658[0n]
Lasiommata megera|LEASS694-17|TLMF Lep 22342||Austria|658[0n]
Lasiommata megera|OXB499-15|OXB-TGS-665||United Kingdom|658[0n]
Lasiommata megera|OXB500-15|OXB-TGS-666||United Kingdom|658[0n]
Lasiommata megera|OXB514-15|OXB-TGS-744||United Kingdom|658[0n]

[illegible]
Lasiommata megera|OXB499-15|OXB-TGS-665||United Kingdom|658[0n]
Lasiommata megera|OXB500-15|OXB-TGS-666||United Kingdom|658[0n]
Lasiommata megera|OXB514-15|OXB-TGS-744||United Kingdom|658[0n]
Lasiommata megera|OXB524-15|OXB-TGS-843||United Kingdom|658[0n]
Lasiommata megera|OXB525-15|OXB-TGS-844||United Kingdom|658[0n]
Lasiommata megera|OXB526-15|OXB-TGS-845||United Kingdom|658[0n]
Lasiommata megera|OXB532-15|OXB-TGS-887||United Kingdom|658[0n]
Lasiommata megera|WMB3579-14|RVcoll.11-E021|Genit. examined|Spain|614[0n]
Lasiommata megera|EZSPN860-09|RVcoll.08-L723||Spain|623[0n]
Lasiommata megera|EZSPC465-09|RVcoll.08-J348||Spain|633[0n]
Lasiommata megera|LEATH754-14|TLMF Lep 15966||Italy|634[0n]
Lasiommata megera|LEATH755-14|TLMF Lep 15967||Italy|634[0n]
Lasiommata megera|LEATI045-15|TLMF Lep 17430||Austria|658[0n]
Lasiommata megera|PHLAW008-13|TLMF Lep 09805||Austria|658[0n]
Lasiommata petropolitana|EULEP904-15|RVcoll.14-C743||Bulgaria|658[0n]
Lasiommata petropolitana|EZSPC864-10|RVcoll.09-T078||Spain|658[0n]
Lasiommata petropolitana|EZSPN1038-11|Rvcoll.07-W120||Spain|658[0n]
Lasiommata petropolitana|EZSPN246-09|RVcoll.07-W119||Spain|658[0n]
Lasiommata petropolitana|EZSPN245-09|RVcoll.07-W118||Spain|658[0n]
Lasiommata petropolitana|EZSPN244-09|RVcoll.07-W116||Spain|658[1n]
Lasiommata petropolitana|EULEP1936-15|RVcoll.14-B908||Bosnia and Herzegovina|658[0n]
Lasiommata petropolitana|OXB318-15|RVcoll.14-N032||Italy|658[0n]
Lasiommata petropolitana|PHLAB291-10|TLMF Lep 01091||Switzerland|658[0n]
Lasiommata petropolitana|LEATD290-13|TLMF Lep 12937||Italy|658[0n]
Lasiommata petropolitana|PHLAI489-13|TLMF Lep 09051||Austria|658[0n]
Lasiommata petropolitana|PHLAB290-10|TLMF Lep 01090||Switzerland|658[0n]
Lasiommata petropolitana|LEATC577-13|TLMF Lep 12559||Austria|658[0n]
Lasiommata petropolitana|LEFIB177-10|MM00591||Finland|658[0n]
Lasiommata petropolitana|LEFID485-10|MM06429||Finland|658[0n]
Lasiommata petropolitana|LEFIJ564-10|MM17189||Finland|658[0n]
Lasiommata petropolitana|WMB6168-18|RVcoll12R274||Greece|658[0n]
Lasiommata petropolitana|WMB6169-18|RVcoll12R275||Greece|658[0n]
Lasiommata petropolitana|WMB6170-18|RVcoll12R276||Greece|658[0n]
Lasiommata petropolitana|WMB6185-18|RVcoll13T325||Italy|658[0n]
Lasiommata petropolitana|WMB6095-18|RVcoll08L308||Sweden|658[0n]
Lasiommata petropolitana|WMB6107-18|RVcoll11D561||Russia|658[0n]
Lasiommata petropolitana|WMB6108-18|RVcoll11D562||Russia|658[0n]
Lasiommata petropolitana|EULEP4126-16|RVcoll15G558||Switzerland|658[0n]
Lasiommata petropolitana|EULEP4758-16|RVcoll16G990||Sweden|658[0n]
Lasiommata petropolitana|EULEP2472-15|RVcoll.14-V299||Switzerland|658[0n]
Lasiommata petropolitana|EULEP1933-15|RVcoll.14-B903||Bosnia and Herzegovina|658[0n]
Lasiommata petropolitana|LEASS749-17|TLMF Lep 22397||Austria|658[0n]
Lasiommata petropolitana|PHLAB347-10|TLMF Lep 01147||Switzerland|627[0n]
Lasiommata petropolitana|GWOSK896-11|BC ZSM Lep 49397||Germany|635[0n]
Lasiommata petropolitana|GWORA2480-09|BC ZSM Lep 30692||Germany|658[0n]
Lasiommata petropolitana|GWOTF673-12|BC ZSM Lep 62379||Germany|658[0n]
Lasiommata petropolitana|LEATG011-14|TLMF Lep 13798||Austria|658[0n]
Lasiommata petropolitana|PHLAH458-12|TLMF Lep 08277||Austria|658[0n]
Pararge aegeria|WMB6006-17|RVcollLD1080|Ponza|Italy|658[0n]
Pararge aegeria|WMB2266-13|RVcoll.12-Z337|Ponza|Italy|658[0n]
Pararge aegeria|WMB1166-13|RVcoll.11-E652|Sardinia|Italy|658[0n]
Pararge aegeria|OXB811-15|14-J278|Sardinia|Italy|658[0n]
Pararge aegeria|OXB806-15|14-J273|Sardinia|Italy|658[0n]
Pararge aegeria|OXB801-15|14-J268|Sardinia|Italy|658[0n]
Pararge aegeria|WMB326-11|RVcoll.11-E619|Santa Maria|Italy|658[0n]
Pararge aegeria|WMB6005-17|RVcollLD1076|Ponza|Italy|658[0n]
Pararge aegeria|WMB1543-13|RVcoll.12-O300|La Maddalena|Italy|658[0n]
Pararge aegeria|WMB3055-14|RVcoll.13-T684|Sardinia|Italy|658[0n]
Pararge aegeria|WMB3004-14|RVcoll.12-O442|Sant Antioco|Italy|658[0n]
Pararge aegeria|WMB3001-14|RVcoll.12-O422|San Pietro|Italy|658[0n]
Pararge aegeria|OXB798-15|14-J264|Sardinia|Italy|658[0n]
Pararge aegeria|WMB2826-13|RVcoll.12-O441|Sardinia|Italy|658[0n]
Pararge aegeria|OXB799-15|14-J266|Sardinia|Italy|658[0n]
Pararge aegeria|OXB807-15|14-J274|Sardinia|Italy|658[0n]
Pararge aegeria|BIBSA1320-15|12-O549|Sardinia|Italy|621[0n]
Pararge aegeria|BIBSA1299-15|LD-1992|Sardinia|Italy|658[0n]
Pararge aegeria|WMB4729-14|RVcoll.LD-3113|Asinara|Italy|658[0n]
Pararge aegeria|WMB3000-14|RVcoll.12-O421|San Pietro|Italy|658[0n]
Pararge aegeria|WMB2989-14|RVcoll.12-O295|La Maddalena|Italy|658[0n]
Pararge aegeria|OXB805-15|14-J272|Sardinia|Italy|658[0n]
Pararge aegeria|WMB2988-14|RVcoll.12-O294|La Maddalena|Italy|658[0n]
Pararge aegeria|OXB794-15|14-J218|Corsica|France|658[0n]
Pararge aegeria|BIBSA1288-15|13-T691|Sardinia|Italy|658[0n]
Pararge aegeria|WMB1585-13|RVcoll.12-O571|Sardinia|Italy|658[0n]
Pararge aegeria|OXB637-15|08-H115||Spain|658[0n]
Pararge aegeria|WMB2897-14|RVcoll.11-E613|Sardinia. Genit. examined|Italy|658[0n]
Pararge aegeria|WMB297-11|RVcoll.11-E313||Italy|658[0n]
Pararge aegeria|OXB800-15|14-J267|Sardinia|Italy|658[0n]
Pararge aegeria|OXB802-15|14-J269|Sardinia|Italy|658[0n]
Pararge aegeria|OXB803-15|14-J270|La Maddalena|Italy|658[0n]
Pararge aegeria|OXB804-15|14-J271|Sardinia|Italy|658[0n]
Pararge aegeria|OXB816-15|14-J283|Sardinia|Italy|658[0n]
Pararge aegeria|WMB1563-13|RVcoll.12-O431|San Pietro|Italy|623[0n]
Pararge aegeria|WMB2853-14|RVcoll.09-T518|Corsica|France|619[0n]
Pararge aegeria|EZSPC798-10|RVcoll.08-P307|Genit. examined|Spain|658[0n]
Pararge aegeria|EZSPC796-10|RVcoll.08-P262||Spain|658[0n]
Pararge aegeria|EZSPM064-09|RVcoll.08-P296|Genit. examined|Spain|658[0n]
Pararge aegeria|EZSPM053-09|RVcoll.08-P273|Genit. examined|Spain|658[0n]
Pararge aegeria|EZSPN323-09|RVcoll.08-H185||Spain|658[0n]
Pararge aegeria|EZSPN301-09|RVcoll.08-H106||Spain|658[0n]
Pararge aegeria|EULEP4371-16|RVcoll14A931||Cyprus|658[0n]
Pararge aegeria|LOWA761-06|2005-LOWA-761||Russia|653[0n]
Pararge aegeria|EZRMN156-08|RVcoll.08-M445||Romania|658[0n]
Pararge aegeria|OXB636-15|06-K533|Genit. examined|Romania|658[0n]
Pararge aegeria|EULEP2495-15|RVcoll.14-V353||Ukraine|658[0n]
Pararge aegeria|EULEP5041-16|RVcoll16J029||Slovakia|658[0n]
Pararge aegeria|EZROM454-08|RV-07-D220|Genit. examined|Romania|658[0n]
Pararge aegeria|EZROM452-08|RV-06-K524|Genit. examined|Romania|658[0n]
Pararge aegeria|EZRMN154-08|RVcoll.08-M232||Romania|658[0n]
Pararge aegeria|WMB2419-13|RVcoll.10-C677|Lipari|Italy|658[0n]
Pararge aegeria|BIBSA1109-15|15-L812||Italy|658[0n]
Pararge aegeria|OXB962-15|15-A912||Italy|658[0n]
[illegible]

Pararge aegeria|WMB2419-13|RVcoll.10-C677|Lipari|Italy|658[0n]
Pararge aegeria|BIBSA1109-15|15-L812||Italy|658[0n]
Pararge aegeria|OXB962-15|15-A912||Italy|658[0n]
Pararge aegeria|EULEP4370-16|RVcoll15J153||Switzerland|658[0n]
Pararge aegeria|BIBSA1599-16|12-R099||Italy|658[0n]
Pararge aegeria|EZROM455-08|RV-07-C975||Romania|658[0n]
Pararge aegeria|WMB2341-13|RVcoll.12-M476||Italy|658[0n]
Pararge aegeria|OXB1267-15|Rvcoll_15-M126||France|658[0n]
Pararge aegeria|OXB1069-15|09-X099||Greece|658[0n]
Pararge aegeria|EULEP695-15|RVcoll.12-M109|Crete|Greece|658[0n]
Pararge aegeria|OXB817-15|14-J284|Corsica|France|658[0n]
Pararge aegeria|OXB810-15|14-J277|Corsica|France|658[0n]
Pararge aegeria|OXB791-15|14-I966|Corsica|France|658[0n]
Pararge aegeria|WMB367-11|RVcoll.11-E975||France|658[0n]
Pararge aegeria|WMB3095-14|RVcoll.LD-2227|Capraia|Italy|658[0n]
Pararge aegeria|EULEP4372-16|RVcoll15P013||Belarus|658[0n]
Pararge aegeria|PHLAW025-13|TLMF Lep 09822||Austria|658[0n]
Pararge aegeria|LEATG002-14|TLMF Lep 13789||Austria|658[0n]
Pararge aegeria|LEATG004-14|TLMF Lep 13791||Austria|658[0n]
Pararge aegeria|LEATF459-14|TLMF Lep 13771||Italy|658[0n]
Pararge aegeria|ABOLD408-16|TLMF Lep 21560||Austria|658[0n]
Pararge aegeria|GWORT461-10|BC ZSM Lep 32147||Germany|658[0n]
Pararge aegeria|GWORZ032-10|BC ZSM Lep 30388||Italy|658[0n]
Pararge aegeria|LEFIL395-10|MM18693||Finland|658[0n]
Pararge aegeria|FBLMX207-11|BC ZSM Lep 50418||Germany|658[0n]
Pararge aegeria|FBLMU489-09|BC ZSM Lep 27139||Germany|658[0n]
Pararge aegeria|FBLMU490-09|BC ZSM Lep 27140||Germany|658[0n]
Pararge aegeria|GWORO996-09|BC ZSM Lep 32303||Italy|658[0n]
Pararge aegeria|GWORA2475-09|BC ZSM Lep 30687||Germany|658[0n]
Pararge aegeria|LEFIJ560-10|MM17185||Finland|658[0n]
Pararge aegeria|LEFIJ561-10|MM17186||Finland|658[0n]
Pararge aegeria|LON950-12|NHMO Lep2011.010||Norway|658[0n]
Pararge aegeria|FBLMT873-09|BC ZSM Lep 25433||Germany|658[0n]
Pararge aegeria|FBLMT891-09|BC ZSM Lep 25451||Germany|658[0n]
Pararge aegeria|EZROM451-08|RV-06-K523|Genit. examined|Romania|658[0n]
Pararge aegeria|EZROM453-08|RV-07-D200|Genit. examined|Romania|658[0n]
Pararge aegeria|EZRMN155-08|RVcoll.08-M358||Romania|658[0n]
Pararge aegeria|EZSPC585-09|RVcoll.08-H233|Genit. examined|Spain|658[0n]
Pararge aegeria|EZSPC586-09|RVcoll.08-M920|Genit. examined|Spain|658[0n]
Pararge aegeria|EZSPN437-09|RVcoll.08-H619||Spain|658[0n]
Pararge aegeria|EZROM745-08|RV-07-C003|Genit. examined|Spain|658[0n]
Pararge aegeria|EZSPN370-09|RVcoll.08-H395|Genit. examined|Spain|658[0n]
Pararge aegeria|EZSPN572-09|RVcoll.08-J119||Portugal|658[0n]
Pararge aegeria|EZSPM276-09|SMcoll.08-J610|Genit. examined|Spain|658[0n]
Pararge aegeria|EZSPM371-09|RVcoll.08-J844||Spain|658[0n]
Pararge aegeria|EZSPC587-09|RVcoll.08-P324||Spain|658[0n]
Pararge aegeria|EZSPC588-09|RVcoll.08-P056||Spain|658[0n]
Pararge aegeria|EZSPM243-09|RVcoll.08-R453|Genit. examined|Spain|658[0n]
Pararge aegeria|EZSPC817-10|RVcoll.08-P648||Spain|658[0n]
Pararge aegeria|EZSPC856-10|RVcoll.08-R452|Genit. examined|Spain|658[0n]
Pararge aegeria|EZSPM921-12|Rvcoll.12-M643||Spain|658[0n]
Pararge aegeria|EZSPC752-10|RVcoll.08-L197|Genit. examined|Spain|658[0n]
Pararge aegeria|WMB157-11|RVcoll.11-D313|Sicily|Italy|658[0n]
Pararge aegeria|WMB178-11|RVcoll.11-D450|Sicily|Italy|658[0n]
Pararge aegeria|WMB183-11|RVcoll.11-D483||Italy|658[0n]
Pararge aegeria|WMB231-11|RVcoll.09-T555|Lipari|Italy|658[0n]
Pararge aegeria|WMB025-11|RVcoll.LD-916|Elba|Italy|658[0n]
Pararge aegeria|WMB026-11|RVcoll.LD-917|Elba|Italy|658[0n]
Pararge aegeria|WMB027-11|RVcoll.LD-918|Elba|Italy|658[0n]
Pararge aegeria|WMB030-11|RVcoll.LD-1113||Italy|658[0n]
Pararge aegeria|WMB031-11|RVcoll.LD-1114||Italy|658[0n]
Pararge aegeria|WMB032-11|RVcoll.LD-1115||Italy|658[0n]
Pararge aegeria|WMB2932-14|RVcoll.11-Y102|Sicily|Italy|658[0n]
Pararge aegeria|WMB2956-14|RVcoll.12-N007|Ibiza|Spain|658[0n]
Pararge aegeria|WMB2959-14|RVcoll.12-N051|Ibiza|Spain|658[0n]
Pararge aegeria|WMB2984-14|RVcoll.12-O268|Corsica|France|658[0n]
Pararge aegeria|WMB3094-14|RVcoll.LD-2226|Capraia|Italy|658[0n]
Pararge aegeria|WMB3160-14|RVcoll.08-H325||Spain|658[0n]
Pararge aegeria|WMB3180-14|RVcoll.08-H601.1|Genit. examined|Spain|658[0n]
Pararge aegeria|WMB3274-14|RVcoll.08-M007||Spain|658[0n]
Pararge aegeria|WMB3459-14|RVcoll.10-A597||France|658[0n]
Pararge aegeria|WMB3580-14|RVcoll.11-E027|Genit. examined|Spain|658[0n]
Pararge aegeria|WMB3642-14|RVcoll.11-I551|Genit. examined|Spain|658[0n]
Pararge aegeria|WMB3699-14|RVcoll.11-J555||Spain|658[0n]
Pararge aegeria|WMB1107-13|RVcoll.10-C799|Ischia|Italy|658[0n]
Pararge aegeria|WMB1109-13|RVcoll.11-D012|Capri|Italy|658[0n]
Pararge aegeria|WMB1236-13|RVcoll.12-L291|Gozo|Malta|658[0n]
Pararge aegeria|WMB1237-13|RVcoll.12-L292|Gozo|Malta|658[0n]
Pararge aegeria|WMB2768-13|RVcoll.11-H833|Vulcano|Italy|658[0n]
Pararge aegeria|WMB2776-13|RVcoll.11-H897|Salina|Italy|658[0n]
Pararge aegeria|WMB1507-13|RVcoll.12-O092|Corsica|France|658[0n]
Pararge aegeria|WMB1770-13|RVcoll.12-Q040||France|658[0n]
Pararge aegeria|WMB1818-13|RVcoll.11-I604|Genit. examined|France|658[0n]
Pararge aegeria|WMB1673-13|RVcoll.12-P314||France|658[0n]
Pararge aegeria|WMB1744-13|RVcoll.12-P956|Levant|France|658[0n]
Pararge aegeria|WMB1745-13|RVcoll.12-P957|Levant|France|658[0n]
Pararge aegeria|WMB3852-14|RVcoll.10-C746||Italy|658[0n]
Pararge aegeria|WMB2330-13|RVcoll.12-L284|Gozo|Malta|658[0n]
Pararge aegeria|WMB2331-13|RVcoll.12-L285|Gozo|Malta|658[0n]
Pararge aegeria|WMB2347-13|RVcoll.12-M496||Italy|658[0n]
Pararge aegeria|WMB2395-13|RVcoll.09-X815||Italy|658[0n]
Pararge aegeria|WMB2420-13|RVcoll.10-C690|Ischia|Italy|658[0n]
Pararge aegeria|WMB2430-13|RVcoll.11-D015|Capri|Italy|658[0n]
Pararge aegeria|WMB6004-17|RVcollLD0441|Capraia|Italy|658[0n]
Pararge aegeria|WMB2633-13|RVcoll.12-O163|Corsica|France|658[0n]
Pararge aegeria|WMB4134-14|RVcoll.13-S638||Italy|658[0n]
Pararge aegeria|WMB4238-14|RVcoll.13-T841||Italy|658[0n]
Pararge aegeria|WMB3872-14|RVcoll.11-Y032||Italy|658[0n]
Pararge aegeria|WMB3900-14|RVcoll.12-P194||France|658[0n]
Pararge aegeria|WMB4506-14|RVcoll.14-E184||Italy|658[0n]
Pararge aegeria|WMB4535-14|RVcoll.LD-0258||Italy|658[0n]
Pararge aegeria|WMB4582-14|RVcoll.150308GV93||Spain|658[0n]
Pararge aegeria|WMB4281-14|RVcoll.14-A318||Italy|658[0n]

Pararge aegeria|WMB4535-14|RVcoll.LD-0258||Italy|658[0n]
Pararge aegeria|WMB4582-14|RVcoll.150308GV93||Spain|658[0n]
Pararge aegeria|WMB4281-14|RVcoll.14-A318||Italy|658[0n]
Pararge aegeria|WMB4398-14|RVcoll.14-B378||Portugal|658[0n]
Pararge aegeria|WMB4425-14|RVcoll.14-B481||Portugal|658[0n]
Pararge aegeria|WMB4609-14|RVcoll.LD-1664|Stromboli|Italy|658[0n]
Pararge aegeria|WMB4610-14|RVcoll.LD-1665|Stromboli|Italy|658[0n]
Pararge aegeria|WMB4616-14|RVcoll.LD-2136||Italy|658[0n]
Pararge aegeria|WMB4625-14|RVcoll.LD-2267|Giglio|Italy|658[0n]
Pararge aegeria|WMB4626-14|RVcoll.LD-2268|Giglio|Italy|658[0n]
Pararge aegeria|WMB487-11|RVcoll.11-H532|Sicily|Italy|658[0n]
Pararge aegeria|WMB516-11|RVcoll.11-H604|Sicily|Italy|658[0n]
Pararge aegeria|WMB576-11|RVcoll.11-H827|Vulcano|Italy|658[0n]
Pararge aegeria|WMB5079-14|RVcoll. 14-L210||Italy|658[0n]
Pararge aegeria|WMB588-11|RVcoll.11-H895|Salina|Italy|658[0n]
Pararge aegeria|WMB637-11|RVcoll.11-I138||Italy|658[0n]
Pararge aegeria|WMB4888-14|RVcoll.14-I435||Italy|658[0n]
Pararge aegeria|WMB5352-14|RVcoll.14-J396|Giannutri|Italy|658[0n]
Pararge aegeria|WMB4935-14|RVcoll.14-I482||Italy|658[0n]
Pararge aegeria|WMB4977-14|RVcoll. 14-I524||Italy|658[0n]
Pararge aegeria|WMB5045-14|RVcoll. 14-L176||Italy|658[0n]
Pararge aegeria|WMB5485-14|RVcoll.14-N731|Stromboli|Italy|658[0n]
Pararge aegeria|WMB5486-14|RVcoll.14-N732|Stromboli|Italy|658[0n]
Pararge aegeria|WMB5507-14|RVcoll.14-J455|Capraia|Italy|658[0n]
Pararge aegeria|EULEP4369-16|RVcoll15G978||Switzerland|658[0n]
Pararge aegeria|EULEP355-14|MM23840||Lithuania|658[0n]
Pararge aegeria|EULEP1134-15|RVcoll.14-F268||Serbia|658[0n]
Pararge aegeria|EULEP786-15|RVcoll.12-Z199||Sweden|658[0n]
Pararge aegeria|EULEP1388-15|RVcoll.14-F995||Greece|658[0n]
Pararge aegeria|EULEP2152-15|RVcoll.14-K051||Switzerland|658[0n]
Pararge aegeria|EULEP2429-15|RVcoll.14-V220||Belgium|658[0n]
Pararge aegeria|EULEP2024-15|RVcoll.14-I873||Poland|658[0n]
Pararge aegeria|BIBSA1239-15|15-F809||France|658[0n]
Pararge aegeria|BIBSA1256-15|15-F854||France|658[0n]
Pararge aegeria|BIBSA923-15|16-A014||France|658[0n]
Pararge aegeria|BIBSA933-15|16-A024||Italy|658[0n]
Pararge aegeria|BIBSA1541-16|11-D332||Italy|658[0n]
Pararge aegeria|BIBSA1099-15|15-L355||Italy|658[0n]
Pararge aegeria|BIBSA1783-16|16-A515||Italy|658[0n]
Pararge aegeria|BIBSA575-15|LEP-SS-00195||Italy|658[0n]
Pararge aegeria|BIBSA121-15|RVcoll.14-D556||Italy|658[0n]
Pararge aegeria|BIBSA682-15|RVcoll. 11-I245||Italy|658[0n]
Pararge aegeria|BIBSA1806-17|RVcoll14I275||Italy|658[0n]
Pararge aegeria|BIBSA514-15|LEP-SS-00134||Italy|658[0n]
Pararge aegeria|BIBSA1717-16|15-N210||Italy|658[0n]
Pararge aegeria|BIBSA145-15|RVcoll.14-D930||Italy|658[0n]
Pararge aegeria|BIBSA334-15|RVcoll.14-I002||Italy|658[0n]
Pararge aegeria|GBLAA347-14|BC ZSM Lep 80343||Germany|658[0n]
Pararge aegeria|BIBSA1835-17|RVcoll16C710||Italy|658[0n]
Pararge aegeria|GBLAB146-13|BC ZSM Lep 75772||Germany|658[0n]
Pararge aegeria|LEASS698-17|TLMF Lep 22346||Austria|658[0n]
Pararge aegeria|OXB1442-16|OXB-TGS-1216||United Kingdom|658[0n]
Pararge aegeria|OXB773-15|14-A714||Italy|658[0n]
Pararge aegeria|OXB792-15|14-J123|Corsica|France|658[0n]
Pararge aegeria|OXB793-15|14-J166|Corsica|France|658[0n]
Pararge aegeria|OXB795-15|14-J219|Corsica|France|658[0n]
Pararge aegeria|OXB796-15|14-J220|Corsica|France|658[0n]
Pararge aegeria|OXB797-15|14-J222|Corsica|France|658[0n]
Pararge aegeria|OXB808-15|14-J275|Corsica|France|658[0n]
Pararge aegeria|OXB809-15|14-J276|Corsica|France|658[0n]
Pararge aegeria|OXB812-15|14-J279|Corsica|France|658[0n]
Pararge aegeria|OXB813-15|14-J280|Corsica|France|658[0n]
Pararge aegeria|OXB814-15|14-J281|Corsica|France|658[0n]
Pararge aegeria|OXB815-15|14-J282|Corsica|France|658[0n]
Pararge aegeria|OXB861-15|15-A505||Italy|658[0n]
Pararge aegeria|OXB862-15|15-A506||Italy|658[0n]
Pararge aegeria|OXB1118-15|15-A651||Italy|658[0n]
Pararge aegeria|OXB638-15|08-H604|Genit. examined|Spain|658[0n]
Pararge aegeria|OXB639-15|11-D453||Italy|658[0n]
Pararge aegeria|OXB640-15|11-E028||Spain|658[0n]
Pararge aegeria|OXB644-15|11-H603||Italy|658[0n]
Pararge aegeria|OXB645-15|11-I139||Italy|658[0n]
Pararge aegeria|OXB646-15|11-I556||Spain|658[0n]
Pararge aegeria|OXB647-15|11-I603|Genit. examined|France|658[0n]
Pararge aegeria|OXB649-15|12-N055||Spain|658[0n]
Pararge aegeria|OXB652-15|12-Q039||France|658[0n]
Pararge aegeria|OXB653-15|12-Z132||Ireland|658[0n]
Pararge aegeria|OXB654-15|12-Z200||Sweden|658[0n]
Pararge aegeria|OXB655-15|12-Z201||Sweden|658[0n]
Pararge aegeria|OXB656-15|14-A346||Italy|658[0n]
Pararge aegeria|OXB657-15|14-A556||Italy|658[0n]
Pararge aegeria|OXB658-15|14-B370||Portugal|658[0n]
Pararge aegeria|OXB659-15|12-Z100||Ireland|658[0n]
Pararge aegeria|OXB660-15|14-A344||Italy|658[0n]
Pararge aegeria|OXB661-15|14-A555||Italy|658[0n]
Pararge aegeria|OXB662-15|14-O025||United Kingdom|658[0n]
Pararge aegeria|OXB663-15|14-O026||United Kingdom|658[0n]
Pararge aegeria|OXB664-15|14-O027||Belgium|658[0n]
Pararge aegeria|OXB665-15|14-B355||Portugal|658[0n]
Pararge aegeria|OXB743-15|13-U316||Italy|658[0n]
Pararge aegeria|OXB909-15|15-A553||Italy|658[0n]
Pararge aegeria|OXB923-15|15-A567||Italy|658[0n]
Pararge aegeria|OXB327-15|RVcoll.14-N041||Italy|658[0n]
Pararge aegeria|OXB1063-15|14-A387||Italy|658[0n]
Pararge aegeria|OXB197-15|OXB-TGS-147||United Kingdom|658[0n]
Pararge aegeria|OXB202-15|OXB-TGS-184||United Kingdom|658[0n]
Pararge aegeria|OXB205-15|OXB-TGS-187||United Kingdom|658[0n]
Pararge aegeria|OXB249-15|OXB-TGS-790||United Kingdom|658[0n]
Pararge aegeria|OXB255-15|OXB-TGS-825||United Kingdom|658[0n]
Pararge aegeria|OXB256-15|OXB-TGS-827||United Kingdom|658[0n]
Pararge aegeria|OXB279-15|OXB-TGS-962||United Kingdom|658[0n]
Pararge aegeria|WMB3546-14|RVcoll.11-D862|Genit. examined|Spain|658[0n]
Pararge aegeria|OXB1309-15|Rvcoll. 15-M573||France|658[0n]

Pararge aegeria|OXB2[illegible]-15|OXB-TGS-[illegible]||United Kingdom|658[0n]
Pararge aegeria|OXB279-15|OXB-TGS-962||United Kingdom|658[0n]
Pararge aegeria|WMB3546-14|RVcoll.11-D862|Genit. examined|Spain|658[0n]
Pararge aegeria|OXB1309-15|Rvcoll_15-M573||France|658[0n]
Pararge aegeria|OXB1312-15|Rvcoll_15-M576||France|658[0n]
Pararge aegeria|OXB1348-15|Rvcoll_15-M700||France|658[0n]
Pararge aegeria|WMB4627-14|RVcoll.LD-2271||Italy|658[0n]
Pararge aegeria|OXB1154-15|Rvcoll_15-N133||Italy|658[0n]
Pararge aegeria|OXB1374-15|Rvcoll_15-M738||France|658[0n]
Pararge aegeria|WMB1982-13|RVcoll.11-I285|Pianosa|Italy|658[0n]
Pararge aegeria|EZROM645-08|RV-07-D597||Romania|652[0n]
Pararge aegeria|GWORA2476-09|BC ZSM Lep 30688||Germany|611[0n]
Pararge aegeria|WMB1949-13|RVcoll.11-H900|Salina|Italy|647[0n]
Pararge aegeria|EULEP4958-16|RVcoll16I259||Poland|636[0n]
Pararge aegeria|WMB1269-13|RVcoll.12-M292|Lipari|Italy|632[0n]
Pararge aegeria|WMB5304-14|RVcoll.14-I626||France|644[0n]
Pararge aegeria|WMB3929-14|RVcoll.12-P528||France|620[0n]
Pararge aegeria|WMB1983-13|RVcoll.11-I286|Pianosa|Italy|632[0n]
Pararge aegeria|OXB1000-15|15-A950||Italy|632[0n]
Pararge aegeria|WMB1169-13|RVcoll.11-E746|Corsica. Genit. examined|France|621[0n]
Pararge aegeria|WMB4624-14|RVcoll.LD-2259|Ischia|Italy|618[0n]
Pararge aegeria|BIBSA513-15|LEP-SS-00133||Italy|622[0n]
Pararge aegeria|WMB1937-13|RVcoll.11-H828|Vulcano|Italy|618[0n]
Pararge aegeria|WMB2892-14|RVcoll.11-D400|Sicily|Italy|625[0n]
Pararge aegeria|WMB123-11|RVcoll.11-D011|Capri|Italy|635[0n]
Pararge aegeria|LENOA1368-11|LN-BD1368||France|632[0n]
Pararge aegeria|OXB1222-15|Rvcoll_15-M650||France|658[0n]
Pararge aegeria|LENOA1367-11|LN-BD1367||France|628[0n]
Pararge aegeria|EULEP1584-15|RVcoll.14-G612||Greece|614[0n]
Pararge aegeria|PHLAC361-10|TLMF Lep 02396||Italy|623[0n]
Hipparchia statilinus|EZROM740-08|RV-06-G531||Spain|650[0n]
Hipparchia statilinus|EZSPM403-09|Rvcoll.09-V805||Spain|658[0n]
Hipparchia statilinus|WMB3999-14|RVcoll.12-Q498||Spain|658[0n]
Hipparchia statilinus|EZSPN1113-11|Rvcoll.10-C310||Spain|658[0n]
Hipparchia statilinus|EZSPC1128-10|RVcoll.08-P694||Spain|658[0n]
Hipparchia statilinus|EZSPC1367-10|RVcoll.09-X056||Spain|658[0n]
Hipparchia statilinus|WMB3499-14|RVcoll.10-B724||France|658[0n]
Hipparchia statilinus|WMB102-11|RVcoll.10-B610||France|658[0n]
Hipparchia statilinus|WMB3949-14|RVcoll.12-P706||France|658[0n]
Hipparchia statilinus|EZSPM291-09|RVcoll.08-J822||Spain|658[0n]
Hipparchia statilinus|WMB4004-14|RVcoll.12-Q530||Spain|658[0n]
Hipparchia statilinus|BIBSA915-15|16-A006||France|658[0n]
Hipparchia statilinus|WMB3647-14|RVcoll.11-I613||France|658[0n]
Hipparchia statilinus|WMB1795-13|RVcoll.12-Q314||France|658[0n]
Hipparchia statilinus|WMB4370-14|RVcoll.14-B140||Portugal|658[0n]
Hipparchia statilinus|GBLAF771-14|BC ZSM Lep 82857||Germany|658[0n]
Hipparchia statilinus|EULEP4873-16|RVcoll16H948||Netherlands|632[0n]
Hipparchia statilinus|WMB1669-13|RVcoll.12-P251||France|624[0n]
Hipparchia statilinus|WMB3531-14|RVcoll.10-C136|Genit. examined|France|658[0n]
Hipparchia statilinus|EZSPC1213-10|RVcoll.09-V506||Spain|658[0n]
Hipparchia statilinus|EZSPC1366-10|RVcoll.09-X055||Spain|658[0n]
Hipparchia statilinus|EZSPM453-09|Rvcoll.09-V589||Spain|658[0n]
Hipparchia statilinus|EZSPM165-09|RVcoll.08-P696||Spain|658[0n]
Hipparchia statilinus|EZSPN1112-11|Rvcoll.10-C309||Spain|658[0n]
Hipparchia statilinus|EZSPC561-09|RVcoll.08-P052||Spain|658[0n]
Hipparchia statilinus|EZSPM116-09|RVcoll.08-P475||Spain|658[3n]
Hipparchia statilinus|WMB3937-14|RVcoll.12-P587||France|658[0n]
Hipparchia statilinus|WMB1731-13|RVcoll.12-P902||France|658[0n]
Hipparchia statilinus|WMB3967-14|RVcoll.12-Q091||France|658[0n]
Hipparchia statilinus|WMB3917-14|RVcoll.12-P394||France|658[0n]
Hipparchia statilinus|EZSPN601-09|RVcoll.08-J193||Portugal|658[0n]
Hipparchia statilinus|EZSPC562-09|RVcoll.08-P053||Spain|658[0n]
Hipparchia statilinus|EZSPC1027-10|RVcoll.08-J075||Spain|658[0n]
Hipparchia statilinus|EZSPN776-09|RVcoll.08-L286||Spain|658[0n]
Hipparchia statilinus|BCLEP282-17|LEP-SS-00708||Italy|658[0n]
Hipparchia statilinus|WMB6336-18|RVcoll14A107|Capri|Italy|658[0n]
Hipparchia statilinus|EULEP4082-16|RVcoll14A944||Russia|658[0n]
Hipparchia statilinus|EULEP2875-15|RVcoll.14-U828||Greece|658[0n]
Hipparchia statilinus|EULEP4827-16|RVcoll16H725||Ukraine|658[0n]
Hipparchia statilinus|EULEP2882-15|RVcoll.14-U835||Albania|658[0n]
Hipparchia statilinus|ABOLD588-17|TLMF Lep 21664||Austria|658[0n]
Hipparchia statilinus|EULEP4080-16|RVcoll15Q171||Ukraine|658[0n]
Hipparchia statilinus|WMB6339-18|RVcoll14C812||Bulgaria|658[2n]
Hipparchia statilinus|EULEP4078-16|RVcoll14W998||Romania|639[0n]
Hipparchia statilinus|EULEP4081-16|RVcoll15Q175||Ukraine|634[0n]
Hipparchia statilinus|EZROM1020-08|RVcoll.08-M582||Romania|609[0n]
Hipparchia statilinus|EZROM603-08|RV-07-F536||Romania|658[0n]
Hipparchia statilinus|WMB2073-13|RVcoll.12-Q932||Italy|658[0n]
Hipparchia statilinus|WMB2364-13|RVcoll.12-Q924||Italy|658[0n]
Hipparchia statilinus|BIBSA1610-16|14-A775||Italy|658[0n]
Hipparchia statilinus|BIBSA709-15|RVcoll. 14-A680||Italy|658[0n]
Hipparchia statilinus|BIBSA700-15|RVcoll. 12-Q856||Italy|658[0n]
Hipparchia statilinus|BIBSA926-15|16-A017||Italy|658[0n]
Hipparchia statilinus|WMB2113-13|RVcoll.12-R183|Sicily|Italy|658[0n]
Hipparchia statilinus|OXB1082-15|15-A615||Italy|658[0n]
Hipparchia statilinus|BIBSA1603-16|12-R179||Italy|658[0n]
Hipparchia statilinus|WMB5286-14|RVcoll.14-D924||Italy|658[0n]
Hipparchia statilinus|WMB6335-18|RVcoll14A106|Capri|Italy|658[0n]
Hipparchia statilinus|WMB3068-14|RVcoll.14-A811|Capri|Italy|658[0n]
Hipparchia statilinus|EULEP5656-17|RVcoll15J998||Italy|658[0n]
Hipparchia statilinus|WMB6334-18|RVcoll14A079||Italy|658[0n]
Hipparchia statilinus|EULEP5659-17|RVcoll15K654||Italy|658[0n]
Hipparchia statilinus|BIBSA133-15|RVcoll.14-D904||Italy|658[0n]
Hipparchia statilinus|BIBSA1594-16|12-Q952||Italy|658[0n]
Hipparchia statilinus|WMB4339-14|RVcoll.14-A730||Italy|619[0n]
Hipparchia statilinus|WMB2592-13|RVcoll.12-R213||Italy|658[0n]
Hipparchia statilinus|WMB2580-13|RVcoll.12-R082|Sicily|Italy|658[0n]
Hipparchia statilinus|WMB2237-13|RVcoll.12-R366|Elba|Italy|658[0n]
Hipparchia statilinus|WMB2129-13|RVcoll.12-R238||Italy|658[0n]
Hipparchia statilinus|WMB2087-13|RVcoll.12-R030|Sicily|Italy|658[0n]
Hipparchia statilinus|WMB2080-13|RVcoll.12-Q999|Sicily|Italy|658[0n]
Hipparchia statilinus|EULEP2881-15|RVcoll.14-U834||Greece|658[0n]
Hipparchia statilinus|WMB3016-14|RVcoll.12-R178|Sicily|Italy|658[0n]

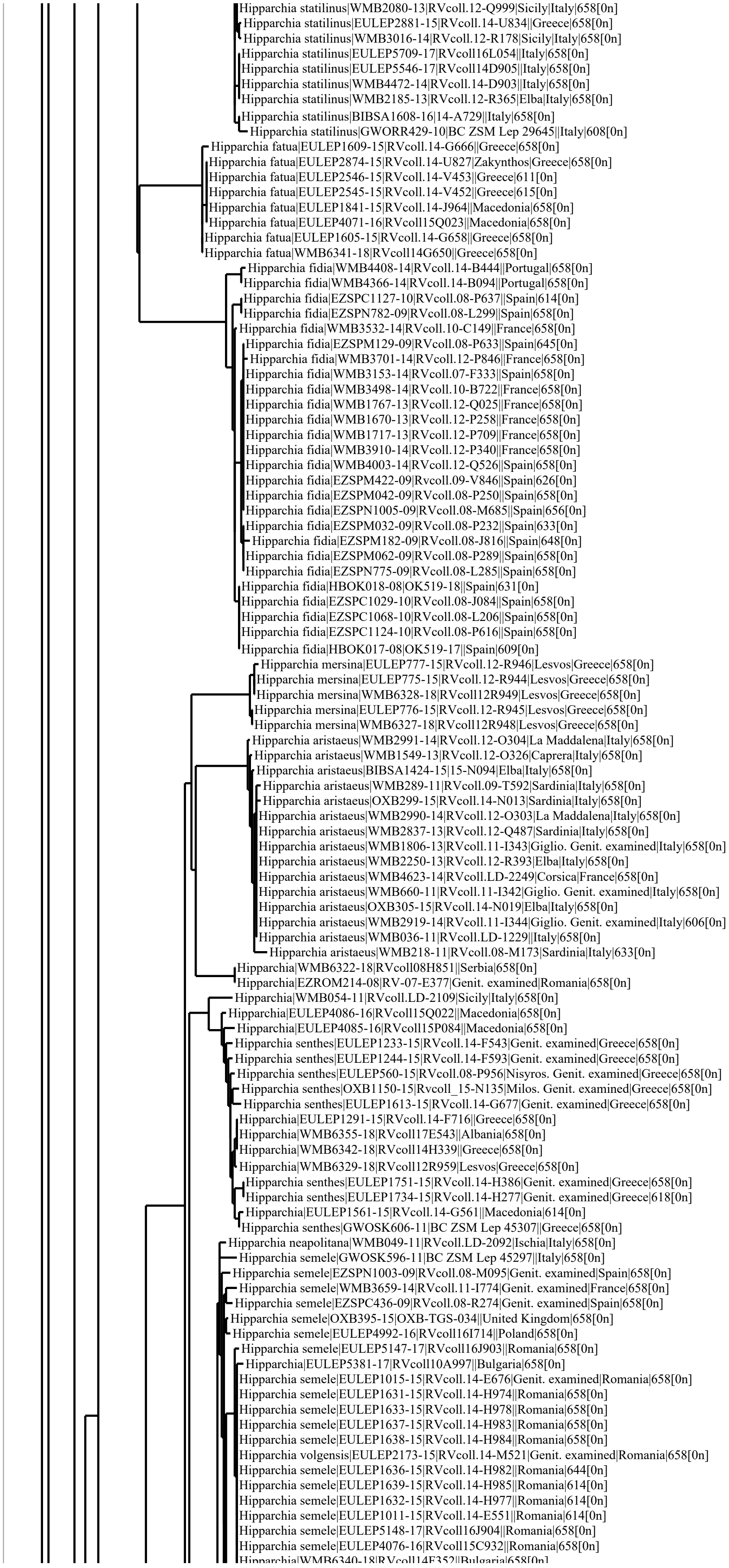
Hipparchia statilinus|WMB2080-13|RVcoll.12-Q999|Sicily|Italy|658[0n]
Hipparchia statilinus|EULEP2881-15|RVcoll.14-U834||Greece|658[0n]
Hipparchia statilinus|WMB3016-14|RVcoll.12-R178|Sicily|Italy|658[0n]
Hipparchia statilinus|EULEP5709-17|RVcoll16L054||Italy|658[0n]
Hipparchia statilinus|EULEP5546-17|RVcoll14D905||Italy|658[0n]
Hipparchia statilinus|WMB4472-14|RVcoll.14-D903||Italy|658[0n]
Hipparchia statilinus|WMB2185-13|RVcoll.12-R365|Elba|Italy|658[0n]
Hipparchia statilinus|BIBSA1608-16|14-A729||Italy|658[0n]
Hipparchia statilinus|GWORR429-10|BC ZSM Lep 29645||Italy|608[0n]
Hipparchia fatua|EULEP1609-15|RVcoll.14-G666||Greece|658[0n]
Hipparchia fatua|EULEP2874-15|RVcoll.14-U827|Zakynthos|Greece|658[0n]
Hipparchia fatua|EULEP2546-15|RVcoll.14-V453||Greece|611[0n]
Hipparchia fatua|EULEP2545-15|RVcoll.14-V452||Greece|615[0n]
Hipparchia fatua|EULEP1841-15|RVcoll.14-J964||Macedonia|658[0n]
Hipparchia fatua|EULEP4071-16|RVcoll15Q023||Macedonia|658[0n]
Hipparchia fatua|EULEP1605-15|RVcoll.14-G658||Greece|658[0n]
Hipparchia fatua|WMB6341-18|RVcoll14G650||Greece|658[0n]
Hipparchia fidia|WMB4408-14|RVcoll.14-B444||Portugal|658[0n]
Hipparchia fidia|WMB4366-14|RVcoll.14-B094||Portugal|658[0n]
Hipparchia fidia|EZSPC1127-10|RVcoll.08-P637||Spain|614[0n]
Hipparchia fidia|EZSPN782-09|RVcoll.08-L299||Spain|658[0n]
Hipparchia fidia|WMB3532-14|RVcoll.10-C149||France|658[0n]
Hipparchia fidia|EZSPM129-09|RVcoll.08-P633||Spain|645[0n]
Hipparchia fidia|WMB3701-14|RVcoll.12-P846||France|658[0n]
Hipparchia fidia|WMB3153-14|RVcoll.07-F333||Spain|658[0n]
Hipparchia fidia|WMB3498-14|RVcoll.10-B722||France|658[0n]
Hipparchia fidia|WMB1767-13|RVcoll.12-Q025||France|658[0n]
Hipparchia fidia|WMB1670-13|RVcoll.12-P258||France|658[0n]
Hipparchia fidia|WMB1717-13|RVcoll.12-P709||France|658[0n]
Hipparchia fidia|WMB3910-14|RVcoll.12-P340||France|658[0n]
Hipparchia fidia|WMB4003-14|RVcoll.12-Q526||Spain|658[0n]
Hipparchia fidia|EZSPM422-09|Rvcoll.09-V846||Spain|626[0n]
Hipparchia fidia|EZSPM042-09|RVcoll.08-P250||Spain|658[0n]
Hipparchia fidia|EZSPN1005-09|RVcoll.08-M685||Spain|656[0n]
Hipparchia fidia|EZSPM032-09|RVcoll.08-P232||Spain|633[0n]
Hipparchia fidia|EZSPM182-09|RVcoll.08-J816||Spain|648[0n]
Hipparchia fidia|EZSPM062-09|RVcoll.08-P289||Spain|658[0n]
Hipparchia fidia|EZSPN775-09|RVcoll.08-L285||Spain|658[0n]
Hipparchia fidia|HBOK018-08|OK519-18||Spain|631[0n]
Hipparchia fidia|EZSPC1029-10|RVcoll.08-J084||Spain|658[0n]
Hipparchia fidia|EZSPC1068-10|RVcoll.08-L206||Spain|658[0n]
Hipparchia fidia|EZSPC1124-10|RVcoll.08-P616||Spain|658[0n]
Hipparchia fidia|HBOK017-08|OK519-17||Spain|609[0n]
Hipparchia mersina|EULEP777-15|RVcoll.12-R946|Lesvos|Greece|658[0n]
Hipparchia mersina|EULEP775-15|RVcoll.12-R944|Lesvos|Greece|658[0n]
Hipparchia mersina|WMB6328-18|RVcoll12R949|Lesvos|Greece|658[0n]
Hipparchia mersina|EULEP776-15|RVcoll.12-R945|Lesvos|Greece|658[0n]
Hipparchia mersina|WMB6327-18|RVcoll12R948|Lesvos|Greece|658[0n]
Hipparchia aristaeus|WMB2991-14|RVcoll.12-O304|La Maddalena|Italy|658[0n]
Hipparchia aristaeus|WMB1549-13|RVcoll.12-O326|Caprera|Italy|658[0n]
Hipparchia aristaeus|BIBSA1424-15|15-N094|Elba|Italy|658[0n]
Hipparchia aristaeus|WMB289-11|RVcoll.09-T592|Sardinia|Italy|658[0n]
Hipparchia aristaeus|OXB299-15|RVcoll.14-N013|Sardinia|Italy|658[0n]
Hipparchia aristaeus|WMB2990-14|RVcoll.12-O303|La Maddalena|Italy|658[0n]
Hipparchia aristaeus|WMB2837-13|RVcoll.12-Q487|Sardinia|Italy|658[0n]
Hipparchia aristaeus|WMB1806-13|RVcoll.11-I343|Giglio. Genit. examined|Italy|658[0n]
Hipparchia aristaeus|WMB2250-13|RVcoll.12-R393|Elba|Italy|658[0n]
Hipparchia aristaeus|WMB4623-14|RVcoll.LD-2249|Corsica|France|658[0n]
Hipparchia aristaeus|WMB660-11|RVcoll.11-I342|Giglio. Genit. examined|Italy|658[0n]
Hipparchia aristaeus|OXB305-15|RVcoll.14-N019|Elba|Italy|658[0n]
Hipparchia aristaeus|WMB2919-14|RVcoll.11-I344|Giglio. Genit. examined|Italy|606[0n]
Hipparchia aristaeus|WMB036-11|RVcoll.LD-1229||Italy|658[0n]
Hipparchia aristaeus|WMB218-11|RVcoll.08-M173|Sardinia|Italy|633[0n]
Hipparchia|WMB6322-18|RVcoll08H851||Serbia|658[0n]
Hipparchia|EZROM214-08|RV-07-E377|Genit. examined|Romania|658[0n]
Hipparchia|WMB054-11|RVcoll.LD-2109|Sicily|Italy|658[0n]
Hipparchia|EULEP4086-16|RVcoll15Q022||Macedonia|658[0n]
Hipparchia|EULEP4085-16|RVcoll15P084||Macedonia|658[0n]
Hipparchia senthes|EULEP1233-15|RVcoll.14-F543|Genit. examined|Greece|658[0n]
Hipparchia senthes|EULEP1244-15|RVcoll.14-F593|Genit. examined|Greece|658[0n]
Hipparchia senthes|EULEP560-15|RVcoll.08-P956|Nisyros. Genit. examined|Greece|658[0n]
Hipparchia senthes|OXB1150-15|Rvcoll_15-N135|Milos. Genit. examined|Greece|658[0n]
Hipparchia senthes|EULEP1613-15|RVcoll.14-G677|Genit. examined|Greece|658[0n]
Hipparchia|EULEP1291-15|RVcoll.14-F716||Greece|658[0n]
Hipparchia|WMB6355-18|RVcoll17E543||Albania|658[0n]
Hipparchia|WMB6342-18|RVcoll14H339||Greece|658[0n]
Hipparchia|WMB6329-18|RVcoll12R959|Lesvos|Greece|658[0n]
Hipparchia senthes|EULEP1751-15|RVcoll.14-H386|Genit. examined|Greece|658[0n]
Hipparchia senthes|EULEP1734-15|RVcoll.14-H277|Genit. examined|Greece|618[0n]
Hipparchia|EULEP1561-15|RVcoll.14-G561||Macedonia|614[0n]
Hipparchia senthes|GWOSK606-11|BC ZSM Lep 45307||Greece|658[0n]
Hipparchia neapolitana|WMB049-11|RVcoll.LD-2092|Ischia|Italy|658[0n]
Hipparchia semele|GWOSK596-11|BC ZSM Lep 45297||Italy|658[0n]
Hipparchia semele|EZSPN1003-09|RVcoll.08-M095|Genit. examined|Spain|658[0n]
Hipparchia semele|WMB3659-14|RVcoll.11-I774|Genit. examined|France|658[0n]
Hipparchia semele|EZSPC436-09|RVcoll.08-R274|Genit. examined|Spain|658[0n]
Hipparchia semele|OXB395-15|OXB-TGS-034||United Kingdom|658[0n]
Hipparchia semele|EULEP4992-16|RVcoll16I714||Poland|658[0n]
Hipparchia semele|EULEP5147-17|RVcoll16J903||Romania|658[0n]
Hipparchia|EULEP5381-17|RVcoll10A997||Bulgaria|658[0n]
Hipparchia semele|EULEP1015-15|RVcoll.14-E676|Genit. examined|Romania|658[0n]
Hipparchia semele|EULEP1631-15|RVcoll.14-H974||Romania|658[0n]
Hipparchia semele|EULEP1633-15|RVcoll.14-H978||Romania|658[0n]
Hipparchia semele|EULEP1637-15|RVcoll.14-H983||Romania|658[0n]
Hipparchia semele|EULEP1638-15|RVcoll.14-H984||Romania|658[0n]
Hipparchia volgensis|EULEP2173-15|RVcoll.14-M521|Genit. examined|Romania|658[0n]
Hipparchia semele|EULEP1636-15|RVcoll.14-H982||Romania|644[0n]
Hipparchia semele|EULEP1639-15|RVcoll.14-H985||Romania|614[0n]
Hipparchia semele|EULEP1632-15|RVcoll.14-H977||Romania|614[0n]
Hipparchia semele|EULEP1011-15|RVcoll.14-E551||Romania|614[0n]
Hipparchia semele|EULEP5148-17|RVcoll16J904||Romania|658[0n]
Hipparchia semele|EULEP4076-16|RVcoll15C932||Romania|658[0n]
Hipparchia|WMB6340-18|RVcoll14E352||Bulgaria|658[0n]

[illegible]
Hipparchia semele|EULEP5148-17|RVcoll16J904||Romania|658[0n]
Hipparchia semele|EULEP4076-16|RVcoll15C932||Romania|658[0n]
Hipparchia|WMB6340-18|RVcoll14F352||Bulgaria|658[0n]
Hipparchia semele|EZROM630-08|RV-07-E378|Genit. examined|Romania|658[0n]
Hipparchia semele|EZRMN420-09|RVcoll.09-V621|Genit. examined|Romania|658[0n]
Hipparchia semele|LEFIJ551-10|MM17176||Finland|658[0n]
Hipparchia semele|EULEP132-14|RVcoll.10-B727||France|658[0n]
Hipparchia semele|EZRMN389-09|RVcoll.08-H032|Genit. examined|Romania|658[0n]
Hipparchia semele|EZSPN1062-11|Rvcoll.08-P717|Genit. examined|Spain|658[0n]
Hipparchia semele|HBOK008-08|OK519-08||Spain|658[0n]
Hipparchia aristaeus|OXB300-15|RVcoll.14-N014|Sardinia|Italy|658[0n]
Hipparchia christenseni|EULEP5433-17|RVcoll11H119|Karpathos. Genit. examined|Greece|658[0n]
Hipparchia semele|EULEP5084-16|RVcoll16J366||Germany|658[0n]
Hipparchia semele|EZSPM906-12|Rvcoll.12-M628||Spain|658[0n]
Hipparchia semele|EZSPN1074-11|Rvcoll.09-V478||Spain|658[0n]
Hipparchia semele|EZSPN737-09|RVcoll.08-L147||Spain|658[0n]
Hipparchia semele|GWOSK598-11|BC ZSM Lep 45299||Spain|658[0n]
Hipparchia|WMB558-11|RVcoll.11-H751|Sicily|Italy|658[0n]
Hipparchia|WMB543-11|RVcoll.11-H731|Sicily|Italy|658[0n]
Hipparchia semele|OXB505-15|OXB-TGS-677||United Kingdom|658[0n]
Hipparchia semele|OXB788-15|LD-2131||Italy|658[0n]
Hipparchia semele|EULEP014-14|RVcoll.07-C295||Belgium|658[0n]
Hipparchia semele|WMB1784-13|RVcoll.12-Q190||France|658[0n]
Hipparchia semele|EZSPM349-09|RVcoll.08-R058||Spain|658[0n]
Hipparchia|WMB602-11|RVcoll.11-H973|Sicily|Italy|658[0n]
Hipparchia|WMB051-11|RVcoll.LD-2100||Italy|658[0n]
Hipparchia semele|EULEP1137-15|RVcoll.14-F297||Serbia|658[0n]
Hipparchia|WMB496-11|RVcoll.11-H546|Sicily|Italy|658[0n]
Hipparchia|WMB6324-18|RVcoll08L371||Ukraine|658[0n]
Hipparchia semele|EULEP049-14|RVcoll.08-H804||Sweden|658[0n]
Hipparchia semele|EULEP050-14|RVcoll.08-H815||Sweden|658[0n]
Hipparchia semele|GBLAB147-13|BC ZSM Lep 75773||Germany|658[0n]
Hipparchia semele|EULEP4807-16|RVcoll16H693||Norway|637[0n]
Hipparchia neapolitana|WMB4615-14|RVcoll.LD-2093|Ischia|Italy|625[0n]
Hipparchia neapolitana|WMB4599-14|RVcoll.LD-1232|Capri|Italy|619[0n]
Hipparchia|WMB5992-17|RVcollLD2102||Italy|658[0n]
Hipparchia|WMB055-11|RVcoll.LD-2112|Sicily|Italy|658[0n]
Hipparchia neapolitana|WMB050-11|RVcoll.LD-2095|Ischia|Italy|658[0n]
Hipparchia|WMB216-11|RVcoll.08-M165|Sicily|Italy|658[0n]
Hipparchia neapolitana|WMB192-11|RVcoll.06-V787||Italy|658[0n]
Hipparchia semele|EZROM1065-09|RVcoll.08-H037||Romania|658[0n]
Hipparchia aristaeus|WMB1545-13|RVcoll.12-O302|La Maddalena|Italy|658[0n]
Hipparchia semele|BIBSA111-15|RVcoll.14-D539|Genit. examined|Italy|632[0n]
Hipparchia aristaeus|WMB4622-14|RVcoll.LD-2244|Sardinia|Italy|658[0n]
Hipparchia aristaeus|WMB4597-14|RVcoll.LD-1187|Corsica|France|658[0n]
Hipparchia aristaeus|WMB2992-14|RVcoll.12-O305|La Maddalena. Genit. examined|Italy|658[0n]
Hipparchia semele|WMB100-11|RVcoll.10-B607||France|658[0n]
Hipparchia semele|EZSPM825-12|Rvcoll. 140611ZG43||Spain|658[0n]
Hipparchia semele|EZSPM917-12|Rvcoll.12-M639||Spain|658[0n]
Hipparchia semele|EZSPN463-09|RVcoll.08-H677||Spain|658[0n]
Hipparchia semele|EZSPN769-09|RVcoll.08-L273||Spain|658[0n]
Hipparchia semele|EZSPC438-09|RVcoll.08-M673||Spain|658[0n]
Hipparchia semele|EZSPC437-09|RVcoll.08-P359||Spain|658[0n]
Hipparchia semele|GWOSK595-11|BC ZSM Lep 45296||Spain|658[0n]
Hipparchia semele|WMB4444-14|RVcoll.14-B657||United Kingdom|658[0n]
Hipparchia blachieri|WMB2750-13|RVcoll.10-C651|Genit. examined|Italy|632[0n]
Hipparchia volgensis|EULEP1158-15|RVcoll.14-F357|Genit. examined|Bulgaria|658[0n]
Hipparchia semele|WMB5294-14|RVcoll.14-I591||France|658[0n]
Hipparchia semele|WMB3704-14|RVcoll.12-L280||Spain|658[0n]
Hipparchia semele|EZSPM151-09|RVcoll.08-P666||Spain|658[0n]
Hipparchia|WMB052-11|RVcoll.LD-2101||Italy|658[0n]
Hipparchia semele|HBOK007-08|OK519-07||Spain|658[0n]
Hipparchia semele|LEFIJ552-10|MM17177||Finland|658[0n]
Hipparchia semele|EZROM196-08|RV-07-E379|Genit. examined|Romania|658[0n]
Hipparchia semele|EZROM631-08|RV-07-C138|Genit. examined|Romania|658[0n]
Hipparchia semele|EZSPC439-09|RVcoll.08-P027||Spain|658[0n]
Hipparchia semele|EZROM213-08|RV-07-D409|Genit. examined|Romania|658[0n]
Hipparchia volgensis|EZROM216-08|RV-07-D072|Genit. examined|Romania|658[0n]
Hipparchia semele|WMB3345-14|RVcoll.08-R035|Genit. examined|Spain|658[0n]
Hipparchia semele|WMB3648-14|RVcoll.11-I622|Genit. examined|France|658[0n]
Hipparchia|WMB2753-13|RVcoll.10-C665||Italy|658[0n]
Hipparchia|WMB2590-13|RVcoll.12-R177|Sicily|Italy|658[0n]
Hipparchia semele|WMB4440-14|RVcoll.14-B634||United Kingdom|658[0n]
Hipparchia|WMB6326-18|RVcoll12R943|Lesvos|Greece|658[0n]
Hipparchia|EULEP1156-15|RVcoll.14-F355||Bulgaria|658[0n]
Hipparchia|EULEP1157-15|RVcoll.14-F356||Bulgaria|658[0n]
Hipparchia|EULEP780-15|RVcoll.12-R961|Lesvos|Greece|658[0n]
Hipparchia|EULEP2070-15|RVcoll.14-J555||Ukraine|658[0n]
Hipparchia semele|OXB1486-16|RVcoll14B667||United Kingdom|658[0n]
Hipparchia semele|OXB1488-16|RVcoll14B642||United Kingdom|658[0n]
Hipparchia semele|OXB1489-16|RVcoll14B637||United Kingdom|658[0n]
Hipparchia|EULEP778-15|RVcoll.12-R950|Lesvos|Greece|658[0n]
Hipparchia|WMB6330-18|RVcoll12R963|Lesvos|Greece|658[0n]
Hipparchia semele|OXB1490-16|RVcoll14B636||United Kingdom|658[0n]
Hipparchia semele|OXB1491-16|RVcoll14B635||United Kingdom|658[0n]
Hipparchia semele|OXB1492-16|RVcoll14B633||United Kingdom|658[0n]
Hipparchia semele|OXB1493-16|RVcoll14B632||United Kingdom|658[0n]
Hipparchia semele|OXB492-15|OXB-TGS-584||United Kingdom|658[0n]
Hipparchia semele|OXB561-15|OXB-TGS-936||United Kingdom|658[0n]
Hipparchia|WMB6323-18|RVcoll08L370||Ukraine|658[0n]
Hipparchia|EULEP1068-15|RVcoll.14-E981||Serbia|623[0n]
Hipparchia semele|WMB1708-13|RVcoll.12-P650||France|627[0n]
Hipparchia semele|OXB1494-16|RVcoll14B631||United Kingdom|626[0n]
Hipparchia semele|GWORA2878-15|BC ZSM Lep 86146||Germany|614[0n]
Hipparchia semele|EULEP4077-16|RVcoll15G049||France|622[0n]
Hipparchia semele|WMB4374-14|RVcoll.14-B159||Portugal|615[0n]
Hipparchia semele|EZROM367-08|07-E624||Spain|641[2n]
Hipparchia semele|EZSPN958-09|RVcoll.08-L955||Spain|647[0n]
Hipparchia semele|LEFID811-10|MM06897||Finland|658[0n]
Hipparchia semele|ABOLD045-16|TLMF Lep 21123||Austria|658[0n]
Hipparchia semele|EZSPN1027-09|RVcoll.08-M961||Spain|658[0n]
Hipparchia semele|WMB3329-14|RVcoll.08-P763||Spain|658[0n]
[illegible]

Hipparchia semele|ABOLD045-16|TLMF Lep 21125||Austria|658[0n]
Hipparchia semele|EZSPN1027-09|RVcoll.08-M961||Spain|658[0n]
Hipparchia semele|WMB3329-14|RVcoll.08-P763||Spain|658[0n]
Hipparchia semele|EZSPN956-09|RVcoll.08-L953||Spain|658[0n]
Hipparchia volgensis|EULEP2585-15|RVcoll.14-V592|Genit. examined|Russia|658[0n]
Hipparchia|EULEP489-14|KN00851||Russia|658[0n]
Hipparchia volgensis|EULEP2586-15|RVcoll.14-V593|Genit. examined|Russia|658[0n]
Hipparchia|EULEP4088-16|RVcoll14A942||Russia|658[0n]
Hipparchia|WMB6348-18|RVcoll14J558||Ukraine|658[0n]
Hipparchia volgensis|EULEP4087-16|RVcoll14A941|Genit. examined|Russia|658[0n]
Hipparchia|LEFIJ6628-17|MM26010||Bulgaria|658[0n]
Hipparchia|EULEP2072-15|RVcoll.14-J557||Ukraine|658[0n]
Hipparchia|EULEP1495-15|RVcoll.14-G336||Greece|658[0n]
Hipparchia|EULEP1372-15|RVcoll.14-F966||Greece|658[0n]
Hipparchia semele|EZROM215-08|RV-06-V719|Genit. examined|Romania|658[0n]
Hipparchia semele|EULEP998-15|RVcoll.14-E161||Romania|658[0n]
Hipparchia|EULEP2073-15|RVcoll.14-J560||Ukraine|658[0n]
Hipparchia|EULEP2074-15|RVcoll.14-J561||Ukraine|658[0n]
Hipparchia|EULEP2525-15|RVcoll.14-V398||Ukraine|621[0n]
Hipparchia semele|EULEP1634-15|RVcoll.14-H980||Romania|641[0n]
Hipparchia volgensis|EULEP4844-16|RVcoll16H753|Genit. examined|Ukraine|639[0n]
Hipparchia pellucida|EULEP2514-15|RVcoll.14-V383|Genit. examined|Ukraine|615[0n]
Hipparchia semele|EZRMN338-08|RVcoll.08-H018|Genit. examined|Romania|649[0n]
Hipparchia sbordonii|WMB6352-18|RVcollLD1087|Ponza|Italy|658[0n]
Hipparchia cretica|EULEP828-15|RVcoll.14-B673|Crete|Greece|658[0n]
Hipparchia cretica|EULEP827-15|RVcoll.14-B672|Crete|Greece|658[0n]
Hipparchia cretica|EULEP826-15|RVcoll.14-B671|Crete|Greece|658[0n]
Hipparchia cretica|WMB6338-18|RVcoll14B670|Crete|Greece|658[0n]
Hipparchia cretica|WMB6337-18|RVcoll14B669|Crete|Greece|658[0n]
Hipparchia|WMB4906-14|RVcoll.14-I453||Italy|658[0n]
Hipparchia cypriensis|EULEP2189-15|RVcoll.14-N265||Cyprus|658[0n]
Hipparchia cypriensis|EULEP4064-16|RVcoll14A926||Cyprus|658[0n]
Hipparchia cypriensis|EULEP4063-16|RVcoll14A925||Cyprus|658[0n]
Hipparchia cypriensis|EULEP4062-16|RVcoll14A924||Cyprus|658[0n]
Hipparchia semele|WMB084-11|RVcoll.LD-3141||Italy|658[0n]
Hipparchia|EULEP1542-15|RVcoll.14-G511||Greece|658[0n]
Hipparchia|EULEP1501-15|RVcoll.14-G379||Greece|658[0n]
Hipparchia|WMB217-11|RVcoll.08-M168|Sicily|Italy|634[0n]
Hipparchia|WMB6356-18|RVcoll17E545||Albania|658[0n]
Hipparchia|BIBSA509-15|LEP-SS-00129||Italy|658[0n]
Hipparchia christenseni|EULEP674-15|RVcoll.11-J514|Karpathos|Greece|658[0n]
Hipparchia christenseni|EULEP5434-17|RVcoll11H120|Karpathos. Genit. examined|Greece|658[0n]
Hipparchia|WMB6325-18|RVcoll12R212||Italy|658[0n]
Hipparchia semele|BIBSA1062-15|14-V317||Italy|658[0n]
Hipparchia sbordonii|WMB2264-13|RVcoll.12-Z335|Ponza|Italy|658[0n]
Hipparchia|WMB3815-14|RVcoll.07-E067||Italy|658[0n]
Hipparchia semele|WMB4143-14|RVcoll.13-S659||Italy|658[0n]
Hipparchia|WMB6331-18|RVcoll12R965||Greece|658[0n]
Hipparchia|EULEP5243-17|RVcoll07E002||Italy|658[0n]
Hipparchia|EULEP781-15|RVcoll.12-R966|Lesvos|Greece|658[0n]
Hipparchia semele|BIBSA1024-15|15-C169||Italy|658[0n]
Hipparchia|BIBSA508-15|LEP-SS-00128||Italy|658[0n]
Hipparchia|BIBSA762-15|LEP-SS-00273||Italy|658[0n]
Hipparchia semele|OXB1487-16|RVcoll14B643||United Kingdom|658[0n]
Hipparchia semele|OXB841-15|14-U804||Italy|658[0n]
Hipparchia semele|OXB464-15|OXB-TGS-404||United Kingdom|658[0n]
Hipparchia semele|OXB465-15|OXB-TGS-405||United Kingdom|658[0n]
Hipparchia semele|OXB475-15|OXB-TGS-450||United Kingdom|658[0n]
Hipparchia semele|OXB476-15|OXB-TGS-451||United Kingdom|658[0n]
Hipparchia semele|OXB491-15|OXB-TGS-583||United Kingdom|658[0n]
Hipparchia semele|OXB506-15|OXB-TGS-678||United Kingdom|658[0n]
Hipparchia|EULEP2069-15|RVcoll.14-J554||Ukraine|627[0n]
Hipparchia semele|EULEP1072-15|RVcoll.14-E991|Genit. examined|Serbia|658[0n]
Hipparchia|EULEP1329-15|RVcoll.14-F809|Genit. examined|Greece|629[0n]
Hipparchia volgensis|EULEP4845-16|RVcoll16H754|Genit. examined|Ukraine|635[0n]
Hipparchia cypriensis|EULEP4880-16|RVcoll16H973||Cyprus|632[0n]
Hipparchia|WMB2118-13|RVcoll.12-R190||Italy|627[0n]
Hipparchia semele|WMB2796-13|RVcoll.11-J744||Italy|658[0n]
Hipparchia sbordonii|WMB3090-14|RVcoll.LD-1089|Ponza|Italy|658[0n]
Hipparchia semele|WMB083-11|RVcoll.LD-3140||Italy|658[0n]
Hipparchia semele|WMB056-11|RVcoll.LD-2130||Italy|658[0n]
Hipparchia sbordonii|HBOK003-08|OK519-03||Italy|658[0n]
Hipparchia semele|LEATG490-14|TLMF Lep 14277||Italy|658[0n]
Hipparchia semele|LEATH674-14|TLMF Lep 15886||Italy|634[0n]
Hipparchia semele|LEATB705-13|TLMF Lep 10882||Italy|658[0n]
Hipparchia semele|ABOLA496-14|TLMF Lep 16183||Austria|658[0n]
Hipparchia semele|ABOLA497-14|TLMF Lep 16184||Austria|658[0n]
Hipparchia|BIBSA676-15|RVcoll. 08-M171|Sicily|Italy|658[0n]
Hipparchia|WMB053-11|RVcoll.LD-2103||Italy|658[0n]
Hipparchia leighebi|WMB583-11|RVcoll.11-H873|Salina|Italy|658[0n]
Hipparchia leighebi|WMB5481-14|RVcoll.14-N706|Alicudi|Italy|658[0n]
Hipparchia leighebi|EULEP555-15|RVcoll.08-M162|Vulcano|Italy|658[0n]
Hipparchia leighebi|WMB1940-13|RVcoll.11-H841|Vulcano|Italy|622[0n]
Hipparchia leighebi|WMB2391-13|RVcoll.09-T558|Stromboli|Italy|627[0n]
Hipparchia leighebi|WMB1064-13|RVcoll.10-C686|Stromboli|Italy|622[0n]
Hipparchia leighebi|WMB868-13|RVcoll.09-T557|Stromboli|Italy|658[0n]
Hipparchia leighebi|WMB2771-13|RVcoll.11-H845|Vulcano|Italy|658[0n]
Hipparchia leighebi|WMB4488-14|RVcoll.LD-3213|Lipari|Italy|613[0n]
Hipparchia leighebi|WMB1939-13|RVcoll.11-H840|Vulcano|Italy|653[0n]
Hipparchia leighebi|HBOK002-08|OK519-02||Italy|658[1n]
Hipparchia leighebi|HBOK001-08|OK519-01||Italy|658[0n]
Hipparchia neomiris|WMB6065-18|RVcoll14A077|Sardinia|Italy|658[0n]
Hipparchia neomiris|WMB6067-18|RVcoll14A110|Sardinia|Italy|658[0n]
Hipparchia neomiris|WMB6066-18|RVcoll14A088|Sardinia|Italy|658[0n]
Hipparchia neomiris|WMB6064-18|RVcoll14A076|Sardinia|Italy|658[0n]
Hipparchia neomiris|WMB865-13|RVcoll.09-T528|Corsica|France|658[0n]
Hipparchia neomiris|OXB1046-15|LD-0833|Elba|Italy|658[0n]
Hipparchia neomiris|OXB1047-15|LD-0834|Elba|Italy|658[0n]
Hipparchia neomiris|WMB4641-14|RVcoll.LD-2415|Corsica|France|632[0n]
Hipparchia neomiris|WMB2252-13|RVcoll.12-R402|Elba|Italy|658[0n]
Hipparchia neomiris|WMB866-13|RVcoll.09-T529|Corsica|France|658[0n]
Hipparchia neomiris|WMB2862-14|RVcoll.09-X881.1|Corsica|France|658[0n]
Hipparchia autonoe|WMB6350-18|RVcoll14V119||Russia|658[0n]

Hipparchia neomiris|WMB866-13|RVcoll.09-T529|Corsica|France|658[0n]
Hipparchia neomiris|WMB2862-14|RVcoll.09-X881.1|Corsica|France|658[0n]
Hipparchia autonoe|WMB6350-18|RVcoll14V119||Russia|658[0n]
Hipparchia fagi|EULEP1460-15|RVcoll.14-G211|Genit. examined|Greece|658[0n]
Hipparchia fagi|EULEP1469-15|RVcoll.14-G236|Genit. examined|Greece|658[0n]
Hipparchia fagi|EULEP1413-15|RVcoll.14-G080|Genit. examined|Greece|658[0n]
Hipparchia fagi|EULEP1337-15|RVcoll.14-F826|Genit. examined|Greece|658[0n]
Hipparchia fagi|EULEP4069-16|RVcoll14A936||Russia|658[0n]
Hipparchia syriaca|EULEP4065-16|RVcoll10B456|Genit. examined|Croatia|658[0n]
Hipparchia fagi|EULEP876-15|RVcoll.14-C407|Genit. examined|Greece|658[0n]
Hipparchia fagi|EZSPM288-09|SMcoll.220708XF16||Spain|658[0n]
Hipparchia fagi|EZSPN1044-11|Rvcoll.08-J677||Spain|658[0n]
Hipparchia fagi|WMB5299-14|RVcoll.14-I613||France|658[0n]
Hipparchia fagi|BIBSA716-15|RVcoll. 14-A774||Italy|658[0n]
Hipparchia fagi|EULEP1555-15|RVcoll.14-G548|Genit. examined|Macedonia|658[0n]
Hipparchia fagi|EULEP1077-15|RVcoll.14-F017|Genit. examined|Serbia|658[0n]
Hipparchia fagi|WMB2128-13|RVcoll.12-R237||Italy|658[0n]
Hipparchia fagi|WMB1087-13|RVcoll.10-C749||Italy|622[0n]
Hipparchia syriaca|WMB6333-18|RVcoll14A040||Romania|658[0n]
Hipparchia syriaca|WMB6332-18|RVcoll14A039|Genit. examined|Romania|658[0n]
Hipparchia syriaca|EULEP2883-15|RVcoll.14-U836|Genit. examined|Croatia|658[0n]
Hipparchia fagi|OXB1083-15|15-A616||Italy|637[0n]
Hipparchia fagi|EULEP959-15|RVcoll.14-D012|Genit. examined|Bulgaria|658[0n]
Hipparchia fagi|EULEP947-15|RVcoll.14-C950||Bulgaria|658[0n]
Hipparchia syriaca|EULEP682-15|RVcoll.11-J534|Rhodes|Greece|658[0n]
Hipparchia syriaca|EULEP681-15|RVcoll.11-J533|Rhodes|Greece|658[0n]
Hipparchia syriaca|EULEP680-15|RVcoll.11-J532|Rhodes|Greece|658[0n]
Hipparchia fagi|WMB2105-13|RVcoll.12-R118|Sicily|Italy|658[0n]
Hipparchia fagi|WMB2571-13|RVcoll.12-Q960|Sicily|Italy|658[0n]
Hipparchia fagi|WMB1876-13|RVcoll.11-J647|Sicily|Italy|658[0n]
Hipparchia fagi|BIBSA761-15|LEP-SS-00262||Italy|658[0n]
Hipparchia fagi|WMB2108-13|RVcoll.12-R150|Sicily|Italy|658[0n]
Hipparchia fagi|WMB2387-13|RVcoll.12-Q955|Sicily|Italy|658[0n]
Hipparchia fagi|WMB6353-18|RVcollLD1729|Capri|Italy|658[0n]
Hipparchia fagi|BCLEP107-17|LEP-SS-00558||Italy|658[0n]
Hipparchia fagi|BIBSA715-15|RVcoll. 14-A773||Italy|658[0n]
Hipparchia fagi|BCLEP106-17|LEP-SS-00557||Italy|622[0n]
Hipparchia fagi|WMB4343-14|RVcoll.14-A771||Italy|658[0n]
Hipparchia fagi|WMB4611-14|RVcoll.LD-1723||Italy|644[0n]
Hipparchia fagi|WMB2101-13|RVcoll.12-R114|Sicily|Italy|623[0n]
Hipparchia fagi|WMB2076-13|RVcoll.12-Q982|Sicily|Italy|658[0n]
Hipparchia fagi|WMB1231-13|RVcoll.11-H331|Sicily|Italy|658[0n]
Hipparchia fagi|WMB3067-14|RVcoll.14-A801|Capri|Italy|658[0n]
Hipparchia fagi|WMB3066-14|RVcoll.14-A800|Capri|Italy|658[0n]
Hipparchia fagi|EZROM724-08|RV-06-G561||Spain|658[0n]
Hipparchia fagi|EULEP258-14|RVcoll.12-Q529||Spain|658[0n]
Hipparchia fagi|WMB3922-14|RVcoll.12-P499||France|658[0n]
Hipparchia fagi|WMB1704-13|RVcoll.12-P634||France|658[0n]
Hipparchia fagi|WMB1691-13|RVcoll.12-P559||France|658[0n]
Hipparchia fagi|WMB1080-13|RVcoll.10-C715||Italy|658[0n]
Hipparchia fagi|WMB3658-14|RVcoll.11-I771||France|658[0n]
Hipparchia fagi|WMB3497-14|RVcoll.10-B721||France|658[0n]
Hipparchia fagi|WMB101-11|RVcoll.10-B608||France|658[0n]
Hipparchia fagi|EZSPM824-12|Rvcoll. 140611ZG25||Spain|658[0n]
Hipparchia fagi|EZSPC434-09|RVcoll.06-V772||Spain|658[0n]
Hipparchia fagi|EZSPC433-09|RVcoll.08-R297||Spain|658[0n]
Hipparchia fagi|EZSPC432-09|RVcoll.08-R152|Genit. examined|Spain|658[0n]
Hipparchia fagi|EZSPC435-09|RVcoll.08-P317||Spain|658[0n]
Hipparchia fagi|ABOLD586-17|TLMF Lep 21662||Austria|658[0n]
Hipparchia fagi|EULEP5027-16|RVcoll16I965||Slovakia|658[0n]
Hipparchia fagi|EULEP4068-16|RVcoll14A935||Russia|658[0n]
Hipparchia fagi|OXB851-15|14-U814||Italy|658[0n]
Hipparchia fagi|EULEP4066-16|RVcoll15H047||Italy|658[0n]
Hipparchia fagi|BIBSA113-15|RVcoll.14-D543||Italy|633[0n]
Hipparchia fagi|WMB2261-13|RVcoll.12-R422||Italy|658[0n]
Hipparchia fagi|EZROM319-08|RV-07-E681|Genit. examined|Romania|658[0n]
Hipparchia fagi|EULEP536-15|RVcoll.08-H880|Genit. examined|Serbia|658[0n]
Hipparchia syriaca|EZROM1076-09|RVcoll.08-M472||Romania|658[0n]
Hipparchia syriaca|EZROM1016-08|RVcoll.08-M481|Genit. examined|Romania|658[0n]
Hipparchia syriaca|EZROM1015-08|RVcoll.08-M480|Genit. examined|Romania|658[0n]
Hipparchia syriaca|EZROM1014-08|RVcoll.08-M464|Genit. examined|Romania|658[0n]
Hipparchia syriaca|EZROM1078-09|RVcoll.08-M479|Genit. examined|Romania|658[0n]
Hipparchia syriaca|EZROM1013-08|RVcoll.08-M463|Genit. examined|Romania|658[0n]
Hipparchia syriaca|EZROM1077-09|RVcoll.08-M476||Romania|658[0n]
Hipparchia syriaca|EZRMN319-08|RVcoll.08-M487|Genit. examined|Romania|658[0n]
Hipparchia syriaca|EZRMN318-08|RVcoll.08-M486|Genit. examined|Romania|658[0n]
Hipparchia syriaca|EZRMN317-08|RVcoll.08-M484|Genit. examined|Romania|658[0n]
Hipparchia syriaca|EZRMN316-08|RVcoll.08-M465|Genit. examined|Romania|658[0n]
Hipparchia fagi|EZROM210-08|RV-06-V696|Genit. examined|Romania|656[0n]
Hipparchia fagi|EULEP874-15|RVcoll.14-C383|Genit. examined|Greece|658[0n]
Hipparchia fagi|EULEP1010-15|RVcoll.14-E550|Genit. examined|Romania|658[0n]
Hipparchia fagi|EZROM211-08|RV-06-V700|Genit. examined|Romania|658[0n]
Hipparchia fagi|EZROM310-08|RV-07-E672||Romania|658[0n]
Hipparchia fagi|EZROM1017-08|RVcoll.08-M570|Genit. examined|Romania|658[0n]
Hipparchia fagi|EZROM629-08|RV-07-E383|Genit. examined|Romania|658[0n]
Hipparchia fagi|EZRMN421-09|RVcoll.09-V622|Genit. examined|Romania|658[0n]
Hipparchia hermione|BIBSA770-15|LEP-SS-00263||Italy|637[0n]
Hipparchia hermione|WMB2570-13|RVcoll.12-Q916||Italy|658[0n]
Hipparchia hermione|WMB2569-13|RVcoll.12-Q915||Italy|658[0n]
Hipparchia hermione|EULEP819-15|RVcoll.14-A324||Italy|658[0n]
Hipparchia hermione|EULEP4074-16|RVcoll14V185||Italy|658[0n]
Hipparchia hermione|BIBSA510-15|LEP-SS-00130||Italy|658[0n]
Hipparchia hermione|WMB916-13|RVcoll.09-X920||Italy|658[0n]
Hipparchia hermione|WMB1787-13|RVcoll.12-Q212||France|658[0n]
Hipparchia hermione|WMB2119-13|RVcoll.12-R191||Italy|658[0n]
Hipparchia hermione|WMB1658-13|RVcoll.12-P102||France|658[0n]
Hipparchia hermione|WMB1884-13|RVcoll.11-J745||Italy|658[0n]
Hipparchia hermione|WMB2004-13|RVcoll.12-Q392||France|658[0n]
Hipparchia hermione|WMB2029-13|RVcoll.12-Q715||Italy|658[0n]
Hipparchia hermione|WMB3981-14|RVcoll.12-Q246||France|658[0n]
Hipparchia hermione|WMB3992-14|RVcoll.12-Q386||France|658[0n]
Hipparchia hermione|WMB4958-14|RVcoll. 14-I505||Italy|658[0n]
[illegible]

Hipparchia hermione|WMB3981-14|RVcoll.12-Q246||France|658[0n]
Hipparchia hermione|WMB3992-14|RVcoll.12-Q386||France|658[0n]
Hipparchia hermione|WMB4958-14|RVcoll. 14-I505||Italy|658[0n]
Hipparchia hermione|WMB5010-14|RVcoll. 14-I557|Genit. examined|Italy|658[0n]
Hipparchia hermione|EULEP4067-16|RVcoll15J667||France|658[0n]
Hipparchia hermione|EULEP4070-16|RVcoll15J144||Switzerland|658[0n]
Hipparchia hermione|EULEP4072-16|RVcoll14W864||Italy|658[0n]
Hipparchia hermione|EULEP4073-16|RVcoll15H317|Genit. examined|Switzerland|658[0n]
Hipparchia hermione|EULEP167-14|RVcoll.11-I657||France|658[0n]
Hipparchia hermione|EULEP168-14|RVcoll.11-I780||France|658[0n]
Hipparchia hermione|EULEP169-14|RVcoll.11-I885||France|658[0n]
Hipparchia hermione|EULEP171-14|RVcoll.11-J138||Switzerland|658[0n]
Hipparchia hermione|EULEP248-14|RVcoll.12-P047||France|658[0n]
Hipparchia hermione|EULEP623-15|RVcoll.11-I785||France|658[0n]
Hipparchia hermione|WMB2056-13|RVcoll.12-Q829||Italy|658[0n]
Hipparchia hermione|EULEP624-15|RVcoll.11-I876||France|658[0n]
Hipparchia hermione|BIBSA1156-15|15-L113||Italy|658[0n]
Hipparchia hermione|BIBSA511-15|LEP-SS-00131||Italy|658[0n]
Hipparchia hermione|OXB1597-16|OXB-TGS-1315||France|658[0n]
Hipparchia hermione|OXB1598-16|OXB-TGS-1316||France|637[0n]
Hipparchia hermione|EULEP4075-16|RVcoll15G463|Genit. examined|Switzerland|636[0n]
Hipparchia hermione|OXB1600-16|OXB-TGS-1318||France|618[0n]
Hipparchia hermione|WMB1079-13|RVcoll.10-C714||Italy|620[0n]
Hipparchia hermione|WMB1653-13|RVcoll.12-P025||France|658[0n]
Hipparchia hermione|WMB2706-13|RVcoll.10-C126||France|629[0n]
Hipparchia hermione|WMB4746-14|RVcoll.07-E096.1||Italy|658[0n]
Hipparchia hermione|GWOSU1042-11|BC Back 0092||France|658[0n]
Hipparchia hermione|WMB3200-14|RVcoll.08-J083||Spain|658[0n]
Hipparchia hermione|EZSPM796-12|RVcoll. 080611XP73||Spain|658[0n]
Hipparchia hermione|EZSPC758-10|RVcoll.08-L267||Spain|658[0n]
Hipparchia hermione|EZSPC1395-10|SMcoll.190608FT66||Spain|658[0n]
Hipparchia hermione|HBOK010-08|OK519-10||Spain|647[0n]
Hipparchia hermione|EZSPN902-09|RVcoll.08-L819||Spain|658[0n]
Hipparchia hermione|EZSPM164-09|RVcoll.08-P695||Spain|658[0n]
Hipparchia hermione|EULEP246-14|RVcoll.12-N741||Spain|658[0n]
Hipparchia hermione|WMB3283-14|RVcoll.08-M676|Genit. examined|Spain|658[0n]
Hipparchia hermione|WMB4387-14|RVcoll.14-B288||Portugal|658[0n]
Hipparchia hermione|EZSPC1020-10|RVcoll.08-P678||Spain|658[0n]
Hipparchia hermione|EZSPM845-12|Rvcoll. 150310ZX49||Spain|658[0n]
Hipparchia hermione|EZSPM1016-12|Rvcoll.12-M765||Spain|658[0n]
Hipparchia hermione|EZSPC895-10|RVcoll.09-V431||Spain|615[0n]
Hipparchia hermione|GWOSU1041-11|BC Back 0091||Spain|658[0n]
Hipparchia hermione|EZSPN551-09|RVcoll.08-J057||Spain|658[0n]
Hipparchia hermione|EZSPC703-10|RVcoll.07-F087||Spain|658[0n]
Hipparchia hermione|EZSPN505-09|RVcoll.08-H962||Spain|658[0n]
Hipparchia hermione|HBOK009-08|OK519-09||Spain|646[0n]
Hipparchia hermione|EZSPM1027-12|Rvcoll.12-M777||Spain|658[0n]
Hipparchia hermione|EZSPM1015-12|Rvcoll.12-M764||Spain|658[0n]
Hipparchia hermione|EZSPN1061-11|Rvcoll.08-P677.1|Genit. examined|Spain|658[0n]
Hipparchia hermione|EZSPC1021-10|RVcoll.08-P044||Spain|658[0n]
Hipparchia hermione|EZSPC960-10|RVcoll.100608DS72||Spain|634[0n]
Hipparchia hermione|EZSPM775-12|Rvcoll. 050111LP62||Spain|658[0n]
Hipparchia hermione|EZSPM303-09|RVcoll.08-J850||Spain|658[0n]
Hipparchia hermione|ABOLD587-17|TLMF Lep 21663||Austria|637[0n]
Hipparchia hermione|EULEP4808-16|RVcoll16H695||Norway|641[0n]
Hipparchia hermione|WMB4519-14|RVcoll.14-E217||Spain|658[0n]
Hipparchia hermione|WMB1705-13|RVcoll.12-P636||France|658[0n]
Hipparchia hermione|EZSPC834-10|RVcoll.08-R165||Spain|658[0n]
Hipparchia hermione|ABOLD022-16|TLMF Lep 21100||Austria|658[0n]
Hipparchia hermione|LEATJ1254-16|TLMF Lep 19577||Austria|658[0n]
Hipparchia hermione|LEATJ1255-16|TLMF Lep 19578||Austria|658[0n]
Minois dryas|LEASS496-17|TLMF Lep 22144||Austria|658[0n]
Minois dryas|EULEP252-14|RVcoll.12-Q446||Spain|658[0n]
Minois dryas|FBLMU377-09|BC ZSM Lep 27027||Germany|658[0n]
Minois dryas|LEATG502-14|TLMF Lep 14289||Italy|658[0n]
Minois dryas|PHLAF623-11|TLMF Lep 05793||Switzerland|658[0n]
Minois dryas|ABOLD025-16|TLMF Lep 21103||Austria|658[0n]
Minois dryas|LEATG503-14|TLMF Lep 14290||Italy|658[0n]
Minois dryas|ABOLA452-14|TLMF Lep 16139||Austria|658[0n]
Minois dryas|ABOLA453-14|TLMF Lep 16140||Austria|658[0n]
Minois dryas|PHLAH710-12|TLMF Lep 08529||Austria|658[0n]
Minois dryas|GWORT469-10|BC ZSM Lep 32155||Germany|658[0n]
Minois dryas|GWORT470-10|BC ZSM Lep 32156||Germany|658[0n]
Minois dryas|FBLMX139-11|BC ZSM Lep 37620||Germany|658[0n]
Minois dryas|GWOSK863-11|BC ZSM Lep 49364||Germany|658[0n]
Minois dryas|GWOSK907-11|BC ZSM Lep 49408||Germany|658[0n]
Minois dryas|FBLMV721-09|BC ZSM Lep 28701||Germany|658[0n]
Minois dryas|EZROM429-08|RV-06-M982||Romania|658[0n]
Minois dryas|EZRMN127-08|RVcoll.08-M571||Romania|658[0n]
Minois dryas|EZROM430-08|RV-07-E398||Romania|658[0n]
Minois dryas|EZROM431-08|RV-07-E430||Romania|658[0n]
Minois dryas|EZROM643-08|RV-07-C207||Romania|658[0n]
Minois dryas|EZRMN126-08|RVcoll.07-E385||Romania|658[0n]
Minois dryas|EZRMN128-08|RVcoll.08-M658||Romania|658[0n]
Minois dryas|EZSPM722-12|Rvcoll. 08-J477||Spain|658[0n]
Minois dryas|EZSPM723-12|Rvcoll. 08-J478||Spain|658[0n]
Minois dryas|EZSPM724-12|Rvcoll. 08-J479||Spain|658[0n]
Minois dryas|EZSPM729-12|Rvcoll. 12-L041||Spain|658[0n]
Minois dryas|EZSPM730-12|Rvcoll. 12-L042||Spain|658[0n]
Minois dryas|WMB1805-13|RVcoll.12-Q343||France|658[0n]
Minois dryas|WMB1831-13|RVcoll.11-I797||France|658[0n]
Minois dryas|WMB1655-13|RVcoll.12-P041||France|658[0n]
Minois dryas|WMB5004-14|RVcoll. 14-I551||Italy|658[0n]
Minois dryas|EULEP4305-16|RVcoll15H737||Switzerland|658[0n]
Minois dryas|EULEP4306-16|RVcoll15I883||Liechtenstein|658[0n]
Minois dryas|EULEP4307-16|RVcoll15J124||Switzerland|658[0n]
Minois dryas|EULEP053-14|RVcoll.08-J480||Spain|658[0n]
Minois dryas|EULEP054-14|RVcoll.08-J481||Spain|658[0n]
Minois dryas|EULEP055-14|RVcoll.08-J482||Spain|658[0n]
Minois dryas|EULEP056-14|RVcoll.08-J483||Spain|658[0n]
Minois dryas|EULEP4843-16|RVcoll16H751||Ukraine|658[0n]
Minois dryas|EULEP5020-16|RVcoll16I939||Slovakia|658[0n]
Minois dryas|EULEP194-14|RVcoll.12-L000||Spain|658[0n]

Minois dryas|EULEP4843-16|RVcoll16H751||Ukraine|658[0n]
Minois dryas|EULEP5020-16|RVcoll16I939||Slovakia|658[0n]
Minois dryas|EULEP194-14|RVcoll.12-L000||Spain|658[0n]
Minois dryas|EULEP195-14|RVcoll.12-L003||Spain|658[0n]
Minois dryas|EULEP215-14|RVcoll.12-L038||Spain|658[0n]
Minois dryas|EULEP216-14|RVcoll.12-L039||Spain|658[0n]
Minois dryas|EULEP217-14|RVcoll.12-L040||Spain|658[0n]
Minois dryas|EULEP251-14|RVcoll.12-Q445||Spain|658[0n]
Minois dryas|EULEP688-15|RVcoll.11-J915||Bulgaria|658[0n]
Minois dryas|EULEP644-15|RVcoll.11-J140||Switzerland|658[0n]
Minois dryas|EULEP2228-15|RVcoll.14-N407||Ukraine|658[0n]
Minois dryas|BIBSA096-15|RVcoll.14-D509||Italy|658[0n]
Minois dryas|BIBSA126-15|RVcoll.14-D580||Italy|658[0n]
Minois dryas|BIBSA449-15|RVcoll.14-I117||Italy|658[0n]
Minois dryas|LEASS894-17|KLM Lep 08399||Austria|658[0n]
Minois dryas|LEASS895-17|KLM Lep 08400||Austria|658[0n]
Minois dryas|OXB1561-16|OXB-TGS-1279||France|658[0n]
Minois dryas|WMB1076-13|RVcoll.10-C705||Italy|632[0n]
Minois dryas|HBOK035-08|OK519-35||Italy|648[1n]
Pyronia cecilia|EZSPC1079-10|RVcoll.08-L663|Genit. examined|Spain|658[0n]
Pyronia cecilia|WMB4418-14|RVcoll.14-B466||Portugal|658[0n]
Pyronia cecilia|EZSPM942-12|Rvcoll.12-M676||Spain|658[0n]
Pyronia cecilia|EZSPM887-12|Rvcoll.12-M605||Spain|658[0n]
Pyronia cecilia|EZSPM1013-12|Rvcoll.12-M762||Spain|658[0n]
Pyronia cecilia|BIBSA1300-15|LD-2241|Sardinia|Italy|658[0n]
Pyronia cecilia|WMB2828-13|RVcoll.12-O474|Sardinia|Italy|658[0n]
Pyronia cecilia|WMB2827-13|RVcoll.12-O455|Sardinia|Italy|658[0n]
Pyronia cecilia|WMB5929-17|RVcoll11H714|Levanzo|Italy|658[0n]
Pyronia cecilia|BIBSA1722-16|LD-2070||Italy|658[0n]
Pyronia cecilia|WMB2578-13|RVcoll.12-R038|Sicily|Italy|658[0n]
Pyronia cecilia|BIBSA1558-16|11-H310||Italy|632[0n]
Pyronia cecilia|WMB517-11|RVcoll.11-H605|Sicily|Italy|658[0n]
Pyronia cecilia|WMB5947-17|RVcoll12R168|Sicily|Italy|658[0n]
Pyronia cecilia|WMB5926-17|RVcoll11H607|Sicily|Italy|658[0n]
Pyronia cecilia|WMB5942-17|RVcoll11J686|Sicily|Italy|658[0n]
Pyronia cecilia|WMB3015-14|RVcoll.12-R166|Sicily|Italy|658[0n]
Pyronia cecilia|EZSPM069-09|RVcoll.08-P309||Spain|658[0n]
Pyronia cecilia|EZSPC1388-10|RVcoll.09-X560||Spain|658[0n]
Pyronia cecilia|WMB3942-14|RVcoll.12-P695||France|658[0n]
Pyronia cecilia|WMB1553-13|RVcoll.12-O383|Sardinia|Italy|658[0n]
Pyronia cecilia|OXB1030-15|LD-0392||France|658[0n]
Pyronia cecilia|WMB4696-14|RVcoll.LD-2876|Asinara|Italy|658[0n]
Pyronia cecilia|WMB2458-13|RVcoll.11-H284|Genit. examined|Italy|658[0n]
Pyronia cecilia|WMB1984-13|RVcoll.11-I288|Pianosa|Italy|658[0n]
Pyronia cecilia|WMB862-13|RVcoll.09-T516|Corsica|France|658[0n]
Pyronia cecilia|EZSPM076-09|RVcoll.08-P325||Spain|658[0n]
Pyronia cecilia|EZSPM1039-12|Rvcoll.12-M790||Spain|658[0n]
Pyronia cecilia|EZSPN406-09|RVcoll.08-H497|Genit. examined|Spain|658[0n]
Pyronia cecilia|WMB4435-14|RVcoll.14-B534||Portugal|658[0n]
Pyronia cecilia|WMB4430-14|RVcoll.14-B505||Portugal|658[0n]
Pyronia cecilia|WMB3563-14|RVcoll.11-D931||Spain|658[0n]
Pyronia cecilia|WMB3005-14|RVcoll.12-O450|Sant Antioco|Italy|658[0n]
Pyronia cecilia|WMB1526-13|RVcoll.12-O220|Corsica|France|658[0n]
Pyronia cecilia|WMB4731-14|RVcoll.LD-3209|Corsica|France|658[0n]
Pyronia cecilia|WMB4608-14|RVcoll.LD-1451|Corsica|France|658[0n]
Pyronia cecilia|WMB1707-13|RVcoll.12-P646||France|658[0n]
Pyronia cecilia|EZSPC1215-10|RVcoll.09-V512|Genit. examined|Spain|658[0n]
Pyronia cecilia|WMB665-12|RVcoll.09-T595.1||Italy|658[0n]
Pyronia cecilia|WMB2838-13|RVcoll.12-Q490|Sardinia|Italy|658[0n]
Pyronia cecilia|EZSPC1237-10|RVcoll.09-V721|Genit. examined|Spain|658[0n]
Pyronia cecilia|EZSPM054-09|RVcoll.08-P275|Genit. examined|Spain|658[0n]
Pyronia cecilia|EZSPM888-12|Rvcoll.12-M606||Spain|658[0n]
Pyronia cecilia|WMB2962-14|RVcoll.12-N673|Menorca|Spain|658[0n]
Pyronia cecilia|EZSPM066-09|RVcoll.08-P299||Spain|658[0n]
Pyronia cecilia|EZSPN316-09|RVcoll.08-H175|Genit. examined|Spain|658[0n]
Pyronia cecilia|WMB1808-13|RVcoll.11-I349|Giglio|Italy|658[0n]
Pyronia cecilia|WMB6071-18|RVcoll14A104||Italy|658[0n]
Pyronia cecilia|WMB1807-13|RVcoll.11-I348|Giglio|Italy|658[0n]
Pyronia cecilia|WMB2788-13|RVcoll.11-I352|Giglio|Italy|658[0n]
Pyronia cecilia|WMB121-11|RVcoll.10-C182||France|658[0n]
Pyronia cecilia|WMB1525-13|RVcoll.12-O204|Corsica|France|658[0n]
Pyronia cecilia|BIBSA982-15|14-W379||Italy|658[0n]
Pyronia cecilia|WMB2926-14|RVcoll.11-I381|Argentario|Italy|658[0n]
Pyronia cecilia|WMB2925-14|RVcoll.11-I380|Argentario|Italy|658[0n]
Pyronia cecilia|WMB1765-13|RVcoll.12-Q007|Levant|France|658[0n]
Pyronia cecilia|WMB3480-14|RVcoll.10-B505||Spain|658[0n]
Pyronia cecilia|WMB4605-14|RVcoll.LD-1414|Corsica|France|658[0n]
Pyronia cecilia|WMB5245-14|RVcoll.140410KV30|Genit. examined|Spain|658[0n]
Pyronia cecilia|WMB1546-13|RVcoll.12-O313|La Maddalena|Italy|658[0n]
Pyronia cecilia|WMB1551-13|RVcoll.12-O328|Caprera|Italy|658[0n]
Pyronia cecilia|WMB1679-13|RVcoll.12-P450||France|658[0n]
Pyronia cecilia|WMB1764-13|RVcoll.12-Q006|Levant|France|658[0n]
Pyronia cecilia|WMB2638-13|RVcoll.09-T517|Corsica|France|658[0n]
Pyronia cecilia|WMB2647-13|RVcoll.12-R338|Elba|Italy|658[0n]
Pyronia cecilia|WMB2648-13|RVcoll.12-R339|Elba|Italy|658[0n]
Pyronia cecilia|WMB3901-14|RVcoll.12-P269||France|658[0n]
Pyronia cecilia|WMB3903-14|RVcoll.12-P293||France|658[0n]
Pyronia cecilia|BIBSA1234-15|15-N076||Italy|658[0n]
Pyronia cecilia|OXB990-15|15-A940||Italy|658[0n]
Pyronia cecilia|OXB303-15|RVcoll.14-N017||Italy|658[0n]
Pyronia cecilia|WMB656-11|RVcoll.11-I287|Pianosa|Italy|658[0n]
Pyronia cecilia|WMB4411-14|RVcoll.14-B450||Portugal|658[0n]
Pyronia cecilia|BIBSA908-15|LD-3520||Italy|658[0n]
Pyronia cecilia|OXB991-15|15-A941||Italy|658[0n]
Pyronia cecilia|WMB2823-13|RVcoll.12-O334|Caprera|Italy|658[0n]
Pyronia cecilia|WMB2822-13|RVcoll.12-O310|La Maddalena|Italy|658[0n]
Pyronia cecilia|WMB3007-14|RVcoll.12-O558|Sardinia|Italy|658[0n]
Pyronia cecilia|WMB087-11|RVcoll.LD-3148|Elba|Italy|658[0n]
Pyronia cecilia|WMB065-11|RVcoll.LD-2613|Elba|Italy|658[0n]
Pyronia cecilia|WMB064-11|RVcoll.LD-2612|Elba|Italy|658[0n]
Pyronia cecilia|EZSPC1028-10|RVcoll.08-J077|Genit. examined|Spain|658[0n]
Pyronia cecilia|WMB4456-14|RVcoll.14-D323||Spain|658[0n]
Pyronia cecilia|EZSPM309-09|RVcoll.08-J858||Spain|658[0n]

Pyronia cecilia|[illegible]|RVcoll.LD-2012|Elba|Italy|658[0n]
Pyronia cecilia|EZSPC1028-10|RVcoll.08-J077|Genit. examined|Spain|658[0n]
Pyronia cecilia|WMB4456-14|RVcoll.14-D323||Spain|658[0n]
Pyronia cecilia|EZSPM309-09|RVcoll.08-J858||Spain|658[0n]
Pyronia cecilia|EZSPM929-12|Rvcoll.12-M655||Spain|658[0n]
Pyronia cecilia|EZSPM963-12|Rvcoll.12-M714||Spain|658[0n]
Pyronia cecilia|EZSPN805-09|RVcoll.08-L463|Genit. examined|Spain|658[0n]
Pyronia cecilia|WMB5162-14|RVcoll.12-R434||Italy|658[0n]
Pyronia cecilia|WMB4877-14|RVcoll.14-I424||Italy|658[0n]
Pyronia cecilia|WMB1811-13|RVcoll.11-I379||Italy|658[0n]
Pyronia cecilia|WMB542-11|RVcoll.11-H710|Levanzo|Italy|658[0n]
Pyronia cecilia|WMB5928-17|RVcoll11H713|Levanzo|Italy|658[0n]
Pyronia cecilia|WMB1947-13|RVcoll.11-H889|Salina|Italy|658[0n]
Pyronia cecilia|WMB2775-13|RVcoll.11-H893|Salina|Italy|658[0n]
Pyronia cecilia|WMB2766-13|RVcoll.11-H814|Vulcano|Italy|658[0n]
Pyronia cecilia|WMB2761-13|RVcoll.11-H717|Levanzo|Italy|658[0n]
Pyronia cecilia|BIBSA1743-16|14-A699||Italy|658[0n]
Pyronia cecilia|WMB4601-14|RVcoll.LD-1340|Lipari|Italy|658[0n]
Pyronia cecilia|WMB1922-13|RVcoll.11-H712|Levanzo|Italy|658[0n]
Pyronia cecilia|WMB5281-14|RVcoll.14-A702||Italy|658[0n]
Pyronia cecilia|WMB2078-13|RVcoll.12-Q991|Sicily|Italy|658[0n]
Pyronia cecilia|WMB5927-17|RVcoll11H711|Levanzo|Italy|658[0n]
Pyronia cecilia|WMB2069-13|RVcoll.12-Q910||Italy|658[0n]
Pyronia cecilia|WMB1934-13|RVcoll.11-H817|Vulcano|Italy|658[0n]
Pyronia cecilia|BIBSA1604-16|12-R435||Italy|658[0n]
Pyronia cecilia|WMB2235-13|RVcoll.11-Y098|Sicily|Italy|658[0n]
Pyronia cecilia|WMB2358-13|RVcoll.12-Q911||Italy|658[0n]
Pyronia cecilia|WMB4607-14|RVcoll.LD-1435|Lipari|Italy|658[0n]
Pyronia cecilia|BIBSA617-15|LEP-SS-00237||Italy|658[0n]
Pyronia cecilia|WMB1948-13|RVcoll.11-H890|Salina|Italy|633[0n]
Pyronia cecilia|WMB4324-14|RVcoll.14-A655||Italy|620[0n]
Pyronia cecilia|WMB2459-13|RVcoll.11-H285|Genit. examined|Italy|627[0n]
Pyronia cecilia|WMB1935-13|RVcoll.11-H818|Vulcano|Italy|632[0n]
Pyronia cecilia|WMB1061-13|RVcoll.10-C681|Lipari|Italy|622[0n]
Pyronia cecilia|BIBSA1721-16|LD-2063||Italy|658[0n]
Pyronia cecilia|BIBSA1656-16|15-C685||Italy|658[0n]
Pyronia cecilia|WMB3012-14|RVcoll.12-Q989|Sicily|Italy|658[0n]
Pyronia cecilia|BIBSA711-15|RVcoll. 14-A701||Italy|658[0n]
Pyronia cecilia|WMB048-11|RVcoll.LD-2066||Italy|658[0n]
Pyronia cecilia|WMB4598-14|RVcoll.LD-1205||Italy|658[0n]
Pyronia cecilia|WMB3034-14|RVcoll.13-S551|San Domino|Italy|658[0n]
Pyronia cecilia|BIBSA1393-15|15-M992||Italy|658[0n]
Pyronia cecilia|WMB3033-14|RVcoll.13-S550|San Domino|Italy|658[0n]
Pyronia cecilia|BIBSA710-15|RVcoll. 14-A700||Italy|658[0n]
Pyronia cecilia|WMB4141-14|RVcoll.13-S656||Italy|658[0n]
Pyronia cecilia|BIBSA1043-15|15-C226||Italy|658[0n]
Pyronia cecilia|BIBSA1387-15|15-M983||Italy|658[0n]
Pyronia cecilia|WMB6582-18|RVcoll14A081||Italy|658[0n]
Pyronia cecilia|BIBSA647-15|RVcoll. 15-A810||Italy|638[0n]
Pyronia cecilia|WMB4602-14|RVcoll.LD-1380||Italy|630[0n]
Pyronia cecilia|WMB4600-14|RVcoll.LD-1303|Capri|Italy|658[0n]
Pyronia cecilia|WMB4303-14|RVcoll.14-A402||Italy|658[0n]
Pyronia cecilia|WMB911-13|RVcoll.09-X900|Capri|Italy|658[0n]
Pyronia cecilia|WMB910-13|RVcoll.09-X899|Capri|Italy|658[0n]
Pyronia cecilia|GWORZ036-10|BC ZSM Lep 30392||Italy|658[0n]
Pyronia bathseba|WMB3389-14|RVcoll.09-V479||Spain|658[0n]
Pyronia bathseba|EZSPM941-12|Rvcoll.12-M675||Spain|658[0n]
Pyronia bathseba|WMB3569-14|RVcoll.11-D986||Spain|658[0n]
Pyronia bathseba|WMB3707-14|RVcoll.12-L582||Portugal|658[0n]
Pyronia bathseba|EZSPN1051-11|Rvcoll.08-L462||Spain|658[0n]
Pyronia bathseba|EZSPN706-09|RVcoll.08-L055||Spain|658[0n]
Pyronia bathseba|WMB265-11|RVcoll.10-A422||France|658[0n]
Pyronia bathseba|EZSPM907-12|Rvcoll.12-M629||Spain|658[0n]
Pyronia bathseba|WMB3286-14|RVcoll.08-M686||Spain|658[0n]
Pyronia bathseba|EZSPN632-09|RVcoll.08-J732||Spain|658[0n]
Pyronia bathseba|EZSPN481-09|RVcoll.08-H918||Spain|658[0n]
Pyronia bathseba|WMB927-13|RVcoll.10-A463||France|658[0n]
Pyronia bathseba|WMB3398-14|RVcoll.09-V720||Spain|658[0n]
Pyronia bathseba|EZSPN1058-11|Rvcoll.08-P339||Spain|658[0n]
Pyronia bathseba|EZSPM927-12|Rvcoll.12-M650||Spain|658[0n]
Pyronia bathseba|EZSPM964-12|Rvcoll.12-M716||Spain|658[0n]
Pyronia bathseba|WMB3567-14|RVcoll.11-D970||Spain|658[0n]
Pyronia bathseba|WMB3217-14|RVcoll.08-J710||Spain|605[0n]
Pyronia bathseba|EZSPN975-09|RVcoll.08-M000||Spain|636[0n]
Pyronia bathseba|EZSPN398-09|RVcoll.08-H463||Spain|658[0n]
Pyronia bathseba|WMB3549-14|RVcoll.11-D875||Spain|658[0n]
Pyronia bathseba|EZSPN083-09|RVcoll.06-A000||Spain|658[0n]
Pyronia bathseba|EZSPM1017-12|Rvcoll.12-M766||Spain|658[0n]
Pyronia bathseba|EZSPN434-09|RVcoll.08-H612||Spain|658[0n]
Pyronia bathseba|EZSPN527-09|RVcoll.08-H997||Spain|658[0n]
Pyronia bathseba|EZSPM1018-12|Rvcoll.12-M767||Spain|658[0n]
Pyronia bathseba|EZSPM358-09|RVcoll.08-R050||Spain|658[0n]
Pyronia bathseba|EZSPM310-09|RVcoll.08-J863||Spain|658[0n]
Pyronia bathseba|WMB3474-14|RVcoll.10-A857||Spain|658[0n]
Pyronia bathseba|WMB3737-14|RVcoll.12-M654||Spain|658[0n]
Pyronia bathseba|WMB1697-13|RVcoll.12-P577||France|658[0n]
Pyronia bathseba|EZSPN340-09|RVcoll.08-H298||Spain|658[0n]
Pyronia bathseba|EZSPN133-09|RVcoll.06-G482||Spain|658[0n]
Pyronia tithonus|EZSPM436-09|Rvcoll.09-V949|Genit. examined|Spain|658[0n]
Pyronia tithonus|EZSPN768-09|RVcoll.08-L272||Spain|658[0n]
Pyronia tithonus|EZSPC302-09|RVcoll.08-R156||Spain|658[0n]
Pyronia tithonus|BIBSA917-15|16-A008||France|658[0n]
Pyronia tithonus|EZSPC303-09|RVcoll.08-P369||Spain|658[0n]
Pyronia tithonus|EZSPN586-09|RVcoll.08-J148||Portugal|627[0n]
Pyronia tithonus|EZSPN467-09|RVcoll.08-H685|Genit. examined|Spain|658[0n]
Pyronia tithonus|EZSPC305-09|RVcoll.08-P320||Spain|658[0n]
Pyronia tithonus|BIBSA1244-15|15-F823||France|658[0n]
Pyronia tithonus|WMB5303-14|RVcoll.14-I625||France|658[0n]
Pyronia tithonus|WMB4419-14|RVcoll.14-B469||Portugal|658[0n]
Pyronia tithonus|WMB4402-14|RVcoll.14-B416||Portugal|658[0n]
Pyronia tithonus|WMB3284-14|RVcoll.08-M679|Genit. examined|Spain|658[0n]
Pyronia tithonus|WMB3270-14|RVcoll.08-L929||Spain|658[0n]
Pyronia tithonus|WMB3228-14|RVcoll.08-J864.1||Spain|658[0n]

Pyronia tithonus|WMB3284-14|RVcoll.08-M679|Genit. examined|Spain|658[0n]
Pyronia tithonus|WMB3270-14|RVcoll.08-L929||Spain|658[0n]
Pyronia tithonus|WMB3228-14|RVcoll.08-J864.1||Spain|658[0n]
Pyronia tithonus|WMB3442-14|RVcoll.09-X557||Spain|658[0n]
Pyronia tithonus|EZSPM216-09|RVcoll.08-R402||Spain|658[0n]
Pyronia tithonus|EZSPC304-09|RVcoll.08-P040||Spain|658[0n]
Pyronia tithonus|EZSPM084-09|RVcoll.08-P406|Genit. examined|Spain|658[0n]
Pyronia tithonus|EZSPN508-09|RVcoll.08-H966||Spain|658[0n]
Pyronia tithonus|EZSPC306-09|RVcoll.08-P021|Genit. examined|Spain|658[0n]
Pyronia tithonus|EZSPN838-09|RVcoll.08-L664||Spain|658[0n]
Pyronia tithonus|WMB4606-14|RVcoll.LD-1420|Corsica|France|658[0n]
Pyronia tithonus|WMB037-11|RVcoll.LD-1395||Italy|658[0n]
Pyronia tithonus|OXB784-15|14-A761||Italy|658[0n]
Pyronia tithonus|BIBSA518-15|LEP-SS-00138||Italy|658[0n]
Pyronia tithonus|WMB3969-14|RVcoll.12-Q136||France|614[0n]
Pyronia tithonus|WMB1682-13|RVcoll.12-P496||France|658[0n]
Pyronia tithonus|WMB5372-14|RVcoll.14-J772||France|658[0n]
Pyronia tithonus|WMB853-13|RVcoll.08-R302|Genit. examined|France|610[0n]
Pyronia tithonus|WMB3404-14|RVcoll.09-V819||Spain|658[0n]
Pyronia tithonus|OXB1311-15|Rvcoll_15-M575||France|658[0n]
Pyronia tithonus|OXB1338-15|Rvcoll_15-M684||France|658[0n]
Pyronia tithonus|OXB225-15|OXB-TGS-436||United Kingdom|658[0n]
Pyronia tithonus|OXB1408-15|Rvcoll_15-M781||France|658[0n]
Pyronia tithonus|OXB1394-15|Rvcoll_15-M761||France|658[0n]
Pyronia tithonus|OXB1362-15|Rvcoll_15-M716||France|658[0n]
Pyronia tithonus|OXB1227-15|Rvcoll_15-M660||France|658[0n]
Pyronia tithonus|OXB1219-15|Rvcoll_15-M645||France|658[0n]
Pyronia tithonus|OXB1341-15|Rvcoll_15-M689||France|658[0n]
Pyronia tithonus|OXB1326-15|Rvcoll_15-M598||France|658[0n]
Pyronia tithonus|GBLAB151-13|BC ZSM Lep 75777||Germany|658[0n]
Pyronia tithonus|EULEP4848-16|RVcoll16H764||Bulgaria|658[0n]
Pyronia tithonus|OXB901-15|15-A545||Italy|658[0n]
Pyronia tithonus|EULEP3129-15|RVcoll.14-O262||Greece|658[0n]
Pyronia tithonus|WMB3643-14|RVcoll.11-I570||Spain|658[0n]
Pyronia tithonus|WMB3495-14|RVcoll.10-B697||France|658[0n]
Pyronia tithonus|WMB1529-13|RVcoll.12-O223|Corsica|France|658[0n]
Pyronia tithonus|WMB1528-13|RVcoll.12-O222|Corsica|France|658[0n]
Pyronia tithonus|WMB1896-13|RVcoll.11-J964|Sardinia|Italy|658[0n]
Pyronia tithonus|WMB2068-13|RVcoll.12-Q898||Italy|658[0n]
Pyronia tithonus|WMB3891-14|RVcoll.12-O914||France|658[0n]
Pyronia tithonus|WMB2649-13|RVcoll.12-R340|Elba|Italy|658[0n]
Pyronia tithonus|WMB4011-14|RVcoll.12-Q863||Italy|658[0n]
Pyronia tithonus|WMB5247-14|RVcoll.14-A196||Italy|658[0n]
Pyronia tithonus|EULEP879-15|RVcoll.14-C455||Greece|658[0n]
Pyronia tithonus|EULEP1476-15|RVcoll.14-G257||Greece|658[0n]
Pyronia tithonus|EULEP1888-15|RVcoll.14-B698||Albania|658[0n]
Pyronia tithonus|BIBSA1292-15|14-E283|Corsica|France|658[0n]
Pyronia tithonus|OXB575-15|14-N964||Italy|658[0n]
Pyronia tithonus|OXB592-15|14-N981||Italy|658[0n]
Pyronia tithonus|OXB1539-16|OXB-TGS-1257||France|658[0n]
Pyronia tithonus|OXB214-15|OXB-TGS-338||United Kingdom|658[0n]
Pyronia tithonus|OXB1049-15|LD-1422||Italy|640[0n]
Pyronia tithonus|EULEP4641-16|RVcoll10B330|Genit. examined|Bulgaria|648[0n]
Pyronia tithonus|WMB4732-14|RVcoll.LD-3211|Corsica|France|603[0n]
Pyronia tithonus|WMB081-11|RVcoll.LD-3138|Elba|Italy|616[0n]
Pyronia tithonus|WMB063-11|RVcoll.LD-2611|Elba|Italy|658[0n]
Pyronia tithonus|WMB039-11|RVcoll.LD-1397||Italy|658[0n]
Pyronia tithonus|WMB038-11|RVcoll.LD-1396||Italy|658[0n]
Pyronia tithonus|EZSPM437-09|Rvcoll.09-V950|Genit. examined|Spain|658[0n]
Pyronia tithonus|EZROM535-08|RV-07-E396||Romania|658[0n]
Pyronia tithonus|EZROM685-08|RV-07-E397||Romania|658[0n]
Pyronia tithonus|EZROM684-08|RV-07-E335|Genit. examined|Romania|658[0n]
Pyronia tithonus|EZRMN250-08|RVcoll.07-E392||Romania|658[0n]
Pyronia tithonus|EZRMN249-08|RVcoll.07-E358|Genit. examined|Romania|658[0n]
Pyronia tithonus|EZRMN248-08|RVcoll.07-E354|Genit. examined|Romania|658[0n]
Pyronia tithonus|EZROM534-08|RV-07-E308|Genit. examined|Romania|658[0n]
Pyronia tithonus|EZRMN247-08|RVcoll.07-E349|Genit. examined|Romania|658[0n]
Aphantopus hyperantus|EZSPC840-10|RVcoll.08-R193||Spain|658[0n]
Aphantopus hyperantus|PHLAF651-11|TLMF Lep 05821||Austria|658[0n]
Aphantopus hyperantus|ABOLD423-16|TLMF Lep 21575||Austria|658[0n]
Aphantopus hyperantus|EULEP2385-15|RVcoll.14-V082||Ukraine|611[0n]
Aphantopus hyperantus|OXB1310-15|Rvcoll_15-M574||France|612[0n]
Aphantopus hyperantus|EZROM333-08|RV-08-A011||Romania|658[0n]
Aphantopus hyperantus|EZSPC089-09|RVcoll.08-M931||Spain|658[0n]
Aphantopus hyperantus|EZROM335-08|RV-08-A013||Romania|658[0n]
Aphantopus hyperantus|EZSPC090-09|RVcoll.08-R269||Spain|658[0n]
Aphantopus hyperantus|EZSPN949-09|RVcoll.08-L939||Spain|658[0n]
Aphantopus hyperantus|EZSPC792-10|RVcoll.08-P093||Spain|658[0n]
Aphantopus hyperantus|EZSPC912-10|RVcoll.09-V812||Spain|658[0n]
Aphantopus hyperantus|EZSPC741-10|RVcoll.08-J859.1||Spain|658[0n]
Aphantopus hyperantus|EULEP3430-16|RVcoll15H176||Switzerland|658[0n]
Aphantopus hyperantus|EULEP3434-16|RVcoll15G182||France|658[0n]
Aphantopus hyperantus|EULEP043-14|RVcoll.08-H021||Romania|658[0n]
Aphantopus hyperantus|EULEP601-15|RVcoll.10-C334||Switzerland|658[0n]
Aphantopus hyperantus|EULEP1998-15|RVcoll.14-I774||Slovakia|658[0n]
Aphantopus hyperantus|EULEP2330-15|RVcoll.14-V011||Denmark|658[0n]
Aphantopus hyperantus|OXB1436-16|OXB-TGS-1210||United Kingdom|658[0n]
Aphantopus hyperantus|OXB1437-16|OXB-TGS-1211||United Kingdom|658[0n]
Aphantopus hyperantus|OXB1438-16|OXB-TGS-1212||United Kingdom|658[0n]
Aphantopus hyperantus|OXB1439-16|OXB-TGS-1213||United Kingdom|658[0n]
Aphantopus hyperantus|OXB1440-16|OXB-TGS-1214||United Kingdom|658[0n]
Aphantopus hyperantus|OXB1516-16|RVcoll14W444||United Kingdom|658[0n]
Aphantopus hyperantus|OXB1517-16|RVcoll14W443||United Kingdom|658[0n]
Aphantopus hyperantus|OXB1518-16|RVcoll14W433||United Kingdom|658[0n]
Aphantopus hyperantus|OXB1525-16|OXB-TGS-1243||United Kingdom|658[0n]
Aphantopus hyperantus|OXB1298-15|Rvcoll_15-M165||France|658[0n]
Aphantopus hyperantus|OXB1333-15|Rvcoll_15-M676||France|658[0n]
Aphantopus hyperantus|OXB1201-15|Rvcoll_15-M617||France|658[0n]
Aphantopus hyperantus|OXB1260-15|Rvcoll_15-M116||France|658[0n]
Aphantopus hyperantus|OXB1226-15|Rvcoll_15-M659||France|613[0n]
Aphantopus hyperantus|OXB1441-16|OXB-TGS-1215||United Kingdom|645[0n]
Aphantopus hyperantus|EULEP2117-15|RVcoll.14-J874||France|624[0n]
Aphantopus hyperantus|EZSPC093-09|RVcoll.08-P094||Spain|627[0n]

Aphantopus hyperantus|OXB1441-16|OXB-TGS-1215||United Kingdom|645[0n]
Aphantopus hyperantus|EULEP2117-15|RVcoll.14-J874||France|624[0n]
Aphantopus hyperantus|EZSPC093-09|RVcoll.08-P094||Spain|627[0n]
Aphantopus hyperantus|EZROM015-08|RV-06-M894||Romania|609[0n]
Aphantopus hyperantus|EZROM766-08|RVcoll.08-M514||Romania|658[0n]
Aphantopus hyperantus|EZROM570-08|RV-07-C351||Romania|658[0n]
Aphantopus hyperantus|EZROM016-08|RV-06-M901||Romania|658[0n]
Aphantopus hyperantus|LEFIC025-10|MM03238||Finland|658[0n]
Aphantopus hyperantus|LEATG491-14|TLMF Lep 14278||Italy|658[0n]
Aphantopus hyperantus|ABOLD009-16|TLMF Lep 21087||Austria|658[0n]
Aphantopus hyperantus|EULEP4930-16|RVcoll16I003||Poland|658[0n]
Aphantopus hyperantus|EULEP791-15|RVcoll.12-Z226||Sweden|658[0n]
Aphantopus hyperantus|OXB1520-16|RVcoll14W431||United Kingdom|658[0n]
Aphantopus hyperantus|OXB1519-16|RVcoll14W432||United Kingdom|658[0n]
Aphantopus hyperantus|EULEP550-15|RVcoll.08-L316||Sweden|658[0n]
Aphantopus hyperantus|EULEP516-15|RVcoll.07-F527||Estonia|658[0n]
Aphantopus hyperantus|EULEP4733-16|RVcoll16G469||Sweden|658[0n]
Aphantopus hyperantus|EULEP3436-16|RVcoll15P005||Belarus|658[0n]
Aphantopus hyperantus|LON842-11|NHMO Lep09040||Norway|658[0n]
Aphantopus hyperantus|EULEP3429-16|RVcoll10A955||Bulgaria|658[0n]
Aphantopus hyperantus|OXB731-15|13-U212||Italy|658[0n]
Aphantopus hyperantus|EULEP2419-15|RVcoll.14-V198||Belgium|633[0n]
Aphantopus hyperantus|LEATG010-14|TLMF Lep 13797||Austria|658[0n]
Aphantopus hyperantus|GWORA2479-09|BC ZSM Lep 30691||Germany|606[0n]
Aphantopus hyperantus|LEASS493-17|TLMF Lep 22141||Austria|658[0n]
Aphantopus hyperantus|OXB331-15|RVcoll.14-N045||Italy|658[0n]
Aphantopus hyperantus|OXB352-15|RVcoll.14-N066||Italy|658[0n]
Aphantopus hyperantus|OXB198-15|OXB-TGS-151||United Kingdom|658[0n]
Aphantopus hyperantus|OXB278-15|OXB-TGS-961||United Kingdom|658[0n]
Aphantopus hyperantus|GBLAA1340-15|BC ZSM Lep 87036||Germany|632[2n]
Aphantopus hyperantus|BIBSA355-15|RVcoll.14-I023||Italy|658[0n]
Aphantopus hyperantus|BIBSA171-15|RVcoll.14-D959||Italy|658[0n]
Aphantopus hyperantus|BIBSA1163-15|15-M181||Italy|658[0n]
Aphantopus hyperantus|BIBSA1121-15|15-L870||Italy|658[0n]
Aphantopus hyperantus|EULEP1511-15|RVcoll.14-G429||Greece|658[0n]
Aphantopus hyperantus|EULEP881-15|RVcoll.14-C464||Greece|658[0n]
Aphantopus hyperantus|EULEP4973-16|RVcoll16I428||Poland|658[0n]
Aphantopus hyperantus|EULEP3431-16|RVcoll15I354||Austria|658[0n]
Aphantopus hyperantus|EZSPC826-10|RVcoll.08-P774||Spain|658[0n]
Aphantopus hyperantus|EZSPC825-10|RVcoll.08-P770||Spain|658[0n]
Aphantopus hyperantus|EZSPC800-10|RVcoll.08-P375||Spain|658[0n]
Aphantopus hyperantus|EZSPM082-09|RVcoll.08-P402||Spain|658[0n]
Aphantopus hyperantus|EZROM354-08|RV-08-A032||Romania|658[0n]
Aphantopus hyperantus|FBLMT907-09|BC ZSM Lep 25467||Germany|658[0n]
Aphantopus hyperantus|GWORA2478-09|BC ZSM Lep 30690||Germany|658[0n]
Aphantopus hyperantus|LEATG492-14|TLMF Lep 14279||Italy|658[0n]
Aphantopus hyperantus|LEATG063-14|TLMF Lep 13850||Austria|600[0n]
Aphantopus hyperantus|GWORA2477-09|BC ZSM Lep 30689||Germany|658[0n]
Aphantopus hyperantus|EZSPC091-09|RVcoll.08-R192||Spain|658[0n]
Aphantopus hyperantus|EULEP618-15|RVcoll.11-H183||Germany|658[0n]
Aphantopus hyperantus|OXB220-15|OXB-TGS-386||United Kingdom|658[0n]
Aphantopus hyperantus|OXB1407-15|Rvcoll_15-M778||France|658[0n]
Aphantopus hyperantus|EULEP4968-16|RVcoll16I389||Poland|639[0n]
Aphantopus hyperantus|EULEP4952-16|RVcoll16I186||Germany|606[0n]
Aphantopus hyperantus|EULEP5072-16|RVcoll16J196||Czech Republic|658[0n]
Aphantopus hyperantus|EULEP3437-16|RVcoll15P031||Ukraine|658[0n]
Aphantopus hyperantus|EULEP3433-16|RVcoll15G088||France|658[0n]
Aphantopus hyperantus|EULEP3432-16|RVcoll15G051||France|658[0n]
Aphantopus hyperantus|LEFIJ559-10|MM17184||Finland|658[0n]
Aphantopus hyperantus|LEATH762-14|TLMF Lep 15974||Italy|658[0n]
Maniola jurtina|OXB547-15|OXB-TGS-922||United Kingdom|658[0n]
Maniola jurtina|OXB546-15|OXB-TGS-921||United Kingdom|658[0n]
Maniola jurtina|OXB545-15|OXB-TGS-920||United Kingdom|658[0n]
Maniola jurtina|OXB442-15|OXB-TGS-174||United Kingdom|658[0n]
Maniola jurtina|OXB1485-16|RVcoll14W401||United Kingdom|658[0n]
Maniola jurtina|OXB1484-16|RVcoll14W402||United Kingdom|658[0n]
Maniola jurtina|OXB1483-16|RVcoll14W403||United Kingdom|658[0n]
Maniola jurtina|OXB1481-16|RVcoll14W406||United Kingdom|658[0n]
Maniola cypricola|EULEP4203-16|RVcoll14A928||Cyprus|658[0n]
Maniola jurtina|OXB450-15|OXB-TGS-267||United Kingdom|658[0n]
Maniola jurtina|OXB056-14|OXB_ACH-0048|(imago)|United Kingdom|658[0n]
Maniola jurtina|OXB181-14|OXB_ACH-0173|(imago)|United Kingdom|658[0n]
Maniola jurtina|OXB040-14|OXB_ACH-0032|(imago)|United Kingdom|658[0n]
Maniola jurtina|OXB033-14|OXB_ACH-0025|(imago)|United Kingdom|658[0n]
Maniola jurtina|OXB031-14|OXB_ACH-0023|(imago)|United Kingdom|658[0n]
Maniola jurtina|OXB567-15|OXB-TGS-942||United Kingdom|658[0n]
Maniola jurtina|OXB520-15|OXB-TGS-776||United Kingdom|658[0n]
Maniola jurtina|OXB519-15|OXB-TGS-775||United Kingdom|658[0n]
Maniola jurtina|OXB518-15|OXB-TGS-774||United Kingdom|658[0n]
Maniola jurtina|OXB481-15|OXB-TGS-470||United Kingdom|658[0n]
Maniola jurtina|OXB112-14|OXB_ACH-0104|(imago)|United Kingdom|658[0n]
Maniola jurtina|OXB110-14|OXB_ACH-0102|(imago)|United Kingdom|658[0n]
Maniola jurtina|OXB109-14|OXB_ACH-0101|(imago)|United Kingdom|658[0n]
Maniola jurtina|OXB073-14|OXB_ACH-0065|(imago)|United Kingdom|658[0n]
Maniola jurtina|OXB1479-16|RVcoll14W424||United Kingdom|658[0n]
Maniola jurtina|WMB6051-18|RVcoll13T354||Albania|658[0n]
Maniola jurtina|WMB6052-18|RVcoll14A016||Italy|658[0n]
Maniola jurtina|OXB1403-15|Rvcoll_15-M772||France|658[0n]
Maniola jurtina|EULEP1198-15|RVcoll.14-F477||Bulgaria|658[0n]
Maniola jurtina|WMB6033-18|RVcoll11H160||Germany|658[0n]
Maniola jurtina|WMB4446-14|RVcoll.14-D230||Austria|658[0n]
Maniola jurtina|LEFID787-10|MM06856||Finland|658[0n]
Maniola telmessia|EULEP675-15|RVcoll.11-J516|Karpathos|Greece|658[0n]
Maniola telmessia|WMB3865-14|RVcoll.11-J512|Karpathos|Greece|658[0n]
Maniola halicarnassus|WMB3863-14|RVcoll.11-J484|Nisyros|Greece|658[0n]
Maniola cypricola|OXB288-15|RVcoll.14-N002|Cyprus|Cyprus|658[0n]
Maniola cypricola|OXB290-15|RVcoll.14-N004|Cyprus|Cyprus|658[0n]
Maniola cypricola|EULEP4205-16|RVcoll14A930||Cyprus|658[0n]
Maniola halicarnassus|EULEP664-15|RVcoll.11-J489|Nisyros. Genit. examined|Greece|658[0n]
Maniola telmessia|EULEP731-15|RVcoll.12-N829|Lesvos|Greece|658[0n]
Maniola telmessia|EULEP5672-17|RVcoll16J794|Ikaria|Greece|658[0n]
Maniola telmessia|EULEP662-15|RVcoll.11-J478|Kos|Greece|658[0n]
[illegible]

Maniola telmessia|EULEP751-15|RVcoll.12-N829|Lesvos|Greece|658[0n]
Maniola telmessia|EULEP5672-17|RVcoll16J794|Ikaria|Greece|658[0n]
Maniola telmessia|EULEP662-15|RVcoll.11-J478|Kos|Greece|658[0n]
Maniola telmessia|EULEP676-15|RVcoll.11-J527|Symi|Greece|658[0n]
Maniola cypricola|OXB286-15|RVcoll.14-N000|Cyprus|Cyprus|658[0n]
Maniola cypricola|OXB287-15|RVcoll.14-N001|Cyprus|Cyprus|658[0n]
Maniola cypricola|OXB289-15|RVcoll.14-N003|Cyprus|Cyprus|658[0n]
Maniola cypricola|EULEP4879-16|RVcoll16H972||Cyprus|644[0n]
Maniola cypricola|EULEP4204-16|RVcoll14A929||Cyprus|658[0n]
Maniola telmessia|EULEP669-15|RVcoll.11-J502|Tilos|Greece|658[0n]
Maniola telmessia|WMB3866-14|RVcoll.11-J526|Symi|Greece|658[0n]
Maniola halicarnassus|EULEP665-15|RVcoll.11-J490|Nisyros|Greece|658[0n]
Maniola halicarnassus|EULEP663-15|RVcoll.11-J488|Nisyros|Greece|658[0n]
Maniola telmessia|WMB3864-14|RVcoll.11-J503|Tilos|Greece|658[0n]
Maniola telmessia|WMB3862-14|RVcoll.11-J468|Kos|Greece|658[0n]
Maniola nurag|EULEP746-15|RVcoll.12-O494|Sardinia|Italy|658[0n]
Maniola nurag|WMB1890-13|RVcoll.11-J873|Sardinia|Italy|658[0n]
Maniola nurag|WMB1584-13|RVcoll.12-O566|Sardinia|Italy|658[0n]
Maniola nurag|EULEP747-15|RVcoll.12-O540|Sardinia|Italy|658[0n]
Maniola nurag|WMB1572-13|RVcoll.12-O514|Sardinia|Italy|622[0n]
Maniola megala|EULEP774-15|RVcoll.12-R940|Lesvos|Greece|658[0n]
Maniola megala|EULEP773-15|RVcoll.12-R938|Lesvos|Greece|658[0n]
Maniola megala|WMB6054-18|RVcoll13T359|Lesvos|Greece|658[0n]
Maniola megala|WMB6053-18|RVcoll13T358|Lesvos|Greece|658[0n]
Maniola jurtina|OXB466-15|OXB-TGS-409||United Kingdom|658[0n]
Maniola jurtina|OXB120-14|OXB_ACH-0112|(imago)|United Kingdom|658[0n]
Maniola jurtina|OXB055-14|OXB_ACH-0047|(imago)|United Kingdom|658[0n]
Maniola jurtina|OXB066-14|OXB_ACH-0058|(imago)|United Kingdom|658[0n]
Maniola jurtina|OXB128-14|OXB_ACH-0120|(imago)|United Kingdom|658[0n]
Maniola jurtina|OXB016-14|OXB_ACH-0008||United Kingdom|658[0n]
Maniola jurtina|OXB012-14|OXB_ACH-0004||United Kingdom|658[0n]
Maniola jurtina|OXB091-14|OXB_ACH-0083|(imago)|United Kingdom|658[0n]
Maniola jurtina|OXB129-14|OXB_ACH-0121|(imago)|United Kingdom|658[0n]
Maniola jurtina|OXB127-14|OXB_ACH-0119|(imago)|United Kingdom|658[0n]
Maniola jurtina|OXB126-14|OXB_ACH-0118|(imago)|United Kingdom|658[0n]
Maniola jurtina|OXB125-14|OXB_ACH-0117|(imago)|United Kingdom|658[0n]
Maniola jurtina|OXB039-14|OXB_ACH-0031|(imago)|United Kingdom|658[0n]
Maniola jurtina|OXB038-14|OXB_ACH-0030|(imago)|United Kingdom|658[0n]
Maniola jurtina|OXB034-14|OXB_ACH-0026|(imago)|United Kingdom|658[0n]
Maniola jurtina|OXB029-14|OXB_ACH-0021|(imago)|United Kingdom|658[0n]
Maniola jurtina|OXB028-14|OXB_ACH-0020|(imago)|United Kingdom|658[0n]
Maniola jurtina|OXB027-14|OXB_ACH-0019|(imago)|United Kingdom|658[0n]
Maniola jurtina|OXB026-14|OXB_ACH-0018|(imago)|United Kingdom|658[0n]
Maniola jurtina|OXB025-14|OXB_ACH-0017|(imago)|United Kingdom|658[0n]
Maniola jurtina|OXB024-14|OXB_ACH-0016|(imago)|United Kingdom|658[0n]
Maniola jurtina|OXB023-14|OXB_ACH-0015|(imago)|United Kingdom|658[0n]
Maniola jurtina|OXB022-14|OXB_ACH-0014|(imago)|United Kingdom|658[0n]
Maniola jurtina|OXB021-14|OXB_ACH-0013|(imago)|United Kingdom|658[0n]
Maniola jurtina|OXB020-14|OXB_ACH-0012|(imago)|United Kingdom|658[0n]
Maniola jurtina|OXB019-14|OXB_ACH-0011|(imago)|United Kingdom|658[0n]
Maniola jurtina|OXB018-14|OXB_ACH-0010|(imago)|United Kingdom|658[0n]
Maniola jurtina|OXB017-14|OXB_ACH-0009|(imago)|United Kingdom|658[0n]
Maniola jurtina|OXB015-14|OXB_ACH-0007||United Kingdom|658[0n]
Maniola jurtina|OXB009-14|OXB_ACH-0001||United Kingdom|658[0n]
Maniola jurtina|OXB560-15|OXB-TGS-935||United Kingdom|658[0n]
Maniola jurtina|OXB557-15|OXB-TGS-932||United Kingdom|658[0n]
Maniola jurtina|OXB555-15|OXB-TGS-930||United Kingdom|658[0n]
Maniola jurtina|OXB533-15|OXB-TGS-888||United Kingdom|658[0n]
Maniola jurtina|OXB515-15|OXB-TGS-768||United Kingdom|658[0n]
Maniola jurtina|OXB508-15|OXB-TGS-703||United Kingdom|658[0n]
Maniola jurtina|OXB507-15|OXB-TGS-702||United Kingdom|658[0n]
Maniola jurtina|OXB498-15|OXB-TGS-663||United Kingdom|658[0n]
Maniola jurtina|OXB495-15|OXB-TGS-622||United Kingdom|658[0n]
Maniola jurtina|OXB494-15|OXB-TGS-621||United Kingdom|658[0n]
Maniola jurtina|OXB493-15|OXB-TGS-620||United Kingdom|658[0n]
Maniola jurtina|OXB483-15|OXB-TGS-472||United Kingdom|658[0n]
Maniola jurtina|OXB482-15|OXB-TGS-471||United Kingdom|658[0n]
Maniola jurtina|OXB474-15|OXB-TGS-428||United Kingdom|658[0n]
Maniola jurtina|OXB473-15|OXB-TGS-427||United Kingdom|658[0n]
Maniola jurtina|OXB472-15|OXB-TGS-426||United Kingdom|658[0n]
Maniola jurtina|OXB471-15|OXB-TGS-425||United Kingdom|658[0n]
Maniola jurtina|OXB470-15|OXB-TGS-424||United Kingdom|658[0n]
Maniola jurtina|OXB468-15|OXB-TGS-411||United Kingdom|658[0n]
Maniola jurtina|OXB467-15|OXB-TGS-410||United Kingdom|658[0n]
Maniola jurtina|OXB463-15|OXB-TGS-401||United Kingdom|658[0n]
Maniola jurtina|OXB453-15|OXB-TGS-270||United Kingdom|658[0n]
Maniola jurtina|OXB452-15|OXB-TGS-269||United Kingdom|658[0n]
Maniola jurtina|OXB451-15|OXB-TGS-268||United Kingdom|658[0n]
Maniola jurtina|OXB449-15|OXB-TGS-266||United Kingdom|658[0n]
Maniola jurtina|OXB445-15|OXB-TGS-177||United Kingdom|658[0n]
Maniola jurtina|OXB118-14|OXB_ACH-0110|(imago)|United Kingdom|658[0n]
Maniola jurtina|OXB115-14|OXB_ACH-0107|(imago)|United Kingdom|658[0n]
Maniola jurtina|OXB114-14|OXB_ACH-0106|(imago)|United Kingdom|658[0n]
Maniola jurtina|OXB111-14|OXB_ACH-0103|(imago)|United Kingdom|658[0n]
Maniola jurtina|OXB106-14|OXB_ACH-0098|(imago)|United Kingdom|658[0n]
Maniola jurtina|OXB104-14|OXB_ACH-0096|(imago)|United Kingdom|658[0n]
Maniola jurtina|OXB102-14|OXB_ACH-0094|(imago)|United Kingdom|658[0n]
Maniola jurtina|OXB100-14|OXB_ACH-0092|(imago)|United Kingdom|658[0n]
Maniola jurtina|OXB098-14|OXB_ACH-0090|(imago)|United Kingdom|658[0n]
Maniola jurtina|OXB097-14|OXB_ACH-0089|(imago)|United Kingdom|658[0n]
Maniola jurtina|OXB096-14|OXB_ACH-0088|(imago)|United Kingdom|658[0n]
Maniola jurtina|OXB095-14|OXB_ACH-0087|(imago)|United Kingdom|658[0n]
Maniola jurtina|OXB094-14|OXB_ACH-0086|(imago)|United Kingdom|658[0n]
Maniola jurtina|OXB093-14|OXB_ACH-0085|(imago)|United Kingdom|658[0n]
Maniola jurtina|OXB092-14|OXB_ACH-0084|(imago)|United Kingdom|658[0n]
Maniola jurtina|OXB090-14|OXB_ACH-0082|(imago)|United Kingdom|658[0n]
Maniola jurtina|OXB089-14|OXB_ACH-0081|(imago)|United Kingdom|658[0n]
Maniola jurtina|OXB087-14|OXB_ACH-0079|(imago)|United Kingdom|658[0n]
Maniola jurtina|OXB086-14|OXB_ACH-0078|(imago)|United Kingdom|658[0n]
Maniola jurtina|OXB085-14|OXB_ACH-0077|(imago)|United Kingdom|658[0n]
Maniola jurtina|OXB084-14|OXB_ACH-0076|(imago)|United Kingdom|658[0n]
Maniola jurtina|OXB083-14|OXB_ACH-0075|(imago)|United Kingdom|658[0n]
Maniola jurtina|OXB082-14|OXB_ACH-0074|(imago)|United Kingdom|658[0n]

Maniola jurtina|OXB084-14|OXB_ACH-0076|(imago)|United Kingdom|658[0n]
Maniola jurtina|OXB083-14|OXB_ACH-0075|(imago)|United Kingdom|658[0n]
Maniola jurtina|OXB082-14|OXB_ACH-0074|(imago)|United Kingdom|658[0n]
Maniola jurtina|OXB079-14|OXB_ACH-0071|(imago)|United Kingdom|658[0n]
Maniola jurtina|OXB076-14|OXB_ACH-0068|(imago)|United Kingdom|658[0n]
Maniola jurtina|OXB075-14|OXB_ACH-0067|(imago)|United Kingdom|658[0n]
Maniola jurtina|OXB074-14|OXB_ACH-0066|(imago)|United Kingdom|658[0n]
Maniola jurtina|OXB070-14|OXB_ACH-0062|(imago)|United Kingdom|658[0n]
Maniola jurtina|OXB068-14|OXB_ACH-0060|(imago)|United Kingdom|658[0n]
Maniola jurtina|OXB067-14|OXB_ACH-0059|(imago)|United Kingdom|658[0n]
Maniola jurtina|OXB063-14|OXB_ACH-0055|(imago)|United Kingdom|658[0n]
Maniola jurtina|OXB062-14|OXB_ACH-0054|(imago)|United Kingdom|658[0n]
Maniola jurtina|OXB061-14|OXB_ACH-0053|(imago)|United Kingdom|658[0n]
Maniola jurtina|OXB060-14|OXB_ACH-0052|(imago)|United Kingdom|658[0n]
Maniola jurtina|OXB054-14|OXB_ACH-0046|(imago)|United Kingdom|658[0n]
Maniola jurtina|OXB052-14|OXB_ACH-0044|(imago)|United Kingdom|658[0n]
Maniola jurtina|OXB050-14|OXB_ACH-0042|(imago)|United Kingdom|658[0n]
Maniola jurtina|OXB047-14|OXB_ACH-0039|(imago)|United Kingdom|658[0n]
Maniola jurtina|OXB046-14|OXB_ACH-0038|(imago)|United Kingdom|658[0n]
Maniola jurtina|OXB043-14|OXB_ACH-0035|(imago)|United Kingdom|658[0n]
Maniola jurtina|OXB392-15|OXB-TGS-028||United Kingdom|658[0n]
Maniola jurtina|OXB386-15|OXB-TGS-014||United Kingdom|658[0n]
Maniola jurtina|OXB1480-16|RVcoll14W423||United Kingdom|658[0n]
Maniola jurtina|OXB1477-16|RVcoll14W426||United Kingdom|658[0n]
Maniola jurtina|OXB1475-16|RVcoll14W441||United Kingdom|658[0n]
Maniola jurtina|OXB1473-16|RVcoll14W448||United Kingdom|658[0n]
Maniola jurtina|OXB1472-16|RVcoll14W449||United Kingdom|658[0n]
Maniola jurtina|WMB492-11|RVcoll.11-H539|Sicily|Italy|658[0n]
Maniola jurtina|WMB4345-14|RVcoll.14-A846||United Kingdom|658[0n]
Maniola jurtina|EZSPM137-09|RVcoll.08-P647||Spain|658[0n]
Maniola jurtina|OXB140-14|OXB_ACH-0132|(imago)|France|658[0n]
Maniola jurtina|GBLN5059-14|KM033892|Mallorca. Genit. examined|Spain|655[0n]
Maniola jurtina|GBLN5058-14|KM033891|Mallorca. Genit. examined|Spain|655[0n]
Maniola jurtina|WMB2657-13|RVcoll.12-M858|Mallorca|Spain|658[0n]
Maniola jurtina|WMB2656-13|RVcoll.12-M857|Mallorca|Spain|658[0n]
Maniola jurtina|WMB2655-13|RVcoll.12-M856|Mallorca|Spain|658[0n]
Maniola jurtina|GBLN5089-14|KM033922|Sardinia|Italy|655[0n]
Maniola jurtina|OXB143-14|OXB_ACH-0135|(imago)|France|658[0n]
Maniola jurtina|WMB237-11|RVcoll.09-T568||Italy|658[0n]
Maniola jurtina|OXB130-14|OXB_ACH-0122|(imago)|United Kingdom|658[0n]
Maniola jurtina|OXB565-15|OXB-TGS-940||United Kingdom|658[0n]
Maniola jurtina|OXB123-14|OXB_ACH-0115|(imago)|United Kingdom|658[0n]
Maniola jurtina|OXB564-15|OXB-TGS-939||United Kingdom|658[0n]
Maniola jurtina|OXB509-15|OXB-TGS-704||United Kingdom|658[0n]
Maniola jurtina|OXB113-14|OXB_ACH-0105|(imago)|United Kingdom|658[0n]
Maniola jurtina|OXB072-14|OXB_ACH-0064|(imago)|United Kingdom|658[0n]
Maniola jurtina|OXB059-14|OXB_ACH-0051|(imago)|United Kingdom|658[0n]
Maniola jurtina|WMB512-11|RVcoll.11-H599|Sicily|Italy|658[0n]
Maniola jurtina|EZSPM1037-12|Rvcoll.12-M788||Spain|658[0n]
Maniola jurtina|OXB691-15|12-M394|Sicily W|Italy|658[0n]
Maniola jurtina|GBLN5018-14|KM033851|Corsica|France|655[0n]
Maniola jurtina|WMB5295-14|RVcoll.14-I593||France|658[0n]
Maniola jurtina|WMB1710-13|RVcoll.12-P656||France|658[0n]
Maniola jurtina|GBLN5066-14|KM033899|Sardinia|Italy|655[0n]
Maniola jurtina|OXB1346-15|Rvcoll_15-M695||France|658[0n]
Maniola jurtina|OXB1202-15|Rvcoll_15-M618||France|658[0n]
Maniola jurtina|OXB189-14|OXB_ACH-0181|(imago)|United Kingdom|658[0n]
Maniola jurtina|OXB1332-15|Rvcoll_15-M674||France|658[0n]
Maniola jurtina|OXB1425-15|Rvcoll_15-M623||France|658[0n]
Maniola jurtina|BIBSA1251-15|15-F835||France|658[0n]
Maniola jurtina|OXB443-15|OXB-TGS-175||United Kingdom|658[0n]
Maniola jurtina|OXB133-14|OXB_ACH-0125|(imago)|France|658[0n]
Maniola jurtina|OXB141-14|OXB_ACH-0133|(imago)|France|658[0n]
Maniola jurtina|OXB132-14|OXB_ACH-0124|(imago)|France|658[0n]
Maniola jurtina|OXB160-14|OXB_ACH-0152|(imago)|France|658[0n]
Maniola jurtina|OXB159-14|OXB_ACH-0151|(imago)|France|658[0n]
Maniola jurtina|OXB150-14|OXB_ACH-0142|(imago)|France|658[0n]
Maniola jurtina|OXB149-14|OXB_ACH-0141|(imago)|France|658[0n]
Maniola jurtina|OXB148-14|OXB_ACH-0140|(imago)|France|658[0n]
Maniola jurtina|OXB147-14|OXB_ACH-0139|(imago)|France|658[0n]
Maniola jurtina|OXB144-14|OXB_ACH-0136|(imago)|France|658[0n]
Maniola jurtina|OXB137-14|OXB_ACH-0129|(imago)|France|658[0n]
Maniola jurtina|OXB136-14|OXB_ACH-0128|(imago)|France|658[0n]
Maniola jurtina|OXB135-14|OXB_ACH-0127|(imago)|France|658[0n]
Maniola jurtina|OXB134-14|OXB_ACH-0126|(imago)|France|658[0n]
Maniola jurtina|OXB131-14|OXB_ACH-0123|(imago)|France|658[0n]
Maniola jurtina|GBLN5014-14|KM033847|Elba|Italy|655[0n]
Maniola jurtina|WMB061-11|RVcoll.LD-2600|Elba|Italy|658[0n]
Maniola jurtina|WMB3411-14|RVcoll.09-V938||Spain|658[0n]
Maniola jurtina|OXB1321-15|Rvcoll_15-M592||France|658[0n]
Maniola jurtina|EZROM373-08|07-E622||Spain|646[0n]
Maniola jurtina|EZSPM081-09|RVcoll.08-P400||Spain|658[0n]
Maniola jurtina|OXB158-14|OXB_ACH-0150|(imago)|France|633[3n]
Maniola jurtina|OXB138-14|OXB_ACH-0130|(imago)|France|658[0n]
Maniola jurtina|OXB162-14|OXB_ACH-0154|(imago)|France|658[0n]
Maniola jurtina|WMB5012-14|RVcoll. 14-I559||Italy|658[0n]
Maniola jurtina|OXB161-14|OXB_ACH-0153|(imago)|France|658[0n]
Maniola jurtina|OXB165-14|OXB_ACH-0157|(imago)|France|658[0n]
Maniola jurtina|OXB1361-15|Rvcoll_15-M715||France|658[0n]
Maniola jurtina|WMB3552-14|RVcoll.11-D888||Spain|658[0n]
Maniola jurtina|OXB163-14|OXB_ACH-0155|(imago)|France|658[0n]
Maniola jurtina|OXB169-14|OXB_ACH-0161|(imago)|France|658[0n]
Maniola jurtina|WMB3198-14|RVcoll.08-J072||Spain|658[0n]
Maniola jurtina|EZSPM871-12|Rvcoll.12-L620||Portugal|658[0n]
Maniola jurtina|EZSPM435-09|Rvcoll.09-X117||Spain|614[0n]
Maniola jurtina|OXB1043-15|LD-2986||Italy|658[0n]
Maniola jurtina|WMB3933-14|RVcoll.12-P535||France|658[0n]
Maniola jurtina|WMB2308-13|RVcoll.11-E145|Gozo. Genit. examined|Malta|658[0n]
Maniola jurtina|EZSPM905-12|Rvcoll.12-M627||Spain|658[0n]
Maniola jurtina|EZSPC530-09|RVcoll.08-P322||Spain|658[0n]
Maniola jurtina|EZSPC529-09|RVcoll.08-R264||Spain|658[0n]
Maniola jurtina|EZSPC528-09|RVcoll.08-M719||Spain|658[0n]
Maniola jurtina|OXB167-14|OXB_ACH-0159|(imago)|France|658[0n]

Maniola jurtina|EZSPC530-09|[illegible]||Spain|658[0n]
Maniola jurtina|EZSPC529-09|RVcoll.08-R264||Spain|658[0n]
Maniola jurtina|EZSPC528-09|RVcoll.08-M719||Spain|658[0n]
Maniola jurtina|OXB167-14|OXB_ACH-0159|(imago)|France|658[0n]
Maniola jurtina|OXB157-14|OXB_ACH-0149|(imago)|France|658[0n]
Maniola jurtina|OXB1335-15|Rvcoll_15-M679||France|658[0n]
Maniola jurtina|BIBSA1048-15|15-M658||France|658[0n]
Maniola jurtina|EZSPN394-09|RVcoll.08-H452|Genit. examined|Spain|658[0n]
Maniola jurtina|GBLN5065-14|KM033898|Sardinia|Italy|655[0n]
Maniola jurtina|OXB108-14|OXB_ACH-0100|(imago)|United Kingdom|658[0n]
Maniola jurtina|GBLN5099-14|KM033932|Ibiza|Spain|655[0n]
Maniola jurtina|WMB2660-13|RVcoll.12-M991|Ibiza|Spain|658[0n]
Maniola jurtina|OXB078-14|OXB_ACH-0070|(imago)|United Kingdom|658[0n]
Maniola jurtina|OXB081-14|OXB_ACH-0073|(imago)|United Kingdom|658[0n]
Maniola jurtina|OXB071-14|OXB_ACH-0063|(imago)|United Kingdom|658[0n]
Maniola jurtina|OXB069-14|OXB_ACH-0061|(imago)|United Kingdom|658[0n]
Maniola jurtina|OXB462-15|OXB-TGS-400||United Kingdom|658[0n]
Maniola jurtina|WMB2658-13|RVcoll.12-N683|Menorca|Spain|658[0n]
Maniola jurtina|WMB5353-14|RVcoll.14-J695||France|658[0n]
Maniola jurtina|GBLN5060-14|KM033893|Genit. examined|France|655[0n]
Maniola jurtina|WMB1144-13|RVcoll.11-E146|Gozo. Genit. examined|Malta|625[0n]
Maniola jurtina|OXB146-14|OXB_ACH-0138|(imago)|France|658[0n]
Maniola jurtina|BIBSA1321-15|12-O551|Sardinia|Italy|658[0n]
Maniola jurtina|OXB124-14|OXB_ACH-0116|(imago)|United Kingdom|633[0n]
Maniola jurtina|WMB370-11|RVcoll.11-E982||France|628[0n]
Maniola jurtina|WMB319-11|RVcoll.11-E563||Italy|631[0n]
Maniola jurtina|WMB2267-13|RVcoll.LD-1334|Lipari|Italy|632[0n]
Maniola jurtina|WMB1268-13|RVcoll.12-M287|Lipari|Italy|632[0n]
Maniola jurtina|WMB1534-13|RVcoll.12-O274|La Maddalena|Italy|658[0n]
Maniola jurtina|WMB1516-13|RVcoll.12-O125|Corsica|France|658[0n]
Maniola jurtina|WMB2787-13|RVcoll.11-I313|Pianosa|Italy|658[0n]
Maniola jurtina|BIBSA1279-15|12-O485|Sardinia|Italy|658[0n]
Maniola jurtina|OXB065-14|OXB_ACH-0057|(imago)|United Kingdom|614[0n]
Maniola jurtina|OXB010-14|OXB_ACH-0002||United Kingdom|614[0n]
Maniola jurtina|OXB088-14|OXB_ACH-0080|(imago)|United Kingdom|614[1n]
Maniola jurtina|OXB058-14|OXB_ACH-0050|(imago)|United Kingdom|610[0n]
Maniola jurtina|WMB3558-14|RVcoll.11-D903||Spain|658[0n]
Maniola jurtina|OXB1380-15|Rvcoll_15-M744||France|613[0n]
Maniola jurtina|OXB1320-15|Rvcoll_15-M591||France|612[0n]
Maniola jurtina|OXB174-14|OXB_ACH-0166|(imago)|United Kingdom|605[0n]
Maniola jurtina|OXB175-14|OXB_ACH-0167|(imago)|United Kingdom|603[0n]
Maniola jurtina|OXB045-14|OXB_ACH-0037|(imago)|United Kingdom|623[0n]
Maniola jurtina|BIBSA1274-15|11-E943|Corsica|France|621[0n]
Maniola jurtina|EZROM372-08|07-E621||Spain|609[0n]
Maniola jurtina|EZSPN682-09|RVcoll.08-L004||Spain|634[0n]
Maniola jurtina|OXB042-14|OXB_ACH-0034|(imago)|United Kingdom|658[0n]
Maniola jurtina|OXB041-14|OXB_ACH-0033|(imago)|United Kingdom|658[0n]
Maniola jurtina|OXB036-14|OXB_ACH-0028|(imago)|United Kingdom|658[0n]
Maniola jurtina|OXB032-14|OXB_ACH-0024|(imago)|United Kingdom|658[0n]
Maniola jurtina|OXB030-14|OXB_ACH-0022|(imago)|United Kingdom|658[0n]
Maniola jurtina|OXB173-14|OXB_ACH-0165|(imago)|United Kingdom|658[0n]
Maniola jurtina|OXB172-14|OXB_ACH-0164|(imago)|United Kingdom|658[0n]
Maniola jurtina|OXB171-14|OXB_ACH-0163|(imago)|United Kingdom|658[0n]
Maniola jurtina|OXB190-14|OXB_ACH-0182|(imago)|United Kingdom|658[0n]
Maniola jurtina|EZSPM351-09|RVcoll.08-R093||Spain|658[0n]
Maniola jurtina|EZSPM979-12|Rvcoll.12-M741||Spain|658[0n]
Maniola jurtina|EZSPN585-09|RVcoll.08-J146||Portugal|658[0n]
Maniola jurtina|EZSPN634-09|RVcoll.08-J740|Genit. examined|Spain|658[0n]
Maniola jurtina|EZSPM380-09|RVcoll.08-J874||Spain|658[0n]
Maniola jurtina|EZSPM712-12|Rvcoll. 12-L542||Spain|658[0n]
Maniola jurtina|EZSPM713-12|Rvcoll. 12-L548||Portugal|658[0n]
Maniola jurtina|EZSPM714-12|Rvcoll. 12-L569||Portugal|658[0n]
Maniola jurtina|EZSPN1022-09|RVcoll.08-M954|Genit. examined|Spain|658[0n]
Maniola jurtina|EZSPM1044-12|Rvcoll.12-M966||Spain|658[0n]
Maniola jurtina|EZSPM901-12|Rvcoll.12-M623||Spain|658[0n]
Maniola jurtina|EZSPM937-12|Rvcoll.12-M671||Spain|658[0n]
Maniola jurtina|EZSPM938-12|Rvcoll.12-M672||Spain|658[0n]
Maniola jurtina|WMB268-11|RVcoll.10-A458|Genit. examined|France|658[0n]
Maniola jurtina|WMB062-11|RVcoll.LD-2601|Elba|Italy|658[0n]
Maniola jurtina|WMB3003-14|RVcoll.12-O440|Sant Antioco|Italy|658[0n]
Maniola jurtina|WMB3070-14|RVcoll.14-E194|Elba|Italy|658[0n]
Maniola jurtina|WMB3279-14|RVcoll.08-M065|Genit. examined|Spain|658[0n]
Maniola jurtina|WMB3318-14|RVcoll.08-P688||Spain|658[0n]
Maniola jurtina|WMB2786-13|RVcoll.11-I311|Pianosa|Italy|658[0n]
Maniola jurtina|WMB1145-13|RVcoll.11-E147|Gozo|Malta|658[0n]
Maniola jurtina|WMB3776-14|RVcoll.12-N720||Spain|658[0n]
Maniola jurtina|WMB1740-13|RVcoll.12-P952|Levant|France|658[0n]
Maniola jurtina|WMB1741-13|RVcoll.12-P953|Levant|France|658[0n]
Maniola jurtina|WMB1994-13|RVcoll.11-I314|Pianosa|Italy|658[0n]
Maniola jurtina|WMB1995-13|RVcoll.11-I315|Pianosa|Italy|658[0n]
Maniola jurtina|WMB2000-13|RVcoll.12-Q368||France|658[0n]
Maniola jurtina|WMB2307-13|RVcoll.11-E144|Gozo. Genit. examined|Malta|658[0n]
Maniola jurtina|WMB2454-13|RVcoll.11-E618|Sardinia|Italy|658[0n]
Maniola jurtina|WMB2659-13|RVcoll.12-M990|Ibiza|Spain|658[0n]
Maniola jurtina|WMB3892-14|RVcoll.12-O917||France|658[0n]
Maniola jurtina|WMB4568-14|RVcoll.130711PX74||Spain|658[0n]
Maniola jurtina|WMB3924-14|RVcoll.12-P509||France|658[0n]
Maniola jurtina|WMB3963-14|RVcoll.12-P901||France|658[0n]
Maniola jurtina|WMB3987-14|RVcoll.12-Q321||France|658[0n]
Maniola jurtina|WMB4384-14|RVcoll.14-B277||Portugal|658[0n]
Maniola jurtina|WMB4404-14|RVcoll.14-B423||Portugal|658[0n]
Maniola jurtina|WMB4413-14|RVcoll.14-B455||Portugal|658[0n]
Maniola jurtina|WMB4428-14|RVcoll.14-B489||Portugal|658[0n]
Maniola jurtina|WMB4694-14|RVcoll.LD-2861|Asinara|Italy|658[0n]
Maniola jurtina|WMB299-11|RVcoll.11-E319|Genit. examined|Italy|658[0n]
Maniola jurtina|WMB343-11|RVcoll.11-E708||France|658[0n]
Maniola jurtina|WMB6035-18|RVcoll11H166||Germany|658[0n]
Maniola jurtina|WMB6036-18|RVcoll12R482||United Kingdom|658[0n]
Maniola jurtina|WMB6037-18|RVcoll12R490||United Kingdom|658[0n]
Maniola jurtina|WMB6038-18|RVcoll12Z216||Sweden|658[0n]
Maniola jurtina|WMB6040-18|RVcoll13S650||Italy|658[0n]
Maniola jurtina|WMB6045-18|RVcoll13S679||Italy|658[0n]
Maniola jurtina|WMB6050-18|RVcoll13S706||Italy|658[0n]

Maniola jurtina|WMB6040-18|RVcoll13S650||Italy|658[0n]
Maniola jurtina|WMB6045-18|RVcoll13S679||Italy|658[0n]
Maniola jurtina|WMB6050-18|RVcoll13S706||Italy|658[0n]
Maniola jurtina|WMB608-11|RVcoll.11-I008|Sicily|Italy|658[0n]
Maniola jurtina|WMB4999-14|RVcoll. 14-I546||Italy|658[0n]
Maniola jurtina|WMB5006-14|RVcoll. 14-I553||Italy|658[0n]
Maniola jurtina|EULEP4732-16|RVcoll16G463||Sweden|658[0n]
Maniola jurtina|EULEP4210-16|RVcoll15G094||France|658[0n]
Maniola jurtina|EULEP4211-16|RVcoll15G172||France|658[0n]
Maniola jurtina|EULEP2427-15|RVcoll.14-V214||Belgium|658[0n]
Maniola jurtina|EULEP2331-15|RVcoll.14-V016||Denmark|658[0n]
Maniola jurtina|BIBSA1258-15|15-F863||France|658[0n]
Maniola jurtina|BIBSA1270-15|11-E669|Sardinia|Italy|658[0n]
Maniola jurtina|BIBSA924-15|16-A015||France|658[0n]
Maniola jurtina|BIBSA935-15|16-A026||Italy|658[0n]
Maniola jurtina|BIBSA1308-15|12-M355|Sicily|Italy|658[0n]
Maniola jurtina|BIBSA1313-15|12-O195|Corsica|France|658[0n]
Maniola jurtina|BIBSA1318-15|12-O461|Sardinia|Italy|658[0n]
Maniola jurtina|BIBSA1327-15|12-Q485|Sardinia|Italy|658[0n]
Maniola jurtina|BIBSA507-15|LEP-SS-00127||Italy|658[0n]
Maniola jurtina|OXB1465-16|RVcoll12R491||United Kingdom|658[0n]
Maniola jurtina|OXB1466-16|RVcoll12R489||United Kingdom|658[0n]
Maniola jurtina|OXB1467-16|RVcoll12R488||United Kingdom|658[0n]
Maniola jurtina|OXB1468-16|RVcoll12R487||United Kingdom|658[0n]
Maniola jurtina|OXB1469-16|RVcoll12R486||United Kingdom|658[0n]
Maniola jurtina|OXB1470-16|RVcoll12R485||United Kingdom|658[0n]
Maniola jurtina|OXB1471-16|RVcoll14W450||United Kingdom|658[0n]
Maniola jurtina|OXB1474-16|RVcoll14W442||United Kingdom|658[0n]
Maniola jurtina|OXB1476-16|RVcoll14W427||United Kingdom|658[0n]
Maniola jurtina|OXB1478-16|RVcoll14W425||United Kingdom|658[0n]
Maniola jurtina|OXB1482-16|RVcoll14W405||United Kingdom|658[0n]
Maniola jurtina|OXB391-15|OXB-TGS-027||United Kingdom|658[0n]
Maniola jurtina|OXB393-15|OXB-TGS-029||United Kingdom|658[0n]
Maniola jurtina|OXB1033-15|LD-2172||Italy|658[0n]
Maniola jurtina|OXB1540-16|OXB-TGS-1258||France|658[0n]
Maniola jurtina|OXB1541-16|OXB-TGS-1259||France|658[0n]
Maniola jurtina|OXB044-14|OXB_ACH-0036|(imago)|United Kingdom|658[0n]
Maniola jurtina|OXB048-14|OXB_ACH-0040|(imago)|United Kingdom|658[0n]
Maniola jurtina|OXB049-14|OXB_ACH-0041|(imago)|United Kingdom|658[0n]
Maniola jurtina|OXB053-14|OXB_ACH-0045|(imago)|United Kingdom|658[0n]
Maniola jurtina|OXB057-14|OXB_ACH-0049|(imago)|United Kingdom|658[0n]
Maniola jurtina|OXB064-14|OXB_ACH-0056|(imago)|United Kingdom|658[0n]
Maniola jurtina|OXB077-14|OXB_ACH-0069|(imago)|United Kingdom|658[0n]
Maniola jurtina|OXB080-14|OXB_ACH-0072|(imago)|United Kingdom|658[0n]
Maniola jurtina|OXB099-14|OXB_ACH-0091|(imago)|United Kingdom|658[0n]
Maniola jurtina|OXB101-14|OXB_ACH-0093|(imago)|United Kingdom|658[0n]
Maniola jurtina|OXB103-14|OXB_ACH-0095|(imago)|United Kingdom|658[0n]
Maniola jurtina|OXB105-14|OXB_ACH-0097|(imago)|United Kingdom|658[0n]
Maniola jurtina|OXB107-14|OXB_ACH-0099|(imago)|United Kingdom|658[0n]
Maniola jurtina|OXB116-14|OXB_ACH-0108|(imago)|United Kingdom|658[0n]
Maniola jurtina|OXB117-14|OXB_ACH-0109|(imago)|United Kingdom|658[0n]
Maniola jurtina|OXB119-14|OXB_ACH-0111|(imago)|United Kingdom|658[0n]
Maniola jurtina|OXB121-14|OXB_ACH-0113|(imago)|United Kingdom|658[0n]
Maniola jurtina|OXB122-14|OXB_ACH-0114|(imago)|United Kingdom|658[0n]
Maniola jurtina|OXB404-15|OXB-TGS-071||United Kingdom|658[0n]
Maniola jurtina|OXB405-15|OXB-TGS-072||United Kingdom|658[0n]
Maniola jurtina|OXB406-15|OXB-TGS-073||United Kingdom|658[0n]
Maniola jurtina|OXB407-15|OXB-TGS-074||United Kingdom|658[0n]
Maniola jurtina|OXB413-15|OXB-TGS-082||United Kingdom|658[0n]
Maniola jurtina|OXB414-15|OXB-TGS-083||United Kingdom|658[0n]
Maniola jurtina|OXB415-15|OXB-TGS-084||United Kingdom|658[0n]
Maniola jurtina|OXB416-15|OXB-TGS-085||United Kingdom|658[0n]
Maniola jurtina|OXB417-15|OXB-TGS-086||United Kingdom|658[0n]
Maniola jurtina|OXB424-15|OXB-TGS-131||United Kingdom|658[0n]
Maniola jurtina|OXB425-15|OXB-TGS-132||United Kingdom|658[0n]
Maniola jurtina|OXB426-15|OXB-TGS-133||United Kingdom|658[0n]
Maniola jurtina|OXB427-15|OXB-TGS-134||United Kingdom|658[0n]
Maniola jurtina|OXB428-15|OXB-TGS-135||United Kingdom|658[0n]
Maniola jurtina|OXB434-15|OXB-TGS-158||United Kingdom|658[0n]
Maniola jurtina|OXB444-15|OXB-TGS-176||United Kingdom|658[0n]
Maniola jurtina|OXB446-15|OXB-TGS-178||United Kingdom|658[0n]
Maniola jurtina|OXB1316-15|Rvcoll_15-M582||France|658[0n]
Maniola jurtina|OXB537-15|OXB-TGS-912||United Kingdom|658[0n]
Maniola jurtina|OXB538-15|OXB-TGS-913||United Kingdom|658[0n]
Maniola jurtina|OXB539-15|OXB-TGS-914||United Kingdom|658[0n]
Maniola jurtina|OXB556-15|OXB-TGS-931||United Kingdom|658[0n]
Maniola jurtina|OXB566-15|OXB-TGS-941||United Kingdom|658[0n]
Maniola jurtina|OXB011-14|OXB_ACH-0003||United Kingdom|658[0n]
Maniola jurtina|OXB013-14|OXB_ACH-0005||United Kingdom|658[0n]
Maniola jurtina|OXB014-14|OXB_ACH-0006||United Kingdom|658[0n]
Maniola jurtina|OXB035-14|OXB_ACH-0027|(imago)|United Kingdom|658[0n]
Maniola jurtina|OXB037-14|OXB_ACH-0029|(imago)|United Kingdom|658[0n]
Maniola jurtina|OXB139-14|OXB_ACH-0131|(imago)|France|658[0n]
Maniola jurtina|OXB142-14|OXB_ACH-0134|(imago)|France|658[0n]
Maniola jurtina|OXB145-14|OXB_ACH-0137|(imago)|France|658[0n]
Maniola jurtina|OXB151-14|OXB_ACH-0143|(imago)|France|658[0n]
Maniola jurtina|OXB152-14|OXB_ACH-0144|(imago)|France|658[0n]
Maniola jurtina|OXB153-14|OXB_ACH-0145|(imago)|France|658[0n]
Maniola jurtina|OXB154-14|OXB_ACH-0146|(imago)|France|658[0n]
Maniola jurtina|OXB155-14|OXB_ACH-0147|(imago)|France|658[0n]
Maniola jurtina|OXB156-14|OXB_ACH-0148|(imago)|France|658[0n]
Maniola jurtina|OXB164-14|OXB_ACH-0156|(imago)|France|658[0n]
Maniola jurtina|OXB166-14|OXB_ACH-0158|(imago)|France|658[0n]
Maniola jurtina|OXB168-14|OXB_ACH-0160|(imago)|France|658[0n]
Maniola jurtina|OXB170-14|OXB_ACH-0162|(imago)|France|658[0n]
Maniola jurtina|OXB176-14|OXB_ACH-0168|(imago)|United Kingdom|658[0n]
Maniola jurtina|OXB177-14|OXB_ACH-0169|(imago)|United Kingdom|658[0n]
Maniola jurtina|OXB178-14|OXB_ACH-0170|(imago)|United Kingdom|658[0n]
Maniola jurtina|OXB179-14|OXB_ACH-0171|(imago)|United Kingdom|658[0n]
Maniola jurtina|OXB180-14|OXB_ACH-0172|(imago)|United Kingdom|658[0n]
Maniola jurtina|OXB182-14|OXB_ACH-0174|(imago)|United Kingdom|658[0n]
Maniola jurtina|OXB183-14|OXB_ACH-0175|(imago)|United Kingdom|658[0n]
Maniola jurtina|OXB184-14|OXB_ACH-0176|(imago)|United Kingdom|658[0n]

[illegible]
Maniola jurtina|OXB182-14|OXB_ACH-0174|(imago)|United Kingdom|658[0n]
Maniola jurtina|OXB183-14|OXB_ACH-0175|(imago)|United Kingdom|658[0n]
Maniola jurtina|OXB184-14|OXB_ACH-0176|(imago)|United Kingdom|658[0n]
Maniola jurtina|OXB185-14|OXB_ACH-0177|(imago)|United Kingdom|658[0n]
Maniola jurtina|OXB186-14|OXB_ACH-0178|(imago)|United Kingdom|658[0n]
Maniola jurtina|OXB187-14|OXB_ACH-0179|(imago)|United Kingdom|658[0n]
Maniola jurtina|OXB188-14|OXB_ACH-0180|(imago)|United Kingdom|658[0n]
Maniola jurtina|OXB1212-15|Rvcoll_15-M637||France|658[0n]
Maniola jurtina|OXB1254-15|Rvcoll_15-M108||France|658[0n]
Maniola jurtina|OXB1276-15|Rvcoll_15-M136||France|658[0n]
Maniola jurtina|OXB1277-15|Rvcoll_15-M137||France|658[0n]
Maniola jurtina|OXB1398-15|Rvcoll_15-M765||France|658[0n]
Maniola jurtina|GBLN5017-14|KM033850||Italy|655[0n]
Maniola jurtina|GBLN5029-14|KM033862||France|655[0n]
Maniola jurtina|GBLN5030-14|KM033863|Mallorca|Spain|655[0n]
Maniola jurtina|GBLN5063-14|KM033896|Sardinia. Genit. examined|Italy|655[0n]
Maniola jurtina|GBLN5064-14|KM033897|Sardinia|Italy|655[0n]
Maniola jurtina|GBLN5067-14|KM033900|Corsica Genit. examined|France|655[0n]
Maniola jurtina|GBLN5068-14|KM033901|Corsica Genit. examined|France|655[0n]
Maniola jurtina|GBLN5076-14|KM033909|Pianosa|Italy|655[0n]
Maniola jurtina|GBLN5077-14|KM033910|Pianosa|Italy|655[0n]
Maniola jurtina|GBLN5078-14|KM033911|Pianosa|Italy|655[0n]
Maniola jurtina|GBLN5079-14|KM033912|Genit. examined|France|655[0n]
Maniola jurtina|GBLN5080-14|KM033913|Genit. examined|France|655[0n]
Maniola jurtina|GBLN5081-14|KM033914|Genit. examined|France|655[0n]
Maniola jurtina|GBLN5087-14|KM033920|Corsica|France|655[0n]
Maniola jurtina|GBLN5088-14|KM033921|Corsica|France|655[0n]
Maniola jurtina|GBLN5090-14|KM033923|Sardinia|Italy|655[0n]
Maniola jurtina|GBLN5092-14|KM033925|Sicily|Italy|655[0n]
Maniola jurtina|GBLN5095-14|KM033928||Italy|655[0n]
Maniola jurtina|GBLN5096-14|KM033929||Italy|655[0n]
Maniola jurtina|GBLN5097-14|KM033930||Italy|655[0n]
Maniola jurtina|GBLN5098-14|KM033931|Mallorca|Spain|655[0n]
Maniola jurtina|OXB051-14|OXB_ACH-0043|(imago)|United Kingdom|658[0n]
Maniola jurtina|GBLN5103-14|KM033936|Lipari|Italy|655[0n]
Maniola jurtina|GBLN5108-14|KM033941||Italy|655[0n]
Maniola jurtina|OXB1181-15|Rvcoll_16-A068||Italy|658[0n]
Maniola jurtina|OXB1418-15|Rvcoll_15-M122||France|658[0n]
Maniola jurtina|OXB1417-15|Rvcoll_15-M111||France|658[0n]
Maniola jurtina|GBLN5019-14|KM033852|Sicily|Italy|655[0n]
Maniola jurtina|WMB5313-14|RVcoll.14-I738||France|658[0n]
Maniola jurtina|EZSPM968-12|Rvcoll.12-M722||Spain|653[0n]
Maniola jurtina|EZSPM770-12|Rvcoll. 050111LP05||Spain|658[0n]
Maniola jurtina|EZSPN319-09|RVcoll.08-H180||Spain|658[0n]
Maniola jurtina|EZSPN815-09|RVcoll.08-L620|Genit. examined|Spain|658[0n]
Maniola jurtina|EZSPN774-09|RVcoll.08-L284|Genit. examined|Spain|658[0n]
Maniola jurtina|EZSPC527-09|RVcoll.08-J414||Spain|658[0n]
Maniola jurtina|LEFID814-10|MM06901||Finland|658[0n]
Maniola jurtina|WMB6034-18|RVcoll11H165||Germany|658[0n]
Maniola jurtina|OXB683-15|12-M351|Sicily E|Italy|658[0n]
Maniola jurtina|WMB577-11|RVcoll.11-H834|Vulcano|Italy|658[0n]
Maniola jurtina|WMB2605-13|RVcoll.LD-1336|Lipari|Italy|658[0n]
Maniola jurtina|GBLN5073-14|KM033906|Vulcano|Italy|655[0n]
Maniola jurtina|BIBSA1580-16|12-M349||Italy|658[0n]
Maniola jurtina|WMB2604-13|RVcoll.LD-1335|Lipari|Italy|658[0n]
Maniola jurtina|WMB1938-13|RVcoll.11-H836|Vulcano|Italy|658[0n]
Maniola jurtina|WMB2770-13|RVcoll.11-H839|Vulcano|Italy|658[0n]
Maniola jurtina|WMB2769-13|RVcoll.11-H838|Vulcano|Italy|658[0n]
Maniola jurtina|GBLN5037-14|KM033870||Bosnia and Herzegovina|655[0n]
Maniola jurtina|EULEP2017-15|RVcoll.14-I847||Poland|658[0n]
Maniola jurtina|GBLN5062-14|KM033895||Switzerland|655[0n]
Maniola jurtina|GBLN5016-14|KM033849||Switzerland|655[0n]
Maniola jurtina|GBLN5015-14|KM033848||Switzerland|655[0n]
Maniola jurtina|LOWA528-06|2005-LOWA-528||Ukraine|658[0n]
Maniola jurtina|BIBSA1186-15|15-M238||Italy|658[0n]
Maniola jurtina|EULEP4206-16|RVcoll15G050||France|658[0n]
Maniola jurtina|GBLN5056-14|KM033889|Genit. examined|Italy|655[0n]
Maniola jurtina|GBLN5040-14|KM033873||Italy|655[0n]
Maniola chia|WMB3885-14|RVcoll.12-N775|Chios. Genit. examined|Greece|658[0n]
Maniola jurtina|GBLN5031-14|KM033864||Italy|655[0n]
Maniola jurtina|WMB6543-18|RVcoll14B779||Albania|658[0n]
Maniola jurtina|WMB2756-13|RVcoll.11-H526|Capri|Italy|658[0n]
Maniola jurtina|LEASS494-17|TLMF Lep 22142||Austria|658[0n]
Maniola jurtina|FBLMU481-09|BC ZSM Lep 27131||Germany|658[0n]
Maniola jurtina|WMB5038-14|RVcoll. 14-L169||Italy|658[0n]
Maniola jurtina|OXB765-15|14-A187||Italy|658[0n]
Maniola jurtina|WMB4278-14|RVcoll.14-A234||Italy|658[0n]
Maniola jurtina|OXB1074-15|15-A607||Italy|658[0n]
Maniola jurtina|GBLN5072-14|KM033905|Capri|Italy|655[0n]
Maniola jurtina|OXB367-15|RVcoll.14-N081||Italy|658[0n]
Maniola jurtina|OXB619-15|14-O008||Italy|658[0n]
Maniola jurtina|WMB5235-14|RVcoll.13-U082||Italy|658[0n]
Maniola jurtina|WMB5088-14|RVcoll. 14-L219||Italy|658[0n]
Maniola jurtina|WMB2470-13|RVcoll.11-H525|Capri|Italy|658[0n]
Maniola jurtina|GBLN5082-14|KM033915||Switzerland|655[0n]
Maniola jurtina|GBLN5035-14|KM033868||Greece|655[0n]
Maniola jurtina|GBLN5022-14|KM033855||Switzerland|655[0n]
Maniola jurtina|GBLN5021-14|KM033854||Switzerland|655[0n]
Maniola jurtina|OXB1171-15|Rvcoll_16-A058||Italy|658[0n]
Maniola jurtina|BIBSA411-15|RVcoll.14-I079||Italy|658[0n]
Maniola jurtina|EZROM587-08|RV-07-C362||Romania|658[0n]
Maniola jurtina|EZRMN093-08|RVcoll.08-M456||Romania|658[0n]
Maniola jurtina|OXB1117-15|15-A650||Italy|658[0n]
Maniola jurtina|BIBSA1813-17|RVcoll14I282||Italy|658[0n]
Maniola jurtina|EULEP4209-16|RVcoll15I353||Austria|658[0n]
Maniola chia|WMB5132-14|RVcoll. 14-L263|Chios|Greece|658[0n]
Maniola chia|WMB3886-14|RVcoll.12-N776|Chios|Greece|658[0n]
Maniola chia|WMB3884-14|RVcoll.12-N774|Chios|Greece|658[0n]
Maniola jurtina|WMB1886-13|RVcoll.11-J752||Italy|658[0n]
Maniola jurtina|GBLN5086-14|KM033919|Corsica|France|655[0n]
Maniola jurtina|GBLN5069-14|KM033902|Corsica Genit. examined|France|655[0n]
Maniola jurtina|GBLN5025-14|KM033858|Argentario|Italy|655[0n]
[illegible]

Maniola jurtina|GBLN5086-14|KM033919|Corsica|France|655[0n]
Maniola jurtina|GBLN5069-14|KM033902|Corsica Genit. examined|France|655[0n]
Maniola jurtina|GBLN5025-14|KM033858|Argentario|Italy|655[0n]
Maniola jurtina|WMB6049-18|RVcoll13S684||Italy|658[0n]
Maniola jurtina|WMB6048-18|RVcoll13S683||Italy|658[0n]
Maniola jurtina|WMB6044-18|RVcoll13S678||Italy|658[0n]
Maniola jurtina|WMB6041-18|RVcoll13S651||Italy|658[0n]
Maniola jurtina|WMB2455-13|RVcoll.11-E879|Corsica|France|658[0n]
Maniola jurtina|GBLN5047-14|KM033880||Italy|655[0n]
Maniola jurtina|WMB6039-18|RVcoll12Z303||Sweden|658[0n]
Maniola jurtina|WMB4036-14|RVcoll.12-Z304||Sweden|658[0n]
Maniola jurtina|GBLN5106-14|KM033939||Italy|655[0n]
Maniola jurtina|GBLN5105-14|KM033938||Italy|655[0n]
Maniola jurtina|GBLN5104-14|KM033937||Italy|655[0n]
Maniola jurtina|WMB6042-18|RVcoll13S676||Italy|658[0n]
Maniola jurtina|OXB849-15|14-U812||Italy|658[0n]
Maniola jurtina|BIBSA995-15|15-C025||Italy|658[0n]
Maniola jurtina|EULEP4207-16|RVcoll15H200||Switzerland|658[0n]
Maniola jurtina|ODOPE241-11|BC ZSM Lep 53112||Germany|658[0n]
Maniola jurtina|GBLN5102-14|KM033935|Elba|Italy|655[0n]
Maniola jurtina|GBLN5049-14|KM033882||Italy|655[0n]
Maniola jurtina|GBLN5042-14|KM033875||Italy|655[0n]
Maniola jurtina|GBLN5052-14|KM033885||Italy|655[0n]
Maniola jurtina|EZRMN090-08|RVcoll.08-M226||Romania|658[0n]
Maniola jurtina|FBLMX211-11|BC ZSM Lep 50422||Germany|658[0n]
Maniola jurtina|GBLN5094-14|KM033927||Italy|655[0n]
Maniola jurtina|EZROM331-08|RV-08-A009||Romania|658[0n]
Maniola jurtina|BIBSA1843-17|RVcoll16C721||Italy|658[0n]
Maniola jurtina|WMB2607-13|RVcoll.LD-2032|Ischia|Italy|658[0n]
Maniola jurtina|WMB2269-13|RVcoll.LD-2029|Ischia|Italy|658[0n]
Maniola jurtina|PHLAF361-11|TLMF Lep 05531||Macedonia|658[0n]
Maniola jurtina|WMB6544-18|RVcoll14D030||Bulgaria|658[0n]
Maniola jurtina|LEATG012-14|TLMF Lep 13799||Austria|658[0n]
Maniola jurtina|ABOLD007-16|TLMF Lep 21085||Austria|658[0n]
Maniola jurtina|LEATG459-14|TLMF Lep 14246||Italy|658[0n]
Maniola jurtina|LEATG458-14|TLMF Lep 14245||Italy|658[0n]
Maniola jurtina|LEATG013-14|TLMF Lep 13800||Austria|658[0n]
Maniola jurtina|ABOLD424-16|TLMF Lep 21576||Austria|658[0n]
Maniola jurtina|GWORR430-10|BC ZSM Lep 29646||Italy|658[0n]
Maniola jurtina|LEFID815-10|MM06902||Finland|658[0n]
Maniola jurtina|EZROM390-08|RV-06-M859||Romania|658[0n]
Maniola jurtina|EZROM391-08|RV-06-M951||Romania|658[0n]
Maniola jurtina|EZROM392-08|RV-07-D474||Romania|658[0n]
Maniola jurtina|EZROM393-08|RV-07-C987|Genit. examined|Romania|658[0n]
Maniola jurtina|EZRMN091-08|RVcoll.08-M276||Romania|658[0n]
Maniola jurtina|WMB014-11|RVcoll.LD-169|Elba|Italy|658[0n]
Maniola jurtina|WMB022-11|RVcoll.LD-856||Italy|658[0n]
Maniola jurtina|WMB023-11|RVcoll.LD-857||Italy|658[0n]
Maniola jurtina|WMB024-11|RVcoll.LD-858||Italy|658[0n]
Maniola jurtina|WMB2818-13|RVcoll.12-O034|Giglio|Italy|658[0n]
Maniola jurtina|WMB2819-13|RVcoll.12-O035|Giglio|Italy|658[0n]
Maniola jurtina|WMB2841-13|RVcoll.12-Q669||Italy|658[0n]
Maniola jurtina|WMB1494-13|RVcoll.12-O036|Giglio|Italy|658[0n]
Maniola jurtina|WMB1495-13|RVcoll.12-O037|Giglio|Italy|658[0n]
Maniola jurtina|WMB2362-13|RVcoll.12-Q919||Italy|658[0n]
Maniola jurtina|WMB2268-13|RVcoll.LD-2028|Ischia|Italy|658[0n]
Maniola jurtina|WMB2356-13|RVcoll.12-Q901||Italy|658[0n]
Maniola jurtina|WMB2388-13|RVcoll.12-Q597|Sicily|Italy|658[0n]
Maniola jurtina|WMB3826-14|RVcoll.09-X833||Italy|658[0n]
Maniola jurtina|WMB2606-13|RVcoll.LD-2030|Ischia|Italy|658[0n]
Maniola jurtina|WMB2608-13|RVcoll.LD-2602|Elba|Italy|658[0n]
Maniola jurtina|WMB4438-14|RVcoll.14-B609||Ukraine|658[0n]
Maniola jurtina|WMB4445-14|RVcoll.14-C033||Sweden|658[0n]
Maniola jurtina|WMB371-11|RVcoll.11-E986||France|658[0n]
Maniola jurtina|WMB6043-18|RVcoll13S677||Italy|658[0n]
Maniola jurtina|WMB6046-18|RVcoll13S680||Italy|658[0n]
Maniola jurtina|WMB6047-18|RVcoll13S681||Italy|658[0n]
Maniola jurtina|WMB485-11|RVcoll.11-H519|Capri|Italy|658[0n]
Maniola jurtina|WMB563-11|RVcoll.11-H761|Sicily|Italy|658[0n]
Maniola jurtina|WMB580-11|RVcoll.11-H868|Salina|Italy|658[0n]
Maniola jurtina|WMB4771-14|RVcoll. 14-I318||Italy|658[0n]
Maniola jurtina|WMB4835-14|RVcoll. 14-I382||Italy|658[0n]
Maniola jurtina|WMB4880-14|RVcoll.14-I427||Italy|658[0n]
Maniola jurtina|WMB4959-14|RVcoll. 14-I506||Italy|658[0n]
Maniola jurtina|LOWA864-06|2005-LOWA-864||Russia|658[0n]
Maniola jurtina|LOWA866-06|2005-LOWA-866||Russia|658[0n]
Maniola jurtina|EULEP4208-16|RVcoll15H718||Switzerland|658[0n]
Maniola jurtina|EULEP5051-16|RVcoll16J071||Czech Republic|658[0n]
Maniola jurtina|EULEP4955-16|RVcoll16I224||Poland|658[0n]
Maniola jurtina|EULEP5718-17|RVcoll16L189||Italy|658[0n]
Maniola chia|EULEP717-15|RVcoll.12-N777|Chios|Greece|658[0n]
Maniola jurtina|EULEP1376-15|RVcoll.14-G286||Greece|658[0n]
Maniola jurtina|BIBSA1113-15|15-L842||Italy|658[0n]
Maniola jurtina|BIBSA1272-15|11-E907|Corsica|France|658[0n]
Maniola jurtina|BIBSA1386-15|15-M982||Italy|658[0n]
Maniola jurtina|BIBSA668-15|RVcoll. 11-I269||Italy|658[0n]
Maniola jurtina|BIBSA506-15|LEP-SS-00126||Italy|658[0n]
Maniola jurtina|BIBSA1742-16|07-E007|Genit. examined|Italy|658[0n]
Maniola jurtina|GBLAA1292-15|BC ZSM Lep 87178||Germany|658[0n]
Maniola jurtina|OXB783-15|14-A758||Italy|658[0n]
Maniola jurtina|OXB1110-15|15-A643||Italy|658[0n]
Maniola jurtina|OXB342-15|RVcoll.14-N056||Italy|658[0n]
Maniola jurtina|OXB1152-15|Rvcoll_15-N111|Milos|Greece|658[0n]
Maniola jurtina|ABOLD599-17|TLMF Lep 21675||Austria|658[0n]
Maniola jurtina|GBLN5020-14|KM033853||Italy|655[0n]
Maniola jurtina|GBLN5027-14|KM033860|Argentario|Italy|655[0n]
Maniola jurtina|GBLN5028-14|KM033861|Argentario|Italy|655[0n]
Maniola jurtina|GBLN5032-14|KM033865||Italy|655[0n]
Maniola jurtina|GBLN5033-14|KM033866||Italy|655[0n]
Maniola jurtina|GBLN5034-14|KM033867||Bulgaria|655[0n]
Maniola jurtina|GBLN5036-14|KM033869||Bosnia and Herzegovina|655[0n]
Maniola jurtina|GBLN5039-14|KM033872||Italy|655[0n]
Maniola jurtina|GBLN5041-14|KM033874||Italy|655[0n]

Maniola jurtina|GBLN5036-14|KM033869||Bosnia and Herzegovina|655[0n]
Maniola jurtina|GBLN5039-14|KM033872||Italy|655[0n]
Maniola jurtina|GBLN5041-14|KM033874||Italy|655[0n]
Maniola jurtina|GBLN5043-14|KM033876||Italy|655[0n]
Maniola jurtina|GBLN5044-14|KM033877||Italy|655[0n]
Maniola jurtina|GBLN5045-14|KM033878||Italy|655[0n]
Maniola jurtina|GBLN5046-14|KM033879||Italy|655[0n]
Maniola jurtina|GBLN5048-14|KM033881||Italy|655[0n]
Maniola jurtina|GBLN5051-14|KM033884||Italy|655[0n]
Maniola jurtina|GBLN5054-14|KM033887|Ischia|Italy|655[0n]
Maniola jurtina|GBLN5074-14|KM033907||Italy|655[0n]
Maniola jurtina|OXB988-15|15-A938||Italy|658[0n]
Maniola jurtina|GBLN5050-14|KM033883|Capri|Italy|655[0n]
Maniola jurtina|GBLN5071-14|KM033904|Capri|Italy|655[0n]
Maniola jurtina|WMB1904-13|RVcoll.11-H522|Capri|Italy|648[0n]
Maniola jurtina|GBLN5061-14|KM033894||Bulgaria|655[0n]
Maniola jurtina|GBLN5075-14|KM033908||Italy|655[0n]
Maniola jurtina|GBLN5083-14|KM033916|Giglio|Italy|655[0n]
Maniola jurtina|GBLN5053-14|KM033886||Italy|655[0n]
Maniola jurtina|GBLN5084-14|KM033917|Giglio|Italy|655[0n]
Maniola jurtina|GBLN5085-14|KM033918|Giglio|Italy|655[0n]
Maniola jurtina|LOWA867-06|2005-LOWA-867||Russia|658[0n]
Maniola jurtina|GBLN5091-14|KM033924|Sicily|Italy|655[0n]
Maniola jurtina|GBLN5055-14|KM033888|Genit. examined|Italy|655[0n]
Maniola jurtina|GBLN5093-14|KM033926|Sicily|Italy|655[0n]
Maniola jurtina|GBLN5107-14|KM033940||Italy|655[0n]
Maniola jurtina|EULEP4212-16|RVcoll15P003||Belarus|636[0n]
Maniola jurtina|WMB2248-13|RVcoll.12-R386||Italy|632[0n]
Maniola jurtina|WMB4770-14|RVcoll. 14-I317||Italy|641[0n]
Maniola jurtina|GBLN5026-14|KM033859|Argentario|Italy|635[0n]
Maniola jurtina|EULEP2377-15|RVcoll.14-V072||Ukraine|613[0n]
Maniola jurtina|LOWA865-06|2005-LOWA-865||Russia|620[0n]
Maniola jurtina|PHLAH472-12|TLMF Lep 08291||Austria|615[0n]
Maniola jurtina|WMB4769-14|RVcoll. 14-I316||Italy|634[0n]
Maniola jurtina|WMB4329-14|RVcoll.14-A697||Italy|621[0n]
Maniola jurtina|EZRMN092-08|RVcoll.08-M368||Romania|609[0n]
Maniola jurtina|LEATH715-14|TLMF Lep 15927||Italy|623[0n]
Maniola jurtina|LEATH716-14|TLMF Lep 15928||Italy|634[0n]
Melanargia ines|EZSPM975-12|Rvcoll.12-M733||Spain|658[0n]
Melanargia ines|EZSPC1138-10|RVcoll.08-P727||Spain|658[0n]
Melanargia ines|EZSPC972-10|RVcoll.06-A002||Spain|658[0n]
Melanargia ines|EZSPN523-09|RVcoll.08-H993||Spain|658[0n]
Melanargia ines|EZSPM715-12|Rvcoll. 12-L559||Portugal|658[0n]
Melanargia ines|EZSPN453-09|RVcoll.08-H657||Spain|658[0n]
Melanargia ines|EZSPN438-09|RVcoll.08-H620||Spain|658[0n]
Melanargia ines|WMB3551-14|RVcoll.11-D885||Spain|658[0n]
Melanargia ines|EZSPM780-12|Rvcoll. 070111GN66||Spain|658[0n]
Melanargia ines|EZSPC1133-10|RVcoll.08-P711||Spain|658[0n]
Melanargia ines|EZSPC1131-10|RVcoll.08-P705||Spain|658[0n]
Melanargia ines|WMB3568-14|RVcoll.11-D977||Spain|658[0n]
Melanargia ines|EZSPM609-12|RVcoll.11-D680||Spain|658[0n]
Melanargia ines|WMB3576-14|RVcoll.11-E000||Spain|658[0n]
Melanargia ines|EZSPC1130-10|RVcoll.08-P704||Spain|625[0n]
Melanargia ines|EZSPC1129-10|RVcoll.08-P700||Spain|658[0n]
Melanargia ines|EZSPM717-12|Rvcoll. 12-L561||Portugal|658[0n]
Melanargia ines|EZSPM716-12|Rvcoll. 12-L560||Portugal|658[0n]
Melanargia ines|EZSPN620-09|RVcoll.08-J702||Spain|658[0n]
Melanargia ines|EZSPN710-09|RVcoll.08-L062||Spain|658[0n]
Melanargia pherusa|WMB490-11|RVcoll.11-H536|Sicily|Italy|658[0n]
Melanargia pherusa|EULEP4224-16|RVcoll15O044|Sicily|Italy|658[0n]
Melanargia pherusa|WMB1905-13|RVcoll.11-H558|Sicily|Italy|633[0n]
Melanargia arge|BIBSA602-15|LEP-SS-00222||Italy|658[0n]
Melanargia arge|EULEP4214-16|RVcoll15C747||Italy|635[0n]
Melanargia arge|WMB5181-14|RVcoll.13-S644||Italy|658[0n]
Melanargia arge|EULEP1848-15|RVcoll.13-S643||Italy|658[0n]
Melanargia arge|EULEP4213-16|RVcoll15C251||Italy|658[0n]
Melanargia arge|WMB5218-14|RVcoll.13-T968||Italy|658[0n]
Melanargia arge|WMB4838-14|RVcoll. 14-I385||Italy|658[0n]
Melanargia arge|WMB4775-14|RVcoll. 14-I322||Italy|658[0n]
Melanargia arge|WMB856-13|RVcoll.08-R608||Italy|658[0n]
Melanargia arge|WMB2718-13|RVcoll.10-C528||Italy|658[0n]
Melanargia arge|WMB2717-13|RVcoll.10-C527||Italy|658[0n]
Melanargia occitanica|WMB877-13|RVcoll.09-X262||France|658[0n]
Melanargia occitanica|OXB833-15|14-U796||Italy|658[0n]
Melanargia occitanica|WMB273-11|RVcoll.10-A501||France|658[0n]
Melanargia occitanica|WMB266-11|RVcoll.10-A424||France|658[0n]
Melanargia occitanica|EZSPC1342-10|RVcoll.130209KL47||Spain|658[0n]
Melanargia occitanica|EZSPN624-09|RVcoll.08-J708||Spain|658[0n]
Melanargia occitanica|EZSPC1187-10|RVcoll.09-T206||Spain|658[0n]
Melanargia occitanica|WMB4549-14|RVcoll.060506BE1||Spain|658[0n]
Melanargia occitanica|EZSPC1009-10|RVcoll.08-H486||Spain|658[0n]
Melanargia occitanica|EZSPN867-09|RVcoll.08-L742||Spain|631[0n]
Melanargia occitanica|EZSPN393-09|RVcoll.08-H451||Spain|658[0n]
Melanargia occitanica|WMB4551-14|RVcoll.070111GN25||Spain|658[0n]
Melanargia occitanica|EZSPM257-09|RVcoll.08-R470||Spain|658[0n]
Melanargia occitanica|EZSPM974-12|Rvcoll.12-M732||Spain|657[0n]
Melanargia occitanica|EZSPC1136-10|RVcoll.08-P723||Spain|658[0n]
Melanargia occitanica|EZSPN361-09|RVcoll.08-H343||Spain|658[0n]
Melanargia occitanica|EZSPN202-09|RVcoll.07-C695||Spain|658[0n]
Melanargia occitanica|EZSPN339-09|RVcoll.08-H288||Spain|658[0n]
Melanargia occitanica|EZSPM318-09|RVcoll.08-J883||Spain|658[0n]
Melanargia occitanica|EZSPN132-09|RVcoll.06-G480||Spain|658[0n]
Melanargia russiae|BIBSA1767-16|LD-3104|Sicily|Italy|658[0n]
Melanargia russiae|WMB1875-13|RVcoll.11-J642|Sicily|Italy|658[0n]
Melanargia russiae|BIBSA1569-16|11-I069|Sicily|Italy|658[0n]
Melanargia russiae|EULEP5097-16|LD3004|Sicily|Italy|658[0n]
Melanargia russiae|EULEP5096-16|LD3001|Sicily|Italy|658[0n]
Melanargia russiae|WMB626-11|RVcoll.11-I068|Sicily|Italy|658[0n]
Melanargia russiae|WMB4717-14|RVcoll.LD-3005|Sicily|Italy|658[0n]
Melanargia russiae|WMB2751-13|RVcoll.10-C655|Sicily|Italy|658[0n]
Melanargia russiae|EULEP4225-16|RVcoll15C246||Italy|658[0n]
Melanargia russiae|BIBSA1016-15|15-C143||Italy|658[0n]
[illegible]

Melanargia russiae|WMB2751-13|RVcoll.10-C655|Sicily|Italy|658[0n]
Melanargia russiae|EULEP4225-16|RVcoll15C246||Italy|658[0n]
Melanargia russiae|BIBSA1016-15|15-C143||Italy|658[0n]
Melanargia russiae|WMB4136-14|RVcoll.13-S640||Italy|658[0n]
Melanargia russiae|EULEP1853-15|RVcoll.13-T960||Italy|658[0n]
Melanargia russiae|WMB2039-13|RVcoll.12-Q759||Italy|658[0n]
Melanargia russiae|WMB2038-13|RVcoll.12-Q758||Italy|658[0n]
Melanargia russiae|EULEP2268-15|RVcoll.14-N453||Ukraine|658[0n]
Melanargia russiae|EULEP3378-16|RVcoll15C258||Italy|658[0n]
Melanargia russiae|EULEP1547-15|RVcoll.14-G526||Greece|658[0n]
Melanargia russiae|EULEP1548-15|RVcoll.14-G529||Greece|658[0n]
Melanargia russiae|EULEP1549-15|RVcoll.14-G530||Greece|649[0n]
Melanargia russiae|EULEP1546-15|RVcoll.14-G523||Greece|650[0n]
Melanargia russiae|OLEP016-15|RVcoll.14-O400||Hungary|658[0n]
Melanargia russiae|EULEP2958-15|RVcoll.14-O091||Russia|658[0n]
Melanargia russiae|EULEP2955-15|RVcoll.14-O088||Albania|650[0n]
Melanargia russiae|EULEP2954-15|RVcoll.14-O087||Albania|658[0n]
Melanargia russiae|EULEP1889-15|RVcoll.14-B700||Albania|658[0n]
Melanargia russiae|EULEP3381-16|RVcoll15Q067||Russia|658[0n]
Melanargia russiae|EULEP3377-16|RVcoll15Q194||Albania|658[0n]
Melanargia russiae|EULEP5093-16|RVcoll14G528||Greece|658[0n]
Melanargia russiae|EULEP5098-16|RVcoll16F172||Italy|658[0n]
Melanargia russiae|EULEP5095-16|RVcoll16F169||Italy|658[0n]
Melanargia russiae|WMB4274-14|RVcoll.14-A219||Italy|658[0n]
Melanargia russiae|EULEP5094-16|RVcoll15M389||Italy|658[0n]
Melanargia russiae|BIBSA696-15|RVcoll. 07-G004||Italy|658[0n]
Melanargia russiae|BIBSA1533-16|07-G473||Italy|658[0n]
Melanargia russiae|BIBSA1373-15|15-M930||Italy|658[0n]
Melanargia russiae|EULEP5092-16|RVcoll14A364||Italy|658[0n]
Melanargia russiae|WMB4908-14|RVcoll.14-I455||Italy|658[0n]
Melanargia russiae|WMB843-13|RVcoll.07-G474||Italy|658[0n]
Melanargia russiae|EULEP2957-15|RVcoll.14-O090||France|658[0n]
Melanargia russiae|EULEP2956-15|RVcoll.14-O089||France|650[0n]
Melanargia russiae|EZSPM302-09|RVcoll.08-J849||Spain|658[0n]
Melanargia russiae|EZSPM157-09|RVcoll.08-P683||Spain|658[0n]
Melanargia russiae|WMB5187-14|RVcoll.13-T165||Spain|658[0n]
Melanargia russiae|EZSPN747-09|RVcoll.08-L185||Spain|658[0n]
Melanargia russiae|EZSPC1183-10|RVcoll.09-T162||Spain|658[0n]
Melanargia russiae|EZSPN575-09|RVcoll.08-J122||Portugal|658[0n]
Melanargia russiae|EZSPC1033-10|RVcoll.08-J176||Portugal|658[0n]
Melanargia russiae|EZSPC1085-10|RVcoll.08-L751||Spain|658[0n]
Melanargia russiae|EZSPC1146-10|RVcoll.08-P764||Spain|658[0n]
Melanargia russiae|EZSPN896-09|RVcoll.08-L808||Spain|658[0n]
Melanargia russiae|EZSPN875-09|RVcoll.08-L768||Spain|658[0n]
Melanargia russiae|WMB4563-14|RVcoll.090611SZ14||Spain|658[0n]
Melanargia russiae|EZSPC1115-10|RVcoll.08-P022||Spain|658[0n]
Melanargia russiae|WMB4554-14|RVcoll.070611MH33||Spain|658[0n]
Melanargia russiae|EULEP242-14|RVcoll.12-N702||Spain|658[0n]
Melanargia russiae|WMB4457-14|RVcoll.14-D325||Spain|636[0n]
Melanargia russiae|EZSPC1158-10|RVcoll.08-R310||Spain|647[0n]
Melanargia russiae|EZSPM019-09|RVcoll.08-P042||Spain|658[0n]
Melanargia russiae|EZSPM203-09|RVcoll.08-R311||Spain|658[0n]
Melanargia russiae|EZSPN866-09|RVcoll.08-L740||Spain|658[0n]
Melanargia russiae|EZSPN179-09|RVcoll.06-V743||Spain|658[0n]
Melanargia larissa|EULEP1310-15|RVcoll.14-F767||Greece|658[0n]
Melanargia larissa|EULEP5579-17|RVcoll14H202||Greece|658[0n]
Melanargia larissa|EULEP1357-15|RVcoll.14-F922||Greece|658[0n]
Melanargia larissa|EULEP1351-15|RVcoll.14-F910||Greece|658[0n]
Melanargia larissa|EULEP1573-15|RVcoll.14-G585||Macedonia|658[0n]
Melanargia larissa|EULEP1816-15|RVcoll.14-H818||Greece|658[0n]
Melanargia larissa|EULEP1896-15|RVcoll.14-B727||Albania|658[0n]
Melanargia larissa|EULEP1545-15|RVcoll.14-G521||Greece|648[0n]
Melanargia larissa|EULEP1503-15|RVcoll.14-G389||Greece|658[0n]
Melanargia larissa|EULEP1606-15|RVcoll.14-G659||Greece|658[0n]
Melanargia larissa|EULEP1290-15|RVcoll.14-F715||Greece|658[0n]
Melanargia larissa|EULEP5564-17|RVcoll14F429||Bulgaria|658[0n]
Melanargia larissa|EULEP5541-17|RVcoll14B798||Albania|658[0n]
Melanargia larissa|EULEP728-15|RVcoll.12-N815|Lesvos|Greece|658[0n]
Melanargia larissa|EULEP772-15|RVcoll.12-R934|Lesvos|Greece|658[0n]
Melanargia larissa|EULEP727-15|RVcoll.12-N814|Lesvos|Greece|658[0n]
Melanargia larissa|EULEP5597-17|RVcoll14O319||Greece|658[0n]
Melanargia larissa|EULEP1654-15|RVcoll.14-G841||Greece|658[0n]
Melanargia larissa|EULEP1401-15|RVcoll.14-G048||Greece|658[0n]
Melanargia larissa|EULEP1164-15|RVcoll.14-F369||Bulgaria|658[0n]
Melanargia larissa|EULEP5545-17|RVcoll14C538||Greece|658[0n]
Melanargia larissa|EULEP5139-17|RVcoll16J895||Serbia|658[0n]
Melanargia larissa|EULEP5101-17|RVcoll16J855||Romania|658[0n]
Melanargia larissa|EULEP5100-17|RVcoll16J854||Romania|658[0n]
Melanargia larissa|EULEP5099-17|RVcoll16J853||Romania|658[0n]
Melanargia larissa|EULEP5669-17|RVcoll15Q064||Romania|658[0n]
Melanargia larissa|EULEP5668-17|RVcoll15Q063||Romania|658[0n]
Melanargia larissa|EULEP5667-17|RVcoll15Q062||Romania|658[0n]
Melanargia larissa|EULEP5566-17|RVcoll14F641||Greece|658[0n]
Melanargia larissa|EULEP5544-17|RVcoll14C294||Greece|658[0n]
Melanargia larissa|EULEP4223-16|RVcoll10B374||Bulgaria|658[0n]
Melanargia galathea|GBLAD224-14|BC ZSM Lep 78795||Germany|658[0n]
Melanargia galathea|EULEP5004-16|RVcoll16I805||Germany|658[0n]
Melanargia galathea|EULEP4218-16|RVcoll15H746||Switzerland|658[0n]
Melanargia galathea|FBLMU478-09|BC ZSM Lep 27128||Germany|658[0n]
Melanargia lachesis|WMB3405-14|RVcoll.09-V820||Spain|658[0n]
Melanargia lachesis|EZSPN1017-09|RVcoll.08-M942||Spain|658[0n]
Melanargia lachesis|EZSPM1020-12|Rvcoll.12-M769||Spain|658[0n]
Melanargia lachesis|EZSPN849-09|RVcoll.08-L693||Spain|629[0n]
Melanargia lachesis|EZSPM255-09|RVcoll.08-R468||Spain|658[0n]
Melanargia lachesis|EZSPC1385-10|RVcoll.09-X535||Spain|658[0n]
Melanargia lachesis|EZSPN491-09|RVcoll.08-H928||Spain|658[0n]
Melanargia lachesis|EZSPM1031-12|Rvcoll.12-M781||Spain|658[0n]
Melanargia lachesis|EZSPN725-09|RVcoll.08-L131||Spain|658[0n]
Melanargia lachesis|EZSPN1039-11|Rvcoll.08-H335.1||Spain|658[0n]
Melanargia lachesis|EZSPN1101-11|Rvcoll.10-A840||Spain|658[0n]
Melanargia lachesis|EZSPN362-09|RVcoll.08-H344||Spain|658[0n]
Melanargia lachesis|EZSPN573-09|RVcoll.08-J120||Portugal|658[0n]
Melanargia lachesis|EZSPN591-09|RVcoll.08-J168||Portugal|658[0n]

Melanargia lachesis|EZSPN362-09|RVcoll.08-H344||Spain|658[0n]
Melanargia lachesis|EZSPN573-09|RVcoll.08-J120||Portugal|658[0n]
Melanargia lachesis|EZSPN591-09|RVcoll.08-J168||Portugal|658[0n]
Melanargia lachesis|EZSPM317-09|RVcoll.08-J878||Spain|658[0n]
Melanargia lachesis|EZSPM444-09|Rvcoll.09-V935||Spain|658[0n]
Melanargia lachesis|EZSPM903-12|Rvcoll.12-M625||Spain|658[0n]
Melanargia lachesis|EZSPN977-09|RVcoll.08-M009||Spain|658[0n]
Melanargia lachesis|EZSPM156-09|RVcoll.08-P682||Spain|658[0n]
Melanargia lachesis|EZSPM256-09|RVcoll.08-R469||Spain|658[0n]
Melanargia lachesis|EZSPM443-09|Rvcoll.09-V470||Spain|658[0n]
Melanargia lachesis|EZSPM902-12|Rvcoll.12-M624||Spain|658[0n]
Melanargia lachesis|EZSPM1026-12|Rvcoll.12-M776||Spain|658[0n]
Melanargia lachesis|WMB3394-14|RVcoll.09-V574||Spain|658[0n]
Melanargia lachesis|WMB3143-14|RVcoll.07-C074||Spain|658[0n]
Melanargia lachesis|WMB3257-14|RVcoll.08-L475||Spain|658[0n]
Melanargia lachesis|WMB3263-14|RVcoll.08-L745||Spain|658[0n]
Melanargia lachesis|WMB3289-14|RVcoll.08-M692||Spain|658[0n]
Melanargia lachesis|WMB3778-14|RVcoll.12-N733||Spain|658[0n]
Melanargia lachesis|WMB1678-13|RVcoll.12-P386||France|658[0n]
Melanargia lachesis|WMB1683-13|RVcoll.12-P501||France|658[0n]
Melanargia lachesis|WMB4381-14|RVcoll.14-B235||Portugal|658[0n]
Melanargia lachesis|WMB5354-14|RVcoll.14-J696||France|658[0n]
Melanargia lachesis|WMB5360-14|RVcoll.14-J708||France|658[0n]
Melanargia lachesis|WMB1677-13|RVcoll.12-P385||France|641[0n]
Melanargia galathea|OXB240-15|OXB-TGS-634||United Kingdom|658[0n]
Melanargia galathea|OXB209-15|OXB-TGS-244||United Kingdom|658[0n]
Melanargia galathea|EZSPN1019-09|RVcoll.08-M951.1||Spain|658[0n]
Melanargia galathea|WMB5456-14|RVcoll.14-N232||Spain|658[0n]
Melanargia galathea|EZSPM314-09|RVcoll.08-J869||Spain|658[0n]
Melanargia galathea|EZSPM816-12|Rvcoll. 130711PX78||Spain|658[0n]
Melanargia galathea|EZSPN899-09|RVcoll.08-L811||Spain|658[0n]
Melanargia galathea|EZSPM846-12|Rvcoll. 150511SD04||Spain|658[0n]
Melanargia galathea|EZSPC1244-10|RVcoll.09-V771||Spain|658[0n]
Melanargia galathea|EZSPC1248-10|RVcoll.09-V793||Spain|658[0n]
Melanargia galathea|WMB5457-14|RVcoll.14-N234||Spain|658[0n]
Melanargia galathea|OXB210-15|OXB-TGS-251||United Kingdom|658[0n]
Melanargia galathea|OXB271-15|OXB-TGS-954||United Kingdom|658[0n]
Melanargia galathea|OXB272-15|OXB-TGS-955||United Kingdom|658[0n]
Melanargia galathea|EZSPC531-09|RVcoll.08-L485||Spain|614[0n]
Melanargia galathea|EZSPN898-09|RVcoll.08-L810||Spain|623[0n]
Melanargia galathea|EZSPN897-09|RVcoll.08-L809||Spain|646[0n]
Melanargia lachesis|EZSPN150-09|RVcoll.06-G570||Spain|658[0n]
Melanargia lachesis|EZSPN840-09|RVcoll.08-L668||Spain|658[0n]
Melanargia galathea|OXB1397-15|Rvcoll_15-M764||France|658[0n]
Melanargia galathea|BIBSA733-15|LD-3334||Italy|658[0n]
Melanargia galathea|WMB4839-14|RVcoll. 14-I386||Italy|658[0n]
Melanargia galathea|WMB557-11|RVcoll.11-H750|Sicily|Italy|658[0n]
Melanargia galathea|BIBSA516-15|LEP-SS-00136||Italy|658[0n]
Melanargia galathea|WMB1715-13|RVcoll.12-P681||France|658[0n]
Melanargia galathea|WMB4318-14|RVcoll.14-A530||Italy|614[0n]
Melanargia galathea|OXB1281-15|Rvcoll_15-M141||France|612[0n]
Melanargia galathea|WMB2109-13|RVcoll.12-R165|Sicily|Italy|627[0n]
Melanargia galathea|BIBSA1091-15|15-L339||Italy|612[0n]
Melanargia galathea|BIBSA1201-15|15-M297||Italy|658[0n]
Melanargia galathea|LEATG464-14|TLMF Lep 14251||Italy|626[0n]
Melanargia galathea|WMB5383-14|RVcoll.14-J842||France|658[0n]
Melanargia galathea|WMB3983-14|RVcoll.12-Q254||France|658[0n]
Melanargia galathea|WMB4135-14|RVcoll.13-S639||Italy|658[0n]
Melanargia galathea|WMB4496-14|RVcoll.14-E172||Italy|658[0n]
Melanargia galathea|WMB3953-14|RVcoll.12-P725||France|658[0n]
Melanargia galathea|WMB4612-14|RVcoll.LD-1805||Italy|658[0n]
Melanargia galathea|WMB4613-14|RVcoll.LD-1807||Italy|658[0n]
Melanargia galathea|WMB4679-14|RVcoll.LD-2655||Italy|658[0n]
Melanargia galathea|WMB4840-14|RVcoll. 14-I387||Italy|658[0n]
Melanargia galathea|WMB4852-14|RVcoll.14-I399||Italy|658[0n]
Melanargia galathea|WMB4875-14|RVcoll.14-I422||Italy|658[0n]
Melanargia galathea|WMB4919-14|RVcoll.14-I466||Italy|658[0n]
Melanargia galathea|WMB5305-14|RVcoll.14-I628||France|658[0n]
Melanargia galathea|WMB5492-14|RVcoll.LD-3331||Italy|658[0n]
Melanargia galathea|BIBSA1169-15|15-M192||Italy|658[0n]
Melanargia galathea|BIBSA1179-15|15-M211||Italy|658[0n]
Melanargia galathea|BIBSA928-15|16-A019||Italy|658[0n]
Melanargia galathea|BIBSA929-15|16-A020||Italy|658[0n]
Melanargia galathea|BIBSA930-15|16-A021||Italy|658[0n]
Melanargia galathea|BIBSA1066-15|14-W655||Italy|658[0n]
Melanargia galathea|BIBSA1399-15|15-N003||Italy|658[0n]
Melanargia galathea|BIBSA695-15|RVcoll. 10-C653||Italy|658[0n]
Melanargia galathea|BIBSA1551-16|LEP-SS-00381||Italy|658[0n]
Melanargia galathea|BIBSA412-15|RVcoll.14-I080||Italy|658[0n]
Melanargia galathea|BIBSA413-15|RVcoll.14-I081||Italy|658[0n]
Melanargia galathea|BIBSA515-15|LEP-SS-00135||Italy|658[0n]
Melanargia galathea|BIBSA1844-17|RVcoll16C722||Italy|658[0n]
Melanargia galathea|BIBSA1050-15|15-M758||France|658[0n]
Melanargia galathea|BIBSA1875-17|RVcoll16C776||Italy|658[0n]
Melanargia galathea|OXB778-15|14-A739||Italy|658[0n]
Melanargia galathea|OXB837-15|14-U800||Italy|658[0n]
Melanargia galathea|OXB853-15|LD-3031|Sicily E|Italy|658[0n]
Melanargia galathea|OXB854-15|LD-3032|Sicily E|Italy|658[0n]
Melanargia galathea|OXB621-15|14-O010||Italy|658[0n]
Melanargia galathea|OXB903-15|15-A547||Italy|658[0n]
Melanargia galathea|OXB989-15|15-A939||Italy|658[0n]
Melanargia galathea|OXB1072-15|15-A605||Italy|658[0n]
Melanargia galathea|OXB1349-15|Rvcoll_15-M701||France|658[0n]
Melanargia galathea|OXB1357-15|Rvcoll_15-M709||France|658[0n]
Melanargia galathea|OXB1224-15|Rvcoll_15-M656||France|658[0n]
Melanargia galathea|OXB1255-15|Rvcoll_15-M109||France|658[0n]
Melanargia galathea|OXB1097-15|15-A630||Italy|658[0n]
Melanargia galathea|OXB1262-15|Rvcoll_15-M118||France|658[0n]
Melanargia galathea|OXB1271-15|Rvcoll_15-M131||France|658[0n]
Melanargia galathea|WMB5244-14|RVcoll.13-U096||Italy|658[0n]
Melanargia galathea|OXB1272-15|Rvcoll_15-M132||France|658[0n]
Melanargia galathea|OXB1278-15|Rvcoll_15-M138||France|658[0n]
Melanargia galathea|BIBSA1598-16|12-R061||Italy|625[0n]

Melanargia galathea|OXB1272-15|Rvcoll_15-M132||France|658[0n]
Melanargia galathea|OXB1278-15|Rvcoll_15-M138||France|658[0n]
Melanargia galathea|BIBSA1598-16|12-R061||Italy|625[0n]
Melanargia galathea|EULEP4221-16|RVcoll15G174||France|636[0n]
Melanargia galathea|WMB3817-14|RVcoll.07-E075||Italy|658[0n]
Melanargia galathea|WMB3811-14|RVcoll.07-E019||Italy|658[0n]
Melanargia galathea|WMB3810-14|RVcoll.07-E008||Italy|658[0n]
Melanargia galathea|WMB3808-14|RVcoll.07-D888||Italy|658[0n]
Melanargia galathea|WMB5994-17|RVcoll10C654|Sicily|Italy|658[0n]
Melanargia galathea|WMB3837-14|RVcoll.09-X919||Italy|658[0n]
Melanargia galathea|WMB2026-13|RVcoll.12-Q703||Italy|658[0n]
Melanargia galathea|WMB2006-13|RVcoll.12-Q396||France|658[0n]
Melanargia galathea|WMB1729-13|RVcoll.12-P897||France|658[0n]
Melanargia galathea|WMB2089-13|RVcoll.12-R033|Sicily|Italy|658[0n]
Melanargia galathea|WMB2063-13|RVcoll.12-Q859||Italy|658[0n]
Melanargia galathea|WMB3801-14|RVcoll.12-O644||France|658[0n]
Melanargia galathea|WMB3652-14|RVcoll.11-I700||France|658[0n]
Melanargia galathea|WMB3512-14|RVcoll.10-B859||France|658[0n]
Melanargia galathea|WMB2872-14|RVcoll.10-C564|Argentario|Italy|658[0n]
Melanargia galathea|LEATB706-13|TLMF Lep 10883||Italy|658[0n]
Melanargia galathea|LEATG463-14|TLMF Lep 14250||Italy|658[0n]
Melanargia galathea|LEATJ1178-16|TLMF Lep 19501||Czech Republic|658[0n]
Melanargia galathea|GBLAC226-13|BC ZSM Lep 78607||Germany|658[0n]
Melanargia galathea|EULEP5060-16|RVcoll16J111||Czech Republic|658[0n]
Melanargia galathea|GWORA2473-09|BC ZSM Lep 30685||Germany|658[0n]
Melanargia galathea|WMB5008-14|RVcoll. 14-I555||Italy|658[0n]
Melanargia galathea|EULEP1268-15|RVcoll.14-F663||Greece|658[0n]
Melanargia galathea|WMB4964-14|RVcoll. 14-I511||Italy|658[0n]
Melanargia galathea|BIBSA242-15|RVcoll.14-E035||Italy|658[0n]
Melanargia galathea|OXB323-15|RVcoll.14-N037||Italy|658[0n]
Melanargia galathea|BIBSA442-15|RVcoll.14-I110||Italy|658[0n]
Melanargia galathea|WMB5007-14|RVcoll. 14-I554||Italy|658[0n]
Melanargia galathea|WMB4955-14|RVcoll. 14-I502||Italy|658[0n]
Melanargia galathea|BIBSA441-15|RVcoll.14-I109||Italy|658[0n]
Melanargia galathea|BIBSA944-15|16-A035||Italy|658[0n]
Melanargia galathea|BIBSA1223-15|15-L957||Italy|658[0n]
Melanargia galathea|WMB5013-14|RVcoll. 14-I560||Italy|658[0n]
Melanargia galathea|OXB1305-15|Rvcoll_15-M568||France|612[0n]
Melanargia galathea|EULEP4215-16|RVcoll10B361||Bulgaria|658[0n]
Melanargia galathea|LOWA527-06|2005-LOWA-527||Ukraine|658[0n]
Melanargia galathea|LOWA526-06|2005-LOWA-526||Ukraine|658[0n]
Melanargia galathea|BIBSA1059-15|13-U102||Italy|658[0n]
Melanargia galathea|OXB819-15|14-U782||France|658[0n]
Melanargia galathea|OXB818-15|14-U781||France|658[0n]
Melanargia galathea|BIBSA1242-15|15-F819||France|658[0n]
Melanargia galathea|EULEP1917-15|RVcoll.14-B837||Bosnia and Herzegovina|658[0n]
Melanargia galathea|WMB5031-14|RVcoll. 14-I578||Italy|658[0n]
Melanargia galathea|EULEP4217-16|RVcoll15G044||France|658[0n]
Melanargia galathea|EULEP4220-16|RVcoll15I352||Austria|658[0n]
Melanargia galathea|EULEP1076-15|RVcoll.14-F015||Serbia|658[0n]
Melanargia galathea|EULEP2388-15|RVcoll.14-V085||Ukraine|658[0n]
Melanargia galathea|BIBSA1243-15|15-F822||France|658[0n]
Melanargia galathea|BIBSA960-15|13-U101||Italy|658[0n]
Melanargia galathea|BIBSA1047-15|15-M629||France|658[0n]
Melanargia galathea|BIBSA1060-15|13-U481||Italy|658[0n]
Melanargia galathea|BIBSA1061-15|13-U482||Italy|658[0n]
Melanargia galathea|BIBSA1100-15|15-L750||Italy|658[0n]
Melanargia galathea|BIBSA1101-15|15-L751||Italy|658[0n]
Melanargia galathea|GBLAB152-13|BC ZSM Lep 75778||Germany|658[0n]
Melanargia galathea|OXB1542-16|OXB-TGS-1260||France|658[0n]
Melanargia galathea|OXB1307-15|Rvcoll_15-M570||France|658[0n]
Melanargia galathea|EZSPC1168-10|RVcoll.09-T073||Spain|658[0n]
Melanargia galathea|OXB1204-15|Rvcoll_15-M620||France|658[0n]
Melanargia galathea|OXB1233-15|Rvcoll_15-M668||France|658[0n]
Melanargia galathea|EULEP4216-16|RVcoll15G032||Belgium|637[0n]
Melanargia galathea|EULEP4222-16|RVcoll15G485||Switzerland|636[0n]
Melanargia galathea|WMB1074-13|RVcoll.10-C703||Italy|624[0n]
Melanargia galathea|EZSPC534-09|RVcoll.08-P374||Spain|641[0n]
Melanargia galathea|EZRMN096-08|RVcoll.08-M421||Romania|658[0n]
Melanargia galathea|EZROM398-08|RV-07-C971||Romania|658[0n]
Melanargia galathea|EZROM396-08|RV-06-M935||Romania|658[0n]
Melanargia galathea|EZROM395-08|RV-06-M927||Romania|658[0n]
Melanargia galathea|EZROM394-08|RV-06-M861||Romania|658[0n]
Melanargia galathea|EZRMN095-08|RVcoll.08-M394||Romania|658[0n]
Melanargia galathea|EZRMN094-08|RVcoll.07-C141||Romania|658[0n]
Melanargia galathea|EZROM397-08|RV-07-D440||Romania|658[0n]
Melanargia galathea|PHLAF652-11|TLMF Lep 05822||Austria|658[0n]
Melanargia galathea|EULEP4219-16|RVcoll15H988||Italy|658[0n]
Melanargia galathea|ABOLD051-16|TLMF Lep 21129||Austria|658[0n]
Melanargia galathea|BIBSA1425-15|16-A042||Italy|658[0n]
Melanargia galathea|EULEP1497-15|RVcoll.14-G362||Greece|658[0n]
Melanargia galathea|EULEP4988-16|RVcoll16I690||Poland|658[0n]
Melanargia galathea|LEATI042-15|TLMF Lep 17427||Austria|658[0n]
Melanargia galathea|LEATI043-15|TLMF Lep 17428||Austria|658[0n]
Oeneis tarpeia|EULEP3021-15|RVcoll.14-O154||Russia|658[0n]
Oeneis tarpeia|EULEP3022-15|RVcoll.14-O155||Russia|658[0n]
Oeneis tarpeia|EULEP3020-15|RVcoll.14-O153||Russia|658[0n]
Oeneis tarpeia|EULEP3019-15|RVcoll.14-O152||Russia|658[0n]
Oeneis tarpeia|EULEP459-14|KN00821||Russia|658[0n]
Oeneis tarpeia|EULEP458-14|KN00820||Russia|658[0n]
Oeneis tarpeia|EULEP421-14|KN00783||Russia|658[0n]
Oeneis bore|EULEP396-14|MM23881||Finland|658[0n]
Oeneis bore|LEFIF130-10|MM10586||Finland|658[0n]
Oeneis bore|LEFIF129-10|MM10585||Finland|658[0n]
Oeneis bore|EULEP4788-16|RVcoll16H441||Sweden|658[0n]
Oeneis bore|EULEP4722-16|RVcoll16B376||Sweden|658[0n]
Oeneis bore|EULEP397-14|MM23882||Finland|658[0n]
Oeneis bore|LEFIJ557-10|MM17182||Finland|658[0n]
Oeneis norna|EULEP3018-15|RVcoll.14-O151||Norway|658[0n]
Oeneis norna|LON804-11|NHMO Lep09002||Norway|658[0n]
Oeneis melissa|EULEP4356-16|RVcoll14V764||Russia|658[0n]
Oeneis melissa|EULEP455-14|KN00817||Russia|658[0n]

Oeneis norna|LON804-11|NHMO Lep09002||Norway|658[0n]
Oeneis melissa|EULEP4356-16|RVcoll14V764||Russia|658[0n]
Oeneis melissa|EULEP455-14|KN00817||Russia|658[0n]
Oeneis melissa|EULEP456-14|KN00818||Russia|658[0n]
Oeneis melissa|EULEP4358-16|RVcoll14V766||Russia|639[0n]
Oeneis melissa|EULEP4357-16|RVcoll14V765||Russia|658[0n]
Oeneis melissa|EULEP4355-16|RVcoll14V763||Russia|658[0n]
Oeneis jutta|EULEP462-14|KN00824||Russia|658[0n]
Oeneis jutta|EULEP4352-16|RVcoll14V767||Russia|658[0n]
Oeneis jutta|EULEP460-14|KN00822||Russia|658[0n]
Oeneis jutta|EULEP4353-16|RVcoll14V768||Russia|658[0n]
Oeneis jutta|EULEP461-14|KN00823||Russia|658[0n]
Oeneis jutta|EULEP2040-15|RVcoll.14-J519||Ukraine|658[0n]
Oeneis jutta|LEFIJ556-10|MM17181||Finland|658[0n]
Oeneis jutta|LON036-08|NHMO-06036||Norway|657[0n]
Oeneis jutta|LON201-08|NHMO-06210||Norway|657[0n]
Oeneis jutta|LON472-08|NHMO-08123||Norway|657[0n]
Oeneis jutta|LEFIC071-10|MM03356||Finland|658[0n]
Oeneis jutta|EULEP4361-16|RVcoll15Q074||Russia|658[0n]
Oeneis jutta|EULEP4745-16|RVcoll16G716||Sweden|658[0n]
Oeneis jutta|EULEP398-14|MM23883||Finland|658[0n]
Oeneis jutta|EULEP399-14|MM23884||Finland|658[0n]
Oeneis jutta|EULEP2276-15|RVcoll.14-N462||Ukraine|658[0n]
Oeneis jutta|EULEP2277-15|RVcoll.14-N463||Ukraine|658[0n]
Oeneis jutta|EULEP3016-15|RVcoll.14-O149||Estonia|658[0n]
Oeneis jutta|LEFIB189-10|MM00618||Finland|643[0n]
Oeneis glacialis|FBLMX212-11|BC ZSM Lep 50423||Germany|602[0n]
Oeneis glacialis|PHLSA387-11|TLMF Lep 05842||Austria|658[0n]
Oeneis glacialis|EULEP1866-15|RVcoll.13-U328||Italy|658[0n]
Oeneis norna|COLFF475-13|MM23185||Finland|658[1n]
Oeneis norna|COLFH608-15|MM24118||Finland|658[0n]
Oeneis norna|EULEP454-14|KN00816||Russia|658[0n]
Oeneis norna|EULEP4783-16|RVcoll16H379||Sweden|658[0n]
Oeneis norna|LEFIC093-10|MM03405||Finland|658[0n]
Oeneis glacialis|GWOSA786-10|BC ZSM Lep 36652||Germany|658[0n]
Oeneis norna|LEFIA745-10|MM04099||Finland|658[0n]
Oeneis norna|LON170-08|NHMO-06171||Norway|657[0n]
Oeneis glacialis|GWOTF672-12|BC ZSM Lep 62378||Germany|658[0n]
Oeneis norna|LEFIA021-10|MM00092||Finland|658[0n]
Oeneis glacialis|PHLAI558-13|TLMF Lep 09120||Austria|658[0n]
Oeneis glacialis|EULEP4345-16|RVcoll15H007||Switzerland|658[0n]
Oeneis glacialis|EULEP4346-16|RVcoll15H466||Switzerland|658[0n]
Oeneis glacialis|EULEP4350-16|RVcoll15J109||Switzerland|658[0n]
Oeneis glacialis|EULEP4351-16|RVcoll15O045||Italy|658[0n]
Oeneis norna|EULEP4764-16|RVcoll16H074||Sweden|658[0n]
Oeneis glacialis|EULEP4347-16|RVcoll15H576||Switzerland|637[0n]
Oeneis glacialis|EULEP4342-16|RVcoll14V907||Switzerland|638[0n]
Oeneis norna|COLFF474-13|MM23184||Finland|658[0n]
Oeneis glacialis|LEATG100-14|TLMF Lep 13887||Austria|658[0n]
Oeneis glacialis|PHLAB287-10|TLMF Lep 01087||Switzerland|658[0n]
Oeneis glacialis|EULEP4344-16|RVcoll15G744||Switzerland|658[0n]
Oeneis glacialis|EULEP4343-16|RVcoll15G686||Italy|658[0n]
Oeneis glacialis|EULEP4349-16|RVcoll15I334||Austria|658[0n]
Oeneis glacialis|EULEP4348-16|RVcoll15I166||Austria|658[0n]
Oeneis glacialis|PHLAI559-13|TLMF Lep 09121||Austria|658[0n]
Oeneis glacialis|LEATC087-13|TLMF Lep 11214||Italy|658[0n]
Oeneis glacialis|LEATC086-13|TLMF Lep 11213||Italy|658[0n]
Oeneis glacialis|BIBSA251-15|RVcoll.14-E047||Italy|658[0n]
Oeneis glacialis|PHLAA392-09|TLMF Lep 00432||Italy|658[0n]
Satyrus ferula|EULEP3137-15|RVcoll.14-O270||Russia|658[0n]
Satyrus ferula|EULEP5569-17|RVcoll14F993||Greece|655[0n]
Satyrus ferula|EULEP5568-17|RVcoll14F917||Greece|658[0n]
Satyrus ferula|EULEP5571-17|RVcoll14G141||Greece|655[0n]
Satyrus ferula|EULEP5567-17|RVcoll14F866||Greece|655[0n]
Satyrus ferula|EULEP5562-17|RVcoll14F350||Bulgaria|655[0n]
Satyrus ferula|EULEP5575-17|RVcoll14G543||Macedonia|655[0n]
Satyrus ferula|EULEP5573-17|RVcoll14G520||Greece|655[0n]
Satyrus ferula|EULEP5540-17|RVcoll14B778||Albania|655[0n]
Satyrus ferula|OXB1560-16|OXB-TGS-1278||France|658[0n]
Satyrus ferula|WMB1660-13|RVcoll.12-P145||France|658[0n]
Satyrus ferula|EULEP5448-17|RVcoll11I661||France|655[0n]
Satyrus ferula|WMB1826-13|RVcoll.11-I706||France|658[0n]
Satyrus ferula|WMB2007-13|RVcoll.12-Q404||France|658[0n]
Satyrus ferula|OXB577-15|14-N966||Italy|658[0n]
Satyrus ferula|BIBSA249-15|RVcoll.14-E045||Italy|658[0n]
Satyrus ferula|BIBSA430-15|RVcoll.14-I098||Italy|658[0n]
Satyrus ferula|WMB5030-14|RVcoll. 14-I577||Italy|658[0n]
Satyrus ferula|WMB1788-13|RVcoll.12-Q214||France|658[0n]
Satyrus ferula|WMB1077-13|RVcoll.10-C711||Italy|658[0n]
Satyrus ferula|WMB3519-14|RVcoll.10-B968||France|658[0n]
Satyrus ferula|LEATG500-14|TLMF Lep 14287||Italy|658[0n]
Satyrus ferula|LEATG501-14|TLMF Lep 14288||Italy|658[0n]
Satyrus virbius|EULEP2527-15|RVcoll.14-V401||Ukraine|658[0n]
Satyrus virbius|EULEP2526-15|RVcoll.14-V400||Ukraine|658[0n]
Satyrus virbius|EULEP2057-15|RVcoll.14-J542||Ukraine|658[0n]
Satyrus virbius|EULEP2056-15|RVcoll.14-J541||Ukraine|658[0n]
Satyrus ferula|EULEP3136-15|RVcoll.14-O269||Russia|658[0n]
Satyrus ferula|EULEP3139-15|RVcoll.14-O272||Russia|658[0n]
Satyrus ferula|EULEP5565-17|RVcoll14F629||Greece|655[0n]
Satyrus ferula|EULEP5380-17|RVcoll10A969||Bulgaria|655[0n]
Satyrus ferula|EULEP5563-17|RVcoll14F387||Bulgaria|658[0n]
Satyrus ferula|EULEP5529-17|RVcoll14A969||Bulgaria|658[0n]
Satyrus ferula|EULEP5102-17|RVcoll16J856||Romania|658[0n]
Satyrus ferula|EULEP5583-17|RVcoll14H997||Romania|655[0n]
Satyrus ferula|EULEP5582-17|RVcoll14H996||Romania|655[0n]
Satyrus ferula|EULEP5581-17|RVcoll14H973||Romania|655[0n]
Satyrus ferula|EULEP5515-17|RVcoll14A038||Romania|658[0n]
Satyrus ferula|EULEP5526-17|RVcoll14A594||Italy|655[0n]
Satyrus ferula|BIBSA759-15|LEP-SS-00260||Italy|658[0n]
Satyrus ferula|BIBSA758-15|LEP-SS-00259||Italy|658[0n]
Satyrus ferula|WMB2379-13|RVcoll.07-E012||Italy|658[0n]
Satyrus ferula|WMB2047-13|RVcoll.12-Q781||Italy|658[0n]
Satyrus ferula|BIBSA952-15|09-X924||Italy|658[0n]

Satyrus ferula|WMB2379-13|RVcoll.07-E012||Italy|658[0n]
Satyrus ferula|WMB2047-13|RVcoll.12-Q781||Italy|658[0n]
Satyrus ferula|BIBSA952-15|09-X924||Italy|658[0n]
Satyrus ferula|WMB2045-13|RVcoll.12-Q779||Italy|658[0n]
Satyrus ferula|EZSPM862-12|Rvcoll.070611MH08||Spain|658[0n]
Satyrus ferula|EZSPM864-12|Rvcoll.070611MH61||Spain|658[0n]
Satyrus ferula|EZSPM078-09|RVcoll.08-P358||Spain|658[0n]
Satyrus ferula|EZSPN1079-11|RVcoll.09-X037||Spain|632[0n]
Satyrus ferula|EZSPN1013-09|RVcoll.08-M933||Spain|658[0n]
Satyrus ferula|EZSPM178-09|RVcoll.08-R124||Spain|658[0n]
Satyrus ferula|EZSPN1068-11|Rvcoll.08-R263||Spain|658[0n]
Satyrus ferula|EZSPN250-09|RVcoll.07-W145||Spain|658[0n]
Satyrus ferula|EZSPN249-09|RVcoll.07-W144||Spain|658[0n]
Satyrus actaea|WMB4436-14|RVcoll.14-B535||Portugal|658[0n]
Satyrus actaea|WMB3195-14|RVcoll.08-J061||Spain|658[0n]
Satyrus actaea|WMB3386-14|RVcoll.09-V467||Spain|658[0n]
Satyrus actaea|EZSPM460-09|Rvcoll.09-V933||Spain|658[0n]
Satyrus actaea|WMB3666-14|RVcoll.11-I877||France|658[0n]
Satyrus actaea|WMB1830-13|RVcoll.11-I788||France|658[0n]
Satyrus actaea|OXB1184-15|Rvcoll_16-A071||Italy|658[0n]
Satyrus actaea|WMB1827-13|RVcoll.11-I711||France|658[0n]
Satyrus actaea|EZSPN729-09|RVcoll.08-L137||Spain|658[0n]
Satyrus actaea|EZSPM128-09|RVcoll.08-P623||Spain|658[0n]
Satyrus actaea|WMB3137-14|RVcoll.06-G654||Spain|658[0n]
Satyrus actaea|EULEP5876-18|RVcoll15O513||Spain|658[0n]
Satyrus actaea|WMB3151-14|RVcoll.07-F086||Spain|621[0n]
Satyrus actaea|EZSPM017-09|RVcoll.08-P014||Spain|658[0n]
Satyrus actaea|EZSPM421-09|Rvcoll.09-V845||Spain|658[0n]
Satyrus actaea|EZSPN186-09|RVcoll.07-C088||Spain|658[0n]
Satyrus actaea|EZSPN770-09|RVcoll.08-L274||Spain|658[0n]
Satyrus actaea|EZSPN504-09|RVcoll.08-H959||Spain|658[0n]
Satyrus actaea|EZSPN503-09|RVcoll.08-H958||Spain|658[0n]
Satyrus actaea|EZSPN550-09|RVcoll.08-J056||Spain|658[0n]
Satyrus actaea|WMB4042-14|RVcoll.12-Z360||Spain|658[0n]
Satyrus actaea|WMB3212-14|RVcoll.08-J408||Spain|658[0n]
Satyrus actaea|EZSPM858-12|Rvcoll.040611HZ71||Spain|658[0n]
Satyrus actaea|HBOK011-08|OK519-11||Spain|658[0n]
Satyrus actaea|HBOK012-08|OK519-12||Spain|658[0n]
Chazara persephone|EULEP2499-15|RVcoll.14-V360||Ukraine|658[0n]
Chazara persephone|EULEP2487-15|RVcoll.14-V344||Ukraine|611[0n]
Chazara persephone|EULEP2060-15|RVcoll.14-J545||Ukraine|658[0n]
Chazara persephone|EULEP2059-15|RVcoll.14-J544||Ukraine|658[0n]
Chazara persephone|EULEP2058-15|RVcoll.14-J543||Ukraine|658[0n]
Chazara briseis|EULEP2190-15|RVcoll.14-N266|Cyprus|Cyprus|658[0n]
Chazara briseis|EZSPC216-09|RVcoll.08-J400||Spain|658[0n]
Chazara briseis|EULEP2191-15|RVcoll.14-N271|Cyprus|Cyprus|658[0n]
Chazara briseis|EULEP3636-16|RVcoll14A927||Cyprus|658[0n]
Chazara briseis|EZSPC1368-10|RVcoll.09-X060||Spain|658[0n]
Chazara briseis|EULEP1544-15|RVcoll.14-G514||Greece|658[0n]
Chazara briseis|EULEP1509-15|RVcoll.14-G423||Greece|658[0n]
Chazara briseis|EULEP1487-15|RVcoll.14-G318||Greece|658[0n]
Chazara briseis|EULEP927-15|RVcoll.14-C816||Bulgaria|658[0n]
Chazara briseis|EULEP1553-15|RVcoll.14-G542||Macedonia|614[0n]
Chazara briseis|EZSPC239-09|RVcoll.08-P026||Spain|656[0n]
Chazara briseis|EZSPC238-09|RVcoll.08-P015||Spain|657[0n]
Chazara briseis|EZROM089-08|RV-06-V673|Genit. examined|Romania|658[0n]
Chazara briseis|WMB2846-13|RVcoll.12-Q756||Italy|658[0n]
Chazara briseis|EZSPC991-10|RVcoll.07-F080.1||Spain|658[0n]
Chazara briseis|EZROM714-08|RV-06-V746||Spain|658[0n]
Chazara briseis|WMB743-12|RVcoll.11-J688|Sicily|Italy|658[0n]
Chazara briseis|WMB701-12|RVcoll.11-H291|Sicily|Italy|658[0n]
Chazara briseis|WMB700-12|RVcoll.11-H289|Sicily|Italy|658[0n]
Chazara briseis|WMB2418-13|RVcoll.10-C657|Sicily|Italy|658[0n]
Chazara briseis|WMB2927-14|RVcoll.11-J725|Sicily|Italy|658[0n]
Chazara briseis|EZSPM767-12|Rvcoll. 040611HZ50||Spain|658[0n]
Chazara briseis|EULEP3634-16|RVcoll15J555||France|638[0n]
Chazara briseis|WMB2009-13|RVcoll.12-Q571||France|658[0n]
Chazara briseis|GBLAF768-14|BC ZSM Lep 82854||Germany|658[0n]
Chazara briseis|BIBSA857-15|LD-3469||Italy|658[0n]
Chazara briseis|EULEP5082-16|RVcoll16J338||Germany|658[0n]
Chazara briseis|WMB736-12|RVcoll.11-I879||France|658[0n]
Chazara briseis|WMB1698-13|RVcoll.12-P581||France|658[0n]
Chazara briseis|WMB1644-13|RVcoll.12-O953||France|658[0n]
Chazara briseis|WMB3272-14|RVcoll.08-L933||Spain|658[0n]
Chazara briseis|EZSPC1134-10|RVcoll.08-P716||Spain|658[0n]
Chazara briseis|EZSPC1339-10|RVcoll.130209KL91||Spain|658[0n]
Chazara briseis|EZSPC1318-10|RVcoll.130209KL92||Spain|658[0n]
Chazara briseis|EZSPM127-09|RVcoll.08-P622||Spain|658[0n]
Chazara briseis|EZSPN957-09|RVcoll.08-L954||Spain|656[0n]
Chazara briseis|EZSPN287-09|RVcoll.07-W240||Spain|658[0n]
Chazara briseis|EZSPN755-09|RVcoll.08-L231||Spain|658[0n]
Chazara briseis|WMB2037-13|RVcoll.12-Q757||Italy|658[0n]
Chazara briseis|WMB2124-13|RVcoll.12-R218||Italy|658[0n]
Chazara briseis|WMB2117-13|RVcoll.12-R189||Italy|658[0n]
Chazara briseis|EZROM958-08|RVcoll.06-V677.1||Romania|646[2n]
Chazara briseis|LEATG457-14|TLMF Lep 14244||Italy|658[0n]
Chazara briseis|GWORU361-10|BC ZSM Lep 31952||Italy|658[0n]
Chazara briseis|GWORZ038-10|BC ZSM Lep 30394||Italy|658[0n]
Chazara briseis|EZROM847-08|RVcoll.06-V672||Romania|658[0n]
Chazara briseis|EZROM848-08|RVcoll.06-V676||Romania|658[0n]
Chazara briseis|EZROM090-08|RV-06-V675||Romania|658[0n]
Chazara briseis|EZROM667-08|RV-06-V674||Romania|658[0n]
Chazara briseis|EZSPC258-09|RVcoll.08-P363||Spain|658[0n]
Chazara briseis|WMB3939-14|RVcoll.12-P624||France|658[0n]
Chazara briseis|EULEP3635-16|RVcoll15Q179||Ukraine|658[0n]
Chazara briseis|EULEP175-14|RVcoll.11-J337||Romania|658[0n]
Chazara briseis|EULEP861-15|RVcoll.14-C211||Greece|658[0n]
Chazara briseis|LEASS990-17|TLMF Lep 22543||Austria|658[0n]
Chazara briseis|LEATG456-14|TLMF Lep 14243||Italy|625[0n]
Chazara briseis|LEATJ1184-16|TLMF Lep 19507||Czech Republic|658[0n]
Chazara briseis|ABOLD037-16|TLMF Lep 21115||Austria|658[0n]
Chazara briseis|ABOLD418-16|TLMF Lep 21570||Austria|658[0n]
[illegible]

Chazara briseis|LEATJ1184-16|TLMF Lep 19507||Czech Republic|658[0n]
Chazara briseis|ABOLD037-16|TLMF Lep 21115||Austria|658[0n]
Chazara briseis|ABOLD418-16|TLMF Lep 21570||Austria|658[0n]
Chazara prieuri|EZSPC1305-10|RVcoll.130209KL2||Spain|658[0n]
Chazara prieuri|EZSPM679-12|Rvcoll. 12-L035||Spain|643[0n]
Chazara prieuri|EZSPC1325-10|RVcoll.130209KL33||Spain|658[0n]
Chazara prieuri|EZSPC805-10|RVcoll.09-X324||Spain|658[0n]
Chazara prieuri|EZSPM680-12|Rvcoll. 12-L036||Spain|658[0n]
Chazara prieuri|EZSPM678-12|Rvcoll. 08-J476||Spain|658[0n]
Chazara prieuri|EZSPM675-12|Rvcoll. 08-J473||Spain|658[0n]
Chazara prieuri|EZSPM674-12|Rvcoll. 08-J472||Spain|658[0n]
Chazara prieuri|EZSPM677-12|Rvcoll. 08-J475||Spain|658[0n]
Chazara prieuri|EZSPM676-12|Rvcoll. 08-J474||Spain|658[0n]
Chazara prieuri|EZSPM826-12|Rvcoll. 140611ZG87||Spain|658[0n]
Chazara prieuri|EZSPM768-12|Rvcoll. 040611HZ54||Spain|658[0n]
Chazara prieuri|HBOK033-08|OK519-33||Spain|655[0n]
Arethusana arethusa|EZSPN1116-11|Rvcoll.10-C315||Spain|658[0n]
Arethusana arethusa|EZSPN1115-11|Rvcoll.10-C314||Spain|658[0n]
Arethusana arethusa|WMB6546-18|RVcoll15D788||Spain|658[0n]
Arethusana arethusa|EZSPC740-10|RVcoll.08-J829.1||Spain|658[0n]
Arethusana arethusa|WMB6548-18|RVcoll15D790||Spain|658[0n]
Arethusana arethusa|WMB3413-14|RVcoll.09-V992||France|658[0n]
Arethusana arethusa|EZSPM582-12|RVcoll.08-P940||Spain|658[0n]
Arethusana arethusa|EZSPM579-12|RVcoll.08-P937||Spain|658[0n]
Arethusana arethusa|EZSPC1397-10|RVcoll.09-V108||Spain|633[0n]
Arethusana arethusa|EZSPC1442-10|RVcoll.09-V154||Spain|658[0n]
Arethusana arethusa|EZSPC1441-10|RVcoll.09-V153||Spain|658[0n]
Arethusana arethusa|EZSPC1396-10|RVcoll.09-V107||Spain|658[0n]
Arethusana arethusa|EZSPN792-09|RVcoll.08-L351||Spain|658[0n]
Arethusana arethusa|EZROM598-08|RV-07-E432||Romania|658[0n]
Arethusana arethusa|OXB1183-15|Rvcoll_16-A070||Italy|658[0n]
Arethusana arethusa|WMB2422-13|RVcoll.10-C713||Italy|658[0n]
Arethusana arethusa|WMB1793-13|RVcoll.12-Q311||France|658[0n]
Arethusana arethusa|WMB1078-13|RVcoll.10-C712||Italy|658[0n]
Arethusana arethusa|WMB3518-14|RVcoll.10-B960||France|658[0n]
Arethusana arethusa|WMB746-12|RVcoll.11-J870||Italy|635[0n]
Arethusana arethusa|WMB745-12|RVcoll.11-J869||Italy|658[0n]
Arethusana arethusa|WMB117-11|RVcoll.10-B679||France|655[0n]
Arethusana arethusa|WMB726-12|RVcoll.11-I638||France|658[0n]
Arethusana arethusa|ABOLD589-17|TLMF Lep 21665||Austria|658[0n]
Arethusana arethusa|LEASS495-17|TLMF Lep 22143||Austria|658[0n]
Arethusana arethusa|LEASS986-17|TLMF Lep 22539||Austria|658[0n]
Arethusana arethusa|LEATJ1231-16|TLMF Lep 19554||Hungary|658[0n]
Arethusana arethusa|EULEP929-15|RVcoll.14-C843||Bulgaria|658[0n]
Arethusana arethusa|EULEP5058-16|RVcoll16J109||Czech Republic|658[0n]
Arethusana arethusa|EZROM660-08|RV-06-V709||Romania|658[0n]
Arethusana arethusa|EZROM023-08|RV-06-V702||Romania|658[0n]
Arethusana arethusa|EZROM774-08|RVcoll.06-V710||Romania|658[0n]
Arethusana arethusa|EZROM773-08|RVcoll.06-V707||Romania|658[0n]
Arethusana arethusa|EZROM957-08|RVcoll.06-V708.1||Romania|658[0n]
Arethusana arethusa|LENOA1370-11|LN-BD1370||France|633[0n]
Arethusana arethusa|EZSPC198-09|RVcoll.06-G565||Spain|656[0n]
Arethusana arethusa|EULEP3450-16|RVcoll14V721||Spain|658[0n]
Arethusana arethusa|WMB6547-18|RVcoll15D789||Spain|658[0n]
Arethusana arethusa|EZSPC956-10|RVcoll.09-X570||Spain|658[0n]
Arethusana arethusa|EULEP2626-15|RVcoll.14-N566||France|658[0n]
Arethusana arethusa|EZSPM764-12|Rvcoll. 040611HZ07||Spain|658[0n]
Arethusana arethusa|EZSPC802-10|RVcoll.08-P428||Spain|658[0n]
Arethusana arethusa|EZSPC801-10|RVcoll.08-P427||Spain|658[0n]
Arethusana arethusa|EZSPM098-09|RVcoll.08-P426||Spain|658[0n]
Arethusana arethusa|EZSPC267-09|RVcoll.08-R155||Spain|656[0n]
Arethusana arethusa|EZSPN756-09|RVcoll.08-L233||Spain|658[0n]
Arethusana arethusa|EZSPN1094-11|Rvcoll.10-A008||Spain|658[0n]
Arethusana arethusa|EZSPN1095-11|Rvcoll.10-A012||Spain|658[0n]
Arethusana arethusa|EULEP253-14|RVcoll.12-Q447||Spain|658[0n]
Arethusana arethusa|EZSPC197-09|RVcoll.06-G545||Spain|654[0n]
Arethusana arethusa|EZROM775-08|RVcoll.07-E431||Romania|658[0n]
Arethusana arethusa|EULEP5190-17|RVcoll16I962||Slovakia|641[0n]
Arethusana arethusa|EZROM024-08|RV-07-E414||Romania|658[0n]
Arethusana arethusa|EZSPC818-10|RVcoll.08-P699||Spain|658[0n]
Arethusana arethusa|EZSPM105-09|RVcoll.08-P440||Spain|658[0n]
Arethusana arethusa|EZSPC816-10|RVcoll.08-P617||Spain|658[0n]
Arethusana arethusa|EZSPC942-10|RVcoll.09-X057||Spain|658[0n]
Arethusana arethusa|EZSPM850-12|Rvcoll. 170410YM76||Spain|658[0n]
Arethusana arethusa|WMB3479-14|RVcoll.10-A898||Spain|658[0n]
Arethusana arethusa|WMB852-13|RVcoll.08-R301||France|658[0n]
Arethusana arethusa|WMB5154-14|RVcoll.12-P605||France|658[0n]
Arethusana arethusa|EULEP254-14|RVcoll.12-Q448||Spain|658[0n]
Arethusana arethusa|EULEP683-15|RVcoll.11-J868||Slovenia|658[0n]
Arethusana arethusa|EULEP887-15|RVcoll.14-C526||Greece|658[0n]
Arethusana arethusa|EZSPN465-09|RVcoll.08-H679||Spain|658[1n]
Arethusana arethusa|EZSPC252-09|RVcoll.08-P333||Spain|655[0n]
Arethusana arethusa|EZSPC202-09|RVcoll.06-V774||Spain|658[0n]
Arethusana arethusa|ABOLD038-16|TLMF Lep 21116||Austria|658[0n]
Brintesia circe|EZSPN985-09|RVcoll.08-M025||Spain|658[0n]
Brintesia circe|EZSPC217-09|RVcoll.08-J416||Spain|658[0n]
Brintesia circe|LEASS690-17|TLMF Lep 22338||Austria|658[0n]
Brintesia circe|LEASS896-17|KLM Lep 08401||Austria|658[0n]
Brintesia circe|EULEP4928-16|RVcoll16J781||Russia|658[0n]
Brintesia circe|EULEP1887-15|RVcoll.14-B697||Albania|658[0n]
Brintesia circe|EULEP1552-15|RVcoll.14-G540||Macedonia|658[0n]
Brintesia circe|EULEP1479-15|RVcoll.14-G266||Greece|658[0n]
Brintesia circe|EULEP4854-16|RVcoll16H786||Bulgaria|658[0n]
Brintesia circe|EZROM822-08|RVcoll.08-M541||Romania|658[0n]
Brintesia circe|EZROM821-08|RVcoll.08-M489||Romania|658[0n]
Brintesia circe|EZROM818-08|RVcoll.08-M468||Romania|658[0n]
Brintesia circe|EZROM066-08|RV-06-V716||Romania|640[0n]
Brintesia circe|EZRMN343-08|RVcoll.08-H039||Romania|649[0n]
Brintesia circe|EZSPN576-09|RVcoll.08-J123||Portugal|658[0n]
Brintesia circe|OXB722-15|13-U128||Italy|658[0n]
Brintesia circe|WMB4921-14|RVcoll.14-I468||Italy|658[0n]
Brintesia circe|BIBSA1869-17|RVcoll16C764||Italy|658[0n]
Brintesia circe|BIBSA1868-17|RVcoll16C762||Italy|658[0n]

Brintesia circe|WMB4921-14|RVcoll.14-I468||Italy|658[0n]
Brintesia circe|BIBSA1869-17|RVcoll16C764||Italy|658[0n]
Brintesia circe|BIBSA1868-17|RVcoll16C762||Italy|658[0n]
Brintesia circe|WMB4285-14|RVcoll.14-A336||Italy|658[0n]
Brintesia circe|EZSPC658-09|RVcoll.08-L383||Spain|658[0n]
Brintesia circe|EULEP1162-15|RVcoll.14-F366||Bulgaria|658[0n]
Brintesia circe|EZSPM372-09|RVcoll.08-J856||Spain|658[0n]
Brintesia circe|BIBSA347-15|RVcoll.14-I015||Italy|658[0n]
Brintesia circe|WMB3199-14|RVcoll.08-J073||Spain|658[0n]
Brintesia circe|WMB1675-13|RVcoll.12-P360||France|658[0n]
Brintesia circe|WMB3256-14|RVcoll.08-L461|Genit. examined|Spain|658[0n]
Brintesia circe|EZSPC1090-10|RVcoll.08-L905||Spain|658[0n]
Brintesia circe|WMB114-11|RVcoll.10-B657||France|658[0n]
Brintesia circe|WMB3657-14|RVcoll.11-I767||France|658[0n]
Brintesia circe|WMB1996-13|RVcoll.12-Q344||France|658[0n]
Brintesia circe|WMB4383-14|RVcoll.14-B242||Portugal|658[0n]
Brintesia circe|EZSPN560-09|RVcoll.08-J088||Spain|636[0n]
Brintesia circe|EZSPN466-09|RVcoll.08-H683||Spain|658[0n]
Brintesia circe|EULEP2107-15|RVcoll.14-J598||Ukraine|658[0n]
Brintesia circe|EULEP4929-16|RVcoll16J782||Russia|658[0n]
Brintesia circe|BIBSA348-15|RVcoll.14-I016||Italy|658[0n]
Brintesia circe|WMB3508-14|RVcoll.10-B786||France|658[0n]
Brintesia circe|BIBSA456-15|RVcoll.14-I124||Italy|658[0n]
Brintesia circe|EULEP743-15|RVcoll.12-N863|Lesvos|Greece|658[0n]
Brintesia circe|EULEP744-15|RVcoll.12-N864|Lesvos|Greece|658[0n]
Brintesia circe|BIBSA118-15|RVcoll.14-D553||Italy|658[0n]
Brintesia circe|BIBSA948-15|16-A039||Italy|658[0n]
Brintesia circe|BIBSA463-15|RVcoll.14-I131||Italy|658[0n]
Brintesia circe|EULEP3583-16|RVcoll15J564||France|639[0n]
Brintesia circe|WMB5290-14|RVcoll.14-H968||France|658[0n]
Brintesia circe|WMB5356-14|RVcoll.14-J699||France|658[0n]
Brintesia circe|WMB3974-14|RVcoll.12-Q204||France|658[0n]
Brintesia circe|EZROM610-08|RV-06-V717||Romania|658[0n]
Brintesia circe|EZROM067-08|RV-07-E381||Romania|658[0n]
Brintesia circe|EZROM820-08|RVcoll.08-M488||Romania|658[0n]
Brintesia circe|EULEP3582-16|RVcoll10A974||Bulgaria|658[0n]
Brintesia circe|EZROM819-08|RVcoll.08-M469||Romania|658[0n]
Brintesia circe|EZROM727-08|RV-07-C035|Genit. examined|Spain|658[0n]
Brintesia circe|WMB2033-13|RVcoll.12-Q742||Italy|658[0n]
Brintesia circe|WMB2840-13|RVcoll.12-Q661||Italy|658[0n]
Brintesia circe|OXB1031-15|LD-1426||France|658[0n]
Brintesia circe|WMB2611-13|RVcoll.12-O200|Corsica|France|658[0n]
Brintesia circe|WMB1530-13|RVcoll.12-O236|Corsica|France|658[0n]
Brintesia circe|WMB1522-13|RVcoll.12-O188|Corsica|France|658[0n]
Brintesia circe|BIBSA627-15|RVcoll. 15-A790||Italy|658[0n]
Brintesia circe|BIBSA1617-16|15-C392||Italy|658[0n]
Brintesia circe|EZSPC1200-10|RVcoll.09-V425||Spain|658[0n]
Brintesia circe|EZSPC1356-10|RVcoll.09-V929||Spain|658[0n]
Brintesia circe|WMB3299-14|RVcoll.08-P343|Genit. examined|Spain|620[0n]
Brintesia circe|EZSPC231-09|RVcoll.08-M694||Spain|656[0n]
Brintesia circe|EZSPC679-09|RVcoll.08-L384||Spain|658[0n]
Brintesia circe|WMB4266-14|RVcoll.14-A144||Italy|658[0n]
Brintesia circe|GWORR422-10|BC ZSM Lep 29638||Italy|658[0n]
Brintesia circe|GWOSK908-11|BC ZSM Lep 49409||Germany|658[0n]
Brintesia circe|EZSPM956-12|Rvcoll.12-M707||Spain|658[0n]
Brintesia circe|WMB236-11|RVcoll.09-T567||Italy|658[0n]
Brintesia circe|WMB2793-13|RVcoll.11-J645|Sicily|Italy|658[0n]
Brintesia circe|WMB1227-13|RVcoll.11-H287|Sicily|Italy|658[0n]
Brintesia circe|WMB1232-13|RVcoll.11-H347|Sicily|Italy|658[0n]
Brintesia circe|WMB864-13|RVcoll.09-T527|Corsica|France|658[0n]
Brintesia circe|WMB1084-13|RVcoll.10-C736||Italy|658[0n]
Brintesia circe|WMB2191-13|RVcoll.09-X870|Sardinia|Italy|658[0n]
Brintesia circe|WMB4150-14|RVcoll.13-S693||Italy|658[0n]
Brintesia circe|WMB4617-14|RVcoll.LD-2138|Sicily|Italy|658[0n]
Brintesia circe|WMB4618-14|RVcoll.LD-2139|Sicily|Italy|658[0n]
Brintesia circe|BIBSA1228-15|16-A107||Italy|658[0n]
Brintesia circe|BIBSA1769-16|LD-1169||Italy|658[0n]
Brintesia circe|BIBSA097-15|RVcoll.14-D513||Italy|658[0n]
Brintesia circe|BIBSA1396-15|15-N000||Italy|658[0n]
Brintesia circe|BIBSA694-15|RVcoll. 07-E079||Italy|658[0n]
Brintesia circe|BIBSA512-15|LEP-SS-00132||Italy|658[0n]
Brintesia circe|BIBSA517-15|LEP-SS-00137||Italy|658[0n]
Brintesia circe|OXB840-15|14-U803||Italy|658[0n]
Brintesia circe|OXB1164-15|Rvcoll_16-A051||Italy|658[0n]
Brintesia circe|ABOLD585-17|TLMF Lep 21661||Austria|658[0n]
Brintesia circe|ABOLD048-16|TLMF Lep 21126||Austria|632[0n]
Pseudochazara graeca|EULEP1430-15|RVcoll.14-G111||Greece|658[0n]
Pseudochazara graeca|EULEP1445-15|RVcoll.14-G146||Greece|658[0n]
Pseudochazara graeca|EULEP1427-15|RVcoll.14-G105||Greece|658[0n]
Pseudochazara graeca|EULEP1410-15|RVcoll.14-G077|Genit. examined|Greece|658[0n]
Pseudochazara graeca|EULEP1409-15|RVcoll.14-G076|Genit. examined|Greece|658[0n]
Pseudochazara geyeri|EULEP3107-15|RVcoll.14-O240||Macedonia|658[0n]
Pseudochazara geyeri|EULEP3106-15|RVcoll.14-O239||Macedonia|658[0n]
Pseudochazara geyeri|EULEP3105-15|RVcoll.14-O238||Macedonia|658[0n]
Pseudochazara geyeri|EULEP2293-15|RVcoll.14-N493||Greece|658[0n]
Pseudochazara graeca|EULEP2180-15|RVcoll.14-N219||Greece|658[0n]
Pseudochazara graeca|EULEP849-15|RVcoll.14-C132|Genit. examined|Greece|658[0n]
Pseudochazara graeca|EULEP848-15|RVcoll.14-C131|Genit. examined|Greece|658[0n]
Pseudochazara amymone|EULEP2186-15|RVcoll.14-N226||Albania|658[0n]
Pseudochazara amymone|EULEP2185-15|RVcoll.14-N225||Albania|658[0n]
Pseudochazara amymone|EULEP4561-16|RVcoll14O350||Albania|658[0n]
Pseudochazara amymone|EULEP4560-16|RVcoll14O349||Albania|658[0n]
Pseudochazara amalthea|EULEP2556-15|RVcoll.14-V479||Greece|658[0n]
Pseudochazara amalthea|EULEP3102-15|RVcoll.14-O235||Albania|658[0n]
Pseudochazara amalthea|EULEP3101-15|RVcoll.14-O234||Macedonia|658[0n]
Pseudochazara amalthea|EULEP1836-15|RVcoll.14-J958||Albania|658[0n]
Pseudochazara amalthea|EULEP1835-15|RVcoll.14-J957||Albania|658[0n]
Pseudochazara amalthea|EULEP1440-15|RVcoll.14-G132||Greece|658[0n]
Pseudochazara amalthea|EULEP1423-15|RVcoll.14-G100||Greece|658[0n]
Pseudochazara amalthea|EULEP1386-15|RVcoll.14-F988||Greece|658[0n]
Pseudochazara amalthea|EULEP1368-15|RVcoll.14-F956||Greece|658[0n]
Pseudochazara amalthea|EULEP507-15|RVcoll.07-C536||Greece|658[0n]
Pseudochazara amalthea|EULEP1614-15|RVcoll.14-G683||Greece|614[0n]

Pseudochazara amalthea|EULEP13[illegible]-15|RVcoll.14-F9[illegible]||Greece|658[0n]
Pseudochazara amalthea|EULEP1368-15|RVcoll.14-F956||Greece|658[0n]
Pseudochazara amalthea|EULEP507-15|RVcoll.07-C536||Greece|658[0n]
Pseudochazara amalthea|EULEP1614-15|RVcoll.14-G683||Greece|614[0n]
Pseudochazara amalthea|EULEP1510-15|RVcoll.14-G426||Greece|658[0n]
Pseudochazara amalthea|EULEP1506-15|RVcoll.14-G414||Greece|658[0n]
Pseudochazara amalthea|EULEP1298-15|RVcoll.14-F731||Greece|658[0n]
Pseudochazara anthelea|EULEP3097-15|RVcoll.14-O230|Cyprus|Cyprus|658[0n]
Pseudochazara anthelea|EULEP739-15|RVcoll.12-N853|Lesvos|Greece|658[0n]
Pseudochazara anthelea|EULEP3099-15|RVcoll.14-O232|Samos|Greece|658[0n]
Pseudochazara anthelea|EULEP679-15|RVcoll.11-J531|Rhodes|Greece|658[0n]
Pseudochazara williamsi|EZSPC1430-10|RVcoll.09-V141||Spain|658[0n]
Pseudochazara williamsi|EZSPC1429-10|RVcoll.09-V140||Spain|658[0n]
Pseudochazara williamsi|EZSPC1440-10|RVcoll.09-V152||Spain|658[0n]
Pseudochazara williamsi|EZSPC1433-10|RVcoll.09-V144||Spain|658[0n]
Pseudochazara williamsi|EZSPN791-09|RVcoll.08-L350||Spain|658[0n]
Pseudochazara mercurius|EULEP488-14|KN00850||Russia|658[0n]
Pseudochazara mercurius|EULEP487-14|KN00849||Russia|658[0n]
Pseudochazara euxina|EULEP2517-15|RVcoll.14-V386||Ukraine|658[0n]
Pseudochazara euxina|EULEP2055-15|RVcoll.14-J540||Ukraine|658[0n]
Pseudochazara euxina|EULEP2054-15|RVcoll.14-J539||Ukraine|658[0n]
Pseudochazara euxina|EULEP2516-15|RVcoll.14-V385||Ukraine|658[0n]
Pseudochazara euxina|EULEP2515-15|RVcoll.14-V384||Ukraine|658[0n]
Pseudochazara euxina|EULEP453-14|KN00815||Ukraine|658[0n]
Pseudochazara euxina|EULEP452-14|KN00814||Ukraine|658[0n]
Pseudochazara euxina|EULEP451-14|KN00813||Ukraine|658[0n]
Pseudochazara williamsi|EZSPC1443-10|RVcoll.09-V155||Spain|606[0n]
Pseudochazara williamsi|EZSPC1444-10|RVcoll.09-V156||Spain|658[0n]
Pseudochazara williamsi|EZSPC1431-10|RVcoll.09-V142||Spain|658[5n]
Pseudochazara williamsi|EZSPM470-09|Rvcoll.09-V432||Spain|658[0n]
Pseudochazara williamsi|EZSPN733-09|RVcoll.08-L141||Spain|658[0n]
Pseudochazara williamsi|EZSPN732-09|RVcoll.08-L140||Spain|658[0n]
Pseudochazara williamsi|EZSPN736-09|RVcoll.08-L145||Spain|658[0n]
Pseudochazara williamsi|EZSPN735-09|RVcoll.08-L144||Spain|650[0n]
Pseudochazara tisiphone|EULEP1626-15|RVcoll.14-G725||Greece|658[0n]
Pseudochazara tisiphone|EULEP1929-15|RVcoll.14-B892||Albania|658[0n]
Pseudochazara tisiphone|EULEP1906-15|RVcoll.14-B801||Albania|658[0n]
Pseudochazara tisiphone|EULEP1897-15|RVcoll.14-B728||Albania|658[0n]
Pseudochazara tisiphone|EULEP1619-15|RVcoll.14-G696||Greece|658[0n]
Pseudochazara tisiphone|EULEP1297-15|RVcoll.14-F729||Greece|658[0n]
Pseudochazara cingovskii|EULEP1556-15|RVcoll.14-G549||Macedonia|658[0n]
Pseudochazara cingovskii|EULEP1566-15|RVcoll.14-G569||Macedonia|658[0n]
Pseudochazara cingovskii|EULEP1565-15|RVcoll.14-G568||Macedonia|614[0n]
Pseudochazara cingovskii|EULEP1564-15|RVcoll.14-G566||Macedonia|614[0n]
Pseudochazara cingovskii|EULEP1554-15|RVcoll.14-G545||Macedonia|618[0n]
Pseudochazara orestes|EULEP2184-15|RVcoll.14-N223||Greece|658[0n]
Pseudochazara orestes|EULEP2183-15|RVcoll.14-N222||Greece|658[0n]
Pseudochazara orestes|EULEP2182-15|RVcoll.14-N221||Greece|658[0n]
Pseudochazara orestes|EULEP893-15|RVcoll.14-C671||Greece|658[0n]
Pseudochazara orestes|EULEP892-15|RVcoll.14-C648||Greece|658[0n]
Pseudochazara orestes|EULEP1260-15|RVcoll.14-F649||Greece|658[0n]
Pseudochazara tisiphone|EULEP5838-18|RVcoll14U547||Albania|658[0n]
Pseudochazara tisiphone|EULEP5837-18|RVcoll14U546||Albania|658[0n]
Pseudochazara tisiphone|EULEP5836-18|RVcoll14U545||Albania|658[0n]
Pseudochazara cingovskii|EULEP1567-15|RVcoll.14-G570||Macedonia|658[0n]
Pseudochazara cingovskii|HBOK048-08|OK519-48||Macedonia|632[0n]
Erebia epistygne|EZSPM812-12|Rvcoll. 130406FP1||Spain|658[0n]
Erebia epistygne|EZSPC1042-10|RVcoll.08-J342||Spain|658[0n]
Erebia epistygne|EZSPC1040-10|RVcoll.08-J340||Spain|658[0n]
Erebia epistygne|EZSPN612-09|RVcoll.08-J339||Spain|658[0n]
Erebia epistygne|EZSPN145-09|RVcoll.06-G515||Spain|658[0n]
Erebia epistygne|WMB947-13|RVcoll.10-A649||France|658[0n]
Erebia epistygne|EZSPM282-09|SMcoll.150308PP73||Spain|658[0n]
Erebia epistygne|EZSPM280-09|SMcoll.150308PP52||Spain|658[0n]
Erebia epistygne|EZSPC1155-10|RVcoll.08-R048||Spain|658[0n]
Erebia epistygne|EZSPC1154-10|RVcoll.08-R047||Spain|658[0n]
Erebia epistygne|EZSPM808-12|Rvcoll. 130211WX03||Spain|658[0n]
Erebia epistygne|HBOK134-08|OK520-40||Spain|632[0n]
Erebia discoidalis|EULEP2788-15|RVcoll.14-N872||Russia|658[0n]
Erebia discoidalis|EULEP2787-15|RVcoll.14-N871||Russia|658[0n]
Erebia discoidalis|EULEP2791-15|RVcoll.14-N875||Russia|658[0n]
Erebia discoidalis|EULEP2790-15|RVcoll.14-N874||Russia|658[0n]
Erebia discoidalis|EULEP448-14|KN00810||Russia|658[0n]
Erebia discoidalis|EULEP449-14|KN00811||Russia|658[0n]
Erebia discoidalis|EULEP420-14|KN00782||Russia|658[0n]
Erebia fasciata|EULEP3817-16|RVcoll14V776||Russia|658[0n]
Erebia fasciata|EULEP446-14|KN00808||Russia|658[0n]
Erebia fasciata|EULEP447-14|KN00809||Russia|658[0n]
Erebia fasciata|EULEP2796-15|RVcoll.14-N880||Russia|658[0n]
Erebia fasciata|EULEP3819-16|RVcoll14V778||Russia|631[0n]
Erebia fasciata|EULEP3818-16|RVcoll14V777||Russia|658[0n]
Erebia rossii|EULEP3962-16|RVcoll14V748||Russia|658[0n]
Erebia rossii|EULEP444-14|KN00806||Russia|658[0n]
Erebia rossii|EULEP3964-16|RVcoll14V750||Russia|600[0n]
Erebia rossii|EULEP3963-16|RVcoll14V749||Russia|606[0n]
Erebia rossii|EULEP3961-16|RVcoll14V747||Russia|647[0n]
Erebia cyclopius|EULEP445-14|KN00807||Russia|658[0n]
Erebia disa|EULEP464-14|KN00826||Russia|658[0n]
Erebia disa|EULEP4762-16|RVcoll16H061||Sweden|658[0n]
Erebia disa|EULEP548-15|RVcoll.08-L312||Sweden|658[0n]
Erebia disa|EULEP547-15|RVcoll.08-L311||Sweden|658[0n]
Erebia disa|LEFIJ555-10|MM17180||Finland|658[0n]
Erebia disa|LEFIJ554-10|MM17179||Finland|658[0n]
Erebia disa|LON165-08|NHMO-06166||Norway|657[0n]
Erebia embla|EULEP5172-17|RVcoll16H009||Sweden|658[0n]
Erebia embla|EULEP4760-16|RVcoll16H035||Sweden|658[0n]
Erebia embla|LEFID493-10|MM06450||Finland|658[0n]
Erebia embla|LEFID492-10|MM06449||Finland|658[0n]
Erebia embla|LEFIG142-10|MM13978||Finland|658[0n]
Erebia embla|LON649-09|NHMO Lep08303||Norway|658[0n]
Erebia albergana|LEASS676-17|TLMF Lep 22324||Austria|609[0n]
Erebia albergana|OXB733-15|13-U235||Italy|658[0n]
Erebia albergana|OXB732-15|13-U231||Italy|658[0n]

Erebia albergana|LEASS676-17|TLMF Lep 22324||Austria|609[0n]
Erebia albergana|OXB733-15|13-U235||Italy|658[0n]
Erebia albergana|OXB732-15|13-U231||Italy|658[0n]
Erebia albergana|LEASS975-17|TLMF Lep 22528||Austria|658[0n]
Erebia albergana|BIBSA383-15|RVcoll.14-I051||Italy|658[0n]
Erebia albergana|EULEP3765-16|RVcoll15J589||France|658[0n]
Erebia albergana|WMB1848-13|RVcoll.11-I924||France|658[0n]
Erebia albergana|EULEP822-15|RVcoll.14-A457||Italy|658[0n]
Erebia albergana|EULEP821-15|RVcoll.14-A456||Italy|658[0n]
Erebia albergana|WMB5278-14|RVcoll.14-A627||Italy|658[0n]
Erebia albergana|BIBSA1196-15|15-M282||Italy|613[0n]
Erebia albergana|EULEP3766-16|RVcoll15G005||Switzerland|648[0n]
Erebia albergana|PHLAI522-13|TLMF Lep 09084||Austria|615[0n]
Erebia albergana|EULEP578-15|RVcoll.10-A392||Bulgaria|658[0n]
Erebia albergana|EULEP1141-15|RVcoll.14-F300||Serbia|658[0n]
Erebia albergana|EULEP1095-15|RVcoll.14-F100||Serbia|658[0n]
Erebia albergana|EULEP3762-16|RVcoll10A900||Bulgaria|658[0n]
Erebia albergana|WMB5121-14|RVcoll. 14-L252||Italy|658[0n]
Erebia albergana|BIBSA390-15|RVcoll.14-I058||Italy|658[0n]
Erebia albergana|WMB1624-13|RVcoll.12-O785||France|658[0n]
Erebia albergana|LEATD289-13|TLMF Lep 12936||Italy|658[0n]
Erebia albergana|WMB5257-14|RVcoll.14-A266||Italy|658[0n]
Erebia albergana|WMB5103-14|RVcoll. 14-L234||Italy|658[0n]
Erebia albergana|EULEP3764-16|RVcoll15I376||Austria|658[0n]
Erebia albergana|EULEP3763-16|RVcoll15G921||Switzerland|658[0n]
Erebia albergana|PHLSA389-11|TLMF Lep 05844||Switzerland|658[0n]
Erebia albergana|LEATG080-14|TLMF Lep 13867||Austria|658[0n]
Erebia albergana|EULEP1876-15|RVcoll.14-A265||Italy|658[0n]
Erebia albergana|EULEP2437-15|RVcoll.14-V240||Switzerland|658[0n]
Erebia albergana|BIBSA154-15|RVcoll.14-I166||Italy|658[0n]
Erebia albergana|BIBSA155-15|RVcoll.14-I167||Italy|658[0n]
Erebia albergana|BIBSA202-15|RVcoll.14-D990||Italy|658[0n]
Erebia albergana|BIBSA203-15|RVcoll.14-D991||Italy|658[0n]
Erebia albergana|LEASS499-17|TLMF Lep 22147||Austria|658[0n]
Erebia albergana|OXB768-15|14-A228||Italy|658[0n]
Erebia albergana|OXB1564-16|OXB-TGS-1282||France|658[0n]
Erebia albergana|OXB1565-16|OXB-TGS-1283||France|658[0n]
Erebia albergana|WMB1623-13|RVcoll.12-O784||France|647[0n]
Erebia albergana|PHLSA403-11|TLMF Lep 05858||France|634[0n]
Erebia albergana|LEATD453-13|TLMF Lep 13100||Italy|658[0n]
Erebia albergana|PHLSA388-11|TLMF Lep 05843||Switzerland|658[0n]
Erebia flavofasciata|EULEP3820-16|RVcoll15G893||Switzerland|658[0n]
Erebia flavofasciata|EULEP3821-16|RVcoll15G895||Switzerland|658[0n]
Erebia flavofasciata|EULEP3822-16|RVcoll15H553||Switzerland|658[0n]
Erebia flavofasciata|EULEP3823-16|RVcoll15H568||Switzerland|658[0n]
Erebia flavofasciata|EULEP2033-15|RVcoll.14-J512||Italy|658[0n]
Erebia flavofasciata|OXB313-15|RVcoll.14-N027||Italy|658[0n]
Erebia flavofasciata|OXB314-15|RVcoll.14-N028||Italy|658[0n]
Erebia flavofasciata|EULEP3824-16|RVcoll15O035||Italy|637[0n]
Erebia flavofasciata|EULEP2034-15|RVcoll.14-J513||Italy|617[0n]
Erebia flavofasciata|HBOK106-08|OK520-12||Austria|609[0n]
Erebia oeme|EZRMN323-08|RVcoll.08-M631|Genit. examined|Romania|658[0n]
Erebia oeme|EZRMN322-08|RVcoll.08-M626|Genit. examined|Romania|658[0n]
Erebia oeme|EZRMN321-08|RVcoll.08-M624|Genit. examined|Romania|658[0n]
Erebia oeme|EZRMN403-09|RVcoll.06-V691|Genit. examined|Romania|627[0n]
Erebia oeme|EZRMN320-08|RVcoll.08-M623|Genit. examined|Romania|658[0n]
Erebia oeme|EULEP3905-16|RVcoll15H783||Switzerland|658[0n]
Erebia oeme|PHLAF656-11|TLMF Lep 05826||Austria|658[0n]
Erebia oeme|GWORK563-09|BC ZSM Lep 21893||Germany|658[0n]
Erebia oeme|PHLAF657-11|TLMF Lep 05827||Austria|658[0n]
Erebia oeme|EULEP520-15|RVcoll.08-H702||Slovenia|658[0n]
Erebia oeme|EULEP3906-16|RVcoll15H999||Italy|658[0n]
Erebia oeme|EULEP3866-16|RVcoll15H992||Italy|658[0n]
Erebia oeme|ODOPE686-11|BC ZSM Lep 50327||Germany|658[0n]
Erebia oeme|LEASS844-17|TLMF Lep 22492||Austria|658[0n]
Erebia oeme|LEATJ1302-16|TLMF Lep 19625||Austria|658[0n]
Erebia oeme|PHLSA696-11|TLMF Lep 06151||Austria|658[0n]
Erebia oeme|PHLSA384-11|TLMF Lep 05839||Austria|658[0n]
Erebia oeme|LEATJ1301-16|TLMF Lep 19624||Austria|658[0n]
Erebia oeme|ODOPE687-11|BC ZSM Lep 50328||Germany|658[0n]
Erebia oeme|GWORR686-10|BC ZSM Lep 32182||Germany|658[0n]
Erebia oeme|GWOSK901-11|BC ZSM Lep 49402||Germany|658[0n]
Erebia oeme|EZSPM003-09|RVcoll.08-M973|Genit. examined|France|658[0n]
Erebia oeme|EZSPM012-09|RVcoll.08-M992|Genit. examined|Andorra|658[0n]
Erebia oeme|EZSPC1371-10|RVcoll.09-X137||Spain|658[0n]
Erebia oeme|EZSPC994-10|RVcoll.07-W131||Spain|658[0n]
Erebia oeme|EZSPC071-09|RVcoll.07-W126|Genit. examined|Spain|658[0n]
Erebia oeme|EZSPC072-09|RVcoll.07-W127||Spain|658[0n]
Erebia oeme|EZSPC073-09|RVcoll.07-W128|Genit. examined|Spain|658[0n]
Erebia oeme|EZSPC074-09|RVcoll.07-W129||Spain|658[0n]
Erebia oeme|EZSPC075-09|RVcoll.07-W130||Spain|658[0n]
Erebia oeme|EULEP3904-16|RVcoll15H214||Switzerland|658[0n]
Erebia oeme|EULEP3907-16|RVcoll15I651||Austria|658[0n]
Erebia oeme|EULEP3908-16|RVcoll15G125||France|658[0n]
Erebia oeme|EULEP598-15|RVcoll.10-C331||Switzerland|658[0n]
Erebia oeme|ABOLD656-17|TLMF Lep 21732||Austria|658[0n]
Erebia oeme|EULEP3909-16|RVcoll15G196||France|658[0n]
Erebia oeme|EZSPC995-10|RVcoll.07-W132||Spain|637[0n]
Erebia oeme|EZSPC1361-10|RVcoll.09-X007||Spain|658[0n]
Erebia oeme|PHLAI564-13|TLMF Lep 09126||Austria|631[0n]
Erebia oeme|EULEP579-15|RVcoll.10-A393||Bulgaria|658[0n]
Erebia oeme|EULEP1152-15|RVcoll.14-F345||Serbia|658[0n]
Erebia oeme|EULEP1092-15|RVcoll.14-F061||Serbia|658[0n]
Erebia oeme|EULEP2829-15|RVcoll.14-N913||Bosnia and Herzegovina|658[0n]
Erebia oeme|EULEP1937-15|RVcoll.14-B909||Bosnia and Herzegovina|658[0n]
Erebia oeme|EULEP3903-16|RVcoll10B422||Bulgaria|658[0n]
Erebia oeme|EULEP3902-16|RVcoll10B413||Bulgaria|658[0n]
Erebia oeme|EULEP5666-17|RVcoll15Q043||Macedonia|658[0n]
Erebia oeme|EULEP3910-16|RVcoll15P079||Macedonia|614[1n]
Erebia oeme|PHLAF345-11|TLMF Lep 05515||Macedonia|658[0n]
Erebia oeme|PHLAF346-11|TLMF Lep 05516||Macedonia|658[0n]
Erebia claudina|EULEP5160-17|RVcoll15I424||Austria|658[0n]
Erebia claudina|EULEP5161-17|RVcoll15I853||Austria|658[0n]

[illegible]
Erebia oeme|PHLAF346-11|TLMF Lep 05516||Macedonia|658[0n]
Erebia claudina|EULEP5160-17|RVcoll15I424||Austria|658[0n]
Erebia claudina|EULEP5161-17|RVcoll15I853||Austria|658[0n]
Erebia claudina|EULEP3784-16|RVcoll15I408||Austria|631[0n]
Erebia claudina|LEATG664-14|TLMF Lep 14451||Austria|600[0n]
Erebia ligea|EULEP3847-16|RVcoll15I146||Austria|658[0n]
Erebia ligea|BIBSA1152-15|15-L089||Italy|658[0n]
Erebia ligea|EULEP1879-15|RVcoll.14-A541||Italy|658[0n]
Erebia ligea|EULEP1877-15|RVcoll.14-A295||Italy|658[0n]
Erebia ligea|WMB5260-14|RVcoll.14-A296||Italy|658[0n]
Erebia ligea|WMB4996-14|RVcoll. 14-I543||Italy|658[0n]
Erebia ligea|ABOLD033-16|TLMF Lep 21111||Austria|658[0n]
Erebia ligea|EULEP4976-16|RVcoll16I473||Poland|658[0n]
Erebia ligea|EULEP948-15|RVcoll.14-C953||Bulgaria|658[0n]
Erebia ligea|LEFID112-10|MM05735||Finland|658[0n]
Erebia ligea|EULEP2174-15|RVcoll.14-M528||Sweden|658[0n]
Erebia ligea|LEFIK299-10|MM17874||Finland|658[0n]
Erebia ligea|LEFIC041-10|MM03272||Finland|658[0n]
Erebia ligea|LEFIC042-10|MM03273||Finland|658[0n]
Erebia ligea|EZROM580-08|RV-07-C317|Genit. examined|Romania|658[0n]
Erebia ligea|EZROM1075-09|RVcoll.08-L379|Genit. examined|Romania|658[0n]
Erebia ligea|EZROM932-08|RVcoll.08-M593|Genit. examined|Romania|658[0n]
Erebia ligea|EULEP4771-16|RVcoll16H171||Norway|658[0n]
Erebia ligea|EULEP1074-15|RVcoll.14-E995||Serbia|658[0n]
Erebia ligea|EZROM579-08|RV-07-C316|Genit. examined|Romania|658[0n]
Erebia ligea|LON125-08|NHMO-06125||Norway|653[0n]
Erebia ligea|LEFIG532-10|MM14658||Finland|658[0n]
Erebia ligea|LEFIG531-10|MM14657||Finland|658[0n]
Erebia ligea|HBOK095-08|OK520-01||Austria|609[0n]
Erebia ligea|PHLAH745-12|TLMF Lep 08564||Austria|658[0n]
Erebia ligea|PHLAF655-11|TLMF Lep 05825||Austria|658[0n]
Erebia ligea|GWOTF670-12|BC ZSM Lep 62376||Germany|658[0n]
Erebia ligea|FBLMU438-09|BC ZSM Lep 27088||Germany|658[0n]
Erebia ligea|EULEP3848-16|RVcoll15I793||Austria|658[0n]
Erebia ligea|BIBSA966-15|13-U417||Italy|658[0n]
Erebia ligea|ODOPE244-11|BC ZSM Lep 53115||Germany|658[0n]
Erebia ligea|EULEP979-15|RVcoll.14-D121||Austria|613[0n]
Erebia ligea|LEFID111-10|MM05734||Finland|624[0n]
Erebia ligea|BIBSA1200-15|15-M296||Italy|612[0n]
Erebia ligea|LEATH758-14|TLMF Lep 15970||Italy|634[0n]
Erebia ligea|PHLAI543-13|TLMF Lep 09105||Austria|658[0n]
Erebia ligea|WMB1645-13|RVcoll.12-O954||France|658[0n]
Erebia ligea|WMB2686-13|RVcoll.10-B984||France|658[0n]
Erebia euryale|EULEP966-15|RVcoll.14-D038|Genit. examined|Bulgaria|658[0n]
Erebia ligea|EULEP532-15|RVcoll.08-H852||Serbia|658[0n]
Erebia euryale|EULEP3806-16|RVcoll10A919||Bulgaria|658[0n]
Erebia euryale|EULEP540-15|RVcoll.08-H898|Genit. examined|Serbia|658[0n]
Erebia euryale|EULEP949-15|RVcoll.14-C954||Bulgaria|658[0n]
Erebia euryale|EULEP898-15|RVcoll.14-C727||Bulgaria|658[0n]
Erebia euryale|EULEP854-15|RVcoll.14-C149||Greece|658[0n]
Erebia euryale|EULEP3816-16|RVcoll15Q014||Macedonia|658[0n]
Erebia euryale|EULEP3815-16|RVcoll15P088||Macedonia|658[0n]
Erebia euryale|EZROM1036-09|RVcoll.06-V690|Genit. examined|Romania|658[0n]
Erebia euryale|PHLAF343-11|TLMF Lep 05513||Macedonia|658[0n]
Erebia euryale|PHLAF344-11|TLMF Lep 05514||Macedonia|658[0n]
Erebia ligea|EZROM1074-09|RVcoll.08-L377|Genit. examined|Romania|658[0n]
Erebia ligea|EZROM935-08|RVcoll.08-M633|Genit. examined|Romania|658[0n]
Erebia ligea|EZROM934-08|RVcoll.08-M632|Genit. examined|Romania|658[0n]
Erebia ligea|EZROM929-08|RVcoll.08-M533|Genit. examined|Romania|658[0n]
Erebia ligea|EZROM930-08|RVcoll.08-M534|Genit. examined|Romania|658[0n]
Erebia ligea|EZROM928-08|RVcoll.08-M532|Genit. examined|Romania|658[0n]
Erebia ligea|EULEP3846-16|RVcoll15I076||Italy|658[0n]
Erebia ligea|EULEP626-15|RVcoll.11-J010||Switzerland|658[0n]
Erebia ligea|EULEP1938-15|RVcoll.14-B910||Bosnia and Herzegovina|658[0n]
Erebia euryale|EULEP2817-15|RVcoll.14-N901||Bosnia and Herzegovina|658[0n]
Erebia ligea|EULEP2011-15|RVcoll.14-I801||Slovakia|658[0n]
Erebia ligea|EULEP3850-16|RVcoll15P077||Macedonia|658[0n]
Erebia ligea|EZROM931-08|RVcoll.08-M535|Genit. examined|Romania|658[0n]
Erebia ligea|EZROM923-08|RVcoll.07-C352|Genit. examined|Romania|658[0n]
Erebia ligea|EULEP3851-16|RVcoll15Q016||Macedonia|658[0n]
Erebia ligea|EULEP3849-16|RVcoll15G165||France|658[0n]
Erebia ligea|WMB1824-13|RVcoll.11-I671|Genit. examined|France|658[0n]
Erebia ligea|EULEP3844-16|RVcoll15H246||Switzerland|658[0n]
Erebia ligea|EULEP2441-15|RVcoll.14-V245||Switzerland|658[0n]
Erebia ligea|OXB1566-16|OXB-TGS-1284||France|658[0n]
Erebia euryale|OXB1282-15|Rvcoll_15-M142||France|612[0n]
Erebia ligea|WMB821-13|RVcoll.07-E185||Italy|634[0n]
Erebia ligea|WMB3668-14|RVcoll.11-I954||France|658[0n]
Erebia ligea|WMB3651-14|RVcoll.11-I673||France|658[0n]
Erebia euryale|LEFIL051-10|MM19051||Russia|658[0n]
Erebia euryale|BIBSA158-15|RVcoll.14-I170||Italy|658[0n]
Erebia ligea|BIBSA210-15|RVcoll.14-D998||Italy|658[0n]
Erebia euryale|BIBSA937-15|16-A028||Italy|658[0n]
Erebia euryale|WMB1640-13|RVcoll.12-O879||France|658[0n]
Erebia euryale|LEASS759-17|TLMF Lep 22407||Austria|658[0n]
Erebia euryale|WMB2400-13|RVcoll.09-X929||Italy|658[0n]
Erebia euryale|WMB2666-13|RVcoll.10-B807||France|658[0n]
Erebia euryale|GWOSK902-11|BC ZSM Lep 49403||Germany|658[0n]
Erebia euryale|LEATD288-13|TLMF Lep 12935||Italy|658[0n]
Erebia euryale|WMB5023-14|RVcoll. 14-I570||Italy|658[0n]
Erebia euryale|OXB1180-15|Rvcoll_16-A067||Italy|658[0n]
Erebia euryale|EZSPC1256-10|RVcoll.09-V880||Spain|658[0n]
Erebia euryale|EZSPC1255-10|RVcoll.09-V879||Spain|658[0n]
Erebia ligea|WMB1603-13|RVcoll.12-O659|Genit. examined|France|658[0n]
Erebia euryale|EZSPC389-09|RVcoll.08-P089||Andorra|658[0n]
Erebia euryale|EZSPM586-12|RVcoll.08-P944||Spain|658[0n]
Erebia euryale|EZSPM092-09|RVcoll.08-P419||Spain|658[0n]
Erebia euryale|EZSPM225-09|RVcoll.08-R417||Spain|658[0n]
Erebia euryale|EZSPM226-09|RVcoll.08-R418||Spain|658[0n]
Erebia euryale|EZSPC1362-10|RVcoll.09-X008||Spain|658[0n]
Erebia euryale|EZSPC1250-10|RVcoll.09-V838||Spain|658[0n]
Erebia euryale|EULEP5888-18|RVcoll15O592||France|658[0n]
[illegible]

Erebia euryale|EZSPC1362-10|RVcoll.09-X008||Spain|658[0n]
Erebia euryale|EZSPC1250-10|RVcoll.09-V838||Spain|658[0n]
Erebia euryale|EULEP5888-18|RVcoll15O592||France|658[0n]
Erebia euryale|EULEP5889-18|RVcoll15O593||France|658[0n]
Erebia euryale|BIBSA207-15|RVcoll.14-D995||Italy|658[0n]
Erebia euryale|BIBSA209-15|RVcoll.14-D997||Italy|658[0n]
Erebia euryale|BIBSA211-15|RVcoll.14-D999||Italy|658[0n]
Erebia euryale|BIBSA214-15|RVcoll.14-E002||Italy|640[0n]
Erebia euryale|EZSPM097-09|RVcoll.08-P424||Spain|632[0n]
Erebia euryale|EZSPM295-09|RVcoll.08-J836||Spain|658[0n]
Erebia euryale|EZSPM585-12|RVcoll.08-P943||Spain|658[0n]
Erebia euryale|EZSPC388-09|RVcoll.08-P393||Spain|658[0n]
Erebia euryale|EZSPC387-09|RVcoll.08-L479||Spain|658[0n]
Erebia euryale|EZSPC386-09|RVcoll.08-L478||Spain|658[0n]
Erebia euryale|EZSPM417-09|Rvcoll.09-V837||Spain|658[0n]
Erebia euryale|EZSPC385-09|RVcoll.08-L477||Spain|658[0n]
Erebia euryale|EULEP1863-15|RVcoll.13-U270||Italy|658[0n]
Erebia euryale|HBOK097-08|OK520-03||Austria|658[0n]
Erebia euryale|EULEP3810-16|RVcoll15H483||Switzerland|658[0n]
Erebia ligea|WMB5269-14|RVcoll.14-A552||Italy|658[0n]
Erebia euryale|PHLAB369-10|TLMF Lep 01169||Switzerland|658[0n]
Erebia euryale|LEATD141-13|TLMF Lep 12788||Italy|658[0n]
Erebia euryale|BIBSA1464-16|RVcoll.14-I218||Italy|658[0n]
Erebia euryale|WMB914-13|RVcoll.09-X911||Italy|658[0n]
Erebia euryale|PHLSA391-11|TLMF Lep 05846||Italy|658[0n]
Erebia euryale|LEFIL052-10|MM19052||Russia|658[0n]
Erebia euryale|GWORR687-10|BC ZSM Lep 32183||Germany|658[0n]
Erebia euryale|FBLMU380-09|BC ZSM Lep 27030||Germany|658[0n]
Erebia euryale|EZROM1062-09|RVcoll.07-E599|Genit. examined|Romania|658[0n]
Erebia euryale|EZROM578-08|RV-07-C318|Genit. examined|Romania|658[0n]
Erebia euryale|EZROM920-08|RVcoll.07-C322|Genit. examined|Romania|658[0n]
Erebia euryale|EZROM924-08|RVcoll.07-D670|Genit. examined|Romania|658[0n]
Erebia euryale|EZROM925-08|RVcoll.07-E482|Genit. examined|Romania|658[0n]
Erebia euryale|EZROM926-08|RVcoll.07-E490||Romania|658[0n]
Erebia euryale|EZROM927-08|RVcoll.08-M495|Genit. examined|Romania|658[0n]
Erebia euryale|EZROM933-08|RVcoll.08-M613|Genit. examined|Romania|658[0n]
Erebia euryale|EZROM919-08|RVcoll.06-N021|Genit. examined|Romania|658[0n]
Erebia euryale|EZROM921-08|RVcoll.07-C325|Genit. examined|Romania|658[0n]
Erebia euryale|EZROM922-08|RVcoll.07-C340|Genit. examined|Romania|658[0n]
Erebia euryale|EZROM1053-09|RVcoll.07-D630|Genit. examined|Romania|658[0n]
Erebia euryale|EZROM153-08|RV-07-E487|Genit. examined|Romania|658[0n]
Erebia euryale|EZROM154-08|RV-07-E581|Genit. examined|Romania|658[0n]
Erebia euryale|EZROM155-08|RV-07-C149|Genit. examined|Romania|658[0n]
Erebia euryale|EZROM1040-09|RVcoll.07-C319|Genit. examined|Romania|658[0n]
Erebia euryale|WMB838-13|RVcoll.07-E234||Italy|658[0n]
Erebia euryale|WMB919-13|RVcoll.09-X928||Italy|658[0n]
Erebia euryale|WMB2399-13|RVcoll.09-X910||Italy|658[0n]
Erebia euryale|WMB5026-14|RVcoll. 14-I573||Italy|658[0n]
Erebia euryale|WMB5029-14|RVcoll. 14-I576||Italy|658[0n]
Erebia ligea|EULEP3845-16|RVcoll15H794||Italy|658[0n]
Erebia euryale|EULEP3803-16|RVcoll15I187||Austria|658[0n]
Erebia euryale|EULEP3808-16|RVcoll14V773||Russia|658[0n]
Erebia euryale|EULEP3809-16|RVcoll15H220||Switzerland|658[0n]
Erebia euryale|EULEP3811-16|RVcoll15H911||Italy|658[0n]
Erebia euryale|EULEP3812-16|RVcoll15I278||Austria|658[0n]
Erebia euryale|EULEP3813-16|RVcoll15I380||Austria|658[0n]
Erebia euryale|EULEP3814-16|RVcoll15I622||Austria|658[0n]
Erebia euryale|EULEP993-15|RVcoll.14-D287||Austria|658[0n]
Erebia euryale|EULEP2010-15|RVcoll.14-I799||Poland|658[0n]
Erebia euryale|BIBSA1462-16|RVcoll.14-I216||Italy|658[0n]
Erebia euryale|BIBSA1075-15|15-K523||Italy|658[0n]
Erebia euryale|BIBSA1105-15|15-L799||Italy|658[0n]
Erebia euryale|BIBSA388-15|RVcoll.14-I056||Italy|658[0n]
Erebia euryale|BIBSA159-15|RVcoll.14-I171||Italy|658[0n]
Erebia euryale|BIBSA208-15|RVcoll.14-D996||Italy|658[0n]
Erebia euryale|LEASS563-17|TLMF Lep 22211||Austria|658[0n]
Erebia euryale|LEASS768-17|TLMF Lep 22416||Austria|658[0n]
Erebia euryale|OXB832-15|14-U795||Italy|658[0n]
Erebia euryale|OXB632-15|14-O021||Italy|658[0n]
Erebia euryale|OXB1615-16|OXB-TGS-1333||France|658[0n]
Erebia euryale|BIBSA1144-15|15-L043||Italy|658[1n]
Erebia euryale|WMB5108-14|RVcoll. 14-L239||Italy|658[0n]
Erebia euryale|EULEP5009-16|RVcoll16I877||Poland|658[0n]
Erebia euryale|EULEP5071-16|RVcoll16J192||Czech Republic|658[0n]
Erebia euryale|EULEP602-15|RVcoll.10-C345||Switzerland|658[0n]
Erebia euryale|LEATJ1257-16|TLMF Lep 19580||Austria|658[0n]
Erebia euryale|EZROM152-08|RV-06-V671|Genit. examined|Romania|650[0n]
Erebia euryale|EULEP3807-16|RVcoll14V772||Russia|631[0n]
Erebia euryale|LEASS504-17|TLMF Lep 22152||Austria|638[0n]
Erebia euryale|LEASS515-17|TLMF Lep 22163||Austria|612[0n]
Erebia euryale|GWORR683-10|BC ZSM Lep 32179||Germany|639[0n]
Erebia euryale|PHLAB370-10|TLMF Lep 01170||Switzerland|637[0n]
Erebia ligea|LEATG504-14|TLMF Lep 14291||Italy|630[0n]
Erebia ligea|LEATG505-14|TLMF Lep 14292||Italy|607[0n]
Erebia euryale|PHLAF653-11|TLMF Lep 05823||Austria|658[0n]
Erebia euryale|PHLAF654-11|TLMF Lep 05824||Austria|658[0n]
Erebia euryale|PHLSA386-11|TLMF Lep 05841||Austria|658[0n]
Erebia euryale|LEATJ1313-16|TLMF Lep 19636||Austria|658[0n]
Erebia eriphyle|EULEP3801-16|RVcoll15H784||Switzerland|658[0n]
Erebia eriphyle|LEASS517-17|TLMF Lep 22165||Austria|658[0n]
Erebia eriphyle|GWOSA779-10|BC ZSM Lep 36645||Germany|658[0n]
Erebia eriphyle|EULEP3800-16|RVcoll15H780||Switzerland|658[0n]
Erebia eriphyle|EULEP3802-16|RVcoll15I186||Austria|658[0n]
Erebia eriphyle|EULEP2795-15|RVcoll.14-N879||Italy|614[0n]
Erebia eriphyle|EULEP2794-15|RVcoll.14-N878||Austria|658[0n]
Erebia eriphyle|EULEP3805-16|RVcoll15I792||Austria|642[0n]
Erebia eriphyle|ODOPE667-11|BC ZSM Lep 50308||Germany|623[0n]
Erebia eriphyle|ODOPE666-11|BC ZSM Lep 50307||Germany|627[0n]
Erebia eriphyle|PHLAH289-12|TLMF Lep 07728||Austria|658[0n]
Erebia eriphyle|LEATJ1309-16|TLMF Lep 19632||Austria|658[0n]
Erebia eriphyle|LEATA408-13|TLMF Lep 10015||Austria|658[0n]
Erebia eriphyle|LEATJ1103-15|TLMF Lep 18963||Austria|658[0n]
Erebia eriphyle|PHLSA411-11|TLMF Lep 05866||Austria|658[0n]

Erebia eriphyle|LEATA408-13|TLMF Lep 10015||Austria|658[0n]
Erebia eriphyle|LEATJ1103-15|TLMF Lep 18963||Austria|658[0n]
Erebia eriphyle|PHLSA411-11|TLMF Lep 05866||Austria|658[0n]
Erebia montana|BIBSA1147-15|15-L052||Italy|658[0n]
Erebia montana|EULEP749-15|RVcoll.12-O778||France|658[0n]
Erebia montana|EULEP5475-17|RVcoll12O777||France|658[0n]
Erebia montana|EULEP5474-17|RVcoll12O776||France|658[0n]
Erebia montana|EULEP5473-17|RVcoll12O775||France|658[0n]
Erebia montana|OXB1185-15|Rvcoll_16-A072||Italy|658[0n]
Erebia montana|OXB786-15|LD-0430||Italy|658[0n]
Erebia montana|EULEP1883-15|RVcoll.14-A620||Italy|658[0n]
Erebia montana|EULEP3893-16|RVcoll14V136||Italy|658[0n]
Erebia montana|WMB5277-14|RVcoll.14-A621||Italy|658[0n]
Erebia montana|EULEP3892-16|RVcoll10C040||France|658[0n]
Erebia montana|EULEP3897-16|RVcoll15J329||France|658[0n]
Erebia montana|EULEP5393-17|RVcoll10C041||France|658[1n]
Erebia montana|BIBSA299-15|RVcoll.14-E101||Italy|639[0n]
Erebia montana|BIBSA897-15|LD-3509||Italy|658[0n]
Erebia montana|PHLAH842-12|TLMF Lep 08646||Italy|658[0n]
Erebia montana|EULEP5454-17|RVcoll11J434||Switzerland|658[0n]
Erebia montana|EULEP3894-16|RVcoll15G983||Switzerland|658[0n]
Erebia montana|PHLSA393-11|TLMF Lep 05848||Switzerland|658[0n]
Erebia montana|LEATG353-14|TLMF Lep 14140||Austria|658[0n]
Erebia montana|LEATG507-14|TLMF Lep 14294||Italy|658[0n]
Erebia montana|LEATC617-13|TLMF Lep 12599||Austria|658[0n]
Erebia montana|EULEP3896-16|RVcoll15J214||Switzerland|658[0n]
Erebia montana|EULEP3895-16|RVcoll15I939||Switzerland|634[0n]
Erebia montana|PHLAI538-13|TLMF Lep 09100||Austria|608[0n]
Erebia montana|LEATF473-14|TLMF Lep 13785||Switzerland|658[0n]
Erebia montana|LEATG506-14|TLMF Lep 14293||Italy|658[0n]
Erebia styx|EULEP3981-16|RVcoll15O040||Italy|658[0n]
Erebia styx|EULEP3980-16|RVcoll15O039||Italy|658[0n]
Erebia styx|EULEP3975-16|RVcoll15I539||Italy|658[0n]
Erebia styx|EULEP3976-16|RVcoll15I576||Italy|658[0n]
Erebia styx|EULEP3984-16|RVcoll15P052||Slovenia|658[0n]
Erebia styx|EULEP3974-16|RVcoll15I538||Italy|637[0n]
Erebia styx|EULEP3971-16|RVcoll15I567||Italy|658[0n]
Erebia styx|EULEP3954-16|RVcoll15I559||Italy|658[0n]
Erebia styx|ODOPE685-11|BC ZSM Lep 50326||Germany|658[0n]
Erebia stiria|WMB884-13|RVcoll.09-X598||Italy|658[0n]
Erebia stiria|EULEP3969-16|RVcoll15H986||Italy|658[0n]
Erebia stiria|EULEP3970-16|RVcoll15I548||Italy|658[0n]
Erebia stiria|EULEP3972-16|RVcoll15P048||Slovenia|658[0n]
Erebia stiria|EULEP3973-16|RVcoll15P051||Slovenia|658[0n]
Erebia styx|EULEP3978-16|RVcoll15J032||Switzerland|658[0n]
Erebia styx|EULEP3979-16|RVcoll15J049||Switzerland|658[0n]
Erebia styx|EULEP5272-17|RVcoll08H700||Italy|658[0n]
Erebia styx|EULEP5273-17|RVcoll08H701||Italy|658[0n]
Erebia styx|EULEP5274-17|RVcoll08H709||Slovenia|658[0n]
Erebia stiria|EULEP5275-17|RVcoll08H710||Slovenia|658[0n]
Erebia stiria|EULEP521-15|RVcoll.08-H707||Slovenia|658[0n]
Erebia stiria|EULEP2111-15|RVcoll.14-J603||Slovenia|658[0n]
Erebia stiria|EULEP522-15|RVcoll.08-H708||Slovenia|658[0n]
Erebia stiria|EULEP1843-15|RVcoll.09-X198||Italy|658[0n]
Erebia styx|LEASS558-17|TLMF Lep 22206||Austria|658[1n]
Erebia styx|EULEP3982-16|RVcoll15O041||Italy|658[0n]
Erebia styx|EULEP3983-16|RVcoll15O042||Italy|658[0n]
Erebia stiria|EULEP523-15|RVcoll.08-H711||Slovenia|613[0n]
Erebia styx|LEATH723-14|TLMF Lep 15935||Italy|634[0n]
Erebia styx|LEATH722-14|TLMF Lep 15934||Italy|634[0n]
Erebia styx|GWOSK904-11|BC ZSM Lep 49405||Germany|658[0n]
Erebia styx|GWOSN575-11|BC ZSM Lep 52686||Germany|658[0n]
Erebia styx|ODOPE684-11|BC ZSM Lep 50325||Germany|658[0n]
Erebia styx|LEATG109-14|TLMF Lep 13896||Austria|658[0n]
Erebia styx|LEATG082-14|TLMF Lep 13869||Austria|658[0n]
Erebia stiria|LEATG428-14|TLMF Lep 14215||Italy|658[0n]
Erebia stiria|LEATG429-14|TLMF Lep 14216||Italy|658[0n]
Erebia scipio|EULEP799-15|RVcoll.13-S255||Italy|658[0n]
Erebia scipio|EULEP3967-16|RVcoll15J464||France|658[0n]
Erebia scipio|EULEP3966-16|RVcoll15J449||France|658[0n]
Erebia scipio|BIBSA895-15|LD-3507||France|658[0n]
Erebia scipio|EULEP5509-17|RVcoll13S254||Italy|658[0n]
Erebia scipio|EULEP2051-15|RVcoll.14-J535||France|658[0n]
Erebia scipio|EULEP2052-15|RVcoll.14-J536||France|658[0n]
Erebia scipio|EULEP3968-16|RVcoll14A994||Italy|649[0n]
Erebia scipio|EULEP3965-16|RVcoll10B935||France|658[0n]
Erebia melampus|EULEP3876-16|RVcoll15I312||Austria|658[0n]
Erebia melampus|EULEP3877-16|RVcoll15I375||Austria|658[0n]
Erebia melampus|EULEP3878-16|RVcoll15I766||Austria|658[0n]
Erebia melampus|LEASS503-17|TLMF Lep 22151||Austria|658[0n]
Erebia melampus|LEASS506-17|TLMF Lep 22154||Austria|658[0n]
Erebia melampus|BIBSA1188-15|15-M248||Italy|658[0n]
Erebia melampus|PHLAI518-13|TLMF Lep 09080||Austria|627[0n]
Erebia melampus|BIBSA1104-15|15-L777||Italy|615[0n]
Erebia melampus|EULEP1870-15|RVcoll.13-U367||Italy|658[0n]
Erebia melampus|BIBSA389-15|RVcoll.14-I057||Italy|658[0n]
Erebia melampus|OXB827-15|14-U790||Italy|658[0n]
Erebia melampus|EULEP3872-16|RVcoll15G982||Switzerland|658[0n]
Erebia melampus|EULEP3873-16|RVcoll15H445||Switzerland|658[0n]
Erebia melampus|EULEP3874-16|RVcoll15H692||Switzerland|658[0n]
Erebia melampus|EULEP3879-16|RVcoll15J260||Switzerland|658[0n]
Erebia melampus|EULEP3880-16|RVcoll15J269||Switzerland|658[0n]
Erebia melampus|EULEP3881-16|RVcoll15J379||France|658[0n]
Erebia melampus|EULEP608-15|RVcoll.11-E231||Switzerland|658[0n]
Erebia melampus|EULEP610-15|RVcoll.11-E233|Genit. examined|Switzerland|658[0n]
Erebia melampus|EULEP2450-15|RVcoll.14-V270|Genit. examined|Switzerland|658[0n]
Erebia melampus|BIBSA1466-16|RVcoll.14-I220||Italy|658[0n]
Erebia melampus|BIBSA1468-16|RVcoll.14-I222||Italy|658[0n]
Erebia melampus|BIBSA386-15|RVcoll.14-I054||Italy|658[0n]
Erebia melampus|EULEP2443-15|RVcoll.14-V249|Genit. examined|Switzerland|658[0n]
Erebia melampus|BIBSA393-15|RVcoll.14-I061||Italy|658[0n]
Erebia melampus|BIBSA213-15|RVcoll.14-E001||Italy|637[0n]
Erebia melampus|ODOPE674-11|BC ZSM Lep 50315||Germany|658[1n]

Erebia melampus|[illegible]|Genit. examined|Switzerland|658[0n]
Erebia melampus|BIBSA393-15|RVcoll.14-I061||Italy|658[0n]
Erebia melampus|BIBSA213-15|RVcoll.14-E001||Italy|637[0n]
Erebia melampus|ODOPE674-11|BC ZSM Lep 50315||Germany|658[1n]
Erebia melampus|PHLAB321-10|TLMF Lep 01121||Switzerland|658[0n]
Erebia melampus|PHLAF633-11|TLMF Lep 05803||Switzerland|658[0n]
Erebia sudetica|EULEP3988-16|RVcoll15J590||France|658[0n]
Erebia sudetica|EULEP3986-16|RVcoll15J569||France|658[0n]
Erebia sudetica|LEATJ1205-16|TLMF Lep 19528||Czech Republic|649[0n]
Erebia sudetica|EULEP5453-17|RVcoll11J422||Switzerland|658[0n]
Erebia sudetica|EULEP5452-17|RVcoll11J421||Switzerland|658[0n]
Erebia sudetica|EULEP5451-17|RVcoll11J420||Switzerland|658[0n]
Erebia sudetica|EULEP3987-16|RVcoll15J583||France|658[0n]
Erebia sudetica|EULEP3985-16|RVcoll15H198||Switzerland|658[0n]
Erebia sudetica|EZROM970-08|RVcoll.08-M608||Romania|658[0n]
Erebia sudetica|EZROM971-08|RVcoll.08-M617||Romania|658[0n]
Erebia sudetica|EZROM173-08|RV-07-E474||Romania|658[0n]
Erebia sudetica|EZROM172-08|RV-07-E473||Romania|658[0n]
Erebia sudetica|EZROM972-08|RVcoll.08-M618||Romania|658[0n]
Erebia sudetica|EZROM175-08|RV-07-C147||Romania|658[0n]
Erebia sudetica|EZROM174-08|RV-07-C146||Romania|658[0n]
Erebia sudetica|EZRMN313-08|RVcoll.08-M797||Romania|658[0n]
Erebia sudetica|EZRMN305-08|RVcoll.08-M789|Genit. examined|Romania|658[0n]
Erebia sudetica|EZRMN326-08|RVcoll.08-H026||Romania|658[0n]
Erebia sudetica|EZRMN325-08|RVcoll.08-H023||Romania|658[0n]
Erebia melampus|BIBSA212-15|RVcoll.14-E000||Italy|658[0n]
Erebia melampus|OXB1151-15|Rvcoll_15-N109||Italy|658[0n]
Erebia melampus|OXB629-15|14-O018||Italy|658[0n]
Erebia melampus|EULEP3875-16|RVcoll15H827||Italy|658[0n]
Erebia melampus|LEATD144-13|TLMF Lep 12791||Italy|658[0n]
Erebia melampus|PHLSA385-11|TLMF Lep 05840||Austria|658[0n]
Erebia melampus|LEATD142-13|TLMF Lep 12789||Italy|658[0n]
Erebia melampus|LEATD291-13|TLMF Lep 12938||Italy|658[0n]
Erebia melampus|PHLSA663-11|TLMF Lep 06118||Austria|658[0n]
Erebia lefebvrei|EZSPC1172-10|RVcoll.09-T120||Spain|658[0n]
Erebia lefebvrei|EZSPC1171-10|RVcoll.09-T119||Spain|658[0n]
Erebia lefebvrei|WMB4532-14|RVcoll.14-E247||Spain|658[0n]
Erebia lefebvrei|WMB4531-14|RVcoll.14-E245||Spain|658[0n]
Erebia lefebvrei|WMB4534-14|RVcoll.14-E249||Spain|658[0n]
Erebia lefebvrei|WMB4533-14|RVcoll.14-E248||Spain|658[0n]
Erebia lefebvrei|EZSPM841-12|Rvcoll. 140711ZG84||Spain|658[0n]
Erebia lefebvrei|EZSPM837-12|Rvcoll. 140711ZG41||Spain|658[0n]
Erebia lefebvrei|EZSPM830-12|Rvcoll. 140711ZG05||Spain|658[0n]
Erebia lefebvrei|EZSPC1165-10|RVcoll.09-T057||Spain|658[0n]
Erebia lefebvrei|WMB4529-14|RVcoll.14-E240||Spain|658[0n]
Erebia lefebvrei|WMB4530-14|RVcoll.14-E244||Spain|658[0n]
Erebia lefebvrei|EULEP2187-15|RVcoll.14-N229||Spain|658[0n]
Erebia lefebvrei|EZSPC1156-10|RVcoll.08-R225||Spain|637[0n]
Erebia lefebvrei|EZSPM193-09|RVcoll.08-R223||Spain|658[0n]
Erebia lefebvrei|EZSPM187-09|RVcoll.08-R200||Spain|658[0n]
Erebia lefebvrei|EZSPM022-09|RVcoll.08-P087||Andorra|658[0n]
Erebia lefebvrei|EZSPM194-09|RVcoll.08-R224||Spain|658[0n]
Erebia lefebvrei|EZSPM186-09|RVcoll.08-R182||Spain|658[0n]
Erebia lefebvrei|EZSPM184-09|RVcoll.08-R173||Spain|658[0n]
Erebia lefebvrei|EZSPM183-09|RVcoll.08-R172||Spain|658[0n]
Erebia pronoe|EZSPC949-10|RVcoll.09-X305||Spain|658[0n]
Erebia pronoe|EZSPC950-10|RVcoll.09-X306||Spain|658[0n]
Erebia pronoe|EZSPC951-10|RVcoll.09-X307|Genit. examined|Spain|658[0n]
Erebia pronoe|EZSPC1041-10|RVcoll.08-M805||Spain|658[0n]
Erebia pronoe|EZSPC1051-10|RVcoll.08-M806||Spain|658[0n]
Erebia pronoe|EZSPC1055-10|RVcoll.08-M807||Spain|658[0n]
Erebia pronoe|EZSPC1057-10|RVcoll.08-M808||Spain|658[0n]
Erebia pronoe|EZSPC1064-10|RVcoll.08-M809||Spain|658[0n]
Erebia pronoe|WMB3632-14|RVcoll.11-G149||Spain|658[0n]
Erebia pronoe|EULEP5639-17|RVcoll15D791||Spain|658[0n]
Erebia pronoe|EULEP5640-17|RVcoll15D792||Spain|658[0n]
Erebia pronoe|EZSPC947-10|RVcoll.09-X303|Genit. examined|Spain|629[0n]
Erebia pronoe|EZSPC946-10|RVcoll.09-X302||Spain|658[0n]
Erebia pronoe|EZSPC871-10|RVcoll.09-T122||Spain|658[0n]
Erebia pronoe|EZSPC870-10|RVcoll.09-T121||Spain|658[0n]
Erebia pronoe|EZSPM583-12|RVcoll.08-P941|Genit. examined|Spain|658[0n]
Erebia pronoe|EZSPM584-12|RVcoll.08-P942||Spain|658[0n]
Erebia melas|EZROM948-08|RVcoll.06-M970||Romania|658[0n]
Erebia melas|EZROM949-08|RVcoll.06-M992||Romania|658[0n]
Erebia melas|EZROM951-08|RVcoll.06-N025|Genit. examined|Romania|658[0n]
Erebia melas|EZROM164-08|RV-06-M981||Romania|653[0n]
Erebia melas|EZROM564-08|RV-06-M994|Genit. examined|Romania|651[0n]
Erebia melas|EZROM950-08|RVcoll.06-M998|Genit. examined|Romania|658[0n]
Erebia melas|EZROM167-08|RV-07-E492||Romania|658[0n]
Erebia melas|EULEP3884-16|RVcoll15Q019||Macedonia|658[0n]
Erebia melas|EULEP913-15|RVcoll.14-C772||Bulgaria|658[0n]
Erebia melas|EULEP857-15|RVcoll.14-C173||Greece|658[0n]
Erebia melas|EULEP997-15|RVcoll.14-E160||Romania|658[0n]
Erebia melas|EULEP5295-17|RVcoll08L398||Slovenia|658[0n]
Erebia melas|EZROM953-08|RVcoll.07-E314||Romania|658[0n]
Erebia melas|EZROM952-08|RVcoll.07-E311||Romania|658[0n]
Erebia melas|EZROM165-08|RV-07-E309||Romania|658[0n]
Erebia melas|EZROM1055-09|RVcoll.07-E315||Romania|658[0n]
Erebia melas|EZROM954-08|RVcoll.07-E319||Romania|658[0n]
Erebia melas|EZRMN366-08|RVcoll.08-M733||Romania|658[0n]
Erebia melas|EZRMN367-08|RVcoll.08-M734||Romania|658[0n]
Erebia melas|EZROM955-08|RVcoll.07-E388||Romania|658[0n]
Erebia melas|EZROM956-08|RVcoll.07-E390||Romania|658[0n]
Erebia melas|EZROM166-08|RV-07-E387||Romania|658[0n]
Erebia melas|EZROM1057-09|RVcoll.07-E389||Romania|658[0n]
Erebia melas|EULEP3883-16|RVcoll15Q018||Macedonia|658[0n]
Erebia melas|EULEP553-15|RVcoll.08-L393||Montenegro|658[0n]
Erebia melas|EULEP2824-15|RVcoll.14-N908||Albania|658[0n]
Erebia melas|EULEP2825-15|RVcoll.14-N909||Albania|658[0n]
Erebia melas|EULEP3882-16|RVcoll15P095||Macedonia|624[0n]
Erebia melas|EZRMN351-08|RVcoll.07-D698||Romania|608[0n]
Erebia melas|PHLAF341-11|TLMF Lep 05511||Macedonia|658[0n]
Erebia melas|PHLAF340-11|TLMF Lep 05510||Macedonia|658[0n]

Erebia melas|EZRMN351-08|RVcoll.07-D698||Romania|608[0n]
Erebia melas|PHLAF341-11|TLMF Lep 05511||Macedonia|658[0n]
Erebia melas|PHLAF340-11|TLMF Lep 05510||Macedonia|658[0n]
Erebia pronoe|EULEP5449-17|RVcoll11J168|Genit. examined|France|658[0n]
Erebia pronoe|EULEP5419-17|RVcoll11E238|Genit. examined|France|658[0n]
Erebia pronoe|EULEP3956-16|RVcoll15I990||Switzerland|658[0n]
Erebia pronoe|GWOTD442-12|BC ZSM Lep 63573||Germany|658[0n]
Erebia pronoe|ODOPE682-11|BC ZSM Lep 50323||Germany|658[0n]
Erebia pronoe|PHLAB350-10|TLMF Lep 01150||Switzerland|658[0n]
Erebia pronoe|PHLAF664-11|TLMF Lep 05834||Switzerland|658[0n]
Erebia pronoe|EULEP5418-17|RVcoll11E229||Switzerland|658[0n]
Erebia pronoe|EULEP5417-17|RVcoll11E228||Switzerland|658[0n]
Erebia pronoe|EULEP5416-17|RVcoll11E227|Genit. examined|Switzerland|658[0n]
Erebia pronoe|EULEP5415-17|RVcoll11E226||Switzerland|658[0n]
Erebia pronoe|EULEP5414-17|RVcoll11E225||Switzerland|658[0n]
Erebia pronoe|EULEP5413-17|RVcoll11E224||Switzerland|658[0n]
Erebia pronoe|EULEP5412-17|RVcoll11E223||Switzerland|658[0n]
Erebia pronoe|EULEP5411-17|RVcoll11E222||Switzerland|658[0n]
Erebia pronoe|EULEP5410-17|RVcoll11E221||Switzerland|658[0n]
Erebia pronoe|EULEP5409-17|RVcoll11E220|Genit. examined|Switzerland|658[0n]
Erebia pronoe|EZROM968-08|RVcoll.07-E572||Romania|658[0n]
Erebia pronoe|EZROM170-08|RV-07-E449||Romania|658[0n]
Erebia pronoe|EZROM969-08|RVcoll.07-E590||Romania|658[0n]
Erebia pronoe|EZROM967-08|RVcoll.07-E568||Romania|658[0n]
Erebia pronoe|EZROM966-08|RVcoll.07-E452||Romania|658[0n]
Erebia pronoe|EZROM965-08|RVcoll.07-E451||Romania|658[0n]
Erebia pronoe|EZROM171-08|RV-07-E550||Romania|658[0n]
Erebia pronoe|EZROM628-08|RV-07-E588||Romania|658[2n]
Erebia pronoe|EULEP3953-16|RVcoll15H996||Italy|658[0n]
Erebia pronoe|ABOLD026-16|TLMF Lep 21104||Austria|658[0n]
Erebia pronoe|GWORL320-09|BC ZSM Lep 22032||Germany|658[0n]
Erebia pronoe|GWOSK903-11|BC ZSM Lep 49404||Germany|658[0n]
Erebia pronoe|GWOSK905-11|BC ZSM Lep 49406||Germany|658[0n]
Erebia pronoe|GWOSK906-11|BC ZSM Lep 49407||Germany|658[0n]
Erebia pronoe|EULEP3955-16|RVcoll15I758||Austria|658[0n]
Erebia pronoe|EULEP5403-17|RVcoll11D545||Austria|658[0n]
Erebia pronoe|GWORA2485-09|BC ZSM Lep 30697|original identification E montana (RS) |Germany...
Erebia pronoe|GWORA2482-09|BC ZSM Lep 30694|changed to pronoe (AH,AS)|Germany|658[0n]
Erebia pronoe|GWORR689-10|BC ZSM Lep 32185||Germany|658[0n]
Erebia pronoe|GWORR682-10|BC ZSM Lep 32178||Germany|658[0n]
Erebia pronoe|ODOPE681-11|BC ZSM Lep 50322||Germany|658[0n]
Erebia pronoe|LEATC624-13|TLMF Lep 12606||Austria|658[0n]
Erebia pronoe|LEATC623-13|TLMF Lep 12605||Austria|658[0n]
Erebia pronoe|LEATG083-14|TLMF Lep 13870||Austria|658[0n]
Erebia pronoe|LEATG091-14|TLMF Lep 13878||Austria|658[0n]
Erebia pronoe|LEATG508-14|TLMF Lep 14295||Italy|658[0n]
Erebia pronoe|EULEP5239-17|RVcoll07D683||Bulgaria|658[0n]
Erebia pronoe|EULEP5238-17|RVcoll07D654||Bulgaria|658[0n]
Erebia pronoe|EULEP5237-17|RVcoll07D632||Bulgaria|658[0n]
Erebia pronoe|EULEP5236-17|RVcoll07D613||Bulgaria|658[0n]
Erebia pronoe|EULEP5235-17|RVcoll07D603|Genit. examined|Bulgaria|658[0n]
Erebia pronoe|EULEP5234-17|RVcoll07D602||Bulgaria|658[0n]
Erebia pronoe|EULEP3958-16|RVcoll15P050||Slovenia|658[0n]
Erebia pronoe|BIBSA1082-15|15-K564||Italy|658[0n]
Erebia pronoe|PHLAA140-09|TLMF Lep 00180||Italy|658[0n]
Erebia pronoe|EULEP3957-16|RVcoll15P047||Slovenia|658[0n]
Erebia pronoe|EULEP5501-17|RVcoll13S246||Italy|658[0n]
Erebia pronoe|EULEP5502-17|RVcoll13S247||Italy|658[0n]
Erebia pronoe|EULEP5503-17|RVcoll13S248||Italy|658[0n]
Erebia pronoe|EULEP5504-17|RVcoll13S249||Italy|658[0n]
Erebia pronoe|EULEP5505-17|RVcoll13S250||Italy|658[0n]
Erebia pronoe|EULEP5506-17|RVcoll13S251||Italy|658[0n]
Erebia pronoe|EULEP5508-17|RVcoll13S253||Italy|658[0n]
Erebia pronoe|EULEP1846-15|RVcoll.13-S245||Italy|658[0n]
Erebia pronoe|EULEP5507-17|RVcoll13S252||Italy|634[0n]
Erebia pronoe|LEASS556-17|TLMF Lep 22204||Austria|636[0n]
Erebia pronoe|HBOK150-08|OK520-56||Austria|609[0n]
Erebia pronoe|HBOK149-08|OK520-55||Austria|630[3n]
Erebia neoridas|EULEP1881-15|RVcoll.14-A562||Italy|658[0n]
Erebia neoridas|WMB5272-14|RVcoll.14-A588||Italy|658[0n]
Erebia neoridas|EZSPN182-09|RVcoll.06-V752||Spain|658[0n]
Erebia neoridas|EZSPC980-10|RVcoll.06-V775||Spain|658[0n]
Erebia neoridas|EZSPC981-10|RVcoll.06-V777||Spain|658[0n]
Erebia neoridas|EZSPN180-09|RVcoll.06-V745||Spain|658[0n]
Erebia zapateri|EULEP5321-17|RVcoll09T104||Spain|658[0n]
Erebia zapateri|EULEP5374-17|RVcoll10A279||Spain|658[0n]
Erebia zapateri|EULEP5300-17|RVcoll08P459||Spain|658[0n]
Erebia zapateri|EULEP5470-17|RVcoll12N723||Spain|658[0n]
Erebia zapateri|EULEP5469-17|RVcoll12N722||Spain|658[0n]
Erebia zapateri|EZSPM546-10|RVcoll.08-P458||Spain|658[0n]
Erebia zapateri|EZSPM543-10|RVcoll.08-P448.1||Spain|658[0n]
Erebia zapateri|EZSPM542-10|RVcoll.08-P447||Spain|658[0n]
Erebia zapateri|EZSPM541-10|RVcoll.08-L259||Spain|658[0n]
Erebia neoridas|EULEP5450-17|RVcoll11J187||France|658[0n]
Erebia neoridas|EULEP5476-17|RVcoll12O934||France|658[0n]
Erebia neoridas|WMB3520-14|RVcoll.10-B969||France|658[0n]
Erebia neoridas|WMB5312-14|RVcoll.14-I731||France|658[0n]
Erebia neoridas|EZSPC979-10|RVcoll.06-V754||Spain|658[0n]
Erebia neoridas|EZSPC978-10|RVcoll.06-V753||Spain|658[0n]
Erebia neoridas|EZSPC696-10|RVcoll.06-V731||Spain|658[0n]
Erebia neoridas|EZSPC695-10|RVcoll.06-V730||Spain|658[0n]
Erebia neoridas|EZSPC1370-10|RVcoll.09-X063||Spain|658[0n]
Erebia neoridas|EZSPC1369-10|RVcoll.09-X062||Spain|658[0n]
Erebia neoridas|EZSPM020-09|RVcoll.08-P043||Spain|658[0n]
Erebia neoridas|EZSPM016-09|RVcoll.08-P013||Spain|658[0n]
Erebia neoridas|EZSPC977-10|RVcoll.06-V748.1||Spain|658[0n]
Erebia neoridas|BIBSA217-15|RVcoll.14-E005||Italy|658[0n]
Erebia neoridas|BIBSA131-15|RVcoll.14-I146||Italy|658[0n]
Erebia neoridas|EULEP5472-17|RVcoll12O639||France|658[0n]
Erebia neoridas|WMB3887-14|RVcoll.12-O660||France|658[0n]
Erebia neoridas|PHLSA392-11|TLMF Lep 05847||Italy|658[0n]
Erebia gorge|EULEP2805-15|RVcoll.14-N889||France|658[0n]
Erebia gorge|EULEP5875-18|RVcoll15O511||Spain|658[0n]

[illegible]
Erebia neoridas|PHLSA392-11|TLMF Lep 05847||Italy|658[0n]
Erebia gorge|EULEP2805-15|RVcoll.14-N889||France|658[0n]
Erebia gorge|EULEP5875-18|RVcoll15O511||Spain|658[0n]
Erebia gorge|EZSPC937-10|RVcoll.09-X030||Spain|658[0n]
Erebia gorge|EZSPN028-09|RVcoll.09-T055||Spain|658[0n]
Erebia gorge|EZSPC390-09|RVcoll.08-P086||Andorra|658[0n]
Erebia gorge|EULEP3842-16|RVcoll14A971||Bulgaria|658[0n]
Erebia gorge|EULEP3757-16|RVcoll10B930||France|658[0n]
Erebia gorge|EULEP2800-15|RVcoll.14-N884||Albania|658[0n]
Erebia gorge|EULEP2799-15|RVcoll.14-N883||Albania|658[0n]
Erebia gorge|EULEP3839-16|RVcoll15P091||Macedonia|658[0n]
Erebia gorge|EULEP3840-16|RVcoll15P092||Macedonia|658[1n]
Erebia gorge|EULEP2801-15|RVcoll.14-N885||Bosnia and Herzegovina|658[0n]
Erebia gorge|EULEP3838-16|RVcoll15P090||Macedonia|658[0n]
Erebia gorge|EULEP3837-16|RVcoll15P089||Macedonia|658[0n]
Erebia gorge|PHLAA226-09|TLMF Lep 00266||Austria|655[0n]
Erebia gorge|PHLSA383-11|TLMF Lep 05838||Austria|658[0n]
Erebia gorge|EULEP3836-16|RVcoll15J357||France|658[0n]
Erebia gorge|ODOPE679-11|BC ZSM Lep 50320||Germany|658[0n]
Erebia gorge|PHLAA364-09|TLMF Lep 00404||Italy|658[0n]
Erebia gorge|LEATG229-14|TLMF Lep 14016||Austria|658[0n]
Erebia gorge|PHLSA401-11|TLMF Lep 05856||Austria|658[0n]
Erebia gorge|LEATC132-13|TLMF Lep 11259||Italy|658[0n]
Erebia gorge|ODOPE680-11|BC ZSM Lep 50321||Germany|658[0n]
Erebia gorge|GWOTF668-12|BC ZSM Lep 62374||Germany|658[0n]
Erebia gorge|GWOSA780-10|BC ZSM Lep 36646||Germany|658[0n]
Erebia gorge|WMB2692-13|RVcoll.10-C018||France|658[0n]
Erebia gorge|EULEP3826-16|RVcoll15G736||Italy|658[0n]
Erebia gorge|EULEP3828-16|RVcoll15H546||Switzerland|658[0n]
Erebia gorge|EULEP3829-16|RVcoll15H822||Italy|658[0n]
Erebia gorge|EULEP3831-16|RVcoll15I149||Austria|658[0n]
Erebia gorge|EULEP3832-16|RVcoll15I150||Austria|658[0n]
Erebia gorge|EULEP3833-16|RVcoll15I633||Austria|658[0n]
Erebia gorge|EULEP3834-16|RVcoll15I635||Austria|658[0n]
Erebia gorge|EULEP3835-16|RVcoll15J037||Switzerland|658[0n]
Erebia gorge|EULEP3830-16|RVcoll15H914||Italy|658[0n]
Erebia gorge|BIBSA1205-15|15-M310||Italy|658[0n]
Erebia gorge|LEASS559-17|TLMF Lep 22207||Austria|658[0n]
Erebia gorge|EZROM326-08|RV-08-A004||Romania|658[0n]
Erebia gorge|EZRMN356-08|RVcoll.08-M723||Romania|604[0n]
Erebia gorge|LEATC111-13|TLMF Lep 11238||Italy|658[0n]
Erebia gorge|EULEP2804-15|RVcoll.14-N888||France|613[0n]
Erebia gorge|BIBSA1148-15|15-L053||Italy|658[0n]
Erebia gorge|BIBSA1145-15|15-L049||Italy|658[0n]
Erebia gorge|LEATJ1314-16|TLMF Lep 19637||Austria|644[0n]
Erebia gorge|WMB2683-13|RVcoll.10-B934||France|631[0n]
Erebia gorge|BIBSA324-15|RVcoll.14-E127||Italy|633[0n]
Erebia gorge|PHLAA305-09|TLMF Lep 00345||Italy|630[0n]
Erebia gorge|LEATJ1258-16|TLMF Lep 19581||Austria|658[0n]
Erebia gorge|HBOK126-08|OK520-32||Austria|658[0n]
Erebia gorge|EULEP3827-16|RVcoll15H451||Switzerland|658[0n]
Erebia gorge|LEATJ1326-16|TLMF Lep 19649||Italy|658[0n]
Erebia gorge|PHLSA400-11|TLMF Lep 05855||Austria|658[0n]
Erebia gorge|EULEP208-14|RVcoll.12-L026||Spain|658[0n]
Erebia gorge|EULEP207-14|RVcoll.12-L025||Spain|658[0n]
Erebia gorge|EZSPC809-10|RVcoll.09-X328||Spain|658[0n]
Erebia gorge|EZSPC808-10|RVcoll.09-X327||Spain|658[0n]
Erebia gorge|EZSPC807-10|RVcoll.09-X326||Spain|658[0n]
Erebia gorge|EZSPC806-10|RVcoll.09-X325||Spain|658[0n]
Erebia gorge|PHLSA382-11|TLMF Lep 05837||Switzerland|658[0n]
Erebia gorge|LEATG230-14|TLMF Lep 14017||Austria|658[0n]
Erebia gorgone|EULEP5878-18|RVcoll15O515||Spain|658[0n]
Erebia gorgone|EZSPC1331-10|RVcoll.130209KL46||Spain|658[0n]
Erebia gorgone|EZSPM842-12|Rvcoll. 140711ZG95||Spain|658[0n]
Erebia gorgone|EZSPM838-12|Rvcoll. 140711ZG44||Spain|658[0n]
Erebia gorgone|EZSPC854-10|RVcoll.08-R258||Spain|658[0n]
Erebia gorgone|EZSPC853-10|RVcoll.08-R256||Spain|658[0n]
Erebia gorgone|EZSPC852-10|RVcoll.08-R255||Spain|658[0n]
Erebia gorgone|EZSPM199-09|RVcoll.08-R251||Spain|658[0n]
Erebia gorgone|EZSPC917-10|RVcoll.09-V878||Spain|658[0n]
Erebia gorgone|EZSPC851-10|RVcoll.08-R253||Spain|658[0n]
Erebia gorgone|EZSPM198-09|RVcoll.08-R250||Spain|658[0n]
Erebia gorgone|EZSPM197-09|RVcoll.08-R248||Spain|658[0n]
Erebia aethiopellus|WMB2685-13|RVcoll.10-B950||France|658[0n]
Erebia aethiopellus|EULEP750-15|RVcoll.12-O883||Italy|658[0n]
Erebia aethiopellus|WMB1641-13|RVcoll.12-O885||France|658[0n]
Erebia aethiopellus|BIBSA898-15|LD-3510||Italy|658[0n]
Erebia aethiopellus|EULEP3758-16|RVcoll10C026||France|658[0n]
Erebia aethiopellus|WMB2694-13|RVcoll.10-C025||France|658[0n]
Erebia aethiopellus|BIBSA296-15|RVcoll.14-E098||Italy|658[0n]
Erebia aethiopellus|PHLAA366-09|TLMF Lep 00406||Italy|658[0n]
Erebia rhodopensis|EULEP1838-15|RVcoll.14-J960||Albania|658[0n]
Erebia rhodopensis|EULEP917-15|RVcoll.14-C784||Bulgaria|658[0n]
Erebia rhodopensis|EULEP922-15|RVcoll.14-C802||Bulgaria|658[0n]
Erebia rhodopensis|EULEP923-15|RVcoll.14-C803||Bulgaria|658[0n]
Erebia rhodopensis|EULEP924-15|RVcoll.14-C806||Bulgaria|658[0n]
Erebia rhodopensis|EULEP925-15|RVcoll.14-C807||Bulgaria|658[0n]
Erebia rhodopensis|EULEP1837-15|RVcoll.14-J959||Albania|658[0n]
Erebia rhodopensis|EULEP3959-16|RVcoll14O348||Bulgaria|635[0n]
Erebia mnestra|EULEP3887-16|RVcoll15G703||Italy|658[0n]
Erebia mnestra|EULEP3891-16|RVcoll15G431||Switzerland|636[0n]
Erebia mnestra|EULEP3888-16|RVcoll15G858||Switzerland|658[0n]
Erebia mnestra|EULEP3886-16|RVcoll15G563||Switzerland|658[0n]
Erebia mnestra|EULEP3885-16|RVcoll14W710||Italy|627[0n]
Erebia mnestra|WMB5107-14|RVcoll. 14-L238||Italy|658[0n]
Erebia mnestra|HBOK130-08|OK520-36||Italy|658[0n]
Erebia mnestra|HBOK129-08|OK520-35||Italy|658[0n]
Erebia mnestra|LEATD153-13|TLMF Lep 12800||Italy|658[0n]
Erebia mnestra|EULEP3890-16|RVcoll15J515||France|658[0n]
Erebia mnestra|LEATC091-13|TLMF Lep 11218||Italy|658[0n]
Erebia mnestra|EULEP3889-16|RVcoll15H538||Switzerland|658[0n]
Erebia mnestra|BIBSA394-15|RVcoll.14-I062||Italy|658[0n]
[illegible]

Erebia mnestra|LEATC091-13|TLMF Lep 11218||Italy|658[0n]
Erebia mnestra|EULEP3889-16|RVcoll15H538||Switzerland|658[0n]
Erebia mnestra|BIBSA394-15|RVcoll.14-I062||Italy|658[0n]
Erebia mnestra|PHLAB378-10|TLMF Lep 01178||Switzerland|658[1n]
Erebia mnestra|ABOLD438-16|TLMF Lep 21590||Austria|658[0n]
Erebia mnestra|PHLAB379-10|TLMF Lep 01179||Switzerland|658[0n]
Erebia mnestra|ABOLD437-16|TLMF Lep 21589||Austria|658[0n]
Erebia pluto|BIBSA1143-15|15-L041||Italy|658[0n]
Erebia pluto|BIBSA1142-15|15-L040||Italy|658[0n]
Erebia pluto|BIBSA1141-15|15-L039||Italy|658[0n]
Erebia pluto|PHLAA428-09|TLMF Lep 00468||France|658[0n]
Erebia pluto|PHLAA427-09|TLMF Lep 00467||France|658[0n]
Erebia pluto|EULEP3946-16|RVcoll15J055||Switzerland|658[0n]
Erebia pluto|EULEP3945-16|RVcoll15J050||Switzerland|658[0n]
Erebia pluto|EULEP3944-16|RVcoll15I958||Switzerland|658[0n]
Erebia pluto|EULEP3943-16|RVcoll15I947||Switzerland|658[0n]
Erebia pluto|LEATC113-13|TLMF Lep 11240||Italy|658[0n]
Erebia pluto|EULEP3948-16|RVcoll15J316||France|658[0n]
Erebia pluto|EULEP3947-16|RVcoll15J314||France|658[0n]
Erebia pluto|EULEP3951-16|RVcoll15O019||Italy|658[0n]
Erebia pluto|EULEP3950-16|RVcoll15O018||Italy|658[0n]
Erebia pluto|EULEP3949-16|RVcoll15O017||Italy|658[0n]
Erebia pluto|EULEP1844-15|RVcoll.11-J440||Italy|658[0n]
Erebia pluto|EULEP5458-17|RVcoll11J445||Italy|658[0n]
Erebia pluto|EULEP5457-17|RVcoll11J444||Italy|658[0n]
Erebia pluto|EULEP5456-17|RVcoll11J443||Italy|658[0n]
Erebia pluto|EULEP5455-17|RVcoll11J442||Italy|658[0n]
Erebia pluto|EULEP3952-16|RVcoll15O037||Italy|658[0n]
Erebia pluto|EULEP3942-16|RVcoll15I703||Austria|658[0n]
Erebia pluto|EULEP3941-16|RVcoll15I637||Austria|658[0n]
Erebia pluto|EULEP3940-16|RVcoll15H825||Italy|658[0n]
Erebia pluto|LEATC616-13|TLMF Lep 12598||Austria|658[0n]
Erebia pluto|ABOLA431-14|TLMF Lep 16118||Austria|658[0n]
Erebia pluto|ABOLA430-14|TLMF Lep 16117||Austria|658[0n]
Erebia pluto|ODOPE678-11|BC ZSM Lep 50319||Germany|615[0n]
Erebia pluto|ODOPE677-11|BC ZSM Lep 50318||Germany|658[0n]
Erebia pluto|PHLAA303-09|TLMF Lep 00343||Italy|658[0n]
Erebia pluto|PHLAA302-09|TLMF Lep 00342||Italy|658[0n]
Erebia pluto|LEATC126-13|TLMF Lep 11253||Italy|658[0n]
Erebia sthennyo|EZSPM835-12|Rvcoll. 140711ZG24||Spain|658[0n]
Erebia sthennyo|EZSPM829-12|Rvcoll. 140711ZG04||Spain|658[0n]
Erebia sthennyo|EZSPM790-12|Rvcoll. 070611MH49||Spain|658[0n]
Erebia sthennyo|EZSPC835-10|RVcoll.07-W122|Genit. examined|Spain|658[0n]
Erebia sthennyo|EZSPC813-10|RVcoll.07-W115|Genit. examined|Spain|658[0n]
Erebia sthennyo|EZSPC843-10|RVcoll.09-T060|Genit. examined|Spain|658[0n]
Erebia sthennyo|EZSPC842-10|RVcoll.09-T059|Genit. examined|Spain|658[0n]
Erebia sthennyo|EZSPC836-10|RVcoll.09-T058|Genit. examined|Spain|658[0n]
Erebia sthennyo|EZSPC812-10|RVcoll.07-W111|Genit. examined|Spain|658[0n]
Erebia sthennyo|EZSPM562-10|RVcoll.07-W107||Spain|658[0n]
Erebia sthennyo|EZSPM561-10|RVcoll.07-W106|Genit. examined|Spain|658[0n]
Erebia pandrose|EULEP811-15|RVcoll.13-U464||Italy|658[0n]
Erebia pandrose|PHLAF587-11|TLMF Lep 05757||Italy|658[0n]
Erebia pandrose|PHLAA304-09|TLMF Lep 00344||Italy|658[0n]
Erebia pandrose|LEATC127-13|TLMF Lep 11254||Italy|658[0n]
Erebia pandrose|EULEP3926-16|RVcoll15G750||Switzerland|658[0n]
Erebia pandrose|EULEP3925-16|RVcoll15G688||Italy|658[0n]
Erebia pandrose|EULEP114-14|RVcoll.09-X338||Andorra|658[0n]
Erebia pandrose|EZSPC397-09|RVcoll.08-P085|Genit. examined|Andorra|658[0n]
Erebia pandrose|EULEP115-14|RVcoll.09-X339||Andorra|658[0n]
Erebia pandrose|EZSPC396-09|RVcoll.08-P084|Genit. examined|Andorra|658[0n]
Erebia pandrose|BIBSA1206-15|15-M319||Italy|658[0n]
Erebia pandrose|EULEP3930-16|RVcoll15I152||Austria|658[0n]
Erebia pandrose|LEASS518-17|TLMF Lep 22166||Austria|658[0n]
Erebia pandrose|EULEP184-14|RVcoll.11-J851||Romania|658[0n]
Erebia pandrose|EZROM322-08|RV-08-A000||Romania|658[0n]
Erebia pandrose|PHLAW016-13|TLMF Lep 09813||Austria|658[0n]
Erebia pandrose|EULEP2835-15|RVcoll.14-N919||Slovakia|658[0n]
Erebia pandrose|EULEP3924-16|RVcoll10B427||Bulgaria|658[0n]
Erebia pandrose|EULEP3923-16|RVcoll10B426||Bulgaria|658[0n]
Erebia pandrose|LEATG064-14|TLMF Lep 13851||Austria|658[0n]
Erebia pandrose|EZROM963-08|RVcoll.08-M652||Romania|658[0n]
Erebia pandrose|EZROM964-08|RVcoll.08-M662||Romania|658[0n]
Erebia pandrose|EULEP3929-16|RVcoll15H823||Italy|658[0n]
Erebia pandrose|EULEP4778-16|RVcoll16H306||Norway|658[0n]
Erebia pandrose|EULEP5090-16|RVcoll16H432||Sweden|658[0n]
Erebia pandrose|EULEP2464-15|RVcoll.14-V290||Switzerland|658[0n]
Erebia pandrose|LEASS793-17|TLMF Lep 22441||Italy|658[0n]
Erebia pandrose|EULEP3927-16|RVcoll15G918||Switzerland|658[0n]
Erebia pandrose|EZROM962-08|RVcoll.08-M490||Romania|658[0n]
Erebia pandrose|LEFID392-10|MM06305||Finland|658[0n]
Erebia pandrose|LON500-08|NHMO-08151||Norway|657[0n]
Erebia pandrose|GWOSZ209-11|BC ZSM Lep 41965||Italy|658[0n]
Erebia pandrose|LON176-08|NHMO-06177||Norway|657[0n]
Erebia pandrose|LEFIG496-10|MM14572||Finland|658[0n]
Erebia pandrose|LEFIA020-10|MM00091||Finland|658[0n]
Erebia pandrose|LEATC112-13|TLMF Lep 11239||Italy|658[0n]
Erebia pandrose|PHLAF349-11|TLMF Lep 05519||Macedonia|658[0n]
Erebia pandrose|PHLAF350-11|TLMF Lep 05520||Macedonia|658[0n]
Erebia pandrose|PHLAC694-10|TLMF Lep 02729||Switzerland|658[0n]
Erebia pandrose|PHLAA367-09|TLMF Lep 00407||Italy|658[0n]
Erebia pandrose|ODOPE688-11|BC ZSM Lep 50329||Germany|658[0n]
Erebia pandrose|EULEP3928-16|RVcoll15H565||Switzerland|658[0n]
Erebia pandrose|GWOTF667-12|BC ZSM Lep 62373||Germany|658[0n]
Erebia pandrose|GWORL287-09|BC ZSM Lep 21999||Germany|658[0n]
Erebia pandrose|LEATG065-14|TLMF Lep 13852||Austria|658[0n]
Erebia pandrose|PHLAB372-10|TLMF Lep 01172||Switzerland|658[0n]
Erebia pandrose|PHLAB1036-10|TLMF Lep 01836||Switzerland|658[0n]
Erebia epiphron|EULEP5638-17|RVcoll15D782|Genit. examined|Spain|658[0n]
Erebia epiphron|EULEP5637-17|RVcoll15D781||Spain|658[0n]
Erebia epiphron|EULEP5636-17|RVcoll15D780||Spain|658[0n]
Erebia epiphron|EULEP5635-17|RVcoll15D779||Spain|658[0n]
Erebia epiphron|EULEP5634-17|RVcoll15D778|Genit. examined|Spain|658[0n]
Erebia epiphron|EULEP5633-17|RVcoll15D777||Spain|658[0n]

Erebia epiphron|EULEP5635-17|RVcoll15D779||Spain|658[0n]
Erebia epiphron|EULEP5634-17|RVcoll15D778|Genit. examined|Spain|658[0n]
Erebia epiphron|EULEP5633-17|RVcoll15D777||Spain|658[0n]
Erebia epiphron|WMB5455-14|RVcoll.14-N230||Spain|658[0n]
Erebia orientalis|EULEP920-15|RVcoll.14-C794||Bulgaria|658[0n]
Erebia orientalis|EULEP915-15|RVcoll.14-C779||Bulgaria|658[0n]
Erebia orientalis|EULEP907-15|RVcoll.14-C754|Genit. examined|Bulgaria|658[0n]
Erebia orientalis|EULEP1093-15|RVcoll.14-F063||Serbia|658[0n]
Erebia orientalis|EULEP1087-15|RVcoll.14-F040||Serbia|658[0n]
Erebia orientalis|EULEP1086-15|RVcoll.14-F039|Genit. examined|Serbia|658[0n]
Erebia orientalis|EULEP3914-16|RVcoll10B424||Bulgaria|658[0n]
Erebia orientalis|EULEP3913-16|RVcoll10A932||Bulgaria|658[0n]
Erebia orientalis|EULEP3912-16|RVcoll10A931||Bulgaria|658[0n]
Erebia epiphron|EULEP5648-17|RVcoll15G867||Switzerland|658[1n]
Erebia epiphron|EZROM151-08|RV-07-E456||Romania|658[0n]
Erebia epiphron|EZROM915-08|RVcoll.07-D631||Romania|658[0n]
Erebia epiphron|EZROM672-08|RV-06-M985||Romania|658[0n]
Erebia epiphron|EZROM150-08|RV-06-V683||Romania|655[0n]
Erebia epiphron|EZROM149-08|RV-06-M974||Romania|658[0n]
Erebia epiphron|EZROM914-08|RVcoll.06-M987||Romania|658[0n]
Erebia epiphron|EZROM918-08|RVcoll.08-M614||Romania|658[0n]
Erebia epiphron|EZROM917-08|RVcoll.08-M607||Romania|658[0n]
Erebia epiphron|EZROM916-08|RVcoll.07-E495|Genit. examined|Romania|658[0n]
Erebia epiphron|EZROM1037-09|RVcoll.06-V706||Romania|658[0n]
Erebia epiphron|EULEP2115-15|RVcoll.14-J771||France|658[0n]
Erebia epiphron|EZSPC1157-10|RVcoll.08-R257||Spain|658[0n]
Erebia epiphron|EZSPC1114-10|RVcoll.08-M995||Andorra|658[0n]
Erebia epiphron|EZSPC1113-10|RVcoll.08-M994|Genit. examined|Andorra|658[0n]
Erebia epiphron|EZSPC1365-10|RVcoll.09-X029||Spain|658[0n]
Erebia epiphron|EZSPM223-09|RVcoll.08-R413||Spain|658[0n]
Erebia epiphron|EZSPM221-09|RVcoll.08-R410|Genit. examined|Spain|658[0n]
Erebia epiphron|EZSPC383-09|RVcoll.08-R260||Spain|658[0n]
Erebia epiphron|EZSPC380-09|RVcoll.08-R174||Spain|658[0n]
Erebia epiphron|EZSPC384-09|RVcoll.08-R261||Spain|658[0n]
Erebia epiphron|EZSPC382-09|RVcoll.08-R259||Spain|658[0n]
Erebia epiphron|EZSPN036-09|RVcoll.09-T080||Spain|658[0n]
Erebia epiphron|EZSPC381-09|RVcoll.07-W121||Spain|658[0n]
Erebia epiphron|OXB616-15|14-O005||Italy|658[0n]
Erebia epiphron|OXB335-15|RVcoll.14-N049||Italy|658[0n]
Erebia epiphron|EULEP5649-17|RVcoll15H312|Genit. examined|Switzerland|658[0n]
Erebia epiphron|PHLAF352-11|TLMF Lep 05522||Macedonia|658[0n]
Erebia epiphron|LEATJ1310-16|TLMF Lep 19633||Austria|614[0n]
Erebia epiphron|EULEP3798-16|RVcoll15P093||Macedonia|658[0n]
Erebia epiphron|EULEP2793-15|RVcoll.14-N877||Macedonia|658[0n]
Erebia epiphron|EULEP5665-17|RVcoll15Q015||Macedonia|658[1n]
Erebia epiphron|LEASS507-17|TLMF Lep 22155||Austria|658[0n]
Erebia epiphron|BIBSA392-15|RVcoll.14-I060||Italy|658[0n]
Erebia epiphron|BIBSA385-15|RVcoll.14-I053||Italy|658[0n]
Erebia epiphron|GBLAC376-13|BC ZSM Lep 75242||Germany|658[0n]
Erebia epiphron|BIBSA1077-15|15-K528||Italy|658[0n]
Erebia epiphron|EULEP5652-17|RVcoll15I602||Austria|658[0n]
Erebia epiphron|EULEP5651-17|RVcoll15I330|Genit. examined|Austria|658[0n]
Erebia epiphron|EULEP5650-17|RVcoll15I016||Italy|658[0n]
Erebia epiphron|EULEP183-14|RVcoll.11-J460||Switzerland|658[0n]
Erebia epiphron|EULEP3799-16|RVcoll15P094||Macedonia|658[0n]
Erebia epiphron|EULEP3796-16|RVcoll15J040||Switzerland|658[0n]
Erebia epiphron|EULEP3795-16|RVcoll15I957||Switzerland|658[0n]
Erebia epiphron|EULEP3794-16|RVcoll15I860||Austria|658[0n]
Erebia epiphron|GWOTF669-12|BC ZSM Lep 62375||Germany|658[0n]
Erebia epiphron|ODOPE671-11|BC ZSM Lep 50312||Germany|658[0n]
Erebia epiphron|ODOPE670-11|BC ZSM Lep 50311||Germany|658[0n]
Erebia epiphron|LEATC104-13|TLMF Lep 11231||Italy|658[0n]
Erebia epiphron|HBOK107-08|OK520-13||Austria|658[0n]
Erebia epiphron|PHLAF351-11|TLMF Lep 05521||Macedonia|658[0n]
Erebia epiphron|LEATG228-14|TLMF Lep 14015||Austria|658[0n]
Erebia epiphron|PHLAA340-09|TLMF Lep 00380||Austria|658[0n]
Erebia epiphron|PHLSA650-11|TLMF Lep 06105||Austria|658[0n]
Erebia epiphron|PHLAB364-10|TLMF Lep 01164||Switzerland|658[0n]
Erebia epiphron|LEATC105-13|TLMF Lep 11232||Italy|658[0n]
Erebia epiphron|EULEP1878-15|RVcoll.14-A446||Italy|658[0n]
Erebia epiphron|WMB5276-14|RVcoll.14-A619||Italy|658[0n]
Erebia epiphron|EULEP1875-15|RVcoll.14-A259||Italy|658[0n]
Erebia epiphron|WMB5256-14|RVcoll.14-A260||Italy|658[0n]
Erebia epiphron|PHLAB1223-10|TLMF Lep 02023||Italy|658[0n]
Erebia epiphron|EULEP264-14|RVcoll.12-R465||United Kingdom|658[0n]
Erebia epiphron|EULEP263-14|RVcoll.12-R464||United Kingdom|658[0n]
Erebia epiphron|EULEP262-14|RVcoll.12-R463||United Kingdom|658[0n]
Erebia epiphron|EULEP261-14|RVcoll.12-R462||United Kingdom|658[0n]
Erebia epiphron|EULEP5491-17|RVcoll12R466||United Kingdom|658[0n]
Erebia epiphron|EULEP267-14|RVcoll.12-R471||United Kingdom|658[0n]
Erebia epiphron|EULEP266-14|RVcoll.12-R469||United Kingdom|658[0n]
Erebia epiphron|EULEP265-14|RVcoll.12-R468||United Kingdom|658[0n]
Erebia epiphron|EULEP170-14|RVcoll.11-I916||France|658[0n]
Erebia epiphron|PHLAB352-10|TLMF Lep 01152||Switzerland|658[0n]
Erebia epiphron|PHLAB320-10|TLMF Lep 01120||Switzerland|658[0n]
Erebia epiphron|PHLAB319-10|TLMF Lep 01119||Switzerland|658[0n]
Erebia epiphron|PHLAB314-10|TLMF Lep 01114||Switzerland|658[0n]
Erebia epiphron|BIBSA206-15|RVcoll.14-D994||Italy|658[0n]
Erebia epiphron|EULEP3797-16|RVcoll15J516||France|658[0n]
Erebia epiphron|WMB2693-13|RVcoll.10-C021|Genit. examined|France|658[0n]
Erebia epiphron|PHLAA411-09|TLMF Lep 00451||France|658[0n]
Erebia epiphron|BIBSA298-15|RVcoll.14-E100||Italy|628[0n]
Erebia epiphron|WMB2684-13|RVcoll.10-B939||France|658[0n]
Erebia epiphron|LEATJ1329-16|TLMF Lep 19652||Italy|658[0n]
Erebia epiphron|PHLAA365-09|TLMF Lep 00405||Italy|658[0n]
Erebia christi|EULEP3782-16|RVcoll15O003||Switzerland|654[0n]
Erebia christi|EULEP3781-16|RVcoll15G833||Italy|658[0n]
Erebia christi|EULEP3780-16|RVcoll15G760||Italy|658[0n]
Erebia christi|EULEP3779-16|RVcoll14V886||Italy|658[0n]
Erebia pharte|EULEP3936-16|RVcoll15I411||Austria|658[0n]
Erebia pharte|PHLAI566-13|TLMF Lep 09128||Austria|658[0n]
Erebia pharte|PHLAF661-11|TLMF Lep 05831||Switzerland|658[0n]
[illegible]

Erebia pharte|EULEP3936-16|RVcoll15I411||Austria|658[0n]
Erebia pharte|PHLAI566-13|TLMF Lep 09128||Austria|658[0n]
Erebia pharte|PHLAF661-11|TLMF Lep 05831||Switzerland|658[0n]
Erebia pharte|PHLAF660-11|TLMF Lep 05830||Austria|658[0n]
Erebia pharte|ODOPE672-11|BC ZSM Lep 50313||Germany|658[0n]
Erebia pharte|ODOPE673-11|BC ZSM Lep 50314||Germany|658[0n]
Erebia pharte|GWOSK899-11|BC ZSM Lep 49400||Germany|658[0n]
Erebia pharte|GWOSA783-10|BC ZSM Lep 36649||Germany|658[0n]
Erebia pharte|EULEP3932-16|RVcoll15G681||Italy|658[0n]
Erebia pharte|EULEP3933-16|RVcoll15H279||Switzerland|658[0n]
Erebia pharte|EULEP3934-16|RVcoll15H691||Switzerland|658[0n]
Erebia pharte|EULEP3935-16|RVcoll15I198||Austria|658[0n]
Erebia pharte|EULEP3937-16|RVcoll15I776||Austria|658[0n]
Erebia pharte|EULEP3938-16|RVcoll15J411||France|658[0n]
Erebia pharte|EULEP2453-15|RVcoll.14-V275||Switzerland|658[0n]
Erebia pharte|EULEP2035-15|RVcoll.14-J514||Italy|658[0n]
Erebia pharte|EULEP2036-15|RVcoll.14-J515||Italy|658[0n]
Erebia pharte|LEASS491-17|TLMF Lep 22139||Austria|658[0n]
Erebia pharte|LEASS516-17|TLMF Lep 22164||Austria|658[0n]
Erebia pharte|LEASS564-17|TLMF Lep 22212||Austria|658[0n]
Erebia pharte|LEASS565-17|TLMF Lep 22213||Austria|658[0n]
Erebia pharte|LEATJ1259-16|TLMF Lep 19582||Austria|658[1n]
Erebia pharte|LEATG493-14|TLMF Lep 14280||Italy|658[0n]
Erebia pharte|PHLAF659-11|TLMF Lep 05829||Austria|658[0n]
Erebia pharte|PHLAG251-12|TLMF Lep 06561||Austria|658[0n]
Erebia pharte|PHLSA394-11|TLMF Lep 05849||Austria|658[0n]
Erebia pharte|PHLAH702-12|TLMF Lep 08521||Austria|658[0n]
Erebia pharte|EZROM169-08|RV-07-C148||Romania|658[0n]
Erebia pharte|EZROM168-08|RV-07-C145|Genit. examined|Romania|658[0n]
Erebia pharte|EULEP3939-16|RVcoll15P049||Slovenia|658[0n]
Erebia pharte|PHLAF588-11|TLMF Lep 05758||Italy|658[0n]
Erebia meolans|WMB5254-14|RVcoll.14-A248||Italy|658[0n]
Erebia meolans|BIBSA1146-15|15-L051||Italy|658[0n]
Erebia meolans|EULEP2123-15|RVcoll.14-J905||France|658[0n]
Erebia meolans|ABOLD598-17|TLMF Lep 21674||Austria|658[0n]
Erebia palarica|WMB3436-14|RVcoll.09-X538||Spain|658[0n]
Erebia palarica|EZSPM776-12|Rvcoll. 050111LP78||Spain|658[0n]
Erebia palarica|EZSPM772-12|Rvcoll. 050111LP32||Spain|658[0n]
Erebia palarica|EZSPM228-09|RVcoll.08-R423||Spain|658[0n]
Erebia palarica|EZSPM222-09|RVcoll.08-R411||Spain|658[0n]
Erebia palarica|EZSPM215-09|RVcoll.08-R400||Spain|658[0n]
Erebia palarica|EZSPM155-09|RVcoll.08-P680||Spain|658[0n]
Erebia palarica|EZSPN973-09|RVcoll.08-L998||Spain|658[0n]
Erebia palarica|EZSPN888-09|RVcoll.08-L799||Spain|658[0n]
Erebia palarica|EZSPM300-09|RVcoll.08-J847||Spain|658[0n]
Erebia palarica|EZSPN887-09|RVcoll.08-L797||Spain|658[0n]
Erebia meolans|EZSPM848-12|Rvcoll. 150511SD34||Spain|658[0n]
Erebia meolans|EZSPM847-12|Rvcoll. 150511SD33||Spain|658[0n]
Erebia meolans|BIBSA215-15|RVcoll.14-E003||Italy|658[0n]
Erebia meolans|EZSPC1386-10|RVcoll.09-X544||Spain|658[0n]
Erebia meolans|EZSPM836-12|Rvcoll. 140711ZG33||Spain|658[0n]
Erebia meolans|EZSPC1254-10|RVcoll.09-V877||Spain|658[0n]
Erebia meolans|EZSPC1264-10|RVcoll.090702PE5||Spain|658[0n]
Erebia meolans|EZSPC1075-10|RVcoll.08-L480||Spain|658[0n]
Erebia meolans|EULEP3990-16|RVcoll14V132||Italy|653[0n]
Erebia meolans|WMB6556-18|RVcoll14A098||Italy|658[0n]
Erebia meolans|WMB3353-14|RVcoll.08-R420||Spain|658[0n]
Erebia meolans|WMB782-13|RVcoll.07-C014||France|658[0n]
Erebia meolans|EZSPC1240-10|RVcoll.09-V752||Spain|658[0n]
Erebia meolans|WMB851-13|RVcoll.08-P201||France|658[0n]
Erebia meolans|EULEP2828-15|RVcoll.14-N912||France|658[0n]
Erebia meolans|WMB783-13|RVcoll.07-C015||France|641[0n]
Erebia meolans|EZSPC1044-10|RVcoll.08-J409||Spain|640[0n]
Erebia meolans|EZSPM774-12|Rvcoll. 050111LP49||Spain|635[0n]
Erebia meolans|EZSPM179-09|RVcoll.08-R144||Spain|658[0n]
Erebia meolans|EZSPM153-09|RVcoll.08-P673||Spain|658[0n]
Erebia meolans|EZSPM152-09|RVcoll.08-P672||Spain|658[0n]
Erebia meolans|EZSPM095-09|RVcoll.08-P422||Spain|658[0n]
Erebia meolans|LEATJ1328-16|TLMF Lep 19651||Italy|658[0n]
Erebia meolans|PHLAB317-10|TLMF Lep 01117||Switzerland|658[0n]
Erebia meolans|PHLAB316-10|TLMF Lep 01116||Switzerland|658[0n]
Erebia meolans|PHLAB318-10|TLMF Lep 01118||Switzerland|658[0n]
Erebia meolans|EZSPC1103-10|RVcoll.08-L978||Spain|658[0n]
Erebia meolans|EZSPC1102-10|RVcoll.08-L975||Spain|658[0n]
Erebia meolans|EZSPN965-09|RVcoll.08-L976||Spain|658[0n]
Erebia meolans|EULEP2118-15|RVcoll.14-J879||France|658[0n]
Erebia meolans|EZSPC1241-10|RVcoll.09-V753||Spain|628[0n]
Erebia meolans|PHLSA381-11|TLMF Lep 05836||Switzerland|658[0n]
Erebia meolans|PHLAW053-13|TLMF Lep 09850||Austria|658[0n]
Erebia meolans|PHLAF665-11|TLMF Lep 05835||Switzerland|658[0n]
Erebia aethiops|OXB603-15|14-N992||Italy|658[0n]
Erebia aethiops|LEATC177-13|TLMF Lep 11304||Italy|658[1n]
Erebia aethiops|BIBSA934-15|16-A025||Italy|658[0n]
Erebia aethiops|OXB259-15|OXB-TGS-836||United Kingdom|658[0n]
Erebia aethiops|EULEP2100-15|RVcoll.14-J589||Ukraine|658[0n]
Erebia aethiops|EULEP956-15|RVcoll.14-D002||Bulgaria|658[0n]
Erebia aethiops|EULEP873-15|RVcoll.14-C373||Greece|658[0n]
Erebia aethiops|WMB1862-13|RVcoll.11-J169||France|658[0n]
Erebia aethiops|EZROM145-08|RV-07-E597||Romania|658[0n]
Erebia aethiops|PHLAI541-13|TLMF Lep 09103||Austria|658[0n]
Erebia aethiops|PHLAA680-09|TLMF Lep 00720||Austria|658[1n]
Erebia aethiops|GBLAA1500-15|BC ZSM Lep 87291||Germany|658[0n]
Erebia aethiops|EULEP3761-16|RVcoll15I836||Austria|637[0n]
Erebia aethiops|EZROM144-08|RV-07-E365||Romania|658[0n]
Erebia aethiops|EZROM146-08|RV-07-C153||Romania|658[0n]
Erebia aethiops|EZROM143-08|RV-06-M983||Romania|658[0n]
Erebia aethiops|LEEUA523-11|MM20582||Latvia|658[0n]
Erebia aethiops|EZROM909-08|RVcoll.07-E488||Romania|658[0n]
Erebia aethiops|EZROM577-08|RV-07-C349||Romania|658[0n]
Erebia aethiops|EZROM906-08|RVcoll.06-V649||Romania|658[0n]
Erebia aethiops|EZROM907-08|RVcoll.07-C355||Romania|658[0n]
Erebia aethiops|EZROM908-08|RVcoll.07-C358||Romania|658[0n]
Erebia aethiops|WMB3680-14|RVcoll.11-J217||France|658[0n]

Erebia aethiops|EZROM907-08|RVcoll.07-C355||Romania|658[0n]
Erebia aethiops|EZROM908-08|RVcoll.07-C358||Romania|658[0n]
Erebia aethiops|WMB3680-14|RVcoll.11-J217||France|658[0n]
Erebia aethiops|WMB1867-13|RVcoll.11-J204||France|658[0n]
Erebia aethiops|EULEP3759-16|RVcoll15H722||Switzerland|658[0n]
Erebia aethiops|EULEP3760-16|RVcoll15I721||Austria|658[0n]
Erebia aethiops|EULEP4974-16|RVcoll16I462||Poland|658[0n]
Erebia aethiops|EULEP5015-16|RVcoll16I909||Slovakia|658[0n]
Erebia aethiops|EULEP593-15|RVcoll.10-C104||France|658[0n]
Erebia aethiops|EULEP617-15|RVcoll.11-H155||Germany|658[0n]
Erebia aethiops|EULEP631-15|RVcoll.11-J027||Switzerland|658[0n]
Erebia aethiops|EULEP980-15|RVcoll.14-D130||Austria|658[0n]
Erebia aethiops|EULEP2778-15|RVcoll.14-N862||United Kingdom|658[0n]
Erebia aethiops|GBLAB766-13|BC ZSM Lep 75537||Germany|658[0n]
Erebia aethiops|GBLAA1438-15|BC ZSM Lep 87229||Germany|658[0n]
Erebia aethiops|OXB1601-16|OXB-TGS-1319||France|658[0n]
Erebia aethiops|OXB258-15|OXB-TGS-835||United Kingdom|658[0n]
Erebia aethiops|OXB260-15|OXB-TGS-837||United Kingdom|658[0n]
Erebia aethiops|BIBSA1199-15|15-M294||Italy|612[0n]
Erebia aethiops|LEASS502-17|TLMF Lep 22150||Austria|633[0n]
Erebia aethiops|GBLAA1506-15|BC ZSM Lep 87297||Germany|623[0n]
Erebia aethiops|WMB1643-13|RVcoll.12-O948||France|655[0n]
Erebia aethiops|FBLMU479-09|BC ZSM Lep 27129||Germany|627[0n]
Erebia aethiops|ODOPE675-11|BC ZSM Lep 50316||Germany|658[0n]
Erebia aethiops|FBLMW375-10|BC ZSM Lep 37476||Germany|658[0n]
Erebia aethiops|GWORR684-10|BC ZSM Lep 32180||Germany|658[0n]
Erebia aethiops|LEATF452-14|TLMF Lep 13764||Austria|658[0n]
Erebia aethiops|PHLAI521-13|TLMF Lep 09083||Austria|658[0n]
Erebia aethiops|WMB837-13|RVcoll.07-E232||Italy|658[0n]
Erebia aethiops|WMB5027-14|RVcoll. 14-I574||Italy|658[0n]
Erebia aethiops|BIBSA1073-15|15-I540||Italy|658[0n]
Erebia aethiops|BIBSA201-15|RVcoll.14-D989||Italy|658[0n]
Erebia aethiops|OXB724-15|13-U142||Italy|658[0n]
Erebia aethiops|BIBSA125-15|RVcoll.14-D579||Italy|644[0n]
Erebia aethiops|HBOK115-08|OK520-21||Austria|609[0n]
Erebia aethiops|HBOK116-08|OK520-22||Austria|658[0n]
Erebia aethiops|ABOLD028-16|TLMF Lep 21106||Austria|658[0n]
Erebia manto|EZSPM644-12|RVcoll.11-H250||Spain|658[0n]
Erebia manto|EZSPM640-12|RVcoll.11-H050||Spain|658[0n]
Erebia manto|EZSPC394-09|RVcoll.08-R108||Spain|658[0n]
Erebia manto|EZSPC393-09|RVcoll.08-P392||Spain|658[0n]
Erebia manto|EZSPC1117-10|RVcoll.08-P390||Spain|658[0n]
Erebia manto|EZSPC395-09|RVcoll.08-R109||Spain|658[0n]
Erebia manto|EZSPC392-09|RVcoll.08-P391||Spain|658[0n]
Erebia manto|EZSPC391-09|RVcoll.07-W203||Spain|658[0n]
Erebia manto|PHLAB348-10|TLMF Lep 01148||Switzerland|658[0n]
Erebia manto|EZROM158-08|RV-07-C208||Romania|658[0n]
Erebia manto|EZROM625-08|RV-07-C209||Romania|658[0n]
Erebia manto|EZROM936-08|RVcoll.07-C206||Romania|658[0n]
Erebia manto|EULEP654-15|RVcoll.11-J429||Switzerland|633[0n]
Erebia manto|EULEP653-15|RVcoll.11-J428||Switzerland|658[0n]
Erebia manto|EULEP3854-16|RVcoll15H314||Switzerland|658[0n]
Erebia manto|EULEP3852-16|RVcoll15H271||Switzerland|658[0n]
Erebia manto|BIBSA1510-16|RVcoll.14-I264||Italy|658[0n]
Erebia manto|WMB1856-13|RVcoll.11-I993||France|658[0n]
Erebia manto|EULEP655-15|RVcoll.11-J430||Switzerland|658[0n]
Erebia manto|EULEP1971-15|RVcoll.14-H844||Switzerland|658[0n]
Erebia manto|WMB1150-13|RVcoll.11-E216||France|614[0n]
Erebia manto|EZROM156-08|RV-07-E470||Romania|658[0n]
Erebia manto|EZROM157-08|RV-07-E585||Romania|658[0n]
Erebia manto|EZROM1061-09|RVcoll.07-E583||Romania|658[0n]
Erebia manto|EZROM940-08|RVcoll.07-E586||Romania|609[0n]
Erebia manto|EZROM939-08|RVcoll.07-E475||Romania|609[0n]
Erebia manto|EZROM938-08|RVcoll.07-E472||Romania|658[0n]
Erebia manto|EZROM937-08|RVcoll.07-E471||Romania|658[0n]
Erebia manto|EZROM1060-09|RVcoll.07-E476||Romania|658[0n]
Erebia manto|EULEP2099-15|RVcoll.14-J588||Ukraine|658[0n]
Erebia manto|EULEP3853-16|RVcoll15H272||Switzerland|658[0n]
Erebia manto|GWOSK900-11|BC ZSM Lep 49401||Germany|658[0n]
Erebia manto|GBLAB774-13|BC ZSM Lep 75545||Austria|658[0n]
Erebia manto|PHLAB349-10|TLMF Lep 01149||Switzerland|658[0n]
Erebia manto|LEATG499-14|TLMF Lep 14286||Italy|658[1n]
Erebia manto|ABOLD035-16|TLMF Lep 21113||Austria|658[0n]
Erebia manto|LEATG473-14|TLMF Lep 14260||Italy|658[0n]
Erebia manto|EULEP3855-16|RVcoll15H984||Italy|658[0n]
Erebia manto|EULEP3856-16|RVcoll15H985||Italy|658[0n]
Erebia manto|EULEP3857-16|RVcoll15I175||Austria|658[0n]
Erebia manto|EULEP3858-16|RVcoll15I176||Austria|658[0n]
Erebia manto|EULEP3859-16|RVcoll15I419||Austria|658[0n]
Erebia manto|EULEP3861-16|RVcoll15I652||Austria|658[0n]
Erebia manto|EULEP3862-16|RVcoll15I788||Austria|658[0n]
Erebia manto|EULEP3804-16|RVcoll15I775||Austria|658[0n]
Erebia manto|EULEP5166-17|RVcoll16G273||Italy|658[0n]
Erebia manto|BIBSA1213-15|15-M338||Italy|658[0n]
Erebia manto|LEATF450-14|TLMF Lep 13762||Austria|658[0n]
Erebia manto|WMB5022-14|RVcoll. 14-I569||Italy|658[0n]
Erebia manto|WMB5018-14|RVcoll. 14-I565||Italy|658[0n]
Erebia manto|EULEP3860-16|RVcoll15I613||Austria|650[1n]
Erebia manto|HBOK099-08|OK520-05||Austria|606[0n]
Erebia manto|HBOK100-08|OK520-06||Austria|609[0n]
Erebia manto|LEATG472-14|TLMF Lep 14259||Italy|621[0n]
Erebia manto|LEATG498-14|TLMF Lep 14285||Italy|658[0n]
Erebia manto|WMB1863-13|RVcoll.11-J175||France|658[0n]
Erebia manto|GWOSA782-10|BC ZSM Lep 36648||Germany|658[0n]
Erebia manto|GWOTD441-12|BC ZSM Lep 63572||Germany|658[0n]
Erebia manto|ODOPE669-11|BC ZSM Lep 50310||Germany|658[0n]
Erebia manto|PHLAH668-12|TLMF Lep 08487||Austria|658[0n]
Erebia dabanensis|EULEP3790-16|RVcoll14V754||Russia|658[0n]
Erebia dabanensis|EULEP450-14|KN00812||Russia|658[0n]
Erebia dabanensis|EULEP3791-16|RVcoll14V755||Russia|638[0n]
Erebia dabanensis|EULEP3793-16|RVcoll14V757||Russia|614[0n]
Erebia dabanensis|EULEP3789-16|RVcoll14V753||Russia|621[0n]
Erebia ottomana|EULEP899-15|RVcoll.14-C729||Bulgaria|658[0n]

[illegible]
Erebia dabanensis|EULEP3793-16|RVcoll14V757||Russia|614[0n]
Erebia dabanensis|EULEP3789-16|RVcoll14V753||Russia|621[0n]
Erebia ottomana|EULEP899-15|RVcoll.14-C729||Bulgaria|658[0n]
Erebia ottomana|EULEP512-15|RVcoll.07-D674||Bulgaria|658[0n]
Erebia ottomana|EULEP3918-16|RVcoll15H903||Italy|658[0n]
Erebia ottomana|EULEP3917-16|RVcoll15H902||Italy|658[0n]
Erebia ottomana|EULEP3916-16|RVcoll15H894||Italy|658[0n]
Erebia ottomana|WMB1894-13|RVcoll.11-J960||Italy|658[0n]
Erebia ottomana|EULEP511-15|RVcoll.07-D662||Bulgaria|658[0n]
Erebia ottomana|EULEP2832-15|RVcoll.14-N916||Bosnia and Herzegovina|658[0n]
Erebia ottomana|EULEP2831-15|RVcoll.14-N915||Albania|658[0n]
Erebia ottomana|EULEP2830-15|RVcoll.14-N914||Albania|658[0n]
Erebia ottomana|EULEP1886-15|RVcoll.14-B695||Albania|658[0n]
Erebia ottomana|EULEP1884-15|RVcoll.14-B692||Albania|658[0n]
Erebia ottomana|EULEP971-15|RVcoll.14-D061||Bulgaria|658[0n]
Erebia ottomana|EULEP944-15|RVcoll.14-C938||Bulgaria|658[0n]
Erebia ottomana|EULEP884-15|RVcoll.14-C479||Greece|658[0n]
Erebia ottomana|EULEP538-15|RVcoll.08-H887|Genit. examined|Serbia|658[0n]
Erebia ottomana|EULEP685-15|RVcoll.11-J900||Bulgaria|658[0n]
Erebia ottomana|EULEP5679-17|RVcoll16J912||France|658[0n]
Erebia ottomana|EULEP5678-17|RVcoll16J911||France|658[0n]
Erebia ottomana|EULEP5677-17|RVcoll16J910||France|658[0n]
Erebia ottomana|EULEP5676-17|RVcoll16J909||France|658[0n]
Erebia ottomana|EULEP3773-16|RVcoll15Q021||Macedonia|658[0n]
Erebia ottomana|EULEP3922-16|RVcoll15P086||Macedonia|658[0n]
Erebia ottomana|EULEP3921-16|RVcoll15P085||Macedonia|658[0n]
Erebia ottomana|EULEP3915-16|RVcoll10B421||Bulgaria|658[0n]
Erebia ottomana|PHLAF347-11|TLMF Lep 05517||Macedonia|658[0n]
Erebia ottomana|PHLAF348-11|TLMF Lep 05518||Macedonia|658[0n]
Erebia hispania|EZSPM650-12|RVcoll.11-I468||Spain|658[0n]
Erebia hispania|EZSPM649-12|RVcoll.11-I466||Spain|658[0n]
Erebia hispania|EZSPN1081-11|Rvcoll.09-X193||Spain|658[0n]
Erebia hispania|EZSPN674-09|RVcoll.08-J985||Spain|658[0n]
Erebia hispania|EZSPN1080-11|Rvcoll.09-X192||Spain|658[0n]
Erebia hispania|WMB3383-14|RVcoll.09-V427||Spain|658[0n]
Erebia hispania|EZSPN1082-11|Rvcoll.09-X194||Spain|658[0n]
Erebia hispania|EZSPN672-09|RVcoll.08-J978||Spain|658[0n]
Erebia hispania|EZSPN675-09|RVcoll.08-J987||Spain|658[0n]
Erebia rondoui|EZSPC850-10|RVcoll.08-R246||Spain|658[0n]
Erebia rondoui|EZSPN617-09|RVcoll.08-J410||Spain|658[3n]
Erebia rondoui|EZSPC915-10|RVcoll.09-V830||Spain|658[0n]
Erebia rondoui|EZSPC697-10|RVcoll.06-V733||Spain|658[0n]
Erebia rondoui|WMB6557-18|RVcoll14B007||Spain|658[0n]
Erebia rondoui|EZSPC849-10|RVcoll.08-R245||Spain|658[0n]
Erebia rondoui|EZSPM565-10|RVcoll.07-C031||France|658[0n]
Erebia rondoui|EZSPC837-10|RVcoll.08-R184||Spain|658[0n]
Erebia rondoui|EZSPC838-10|RVcoll.08-R187||Spain|658[0n]
Erebia rondoui|EZSPC839-10|RVcoll.08-R188||Spain|658[0n]
Erebia rondoui|EZSPC914-10|RVcoll.09-V829||Spain|658[0n]
Erebia rondoui|EZSPC1335-10|RVcoll.130209KL76||Spain|658[0n]
Erebia rondoui|EULEP1991-15|RVcoll.14-I707||France|658[0n]
Erebia rondoui|WMB784-13|RVcoll.07-C030||France|635[0n]
Erebia rondoui|EZSPM568-10|RVcoll.08-R183|Genit. examined|Spain|630[0n]
Erebia rondoui|EZSPM567-10|RVcoll.08-R175|Genit. examined|Spain|632[0n]
Erebia rondoui|EZSPM566-10|RVcoll.07-C034||France|652[0n]
Erebia cassioides|EULEP2779-15|RVcoll.14-N863||Italy|658[0n]
Erebia cassioides|EULEP1874-15|RVcoll.14-A244||Italy|658[0n]
Erebia cassioides|WMB5252-14|RVcoll.14-A245||Italy|658[0n]
Erebia cassioides|EULEP3776-16|RVcoll15I778||Austria|658[0n]
Erebia tyndarus|GBLAC377-13|BC ZSM Lep 75243||Germany|658[0n]
Erebia neleus|EZROM911-08|RVcoll.07-E494||Romania|658[0n]
Erebia neleus|EZROM147-08|RV-06-V703|Genit. examined|Romania|647[0n]
Erebia neleus|EZROM913-08|RVcoll.08-M635||Romania|658[0n]
Erebia neleus|EZROM912-08|RVcoll.07-E498||Romania|658[0n]
Erebia neleus|EZROM910-08|RVcoll.06-V701||Romania|658[0n]
Erebia neleus|EZROM671-08|RV-07-E493||Romania|658[0n]
Erebia tyndarus|ODOPE683-11|BC ZSM Lep 50324||Germany|658[0n]
Erebia tyndarus|LEATG103-14|TLMF Lep 13890||Austria|658[0n]
Erebia tyndarus|EULEP2469-15|RVcoll.14-V296||Switzerland|658[0n]
Erebia cassioides|EULEP657-15|RVcoll.11-J451||Switzerland|658[0n]
Erebia tyndarus|EULEP3998-16|RVcoll15J041||Switzerland|658[0n]
Erebia tyndarus|EULEP3996-16|RVcoll15I921||Switzerland|658[0n]
Erebia tyndarus|EULEP3995-16|RVcoll15H650||Switzerland|658[0n]
Erebia tyndarus|FBLMW289-10|BC ZSM Lep 37390||Austria|658[0n]
Erebia tyndarus|GWOSA785-10|BC ZSM Lep 36651||Germany|658[0n]
Erebia tyndarus|FBLMW293-10|BC ZSM Lep 37394||Austria|658[0n]
Erebia tyndarus|FBLMW286-10|BC ZSM Lep 37387||Austria|658[0n]
Erebia tyndarus|HBOK138-08|OK520-44||Austria|658[0n]
Erebia tyndarus|PHLSA649-11|TLMF Lep 06104||Austria|658[0n]
Erebia tyndarus|PHLAI493-13|TLMF Lep 09055||Austria|658[0n]
Erebia tyndarus|HBOK137-08|OK520-43||Austria|658[0n]
Erebia tyndarus|PHLAB376-10|TLMF Lep 01176||Switzerland|658[0n]
Erebia arvernensis|BIBSA387-15|RVcoll.14-I055||Italy|658[0n]
Erebia nivalis|LEASS560-17|TLMF Lep 22208||Austria|658[0n]
Erebia cassioides|BIBSA1079-15|15-K533||Italy|658[0n]
Erebia cassioides|EULEP3774-16|RVcoll15H843||Italy|658[0n]
Erebia cassioides|PHLAI542-13|TLMF Lep 09104||Austria|658[0n]
Erebia cassioides|PHLAF658-11|TLMF Lep 05828||Austria|658[0n]
Erebia cassioides|LEATF449-14|TLMF Lep 13761||Austria|658[0n]
Erebia calcarius|PHLAJ644-15|KLM Lep 03779||Austria|658[0n]
Erebia arvernensis|BIBSA384-15|RVcoll.14-I052||Italy|658[0n]
Erebia tyndarus|EULEP3994-16|RVcoll15H313||Switzerland|658[0n]
Erebia tyndarus|EULEP3993-16|RVcoll15G898||Switzerland|658[0n]
Erebia tyndarus|EULEP3992-16|RVcoll15G637||Switzerland|658[0n]
Erebia tyndarus|WMB883-13|RVcoll.09-X291|Genit. examined|Italy|658[0n]
Erebia cassioides|LEASS526-17|TLMF Lep 22174||Austria|658[0n]
Erebia tyndarus|EULEP609-15|RVcoll.11-E232||Switzerland|628[0n]
Erebia cassioides|OXB695-15|12-Q695||Italy|658[0n]
Erebia cassioides|EULEP1845-15|RVcoll.12-Q693||Italy|658[0n]
Erebia arvernensis|BIBSA323-15|RVcoll.14-E126||Italy|658[0n]
Erebia arvernensis|BIBSA297-15|RVcoll.14-E099||Italy|658[0n]
Erebia arvernensis|BIBSA205-15|RVcoll.14-D993||Italy|658[0n]
[illegible]

Erebia arvernensis|BIBSA323-15|RVcoll.14-E126||Italy|658[0n]
Erebia arvernensis|BIBSA297-15|RVcoll.14-E099||Italy|658[0n]
Erebia arvernensis|BIBSA205-15|RVcoll.14-D993||Italy|658[0n]
Erebia arvernensis|BIBSA204-15|RVcoll.14-D992||Italy|658[0n]
Erebia cassioides|EULEP1880-15|RVcoll.14-A545||Italy|658[0n]
Erebia arvernensis|EULEP3777-16|RVcoll15J289||France|658[0n]
Erebia cassioides|WMB5268-14|RVcoll.14-A548||Italy|658[0n]
Erebia cassioides|WMB5158-14|RVcoll.12-Q791||Italy|658[0n]
Erebia arvernensis|WMB1626-13|RVcoll.12-O795||France|658[0n]
Erebia arvernensis|WMB2695-13|RVcoll.10-C029||France|658[0n]
Erebia cassioides|PHLSA440-11|TLMF Lep 05895||Italy|658[0n]
Erebia arvernensis|PHLAI326-13|TLMF Lep 08821||Italy|658[0n]
Erebia arvernensis|LEATJ1327-16|TLMF Lep 19650||Italy|658[0n]
Erebia arvernensis|PHLAI327-13|TLMF Lep 08822||Italy|658[0n]
Erebia arvernensis|PHLAA412-09|TLMF Lep 00452||France|658[0n]
Erebia tyndarus|EULEP3997-16|RVcoll15J028||Switzerland|658[0n]
Erebia tyndarus|PHLAF662-11|TLMF Lep 05832||Switzerland|658[0n]
Erebia tyndarus|PHLAF663-11|TLMF Lep 05833||Switzerland|658[0n]
Erebia calcarius|EULEP3767-16|RVcoll15I004||Italy|658[0n]
Erebia calcarius|WMB845-13|RVcoll.08-H715||Italy|658[0n]
Erebia cassioides|HBOK140-08|OK520-46||Austria|658[0n]
Erebia cassioides|PHLAA339-09|TLMF Lep 00379||Austria|658[0n]
Erebia nivalis|LEATG495-14|TLMF Lep 14282||Italy|658[0n]
Erebia nivalis|PHLSA402-11|TLMF Lep 05857||Austria|658[0n]
Erebia nivalis|HBOK146-08|OK520-52||Austria|658[0n]
Erebia nivalis|GWOSU031-11|BC ZSM Lep 53282||Switzerland|658[0n]
Erebia nivalis|GWOSU035-11|BC ZSM Lep 53286||Switzerland|658[0n]
Erebia neleus|EZROM148-08|RV-07-E497|Genit. examined|Romania|658[0n]
Erebia arvernensis|EZSPM096-09|RVcoll.08-P423||Spain|658[0n]
Erebia arvernensis|EZSPM296-09|RVcoll.08-J838||Spain|658[0n]
Erebia arvernensis|EZSPM558-10|RVcoll.09-X054||Spain|658[0n]
Erebia arvernensis|EZSPM559-10|RVcoll.09-X027||Spain|658[0n]
Erebia arvernensis|EZSPM563-10|RVcoll.09-X024||Spain|658[0n]
Erebia arvernensis|EZSPM564-10|RVcoll.09-X025||Spain|658[0n]
Erebia arvernensis|EZSPM093-09|RVcoll.08-P420|Genit. examined|Spain|658[0n]
Erebia arvernensis|EZSPM094-09|RVcoll.08-P421||Spain|658[0n]
Erebia arvernensis|EZSPM217-09|RVcoll.08-R404||Spain|658[0n]
Erebia arvernensis|EZSPM218-09|RVcoll.08-R405||Spain|658[0n]
Erebia arvernensis|EZSPC940-10|RVcoll.09-X052||Spain|658[0n]
Erebia arvernensis|WMB3677-14|RVcoll.11-J172||France|658[0n]
Erebia calcarius|WMB846-13|RVcoll.08-H716||Italy|658[0n]
Erebia nivalis|WMB1869-13|RVcoll.11-J446||Italy|658[0n]
Erebia nivalis|WMB1870-13|RVcoll.11-J447||Italy|658[0n]
Erebia nivalis|EULEP3898-16|RVcoll15H274||Switzerland|658[0n]
Erebia nivalis|EULEP3899-16|RVcoll15H275||Switzerland|658[0n]
Erebia nivalis|EULEP3900-16|RVcoll15I162||Austria|658[0n]
Erebia nivalis|EULEP3901-16|RVcoll15I163||Austria|658[0n]
Erebia calcarius|EULEP3768-16|RVcoll15I019||Italy|658[0n]
Erebia calcarius|EULEP3771-16|RVcoll15P046||Slovenia|658[0n]
Erebia neleus|EULEP3772-16|RVcoll15P087||Macedonia|658[0n]
Erebia nivalis|EULEP3775-16|RVcoll15I346||Austria|658[0n]
Erebia arvernensis|EULEP5874-18|RVcoll15O510||Spain|658[0n]
Erebia nivalis|EULEP656-15|RVcoll.11-J450||Italy|658[0n]
Erebia neleus|EULEP918-15|RVcoll.14-C786||Bulgaria|658[0n]
Erebia arvernensis|BIBSA391-15|RVcoll.14-I059||Italy|658[0n]
Erebia cassioides|PHLAA139-09|TLMF Lep 00179||Italy|621[0n]
Erebia tyndarus|EULEP3999-16|RVcoll15G402||Switzerland|625[0n]
Erebia arvernensis|EULEP3778-16|RVcoll15J424||France|634[0n]
Erebia arvernensis|WMB1664-13|RVcoll.12-P213||France|634[0n]
Erebia arvernensis|EZSPM557-10|RVcoll.09-X053||Spain|614[0n]
Erebia nivalis|LEATG494-14|TLMF Lep 14281||Italy|600[0n]
Erebia arvernensis|EZSPM560-10|RVcoll.09-X026||Spain|632[0n]
Erebia tyndarus|LEATD145-13|TLMF Lep 12792||Italy|613[0n]
Erebia arvernensis|EZSPC889-10|RVcoll.09-T171||Spain|623[0n]
Erebia nivalis|HBOK145-08|OK520-51||Austria|609[0n]
Erebia calcarius|HBOK143-08|OK520-49||Slovenia|609[0n]
Erebia calcarius|HBOK144-08|OK520-50||Slovenia|609[0n]
Erebia cassioides|HBOK139-08|OK520-45||Austria|611[0n]
Erebia triarius|LASTS142-14|TLMF Lep 14594||Italy|658[0n]
Erebia triarius|EULEP2816-15|RVcoll.14-N900||Macedonia|658[0n]
Erebia triarius|EULEP2815-15|RVcoll.14-N899||Macedonia|658[0n]
Erebia triarius|EULEP2814-15|RVcoll.14-N898||Macedonia|658[0n]
Erebia triarius|EULEP3991-16|RVcoll15P078||Macedonia|658[0n]
Erebia triarius|EZSPC1060-10|RVcoll.08-L073||Spain|658[0n]
Erebia triarius|EZSPN812-09|RVcoll.08-L526||Spain|658[0n]
Erebia triarius|EZSPM258-09|RVcoll.08-R471||Spain|658[0n]
Erebia triarius|EZSPM220-09|RVcoll.08-R408||Spain|658[0n]
Erebia triarius|WMB5511-14|RVcoll.14-M308||France|658[0n]
Erebia triarius|EZSPN644-09|RVcoll.08-J781||Spain|658[0n]
Erebia triarius|EULEP3843-16|RVcoll14M304||Spain|658[0n]
Erebia triarius|WMB4041-14|RVcoll.12-Z359||Spain|658[0n]
Erebia triarius|WMB949-13|RVcoll.10-A651||France|658[0n]
Erebia triarius|WMB948-13|RVcoll.10-A650||France|658[0n]
Erebia triarius|WMB938-13|RVcoll.10-A602||France|658[0n]
Erebia triarius|WMB3589-14|RVcoll.11-E167||Spain|658[0n]
Erebia triarius|EZSPC1179-10|RVcoll.09-T151||Spain|658[0n]
Erebia triarius|EZSPC1169-10|RVcoll.09-T079||Spain|658[0n]
Erebia triarius|EZSPC1052-10|RVcoll.08-J782||Spain|658[0n]
Erebia triarius|EZSPC1242-10|RVcoll.09-V754||Spain|658[0n]
Erebia triarius|EZSPC1372-10|RVcoll.09-X139||Spain|658[0n]
Erebia triarius|EZSPC1360-10|RVcoll.09-X001||Spain|658[0n]
Erebia triarius|EZSPM811-12|Rvcoll. 130211WX96||Spain|658[0n]
Erebia triarius|EZSPM224-09|RVcoll.08-R415||Spain|658[0n]
Erebia triarius|EZSPN276-09|RVcoll.08-J655||Spain|658[0n]
Erebia triarius|EZSPN859-09|RVcoll.08-L720||Spain|658[0n]
Erebia triarius|EZSPN653-09|RVcoll.08-J796||Spain|658[0n]
Erebia triarius|EZSPM013-09|RVcoll.08-M993||Andorra|658[0n]
Erebia triarius|EZSPN811-09|RVcoll.08-L524||Spain|658[0n]
Erebia triarius|BIBSA901-15|LD-3513||Italy|658[0n]
Erebia triarius|BIBSA896-15|LD-3508||Italy|658[0n]
Erebia triarius|EULEP2142-15|RVcoll.14-J996||Switzerland|658[0n]
Erebia triarius|BIBSA132-15|RVcoll.14-I147||Italy|658[0n]
Erebia triarius|BIBSA218-15|RVcoll.14-E006||Italy|658[0n]

Erebia triarius|EULEP2142-15|RVcoll.14-J996||Switzerland|658[0n]
Erebia triarius|BIBSA132-15|RVcoll.14-I147||Italy|658[0n]
Erebia triarius|BIBSA218-15|RVcoll.14-E006||Italy|658[0n]
Erebia triarius|EULEP3989-16|RVcoll15G557||Switzerland|636[0n]
Erebia triarius|LASTS141-14|TLMF Lep 14593||Italy|658[0n]
Erebia medusa|EULEP3911-16|RVcoll15Q020||Macedonia|658[0n]
Erebia medusa|EULEP3871-16|RVcoll15Q017||Macedonia|658[0n]
Erebia medusa|EULEP3867-16|RVcoll15I178||Austria|658[0n]
Erebia medusa|EULEP808-15|RVcoll.13-U392||Italy|658[0n]
Erebia medusa|BIBSA965-15|13-U400||Italy|658[0n]
Erebia medusa|LEASS500-17|TLMF Lep 22148||Austria|658[0n]
Erebia medusa|EULEP3868-16|RVcoll15I430||Austria|658[0n]
Erebia medusa|HBOK119-08|OK520-25||Austria|632[1n]
Erebia medusa|LEATG497-14|TLMF Lep 14284||Italy|658[0n]
Erebia medusa|WMB5014-14|RVcoll. 14-I561||Italy|658[0n]
Erebia medusa|EZROM947-08|RVcoll.08-M615||Romania|658[0n]
Erebia medusa|EZROM943-08|RVcoll.06-N019|Genit. examined|Romania|658[0n]
Erebia medusa|EULEP3863-16|RVcoll10B411||Bulgaria|658[0n]
Erebia medusa|EZROM626-08|RV-06-N015|Genit. examined|Romania|658[0n]
Erebia medusa|EULEP1069-15|RVcoll.14-E986||Serbia|658[0n]
Erebia medusa|EULEP3869-16|RVcoll15P080||Macedonia|658[0n]
Erebia medusa|EULEP1085-15|RVcoll.14-F037||Serbia|658[0n]
Erebia medusa|EULEP1150-15|RVcoll.14-F328||Serbia|658[0n]
Erebia medusa|EULEP1225-15|RVcoll.14-F528||Greece|658[0n]
Erebia medusa|EULEP539-15|RVcoll.08-H891|Genit. examined|Serbia|658[0n]
Erebia medusa|EULEP1891-15|RVcoll.14-B705||Albania|658[0n]
Erebia medusa|EULEP1924-15|RVcoll.14-B863||Bosnia and Herzegovina|658[0n]
Erebia medusa|EULEP2822-15|RVcoll.14-N906||Albania|658[0n]
Erebia medusa|EULEP2823-15|RVcoll.14-N907||Albania|658[0n]
Erebia medusa|EZRMN346-08|RVcoll.08-M625|Genit. examined|Romania|649[0n]
Erebia medusa|EZROM945-08|RVcoll.08-M341||Romania|658[0n]
Erebia medusa|ABOLD080-16|TLMF Lep 21158||Austria|658[0n]
Erebia medusa|PHLAW006-13|TLMF Lep 09803||Austria|658[0n]
Erebia medusa|EULEP3870-16|RVcoll15P081||Macedonia|658[0n]
Erebia polaris|EULEP4767-16|RVcoll16H131||Norway|658[0n]
Erebia medusa|BIBSA146-15|RVcoll.14-I158||Italy|658[0n]
Erebia medusa|EZROM162-08|RV-07-D282||Romania|658[0n]
Erebia medusa|EZROM946-08|RVcoll.08-M522||Romania|658[0n]
Erebia polaris|LEFIF128-10|MM10584||Finland|658[0n]
Erebia polaris|LEFIF127-10|MM10583||Finland|658[0n]
Erebia medusa|EULEP3864-16|RVcoll15H000||Switzerland|658[0n]
Erebia medusa|WMB5180-14|RVcoll.13-S602||Italy|658[0n]
Erebia polaris|LEFIJ553-10|MM17178||Finland|658[0n]
Erebia medusa|GWORA2484-09|BC ZSM Lep 30696||Germany|658[0n]
Erebia medusa|GWORA2483-09|BC ZSM Lep 30695||Germany|658[0n]
Erebia medusa|LON171-08|NHMO-06172||Norway|657[0n]
Erebia medusa|FBLMX271-11|BC ZSM Lep 50482||Germany|658[0n]
Erebia medusa|GWORR685-10|BC ZSM Lep 32181||Germany|658[0n]
Erebia medusa|WMB5117-14|RVcoll. 14-L248||Italy|658[0n]
Erebia medusa|EZROM581-08|RV-07-C306||Romania|658[0n]
Erebia medusa|GWORR681-10|BC ZSM Lep 32177||Germany|658[0n]
Erebia medusa|LEATF451-14|TLMF Lep 13763||Austria|658[0n]
Erebia medusa|EZROM942-08|RVcoll.06-K622||Romania|658[0n]
Erebia medusa|EZRMN339-08|RVcoll.08-H025|Genit. examined|Romania|658[0n]
Erebia medusa|EZROM944-08|RVcoll.07-C112||Romania|658[0n]
Erebia medusa|EZROM159-08|RV-06-K618||Romania|658[0n]
Erebia polaris|EULEP5091-16|RVcoll16H215||Norway|658[0n]
Erebia medusa|EULEP3865-16|RVcoll15H916||Italy|658[0n]
Erebia medusa|EULEP4819-16|RVcoll16H715||Belgium|658[0n]
Erebia medusa|EULEP1850-15|RVcoll.13-S792||Italy|658[0n]
Erebia medusa|EULEP2272-15|RVcoll.14-N458||Ukraine|658[0n]
Erebia polaris|EULEP2836-15|RVcoll.14-N920||Norway|658[0n]
Erebia polaris|EULEP2837-15|RVcoll.14-N921||Norway|658[0n]
Erebia medusa|BIBSA986-15|14-W574||Italy|658[0n]
Erebia medusa|GBLAD266-14|BC ZSM Lep 78837||Germany|658[0n]
Erebia medusa|GBLAB169-13|BC ZSM Lep 75795||Germany|658[0n]
Erebia medusa|EULEP2244-15|RVcoll.14-N426||Ukraine|658[0n]
Erebia medusa|OXB831-15|14-U794||Italy|658[0n]
Erebia medusa|OXB630-15|14-O019||Italy|658[0n]
Erebia medusa|EZROM627-08|RV-07-C150|Genit. examined|Romania|652[0n]
Erebia medusa|GWOSN714-11|BC ZSM Lep 52920|changed from aethiops to medusa (AS)|Austria|618...
Erebia medusa|LEATG496-14|TLMF Lep 14283||Italy|632[0n]
Erebia medusa|LEATD454-13|TLMF Lep 13101||Italy|658[0n]
Erebia medusa|FBLMT878-09|BC ZSM Lep 25438||Germany|658[0n]
Erebia medusa|PHLAB315-10|TLMF Lep 01115||Switzerland|658[0n]
Erebia medusa|PHLAB351-10|TLMF Lep 01151||Switzerland|658[0n]
Erebia medusa|EULEP2442-15|RVcoll.14-V247||Switzerland|658[0n]
Erebia medusa|GWOSZ210-11|BC ZSM Lep 41966||Italy|658[0n]
Erebia medusa|LEATG017-14|TLMF Lep 13804||Austria|658[0n]
Erebia medusa|PHLAC363-10|TLMF Lep 02398||Italy|658[0n]
Proterebia phegea|EULEP1688-15|RVcoll.14-H024||Greece|658[0n]
Proterebia phegea|EULEP1687-15|RVcoll.14-H018||Greece|658[0n]
Proterebia phegea|EULEP1679-15|RVcoll.14-G980||Greece|658[0n]
Proterebia phegea|EULEP1677-15|RVcoll.14-G970||Greece|658[0n]
Proterebia phegea|EULEP2510-15|RVcoll.14-V378||Ukraine|658[0n]
Proterebia phegea|EULEP2511-15|RVcoll.14-V379||Ukraine|658[0n]
Proterebia phegea|EULEP2512-15|RVcoll.14-V380||Ukraine|658[0n]
Proterebia phegea|EULEP2509-15|RVcoll.14-V377||Ukraine|647[0n]
Proterebia phegea|EULEP2077-15|RVcoll.14-J565||Ukraine|658[0n]
Proterebia phegea|EULEP477-14|KN00839||Russia|658[0n]
Proterebia phegea|EULEP476-14|KN00838||Russia|658[0n]
Proterebia phegea|EULEP478-14|KN00840||Russia|658[0n]
Proterebia phegea|EULEP4555-16|RVcoll14A949||Russia|658[0n]
Hyponephele lupina|EULEP1472-15|RVcoll.14-G239||Greece|658[0n]
Hyponephele lupina|EULEP2890-15|RVcoll.14-U843|Symi|Greece|658[0n]
Hyponephele lupina|EULEP1543-15|RVcoll.14-G512||Greece|641[0n]
Hyponephele lupina|EULEP1540-15|RVcoll.14-G507||Greece|658[0n]
Hyponephele lupina|EULEP2891-15|RVcoll.14-U844|Symi|Greece|658[0n]
Hyponephele lupina|EULEP4927-16|RVcoll16J780||Russia|658[0n]
Hyponephele lupina|EULEP2892-15|RVcoll.14-U845|Thassos|Greece|658[0n]
Hyponephele lupina|EULEP745-15|RVcoll.12-N868|Lesvos|Greece|658[0n]
Hyponephele lupina|EULEP1475-15|RVcoll.14-G249||Greece|658[0n]
[illegible]

Hyponephele lupina|EULEP2892-15|RVcoll.14-U845|Thassos|Greece|658[0n]
Hyponephele lupina|EULEP745-15|RVcoll.12-N868|Lesvos|Greece|658[0n]
Hyponephele lupina|EULEP1475-15|RVcoll.14-G249||Greece|658[0n]
Hyponephele lupina|EULEP1293-15|RVcoll.14-F719||Greece|658[0n]
Hyponephele lupina|EULEP1839-15|RVcoll.14-J962||Macedonia|658[0n]
Hyponephele lupina|EULEP1478-15|RVcoll.14-G261||Greece|658[0n]
Hyponephele lupina|EULEP2887-15|RVcoll.14-U840||Macedonia|635[0n]
Hyponephele lupina|EULEP1840-15|RVcoll.14-J963||Macedonia|622[0n]
Hyponephele lupina|EULEP4926-16|RVcoll16J779||Russia|644[0n]
Hyponephele lupina|EULEP4925-16|RVcoll16J778||Russia|639[1n]
Hyponephele lupina|EULEP4089-16|RVcoll14O316||Greece|658[0n]
Hyponephele lupina|EULEP1462-15|RVcoll.14-G220||Greece|612[0n]
Hyponephele lupina|WMB4722-14|RVcoll.LD-3015|Sicily|Italy|658[0n]
Hyponephele lupina|BIBSA703-15|RVcoll. 12-R176|Sicily|Italy|658[0n]
Hyponephele lupina|WMB4716-14|RVcoll.LD-2974|Sicily|Italy|629[0n]
Hyponephele lupina|WMB2746-13|RVcoll.10-C625|Sicily|Italy|632[0n]
Hyponephele lupina|WMB4521-14|RVcoll.14-E221||Spain|611[0n]
Hyponephele lupina|EZSPM911-12|Rvcoll.12-M633||Spain|658[0n]
Hyponephele lupina|WMB4455-14|RVcoll.14-D322||Spain|658[0n]
Hyponephele lupina|EZSPC1109-10|RVcoll.08-M064||Spain|658[0n]
Hyponephele lupina|EZSPN980-09|RVcoll.08-M019||Spain|655[0n]
Hyponephele lupina|EZSPC1108-10|RVcoll.08-M046||Spain|658[0n]
Hyponephele lupina|EZSPM910-12|Rvcoll.12-M632||Spain|658[0n]
Hyponephele lupina|EZSPM662-12|RVcoll.11-I531||Spain|658[0n]
Hyponephele lupina|EZSPC1212-10|RVcoll.09-V504||Spain|658[0n]
Hyponephele lupina|EZSPN548-09|RVcoll.08-J052||Spain|658[0n]
Hyponephele lupina|EZSPC1093-10|RVcoll.08-L920.1||Spain|624[0n]
Hyponephele lupina|EZSPC1279-10|RVcoll.290909HG13||Spain|658[0n]
Hyponephele lupina|EZSPN990-09|RVcoll.08-M045||Spain|658[0n]
Hyponephele lupina|EZSPN1053-11|Rvcoll.08-L921||Spain|658[0n]
Hyponephele lupina|EZSPN1048-11|Rvcoll.08-L268||Spain|658[0n]
Hyponephele lupina|EZSPM661-12|RVcoll.11-I530||Spain|658[0n]
Hyponephele lycaon|EULEP2474-15|RVcoll.14-V328|Genit. examined|Ukraine|658[0n]
Hyponephele lycaon|WMB1699-13|RVcoll.12-P584||France|658[0n]
Hyponephele lycaon|WMB4388-14|RVcoll.14-B290||Portugal|658[0n]
Hyponephele lycaon|EZSPN942-09|RVcoll.08-L918||Spain|658[0n]
Hyponephele lycaon|EZSPN469-09|RVcoll.08-H694||Spain|658[0n]
Hyponephele lycaon|EZSPC442-09|RVcoll.08-P025||Spain|658[0n]
Hyponephele lycaon|EZSPC441-09|RVcoll.08-P024||Spain|658[0n]
Hyponephele lycaon|EZSPC440-09|RVcoll.08-P023|Genit. examined|Spain|658[0n]
Hyponephele lycaon|EZSPN509-09|RVcoll.08-H967||Spain|658[0n]
Hyponephele lycaon|EZSPN752-09|RVcoll.08-L219||Spain|658[0n]
Hyponephele lycaon|EZSPC1223-10|RVcoll.09-V557||Spain|658[0n]
Hyponephele lycaon|EZSPM463-09|Rvcoll.09-V538||Spain|656[0n]
Hyponephele lycaon|EZSPC1219-10|RVcoll.09-V539||Spain|658[0n]
Hyponephele lycaon|EZSPC1217-10|RVcoll.09-V525||Spain|658[0n]
Hyponephele lycaon|EZSPC1216-10|RVcoll.09-V524||Spain|658[0n]
Hyponephele lycaon|WMB3412-14|RVcoll.09-V943||Spain|658[0n]
Hyponephele lycaon|EZSPC1381-10|RVcoll.09-X517||Spain|658[0n]
Hyponephele lycaon|EZSPC1019-10|RVcoll.08-H695||Spain|658[0n]
Hyponephele lycaon|EZSPN751-09|RVcoll.08-L215||Spain|658[0n]
Hyponephele lycaon|EZSPN745-09|RVcoll.08-L173||Spain|658[0n]
Hyponephele lycaon|WMB3334-14|RVcoll.08-P779||Spain|658[0n]
Hyponephele lycaon|WMB3435-14|RVcoll.09-X534||Spain|658[0n]
Hyponephele lycaon|EZSPM293-09|RVcoll.08-J831||Spain|658[0n]
Hyponephele lycaon|EULEP1928-15|RVcoll.14-B887||Albania|658[0n]
Hyponephele lycaon|WMB3984-14|RVcoll.12-Q263||France|651[0n]
Hyponephele lycaon|EULEP4091-16|RVcoll15J553||France|658[1n]
Hyponephele lycaon|EULEP1491-15|RVcoll.14-G325||Greece|658[0n]
Hyponephele lycaon|EULEP1485-15|RVcoll.14-G296||Greece|613[2n]
Hyponephele lycaon|EULEP1531-15|RVcoll.14-G483||Greece|658[0n]
Hyponephele lycaon|EULEP1436-15|RVcoll.14-G126||Greece|658[0n]
Hyponephele lycaon|EULEP189-14|RVcoll.11-J887||Romania|658[0n]
Hyponephele lycaon|BIBSA931-15|16-A022||Italy|658[0n]
Hyponephele lycaon|EZROM341-08|RV-08-A019||Romania|658[0n]
Hyponephele lycaon|EZROM1024-08|RVcoll.08-M573||Romania|658[0n]
Hyponephele lycaon|EZROM1023-08|RVcoll.08-M564||Romania|658[0n]
Hyponephele lycaon|EULEP963-15|RVcoll.14-D026||Bulgaria|658[0n]
Hyponephele lycaon|EULEP852-15|RVcoll.14-C143||Greece|658[0n]
Hyponephele lycaon|LEFIK298-10|MM17873||Finland|658[0n]
Hyponephele lycaon|EULEP4092-16|RVcoll15G501||Switzerland|632[0n]
Hyponephele lycaon|EZSPC443-09|RVcoll.07-E640|Genit. examined|Spain|602[0n]
Hyponephele lycaon|LEFIL235-10|MM19235||Russia|658[0n]
Hyponephele lycaon|LEFIL234-10|MM19234||Russia|657[0n]
Hyponephele lycaon|EULEP1629-15|RVcoll.14-G731||Greece|658[0n]
Hyponephele lycaon|WMB3362-14|RVcoll.09-T166||Spain|658[0n]
Hyponephele lycaon|EZSPN960-09|RVcoll.08-L963||Spain|658[0n]
Hyponephele lycaon|EULEP2889-15|RVcoll.14-U842|Genit. examined|Russia|658[0n]
Hyponephele lycaon|EULEP4094-16|RVcoll15Q084||Russia|658[0n]
Hyponephele lycaon|EULEP4095-16|RVcoll15Q085||Russia|658[0n]
Hyponephele lycaon|EULEP4096-16|RVcoll15Q086||Russia|658[0n]
Hyponephele lycaon|EULEP2524-15|RVcoll.14-V397||Ukraine|658[0n]
Hyponephele lycaon|EULEP2886-15|RVcoll.14-U839|Lesvos|Greece|658[0n]
Hyponephele lycaon|EULEP4093-16|RVcoll15Q145||Russia|636[0n]
Hyponephele lycaon|EULEP5085-16|RVcoll16J377||Germany|636[0n]
Hyponephele lycaon|LEASS769-17|TLMF Lep 22417||Austria|637[0n]
Hyponephele lycaon|LEASS977-17|TLMF Lep 22530||Austria|658[0n]
Hyponephele lycaon|WMB1832-13|RVcoll.11-I804||France|632[0n]
Hyponephele lycaon|GWORZ035-10|BC ZSM Lep 30391||Italy|658[0n]
Hyponephele lycaon|WMB1612-13|RVcoll.12-O723||France|638[0n]
Hyponephele lycaon|EULEP4090-16|RVcoll15H109||Italy|658[0n]
Hyponephele lycaon|BIBSA1757-16|12-Q820||Italy|658[0n]
Hyponephele lycaon|WMB2849-13|RVcoll.12-Q816||Italy|632[0n]
Hyponephele lycaon|WMB4748-14|RVcoll.12-Q676.1||Italy|658[0n]
Hyponephele lycaon|WMB1880-13|RVcoll.11-J698|Sicily|Italy|658[0n]
Hyponephele lycaon|EULEP4866-16|RVcoll16H891||Poland|658[0n]
Hyponephele lycaon|BIBSA1553-16|11-H305||Italy|658[0n]
Hyponephele lycaon|BIBSA1732-16|LD-2579||Italy|658[0n]
Hyponephele lycaon|WMB808-13|RVcoll.07-E093||Italy|618[0n]
Hyponephele lycaon|WMB4280-14|RVcoll.14-A307||Italy|636[0n]
Hyponephele lycaon|WMB823-13|RVcoll.07-E190||Italy|613[0n]
Hyponephele lycaon|LEATG460-14|TLMF Lep 14247||Italy|658[0n]
Hyponephele lycaon|EULEP1557-15|RVcoll.14-G550||Macedonia|658[0n]

Hyponephele lycaon|WMB823-13|RVcoll.07-E190||Italy|613[0n]
Hyponephele lycaon|LEATG460-14|TLMF Lep 14247||Italy|658[0n]
Hyponephele lycaon|EULEP1557-15|RVcoll.14-G550||Macedonia|658[0n]
Hyponephele lycaon|PHLAF325-11|TLMF Lep 05495||Macedonia|658[0n]
Coenonympha glycerion|EZSPC771-10|RVcoll.08-L769||Spain|658[0n]
Coenonympha glycerion|EZSPM377-09|RVcoll.08-J867||Spain|658[0n]
Coenonympha glycerion|EZSPM308-09|RVcoll.08-J857||Spain|658[0n]
Coenonympha glycerion|EZSPC779-10|RVcoll.08-L859||Spain|658[0n]
Coenonympha glycerion|EZSPN650-09|RVcoll.08-J792||Spain|658[0n]
Coenonympha glycerion|EULEP5535-17|RVcoll14B597||Spain|658[0n]
Coenonympha glycerion|WMB3434-14|RVcoll.09-X510||Spain|658[0n]
Coenonympha glycerion|EZSPC772-10|RVcoll.08-L770||Spain|658[0n]
Coenonympha glycerion|EZSPN638-09|RVcoll.08-J762||Spain|658[0n]
Coenonympha glycerion|EZSPN596-09|RVcoll.08-J184||Portugal|658[0n]
Coenonympha glycerion|EZSPN595-09|RVcoll.08-J183||Portugal|658[0n]
Coenonympha glycerion|WMB4458-14|RVcoll.14-D326||Spain|658[0n]
Coenonympha glycerion|WMB3361-14|RVcoll.09-T164||Spain|658[0n]
Coenonympha glycerion|EZSPN871-09|RVcoll.08-L755||Spain|658[0n]
Coenonympha glycerion|EZSPN824-09|RVcoll.08-L633||Spain|658[0n]
Coenonympha glycerion|EULEP517-15|RVcoll.07-F528||Estonia|658[0n]
Coenonympha glycerion|FBLMV676-09|BC ZSM Lep 28656||Germany|658[1n]
Coenonympha glycerion|FBLMU488-09|BC ZSM Lep 27138||Germany|658[0n]
Coenonympha glycerion|FBLMU382-09|BC ZSM Lep 27032||Germany|658[0n]
Coenonympha glycerion|LEATF464-14|TLMF Lep 13776||Austria|658[0n]
Coenonympha glycerion|LEFIE850-10|MM10081||Finland|658[0n]
Coenonympha glycerion|OXB1296-15|Rvcoll_15-M161||France|658[0n]
Coenonympha glycerion|EULEP3664-16|RVcoll15G135||France|658[0n]
Coenonympha glycerion|OXB1295-15|Rvcoll_15-M160||France|626[0n]
Coenonympha glycerion|GBLAA375-14|BC ZSM Lep 80371||Germany|614[0n]
Coenonympha glycerion|PHLAH470-12|TLMF Lep 08289||Austria|615[0n]
Coenonympha glycerion|LEATF463-14|TLMF Lep 13775||Austria|658[0n]
Coenonympha glycerion|GWORT467-10|BC ZSM Lep 32153||Germany|658[0n]
Coenonympha glycerion|EZSPC794-10|RVcoll.08-P218||Spain|658[0n]
Coenonympha glycerion|EULEP5064-16|RVcoll16J126||Czech Republic|658[0n]
Coenonympha glycerion|GWORT464-10|BC ZSM Lep 32150||Germany|658[0n]
Coenonympha glycerion|EZROM870-08|RVcoll.08-M277||Romania|658[0n]
Coenonympha glycerion|EZROM108-08|RV-07-C955||Romania|658[0n]
Coenonympha glycerion|EZSPN251-09|RVcoll.07-W148||Spain|658[0n]
Coenonympha glycerion|EZSPC352-09|RVcoll.07-C497||Spain|658[0n]
Coenonympha glycerion|EZSPC353-09|RVcoll.07-C682||Spain|658[0n]
Coenonympha glycerion|EZSPC930-10|RVcoll.09-X004||Spain|658[0n]
Coenonympha glycerion|EZSPC932-10|RVcoll.09-X010||Spain|658[0n]
Coenonympha glycerion|EZSPC707-10|RVcoll.07-W146||Spain|658[0n]
Coenonympha glycerion|EZSPC708-10|RVcoll.07-W147||Spain|658[0n]
Coenonympha glycerion|WMB2676-13|RVcoll.10-B877||France|658[0n]
Coenonympha glycerion|EULEP5047-16|RVcoll16J043||Slovakia|658[0n]
Coenonympha glycerion|EULEP5877-18|RVcoll15O514||Spain|658[0n]
Coenonympha glycerion|EULEP1054-15|RVcoll.14-E936||Serbia|658[0n]
Coenonympha glycerion|EULEP1925-15|RVcoll.14-B865||Bosnia and Herzegovina|658[0n]
Coenonympha glycerion|EULEP1996-15|RVcoll.14-I767||Poland|658[0n]
Coenonympha glycerion|BIBSA888-15|LD-3500||Italy|658[0n]
Coenonympha glycerion|BIBSA889-15|LD-3501||Italy|658[0n]
Coenonympha glycerion|BIBSA899-15|LD-3511||Italy|658[0n]
Coenonympha glycerion|BIBSA900-15|LD-3512||Italy|658[0n]
Coenonympha glycerion|WMB2664-13|RVcoll.10-B792||France|613[0n]
Coenonympha glycerion|EZROM106-08|RV-06-K649||Romania|635[1n]
Coenonympha glycerion|PHLAA641-09|TLMF Lep 00681||France|632[0n]
Coenonympha glycerion|ABOLD428-16|TLMF Lep 21580||Austria|658[0n]
Coenonympha glycerion|PHLAB1222-10|TLMF Lep 02022||Italy|658[0n]
Coenonympha glycerion|PHLAA642-09|TLMF Lep 00682||France|658[0n]
Coenonympha glycerion|EZROM867-08|RVcoll.08-M217||Romania|658[0n]
Coenonympha glycerion|EZROM616-08|RV-07-D906||Romania|658[0n]
Coenonympha glycerion|LEFIG385-10|MM14398||Finland|658[0n]
Coenonympha glycerion|EZROM107-08|RV-07-D191|Genit. examined|Romania|658[0n]
Coenonympha glycerion|EULEP385-14|MM23870||Finland|658[0n]
Coenonympha glycerion|EULEP1139-15|RVcoll.14-F298||Serbia|658[0n]
Coenonympha glycerion|EULEP973-15|RVcoll.14-D066||Bulgaria|658[0n]
Coenonympha glycerion|EULEP1970-15|RVcoll.14-H834||Serbia|658[0n]
Coenonympha glycerion|EULEP4889-16|RVcoll16J581||Moldova|644[0n]
Coenonympha glycerion|EULEP4868-16|RVcoll16H901||Poland|639[0n]
Coenonympha glycerion|EULEP3665-16|RVcoll15Q081||Russia|658[0n]
Coenonympha glycerion|EZROM871-08|RVcoll.08-M381||Romania|658[0n]
Coenonympha glycerion|EZROM869-08|RVcoll.08-M240||Romania|658[0n]
Coenonympha glycerion|EZROM868-08|RVcoll.08-M221||Romania|658[0n]
Coenonympha glycerion|LEFID313-10|MM06198||Finland|658[0n]
Coenonympha glycerion|ABOLD069-16|TLMF Lep 21147||Austria|658[0n]
Coenonympha dorus|BCLEP121-17|LEP-SS-00572||Italy|658[0n]
Coenonympha dorus|WMB5259-14|RVcoll.14-A290||Italy|658[0n]
Coenonympha dorus|WMB1825-13|RVcoll.11-I695||France|658[0n]
Coenonympha dorus|WMB3503-14|RVcoll.10-B756||France|658[0n]
Coenonympha dorus|WMB283-11|RVcoll.10-A677||France|658[0n]
Coenonympha dorus|EZSPN1037-11|Rvcoll.07-F014||Spain|658[0n]
Coenonympha dorus|EZSPN807-09|RVcoll.08-L472||Spain|634[0n]
Coenonympha dorus|WMB4517-14|RVcoll.14-E214||Spain|658[0n]
Coenonympha dorus|WMB3577-14|RVcoll.11-E004||Spain|658[0n]
Coenonympha dorus|WMB4371-14|RVcoll.14-B151||Portugal|658[0n]
Coenonympha dorus|EULEP5646-17|RVcoll15F193||Portugal|658[0n]
Coenonympha dorus|EULEP5645-17|RVcoll15F192||Portugal|658[0n]
Coenonympha dorus|EZSPM458-09|Rvcoll.09-V508||Spain|621[0n]
Coenonympha dorus|WMB3395-14|RVcoll.09-V580||Spain|658[0n]
Coenonympha dorus|EZSPM457-09|Rvcoll.09-V429||Spain|658[0n]
Coenonympha dorus|EZSPM598-12|RVcoll.10-C392||Spain|658[0n]
Coenonympha dorus|EZSPN939-09|RVcoll.08-L908||Spain|658[0n]
Coenonympha dorus|EZSPM596-12|RVcoll.10-C389||Spain|658[0n]
Coenonympha dorus|EZSPN1118-11|Rvcoll.10-C317||Spain|658[0n]
Coenonympha dorus|EZSPN594-09|RVcoll.08-J181||Portugal|654[0n]
Coenonympha dorus|EZSPN1120-11|Rvcoll.10-C319||Spain|658[0n]
Coenonympha dorus|EZSPM904-12|Rvcoll.12-M626||Spain|658[0n]
Coenonympha dorus|EZSPM018-09|RVcoll.08-P018||Spain|658[0n]
Coenonympha dorus|EZSPC1380-10|RVcoll.09-X516||Spain|658[0n]
Coenonympha dorus|EZSPC987-10|RVcoll.07-C696||Spain|658[0n]
Coenonympha dorus|EZSPC1034-10|RVcoll.08-J192||Portugal|658[0n]
Coenonympha dorus|WMB3290-14|RVcoll.08-M948||Spain|658[0n]

Coenonympha dorus|EZSPC1[illegible]-10|RVcoll.09-[illegible]||Spain|658[0n]
Coenonympha dorus|EZSPC987-10|RVcoll.07-C696||Spain|658[0n]
Coenonympha dorus|EZSPC1034-10|RVcoll.08-J192||Portugal|658[0n]
Coenonympha dorus|WMB3290-14|RVcoll.08-M948||Spain|658[0n]
Coenonympha dorus|EZSPN549-09|RVcoll.08-J054||Spain|629[0n]
Coenonympha dorus|EZSPM597-12|RVcoll.10-C390||Spain|658[0n]
Coenonympha dorus|EZSPM595-12|RVcoll.10-C388||Spain|658[0n]
Coenonympha dorus|EZSPN201-09|RVcoll.07-C694||Spain|658[0n]
Coenonympha dorus|EZSPN762-09|RVcoll.08-L243||Spain|658[0n]
Coenonympha rhodopensis|EULEP3682-16|RVcoll13U065||Italy|658[0n]
Coenonympha rhodopensis|LEATJ1206-16|TLMF Lep 19529||Italy|658[0n]
Coenonympha tullia|EULEP4721-16|RVcoll16B370||Sweden|658[0n]
Coenonympha tullia|EULEP401-14|MM23886||Finland|658[0n]
Coenonympha tullia|EULEP400-14|MM23885||Finland|658[0n]
Coenonympha tullia|LEFIC021-10|MM03232||Finland|658[0n]
Coenonympha tullia|LEFID529-10|MM06510||Finland|658[0n]
Coenonympha rhodopensis|EULEP130-14|RVcoll.10-B420||Bulgaria|658[0n]
Coenonympha rhodopensis|EULEP1649-15|RVcoll.14-F564||Greece|614[0n]
Coenonympha rhodopensis|EULEP902-15|RVcoll.14-C734||Bulgaria|658[0n]
Coenonympha rhodopensis|EULEP851-15|RVcoll.14-C141||Greece|658[0n]
Coenonympha rhodopensis|EULEP129-14|RVcoll.10-B415||Bulgaria|658[0n]
Coenonympha rhodopensis|EULEP1084-15|RVcoll.14-F036||Serbia|658[0n]
Coenonympha rhodopensis|EULEP2811-15|RVcoll.14-N895||Macedonia|658[0n]
Coenonympha rhodopensis|EULEP1083-15|RVcoll.14-F035||Serbia|628[0n]
Coenonympha rhodopensis|EULEP128-14|RVcoll.10-A395||Bulgaria|658[0n]
Coenonympha rhodopensis|EULEP815-15|RVcoll.14-A257||Italy|658[0n]
Coenonympha rhodopensis|EULEP820-15|RVcoll.14-A361||Italy|658[0n]
Coenonympha rhodopensis|EULEP3684-16|RVcoll15O030||Italy|658[0n]
Coenonympha rhodopensis|EULEP3683-16|RVcoll15O029||Italy|658[0n]
Coenonympha rhodopensis|LEATJ1207-16|TLMF Lep 19530||Italy|658[0n]
Coenonympha tullia|OXB1502-16|RVcoll14B651||United Kingdom|658[0n]
Coenonympha tullia|OXB1500-16|RVcoll14B653||United Kingdom|658[0n]
Coenonympha tullia|OXB1498-16|RVcoll14B655||United Kingdom|658[0n]
Coenonympha tullia|OXB200-15|OXB-TGS-153||United Kingdom|658[0n]
Coenonympha tullia|OXB1507-16|RVcoll14B639||United Kingdom|658[0n]
Coenonympha tullia|OXB1506-16|RVcoll14B640||United Kingdom|658[0n]
Coenonympha tullia|OXB1505-16|RVcoll14B641||United Kingdom|658[0n]
Coenonympha tullia|OXB1503-16|RVcoll14B649||United Kingdom|658[0n]
Coenonympha tullia|OXB1501-16|RVcoll14B652||United Kingdom|658[0n]
Coenonympha tullia|OXB1499-16|RVcoll14B654||United Kingdom|658[0n]
Coenonympha tullia|OXB1496-16|RVcoll14B665||United Kingdom|658[0n]
Coenonympha tullia|OXB1495-16|RVcoll14B666||United Kingdom|658[0n]
Coenonympha amaryllis|EULEP404-14|KN00766||Russia|658[0n]
Coenonympha amaryllis|EULEP403-14|KN00765||Russia|658[0n]
Coenonympha tullia|WMB4443-14|RVcoll.14-B650||United Kingdom|658[0n]
Coenonympha tullia|WMB4442-14|RVcoll.14-B648||United Kingdom|658[0n]
Coenonympha tullia|WMB4441-14|RVcoll.14-B638||United Kingdom|658[0n]
Coenonympha rhodopensis|EZROM883-08|RVcoll.08-M622||Romania|658[0n]
Coenonympha rhodopensis|EZROM881-08|RVcoll.08-M620||Romania|658[0n]
Coenonympha rhodopensis|EZROM880-08|RVcoll.08-M619|Genit. examined|Romania|658[0n]
Coenonympha rhodopensis|EULEP2750-15|RVcoll.14-N834||Albania|658[0n]
Coenonympha rhodopensis|EULEP2749-15|RVcoll.14-N833||Albania|658[0n]
Coenonympha rhodopensis|EZROM884-08|RVcoll.08-M638|Genit. examined|Romania|658[0n]
Coenonympha rhodopensis|EZROM882-08|RVcoll.08-M621|Genit. examined|Romania|658[0n]
Coenonympha tullia|OXB1504-16|RVcoll14B647||United Kingdom|658[0n]
Coenonympha rhodopensis|EULEP1913-15|RVcoll.14-B811||Bosnia and Herzegovina|658[0n]
Coenonympha rhodopensis|EULEP1922-15|RVcoll.14-B854||Bosnia and Herzegovina|658[0n]
Coenonympha rhodopensis|PHLAF417-11|TLMF Lep 05587||Macedonia|658[0n]
Coenonympha rhodopensis|PHLAF416-11|TLMF Lep 05586||Macedonia|658[0n]
Coenonympha tullia|EULEP2716-15|RVcoll.14-N800||Bosnia and Herzegovina|658[0n]
Coenonympha tullia|EULEP2715-15|RVcoll.14-N799||Bosnia and Herzegovina|658[0n]
Coenonympha tullia|EULEP3685-16|RVcoll14O320||Bosnia and Herzegovina|658[0n]
Coenonympha tullia|EULEP758-15|RVcoll.12-R656||Romania|658[0n]
Coenonympha tullia|EULEP759-15|RVcoll.12-R657||Romania|658[0n]
Coenonympha tullia|EULEP4870-16|RVcoll16I284||Poland|658[0n]
Coenonympha tullia|LEATI050-15|TLMF Lep 17435||Austria|658[1n]
Coenonympha tullia|EZRMN359-08|RVcoll.08-M726||Romania|658[0n]
Coenonympha tullia|EZRMN360-08|RVcoll.08-M727||Romania|658[0n]
Coenonympha tullia|EZRMN361-08|RVcoll.08-M728||Romania|658[0n]
Coenonympha tullia|EZROM114-08|RV-06-N023|Genit. examined|Romania|658[0n]
Coenonympha tullia|EULEP3686-16|RVcoll15G048||France|658[0n]
Coenonympha tullia|EULEP3687-16|RVcoll15G070||France|658[0n]
Coenonympha tullia|EULEP3688-16|RVcoll15G081||France|658[0n]
Coenonympha tullia|EULEP1909-15|RVcoll.14-B806||Bosnia and Herzegovina|658[0n]
Coenonympha tullia|EULEP1910-15|RVcoll.14-B807||Bosnia and Herzegovina|658[0n]
Coenonympha tullia|GBLAB768-13|BC ZSM Lep 75539||Germany|658[0n]
Coenonympha tullia|ABOLD600-17|TLMF Lep 21676||Austria|658[0n]
Coenonympha tullia|EULEP3689-16|RVcoll15G083||France|637[0n]
Coenonympha tullia|GWORK321-09|BC ZSM Lep 21556||Germany|640[0n]
Coenonympha tullia|FBLMU482-09|BC ZSM Lep 27132||Germany|658[0n]
Coenonympha tullia|LEFIG583-10|MM14772||Finland|658[0n]
Coenonympha tullia|GWORT465-10|BC ZSM Lep 32151||Germany|658[0n]
Coenonympha tullia|LEATI051-15|TLMF Lep 17436||Austria|658[0n]
Coenonympha tullia|LEATJ1252-16|TLMF Lep 19575||Austria|658[0n]
Coenonympha tullia|LEATA404-13|TLMF Lep 10011||Austria|658[0n]
Coenonympha tullia|LEATJ1253-16|TLMF Lep 19576||Austria|658[0n]
Coenonympha corinna|WMB2274-13|RVcoll.LD-3383|Giannutri|Italy|658[0n]
Coenonympha corinna|WMB2602-13|RVcoll.12-R526|Capraia|Italy|658[0n]
Coenonympha corinna|WMB2247-13|RVcoll.12-R383|Capraia|Italy|658[0n]
Coenonympha corinna|WMB2246-13|RVcoll.12-R382|Capraia|Italy|658[0n]
Coenonympha corinna|WMB2245-13|RVcoll.12-R381|Capraia|Italy|658[0n]
Coenonympha corinna|WMB3019-14|RVcoll.12-R385|Capraia|Italy|658[0n]
Coenonympha corinna|WMB3018-14|RVcoll.12-R384|Capraia|Italy|658[0n]
Coenonympha corinna|WMB2619-13|RVcoll.12-O153|Corsica|France|658[0n]
Coenonympha corinna|WMB2905-14|RVcoll.11-E790|Corsica|France|621[0n]
Coenonympha corinna|BIBSA1273-15|11-E934|Corsica|France|658[0n]
Coenonympha corinna|WMB682-12|RVcoll.11-E901|Corsica|France|658[0n]
Coenonympha corinna|WMB362-11|RVcoll.11-E962|Corsica|France|658[0n]
Coenonympha corinna|WMB345-11|RVcoll.11-E733|Corsica|France|658[0n]
Coenonympha corinna|WMB2903-14|RVcoll.11-E757|Corsica|France|658[0n]
Coenonympha corinna|WMB1550-13|RVcoll.12-O327|Caprera|Italy|658[0n]
Coenonympha corinna|WMB2999-14|RVcoll.12-O416|San Pietro|Italy|658[0n]
Coenonympha corinna|WMB1567-13|RVcoll.12-O496|Sardinia|Italy|658[0n]

Coenonympha corinna|WMB1550-13|RVcoll.12-O327|Caprera|Italy|658[0n]
Coenonympha corinna|WMB2999-14|RVcoll.12-O416|San Pietro|Italy|658[0n]
Coenonympha corinna|WMB1567-13|RVcoll.12-O496|Sardinia|Italy|658[0n]
Coenonympha corinna|WMB1562-13|RVcoll.12-O414|San Pietro|Italy|658[0n]
Coenonympha corinna|WMB2998-14|RVcoll.12-O415|San Pietro|Italy|658[0n]
Coenonympha corinna|WMB1541-13|RVcoll.12-O288|La Maddalena|Italy|658[0n]
Coenonympha corinna|WMB332-11|RVcoll.11-E637|Spargi|Italy|658[0n]
Coenonympha corinna|WMB329-11|RVcoll.11-E625|Santa Maria|Italy|658[0n]
Coenonympha corinna|WMB1542-13|RVcoll.12-O289|La Maddalena|Italy|658[0n]
Coenonympha corinna|WMB3080-14|RVcoll.14-D947|Santa Maria|Italy|658[0n]
Coenonympha corinna|WMB2899-14|RVcoll.11-E627|Santa Maria|Italy|658[0n]
Coenonympha corinna|WMB2898-14|RVcoll.11-E626.1|Santa Maria|Italy|658[0n]
Coenonympha corinna|BIBSA1326-15|12-Q483|Sardinia|Italy|658[0n]
Coenonympha corinna|WMB6059-18|RVcoll12O542|Sardinia|Italy|658[0n]
Coenonympha corinna|WMB2834-13|RVcoll.12-O563|Sardinia|Italy|658[0n]
Coenonympha corinna|WMB242-11|RVcoll.09-T599|Sardinia|Italy|658[0n]
Coenonympha corinna|OXB894-15|15-A538|Elba|Italy|658[2n]
Coenonympha corinna|WMB4471-14|RVcoll.14-D902||Italy|658[0n]
Coenonympha corinna|EULEP5698-17|RVcoll16L041||Italy|658[0n]
Coenonympha corinna|WMB658-11|RVcoll.11-I339|Argentario|Italy|658[0n]
Coenonympha corinna|WMB725-12|RVcoll.11-I368|Argentario|Italy|658[0n]
Coenonympha corinna|WMB6058-18|RVcoll08R613||Italy|658[0n]
Coenonympha corinna|WMB4479-14|RVcoll.14-D920||Italy|658[0n]
Coenonympha corinna|WMB4478-14|RVcoll.14-D919||Italy|658[0n]
Coenonympha corinna|WMB4470-14|RVcoll.14-D901||Italy|658[0n]
Coenonympha corinna|WMB4469-14|RVcoll.14-D900||Italy|658[0n]
Coenonympha corinna|WMB2594-13|RVcoll.12-R330|Elba|Italy|658[0n]
Coenonympha corinna|WMB2725-13|RVcoll.10-C572||Italy|658[0n]
Coenonympha corinna|WMB015-11|RVcoll.LD-221|Elba|Italy|658[0n]
Coenonympha corinna|WMB2875-14|RVcoll.10-C573|Argentario|Italy|658[0n]
Coenonympha corinna|WMB4477-14|RVcoll.14-D918||Italy|658[0n]
Coenonympha corinna|WMB6057-18|RVcoll08R612||Italy|658[0n]
Coenonympha corinna|WMB195-11|RVcoll.06-V799|Elba|Italy|658[0n]
Coenonympha hero|LON927-12|NHMO Lep09104||Norway|658[0n]
Coenonympha hero|EULEP4730-16|RVcoll16G425||Sweden|658[0n]
Coenonympha hero|EULEP5167-17|RVcoll16G604||Sweden|658[0n]
Coenonympha hero|GWORT466-10|BC ZSM Lep 32152||Germany|622[1n]
Coenonympha hero|EULEP479-14|KN00841||Russia|658[0n]
Coenonympha hero|EULEP2243-15|RVcoll.14-N425||Ukraine|658[0n]
Coenonympha hero|EULEP2719-15|RVcoll.14-N803||France|658[0n]
Coenonympha hero|EULEP2720-15|RVcoll.14-N804||Estonia|658[0n]
Coenonympha hero|GBLAD461-14|BC ZSM Lep 77987||Germany|658[0n]
Coenonympha hero|EULEP2717-15|RVcoll.14-N801||France|617[0n]
Coenonympha hero|EULEP4864-16|RVcoll16H872||Poland|640[0n]
Coenonympha hero|EULEP3666-16|RVcoll10A787||Estonia|658[0n]
Coenonympha hero|FBLMT911-09|BC ZSM Lep 25471||Germany|658[0n]
Coenonympha hero|LEEUA525-11|MM20584||Latvia|658[0n]
Coenonympha hero|GWORT463-10|BC ZSM Lep 32149||Germany|658[0n]
Coenonympha hero|GWORK516-09|BC ZSM Lep 21846||Germany|658[0n]
Coenonympha hero|LEFIL047-10|MM19047||Estonia|658[0n]
Coenonympha hero|LEFIL046-10|MM19046||Russia|658[0n]
Coenonympha hero|LEFIL045-10|MM19045||Estonia|658[0n]
Coenonympha leander|EULEP2732-15|RVcoll.14-N816||Bulgaria|658[0n]
Coenonympha leander|EULEP2731-15|RVcoll.14-N815||Bulgaria|658[0n]
Coenonympha leander|EULEP2728-15|RVcoll.14-N812||Russia|658[0n]
Coenonympha leander|EULEP2729-15|RVcoll.14-N813||Russia|658[0n]
Coenonympha leander|EULEP2730-15|RVcoll.14-N814||Russia|658[0n]
Coenonympha leander|EULEP2727-15|RVcoll.14-N811||Russia|658[0n]
Coenonympha leander|EULEP1014-15|RVcoll.14-E659||Romania|614[0n]
Coenonympha leander|EULEP483-14|KN00845||Russia|658[0n]
Coenonympha leander|EZROM876-08|RVcoll.08-M255|Genit. examined|Romania|658[0n]
Coenonympha leander|EULEP2726-15|RVcoll.14-N810||Macedonia|658[0n]
Coenonympha leander|EULEP1088-15|RVcoll.14-F048||Serbia|658[0n]
Coenonympha leander|EULEP2724-15|RVcoll.14-N808||Macedonia|658[0n]
Coenonympha leander|EULEP2725-15|RVcoll.14-N809||Macedonia|658[0n]
Coenonympha leander|EULEP3668-16|RVcoll15G022||Greece|658[0n]
Coenonympha leander|EZROM874-08|RVcoll.08-M253||Romania|658[0n]
Coenonympha leander|EZROM873-08|RVcoll.08-M252||Romania|658[0n]
Coenonympha leander|EULEP1827-15|RVcoll.14-J948||Bulgaria|658[0n]
Coenonympha leander|EZROM875-08|RVcoll.08-M254||Romania|658[0n]
Coenonympha leander|EZROM872-08|RVcoll.08-M245||Romania|658[0n]
Coenonympha gardetta|EULEP641-15|RVcoll.11-J122||Switzerland|658[0n]
Coenonympha gardetta|EULEP3663-16|RVcoll15G418||Switzerland|632[0n]
Coenonympha gardetta|EULEP3652-16|RVcoll15H194||Switzerland|658[0n]
Coenonympha gardetta|EULEP3646-16|RVcoll15G745||Switzerland|658[0n]
Coenonympha gardetta|BIBSA376-15|RVcoll.14-I044||Italy|658[0n]
Coenonympha gardetta|BIBSA375-15|RVcoll.14-I043||Italy|658[0n]
Coenonympha gardetta|EULEP3650-16|RVcoll15G739||Switzerland|658[0n]
Coenonympha gardetta|EULEP3662-16|RVcoll15J520||France|658[0n]
Coenonympha arcania|WMB5258-14|RVcoll.14-A288||Italy|658[0n]
Coenonympha gardetta|BIBSA194-15|RVcoll.14-D982||Italy|658[0n]
Coenonympha gardetta|EULEP5394-17|RVcoll10C075||France|658[0n]
Coenonympha arcania|WMB5215-14|RVcoll.13-T963||Italy|658[0n]
Coenonympha arcania|WMB4945-14|RVcoll. 14-I492||Italy|658[0n]
Coenonympha arcania|WMB4862-14|RVcoll.14-I409||Italy|658[0n]
Coenonympha arcania|WMB800-13|RVcoll.07-E016||Italy|658[0n]
Coenonympha arcania|GWOSA778-10|BC ZSM Lep 36644||Germany|658[0n]
Coenonympha gardetta|PHLAI492-13|TLMF Lep 09054||Austria|658[0n]
Coenonympha gardetta|PHLSA729-11|TLMF Lep 06184||Austria|658[0n]
Coenonympha gardetta|LEATC102-13|TLMF Lep 11229||Italy|658[0n]
Coenonympha arcania|FBLMX208-11|BC ZSM Lep 50419||Germany|658[0n]
Coenonympha arcania|OXB594-15|14-N983||Italy|658[0n]
Coenonympha arcania|EULEP933-15|RVcoll.14-C875||Bulgaria|658[0n]
Coenonympha arcania|EZROM865-08|RVcoll.08-M457||Romania|658[0n]
Coenonympha arcania|EULEP1263-15|RVcoll.14-F655||Greece|658[0n]
Coenonympha arcania|EZROM103-08|RV-06-M941||Romania|658[0n]
Coenonympha arcania|EZROM863-08|RVcoll.07-D569||Romania|658[0n]
Coenonympha arcania|EULEP1522-15|RVcoll.14-G450||Greece|658[0n]
Coenonympha arcania|EZROM105-08|RV-07-C930||Romania|655[0n]
Coenonympha arcania|EULEP1036-15|RVcoll.14-E889||Serbia|658[0n]
Coenonympha arcania|EULEP1075-15|RVcoll.14-F011||Serbia|658[0n]
Coenonympha arcania|EULEP1100-15|RVcoll.14-F148||Serbia|658[0n]
Coenonympha arcania|EULEP1904-15|RVcoll.14-B794||Albania|658[0n]

[illegible]
Coenonympha arcania|EULEP1075-15|RVcoll.14-F011||Serbia|658[0n]
Coenonympha arcania|EULEP1100-15|RVcoll.14-F148||Serbia|658[0n]
Coenonympha arcania|EULEP1904-15|RVcoll.14-B794||Albania|658[0n]
Coenonympha arcania|EULEP1969-15|RVcoll.14-H833||Serbia|658[0n]
Coenonympha arcania|EULEP2352-15|RVcoll.14-V041||Ukraine|613[0n]
Coenonympha arcania|EULEP4815-16|RVcoll16H710||Belgium|637[0n]
Coenonympha arcania|EZROM104-08|RV-06-M954||Romania|644[0n]
Coenonympha arcania|EZROM102-08|RV-06-M843|Genit. examined|Romania|658[0n]
Coenonympha arcania|EZROM866-08|RVcoll.08-M568||Romania|658[0n]
Coenonympha arcania|EZROM864-08|RVcoll.08-M442||Romania|658[0n]
Coenonympha arcania|EZROM862-08|RVcoll.07-D491||Romania|658[0n]
Coenonympha arcania|LEATG073-14|TLMF Lep 13860||Austria|658[0n]
Coenonympha arcania|PHLAI562-13|TLMF Lep 09124||Austria|658[0n]
Coenonympha arcania|LEATJ1312-16|TLMF Lep 19635||Austria|658[0n]
Coenonympha arcania|LEATJ1247-16|TLMF Lep 19570||Austria|658[0n]
Coenonympha arcania|OXB635-15|14-O024||Italy|658[0n]
Coenonympha arcania|EULEP802-15|RVcoll.13-U203||Italy|658[0n]
Coenonympha arcania|BIBSA1094-15|15-L349||Italy|658[0n]
Coenonympha arcania|WMB2423-13|RVcoll.10-C734||Italy|658[0n]
Coenonympha arcania|BIBSA1839-17|RVcoll16C715||Italy|658[0n]
Coenonympha arcania|OXB940-15|15-A584||Italy|658[0n]
Coenonympha arcania|OXB311-15|RVcoll.14-N025||Italy|658[0n]
Coenonympha arcania|WMB1083-13|RVcoll.10-C732||Italy|633[0n]
Coenonympha arcania|WMB901-13|RVcoll.09-X857||Italy|658[0n]
Coenonympha arcania|EULEP3641-16|RVcoll15I566||Italy|658[0n]
Coenonympha arcania|EULEP3640-16|RVcoll15I072||Italy|658[0n]
Coenonympha gardetta|OXB738-15|13-U254||Italy|658[0n]
Coenonympha arcania|OXB757-15|13-U492||Italy|658[0n]
Coenonympha arcania|LEATH731-14|TLMF Lep 15943||Italy|634[0n]
Coenonympha arcania|LEATG513-14|TLMF Lep 14300||Italy|658[0n]
Coenonympha arcania|LEATG512-14|TLMF Lep 14299||Italy|658[0n]
Coenonympha arcania|ABOLD059-16|TLMF Lep 21137||Austria|658[0n]
Coenonympha gardetta|EULEP2089-15|RVcoll.14-J577||Italy|658[0n]
Coenonympha arcania|BIBSA1409-15|15-N016||Italy|658[0n]
Coenonympha arcania|OXB670-15|07-E015||Italy|658[0n]
Coenonympha arcania|BIBSA520-15|LEP-SS-00140||Italy|658[0n]
Coenonympha arcania|BIBSA519-15|LEP-SS-00139||Italy|658[0n]
Coenonympha arcania|BIBSA1372-15|15-M917||Italy|658[0n]
Coenonympha arcania|BIBSA1033-15|15-C192||Italy|658[0n]
Coenonympha arcania|WMB4882-14|RVcoll.14-I429||Italy|658[0n]
Coenonympha gardetta|FBLMX209-11|BC ZSM Lep 50420||Germany|658[0n]
Coenonympha arcania|WMB1616-13|RVcoll.12-O748||France|658[0n]
Coenonympha gardetta|EULEP2090-15|RVcoll.14-J578||Italy|617[0n]
Coenonympha gardetta|EULEP3654-16|RVcoll15H778||Switzerland|658[0n]
Coenonympha gardetta|EULEP3651-16|RVcoll15G768||Italy|658[0n]
Coenonympha gardetta|EULEP3648-16|RVcoll15G874||Switzerland|658[0n]
Coenonympha gardetta|EULEP3647-16|RVcoll15G796||Italy|658[0n]
Coenonympha gardetta|EULEP3644-16|RVcoll14V889||Switzerland|658[0n]
Coenonympha arcania|EULEP3642-16|RVcoll15I728||Austria|658[0n]
Coenonympha arcania|WMB5487-14|RVcoll.LD-2538||Italy|658[0n]
Coenonympha gardetta|WMB5120-14|RVcoll. 14-L251||Italy|658[0n]
Coenonympha gardetta|PHLAB322-10|TLMF Lep 01122||Switzerland|658[0n]
Coenonympha gardetta|EULEP3660-16|RVcoll15I862||Austria|658[0n]
Coenonympha arcania|EULEP3649-16|RVcoll15J227||Switzerland|658[0n]
Coenonympha arcania|BIBSA160-15|RVcoll.14-I172||Italy|645[0n]
Coenonympha gardetta|EULEP3657-16|RVcoll15I237||Austria|658[0n]
Coenonympha gardetta|EULEP3645-16|RVcoll15G612||Switzerland|658[0n]
Coenonympha gardetta|EULEP803-15|RVcoll.13-U244||Italy|658[0n]
Coenonympha gardetta|EULEP3656-16|RVcoll15H953||Italy|658[0n]
Coenonympha gardetta|EULEP3658-16|RVcoll15I378||Austria|658[0n]
Coenonympha gardetta|EULEP3659-16|RVcoll15I555||Italy|658[0n]
Coenonympha gardetta|EULEP2249-15|RVcoll.14-N432||Slovenia|658[0n]
Coenonympha gardetta|LEASS492-17|TLMF Lep 22140||Austria|642[0n]
Coenonympha gardetta|PHLAB374-10|TLMF Lep 01174||Switzerland|627[0n]
Coenonympha gardetta|PHLAA709-09|TLMF Lep 00749||Austria|658[0n]
Coenonympha gardetta|PHLAA710-09|TLMF Lep 00750||Austria|658[0n]
Coenonympha gardetta|EULEP2436-15|RVcoll.14-V238||Switzerland|658[0n]
Coenonympha gardetta|EULEP690-15|RVcoll.11-J958||Italy|658[0n]
Coenonympha gardetta|EULEP3661-16|RVcoll15J036||Switzerland|658[0n]
Coenonympha gardetta|EULEP3653-16|RVcoll15H423||Switzerland|658[0n]
Coenonympha gardetta|PHLAB298-10|TLMF Lep 01098||Switzerland|658[0n]
Coenonympha gardetta|LEATD302-13|TLMF Lep 12949||Italy|658[0n]
Coenonympha orientalis|EULEP1820-15|RVcoll.14-J940||Albania|658[0n]
Coenonympha orientalis|EULEP1829-15|RVcoll.14-J950||Albania|658[0n]
Coenonympha arcania|EZSPC255-09|RVcoll.08-P347||Spain|656[0n]
Coenonympha arcania|EZSPM138-09|RVcoll.08-P649||Spain|658[0n]
Coenonympha orientalis|EULEP1082-15|RVcoll.14-F030||Serbia|658[0n]
Coenonympha orientalis|EULEP1081-15|RVcoll.14-F029||Serbia|658[0n]
Coenonympha orientalis|EULEP1080-15|RVcoll.14-F027||Serbia|658[0n]
Coenonympha orientalis|EULEP2740-15|RVcoll.14-N824||Serbia|658[0n]
Coenonympha orientalis|EULEP2741-15|RVcoll.14-N825||Serbia|658[0n]
Coenonympha orientalis|EULEP1079-15|RVcoll.14-F025||Serbia|658[0n]
Coenonympha arcania|LON237-08|NHMO-07026||Norway|657[1n]
Coenonympha arcania|EZSPM088-09|RVcoll.08-P412||Spain|658[0n]
Coenonympha arcania|EULEP4729-16|RVcoll16G413||Sweden|658[0n]
Coenonympha arcania|EULEP1993-15|RVcoll.14-I724||France|658[0n]
Coenonympha arcania|WMB3653-14|RVcoll.11-I704||France|658[0n]
Coenonympha arcania|OXB1562-16|OXB-TGS-1280||France|658[0n]
Coenonympha arcania|OXB1563-16|OXB-TGS-1281||France|658[0n]
Coenonympha arcania|OXB1351-15|Rvcoll_15-M703||France|658[0n]
Coenonympha arcania|EULEP3643-16|RVcoll15G170||France|636[0n]
Coenonympha arcania|WMB270-11|RVcoll.10-A472||France|658[0n]
Coenonympha arcania|EZSPN722-09|RVcoll.08-L113||Spain|658[0n]
Coenonympha arcania|EZSPN905-09|RVcoll.08-L824||Spain|658[0n]
Coenonympha arcania|EZSPN906-09|RVcoll.08-L828||Spain|658[0n]
Coenonympha arcania|EZSPC199-09|RVcoll.06-G574||Spain|655[0n]
Coenonympha arcania|EZSPC222-09|RVcoll.08-L445||Spain|655[0n]
Coenonympha arcania|EZSPN480-09|RVcoll.08-H915||Spain|658[0n]
Coenonympha arcania|EZSPN506-09|RVcoll.08-H964||Spain|658[0n]
Coenonympha arcania|EZSPM266-09|RVcoll.08-R481||Spain|658[0n]
Coenonympha arcania|EZSPC1389-10|RVcoll.09-X571||Spain|658[0n]
Coenonympha arcania|WMB3308-14|RVcoll.08-P490||Spain|658[0n]
[illegible]

Coenonympha arcania|EZSPM266-09|RVcoll.08-K481||Spain|658[0n]
Coenonympha arcania|EZSPC1389-10|RVcoll.09-X571||Spain|658[0n]
Coenonympha arcania|WMB3308-14|RVcoll.08-P490||Spain|658[0n]
Coenonympha arcania|WMB4572-14|RVcoll.140410KV49||Spain|658[0n]
Coenonympha arcania|WMB5366-14|RVcoll.14-J739||France|658[0n]
Coenonympha arcania|WMB4962-14|RVcoll. 14-I509||Italy|658[0n]
Coenonympha arcania|EULEP3638-16|RVcoll15G041||France|658[0n]
Coenonympha arcania|EULEP3639-16|RVcoll15G843||Italy|658[0n]
Coenonympha arcania|BIBSA192-15|RVcoll.14-D980||Italy|658[0n]
Coenonympha arcania|WMB667-12|RVcoll.07-C638||France|658[0n]
Coenonympha arcania|BIBSA193-15|RVcoll.14-D981||Italy|658[0n]
Coenonympha arcania|OXB328-15|RVcoll.14-N042||Italy|658[0n]
Coenonympha arcania|BIBSA438-15|RVcoll.14-I106||Italy|637[0n]
Coenonympha arcania|EZSPC227-09|RVcoll.08-L495||Spain|645[0n]
Coenonympha arcania|EZSPM306-09|RVcoll.08-J853||Spain|614[0n]
Coenonympha arcania|EZSPC250-09|RVcoll.08-P224||Spain|648[0n]
Coenonympha arcania|LENOA1377-11|LN-BD1377||France|626[0n]
Coenonympha arcania|GBLAA1442-15|BC ZSM Lep 87233||Germany|658[0n]
Coenonympha arcania|GWORA2879-15|BC ZSM Lep 86147||Germany|638[0n]
Coenonympha arcania|EULEP586-15|RVcoll.10-A701||Estonia|658[0n]
Coenonympha arcania|EULEP4901-16|RVcoll16J604||Russia|658[0n]
Coenonympha arcania|LEEUA526-11|MM20585||Latvia|658[0n]
Coenonympha arcania|GWORA2481-09|BC ZSM Lep 30693||Germany|658[0n]
Coenonympha arcania|GWORT462-10|BC ZSM Lep 32148||Germany|658[0n]
Coenonympha gardetta|OXB618-15|14-O007||Italy|658[0n]
Coenonympha gardetta|EULEP3655-16|RVcoll15H844||Italy|658[0n]
Coenonympha gardetta|BIBSA1214-15|15-M341||Italy|658[0n]
Coenonympha gardetta|EULEP807-15|RVcoll.13-U356||Italy|658[0n]
Coenonympha gardetta|LEATF465-14|TLMF Lep 13777||Austria|658[0n]
Coenonympha phryne|EULEP2201-15|RVcoll.14-N361||Ukraine|658[0n]
Coenonympha phryne|EULEP2200-15|RVcoll.14-N360||Ukraine|658[0n]
Coenonympha phryne|EULEP475-14|KN00837||Russia|658[0n]
Coenonympha oedippus|EULEP4860-16|RVcoll16H841||Poland|658[0n]
Coenonympha oedippus|EULEP2238-15|RVcoll.14-N418||Ukraine|658[0n]
Coenonympha oedippus|EULEP2220-15|RVcoll.14-N398||Ukraine|658[0n]
Coenonympha oedippus|EULEP443-14|KN00805||Poland|658[1n]
Coenonympha oedippus|EULEP2734-15|RVcoll.14-N818||Poland|658[0n]
Coenonympha oedippus|LEASS739-17|TLMF Lep 22387||Austria|658[0n]
Coenonympha oedippus|LEASS740-17|TLMF Lep 22388||Austria|658[0n]
Coenonympha oedippus|ABOLD646-17|TLMF Lep 21722||Austria|658[0n]
Coenonympha oedippus|EULEP2219-15|RVcoll.14-N397||Ukraine|614[0n]
Coenonympha oedippus|EULEP2733-15|RVcoll.14-N817||Poland|658[0n]
Coenonympha oedippus|EULEP3669-16|RVcoll15I876||Liechtenstein|658[0n]
Coenonympha oedippus|PHLAF625-11|TLMF Lep 05795||Liechtenstein|658[0n]
Coenonympha oedippus|EULEP3670-16|RVcoll15O001||Italy|658[0n]
Coenonympha oedippus|BIBSA1817-17|RVcoll14I286||Italy|658[2n]
Coenonympha oedippus|EULEP2739-15|RVcoll.14-N823||France|658[0n]
Coenonympha oedippus|EULEP2738-15|RVcoll.14-N822||France|658[0n]
Coenonympha oedippus|BIBSA1808-17|RVcoll14I277||Italy|658[1n]
Coenonympha oedippus|BIBSA1812-17|RVcoll14I281||Italy|658[1n]
Coenonympha oedippus|BIBSA1818-17|RVcoll14I287||Italy|658[1n]
Coenonympha oedippus|EULEP3671-16|RVcoll15O002||Italy|658[0n]
Coenonympha oedippus|PHLSA390-11|TLMF Lep 05845||Italy|658[1n]
Coenonympha oedippus|FBLMU190-09|BC ZSM Lep 25700||Germany|658[0n]
Coenonympha oedippus|PHLAF624-11|TLMF Lep 05794||Liechtenstein|658[0n]
Coenonympha thyrsis|EULEP2300-15|RVcoll.14-N501|Crete|Greece|658[0n]
Coenonympha thyrsis|EULEP2299-15|RVcoll.14-N500|Crete|Greece|658[0n]
Coenonympha thyrsis|EULEP2301-15|RVcoll.14-N502|Crete|Greece|658[0n]
Coenonympha thyrsis|EULEP2297-15|RVcoll.14-N498|Crete|Greece|658[0n]
Coenonympha pamphilus|WMB2652-13|RVcoll.12-O385|Sardinia|Italy|658[0n]
Coenonympha pamphilus|OXB1249-15|Rvcoll_13-T741|Sardinia|Italy|658[0n]
Coenonympha pamphilus|BIBSA1280-15|12-O548|Sardinia|Italy|658[0n]
Coenonympha pamphilus|WMB310-11|RVcoll.11-E430|Sardinia|Italy|658[0n]
Coenonympha pamphilus|WMB2651-13|RVcoll.12-O367|Sardinia|Italy|658[0n]
Coenonympha pamphilus|WMB2650-13|RVcoll.12-O350|Sardinia|Italy|658[0n]
Coenonympha pamphilus|WMB1571-13|RVcoll.12-O508|Sardinia|Italy|658[0n]
Coenonympha pamphilus|WMB2895-14|RVcoll.11-E340|Sardinia. Genit. examined|Italy|658[0n]
Coenonympha pamphilus|EZSPC713-10|RVcoll.08-H112||Spain|658[0n]
Coenonympha pamphilus|EZSPM068-09|RVcoll.08-P304|Genit. examined|Spain|658[0n]
Coenonympha pamphilus|EZSPM067-09|RVcoll.08-P300||Spain|658[0n]
Coenonympha pamphilus|EZSPN303-09|RVcoll.08-H108||Spain|658[0n]
Coenonympha pamphilus|EZSPM044-09|RVcoll.08-P257|Genit. examined|Spain|658[0n]
Coenonympha pamphilus|WMB239-11|RVcoll.09-T591|Sardinia|Italy|658[0n]
Coenonympha pamphilus|WMB290-11|RVcoll.09-T596|Sardinia|Italy|658[0n]
Coenonympha pamphilus|EZSPN304-09|RVcoll.08-H109|Genit. examined|Spain|658[0n]
Coenonympha pamphilus|EZSPM1002-12|Rvcoll.12-L888||Spain|634[0n]
Coenonympha pamphilus|WMB044-11|RVcoll.LD-1687|Mallorca|Spain|615[0n]
Coenonympha pamphilus|BIBSA1525-16|LEP-SS-00370||Italy|611[2n]
Coenonympha pamphilus|EZSPM102-09|RVcoll.08-P434||Spain|658[0n]
Coenonympha pamphilus|WMB3678-14|RVcoll.11-J181||France|658[0n]
Coenonympha pamphilus|EULEP1180-15|RVcoll.14-F421||Bulgaria|658[0n]
Coenonympha pamphilus|EZSPM1014-12|Rvcoll.12-M763||Spain|658[0n]
Coenonympha pamphilus|WMB1921-13|RVcoll.11-H705|Levanzo|Italy|643[0n]
Coenonympha pamphilus|EZROM574-08|RV-07-C311||Romania|658[0n]
Coenonympha pamphilus|WMB339-11|RVcoll.11-E678||France|658[0n]
Coenonympha pamphilus|BIBSA1754-16|14-A705||Italy|658[0n]
Coenonympha pamphilus|EZROM111-08|RV-07-D118||Romania|658[0n]
Coenonympha pamphilus|EZSPN704-09|RVcoll.08-L050||Spain|658[0n]
Coenonympha pamphilus|WMB5058-14|RVcoll. 14-L189||Italy|658[0n]
Coenonympha pamphilus|EZROM112-08|RV-07-D208||Romania|658[0n]
Coenonympha pamphilus|WMB042-11|RVcoll.LD-1677|Elba|Italy|658[0n]
Coenonympha pamphilus|GBLAD273-14|BC ZSM Lep 78844||Germany|658[0n]
Coenonympha pamphilus|OXB329-15|RVcoll.14-N043||Italy|658[0n]
Coenonympha pamphilus|BIBSA339-15|RVcoll.14-I007||Italy|623[0n]
Coenonympha pamphilus|WMB4737-14|RVcoll.LD-3382||Italy|658[0n]
Coenonympha pamphilus|WMB2760-13|RVcoll.11-H709|Levanzo|Italy|658[0n]
Coenonympha pamphilus|OXB195-15|OXB-TGS-060||United Kingdom|658[0n]
Coenonympha pamphilus|OXB194-15|OXB-TGS-059||United Kingdom|658[0n]
Coenonympha pamphilus|EZSPN566-09|RVcoll.08-J107||Portugal|658[0n]
Coenonympha pamphilus|OXB1432-16|OXB-TGS-1206||United Kingdom|658[0n]
Coenonympha pamphilus|LON948-12|NHMO Lep2011.008||Norway|658[0n]
Coenonympha pamphilus|LEFIJ558-10|MM17183||Finland|658[0n]
Coenonympha pamphilus|OXB230-15|OXB-TGS-491||United Kingdom|656[0n]

Coenonympha pamphilus|LON948-12|NHMO Lep2011.008||Norway|658[0n]
Coenonympha pamphilus|LEFIJ558-10|MM17183||Finland|658[0n]
Coenonympha pamphilus|OXB230-15|OXB-TGS-491||United Kingdom|656[0n]
Coenonympha pamphilus|OXB281-15|OXB-TGS-964||United Kingdom|656[0n]
Coenonympha pamphilus|EZSPM245-09|RVcoll.08-R456||Spain|658[0n]
Coenonympha pamphilus|EZSPM244-09|RVcoll.08-R455||Spain|658[0n]
Coenonympha pamphilus|LON013-08|NHMO-06013||Norway|657[0n]
Coenonympha pamphilus|LON965-12|NHMO Lep2011.025||Norway|658[0n]
Coenonympha pamphilus|EZSPM324-09|RVcoll.08-J898||Spain|658[0n]
Coenonympha pamphilus|WMB3633-14|RVcoll.11-H010||Spain|658[0n]
Coenonympha pamphilus|WMB4021-14|RVcoll.12-R504||United Kingdom|658[0n]
Coenonympha pamphilus|WMB4031-14|RVcoll.12-Z160||Ireland|658[0n]
Coenonympha pamphilus|EULEP4737-16|RVcoll16G563||Sweden|658[0n]
Coenonympha pamphilus|EULEP545-15|RVcoll.08-L304||Denmark|658[0n]
Coenonympha pamphilus|EULEP844-15|RVcoll.14-C036||Sweden|658[0n]
Coenonympha pamphilus|OXB1431-16|OXB-TGS-1205||United Kingdom|658[0n]
Coenonympha pamphilus|OXB1433-16|OXB-TGS-1207||United Kingdom|658[0n]
Coenonympha pamphilus|OXB1434-16|OXB-TGS-1208||United Kingdom|658[0n]
Coenonympha pamphilus|EZSPC857-10|RVcoll.08-R457||Spain|658[0n]
Coenonympha pamphilus|OXB192-15|OXB-TGS-015||United Kingdom|658[0n]
Coenonympha pamphilus|OXB216-15|OXB-TGS-352||United Kingdom|658[0n]
Coenonympha pamphilus|WMB4035-14|RVcoll.12-Z183||Ireland|631[0n]
Coenonympha pamphilus|WMB3312-14|RVcoll.08-P495||Spain|620[0n]
Coenonympha pamphilus|LEFIE892-10|MM10171||Finland|649[0n]
Coenonympha pamphilus|EULEP3675-16|RVcoll15I018||Italy|658[0n]
Coenonympha pamphilus|WMB541-11|RVcoll.11-H704|Levanzo|Italy|658[0n]
Coenonympha pamphilus|EULEP1668-15|RVcoll.14-G895||Greece|658[0n]
Coenonympha pamphilus|OXB1213-15|Rvcoll_15-M639||France|658[0n]
Coenonympha pamphilus|ABOLD425-16|TLMF Lep 21577||Austria|658[0n]
Coenonympha pamphilus|ABOLD079-16|TLMF Lep 21157||Austria|658[0n]
Coenonympha pamphilus|LEFIG356-10|MM14354||Finland|656[0n]
Coenonympha pamphilus|EZSPC355-09|RVcoll.08-M716|Genit. examined|Spain|658[0n]
Coenonympha pamphilus|FBLMU483-09|BC ZSM Lep 27133||Germany|657[0n]
Coenonympha pamphilus|FBLMU484-09|BC ZSM Lep 27134||Germany|658[0n]
Coenonympha pamphilus|FBLMT883-09|BC ZSM Lep 25443||Germany|658[0n]
Coenonympha pamphilus|EZROM877-08|RVcoll.08-M228||Romania|658[0n]
Coenonympha pamphilus|EZROM878-08|RVcoll.08-M348||Romania|658[0n]
Coenonympha pamphilus|EZROM879-08|RVcoll.08-M365||Romania|658[0n]
Coenonympha pamphilus|EZROM109-08|RV-06-K637|Genit. examined|Romania|658[0n]
Coenonympha pamphilus|EZROM110-08|RV-06-M836|Genit. examined|Romania|658[0n]
Coenonympha pamphilus|EZROM113-08|RV-07-D212||Romania|658[0n]
Coenonympha pamphilus|EZSPC354-09|RVcoll.06-G663||Spain|658[0n]
Coenonympha pamphilus|EZSPC356-09|RVcoll.06-G527|Genit. examined|Spain|658[0n]
Coenonympha pamphilus|EZSPN378-09|RVcoll.08-H422|Genit. examined|Spain|658[0n]
Coenonympha pamphilus|EZSPN444-09|RVcoll.08-H630|Genit. examined|Spain|658[0n]
Coenonympha pamphilus|EZSPM969-12|Rvcoll.12-M723||Spain|658[0n]
Coenonympha pamphilus|EZSPM972-12|Rvcoll.12-M730||Spain|658[0n]
Coenonympha pamphilus|EZSPM1021-12|Rvcoll.12-M770||Spain|658[0n]
Coenonympha pamphilus|EZSPM1035-12|Rvcoll.12-M786||Spain|658[0n]
Coenonympha pamphilus|EZSPN541-09|RVcoll.08-J033|Genit. examined|Spain|658[0n]
Coenonympha pamphilus|EZSPC357-09|RVcoll.07-C669||Spain|658[0n]
Coenonympha pamphilus|EZSPM681-12|Rvcoll. 12-L282||Spain|658[0n]
Coenonympha pamphilus|EZSPM682-12|Rvcoll. 12-L283||Spain|658[0n]
Coenonympha pamphilus|EZSPC905-10|RVcoll.09-V595|Genit. examined|Spain|658[0n]
Coenonympha pamphilus|EZSPM920-12|Rvcoll.12-M642||Spain|658[0n]
Coenonympha pamphilus|EZSPM949-12|Rvcoll.12-M688||Spain|658[0n]
Coenonympha pamphilus|WMB158-11|RVcoll.11-D316|Sicily|Italy|658[0n]
Coenonympha pamphilus|WMB184-11|RVcoll.11-D486|Genit. examined|Italy|658[0n]
Coenonympha pamphilus|EZROM715-08|RV-07-C622||Spain|658[0n]
Coenonympha pamphilus|WMB041-11|RVcoll.LD-1676|Elba|Italy|658[0n]
Coenonympha pamphilus|WMB043-11|RVcoll.LD-1678|Elba|Italy|658[0n]
Coenonympha pamphilus|WMB045-11|RVcoll.LD-1709||Italy|658[0n]
Coenonympha pamphilus|WMB046-11|RVcoll.LD-1710||Italy|658[0n]
Coenonympha pamphilus|WMB047-11|RVcoll.LD-1711||Italy|658[0n]
Coenonympha pamphilus|WMB3425-14|RVcoll.09-X272||France|658[0n]
Coenonympha pamphilus|WMB3431-14|RVcoll.09-X282||France|658[0n]
Coenonympha pamphilus|WMB3694-14|RVcoll.11-J251||Spain|658[0n]
Coenonympha pamphilus|WMB3171-14|RVcoll.08-H524|Genit. examined|Spain|658[0n]
Coenonympha pamphilus|WMB3211-14|RVcoll.08-J364|Genit. examined|Spain|658[0n]
Coenonympha pamphilus|WMB3306-14|RVcoll.08-P476||Spain|658[0n]
Coenonympha pamphilus|WMB3470-14|RVcoll.10-A633||France|658[0n]
Coenonympha pamphilus|WMB3487-14|RVcoll.10-B668.1||France|658[0n]
Coenonympha pamphilus|WMB3670-14|RVcoll.11-I972||France|658[0n]
Coenonympha pamphilus|WMB3736-14|RVcoll.12-M653||Spain|658[0n]
Coenonympha pamphilus|WMB2722-13|RVcoll.10-C557||Italy|658[0n]
Coenonympha pamphilus|WMB1700-13|RVcoll.12-P600||France|658[0n]
Coenonympha pamphilus|WMB1888-13|RVcoll.11-J757||Italy|658[0n]
Coenonympha pamphilus|WMB1999-13|RVcoll.12-Q356||France|658[0n]
Coenonympha pamphilus|WMB3858-14|RVcoll.11-I220||Italy|658[0n]
Coenonympha pamphilus|WMB2354-13|RVcoll.12-Q890||Italy|658[0n]
Coenonympha pamphilus|WMB2355-13|RVcoll.12-Q891||Italy|658[0n]
Coenonympha pamphilus|WMB5961-17|RVcoll14E251|Malta|Malta|658[0n]
Coenonympha pamphilus|WMB5962-17|RVcoll14E255|Malta|Malta|658[0n]
Coenonympha pamphilus|WMB5963-17|RVcoll14E258|Malta|Malta|658[0n]
Coenonympha pamphilus|WMB5964-17|RVcoll14E259|Malta|Malta|658[0n]
Coenonympha pamphilus|WMB3816-14|RVcoll.07-E070||Italy|658[0n]
Coenonympha pamphilus|WMB2620-13|RVcoll.12-O154|Corsica|France|658[0n]
Coenonympha pamphilus|WMB2653-13|RVcoll.12-O245|Corsica|France|658[0n]
Coenonympha pamphilus|WMB2654-13|RVcoll.12-O186|Corsica|France|658[0n]
Coenonympha pamphilus|WMB4165-14|RVcoll.13-S724||Italy|658[0n]
Coenonympha pamphilus|WMB3875-14|RVcoll.11-Y084||Italy|658[0n]
Coenonympha pamphilus|WMB3919-14|RVcoll.12-P436||France|658[0n]
Coenonympha pamphilus|WMB3934-14|RVcoll.12-P538||France|658[0n]
Coenonympha pamphilus|WMB3954-14|RVcoll.12-P729||France|658[0n]
Coenonympha pamphilus|WMB3973-14|RVcoll.12-Q184||France|658[0n]
Coenonympha pamphilus|WMB4009-14|RVcoll.12-Q752||Italy|658[0n]
Coenonympha pamphilus|WMB4016-14|RVcoll.12-R210||Italy|658[0n]
Coenonympha pamphilus|WMB4298-14|RVcoll.14-A395||Italy|658[0n]
Coenonympha pamphilus|WMB4341-14|RVcoll.14-A733||Italy|658[0n]
Coenonympha pamphilus|WMB4389-14|RVcoll.14-B298||Portugal|658[0n]
Coenonympha pamphilus|WMB4407-14|RVcoll.14-B441||Portugal|658[0n]
Coenonympha pamphilus|WMB4481-14|RVcoll.14-D926||Italy|658[0n]
Coenonympha pamphilus|WMB4619-14|RVcoll.LD-2154||Italy|658[0n]

Coenonympha pamphilus|WMB43[illegible]-14|RVcoll.14-B29[illegible]||Portugal|658[0n]
Coenonympha pamphilus|WMB4407-14|RVcoll.14-B441||Portugal|658[0n]
Coenonympha pamphilus|WMB4481-14|RVcoll.14-D926||Italy|658[0n]
Coenonympha pamphilus|WMB4619-14|RVcoll.LD-2154||Italy|658[0n]
Coenonympha pamphilus|WMB4620-14|RVcoll.LD-2223||Italy|658[0n]
Coenonympha pamphilus|WMB4621-14|RVcoll.LD-2224||Italy|658[0n]
Coenonympha pamphilus|WMB358-11|RVcoll.11-E889|Genit. examined|France|658[0n]
Coenonympha pamphilus|WMB499-11|RVcoll.11-H555|Sicily|Italy|658[0n]
Coenonympha pamphilus|WMB509-11|RVcoll.11-H594|Sicily|Italy|658[0n]
Coenonympha pamphilus|WMB545-11|RVcoll.11-H733|Sicily|Italy|658[0n]
Coenonympha pamphilus|WMB552-11|RVcoll.11-H745|Sicily|Italy|658[0n]
Coenonympha pamphilus|WMB592-11|RVcoll.11-H921|Sicily|Italy|658[0n]
Coenonympha pamphilus|WMB624-11|RVcoll.11-I063|Sicily|Italy|658[0n]
Coenonympha pamphilus|WMB4755-14|RVcoll. 14-I302||Italy|658[0n]
Coenonympha pamphilus|WMB5367-14|RVcoll.14-J740||France|658[0n]
Coenonympha pamphilus|WMB4956-14|RVcoll. 14-I503||Italy|658[0n]
Coenonympha pamphilus|WMB5067-14|RVcoll. 14-L198||Italy|658[0n]
Coenonympha pamphilus|WMB5179-14|RVcoll.13-S530||Italy|658[0n]
Coenonympha pamphilus|WMB5196-14|RVcoll.13-T911||Italy|658[0n]
Coenonympha pamphilus|EULEP3672-16|RVcoll10B331||Bulgaria|658[0n]
Coenonympha pamphilus|EULEP3673-16|RVcoll10B455|Genit. examined|Croatia|658[0n]
Coenonympha pamphilus|EULEP3674-16|RVcoll15H900||Italy|658[0n]
Coenonympha pamphilus|EULEP3677-16|RVcoll15I897||Liechtenstein|658[0n]
Coenonympha pamphilus|EULEP3678-16|RVcoll15G095||France|658[0n]
Coenonympha pamphilus|EULEP3680-16|RVcoll15P002||Belarus|658[0n]
Coenonympha pamphilus|EULEP5070-16|RVcoll16J178||Czech Republic|658[0n]
Coenonympha pamphilus|EULEP4922-16|RVcoll16J773||Russia|658[0n]
Coenonympha pamphilus|EULEP4933-16|RVcoll16I012||Poland|658[0n]
Coenonympha pamphilus|EULEP4972-16|RVcoll16I424||Poland|658[0n]
Coenonympha pamphilus|EULEP5005-16|RVcoll16I808||Germany|658[0n]
Coenonympha pamphilus|EULEP741-15|RVcoll.12-N857|Lesvos|Greece|658[0n]
Coenonympha pamphilus|EULEP771-15|RVcoll.12-R932|Lesvos|Greece|658[0n]
Coenonympha pamphilus|EULEP1349-15|RVcoll.14-F896||Greece|658[0n]
Coenonympha pamphilus|EULEP518-15|RVcoll.07-F531||Estonia|658[0n]
Coenonympha pamphilus|EULEP622-15|RVcoll.11-I563|Genit. examined|Spain|658[0n]
Coenonympha pamphilus|EULEP1587-15|RVcoll.14-G617||Greece|658[0n]
Coenonympha pamphilus|EULEP1466-15|RVcoll.14-G227||Greece|658[0n]
Coenonympha pamphilus|EULEP2146-15|RVcoll.14-K025||Switzerland|658[0n]
Coenonympha pamphilus|EULEP1757-15|RVcoll.14-H419||Greece|658[0n]
Coenonympha pamphilus|EULEP1923-15|RVcoll.14-B856||Bosnia and Herzegovina|658[0n]
Coenonympha pamphilus|EULEP2019-15|RVcoll.14-I850||Poland|658[0n]
Coenonympha pamphilus|EULEP2202-15|RVcoll.14-N362||Ukraine|658[0n]
Coenonympha pamphilus|BIBSA1172-15|15-M198||Italy|658[0n]
Coenonympha pamphilus|BIBSA1224-15|15-L958||Italy|658[0n]
Coenonympha pamphilus|BIBSA1265-15|LD-2285||Italy|658[0n]
Coenonympha pamphilus|BIBSA925-15|16-A016||France|658[0n]
Coenonympha pamphilus|BIBSA989-15|15-C318||Italy|658[0n]
Coenonympha pamphilus|BIBSA1307-15|12-M342|Sicily|Italy|658[0n]
Coenonympha pamphilus|BIBSA1784-16|16-A517||Italy|658[0n]
Coenonympha pamphilus|BIBSA1366-15|15-M896||Italy|658[0n]
Coenonympha pamphilus|BIBSA1526-16|LEP-SS-00371||Italy|658[0n]
Coenonympha pamphilus|BIBSA1851-17|RVcoll16C730||Italy|658[0n]
Coenonympha pamphilus|BIBSA443-15|RVcoll.14-I111||Italy|658[0n]
Coenonympha pamphilus|BIBSA500-15|LEP-SS-00120||Italy|658[0n]
Coenonympha pamphilus|BIBSA501-15|LEP-SS-00121||Italy|658[0n]
Coenonympha pamphilus|BIBSA195-15|RVcoll.14-D983||Italy|658[0n]
Coenonympha pamphilus|BIBSA1849-17|RVcoll16C727||Italy|658[0n]
Coenonympha pamphilus|BIBSA373-15|RVcoll.14-I041||Italy|658[0n]
Coenonympha pamphilus|BIBSA374-15|RVcoll.14-I042||Italy|658[0n]
Coenonympha pamphilus|BIBSA1029-15|15-C183||Italy|658[0n]
Coenonympha pamphilus|GBLAB150-13|BC ZSM Lep 75776||Germany|658[0n]
Coenonympha pamphilus|GBLAC283-13|BC ZSM Lep 78664||Germany|658[0n]
Coenonympha pamphilus|OXB821-15|14-U784||France|658[0n]
Coenonympha pamphilus|OXB881-15|15-A523||Italy|658[0n]
Coenonympha pamphilus|OXB583-15|14-N972||Italy|658[0n]
Coenonympha pamphilus|OXB595-15|14-N984||Italy|658[0n]
Coenonympha pamphilus|OXB1105-15|15-A638||Italy|658[0n]
Coenonympha pamphilus|OXB1113-15|15-A646||Italy|658[0n]
Coenonympha pamphilus|OXB1131-15|15-A664||Italy|658[0n]
Coenonympha pamphilus|OXB667-15|07-D804||Italy|658[0n]
Coenonympha pamphilus|OXB680-15|11-Y107|Sicily W|Italy|658[0n]
Coenonympha pamphilus|OXB687-15|12-M381|Sicily W|Italy|658[0n]
Coenonympha pamphilus|OXB723-15|13-U141||Italy|658[0n]
Coenonympha pamphilus|OXB368-15|RVcoll.14-N082||Italy|658[0n]
Coenonympha pamphilus|OXB960-15|15-A910||Italy|658[0n]
Coenonympha pamphilus|OXB1001-15|15-A951||Italy|658[0n]
Coenonympha pamphilus|OXB1045-15|LD-3208||France|658[0n]
Coenonympha pamphilus|OXB1075-15|15-A608||Italy|658[0n]
Coenonympha pamphilus|OXB1545-16|OXB-TGS-1263||France|658[0n]
Coenonympha pamphilus|OXB1546-16|OXB-TGS-1264||France|658[0n]
Coenonympha pamphilus|OXB1324-15|Rvcoll_15-M595||France|658[0n]
Coenonympha pamphilus|OXB1330-15|Rvcoll_15-M602||France|658[0n]
Coenonympha pamphilus|OXB1234-15|Rvcoll_15-M669||France|658[0n]
Coenonympha pamphilus|OXB1235-15|Rvcoll_15-M671||France|658[0n]
Coenonympha pamphilus|OXB1268-15|Rvcoll_15-M127||France|658[0n]
Coenonympha pamphilus|OXB1372-15|Rvcoll_15-M735||France|658[0n]
Coenonympha pamphilus|OXB1384-15|Rvcoll_15-M749||France|658[0n]
Coenonympha pamphilus|WMB3821-14|RVcoll.09-T544||Italy|658[0n]
Coenonympha pamphilus|WMB1506-13|RVcoll.12-O091|Corsica|France|658[0n]
Coenonympha pamphilus|OXB1411-15|Rvcoll_15-M785||France|658[0n]
Coenonympha pamphilus|LENOA1379-11|LN-BD1379||France|649[0n]
Coenonympha pamphilus|WMB188-11|RVcoll.11-D497||Italy|651[0n]
Coenonympha pamphilus|OXB927-15|15-A571||Italy|643[0n]
Coenonympha pamphilus|EZSPC739-10|RVcoll.08-J717|Genit. examined|Spain|640[0n]
Coenonympha pamphilus|WMB180-11|RVcoll.11-D459|Sicily|Italy|634[0n]
Coenonympha pamphilus|OXB1292-15|Rvcoll_15-M155||France|647[0n]
Coenonympha pamphilus|BIBSA123-15|RVcoll.14-D571||Italy|621[0n]
Coenonympha pamphilus|OXB675-15|11-D346|Sicily E|Italy|620[0n]
Coenonympha pamphilus|WMB5133-14|RVcoll.07-C029||France|637[0n]
Coenonympha pamphilus|GBLAA379-14|BC ZSM Lep 80375||Germany|622[0n]
Coenonympha pamphilus|WMB3840-14|RVcoll.10-C520||Italy|614[0n]
Coenonympha pamphilus|EULEP1041-15|RVcoll.14-E905||Serbia|614[0n]
Coenonympha pamphilus|EULEP3676-16|RVcoll15I610||Austria|638[0n]

Coenonympha pamphilus|WMB3840-14|RVcoll.10-C520||Italy|614[0n]
Coenonympha pamphilus|EULEP1041-15|RVcoll.14-E905||Serbia|614[0n]
Coenonympha pamphilus|EULEP3676-16|RVcoll15I610||Austria|638[0n]
Coenonympha pamphilus|EULEP3679-16|RVcoll15Q140||Russia|639[0n]
Coenonympha pamphilus|EULEP2422-15|RVcoll.14-V204||Belgium|617[0n]
Coenonympha pamphilus|WMB3988-14|RVcoll.12-Q325||France|620[0n]
Coenonympha pamphilus|WMB1647-13|RVcoll.12-O975||France|634[0n]
Coenonympha pamphilus|GWORR407-10|BC ZSM Lep 29623||Italy|622[0n]
Coenonympha pamphilus|GWORR403-10|BC ZSM Lep 29619||Italy|658[0n]
Coenonympha pamphilus|LEATD020-13|TLMF Lep 12667||Italy|658[0n]
Coenonympha pamphilus|LEATG071-14|TLMF Lep 13858||Austria|658[0n]
Coenonympha pamphilus|PHLAW018-13|TLMF Lep 09815||Austria|658[0n]
Coenonympha pamphilus|LEATG072-14|TLMF Lep 13859||Austria|658[0n]
Coenonympha pamphilus|PHLAC365-10|TLMF Lep 02400||Italy|658[0n]
Zerynthia cretica|EULEP710-15|RVcoll.12-M221|Crete|Greece|658[0n]
Zerynthia cretica|EULEP711-15|RVcoll.12-M237|Crete|Greece|658[0n]
Zerynthia cretica|EULEP708-15|RVcoll.12-M207|Crete|Greece|658[0n]
Zerynthia cretica|EULEP703-15|RVcoll.12-M173|Crete|Greece|658[0n]
Zerynthia cretica|EULEP699-15|RVcoll.12-M139|Crete|Greece|658[0n]
Zerynthia cretica|EULEP694-15|RVcoll.12-M107|Crete|Greece|658[0n]
Zerynthia caucasica|EULEP2039-15|RVcoll.14-J518||Russia|658[0n]
Zerynthia caucasica|EULEP2038-15|RVcoll.14-J517||Russia|658[0n]
Zerynthia caucasica|EULEP2037-15|RVcoll.14-J516||Russia|658[0n]
Zerynthia caucasica|EULEP424-14|KN00786||Russia|658[0n]
Zerynthia caucasica|EULEP425-14|KN00787||Russia|658[0n]
Zerynthia caucasica|EULEP3420-16|RVcoll15Q147||Russia|633[0n]
Zerynthia caucasica|EULEP3419-16|RVcoll15Q146||Russia|643[0n]
Zerynthia cerisy|EULEP5590-17|RVcoll14N515|Lesvos|Greece|655[0n]
Zerynthia cerisy|EULEP5306-17|RVcoll08R595|Samos|Greece|655[0n]
Zerynthia cerisy|EULEP5308-17|RVcoll08R597|Samos|Greece|655[0n]
Zerynthia cerisy|EULEP5307-17|RVcoll08R596|Samos|Greece|655[0n]
Zerynthia cerisy|EULEP5549-17|RVcoll14E331||Bulgaria|655[0n]
Zerynthia cerisy|EULEP5591-17|RVcoll14N516||Macedonia|655[0n]
Zerynthia cerisy|EULEP5432-17|RVcoll11G199||Bulgaria|658[0n]
Zerynthia cerisy|EULEP5369-17|RVcoll10A241||Bulgaria|655[0n]
Zerynthia cerisy|EULEP5134-17|RVcoll16J890||Serbia|658[0n]
Zerynthia cerisy|EULEP5631-17|RVcoll15C891||Romania|658[0n]
Zerynthia cerisy|EULEP5630-17|RVcoll15C889||Romania|658[0n]
Zerynthia cerisy|EULEP5629-17|RVcoll15C888||Romania|658[0n]
Zerynthia cerisy|EULEP5628-17|RVcoll15C887||Romania|658[0n]
Zerynthia cerisy|EULEP5627-17|RVcoll15C886||Romania|658[0n]
Zerynthia cerisy|EULEP5605-17|RVcoll14U882||Romania|658[0n]
Zerynthia cerisy|EULEP5604-17|RVcoll14U881||Romania|658[0n]
Zerynthia cerisy|EULEP5603-17|RVcoll14U880||Romania|658[0n]
Zerynthia cerisy|EULEP5602-17|RVcoll14U879||Romania|658[0n]
Zerynthia cerisy|EULEP5601-17|RVcoll14U878||Romania|658[0n]
Zerynthia cerisy|EULEP5550-17|RVcoll14E439||Bulgaria|658[0n]
Zerynthia cerisy|EZRMN410-09|RVcoll.07-D031||Romania|650[0n]
Zerynthia rumina|EZSPN1041-11|Rvcoll.08-J350||Spain|658[0n]
Zerynthia rumina|WMB262-11|RVcoll.10-A416||France|658[0n]
Zerynthia rumina|EZSPN604-09|RVcoll.08-J311||Spain|658[0n]
Zerynthia rumina|WMB933-13|RVcoll.10-A577||France|658[0n]
Zerynthia rumina|WMB272-11|RVcoll.10-A496||France|658[0n]
Zerynthia rumina|WMB3422-14|RVcoll.09-X247||France|658[0n]
Zerynthia rumina|WMB3710-14|RVcoll.12-L619||Portugal|658[0n]
Zerynthia rumina|EZSPN1123-11|Rvcoll.10-C355||Spain|643[0n]
Zerynthia rumina|EZSPN191-09|RVcoll.07-C402||Spain|658[1n]
Zerynthia rumina|WMB3359-14|RVcoll.09-T141||Spain|658[0n]
Zerynthia rumina|EZSPN460-09|RVcoll.08-H668||Spain|658[0n]
Zerynthia rumina|EZSPN456-09|RVcoll.08-H662||Spain|658[0n]
Zerynthia rumina|EZSPN402-09|RVcoll.08-H472||Spain|658[0n]
Zerynthia rumina|EZSPN382-09|RVcoll.08-H430||Spain|658[0n]
Zerynthia rumina|WMB4586-14|RVcoll.190309FY22||Spain|658[0n]
Zerynthia rumina|EZSPM346-09|RVcoll.08-R042||Spain|658[0n]
Zerynthia rumina|EZSPN383-09|RVcoll.08-H431||Spain|658[0n]
Zerynthia rumina|WMB4464-14|RVcoll.14-D337||Spain|658[0n]
Zerynthia rumina|EZSPN498-09|RVcoll.08-H947||Spain|658[0n]
Zerynthia rumina|WMB4583-14|RVcoll.150308PP2||Spain|658[0n]
Zerynthia rumina|WMB4579-14|RVcoll.150308GV83||Spain|658[0n]
Zerynthia rumina|WMB4559-14|RVcoll.090211SD22||Spain|658[0n]
Zerynthia rumina|EZSPN366-09|RVcoll.08-H391||Spain|658[0n]
Zerynthia rumina|EZSPN352-09|RVcoll.08-H323||Spain|653[0n]
Zerynthia rumina|WMB4513-14|RVcoll.14-E203||Spain|658[0n]
Zerynthia rumina|EZSPN089-09|RVcoll.06-A018||Spain|658[0n]
Zerynthia cassandra|BIBSA665-15|RVcoll. 15-A828||Italy|658[0n]
Zerynthia cassandra|BIBSA003-14|LEP-SS-00003||Italy|658[0n]
Zerynthia cassandra|GBMIN12589-13|KC119728|Elba. Genit. examined|Italy|657[0n]
Zerynthia cassandra|GBMIN12597-13|KC119712||Italy|657[0n]
Zerynthia cassandra|WMB5035-14|RVcoll. 14-I582||Italy|658[0n]
Zerynthia cassandra|BIBSA020-14|LEP-SS-00020||Italy|658[0n]
Zerynthia cassandra|BIBSA601-15|LEP-SS-00221||Italy|658[0n]
Zerynthia cassandra|BIBSA004-14|LEP-SS-00004||Italy|658[0n]
Zerynthia cassandra|BIBSA600-15|LEP-SS-00220||Italy|658[0n]
Zerynthia cassandra|WMB5037-14|RVcoll. 14-I584||Italy|658[0n]
Zerynthia cassandra|WMB5036-14|RVcoll. 14-I583||Italy|658[0n]
Zerynthia cassandra|WMB4793-14|RVcoll. 14-I340||Italy|658[0n]
Zerynthia cassandra|WMB6091-18|RVcollLD3454||Italy|657[0n]
Zerynthia cassandra|WMB6088-18|RVcollLD3443||Italy|657[0n]
Zerynthia cassandra|WMB6087-18|RVcollLD3442||Italy|657[0n]
Zerynthia cassandra|WMB6086-18|RVcollLD3441||Italy|657[0n]
Zerynthia cassandra|WMB6085-18|RVcollLD3440||Italy|657[0n]
Zerynthia cassandra|WMB6084-18|RVcollLD3439||Italy|657[0n]
Zerynthia cassandra|WMB6083-18|RVcollLD3438||Italy|657[0n]
Zerynthia cassandra|WMB6082-18|RVcollLD3437||Italy|657[0n]
Zerynthia cassandra|WMB6081-18|RVcollLD3436||Italy|657[0n]
Zerynthia cassandra|WMB6080-18|RVcollLD1003||Italy|657[0n]
Zerynthia cassandra|WMB6076-18|RVcollLD0986||Italy|657[0n]
Zerynthia cassandra|GBMIN12611-13|KC119707|Genit. examined|Italy|657[0n]
Zerynthia cassandra|EULEP800-15|RVcoll.13-T755||Italy|658[0n]
Zerynthia cassandra|WMB6092-18|RVcollLD3455||Italy|657[0n]
Zerynthia cassandra|WMB6090-18|RVcollLD3453||Italy|657[0n]
Zerynthia cassandra|WMB6089-18|RVcollLD3452||Italy|657[0n]
Zerynthia cassandra|WMB6079-18|RVcollLD1002||Italy|657[0n]

Zerynthia cassandra|WMB6090-18|RVcollLD3453||Italy|657[0n]
Zerynthia cassandra|WMB6089-18|RVcollLD3452||Italy|657[0n]
Zerynthia cassandra|WMB6079-18|RVcollLD1002||Italy|657[0n]
Zerynthia cassandra|WMB6078-18|RVcollLD0989||Italy|657[0n]
Zerynthia cassandra|WMB6077-18|RVcollLD0987||Italy|657[0n]
Zerynthia cassandra|WMB6075-18|RVcollLD0983||Italy|657[0n]
Zerynthia cassandra|WMB6074-18|RVcollLD0978||Italy|657[0n]
Zerynthia cassandra|WMB6073-18|RVcollLD0019||Italy|657[0n]
Zerynthia cassandra|GBMIN12598-13|KC119710|Sicily|Italy|657[0n]
Zerynthia cassandra|GBMIN12599-13|KC119708|Sicily|Italy|657[0n]
Zerynthia cassandra|EULEP607-15|RVcoll.11-D292|Sicily|Italy|658[0n]
Zerynthia cassandra|GBMIN12609-13|KC119711|Sicily|Italy|657[0n]
Zerynthia cassandra|BIBSA1791-16|16-A528||Italy|658[0n]
Zerynthia cassandra|BIBSA1777-16|16-A480||Italy|658[0n]
Zerynthia cassandra|WMB622-11|RVcoll.11-I061|Sicily|Italy|658[0n]
Zerynthia cassandra|GBMIN12610-13|KC119709|Sicily|Italy|657[0n]
Zerynthia cassandra|WMB153-11|RVcoll.11-D290|Sicily|Italy|630[0n]
Zerynthia polyxena|GBMIN12602-13|KC119725|Genit. examined|France|657[0n]
Zerynthia polyxena|GBMIN12590-13|KC119726|Genit. examined|France|657[0n]
Zerynthia polyxena|GBMIN12592-13|KC119722|Genit. examined|France|657[0n]
Zerynthia polyxena|GBMIN12593-13|KC119720|Genit. examined|France|657[0n]
Zerynthia polyxena|GBMIN12604-13|KC119721|Genit. examined|France|657[0n]
Zerynthia polyxena|GBMIN12603-13|KC119723|Genit. examined|Italy|657[0n]
Zerynthia polyxena|GBMIN12605-13|KC119719|Genit. examined|France|657[0n]
Zerynthia polyxena|EULEP2312-15|RVcoll.14-O298||France|658[0n]
Zerynthia polyxena|EULEP2311-15|RVcoll.14-O297||France|658[0n]
Zerynthia polyxena|ABOLD573-17|TLMF Lep 21649||Austria|658[0n]
Zerynthia polyxena|LEASS465-17|TLMF Lep 21923||Austria|658[0n]
Zerynthia polyxena|LEASS464-17|TLMF Lep 21922||Austria|658[0n]
Zerynthia polyxena|LEASS463-17|TLMF Lep 21921||Austria|658[0n]
Zerynthia polyxena|EULEP2314-15|RVcoll.14-O300||Slovenia|658[0n]
Zerynthia polyxena|EULEP603-15|RVcoll.10-C400||Romania|658[0n]
Zerynthia polyxena|GBMIN12606-13|KC119717|Genit. examined|Italy|657[0n]
Zerynthia polyxena|GBMIN12594-13|KC119718|Genit. examined|Italy|657[0n]
Zerynthia polyxena|BIBSA1451-16|RVcoll.14-I205||Italy|658[0n]
Zerynthia polyxena|GBMIN12591-13|KC119724|Genit. examined|Italy|657[0n]
Zerynthia polyxena|BIBSA1450-16|RVcoll.14-I204||Italy|658[0n]
Zerynthia polyxena|EULEP2316-15|RVcoll.14-O302||Romania|618[1n]
Zerynthia polyxena|EZROM695-08|RV-07-F537||Romania|658[0n]
Zerynthia polyxena|EULEP2318-15|RVcoll.14-O304||Greece|658[0n]
Zerynthia polyxena|EULEP2317-15|RVcoll.14-O303||Greece|658[0n]
Zerynthia polyxena|EULEP2313-15|RVcoll.14-O299||Serbia|658[0n]
Zerynthia polyxena|EZROM566-08|RV-07-D395||Romania|658[0n]
Zerynthia polyxena|EULEP760-15|RVcoll.12-R683||Romania|658[0n]
Zerynthia polyxena|EULEP2337-15|RVcoll.14-V022||Ukraine|658[0n]
Zerynthia polyxena|EZROM1069-09|RVcoll.08-L358||Romania|658[0n]
Zerynthia polyxena|EZRMN405-09|RVcoll.07-D023||Romania|650[0n]
Zerynthia polyxena|EZRMN404-09|RVcoll.07-D396||Romania|650[0n]
Zerynthia polyxena|EZROM1064-09|RVcoll.07-F560||Romania|658[0n]
Papilio alexanor|EULEP3026-15|RVcoll.14-O159|Lesvos|Greece|658[0n]
Papilio alexanor|EULEP3024-15|RVcoll.14-O157||Macedonia|658[0n]
Papilio alexanor|EULEP3030-15|RVcoll.14-O163|Samos|Greece|658[0n]
Papilio alexanor|EULEP3028-15|RVcoll.14-O161||France|658[0n]
Papilio alexanor|WMB6028-18|PAX1||Italy|658[0n]
Papilio alexanor|EULEP3027-15|RVcoll.14-O160||France|658[0n]
Papilio alexanor|WMB6030-18|PAX3||Italy|658[0n]
Papilio alexanor|WMB6029-18|PAX2||Italy|658[0n]
Papilio alexanor|BIBSA859-15|LD-3471||Italy|658[0n]
Papilio alexanor|EULEP3025-15|RVcoll.14-O158||Macedonia|658[0n]
Papilio alexanor|EULEP1775-15|RVcoll.14-H540||Greece|658[0n]
Papilio alexanor|EULEP1736-15|RVcoll.14-H296||Greece|658[0n]
Papilio alexanor|EULEP1779-15|RVcoll.14-H570||Greece|658[1n]
Papilio alexanor|EULEP1727-15|RVcoll.14-H235||Greece|658[1n]
Papilio hospiton|EULEP813-15|RVcoll.13-U519|Sardinia|Italy|658[0n]
Papilio hospiton|WMB4741-14|RVcoll.13-T218|Corsica|France|658[0n]
Papilio hospiton|WMB4740-14|RVcoll.13-T209|Corsica|France|658[0n]
Papilio hospiton|WMB4555-14|RVcoll.14-E400|Corsica|France|658[0n]
Papilio hospiton|WMB3051-14|RVcoll.13-T642|Sardinia|Italy|658[0n]
Papilio hospiton|WMB3050-14|RVcoll.13-T635|Sardinia|Italy|658[0n]
Papilio machaon|WMB5118-14|RVcoll. 14-L249||Italy|658[0n]
Papilio machaon|EULEP742-15|RVcoll.12-N861|Lesvos|Greece|658[0n]
Papilio machaon|EULEP705-15|RVcoll.12-M179|Crete|Greece|658[0n]
Papilio machaon|WMB3530-14|RVcoll.10-C134||France|658[0n]
Papilio machaon|EZSPC1199-10|RVcoll.09-V419||Spain|658[0n]
Papilio machaon|WMB3548-14|RVcoll.11-D873||Spain|658[0n]
Papilio machaon|WMB096-11|RVcoll.10-B602||France|658[0n]
Papilio machaon|EZRMN153-08|RVcoll.08-M392||Romania|658[0n]
Papilio machaon|OXB952-15|15-A902||Italy|658[0n]
Papilio machaon|EULEP2148-15|RVcoll.14-K030||Switzerland|658[0n]
Papilio machaon|EULEP4368-16|RVcoll15P011||Belarus|658[0n]
Papilio machaon|EZSPN442-09|RVcoll.08-H627||Spain|658[0n]
Papilio machaon|FBLMU410-09|BC ZSM Lep 27060||Germany|658[0n]
Papilio machaon|LENOA1337-11|LN-BD1337||France|658[0n]
Papilio machaon|LEFID021-10|MM05450||Finland|658[0n]
Papilio machaon|EZRMN152-08|RVcoll.08-M338||Romania|658[0n]
Papilio machaon|EZROM448-08|RV-06-K638||Romania|658[0n]
Papilio machaon|EZROM450-08|RV-07-D302||Romania|658[0n]
Papilio machaon|EZSPC583-09|RVcoll.08-J346||Spain|658[0n]
Papilio machaon|EZSPC584-09|RVcoll.08-R265||Spain|658[0n]
Papilio machaon|WMB1666-13|RVcoll.12-P221||France|658[0n]
Papilio machaon|EULEP5076-16|RVcoll16J251||Germany|658[0n]
Papilio machaon|EULEP941-15|RVcoll.14-C930||Bulgaria|658[0n]
Papilio machaon|EULEP1750-15|RVcoll.14-H384||Greece|658[0n]
Papilio machaon|BIBSA1124-15|15-L906||Italy|658[0n]
Papilio machaon|GBLAA1486-15|BC ZSM Lep 87277||Germany|658[0n]
Papilio machaon|EULEP1612-15|RVcoll.14-G673||Greece|614[0n]
Papilio machaon|EULEP1352-15|RVcoll.14-F912||Greece|658[0n]
Papilio machaon|EULEP1784-15|RVcoll.14-H617||Greece|658[0n]
Papilio machaon|EULEP1586-15|RVcoll.14-G615||Greece|658[0n]
Papilio machaon|EULEP5685-17|RVcoll16L020|Corsica|France|658[0n]
Papilio machaon|WMB294-11|RVcoll.11-E302||Italy|658[0n]
Papilio machaon|WMB4724-14|RVcoll.LD-3078|Capri|Italy|658[0n]

Papilio machaon|EULEP5685-17|RVcoll16L020|Corsica|France|658[0n]
Papilio machaon|WMB294-11|RVcoll.11-E302||Italy|658[0n]
Papilio machaon|WMB4724-14|RVcoll.LD-3078|Capri|Italy|658[0n]
Papilio machaon|WMB4723-14|RVcoll.LD-3077|Capri|Italy|658[0n]
Papilio machaon|WMB4320-14|RVcoll.14-A553||Italy|658[0n]
Papilio machaon|WMB1802-13|RVcoll.12-Q338||France|658[0n]
Papilio machaon|EZSPC1049-10|RVcoll.08-J745||Spain|658[0n]
Papilio machaon|LEATG387-14|TLMF Lep 14174||Italy|658[0n]
Papilio machaon|WMB3173-14|RVcoll.08-H555||Spain|658[0n]
Papilio machaon|WMB3902-14|RVcoll.12-P290||France|658[0n]
Papilio machaon|EULEP4363-16|RVcoll14F023||Serbia|658[0n]
Papilio machaon|EULEP4364-16|RVcoll15H201||Switzerland|658[0n]
Papilio machaon|EULEP4367-16|RVcoll15G486||Switzerland|658[0n]
Papilio machaon|EULEP1305-15|RVcoll.14-F753||Greece|658[0n]
Papilio machaon|OXB1059-15|11-H368||Italy|658[0n]
Papilio machaon|EZSPM207-09|RVcoll.08-R325||Spain|658[0n]
Papilio machaon|EZSPM043-09|RVcoll.08-P251||Spain|658[0n]
Papilio machaon|EZSPM060-09|RVcoll.08-P286||Spain|658[0n]
Papilio machaon|EZSPM366-09|RVcoll.08-J830||Spain|658[0n]
Papilio machaon|EZSPM287-09|SMcoll.311007WR55||Spain|658[0n]
Papilio machaon|EZSPN609-09|RVcoll.08-J326||Spain|658[0n]
Papilio machaon|EZSPM1009-12|Rvcoll.12-L899||Spain|658[0n]
Papilio machaon|EZSPM996-12|Rvcoll.12-L882||Spain|658[0n]
Papilio machaon|EZSPM993-12|Rvcoll.12-L879||Spain|658[0n]
Papilio machaon|EZSPM049-09|RVcoll.08-P267||Spain|658[0n]
Papilio machaon|LEFIB255-10|MM00727||Finland|658[0n]
Papilio machaon|LEATJ1305-16|TLMF Lep 19628||Austria|658[0n]
Papilio machaon|WMB526-11|RVcoll.11-H630|Lampedusa|Italy|658[0n]
Papilio machaon|WMB2318-13|RVcoll.11-H629|Lampedusa|Italy|658[0n]
Papilio machaon|WMB2317-13|RVcoll.11-H628|Lampedusa|Italy|658[0n]
Papilio machaon|WMB1912-13|RVcoll.11-H627|Lampedusa|Italy|658[0n]
Papilio machaon|WMB1926-13|RVcoll.11-H792|Vulcano|Italy|658[0n]
Papilio machaon|OXB697-15|12-R119|Sicily E|Italy|658[0n]
Papilio machaon|WMB569-11|RVcoll.11-H791|Vulcano|Italy|658[0n]
Papilio machaon|WMB4629-14|RVcoll.LD-2381|Ustica|Italy|658[0n]
Papilio machaon|BIBSA1417-15|15-N087||Italy|658[0n]
Papilio machaon|BIBSA521-15|LEP-SS-00141||Italy|658[0n]
Papilio machaon|BIBSA1799-16|16-A553||Italy|658[0n]
Papilio machaon|OXB852-15|14-U815||Italy|658[0n]
Papilio machaon|BIBSA1040-15|15-C218||Italy|623[0n]
Papilio machaon|WMB4924-14|RVcoll.14-I471||Italy|658[0n]
Papilio machaon|WMB5342-14|RVcoll.14-J356|Pianosa|Italy|658[0n]
Papilio machaon|WMB578-11|RVcoll.11-H866|Salina|Italy|658[0n]
Papilio machaon|WMB498-11|RVcoll.11-H554|Sicily|Italy|658[0n]
Papilio machaon|WMB2457-13|RVcoll.11-H279||Italy|658[0n]
Papilio machaon|WMB2121-13|RVcoll.12-R196||Italy|658[0n]
Papilio machaon|WMB1136-13|RVcoll.11-E122|Gozo|Malta|658[0n]
Papilio machaon|WMB2893-14|RVcoll.11-D403|Sicily|Italy|658[0n]
Papilio machaon|WMB161-11|RVcoll.11-D330|Sicily|Italy|658[0n]
Papilio machaon|EZSPM289-09|SMcoll.270708ZB68||Spain|658[0n]
Papilio machaon|OXB1265-15|Rvcoll_15-M121||France|658[0n]
Papilio machaon|WMB3025-14|RVcoll.13-S385|Ibiza|Spain|658[0n]
Papilio machaon|EZSPM1043-12|Rvcoll.12-M963||Spain|658[0n]
Papilio machaon|EZSPN395-09|RVcoll.08-H454||Spain|658[0n]
Papilio machaon|WMB6566-18|RVcoll14A078||Italy|658[0n]
Papilio machaon|OXB1162-15|Rvcoll_16-A049||Italy|658[0n]
Papilio machaon|OXB976-15|15-A926||Italy|658[0n]
Papilio machaon|BIBSA1602-16|12-R161||Italy|658[0n]
Papilio machaon|OXB911-15|15-A555||Italy|658[0n]
Papilio machaon|WMB171-11|RVcoll.11-D414|Sicily|Italy|658[0n]
Papilio machaon|BIBSA522-15|LEP-SS-00142||Italy|658[0n]
Papilio machaon|WMB5466-14|RVcoll.14-N657|Salina|Italy|658[0n]
Papilio machaon|WMB5051-14|RVcoll. 14-L182||Italy|658[0n]
Papilio machaon|WMB4967-14|RVcoll. 14-I514||Italy|658[0n]
Papilio machaon|WMB4834-14|RVcoll. 14-I381||Italy|658[0n]
Papilio machaon|WMB3832-14|RVcoll.09-X914||Italy|658[0n]
Papilio machaon|WMB1267-13|RVcoll.12-M286|Lipari|Italy|658[0n]
Papilio machaon|WMB766-13|RVcoll.11-Y005|Elba|Italy|658[0n]
Papilio machaon|WMB2876-14|RVcoll.10-C576|Argentario|Italy|658[0n]
Papilio machaon|GWORU074-10|BC ZSM Lep 30335||Italy|658[0n]
Papilio machaon|WMB515-11|RVcoll.11-H602|Sicily|Italy|658[0n]
Papilio machaon|EULEP5674-17|RVcoll16J798|Ikaria|Greece|658[0n]
Papilio machaon|WMB4229-14|RVcoll.13-T773||Italy|658[0n]
Papilio machaon|WMB1583-13|RVcoll.12-O552|Sardinia|Italy|658[0n]
Papilio machaon|WMB5324-14|RVcoll.14-I993|La Maddalena|Italy|658[0n]
Papilio machaon|WMB6633-18|RVcoll17A026||Malta|658[0n]
Papilio machaon|WMB335-11|RVcoll.11-E673||Italy|658[0n]
Papilio machaon|WMB4708-14|RVcoll.LD-2914|Asinara|Italy|658[0n]
Papilio machaon|BIBSA1390-15|15-M986||Italy|658[0n]
Papilio machaon|BIBSA1286-15|13-T686|Sardinia|Italy|658[0n]
Papilio machaon|WMB4501-14|RVcoll.14-E178||Italy|658[0n]
Papilio machaon|WMB2450-13|RVcoll.11-E398|Sardinia|Italy|658[0n]
Papilio machaon|WMB2403-13|RVcoll.09-X938|Ponza|Italy|658[0n]
Papilio machaon|WMB2301-13|RVcoll.11-E120|Gozo|Malta|658[0n]
Papilio machaon|WMB2192-13|RVcoll.09-X936|Ponza|Italy|658[0n]
Papilio machaon|WMB3843-14|RVcoll.10-C608||Italy|658[0n]
Papilio machaon|OXB893-15|15-A537||Italy|658[0n]
Papilio machaon|OXB863-15|15-A507||Italy|658[0n]
Papilio machaon|BIBSA1277-15|12-O366|Sardinia|Italy|658[0n]
Papilio machaon|WMB5343-14|RVcoll.14-J357|Pianosa|Italy|658[0n]
Papilio machaon|WMB1674-13|RVcoll.11-E119|Gozo|Malta|658[0n]
Papilio machaon|WMB1593-13|RVcoll.12-O597|Sardinia|Italy|658[0n]
Papilio machaon|WMB1556-13|RVcoll.12-O397|San Pietro|Italy|658[0n]
Papilio machaon|WMB920-13|RVcoll.09-X932|Elba|Italy|658[0n]
Papilio machaon|WMB1501-13|RVcoll.12-O048||Italy|658[0n]
Papilio machaon|WMB1135-13|RVcoll.11-E121|Gozo|Malta|658[0n]
Papilio machaon|WMB3057-14|RVcoll.13-T735|Sardinia|Italy|658[0n]
Papilio machaon|WMB2865-14|RVcoll.09-X937.1|Ponza|Italy|658[0n]
Papilio machaon|WMB3043-14|RVcoll.13-S575|San Domino|Italy|658[0n]
Papilio machaon|EZSPC582-09|RVcoll.08-J361||Spain|658[0n]
Papilio machaon|WMB186-11|RVcoll.11-D492||Italy|658[0n]
Papilio machaon|EZSPC1170-10|RVcoll.09-T105||Spain|658[0n]
Papilio machaon|WMB2967-14|RVcoll.12-O049|Argentario|Italy|658[0n]

Papilio machaon|WMB186-11|RVcoll.11-D492||Italy|658[0n]
Papilio machaon|EZSPC1170-10|RVcoll.09-T105||Spain|658[0n]
Papilio machaon|WMB2967-14|RVcoll.12-O049|Argentario|Italy|658[0n]
Papilio machaon|WMB1523-13|RVcoll.12-O197|Corsica|France|658[0n]
Papilio machaon|WMB1527-13|RVcoll.12-O221|Corsica|France|658[0n]
Papilio machaon|WMB906-13|RVcoll.09-X895|Capri|Italy|658[0n]
Papilio machaon|WMB1736-13|RVcoll.12-P948|Levant|France|658[0n]
Papilio machaon|WMB4757-14|RVcoll. 14-I304||Italy|658[0n]
Papilio machaon|WMB5157-14|RVcoll.12-Q790||Italy|658[0n]
Papilio machaon|EULEP5000-16|RVcoll16I789||Germany|658[0n]
Papilio machaon|EULEP176-14|RVcoll.11-J350||Romania|658[0n]
Papilio machaon|EULEP179-14|RVcoll.11-J399||Romania|658[0n]
Papilio machaon|BIBSA693-15|RVcoll. 11-D511||Italy|658[0n]
Papilio machaon|GBLAA1350-15|BC ZSM Lep 87046||Germany|658[0n]
Papilio machaon|GBLAB310-13|BC ZSM Lep 72611||Germany|658[0n]
Papilio machaon|OXB835-15|14-U798||Italy|658[0n]
Papilio machaon|OXB574-15|14-N963||Italy|658[0n]
Papilio machaon|OXB678-15|11-Y095|Sicily W|Italy|658[0n]
Papilio machaon|EZROM449-08|RV-06-M955||Romania|652[0n]
Papilio machaon|EZROM338-08|RV-08-A016||Romania|646[0n]
Papilio machaon|OXB895-15|15-A539||Italy|658[0n]
Papilio machaon|LEATG388-14|TLMF Lep 14175||Italy|658[0n]
Papilio machaon|LEFIJ4741-16|ZMBSU-02123||Belarus|658[0n]
Papilio machaon|LEFID090-10|MM05702||Finland|658[0n]
Papilio machaon|EULEP4986-16|RVcoll16I684||Poland|640[0n]
Papilio machaon|WMB5501-14|RVcoll.14-J421|Giglio|Italy|658[0n]
Papilio machaon|WMB4682-14|RVcoll.LD-2673|Giglio|Italy|658[0n]
Papilio machaon|EULEP1984-15|RVcoll.14-H959||France|658[0n]
Papilio machaon|WMB3292-14|RVcoll.08-M966||France|658[0n]
Papilio machaon|EZROM447-08|RV-06-K527||Romania|658[0n]
Papilio machaon|ABOLD047-16|TLMF Lep 21125||Austria|658[0n]
Papilio machaon|BIBSA1057-15|15-M997||Italy|658[0n]
Papilio machaon|EULEP4366-16|RVcoll15J461||France|658[0n]
Papilio machaon|EULEP4365-16|RVcoll15I902||Liechtenstein|633[0n]
Papilio machaon|GWORK513-09|BC ZSM Lep 21843||Germany|658[0n]
Papilio machaon|LEATG235-14|TLMF Lep 14022||Austria|658[0n]
Papilio machaon|LEATG079-14|TLMF Lep 13866||Austria|658[0n]
Papilio machaon|PHLAI551-13|TLMF Lep 09113||Austria|658[0n]
Charaxes jasius|LEATJ1290-16|TLMF Lep 19613||Croatia|658[0n]
Charaxes jasius|WMB3088-14|RVcoll.14-D955|Sardinia|Italy|658[0n]
Charaxes jasius|WMB1095-13|RVcoll.10-C763|Capraia|Italy|655[0n]
Charaxes jasius|WMB2912-14|RVcoll.11-E988|Corsica|France|658[0n]
Charaxes jasius|WMB3069-14|RVcoll.14-D935|Elba|Italy|658[0n]
Charaxes jasius|WMB3083-14|RVcoll.14-D950|Giglio|Italy|658[0n]
Charaxes jasius|WMB3084-14|RVcoll.14-D951|Giglio|Italy|658[0n]
Charaxes jasius|WMB3096-14|RVcoll.LD-2557|Lipari|Italy|658[0n]
Charaxes jasius|WMB1535-13|RVcoll.12-O275|La Maddalena|Italy|658[0n]
Charaxes jasius|WMB1746-13|RVcoll.12-P958|Levant|France|658[0n]
Charaxes jasius|WMB1766-13|RVcoll.12-Q019||France|658[0n]
Charaxes jasius|WMB5972-17|RVcoll14V917||Italy|658[0n]
Charaxes jasius|WMB2618-13|RVcoll.12-O273|Corsica|France|658[0n]
Charaxes jasius|WMB361-11|RVcoll.11-E960||France|658[0n]
Charaxes jasius|WMB5339-14|RVcoll.14-J325|Elba|Italy|658[0n]
Charaxes jasius|WMB5347-14|RVcoll.14-J379|Giannutri|Italy|658[0n]
Charaxes jasius|WMB5468-14|RVcoll.14-N672|Salina|Italy|658[0n]
Charaxes jasius|WMB5469-14|RVcoll.14-N673|Salina|Italy|658[0n]
Charaxes jasius|BIBSA856-15|LD-3468||Italy|658[0n]
Charaxes jasius|BIBSA976-15|14-V916||Italy|658[0n]
Charaxes jasius|BIBSA1415-15|15-N085||Italy|658[0n]
Charaxes jasius|BIBSA1416-15|15-N086||Italy|658[0n]
Charaxes jasius|OLEP060-15|RVcoll.14-V864|Sicily|Italy|658[0n]
Charaxes jasius|WMB1094-13|RVcoll.10-C762|Capraia|Italy|601[0n]
Charaxes jasius|WMB1533-13|RVcoll.12-O272|Corsica|France|633[0n]
Charaxes jasius|EZSPC1119-10|RVcoll.08-P463||Spain|611[0n]
Charaxes jasius|EZSPC1334-10|RVcoll.130209KL7||Spain|658[0n]
Charaxes jasius|EZSPC844-10|RVcoll.08-P464.1||Spain|658[0n]
Charaxes jasius|EZSPM210-09|RVcoll.08-R330||Spain|658[0n]
Charaxes jasius|EZSPM209-09|RVcoll.08-R329||Spain|658[0n]
Charaxes jasius|EZSPM204-09|RVcoll.08-R314||Spain|658[0n]
Charaxes jasius|EZSPM040-09|RVcoll.08-P248||Spain|658[0n]
Charaxes jasius|EZSPM1008-12|Rvcoll.12-L898||Spain|658[0n]
Charaxes jasius|WMB1747-13|RVcoll.12-P959|Levant|France|658[0n]
Charaxes jasius|EZSPC992-10|RVcoll.07-F534.1||Spain|658[0n]
Charaxes jasius|EZSPC993-10|RVcoll.07-F535||Spain|658[0n]
Charaxes jasius|EZSPM420-09|Rvcoll.09-V843||Spain|658[0n]
Charaxes jasius|EZSPN079-09|SMcoll.020808CB85||Spain|658[0n]
Parnassius mnemosyne|EZRMN159-08|RVcoll.08-M274||Romania|658[0n]
Parnassius mnemosyne|EULEP1229-15|RVcoll.14-F532||Greece|658[0n]
Parnassius mnemosyne|EZROM646-08|RV-07-D060||Romania|658[0n]
Parnassius mnemosyne|EULEP1438-15|RVcoll.14-G129||Greece|658[0n]
Parnassius mnemosyne|EULEP1434-15|RVcoll.14-G121||Greece|658[0n]
Parnassius mnemosyne|LEATJ1187-16|TLMF Lep 19510||Russia|658[0n]
Parnassius mnemosyne|EULEP2080-15|RVcoll.14-J568||Ukraine|658[0n]
Parnassius mnemosyne|LEASS729-17|TLMF Lep 22377||Austria|658[0n]
Parnassius mnemosyne|EULEP4382-16|RVcoll15I805||Austria|658[0n]
Parnassius mnemosyne|LEASS858-17|KLM Lep 08363||Austria|658[0n]
Parnassius mnemosyne|EULEP4833-16|RVcoll16H735||Ukraine|658[0n]
Parnassius mnemosyne|EZRMN160-08|RVcoll.08-M361||Romania|658[0n]
Parnassius mnemosyne|EULEP580-15|RVcoll.10-A396||Bulgaria|658[0n]
Parnassius mnemosyne|EULEP1892-15|RVcoll.14-B709||Albania|658[0n]
Parnassius mnemosyne|EULEP1341-15|RVcoll.14-F835||Greece|658[0n]
Parnassius mnemosyne|EULEP1335-15|RVcoll.14-F822||Greece|658[0n]
Parnassius mnemosyne|BIBSA1120-15|15-L865||Italy|658[0n]
Parnassius mnemosyne|LEFIJ4740-16|ZMBSU-02101||Belarus|658[0n]
Parnassius mnemosyne|LEATJ1239-16|TLMF Lep 19562||Hungary|658[0n]
Parnassius mnemosyne|EULEP2109-15|RVcoll.14-J601||Ukraine|658[0n]
Parnassius mnemosyne|EULEP4859-16|RVcoll16H834||Poland|658[0n]
Parnassius mnemosyne|EULEP4378-16|RVcoll10A781||Estonia|658[0n]
Parnassius mnemosyne|EZRMN163-08|RVcoll.08-M642||Romania|658[0n]
Parnassius mnemosyne|EZRMN158-08|RVcoll.08-M246||Romania|658[0n]
Parnassius mnemosyne|EZROM458-08|RV-07-C107||Romania|658[0n]
Parnassius mnemosyne|EZRMN162-08|RVcoll.08-M629||Romania|658[0n]
[illegible]

Parnassius mnemosyne|EZRMN158-08|RVcoll.08-M246||Romania|658[0n]
Parnassius mnemosyne|EZROM458-08|RV-07-C107||Romania|658[0n]
Parnassius mnemosyne|EZRMN162-08|RVcoll.08-M629||Romania|658[0n]
Parnassius mnemosyne|EZRMN161-08|RVcoll.08-M417||Romania|658[0n]
Parnassius mnemosyne|LEFID119-10|MM05853||Estonia|658[0n]
Parnassius mnemosyne|LEFIJ795-10|MM17420||Finland|658[0n]
Parnassius mnemosyne|EZROM456-08|RV-06-K661||Romania|658[0n]
Parnassius mnemosyne|EZROM457-08|RV-07-D136||Romania|656[0n]
Parnassius mnemosyne|LEFIJ794-10|MM17419||Finland|658[0n]
Parnassius mnemosyne|LEASS538-17|TLMF Lep 22186||Austria|658[0n]
Parnassius mnemosyne|ODOPE191-11|BC ZSM Lep 53062||Germany|658[0n]
Parnassius mnemosyne|PHLAG184-12|TLMF Lep 06494||Austria|658[0n]
Parnassius mnemosyne|ODOPE194-11|BC ZSM Lep 53065||Germany|658[0n]
Parnassius mnemosyne|LON926-12|NHMO Lep09103||Norway|658[0n]
Parnassius mnemosyne|PHLAG183-12|TLMF Lep 06493||Austria|658[0n]
Parnassius mnemosyne|GWORA2437-09|BC ZSM Lep 30649||Austria|658[0n]
Parnassius mnemosyne|PHLAG181-12|TLMF Lep 06491||Austria|658[0n]
Parnassius mnemosyne|PHLAG182-12|TLMF Lep 06492||Austria|658[0n]
Parnassius mnemosyne|BIBSA001-14|LEP-SS-00001||Italy|658[0n]
Parnassius mnemosyne|BIBSA1676-16|15-C867||Italy|658[0n]
Parnassius mnemosyne|BIBSA002-14|LEP-SS-00002||Italy|632[0n]
Parnassius mnemosyne|BIBSA1675-16|15-C862||Italy|618[0n]
Parnassius mnemosyne|BIBSA1561-16|11-H738||Italy|658[0n]
Parnassius mnemosyne|BIBSA1564-16|11-H953||Italy|618[0n]
Parnassius mnemosyne|WMB607-11|RVcoll.11-I006|Sicily|Italy|658[0n]
Parnassius mnemosyne|WMB597-11|RVcoll.11-H952|Sicily|Italy|658[0n]
Parnassius mnemosyne|WMB548-11|RVcoll.11-H737|Sicily|Italy|658[0n]
Parnassius mnemosyne|EZSPN432-09|RVcoll.08-H609||Spain|658[0n]
Parnassius mnemosyne|EZSPN431-09|RVcoll.08-H608||Spain|658[0n]
Parnassius mnemosyne|EZSPC594-09|RVcoll.07-W141||Spain|658[0n]
Parnassius mnemosyne|EZSPN035-09|RVcoll.09-T076||Spain|658[0n]
Parnassius mnemosyne|EZSPC928-10|RVcoll.09-X000||Spain|627[0n]
Parnassius mnemosyne|EZSPN060-09|RVcoll.09-V684||Spain|658[0n]
Parnassius mnemosyne|EZSPN059-09|RVcoll.09-V683||Spain|658[0n]
Parnassius mnemosyne|EZSPC645-09|RVcoll.07-C024||France|658[0n]
Parnassius mnemosyne|BIBSA1065-15|14-V321||Italy|658[0n]
Parnassius mnemosyne|EULEP4381-16|RVcoll15H297||Switzerland|658[0n]
Parnassius mnemosyne|BCLEP112-17|LEP-SS-00563||Italy|658[0n]
Parnassius mnemosyne|GWORU075-10|BC ZSM Lep 30336||Italy|658[0n]
Parnassius mnemosyne|GWORT458-10|BC ZSM Lep 32144||Germany|658[0n]
Parnassius mnemosyne|PHLSA695-11|TLMF Lep 06150||Austria|658[0n]
Parnassius mnemosyne|ODOPE739-11|BC ZSM Lep 50380|ssp. korbi|Germany|658[0n]
Parnassius mnemosyne|LEASS667-17|TLMF Lep 22315||Austria|658[0n]
Parnassius mnemosyne|LEASS666-17|TLMF Lep 22314||Austria|658[0n]
Parnassius mnemosyne|LEASS668-17|TLMF Lep 22316||Austria|658[0n]
Parnassius mnemosyne|LEASS741-17|TLMF Lep 22389||Austria|658[0n]
Parnassius mnemosyne|PHLAD681-11|TLMF Lep 04046||Switzerland|658[0n]
Parnassius mnemosyne|BIBSA1576-16|11-I228||Italy|658[0n]
Parnassius mnemosyne|WMB1976-13|RVcoll.11-I226||Italy|658[0n]
Parnassius mnemosyne|WMB1975-13|RVcoll.11-I225||Italy|658[0n]
Parnassius mnemosyne|BIBSA1026-15|15-C171||Italy|658[0n]
Parnassius mnemosyne|BIBSA1359-15|15-M863||Italy|658[0n]
Parnassius mnemosyne|BIBSA861-15|LD-3473||Italy|642[0n]
Parnassius mnemosyne|BIBSA860-15|LD-3472||Italy|658[0n]
Parnassius mnemosyne|BIBSA1430-16|RVcoll.14-I184||Italy|658[0n]
Parnassius mnemosyne|BIBSA1429-16|RVcoll.14-I183||Italy|658[0n]
Parnassius mnemosyne|EULEP4380-16|RVcoll15H181||Switzerland|658[0n]
Parnassius mnemosyne|EULEP4379-16|RVcoll15G910||Switzerland|658[0n]
Parnassius mnemosyne|WMB5233-14|RVcoll.13-U073||Italy|658[0n]
Parnassius mnemosyne|WMB5208-14|RVcoll.13-T947||Italy|658[0n]
Parnassius mnemosyne|WMB4939-14|RVcoll.14-I486||Italy|658[0n]
Parnassius mnemosyne|PHLAD682-11|TLMF Lep 04047||Switzerland|658[0n]
Parnassius apollo|EULEP969-15|RVcoll.14-D048||Bulgaria|658[0n]
Parnassius apollo|LON940-12|NHMO Lep2011.004||Norway|658[0n]
Parnassius apollo|LON939-12|NHMO Lep2011.003||Norway|658[0n]
Parnassius apollo|LEATJ1189-16|TLMF Lep 19512||Czech Republic|658[0n]
Parnassius apollo|EZRMN365-08|RVcoll.08-M732||Romania|609[0n]
Parnassius apollo|LON943-12|NHMO Lep2011.240||Norway|658[0n]
Parnassius apollo|LON942-12|NHMO Lep2011.239||Norway|658[0n]
Parnassius apollo|LON941-12|NHMO Lep2011.238||Norway|658[0n]
Parnassius apollo|LON938-12|NHMO Lep2011.002||Norway|658[0n]
Parnassius apollo|LON937-12|NHMO Lep2011.001||Norway|658[0n]
Parnassius apollo|LON936-12|NHMO Lep2010.009||Norway|658[0n]
Parnassius phoebus|EULEP4385-16|RVcoll15I320||Austria|658[0n]
Parnassius phoebus|EULEP4387-16|RVcoll15J308||France|658[0n]
Parnassius phoebus|EULEP2110-15|RVcoll.14-J602||Switzerland|658[0n]
Parnassius phoebus|EULEP2085-15|RVcoll.14-J573||Switzerland|658[0n]
Parnassius phoebus|EULEP4388-16|RVcoll15O005||France|658[0n]
Parnassius phoebus|EULEP4383-16|RVcoll15G403||Switzerland|658[0n]
Parnassius phoebus|PHLAA421-09|TLMF Lep 00461|phoebus gazeli PRAVIEL, 1936|France|658[0n]
Parnassius phoebus|PHLAC933-10|TLMF Lep 02968||Switzerland|658[0n]
Parnassius phoebus|PHLAC932-10|TLMF Lep 02967||Switzerland|658[0n]
Parnassius phoebus|PHLAC726-10|TLMF Lep 02761||Switzerland|658[0n]
Parnassius phoebus|EULEP4384-16|RVcoll15H706||Switzerland|658[0n]
Parnassius phoebus|EULEP4386-16|RVcoll15J038||Switzerland|658[0n]
Parnassius phoebus|EULEP4389-16|RVcoll15O008||Italy|658[0n]
Parnassius phoebus|EULEP4390-16|RVcoll15O009||Italy|658[0n]
Parnassius phoebus|EULEP2456-15|RVcoll.14-V279||Switzerland|658[0n]
Parnassius phoebus|LEASS978-17|TLMF Lep 22531||Austria|658[0n]
Parnassius phoebus|LEASS539-17|TLMF Lep 22187||Austria|658[0n]
Parnassius phoebus|LEATI044-15|TLMF Lep 17429||Austria|658[0n]
Parnassius phoebus|EULEP2087-15|RVcoll.14-J575||Russia|617[0n]
Parnassius phoebus|LEATG384-14|TLMF Lep 14171||Italy|618[0n]
Parnassius phoebus|LEATI004-15|TLMF Lep 17389||Austria|658[0n]
Parnassius phoebus|LEATG383-14|TLMF Lep 14170||Italy|658[0n]
Parnassius apollo|EZSPM428-09|Rvcoll.09-X530||Spain|658[0n]
Parnassius apollo|EZSPC968-10|RVcoll.220708XF62||Spain|658[0n]
Parnassius apollo|EZSPM091-09|RVcoll.08-P417||Spain|658[0n]
Parnassius apollo|EZSPC891-10|RVcoll.09-V401||Spain|658[0n]
Parnassius apollo|EZSPC961-10|RVcoll.100608DS77||Spain|621[0n]
Parnassius apollo|EZSPC959-10|RVcoll.100608DS55||Spain|658[0n]
Parnassius apollo|EZSPM469-09|Rvcoll.09-V528||Spain|658[0n]
Parnassius apollo|EZSPN933-09|RVcoll.08-L893||Spain|650[0n]

Parnassius apollo|EZSPC959-10|RVcoll.100608DS55||Spain|658[0n]
Parnassius apollo|EZSPM469-09|Rvcoll.09-V528||Spain|658[0n]
Parnassius apollo|EZSPN933-09|RVcoll.08-L893||Spain|650[0n]
Parnassius apollo|EZSPC965-10|RVcoll.190608FT56||Spain|658[0n]
Parnassius apollo|EZSPN364-09|RVcoll.08-H348||Spain|658[0n]
Parnassius apollo|EULEP5882-18|RVcoll15O578||Spain|658[0n]
Parnassius apollo|EULEP210-14|RVcoll.12-L028||Spain|658[0n]
Parnassius apollo|EZSPM299-09|RVcoll.08-J846||Spain|658[0n]
Parnassius apollo|EZSPN359-09|RVcoll.08-H337||Spain|658[0n]
Parnassius apollo|WMB1843-13|RVcoll.11-I896||France|658[0n]
Parnassius apollo|EZSPM014-09|RVcoll.08-M999||France|658[0n]
Parnassius apollo|EZSPC916-10|RVcoll.09-V869||Spain|658[0n]
Parnassius apollo|EZSPC866-10|RVcoll.09-T088||Spain|658[0n]
Parnassius apollo|EZSPC591-09|RVcoll.08-R195||Spain|658[0n]
Parnassius apollo|EZSPC589-09|RVcoll.07-C489||Spain|658[0n]
Parnassius apollo|EZSPC590-09|RVcoll.07-C492||Spain|658[0n]
Parnassius apollo|WMB5274-14|RVcoll.14-A614||Italy|658[0n]
Parnassius apollo|WMB5124-14|RVcoll. 14-L255||Italy|658[0n]
Parnassius apollo|BIBSA722-15|LD-2549||Italy|658[0n]
Parnassius apollo|EZSPC593-09|RVcoll.08-R189||Spain|658[0n]
Parnassius apollo|WMB5032-14|RVcoll. 14-I579||Italy|658[0n]
Parnassius apollo|LEASS857-17|KLM Lep 08362||Austria|658[0n]
Parnassius apollo|EULEP2161-15|RVcoll.14-K073||Switzerland|658[0n]
Parnassius apollo|ABOLD061-16|TLMF Lep 21139||Austria|658[0n]
Parnassius apollo|GWORA2431-09|BC ZSM Lep 30643|ssp. bartholomaeus|Germany|658[0n]
Parnassius apollo|GWORA2432-09|BC ZSM Lep 30644|ssp. adonais|Germany|658[0n]
Parnassius apollo|GWORA2435-09|BC ZSM Lep 30647|ssp. melliculus|Germany|658[0n]
Parnassius apollo|GWORK287-09|BC ZSM Lep 21522||Germany|658[0n]
Parnassius apollo|EZSPC592-09|RVcoll.08-R196||Spain|658[0n]
Parnassius apollo|EZSPC907-10|RVcoll.09-V757||Spain|658[0n]
Parnassius apollo|WMB2688-13|RVcoll.10-B992||France|658[0n]
Parnassius apollo|WMB824-13|RVcoll.07-E194||Italy|658[0n]
Parnassius apollo|WMB826-13|RVcoll.07-E196||Italy|658[0n]
Parnassius apollo|WMB1613-13|RVcoll.12-O731||France|658[0n]
Parnassius apollo|WMB1874-13|RVcoll.11-J638|Sicily|Italy|658[0n]
Parnassius apollo|WMB1881-13|RVcoll.11-J706|Sicily|Italy|658[0n]
Parnassius apollo|WMB1882-13|RVcoll.11-J710|Sicily|Italy|658[0n]
Parnassius apollo|WMB4987-14|RVcoll. 14-I534||Italy|658[0n]
Parnassius apollo|WMB5488-14|RVcoll.LD-2548||Italy|658[0n]
Parnassius apollo|EULEP4373-16|RVcoll14V902||Italy|658[0n]
Parnassius apollo|EULEP4374-16|RVcoll15G987||Switzerland|658[0n]
Parnassius apollo|EULEP4376-16|RVcoll15H668||Switzerland|658[0n]
Parnassius apollo|LEATJ1188-16|TLMF Lep 19511||Croatia|658[0n]
Parnassius apollo|PHLAG188-12|TLMF Lep 06498||Austria|658[0n]
Parnassius apollo|LEATJ1190-16|TLMF Lep 19513||Russia|658[0n]
Parnassius apollo|OXB753-15|13-U476||Italy|658[0n]
Parnassius apollo|EULEP4377-16|RVcoll15J483||France|637[0n]
Parnassius apollo|EULEP4375-16|RVcoll15H101||Italy|658[0n]
Parnassius apollo|WMB840-13|RVcoll.07-E238||Italy|645[0n]
Parnassius apollo|LEATG382-14|TLMF Lep 14169||Italy|618[0n]
Parnassius apollo|LEATG102-14|TLMF Lep 13889||Austria|658[0n]
Parnassius apollo|LEATG101-14|TLMF Lep 13888||Austria|658[0n]
Parnassius apollo|PHLAG189-12|TLMF Lep 06499||Austria|658[0n]
Parnassius apollo|PHLAG190-12|TLMF Lep 06500||Austria|658[0n]
Limenitis reducta|OXB715-15|11-J685|Sicily W|Italy|658[0n]
Limenitis reducta|WMB2589-13|RVcoll.12-R159|Sicily|Italy|658[0n]
Limenitis reducta|BIBSA1765-16|LD-3103|Sicily|Italy|658[0n]
Limenitis reducta|WMB2110-13|RVcoll.12-R170|Sicily|Italy|622[0n]
Limenitis reducta|WMB2091-13|RVcoll.12-R069|Sicily|Italy|658[0n]
Limenitis reducta|WMB2748-13|RVcoll.10-C640|Sicily|Italy|658[0n]
Limenitis reducta|WMB2878-14|RVcoll.10-C639|Sicily|Italy|658[0n]
Limenitis reducta|BIBSA654-15|RVcoll. 15-A817||Italy|658[0n]
Limenitis reducta|BIBSA1660-16|15-C738||Italy|658[0n]
Limenitis reducta|BIBSA748-15|LEP-SS-00249||Italy|658[0n]
Limenitis reducta|EZSPC491-09|RVcoll.08-P068||Spain|658[0n]
Limenitis reducta|EZSPC1382-10|RVcoll.09-X518||Spain|658[0n]
Limenitis reducta|EZSPN633-09|RVcoll.08-J734||Spain|658[0n]
Limenitis reducta|WMB4726-14|RVcoll.LD-3087|Capri|Italy|658[0n]
Limenitis reducta|GWORU089-10|BC ZSM Lep 30350||Italy|658[0n]
Limenitis reducta|BIBSA1422-15|15-N092||Italy|658[0n]
Limenitis reducta|WMB2253-13|RVcoll.12-R403|Elba|Italy|658[0n]
Limenitis reducta|BIBSA1620-16|15-C422||Italy|658[0n]
Limenitis reducta|BCLEP285-17|LEP-SS-00732||Italy|658[0n]
Limenitis reducta|GWORO993-09|BC ZSM Lep 32300||Italy|658[0n]
Limenitis reducta|EULEP1196-15|RVcoll.14-F474||Bulgaria|658[0n]
Limenitis reducta|WMB3908-14|RVcoll.12-P329||France|658[0n]
Limenitis reducta|WMB1085-13|RVcoll.10-C739||Italy|658[0n]
Limenitis reducta|BIBSA919-15|16-A010||France|658[0n]
Limenitis reducta|BIBSA1263-15|15-F921||France|658[0n]
Limenitis reducta|WMB3959-14|RVcoll.12-P773||France|658[0n]
Limenitis reducta|WMB3943-14|RVcoll.12-P696||France|658[0n]
Limenitis reducta|WMB3663-14|RVcoll.11-I825||France|658[0n]
Limenitis reducta|WMB3460-14|RVcoll.10-A598||France|658[0n]
Limenitis reducta|WMB097-11|RVcoll.10-B603||France|658[0n]
Limenitis reducta|WMB664-11|RVcoll.11-I373|Argentario|Italy|658[0n]
Limenitis reducta|WMB2924-14|RVcoll.11-I376|Argentario|Italy|658[0n]
Limenitis reducta|EZSPC490-09|RVcoll.08-R316||Spain|658[0n]
Limenitis reducta|EZSPN995-09|RVcoll.08-M066||Spain|658[0n]
Limenitis reducta|EZSPC1391-10|RVcoll.09-X582||Spain|658[0n]
Limenitis reducta|EZSPC1193-10|RVcoll.09-V348||Spain|658[0n]
Limenitis reducta|EZSPC1194-10|RVcoll.09-V350||Spain|658[0n]
Limenitis reducta|WMB2969-14|RVcoll.12-O056|Argentario|Italy|658[0n]
Limenitis reducta|WMB2979-14|RVcoll.12-O171|Corsica|France|658[0n]
Limenitis reducta|WMB3058-14|RVcoll.13-T744|Elba|Italy|658[0n]
Limenitis reducta|WMB3645-14|RVcoll.11-I600||France|658[0n]
Limenitis reducta|WMB1532-13|RVcoll.12-O267|Corsica|France|658[0n]
Limenitis reducta|WMB904-13|RVcoll.09-X866|Sardinia|Italy|658[0n]
Limenitis reducta|WMB1781-13|RVcoll.12-Q137||France|658[0n]
Limenitis reducta|WMB1681-13|RVcoll.12-P488||France|658[0n]
Limenitis reducta|WMB2050-13|RVcoll.12-Q789||Italy|658[0n]
Limenitis reducta|WMB2591-13|RVcoll.12-R195||Italy|658[0n]
Limenitis reducta|WMB2631-13|RVcoll.11-E906|Corsica|France|658[0n]
Limenitis reducta|WMB4149-14|RVcoll.13-S692||Italy|658[0n]

[illegible]
Limenitis reducta|WMB2591-13|RVcoll.12-R195||Italy|658[0n]
Limenitis reducta|WMB2631-13|RVcoll.11-E906|Corsica|France|658[0n]
Limenitis reducta|WMB4149-14|RVcoll.13-S692||Italy|658[0n]
Limenitis reducta|WMB3876-14|RVcoll.11-Y093||Italy|658[0n]
Limenitis reducta|WMB4487-14|RVcoll.14-D934||Italy|658[0n]
Limenitis reducta|WMB4301-14|RVcoll.14-A398||Italy|658[0n]
Limenitis reducta|WMB4396-14|RVcoll.14-B352||Portugal|658[0n]
Limenitis reducta|WMB344-11|RVcoll.11-E718||France|658[0n]
Limenitis reducta|WMB5176-14|RVcoll.13-S523||Italy|658[0n]
Limenitis reducta|WMB5317-14|RVcoll.14-I901|Sardinia|Italy|658[0n]
Limenitis reducta|EULEP5514-17|RVcoll13T858||Italy|658[0n]
Limenitis reducta|EULEP5705-17|RVcoll16L048||Italy|658[0n]
Limenitis reducta|EULEP5710-17|RVcoll16L055||Italy|658[0n]
Limenitis reducta|EULEP5712-17|RVcoll16L057||Italy|658[0n]
Limenitis reducta|EULEP1135-15|RVcoll.14-F283||Serbia|658[0n]
Limenitis reducta|EULEP1206-15|RVcoll.14-F501||Greece|658[0n]
Limenitis reducta|EULEP1450-15|RVcoll.14-G171||Greece|658[0n]
Limenitis reducta|EULEP1707-15|RVcoll.14-H143||Greece|658[0n]
Limenitis reducta|EULEP1733-15|RVcoll.14-H273||Greece|658[0n]
Limenitis reducta|BIBSA1227-15|15-L967||Italy|658[0n]
Limenitis reducta|BIBSA981-15|14-W177||Italy|658[0n]
Limenitis reducta|BIBSA1301-15|LD-2489|Sardinia|Italy|658[0n]
Limenitis reducta|BIBSA1012-15|15-C123||Italy|658[0n]
Limenitis reducta|LEASS999-17|TLMF Lep 22552||Austria|658[0n]
Limenitis reducta|LEASS891-17|KLM Lep 08396||Austria|658[0n]
Limenitis reducta|LEASS689-17|TLMF Lep 22337||Austria|658[0n]
Limenitis reducta|OXB584-15|14-N973||Italy|658[0n]
Limenitis reducta|OXB713-15|13-T906||Italy|658[0n]
Limenitis reducta|OXB1053-15|15-A598||Italy|658[0n]
Limenitis reducta|OXB304-15|RVcoll.14-N018||Italy|658[0n]
Limenitis reducta|OXB1163-15|Rvcoll_16-A050||Italy|658[0n]
Limenitis reducta|ABOLD661-17|TLMF Lep 21737||Austria|658[0n]
Limenitis reducta|EZSPN924-09|RVcoll.08-L871||Spain|653[0n]
Limenitis reducta|WMB366-11|RVcoll.11-E973||France|658[0n]
Limenitis reducta|BIBSA1297-15|14-E304|Corsica|France|658[0n]
Limenitis reducta|EULEP4138-16|RVcoll14E303|Corsica|France|632[0n]
Limenitis reducta|EULEP4137-16|RVcoll10B317||Bulgaria|632[0n]
Limenitis reducta|WMB2829-13|RVcoll.12-O484|Sardinia|Italy|628[0n]
Limenitis reducta|EZSPN850-09|RVcoll.08-L695||Spain|630[0n]
Limenitis reducta|EZSPC489-09|RVcoll.08-H272||Spain|658[0n]
Limenitis reducta|EZRMN034-08|RVcoll.08-M554||Romania|658[0n]
Limenitis reducta|GWOTF690-12|BC ZSM Lep 62396||Croatia|658[0n]
Limenitis reducta|LEATG390-14|TLMF Lep 14177||Italy|658[0n]
Limenitis reducta|LEATI002-15|TLMF Lep 17387||Austria|658[0n]
Limenitis populi|EULEP1641-15|RVcoll.14-H987||Sweden|658[0n]
Limenitis populi|EULEP382-14|MM23867||Finland|658[0n]
Limenitis populi|LEFIC084-10|MM03383||Finland|658[0n]
Limenitis populi|LEFIJ534-10|MM17159||Finland|658[0n]
Limenitis populi|ABOLD660-17|TLMF Lep 21736||Austria|658[0n]
Limenitis populi|EULEP2920-15|RVcoll.14-U873||Serbia|658[0n]
Limenitis populi|EULEP342-14|MM23827||Lithuania|658[0n]
Limenitis populi|EULEP4869-16|RVcoll16H921||Poland|658[0n]
Limenitis populi|EZROM247-08|RV-07-C965||Romania|658[0n]
Limenitis populi|GWOSA734-10|BC ZSM Lep 35745||Germany|658[0n]
Limenitis populi|FBLMU110-09|BC ZSM Lep 25620||Germany|658[0n]
Limenitis populi|LEFID365-10|MM06273||Finland|658[0n]
Limenitis populi|LEATG431-14|TLMF Lep 14218||Italy|658[0n]
Limenitis populi|LEATG432-14|TLMF Lep 14219||Italy|658[0n]
Limenitis camilla|EULEP2424-15|RVcoll.14-V208||Belgium|658[0n]
Limenitis camilla|OXB1232-15|Rvcoll_15-M667||France|658[0n]
Limenitis camilla|EULEP4134-16|RVcoll15G077||France|658[0n]
Limenitis camilla|EULEP4132-16|RVcoll15H720||Switzerland|658[0n]
Limenitis camilla|EULEP4902-16|RVcoll16J605||Russia|631[0n]
Limenitis camilla|LEATG386-14|TLMF Lep 14173||Italy|658[0n]
Limenitis camilla|LEATG385-14|TLMF Lep 14172||Italy|658[0n]
Limenitis camilla|ABOLB042-15|TLMF Lep 17047||Italy|658[0n]
Limenitis camilla|EZRMN029-08|RVcoll.06-M856||Romania|658[1n]
Limenitis camilla|EULEP090-14|RVcoll.08-P977||Romania|658[0n]
Limenitis camilla|EZRMN030-08|RVcoll.08-M504||Romania|658[0n]
Limenitis camilla|EZROM245-08|RV-06-M875||Romania|658[0n]
Limenitis camilla|EZROM244-08|RV-06-M853||Romania|658[0n]
Limenitis camilla|EZSPN1059-11|Rvcoll.08-P348.1||Spain|629[0n]
Limenitis camilla|ABOLB037-15|TLMF Lep 17042||Austria|658[2n]
Limenitis camilla|OXB1258-15|Rvcoll_15-M114||France|658[0n]
Limenitis camilla|BIBSA737-15|LEP-SS-00264||Italy|658[0n]
Limenitis camilla|LEFIJ4717-16|ZMBSU-02116||Belarus|658[0n]
Limenitis camilla|EULEP2425-15|RVcoll.14-V210||Belgium|616[0n]
Limenitis camilla|EULEP5189-17|RVcoll16J260||Germany|658[0n]
Limenitis camilla|EULEP5044-16|RVcoll16J040||Slovakia|658[0n]
Limenitis camilla|LOWA857-06|2005-LOWA-857||Russia|658[0n]
Limenitis camilla|WMB4806-14|RVcoll. 14-I353||Italy|658[0n]
Limenitis camilla|WMB3296-14|RVcoll.08-P067.1||Spain|658[0n]
Limenitis camilla|EZSPC913-10|RVcoll.09-V822||Spain|658[0n]
Limenitis camilla|EZSPC911-10|RVcoll.09-V808||Spain|658[0n]
Limenitis camilla|EZSPC860-10|RVcoll.09-T052||Spain|658[0n]
Limenitis camilla|EZSPC765-10|RVcoll.08-L534||Spain|658[0n]
Limenitis camilla|EZSPM777-12|Rvcoll. 050111LP98||Spain|658[0n]
Limenitis camilla|EZSPM265-09|RVcoll.08-R480||Spain|658[0n]
Limenitis camilla|EZSPN278-09|RVcoll.07-W210|Genit. examined|Spain|658[0n]
Limenitis camilla|EZROM730-08|RV-07-F310||Spain|658[0n]
Limenitis camilla|EZRMN031-08|RVcoll.08-M505||Romania|658[0n]
Limenitis camilla|EZSPC486-09|RVcoll.08-M917||Spain|658[0n]
Limenitis camilla|EZRMN032-08|RVcoll.08-M540||Romania|658[0n]
Limenitis camilla|LEFIJ1008-11|MM21248||Estonia|658[0n]
Limenitis camilla|ABOLB035-15|TLMF Lep 17040||Austria|658[0n]
Limenitis camilla|PHLAW052-13|TLMF Lep 09849||Austria|658[0n]
Boloria selene|EULEP4957-16|RVcoll16I247||Poland|658[0n]
Boloria selene|EULEP4824-16|RVcoll16H720||Belgium|658[0n]
Boloria selene|EULEP3549-16|RVcoll15G042||France|658[0n]
Boloria selene|EZROM861-08|RVcoll.08-M344||Romania|658[0n]
Boloria selene|OXB611-15|14-O000||Italy|658[0n]
Boloria selene|EZROM101-08|RV-07-C902||Romania|658[0n]
[illegible]

Boloria selene|EZROM861-08|RVcoll.08-M344||Romania|658[0n]
Boloria selene|OXB611-15|14-O000||Italy|658[0n]
Boloria selene|EZROM101-08|RV-07-C902||Romania|658[0n]
Boloria selene|EZROM097-08|RV-06-K635||Romania|658[0n]
Boloria selene|EULEP796-15|RVcoll.12-Z281||Sweden|658[0n]
Boloria selene|LEFIJ547-10|MM17172||Finland|658[0n]
Boloria selene|LEFIB997-10|MM03195||Finland|657[0n]
Boloria selene|EULEP4735-16|RVcoll16G520||Sweden|658[0n]
Boloria selene|OXB283-15|OXB-TGS-966||United Kingdom|658[0n]
Boloria selene|OXB191-15|OXB-TGS-013||United Kingdom|658[0n]
Boloria selene|WMB4023-14|RVcoll.12-R523||United Kingdom|658[0n]
Boloria selene|OXB1430-16|OXB-TGS-1204||United Kingdom|658[0n]
Boloria selene|OXB1426-16|OXB-TGS-1200||United Kingdom|658[0n]
Boloria selene|LEATJ1306-16|TLMF Lep 19629||Austria|658[0n]
Boloria selene|WMB3335-14|RVcoll.08-P781||Spain|658[0n]
Boloria selene|WMB3330-14|RVcoll.08-P765||Spain|658[0n]
Boloria selene|EZSPC855-10|RVcoll.08-R445||Spain|658[0n]
Boloria selene|EZSPM240-09|RVcoll.08-R444||Spain|658[0n]
Boloria selene|EZSPN790-09|RVcoll.08-J893||Spain|658[0n]
Boloria selene|WMB3414-14|RVcoll.09-X006||Spain|658[0n]
Boloria selene|EZSPN062-09|RVcoll.09-V690||Spain|658[0n]
Boloria selene|EZSPC675-09|RVcoll.08-M969||France|658[0n]
Boloria selene|OXB1396-15|Rvcoll_15-M763||France|658[0n]
Boloria selene|WMB879-13|RVcoll.09-X281||France|658[0n]
Boloria selene|EZSPC865-10|RVcoll.09-T082||Spain|658[0n]
Boloria selene|EZSPN874-09|RVcoll.08-L765||Spain|656[0n]
Boloria selene|EZSPN497-09|RVcoll.08-H943||Spain|658[0n]
Boloria selene|EZSPN831-09|RVcoll.08-L649||Spain|658[0n]
Boloria selene|EZSPC676-09|RVcoll.08-M970||France|658[0n]
Boloria selene|EZSPC654-09|RVcoll.07-C659||France|658[0n]
Boloria selene|LEFIC043-10|MM03274||Finland|658[0n]
Boloria selene|LEATF456-14|TLMF Lep 13768||Italy|654[0n]
Boloria selene|GWORR667-10|BC ZSM Lep 32163||Germany|658[0n]
Boloria selene|LEATG040-14|TLMF Lep 13827||Austria|658[0n]
Boloria selene|ABOLB316-15|TLMF Lep 17321||Austria|658[0n]
Boloria selene|EULEP5016-16|RVcoll16I913||Slovakia|658[0n]
Boloria selene|EZROM100-08|RV-07-D973||Romania|658[0n]
Boloria selene|EZROM099-08|RV-07-C421||Romania|657[0n]
Boloria selene|LEATF457-14|TLMF Lep 13769||Italy|658[0n]
Boloria selene|GWORR668-10|BC ZSM Lep 32164||Germany|658[0n]
Boloria selene|LON001-08|NHMO-06001||Norway|657[0n]
Boloria selene|LON867-11|NHMO Lep09065||Norway|658[0n]
Boloria selene|LON210-08|NHMO-06222||Norway|657[0n]
Boloria selene|GWORA2494-09|BC ZSM Lep 30706||Germany|658[0n]
Boloria selene|LEFIJ545-10|MM17170||Finland|658[0n]
Boloria selene|GWORK309-09|BC ZSM Lep 21544||Germany|658[0n]
Boloria selene|EZROM860-08|RVcoll.08-M223||Romania|658[0n]
Boloria selene|EZROM615-08|RV-07-D313||Romania|658[0n]
Boloria selene|EZSPN588-09|RVcoll.08-J151||Portugal|658[0n]
Boloria selene|LOWA756-06|2005-LOWA-756||Russia|658[0n]
Boloria selene|EULEP3550-16|RVcoll15H005||Switzerland|658[0n]
Boloria selene|EULEP3551-16|RVcoll15H212||Switzerland|658[0n]
Boloria selene|EULEP3552-16|RVcoll15H389||Switzerland|658[0n]
Boloria selene|EULEP542-15|RVcoll.08-L300||Denmark|658[0n]
Boloria selene|EULEP585-15|RVcoll.10-A697||Estonia|658[0n]
Boloria selene|EULEP2446-15|RVcoll.14-V256||Switzerland|658[0n]
Boloria selene|EULEP2204-15|RVcoll.14-N364||Ukraine|658[0n]
Boloria selene|GBLAB325-13|BC ZSM Lep 72626||Germany|658[0n]
Boloria selene|OXB1428-16|OXB-TGS-1202||United Kingdom|658[0n]
Boloria selene|OXB1429-16|OXB-TGS-1203||United Kingdom|658[0n]
Boloria selene|OXB350-15|RVcoll.14-N064||Italy|658[0n]
Boloria selene|OXB1395-15|Rvcoll_15-M762||France|658[3n]
Boloria selene|FBLMU471-09|BC ZSM Lep 27121||Germany|630[0n]
Boloria selene|PHLSA700-11|TLMF Lep 06155||Austria|658[0n]
Boloria selene|ABOLD601-17|TLMF Lep 21677||Austria|658[0n]
Boloria selene|OXB1427-16|OXB-TGS-1201||United Kingdom|658[0n]
Boloria selene|EULEP5182-17|RVcoll16H352||Norway|658[0n]
Boloria selene|EULEP4754-16|RVcoll16G917||Sweden|658[0n]
Boloria selene|EZROM098-08|RV-06-K642||Romania|658[0n]
Boloria selene|EZROM573-08|RV-07-C336||Romania|658[0n]
Boloria selene|LEATG039-14|TLMF Lep 13826||Austria|658[0n]
Boloria thore|EULEP4791-16|RVcoll16H498||Sweden|658[0n]
Boloria thore|EULEP3558-16|RVcoll15H699||Switzerland|658[0n]
Boloria thore|LEASS877-17|KLM Lep 08382||Austria|658[0n]
Boloria thore|GWORA2495-09|BC ZSM Lep 30707||Germany|658[0n]
Boloria thore|LEATJ1244-16|TLMF Lep 19567||Austria|658[0n]
Boloria thore|PHLAB292-10|TLMF Lep 01092||Switzerland|603[0n]
Boloria thore|EULEP3559-16|RVcoll15I182||Austria|658[0n]
Boloria thore|LEATG518-14|TLMF Lep 14305||Italy|658[0n]
Boloria thore|LEATG517-14|TLMF Lep 14304||Italy|658[0n]
Boloria thore|BIBSA1112-15|15-L832||Italy|658[0n]
Boloria thore|LEFIA740-10|MM04094||Finland|658[1n]
Boloria thore|LEFIA739-10|MM04093||Finland|627[0n]
Boloria thore|LEATG016-14|TLMF Lep 13803||Austria|658[0n]
Boloria thore|EULEP3561-16|RVcoll15O015||Italy|658[0n]
Boloria thore|EULEP5184-17|RVcoll16H394||Sweden|658[0n]
Boloria thore|LEFIC095-10|MM03408||Finland|658[0n]
Boloria thore|FBLMX199-11|BC ZSM Lep 50410||Germany|658[0n]
Boloria thore|FBLMU475-09|BC ZSM Lep 27125||Germany|658[0n]
Boloria thore|GWORO851-09|BC ZSM Lep 30543||Germany|658[0n]
Boloria thore|GWORK566-09|BC ZSM Lep 21896||Germany|658[0n]
Boloria thore|GWOSK893-11|BC ZSM Lep 49394||Germany|658[0n]
Boloria thore|WMB5110-14|RVcoll. 14-L241||Italy|658[0n]
Boloria thore|EULEP3555-16|RVcoll14W721||Italy|658[0n]
Boloria thore|EULEP3556-16|RVcoll15G789||Italy|658[0n]
Boloria thore|EULEP3557-16|RVcoll15H440||Switzerland|658[0n]
Boloria thore|EULEP3560-16|RVcoll15I670||Austria|658[0n]
Boloria thore|EULEP4715-16|RVcoll16B324||Sweden|658[0n]
Boloria thore|EULEP5183-17|RVcoll16H362||Sweden|658[0n]
Boloria thore|EULEP2451-15|RVcoll.14-V273||Switzerland|658[0n]
Boloria thore|EULEP2678-15|RVcoll.14-N762||Norway|658[0n]
Boloria thore|LEASS878-17|KLM Lep 08383||Austria|658[0n]
Boloria thore|ABOLD594-17|TLMF Lep 21670||Austria|658[0n]

Boloria thore|EULEP2678-15|RVcoll.14-N762||Norway|658[0n]
Boloria thore|LEASS878-17|KLM Lep 08383||Austria|658[0n]
Boloria thore|ABOLD594-17|TLMF Lep 21670||Austria|658[0n]
Boloria thore|EULEP2677-15|RVcoll.14-N761||Norway|603[0n]
Boloria thore|GRAFW2418-13|LE-JS-2013-0009||Finland|622[0n]
Boloria thore|GWORR673-10|BC ZSM Lep 32169||Germany|636[1n]
Boloria thore|PHLAB1031-10|TLMF Lep 01831||Switzerland|658[0n]
Boloria thore|LEATG047-14|TLMF Lep 13834||Austria|658[0n]
Boloria thore|PHLAB1030-10|TLMF Lep 01830||Switzerland|658[0n]
Boloria thore|PHLAB1032-10|TLMF Lep 01832||Switzerland|658[0n]
Boloria thore|PHLSA699-11|TLMF Lep 06154||Austria|658[0n]
Boloria thore|LEATJ1245-16|TLMF Lep 19568||Austria|658[0n]
Boloria thore|PHLAC686-10|TLMF Lep 02721||Switzerland|658[0n]
Boloria frigga|EULEP5174-17|RVcoll16H073||Sweden|658[0n]
Boloria frigga|EULEP5173-17|RVcoll16H051||Sweden|658[0n]
Boloria frigga|EULEP390-14|MM23875||Finland|658[0n]
Boloria frigga|EULEP4747-16|RVcoll16G756||Sweden|658[0n]
Boloria frigga|EULEP5181-17|RVcoll16H285||Norway|658[0n]
Boloria frigga|EULEP332-14|MM23817||Estonia|658[0n]
Boloria frigga|EULEP387-14|MM23872||Finland|658[0n]
Boloria frigga|LON466-08|NHMO-08117||Norway|657[0n]
Boloria frigga|EULEP5176-17|RVcoll16H119||Norway|658[0n]
Boloria frigga|LEFIJ546-10|MM17171||Finland|658[0n]
Boloria improba|LON934-12|NHMO Lep2010.007||Norway|658[0n]
Boloria improba|EULEP4790-16|RVcoll16H488||Sweden|658[0n]
Boloria improba|LEFIG491-10|MM14556||Finland|658[0n]
Boloria improba|LEFIG489-10|MM14554||Finland|658[0n]
Boloria improba|LEFIG490-10|MM14555||Finland|658[0n]
Boloria improba|LEFIG488-10|MM14553||Finland|658[0n]
Boloria frigga|LEFIG159-10|MM14016||Finland|658[0n]
Boloria frigga|LEFIG146-10|MM13985||Finland|658[0n]
Boloria frigga|EULEP389-14|MM23874||Finland|658[0n]
Boloria frigga|EULEP388-14|MM23873||Finland|658[0n]
Boloria frigga|LEFIG145-10|MM13984||Finland|658[0n]
Boloria dia|EULEP2374-15|RVcoll.14-V069||Ukraine|608[0n]
Boloria dia|FBLMV675-09|BC ZSM Lep 28655||Germany|658[0n]
Boloria dia|GWORR671-10|BC ZSM Lep 32167||Germany|622[0n]
Boloria dia|LEFID124-10|MM05860||Estonia|658[0n]
Boloria dia|LEASS583-17|TLMF Lep 22231||Austria|658[0n]
Boloria dia|LEASS876-17|KLM Lep 08381||Austria|658[0n]
Boloria dia|OXB1178-15|Rvcoll_16-A065||Italy|658[0n]
Boloria dia|BIBSA745-15|LEP-SS-00272||Italy|658[0n]
Boloria dia|EULEP5708-17|RVcoll16L053||Italy|658[0n]
Boloria dia|WMB4952-14|RVcoll. 14-I499||Italy|658[0n]
Boloria dia|LEATI047-15|TLMF Lep 17432||Austria|658[0n]
Boloria dia|ABOLD077-16|TLMF Lep 21155||Austria|658[0n]
Boloria dia|LEEUA529-11|MM20588||Latvia|655[0n]
Boloria dia|EZROM849-08|RVcoll.08-M354||Romania|658[0n]
Boloria dia|EZROM850-08|RVcoll.06-N012||Romania|658[0n]
Boloria dia|EZROM851-08|RVcoll.07-C423||Romania|658[0n]
Boloria dia|EZROM852-08|RVcoll.07-D504||Romania|658[0n]
Boloria dia|EZROM853-08|RVcoll.07-D562||Romania|658[0n]
Boloria dia|EZROM854-08|RVcoll.07-D909||Romania|658[0n]
Boloria dia|EZROM855-08|RVcoll.07-D922||Romania|658[0n]
Boloria dia|EZROM091-08|RV-06-K536||Romania|658[0n]
Boloria dia|EZROM092-08|RV-06-K538||Romania|658[0n]
Boloria dia|EZROM093-08|RV-06-M957||Romania|658[0n]
Boloria dia|EZROM094-08|RV-07-C954||Romania|658[0n]
Boloria dia|EZSPN914-09|RVcoll.08-L854||Spain|658[0n]
Boloria dia|EZSPC218-09|RVcoll.08-L416||Spain|655[0n]
Boloria dia|EZSPC249-09|RVcoll.08-P223||Spain|658[0n]
Boloria dia|EZSPC253-09|RVcoll.08-P334||Spain|655[0n]
Boloria dia|EZROM709-08|RV-06-H866||Spain|658[0n]
Boloria dia|EZSPN789-09|RVcoll.08-J889||Spain|658[0n]
Boloria dia|EZSPN923-09|RVcoll.08-L870||Spain|658[0n]
Boloria dia|EZSPM795-12|Rvcoll. 070611MH85||Spain|658[0n]
Boloria dia|EZSPC778-10|RVcoll.08-L853||Spain|658[0n]
Boloria dia|EZSPC827-10|RVcoll.08-P776||Spain|658[0n]
Boloria dia|WMB3679-14|RVcoll.11-J214||France|658[0n]
Boloria dia|WMB1794-13|RVcoll.12-Q312||France|658[0n]
Boloria dia|WMB1648-13|RVcoll.12-O978||France|658[0n]
Boloria dia|WMB5155-14|RVcoll.12-P906||France|658[0n]
Boloria dia|EULEP3500-16|RVcoll15H741||Switzerland|658[0n]
Boloria dia|EULEP3501-16|RVcoll15Q090||Russia|658[0n]
Boloria dia|EULEP5039-16|RVcoll16J012||Slovakia|658[0n]
Boloria dia|EULEP5056-16|RVcoll16J102||Czech Republic|658[0n]
Boloria dia|EULEP4932-16|RVcoll16I011||Poland|658[0n]
Boloria dia|EULEP135-14|RVcoll.10-C381||Romania|658[0n]
Boloria dia|EULEP321-14|MM23806||Latvia|658[0n]
Boloria dia|EULEP346-14|MM23831||Lithuania|658[0n]
Boloria dia|EULEP1132-15|RVcoll.14-F262||Serbia|658[0n]
Boloria dia|EULEP1178-15|RVcoll.14-F417||Bulgaria|658[0n]
Boloria dia|EULEP888-15|RVcoll.14-C527||Greece|658[0n]
Boloria dia|EULEP2260-15|RVcoll.14-N444||Ukraine|658[0n]
Boloria dia|BIBSA1863-17|RVcoll16C756||Italy|658[0n]
Boloria dia|BIBSA1864-17|RVcoll16C757||Italy|658[0n]
Boloria dia|GBLAB321-13|BC ZSM Lep 72622||Germany|658[0n]
Boloria dia|GBLAA349-14|BC ZSM Lep 80345||Germany|658[0n]
Boloria dia|GBLAD222-14|BC ZSM Lep 78793||Germany|658[0n]
Boloria dia|GBLAF769-14|BC ZSM Lep 82855||Germany|658[0n]
Boloria dia|GBLAA1377-15|BC ZSM Lep 87073||Germany|658[0n]
Boloria dia|LEASS483-17|TLMF Lep 22131||Austria|658[0n]
Boloria dia|OXB1569-16|OXB-TGS-1287||France|658[0n]
Boloria dia|OXB1350-15|Rvcoll_15-M702||France|658[0n]
Boloria dia|OXB1401-15|Rvcoll_15-M768||France|658[0n]
Boloria dia|EZSPC196-09|RVcoll.06-G467||Spain|654[0n]
Boloria dia|EULEP2166-15|RVcoll.14-K087||Switzerland|642[0n]
Boloria dia|LENOA1373-11|LN-BD1373||France|631[0n]
Boloria dia|WMB1686-13|RVcoll.12-P531||France|627[0n]
Boloria dia|PHLAH474-12|TLMF Lep 08293||Austria|615[0n]
Boloria dia|LEATG056-14|TLMF Lep 13843||Austria|658[0n]
Boloria freija|EULEP484-14|KN00846||Russia|658[0n]
[illegible]

Boloria dia|PHLAH474-12|TLMF Lep 08295||Austria|615[0n]
Boloria dia|LEATG056-14|TLMF Lep 13843||Austria|658[0n]
Boloria freija|EULEP484-14|KN00846||Russia|658[0n]
Boloria freija|EULEP486-14|KN00848||Russia|658[0n]
Boloria freija|EULEP485-14|KN00847||Russia|658[1n]
Boloria freija|LEFIB217-10|MM00671||Finland|658[0n]
Boloria freija|EULEP5178-17|RVcoll16H170||Norway|658[0n]
Boloria freija|EULEP394-14|MM23879||Finland|658[0n]
Boloria freija|EULEP393-14|MM23878||Finland|658[0n]
Boloria freija|EULEP392-14|MM23877||Finland|658[0n]
Boloria freija|EULEP391-14|MM23876||Finland|658[0n]
Boloria freija|EULEP4765-16|RVcoll16H102||Sweden|658[0n]
Boloria freija|LEFID419-10|MM06344||Finland|658[0n]
Boloria freija|LEFIA741-10|MM04095||Finland|658[0n]
Boloria freija|LEFIA742-10|MM04096||Finland|658[0n]
Boloria polaris|LEFIJ2011-13|MM22827||Finland|658[0n]
Boloria polaris|EULEP438-14|KN00800||Russia|658[0n]
Boloria polaris|EULEP395-14|MM23880||Finland|658[0n]
Boloria polaris|LON184-08|NHMO-06185||Norway|657[0n]
Boloria polaris|LEFID422-10|MM06348||Finland|658[0n]
Boloria polaris|LEFID421-10|MM06347||Finland|658[0n]
Boloria polaris|LEFID420-10|MM06346||Finland|658[0n]
Boloria chariclea|EULEP4776-16|RVcoll16H296||Norway|658[0n]
Boloria angarensis|EULEP3497-16|RVcoll14V785||Russia|658[1n]
Boloria chariclea|EULEP4787-16|RVcoll16H439||Sweden|658[0n]
Boloria angarensis|EULEP3496-16|RVcoll14V784||Russia|658[0n]
Boloria angarensis|EULEP3495-16|RVcoll14V783||Russia|658[0n]
Boloria chariclea|LEFID418-10|MM06342||Finland|658[0n]
Boloria chariclea|LEFID417-10|MM06341||Finland|658[0n]
Boloria chariclea|LON183-08|NHMO-06184||Norway|657[0n]
Boloria chariclea|LEFIG495-10|MM14570||Finland|658[0n]
Boloria chariclea|LEFIG494-10|MM14569||Finland|658[0n]
Boloria titania|WMB1636-13|RVcoll.12-O847||France|658[0n]
Boloria titania|BIBSA188-15|RVcoll.14-D976||Italy|658[0n]
Boloria titania|WMB2667-13|RVcoll.10-B820||France|627[0n]
Boloria titania|PHLAF622-11|TLMF Lep 05792||France|658[0n]
Boloria titania|EULEP3562-16|RVcoll15G577||Switzerland|658[0n]
Boloria titania|LEATF453-14|TLMF Lep 13765||Austria|658[0n]
Boloria titania|EULEP2680-15|RVcoll.14-N764||Serbia|658[0n]
Boloria titania|EULEP2679-15|RVcoll.14-N763||Serbia|658[0n]
Boloria titania|EULEP3570-16|RVcoll15Q042||Serbia|658[0n]
Boloria titania|EZROM055-08|RV-06-N010||Romania|658[0n]
Boloria titania|EZROM806-08|RVcoll.08-M604||Romania|658[0n]
Boloria titania|EZROM805-08|RVcoll.08-M603||Romania|658[0n]
Boloria titania|EZROM804-08|RVcoll.08-M602||Romania|658[0n]
Boloria titania|EZROM803-08|RVcoll.08-M601||Romania|658[0n]
Boloria titania|EZRMN428-09|RVcoll.09-V681||Romania|658[0n]
Boloria titania|BIBSA369-15|RVcoll.14-I037||Italy|658[0n]
Boloria titania|BIBSA366-15|RVcoll.14-I034||Italy|658[0n]
Boloria titania|EULEP3567-16|RVcoll15I663||Austria|658[0n]
Boloria titania|EULEP3566-16|RVcoll15H803||Italy|658[0n]
Boloria titania|EULEP3565-16|RVcoll15H350||Switzerland|658[0n]
Boloria titania|PHLAI487-13|TLMF Lep 09049||Austria|658[0n]
Boloria titania|PHLAB293-10|TLMF Lep 01093||Switzerland|658[0n]
Boloria titania|PHLAA705-09|TLMF Lep 00745||Austria|658[0n]
Boloria titania|GWORR672-10|BC ZSM Lep 32168||Germany|658[0n]
Boloria titania|FBLMU472-09|BC ZSM Lep 27122||Germany|658[0n]
Boloria titania|GWORA2496-09|BC ZSM Lep 30708||Germany|658[0n]
Boloria titania|ODOPE243-11|BC ZSM Lep 53114||Germany|658[0n]
Boloria titania|WMB1852-13|RVcoll.11-I966||France|658[0n]
Boloria titania|WMB5119-14|RVcoll. 14-L250||Italy|658[0n]
Boloria titania|EULEP3563-16|RVcoll15G970||Switzerland|658[0n]
Boloria titania|EULEP3568-16|RVcoll15I774||Austria|658[0n]
Boloria titania|EULEP2126-15|RVcoll.14-J928||France|658[0n]
Boloria titania|EULEP3569-16|RVcoll15J581||France|658[0n]
Boloria titania|EULEP649-15|RVcoll.11-J153||Switzerland|658[0n]
Boloria titania|EULEP2467-15|RVcoll.14-V293||Switzerland|658[0n]
Boloria titania|BIBSA1211-15|15-M335||Italy|658[0n]
Boloria titania|LEASS776-17|TLMF Lep 22424||Austria|658[0n]
Boloria titania|FBLMX197-11|BC ZSM Lep 50408||Germany|658[0n]
Boloria titania|OXB828-15|14-U791||Italy|658[0n]
Boloria titania|OXB609-15|14-N998||Italy|658[0n]
Boloria titania|EULEP3564-16|RVcoll15H188||Switzerland|638[0n]
Boloria titania|PHLAA681-09|TLMF Lep 00721||Austria|634[0n]
Boloria titania|LEATD455-13|TLMF Lep 13102||Italy|658[0n]
Boloria titania|ABOLD040-16|TLMF Lep 21118||Austria|658[0n]
Boloria titania|PHLAB1033-10|TLMF Lep 01833||Switzerland|658[0n]
Boloria titania|PHLAB1035-10|TLMF Lep 01835||Switzerland|658[0n]
Boloria titania|PHLAC685-10|TLMF Lep 02720||Switzerland|658[0n]
Boloria titania|LEATF455-14|TLMF Lep 13767||Italy|658[0n]
Boloria titania|PHLAB1034-10|TLMF Lep 01834||Switzerland|658[0n]
Boloria euphrosyne|EULEP3515-16|RVcoll15I249||Austria|658[0n]
Boloria euphrosyne|EULEP3516-16|RVcoll15I367||Austria|658[0n]
Boloria euphrosyne|EULEP901-15|RVcoll.14-C732||Bulgaria|658[0n]
Boloria euphrosyne|EULEP3506-16|RVcoll14W677||Italy|658[0n]
Boloria euphrosyne|EULEP3504-16|RVcoll10B418||Bulgaria|658[0n]
Boloria euphrosyne|WMB5021-14|RVcoll. 14-I568||Italy|658[0n]
Boloria euphrosyne|BIBSA1111-15|15-L818||Italy|658[0n]
Boloria euphrosyne|EULEP1932-15|RVcoll.14-B900||Bosnia and Herzegovina|658[0n]
Boloria euphrosyne|EULEP1926-15|RVcoll.14-B868||Bosnia and Herzegovina|658[0n]
Boloria euphrosyne|EULEP1266-15|RVcoll.14-F659||Greece|658[0n]
Boloria euphrosyne|EULEP1066-15|RVcoll.14-E971||Serbia|658[0n]
Boloria euphrosyne|EULEP1013-15|RVcoll.14-E647||Romania|658[0n]
Boloria euphrosyne|EULEP3508-16|RVcoll15C912||Romania|658[0n]
Boloria euphrosyne|EZROM859-08|RVcoll.08-M645|Genit. examined|Romania|658[0n]
Boloria euphrosyne|EULEP3507-16|RVcoll15C573||Italy|658[0n]
Boloria euphrosyne|WMB1970-13|RVcoll.11-I194||Italy|658[0n]
Boloria euphrosyne|BIBSA730-15|LD-2995||Italy|658[0n]
Boloria euphrosyne|WMB636-11|RVcoll.11-I133||Italy|658[0n]
Boloria euphrosyne|WMB886-13|RVcoll.09-X781|Sicily|Italy|658[0n]
Boloria euphrosyne|BIBSA663-15|RVcoll. 15-A826||Italy|658[0n]
Boloria euphrosyne|BIBSA087-14|LEP-SS-00087||Italy|658[0n]
Boloria euphrosyne|BIBSA088-14|LEP-SS-00088||Italy|658[0n]

Boloria euphrosyne|BIBSA663-15|RVcoll. 15-A826||Italy|658[0n]
Boloria euphrosyne|BIBSA087-14|LEP-SS-00087||Italy|658[0n]
Boloria euphrosyne|BIBSA088-14|LEP-SS-00088||Italy|658[0n]
Boloria euphrosyne|BIBSA666-15|RVcoll. 15-A829||Italy|658[0n]
Boloria euphrosyne|BIBSA636-15|RVcoll. 15-A799||Italy|636[0n]
Boloria euphrosyne|BIBSA586-15|LEP-SS-00206||Italy|631[0n]
Boloria euphrosyne|BIBSA585-15|LEP-SS-00205||Italy|658[0n]
Boloria euphrosyne|BIBSA584-15|LEP-SS-00204||Italy|658[0n]
Boloria euphrosyne|BIBSA583-15|LEP-SS-00203||Italy|658[0n]
Boloria euphrosyne|WMB2803-13|RVcoll.12-M283||Italy|658[0n]
Boloria euphrosyne|GWORZ024-10|BC ZSM Lep 30380||Italy|658[0n]
Boloria euphrosyne|BIBSA1369-15|15-M912||Italy|658[0n]
Boloria euphrosyne|BIBSA1349-15|15-M845||Italy|658[0n]
Boloria euphrosyne|OXB1022-15|15-A972||Italy|658[0n]
Boloria euphrosyne|OXB1006-15|15-A956||Italy|658[0n]
Boloria euphrosyne|BIBSA186-15|RVcoll.14-D974||Italy|658[0n]
Boloria euphrosyne|WMB937-13|RVcoll.10-A600||France|658[0n]
Boloria euphrosyne|WMB857-13|RVcoll.09-T265||France|658[0n]
Boloria euphrosyne|GBLAA1947-15|BC ZSM Lep 89068||Italy|658[0n]
Boloria euphrosyne|WMB5231-14|RVcoll.13-U050||Italy|658[0n]
Boloria euphrosyne|WMB5209-14|RVcoll.13-T948||Italy|658[0n]
Boloria euphrosyne|WMB4172-14|RVcoll.13-S747||Italy|658[0n]
Boloria euphrosyne|WMB4175-14|RVcoll.13-S768||Italy|658[0n]
Boloria euphrosyne|OXB868-15|15-A535||Italy|630[0n]
Boloria euphrosyne|WMB2229-13|RVcoll.11-Y068||Italy|632[0n]
Boloria euphrosyne|WMB279-11|RVcoll.10-A556||France|658[0n]
Boloria euphrosyne|WMB4848-14|RVcoll.14-I395||Italy|634[0n]
Boloria euphrosyne|WMB903-13|RVcoll.09-X865||Italy|658[0n]
Boloria euphrosyne|GWORR669-10|BC ZSM Lep 32165||Germany|658[0n]
Boloria euphrosyne|EZSPC880-10|RVcoll.09-T138||Spain|658[0n]
Boloria euphrosyne|EZSPC879-10|RVcoll.09-T137||Spain|658[0n]
Boloria euphrosyne|BIBSA184-15|RVcoll.14-D972||Italy|658[0n]
Boloria euphrosyne|BIBSA962-15|13-U325||Italy|658[0n]
Boloria euphrosyne|EULEP3505-16|RVcoll13U259||Italy|658[0n]
Boloria euphrosyne|EZSPC652-09|RVcoll.07-C646||France|658[0n]
Boloria euphrosyne|EULEP044-14|RVcoll.08-H024||Romania|658[0n]
Boloria euphrosyne|EZROM095-08|RV-07-D221||Romania|656[0n]
Boloria euphrosyne|EZROM096-08|RV-07-D234||Romania|621[0n]
Boloria euphrosyne|EZROM856-08|RVcoll.08-M343||Romania|658[0n]
Boloria euphrosyne|EZROM614-08|RV-06-N006||Romania|658[0n]
Boloria euphrosyne|EULEP086-14|RVcoll.08-P972||Romania|658[0n]
Boloria euphrosyne|EZROM301-08|RV-07-E663||Romania|658[0n]
Boloria euphrosyne|EZROM858-08|RVcoll.07-D486||Romania|658[0n]
Boloria euphrosyne|EZROM857-08|RVcoll.07-D314||Romania|658[0n]
Boloria euphrosyne|LEFIJ548-10|MM17173||Finland|658[0n]
Boloria euphrosyne|GWORR670-10|BC ZSM Lep 32166||Germany|658[0n]
Boloria euphrosyne|LEFIL378-10|MM18688||Finland|658[0n]
Boloria euphrosyne|EULEP4773-16|RVcoll16H223||Norway|658[0n]
Boloria euphrosyne|EULEP4748-16|RVcoll16G782||Sweden|658[0n]
Boloria euphrosyne|EULEP583-15|RVcoll.10-A692||Estonia|658[0n]
Boloria euphrosyne|EULEP3519-16|RVcoll14O552||Estonia|658[0n]
Boloria euphrosyne|LEFID495-10|MM06452||Finland|658[0n]
Boloria euphrosyne|EULEP2205-15|RVcoll.14-N365||Ukraine|658[0n]
Boloria euphrosyne|EZSPN258-09|RVcoll.07-W162||Spain|658[0n]
Boloria euphrosyne|EULEP2209-15|RVcoll.14-N376||Belarus|658[0n]
Boloria euphrosyne|EZSPC225-09|RVcoll.08-L492||Spain|655[0n]
Boloria euphrosyne|EZSPN1072-11|Rvcoll.09-V303||France|658[0n]
Boloria euphrosyne|EZSPC944-10|RVcoll.09-X132||Spain|658[0n]
Boloria euphrosyne|WMB3379-14|RVcoll.09-V316||Spain|658[0n]
Boloria euphrosyne|EZSPC226-09|RVcoll.08-L493||Spain|654[0n]
Boloria euphrosyne|EZSPC647-09|RVcoll.07-C026||France|658[0n]
Boloria euphrosyne|EZSPC646-09|RVcoll.07-C025||France|658[0n]
Boloria euphrosyne|EULEP845-15|RVcoll.14-C062||Sweden|658[0n]
Boloria euphrosyne|EULEP5088-16|RVcoll16H001||Sweden|658[0n]
Boloria euphrosyne|WMB4032-14|RVcoll.12-Z161||Ireland|658[0n]
Boloria euphrosyne|EZSPC909-10|RVcoll.09-V790||Spain|658[0n]
Boloria euphrosyne|LON866-11|NHMO Lep09064||Norway|658[0n]
Boloria euphrosyne|LON819-11|NHMO Lep09017||Norway|658[0n]
Boloria euphrosyne|LON052-08|NHMO-06052||Norway|657[0n]
Boloria euphrosyne|GWORL285-09|BC ZSM Lep 21997||Germany|658[0n]
Boloria euphrosyne|GBLAA1363-15|BC ZSM Lep 87059||Germany|658[0n]
Boloria euphrosyne|OXB825-15|14-U788||Italy|658[0n]
Boloria euphrosyne|EULEP2136-15|RVcoll.14-J981||Switzerland|658[0n]
Boloria euphrosyne|EULEP3509-16|RVcoll15G743||Switzerland|658[0n]
Boloria euphrosyne|LEATF461-14|TLMF Lep 13773||Austria|658[0n]
Boloria euphrosyne|EULEP599-15|RVcoll.10-C332||Switzerland|658[0n]
Boloria euphrosyne|BIBSA1195-15|15-M281||Italy|658[0n]
Boloria euphrosyne|OXB623-15|14-O012||Italy|658[0n]
Boloria euphrosyne|OXB357-15|RVcoll.14-N071||Italy|658[0n]
Boloria euphrosyne|EULEP2561-15|RVcoll.14-V533||Ukraine|627[0n]
Boloria euphrosyne|EULEP4812-16|RVcoll16H705||Belgium|613[0n]
Boloria euphrosyne|EULEP3517-16|RVcoll15I600||Austria|658[0n]
Boloria euphrosyne|EULEP3514-16|RVcoll15H776||Switzerland|658[0n]
Boloria euphrosyne|EULEP3513-16|RVcoll15H502||Switzerland|658[0n]
Boloria euphrosyne|EULEP3512-16|RVcoll15H174||Switzerland|658[0n]
Boloria euphrosyne|EULEP3511-16|RVcoll15G881||Switzerland|658[0n]
Boloria euphrosyne|EULEP3510-16|RVcoll15G857||Italy|658[0n]
Boloria euphrosyne|LEATG044-14|TLMF Lep 13831||Austria|658[0n]
Boloria euphrosyne|PHLAH746-12|TLMF Lep 08565||Austria|658[0n]
Boloria euphrosyne|ABOLD078-16|TLMF Lep 21156||Austria|658[0n]
Boloria euphrosyne|LEATJ1243-16|TLMF Lep 19566||Austria|658[0n]
Boloria euphrosyne|LEATC576-13|TLMF Lep 12558||Austria|658[0n]
Boloria euphrosyne|PHLAC362-10|TLMF Lep 02397||Italy|658[0n]
Issoria lathonia|WMB2367-13|RVcoll.12-Q933||Italy|658[0n]
Issoria lathonia|LOWA287-06|2005-LOWA-287||Russia|658[0n]
Issoria lathonia|FBLMX196-11|BC ZSM Lep 50407||Germany|658[0n]
Issoria lathonia|EULEP4100-16|RVcoll15H897||Italy|633[0n]
Issoria lathonia|LEFIG105-10|MM13889||Finland|658[1n]
Issoria lathonia|EZROM223-08|RV-07-D165||Romania|658[0n]
Issoria lathonia|BIBSA397-15|RVcoll.14-I065||Italy|658[0n]
Issoria lathonia|EULEP1398-15|RVcoll.14-G032||Greece|658[0n]
Issoria lathonia|BIBSA1298-15|14-E310|Corsica|France|658[0n]
Issoria lathonia|WMB1517-13|RVcoll.12-Q129|Corsica|France|658[0n]

Issoria lathonia|BIBSA397-15|RVcoll.14-I065||Italy|658[0n]
Issoria lathonia|EULEP1398-15|RVcoll.14-G032||Greece|658[0n]
Issoria lathonia|BIBSA1298-15|14-E310|Corsica|France|658[0n]
Issoria lathonia|WMB1517-13|RVcoll.12-O129|Corsica|France|658[0n]
Issoria lathonia|LEATG516-14|TLMF Lep 14303||Italy|658[0n]
Issoria lathonia|LEATG515-14|TLMF Lep 14302||Italy|658[0n]
Issoria lathonia|GWORU347-10|BC ZSM Lep 31938||Italy|658[0n]
Issoria lathonia|LEFIG353-10|MM14350||Finland|658[0n]
Issoria lathonia|FBLMW377-10|BC ZSM Lep 37478||Germany|658[0n]
Issoria lathonia|GWORZ025-10|BC ZSM Lep 30381||Italy|658[0n]
Issoria lathonia|GWORA2501-09|BC ZSM Lep 30713||Germany|658[0n]
Issoria lathonia|LEFIJ541-10|MM17166||Finland|658[0n]
Issoria lathonia|EZROM221-08|RV-06-M895||Romania|658[0n]
Issoria lathonia|EZROM224-08|RV-07-D237||Romania|658[0n]
Issoria lathonia|EZROM1031-08|RVcoll.08-M211||Romania|658[0n]
Issoria lathonia|EZROM334-08|RV-08-A012||Romania|658[0n]
Issoria lathonia|EZROM632-08|RV-07-D953||Romania|658[0n]
Issoria lathonia|EZROM1032-08|RVcoll.08-M239||Romania|658[0n]
Issoria lathonia|EZROM332-08|RV-08-A010||Romania|657[0n]
Issoria lathonia|EZROM1033-08|RVcoll.08-M366||Romania|658[0n]
Issoria lathonia|EZROM222-08|RV-06-M914||Romania|658[0n]
Issoria lathonia|EZROM726-08|RV-06-G427||Spain|658[0n]
Issoria lathonia|EZSPN100-09|RVcoll.06-A039||Spain|658[0n]
Issoria lathonia|EZSPN734-09|RVcoll.08-L142||Spain|658[0n]
Issoria lathonia|EZSPC453-09|RVcoll.08-H210||Spain|658[0n]
Issoria lathonia|EZSPN367-09|RVcoll.08-H392||Spain|658[0n]
Issoria lathonia|EZSPN397-09|RVcoll.08-H462||Spain|658[0n]
Issoria lathonia|EZSPM958-12|Rvcoll.12-M709||Spain|658[0n]
Issoria lathonia|EZSPN841-09|RVcoll.08-L670||Spain|658[0n]
Issoria lathonia|EZSPN333-09|RVcoll.08-H258||Spain|658[0n]
Issoria lathonia|EZSPM131-09|RVcoll.08-P640||Spain|658[0n]
Issoria lathonia|WMB256-11|RVcoll.09-V290||France|658[0n]
Issoria lathonia|WMB3391-14|RVcoll.09-V561||Spain|658[0n]
Issoria lathonia|WMB2980-14|RVcoll.12-O172|Corsica|France|658[0n]
Issoria lathonia|WMB3176-14|RVcoll.08-H573||Spain|658[0n]
Issoria lathonia|WMB3235-14|RVcoll.08-J891||Spain|658[0n]
Issoria lathonia|WMB3320-14|RVcoll.08-P702||Spain|658[0n]
Issoria lathonia|WMB3500-14|RVcoll.10-B729||France|658[0n]
Issoria lathonia|WMB2836-13|RVcoll.12-O590|Sardinia|Italy|658[0n]
Issoria lathonia|WMB3637-14|RVcoll.11-I450||Spain|658[0n]
Issoria lathonia|WMB3673-14|RVcoll.11-I976||France|658[0n]
Issoria lathonia|WMB1180-13|RVcoll.11-E868|Corsica|France|658[0n]
Issoria lathonia|WMB1589-13|RVcoll.12-O587|Sardinia|Italy|658[0n]
Issoria lathonia|WMB2075-13|RVcoll.12-Q975|Sicily|Italy|658[0n]
Issoria lathonia|WMB2077-13|RVcoll.12-Q984|Sicily|Italy|658[0n]
Issoria lathonia|WMB1621-13|RVcoll.12-O769||France|658[0n]
Issoria lathonia|WMB1845-13|RVcoll.11-I907||France|658[0n]
Issoria lathonia|WMB2238-13|RVcoll.12-R369|Elba|Italy|658[0n]
Issoria lathonia|WMB2294-13|RVcoll.10-C668||Italy|658[0n]
Issoria lathonia|WMB2581-13|RVcoll.12-R098|Sicily|Italy|658[0n]
Issoria lathonia|WMB2624-13|RVcoll.12-O097|Corsica|France|658[0n]
Issoria lathonia|WMB4128-14|RVcoll.13-S613||Italy|658[0n]
Issoria lathonia|WMB4169-14|RVcoll.13-S740||Italy|658[0n]
Issoria lathonia|WMB4510-14|RVcoll.14-E198||Spain|658[0n]
Issoria lathonia|WMB4008-14|RVcoll.12-Q743||Italy|658[0n]
Issoria lathonia|WMB4265-14|RVcoll.14-A131||Italy|658[0n]
Issoria lathonia|WMB4276-14|RVcoll.14-A224||Italy|658[0n]
Issoria lathonia|WMB4309-14|RVcoll.14-A425||Italy|658[0n]
Issoria lathonia|WMB4367-14|RVcoll.14-B098||Portugal|658[0n]
Issoria lathonia|WMB4399-14|RVcoll.14-B401||Portugal|658[0n]
Issoria lathonia|WMB4647-14|RVcoll.LD-2443|Corsica|France|658[0n]
Issoria lathonia|WMB4725-14|RVcoll.LD-3086|Capri|Italy|658[0n]
Issoria lathonia|WMB311-11|RVcoll.11-E442||Italy|658[0n]
Issoria lathonia|WMB350-11|RVcoll.11-E774||France|658[0n]
Issoria lathonia|WMB708-12|RVcoll.11-H336|Sicily|Italy|658[0n]
Issoria lathonia|WMB721-12|RVcoll.11-I191||Italy|658[0n]
Issoria lathonia|WMB4813-14|RVcoll. 14-I360||Italy|658[0n]
Issoria lathonia|WMB4904-14|RVcoll.14-I451||Italy|658[0n]
Issoria lathonia|WMB4966-14|RVcoll. 14-I513||Italy|658[0n]
Issoria lathonia|WMB5039-14|RVcoll. 14-L170||Italy|658[0n]
Issoria lathonia|WMB5306-14|RVcoll.14-I642||France|658[0n]
Issoria lathonia|WMB5502-14|RVcoll.14-J422|Giglio|Italy|658[0n]
Issoria lathonia|EULEP4101-16|RVcoll15I372||Austria|658[0n]
Issoria lathonia|EULEP4102-16|RVcoll15J307||France|658[0n]
Issoria lathonia|EULEP4951-16|RVcoll16I177||Germany|658[0n]
Issoria lathonia|EULEP4971-16|RVcoll16I420||Poland|658[0n]
Issoria lathonia|EULEP1179-15|RVcoll.14-F419||Bulgaria|658[0n]
Issoria lathonia|EULEP961-15|RVcoll.14-D021||Bulgaria|658[0n]
Issoria lathonia|EULEP1670-15|RVcoll.14-G908||Greece|658[0n]
Issoria lathonia|EULEP1908-15|RVcoll.14-B805||Bosnia and Herzegovina|658[0n]
Issoria lathonia|EULEP2013-15|RVcoll.14-I805||Slovakia|658[0n]
Issoria lathonia|EULEP2247-15|RVcoll.14-N429||Ukraine|658[0n]
Issoria lathonia|BIBSA1125-15|15-L908||Italy|658[0n]
Issoria lathonia|BIBSA1138-15|15-L951||Italy|658[0n]
Issoria lathonia|BIBSA1542-16|LEP-SS-00377||Italy|658[0n]
Issoria lathonia|BIBSA1606-16|14-A713||Italy|658[0n]
Issoria lathonia|BIBSA1019-15|15-C161||Italy|658[0n]
Issoria lathonia|BIBSA1331-15|15-M823||Italy|658[0n]
Issoria lathonia|BIBSA1405-15|15-N011||Italy|658[0n]
Issoria lathonia|BIBSA398-15|RVcoll.14-I066||Italy|658[0n]
Issoria lathonia|BIBSA1856-17|RVcoll16C738||Italy|658[0n]
Issoria lathonia|BIBSA481-15|LEP-SS-00101||Italy|658[0n]
Issoria lathonia|BIBSA482-15|LEP-SS-00102||Italy|658[0n]
Issoria lathonia|BIBSA1733-16|LD-2776||Italy|658[0n]
Issoria lathonia|BIBSA302-15|RVcoll.14-E104||Italy|658[0n]
Issoria lathonia|GBLAA1076-15|BC ZSM Lep 86582||Germany|658[0n]
Issoria lathonia|WMB2048-13|RVcoll.12-Q782||Italy|658[0n]
Issoria lathonia|LEASS695-17|TLMF Lep 22343||Austria|658[0n]
Issoria lathonia|OXB1115-15|15-A648||Italy|658[0n]
Issoria lathonia|OXB362-15|RVcoll.14-N076||Italy|658[0n]
Issoria lathonia|OXB291-15|RVcoll.14-N005||Italy|658[0n]
Issoria lathonia|EULEP5069-16|RVcoll16J172||Czech Republic|658[0n]
Issoria lathonia|WMB1160-13|RVcoll.11-E557|Sardinia|Italy|651[0n]

Issoria lathonia|OXB291-15|RVcoll.14-N005||Italy|658[0n]
Issoria lathonia|EULEP5069-16|RVcoll16J172||Czech Republic|658[0n]
Issoria lathonia|WMB1160-13|RVcoll.11-E557|Sardinia|Italy|651[0n]
Issoria lathonia|WMB2212-13|RVcoll.11-Y025||Italy|627[0n]
Issoria lathonia|EZSPM085-09|RVcoll.08-P408||Spain|634[0n]
Issoria lathonia|EZSPC454-09|RVcoll.08-M903||Spain|632[0n]
Issoria lathonia|EULEP4980-16|RVcoll16I525||Poland|633[0n]
Issoria lathonia|WMB4160-14|RVcoll.13-S717||Italy|617[0n]
Issoria lathonia|EZSPC455-09|RVcoll.08-P389||Spain|630[0n]
Issoria lathonia|PHLAI486-13|TLMF Lep 09048||Austria|612[0n]
Issoria lathonia|ABOLD407-16|TLMF Lep 21559||Austria|658[0n]
Issoria eugenia|EULEP2897-15|RVcoll.14-U850||Russia|620[0n]
Issoria eugenia|EULEP2895-15|RVcoll.14-U848||Russia|658[0n]
Issoria eugenia|EULEP442-14|KN00804||Russia|658[0n]
Issoria eugenia|EULEP441-14|KN00803||Russia|658[0n]
Boloria eunomia|EULEP5180-17|RVcoll16H249||Norway|658[0n]
Boloria eunomia|EULEP5168-17|RVcoll16G707||Sweden|658[0n]
Boloria eunomia|EULEP4766-16|RVcoll16H128||Norway|658[0n]
Boloria eunomia|EZSPC763-10|RVcoll.07-C632.1||Spain|658[0n]
Boloria eunomia|EZSPC757-10|RVcoll.09-V672||Spain|658[0n]
Boloria eunomia|EZSPN198-09|RVcoll.07-C679||Spain|658[0n]
Boloria eunomia|EZSPN894-09|RVcoll.08-L805||Spain|658[0n]
Boloria eunomia|EZSPN891-09|RVcoll.08-L802||Spain|658[0n]
Boloria eunomia|EZSPN893-09|RVcoll.08-L804||Spain|658[0n]
Boloria eunomia|EZSPN892-09|RVcoll.08-L803||Spain|658[0n]
Boloria eunomia|EZSPN810-09|RVcoll.08-L505||Andorra|658[0n]
Boloria eunomia|GBLAB324-13|BC ZSM Lep 72625||Germany|647[0n]
Boloria eunomia|EULEP4822-16|RVcoll16H718||Belgium|658[0n]
Boloria eunomia|LEFIJ544-10|MM17169||Finland|658[0n]
Boloria eunomia|EULEP4743-16|RVcoll16G757||Sweden|658[0n]
Boloria eunomia|LON467-08|NHMO-08118||Norway|657[0n]
Boloria eunomia|LEFIG144-10|MM13983||Finland|658[0n]
Boloria eunomia|LEFIG143-10|MM13982||Finland|658[0n]
Boloria eunomia|EULEP1094-15|RVcoll.14-F093||Serbia|658[0n]
Boloria eunomia|EULEP1091-15|RVcoll.14-F059||Serbia|658[0n]
Boloria eunomia|EULEP1090-15|RVcoll.14-F053||Serbia|658[0n]
Boloria eunomia|EULEP2275-15|RVcoll.14-N461||Ukraine|658[0n]
Boloria eunomia|EULEP3503-16|RVcoll10A811||Estonia|658[0n]
Boloria eunomia|EULEP3502-16|RVcoll10A810||Estonia|658[0n]
Boloria eunomia|LOWA758-06|2005-LOWA-758||Russia|658[0n]
Boloria eunomia|LOWA293-06|2005-LOWA-293||Russia|658[0n]
Boloria eunomia|LOWA292-06|2005-LOWA-292||Russia|658[0n]
Boloria eunomia|PHLAI555-13|TLMF Lep 09117||Austria|658[0n]
Boloria eunomia|LEASS1049-17|TLMF Lep 22602||Austria|658[0n]
Boloria eunomia|ODOPE250-11|BC ZSM Lep 53121||Germany|658[0n]
Boloria eunomia|PHLAI571-13|TLMF Lep 09133||Italy|658[0n]
Boloria eunomia|PHLAI570-13|TLMF Lep 09132||Italy|658[0n]
Boloria eunomia|FBLMT910-09|BC ZSM Lep 25470||Germany|658[0n]
Boloria eunomia|GWORR675-10|BC ZSM Lep 32171||Germany|658[0n]
Boloria eunomia|ABOLD596-17|TLMF Lep 21672||Austria|658[0n]
Boloria eunomia|GBLAB780-13|BC ZSM Lep 75551||Germany|658[0n]
Boloria eunomia|GBLAC368-13|BC ZSM Lep 75234||Germany|658[0n]
Boloria eunomia|GWORK308-09|BC ZSM Lep 21543||Germany|658[0n]
Boloria eunomia|ODOPE249-11|BC ZSM Lep 53120||Germany|658[0n]
Boloria eunomia|GWOTF680-12|BC ZSM Lep 62386||Germany|658[0n]
Boloria eunomia|PHLAW004-13|TLMF Lep 09801||Austria|658[0n]
Boloria eunomia|PHLAI572-13|TLMF Lep 09134||Austria|658[0n]
Boloria graeca|BIBSA881-15|LD-3493||Italy|658[0n]
Boloria graeca|WMB1868-13|RVcoll.11-J435||France|658[0n]
Boloria graeca|WMB1628-13|RVcoll.12-O810||France|639[0n]
Boloria graeca|WMB1627-13|RVcoll.12-O809||France|658[0n]
Boloria graeca|EULEP908-15|RVcoll.14-C765||Bulgaria|658[0n]
Boloria graeca|EULEP863-15|RVcoll.14-C223||Greece|658[0n]
Boloria graeca|EULEP862-15|RVcoll.14-C221||Greece|658[0n]
Boloria graeca|EULEP505-15|RVcoll.07-C530||Bulgaria|658[0n]
Boloria graeca|EULEP3520-16|RVcoll10B414||Bulgaria|658[0n]
Boloria graeca|PHLAF404-11|TLMF Lep 05574||Macedonia|658[0n]
Boloria|EULEP3529-16|RVcoll15G749|pales/napaea|Switzerland|658[0n]
Boloria|EULEP3532-16|RVcoll15G756|pales/napaea|Switzerland|658[0n]
Boloria|EULEP3540-16|RVcoll15J474|pales/napaea|France|658[0n]
Boloria|BIBSA291-15|RVcoll.14-E091|pales/napaea|Italy|658[0n]
Boloria|EULEP3548-16|RVcoll15G215|pales/napaea|Switzerland|622[0n]
Boloria|EULEP3534-16|RVcoll15H474|pales/napaea|Switzerland|630[0n]
Boloria|EULEP3521-16|RVcoll10C011|pales/napaea|France|658[0n]
Boloria napaea|EULEP5185-17|RVcoll16H509||Sweden|658[0n]
Boloria napaea|EULEP4786-16|RVcoll16H437||Sweden|658[0n]
Boloria napaea|LEFIC094-10|MM03407||Finland|658[0n]
Boloria napaea|LEFIA746-10|MM04100||Finland|658[0n]
Boloria napaea|LEFIJ543-10|MM17168||Finland|658[0n]
Boloria napaea|OXB374-15|RVcoll.14-N088||Italy|658[0n]
Boloria napaea|LEATC570-13|TLMF Lep 12552||Austria|658[0n]
Boloria napaea|PHLSA407-11|TLMF Lep 05862||Austria|658[0n]
Boloria napaea|EULEP182-14|RVcoll.11-J433||Switzerland|658[0n]
Boloria napaea|EULEP3530-16|RVcoll15H267||Switzerland|636[1n]
Boloria napaea|BIBSA367-15|RVcoll.14-I035||Italy|658[0n]
Boloria napaea|BIBSA290-15|RVcoll.14-E090||Italy|658[0n]
Boloria napaea|BIBSA1076-15|15-K526||Italy|658[0n]
Boloria napaea|EULEP2084-15|RVcoll.14-J572||Switzerland|658[0n]
Boloria napaea|EULEP3523-16|RVcoll15I318||Austria|658[0n]
Boloria napaea|PHLAH356-12|TLMF Lep 07795||Austria|658[0n]
Boloria napaea|PHLAH355-12|TLMF Lep 07794||Austria|658[0n]
Boloria napaea|EULEP2455-15|RVcoll.14-V277||Switzerland|658[0n]
Boloria napaea|EULEP3522-16|RVcoll15H411||Switzerland|658[0n]
Boloria napaea|EULEP3524-16|RVcoll15I913||Switzerland|658[0n]
Boloria napaea|EULEP3525-16|RVcoll15J075||Switzerland|658[0n]
Boloria napaea|EULEP2468-15|RVcoll.14-V294||Switzerland|658[0n]
Boloria napaea|EULEP2458-15|RVcoll.14-V281||Switzerland|614[0n]
Boloria napaea|EULEP3531-16|RVcoll15G227||Switzerland|623[0n]
Boloria napaea|EULEP3528-16|RVcoll15G216||Switzerland|612[0n]
Boloria napaea|LEATJ1331-16|TLMF Lep 19654||Italy|632[1n]
Boloria napaea|LEATH794-14|TLMF Lep 16006||Italy|658[0n]
Boloria napaea|EULEP3527-16|RVcoll15J541||France|658[0n]
Boloria napaea|WMB2681-13|RVcoll.10-B923||France|658[0n]

[illegible]
Boloria napaea|LEATH794-14|TLMF Lep 16006||Italy|658[0n]
Boloria napaea|EULEP3527-16|RVcoll15J541||France|658[0n]
Boloria napaea|WMB2681-13|RVcoll.10-B923||France|658[0n]
Boloria napaea|WMB2682-13|RVcoll.10-B929||France|658[0n]
Boloria napaea|EULEP3526-16|RVcoll15J365||France|658[0n]
Boloria napaea|EULEP2083-15|RVcoll.14-J571||Switzerland|658[0n]
Boloria napaea|WMB2680-13|RVcoll.10-B920||France|632[0n]
Boloria napaea|LEATG041-14|TLMF Lep 13828||Austria|658[0n]
Boloria alaskensis|EULEP2666-15|RVcoll.14-N750||Russia|643[0n]
Boloria alaskensis|EULEP440-14|KN00802||Russia|634[0n]
Boloria alaskensis|EULEP439-14|KN00801||Russia|643[0n]
Boloria aquilonaris|EULEP4746-16|RVcoll16G744||Sweden|658[0n]
Boloria aquilonaris|LEFIG493-10|MM14566||Finland|658[0n]
Boloria aquilonaris|EZROM798-08|RVcoll.08-M527||Romania|658[0n]
Boloria aquilonaris|EZROM797-08|RVcoll.08-M526||Romania|658[0n]
Boloria aquilonaris|EZROM795-08|RVcoll.08-M524|Genit. examined|Romania|658[0n]
Boloria aquilonaris|EZROM796-08|RVcoll.08-M525|Genit. examined|Romania|658[0n]
Boloria aquilonaris|EZROM794-08|RVcoll.08-M523||Romania|658[0n]
Boloria aquilonaris|EULEP5177-17|RVcoll16H126||Norway|658[0n]
Boloria aquilonaris|EULEP4775-16|RVcoll16H292||Norway|658[0n]
Boloria aquilonaris|LOWA295-06|2005-LOWA-295||Russia|657[0n]
Boloria aquilonaris|LOWA294-06|2005-LOWA-294||Russia|657[0n]
Boloria aquilonaris|LEFIC067-10|MM03352||Finland|658[0n]
Boloria aquilonaris|LEFIC066-10|MM03351||Finland|658[0n]
Boloria aquilonaris|EULEP3499-16|RVcoll15G075||France|658[0n]
Boloria aquilonaris|GWORR674-10|BC ZSM Lep 32170||Germany|658[0n]
Boloria aquilonaris|FBLMU469-09|BC ZSM Lep 27119||Germany|658[0n]
Boloria aquilonaris|GWOSA737-10|BC ZSM Lep 35748||Germany|658[0n]
Boloria aquilonaris|EULEP3498-16|RVcoll15G074||France|658[0n]
Boloria aquilonaris|GBLAB771-13|BC ZSM Lep 75542||Germany|658[0n]
Boloria aquilonaris|LEASS883-17|KLM Lep 08388||Austria|658[0n]
Boloria aquilonaris|LEASS486-17|TLMF Lep 22134||Austria|658[0n]
Boloria aquilonaris|LEASS677-17|TLMF Lep 22325||Austria|658[0n]
Boloria aquilonaris|GWOTF677-12|BC ZSM Lep 62383||Germany|629[0n]
Boloria aquilonaris|GWORL401-09|BC ZSM Lep 22303||Germany|614[0n]
Boloria aquilonaris|PHLAH291-12|TLMF Lep 07730||Austria|658[0n]
Boloria aquilonaris|PHLAH292-12|TLMF Lep 07731||Austria|658[0n]
Boloria aquilonaris|PHLAA706-09|TLMF Lep 00746||Austria|658[0n]
Boloria aquilonaris|ABOLA858-15|TLMF Lep 16818||Austria|658[0n]
Boloria aquilonaris|ABOLD021-16|TLMF Lep 21099||Austria|658[0n]
Boloria pales|EZSPC271-09|RVcoll.08-R203|Genit. examined|Spain|656[0n]
Boloria pales|EZSPC270-09|RVcoll.08-R202|Genit. examined|Spain|658[0n]
Boloria pales|EZSPC938-10|RVcoll.09-X039|Genit. examined|Spain|658[0n]
Boloria pales|EZSPM411-09|Rvcoll.09-V831|Genit. examined|Spain|658[0n]
Boloria pales|EZSPM413-09|Rvcoll.09-V833|Genit. examined|Spain|655[0n]
Boloria pales|EZSPC848-10|RVcoll.08-R237.1|Genit. examined|Spain|657[0n]
Boloria pales|EZSPM414-09|Rvcoll.09-V834|Genit. examined|Spain|658[0n]
Boloria pales|EZSPM794-12|Rvcoll. 070611MH78||Spain|658[0n]
Boloria pales|EZSPM412-09|Rvcoll.09-V832|Genit. examined|Spain|658[0n]
Boloria pales|EZSPM789-12|Rvcoll. 070611MH21||Spain|658[0n]
Boloria pales|EZSPC875-10|RVcoll.09-T126|Genit. examined|Spain|658[0n]
Boloria pales|EZSPC876-10|RVcoll.09-T127|Genit. examined|Spain|658[0n]
Boloria pales|EZSPM831-12|Rvcoll. 140711ZG08||Spain|658[0n]
Boloria pales|EZSPM833-12|Rvcoll. 140711ZG20||Spain|658[0n]
Boloria pales|EZSPM840-12|Rvcoll. 140711ZG55||Spain|658[0n]
Boloria pales|EZSPC269-09|RVcoll.08-R201||Spain|657[0n]
Boloria pales|EULEP2113-15|RVcoll.14-J669||Spain|658[0n]
Boloria pales|EZSPM416-09|Rvcoll.09-V836|Genit. examined|Spain|654[0n]
Boloria pales|EZSPM415-09|Rvcoll.09-V835|Genit. examined|Spain|651[0n]
Boloria pales|EZSPC648-09|RVcoll.07-C027|Genit. examined|France|658[0n]
Boloria pales|PHLAH351-12|TLMF Lep 07790||Switzerland|658[0n]
Boloria pales|WMB6594-18|RVcoll14V685||Italy|658[1n]
Boloria pales|WMB6593-18|RVcoll14V684||Italy|639[1n]
Boloria pales|WMB6592-18|RVcoll14V683||Italy|632[1n]
Boloria pales|WMB6591-18|RVcoll14V681||Italy|658[0n]
Boloria pales|PHLSA441-11|TLMF Lep 05896||Italy|658[0n]
Boloria pales|BIBSA371-15|RVcoll.14-I039||Italy|658[0n]
Boloria pales|PHLAB1038-10|TLMF Lep 01838||Switzerland|658[0n]
Boloria pales|EULEP3536-16|RVcoll15I349||Austria|658[0n]
Boloria pales|GBLAD932-14|BC ZSM Lep 84538||Germany|658[0n]
Boloria pales|LEATG042-14|TLMF Lep 13829||Austria|657[0n]
Boloria pales|BIBSA1192-15|15-M267||Italy|658[0n]
Boloria pales|LEATC131-13|TLMF Lep 11258||Italy|658[0n]
Boloria pales|EULEP3538-16|RVcoll15J092||Switzerland|658[0n]
Boloria pales|EULEP3535-16|RVcoll15H865||Italy|658[0n]
Boloria pales|PHLAB1039-10|TLMF Lep 01839||Switzerland|658[0n]
Boloria pales|WMB5104-14|RVcoll. 14-L235||Italy|658[0n]
Boloria pales|EULEP3546-16|RVcoll14A972||Bulgaria|658[0n]
Boloria pales|EULEP2676-15|RVcoll.14-N760||Macedonia|658[0n]
Boloria pales|EULEP3545-16|RVcoll15Q009||Macedonia|637[0n]
Boloria pales|EULEP3543-16|RVcoll15P076||Macedonia|658[0n]
Boloria pales|EULEP3542-16|RVcoll15P075||Macedonia|641[0n]
Boloria pales|EULEP910-15|RVcoll.14-C768||Bulgaria|613[0n]
Boloria pales|EULEP990-15|RVcoll.14-D236||Austria|614[0n]
Boloria pales|BIBSA368-15|RVcoll.14-I036||Italy|629[0n]
Boloria pales|EULEP3541-16|RVcoll15G292||Switzerland|623[0n]
Boloria pales|GWOTF676-12|BC ZSM Lep 62382||Germany|633[0n]
Boloria pales|PHLAB1037-10|TLMF Lep 01837||Switzerland|658[0n]
Boloria pales|EZROM053-08|RV-07-E454||Romania|656[1n]
Boloria pales|EULEP3537-16|RVcoll15I601||Austria|658[0n]
Boloria pales|ABOLD030-16|TLMF Lep 21108||Austria|658[0n]
Boloria pales|LEATC099-13|TLMF Lep 11226||Italy|658[0n]
Boloria pales|LEATJ1260-16|TLMF Lep 19583||Austria|658[0n]
Boloria pales|EULEP3547-16|RVcoll15H813||Italy|658[0n]
Boloria pales|EULEP600-15|RVcoll.10-C333||Switzerland|658[0n]
Boloria pales|LEASS484-17|TLMF Lep 22132||Austria|658[0n]
Boloria pales|BIBSA370-15|RVcoll.14-I038||Italy|658[0n]
Boloria pales|EULEP024-14|RVcoll.07-D690||Romania|658[0n]
Boloria pales|EULEP3539-16|RVcoll15J346||France|658[0n]
Boloria pales|EZROM663-08|RV-06-M965||Romania|658[0n]
Boloria pales|EZROM054-08|RV-07-E575|Genit. examined|Romania|658[0n]
Boloria pales|EZROM802-08|RVcoll.08-M611||Romania|658[0n]
[illegible]

Boloria pales|EZROM663-08|RV-06-M965||Romania|658[0n]
Boloria pales|EZROM054-08|RV-07-E575|Genit. examined|Romania|658[0n]
Boloria pales|EZROM802-08|RVcoll.08-M611||Romania|658[0n]
Boloria pales|EZROM801-08|RVcoll.07-D650|Genit. examined|Romania|658[0n]
Boloria pales|EZROM800-08|RVcoll.06-M996||Romania|658[0n]
Boloria pales|EZROM799-08|RVcoll.06-M989|Genit. examined|Romania|658[0n]
Boloria pales|GWOSA777-10|BC ZSM Lep 36643||Germany|658[0n]
Boloria pales|FBLMX200-11|BC ZSM Lep 50411||Germany|658[0n]
Boloria pales|GWOTD440-12|BC ZSM Lep 63571||Germany|658[0n]
Boloria pales|LEATC098-13|TLMF Lep 11225||Italy|658[0n]
Boloria pales|LEATJ1332-16|TLMF Lep 19655||Italy|658[0n]
Boloria pales|PHLSA651-11|TLMF Lep 06106||Austria|658[0n]
Boloria pales|EULEP639-15|RVcoll.11-J107||Switzerland|658[0n]
Boloria pales|EULEP3533-16|RVcoll15G875||Switzerland|658[0n]
Boloria pales|EZSPC945-10|RVcoll.09-X290|Genit. examined|Italy|658[0n]
Boloria pales|PHLAH352-12|TLMF Lep 07791||Switzerland|658[0n]
Boloria pales|WMB6598-18|RVcoll14V689||Italy|658[1n]
Boloria pales|WMB6597-18|RVcoll14V688||Italy|657[0n]
Boloria pales|WMB6596-18|RVcoll14V687||Italy|658[1n]
Boloria pales|WMB6595-18|RVcoll14V686||Italy|658[1n]
Boloria pales|BIBSA882-15|LD-3494||France|658[0n]
Boloria pales|WMB2691-13|RVcoll.10-C016||France|658[0n]
Boloria pales|PHLAH353-12|TLMF Lep 07792||France|658[0n]
Boloria pales|PHLAH354-12|TLMF Lep 07793||France|658[0n]
Brenthis daphne|EZROM808-08|RVcoll.08-M452||Romania|658[0n]
Brenthis daphne|EZSPN475-09|RVcoll.08-H909|Genit. examined|Spain|634[1n]
Brenthis daphne|EZSPC228-09|RVcoll.08-L518|Genit. examined|Spain|651[0n]
Brenthis daphne|EZROM809-08|RVcoll.08-M503||Romania|658[0n]
Brenthis daphne|EZROM810-08|RVcoll.08-M548||Romania|658[0n]
Brenthis daphne|EZROM607-08|RV-06-M910|Genit. examined|Romania|658[0n]
Brenthis daphne|EZROM056-08|RV-06-M834|Genit. examined|Romania|658[0n]
Brenthis daphne|EZROM060-08|RV-07-C912||Romania|658[0n]
Brenthis daphne|EZROM058-08|RV-06-M960|Genit. examined|Romania|658[0n]
Brenthis daphne|WMB3313-14|RVcoll.08-P496||Spain|658[0n]
Brenthis daphne|WMB1646-13|RVcoll.12-O955||France|658[0n]
Brenthis daphne|EULEP2682-15|RVcoll.14-N766||Croatia|658[0n]
Brenthis daphne|LEASS888-17|KLM Lep 08393||Austria|658[0n]
Brenthis daphne|LEASS487-17|TLMF Lep 22135||Austria|658[0n]
Brenthis daphne|OXB1570-16|OXB-TGS-1288||France|658[0n]
Brenthis daphne|ABOLD584-17|TLMF Lep 21660||Austria|658[0n]
Brenthis daphne|EZROM057-08|RV-06-M873|Genit. examined|Romania|658[1n]
Brenthis daphne|EULEP3574-16|RVcoll10A954||Bulgaria|645[0n]
Brenthis daphne|LEATH718-14|TLMF Lep 15930||Italy|634[0n]
Brenthis daphne|LEATG520-14|TLMF Lep 14307||Italy|658[0n]
Brenthis ino|EZSPC234-09|RVcoll.08-M913|Genit. examined|Spain|656[0n]
Brenthis ino|EZSPC918-10|RVcoll.09-V883|Genit. examined|Spain|658[0n]
Brenthis ino|EZSPC206-09|RVcoll.07-C675||Spain|654[0n]
Brenthis ino|BIBSA372-15|RVcoll.14-I040||Italy|658[0n]
Brenthis ino|WMB1635-13|RVcoll.12-O846||France|648[0n]
Brenthis ino|LEATG009-14|TLMF Lep 13796||Austria|658[0n]
Brenthis daphne|BIBSA1811-17|RVcoll14I280||Italy|658[0n]
Brenthis daphne|WMB668-12|RVcoll.07-D854||Italy|658[0n]
Brenthis daphne|WMB3835-14|RVcoll.09-X917||Italy|658[0n]
Brenthis daphne|BIBSA084-14|LEP-SS-00084||Italy|658[0n]
Brenthis daphne|WMB2369-13|RVcoll.12-R439||Italy|658[0n]
Brenthis daphne|WMB2298-13|RVcoll.10-C674||Italy|658[0n]
Brenthis daphne|WMB1057-13|RVcoll.10-C673||Italy|632[0n]
Brenthis daphne|BIBSA1633-16|15-C481||Italy|658[0n]
Brenthis daphne|BIBSA604-15|LEP-SS-00224||Italy|641[0n]
Brenthis daphne|BIBSA083-14|LEP-SS-00083||Italy|658[0n]
Brenthis daphne|BIBSA1632-16|15-C480||Italy|658[0n]
Brenthis daphne|BIBSA189-15|RVcoll.14-D977||Italy|658[0n]
Brenthis daphne|BIBSA1870-17|RVcoll16C765||Italy|658[0n]
Brenthis daphne|BIBSA1403-15|15-N009||Italy|658[0n]
Brenthis daphne|BIBSA999-15|15-C036||Italy|658[0n]
Brenthis daphne|WMB4291-14|RVcoll.14-A365||Italy|658[0n]
Brenthis daphne|WMB4284-14|RVcoll.14-A333||Italy|658[0n]
Brenthis daphne|WMB898-13|RVcoll.09-X842||Italy|658[0n]
Brenthis daphne|EZSPC246-09|RVcoll.08-P069||Spain|655[0n]
Brenthis daphne|EZSPC254-09|RVcoll.08-P345||Spain|658[0n]
Brenthis daphne|EZROM708-08|RV-06-H895|Genit. examined|Spain|658[0n]
Brenthis daphne|EZSPN1025-09|RVcoll.08-M958||Spain|658[0n]
Brenthis daphne|EZSPM786-12|Rvcoll. 070611MH06||Spain|658[0n]
Brenthis daphne|EZSPC764-10|RVcoll.08-L511||Spain|658[0n]
Brenthis daphne|EZSPM792-12|Rvcoll. 070611MH67||Spain|658[0n]
Brenthis daphne|EZSPM408-09|Rvcoll.09-V825|Genit. examined|Spain|638[0n]
Brenthis daphne|EZSPC229-09|RVcoll.08-L519|Genit. examined|Spain|648[0n]
Brenthis daphne|OXB1375-15|Rvcoll_15-M739||France|658[0n]
Brenthis daphne|OXB1288-15|Rvcoll_15-M151||France|658[0n]
Brenthis daphne|WMB5388-14|RVcoll.14-J868||France|658[0n]
Brenthis daphne|WMB727-12|RVcoll.11-I640||France|658[0n]
Brenthis daphne|WMB1786-13|RVcoll.12-Q207||France|658[0n]
Brenthis daphne|WMB1075-13|RVcoll.10-C704||Italy|658[0n]
Brenthis daphne|WMB3521-14|RVcoll.10-B974||France|658[0n]
Brenthis daphne|EZSPC734-10|RVcoll.08-H920.1||Spain|658[0n]
Brenthis daphne|EZSPC733-10|RVcoll.08-H908||Spain|658[0n]
Brenthis daphne|EZSPM839-12|Rvcoll. 140711ZG48||Spain|658[0n]
Brenthis daphne|EZSPC781-10|RVcoll.08-L902||Spain|658[0n]
Brenthis daphne|EZSPN971-09|RVcoll.08-L991||Spain|658[0n]
Brenthis daphne|EZSPN907-09|RVcoll.08-L831||Spain|658[0n]
Brenthis daphne|GWOTF691-12|BC ZSM Lep 62397||Croatia|658[0n]
Brenthis daphne|WMB5144-14|RVcoll.10-C632||Italy|658[0n]
Brenthis daphne|BIBSA1117-15|15-L858||Italy|658[0n]
Brenthis daphne|EULEP886-15|RVcoll.14-C506||Greece|658[0n]
Brenthis daphne|EZROM059-08|RV-07-D978|Genit. examined|Romania|658[0n]
Brenthis daphne|LEATG519-14|TLMF Lep 14306||Italy|658[0n]
Brenthis daphne|WMB706-12|RVcoll.11-H320|Sicily|Italy|658[0n]
Brenthis daphne|WMB707-12|RVcoll.11-H321|Sicily|Italy|658[0n]
Brenthis daphne|WMB742-12|RVcoll.11-J658|Sicily|Italy|658[0n]
Brenthis daphne|EULEP3575-16|RVcoll15H080||Italy|658[0n]
Brenthis daphne|EULEP4906-16|RVcoll16J609||Russia|658[0n]
Brenthis daphne|EULEP734-15|RVcoll.12-N840|Lesvos|Greece|658[0n]
Brenthis daphne|EULEP737-15|RVcoll.12-N848|Lesvos|Greece|658[0n]

Brenthis daphne|EULEP4906-16|RVcoll16J609||Russia|658[0n]
Brenthis daphne|EULEP734-15|RVcoll.12-N840|Lesvos|Greece|658[0n]
Brenthis daphne|EULEP737-15|RVcoll.12-N848|Lesvos|Greece|658[0n]
Brenthis daphne|EULEP1186-15|RVcoll.14-F452||Bulgaria|658[0n]
Brenthis daphne|EULEP1064-15|RVcoll.14-E968||Serbia|658[0n]
Brenthis daphne|EULEP1114-15|RVcoll.14-F191||Serbia|658[0n]
Brenthis daphne|EULEP1269-15|RVcoll.14-F665||Greece|658[0n]
Brenthis daphne|BIBSA685-15|RVcoll. 10-C633||Italy|658[0n]
Brenthis daphne|EULEP2560-15|RVcoll.14-V530||Ukraine|613[0n]
Brenthis daphne|WMB741-12|RVcoll.11-J657|Sicily|Italy|626[0n]
Brenthis daphne|WMB4164-14|RVcoll.13-S722||Italy|617[0n]
Brenthis daphne|WMB2081-13|RVcoll.12-R004|Sicily|Italy|658[0n]
Brenthis daphne|ABOLD003-16|TLMF Lep 21081||Austria|658[0n]
Brenthis ino|OXB1302-15|Rvcoll_15-M170||France|658[0n]
Brenthis ino|EULEP3580-16|RVcoll15G097||France|658[0n]
Brenthis ino|EZROM064-08|RV-06-M906|Genit. examined|Romania|658[0n]
Brenthis ino|EZROM814-08|RVcoll.06-M899|Genit. examined|Romania|658[0n]
Brenthis ino|EZSPC863-10|RVcoll.09-T068||Spain|658[0n]
Brenthis ino|EZSPC261-09|RVcoll.08-P384||Spain|658[0n]
Brenthis ino|EULEP597-15|RVcoll.10-C328||Switzerland|628[0n]
Brenthis ino|EULEP1129-15|RVcoll.14-F257||Serbia|658[0n]
Brenthis ino|EULEP1097-15|RVcoll.14-F121||Serbia|658[0n]
Brenthis ino|EZROM065-08|RV-07-D345|Genit. examined|Romania|658[0n]
Brenthis ino|EZROM063-08|RV-06-M865|Genit. examined|Romania|658[0n]
Brenthis ino|GBLAF776-14|BC ZSM Lep 82862||Germany|658[0n]
Brenthis ino|WMB2704-13|RVcoll.10-C120||France|658[0n]
Brenthis ino|EZSPM788-12|Rvcoll. 070611MH13||Spain|658[0n]
Brenthis ino|EZSPC931-10|RVcoll.09-X005||Spain|658[0n]
Brenthis ino|EZSPC237-09|RVcoll.08-P011||Spain|658[0n]
Brenthis ino|EZSPC205-09|RVcoll.07-C670|Genit. examined|Spain|658[0n]
Brenthis ino|EULEP3581-16|RVcoll15G155||France|629[0n]
Brenthis ino|PHLAW007-13|TLMF Lep 09804||Austria|658[0n]
Brenthis ino|BIBSA082-14|LEP-SS-00082||Italy|658[0n]
Brenthis ino|BIBSA081-14|LEP-SS-00081||Italy|658[0n]
Brenthis ino|BIBSA080-14|LEP-SS-00080||Italy|658[0n]
Brenthis ino|BCLEP120-17|LEP-SS-00571||Italy|658[0n]
Brenthis ino|WMB3332-14|RVcoll.08-P772||Spain|658[0n]
Brenthis ino|EZSPM817-12|Rvcoll. 130711PX83||Spain|642[0n]
Brenthis ino|EZSPC967-10|RVcoll.210207TM83||Spain|658[0n]
Brenthis ino|EZSPM818-12|Rvcoll. 130711PX89||Spain|658[0n]
Brenthis ino|EZSPM086-09|RVcoll.08-P409||Spain|658[0n]
Brenthis ino|EZSPN837-09|RVcoll.08-J835||Spain|658[0n]
Brenthis ino|EZROM815-08|RVcoll.07-D309||Romania|658[0n]
Brenthis ino|EZROM816-08|RVcoll.07-D318|Genit. examined|Romania|658[0n]
Brenthis ino|EZROM817-08|RVcoll.07-D346||Romania|658[0n]
Brenthis ino|EZSPN744-09|RVcoll.08-L171||Spain|658[0n]
Brenthis ino|EZSPN753-09|RVcoll.08-L225||Spain|658[0n]
Brenthis ino|EZSPN471-09|RVcoll.08-H699||Spain|658[0n]
Brenthis ino|EZSPM807-12|Rvcoll. 090611SZ94||Spain|658[0n]
Brenthis ino|EZSPM763-12|Rvcoll. 030111FB83||Spain|658[0n]
Brenthis ino|EULEP4794-16|RVcoll16H536||Sweden|658[0n]
Brenthis ino|EULEP243-14|RVcoll.12-N703||Spain|658[0n]
Brenthis ino|EZSPN925-09|RVcoll.08-L874||Spain|621[0n]
Brenthis ino|LEFIC069-10|MM03354||Finland|632[0n]
Brenthis ino|EULEP4862-16|RVcoll16H856||Poland|658[0n]
Brenthis ino|GWOSK888-11|BC ZSM Lep 49389||Germany|658[0n]
Brenthis ino|ODOPE247-11|BC ZSM Lep 53118||Germany|658[0n]
Brenthis ino|ABOLD091-16|TLMF Lep 21169||Austria|658[0n]
Brenthis ino|EULEP1943-15|RVcoll.14-B918||Bosnia and Herzegovina|658[0n]
Brenthis ino|BIBSA1103-15|15-L768||Italy|647[0n]
Brenthis ino|EULEP2128-15|RVcoll.14-J936||France|658[0n]
Brenthis ino|EULEP794-15|RVcoll.12-Z269||Sweden|658[0n]
Brenthis ino|EULEP331-14|MM23816||Estonia|658[0n]
Brenthis ino|EULEP3578-16|RVcoll15I465||Austria|658[0n]
Brenthis ino|LEFIC068-10|MM03353||Finland|658[0n]
Brenthis ino|LEFIJ542-10|MM17167||Finland|658[0n]
Brenthis ino|LEATG514-14|TLMF Lep 14301||Italy|658[0n]
Brenthis ino|GWORA2493-09|BC ZSM Lep 30705||Germany|658[0n]
Brenthis ino|GBLAB322-13|BC ZSM Lep 72623||Germany|658[0n]
Brenthis ino|EULEP1872-15|RVcoll.13-U488||Italy|658[0n]
Brenthis ino|EULEP1858-15|RVcoll.13-U221||Italy|658[0n]
Brenthis ino|EULEP3577-16|RVcoll15H681||Switzerland|658[0n]
Brenthis ino|EULEP140-14|RVcoll.10-C414||Romania|658[0n]
Brenthis ino|EULEP2376-15|RVcoll.14-V071||Ukraine|658[0n]
Brenthis ino|EULEP2439-15|RVcoll.14-V243||Switzerland|658[0n]
Brenthis ino|EULEP2258-15|RVcoll.14-N442||Ukraine|658[0n]
Brenthis ino|GBLAC367-13|BC ZSM Lep 75233||Germany|658[0n]
Brenthis ino|EULEP3576-16|RVcoll15G031||Belgium|648[0n]
Brenthis ino|EULEP3579-16|RVcoll15I791||Austria|638[0n]
Brenthis ino|WMB5114-14|RVcoll. 14-L245||Italy|635[0n]
Brenthis ino|WMB1858-13|RVcoll.11-J000||France|658[0n]
Brenthis ino|EZROM807-08|RVcoll.08-M478||Romania|658[0n]
Brenthis ino|GWOSA736-10|BC ZSM Lep 35747||Germany|658[0n]
Brenthis ino|GWORR666-10|BC ZSM Lep 32162||Germany|658[0n]
Brenthis ino|PHLAI546-13|TLMF Lep 09108||Austria|658[0n]
Brenthis ino|LEATJ1246-16|TLMF Lep 19569||Austria|658[0n]
Brenthis ino|LEATF460-14|TLMF Lep 13772||Italy|658[0n]
Brenthis hecate|EULEP013-14|RVcoll.06-M911||Romania|658[0n]
Brenthis hecate|EULEP1120-15|RVcoll.14-F212||Serbia|658[0n]
Brenthis hecate|EZROM288-08|RV-07-E650||Romania|644[1n]
Brenthis hecate|EZSPC1069-10|RVcoll.08-L220||Spain|658[0n]
Brenthis hecate|EZSPC1070-10|RVcoll.08-L238||Spain|658[0n]
Brenthis hecate|EZSPC1086-10|RVcoll.08-L839||Spain|658[0n]
Brenthis hecate|EZSPC1087-10|RVcoll.08-L840||Spain|658[0n]
Brenthis hecate|EZROM363-08|07-E631||Spain|658[0n]
Brenthis hecate|EZROM362-08|RVcoll. 07-E630||Spain|658[0n]
Brenthis hecate|EZSPN1100-11|Rvcoll.10-A831||Spain|658[0n]
Brenthis hecate|EZSPN951-09|RVcoll.08-L943||Spain|658[0n]
Brenthis hecate|EZSPN759-09|RVcoll.08-L237||Spain|658[0n]
Brenthis hecate|EZSPN909-09|RVcoll.08-L838||Spain|658[0n]
Brenthis hecate|WMB4275-14|RVcoll.14-A221||Italy|658[0n]
Brenthis hecate|EZROM186-08|RV-07-D344||Romania|656[0n]
Brenthis hecate|EZROM609-08|RV-07-D543||Romania|658[0n]

Brenthis hecate|EZSPN909-09|RVcoll.08-L838||Spain|658[0n]
Brenthis hecate|WMB4275-14|RVcoll.14-A221||Italy|658[0n]
Brenthis hecate|EZROM186-08|RV-07-D344||Romania|656[0n]
Brenthis hecate|EZROM609-08|RV-07-D543||Romania|658[0n]
Brenthis hecate|BIBSA1118-15|15-L862||Italy|658[0n]
Brenthis hecate|EULEP1855-15|RVcoll.13-U098||Italy|658[0n]
Brenthis hecate|GWOTF692-12|BC ZSM Lep 62398||Croatia|658[0n]
Brenthis hecate|EULEP1940-15|RVcoll.14-B912||Bosnia and Herzegovina|658[0n]
Brenthis hecate|EULEP1254-15|RVcoll.14-F624||Greece|658[0n]
Brenthis hecate|EULEP1035-15|RVcoll.14-E886||Serbia|658[0n]
Brenthis hecate|EULEP2101-15|RVcoll.14-J590||Ukraine|658[0n]
Brenthis hecate|WMB248-11|RVcoll.09-V224||France|658[0n]
Brenthis hecate|EZROM061-08|RV-06-M850|Genit. examined|Romania|658[0n]
Brenthis hecate|EZROM062-08|RV-07-C970|Genit. examined|Romania|658[0n]
Brenthis hecate|EZROM813-08|RVcoll.08-M516||Romania|658[0n]
Brenthis hecate|EULEP503-15|RVcoll.07-C527||Greece|658[0n]
Brenthis hecate|ABOLD602-17|TLMF Lep 21678||Austria|658[0n]
Brenthis hecate|EZROM812-08|RVcoll.08-M461||Romania|658[0n]
Brenthis hecate|EZROM811-08|RVcoll.08-M433||Romania|658[0n]
Brenthis hecate|ABOLD485-16|TLMF Lep 21468||Austria|658[0n]
Argynnis laodice|EULEP2629-15|RVcoll.14-N569||Estonia|658[0n]
Argynnis laodice|EULEP767-15|RVcoll.12-R780||Romania|658[0n]
Argynnis laodice|EULEP2630-15|RVcoll.14-N570||Estonia|658[0n]
Argynnis laodice|EULEP2632-15|RVcoll.14-N572||Slovakia|658[0n]
Argynnis laodice|EZROM033-08|RV-07-E465||Romania|658[1n]
Argynnis laodice|EULEP689-15|RVcoll.11-J953||Romania|658[0n]
Argynnis laodice|EULEP4967-16|RVcoll16I374||Poland|658[0n]
Argynnis laodice|EZROM032-08|RV-06-M971||Romania|658[0n]
Argynnis laodice|EZROM780-08|RVcoll.08-M654||Romania|658[0n]
Argynnis laodice|EZROM779-08|RVcoll.08-M653||Romania|658[0n]
Argynnis laodice|LEFIJ312-10|MM15912||Finland|658[0n]
Argynnis laodice|LEFIJ311-10|MM15911||Finland|658[0n]
Argynnis laodice|LEFIL377-10|MM18687||Finland|658[0n]
Argynnis pandora|EZSPM658-12|RVcoll.11-I494||Spain|658[0n]
Argynnis pandora|EZSPC642-09|RVcoll.08-L375||Spain|658[0n]
Argynnis pandora|WMB2095-13|RVcoll.12-R097|Sicily|Italy|658[0n]
Argynnis pandora|EZSPM898-12|Rvcoll.12-M620||Spain|658[0n]
Argynnis pandora|EULEP1125-15|RVcoll.14-F237||Serbia|658[0n]
Argynnis pandora|WMB659-11|RVcoll.11-I341|Giglio|Italy|658[0n]
Argynnis pandora|WMB2401-13|RVcoll.09-X930|Elba|Italy|658[0n]
Argynnis pandora|WMB1102-13|RVcoll.10-C774|Giglio|Italy|658[0n]
Argynnis pandora|EZROM785-08|RVcoll.08-M404||Romania|658[0n]
Argynnis pandora|EZSPM933-12|Rvcoll.12-M667||Spain|658[0n]
Argynnis pandora|WMB1505-13|RVcoll.12-O089|Corsica|France|658[0n]
Argynnis pandora|WMB2588-13|RVcoll.12-R138|Sicily|Italy|658[0n]
Argynnis pandora|WMB4644-14|RVcoll.LD-2430|Corsica|France|658[0n]
Argynnis pandora|WMB4655-14|RVcoll.LD-2544||Italy|658[0n]
Argynnis pandora|EULEP177-14|RVcoll.11-J354||Romania|658[0n]
Argynnis pandora|EULEP952-15|RVcoll.14-C965||Bulgaria|658[0n]
Argynnis pandora|OXB1037-15|LD-2586||Italy|658[0n]
Argynnis pandora|EULEP2129-15|RVcoll.14-J969||Switzerland|624[0n]
Argynnis pandora|WMB1899-13|RVcoll.11-J971|Sardinia|Italy|635[0n]
Argynnis pandora|EZSPC966-10|RVcoll.210207TM10||Spain|636[0n]
Argynnis pandora|EZSPN645-09|RVcoll.08-J826||Spain|658[0n]
Argynnis pandora|EZROM744-08|RV-06-H898||Spain|658[0n]
Argynnis pandora|EZSPC579-09|RVcoll.07-C047||Spain|658[0n]
Argynnis pandora|EZROM037-08|RV-07-D071||Romania|658[0n]
Argynnis pandora|EZROM786-08|RVcoll.08-M436||Romania|658[0n]
Argynnis pandora|EZROM784-08|RVcoll.08-M273||Romania|658[0n]
Argynnis pandora|WMB4652-14|RVcoll.LD-2485|Sardinia|Italy|658[0n]
Argynnis pandora|WMB4651-14|RVcoll.LD-2484|Sardinia|Italy|658[0n]
Argynnis pandora|BCLEP113-17|LEP-SS-00564||Italy|658[0n]
Argynnis pandora|EULEP1209-15|RVcoll.14-F505||Greece|658[0n]
Argynnis pandora|EULEP178-14|RVcoll.11-J355||Romania|658[0n]
Argynnis pandora|LEATJ1168-16|TLMF Lep 19491||Hungary|658[0n]
Argynnis pandora|WMB2251-13|RVcoll.12-R401|Elba|Italy|658[0n]
Argynnis pandora|EULEP2181-15|RVcoll.14-N220||Greece|658[0n]
Argynnis pandora|EZSPC724-10|RVcoll.08-H482||Spain|615[0n]
Argynnis pandora|EZSPN401-09|RVcoll.08-H470||Spain|658[0n]
Argynnis pandora|EZROM036-08|RV-07-D069||Romania|658[0n]
Argynnis pandora|EZSPC722-10|RVcoll.08-H469||Spain|658[0n]
Argynnis pandora|EULEP1024-15|RVcoll.14-E819||Serbia|658[0n]
Argynnis pandora|WMB6055-18|RVcoll14A087||Italy|658[0n]
Argynnis pandora|EZROM661-08|RV-07-D073||Romania|658[0n]
Argynnis pandora|WMB2425-13|RVcoll.10-C775|Giglio|Italy|658[0n]
Argynnis pandora|WMB4386-14|RVcoll.14-B283||Portugal|658[0n]
Argynnis pandora|WMB2239-13|RVcoll.12-R370|Elba|Italy|658[0n]
Argynnis pandora|EZSPC723-10|RVcoll.08-H471||Spain|658[0n]
Argynnis pandora|BIBSA1592-16|12-Q838||Italy|658[0n]
Argynnis pandora|WMB2090-13|RVcoll.12-R060|Sicily|Italy|658[0n]
Argynnis pandora|WMB2058-13|RVcoll.12-Q835||Italy|658[0n]
Argynnis pandora|WMB2059-13|RVcoll.12-Q836||Italy|658[0n]
Argynnis pandora|GWORZ028-10|BC ZSM Lep 30384||Italy|658[0n]
Argynnis paphia|EZSPN1103-11|Rvcoll.10-A877||Spain|658[0n]
Argynnis paphia|WMB2747-13|RVcoll.10-C637|Sicily|Italy|658[0n]
Argynnis paphia|WMB2057-13|RVcoll.12-Q834||Italy|658[0n]
Argynnis paphia|GWORA2503-09|BC ZSM Lep 30715||Germany|658[0n]
Argynnis paphia|GWORZ027-10|BC ZSM Lep 30383||Italy|658[1n]
Argynnis paphia|OXB1535-16|OXB-TGS-1253||France|658[0n]
Argynnis paphia|ABOLD422-16|TLMF Lep 21574||Austria|658[0n]
Argynnis paphia|PHLAF332-11|TLMF Lep 05502||Macedonia|658[0n]
Argynnis paphia|LEATG444-14|TLMF Lep 14231||Italy|658[0n]
Argynnis paphia|LEATC620-13|TLMF Lep 12602||Austria|658[0n]
Argynnis paphia|ABOLD046-16|TLMF Lep 21124||Austria|658[0n]
Argynnis paphia|ABOLB036-15|TLMF Lep 17041||Austria|658[0n]
Argynnis paphia|GWORU371-10|BC ZSM Lep 31962||Italy|658[0n]
Argynnis paphia|LON252-08|NHMO-07041||Norway|657[0n]
Argynnis paphia|GWORA2502-09|BC ZSM Lep 30714||Germany|658[0n]
Argynnis paphia|LEFIC085-10|MM03384||Finland|658[0n]
Argynnis paphia|EZROM787-08|RVcoll.08-M545||Romania|658[0n]
Argynnis paphia|EZROM789-08|RVcoll.08-M589||Romania|658[0n]
Argynnis paphia|EZROM788-08|RVcoll.08-M546||Romania|658[0n]
Argynnis paphia|EZROM038-08|RV-07-D972||Romania|658[0n]

Argynnis paphia|EZROM789-08|RVcoll.08-M589||Romania|658[0n]
Argynnis paphia|EZROM788-08|RVcoll.08-M546||Romania|658[0n]
Argynnis paphia|EZROM038-08|RV-07-D972||Romania|658[0n]
Argynnis paphia|EZSPN901-09|RVcoll.08-L817||Spain|658[0n]
Argynnis paphia|EZSPC241-09|RVcoll.08-P047||Spain|658[0n]
Argynnis paphia|EZSPC260-09|RVcoll.08-P372||Spain|658[0n]
Argynnis paphia|EZSPN210-09|RVcoll.07-F063||Spain|658[0n]
Argynnis paphia|EZSPN476-09|RVcoll.08-H910||Spain|658[0n]
Argynnis paphia|EZSPN955-09|RVcoll.08-L949||Spain|658[0n]
Argynnis paphia|EZROM360-08|07-E600||Spain|658[0n]
Argynnis paphia|EZROM361-08|07-E601||Spain|657[0n]
Argynnis paphia|EZSPC1357-10|RVcoll.09-V931||Spain|658[0n]
Argynnis paphia|EZSPC1016-10|RVcoll.08-H676||Spain|658[0n]
Argynnis paphia|WMB235-11|RVcoll.09-T566||Italy|658[0n]
Argynnis paphia|EZSPC1123-10|RVcoll.08-P605||Spain|658[0n]
Argynnis paphia|WMB3440-14|RVcoll.09-X555||Spain|658[0n]
Argynnis paphia|WMB3146-14|RVcoll.07-E258||France|658[0n]
Argynnis paphia|WMB2797-13|RVcoll.11-J758||Italy|658[0n]
Argynnis paphia|WMB2832-13|RVcoll.12-O532|Sardinia|Italy|658[0n]
Argynnis paphia|WMB2662-13|RVcoll.10-B742||France|658[0n]
Argynnis paphia|WMB2074-13|RVcoll.12-Q971|Sicily|Italy|658[0n]
Argynnis paphia|WMB1607-13|RVcoll.12-O681||France|658[0n]
Argynnis paphia|WMB1735-13|RVcoll.12-P943||France|658[0n]
Argynnis paphia|WMB1900-13|RVcoll.11-J972|Sardinia|Italy|658[0n]
Argynnis paphia|WMB3833-14|RVcoll.09-X915||Italy|658[0n]
Argynnis paphia|WMB3851-14|RVcoll.10-C740||Italy|658[0n]
Argynnis paphia|WMB2577-13|RVcoll.12-R031|Sicily|Italy|658[0n]
Argynnis paphia|WMB3868-14|RVcoll.11-J594||Italy|658[0n]
Argynnis paphia|WMB4593-14|RVcoll.LD-469|Corsica|France|658[0n]
Argynnis paphia|WMB3941-14|RVcoll.12-P642||France|658[0n]
Argynnis paphia|WMB4015-14|RVcoll.12-R207||Italy|658[0n]
Argynnis paphia|WMB4279-14|RVcoll.14-A294||Italy|658[0n]
Argynnis paphia|WMB4323-14|RVcoll.14-A653||Italy|658[0n]
Argynnis paphia|WMB4397-14|RVcoll.14-B357||Portugal|658[0n]
Argynnis paphia|WMB4474-14|RVcoll.14-D910||Italy|658[0n]
Argynnis paphia|WMB4639-14|RVcoll.LD-2411|Corsica|France|658[0n]
Argynnis paphia|WMB4640-14|RVcoll.LD-2412|Corsica|France|658[0n]
Argynnis paphia|WMB4645-14|RVcoll.LD-2431|Corsica|France|658[0n]
Argynnis paphia|WMB4646-14|RVcoll.LD-2432|Corsica|France|658[0n]
Argynnis paphia|WMB4876-14|RVcoll.14-I423||Italy|658[0n]
Argynnis paphia|WMB5368-14|RVcoll.14-J748||France|658[0n]
Argynnis paphia|WMB4963-14|RVcoll. 14-I510||Italy|658[0n]
Argynnis paphia|WMB5270-14|RVcoll.14-A554||Italy|658[0n]
Argynnis paphia|WMB5300-14|RVcoll.14-I615||France|658[0n]
Argynnis paphia|EULEP3474-16|RVcoll15H704||Switzerland|658[0n]
Argynnis paphia|EULEP3475-16|RVcoll15I081||Italy|658[0n]
Argynnis paphia|EULEP3476-16|RVcoll15I455||Austria|658[0n]
Argynnis paphia|EULEP3477-16|RVcoll15I720||Austria|658[0n]
Argynnis paphia|EULEP3478-16|RVcoll15Q120||Russia|658[0n]
Argynnis paphia|EULEP3479-16|RVcoll15P038||Ukraine|658[0n]
Argynnis paphia|EULEP5030-16|RVcoll16I970||Slovakia|658[0n]
Argynnis paphia|EULEP5050-16|RVcoll16J051||Czech Republic|658[0n]
Argynnis paphia|EULEP4905-16|RVcoll16J608||Russia|658[0n]
Argynnis paphia|EULEP4956-16|RVcoll16I244||Poland|658[0n]
Argynnis paphia|EULEP5001-16|RVcoll16I797||Germany|658[0n]
Argynnis paphia|EULEP085-14|RVcoll.08-P971||Romania|658[0n]
Argynnis paphia|EULEP172-14|RVcoll.11-J316||Romania|658[0n]
Argynnis paphia|EULEP1136-15|RVcoll.14-F292||Serbia|658[0n]
Argynnis paphia|EULEP1354-15|RVcoll.14-F916||Greece|658[0n]
Argynnis paphia|EULEP628-15|RVcoll.11-J014||Switzerland|658[0n]
Argynnis paphia|EULEP797-15|RVcoll.12-Z295||Sweden|658[0n]
Argynnis paphia|EULEP831-15|RVcoll.14-B946||Sweden|658[0n]
Argynnis paphia|EULEP866-15|RVcoll.14-C308||Greece|658[0n]
Argynnis paphia|EULEP977-15|RVcoll.14-D102||Austria|658[0n]
Argynnis paphia|EULEP1380-15|RVcoll.14-F979||Greece|658[0n]
Argynnis paphia|EULEP2375-15|RVcoll.14-V070||Ukraine|658[0n]
Argynnis paphia|EULEP2412-15|RVcoll.14-V187||Belgium|658[0n]
Argynnis paphia|EULEP1977-15|RVcoll.14-H901||France|658[0n]
Argynnis paphia|EULEP2025-15|RVcoll.14-I877||Poland|658[0n]
Argynnis paphia|EULEP2329-15|RVcoll.14-V006||Denmark|658[0n]
Argynnis paphia|BIBSA728-15|LD-2958||Italy|658[0n]
Argynnis paphia|BIBSA942-15|16-A033||Italy|658[0n]
Argynnis paphia|BIBSA1596-16|12-R022||Italy|658[0n]
Argynnis paphia|BIBSA1095-15|15-L350||Italy|658[0n]
Argynnis paphia|BIBSA1291-15|14-E278|Corsica|France|658[0n]
Argynnis paphia|BIBSA101-15|RVcoll.14-D524||Italy|658[0n]
Argynnis paphia|BIBSA122-15|RVcoll.14-D568||Italy|658[0n]
Argynnis paphia|BIBSA1522-16|LEP-SS-00367||Italy|658[0n]
Argynnis paphia|BIBSA497-15|LEP-SS-00117||Italy|658[0n]
Argynnis paphia|BIBSA498-15|LEP-SS-00118||Italy|658[0n]
Argynnis paphia|BIBSA765-15|LEP-SS-00276||Italy|658[0n]
Argynnis paphia|BIBSA179-15|RVcoll.14-D967||Italy|658[0n]
Argynnis paphia|BIBSA180-15|RVcoll.14-D968||Italy|658[0n]
Argynnis paphia|BIBSA345-15|RVcoll.14-I013||Italy|658[0n]
Argynnis paphia|GBLAB327-13|BC ZSM Lep 72628||Germany|658[0n]
Argynnis paphia|GBLAA354-14|BC ZSM Lep 80350||Germany|658[0n]
Argynnis paphia|GBLAA1474-15|BC ZSM Lep 87265||Germany|658[0n]
Argynnis paphia|BIBSA1597-16|12-R023||Italy|658[0n]
Argynnis paphia|GBLAA055-14|BC ZSM Lep 80716||Germany|658[0n]
Argynnis paphia|LEASS1048-17|TLMF Lep 22601||Austria|658[0n]
Argynnis paphia|LEFIJ4779-16|ZMBSU-02140||Belarus|658[0n]
Argynnis paphia|LEASS541-17|TLMF Lep 22189||Austria|658[0n]
Argynnis paphia|OXB1534-16|OXB-TGS-1252||France|658[0n]
Argynnis paphia|OXB762-15|13-U505||Italy|658[0n]
Argynnis paphia|OXB899-15|15-A543||Italy|658[0n]
Argynnis paphia|OXB233-15|OXB-TGS-506||United Kingdom|658[0n]
Argynnis paphia|OXB239-15|OXB-TGS-597||United Kingdom|658[0n]
Argynnis paphia|OXB1217-15|Rvcoll_15-M643||France|658[0n]
Argynnis paphia|GBLAA1334-15|BC ZSM Lep 87030||Germany|658[0n]
Argynnis paphia|LEFID864-10|MM06997||Finland|654[0n]
Argynnis paphia|EZSPC230-09|RVcoll.08-M682||Spain|654[0n]
Argynnis paphia|EZRMN340-08|RVcoll.08-H027||Romania|649[0n]
Argynnis paphia|EZRMN341-08|RVcoll.08-H028||Romania|649[0n]

[illegible]
Argynnis paphia|EZSPC230-09|RVcoll.08-M682||Spain|654[0n]
Argynnis paphia|EZRMN340-08|RVcoll.08-H027||Romania|649[0n]
Argynnis paphia|EZRMN341-08|RVcoll.08-H028||Romania|649[0n]
Argynnis paphia|EZSPC281-09|RVcoll.08-R303||Spain|651[0n]
Argynnis paphia|LEFIJ533-10|MM17158||Finland|638[0n]
Argynnis paphia|WMB820-13|RVcoll.07-E184||Italy|637[0n]
Argynnis paphia|WMB1103-13|RVcoll.10-C776||Italy|631[0n]
Argynnis paphia|EZSPM144-09|RVcoll.08-P656||Spain|632[0n]
Argynnis paphia|WMB3966-14|RVcoll.12-Q027||France|620[0n]
Argynnis paphia|OXB776-15|14-A727||Italy|622[0n]
Argynnis paphia|BIBSA1135-15|15-L946||Italy|623[0n]
Argynnis paphia|EZSPN651-09|RVcoll.08-J840||Spain|623[0n]
Argynnis paphia|LEATG445-14|TLMF Lep 14232||Italy|614[0n]
Argynnis paphia|LEATG062-14|TLMF Lep 13849||Austria|658[0n]
Fabriciana adippe|EZSPN621-09|RVcoll.08-J819||Spain|649[0n]
Fabriciana adippe|EZSPN1107-11|Rvcoll.10-B511||Spain|658[0n]
Fabriciana adippe|EZSPN1110-11|Rvcoll.10-B543||Spain|658[0n]
Fabriciana adippe|EZSPN582-09|RVcoll.08-J141||Portugal|658[0n]
Fabriciana adippe|EZSPN938-09|RVcoll.08-L901||Spain|658[0n]
Fabriciana adippe|EZSPM133-09|RVcoll.08-P643||Spain|658[0n]
Fabriciana adippe|EZSPC1378-10|RVcoll.09-X507||Spain|658[0n]
Fabriciana adippe|WMB3482-14|RVcoll.10-B542||Spain|658[0n]
Fabriciana adippe|WMB3639-14|RVcoll.11-I498||Spain|658[0n]
Fabriciana adippe|EZSPC927-10|RVcoll.09-V939||Spain|638[0n]
Fabriciana adippe|EZSPN559-09|RVcoll.08-J079||Spain|627[0n]
Fabriciana adippe|EZSPN468-09|RVcoll.08-H689||Spain|658[0n]
Fabriciana adippe|EZSPN1108-11|Rvcoll.10-B512||Spain|658[0n]
Fabriciana adippe|EZSPN1060-11|Rvcoll.08-P610|Genit. examined|Spain|658[0n]
Fabriciana adippe|EZSPN1047-11|Rvcoll.08-L177||Spain|658[0n]
Fabriciana adippe|EZSPC268-09|RVcoll.08-R163||Spain|658[0n]
Fabriciana adippe|GBLAA1462-15|BC ZSM Lep 87253||Germany|658[0n]
Fabriciana adippe|EZROM776-08|RVcoll.07-C913||Romania|658[0n]
Fabriciana adippe|OXB1387-15|Rvcoll_15-M752||France|658[0n]
Fabriciana adippe|GBLAD449-14|BC ZSM Lep 77975||Germany|657[0n]
Fabriciana adippe|GBLAA1333-15|BC ZSM Lep 87029||Germany|640[0n]
Fabriciana adippe|BIBSA1241-15|15-F811||France|658[0n]
Fabriciana adippe|EULEP3461-16|RVcoll15Q125||Russia|643[0n]
Fabriciana adippe|EULEP3456-16|RVcoll15G060||France|658[0n]
Fabriciana adippe|GWORK514-09|BC ZSM Lep 21844||Germany|655[0n]
Fabriciana adippe|GWORO720-09|BC ZSM Lep 27656|to adippe (AH)|Germany|658[0n]
Fabriciana adippe|EULEP4821-16|RVcoll16H717||Belgium|658[0n]
Fabriciana adippe|EULEP5083-16|RVcoll16J339||Germany|658[0n]
Fabriciana adippe|FBLMU446-09|BC ZSM Lep 27096||Germany|657[0n]
Fabriciana adippe|EZROM025-08|RV-06-M882||Romania|640[0n]
Fabriciana adippe|EULEP4806-16|RVcoll16H692||Norway|658[0n]
Fabriciana adippe|LEFIJ539-10|MM17164||Finland|658[0n]
Fabriciana adippe|EULEP5017-16|RVcoll16I918||Slovakia|658[0n]
Fabriciana adippe|EULEP133-14|RVcoll.10-C373||Romania|658[0n]
Fabriciana adippe|GBLAD223-14|BC ZSM Lep 78794||Germany|658[0n]
Fabriciana adippe|EZROM027-08|RV-07-C968||Romania|655[0n]
Fabriciana adippe|EZROM608-08|RV-07-D509|Genit. examined|Romania|658[0n]
Fabriciana adippe|EZROM026-08|RV-06-M950||Romania|658[0n]
Fabriciana adippe|EZROM572-08|RV-07-C347||Romania|658[0n]
Fabriciana adippe|EULEP3460-16|RVcoll15Q124||Russia|658[0n]
Fabriciana adippe|EULEP4793-16|RVcoll16H529||Sweden|658[0n]
Fabriciana adippe|EULEP4912-16|RVcoll16J615||Russia|658[0n]
Fabriciana adippe|EULEP4934-16|RVcoll16I033||Poland|658[0n]
Fabriciana adippe|EULEP4997-16|RVcoll16I759||Poland|658[0n]
Fabriciana adippe|EULEP1946-15|RVcoll.14-D662||Slovakia|658[0n]
Fabriciana adippe|GBLAD463-14|BC ZSM Lep 77989||Germany|657[0n]
Fabriciana adippe|EULEP312-14|MM23797||Latvia|618[0n]
Fabriciana adippe|EULEP3459-16|RVcoll15P007||Belarus|658[0n]
Fabriciana adippe|EULEP3458-16|RVcoll15Q105||Russia|658[0n]
Fabriciana adippe|LEFIJ538-10|MM17163||Finland|658[0n]
Fabriciana adippe|GWORO811-09|BC ZSM Lep 30503||Germany|658[0n]
Fabriciana adippe|FBLMU403-09|BC ZSM Lep 27053||Germany|657[0n]
Fabriciana adippe|LEFIG384-10|MM14397||Finland|658[0n]
Fabriciana adippe|BIBSA1601-16|12-R139||Italy|658[0n]
Fabriciana adippe|WMB2103-13|RVcoll.12-R116|Sicily|Italy|658[0n]
Fabriciana adippe|WMB2085-13|RVcoll.12-R024|Sicily|Italy|658[0n]
Fabriciana adippe|WMB2082-13|RVcoll.12-R010|Sicily|Italy|658[0n]
Fabriciana adippe|EZROM357-08|07-E611||Spain|658[0n]
Fabriciana adippe|EZSPN1099-11|Rvcoll.10-A823||Spain|658[0n]
Fabriciana adippe|EZSPC279-09|RVcoll.08-R279||Spain|656[0n]
Fabriciana adippe|BIBSA094-14|LEP-SS-00094||Italy|658[0n]
Fabriciana adippe|BCLEP109-17|LEP-SS-00560||Italy|658[0n]
Fabriciana adippe|BCLEP108-17|LEP-SS-00559||Italy|658[0n]
Fabriciana adippe|WMB4338-14|RVcoll.14-A728||Italy|658[0n]
Fabriciana adippe|WMB2062-13|RVcoll.12-Q849||Italy|658[0n]
Fabriciana adippe|GWORZ026-10|BC ZSM Lep 30382||Italy|658[0n]
Fabriciana adippe|GWORU354-10|BC ZSM Lep 31945||Italy|658[0n]
Fabriciana adippe|EULEP5707-17|RVcoll16L052||Italy|658[0n]
Fabriciana adippe|EZSPC282-09|RVcoll.08-R307||Spain|653[0n]
Fabriciana adippe|LEATG060-14|TLMF Lep 13847||Austria|658[0n]
Fabriciana adippe|EZROM356-08|07-E610||Spain|658[0n]
Fabriciana adippe|WMB1734-13|RVcoll.12-P941||France|658[0n]
Fabriciana adippe|WMB1835-13|RVcoll.11-I839||France|658[0n]
Fabriciana adippe|EULEP3451-16|RVcoll15H325||Switzerland|658[0n]
Fabriciana adippe|EULEP3452-16|RVcoll15H724||Switzerland|658[0n]
Fabriciana adippe|EULEP3454-16|RVcoll15I730||Austria|658[0n]
Fabriciana adippe|EULEP3455-16|RVcoll15J575||France|658[0n]
Fabriciana adippe|BIBSA1170-15|15-M194||Italy|658[0n]
Fabriciana adippe|BIBSA435-15|RVcoll.14-I103||Italy|658[0n]
Fabriciana adippe|GBLAA1440-15|BC ZSM Lep 87231||Germany|658[0n]
Fabriciana adippe|OXB1536-16|OXB-TGS-1254||France|658[0n]
Fabriciana adippe|OXB1301-15|Rvcoll_15-M168||France|658[0n]
Fabriciana adippe|WMB5049-14|RVcoll. 14-L180||Italy|656[0n]
Fabriciana adippe|EULEP3457-16|RVcoll15G140||France|635[0n]
Fabriciana adippe|LEATH712-14|TLMF Lep 15924||Italy|634[0n]
Fabriciana adippe|LEATC622-13|TLMF Lep 12604||Austria|658[0n]
Fabriciana adippe|LEATG061-14|TLMF Lep 13848||Austria|658[0n]
Fabriciana adippe|EULEP1951-15|RVcoll.14-D697||Greece|658[0n]
[illegible]

Fabriciana adippe|LEATC622-13|TLMF Lep 12604||Austria|658[0n]
Fabriciana adippe|LEATG061-14|TLMF Lep 13848||Austria|658[0n]
Fabriciana adippe|EULEP1951-15|RVcoll.14-D697||Greece|658[0n]
Fabriciana adippe|EULEP1949-15|RVcoll.14-D688||Serbia|658[0n]
Fabriciana adippe|OXB346-15|RVcoll.14-N060||Italy|658[0n]
Fabriciana adippe|BIBSA1432-16|RVcoll.14-I186||Italy|657[1n]
Fabriciana adippe|EULEP1898-15|RVcoll.14-B768||Albania|658[0n]
Fabriciana adippe|WMB6545-18|RVcoll13T322||Italy|658[0n]
Fabriciana adippe|WMB4321-14|RVcoll.14-A579||Italy|658[0n]
Fabriciana adippe|WMB4312-14|RVcoll.14-A467||Italy|658[0n]
Fabriciana adippe|WMB3972-14|RVcoll.12-Q143||France|658[0n]
Fabriciana adippe|WMB1597-13|RVcoll.12-O618||France|658[0n]
Fabriciana adippe|WMB3501-14|RVcoll.10-B745||France|658[0n]
Fabriciana adippe|BIBSA967-15|13-U477||Italy|658[0n]
Fabriciana adippe|ABOLD014-16|TLMF Lep 21092||Austria|658[0n]
Fabriciana elisa|EULEP3469-16|RVcoll14E315|Corsica|France|658[0n]
Fabriciana elisa|EULEP2628-15|RVcoll.14-N568|Corsica|France|658[0n]
Fabriciana elisa|EULEP2627-15|RVcoll.14-N567|Corsica|France|658[0n]
Fabriciana elisa|WMB4649-14|RVcoll.LD-2447|Corsica|France|621[0n]
Fabriciana elisa|WMB4648-14|RVcoll.LD-2446|Corsica|France|658[0n]
Fabriciana elisa|WMB760-12|RVcoll.11-J967|Sardinia|Italy|640[0n]
Fabriciana elisa|WMB1897-13|RVcoll.11-J968|Sardinia|Italy|658[0n]
Fabriciana elisa|WMB905-13|RVcoll.09-X872|Sardinia|Italy|658[0n]
Fabriciana elisa|WMB233-11|RVcoll.09-T562|Sardinia|Italy|658[0n]
Fabriciana niobe|EULEP1953-15|RVcoll.14-E500||Greece|658[0n]
Fabriciana niobe|EULEP1952-15|RVcoll.14-D699||Greece|658[0n]
Fabriciana niobe|BIBSA095-14|LEP-SS-00095||Italy|658[0n]
Fabriciana niobe|BIBSA495-15|LEP-SS-00115||Italy|658[0n]
Fabriciana niobe|BIBSA724-15|LD-2766||Italy|658[0n]
Fabriciana niobe|BIBSA723-15|LD-2765||Italy|658[0n]
Fabriciana niobe|BIBSA671-15|RVcoll. 07-G480||Italy|658[0n]
Fabriciana niobe|BIBSA1339-15|15-M831||Italy|658[0n]
Fabriciana niobe|BIBSA760-15|LEP-SS-00261||Italy|658[0n]
Fabriciana niobe|BIBSA1154-15|15-L100||Italy|658[0n]
Fabriciana niobe|BIBSA1153-15|15-L094||Italy|658[0n]
Fabriciana niobe|WMB4322-14|RVcoll.14-A611||Italy|658[0n]
Fabriciana niobe|WMB4007-14|RVcoll.12-Q701||Italy|658[0n]
Fabriciana niobe|WMB2259-13|RVcoll.12-R420||Italy|658[0n]
Fabriciana niobe|WMB2022-13|RVcoll.12-Q691||Italy|658[0n]
Fabriciana niobe|WMB2061-13|RVcoll.12-Q848||Italy|658[0n]
Fabriciana niobe|WMB844-13|RVcoll.07-G478||Italy|658[0n]
Fabriciana niobe|WMB802-13|RVcoll.07-E024||Italy|658[0n]
Fabriciana niobe|EZROM781-08|RVcoll.07-D049||Romania|658[0n]
Fabriciana niobe|EZROM783-08|RVcoll.08-M427||Romania|658[0n]
Fabriciana niobe|EZROM782-08|RVcoll.08-M473||Romania|658[0n]
Fabriciana niobe|GBLAD464-14|BC ZSM Lep 77990||Germany|658[0n]
Fabriciana niobe|EZROM599-08|RV-07-D052||Romania|638[0n]
Fabriciana niobe|EZSPC1083-10|RVcoll.08-L736||Spain|658[0n]
Fabriciana niobe|EZSPN904-09|RVcoll.08-L823||Spain|658[0n]
Fabriciana niobe|EZSPN730-09|RVcoll.08-L138||Spain|658[0n]
Fabriciana niobe|EZSPN528-09|RVcoll.08-H998||Spain|658[0n]
Fabriciana niobe|EZSPN655-09|RVcoll.08-J951||Spain|658[0n]
Fabriciana niobe|EZSPC886-10|RVcoll.09-T149||Spain|658[0n]
Fabriciana niobe|EZSPC1201-10|RVcoll.09-V426||Spain|658[0n]
Fabriciana niobe|EZSPC1218-10|RVcoll.09-V531||Spain|658[0n]
Fabriciana niobe|EULEP1954-15|RVcoll.14-E517||Greece|658[0n]
Fabriciana niobe|EZSPC233-09|RVcoll.08-M901||Spain|629[0n]
Fabriciana niobe|EZSPN825-09|RVcoll.08-L637||Spain|658[0n]
Fabriciana niobe|LEFIJ280-10|MM14896||Finland|658[0n]
Fabriciana niobe|LEFIJ279-10|MM14895||Finland|658[0n]
Fabriciana niobe|LEFIJ540-10|MM17165||Finland|658[0n]
Fabriciana niobe|LEFIJ4777-16|ZMBSU-02139||Belarus|612[0n]
Fabriciana niobe|EULEP3472-16|RVcoll15I405||Austria|658[0n]
Fabriciana niobe|WMB3513-14|RVcoll.10-B860||France|658[0n]
Fabriciana niobe|BIBSA178-15|RVcoll.14-D966||Italy|658[0n]
Fabriciana niobe|WMB3951-14|RVcoll.12-P714||France|658[0n]
Fabriciana niobe|EZROM359-08|07-E613||Spain|618[0n]
Fabriciana niobe|EZROM358-08|07-E612||France|658[0n]
Fabriciana niobe|EZSPC264-09|RVcoll.08-R114||Spain|658[0n]
Fabriciana niobe|EZSPC247-09|RVcoll.08-P220||Spain|656[0n]
Fabriciana niobe|EZSPC242-09|RVcoll.08-P050||Spain|657[0n]
Fabriciana niobe|EZROM034-08|RV-06-M980||Romania|658[0n]
Fabriciana niobe|WMB1849-13|RVcoll.11-I938||France|658[0n]
Fabriciana niobe|WMB3888-14|RVcoll.12-O684||France|658[0n]
Fabriciana niobe|WMB3982-14|RVcoll.12-Q250||France|658[0n]
Fabriciana niobe|WMB4993-14|RVcoll. 14-I540||Italy|658[0n]
Fabriciana niobe|WMB4994-14|RVcoll. 14-I541||Italy|658[0n]
Fabriciana niobe|EULEP3473-16|RVcoll15J425||France|658[0n]
Fabriciana niobe|EULEP139-14|RVcoll.10-C407||Romania|658[0n]
Fabriciana niobe|EULEP1948-15|RVcoll.14-D681||Serbia|658[0n]
Fabriciana niobe|EULEP1950-15|RVcoll.14-D691||Bulgaria|658[0n]
Fabriciana niobe|BIBSA1431-16|RVcoll.14-I185||Italy|658[0n]
Fabriciana niobe|BIBSA285-15|RVcoll.14-E085||Italy|658[0n]
Fabriciana niobe|BIBSA287-15|RVcoll.14-E087||Italy|658[0n]
Fabriciana niobe|BIBSA359-15|RVcoll.14-I027||Italy|658[0n]
Fabriciana niobe|BIBSA360-15|RVcoll.14-I028||Italy|658[0n]
Fabriciana niobe|BIBSA361-15|RVcoll.14-I029||Italy|658[0n]
Fabriciana niobe|BIBSA362-15|RVcoll.14-I030||Italy|658[0n]
Fabriciana niobe|LEFIJ4726-16|ZMBSU-02138||Belarus|658[0n]
Fabriciana niobe|LEASS488-17|TLMF Lep 22136||Austria|658[0n]
Fabriciana niobe|OXB1179-15|Rvcoll_16-A066||Italy|658[0n]
Fabriciana niobe|BIBSA177-15|RVcoll.14-D965||Italy|615[0n]
Fabriciana niobe|EULEP1947-15|RVcoll.14-D664||France|614[0n]
Fabriciana niobe|WMB1617-13|RVcoll.12-O750||France|639[0n]
Fabriciana niobe|WMB3517-14|RVcoll.10-B914||France|658[0n]
Fabriciana niobe|WMB3511-14|RVcoll.10-B817||France|658[0n]
Fabriciana niobe|WMB3676-14|RVcoll.11-J158||France|658[0n]
Fabriciana niobe|EZROM035-08|RV-07-D499||Romania|658[0n]
Fabriciana niobe|EZRMN337-08|RVcoll.08-H017||Romania|658[0n]
Fabriciana niobe|GWORA2504-09|BC ZSM Lep 30716||Germany|658[0n]
Fabriciana niobe|GWORO812-09|BC ZSM Lep 30504||Germany|658[0n]
Fabriciana niobe|LEATG058-14|TLMF Lep 13845||Austria|658[0n]
Fabriciana niobe|ABOLD032-16|TLMF Lep 21110||Austria|658[0n]

[illegible]
Fabriciana niobe|GWORO812-09|BC ZSM Lep 30504||Germany|658[0n]
Fabriciana niobe|LEATG058-14|TLMF Lep 13845||Austria|658[0n]
Fabriciana niobe|ABOLD032-16|TLMF Lep 21110||Austria|658[0n]
Fabriciana niobe|EULEP3471-16|RVcoll15H598||Switzerland|658[0n]
Fabriciana niobe|EULEP3470-16|RVcoll15G855||Italy|658[0n]
Fabriciana niobe|WMB5122-14|RVcoll. 14-L253||Italy|658[0n]
Fabriciana niobe|LEATH793-14|TLMF Lep 16005||Italy|658[0n]
Fabriciana niobe|LEATC096-13|TLMF Lep 11223||Italy|658[0n]
Fabriciana niobe|LEATG059-14|TLMF Lep 13846||Austria|658[0n]
Fabriciana niobe|PHLAH743-12|TLMF Lep 08562||Austria|658[0n]
Speyeria aglaja|EULEP2016-15|RVcoll.14-I823||Slovakia|658[0n]
Speyeria aglaja|EULEP041-14|RVcoll.08-H011||Romania|658[0n]
Speyeria aglaja|EULEP2257-15|RVcoll.14-N441||Ukraine|658[0n]
Speyeria aglaja|WMB3301-14|RVcoll.08-P411||Spain|616[0n]
Speyeria aglaja|WMB3403-14|RVcoll.09-V809||Spain|658[0n]
Speyeria aglaja|EZSPM227-09|RVcoll.08-R419||Spain|658[0n]
Speyeria aglaja|EZSPN622-09|RVcoll.08-J854||Spain|658[0n]
Speyeria aglaja|EZSPN721-09|RVcoll.08-L110||Spain|658[0n]
Speyeria aglaja|EZSPC266-09|RVcoll.08-R150||Spain|658[0n]
Speyeria aglaja|EZSPC236-09|RVcoll.08-M928||Spain|658[0n]
Speyeria aglaja|EZSPN879-09|RVcoll.08-L782||Spain|658[0n]
Speyeria aglaja|OXB1514-16|RVcoll14W391||United Kingdom|658[0n]
Speyeria aglaja|OXB1511-16|RVcoll14W404||United Kingdom|658[0n]
Speyeria aglaja|EZSPC1036-10|RVcoll.08-J217||Spain|658[0n]
Speyeria aglaja|EZSPC1202-10|RVcoll.09-V428||Spain|658[0n]
Speyeria aglaja|EULEP789-15|RVcoll.12-Z221||Sweden|658[0n]
Speyeria aglaja|EULEP4798-16|RVcoll16H597||Sweden|658[0n]
Speyeria aglaja|OXB1513-16|RVcoll14W392||United Kingdom|658[0n]
Speyeria aglaja|EULEP2328-15|RVcoll.14-V000||Denmark|658[0n]
Speyeria aglaja|EULEP2625-15|RVcoll.14-N565||United Kingdom|658[0n]
Speyeria aglaja|GBLAB326-13|BC ZSM Lep 72627||Germany|658[0n]
Speyeria aglaja|OXB1508-16|RVcoll14W447||United Kingdom|658[0n]
Speyeria aglaja|OXB1509-16|RVcoll14W446||United Kingdom|658[0n]
Speyeria aglaja|OXB1510-16|RVcoll14W445||United Kingdom|658[0n]
Speyeria aglaja|OXB1512-16|RVcoll14W393||United Kingdom|658[0n]
Speyeria aglaja|OXB1515-16|RVcoll14W390||United Kingdom|658[0n]
Speyeria aglaja|OXB199-15|OXB-TGS-152||United Kingdom|658[0n]
Speyeria aglaja|OXB201-15|OXB-TGS-171||United Kingdom|658[0n]
Speyeria aglaja|OXB217-15|OXB-TGS-355||United Kingdom|658[0n]
Speyeria aglaja|OXB280-15|OXB-TGS-963||United Kingdom|658[0n]
Speyeria aglaja|OXB1353-15|Rvcoll_15-M705||France|658[0n]
Speyeria aglaja|OXB1381-15|Rvcoll_15-M745||France|658[0n]
Speyeria aglaja|EZSPN851-09|RVcoll.08-L698||Spain|646[0n]
Speyeria aglaja|LEFIC070-10|MM03355||Finland|658[0n]
Speyeria aglaja|LEFID541-10|MM06526||Finland|658[0n]
Speyeria aglaja|LEFIJ537-10|MM17162||Finland|658[0n]
Speyeria aglaja|BIBSA093-14|LEP-SS-00093||Italy|632[0n]
Speyeria aglaja|BIBSA615-15|LEP-SS-00235||Italy|658[0n]
Speyeria aglaja|BIBSA496-15|LEP-SS-00116||Italy|658[0n]
Speyeria aglaja|BIBSA1371-15|15-M916||Italy|658[0n]
Speyeria aglaja|WMB4290-14|RVcoll.14-A359||Italy|658[0n]
Speyeria aglaja|WMB2060-13|RVcoll.12-Q839||Italy|658[0n]
Speyeria aglaja|WMB2781-13|RVcoll.11-I196||Italy|658[0n]
Speyeria aglaja|EZSPC256-09|RVcoll.08-P360||Spain|655[0n]
Speyeria aglaja|EZSPN999-09|RVcoll.08-M082||Spain|658[0n]
Speyeria aglaja|WMB3182-14|RVcoll.08-H691||Spain|658[0n]
Speyeria aglaja|WMB3324-14|RVcoll.08-P741||Spain|658[0n]
Speyeria aglaja|WMB640-11|RVcoll.11-I152||Italy|658[0n]
Speyeria aglaja|WMB4914-14|RVcoll.14-I461||Italy|658[0n]
Speyeria aglaja|EZSPC248-09|RVcoll.08-P222||Spain|648[0n]
Speyeria aglaja|EZSPC208-09|RVcoll.07-E637||Spain|656[0n]
Speyeria aglaja|EZROM029-08|RV-06-M932||Romania|658[0n]
Speyeria aglaja|EZROM028-08|RV-06-M881||Romania|658[0n]
Speyeria aglaja|GBLAD164-14|BC ZSM Lep 78735||Germany|658[0n]
Speyeria aglaja|EULEP193-14|RVcoll.11-J955||Romania|658[0n]
Speyeria aglaja|EULEP5019-16|RVcoll16I931||Slovakia|658[0n]
Speyeria aglaja|EULEP5003-16|RVcoll16I804||Germany|658[0n]
Speyeria aglaja|EULEP3468-16|RVcoll15P006||Belarus|658[0n]
Speyeria aglaja|EULEP3462-16|RVcoll15G046||France|658[0n]
Speyeria aglaja|EZROM030-08|RV-06-M936||Romania|658[0n]
Speyeria aglaja|EZROM778-08|RVcoll.07-D526||Romania|658[0n]
Speyeria aglaja|EZROM600-08|RV-07-D517||Romania|658[0n]
Speyeria aglaja|FBLMT912-09|BC ZSM Lep 25472||Germany|658[0n]
Speyeria aglaja|GWOSA735-10|BC ZSM Lep 35746||Germany|658[0n]
Speyeria aglaja|FBLMU111-09|BC ZSM Lep 25621||Germany|658[0n]
Speyeria aglaja|GWORO810-09|BC ZSM Lep 30502||Germany|658[0n]
Speyeria aglaja|FBLMU414-09|BC ZSM Lep 27064||Germany|658[0n]
Speyeria aglaja|GWOTF681-12|BC ZSM Lep 62387||Germany|658[0n]
Speyeria aglaja|EULEP3463-16|RVcoll15G846||Italy|658[0n]
Speyeria aglaja|EULEP967-15|RVcoll.14-D040||Bulgaria|658[0n]
Speyeria aglaja|WMB839-13|RVcoll.07-E236||Italy|649[0n]
Speyeria aglaja|LEATF458-14|TLMF Lep 13770||Austria|658[0n]
Speyeria aglaja|EULEP860-15|RVcoll.14-C207||Greece|658[0n]
Speyeria aglaja|BIBSA176-15|RVcoll.14-D964||Italy|658[0n]
Speyeria aglaja|BIBSA286-15|RVcoll.14-E086||Italy|658[0n]
Speyeria aglaja|BIBSA358-15|RVcoll.14-I026||Italy|658[0n]
Speyeria aglaja|BIBSA436-15|RVcoll.14-I104||Italy|658[0n]
Speyeria aglaja|LEASS481-17|TLMF Lep 22129||Austria|658[0n]
Speyeria aglaja|OXB601-15|14-N990||Italy|658[0n]
Speyeria aglaja|OXB610-15|14-N999||Italy|658[0n]
Speyeria aglaja|OXB326-15|RVcoll.14-N040||Italy|658[0n]
Speyeria aglaja|ABOLD662-17|TLMF Lep 21738||Austria|658[0n]
Speyeria aglaja|OXB1285-15|Rvcoll_15-M148||France|622[0n]
Speyeria aglaja|BIBSA175-15|RVcoll.14-D963||Italy|613[0n]
Speyeria aglaja|BIBSA1857-17|RVcoll16C741||Italy|658[0n]
Speyeria aglaja|BIBSA1098-15|15-L354||Italy|658[0n]
Speyeria aglaja|BIBSA1086-15|15-K575||Italy|658[0n]
Speyeria aglaja|BIBSA1071-15|15-H947||Italy|658[0n]
Speyeria aglaja|BIBSA1492-16|RVcoll.14-I246||Italy|658[0n]
Speyeria aglaja|EULEP1915-15|RVcoll.14-B819||Bosnia and Herzegovina|658[0n]
Speyeria aglaja|EULEP2444-15|RVcoll.14-V251||Switzerland|658[0n]
Speyeria aglaja|EULEP1651-15|RVcoll.14-G437||Greece|658[0n]
[illegible]

Speyeria aglaja|EULEP1913-15|RVcoll.14-B819||Bosnia and Herzegovina|658[0n]
Speyeria aglaja|EULEP2444-15|RVcoll.14-V251||Switzerland|658[0n]
Speyeria aglaja|EULEP1651-15|RVcoll.14-G437||Greece|658[0n]
Speyeria aglaja|EULEP1448-15|RVcoll.14-G164||Greece|658[0n]
Speyeria aglaja|EULEP986-15|RVcoll.14-D159||Austria|658[0n]
Speyeria aglaja|EULEP629-15|RVcoll.11-J015||Switzerland|658[0n]
Speyeria aglaja|EULEP1347-15|RVcoll.14-F892||Greece|658[0n]
Speyeria aglaja|EULEP1048-15|RVcoll.14-E917||Serbia|658[0n]
Speyeria aglaja|EULEP3467-16|RVcoll15J456||France|658[0n]
Speyeria aglaja|EULEP3466-16|RVcoll15I734||Austria|658[0n]
Speyeria aglaja|EULEP3465-16|RVcoll15I036||Italy|658[0n]
Speyeria aglaja|EULEP3464-16|RVcoll15H586||Switzerland|658[0n]
Speyeria aglaja|WMB5024-14|RVcoll. 14-I571||Italy|658[0n]
Speyeria aglaja|WMB4998-14|RVcoll. 14-I545||Italy|658[0n]
Speyeria aglaja|WMB733-12|RVcoll.11-I712||France|658[0n]
Speyeria aglaja|WMB3979-14|RVcoll.12-Q229||France|658[0n]
Speyeria aglaja|WMB3978-14|RVcoll.12-Q228||France|658[0n]
Speyeria aglaja|WMB3829-14|RVcoll.09-X861||Italy|658[0n]
Speyeria aglaja|WMB2002-13|RVcoll.12-Q389||France|658[0n]
Speyeria aglaja|WMB3515-14|RVcoll.10-B874||France|658[0n]
Speyeria aglaja|WMB3675-14|RVcoll.11-I998||France|658[0n]
Speyeria aglaja|EZROM031-08|RV-07-D935||Romania|658[0n]
Speyeria aglaja|EZROM777-08|RVcoll.07-D496||Romania|658[0n]
Speyeria aglaja|PHLAH742-12|TLMF Lep 08561||Austria|658[0n]
Speyeria aglaja|PHLAI483-13|TLMF Lep 09045||Austria|658[0n]
Speyeria aglaja|LEATC571-13|TLMF Lep 12553||Italy|658[0n]
Speyeria aglaja|PHLAC443-10|TLMF Lep 02478||Italy|658[0n]
Apatura metis|EULEP1594-15|RVcoll.14-G637||Greece|658[1n]
Apatura metis|EULEP1592-15|RVcoll.14-G631||Greece|658[0n]
Apatura metis|EULEP1582-15|RVcoll.14-G609||Greece|649[0n]
Apatura ilia|EULEP1123-15|RVcoll.14-F224||Serbia|658[0n]
Apatura metis|EULEP2606-15|RVcoll.14-N546||Albania|658[0n]
Apatura metis|EULEP2605-15|RVcoll.14-N545||Montenegro|658[0n]
Apatura metis|EULEP2610-15|RVcoll.14-N550||Hungary|647[0n]
Apatura metis|EULEP2609-15|RVcoll.14-N549||Hungary|658[0n]
Apatura metis|EULEP2608-15|RVcoll.14-N548||Serbia|658[0n]
Apatura metis|EULEP2607-15|RVcoll.14-N547||Serbia|658[0n]
Apatura metis|EULEP2534-15|RVcoll.14-V421||Greece|620[0n]
Apatura metis|EULEP1965-15|RVcoll.14-G828||Romania|658[0n]
Apatura metis|EULEP1824-15|RVcoll.14-J944||Bulgaria|658[0n]
Apatura metis|EZROM702-08|RV-07-F566|Genit. examined|Romania|658[0n]
Apatura metis|EZROM701-08|RV-07-F565||Romania|658[0n]
Apatura metis|EZROM014-08|RV-07-E441||Romania|658[0n]
Apatura metis|EZRMN376-08|RVcoll.08-M779||Romania|658[0n]
Apatura metis|EZROM013-08|RV-07-D010|Genit. examined|Romania|658[0n]
Apatura ilia|FBLMU464-09|BC ZSM Lep 27114||Germany|658[0n]
Apatura ilia|EZSPC872-10|RVcoll.09-T123||Spain|658[0n]
Apatura ilia|EULEP4838-16|RVcoll16H744||Ukraine|658[0n]
Apatura ilia|EULEP3426-16|RVcoll15P012||Belarus|641[0n]
Apatura ilia|LOWA861-06|2005-LOWA-861||Russia|658[0n]
Apatura ilia|LEFIJ4715-16|ZMBSU-02134||Belarus|658[0n]
Apatura ilia|LEASS997-17|TLMF Lep 22550||Austria|658[0n]
Apatura ilia|EULEP2603-15|RVcoll.14-N543||Estonia|658[0n]
Apatura ilia|EULEP2264-15|RVcoll.14-N449||Ukraine|658[0n]
Apatura ilia|EULEP939-15|RVcoll.14-C920||Bulgaria|658[0n]
Apatura ilia|EULEP1026-15|RVcoll.14-E824||Serbia|658[0n]
Apatura ilia|EULEP5890-18|RVcoll15O595||Spain|658[0n]
Apatura ilia|LOWA862-06|2005-LOWA-862||Russia|658[0n]
Apatura ilia|EZRMN371-08|RVcoll.08-M101|Genit. examined|Romania|658[0n]
Apatura ilia|ABOLD664-17|TLMF Lep 21740||Austria|658[0n]
Apatura ilia|ABOLD579-17|TLMF Lep 21655||Austria|658[0n]
Apatura ilia|LEFIJ4766-16|ZMBSU-02135||Belarus|621[0n]
Apatura ilia|EZSPC874-10|RVcoll.09-T125||Spain|658[0n]
Apatura ilia|LEFID153-10|MM05916||Finland|658[0n]
Apatura ilia|LEFIJ760-10|MM17385||Finland|658[0n]
Apatura ilia|LEFIJ535-10|MM17160||Finland|658[0n]
Apatura ilia|ABOLD012-16|TLMF Lep 21090||Austria|658[0n]
Apatura ilia|BIBSA337-15|RVcoll.14-I005||Italy|658[0n]
Apatura ilia|EZROM760-08|RVcoll.08-M661||Romania|658[0n]
Apatura ilia|EZROM759-08|RVcoll.06-M893||Romania|658[0n]
Apatura ilia|EZSPC086-09|RVcoll.08-L449||Spain|658[0n]
Apatura ilia|EZSPC640-09|RVcoll.08-L381||Spain|658[0n]
Apatura ilia|EZSPM587-12|RVcoll.08-P960||Spain|658[0n]
Apatura ilia|EZSPN279-09|RVcoll.07-W211||Spain|658[0n]
Apatura ilia|WMB5126-14|RVcoll. 14-L257||Italy|658[0n]
Apatura ilia|EZSPC087-09|RVcoll.08-R194||Spain|658[0n]
Apatura ilia|EULEP186-14|RVcoll.11-J883||Romania|658[0n]
Apatura ilia|EULEP142-14|RVcoll.10-C420||Romania|658[0n]
Apatura ilia|EULEP1017-15|RVcoll.14-E680||Romania|658[0n]
Apatura ilia|EULEP994-15|RVcoll.14-E156||Romania|658[0n]
Apatura ilia|BIBSA738-15|LEP-SS-00265||Italy|658[0n]
Apatura ilia|BIBSA739-15|LEP-SS-00266||Italy|658[0n]
Apatura ilia|BIBSA743-15|LEP-SS-00270||Italy|658[0n]
Apatura ilia|BIBSA744-15|LEP-SS-00271||Italy|658[0n]
Apatura ilia|BIBSA947-15|16-A038||Italy|658[0n]
Apatura ilia|GBLAC571-13|BC ZSM Lep 78192||Germany|658[0n]
Apatura ilia|LEASS897-17|KLM Lep 08402||Austria|658[0n]
Apatura ilia|OXB847-15|14-U810||Italy|658[0n]
Apatura ilia|EZSPN027-09|RVcoll.09-T050||Spain|658[3n]
Apatura ilia|EULEP2604-15|RVcoll.14-N544||France|644[0n]
Apatura ilia|EZROM009-08|RV-06-M872|Genit. examined|Romania|658[1n]
Apatura ilia|EZROM010-08|RV-06-M898||Romania|658[0n]
Apatura ilia|GWOSA731-10|BC ZSM Lep 35742||Germany|658[0n]
Apatura ilia|FBLMU109-09|BC ZSM Lep 25619||Germany|658[0n]
Apatura ilia|GWORA2515-09|BC ZSM Lep 30727||Germany|658[0n]
Apatura ilia|FBLMU463-09|BC ZSM Lep 27113||Germany|658[0n]
Apatura ilia|PHLAW056-13|TLMF Lep 09853||Austria|658[0n]
Apatura iris|EZROM762-08|RVcoll.08-M553||Romania|658[0n]
Apatura iris|EZROM012-08|RV-06-M870||Romania|632[0n]
Apatura iris|EULEP1027-15|RVcoll.14-E831||Serbia|658[0n]
Apatura iris|EZROM011-08|RV-06-M869||Romania|658[0n]
Apatura iris|EZROM765-08|RVcoll.08-M606||Romania|658[0n]
Apatura iris|EZROM761-08|RVcoll.06-M871||Romania|658[0n]

Apatura iris|EZROM011-08|RV-06-M869||Romania|658[0n]
Apatura iris|EZROM765-08|RVcoll.08-M606||Romania|658[0n]
Apatura iris|EZROM761-08|RVcoll.06-M871||Romania|658[0n]
Apatura iris|EULEP877-15|RVcoll.14-C408||Greece|658[0n]
Apatura iris|EZROM764-08|RVcoll.08-M595||Romania|658[0n]
Apatura iris|EZROM763-08|RVcoll.08-M591||Romania|658[0n]
Apatura iris|EZSPC919-10|RVcoll.09-V892||Spain|658[0n]
Apatura iris|LEATJ1200-16|TLMF Lep 19523||Czech Republic|658[0n]
Apatura iris|EULEP3428-16|RVcoll15G065||France|658[0n]
Apatura iris|GWOSI560-10|BC ZSM Lep 44406||Germany|658[0n]
Apatura iris|FBLMX206-11|BC ZSM Lep 50417||Germany|658[0n]
Apatura iris|EULEP214-14|RVcoll.12-L037||Spain|658[0n]
Apatura iris|EULEP079-14|RVcoll.08-P964||Spain|658[0n]
Apatura iris|EZSPM844-12|Rvcoll. 150111HJ81||Spain|658[0n]
Apatura iris|LEASS763-17|TLMF Lep 22411||Austria|658[0n]
Apatura iris|LEFIJ4765-16|ZMBSU-02114||Belarus|658[0n]
Apatura iris|GWOTL176-13|BC ZSM Lep 67107||Germany|658[0n]
Apatura iris|GWOTL175-13|BC ZSM Lep 67106||Germany|658[0n]
Apatura iris|EULEP809-15|RVcoll.13-U448||Italy|658[0n]
Apatura iris|EULEP199-14|RVcoll.12-L010||Spain|658[0n]
Apatura iris|EULEP198-14|RVcoll.12-L009||Spain|658[0n]
Apatura iris|EULEP3427-16|RVcoll15I456||Austria|658[0n]
Apatura iris|EZSPM855-12|Rvcoll. 300511SB99||Spain|658[0n]
Apatura iris|EZSPC873-10|RVcoll.09-T124||Spain|658[0n]
Apatura iris|EZSPM854-12|Rvcoll. 300511SB98||Spain|658[0n]
Apatura iris|EZSPM666-12|Rvcoll. 12-L279||Spain|658[0n]
Apatura iris|EZSPN063-09|RVcoll.09-V716||Spain|658[0n]
Apatura iris|EZSPN031-09|RVcoll.09-T066||Spain|658[0n]
Apatura iris|EZSPC088-09|RVcoll.08-L533||Spain|658[0n]
Apatura iris|GWOSA729-10|BC ZSM Lep 35740||Germany|658[0n]
Apatura iris|LEFID151-10|MM05914||Finland|658[0n]
Apatura iris|LEFIB065-10|MM00353||Finland|658[0n]
Apatura iris|LEFIJ759-10|MM17384||Finland|658[0n]
Apatura iris|GWORA2513-09|BC ZSM Lep 30725||Germany|658[0n]
Apatura iris|GWORA2512-09|BC ZSM Lep 30724||Germany|658[0n]
Apatura iris|LASTS096-14|TLMF Lep 14548||Austria|658[0n]
Apatura iris|LEATG220-14|TLMF Lep 14007||Austria|658[0n]
Apatura iris|LEATA079-13|TLMF Lep 09496||Italy|658[0n]
Apatura iris|LEATA414-13|TLMF Lep 10021||Austria|658[0n]
Apatura iris|LEATG396-14|TLMF Lep 14183||Italy|658[0n]
Araschnia levana|PHLAI565-13|TLMF Lep 09127||Austria|658[0n]
Araschnia levana|EZROM021-08|RV-06-M926||Romania|656[0n]
Araschnia levana|LEFIG238-10|MM14152||Finland|658[0n]
Araschnia levana|FBLMU447-09|BC ZSM Lep 27097||Germany|658[0n]
Araschnia levana|LEFID200-10|MM06026||Finland|658[0n]
Araschnia levana|LEFID201-10|MM06027||Finland|658[0n]
Araschnia levana|FBLMT893-09|BC ZSM Lep 25453||Germany|658[0n]
Araschnia levana|EZROM020-08|RV-06-K530||Romania|658[0n]
Araschnia levana|EZROM771-08|RVcoll.07-C999||Romania|658[0n]
Araschnia levana|EZROM772-08|RVcoll.07-D566||Romania|658[0n]
Araschnia levana|EZROM571-08|RV-07-C348||Romania|658[0n]
Araschnia levana|EZROM019-08|RV-06-K528||Romania|658[0n]
Araschnia levana|EZROM022-08|RV-07-D599||Romania|658[0n]
Araschnia levana|EZSPC245-09|RVcoll.08-P062||Spain|657[0n]
Araschnia levana|EZSPM404-09|Rvcoll.09-V810||Spain|658[0n]
Araschnia levana|EZSPM405-09|Rvcoll.09-V811||Spain|658[0n]
Araschnia levana|EZSPC1377-10|RVcoll.09-X300||Spain|658[0n]
Araschnia levana|LOWA778-06|2005-LOWA-778||Russia|658[0n]
Araschnia levana|LOWA779-06|2005-LOWA-779||Russia|658[0n]
Araschnia levana|EULEP3443-16|RVcoll10A769||Estonia|658[0n]
Araschnia levana|EULEP3444-16|RVcoll15I528||Austria|658[0n]
Araschnia levana|EULEP3445-16|RVcoll15I745||Austria|658[0n]
Araschnia levana|EULEP3448-16|RVcoll15P035||Ukraine|658[0n]
Araschnia levana|EULEP3449-16|RVcoll15Q195||Ukraine|658[0n]
Araschnia levana|EULEP5045-16|RVcoll16J041||Slovakia|658[0n]
Araschnia levana|EULEP107-14|RVcoll.09-V628||Romania|658[0n]
Araschnia levana|EULEP173-14|RVcoll.11-J322||Romania|658[0n]
Araschnia levana|EULEP1201-15|RVcoll.14-F493||Serbia|658[0n]
Araschnia levana|EULEP615-15|RVcoll.11-H129||Germany|658[0n]
Araschnia levana|EULEP787-15|RVcoll.12-Z205||Sweden|658[0n]
Araschnia levana|EULEP2420-15|RVcoll.14-V200||Belgium|658[0n]
Araschnia levana|EULEP1964-15|RVcoll.14-G812||Romania|658[0n]
Araschnia levana|EULEP2617-15|RVcoll.14-N557||Greece|658[0n]
Araschnia levana|GBLAD099-14|BC ZSM Lep 78670||Germany|658[0n]
Araschnia levana|GBLAD208-14|BC ZSM Lep 78779||Germany|658[0n]
Araschnia levana|GBLAB319-13|BC ZSM Lep 72620||Germany|658[0n]
Araschnia levana|GBLAA373-14|BC ZSM Lep 80369||Germany|658[0n]
Araschnia levana|LEATJ1222-16|TLMF Lep 19545||Hungary|658[0n]
Araschnia levana|LEFIJ4718-16|ZMBSU-02119||Belarus|658[0n]
Araschnia levana|OXB1210-15|Rvcoll_15-M635||France|658[0n]
Araschnia levana|OXB1379-15|Rvcoll_15-M743||France|658[0n]
Araschnia levana|EULEP4931-16|RVcoll16I004||Poland|634[0n]
Araschnia levana|EULEP4853-16|RVcoll16H780||Bulgaria|634[0n]
Araschnia levana|EULEP3447-16|RVcoll15Q123||Russia|622[0n]
Araschnia levana|EULEP3446-16|RVcoll15Q119||Russia|626[0n]
Araschnia levana|EZROM355-08|07-E609||Spain|605[1n]
Araschnia levana|EZSPC244-09|RVcoll.08-P061||Spain|646[0n]
Araschnia levana|EZSPC243-09|RVcoll.08-P060||Spain|646[0n]
Araschnia levana|LENOA1363-11|LN-BD1363||France|636[0n]
Araschnia levana|LENOA1362-11|LN-BD1362||France|658[0n]
Araschnia levana|ABOLD017-16|TLMF Lep 21095||Austria|658[0n]
Araschnia levana|ABOLD427-16|TLMF Lep 21579||Austria|658[0n]
Araschnia levana|PHLAB327-10|TLMF Lep 01127||Austria|658[0n]
Araschnia levana|LEATA080-13|TLMF Lep 09497||Austria|658[0n]
Polygonia egea|EULEP740-15|RVcoll.12-N854|Lesvos|Greece|658[0n]
Polygonia egea|EULEP1770-15|RVcoll.14-H492||Greece|658[0n]
Polygonia egea|EULEP3058-15|RVcoll.14-O191|Thassos|Greece|658[0n]
Polygonia egea|BIBSA1595-16|12-Q953||Italy|658[0n]
Polygonia egea|BCLEP098-17|LEP-SS-00549||Italy|658[0n]
Polygonia egea|EULEP1724-15|RVcoll.14-H230||Greece|622[0n]
Polygonia egea|WMB2100-13|RVcoll.12-R113|Sicily|Italy|658[0n]
Polygonia egea|WMB4693-14|RVcoll.LD-2818||Italy|658[0n]
Polygonia egea|EULEP1780-15|RVcoll.14-H583||Greece|658[0n]

Polygonia egea|EULEP1724-15|RVcoll.14-H250||Greece|622[0n]
Polygonia egea|WMB2100-13|RVcoll.12-R113|Sicily|Italy|658[0n]
Polygonia egea|WMB4693-14|RVcoll.LD-2818||Italy|658[0n]
Polygonia egea|EULEP1780-15|RVcoll.14-H583||Greece|658[0n]
Polygonia egea|EULEP1788-15|RVcoll.14-H644||Greece|658[0n]
Polygonia egea|EULEP1806-15|RVcoll.14-H753||Greece|658[0n]
Polygonia egea|BIBSA1267-15|LD-3424||Italy|658[0n]
Polygonia egea|BCLEP099-17|LEP-SS-00550||Italy|658[0n]
Polygonia egea|BIBSA616-15|LEP-SS-00236||Italy|658[0n]
Polygonia egea|BIBSA646-15|RVcoll. 15-A809||Italy|658[0n]
Polygonia egea|EULEP1353-15|RVcoll.14-F913||Greece|614[0n]
Polygonia egea|EULEP5164-17|RVcoll16F105||Italy|658[0n]
Polygonia egea|EULEP4500-16|RVcoll15C230||Italy|658[0n]
Polygonia egea|WMB2106-13|RVcoll.12-R124|Sicily|Italy|658[0n]
Polygonia egea|WMB1298-13|RVcoll.12-M594|Sicily|Italy|658[0n]
Polygonia egea|GWORZ029-10|BC ZSM Lep 30385||Italy|658[0n]
Polygonia c-album|OXB836-15|14-U799||Italy|658[1n]
Polygonia c-album|BIBSA1745-16|11-H343||Italy|658[0n]
Polygonia c-album|BIBSA484-15|LEP-SS-00104||Italy|658[0n]
Polygonia c-album|BCLEP096-17|LEP-SS-00547||Italy|658[0n]
Polygonia c-album|WMB2585-13|RVcoll.12-R108|Sicily|Italy|658[0n]
Polygonia c-album|WMB1956-13|RVcoll.11-I058|Sicily|Italy|658[0n]
Polygonia c-album|WMB2097-13|RVcoll.12-R107|Sicily|Italy|658[0n]
Polygonia c-album|EZSPN948-09|RVcoll.08-L938||Spain|658[0n]
Polygonia c-album|GWORZ030-10|BC ZSM Lep 30386||Italy|658[0n]
Polygonia c-album|OXB902-15|15-A546||Italy|658[0n]
Polygonia c-album|WMB1650-13|RVcoll.12-O993||France|658[0n]
Polygonia c-album|BIBSA1296-15|14-E298|Corsica|France|658[0n]
Polygonia c-album|BIBSA1096-15|15-L351||Italy|658[0n]
Polygonia c-album|BIBSA1074-15|15-I579||Italy|658[0n]
Polygonia c-album|BIBSA1220-15|15-L953||Italy|658[0n]
Polygonia c-album|BIBSA1204-15|15-M303||Italy|658[0n]
Polygonia c-album|EULEP1720-15|RVcoll.14-H209||Greece|658[0n]
Polygonia c-album|EULEP2138-15|RVcoll.14-J986||Switzerland|658[0n]
Polygonia c-album|EULEP1580-15|RVcoll.14-G606||Greece|658[0n]
Polygonia c-album|EULEP1402-15|RVcoll.14-G054||Greece|658[0n]
Polygonia c-album|EULEP935-15|RVcoll.14-C885||Bulgaria|658[0n]
Polygonia c-album|EULEP1067-15|RVcoll.14-E974||Serbia|658[0n]
Polygonia c-album|EULEP350-14|MM23835||Lithuania|658[0n]
Polygonia c-album|EULEP144-14|RVcoll.10-C427||Romania|658[0n]
Polygonia c-album|EULEP4989-16|RVcoll16I692||Poland|658[0n]
Polygonia c-album|EULEP4936-16|RVcoll16I035||Poland|658[0n]
Polygonia c-album|EULEP4810-16|RVcoll16H702||Belgium|658[0n]
Polygonia c-album|EULEP4499-16|RVcoll15P036||Ukraine|658[0n]
Polygonia c-album|EULEP4498-16|RVcoll15Q141||Russia|658[0n]
Polygonia c-album|EULEP4497-16|RVcoll15I717||Austria|658[0n]
Polygonia c-album|EULEP4496-16|RVcoll15I361||Austria|658[0n]
Polygonia c-album|EULEP4753-16|RVcoll16G916||Sweden|658[0n]
Polygonia c-album|LOWA786-06|2005-LOWA-786||Russia|658[0n]
Polygonia c-album|LOWA785-06|2005-LOWA-785||Russia|658[0n]
Polygonia c-album|LOWA164-06|2005-LOWA-164||Russia|658[0n]
Polygonia c-album|WMB5318-14|RVcoll.14-I921|Sardinia|Italy|658[0n]
Polygonia c-album|WMB5143-14|RVcoll.10-C530||Italy|658[0n]
Polygonia c-album|WMB5054-14|RVcoll. 14-L185||Italy|658[0n]
Polygonia c-album|WMB5363-14|RVcoll.14-J732||France|658[0n]
Polygonia c-album|WMB4886-14|RVcoll.14-I433||Italy|658[0n]
Polygonia c-album|WMB605-11|RVcoll.11-I004|Sicily|Italy|658[0n]
Polygonia c-album|WMB4299-14|RVcoll.14-A396||Italy|658[0n]
Polygonia c-album|WMB4268-14|RVcoll.14-A169||Italy|658[0n]
Polygonia c-album|WMB4590-14|RVcoll.LD-231|Corsica|France|658[0n]
Polygonia c-album|WMB4537-14|RVcoll.LD-2550||Italy|658[0n]
Polygonia c-album|BIBSA1022-15|15-C166||Italy|658[0n]
Polygonia c-album|GBLAB328-13|BC ZSM Lep 72629||Germany|658[0n]
Polygonia c-album|WMB4171-14|RVcoll.13-S743||Italy|658[0n]
Polygonia c-album|WMB2396-13|RVcoll.09-X835||Italy|658[0n]
Polygonia c-album|BIBSA462-15|RVcoll.14-I130||Italy|658[0n]
Polygonia c-album|BIBSA262-15|RVcoll.14-E059||Italy|658[0n]
Polygonia c-album|WMB2262-13|RVcoll.12-R423||Italy|658[0n]
Polygonia c-album|WMB2187-13|RVcoll.07-E192||Italy|658[0n]
Polygonia c-album|WMB2049-13|RVcoll.12-Q784||Italy|658[0n]
Polygonia c-album|WMB1873-13|RVcoll.11-J619||Italy|658[0n]
Polygonia c-album|WMB1595-13|RVcoll.12-O612||France|658[0n]
Polygonia c-album|WMB1587-13|RVcoll.12-O574|Sardinia|Italy|658[0n]
Polygonia c-album|WMB1586-13|RVcoll.12-O573|Sardinia|Italy|658[0n]
Polygonia c-album|WMB1769-13|RVcoll.12-Q036||France|658[0n]
Polygonia c-album|WMB3488-14|RVcoll.10-B682||France|658[0n]
Polygonia c-album|WMB3477-14|RVcoll.10-A874||Spain|658[0n]
Polygonia c-album|WMB3227-14|RVcoll.08-J833||Spain|658[0n]
Polygonia c-album|WMB2931-14|RVcoll.11-J742|Sicily|Italy|658[0n]
Polygonia c-album|WMB3373-14|RVcoll.09-V200||France|658[0n]
Polygonia c-album|EZSPC1089-10|RVcoll.08-L857||Spain|658[0n]
Polygonia c-album|EZSPC1054-10|RVcoll.08-J817||Spain|658[0n]
Polygonia c-album|EZSPM259-09|RVcoll.08-R473||Spain|658[0n]
Polygonia c-album|EZSPM147-09|RVcoll.08-P660||Spain|658[0n]
Polygonia c-album|EZSPN589-09|RVcoll.08-J152||Portugal|658[0n]
Polygonia c-album|EZROM750-08|RV-06-G442||Spain|658[0n]
Polygonia c-album|EZSPC678-09|RVcoll.08-P336||Spain|658[0n]
Polygonia c-album|EZSPC677-09|RVcoll.08-P078||Spain|658[0n]
Polygonia c-album|EZSPC668-09|RVcoll.08-L494||Spain|658[0n]
Polygonia c-album|EZSPC661-09|RVcoll.08-H209||Spain|658[0n]
Polygonia c-album|EZROM327-08|RV-08-A005||Romania|658[0n]
Polygonia c-album|EZROM493-08|RV-06-M874||Romania|658[0n]
Polygonia c-album|EZROM492-08|RV-06-K608||Romania|658[0n]
Polygonia c-album|GWOSU1045-11|BC Back 0095||France|658[0n]
Polygonia c-album|GWOSU1044-11|BC Back 0094||Germany|658[0n]
Polygonia c-album|FBLMU451-09|BC ZSM Lep 27101||Germany|658[0n]
Polygonia c-album|LENOA1353-11|LN-BD1353||France|658[0n]
Polygonia c-album|LEFIB082-10|MM00384||Finland|658[0n]
Polygonia c-album|ABOLB004-15|TLMF Lep 17009||Austria|658[0n]
Polygonia c-album|LEATG474-14|TLMF Lep 14261||Italy|658[0n]
Polygonia c-album|ABOLA498-14|TLMF Lep 16185||Austria|658[0n]
Polygonia c-album|EZROM494-08|RV-07-D905||Romania|658[0n]
Polygonia c-album|EZRMN205-08|RVcoll.08-M441||Romania|658[0n]

Polygonia c-album|ABOLA498-14|TLMF Lep 16185||Austria|658[0n]
Polygonia c-album|EZROM494-08|RV-07-D905||Romania|658[0n]
Polygonia c-album|EZRMN205-08|RVcoll.08-M441||Romania|658[0n]
Polygonia c-album|EZRMN204-08|RVcoll.07-D936||Romania|658[0n]
Polygonia c-album|EZRMN203-08|RVcoll.07-D564||Romania|658[0n]
Polygonia c-album|LEATG021-14|TLMF Lep 13808||Austria|658[0n]
Polygonia c-album|ABOLB005-15|TLMF Lep 17010||Austria|658[0n]
Polygonia c-album|PHLAA441-09|TLMF Lep 00481||France|658[0n]
Polygonia c-album|ABOLD417-16|TLMF Lep 21569||Austria|658[0n]
Polygonia c-album|BIBSA1357-15|15-M860||Italy|658[0n]
Polygonia c-album|BCLEP097-17|LEP-SS-00548||Italy|658[0n]
Polygonia c-album|BIBSA764-15|LEP-SS-00275||Italy|658[0n]
Polygonia c-album|BIBSA483-15|LEP-SS-00103||Italy|658[0n]
Polygonia c-album|BIBSA453-15|RVcoll.14-I121||Italy|658[0n]
Polygonia c-album|BIBSA1619-16|LEP-SS-00391||Italy|658[0n]
Polygonia c-album|WMB3870-14|RVcoll.11-Y027||Italy|658[0n]
Polygonia c-album|WMB4245-14|RVcoll.13-U019||Italy|658[0n]
Polygonia c-album|GBLAC962-13|BC ZSM Lep 76493||Germany|658[0n]
Polygonia c-album|BIBSA424-15|RVcoll.14-I092||Italy|658[0n]
Polygonia c-album|EULEP343-14|MM23828||Lithuania|658[0n]
Polygonia c-album|EULEP314-14|MM23799||Latvia|658[0n]
Polygonia c-album|EULEP835-15|RVcoll.14-C008||Sweden|658[0n]
Polygonia c-album|EULEP1283-15|RVcoll.14-F695||Greece|658[0n]
Polygonia c-album|GBLAA1291-15|BC ZSM Lep 87177||Germany|658[0n]
Polygonia c-album|OXB779-15|14-A742||Italy|658[0n]
Polygonia c-album|OXB597-15|14-N986||Italy|658[0n]
Polygonia c-album|OXB1521-16|OXB-TGS-1239||United Kingdom|658[0n]
Polygonia c-album|OXB604-15|14-N993||Italy|658[0n]
Polygonia c-album|OXB345-15|RVcoll.14-N059||Italy|658[0n]
Polygonia c-album|OXB1013-15|15-A963||Italy|658[0n]
Polygonia c-album|OXB277-15|OXB-TGS-960||United Kingdom|658[0n]
Polygonia c-album|OXB1290-15|Rvcoll_15-M153||France|658[0n]
Polygonia c-album|OXB1318-15|Rvcoll_15-M584||France|658[0n]
Polygonia c-album|OXB1377-15|Rvcoll_15-M741||France|658[0n]
Polygonia c-album|GBLAA1258-15|BC ZSM Lep 87144||Germany|658[1n]
Polygonia c-album|LOWA863-06|2005-LOWA-863||Russia|658[0n]
Polygonia c-album|PHLAW015-13|TLMF Lep 09812||Austria|650[0n]
Polygonia c-album|EZSPN993-09|RVcoll.08-M062||Spain|632[0n]
Polygonia c-album|LEFIB083-10|MM00385||Finland|633[0n]
Polygonia c-album|EULEP4495-16|RVcoll10A756||Estonia|622[0n]
Polygonia c-album|LEFIJ536-10|MM17161||Finland|658[0n]
Polygonia c-album|LEATH713-14|TLMF Lep 15925||Italy|634[0n]
Polygonia c-album|LEATG018-14|TLMF Lep 13805||Austria|658[0n]
Nymphalis vaualbum|EULEP3012-15|RVcoll.14-O145||Serbia|658[0n]
Nymphalis vaualbum|EULEP3013-15|RVcoll.14-O146||Serbia|658[0n]
Nymphalis vaualbum|EULEP4331-16|RVcoll14E330||Serbia|658[0n]
Nymphalis vaualbum|EZRMN419-09|RVcoll.07-D628||Romania|658[0n]
Nymphalis polychloros|WMB2415-13|RVcoll.10-C634|Sicily|Italy|658[0n]
Nymphalis polychloros|EZSPN631-09|RVcoll.08-J730||Spain|658[0n]
Nymphalis polychloros|EZSPM735-12|Rvcoll. 12-L621||Spain|658[1n]
Nymphalis polychloros|OXB1014-15|15-A964||Italy|658[0n]
Nymphalis polychloros|BIBSA1719-16|LD-0240||Italy|658[0n]
Nymphalis polychloros|WMB5319-14|RVcoll.14-I954|Sardinia|Italy|658[0n]
Nymphalis polychloros|WMB5332-14|RVcoll.14-J194|Corsica|France|658[0n]
Nymphalis polychloros|WMB305-11|RVcoll.11-E349||Italy|658[0n]
Nymphalis polychloros|WMB4650-14|RVcoll.LD-2469|Sardinia|Italy|658[0n]
Nymphalis polychloros|WMB1580-13|RVcoll.12-O535|Sardinia|Italy|658[0n]
Nymphalis polychloros|WMB1122-13|RVcoll.11-D222|Sicily|Italy|658[0n]
Nymphalis polychloros|EZSPN345-09|RVcoll.08-H310||Spain|658[1n]
Nymphalis polychloros|EULEP308-14|MM23793||Latvia|614[0n]
Nymphalis polychloros|WMB151-11|RVcoll.11-D270|Sicily|Italy|658[0n]
Nymphalis polychloros|EZSPM736-12|Rvcoll. 12-L622||Spain|630[0n]
Nymphalis polychloros|LEATG237-14|TLMF Lep 14024||Austria|658[0n]
Nymphalis polychloros|LEATG447-14|TLMF Lep 14234||Italy|658[0n]
Nymphalis polychloros|ABOLD042-16|TLMF Lep 21120||Austria|658[0n]
Nymphalis polychloros|LEATG238-14|TLMF Lep 14025||Austria|658[0n]
Nymphalis polychloros|GWORU359-10|BC ZSM Lep 31950||Italy|658[0n]
Nymphalis polychloros|LENOA1360-11|LN-BD1360||France|658[0n]
Nymphalis polychloros|FBLMX201-11|BC ZSM Lep 50412||Germany|658[0n]
Nymphalis polychloros|GWORA2506-09|BC ZSM Lep 30718||Germany|658[0n]
Nymphalis polychloros|GWORA2507-09|BC ZSM Lep 30719||Germany|658[0n]
Nymphalis polychloros|GWOR3922-09|BC ZSM Lep 21206||Germany|658[0n]
Nymphalis polychloros|LEFID220-10|MM06077||Estonia|658[0n]
Nymphalis polychloros|LEFIJ315-10|MM15915||Finland|658[0n]
Nymphalis polychloros|EZRMN146-08|RVcoll.08-M477||Romania|658[0n]
Nymphalis polychloros|EZROM285-08|RV-07-E647||Romania|658[0n]
Nymphalis polychloros|EZROM302-08|RV-07-E664||Romania|658[0n]
Nymphalis polychloros|EZROM303-08|RV-07-E665||Romania|658[0n]
Nymphalis polychloros|EZROM314-08|RV-07-E676||Romania|658[0n]
Nymphalis polychloros|EZROM352-08|RV-08-A030||Romania|658[0n]
Nymphalis polychloros|EZROM353-08|RV-08-A031||Romania|658[0n]
Nymphalis polychloros|EZSPN698-09|RVcoll.08-L040||Spain|658[0n]
Nymphalis polychloros|EZSPN032-09|RVcoll.09-T067||Spain|658[0n]
Nymphalis polychloros|EZSPN423-09|RVcoll.08-H579||Spain|658[0n]
Nymphalis polychloros|EZSPC571-09|RVcoll.07-C007||Spain|658[0n]
Nymphalis polychloros|EZSPC572-09|RVcoll.08-H223||Spain|658[0n]
Nymphalis polychloros|EZSPC573-09|RVcoll.08-H228||Spain|658[0n]
Nymphalis polychloros|EZSPM737-12|Rvcoll. 12-L623||Spain|658[0n]
Nymphalis polychloros|EZROM743-08|RV-06-H867||Spain|658[0n]
Nymphalis polychloros|EZSPC1320-10|RVcoll.130209KL80||Spain|658[0n]
Nymphalis polychloros|WMB3540-14|RVcoll.11-D645||Spain|658[0n]
Nymphalis polychloros|WMB4548-14|RVcoll.040611HZ98||Spain|658[0n]
Nymphalis polychloros|WMB4588-14|RVcoll.280111MJ47||Spain|658[0n]
Nymphalis polychloros|WMB601-11|RVcoll.11-H972|Sicily|Italy|658[0n]
Nymphalis polychloros|WMB630-11|RVcoll.11-I113||Italy|658[0n]
Nymphalis polychloros|WMB4794-14|RVcoll. 14-I341||Italy|658[0n]
Nymphalis polychloros|WMB5128-14|RVcoll. 14-L259||Italy|658[0n]
Nymphalis polychloros|EULEP4330-16|RVcoll14V322||Italy|658[0n]
Nymphalis polychloros|EULEP1130-15|RVcoll.14-F258||Serbia|658[0n]
Nymphalis polychloros|EULEP1327-15|RVcoll.14-F802||Greece|658[0n]
Nymphalis polychloros|EULEP1907-15|RVcoll.14-B804||Bosnia and Herzegovina|658[0n]
Nymphalis polychloros|BIBSA858-15|LD-3470||Italy|658[0n]
Nymphalis polychloros|BIBSA1197-15|15-M289||Italy|658[0n]

Nymphalis polychloros|EULEP1907-15|RVcoll.14-B804||Bosnia and Herzegovina|658[0n]
Nymphalis polychloros|BIBSA858-15|LD-3470||Italy|658[0n]
Nymphalis polychloros|BIBSA1197-15|15-M289||Italy|658[0n]
Nymphalis polychloros|BCLEP100-17|LEP-SS-00551||Italy|658[0n]
Nymphalis polychloros|GBLAC996-13|BC ZSM Lep 76527||Germany|658[0n]
Nymphalis polychloros|GWORA2877-15|BC ZSM Lep 86145||Germany|658[0n]
Nymphalis polychloros|LEASS892-17|KLM Lep 08397||Austria|658[0n]
Nymphalis polychloros|LEASS847-17|TLMF Lep 22495||Austria|658[0n]
Nymphalis polychloros|OXB607-15|14-N996||Italy|658[0n]
Nymphalis polychloros|OXB306-15|RVcoll.14-N020||Italy|658[0n]
Nymphalis polychloros|OXB1153-15|Rvcoll_15-N116||Italy|658[0n]
Nymphalis polychloros|GWORZ031-10|BC ZSM Lep 30387||Italy|658[1n]
Nymphalis polychloros|WMB819-13|RVcoll.07-E182||Italy|633[0n]
Nymphalis polychloros|GWOTL177-13|BC ZSM Lep 67108||Germany|642[0n]
Nymphalis polychloros|EULEP4814-16|RVcoll16H709||Belgium|658[0n]
Nymphalis polychloros|EZSPC725-10|RVcoll.08-H553||Spain|640[0n]
Nymphalis polychloros|LEATG446-14|TLMF Lep 14233||Italy|624[0n]
Nymphalis xanthomelas|LEFIJ314-10|MM15914||Finland|651[0n]
Nymphalis xanthomelas|EULEP563-15|RVcoll.09-V359||Romania|658[0n]
Nymphalis xanthomelas|EULEP829-15|RVcoll.14-B925||Sweden|658[0n]
Nymphalis xanthomelas|EULEP834-15|RVcoll.14-B996||Sweden|658[0n]
Nymphalis xanthomelas|EULEP2335-15|RVcoll.14-V020||Ukraine|658[0n]
Nymphalis xanthomelas|EULEP3014-15|RVcoll.14-O147||Macedonia|658[0n]
Nymphalis xanthomelas|EZRMN147-08|RVcoll.08-M670||Romania|647[0n]
Nymphalis xanthomelas|EZROM299-08|RV-07-E661||Romania|655[0n]
Nymphalis xanthomelas|EULEP356-14|MM23841||Lithuania|658[0n]
Nymphalis xanthomelas|EULEP339-14|MM23824||Estonia|658[0n]
Nymphalis xanthomelas|EULEP309-14|MM23794||Latvia|658[0n]
Nymphalis xanthomelas|EZROM313-08|RV-07-E675||Romania|658[0n]
Nymphalis xanthomelas|EZROM284-08|RV-07-E646||Romania|658[0n]
Nymphalis xanthomelas|EZROM442-08|RV-07-C961|ex. pupa|Romania|658[0n]
Nymphalis xanthomelas|LEFIJ313-10|MM15913||Finland|658[0n]
Nymphalis xanthomelas|LEFIJ1026-11|MM14865||Finland|658[0n]
Nymphalis antiopa|EULEP983-15|RVcoll.14-D141||Austria|658[0n]
Nymphalis antiopa|WMB4587-14|RVcoll.190511WX22||Spain|630[0n]
Nymphalis antiopa|LEATG019-14|TLMF Lep 13806||Austria|628[0n]
Nymphalis antiopa|LEATG020-14|TLMF Lep 13807||Austria|658[0n]
Nymphalis antiopa|EULEP200-14|RVcoll.12-L011||Spain|658[0n]
Nymphalis antiopa|EZSPM799-12|Rvcoll. 090111XZ43||Spain|658[0n]
Nymphalis antiopa|EZSPM798-12|Rvcoll. 090111XZ40||Spain|658[0n]
Nymphalis antiopa|EZSPM800-12|Rvcoll. 090111XZ44||Spain|658[0n]
Nymphalis antiopa|EZSPM732-12|Rvcoll. 08-J461||Spain|658[1n]
Nymphalis antiopa|EULEP4328-16|RVcoll14W587||Italy|637[0n]
Nymphalis antiopa|EULEP4978-16|RVcoll16I518||Poland|637[0n]
Nymphalis antiopa|ABOLD582-17|TLMF Lep 21658||Austria|658[0n]
Nymphalis antiopa|BCLEP119-17|LEP-SS-00570||Italy|658[0n]
Nymphalis antiopa|EULEP1481-15|RVcoll.14-G279||Greece|658[0n]
Nymphalis antiopa|EULEP840-15|RVcoll.14-C020||Sweden|658[0n]
Nymphalis antiopa|EULEP4999-16|RVcoll16I784||Germany|658[0n]
Nymphalis antiopa|EULEP4856-16|RVcoll16H829||Poland|658[0n]
Nymphalis antiopa|EULEP015-14|RVcoll.07-C393||Romania|658[0n]
Nymphalis antiopa|EULEP4329-16|RVcoll15Q044||Austria|658[0n]
Nymphalis antiopa|WMB5125-14|RVcoll. 14-L256||Italy|658[0n]
Nymphalis antiopa|EZROM742-08|RV-07-C000||Spain|658[0n]
Nymphalis antiopa|EZSPM733-12|Rvcoll. 08-J462||Spain|658[0n]
Nymphalis antiopa|EZSPC570-09|RVcoll.08-H227||Spain|658[0n]
Nymphalis antiopa|EZRMN145-08|RVcoll.08-M669||Romania|658[0n]
Nymphalis antiopa|EZRMN144-08|RVcoll.08-M663||Romania|658[0n]
Nymphalis antiopa|EZSPC569-09|RVcoll.08-H226||Spain|658[0n]
Nymphalis antiopa|EZSPC568-09|RVcoll.08-H219||Spain|658[0n]
Nymphalis antiopa|EZSPC567-09|RVcoll.08-H218||Spain|658[0n]
Nymphalis antiopa|EZROM441-08|RV-06-M842||Romania|658[0n]
Nymphalis antiopa|EZRMN423-09|RVcoll.09-V660||Romania|658[0n]
Nymphalis antiopa|LON949-12|NHMO Lep2011.009||Norway|658[0n]
Nymphalis antiopa|LEFIJ531-10|MM17156||Finland|658[0n]
Nymphalis antiopa|LEFIJ530-10|MM17155||Finland|658[0n]
Nymphalis antiopa|GWORA2509-09|BC ZSM Lep 30721||Germany|658[0n]
Nymphalis antiopa|GWORA2508-09|BC ZSM Lep 30720||Germany|658[0n]
Nymphalis antiopa|LEATG448-14|TLMF Lep 14235||Italy|658[0n]
Nymphalis antiopa|LEATA048-13|TLMF Lep 09465||Italy|658[0n]
Aglais ichnusa|WMB4744-14|RVcoll.13-T229|Corsica|France|658[0n]
Aglais ichnusa|WMB4743-14|RVcoll.13-T223|Corsica|France|658[0n]
Aglais ichnusa|WMB4742-14|RVcoll.13-T222|Corsica|France|658[0n]
Aglais ichnusa|WMB2452-13|RVcoll.11-E500.1|Sardinia|Italy|658[0n]
Aglais ichnusa|WMB679-12|RVcoll.11-E480|Sardinia|Italy|658[0n]
Aglais ichnusa|WMB318-11|RVcoll.11-E522|Sardinia|Italy|658[0n]
Aglais ichnusa|WMB1578-13|RVcoll.12-O526|Sardinia|Italy|658[0n]
Aglais ichnusa|WMB1577-13|RVcoll.12-O525|Sardinia|Italy|658[0n]
Aglais urticae|EULEP1418-15|RVcoll.14-G088||Greece|658[0n]
Aglais urticae|EZSPC750-10|RVcoll.08-L143|Genit. examined|Spain|658[0n]
Aglais urticae|EZSPM654-12|RVcoll.11-I474||Spain|658[0n]
Aglais urticae|EZSPM653-12|RVcoll.11-I473||Spain|658[0n]
Aglais urticae|EZSPN727-09|RVcoll.08-L134|Genit. examined|Spain|658[0n]
Aglais urticae|EZSPN098-09|RVcoll.06-A037|Genit. examined|Spain|658[0n]
Aglais urticae|EZSPC958-10|RVcoll.100608DS47||Spain|658[0n]
Aglais urticae|WMB1959-13|RVcoll.11-I082|Sicily|Italy|656[0n]
Aglais urticae|WMB609-11|RVcoll.11-I009|Sicily|Italy|658[0n]
Aglais urticae|WMB561-11|RVcoll.11-H755|Sicily|Italy|658[0n]
Aglais urticae|WMB719-12|RVcoll.11-I095|Sicily|Italy|658[0n]
Aglais urticae|WMB718-12|RVcoll.11-I085|Sicily|Italy|658[0n]
Aglais urticae|WMB4715-14|RVcoll.LD-2956|Sicily|Italy|658[0n]
Aglais urticae|WMB4536-14|RVcoll.LD-2535||Italy|658[0n]
Aglais urticae|WMB1953-13|RVcoll.11-I010|Sicily|Italy|658[0n]
Aglais urticae|EZSPM822-12|Rvcoll. 140410KV47||Spain|658[0n]
Aglais urticae|EZSPM652-12|RVcoll.11-I472||Spain|658[0n]
Aglais urticae|EZSPM651-12|RVcoll.11-I470||Spain|658[0n]
Aglais urticae|EZSPC1309-10|RVcoll.130209KL69||Spain|658[0n]
Aglais urticae|EZSPM615-12|RVcoll.11-D824||Spain|658[0n]
Aglais urticae|EZSPN091-09|RVcoll.06-A020|Genit. examined|Spain|635[0n]
Aglais urticae|EULEP4768-16|RVcoll16H162||Norway|658[0n]
Aglais urticae|EULEP4975-16|RVcoll16I467||Poland|658[0n]
Aglais urticae|WMB874-13|RVcoll.09-X249||France|658[0n]
[illegible]

Aglais urticae|EULEP4768-16|RVcoll16H162||Norway|658[0n]
Aglais urticae|EULEP4975-16|RVcoll16I467||Poland|658[0n]
Aglais urticae|WMB874-13|RVcoll.09-X249||France|658[0n]
Aglais urticae|OXB744-15|13-U317||Italy|658[0n]
Aglais urticae|EZROM754-08|RVcoll.08-M491|Genit. examined|Romania|658[0n]
Aglais urticae|OXB1343-15|Rvcoll_15-M691||France|658[0n]
Aglais urticae|LEFIJ4722-16|ZMBSU-02165||Belarus|658[0n]
Aglais urticae|GBLAA1057-15|BC ZSM Lep 86563||Germany|658[0n]
Aglais urticae|GBLAB329-13|BC ZSM Lep 72630||Germany|658[0n]
Aglais urticae|EULEP905-15|RVcoll.14-C746||Bulgaria|658[0n]
Aglais urticae|EULEP1148-15|RVcoll.14-F323||Serbia|658[0n]
Aglais urticae|EULEP313-14|MM23798||Latvia|658[0n]
Aglais urticae|EULEP134-14|RVcoll.10-C378||Romania|658[0n]
Aglais urticae|EULEP5013-16|RVcoll16I898||Slovakia|658[0n]
Aglais urticae|EULEP4954-16|RVcoll16I208||Germany|658[0n]
Aglais urticae|EULEP4804-16|RVcoll16H690||Norway|658[0n]
Aglais urticae|EULEP3388-16|RVcoll15I021||Italy|658[0n]
Aglais urticae|EZROM003-08|RV-07-C204|Genit. examined|Romania|658[0n]
Aglais urticae|LEFIJ529-10|MM17154||Finland|658[0n]
Aglais urticae|LEFIB079-10|MM00381||Finland|634[0n]
Aglais urticae|EZSPM260-09|RVcoll.08-R474|Genit. examined|Spain|658[0n]
Aglais urticae|OXB613-15|14-O002||Italy|658[2n]
Aglais urticae|EZSPC077-09|RVcoll.07-C631|Genit. examined|Spain|658[0n]
Aglais urticae|WMB4346-14|RVcoll.14-A847||United Kingdom|658[0n]
Aglais urticae|EULEP1062-15|RVcoll.14-E964||Serbia|613[0n]
Aglais urticae|EZROM001-08|RV-06-M855||Romania|658[0n]
Aglais urticae|EZROM597-08|RV-07-D352||Romania|658[0n]
Aglais urticae|EZROM283-08|RV-07-E645||Romania|658[0n]
Aglais urticae|EZROM002-08|RV-07-D548||Romania|658[0n]
Aglais urticae|EZSPC076-09|RVcoll.08-P217||Spain|657[0n]
Aglais urticae|EZSPC079-09|RVcoll.08-L484||Spain|658[0n]
Aglais urticae|EZSPC092-09|RVcoll.07-E603.1||Spain|658[0n]
Aglais urticae|EZSPN184-09|RVcoll.08-J882||Spain|658[0n]
Aglais urticae|EZSPN835-09|RVcoll.08-L657|Genit. examined|Spain|658[0n]
Aglais urticae|EZSPN360-09|RVcoll.08-H339|Genit. examined|Spain|658[0n]
Aglais urticae|EZSPC078-09|RVcoll.08-R180||Spain|658[0n]
Aglais urticae|EZSPM806-12|Rvcoll. 090611SZ73||Spain|658[0n]
Aglais urticae|EZSPM820-12|Rvcoll. 140410KV21||Spain|658[0n]
Aglais urticae|EZSPN967-09|RVcoll.08-L981|Genit. examined|Spain|658[0n]
Aglais urticae|EZSPC964-10|RVcoll.190608FT45||Spain|658[0n]
Aglais urticae|EZSPM139-09|RVcoll.08-P651|Genit. examined|Spain|658[0n]
Aglais urticae|EZSPM761-12|Rvcoll. 030111FB20||Spain|658[0n]
Aglais urticae|EZSPM783-12|Rvcoll. 070211PL18||Spain|658[0n]
Aglais urticae|EZSPM784-12|Rvcoll. 070211PL40||Spain|658[0n]
Aglais urticae|EZSPC769-10|RVcoll.08-L703|Genit. examined|Spain|658[0n]
Aglais urticae|EZSPC784-10|RVcoll.08-L982|Genit. examined|Spain|658[0n]
Aglais urticae|WMB4749-14|RVcoll.12-Q814.1||Italy|658[0n]
Aglais urticae|WMB2023-13|RVcoll.12-Q692||Italy|658[0n]
Aglais urticae|WMB2223-13|RVcoll.11-Y051||Italy|658[0n]
Aglais urticae|WMB4243-14|RVcoll.13-U011||Italy|658[0n]
Aglais urticae|WMB737-12|RVcoll.11-I985||France|658[0n]
Aglais urticae|WMB4849-14|RVcoll.14-I396||Italy|658[0n]
Aglais urticae|WMB4992-14|RVcoll. 14-I539||Italy|658[0n]
Aglais urticae|WMB5210-14|RVcoll.13-T949||Italy|658[0n]
Aglais urticae|EULEP3385-16|RVcoll15G859||Switzerland|658[0n]
Aglais urticae|EULEP3386-16|RVcoll15H084||Italy|658[0n]
Aglais urticae|EULEP3387-16|RVcoll15H912||Italy|658[0n]
Aglais urticae|EULEP3389-16|RVcoll15I153||Austria|658[0n]
Aglais urticae|EULEP045-14|RVcoll.08-H029||Romania|658[0n]
Aglais urticae|EULEP1284-15|RVcoll.14-F697||Greece|658[0n]
Aglais urticae|EULEP616-15|RVcoll.11-H147||Germany|658[0n]
Aglais urticae|EULEP638-15|RVcoll.11-J106||Switzerland|658[0n]
Aglais urticae|EULEP858-15|RVcoll.14-C177||Greece|658[0n]
Aglais urticae|EULEP2120-15|RVcoll.14-J890||France|658[0n]
Aglais urticae|EULEP2150-15|RVcoll.14-K040||Switzerland|658[0n]
Aglais urticae|EULEP2417-15|RVcoll.14-V192||Belgium|658[0n]
Aglais urticae|EULEP2003-15|RVcoll.14-I782||Slovakia|658[0n]
Aglais urticae|BIBSA746-15|LEP-SS-00247||Italy|658[0n]
Aglais urticae|BIBSA1080-15|15-K543||Italy|658[0n]
Aglais urticae|BIBSA1368-15|15-M911||Italy|658[0n]
Aglais urticae|BIBSA690-15|RVcoll. 12-Q815||Italy|658[0n]
Aglais urticae|BIBSA499-15|LEP-SS-00119||Italy|658[0n]
Aglais urticae|BIBSA168-15|RVcoll.14-D956||Italy|658[0n]
Aglais urticae|BIBSA328-15|RVcoll.14-E133||Italy|658[0n]
Aglais urticae|BIBSA1826-17|RVcoll16C700||Italy|658[0n]
Aglais urticae|OXB337-15|RVcoll.14-N051||Italy|658[0n]
Aglais urticae|OXB232-15|OXB-TGS-505||United Kingdom|658[0n]
Aglais urticae|OXB263-15|OXB-TGS-946||United Kingdom|658[0n]
Aglais urticae|OXB1314-15|Rvcoll_15-M579||France|658[0n]
Aglais urticae|OXB1196-15|Rvcoll_15-M611||France|658[0n]
Aglais urticae|EULEP3390-16|RVcoll15I603||Austria|632[0n]
Aglais urticae|WMB4940-14|RVcoll.14-I487||Italy|639[0n]
Aglais urticae|GBLAA1413-15|BC ZSM Lep 87109||Germany|637[0n]
Aglais urticae|OXB208-15|OXB-TGS-190||United Kingdom|632[1n]
Aglais urticae|EULEP3393-16|RVcoll15Q177||Ukraine|637[0n]
Aglais urticae|EULEP3392-16|RVcoll15G307||France|619[0n]
Aglais urticae|EULEP3391-16|RVcoll15G181||France|618[0n]
Aglais urticae|LENOA1356-11|LN-BD1356||France|644[0n]
Aglais urticae|LEFIB080-10|MM00382||Finland|658[0n]
Aglais urticae|LEATG239-14|TLMF Lep 14026||Austria|658[0n]
Aglais urticae|LEATG441-14|TLMF Lep 14228||Italy|658[0n]
Aglais urticae|LEATA047-13|TLMF Lep 09464||Italy|658[0n]
Aglais urticae|ABOLD054-16|TLMF Lep 21132||Austria|658[0n]
Aglais urticae|PHLAH744-12|TLMF Lep 08563||Austria|658[0n]
Aglais urticae|LEATG001-14|TLMF Lep 13788||Austria|658[0n]
Aglais io|GBLAC959-13|BC ZSM Lep 76490||Germany|658[0n]
Aglais io|LEATJ1277-16|TLMF Lep 19600||Czech Republic|658[0n]
Aglais io|EULEP1237-15|RVcoll.14-F553||Greece|658[0n]
Aglais io|EZSPM572-12|RVcoll.08-H225||Spain|658[0n]
Aglais io|EZROM218-08|RV-06-K659||Romania|658[0n]
Aglais io|EZROM1026-08|RVcoll.08-M530||Romania|658[0n]
Aglais io|WMB1971-13|RVcoll.11-I200||Italy|658[0n]
Aglais io|EZSPN848-09|RVcoll.08-L692||Spain|658[0n]

Aglais io|EZROM1026-08|RVcoll.08-M530||Romania|658[0n]
Aglais io|WMB1971-13|RVcoll.11-I200||Italy|658[0n]
Aglais io|EZSPN848-09|RVcoll.08-L692||Spain|658[0n]
Aglais io|EZSPM574-12|RVcoll.08-L836||Spain|658[0n]
Aglais io|EZSPN911-09|RVcoll.08-L848||Spain|658[0n]
Aglais io|EZSPC447-09|RVcoll.08-R146||Spain|658[0n]
Aglais io|EZSPC1096-10|RVcoll.08-L935||Spain|658[0n]
Aglais io|EZSPC448-09|RVcoll.08-R287||Spain|658[0n]
Aglais io|OXB274-15|OXB-TGS-957||United Kingdom|658[0n]
Aglais io|LEFIJ527-10|MM17152||Finland|658[0n]
Aglais io|LEFIJ528-10|MM17153||Finland|658[0n]
Aglais io|EZROM1027-08|RVcoll.08-M640||Romania|658[0n]
Aglais io|EZROM217-08|RV-06-K655||Romania|658[0n]
Aglais io|EZSPC446-09|RVcoll.08-L515||Spain|658[0n]
Aglais io|EZSPN918-09|RVcoll.08-L862||Spain|658[0n]
Aglais io|EZSPN994-09|RVcoll.08-M063||Spain|658[0n]
Aglais io|EZSPM241-09|RVcoll.08-R448||Spain|658[0n]
Aglais io|WMB1508-13|RVcoll.12-O093|Corsica|France|658[0n]
Aglais io|WMB1519-13|RVcoll.12-O150|Corsica|France|658[0n]
Aglais io|WMB917-13|RVcoll.09-X926||Italy|658[0n]
Aglais io|WMB1592-13|RVcoll.12-O595|Sardinia|Italy|658[0n]
Aglais io|WMB4250-14|RVcoll.13-U063||Italy|658[0n]
Aglais io|WMB4653-14|RVcoll.LD-2501|Sardinia|Italy|658[0n]
Aglais io|WMB4727-14|RVcoll.LD-3107||Italy|658[0n]
Aglais io|WMB728-12|RVcoll.11-I641||France|658[0n]
Aglais io|WMB650-11|RVcoll.11-I199||Italy|658[0n]
Aglais io|WMB5329-14|RVcoll.14-J141|Corsica|France|658[0n]
Aglais io|WMB4932-14|RVcoll.14-I479||Italy|658[0n]
Aglais io|WMB4995-14|RVcoll. 14-I542||Italy|658[0n]
Aglais io|LOWA167-06|2005-LOWA-167||Russia|658[0n]
Aglais io|EULEP4097-16|RVcoll15P020||Belarus|658[0n]
Aglais io|LOWA783-06|2005-LOWA-783||Russia|658[0n]
Aglais io|EULEP3382-16|RVcoll10A759||Estonia|658[0n]
Aglais io|EULEP3383-16|RVcoll15I422||Austria|658[0n]
Aglais io|EULEP3384-16|RVcoll15I750||Austria|658[0n]
Aglais io|EULEP4731-16|RVcoll16G431||Sweden|658[0n]
Aglais io|EULEP5012-16|RVcoll16I895||Slovakia|658[0n]
Aglais io|EULEP315-14|MM23800||Latvia|658[0n]
Aglais io|EULEP859-15|RVcoll.14-C187||Greece|658[0n]
Aglais io|EULEP2416-15|RVcoll.14-V191||Belgium|658[0n]
Aglais io|EULEP2021-15|RVcoll.14-I855||Poland|658[0n]
Aglais io|EULEP2158-15|RVcoll.14-K070||Switzerland|658[0n]
Aglais io|BIBSA1216-15|15-M350||Italy|658[0n]
Aglais io|BIBSA1108-15|15-L809||Italy|658[0n]
Aglais io|BIBSA1289-15|13-U512|Sardinia|Italy|658[0n]
Aglais io|GBLAB330-13|BC ZSM Lep 72631||Germany|658[0n]
Aglais io|GBLAA1351-15|BC ZSM Lep 87047||Germany|658[0n]
Aglais io|LEFIJ4772-16|ZMBSU-02163||Belarus|658[0n]
Aglais io|LEASS476-17|TLMF Lep 22124||Austria|658[0n]
Aglais io|OXB734-15|13-U236||Italy|658[0n]
Aglais io|OXB1012-15|15-A962|Sardinia|Italy|658[0n]
Aglais io|OXB1543-16|OXB-TGS-1261||France|658[0n]
Aglais io|OXB252-15|OXB-TGS-816||United Kingdom|658[0n]
Aglais io|OXB257-15|OXB-TGS-834||United Kingdom|658[0n]
Aglais io|OXB301-15|RVcoll.14-N015|Sicily E|Italy|658[0n]
Aglais io|OXB302-15|RVcoll.14-N016|Sicily E|Italy|658[0n]
Aglais io|GBLAA1102-15|BC ZSM Lep 86608||Germany|658[0n]
Aglais io|OXB1323-15|Rvcoll_15-M594||France|658[0n]
Aglais io|OXB1337-15|Rvcoll_15-M682||France|658[0n]
Aglais io|EULEP842-15|RVcoll.14-C023||Sweden|658[0n]
Aglais io|OXB1342-15|Rvcoll_15-M690||France|658[0n]
Aglais io|OXB1416-15|Rvcoll_15-M794||France|658[0n]
Aglais io|EULEP4946-16|RVcoll16I163||Germany|637[0n]
Aglais io|EULEP1046-15|RVcoll.14-E914||Serbia|614[0n]
Aglais io|BIBSA487-15|LEP-SS-00107||Italy|627[0n]
Aglais io|EZROM1025-08|RVcoll.08-M493||Romania|658[0n]
Aglais io|EULEP4839-16|RVcoll16H745||Ukraine|632[0n]
Aglais io|EULEP4941-16|RVcoll16I092||Poland|635[0n]
Aglais io|WMB738-12|RVcoll.11-J161||France|632[0n]
Aglais io|EZSPM571-12|RVcoll.08-H222||Spain|634[0n]
Aglais io|EZROM330-08|RV-08-A008||Romania|609[0n]
Aglais io|LON197-08|NHMO-06205||Norway|608[0n]
Aglais io|LON053-08|NHMO-06053||Norway|657[0n]
Aglais io|FBLMU443-09|BC ZSM Lep 27093||Germany|658[0n]
Aglais io|LENOA1354-11|LN-BD1354||France|658[0n]
Aglais io|LEFIB081-10|MM00383||Finland|658[0n]
Aglais io|ABOLB008-15|TLMF Lep 17013||Austria|658[0n]
Aglais io|LEATI064-15|TLMF Lep 17449||Austria|658[0n]
Aglais io|PHLAW023-13|TLMF Lep 09820||Austria|658[0n]
Aglais io|ABOLA874-15|TLMF Lep 16834||Austria|658[0n]
Aglais io|ABOLD087-16|TLMF Lep 21165||Austria|658[0n]
Aglais io|LEATG440-14|TLMF Lep 14227||Italy|658[0n]
Aglais io|PHLAI556-13|TLMF Lep 09118||Italy|635[0n]
Vanessa atalanta|OXB1238-15|Rvcoll_13-T664|Sardinia|Italy|658[0n]
Vanessa atalanta|EULEP4935-16|RVcoll16I034||Poland|658[0n]
Vanessa atalanta|EZSPC351-09|RVcoll.08-P077||Spain|658[0n]
Vanessa atalanta|LEFIJ526-10|MM17151||Finland|658[0n]
Vanessa atalanta|WMB4334-14|RVcoll.14-A719||Italy|658[0n]
Vanessa atalanta|EULEP648-15|RVcoll.11-J149||Switzerland|658[0n]
Vanessa atalanta|WMB5470-14|RVcoll.14-N674|Salina|Italy|658[0n]
Vanessa atalanta|OXB875-15|15-A517||Italy|658[0n]
Vanessa atalanta|BIBSA1354-15|15-M855||Italy|658[0n]
Vanessa atalanta|EULEP1672-15|RVcoll.14-G912||Greece|658[0n]
Vanessa atalanta|EULEP4707-16|RVcoll15I653||Austria|658[0n]
Vanessa atalanta|WMB1112-13|RVcoll.11-D114|Pantelleria|Italy|658[0n]
Vanessa atalanta|EZSPM205-09|RVcoll.08-R323||Spain|658[0n]
Vanessa atalanta|EULEP1444-15|RVcoll.14-G144||Greece|658[0n]
Vanessa atalanta|WMB1997-13|RVcoll.12-Q347||France|658[0n]
Vanessa atalanta|EZROM596-08|RV-07-C360||Romania|658[0n]
Vanessa atalanta|EZROM560-08|RV-06-M897||Romania|658[0n]
Vanessa atalanta|LON022-08|NHMO-06022||Norway|657[0n]
Vanessa atalanta|WMB849-13|RVcoll.11-Y006|Elba|Italy|658[0n]
Vanessa atalanta|LON061-12|NHMO-Lep2011-021||Norway|658[0n]

Vanessa atalanta|[illegible]
Vanessa atalanta|LON022-08|NHMO-06022||Norway|657[0n]
Vanessa atalanta|WMB849-13|RVcoll.11-Y006|Elba|Italy|658[0n]
Vanessa atalanta|LON961-12|NHMO Lep2011.021||Norway|658[0n]
Vanessa atalanta|WMB1081-13|RVcoll.10-C717||Italy|658[0n]
Vanessa atalanta|OXB699-15|13-T871||Italy|658[0n]
Vanessa atalanta|EULEP5014-16|RVcoll16I905||Slovakia|658[0n]
Vanessa atalanta|WMB4247-14|RVcoll.13-U045||Italy|658[0n]
Vanessa atalanta|WMB1088-13|RVcoll.10-C752|Capraia|Italy|658[0n]
Vanessa atalanta|WMB3041-14|RVcoll.13-S571|San Domino|Italy|658[0n]
Vanessa atalanta|WMB001-11|RVcoll.11-D512||Italy|658[0n]
Vanessa atalanta|EZSPC350-09|RVcoll.08-R308||Spain|658[0n]
Vanessa atalanta|EZROM561-08|RV-07-D077||Romania|658[0n]
Vanessa atalanta|LEATG243-14|TLMF Lep 14030||Austria|658[0n]
Vanessa atalanta|WMB3049-14|RVcoll.13-S635|Capraia|Italy|658[0n]
Vanessa atalanta|WMB1688-13|RVcoll.12-P542||France|658[0n]
Vanessa atalanta|ABOLD420-16|TLMF Lep 21572||Austria|658[0n]
Vanessa atalanta|LOWA784-06|2005-LOWA-784||Russia|655[0n]
Vanessa atalanta|EZSPN448-09|RVcoll.08-H640||Spain|658[0n]
Vanessa atalanta|EULEP4706-16|RVcoll15I298||Austria|658[0n]
Vanessa atalanta|WMB475-11|RVcoll.11-H500|Capri|Italy|658[0n]
Vanessa atalanta|GWORU353-10|BC ZSM Lep 31944||Italy|658[0n]
Vanessa atalanta|EULEP310-14|MM23795||Latvia|658[0n]
Vanessa atalanta|LOWA163-06|2005-LOWA-163||Russia|658[0n]
Vanessa atalanta|FBLMX236-11|BC ZSM Lep 50447||Germany|658[0n]
Vanessa atalanta|GWORA2510-09|BC ZSM Lep 30722||Germany|658[0n]
Vanessa atalanta|GWORA2511-09|BC ZSM Lep 30723||Germany|658[0n]
Vanessa atalanta|EZRMN297-08|RVcoll.08-M395||Romania|658[0n]
Vanessa atalanta|EZRMN298-08|RVcoll.08-M423||Romania|658[0n]
Vanessa atalanta|EZRMN299-08|RVcoll.08-M443||Romania|658[0n]
Vanessa atalanta|EZROM562-08|RV-07-D226||Romania|658[0n]
Vanessa atalanta|EZROM328-08|RV-08-A006||Romania|658[0n]
Vanessa atalanta|EZSPN298-09|RVcoll.08-H101||Spain|658[0n]
Vanessa atalanta|EZSPN365-09|RVcoll.08-H389||Spain|658[0n]
Vanessa atalanta|EZSPN405-09|RVcoll.08-H491||Spain|658[0n]
Vanessa atalanta|EZSPN490-09|RVcoll.08-H927||Spain|658[0n]
Vanessa atalanta|EZSPM1004-12|Rvcoll.12-L890||Spain|658[0n]
Vanessa atalanta|EZSPN297-09|RVcoll.08-H100||Spain|658[0n]
Vanessa atalanta|EZSPM160-09|RVcoll.08-P687||Spain|658[0n]
Vanessa atalanta|EZSPC348-09|RVcoll.08-R278||Spain|658[0n]
Vanessa atalanta|EZSPC347-09|RVcoll.08-H295||Spain|658[0n]
Vanessa atalanta|EZSPC349-09|RVcoll.08-L489||Spain|658[0n]
Vanessa atalanta|WMB145-11|RVcoll.11-D185|Marettimo|Italy|658[0n]
Vanessa atalanta|WMB173-11|RVcoll.11-D433|Sicily|Italy|658[0n]
Vanessa atalanta|WMB130-11|RVcoll.11-D128|Pantelleria|Italy|658[0n]
Vanessa atalanta|WMB2884-14|RVcoll.10-C781|Argentario|Italy|658[0n]
Vanessa atalanta|WMB2950-14|RVcoll.12-M535.1|Capraia|Italy|658[0n]
Vanessa atalanta|WMB3008-14|RVcoll.12-P951.1|Levant|France|658[0n]
Vanessa atalanta|WMB3062-14|RVcoll.14-A792|Capri|Italy|658[0n]
Vanessa atalanta|WMB3186-14|RVcoll.08-J005||Spain|658[0n]
Vanessa atalanta|WMB3491-14|RVcoll.10-B686||France|658[0n]
Vanessa atalanta|WMB3706-14|RVcoll.12-L581||Portugal|658[0n]
Vanessa atalanta|WMB1121-13|RVcoll.11-D184|Marettimo|Italy|658[0n]
Vanessa atalanta|WMB1129-13|RVcoll.11-E104||Malta|658[0n]
Vanessa atalanta|WMB1131-13|RVcoll.11-E109|Gozo|Malta|658[0n]
Vanessa atalanta|WMB1152-13|RVcoll.11-E440|Sardinia|Italy|658[0n]
Vanessa atalanta|WMB1183-13|RVcoll.11-E981|Corsica|France|658[0n]
Vanessa atalanta|WMB1579-13|RVcoll.12-O530|Sardinia|Italy|658[0n]
Vanessa atalanta|WMB2070-13|RVcoll.12-Q913||Italy|658[0n]
Vanessa atalanta|WMB2093-13|RVcoll.12-R094|Sicily|Italy|658[0n]
Vanessa atalanta|WMB1702-13|RVcoll.12-P627||France|658[0n]
Vanessa atalanta|WMB2010-13|RVcoll.12-Q596|Sicily|Italy|658[0n]
Vanessa atalanta|WMB2299-13|RVcoll.11-E103||Malta|658[0n]
Vanessa atalanta|WMB2434-13|RVcoll.11-D135|Pantelleria|Italy|658[0n]
Vanessa atalanta|WMB2441-13|RVcoll.11-D189|Marettimo|Italy|658[0n]
Vanessa atalanta|WMB2568-13|RVcoll.12-Q900||Italy|658[0n]
Vanessa atalanta|WMB2639-13|RVcoll.11-E980|Corsica|France|658[0n]
Vanessa atalanta|WMB4594-14|RVcoll.LD-759|Giglio|Italy|658[0n]
Vanessa atalanta|WMB4305-14|RVcoll.14-A404||Italy|658[0n]
Vanessa atalanta|WMB4689-14|RVcoll.LD-2690|Giglio|Italy|658[0n]
Vanessa atalanta|WMB4700-14|RVcoll.LD-2894|Asinara|Italy|658[0n]
Vanessa atalanta|WMB4738-14|RVcoll.LD-3405|Montecristo|Italy|658[0n]
Vanessa atalanta|WMB303-11|RVcoll.11-E347||Italy|658[0n]
Vanessa atalanta|WMB369-11|RVcoll.11-E979||France|658[0n]
Vanessa atalanta|WMB5078-14|RVcoll. 14-L209||Italy|658[0n]
Vanessa atalanta|WMB4759-14|RVcoll. 14-I306||Italy|658[0n]
Vanessa atalanta|WMB4784-14|RVcoll. 14-I331||Italy|658[0n]
Vanessa atalanta|WMB4801-14|RVcoll. 14-I348||Italy|658[0n]
Vanessa atalanta|WMB4847-14|RVcoll. 14-I394||Italy|658[0n]
Vanessa atalanta|WMB5371-14|RVcoll.14-J768||France|658[0n]
Vanessa atalanta|WMB5005-14|RVcoll. 14-I552||Italy|658[0n]
Vanessa atalanta|WMB5320-14|RVcoll.14-I977|La Maddalena|Italy|658[0n]
Vanessa atalanta|WMB5465-14|RVcoll.14-N646|Vulcano|Italy|658[0n]
Vanessa atalanta|WMB5479-14|RVcoll.14-N704|Alicudi|Italy|658[0n]
Vanessa atalanta|WMB5505-14|RVcoll.14-J434|Montecristo|Italy|658[0n]
Vanessa atalanta|LOWA162-06|2005-LOWA-162||Russia|658[0n]
Vanessa atalanta|EULEP4705-16|RVcoll15G576||Switzerland|658[0n]
Vanessa atalanta|EULEP338-14|MM23823||Estonia|658[0n]
Vanessa atalanta|EULEP1071-15|RVcoll.14-E990||Serbia|658[0n]
Vanessa atalanta|EULEP847-15|RVcoll.14-C075||Sweden|658[0n]
Vanessa atalanta|EULEP940-15|RVcoll.14-C924||Bulgaria|658[0n]
Vanessa atalanta|EULEP2434-15|RVcoll.14-V233||Belgium|658[0n]
Vanessa atalanta|EULEP2004-15|RVcoll.14-I786||Slovakia|658[0n]
Vanessa atalanta|BIBSA1184-15|15-M232||Italy|658[0n]
Vanessa atalanta|BIBSA1257-15|15-F862||France|658[0n]
Vanessa atalanta|BIBSA1025-15|15-C170||Italy|658[0n]
Vanessa atalanta|BIBSA672-15|RVcoll. 12-Q788||Italy|658[0n]
Vanessa atalanta|BIBSA488-15|LEP-SS-00108||Italy|658[0n]
Vanessa atalanta|BIBSA639-15|RVcoll. 15-A802||Italy|658[0n]
Vanessa atalanta|BIBSA661-15|RVcoll. 15-A824||Italy|658[0n]
Vanessa atalanta|GBLAF585-14|BC ZSM Lep 82291||Germany|658[0n]
Vanessa atalanta|GBLAC961-13|BC ZSM Lep 76492||Germany|658[0n]
Vanessa atalanta|LEATJ1289-16|TLMF Lep 19612||Croatia|658[0n]

Vanessa atalanta|GBLAF585-14|BC ZSM Lep 82291||Germany|658[0n]
Vanessa atalanta|GBLAC961-13|BC ZSM Lep 76492||Germany|658[0n]
Vanessa atalanta|LEATJ1289-16|TLMF Lep 19612||Croatia|658[0n]
Vanessa atalanta|OXB1104-15|15-A637||Italy|658[0n]
Vanessa atalanta|OXB1522-16|OXB-TGS-1240||United Kingdom|658[0n]
Vanessa atalanta|OXB628-15|14-O017||Italy|658[0n]
Vanessa atalanta|OXB737-15|13-U246||Italy|658[0n]
Vanessa atalanta|OXB905-15|15-A549||Italy|658[0n]
Vanessa atalanta|OXB932-15|15-A576||Italy|658[0n]
Vanessa atalanta|OXB355-15|RVcoll.14-N069||Italy|658[0n]
Vanessa atalanta|OXB1050-15|15-A595||Italy|658[0n]
Vanessa atalanta|OXB207-15|OXB-TGS-189||United Kingdom|658[0n]
Vanessa atalanta|OXB251-15|OXB-TGS-815||United Kingdom|658[0n]
Vanessa atalanta|OXB1194-15|Rvcoll_15-M607||France|658[0n]
Vanessa atalanta|OXB1248-15|Rvcoll_13-T733|Sardinia|Italy|612[0n]
Vanessa atalanta|OXB1306-15|Rvcoll_15-M569||France|621[0n]
Vanessa atalanta|WMB4745-14|RVcoll.13-T268|Corsica|France|613[0n]
Vanessa atalanta|BIBSA1107-15|15-L807||Italy|623[0n]
Vanessa atalanta|BIBSA1795-16|16-A543||Italy|617[0n]
Vanessa atalanta|WMB2435-13|RVcoll.11-D136|Pantelleria|Italy|621[0n]
Vanessa atalanta|WMB3882-14|RVcoll.12-M517||Italy|658[0n]
Vanessa atalanta|WMB2398-13|RVcoll.09-X874|Sardinia|Italy|658[0n]
Vanessa atalanta|EZROM563-08|RV-07-D596||Romania|648[0n]
Vanessa atalanta|EZSPM997-12|Rvcoll.12-L883||Spain|658[0n]
Vanessa atalanta|LEFIB379-10|MM00953||Finland|632[0n]
Vanessa atalanta|LEFIG261-10|MM14193||Finland|658[0n]
Vanessa atalanta|GWORR423-10|BC ZSM Lep 29639||Italy|658[0n]
Vanessa atalanta|LEATG443-14|TLMF Lep 14230||Italy|658[0n]
Vanessa atalanta|PHLAW054-13|TLMF Lep 09851||Austria|658[0n]
Vanessa atalanta|LEATG244-14|TLMF Lep 14031||Austria|658[0n]
Vanessa virginiensis|EZSPN1089-11|Rvcoll.09-X679||Spain|658[0n]
Vanessa virginiensis|EZSPN462-09|RVcoll.08-H675||Spain|658[0n]
Vanessa cardui|WMB3042-14|RVcoll.13-S572|San Domino|Italy|658[0n]
Vanessa cardui|WMB3377-14|RVcoll.09-V265||France|658[0n]
Vanessa cardui|WMB4375-14|RVcoll.14-B160||Portugal|658[0n]
Vanessa cardui|WMB1511-13|RVcoll.12-O101|Corsica|France|658[0n]
Vanessa cardui|LEATG240-14|TLMF Lep 14027||Austria|658[0n]
Vanessa cardui|BIBSA1345-15|15-M839||Italy|658[0n]
Vanessa cardui|WMB5310-14|RVcoll.14-I683||France|658[0n]
Vanessa cardui|WMB3268-14|RVcoll.08-L872||Spain|658[0n]
Vanessa cardui|WMB3229-14|RVcoll.08-J872||Spain|658[0n]
Vanessa cardui|WMB3085-14|RVcoll.14-D952|Giglio|Italy|658[0n]
Vanessa cardui|EZSPM985-12|Rvcoll.12-M753||Spain|658[0n]
Vanessa cardui|OXB254-15|OXB-TGS-822||United Kingdom|658[0n]
Vanessa cardui|WMB5960-17|RVcoll14D501|Malta|Malta|658[0n]
Vanessa cardui|WMB825-13|RVcoll.07-E195||Italy|658[0n]
Vanessa cardui|LENOA1355-11|LN-BD1355||France|658[0n]
Vanessa cardui|BIBSA1642-16|15-C576||Italy|658[0n]
Vanessa cardui|OXB924-15|15-A568||Italy|658[0n]
Vanessa cardui|OXB1123-15|15-A656||Italy|658[0n]
Vanessa cardui|WMB4000-14|RVcoll.12-Q508||Spain|658[0n]
Vanessa cardui|FBLMU450-09|BC ZSM Lep 27100||Germany|658[0n]
Vanessa cardui|WMB3571-14|RVcoll.11-D988||Spain|658[0n]
Vanessa cardui|EULEP1591-15|RVcoll.14-G630||Greece|658[0n]
Vanessa cardui|WMB2723-13|RVcoll.10-C561||Italy|637[0n]
Vanessa cardui|WMB269-11|RVcoll.10-A469||France|658[0n]
Vanessa cardui|LEATG467-14|TLMF Lep 14254||Italy|619[1n]
Vanessa cardui|WMB1290-13|RVcoll.12-M534|Capraia|Italy|658[0n]
Vanessa cardui|WMB1728-13|RVcoll.12-P893||France|658[0n]
Vanessa cardui|WMB2180-13|RVcoll.12-R352|Elba|Italy|658[0n]
Vanessa cardui|WMB2199-13|RVcoll.09-X948|Ponza|Italy|658[0n]
Vanessa cardui|WMB5959-17|RVcoll14D500|Malta|Malta|658[0n]
Vanessa cardui|WMB2587-13|RVcoll.12-R130|Sicily|Italy|658[0n]
Vanessa cardui|WMB2640-13|RVcoll.12-R448|Corsica|France|658[0n]
Vanessa cardui|WMB4162-14|RVcoll.13-S719||Italy|658[0n]
Vanessa cardui|WMB4500-14|RVcoll.14-E177||Italy|658[0n]
Vanessa cardui|WMB4311-14|RVcoll.14-A441||Italy|658[0n]
Vanessa cardui|WMB4432-14|RVcoll.14-B525||Portugal|658[0n]
Vanessa cardui|WMB313-11|RVcoll.11-E479||Italy|658[0n]
Vanessa cardui|WMB5072-14|RVcoll. 14-L203||Italy|658[0n]
Vanessa cardui|WMB4756-14|RVcoll. 14-I303||Italy|658[0n]
Vanessa cardui|WMB5345-14|RVcoll.14-J365|Pianosa|Italy|658[0n]
Vanessa cardui|WMB4922-14|RVcoll.14-I469||Italy|658[0n]
Vanessa cardui|WMB5055-14|RVcoll. 14-L186||Italy|658[0n]
Vanessa cardui|WMB5216-14|RVcoll.13-T965||Italy|658[0n]
Vanessa cardui|WMB5467-14|RVcoll.14-N671|Salina|Italy|658[0n]
Vanessa cardui|EULEP4982-16|RVcoll16I588||Poland|658[0n]
Vanessa cardui|EULEP5695-17|RVcoll16L038|Elba|Italy|658[0n]
Vanessa cardui|EULEP707-15|RVcoll.12-M184|Crete|Greece|658[0n]
Vanessa cardui|EULEP1207-15|RVcoll.14-F503||Greece|658[0n]
Vanessa cardui|EULEP951-15|RVcoll.14-C960||Bulgaria|658[0n]
Vanessa cardui|BIBSA1114-15|15-L844||Italy|658[0n]
Vanessa cardui|BIBSA1203-15|15-M301||Italy|658[0n]
Vanessa cardui|BIBSA1587-16|12-M545||Italy|658[0n]
Vanessa cardui|BIBSA1753-16|12-Q599||Italy|658[0n]
Vanessa cardui|BIBSA670-15|RVcoll. 11-I192||Italy|658[0n]
Vanessa cardui|BIBSA643-15|RVcoll. 15-A806||Italy|658[0n]
Vanessa cardui|BIBSA485-15|LEP-SS-00105||Italy|658[0n]
Vanessa cardui|BIBSA486-15|LEP-SS-00106||Italy|658[0n]
Vanessa cardui|BIBSA1538-16|11-D071||Italy|658[0n]
Vanessa cardui|BIBSA1709-16|15-N148||Italy|658[0n]
Vanessa cardui|BIBSA144-15|RVcoll.14-I157||Italy|658[0n]
Vanessa cardui|BIBSA1855-17|RVcoll16C737||Italy|658[0n]
Vanessa cardui|BIBSA1871-17|RVcoll16C767||Italy|658[0n]
Vanessa cardui|BIBSA1665-16|15-C769||Italy|658[0n]
Vanessa cardui|LEATJ1272-16|TLMF Lep 19595||Czech Republic|658[0n]
Vanessa cardui|OXB714-15|13-T909||Italy|658[0n]
Vanessa cardui|OXB914-15|15-A558||Italy|658[0n]
Vanessa cardui|OXB941-15|15-A585||Italy|658[0n]
Vanessa cardui|OXB959-15|15-A909||Italy|658[0n]
Vanessa cardui|OXB982-15|15-A932||Italy|658[0n]
Vanessa cardui|OXB1068-15|14j461||Italy|658[0n]
Vanessa cardui|OXB1076-15|15-A609||Italy|658[0n]

[illegible]
Vanessa cardui|OXB982-15|15-A932||Italy|658[0n]
Vanessa cardui|OXB1068-15|14j461||Italy|658[0n]
Vanessa cardui|OXB1076-15|15-A609||Italy|658[0n]
Vanessa cardui|OXB1415-15|Rvcoll_15-M793||France|658[0n]
Vanessa cardui|LEFIJ4720-16|ZMBSU-01172||Belarus|658[2n]
Vanessa cardui|BIBSA283-15|RVcoll.14-E083||Italy|614[0n]
Vanessa cardui|WMB1063-13|RVcoll.10-C684|Stromboli|Italy|630[0n]
Vanessa cardui|WMB2738-13|RVcoll.10-C596||Malta|658[0n]
Vanessa cardui|WMB1120-13|RVcoll.11-D183|Marettimo|Italy|658[0n]
Vanessa cardui|WMB2830-13|RVcoll.12-O488|Sardinia|Italy|658[0n]
Vanessa cardui|WMB2807-13|RVcoll.12-M368|Linosa|Italy|658[0n]
Vanessa cardui|WMB3026-14|RVcoll.13-S390|Ibiza|Spain|658[0n]
Vanessa cardui|WMB3021-14|RVcoll.13-S337|Mallorca|Spain|658[0n]
Vanessa cardui|WMB3402-14|RVcoll.09-V799||Spain|658[0n]
Vanessa cardui|WMB3382-14|RVcoll.09-V399||Spain|658[0n]
Vanessa cardui|WMB129-11|RVcoll.11-D127|Pantelleria|Italy|658[0n]
Vanessa cardui|WMB232-11|RVcoll.09-T561||Italy|658[0n]
Vanessa cardui|WMB147-11|RVcoll.11-D209|Sicily|Italy|658[0n]
Vanessa cardui|WMB144-11|RVcoll.11-D182|Marettimo|Italy|658[0n]
Vanessa cardui|EZSPN998-09|RVcoll.08-M077||Spain|658[0n]
Vanessa cardui|EZSPN510-09|RVcoll.08-H968||Spain|658[0n]
Vanessa cardui|EZSPM1005-12|Rvcoll.12-L893||Spain|658[0n]
Vanessa cardui|EZSPM984-12|Rvcoll.12-M752||Spain|658[0n]
Vanessa cardui|EZSPM031-09|RVcoll.08-P230||Spain|658[0n]
Vanessa cardui|EZSPN447-09|RVcoll.08-H639||Spain|658[0n]
Vanessa cardui|EZSPN312-09|RVcoll.08-H121||Spain|658[0n]
Vanessa cardui|EZSPC378-09|RVcoll.07-C077||Spain|658[0n]
Vanessa cardui|EZSPC377-09|RVcoll.08-R296||Spain|658[0n]
Vanessa cardui|EZSPN436-09|RVcoll.08-H618||Spain|658[0n]
Vanessa cardui|EZSPC379-09|RVcoll.07-F334||Spain|658[0n]
Vanessa cardui|EZROM141-08|RV-07-D416||Romania|658[0n]
Vanessa cardui|EZROM140-08|RV-07-D063||Romania|657[0n]
Vanessa cardui|EZROM624-08|RV-07-E382||Romania|658[0n]
Vanessa cardui|EZRMN303-08|RVcoll.08-M382||Romania|658[0n]
Vanessa cardui|EZRMN301-08|RVcoll.07-E484||Romania|658[0n]
Vanessa cardui|EZROM142-08|RV-07-D426||Romania|658[0n]
Vanessa cardui|GWORK320-09|BC ZSM Lep 21555||Germany|658[0n]
Vanessa cardui|LEFIJ525-10|MM17150||Finland|658[0n]
Vanessa cardui|GWORR412-10|BC ZSM Lep 29628||Italy|658[0n]
Vanessa cardui|PHLAW055-13|TLMF Lep 09852||Austria|658[0n]
Vanessa cardui|LEATJ1317-16|TLMF Lep 19640||Austria|658[0n]
Vanessa cardui|LEATG075-14|TLMF Lep 13862||Austria|658[0n]
Vanessa cardui|OXB999-15|15-A949||Italy|658[0n]
Vanessa cardui|OXB866-15|15-A510||Italy|658[0n]
Vanessa cardui|EULEP1378-15|RVcoll.14-F976||Greece|658[0n]
Vanessa cardui|WMB5464-14|RVcoll.14-N645|Vulcano|Italy|658[0n]
Vanessa cardui|WMB5346-14|RVcoll.14-J378|Giannutri|Italy|658[0n]
Vanessa cardui|WMB632-11|RVcoll.11-I117||Italy|658[0n]
Vanessa cardui|WMB322-11|RVcoll.11-E607||Italy|658[0n]
Vanessa cardui|WMB1295-13|RVcoll.12-M567|Sicily|Italy|658[0n]
Vanessa cardui|WMB1510-13|RVcoll.12-O100|Corsica|France|658[0n]
Vanessa cardui|WMB2739-13|RVcoll.10-C597||Malta|658[0n]
Vanessa cardui|EZSPM214-09|RVcoll.08-R335||Spain|658[0n]
Vanessa cardui|BIBSA1785-16|16-A518||Italy|610[0n]
Vanessa cardui|WMB4638-14|RVcoll.LD-2404|Corsica|France|658[0n]
Vanessa cardui|WMB5323-14|RVcoll.14-I990|La Maddalena|Italy|658[0n]
Vanessa cardui|BIBSA099-15|RVcoll.14-D521||Italy|658[0n]
Vanessa cardui|OXB1128-15|15-A661||Italy|658[0n]
Vanessa cardui|OXB867-15|15-A511||Italy|630[0n]
Vanessa cardui|LEFIB378-10|MM00952||Finland|621[0n]
Vanessa cardui|EULEP824-15|RVcoll.14-B610||Ukraine|658[1n]
Vanessa cardui|WMB4864-14|RVcoll.14-I411||Italy|658[0n]
Vanessa cardui|WMB4916-14|RVcoll.14-I463||Italy|658[0n]
Vanessa cardui|WMB5140-14|RVcoll.07-E143||Italy|658[0n]
Vanessa cardui|WMB5478-14|RVcoll.14-N703|Alicudi|Italy|658[0n]
Vanessa cardui|EULEP5661-17|RVcoll15L622||Italy|658[0n]
Vanessa cardui|EULEP754-15|RVcoll.12-Q564||Sweden|658[0n]
Vanessa cardui|BIBSA1159-15|15-L125||Italy|658[0n]
Vanessa cardui|BIBSA1093-15|15-L342||Italy|658[0n]
Vanessa cardui|BIBSA1583-16|12-M403||Italy|658[0n]
Vanessa cardui|OXB356-15|RVcoll.14-N070||Italy|658[0n]
Vanessa cardui|OXB1538-16|OXB-TGS-1256||France|658[0n]
Vanessa cardui|OXB253-15|OXB-TGS-821||United Kingdom|658[0n]
Vanessa cardui|OXB1345-15|Rvcoll_15-M693||France|658[0n]
Vanessa cardui|WMB4802-14|RVcoll. 14-I349||Italy|632[0n]
Vanessa cardui|WMB488-11|RVcoll.11-H533|Sicily|Italy|658[0n]
Vanessa cardui|WMB4701-14|RVcoll.LD-2895|Asinara|Italy|658[0n]
Vanessa cardui|WMB4333-14|RVcoll.14-A717||Italy|658[0n]
Vanessa cardui|WMB4325-14|RVcoll.14-A662||Italy|658[0n]
Vanessa cardui|WMB4234-14|RVcoll.13-T834||Italy|658[0n]
Vanessa cardui|WMB2641-13|RVcoll.12-R449|Corsica|France|658[0n]
Vanessa cardui|WMB3827-14|RVcoll.09-X836||Italy|658[0n]
Vanessa cardui|WMB2198-13|RVcoll.09-X947|Ponza|Italy|658[0n]
Vanessa cardui|WMB908-13|RVcoll.09-X897|Capri|Italy|658[0n]
Vanessa cardui|WMB876-13|RVcoll.09-X259||France|658[0n]
Vanessa cardui|WMB3638-14|RVcoll.11-I451||Spain|658[0n]
Vanessa cardui|EZSPN305-09|RVcoll.08-H110||Spain|658[0n]
Vanessa cardui|EZSPM983-12|Rvcoll.12-M751||Spain|658[0n]
Vanessa cardui|EZSPC376-09|RVcoll.08-M717||Spain|658[0n]
Vanessa cardui|EZRMN304-08|RVcoll.08-M398||Romania|658[0n]
Vanessa cardui|EZRMN302-08|RVcoll.08-M362||Romania|658[0n]
Vanessa cardui|EZRMN300-08|RVcoll.07-D934||Romania|658[0n]
Vanessa cardui|LEFIG133-10|MM13949||Finland|658[0n]
Vanessa cardui|GWORL445-09|BC ZSM Lep 22347||Germany|658[0n]
Vanessa cardui|ABOLD006-16|TLMF Lep 21084||Austria|658[0n]
Vanessa cardui|LEATJ259-15|TLMF Lep 18689||Italy|658[0n]
Aricia agestis|WMB5289-14|RVcoll.14-H966||France|658[0n]
Aricia agestis|GBGL18182-15|KM459044||Italy|655[0n]
Aricia agestis|GBMIN16312-13|JX678146||Spain|646[0n]
Aricia agestis|EULEP4985-16|RVcoll16I675||Poland|658[0n]
Aricia agestis|EULEP5057-16|RVcoll16J105||Czech Republic|658[0n]
Aricia agestis|WMB6379-18|RVcoll06G510|Genit. examined|Spain|658[0n]
[illegible]

Aricia agestis|EULEP4985-16|RVcoll16I675||Poland|658[0n]
Aricia agestis|EULEP5057-16|RVcoll16J105||Czech Republic|658[0n]
Aricia agestis|WMB6379-18|RVcoll06G510|Genit. examined|Spain|658[0n]
Aricia agestis|WMB6362-18|RVcoll06A202|Genit. examined|Spain|658[0n]
Aricia agestis|WMB6360-18|RVcoll06A151|Genit. examined|Spain|658[0n]
Aricia agestis|WMB6477-18|RVcoll12Q430|Genit. examined|Spain|658[0n]
Aricia agestis|WMB6471-18|RVcoll11D594|Genit. examined|Spain|658[0n]
Aricia agestis|WMB6458-18|RVcoll09X310|Genit. examined|Spain|658[0n]
Aricia agestis|WMB6450-18|RVcoll09X033|Genit. examined|Spain|658[0n]
Aricia agestis|WMB6433-18|RVcoll08P096|Genit. examined|Spain|658[0n]
Aricia agestis|GWOSU023-11|BC ZSM Lep 53274||Germany|658[0n]
Aricia agestis|GWOSU020-11|BC ZSM Lep 53271||Germany|658[0n]
Aricia agestis|GWORL215-09|BC ZSM Lep 18621||Germany|658[0n]
Aricia agestis|GWORL211-09|BC ZSM Lep 18617||Germany|658[0n]
Aricia agestis|FBLMW309-10|BC ZSM Lep 37410||Germany|658[0n]
Aricia agestis|FBLMW308-10|BC ZSM Lep 37409||Germany|658[0n]
Aricia agestis|FBLMW306-10|BC ZSM Lep 37407||Germany|658[0n]
Aricia agestis|FBLMW287-10|BC ZSM Lep 37388||Germany|658[0n]
Aricia agestis|GWORO799-09|BC ZSM Lep 30491||Germany|658[0n]
Aricia agestis|GWORZ054-10|BC ZSM Lep 30410||Italy|658[0n]
Aricia agestis|GWORL221-09|BC ZSM Lep 18627||Germany|658[0n]
Aricia agestis|GWORL213-09|BC ZSM Lep 18619||Germany|658[0n]
Aricia agestis|GWORL195-09|BC ZSM Lep 18601||Italy|658[0n]
Aricia agestis|PHLSA627-11|TLMF Lep 06082||Italy|658[0n]
Aricia agestis|WMB3490-14|RVcoll.10-B684||France|658[0n]
Aricia agestis|GWOSK861-11|BC ZSM Lep 49362||Germany|658[0n]
Aricia agestis|WMB6431-18|RVcoll08M923|Genit. examined|Spain|658[0n]
Aricia agestis|WMB4038-14|RVcoll.12-Z345||Spain|658[0n]
Aricia agestis|WMB6407-18|RVcoll07F500|Genit. examined|Spain|658[0n]
Aricia agestis|WMB6389-18|RVcoll07C284|Genit. examined|Spain|658[0n]
Aricia agestis|WMB5362-14|RVcoll.14-J728||France|658[0n]
Aricia agestis|WMB6524-18|RVcoll12Q618|Genit. examined|Spain|658[0n]
Aricia agestis|EULEP3482-16|RVcoll15G131||France|658[0n]
Aricia agestis|WMB5053-14|RVcoll. 14-L184||Italy|658[0n]
Aricia agestis|EULEP960-15|RVcoll.14-D018||Bulgaria|658[0n]
Aricia agestis|EULEP1205-15|RVcoll.14-F500||Greece|658[0n]
Aricia agestis|BIBSA1221-15|15-L954||Italy|658[0n]
Aricia agestis|EULEP1710-15|RVcoll.14-H159||Greece|658[0n]
Aricia agestis|BIBSA1051-15|15-M760||France|658[0n]
Aricia agestis|BIBSA1609-16|14-A735||Italy|658[0n]
Aricia agestis|GBLAA386-14|BC ZSM Lep 80477||Germany|658[0n]
Aricia agestis|BIBSA1758-16|12-Q858||Italy|658[0n]
Aricia agestis|BIBSA045-14|LEP-SS-00044||Italy|658[0n]
Aricia agestis|BIBSA044-14|LEP-SS-00043||Italy|658[0n]
Aricia agestis|OXB1531-16|OXB-TGS-1249||United Kingdom|658[0n]
Aricia agestis|LEASS837-17|TLMF Lep 22485||Austria|658[0n]
Aricia agestis|OXB704-15|13-T881||Italy|658[0n]
Aricia agestis|OXB622-15|14-O011||Italy|658[0n]
Aricia agestis|OXB215-15|OXB-TGS-346||United Kingdom|658[0n]
Aricia agestis|OXB351-15|RVcoll.14-N065||Italy|658[0n]
Aricia agestis|OXB273-15|OXB-TGS-956||United Kingdom|658[0n]
Aricia agestis|OXB247-15|OXB-TGS-763||United Kingdom|658[0n]
Aricia agestis|GWORL253-09|BC ZSM Lep 18659||Germany|658[0n]
Aricia agestis|GWORL251-09|BC ZSM Lep 18657||Germany|658[0n]
Aricia agestis|GWOSU025-11|BC ZSM Lep 53276||Germany|658[0n]
Aricia agestis|GWOSU024-11|BC ZSM Lep 53275||Germany|658[0n]
Aricia agestis|GBGL18168-15|KM459030||Italy|655[0n]
Aricia agestis|EZSPC189-09|RVcoll.06-A100|Genit. examined|Spain|632[0n]
Aricia agestis|GWORL245-09|BC ZSM Lep 18651||Germany|641[0n]
Aricia agestis|GWORL225-09|BC ZSM Lep 18631||Germany|641[0n]
Aricia agestis|EULEP4947-16|RVcoll16I165||Germany|629[0n]
Aricia agestis|GBGL18216-15|KM459078||France|655[0n]
Aricia agestis|BIBSA997-15|15-C033||Italy|624[0n]
Aricia agestis|EULEP3480-16|RVcoll10A945||Bulgaria|624[0n]
Aricia agestis|GWORL207-09|BC ZSM Lep 18613||Germany|632[0n]
Aricia agestis|GWORL209-09|BC ZSM Lep 18615||Germany|658[0n]
Aricia agestis|GWORL205-09|BC ZSM Lep 18611||Germany|658[0n]
Aricia agestis|OXB1218-15|Rvcoll_15-M644||France|658[0n]
Aricia agestis|OXB1354-15|Rvcoll_15-M706||France|658[0n]
Aricia agestis|GBGL18220-15|KM459082||Italy|655[0n]
Aricia agestis|GBGL18219-15|KM459081||Italy|655[0n]
Aricia agestis|WMB6487-18|RVcoll12Q441|Genit. examined|Spain|658[0n]
Aricia agestis|GBGL18234-15|KM459096||Spain|655[0n]
Aricia agestis|GBGL18235-15|KM459097||Spain|655[0n]
Aricia agestis|GBGL18236-15|KM459098||Spain|655[0n]
Aricia agestis|WMB6479-18|RVcoll12Q433|Genit. examined|Spain|658[0n]
Aricia agestis|GBMIN16172-13|JX678149||Spain|645[3n]
Aricia agestis|EZSPC193-09|RVcoll.06-A251|Genit. examined|Spain|648[0n]
Aricia agestis|GWORL257-09|BC ZSM Lep 18663||Germany|658[0n]
Aricia agestis|GWORL255-09|BC ZSM Lep 18661||Germany|658[0n]
Aricia agestis|GWORL219-09|BC ZSM Lep 18625||Germany|658[0n]
Aricia agestis|GWORL217-09|BC ZSM Lep 18623||Germany|658[0n]
Aricia agestis|GBGL18181-15|KM459043||Italy|655[0n]
Aricia agestis|GBGL18177-15|KM459039||Spain|655[0n]
Aricia agestis|GWOSU017-11|BC ZSM Lep 53268||Germany|658[0n]
Aricia agestis|GWOSU014-11|BC ZSM Lep 53265||Germany|658[0n]
Aricia agestis|GBGL18178-15|KM459040||Spain|655[0n]
Aricia agestis|GWORL265-09|BC ZSM Lep 18671||Germany|658[1n]
Aricia agestis|GWORL249-09|BC ZSM Lep 18655||Germany|658[0n]
Aricia agestis|GWORL247-09|BC ZSM Lep 18653||Germany|658[0n]
Aricia agestis|GBGL18172-15|KM459034||France|655[0n]
Aricia agestis|GBGL18171-15|KM459033||Italy|655[0n]
Aricia agestis|GWORL243-09|BC ZSM Lep 18649||Germany|658[0n]
Aricia agestis|GWORL241-09|BC ZSM Lep 18647||Germany|658[0n]
Aricia agestis|GWORL279-09|BC ZSM Lep 18685||Germany|658[0n]
Aricia agestis|GWORL275-09|BC ZSM Lep 18681|Identified by barcode|Poland|658[0n]
Aricia agestis|EULEP1425-15|RVcoll.14-G102||Greece|658[0n]
Aricia agestis|ABOLD068-16|TLMF Lep 21146||Austria|658[0n]
Aricia agestis|GBGL18175-15|KM459037||France|655[0n]
Aricia agestis|GBGL18173-15|KM459035||Italy|655[0n]
Aricia agestis|GBGL18238-15|KM459100|Elba|Italy|655[0n]
Aricia agestis|GBGL18237-15|KM459099||Spain|655[0n]
Aricia agestis|EULEP926-15|RVcoll.14-C809||Bulgaria|658[0n]

Aricia agestis|GBGL18238-15|KM459100|Elba|Italy|655[0n]
Aricia agestis|GBGL18237-15|KM459099||Spain|655[0n]
Aricia agestis|EULEP926-15|RVcoll.14-C809||Bulgaria|658[0n]
Aricia agestis|GWORL271-09|BC ZSM Lep 18677||Germany|658[0n]
Aricia agestis|GWORL270-09|BC ZSM Lep 18676||Germany|658[0n]
Aricia agestis|GWORL239-09|BC ZSM Lep 18645||Germany|658[0n]
Aricia agestis|GWORL237-09|BC ZSM Lep 18643||Germany|658[0n]
Aricia agestis|GBMIN16323-13|JX678124||France|657[0n]
Aricia agestis|GBMIN16182-13|JX678129||United Kingdom|657[0n]
Aricia agestis|GBGL18214-15|KM459076|Argentario|Italy|655[0n]
Aricia agestis|GBGL18213-15|KM459075|Argentario|Italy|655[0n]
Aricia agestis|GWORL268-09|BC ZSM Lep 18674||Germany|658[0n]
Aricia agestis|GWORL267-09|BC ZSM Lep 18673||Germany|658[0n]
Aricia agestis|GBGL18233-15|KM459095||Spain|655[0n]
Aricia agestis|GBGL18226-15|KM459088||Italy|655[0n]
Aricia agestis|GBGL18207-15|KM459069|Pianosa|Italy|655[0n]
Aricia agestis|BIBSA046-14|LEP-SS-00045||Italy|658[0n]
Aricia agestis|BIBSA1363-15|15-M886||Italy|658[0n]
Aricia agestis|WMB5081-14|RVcoll. 14-L212||Italy|658[0n]
Aricia agestis|WMB4223-14|RVcoll.13-T758||Italy|658[0n]
Aricia agestis|GWORL192-09|BC ZSM Lep 18598||Italy|658[0n]
Aricia agestis|GBMIN16321-13|JX678128||United Kingdom|657[0n]
Aricia agestis|GBMIN16183-13|JX678127||Greece|657[0n]
Aricia agestis|GBGL18210-15|KM459072|Pianosa|Italy|655[0n]
Aricia agestis|GWORL269-09|BC ZSM Lep 18675||Germany|658[0n]
Aricia agestis|GWORL266-09|BC ZSM Lep 18672||Germany|658[0n]
Aricia agestis|GWORL264-09|BC ZSM Lep 18670||Germany|658[0n]
Aricia agestis|GWORL235-09|BC ZSM Lep 18641||Germany|658[0n]
Aricia agestis|GWORL233-09|BC ZSM Lep 18639||Germany|658[0n]
Aricia agestis|GBMIN16184-13|JX678125||Serbia|657[0n]
Aricia agestis|OXB1391-15|Rvcoll_15-M756||France|658[0n]
Aricia agestis|GBGL18225-15|KM459087||France|655[0n]
Aricia agestis|GBGL18224-15|KM459086||France|655[0n]
Aricia agestis|GBGL18174-15|KM459036||France|655[0n]
Aricia agestis|GBGL18215-15|KM459077||France|655[0n]
Aricia agestis|GWORL263-09|BC ZSM Lep 18669||Germany|658[0n]
Aricia agestis|GWORL262-09|BC ZSM Lep 18668||Germany|658[0n]
Aricia agestis|GWORL231-09|BC ZSM Lep 18637||Germany|658[0n]
Aricia agestis|GWORL229-09|BC ZSM Lep 18635||Germany|658[0n]
Aricia agestis|GWORL227-09|BC ZSM Lep 18633||Germany|658[0n]
Aricia agestis|GWORL223-09|BC ZSM Lep 18629||Germany|658[0n]
Aricia agestis|GWORL260-09|BC ZSM Lep 18666||Germany|658[0n]
Aricia agestis|GWORL259-09|BC ZSM Lep 18665||Germany|658[0n]
Aricia agestis|BIBSA660-15|RVcoll. 15-A823||Italy|658[0n]
Aricia agestis|EZROM041-08|RV-07-D155|Genit. examined|Romania|658[0n]
Aricia agestis|GBGL18212-15|KM459074|Argentario|Italy|655[0n]
Aricia agestis|LEASS701-17|TLMF Lep 22349||Austria|658[0n]
Aricia agestis|GBMIN16319-13|JX678132||Slovenia|657[0n]
Aricia agestis|LEASS699-17|TLMF Lep 22347||Austria|658[0n]
Aricia agestis|EZROM048-08|RV-07-E536|Genit. examined|Romania|658[0n]
Aricia agestis|EZROM044-08|RV-06-K652|Genit. examined|Romania|658[0n]
Aricia agestis|EZROM043-08|RV-07-C131|Genit. examined|Romania|658[0n]
Aricia agestis|EZROM042-08|RV-06-K666|Genit. examined|Romania|658[0n]
Aricia agestis|EZROM606-08|RV-07-E466|Genit. examined|Romania|658[0n]
Aricia agestis|EZROM605-08|RV-07-D523|Genit. examined|Romania|658[0n]
Aricia agestis|EZROM601-08|RV-06-M830|Genit. examined|Romania|658[0n]
Aricia agestis|EZROM793-08|RVcoll.08-M519|Genit. examined|Romania|658[0n]
Aricia agestis|ABOLD655-17|TLMF Lep 21731||Austria|658[0n]
Aricia agestis|BIBSA1260-15|15-F880||France|658[0n]
Aricia agestis|OXB573-15|14-N962||Italy|658[0n]
Aricia agestis|WMB6504-18|RVcoll12Q539|Genit. examined|Spain|658[0n]
Aricia agestis|WMB6510-18|RVcoll12Q545||Spain|658[0n]
Aricia agestis|WMB6357-18|RVcoll06A102|Genit. examined|Spain|658[0n]
Aricia agestis|WMB6372-18|RVcoll06A230||Spain|658[0n]
Aricia agestis|WMB6387-18|RVcoll07C070|Genit. examined|Spain|658[0n]
Aricia agestis|BIBSA1181-15|15-M219||Italy|658[0n]
Aricia agestis|BIBSA1247-15|15-F826||France|658[0n]
Aricia agestis|BIBSA980-15|14-W154||Italy|658[0n]
Aricia agestis|BIBSA1049-15|15-M662||France|658[0n]
Aricia agestis|BIBSA181-15|RVcoll.14-D969||Italy|658[0n]
Aricia agestis|BIBSA072-14|LEP-SS-00072||Italy|658[0n]
Aricia agestis|OXB823-15|14-U786||France|658[0n]
Aricia agestis|OXB880-15|15-A522||Italy|658[0n]
Aricia agestis|OXB1530-16|OXB-TGS-1248||United Kingdom|658[0n]
Aricia agestis|OXB957-15|15-A907||Italy|658[0n]
Aricia agestis|OXB1100-15|15-A633||Italy|658[0n]
Aricia agestis|OXB250-15|OXB-TGS-791||United Kingdom|658[0n]
Aricia agestis|OXB1325-15|Rvcoll_15-M597||France|658[0n]
Aricia agestis|GBMIN16315-13|JX678140||Spain|657[0n]
Aricia agestis|GBMIN16320-13|JX678130||Belgium|657[0n]
Aricia agestis|GBMIN16181-13|JX678131||Italy|657[0n]
Aricia agestis|GBGL18167-15|KM459029||Spain|655[0n]
Aricia agestis|GBGL18169-15|KM459031||Italy|655[0n]
Aricia agestis|GBGL18206-15|KM459068||Italy|655[0n]
Aricia agestis|GBGL18211-15|KM459073|Pianosa|Italy|655[0n]
Aricia agestis|GBGL18241-15|KM459103|Elba|Italy|655[0n]
Aricia agestis|GBMIN16215-13|JX678063||Spain|646[0n]
Aricia agestis|GBMIN16353-13|JX678064||Spain|646[0n]
Aricia agestis|WMB6486-18|RVcoll12Q440|Genit. examined|Spain|658[1n]
Aricia agestis|WMB6485-18|RVcoll12Q439|Genit. examined|Spain|658[0n]
Aricia agestis|WMB6483-18|RVcoll12Q437|Genit. examined|Spain|658[0n]
Aricia agestis|WMB6482-18|RVcoll12Q436|Genit. examined|Spain|658[0n]
Aricia agestis|WMB6481-18|RVcoll12Q435|Genit. examined|Spain|658[0n]
Aricia agestis|WMB6478-18|RVcoll12Q432|Genit. examined|Spain|658[0n]
Aricia agestis|WMB6473-18|RVcoll11J240|Genit. examined|Spain|658[0n]
Aricia agestis|WMB6472-18|RVcoll11J239|Genit. examined|Spain|658[0n]
Aricia agestis|WMB6455-18|RVcoll09X070|Genit. examined|Spain|658[0n]
Aricia agestis|EZROM039-08|RV-07-E399|Genit. examined|Romania|658[0n]
Aricia agestis|EZROM790-08|RVcoll.08-M373|Genit. examined|Romania|658[0n]
Aricia agestis|FBLMW341-10|BC ZSM Lep 37442|Identified by barcode|Switzerland|658[0n]
Aricia agestis|FBLMW297-10|BC ZSM Lep 37398|Identified by barcode|Germany|658[0n]
Aricia agestis|GBGL18223-15|KM459085||France|655[0n]

Aricia agestis|FBLMW341-10|BC ZSM Lep 37442|Identified by barcode|Switzerland|658[0n]
Aricia agestis|FBLMW297-10|BC ZSM Lep 37398|Identified by barcode|Germany|658[0n]
Aricia agestis|GBGL18223-15|KM459085||France|655[0n]
Aricia agestis|GBGL18184-15|KM459046||Italy|655[0n]
Aricia agestis|WMB5219-14|RVcoll.13-T969||Italy|658[0n]
Aricia agestis|GBGL18227-15|KM459089||Italy|655[0n]
Aricia agestis|WMB4803-14|RVcoll. 14-I350||Italy|658[0n]
Aricia agestis|WMB4776-14|RVcoll. 14-I323||Italy|658[0n]
Aricia agestis|EULEP5689-17|RVcoll16L032||Italy|658[0n]
Aricia agestis|WMB3948-14|RVcoll.12-P705||France|620[0n]
Aricia agestis|GWORL191-09|BC ZSM Lep 18597||Germany|658[0n]
Aricia agestis|GWORL189-09|BC ZSM Lep 18595||Germany|658[0n]
Aricia agestis|GBMIN16313-13|JX678144||Greece|657[0n]
Aricia agestis|EZROM040-08|RV-07-E419|Genit. examined|Romania|658[0n]
Aricia agestis|BIBSA1041-15|15-C219||Italy|658[0n]
Aricia agestis|GBGL18209-15|KM459071|Pianosa|Italy|655[0n]
Aricia agestis|BIBSA1713-16|15-N173||Italy|658[0n]
Aricia agestis|EULEP723-15|RVcoll.12-N804|Lesvos|Greece|658[0n]
Aricia agestis|WMB4154-14|RVcoll.13-S710||Italy|617[0n]
Aricia agestis|GBGL18170-15|KM459032||France|655[0n]
Aricia agestis|GBMIN16187-13|JX678119||Italy|657[0n]
Aricia agestis|GBGL18183-15|KM459045||Italy|655[0n]
Aricia agestis|GBGL18179-15|KM459041||France|655[0n]
Aricia agestis|GBMIN16326-13|JX678118||Italy|657[0n]
Aricia agestis|GBMIN16325-13|JX678120||Italy|657[0n]
Aricia agestis|GBMIN16324-13|JX678122||France|657[0n]
Aricia agestis|GBMIN16186-13|JX678121||Greece|657[0n]
Aricia agestis|WMB4714-14|RVcoll.LD-2949||Italy|658[0n]
Aricia agestis|WMB3880-14|RVcoll.12-M512||Italy|658[0n]
Aricia agestis|WMB276-11|RVcoll.10-A523||France|658[0n]
Aricia agestis|GWORZ055-10|BC ZSM Lep 30411||Italy|658[0n]
Aricia agestis|OXB366-15|RVcoll.14-N080||Italy|658[0n]
Aricia agestis|EULEP712-15|RVcoll.12-M246|Crete|Greece|658[0n]
Aricia agestis|GBGL18194-15|KM459056|Corsica|France|655[0n]
Aricia agestis|GBMIN16179-13|JX678135|Corsica|France|657[0n]
Aricia agestis|GBGL18193-15|KM459055|Corsica|France|655[0n]
Aricia agestis|GBGL18243-15|KP052710|Capraia|Italy|655[0n]
Aricia agestis|GBGL18190-15|KM459052|Corsica|France|655[0n]
Aricia agestis|GBMIN16174-13|JX678145||Spain|646[0n]
Aricia agestis|GBMIN16211-13|JX678071||Spain|657[0n]
Aricia agestis|GBGL18176-15|KM459038||France|655[0n]
Aricia agestis|GBMIN16349-13|JX678072||Spain|657[0n]
Aricia agestis|OXB1203-15|Rvcoll_15-M619||France|658[0n]
Aricia agestis|WMB6393-18|RVcoll07C288|Genit. examined|Spain|658[0n]
Aricia agestis|WMB6480-18|RVcoll12Q434|Genit. examined|Spain|658[0n]
Aricia agestis|WMB6476-18|RVcoll12Q421|Genit. examined|Spain|658[0n]
Aricia agestis|WMB6451-18|RVcoll09X034|Genit. examined|Spain|658[0n]
Aricia agestis|WMB6429-18|RVcoll08L531|Genit. examined|Spain|658[0n]
Aricia agestis|WMB6428-18|RVcoll08L523|Genit. examined|Spain|658[0n]
Aricia agestis|WMB6427-18|RVcoll08L522|Genit. examined|Spain|658[0n]
Aricia agestis|WMB6422-18|RVcoll08L499|Genit. examined|Spain|658[0n]
Aricia agestis|EZSPC190-09|RVcoll.06-A218|Genit. examined|Spain|658[0n]
Aricia agestis|GBGL18228-15|KM459090||Italy|655[0n]
Aricia agestis|GBGL18204-15|KM459066|Sicily|Italy|655[0n]
Aricia agestis|GBGL18217-15|KM459079|Sicily|Italy|655[0n]
Aricia agestis|GBGL18218-15|KM459080|Sicily|Italy|655[0n]
Aricia agestis|GBGL18232-15|KM459094|Sicily|Italy|655[0n]
Aricia agestis|WMB715-12|RVcoll.11-H951|Sicily|Italy|658[0n]
Aricia agestis|WMB710-12|RVcoll.11-H342|Sicily|Italy|658[0n]
Aricia agestis|WMB744-12|RVcoll.11-J702|Sicily|Italy|658[0n]
Aricia agestis|WMB717-12|RVcoll.11-I007|Sicily|Italy|658[0n]
Aricia agestis|GBMIN16316-13|JX678138|Sicily|Italy|657[0n]
Aricia agestis|OXB689-15|12-M384|Sicily W|Italy|658[0n]
Aricia agestis|GBGL18187-15|KM459049|Sicily|Italy|655[0n]
Aricia agestis|GBGL18185-15|KM459047|Sicily|Italy|655[0n]
Aricia agestis|GBGL18189-15|KM459051|Sicily|Italy|655[0n]
Aricia agestis|GBGL18188-15|KM459050|Sicily|Italy|655[0n]
Aricia agestis|GBGL18199-15|KM459061|Vulcano|Italy|655[0n]
Aricia agestis|GBGL18198-15|KM459060|Sicily|Italy|655[0n]
Aricia agestis|GBGL18202-15|KM459064|Vulcano|Italy|655[0n]
Aricia agestis|GBGL18201-15|KM459063|Vulcano|Italy|655[0n]
Aricia agestis|GBGL18229-15|KM459091|Sicily|Italy|655[0n]
Aricia agestis|GBGL18231-15|KM459093|Sicily|Italy|655[0n]
Aricia agestis|WMB074-11|RVcoll.LD-2969|Sicily|Italy|658[0n]
Aricia agestis|WMB073-11|RVcoll.LD-2968|Sicily|Italy|658[0n]
Aricia agestis|GBMIN16178-13|JX678137|Sicily|Italy|657[0n]
Aricia agestis|GBGL18239-15|KM459101|Sicily|Italy|655[0n]
Aricia agestis|GBGL18230-15|KM459092|Sicily|Italy|655[0n]
Aricia agestis|GBGL18203-15|KM459065|Vulcano|Italy|655[0n]
Aricia agestis|GBGL18205-15|KM459067|Sicily|Italy|655[0n]
Aricia agestis|GBGL18240-15|KM459102|Sicily|Italy|654[0n]
Aricia agestis|GBGL18186-15|KM459048|Sicily|Italy|655[0n]
Aricia agestis|GBGL18200-15|KM459062|Vulcano|Italy|655[0n]
Aricia agestis|WMB2879-14|RVcoll.10-C676|Lipari|Italy|658[0n]
Aricia agestis|WMB230-11|RVcoll.09-T554|Lipari|Italy|658[0n]
Aricia agestis|GBGL18191-15|KM459053|Corsica|France|655[0n]
Aricia agestis|GBGL18196-15|KM459058|Corsica|France|655[0n]
Aricia agestis|GBGL18208-15|KM459070|Pianosa|Italy|655[0n]
Aricia agestis|GBGL18221-15|KM459083|Corsica|France|655[0n]
Aricia agestis|GBGL18222-15|KM459084|Corsica|France|655[0n]
Aricia agestis|GBGL18242-15|KM459104|Elba|Italy|655[0n]
Aricia agestis|GBGL18195-15|KM459057|Corsica|France|630[0n]
Aricia agestis|GBGL18180-15|KM459042|Elba|Italy|655[0n]
Aricia agestis|GBGL18192-15|KM459054|Corsica|France|655[0n]
Aricia agestis|GBGL18197-15|KM459059|Corsica|France|655[0n]
Aricia agestis|GBMIN16318-13|JX678134|Corsica|France|657[0n]
Aricia agestis|OXB1034-15|LD-2414||France|658[0n]
Aricia agestis|BIBSA1290-15|14-E275|Corsica|France|658[0n]
Aricia montensis|WMB6453-18|RVcoll09X068|Genit. examined|Spain|658[0n]
Aricia montensis|WMB6459-18|RVcoll09X318|Genit. examined|Spain|658[0n]
Aricia montensis|WMB6454-18|RVcoll09X069|Genit. examined|Spain|658[0n]
Aricia montensis|WMB6452-18|RVcoll09X038|Genit. examined|Spain|658[0n]
Aricia montensis|WMB6449-18|RVcoll09X014|Genit. examined|Spain|658[0n]

Aricia montensis|WMB6454-18|RVcoll09X069|Genit. examined|Spain|658[0n]
Aricia montensis|WMB6452-18|RVcoll09X038|Genit. examined|Spain|658[0n]
Aricia montensis|WMB6449-18|RVcoll09X014|Genit. examined|Spain|658[0n]
Aricia montensis|WMB6448-18|RVcoll09X002|Genit. examined|Spain|658[0n]
Aricia montensis|WMB6436-18|RVcoll08R179|Genit. examined|Spain|658[0n]
Aricia montensis|WMB6432-18|RVcoll08P082|Genit. examined|Spain|658[0n]
Aricia montensis|WMB6426-18|RVcoll08L520|Genit. examined|Spain|658[0n]
Aricia montensis|EZSPC710-10|RVcoll.08-R145|Genit. examined|Spain|658[0n]
Aricia montensis|EZSPC262-09|RVcoll.08-P398|Genit. examined|Spain|658[0n]
Aricia montensis|EZSPC191-09|RVcoll.06-A219|Genit. examined|Spain|658[0n]
Aricia montensis|WMB3384-14|RVcoll.09-V438||Spain|658[0n]
Aricia montensis|EZSPN078-09|RVcoll.09-V560|Genit. examined|Spain|658[0n]
Aricia montensis|WMB6402-18|RVcoll07F076|Genit. examined|Spain|658[0n]
Aricia montensis|GBMIN16352-13|JX678066||Spain|657[0n]
Aricia montensis|WMB6470-18|RVcoll10B507|Genit. examined|Spain|658[0n]
Aricia montensis|WMB6469-18|RVcoll10A883|Genit. examined|Spain|658[0n]
Aricia montensis|WMB6468-18|RVcoll10A882|Genit. examined|Spain|658[0n]
Aricia montensis|WMB6467-18|RVcoll10A862|Genit. examined|Spain|658[0n]
Aricia montensis|WMB6466-18|RVcoll10A853|Genit. examined|Spain|658[0n]
Aricia montensis|WMB3243-14|RVcoll.08-L180||Spain|658[0n]
Aricia montensis|EZSPC749-10|RVcoll.08-L117|Genit. examined|Spain|658[0n]
Aricia montensis|WMB6404-18|RVcoll07F088||Spain|658[0n]
Aricia montensis|WMB6371-18|RVcoll06A227|Genit. examined|Spain|658[0n]
Aricia montensis|WMB6370-18|RVcoll06A225|Genit. examined|Spain|658[0n]
Aricia montensis|WMB6378-18|RVcoll06A250|Genit. examined|Spain|658[0n]
Aricia montensis|EZSPC829-10|RVcoll.08-P927||Spain|619[0n]
Aricia montensis|EZSPN658-09|RVcoll.08-J843||Spain|626[0n]
Aricia montensis|EULEP119-14|RVcoll.09-X375||Spain|658[0n]
Aricia montensis|WMB6406-18|RVcoll07F090|Genit. examined|Spain|658[2n]
Aricia montensis|WMB6405-18|RVcoll07F089|Genit. examined|Spain|658[1n]
Aricia montensis|EZSPC777-10|RVcoll.08-L842||Spain|658[0n]
Aricia montensis|EZSPC788-10|RVcoll.08-M042||Spain|658[0n]
Aricia montensis|EZSPC692-10|RVcoll.06-A247|Genit. examined|Spain|658[0n]
Aricia montensis|WMB3138-14|RVcoll.06-K691||Spain|658[0n]
Aricia montensis|WMB3266-14|RVcoll.08-L843||Spain|658[0n]
Aricia montensis|WMB3280-14|RVcoll.08-M072||Spain|658[0n]
Aricia montensis|WMB3317-14|RVcoll.08-P686||Spain|658[0n]
Aricia montensis|WMB3775-14|RVcoll.12-N718||Spain|658[0n]
Aricia montensis|WMB4039-14|RVcoll.12-Z353||Spain|658[0n]
Aricia montensis|WMB6414-18|RVcoll07Z076|Genit. examined|Spain|658[0n]
Aricia montensis|WMB6419-18|RVcoll08L329|Genit. examined|Spain|658[0n]
Aricia montensis|WMB6420-18|RVcoll08L332|Genit. examined|Spain|658[0n]
Aricia montensis|WMB6456-18|RVcoll09X308|Genit. examined|Spain|658[0n]
Aricia montensis|WMB6457-18|RVcoll09X309|Genit. examined|Spain|658[0n]
Aricia montensis|WMB6461-18|RVcoll09X335||Andorra|658[0n]
Aricia montensis|WMB6462-18|RVcoll09X336||Andorra|658[0n]
Aricia montensis|WMB6463-18|RVcoll10A828|Genit. examined|Spain|658[0n]
Aricia montensis|WMB6464-18|RVcoll10A829|Genit. examined|Spain|658[0n]
Aricia montensis|WMB6465-18|RVcoll10A830|Genit. examined|Spain|658[0n]
Aricia montensis|WMB6377-18|RVcoll06A240|Genit. examined|Andorra|658[0n]
Aricia montensis|WMB6400-18|RVcoll07C693|Genit. examined|Spain|658[0n]
Aricia montensis|WMB6401-18|RVcoll07F060|Genit. examined|Spain|658[0n]
Aricia montensis|EULEP019-14|RVcoll.07-C692|Genit. examined|Spain|658[0n]
Aricia montensis|WMB6403-18|RVcoll07F085|Genit. examined|Spain|658[0n]
Aricia montensis|EULEP017-14|RVcoll.07-C688|Genit. examined|Spain|658[0n]
Aricia montensis|GBMIN16355-13|JX678060||Andorra|646[0n]
Aricia montensis|GBMIN16354-13|JX678062||Spain|646[0n]
Aricia montensis|GBMIN16217-13|JX678059||Andorra|646[0n]
Aricia montensis|GBMIN16216-13|JX678061||Andorra|646[0n]
Aricia montensis|GBMIN16214-13|JX678065||Spain|657[0n]
Aricia montensis|EULEP018-14|RVcoll.07-C689||Spain|658[0n]
Aricia montensis|EZSPM773-12|Rvcoll. 050111LP38||Spain|658[0n]
Aricia montensis|EZSPC272-09|RVcoll.08-R208|Genit. examined|Spain|655[0n]
Aricia montensis|EZSPC192-09|RVcoll.06-A233|Genit. examined|Spain|655[0n]
Aricia montensis|WMB3409-14|RVcoll.09-V922||Spain|658[0n]
Aricia montensis|EZSPN075-09|RVcoll.09-V919|Genit. examined|Spain|658[0n]
Aricia artaxerxes|BIBSA946-15|16-A037||Italy|658[0n]
Aricia artaxerxes|EULEP3485-16|RVcoll15G699||Italy|658[0n]
Aricia artaxerxes|GBMIN16332-13|JX678106||France|657[0n]
Aricia artaxerxes|WMB1662-13|RVcoll.12-P202||France|658[0n]
Aricia artaxerxes|WMB4843-14|RVcoll. 14-I390||Italy|658[0n]
Aricia artaxerxes|WMB3527-14|RVcoll.10-C083||France|658[0n]
Aricia artaxerxes|FBLMW347-10|BC ZSM Lep 37448|Identified by barcode; ssp. allous|Germany|658[0n]
Aricia artaxerxes|FBLMW345-10|BC ZSM Lep 37446||Germany|658[0n]
Aricia artaxerxes|FBLMW330-10|BC ZSM Lep 37431|Identified by barcode|Germany|658[0n]
Aricia artaxerxes|PHLAB324-10|TLMF Lep 01124||Switzerland|623[0n]
Aricia artaxerxes|GBMIN16335-13|JX678100||Greece|657[0n]
Aricia artaxerxes|EULEP1385-15|RVcoll.14-F987||Greece|658[0n]
Aricia artaxerxes|PHLAB305-10|TLMF Lep 01105||Switzerland|639[0n]
Aricia artaxerxes|GWOSK797-11|BC ZSM Lep 49013||Germany|633[1n]
Aricia artaxerxes|EULEP3488-16|RVcoll15I715||Austria|658[0n]
Aricia artaxerxes|LEFIC063-10|MM03347||Finland|658[0n]
Aricia artaxerxes|FBLMW292-10|BC ZSM Lep 37393||Germany|658[0n]
Aricia artaxerxes|FBLMW290-10|BC ZSM Lep 37391||Germany|658[0n]
Aricia artaxerxes|GWOSU019-11|BC ZSM Lep 53270|externally misidentified as A. agestis|Germany|658...
Aricia artaxerxes|GWORL203-09|BC ZSM Lep 18609||Germany|658[0n]
Aricia artaxerxes|GWORL199-09|BC ZSM Lep 18605|ssp. allous|Germany|658[0n]
Aricia artaxerxes|GWORL197-09|BC ZSM Lep 18603||Germany|658[0n]
Aricia artaxerxes|LEATG096-14|TLMF Lep 13883||Austria|658[0n]
Aricia artaxerxes|PHLAW044-13|TLMF Lep 09841||Austria|658[0n]
Aricia artaxerxes|LEATG465-14|TLMF Lep 14252||Italy|658[0n]
Aricia artaxerxes|LEATG095-14|TLMF Lep 13882||Austria|658[0n]
Aricia artaxerxes|GWORA2463-09|BC ZSM Lep 30675||Austria|658[0n]
Aricia artaxerxes|FBLMX230-11|BC ZSM Lep 50441||Austria|658[0n]
Aricia artaxerxes|PHLAH703-12|TLMF Lep 08522||Austria|658[0n]
Aricia artaxerxes|FBLMX225-11|BC ZSM Lep 50436||Austria|658[0n]
Aricia artaxerxes|EULEP4832-16|RVcoll16H733||Ukraine|658[0n]
Aricia artaxerxes|EULEP4782-16|RVcoll16H377||Sweden|658[0n]
Aricia artaxerxes|FBLMW339-10|BC ZSM Lep 37440||Germany|658[0n]
Aricia artaxerxes|FBLMW337-10|BC ZSM Lep 37438||Germany|658[0n]
Aricia artaxerxes|FBLMX193-11|BC ZSM Lep 50404||Germany|658[0n]
Aricia artaxerxes|FBLMX219-11|BC ZSM Lep 50430||Germany|658[0n]
Aricia artaxerxes|FBLMX217-11|BC ZSM Lep 50428||Germany|658[0n]

[illegible]
Aricia artaxerxes|FBLMX193-11|BC ZSM Lep 50404||Germany|658[0n]
Aricia artaxerxes|FBLMX219-11|BC ZSM Lep 50430||Germany|658[0n]
Aricia artaxerxes|FBLMX217-11|BC ZSM Lep 50428||Germany|658[0n]
Aricia artaxerxes|FBLMX194-11|BC ZSM Lep 50405||Germany|658[0n]
Aricia artaxerxes|EULEP843-15|RVcoll.14-C034||Sweden|658[0n]
Aricia artaxerxes|EULEP166-14|RVcoll.11-I597||Italy|658[0n]
Aricia artaxerxes|LON821-11|NHMO Lep09019||Norway|658[0n]
Aricia artaxerxes|FBLMX234-11|BC ZSM Lep 50445||Switzerland|658[0n]
Aricia artaxerxes|EULEP2575-15|RVcoll.14-V553||Ukraine|658[0n]
Aricia artaxerxes|EULEP2350-15|RVcoll.14-V038||Ukraine|658[0n]
Aricia artaxerxes|LEASS743-17|TLMF Lep 22391||Austria|658[0n]
Aricia artaxerxes|LEASS736-17|TLMF Lep 22384||Austria|658[0n]
Aricia artaxerxes|GBMIN16204-13|JX678085||Russia|657[0n]
Aricia artaxerxes|LEASS836-17|TLMF Lep 22484||Austria|658[0n]
Aricia artaxerxes|LEFIJ514-10|MM17139||Finland|658[0n]
Aricia artaxerxes|LON218-08|NHMO-07007||Norway|657[0n]
Aricia artaxerxes|FBLMX213-11|BC ZSM Lep 50424||Germany|658[0n]
Aricia artaxerxes|GWOSK798-11|BC ZSM Lep 49014||Germany|658[0n]
Aricia artaxerxes|LEASS835-17|TLMF Lep 22483||Austria|658[0n]
Aricia artaxerxes|LEFIE772-10|MM09926||Finland|658[0n]
Aricia artaxerxes|FBLMX215-11|BC ZSM Lep 50426||Germany|658[0n]
Aricia artaxerxes|FBLMX221-11|BC ZSM Lep 50432||Austria|658[0n]
Aricia artaxerxes|FBLMX223-11|BC ZSM Lep 50434||Austria|658[0n]
Aricia artaxerxes|GBMIN16206-13|JX678081||Sweden|657[0n]
Aricia artaxerxes|GBMIN16205-13|JX678083||Russia|657[0n]
Aricia artaxerxes|GBMIN16219-13|JX678055||Norway|657[0n]
Aricia artaxerxes|GBMIN16341-13|JX678088||Sweden|657[0n]
Aricia artaxerxes|GBMIN16203-13|JX678087||Sweden|657[0n]
Aricia artaxerxes|GBMIN16344-13|JX678082||Sweden|657[0n]
Aricia artaxerxes|GBMIN16345-13|JX678080||Sweden|657[0n]
Aricia artaxerxes|GBMIN16346-13|JX678078||Estonia|657[0n]
Aricia artaxerxes|GBMIN16357-13|JX678056||Norway|657[0n]
Aricia artaxerxes|PHLAB323-10|TLMF Lep 01123||Switzerland|641[0n]
Aricia artaxerxes|FBLMW335-10|BC ZSM Lep 37436||Germany|658[0n]
Aricia artaxerxes|FBLMW332-10|BC ZSM Lep 37433|Identified by barcode; ssp. allous|Germany|658[0n]
Aricia artaxerxes|FBLMW311-10|BC ZSM Lep 37412||Austria|658[0n]
Aricia artaxerxes|FBLMW305-10|BC ZSM Lep 37406||Austria|658[0n]
Aricia artaxerxes|GWOSU027-11|BC ZSM Lep 53278|externally misidentified as A. agestis|Germany|658...
Aricia artaxerxes|GWOSU022-11|BC ZSM Lep 53273|externally misidentified as A. agestis|Germany|658...
Aricia artaxerxes|FBLMX228-11|BC ZSM Lep 50439||Austria|658[0n]
Aricia artaxerxes|FBLMX232-11|BC ZSM Lep 50443||Austria|658[0n]
Aricia artaxerxes|GBMIN16194-13|JX678105||France|657[0n]
Aricia artaxerxes|BIBSA363-15|RVcoll.14-I031||Italy|658[0n]
Aricia artaxerxes|BIBSA289-15|RVcoll.14-E089||Italy|658[0n]
Aricia artaxerxes|BIBSA288-15|RVcoll.14-E088||Italy|658[0n]
Aricia artaxerxes|BIBSA183-15|RVcoll.14-D971||Italy|658[0n]
Aricia artaxerxes|BIBSA182-15|RVcoll.14-D970||Italy|658[0n]
Aricia artaxerxes|BIBSA427-15|RVcoll.14-I095||Italy|658[0n]
Aricia artaxerxes|EULEP3481-16|RVcoll15H067||Italy|658[0n]
Aricia artaxerxes|BIBSA1342-15|15-M835||Italy|658[0n]
Aricia artaxerxes|WMB5251-14|RVcoll.14-A237||Italy|658[0n]
Aricia artaxerxes|WMB2032-13|RVcoll.12-Q732||Italy|658[0n]
Aricia artaxerxes|WMB2031-13|RVcoll.12-Q731||Italy|658[0n]
Aricia artaxerxes|LEASS1059-17|TLMF Lep 22612||Austria|658[0n]
Aricia artaxerxes|OXB730-15|13-U205||Italy|658[0n]
Aricia artaxerxes|OXB262-15|OXB-TGS-874||United Kingdom|658[0n]
Aricia artaxerxes|GBMIN16202-13|JX678089||Serbia|657[0n]
Aricia artaxerxes|GBMIN16220-13|JX678053||United Kingdom|657[0n]
Aricia artaxerxes|GBMIN16340-13|JX678090||Greece|657[0n]
Aricia artaxerxes|EULEP3486-16|RVcoll15H591||Switzerland|658[0n]
Aricia artaxerxes|GBMIN16359-13|JX678052||United Kingdom|657[0n]
Aricia artaxerxes|GBMIN16356-13|JX678058||United Kingdom|657[0n]
Aricia artaxerxes|GBMIN16358-13|JX678054||United Kingdom|657[0n]
Aricia artaxerxes|EZROM045-08|RV-07-E380|Genit. examined|Romania|658[0n]
Aricia artaxerxes|BIBSA1069-15|15-H937||Italy|658[0n]
Aricia artaxerxes|EULEP1860-15|RVcoll.13-U234||Italy|658[0n]
Aricia artaxerxes|EULEP1153-15|RVcoll.14-F346||Serbia|658[0n]
Aricia artaxerxes|EULEP3491-16|RVcoll15H994||Italy|658[0n]
Aricia artaxerxes|EULEP3490-16|RVcoll15H207||Switzerland|658[0n]
Aricia artaxerxes|EULEP3489-16|RVcoll15Q000||Macedonia|658[0n]
Aricia artaxerxes|EULEP3487-16|RVcoll15I217||Austria|658[0n]
Aricia artaxerxes|WMB4022-14|RVcoll.12-R518||United Kingdom|658[0n]
Aricia artaxerxes|EZROM050-08|RV-07-C201|Genit. examined|Romania|658[0n]
Aricia artaxerxes|EZROM049-08|RV-07-E576|Genit. examined|Romania|658[0n]
Aricia artaxerxes|EZROM047-08|RV-07-E582|Genit. examined|Romania|658[0n]
Aricia artaxerxes|EZROM046-08|RV-07-E580|Genit. examined|Romania|658[0n]
Aricia artaxerxes|EZROM792-08|RVcoll.08-M628|Genit. examined|Romania|658[0n]
Aricia artaxerxes|EZROM791-08|RVcoll.08-M627|Genit. examined|Romania|658[0n]
Aricia artaxerxes|EZROM602-08|RV-07-E579|Genit. examined|Romania|658[0n]
Aricia artaxerxes|EZRMN213-08|RVcoll.08-M552|Genit. examined|Romania|658[0n]
Aricia artaxerxes|LEATD140-13|TLMF Lep 12787||Italy|658[0n]
Aricia artaxerxes|LEATD459-13|TLMF Lep 13106||Italy|658[0n]
Aricia artaxerxes|PHLAB367-10|TLMF Lep 01167||Switzerland|658[0n]
Aricia nicias|EZSPM554-10|RVcoll.09-V887||Spain|619[0n]
Aricia nicias|EZSPM555-10|RVcoll.09-V889||Spain|623[0n]
Aricia nicias|EZSPM556-10|RVcoll.09-V890||Spain|653[0n]
Aricia nicias|EZSPM553-10|RVcoll.09-V886||Spain|658[0n]
Aricia nicias|EZSPM552-10|RVcoll.09-V885||Spain|658[0n]
Aricia nicias|EZSPM551-10|RVcoll.09-V884||Spain|658[0n]
Aricia nicias|EZSPM643-12|RVcoll.11-H246||Spain|658[0n]
Aricia nicias|EZSPM642-12|RVcoll.11-H245||Spain|658[0n]
Aricia nicias|EZSPM641-12|RVcoll.11-H244||Spain|658[0n]
Aricia nicias|BIBSA364-15|RVcoll.14-I032||Italy|658[0n]
Aricia nicias|BIBSA271-15|RVcoll.14-E070||Italy|638[0n]
Aricia nicias|WMB3528-14|RVcoll.10-C084||France|658[0n]
Aricia nicias|LENOA1443-11|LN-BD1437||France|658[0n]
Aricia nicias|WMB1605-13|RVcoll.12-O669||France|658[0n]
Aricia nicias|BIBSA143-15|RVcoll.14-I156||Italy|658[0n]
Aricia nicias|PHLAF619-11|TLMF Lep 05789||France|658[0n]
Aricia nicias|WMB2668-13|RVcoll.10-B835||France|658[0n]
Aricia nicias|WMB2669-13|RVcoll.10-B836||France|658[0n]
Aricia nicias|WMB2670-13|RVcoll.10-B841||France|658[0n]
[illegible]

Aricia nicias|WMB2668-13|RVcoll.10-B835||France|658[0n]
Aricia nicias|WMB2669-13|RVcoll.10-B836||France|658[0n]
Aricia nicias|WMB2670-13|RVcoll.10-B841||France|658[0n]
Aricia nicias|WMB2671-13|RVcoll.10-B842||France|658[0n]
Aricia nicias|PHLAF618-11|TLMF Lep 05788||France|658[0n]
Aricia nicias|EULEP3090-15|RVcoll.14-O223||Sweden|658[0n]
Aricia nicias|EULEP4797-16|RVcoll16H587||Sweden|658[0n]
Aricia nicias|EULEP4796-16|RVcoll16H567||Sweden|658[0n]
Aricia nicias|LEFID248-10|MM06112||Finland|658[0n]
Aricia nicias|LON803-11|NHMO Lep09001||Norway|658[0n]
Aricia nicias|GBMIN16197-13|JX678099||Sweden|657[0n]
Aricia nicias|LEFIC059-10|MM03341||Finland|658[0n]
Aricia nicias|LEFIG534-10|MM14661||Finland|658[0n]
Aricia nicias|EULEP3492-16|RVcoll15H679||Switzerland|658[0n]
Aricia nicias|PHLAB1019-10|TLMF Lep 01819||Switzerland|658[0n]
Aricia nicias|PHLAB1020-10|TLMF Lep 01820||Switzerland|658[0n]
Aricia nicias|PHLAB1021-10|TLMF Lep 01821||Switzerland|658[0n]
Aricia nicias|PHLAC716-10|TLMF Lep 02751||Switzerland|658[0n]
Aricia cramera|WMB6398-18|RVcoll07C472|Genit. examined|Spain|658[0n]
Aricia cramera|WMB6503-18|RVcoll12Q538|Genit. examined|Spain|658[0n]
Aricia cramera|EZSPM891-12|Rvcoll.12-M610||Spain|658[0n]
Aricia cramera|WMB6514-18|RVcoll12Q549||Spain|658[0n]
Aricia cramera|WMB6425-18|RVcoll08L504|Genit. examined|Spain|658[0n]
Aricia cramera|WMB6395-18|RVcoll07C407|Genit. examined|Spain|658[0n]
Aricia cramera|WMB6368-18|RVcoll06A217|Genit. examined|Spain|658[0n]
Aricia cramera|WMB6367-18|RVcoll06A216|Genit. examined|Spain|658[0n]
Aricia cramera|WMB6365-18|RVcoll06A211|Genit. examined|Spain|658[0n]
Aricia cramera|WMB6358-18|RVcoll06A148|Genit. examined|Spain|658[0n]
Aricia cramera|WMB4043-14|RVcoll.12-Z361||Spain|658[0n]
Aricia cramera|WMB6505-18|RVcoll12Q540|Genit. examined|Spain|658[0n]
Aricia cramera|GBMIN16225-13|JX678043||Spain|657[0n]
Aricia cramera|GBGL18273-15|KM459135|Sardinia|Italy|655[0n]
Aricia cramera|GBMIN16310-13|JX678150||Spain|657[0n]
Aricia cramera|WMB4412-14|RVcoll.14-B453||Portugal|658[0n]
Aricia cramera|WMB6380-18|RVcoll06G524|Genit. examined|Spain|658[0n]
Aricia cramera|WMB3343-14|RVcoll.08-P926||Spain|658[0n]
Aricia cramera|WMB2994-14|RVcoll.12-O399|San Pietro|Italy|658[0n]
Aricia cramera|WMB3163-14|RVcoll.08-H417||Spain|658[0n]
Aricia cramera|WMB3169-14|RVcoll.08-H517||Spain|658[0n]
Aricia cramera|WMB3206-14|RVcoll.08-J214||Spain|658[0n]
Aricia cramera|WMB3219-14|RVcoll.08-J722||Spain|658[0n]
Aricia cramera|WMB3260-14|RVcoll.08-L661||Spain|658[0n]
Aricia cramera|WMB4508-14|RVcoll.14-E196||Spain|658[0n]
Aricia cramera|WMB4380-14|RVcoll.14-B231||Portugal|658[0n]
Aricia cramera|WMB4410-14|RVcoll.14-B448||Portugal|658[0n]
Aricia cramera|WMB4427-14|RVcoll.14-B487||Portugal|658[0n]
Aricia cramera|WMB6411-18|RVcoll07Z065|Genit. examined|Spain|658[0n]
Aricia cramera|WMB6412-18|RVcoll07Z068|Genit. examined|Spain|658[0n]
Aricia cramera|WMB6413-18|RVcoll07Z070|Genit. examined|Spain|658[0n]
Aricia cramera|WMB6415-18|RVcoll07Z079|Genit. examined|Spain|658[0n]
Aricia cramera|WMB6416-18|RVcoll07Z080|Genit. examined|Spain|658[0n]
Aricia cramera|WMB6418-18|RVcoll08J397|Genit. examined|Spain|658[0n]
Aricia cramera|WMB6421-18|RVcoll08L467|Genit. examined|Spain|658[0n]
Aricia cramera|WMB6424-18|RVcoll08L503|Genit. examined|Spain|658[0n]
Aricia cramera|WMB6460-18|RVcoll09X322|Genit. examined|Spain|658[0n]
Aricia cramera|WMB6475-18|RVcoll12N699|Genit. examined|Spain|658[0n]
Aricia cramera|WMB6484-18|RVcoll12Q438|Genit. examined|Spain|658[0n]
Aricia cramera|WMB6488-18|RVcoll12Q502|Genit. examined|Spain|658[0n]
Aricia cramera|WMB6489-18|RVcoll12Q503|Genit. examined|Spain|658[0n]
Aricia cramera|WMB6490-18|RVcoll12Q504|Genit. examined|Spain|658[0n]
Aricia cramera|WMB6492-18|RVcoll12Q506||Spain|658[0n]
Aricia cramera|WMB6493-18|RVcoll12Q522|Genit. examined|Spain|658[0n]
Aricia cramera|WMB6494-18|RVcoll12Q523|Genit. examined|Spain|658[0n]
Aricia cramera|WMB6496-18|RVcoll12Q528|Genit. examined|Spain|658[0n]
Aricia cramera|WMB6497-18|RVcoll12Q532|Genit. examined|Spain|658[0n]
Aricia cramera|WMB6498-18|RVcoll12Q533|Genit. examined|Spain|658[0n]
Aricia cramera|WMB6499-18|RVcoll12Q534|Genit. examined|Spain|658[0n]
Aricia cramera|WMB6500-18|RVcoll12Q535|Genit. examined|Spain|658[0n]
Aricia cramera|WMB6501-18|RVcoll12Q536||Spain|658[0n]
Aricia cramera|WMB6502-18|RVcoll12Q537|Genit. examined|Spain|658[0n]
Aricia cramera|WMB6506-18|RVcoll12Q541|Genit. examined|Spain|658[0n]
Aricia cramera|WMB6507-18|RVcoll12Q542|Genit. examined|Spain|658[0n]
Aricia cramera|WMB6508-18|RVcoll12Q543||Spain|658[0n]
Aricia cramera|WMB6509-18|RVcoll12Q544||Spain|658[0n]
Aricia cramera|WMB6512-18|RVcoll12Q547||Spain|658[0n]
Aricia cramera|WMB6513-18|RVcoll12Q548||Spain|658[0n]
Aricia cramera|WMB6515-18|RVcoll12Q550||Spain|658[0n]
Aricia cramera|WMB6516-18|RVcoll12Q553||Spain|658[0n]
Aricia cramera|WMB6517-18|RVcoll12Q555||Spain|658[0n]
Aricia cramera|WMB6518-18|RVcoll12Q556||Spain|658[0n]
Aricia cramera|WMB6519-18|RVcoll12Q557|Genit. examined|Spain|657[0n]
Aricia cramera|WMB6359-18|RVcoll06A150|Genit. examined|Spain|658[0n]
Aricia cramera|WMB6361-18|RVcoll06A152|Genit. examined|Spain|658[0n]
Aricia cramera|WMB6363-18|RVcoll06A203|Genit. examined|Spain|658[0n]
Aricia cramera|WMB6364-18|RVcoll06A207|Genit. examined|Spain|658[0n]
Aricia cramera|WMB6366-18|RVcoll06A212|Genit. examined|Spain|658[0n]
Aricia cramera|WMB6369-18|RVcoll06A223|Genit. examined|Spain|658[0n]
Aricia cramera|WMB6373-18|RVcoll06A231|Genit. examined|Spain|658[0n]
Aricia cramera|WMB6375-18|RVcoll06A236|Genit. examined|Spain|658[0n]
Aricia cramera|WMB6376-18|RVcoll06A237|Genit. examined|Spain|658[0n]
Aricia cramera|WMB6381-18|RVcoll06G525|Genit. examined|Spain|658[0n]
Aricia cramera|WMB6382-18|RVcoll06G554|Genit. examined|Spain|658[0n]
Aricia cramera|WMB6383-18|RVcoll06G568|Genit. examined|Spain|658[0n]
Aricia cramera|WMB6385-18|RVcoll06G662|Genit. examined|Spain|658[0n]
Aricia cramera|WMB6390-18|RVcoll07C285|Genit. examined|Spain|658[0n]
Aricia cramera|WMB6391-18|RVcoll07C286|Genit. examined|Spain|658[0n]
Aricia cramera|WMB6392-18|RVcoll07C287|Genit. examined|Spain|658[0n]
Aricia cramera|WMB6394-18|RVcoll07C289|Genit. examined|Spain|658[0n]
Aricia cramera|WMB6397-18|RVcoll07C471|Genit. examined|Spain|658[0n]
Aricia cramera|WMB6399-18|RVcoll07C683|Genit. examined|Spain|658[0n]
Aricia cramera|WMB6408-18|RVcoll07F501|Genit. examined|Spain|658[0n]
Aricia cramera|WMB6410-18|RVcoll07Z064|Genit. examined|Spain|658[0n]
Aricia cramera|WMB6522-18|RVcoll12Q560|Genit. examined|Spain|658[0n]

Aricia cramera|WMB6408-18|RVcoll07F501|Genit. examined|Spain|658[0n]
Aricia cramera|WMB6410-18|RVcoll07Z064|Genit. examined|Spain|658[0n]
Aricia cramera|WMB6522-18|RVcoll12Q560|Genit. examined|Spain|658[0n]
Aricia cramera|WMB6523-18|RVcoll12Q561|Genit. examined|Spain|658[0n]
Aricia cramera|EULEP147-14|RVcoll.10-C442||Spain|658[0n]
Aricia cramera|BIBSA1283-15|13-T679|Sardinia|Italy|658[0n]
Aricia cramera|OXB1242-15|Rvcoll_13-T689|Sardinia|Italy|658[0n]
Aricia cramera|GBMIN16231-13|JX678031||Spain|657[0n]
Aricia cramera|GBMIN16367-13|JX678036|Sardinia|Italy|657[0n]
Aricia cramera|GBMIN16371-13|JX678028||Spain|657[0n]
Aricia cramera|GBMIN16229-13|JX678035|Mallorca|Spain|657[0n]
Aricia cramera|GBGL18244-15|KM459105||Spain|655[0n]
Aricia cramera|GBGL18245-15|KM459106||Spain|655[0n]
Aricia cramera|GBGL18246-15|KM459107|Menorca|Spain|655[0n]
Aricia cramera|GBGL18247-15|KM459108|Menorca|Spain|655[0n]
Aricia cramera|GBGL18249-15|KM459110||Spain|655[0n]
Aricia cramera|GBGL18253-15|KM459114|Mallorca|Spain|655[0n]
Aricia cramera|GBGL18256-15|KM459117||Spain|655[0n]
Aricia cramera|GBGL18257-15|KM459118||Spain|655[0n]
Aricia cramera|GBGL18258-15|KM459119||Spain|655[0n]
Aricia cramera|GBGL18259-15|KM459121|Ibiza|Spain|655[0n]
Aricia cramera|GBGL18260-15|KM459122|Ibiza|Spain|655[0n]
Aricia cramera|GBGL18263-15|KM459125||Spain|655[0n]
Aricia cramera|GBGL18264-15|KM459126||Spain|655[0n]
Aricia cramera|GBGL18265-15|KM459127|Sardinia|Italy|655[0n]
Aricia cramera|GBGL18266-15|KM459128|Sardinia|Italy|655[0n]
Aricia cramera|GBGL18267-15|KM459129|Sardinia|Italy|655[0n]
Aricia cramera|GBGL18268-15|KM459130|Sardinia|Italy|655[0n]
Aricia cramera|GBGL18269-15|KM459131|Sardinia|Italy|655[0n]
Aricia cramera|GBGL18272-15|KM459134|Sardinia|Italy|655[0n]
Aricia cramera|GBGL18283-15|KM459149|Menorca|Spain|655[0n]
Aricia cramera|GBGL18285-15|KM459151|Sardinia|Italy|655[0n]
Aricia cramera|GBMIN16227-13|JX678039||Spain|657[0n]
Aricia cramera|WMB6521-18|RVcoll12Q559|Genit. examined|Spain|658[0n]
Aricia cramera|WMB6520-18|RVcoll12Q558|Genit. examined|Spain|658[0n]
Aricia cramera|GBGL18288-15|KM459154||Spain|655[0n]
Aricia cramera|GBGL18289-15|KM459155||Spain|655[0n]
Aricia cramera|GBGL18295-15|KM459161||Spain|655[0n]
Aricia cramera|WMB6495-18|RVcoll12Q524|Genit. examined|Spain|658[1n]
Aricia cramera|GBGL18287-15|KM459153||Spain|655[0n]
Aricia cramera|GBGL18290-15|KM459156||Spain|655[0n]
Aricia cramera|GBGL18291-15|KM459157||Spain|655[0n]
Aricia cramera|GBGL18292-15|KM459158||Spain|655[0n]
Aricia cramera|GBGL18299-15|KM459165||Spain|655[0n]
Aricia cramera|GBGL18293-15|KM459159||Spain|655[0n]
Aricia cramera|GBGL18294-15|KM459160||Spain|655[0n]
Aricia cramera|GBGL18296-15|KM459162||Spain|655[0n]
Aricia cramera|GBGL18298-15|KM459164||Spain|655[0n]
Aricia cramera|GBGL18309-15|KM459175|Asinara|Italy|655[0n]
Aricia cramera|GBMIN16226-13|JX678041|Sardinia|Italy|657[0n]
Aricia cramera|GBGL18300-15|KM459166||Spain|655[0n]
Aricia cramera|GBGL18308-15|KM459174|Asinara|Italy|655[0n]
Aricia cramera|WMB6491-18|RVcoll12Q505|Genit. examined|Spain|658[0n]
Aricia cramera|GBGL18301-15|KM459167||Spain|655[0n]
Aricia cramera|GBGL18305-15|KM459171||Spain|655[0n]
Aricia cramera|GBMIN16365-13|JX678040|Sardinia|Italy|657[0n]
Aricia cramera|GBGL18284-15|KM459150|Sardinia|Italy|655[0n]
Aricia cramera|GBGL18270-15|KM459132|Sardinia|Italy|655[0n]
Aricia cramera|GBGL18302-15|KM459168||Spain|655[0n]
Aricia cramera|GBGL18306-15|KM459172|Asinara|Italy|655[0n]
Aricia cramera|GBGL18248-15|KM459109|Menorca|Spain|655[0n]
Aricia cramera|GBMIN16368-13|JX678034|Mallorca|Spain|657[0n]
Aricia cramera|GBGL18255-15|KM459116|Mallorca|Spain|655[0n]
Aricia cramera|GBGL18254-15|KM459115|Mallorca|Spain|655[0n]
Aricia cramera|GBGL18262-15|KM459124|Ibiza|Spain|655[0n]
Aricia cramera|GBGL18261-15|KM459123|Ibiza|Spain|655[0n]
Aricia cramera|GBMIN16230-13|JX678033|Menorca|Spain|657[0n]
Aricia cramera|GBGL18304-15|KM459170||Spain|655[0n]
Aricia cramera|GBGL18271-15|KM459133|Sardinia|Italy|655[0n]
Aricia cramera|GBGL18303-15|KM459169||Spain|655[0n]
Aricia cramera|GBGL18307-15|KM459173|Asinara|Italy|655[0n]
Aricia cramera|GBGL18286-15|KM459152|Sant Antioco|Italy|655[0n]
Aricia cramera|GBGL18297-15|KM459163||Spain|655[0n]
Aricia cramera|WMB6511-18|RVcoll12Q546||Spain|658[0n]
Aricia cramera|GBMIN16369-13|JX678032||Spain|657[0n]
Aricia cramera|GBMIN16370-13|JX678030||Spain|657[0n]
Aricia cramera|WMB3250-14|RVcoll.08-L289||Spain|621[0n]
Aricia cramera|GBGL18250-15|KM459111||Spain|619[0n]
Aricia cramera|GBMIN16373-13|JX678024||Spain|646[0n]
Aricia cramera|GBMIN16372-13|JX678026|Menorca|Spain|646[0n]
Aricia cramera|GBMIN16235-13|JX678023||Spain|646[0n]
Aricia cramera|GBMIN16224-13|JX678045||Spain|646[0n]
Aricia cramera|GBMIN16363-13|JX678044||Spain|646[0n]
Aricia cramera|GBMIN16361-13|JX678048||Spain|646[0n]
Aricia cramera|GBMIN16234-13|JX678025||Spain|646[0n]
Aricia cramera|GBMIN16222-13|JX678049||Spain|646[1n]
Aricia cramera|GBMIN16360-13|JX678050||Spain|646[1n]
Aricia cramera|WMB3342-14|RVcoll.08-P925||Spain|658[0n]
Aricia cramera|EZSPC753-10|RVcoll.06-A205||Spain|658[0n]
Aricia cramera|EZSPC706-10|RVcoll.08-H635||Spain|658[0n]
Aricia cramera|EZSPC705-10|RVcoll.09-V439||Spain|658[0n]
Aricia cramera|EZSPM943-12|Rvcoll.12-M678||Spain|658[0n]
Aricia cramera|EZSPM912-12|Rvcoll.12-M634||Spain|658[0n]
Aricia cramera|EZSPM892-12|Rvcoll.12-M611||Spain|658[0n]
Aricia cramera|EZSPC756-10|RVcoll.06-G526|Genit. examined|Spain|658[0n]
Aricia cramera|EZSPM1025-12|Rvcoll.12-M775||Spain|658[0n]
Aricia cramera|EZSPM1022-12|Rvcoll.12-M771||Spain|658[0n]
Aricia cramera|EZSPM967-12|Rvcoll.12-M719||Spain|658[0n]
Aricia cramera|EZSPN703-09|RVcoll.08-J884||Spain|658[0n]
Aricia cramera|EZROM707-08|RV-06-A215|Genit. examined|Spain|658[0n]
Aricia cramera|WMB6409-18|RVcoll07F502|Genit. examined|Spain|658[0n]
Aricia cramera|WMB6396-18|RVcoll07C413|Genit. examined|Spain|658[1n]

Aricia cramera|EZROM707-08|RV-06-A215|Genit. examined|Spain|658[0n]
Aricia cramera|WMB6409-18|RVcoll07F502|Genit. examined|Spain|658[0n]
Aricia cramera|WMB6396-18|RVcoll07C413|Genit. examined|Spain|658[1n]
Aricia cramera|WMB6374-18|RVcoll06A232|Genit. examined|Spain|658[0n]
Aricia cramera|WMB6423-18|RVcoll08L502|Genit. examined|Spain|658[0n]
Aricia cramera|EZSPM1036-12|Rvcoll.12-M787||Spain|643[0n]
Aricia cramera|EZSPM899-12|Rvcoll.12-M621||Spain|634[0n]
Aricia cramera|WMB6384-18|RVcoll06G661|Genit. examined|Spain|658[1n]
Aricia cramera|EZSPM668-12|Rvcoll. 12-L566||Portugal|621[0n]
Aricia cramera|EZSPM667-12|Rvcoll. 12-L547||Portugal|620[0n]
Aricia anteros|EULEP2227-15|RVcoll.14-N406||Ukraine|658[0n]
Aricia anteros|EULEP2409-15|RVcoll.14-V121||Russia|658[0n]
Aricia anteros|EULEP2408-15|RVcoll.14-V120||Russia|658[0n]
Aricia anteros|GBMIN16170-13|JX678153||Serbia|657[0n]
Aricia anteros|EULEP1809-15|RVcoll.14-H764||Greece|658[0n]
Aricia anteros|EULEP1808-15|RVcoll.14-H763||Greece|658[0n]
Aricia anteros|GBMIN16308-13|JX678154||Bulgaria|657[0n]
Aricia anteros|EULEP1683-15|RVcoll.14-G991||Greece|658[0n]
Aricia anteros|EULEP1828-15|RVcoll.14-J949||Albania|658[0n]
Aricia anteros|EULEP1826-15|RVcoll.14-J946||Bulgaria|658[0n]
Aricia anteros|EULEP1825-15|RVcoll.14-J945||Bulgaria|658[0n]
Aricia anteros|EULEP1226-15|RVcoll.14-F529||Greece|658[0n]
Aricia anteros|EULEP3483-16|RVcoll10B369||Bulgaria|658[0n]
Aricia morronensis|WMB6435-18|RVcoll08P671||Spain|658[0n]
Aricia morronensis|WMB6440-18|RVcoll09V174||Spain|658[0n]
Aricia morronensis|WMB6439-18|RVcoll09V173||Spain|658[0n]
Aricia morronensis|EZSPC1315-10|RVcoll.130209KL8||Spain|658[0n]
Aricia morronensis|WMB6430-18|RVcoll08L886||Spain|658[0n]
Aricia morronensis|EZSPC751-10|RVcoll.08-L889||Spain|658[0n]
Aricia morronensis|EZSPN931-09|RVcoll.08-L887||Spain|658[0n]
Aricia morronensis|WMB6434-18|RVcoll08P670||Spain|658[0n]
Aricia morronensis|WMB6438-18|RVcoll09V172||Spain|658[0n]
Aricia morronensis|WMB6437-18|RVcoll09V171||Spain|658[0n]
Aricia morronensis|WMB6441-18|RVcoll09V175||Spain|658[0n]
Aricia morronensis|WMB6442-18|RVcoll09V176||Spain|658[0n]
Aricia morronensis|GBMIN16240-13|JX678013||Spain|657[0n]
Aricia morronensis|GBMIN16378-13|JX678014||Spain|657[0n]
Aricia morronensis|WMB6417-18|RVcoll08H975||Spain|658[0n]
Aricia morronensis|EZSPC746-10|RVcoll.08-H973||Spain|658[0n]
Aricia morronensis|EZSPN513-09|RVcoll.08-H974||Spain|658[0n]
Aricia morronensis|EZSPC717-10|RVcoll.09-X041||Spain|658[0n]
Aricia morronensis|EZSPC712-10|RVcoll.09-X040||Spain|658[0n]
Aricia morronensis|EZSPN785-09|RVcoll.08-L328|Genit. examined|Spain|658[0n]
Aricia morronensis|WMB6444-18|RVcoll09V179||Spain|658[0n]
Aricia morronensis|WMB6443-18|RVcoll09V178||Spain|658[0n]
Aricia morronensis|WMB6386-18|RVcoll070207MC31||Spain|658[0n]
Aricia morronensis|EZSPC1301-10|RVcoll.171106RP4||Spain|658[0n]
Aricia morronensis|EZSPC1276-10|RVcoll.171106RP5||Spain|658[0n]
Aricia morronensis|EZSPC1265-10|RVcoll.070207MC36||Spain|658[0n]
Aricia morronensis|WMB6447-18|RVcoll09V424||Spain|658[0n]
Aricia morronensis|WMB6446-18|RVcoll09V423||Spain|658[0n]
Aricia morronensis|WMB6445-18|RVcoll09V422||Spain|658[0n]
Aricia morronensis|GBMIN16238-13|JX678017||Spain|657[0n]
Aricia morronensis|EZSPN077-09|RVcoll.09-V556||Spain|658[0n]
Aricia morronensis|EZSPN076-09|RVcoll.09-V516||Spain|658[0n]
Aricia morronensis|EZSPN676-09|RVcoll.08-J988||Spain|658[0n]
Plebejidea loewii|EULEP666-15|RVcoll.11-J498|Tilos|Greece|658[0n]
Plebejidea loewii|EULEP3050-15|RVcoll.14-O183|Rhodes|Greece|658[0n]
Plebejidea loewii|EULEP661-15|RVcoll.11-J474|Kos|Greece|658[0n]
Plebejidea loewii|EULEP660-15|RVcoll.11-J473|Kos|Greece|658[0n]
Plebejidea loewii|EULEP668-15|RVcoll.11-J500|Tilos|Greece|658[0n]
Plebejidea loewii|EULEP667-15|RVcoll.11-J499|Tilos|Greece|658[0n]
Plebejidea loewii|EULEP659-15|RVcoll.11-J472|Kos|Greece|658[0n]
Kretania hesperica|EZSPN166-09|RVcoll.06-K690||Spain|658[1n]
Kretania hesperica|EZSPC1132-10|RVcoll.08-P710||Spain|658[0n]
Kretania hesperica|EZSPN355-09|RVcoll.08-H330||Spain|658[0n]
Kretania hesperica|EZSPN699-09|RVcoll.08-L042||Spain|633[0n]
Kretania hesperica|EZSPC1008-10|RVcoll.08-H466||Spain|658[0n]
Kretania hesperica|EZSPN399-09|RVcoll.08-H465||Spain|658[0n]
Kretania hesperica|EZSPN708-09|RVcoll.08-L057||Spain|658[0n]
Kretania hesperica|EZSPN707-09|RVcoll.08-L056||Spain|658[0n]
Kretania hesperica|EZSPN165-09|RVcoll.06-K689||Spain|658[0n]
Kretania hesperica|WMB4562-14|RVcoll.090211SD77||Spain|642[0n]
Kretania hesperica|EZSPN717-09|RVcoll.08-L095|Genit. examined|Spain|658[0n]
Kretania hesperica|EZSPN715-09|RVcoll.08-L091||Spain|658[0n]
Kretania eurypilus|EULEP2583-15|RVcoll.14-V589||Russia|658[0n]
Kretania eurypilus|EULEP2582-15|RVcoll.14-V588||Russia|658[0n]
Kretania eurypilus|EULEP1400-15|RVcoll.14-G043||Greece|658[0n]
Kretania eurypilus|EULEP1396-15|RVcoll.14-G019||Greece|658[0n]
Kretania eurypilus|EULEP1393-15|RVcoll.14-G008||Greece|658[0n]
Kretania eurypilus|EULEP1389-15|RVcoll.14-F996||Greece|614[0n]
Kretania eurypilus|EULEP3047-15|RVcoll.14-O180|Samos|Greece|658[0n]
Kretania eurypilus|EULEP5115-17|RVcoll16J870|Samos|Greece|658[0n]
Kretania sephirus|EULEP1330-15|RVcoll.14-F810||Greece|658[0n]
Kretania sephirus|EULEP1321-15|RVcoll.14-F788||Greece|658[0n]
Kretania sephirus|EULEP5120-17|RVcoll16J875||Greece|658[0n]
Kretania sephirus|EULEP2029-15|RVcoll.14-J504||Ukraine|658[0n]
Kretania sephirus|EULEP5119-17|RVcoll16J874||Macedonia|658[0n]
Kretania sephirus|EULEP1627-15|RVcoll.14-G729||Greece|658[0n]
Kretania sephirus|EULEP3056-15|RVcoll.14-O189||Albania|658[0n]
Kretania sephirus|EULEP3057-15|RVcoll.14-O190||Macedonia|658[0n]
Kretania sephirus|EULEP1684-15|RVcoll.14-G994||Greece|658[0n]
Kretania sephirus|EULEP1682-15|RVcoll.14-G988||Greece|614[0n]
Kretania sephirus|EULEP1166-15|RVcoll.14-F373||Bulgaria|658[0n]
Kretania sephirus|EULEP1165-15|RVcoll.14-F372||Bulgaria|658[0n]
Kretania sephirus|EULEP4114-16|RVcoll15Q001||Macedonia|658[0n]
Kretania sephirus|EULEP4115-16|RVcoll14A945||Russia|658[0n]
Kretania sephirus|EULEP2048-15|RVcoll.14-J530||Ukraine|658[0n]
Kretania sephirus|EULEP2044-15|RVcoll.14-J525||Ukraine|658[0n]
Kretania pylaon|EULEP431-14|KN00793||Russia|658[0n]
Kretania sephirus|EULEP4116-16|RVcoll14A946||Russia|658[0n]
Kretania pylaon|EULEP3054-15|RVcoll.14-O187||Russia|658[0n]
Kretania sephirus|EULEP2045-15|RVcoll.14-J526||Ukraine|658[0n]

Kretania sephirus|EULEP4116-16|RVcoll14A946||Russia|658[0n]
Kretania pylaon|EULEP3054-15|RVcoll.14-O187||Russia|658[0n]
Kretania sephirus|EULEP2045-15|RVcoll.14-J526||Ukraine|658[0n]
Kretania sephirus|EULEP2046-15|RVcoll.14-J527||Ukraine|658[0n]
Kretania sephirus|EULEP2047-15|RVcoll.14-J529||Ukraine|658[0n]
Kretania sephirus|EULEP2288-15|RVcoll.14-N486||Ukraine|658[0n]
Kretania pylaon|EULEP3055-15|RVcoll.14-O188||Russia|658[0n]
Kretania sephirus|EULEP2503-15|RVcoll.14-V368||Ukraine|624[0n]
Kretania pylaon|EULEP432-14|KN00794||Russia|658[0n]
Kretania pylaon|EULEP430-14|KN00792||Russia|658[0n]
Kretania sephirus|EULEP4107-16|RVcoll14V603||Russia|658[0n]
Kretania sephirus|EZRMN199-08|RVcoll.08-M288||Romania|658[0n]
Kretania sephirus|EZRMN198-08|RVcoll.08-M287||Romania|658[0n]
Kretania sephirus|EZRMN197-08|RVcoll.08-M286||Romania|658[0n]
Kretania sephirus|EZRMN196-08|RVcoll.08-M285||Romania|658[0n]
Kretania sephirus|EZRMN195-08|RVcoll.08-M284||Romania|658[0n]
Kretania sephirus|EZROM160-08|RV-07-D404|Genit. examined|Romania|658[0n]
Kretania sephirus|EULEP2478-15|RVcoll.14-V335||Ukraine|614[0n]
Kretania sephirus|EZROM490-08|RV-07-D428||Romania|658[0n]
Kretania sephirus|EZROM491-08|RV-07-D436||Romania|658[0n]
Kretania trappi|EULEP4113-16|RVcoll15G495||Switzerland|632[0n]
Kretania trappi|EULEP4112-16|RVcoll15G482||Switzerland|658[0n]
Kretania trappi|EULEP4111-16|RVcoll15G472||Switzerland|658[0n]
Kretania trappi|EULEP4110-16|RVcoll15H144||Italy|658[0n]
Kretania trappi|EULEP4109-16|RVcoll15H141||Italy|658[0n]
Kretania trappi|EULEP4108-16|RVcoll15H137||Italy|658[0n]
Kretania trappi|LEATG566-14|TLMF Lep 14353||Italy|658[0n]
Kretania trappi|LEATG565-14|TLMF Lep 14352||Italy|658[0n]
Agriades dardanus|EULEP2636-15|RVcoll.14-N576||Bosnia and Herzegovina|658[0n]
Agriades dardanus|EULEP2635-15|RVcoll.14-N575||Bosnia and Herzegovina|658[0n]
Agriades dardanus|EULEP2348-15|RVcoll.14-V034||Ukraine|626[0n]
Agriades dardanus|EULEP2031-15|RVcoll.14-J509||Ukraine|617[0n]
Agriades dardanus|EULEP2347-15|RVcoll.14-V033||Ukraine|658[0n]
Agriades dardanus|EULEP2346-15|RVcoll.14-V032||Ukraine|658[0n]
Agriades dardanus|EULEP2345-15|RVcoll.14-V031||Ukraine|658[0n]
Agriades dardanus|EULEP2639-15|RVcoll.14-N579||Greece|658[0n]
Agriades dardanus|EULEP2638-15|RVcoll.14-N578||Greece|658[0n]
Agriades dardanus|EULEP2637-15|RVcoll.14-N577||Greece|658[0n]
Agriades dardanus|EULEP2634-15|RVcoll.14-N574||Bulgaria|658[0n]
Agriades dardanus|EULEP2633-15|RVcoll.14-N573||Bulgaria|658[0n]
Agriades dardanus|EULEP2287-15|RVcoll.14-N513||Greece|658[0n]
Agriades dardanus|EULEP3404-16|RVcoll14A966||Bulgaria|658[0n]
Agriades dardanus|EULEP3403-16|RVcoll14A965||Bulgaria|658[0n]
Agriades dardanus|EULEP3402-16|RVcoll14A964||Bulgaria|658[0n]
Agriades dardanus|EULEP3401-16|RVcoll14A963||Bulgaria|658[0n]
Agriades pyrenaicus|EZSPC882-10|RVcoll.09-T143||Spain|658[0n]
Agriades pyrenaicus|EZSPN1084-11|Rvcoll.09-X298||Spain|658[0n]
Agriades pyrenaicus|EZSPN269-09|RVcoll.07-W186||Spain|658[0n]
Agriades pyrenaicus|EZSPN1083-11|Rvcoll.09-X297||Spain|658[0n]
Agriades pyrenaicus|EZSPN268-09|RVcoll.07-W185||Spain|658[0n]
Agriades pyrenaicus|EZSPN237-09|RVcoll.08-J811||Spain|658[0n]
Eumedonia eumedon|EULEP524-15|RVcoll.08-H803||Sweden|658[0n]
Eumedonia eumedon|EULEP5622-17|RVcoll14V740||Spain|658[0n]
Eumedonia eumedon|EULEP5620-17|RVcoll14V738||Spain|658[0n]
Eumedonia eumedon|EULEP5617-17|RVcoll14V732||Spain|658[0n]
Eumedonia eumedon|GWOSI554-10|BC ZSM Lep 44400||Germany|658[0n]
Eumedonia eumedon|EZSPC1436-10|RVcoll.09-V147||Spain|658[2n]
Eumedonia eumedon|EZSPN797-09|RVcoll.08-L357||Spain|658[0n]
Eumedonia eumedon|EZSPN796-09|RVcoll.08-L356||Spain|658[0n]
Eumedonia eumedon|BIBSA1625-16|15-C450||Italy|658[0n]
Eumedonia eumedon|BIBSA632-15|RVcoll. 15-A795||Italy|629[0n]
Eumedonia eumedon|BIBSA629-15|RVcoll. 15-A792||Italy|629[0n]
Eumedonia eumedon|BIBSA1333-15|15-M825||Italy|658[0n]
Eumedonia eumedon|BIBSA1155-15|15-L101||Italy|658[0n]
Eumedonia eumedon|BIBSA1151-15|15-L087||Italy|658[0n]
Eumedonia eumedon|EULEP5653-17|RVcoll15J705||Spain|658[0n]
Eumedonia eumedon|EULEP5618-17|RVcoll14V733||Spain|658[0n]
Eumedonia eumedon|EULEP5619-17|RVcoll14V735||Spain|658[0n]
Eumedonia eumedon|EULEP5621-17|RVcoll14V739||Spain|658[0n]
Eumedonia eumedon|EZSPC1177-10|RVcoll.09-T145||Spain|658[0n]
Eumedonia eumedon|EZSPN827-09|RVcoll.08-L641||Spain|658[0n]
Eumedonia eumedon|EZSPN826-09|RVcoll.08-L640||Spain|658[0n]
Eumedonia eumedon|EULEP2360-15|RVcoll.14-V052||Ukraine|618[0n]
Eumedonia eumedon|EZROM052-08|RV-06-K675||Romania|658[0n]
Eumedonia eumedon|EULEP150-14|RVcoll.10-C490||Romania|658[0n]
Eumedonia eumedon|EZROM662-08|RV-06-K674||Romania|658[0n]
Eumedonia eumedon|EULEP087-14|RVcoll.08-P973||Romania|658[1n]
Eumedonia eumedon|EZSPN1104-11|Rvcoll.10-B441||Romania|658[0n]
Eumedonia eumedon|EZROM051-08|RV-06-K670||Romania|658[0n]
Eumedonia eumedon|EZROM604-08|RV-06-M828||Romania|658[0n]
Eumedonia eumedon|EZSPC276-09|RVcoll.08-R236||Spain|655[0n]
Eumedonia eumedon|EZSPC274-09|RVcoll.08-R234||Spain|655[0n]
Eumedonia eumedon|GBLAA1410-15|BC ZSM Lep 87106||Germany|658[0n]
Eumedonia eumedon|WMB593-11|RVcoll.11-H925|Sicily|Italy|658[0n]
Eumedonia eumedon|WMB714-12|RVcoll.11-H924|Sicily|Italy|658[0n]
Eumedonia eumedon|BIBSA1567-16|11-I017||Italy|614[0n]
Eumedonia eumedon|WMB611-11|RVcoll.11-I016|Sicily|Italy|658[0n]
Eumedonia eumedon|WMB713-12|RVcoll.11-H923|Sicily|Italy|658[0n]
Eumedonia eumedon|EULEP4893-16|RVcoll16J596||Russia|658[0n]
Eumedonia eumedon|LEASS527-17|TLMF Lep 22175||Austria|612[0n]
Eumedonia eumedon|EULEP4011-16|RVcoll15I789||Austria|658[0n]
Eumedonia eumedon|EULEP4010-16|RVcoll15I245||Austria|658[0n]
Eumedonia eumedon|EULEP2125-15|RVcoll.14-J916||France|658[0n]
Eumedonia eumedon|EULEP5171-17|RVcoll16G982||Sweden|658[0n]
Eumedonia eumedon|LON481-08|NHMO-08132||Norway|657[0n]
Eumedonia eumedon|LON871-11|NHMO Lep09069||Norway|658[0n]
Eumedonia eumedon|LEFIC009-10|MM03212||Finland|658[0n]
Eumedonia eumedon|LEFIJ515-10|MM17140||Finland|658[0n]
Eumedonia eumedon|EULEP345-14|MM23830||Lithuania|658[0n]
Eumedonia eumedon|LEFIG228-10|MM14128||Finland|658[0n]
Eumedonia eumedon|PHLAB310-10|TLMF Lep 01110||Switzerland|658[0n]
Eumedonia eumedon|EULEP2435-15|RVcoll.14-V237||Switzerland|658[0n]
Eumedonia eumedon|EULEP4009-16|RVcoll15H588||Switzerland|658[0n]

Eumedonia eumedon|[illegible]
Eumedonia eumedon|PHLAB310-10|TLMF Lep 01110||Switzerland|658[0n]
Eumedonia eumedon|EULEP2435-15|RVcoll.14-V237||Switzerland|658[0n]
Eumedonia eumedon|EULEP4009-16|RVcoll15H588||Switzerland|658[0n]
Eumedonia eumedon|PHLAW046-13|TLMF Lep 09843||Austria|658[0n]
Eumedonia eumedon|PHLSA697-11|TLMF Lep 06152||Austria|658[0n]
Eumedonia eumedon|PHLAB358-10|TLMF Lep 01158||Switzerland|658[0n]
Eumedonia eumedon|OXB826-15|14-U789||Italy|658[0n]
Eumedonia eumedon|EULEP4006-16|RVcoll15G929||Switzerland|658[0n]
Eumedonia eumedon|EULEP4007-16|RVcoll15H300||Switzerland|658[0n]
Eumedonia eumedon|BIBSA365-15|RVcoll.14-I033||Italy|658[0n]
Eumedonia eumedon|EULEP4012-16|RVcoll15G388||Switzerland|634[0n]
Eumedonia eumedon|WMB200-11|RVcoll.07-W061||France|658[0n]
Eumedonia eumedon|PHLAB311-10|TLMF Lep 01111||Switzerland|658[0n]
Eumedonia eumedon|GBLAA1412-15|BC ZSM Lep 87108||Germany|658[0n]
Eumedonia eumedon|BIBSA222-15|RVcoll.14-E010||Italy|658[0n]
Eumedonia eumedon|BIBSA221-15|RVcoll.14-E009||Italy|658[0n]
Eumedonia eumedon|BIBSA1193-15|15-M276||Italy|658[0n]
Eumedonia eumedon|WMB5112-14|RVcoll. 14-L243||Italy|658[0n]
Eumedonia eumedon|LEATD297-13|TLMF Lep 12944||Italy|658[0n]
Eumedonia eumedon|LEATD150-13|TLMF Lep 12797||Italy|658[0n]
Eumedonia eumedon|EULEP1242-15|RVcoll.14-F586||Greece|658[0n]
Eumedonia eumedon|EULEP1235-15|RVcoll.14-F547||Greece|658[0n]
Eumedonia eumedon|EULEP4008-16|RVcoll15H431||Switzerland|658[0n]
Eumedonia eumedon|PHLAF433-11|TLMF Lep 05603||Macedonia|658[0n]
Eumedonia eumedon|EULEP2848-15|RVcoll.14-N932||France|658[0n]
Eumedonia eumedon|EULEP1098-15|RVcoll.14-F124||Serbia|658[0n]
Eumedonia eumedon|EULEP5137-17|RVcoll16J893||Serbia|658[0n]
Eumedonia eumedon|ABOLD622-17|TLMF Lep 21698||Austria|658[0n]
Eumedonia eumedon|EULEP4738-16|RVcoll16G568||Sweden|658[0n]
Eumedonia eumedon|GWOTF682-12|BC ZSM Lep 62388||Germany|658[0n]
Eumedonia eumedon|EZSPC1245-10|RVcoll.09-V779||Spain|658[0n]
Eumedonia eumedon|EZSPC275-09|RVcoll.08-R235||Spain|654[0n]
Eumedonia eumedon|EZSPC1229-10|RVcoll.09-V689||Spain|658[0n]
Eumedonia eumedon|EULEP5873-18|RVcoll15O509||Spain|658[0n]
Eumedonia eumedon|EZSPC273-09|RVcoll.08-R229||Spain|654[0n]
Eumedonia eumedon|EZSPC1247-10|RVcoll.09-V786||Spain|658[0n]
Eumedonia eumedon|EZSPC1228-10|RVcoll.09-V688||Spain|658[0n]
Eumedonia eumedon|EZSPN061-09|RVcoll.09-V686||Spain|658[0n]
Eumedonia eumedon|GWOTF683-12|BC ZSM Lep 62389||Germany|658[0n]
Eumedonia eumedon|LEATG224-14|TLMF Lep 14011||Austria|658[0n]
Eumedonia eumedon|BIBSA074-14|LEP-SS-00074||Italy|658[0n]
Eumedonia eumedon|BIBSA073-14|LEP-SS-00073||Italy|658[0n]
Eumedonia eumedon|BIBSA1671-16|15-C804||Italy|658[0n]
Eumedonia eumedon|BIBSA1624-16|15-C449||Italy|658[0n]
Eumedonia eumedon|BIBSA598-15|LEP-SS-00218||Italy|658[0n]
Eumedonia eumedon|BIBSA1623-16|15-C448||Italy|658[0n]
Eumedonia eumedon|FBLMX191-11|BC ZSM Lep 50402||Germany|658[0n]
Eumedonia eumedon|FBLMX192-11|BC ZSM Lep 50403||Germany|658[0n]
Eumedonia eumedon|LASTS338-14|TLMF Lep 14790||Austria|635[0n]
Cyaniris semiargus|EZSPM431-09|Rvcoll.09-X266||Spain|658[0n]
Cyaniris semiargus|EZSPN170-09|RVcoll.08-R060|Genit. examined|Spain|658[0n]
Cyaniris semiargus|EZSPM456-09|Rvcoll.09-V535||Spain|658[0n]
Cyaniris semiargus|EZSPM455-09|Rvcoll.09-V585||Spain|658[0n]
Cyaniris semiargus|EZSPC904-10|RVcoll.09-V550||Spain|658[0n]
Cyaniris semiargus|EZSPC903-10|RVcoll.09-V540||Spain|658[0n]
Cyaniris semiargus|WMB4889-14|RVcoll.14-I436||Italy|658[0n]
Cyaniris semiargus|BIBSA678-15|RVcoll. 11-I243||Italy|658[0n]
Cyaniris semiargus|EZROM138-08|RV-07-D334||Romania|658[0n]
Cyaniris semiargus|EZROM568-08|RV-07-D530||Romania|658[0n]
Cyaniris semiargus|BIBSA1674-16|15-C843||Italy|658[0n]
Cyaniris semiargus|WMB4931-14|RVcoll.14-I478||Italy|658[0n]
Cyaniris semiargus|BIBSA1361-15|15-M876||Italy|658[0n]
Cyaniris semiargus|BIBSA059-14|LEP-SS-00059||Italy|631[0n]
Cyaniris semiargus|BIBSA1529-16|LEP-SS-00374||Italy|613[0n]
Cyaniris semiargus|WMB1969-13|RVcoll.11-I188||Italy|658[0n]
Cyaniris semiargus|WMB4252-14|RVcoll.13-U069||Italy|658[0n]
Cyaniris semiargus|BIBSA060-14|LEP-SS-00060||Italy|658[0n]
Cyaniris semiargus|BIBSA1004-15|15-C092||Italy|658[0n]
Cyaniris semiargus|BIBSA726-15|LD-2770||Italy|658[0n]
Cyaniris semiargus|WMB5177-14|RVcoll.13-S524||Italy|658[0n]
Cyaniris semiargus|BIBSA1711-16|15-N160||Italy|658[0n]
Cyaniris semiargus|BIBSA974-15|14-V179||Italy|658[0n]
Cyaniris semiargus|WMB1967-13|RVcoll.11-I156||Italy|658[0n]
Cyaniris semiargus|BIBSA295-15|RVcoll.14-E097||Italy|634[0n]
Cyaniris semiargus|WMB2665-13|RVcoll.10-B795||France|658[0n]
Cyaniris semiargus|PHLSA438-11|TLMF Lep 05893||Italy|658[0n]
Cyaniris semiargus|EULEP3750-16|RVcoll15I014||Italy|658[0n]
Cyaniris semiargus|BIBSA1089-15|15-L337||Italy|658[0n]
Cyaniris semiargus|WMB814-13|RVcoll.07-E131||Italy|626[0n]
Cyaniris semiargus|EULEP4882-16|RVcoll16H984||Denmark|656[0n]
Cyaniris semiargus|EZSPN261-09|RVcoll.07-W168||Spain|658[0n]
Cyaniris semiargus|EZSPC374-09|RVcoll.08-R121||Spain|658[0n]
Cyaniris semiargus|EZSPC375-09|RVcoll.08-R122||Spain|658[0n]
Cyaniris semiargus|EZSPC373-09|RVcoll.08-L498||Spain|658[0n]
Cyaniris semiargus|EZSPC653-09|RVcoll.07-C654||France|658[0n]
Cyaniris semiargus|EULEP1765-15|RVcoll.14-H436||Greece|658[0n]
Cyaniris semiargus|EULEP1764-15|RVcoll.14-H433||Greece|658[0n]
Cyaniris semiargus|EULEP1728-15|RVcoll.14-H236||Greece|658[0n]
Cyaniris semiargus|EULEP1726-15|RVcoll.14-H232||Greece|658[0n]
Cyaniris semiargus|EULEP1395-15|RVcoll.14-G017||Greece|658[0n]
Cyaniris semiargus|EULEP1050-15|RVcoll.14-E920||Serbia|658[0n]
Cyaniris semiargus|EULEP1663-15|RVcoll.14-G880||Greece|658[0n]
Cyaniris semiargus|EULEP3755-16|RVcoll15G129||France|658[0n]
Cyaniris semiargus|EZROM139-08|RV-07-C985||Romania|658[0n]
Cyaniris semiargus|OXB824-15|14-U787||France|658[0n]
Cyaniris semiargus|EULEP2177-15|RVcoll.14-N213||Greece|658[0n]
Cyaniris semiargus|EULEP1945-15|RVcoll.14-B924||Bosnia and Herzegovina|658[0n]
Cyaniris semiargus|EZROM902-08|RVcoll.08-M251||Romania|658[0n]
Cyaniris semiargus|FBLMW312-10|BC ZSM Lep 37413||Germany|658[0n]
Cyaniris semiargus|GWORL258-09|BC ZSM Lep 18664||Germany|658[0n]
Cyaniris semiargus|GBLAB128-13|BC ZSM Lep 75754||Germany|619[0n]
Cyaniris semiargus|GBLAB776-13|BC ZSM Lep 75547||Germany|658[0n]

Cyaniris semiargus|GWORL258-09|BC ZSM Lep 18664||Germany|658[0n]
Cyaniris semiargus|GBLAB128-13|BC ZSM Lep 75754||Germany|619[0n]
Cyaniris semiargus|GBLAB776-13|BC ZSM Lep 75547||Germany|658[0n]
Cyaniris semiargus|EULEP2002-15|RVcoll.14-I781||Slovakia|658[0n]
Cyaniris semiargus|EULEP4539-16|RVcoll15Q083||Russia|658[0n]
Cyaniris semiargus|LEATC574-13|TLMF Lep 12556||Italy|658[0n]
Cyaniris semiargus|EULEP588-15|RVcoll.10-A706||Estonia|658[0n]
Cyaniris semiargus|EULEP792-15|RVcoll.12-Z255||Sweden|658[0n]
Cyaniris semiargus|EULEP363-14|MM23848||Lithuania|658[0n]
Cyaniris semiargus|EULEP4907-16|RVcoll16J610||Russia|658[0n]
Cyaniris semiargus|LEFIJ520-10|MM17145||Finland|658[0n]
Cyaniris semiargus|LEFIJ518-10|MM17143||Finland|658[0n]
Cyaniris semiargus|WMB617-11|RVcoll.11-I034|Sicily|Italy|658[0n]
Cyaniris semiargus|WMB594-11|RVcoll.11-H926|Sicily|Italy|658[0n]
Cyaniris semiargus|WMB560-11|RVcoll.11-H753|Sicily|Italy|658[0n]
Cyaniris semiargus|WMB547-11|RVcoll.11-H736|Sicily|Italy|658[0n]
Cyaniris semiargus|WMB2942-14|RVcoll.12-M397|Sicily|Italy|658[0n]
Cyaniris semiargus|GWORO804-09|BC ZSM Lep 30496||Germany|634[0n]
Cyaniris semiargus|EULEP1447-15|RVcoll.14-G155||Greece|658[0n]
Cyaniris semiargus|EULEP4816-16|RVcoll16H711||Belgium|658[0n]
Cyaniris semiargus|EZROM623-08|RV-07-C925||Romania|658[0n]
Cyaniris semiargus|EZROM903-08|RVcoll.08-M347||Romania|658[0n]
Cyaniris semiargus|BIBSA1187-15|15-M243||Italy|612[0n]
Cyaniris semiargus|OXB631-15|14-O020||Italy|658[0n]
Cyaniris semiargus|EULEP2440-15|RVcoll.14-V244||Switzerland|658[0n]
Cyaniris semiargus|FBLMX195-11|BC ZSM Lep 50406||Germany|658[0n]
Cyaniris semiargus|WMB5239-14|RVcoll.13-U091||Italy|658[0n]
Cyaniris semiargus|EZSPN963-09|RVcoll.08-L970||Spain|637[0n]
Cyaniris semiargus|EULEP1730-15|RVcoll.14-H255||Greece|658[0n]
Cyaniris semiargus|EULEP1729-15|RVcoll.14-H251||Greece|658[0n]
Cyaniris semiargus|EZSPN652-09|RVcoll.08-J795||Spain|626[0n]
Cyaniris semiargus|EZSPN829-09|RVcoll.08-L644||Spain|658[0n]
Cyaniris semiargus|BIBSA200-15|RVcoll.14-D988||Italy|637[0n]
Cyaniris semiargus|FBLMW316-10|BC ZSM Lep 37417||Austria|658[0n]
Cyaniris semiargus|LON820-11|NHMO Lep09018||Norway|658[0n]
Cyaniris semiargus|EZROM904-08|RVcoll.08-M599||Romania|658[0n]
Cyaniris semiargus|EZROM905-08|RVcoll.08-M643||Romania|658[0n]
Cyaniris semiargus|EZSPC372-09|RVcoll.07-C473||Spain|658[0n]
Cyaniris semiargus|EZSPN496-09|RVcoll.08-H940||Spain|658[0n]
Cyaniris semiargus|EZSPN989-09|RVcoll.08-M033||Spain|658[0n]
Cyaniris semiargus|EZSPC773-10|RVcoll.08-L786.1||Spain|658[0n]
Cyaniris semiargus|EZSPC1097-10|RVcoll.08-L940.1||Spain|658[0n]
Cyaniris semiargus|EZSPC1098-10|RVcoll.08-L941||Spain|658[0n]
Cyaniris semiargus|WMB3360-14|RVcoll.09-T152||Spain|658[0n]
Cyaniris semiargus|WMB2945-14|RVcoll.12-M419|Sicily|Italy|658[0n]
Cyaniris semiargus|WMB2698-13|RVcoll.10-C073||France|658[0n]
Cyaniris semiargus|WMB1842-13|RVcoll.11-I890||France|658[0n]
Cyaniris semiargus|WMB4566-14|RVcoll.130211WX02||Spain|658[0n]
Cyaniris semiargus|EULEP3744-16|RVcoll14W529||Italy|658[0n]
Cyaniris semiargus|EULEP3748-16|RVcoll15H180||Switzerland|658[0n]
Cyaniris semiargus|EULEP3749-16|RVcoll15H798||Italy|658[0n]
Cyaniris semiargus|EULEP3751-16|RVcoll15I147||Austria|658[0n]
Cyaniris semiargus|EULEP3752-16|RVcoll15I391||Austria|658[0n]
Cyaniris semiargus|EULEP3753-16|RVcoll15I606||Austria|658[0n]
Cyaniris semiargus|EULEP3754-16|RVcoll15I892||Liechtenstein|658[0n]
Cyaniris semiargus|EULEP4757-16|RVcoll16G984||Sweden|658[0n]
Cyaniris semiargus|EULEP4977-16|RVcoll16I494||Poland|658[0n]
Cyaniris semiargus|EULEP1802-15|RVcoll.14-H706||Greece|658[0n]
Cyaniris semiargus|EULEP1807-15|RVcoll.14-H758||Greece|658[0n]
Cyaniris semiargus|EULEP2342-15|RVcoll.14-V028||Ukraine|658[0n]
Cyaniris semiargus|BIBSA381-15|RVcoll.14-I049||Italy|658[0n]
Cyaniris semiargus|BIBSA382-15|RVcoll.14-I050||Italy|658[0n]
Cyaniris semiargus|LEASS535-17|TLMF Lep 22183||Austria|658[0n]
Cyaniris semiargus|OXB830-15|14-U793||Italy|658[0n]
Cyaniris semiargus|EZSPC776-10|RVcoll.08-L807||Spain|658[0n]
Cyaniris semiargus|LEASS460-17|TLMF Lep 21918||Austria|658[0n]
Cyaniris semiargus|OXB745-15|13-U404||Italy|658[0n]
Cyaniris semiargus|GWOTF684-12|BC ZSM Lep 62390||Germany|658[0n]
Cyaniris semiargus|GWORL261-09|BC ZSM Lep 18667||Germany|658[0n]
Cyaniris semiargus|LEATD458-13|TLMF Lep 13105||Italy|658[0n]
Cyaniris semiargus|LEATA409-13|TLMF Lep 10016||Austria|658[0n]
Cyaniris semiargus|LEATG104-14|TLMF Lep 13891||Austria|658[0n]
Cyaniris semiargus|GWORA2524-09|BC ZSM Lep 30736||Austria|658[0n]
Cyaniris semiargus|LEATG014-14|TLMF Lep 13801||Austria|658[0n]
Cyaniris semiargus|LEATG225-14|TLMF Lep 14012||Austria|658[0n]
Cyaniris semiargus|ABOLD008-16|TLMF Lep 21086||Austria|658[0n]
Kretania psylorita|EULEP5123-17|RVcoll16J878|Crete|Greece|658[0n]
Kretania psylorita|EULEP2904-15|RVcoll.14-U857|Crete|Greece|621[0n]
Kretania psylorita|EULEP5122-17|RVcoll16J877|Crete|Greece|658[0n]
Kretania psylorita|EULEP5121-17|RVcoll16J876|Crete|Greece|658[0n]
Agriades orbitulus|EULEP3418-16|RVcoll15G251||Switzerland|642[0n]
Agriades orbitulus|EULEP2028-15|RVcoll.14-J503||Switzerland|658[0n]
Agriades orbitulus|EULEP3410-16|RVcoll14W468||Italy|658[0n]
Agriades orbitulus|BIBSA1078-15|15-K529||Italy|658[0n]
Agriades orbitulus|PHLAA136-09|TLMF Lep 00176||Italy|658[0n]
Agriades orbitulus|EULEP2448-15|RVcoll.14-V264||Switzerland|658[0n]
Agriades orbitulus|EULEP3417-16|RVcoll15J401||France|658[0n]
Agriades orbitulus|BIBSA284-15|RVcoll.14-E084||Italy|658[0n]
Agriades orbitulus|BIBSA354-15|RVcoll.14-I022||Italy|658[0n]
Agriades orbitulus|GBLAC373-13|BC ZSM Lep 75239||Germany|658[0n]
Agriades orbitulus|EULEP4800-16|RVcoll16H631||Sweden|658[0n]
Agriades orbitulus|EULEP3416-16|RVcoll15I629||Austria|658[0n]
Agriades orbitulus|EULEP1867-15|RVcoll.13-U334||Italy|658[0n]
Agriades orbitulus|EULEP4801-16|RVcoll16H632||Sweden|658[0n]
Agriades orbitulus|EULEP3415-16|RVcoll15I236||Austria|658[0n]
Agriades orbitulus|EULEP3414-16|RVcoll15H817||Italy|658[0n]
Agriades orbitulus|EULEP3413-16|RVcoll15H469||Switzerland|658[0n]
Agriades orbitulus|EULEP3412-16|RVcoll15H283||Switzerland|658[0n]
Agriades orbitulus|EULEP3411-16|RVcoll15G820||Italy|658[0n]
Agriades orbitulus|WMB2690-13|RVcoll.10-B998||France|658[0n]
Agriades orbitulus|GWOSA773-10|BC ZSM Lep 36639||Germany|658[0n]
Agriades orbitulus|GWOTF675-12|BC ZSM Lep 62381||Germany|658[0n]
Agriades orbitulus|ODOPE760-11|BC ZSM Lep 50401||Germany|658[0n]

Agriades orbitulus|GWOSA773-10|BC ZSM Lep 36639||Germany|658[0n]
Agriades orbitulus|GWOTF675-12|BC ZSM Lep 62381||Germany|658[0n]
Agriades orbitulus|ODOPE760-11|BC ZSM Lep 50401||Germany|658[0n]
Agriades orbitulus|PHLAC715-10|TLMF Lep 02750||Switzerland|658[0n]
Agriades orbitulus|LEATG093-14|TLMF Lep 13880||Austria|658[0n]
Agriades orbitulus|LEATG098-14|TLMF Lep 13885||Austria|658[0n]
Agriades orbitulus|PHLSA728-11|TLMF Lep 06183||Austria|658[0n]
Agriades optilete|EULEP4779-16|RVcoll16H338||Norway|658[0n]
Agriades optilete|LON177-08|NHMO-06178||Norway|657[0n]
Agriades optilete|LYCAE090-15|VL01B425||Russia|676[1n]
Agriades optilete|ABOLD619-17|TLMF Lep 21695||Austria|658[0n]
Agriades optilete|LYCAE091-15|VL01B430||Russia|676[0n]
Agriades optilete|EULEP380-14|MM23865||Finland|658[0n]
Agriades optilete|LYCAE025-15|SS12745||Denmark|676[0n]
Agriades optilete|LYCAE024-15|SS12744||Denmark|676[0n]
Agriades optilete|LYCAE023-15|SS12743||Denmark|676[0n]
Agriades optilete|LYCAE022-15|SS12742||Denmark|676[0n]
Agriades optilete|LYCAE021-15|SS12741||Denmark|676[0n]
Agriades optilete|LYCAE020-15|SS12740||Denmark|676[0n]
Agriades optilete|LYCAE089-15|VL01B432||Russia|676[0n]
Agriades optilete|LOWA757-06|2005-LOWA-757||Russia|658[0n]
Agriades optilete|LOWA297-06|2005-LOWA-297||Russia|658[0n]
Agriades optilete|LYCAE074-15|SS12807||Denmark|676[0n]
Agriades optilete|LYCAE043-15|SS12915||Denmark|676[0n]
Agriades optilete|LYCAE034-15|SS12886||Denmark|676[0n]
Agriades optilete|LYCAE033-15|SS12839||Denmark|676[0n]
Agriades optilete|EULEP3409-16|RVcoll15Q134||Russia|636[0n]
Agriades optilete|LEFIC061-10|MM03345||Finland|658[0n]
Agriades optilete|LEFIC060-10|MM03344||Finland|658[0n]
Agriades optilete|LOWA296-06|2005-LOWA-296||Russia|658[0n]
Agriades optilete|EULEP379-14|MM23864||Finland|658[0n]
Agriades optilete|EULEP361-14|MM23846||Lithuania|658[0n]
Agriades optilete|EULEP590-15|RVcoll.10-A722||Estonia|658[0n]
Agriades optilete|EULEP2280-15|RVcoll.14-N466||Ukraine|658[0n]
Agriades optilete|EULEP2281-15|RVcoll.14-N467||Ukraine|658[0n]
Agriades optilete|LYCAE016-15|SS12966||Denmark|668[1n]
Agriades optilete|LYCAE002-15|SS121000||Denmark|676[0n]
Agriades optilete|LYCAE003-15|SS121001||Denmark|676[0n]
Agriades optilete|LYCAE004-15|SS121002||Denmark|676[0n]
Agriades optilete|LYCAE005-15|SS12989||Denmark|676[0n]
Agriades optilete|LYCAE006-15|SS12990||Denmark|676[0n]
Agriades optilete|LYCAE007-15|SS12991||Denmark|676[0n]
Agriades optilete|LYCAE008-15|SS12993||Denmark|676[0n]
Agriades optilete|LYCAE009-15|SS12996||Denmark|676[0n]
Agriades optilete|LYCAE010-15|SS12999||Denmark|676[0n]
Agriades optilete|LYCAE011-15|SS12941||Denmark|676[0n]
Agriades optilete|LYCAE012-15|SS12946||Denmark|676[0n]
Agriades optilete|LYCAE013-15|SS12950||Denmark|676[0n]
Agriades optilete|LYCAE014-15|SS12954||Denmark|676[0n]
Agriades optilete|LYCAE017-15|SS12967||Denmark|676[0n]
Agriades optilete|LYCAE018-15|SS12968||Denmark|676[0n]
Agriades optilete|LYCAE026-15|SS12836||Denmark|676[0n]
Agriades optilete|LYCAE027-15|SS12837||Denmark|676[0n]
Agriades optilete|LYCAE028-15|SS12840||Denmark|676[0n]
Agriades optilete|LYCAE029-15|SS12841||Denmark|676[0n]
Agriades optilete|LYCAE030-15|SS12842||Denmark|676[0n]
Agriades optilete|LYCAE031-15|SS12878||Denmark|676[0n]
Agriades optilete|LYCAE032-15|SS12879||Denmark|676[0n]
Agriades optilete|LYCAE035-15|SS12901||Denmark|676[0n]
Agriades optilete|LYCAE036-15|SS12904||Denmark|676[0n]
Agriades optilete|LYCAE037-15|SS12908||Denmark|676[0n]
Agriades optilete|LYCAE038-15|SS12909||Denmark|676[0n]
Agriades optilete|LYCAE039-15|SS12910||Denmark|676[0n]
Agriades optilete|LYCAE040-15|SS12912||Denmark|676[0n]
Agriades optilete|LYCAE041-15|SS12913||Denmark|676[0n]
Agriades optilete|LYCAE042-15|SS12917||Denmark|676[0n]
Agriades optilete|LYCAE044-15|SS12754||Denmark|676[0n]
Agriades optilete|LYCAE045-15|SS12758||Denmark|676[0n]
Agriades optilete|LYCAE046-15|SS12762||Denmark|676[0n]
Agriades optilete|LYCAE047-15|SS12763||Denmark|676[0n]
Agriades optilete|LYCAE048-15|SS12766||Denmark|676[0n]
Agriades optilete|LYCAE049-15|SS12770||Denmark|676[0n]
Agriades optilete|LYCAE050-15|SS12772||Denmark|676[0n]
Agriades optilete|LYCAE051-15|SS12783||Denmark|676[0n]
Agriades optilete|LYCAE053-15|SS12746||Denmark|676[0n]
Agriades optilete|LYCAE054-15|SS12747||Denmark|676[0n]
Agriades optilete|LYCAE055-15|SS12748||Denmark|676[0n]
Agriades optilete|LYCAE056-15|SS12750||Denmark|676[0n]
Agriades optilete|LYCAE057-15|SS12751||Denmark|676[0n]
Agriades optilete|LYCAE058-15|SS12752||Denmark|676[0n]
Agriades optilete|LYCAE059-15|SS12753||Denmark|676[0n]
Agriades optilete|LYCAE060-15|SS11164||Denmark|676[0n]
Agriades optilete|LYCAE061-15|SS11167||Denmark|676[0n]
Agriades optilete|LYCAE062-15|SS11168||Denmark|676[0n]
Agriades optilete|LYCAE063-15|SS11169||Denmark|676[0n]
Agriades optilete|LYCAE064-15|SS11170||Denmark|676[0n]
Agriades optilete|LYCAE065-15|SS11171||Denmark|676[0n]
Agriades optilete|LYCAE066-15|SS12854||Denmark|676[0n]
Agriades optilete|LYCAE067-15|SS12862||Denmark|676[0n]
Agriades optilete|LYCAE068-15|SS12798||Denmark|676[0n]
Agriades optilete|LYCAE069-15|SS12799||Denmark|676[0n]
Agriades optilete|LYCAE070-15|SS12805||Denmark|676[0n]
Agriades optilete|LYCAE071-15|SS12808||Denmark|676[0n]
Agriades optilete|LYCAE072-15|SS12810||Denmark|676[0n]
Agriades optilete|LYCAE073-15|SS12816||Denmark|676[0n]
Agriades optilete|LYCAE076-15|SS12970||Denmark|676[0n]
Agriades optilete|LYCAE077-15|SS12971||Denmark|676[0n]
Agriades optilete|LYCAE078-15|SS12974||Denmark|676[0n]
Agriades optilete|LYCAE079-15|SS12975||Denmark|676[0n]
Agriades optilete|LYCAE080-15|SS12976||Denmark|676[0n]
Agriades optilete|LYCAE081-15|SS12977||Denmark|676[0n]
Agriades optilete|LYCAE082-15|SS12978||Denmark|676[0n]
[illegible]

Agriades optilete|LYCAE080-15|SS12976||Denmark|676[0n]
Agriades optilete|LYCAE081-15|SS12977||Denmark|676[0n]
Agriades optilete|LYCAE082-15|SS12978||Denmark|676[0n]
Agriades optilete|LYCAE083-15|SS12979||Denmark|676[0n]
Agriades optilete|LYCAE084-15|VL01B426||Russia|676[0n]
Agriades optilete|LYCAE085-15|VL01B427||Russia|676[0n]
Agriades optilete|LYCAE086-15|VL01B428||Russia|676[0n]
Agriades optilete|LYCAE075-15|SS12809||Denmark|676[0n]
Agriades optilete|LYCAE087-15|VL01B431||Russia|676[0n]
Agriades optilete|LYCAE088-15|VL01B433||Russia|676[0n]
Agriades optilete|LYCAE019-15|SS12969||Denmark|644[0n]
Agriades optilete|LYCAE015-15|SS12961||Denmark|640[0n]
Agriades optilete|EULEP798-15|RVcoll.12-Z315||Sweden|634[0n]
Agriades optilete|EULEP527-15|RVcoll.08-H828||Sweden|658[0n]
Agriades optilete|LEFIJ517-10|MM17142||Finland|655[0n]
Agriades optilete|LON088-08|NHMO-06088||Norway|657[0n]
Agriades optilete|ABOLD020-16|TLMF Lep 21098||Austria|658[0n]
Agriades optilete|EULEP2026-15|RVcoll.14-J501||Switzerland|658[0n]
Agriades optilete|EULEP3405-16|RVcoll15G753||Switzerland|658[0n]
Agriades optilete|LEASS571-17|TLMF Lep 22219||Austria|625[0n]
Agriades optilete|PHLAW042-13|TLMF Lep 09839||Austria|658[0n]
Agriades optilete|WMB5123-14|RVcoll. 14-L254||Italy|658[0n]
Agriades optilete|LEATD143-13|TLMF Lep 12790||Italy|658[0n]
Agriades optilete|EULEP3406-16|RVcoll15H627||Switzerland|658[0n]
Agriades optilete|GWORO798-09|BC ZSM Lep 30490||Germany|658[0n]
Agriades optilete|ODOPE757-11|BC ZSM Lep 50398||Germany|658[0n]
Agriades optilete|EULEP2470-15|RVcoll.14-V297||Switzerland|658[0n]
Agriades optilete|EULEP2027-15|RVcoll.14-J502||Switzerland|658[0n]
Agriades optilete|EULEP5118-17|RVcoll16J873||Macedonia|621[0n]
Agriades optilete|EULEP5117-17|RVcoll16J872||Macedonia|658[0n]
Agriades optilete|EULEP3408-16|RVcoll15G277||Switzerland|635[0n]
Agriades optilete|EULEP3407-16|RVcoll15I313||Austria|658[0n]
Agriades optilete|LEATG483-14|TLMF Lep 14270||Italy|658[0n]
Agriades optilete|PHLAB1026-10|TLMF Lep 01826||Switzerland|658[0n]
Agriades optilete|LEATF468-14|TLMF Lep 13780||Austria|658[0n]
Agriades zullichi|EZSPM472-09|Rvcoll.09-V418||Spain|658[0n]
Agriades zullichi|EZSPM473-09|Rvcoll.09-V414||Spain|658[0n]
Agriades zullichi|EZSPM471-09|Rvcoll.09-V416||Spain|658[0n]
Agriades zullichi|EZSPC893-10|RVcoll.09-V417||Spain|658[0n]
Agriades zullichi|EZSPC892-10|RVcoll.09-V415||Spain|658[0n]
Agriades zullichi|EZSPN659-09|RVcoll.08-J957||Spain|658[0n]
Agriades zullichi|EZSPN678-09|RVcoll.08-J990||Spain|658[0n]
Agriades zullichi|EZSPN677-09|RVcoll.08-J989||Spain|626[0n]
Agriades glandon|EULEP3396-16|RVcoll15H280||Switzerland|658[0n]
Agriades glandon|WMB1633-13|RVcoll.12-O844||France|658[0n]
Agriades glandon|EULEP3399-16|RVcoll15J552||France|602[1n]
Agriades glandon|BIBSA330-15|RVcoll.14-E135||Italy|612[0n]
Agriades glandon|PHLAA368-09|TLMF Lep 00408||Italy|600[3n]
Agriades glandon|PHLAB1024-10|TLMF Lep 01824||Italy|658[0n]
Agriades glandon|GWOTI468-12|BC ZSM Lep 66734||Switzerland|658[0n]
Agriades glandon|ODOPE758-11|BC ZSM Lep 50399||Germany|658[0n]
Agriades glandon|GWOTI464-12|BC ZSM Lep 66730||Germany|658[0n]
Agriades glandon|GWOTI467-12|BC ZSM Lep 66733||Germany|658[0n]
Agriades glandon|EZSPN247-09|RVcoll.07-W123||Spain|658[0n]
Agriades glandon|EZSPM634-12|RVcoll.11-H025||Spain|658[0n]
Agriades glandon|EZSPM636-12|RVcoll.11-H027||Spain|658[0n]
Agriades glandon|EZSPM538-10|RVcoll.08-R215.1||Spain|648[0n]
Agriades glandon|EZSPM537-10|RVcoll.08-R214.1||Spain|616[0n]
Agriades glandon|LEATG232-14|TLMF Lep 14019||Austria|658[0n]
Agriades glandon|PHLAB1022-10|TLMF Lep 01822||Switzerland|658[0n]
Agriades glandon|EULEP3397-16|RVcoll15I306||Austria|658[0n]
Agriades glandon|EULEP3398-16|RVcoll15J100||Switzerland|658[0n]
Agriades glandon|EZSPM548-10|RVcoll.07-W204||Spain|623[0n]
Agriades glandon|GWOTI472-12|BC ZSM Lep 66738||Switzerland|658[0n]
Agriades glandon|GWOTI471-12|BC ZSM Lep 66737||Switzerland|658[0n]
Agriades glandon|LEATG231-14|TLMF Lep 14018||Austria|658[0n]
Agriades glandon|LEATC093-13|TLMF Lep 11220||Italy|658[0n]
Agriades glandon|PHLAB1023-10|TLMF Lep 01823||Switzerland|658[0n]
Agriades glandon|LEATC094-13|TLMF Lep 11221||Italy|658[0n]
Agriades glandon|EZSPM550-10|RVcoll.08-P083.1||Andorra|652[0n]
Agriades glandon|EULEP5872-18|RVcoll15O508||Spain|658[0n]
Agriades glandon|EZSPC845-10|RVcoll.08-R217||Spain|658[0n]
Agriades glandon|EZSPM539-10|RVcoll.08-R216.1||Spain|658[0n]
Agriades aquilo|EULEP4769-16|RVcoll16H164||Norway|658[0n]
Agriades aquilo|EULEP4770-16|RVcoll16H167||Norway|658[0n]
Agriades aquilo|EULEP4772-16|RVcoll16H183||Norway|658[0n]
Agriades aquilo|LEFIJ3157-15|MM24727||Norway|658[0n]
Agriades aquilo|LEFIJ3158-15|MM24728||Norway|658[0n]
Agriades aquilo|LEFIJ3159-15|MM24729||Norway|658[0n]
Agriades aquilo|DIFIA665-12|MM22816||Finland|641[0n]
Agriades aquilo|FIDIP3610-12|MM22817||Finland|621[0n]
Agriades aquilo|LON178-08|NHMO-06179||Norway|608[0n]
Agriades glandon|EULEP3400-16|RVcoll15G348||Switzerland|637[0n]
Agriades glandon|EULEP3395-16|RVcoll15H130||Italy|658[0n]
Agriades glandon|WMB1634-13|RVcoll.12-O845||France|658[0n]
Agriades glandon|PHLAB1025-10|TLMF Lep 01825||Italy|658[0n]
Glabroculus cyane|EULEP3062-15|RVcoll.14-O195||Russia|658[0n]
Glabroculus cyane|EULEP3061-15|RVcoll.14-O194||Russia|658[0n]
Glabroculus cyane|EULEP433-14|KN00795||Russia|658[0n]
Plebejus argus|LYCAE451-15|ZT111||Serbia|676[0n]
Plebejus argus|EULEP245-14|RVcoll.12-N714||Spain|658[0n]
Plebejus argus|WMB3340-14|RVcoll.08-P917||Spain|658[0n]
Plebejus argus|EZSPC1292-10|RVcoll.121109PY13||Spain|658[0n]
Plebejus argus|EZSPN986-09|RVcoll.08-M028||Spain|658[0n]
Plebejus argus|EZSPN363-09|RVcoll.08-H347||Spain|657[0n]
Plebejus argus|EZSPM872-12|Rvcoll.130410FG44||Spain|658[0n]
Plebejus argus|EZSPC1293-10|RVcoll.050208ZX5||Spain|658[0n]
Plebejus argus|EZSPN718-09|RVcoll.08-L104||Spain|658[0n]
Plebejus argus|EZSPM451-09|Rvcoll.09-V591|Genit. examined|Spain|658[0n]
Plebejus argus|EZSPN739-09|RVcoll.08-L152||Spain|658[0n]
Plebejus argus|EZSPN726-09|RVcoll.08-L133||Spain|658[0n]
Plebejus argus|BIBSA065-14|LEP-SS-00065||Italy|632[0n]
Plebejus argus|BIBSA079-14|LEP-SS-00079||Italy|658[0n]

Plebejus argus|EZSPN726-09|RVcoll.08-L133||Spain|658[0n]
Plebejus argus|BIBSA065-14|LEP-SS-00065||Italy|632[0n]
Plebejus argus|BIBSA079-14|LEP-SS-00079||Italy|658[0n]
Plebejus argus|BIBSA750-15|LEP-SS-00251||Italy|658[0n]
Plebejus argus|WMB3813-14|RVcoll.07-E034||Italy|658[0n]
Plebejus argus|WMB3809-14|RVcoll.07-D894||Italy|658[0n]
Plebejus argus|LYCAE380-15|AD00P234||Russia|676[0n]
Plebejus argus|LYCAE378-15|AD00P232||Russia|676[0n]
Plebejus argus|WMB655-11|RVcoll.11-I270||Italy|658[0n]
Plebejus argus|WMB3814-14|RVcoll.07-E060||Italy|658[0n]
Plebejus argus|EZSPM432-09|Rvcoll.09-X267||Spain|658[0n]
Plebejus argus|WMB4376-14|RVcoll.14-B174||Portugal|658[0n]
Plebejus argus|EZSPM876-12|Rvcoll.130711PX72||Spain|658[0n]
Plebejus argus|EZSPM305-09|RVcoll.08-J852||Spain|658[0n]
Plebejus argus|WMB4462-14|RVcoll.14-D332||Spain|658[0n]
Plebejus argus|EZSPN869-09|RVcoll.08-L750|Genit. examined|Spain|621[0n]
Plebejus argus|EZSPN599-09|RVcoll.08-J189||Portugal|658[0n]
Plebejus argus|EZSPN1087-11|Rvcoll.09-X536||Spain|658[0n]
Plebejus argus|EZSPN1054-11|Rvcoll.08-L962||Spain|658[0n]
Plebejus argus|LYCAE369-15|SS121096||Denmark|676[0n]
Plebejus argus|EULEP1222-15|RVcoll.14-F525||Greece|658[0n]
Plebejus argus|EULEP4888-16|RVcoll16J576||Moldova|658[0n]
Plebejus argus|EZRMN186-08|RVcoll.08-M216||Romania|658[0n]
Plebejus argus|EZRMN185-08|RVcoll.08-M380||Romania|658[0n]
Plebejus argus|LYCAE339-15|SS11134||Denmark|676[0n]
Plebejus argus|OXB501-15|OXB-TGS-668||United Kingdom|658[0n]
Plebejus argus|OXB455-15|OXB-TGS-290||United Kingdom|658[0n]
Plebejus argus|OXB454-15|OXB-TGS-289||United Kingdom|658[0n]
Plebejus argus|OXB502-15|OXB-TGS-669||United Kingdom|658[0n]
Plebejus argus|LYCAE337-15|SS11132||Denmark|676[0n]
Plebejus argus|OXB672-15|07-E088||Italy|658[0n]
Plebejus argus|LOWA534-06|2005-LOWA-534||Ukraine|658[0n]
Plebejus argus|EULEP4802-16|RVcoll16H660||Norway|658[0n]
Plebejus argus|GBLAB124-13|BC ZSM Lep 75750||Germany|602[0n]
Plebejus argus|GWOSU069-11|BC ZSM Lep 53320||Germany|658[2n]
Plebejus argus|FBLMU499-09|BC ZSM Lep 27149||Germany|615[0n]
Plebejus argus|OXB1294-15|Rvcoll_15-M159||France|612[0n]
Plebejus argus|FBLMU498-09|BC ZSM Lep 27148||Germany|632[0n]
Plebejus argus|LYCAE372-15|SS121099||Denmark|676[0n]
Plebejus argus|LYCAE336-15|SS11131||Denmark|676[0n]
Plebejus argus|LYCAE338-15|SS11133||Denmark|676[0n]
Plebejus argus|LYCAE340-15|SS11135||Denmark|676[0n]
Plebejus argus|LYCAE364-15|MAT99Q874||Spain|676[0n]
Plebejus argus|LYCAE367-15|SS121092||Denmark|676[0n]
Plebejus argus|LYCAE368-15|SS121094||Denmark|676[0n]
Plebejus argus|LYCAE370-15|SS121097||Denmark|676[0n]
Plebejus argus|LYCAE371-15|SS121098||Denmark|676[0n]
Plebejus argus|LYCAE373-15|SS121105||Denmark|676[0n]
Plebejus argus|OXB563-15|OXB-TGS-938||United Kingdom|658[0n]
Plebejus argus|LYCAE414-15|MAT99Q851||Spain|676[0n]
Plebejus argus|EULEP4466-16|RVcoll15G517||Switzerland|658[0n]
Plebejus argus|WMB4439-14|RVcoll.14-B630||United Kingdom|658[0n]
Plebejus argus|EZSPC649-09|RVcoll.07-C493||Spain|658[0n]
Plebejus argus|ABOLD654-17|TLMF Lep 21730||Austria|658[0n]
Plebejus argus|GWORL274-09|BC ZSM Lep 18680|ID verified (AS)|Germany|658[0n]
Plebejus argus|LEATG076-14|TLMF Lep 13863||Austria|658[0n]
Plebejus argus|PHLAW012-13|TLMF Lep 09809||Austria|658[0n]
Plebejus argus|EZSPC671-09|RVcoll.08-M912||Spain|658[0n]
Plebejus argus|EZSPC670-09|RVcoll.08-M904|Genit. examined|Spain|658[0n]
Plebejus argus|FBLMV692-09|BC ZSM Lep 28672||Germany|658[0n]
Plebejus argus|OXB479-15|OXB-TGS-467||United Kingdom|658[0n]
Plebejus argus|OXB469-15|OXB-TGS-417||United Kingdom|658[0n]
Plebejus argus|LEATG077-14|TLMF Lep 13864||Austria|658[0n]
Plebejus argus|LEFIC056-10|MM03336||Finland|658[0n]
Plebejus argus|EULEP1647-15|LR-08-D694||Slovenia|658[0n]
Plebejus argus|EULEP1286-15|RVcoll.14-F701||Greece|658[0n]
Plebejus argus|EULEP853-15|RVcoll.14-C144||Greece|627[0n]
Plebejus argus|EULEP2178-15|RVcoll.14-N214||Greece|658[0n]
Plebejus argus|EULEP943-15|RVcoll.14-C937||Bulgaria|658[0n]
Plebejus argus|EULEP1247-15|RVcoll.14-F603||Greece|658[0n]
Plebejus argus|EULEP4475-16|RVcoll10B410|Genit. examined|Bulgaria|658[0n]
Plebejus argus|LYCAE419-15|ZTnisevac3||Serbia|637[0n]
Plebejus argus|EULEP1895-15|RVcoll.14-B721||Albania|658[0n]
Plebejus argus|EULEP562-15|RVcoll.08-R602|Corsica Genit. examined|France|658[0n]
Plebejus argus|WMB2609-13|RVcoll.08-R601|Corsica|France|658[0n]
Plebejus argus|WMB855-13|RVcoll.08-R606|Corsica. Genit. examined|France|658[0n]
Plebejus argus|WMB1520-13|RVcoll.12-O157|Corsica. Genit. examined|France|658[0n]
Plebejus argus|WMB3802-14|RVcoll.07-D822||Italy|658[0n]
Plebejus argus|WMB2021-13|RVcoll.12-Q684||Italy|658[0n]
Plebejus argus|WMB1504-13|RVcoll.12-O082|Corsica. Genit. examined|France|658[0n]
Plebejus argus|LEASS919-17|KLM Lep 08424||Austria|658[0n]
Plebejus argus|LYCAE365-15|SS1155||Sweden|676[0n]
Plebejus argus|BIBSA259-15|RVcoll.14-E056||Italy|658[0n]
Plebejus argus|EULEP4467-16|RVcoll15H989||Italy|658[0n]
Plebejus argus|WMB1791-13|RVcoll.12-Q242||France|658[0n]
Plebejus argus|WMB3525-14|RVcoll.10-C065||France|658[0n]
Plebejus argus|EULEP1885-15|RVcoll.14-B693||Albania|658[0n]
Plebejus argus|WMB4539-14|RVcoll.LD-2763||Italy|658[0n]
Plebejus argus|EZSPM452-09|Rvcoll.09-V925||Spain|658[0n]
Plebejus argus|EULEP1399-15|RVcoll.14-G033||Greece|658[0n]
Plebejus argus|EULEP1339-15|RVcoll.14-F828|Genit. examined|Greece|658[0n]
Plebejus argus|LYCAE444-15|MAT99Q901||Spain|676[0n]
Plebejus argus|WMB4915-14|RVcoll.14-I462||Italy|658[0n]
Plebejus argus|WMB841-13|RVcoll.07-E266|Genit. examined|France|658[0n]
Plebejus argus|EZROM375-08|07-E625||Spain|658[0n]
Plebejus argus|EZSPN1036-11|Rvcoll.07-C698|Genit. examined|Spain|658[0n]
Plebejus argus|EZROM749-08|RV-07-C485|Genit. examined|Spain|658[0n]
Plebejus argus|EZSPC665-09|RVcoll.08-L468||Spain|658[0n]
Plebejus argus|WMB4918-14|RVcoll.14-I465||Italy|658[0n]
Plebejus argus|BIBSA1846-17|RVcoll16C724||Italy|658[0n]
Plebejus argus|EULEP4469-16|RVcoll15G124||France|632[0n]
Plebejus argus|GBLAA385-14|BC ZSM Lep 80476||Germany|658[0n]
Plebejus argus|OXB562-15|OXB-TGS-937||United Kingdom|658[0n]

Plebejus argus|BIBSA1640-17|RVcoll16C724||Italy|658[0n]
Plebejus argus|EULEP4469-16|RVcoll15G124||France|632[0n]
Plebejus argus|GBLAA385-14|BC ZSM Lep 80476||Germany|658[0n]
Plebejus argus|OXB562-15|OXB-TGS-937||United Kingdom|658[0n]
Plebejus argus|EULEP4465-16|RVcoll15G047||France|658[0n]
Plebejus argus|WMB1649-13|RVcoll.12-O984||France|658[0n]
Plebejus argus|LON540-08|NHMO-08192||Norway|657[0n]
Plebejus argus|EULEP1441-15|RVcoll.14-G133||Greece|658[0n]
Plebejus argus|BIBSA258-15|RVcoll.14-E055||Italy|658[0n]
Plebejus argus|LYCAE435-15|SS11228||Denmark|676[2n]
Plebejus argus|BIBSA1122-15|15-L895||Italy|658[0n]
Plebejus argus|LYCAE394-15|SS11281||Denmark|676[0n]
Plebejus argus|LYCAE393-15|SS11280||Denmark|676[0n]
Plebejus argus|LYCAE403-15|SS121597||Denmark|676[0n]
Plebejus argus|LYCAE402-15|SS121596||Denmark|676[0n]
Plebejus argus|LYCAE392-15|SS11279||Denmark|676[0n]
Plebejus argus|LYCAE341-15|SS11136||Denmark|676[0n]
Plebejus argus|LYCAE342-15|SS11137||Denmark|676[0n]
Plebejus argus|LYCAE346-15|SS121901||Serbia|676[0n]
Plebejus argus|LYCAE356-15|ZT80||Serbia|676[0n]
Plebejus argus|LYCAE357-15|ZT81||Serbia|676[0n]
Plebejus argus|LYCAE358-15|ZT83||Serbia|676[0n]
Plebejus argus|LYCAE359-15|ZT86||Serbia|676[0n]
Plebejus argus|LYCAE360-15|ZT87||Serbia|676[0n]
Plebejus argus|LYCAE361-15|ZT88||Serbia|676[0n]
Plebejus argus|LYCAE362-15|ZT89||Serbia|676[0n]
Plebejus argus|LYCAE363-15|ZT90||Serbia|676[0n]
Plebejus argus|LYCAE390-15|SS11277||Denmark|676[0n]
Plebejus argus|LYCAE391-15|SS11278||Denmark|676[0n]
Plebejus argus|LYCAE395-15|SS11282||Denmark|676[0n]
Plebejus argus|LYCAE396-15|SS11283||Denmark|676[0n]
Plebejus argus|LYCAE397-15|SS11284||Denmark|676[0n]
Plebejus argus|LYCAE398-15|SS121591||Denmark|676[0n]
Plebejus argus|LYCAE399-15|SS121592||Denmark|676[0n]
Plebejus argus|LYCAE400-15|SS121593||Denmark|676[0n]
Plebejus argus|LYCAE401-15|SS121595||Denmark|676[0n]
Plebejus argus|LYCAE404-15|SS121598||Denmark|676[0n]
Plebejus argus|LYCAE405-15|SS121599||Denmark|676[0n]
Plebejus argus|LYCAE407-15|SS121954||Serbia|676[0n]
Plebejus argus|LYCAE412-15|SS121959||Serbia|676[0n]
Plebejus argus|LYCAE433-15|SS11225||Denmark|676[0n]
Plebejus argus|LYCAE434-15|SS11226||Denmark|676[0n]
Plebejus argus|LYCAE436-15|SS11231||Denmark|676[0n]
Plebejus argus|LYCAE437-15|SS11232||Denmark|676[0n]
Plebejus argus|LYCAE438-15|SS11235||Denmark|676[0n]
Plebejus argus|LYCAE439-15|SS11236||Denmark|676[0n]
Plebejus argus|LYCAE442-15|SS121076||Denmark|676[0n]
Plebejus argus|LYCAE443-15|SS12802||Denmark|676[0n]
Plebejus argus|LYCAE445-15|SS121498||Denmark|676[0n]
Plebejus argus|LYCAE446-15|SS121502||Denmark|676[0n]
Plebejus argus|LYCAE447-15|SS121505||Denmark|676[0n]
Plebejus argus|LYCAE448-15|SS121517||Denmark|676[0n]
Plebejus argus|LYCAE449-15|SS121518||Denmark|676[0n]
Plebejus argus|LYCAE459-15|ZT121||Serbia|676[0n]
Plebejus argus|LYCAE460-15|ZT176||Serbia|676[0n]
Plebejus argus|LYCAE464-15|ZT331||Serbia|676[0n]
Plebejus argus|LYCAE465-15|ZT333||Serbia|676[0n]
Plebejus argus|LYCAE466-15|ZT349||Serbia|676[0n]
Plebejus argus|EZRMN392-09|RVcoll.07-E355|Genit. examined|Romania|658[0n]
Plebejus argus|WMB889-13|RVcoll.09-X807|Genit. examined|Italy|658[0n]
Plebejus argus|WMB897-13|RVcoll.09-X839||Italy|658[0n]
Plebejus argus|WMB3823-14|RVcoll.09-X804||Italy|658[0n]
Plebejus argus|WMB4310-14|RVcoll.14-A426||Italy|658[0n]
Plebejus argus|WMB4314-14|RVcoll.14-A488||Italy|658[0n]
Plebejus argus|EULEP4494-16|RVcoll15Q128||Russia|658[0n]
Plebejus argus|EULEP5522-17|RVcoll14A071||Ukraine|658[0n]
Plebejus argus|EULEP334-14|MM23819||Estonia|658[0n]
Plebejus argus|EULEP364-14|MM23849||Lithuania|658[0n]
Plebejus argus|EULEP365-14|MM23850||Lithuania|658[0n]
Plebejus argus|EULEP525-15|RVcoll.08-H806||Sweden|658[0n]
Plebejus argus|EULEP832-15|RVcoll.14-B978||Sweden|658[0n]
Plebejus argus|EULEP2225-15|RVcoll.14-N404||Ukraine|658[0n]
Plebejus argus|BIBSA1088-15|15-L334||Italy|658[0n]
Plebejus argus|BIBSA108-15|RVcoll.14-D536||Italy|658[0n]
Plebejus argus|BIBSA156-15|RVcoll.14-I168||Italy|658[0n]
Plebejus argus|BIBSA157-15|RVcoll.14-I169||Italy|658[0n]
Plebejus argus|OXB1062-15|13-U025||Italy|658[0n]
Plebejus argus|WMB4946-14|RVcoll. 14-I493||Italy|658[0n]
Plebejus argus|WMB3824-14|RVcoll.09-X812|Genit. examined|Italy|658[0n]
Plebejus argus|LYCAE377-15|AD00P231||Russia|676[0n]
Plebejus argus|EULEP2344-15|RVcoll.14-V030||Ukraine|658[0n]
Plebejus argus|EZROM592-08|RV-07-C302||Romania|658[0n]
Plebejus argus|LEFIE771-10|MM09925||Finland|658[0n]
Plebejus argus|LEFIJ516-10|MM17141||Finland|658[0n]
Plebejus argus|GWORO795-09|BC ZSM Lep 30487||Germany|658[0n]
Plebejus argus|LEATH464-14|TLMF Lep 15676||Italy|658[0n]
Plebejus argus|LYCAE461-15|ZTUMCARI1||Serbia|676[0n]
Plebejus argus|EULEP4470-16|RVcoll15Q111||Russia|635[0n]
Plebejus argus|LYCAE352-15|SS121908||Serbia|676[0n]
Plebejus argus|LEATH465-14|TLMF Lep 15677||Italy|634[0n]
Plebejus argus|BIBSA1177-15|15-M208||Italy|658[0n]
Plebejus argus|OXB1102-15|15-A635||Italy|658[0n]
Plebejus argus|OXB1140-15|15-A673||Italy|658[0n]
Plebejus argus|OXB728-15|13-U177||Italy|658[0n]
Plebejus argus|LEATG415-14|TLMF Lep 14202||Italy|658[0n]
Plebejus argus|LEASS490-17|TLMF Lep 22138||Austria|658[0n]
Plebejus argus|EULEP1646-15|LR-08-D369||Greece|614[0n]
Plebejus argus|LYCAE462-15|ZTUMCARI5||Serbia|648[0n]
Plebejus argus|LYCAE450-15|ZT109||Serbia|676[0n]
Plebejus argus|EULEP1295-15|RVcoll.14-F727||Greece|616[0n]
Plebejus argus|LYCAE347-15|SS121903||Serbia|676[0n]
Plebejus argus|LYCAE351-15|SS121907||Serbia|676[0n]
Plebejus argus|LYCAE376-15|AD00P060||Russia|676[0n]

Plebejus argus|LYCAE347-15|SS121903||Serbia|676[0n]
Plebejus argus|LYCAE351-15|SS121907||Serbia|676[0n]
Plebejus argus|LYCAE376-15|AD00P060||Russia|676[0n]
Plebejus argus|LYCAE379-15|AD00P233||Russia|676[0n]
Plebejus argus|LYCAE406-15|SS121953||Serbia|676[0n]
Plebejus argus|LYCAE408-15|SS121955||Serbia|676[0n]
Plebejus argus|LYCAE409-15|SS121956||Serbia|676[0n]
Plebejus argus|LYCAE410-15|SS121957||Serbia|676[0n]
Plebejus argus|LYCAE411-15|SS121958||Serbia|676[0n]
Plebejus argus|LYCAE413-15|SS121960||Serbia|676[0n]
Plebejus argus|LYCAE417-15|ZTnisevac1||Serbia|676[0n]
Plebejus argus|LYCAE420-15|ZTnisevac4||Serbia|676[0n]
Plebejus argus|LYCAE421-15|ZTnisevac5||Serbia|676[0n]
Plebejus argus|LYCAE422-15|ZTnisevac7||Serbia|676[0n]
Plebejus argus|LYCAE423-15|ZTnisevac8||Serbia|676[0n]
Plebejus argus|LYCAE452-15|ZT114||Serbia|676[0n]
Plebejus argus|LYCAE454-15|ZT116||Serbia|676[0n]
Plebejus argus|LYCAE455-15|ZT117||Serbia|676[0n]
Plebejus argus|LYCAE456-15|ZT118||Serbia|676[0n]
Plebejus argus|EZROM481-08|RV-07-D442||Romania|658[0n]
Plebejus argus|EZROM482-08|RV-07-C109||Romania|658[0n]
Plebejus argus|EZROM651-08|RV-07-D545||Romania|658[0n]
Plebejus argus|EZROM652-08|RV-07-E436||Romania|658[0n]
Plebejus argus|EZROM658-08|RV-07-E516||Romania|658[0n]
Plebejus argus|EZRMN393-09|RVcoll.07-E395|Genit. examined|Romania|658[0n]
Plebejus argus|EZRMN188-08|RVcoll.08-M281||Romania|658[0n]
Plebejus argus|EULEP3606-16|RVcoll14V856||Bulgaria|658[0n]
Plebejus argus|EULEP4464-16|RVcoll10B336||Bulgaria|658[0n]
Plebejus argus|EULEP5523-17|RVcoll14A072||Ukraine|658[0n]
Plebejus argus|EULEP1575-15|RVcoll.14-G598||Macedonia|658[0n]
Plebejus argus|EULEP541-15|RVcoll.08-H899|Genit. examined|Serbia|658[0n]
Plebejus argus|EULEP1170-15|RVcoll.14-F380||Bulgaria|658[0n]
Plebejus argus|LYCAE349-15|SS121905||Serbia|676[0n]
Plebejus argus|LYCAE348-15|SS121904||Serbia|676[0n]
Plebejus argus|LYCAE350-15|SS121906||Serbia|676[1n]
Plebejus argus|LYCAE453-15|ZT115||Serbia|676[0n]
Plebejus argus|LYCAE415-15|ZT337||Serbia|676[0n]
Plebejus argus|LYCAE457-15|ZT119||Serbia|676[0n]
Plebejus argus|LYCAE458-15|ZT120||Serbia|676[0n]
Plebejus argus|EULEP1106-15|RVcoll.14-F170||Serbia|658[0n]
Plebejus argus|EZRMN187-08|RVcoll.08-M269||Romania|658[0n]
Plebejus argus|EULEP1713-15|RVcoll.14-H170||Greece|658[0n]
Plebejus argus|EZROM479-08|RV-07-D097||Romania|658[0n]
Plebejus argus|EZROM478-08|RV-07-D020||Romania|658[0n]
Plebejus argus|ABOLD018-16|TLMF Lep 21096||Austria|658[0n]
Plebejus argyrognomon|EULEP3053-15|RVcoll.14-O186|Genit. examined|Greece|658[0n]
Plebejus argyrognomon|EULEP3052-15|RVcoll.14-O185|Genit. examined|Greece|658[0n]
Plebejus idas|BIBSA310-15|RVcoll.14-E112||Italy|658[0n]
Plebejus idas|EULEP4480-16|RVcoll15J002||Switzerland|658[0n]
Plebejus idas|WMB2678-13|RVcoll.10-B891|Genit. examined|France|658[0n]
Plebejus idas|WMB3348-14|RVcoll.08-R273|Genit. examined|Spain|658[0n]
Plebejus idas|EZSPN1076-11|Rvcoll.09-V718.1|Genit. examined|Spain|658[0n]
Plebejus idas|EULEP651-15|RVcoll.14-F642||Greece|658[0n]
Plebejus idas|EULEP1533-15|RVcoll.14-G488||Greece|658[0n]
Plebejus idas|EULEP1618-15|RVcoll.14-G693||Greece|658[0n]
Plebejus idas|EULEP1256-15|RVcoll.14-F631||Greece|658[0n]
Plebejus bellieri|WMB1512-13|RVcoll.12-O113|Corsica. Genit. examined|France|658[0n]
Plebejus idas|EULEP1912-15|RVcoll.14-B810||Bosnia and Herzegovina|658[0n]
Plebejus bellieri|WMB1566-13|RVcoll.12-O467|Sardinia|Italy|658[0n]
Plebejus bellieri|WMB328-11|RVcoll.11-E623|Santa Maria|Italy|658[0n]
Plebejus bellieri|WMB1162-13|RVcoll.11-E624|Santa Maria|Italy|622[0n]
Plebejus bellieri|EULEP613-15|RVcoll.11-E915|Corsica|France|658[0n]
Plebejus bellieri|WMB363-11|RVcoll.11-E968|Corsica|France|658[0n]
Plebejus bellieri|WMB887-13|RVcoll.09-X802|Sardinia. Genit. examined|Italy|658[0n]
Plebejus argyrognomon|BIBSA064-14|LEP-SS-00064||Italy|658[0n]
Plebejus argyrognomon|BIBSA063-14|LEP-SS-00063||Italy|632[0n]
Plebejus idas|BIBSA053-14|LEP-SS-00053||Italy|658[0n]
Plebejus idas|BIBSA052-14|LEP-SS-00052||Italy|658[0n]
Plebejus argyrognomon|EULEP4990-16|RVcoll16I705|Genit. examined|Poland|658[0n]
Plebejus argyrognomon|EULEP4472-16|RVcoll15J650||France|658[0n]
Plebejus idas|WMB5279-14|RVcoll.14-A673||Italy|658[0n]
Plebejus idas|WMB6632-18|RVcoll14A007|Genit. examined|Italy|658[0n]
Plebejus bellieri|WMB331-11|RVcoll.11-E635|Spargi|Italy|658[0n]
Plebejus argyrognomon|WMB1841-13|RVcoll.11-I887|Genit. examined|France|658[0n]
Plebejus argyrognomon|WMB1659-13|RVcoll.12-P110|Genit. examined|France|658[0n]
Plebejus argyrognomon|WMB1654-13|RVcoll.12-P031|Genit. examined|France|658[0n]
Plebejus bellieri|WMB1548-13|RVcoll.12-O325|Caprera|Italy|658[0n]
Plebejus bellieri|WMB888-13|RVcoll.09-X803|Sardinia. Genit. examined|Italy|658[0n]
Plebejus bellieri|WMB867-13|RVcoll.09-T530|Corsica. Genit. examined|France|658[0n]
Plebejus bellieri|WMB1540-13|RVcoll.12-O284|La Maddalena|Italy|658[0n]
Plebejus bellieri|WMB781-13|RVcoll.06-V802|Elba|Italy|658[0n]
Plebejus bellieri|WMB780-13|RVcoll.06-V801|Elba|Italy|658[0n]
Plebejus bellieri|WMB1164-13|RVcoll.11-E636|Spargi|Italy|657[0n]
Plebejus bellieri|WMB3009-14|RVcoll.12-Q481|Sardinia|Italy|658[0n]
Plebejus bellieri|WMB2987-14|RVcoll.12-O285|La Maddalena|Italy|658[0n]
Plebejus bellieri|WMB234-11|RVcoll.09-T563|Sardinia|Italy|658[0n]
Plebejus bellieri|WMB194-11|RVcoll.06-V795|Elba|Italy|658[0n]
Plebejus argyrognomon|LEEUA658-11|MM20717||Sweden|658[0n]
Plebejus argyrognomon|GWORZ053-10|BC ZSM Lep 30409||Italy|658[0n]
Plebejus argyrognomon|GWORZ052-10|BC ZSM Lep 30408||Italy|658[0n]
Plebejus bellieri|WMB1513-13|RVcoll.12-O117|Corsica. Genit. examined|France|658[0n]
Plebejus argyrognomon|BIBSA448-15|RVcoll.14-I116||Italy|632[0n]
Plebejus argyrognomon|LEATJ1176-16|TLMF Lep 19499||Czech Republic|658[0n]
Plebejus argyrognomon|EZROM486-08|RV-07-D214|Genit. examined|Romania|658[0n]
Plebejus argyrognomon|EZRMN394-09|RVcoll.06-M953|Genit. examined|Romania|658[0n]
Plebejus argyrognomon|EZRMN191-08|RVcoll.08-M440|Genit. examined|Romania|658[0n]
Plebejus argyrognomon|EZROM487-08|RV-07-D233|Genit. examined|Romania|658[0n]
Plebejus argyrognomon|LEATJ1223-16|TLMF Lep 19546||Hungary|658[0n]
Plebejus idas|BIBSA1230-15|15-N037||Italy|658[0n]
Plebejus argyrognomon|LEASS510-17|TLMF Lep 22158||Austria|658[0n]
Plebejus argyrognomon|LEASS1026-17|TLMF Lep 22579||Austria|658[0n]
Plebejus argyrognomon|WMB2013-13|RVcoll.12-Q651|Genit. examined|Italy|658[0n]
Plebejus argyrognomon|EZRMN192-08|RVcoll.08-M238|Genit. examined|Romania|658[0n]

[illegible]
Plebejus argyrognomon|LEASS1026-17|TLMF Lep 22579||Austria|658[0n]
Plebejus argyrognomon|WMB2013-13|RVcoll.12-Q651|Genit. examined|Italy|658[0n]
Plebejus argyrognomon|EZRMN192-08|RVcoll.08-M238|Genit. examined|Romania|658[0n]
Plebejus argyrognomon|LOWA860-06|2005-LOWA-860||Russia|658[0n]
Plebejus argyrognomon|LOWA859-06|2005-LOWA-859||Russia|658[0n]
Plebejus argyrognomon|EULEP5524-17|RVcoll14A073|Genit. examined|Ukraine|658[0n]
Plebejus argyrognomon|EULEP5519-17|RVcoll14A068|Genit. examined|Ukraine|658[0n]
Plebejus argyrognomon|FBLMT888-09|BC ZSM Lep 25448||Germany|658[0n]
Plebejus argyrognomon|FBLMV715-09|BC ZSM Lep 28695||Germany|658[0n]
Plebejus argyrognomon|EZRMN193-08|RVcoll.08-M656|Genit. examined|Romania|658[0n]
Plebejus argyrognomon|EZRMN190-08|RVcoll.08-M367|Genit. examined|Romania|658[0n]
Plebejus argyrognomon|EZROM483-08|RV-07-C159|Genit. examined|Romania|658[0n]
Plebejus argyrognomon|EZROM1070-09|RVcoll.08-L362|Genit. examined|Romania|658[0n]
Plebejus argyrognomon|EULEP5537-17|RVcoll14B626|Genit. examined|Ukraine|658[0n]
Plebejus argyrognomon|EULEP5525-17|RVcoll14A074|Genit. examined|Ukraine|658[0n]
Plebejus argyrognomon|EULEP534-15|RVcoll.08-H876||Serbia|658[0n]
Plebejus argyrognomon|EULEP5538-17|RVcoll14B627|Genit. examined|Ukraine|658[0n]
Plebejus argyrognomon|EZROM485-08|RV-07-D975|Genit. examined|Romania|658[0n]
Plebejus argyrognomon|EZROM484-08|RV-07-D407|Genit. examined|Romania|658[0n]
Plebejus argyrognomon|LEASS542-17|TLMF Lep 22190||Austria|658[0n]
Plebejus argyrognomon|LEASS520-17|TLMF Lep 22168||Austria|658[0n]
Plebejus argyrognomon|EULEP2404-15|RVcoll.14-V112|Genit. examined|Ukraine|658[0n]
Plebejus argyrognomon|EULEP2403-15|RVcoll.14-V111||Ukraine|658[0n]
Plebejus argyrognomon|EZROM489-08|RV-07-E506|Genit. examined|Romania|658[0n]
Plebejus argyrognomon|EZROM488-08|RV-07-E357|Genit. examined|Romania|658[0n]
Plebejus argyrognomon|FBLMU384-09|BC ZSM Lep 27034||Germany|658[0n]
Plebejus argyrognomon|ABOLD093-16|TLMF Lep 21171||Austria|658[0n]
Plebejus argyrognomon|ABOLD649-17|TLMF Lep 21725||Austria|658[0n]
Plebejus argyrognomon|FBLMU393-09|BC ZSM Lep 27043||Germany|658[0n]
Plebejus argyrognomon|EZRMN390-09|RVcoll.07-C129|Genit. examined|Romania|658[0n]
Plebejus argyrognomon|EZROM653-08|RV-07-D310|Genit. examined|Romania|658[0n]
Plebejus argyrognomon|LEASS764-17|TLMF Lep 22412||Austria|658[0n]
Plebejus argyrognomon|LEASS765-17|TLMF Lep 22413||Austria|658[0n]
Plebejus argyrognomon|OXB590-15|14-N979|Genit. examined|Italy|658[0n]
Plebejus idas|OXB1086-15|15-A619||Italy|658[0n]
Plebejus argyrognomon|OXB1369-15|Rvcoll_15-M730|Genit. examined|France|658[0n]
Plebejus idas|LYCAE491-15|ZT121659||Bosnia and Herzegovina|676[0n]
Plebejus idas|BIBSA1225-15|15-L961||Italy|612[0n]
Plebejus argyrognomon|EULEP2362-15|RVcoll.14-V054|Genit. examined|Ukraine|621[0n]
Plebejus argyrognomon|EZRMN391-09|RVcoll.07-D382|Genit. examined|Romania|658[0n]
Plebejus argyrognomon|ABOLD419-16|TLMF Lep 21571||Austria|658[0n]
Plebejus idas|LYCAE477-15|ZT84||Serbia|676[0n]
Plebejus idas|EZSPN1105-11|Rvcoll.10-B445|Genit. examined|Romania|658[0n]
Plebejus idas|EZSPC1221-10|RVcoll.09-V545||Spain|658[0n]
Plebejus idas|EZSPC1220-10|RVcoll.09-V543|Genit. examined|Spain|658[0n]
Plebejus idas|EZSPN738-09|RVcoll.08-L151|Genit. examined|Spain|658[0n]
Plebejus idas|EZSPN662-09|RVcoll.08-J964|Genit. examined|Spain|658[0n]
Plebejus idas|LYCAE490-15|ZT121657||Bosnia and Herzegovina|676[0n]
Plebejus idas|EULEP1251-15|RVcoll.14-F611||Greece|658[0n]
Plebejus idas|EULEP1245-15|RVcoll.14-F595||Greece|658[0n]
Plebejus idas|LYCAE502-15|SS121585||Denmark|676[0n]
Plebejus idas|WMB1864-13|RVcoll.11-J195|Genit. examined|France|658[0n]
Plebejus idas|EZSPN1070-11|Rvcoll.09-T175|Genit. examined|Spain|658[0n]
Plebejus idas|EZSPC1182-10|RVcoll.09-T161|Genit. examined|Spain|658[0n]
Plebejus idas|EZSPN930-09|RVcoll.08-L885|Genit. examined|Spain|622[0n]
Plebejus idas|EZSPN921-09|RVcoll.08-L867|Genit. examined|Spain|658[0n]
Plebejus idas|LYCAE507-15|SS1113||Sweden|676[0n]
Plebejus idas|LYCAE506-15|SS1112||Sweden|676[0n]
Plebejus idas|WMB4123-14|RVcoll.13-S591|Genit. examined|Italy|658[0n]
Plebejus idas|BIBSA458-15|RVcoll.14-I126|Genit. examined|Italy|658[0n]
Plebejus idas|WMB4269-14|RVcoll.14-A170|Genit. examined|Italy|658[0n]
Plebejus idas|WMB3819-14|RVcoll.07-E117|Genit. examined|Italy|658[0n]
Plebejus idas|LEFIC057-10|MM03337||Finland|658[0n]
Plebejus idas|LYCAE524-15|SS121494||Denmark|601[1n]
Plebejus idas|EZSPM009-09|RVcoll.08-M989|Genit. examined|Andorra|634[0n]
Plebejus idas|EULEP4486-16|RVcoll15Q137|Genit. examined|Russia|658[0n]
Plebejus idas|EULEP4484-16|RVcoll15Q135|Genit. examined|Russia|635[0n]
Plebejus idas|EULEP4482-16|RVcoll15G270||Switzerland|632[0n]
Plebejus idas|WMB1619-13|RVcoll.12-O754|Genit. examined|France|658[0n]
Plebejus idas|EZROM480-08|RV-07-D113|Genit. examined|Romania|658[0n]
Plebejus idas|EZRMN422-09|RVcoll.09-V629|Genit. examined|Romania|658[0n]
Plebejus idas|EZSPC686-09|RVcoll.08-R228|Genit. examined|Spain|658[0n]
Plebejus idas|EZSPC685-09|RVcoll.08-R227|Genit. examined|Spain|658[0n]
Plebejus idas|WMB4565-14|RVcoll.090611SZ35||Spain|658[0n]
Plebejus idas|WMB881-13|RVcoll.09-X289|Genit. examined|Italy|658[0n]
Plebejus idas|WMB3295-14|RVcoll.08-M975|Genit. examined|France|658[0n]
Plebejus idas|EZSPC1180-10|RVcoll.09-T153|Genit. examined|Spain|658[0n]
Plebejus idas|EZSPC1178-10|RVcoll.09-T146|Genit. examined|Spain|658[0n]
Plebejus idas|EZSPC1144-10|RVcoll.08-P749|Genit. examined|Spain|658[0n]
Plebejus idas|EZSPM011-09|RVcoll.08-M991|Genit. examined|Andorra|658[0n]
Plebejus idas|EZROM376-08|07-E626||France|658[0n]
Plebejus idas|EZSPM119-09|RVcoll.08-P602|Genit. examined|Spain|658[0n]
Plebejus idas|EZSPC402-09|RVcoll.08-R238|Genit. examined|Spain|658[0n]
Plebejus idas|EZSPC684-09|RVcoll.08-R205||Spain|658[0n]
Plebejus idas|EZSPC683-09|RVcoll.08-R204|Genit. examined|Spain|658[0n]
Plebejus idas|LEFIC062-10|MM03346||Finland|658[0n]
Plebejus idas|LEFIC058-10|MM03338||Finland|658[0n]
Plebejus idas|GWOR3912-09|BC ZSM Lep 21196||Germany|658[0n]
Plebejus idas|GWOSK796-11|BC ZSM Lep 49012||Germany|658[0n]
Plebejus idas|GWORO797-09|BC ZSM Lep 30489||Germany|658[0n]
Plebejus idas|GWORO796-09|BC ZSM Lep 30488||Germany|658[0n]
Plebejus idas|LON872-11|NHMO Lep09070||Norway|658[0n]
Plebejus idas|LON085-08|NHMO-06085||Norway|657[0n]
Plebejus idas|ABOLD432-16|TLMF Lep 21584||Austria|658[0n]
Plebejus idas|LEATG568-14|TLMF Lep 14355||Austria|658[0n]
Plebejus idas|EULEP4479-16|RVcoll15G888||Switzerland|658[0n]
Plebejus idas|EULEP4478-16|RVcoll15G664||Switzerland|658[0n]
Plebejus idas|EULEP4474-16|RVcoll10B390||Bulgaria|658[0n]
Plebejus idas|EULEP4473-16|RVcoll10B389||Bulgaria|658[0n]
Plebejus idas|EULEP4471-16|RVcoll15G527||Switzerland|658[0n]
Plebejus idas|EULEP4468-16|RVcoll15I468||Austria|658[0n]
Plebejus idas|WMB5134-14|RVcoll.07-C039||France|658[0n]
[illegible]

Plebejus idas|EULEP4471-16|RVcoll15G527||Switzerland|658[0n]
Plebejus idas|EULEP4468-16|RVcoll15I468||Austria|658[0n]
Plebejus idas|WMB5134-14|RVcoll.07-C039||France|658[0n]
Plebejus idas|WMB4957-14|RVcoll. 14-I504|Genit. examined|Italy|658[0n]
Plebejus idas|EULEP4789-16|RVcoll16H482||Sweden|658[0n]
Plebejus idas|EULEP4481-16|RVcoll15J360||France|658[0n]
Plebejus idas|EULEP5038-16|RVcoll16J006||Slovakia|658[0n]
Plebejus idas|EULEP4803-16|RVcoll16H677||Norway|658[0n]
Plebejus idas|EULEP976-15|RVcoll.14-D091||Austria|658[0n]
Plebejus idas|EULEP793-15|RVcoll.12-Z261||Sweden|658[0n]
Plebejus idas|EULEP5520-17|RVcoll14A069|Genit. examined|Ukraine|658[0n]
Plebejus idas|EULEP4908-16|RVcoll16J611||Russia|658[0n]
Plebejus idas|EULEP5539-17|RVcoll14B628|Genit. examined|Ukraine|658[0n]
Plebejus idas|EULEP5521-17|RVcoll14A070|Genit. examined|Ukraine|658[0n]
Plebejus idas|EULEP509-15|RVcoll.07-C800||Switzerland|658[0n]
Plebejus idas|EULEP1249-15|RVcoll.14-F606||Greece|658[0n]
Plebejus idas|EULEP530-15|RVcoll.08-H839||Sweden|658[0n]
Plebejus idas|EULEP529-15|RVcoll.08-H835||Sweden|658[0n]
Plebejus idas|OXB739-15|13-U279||Italy|658[0n]
Plebejus idas|LYCAE481-15|SS121526||Denmark|676[0n]
Plebejus idas|LYCAE482-15|SS121527||Denmark|676[0n]
Plebejus idas|LYCAE483-15|SS121528||Denmark|676[0n]
Plebejus idas|LYCAE484-15|SS121531||Denmark|676[0n]
Plebejus idas|LYCAE485-15|SS121532||Denmark|676[0n]
Plebejus idas|LYCAE486-15|SS121533||Denmark|676[0n]
Plebejus idas|LYCAE487-15|SS121534||Denmark|676[0n]
Plebejus idas|LYCAE492-15|MAT99Q846||Spain|676[0n]
Plebejus idas|LYCAE493-15|SS121561||Denmark|676[0n]
Plebejus idas|LYCAE494-15|SS121564||Denmark|676[0n]
Plebejus idas|LYCAE495-15|SS121567||Denmark|676[0n]
Plebejus idas|LYCAE496-15|SS121569||Denmark|676[0n]
Plebejus idas|LYCAE498-15|SS121574||Denmark|676[0n]
Plebejus idas|LYCAE499-15|SS121575||Denmark|676[0n]
Plebejus idas|LYCAE500-15|SS121577||Denmark|676[0n]
Plebejus idas|LYCAE501-15|SS121579||Denmark|676[0n]
Plebejus idas|LYCAE505-15|SS1111||Sweden|676[0n]
Plebejus idas|LYCAE513-15|SS121074||Denmark|676[0n]
Plebejus idas|LYCAE514-15|SS121079||Denmark|676[0n]
Plebejus idas|LYCAE515-15|SS121082||Denmark|676[0n]
Plebejus idas|LYCAE527-15|SS121503||Denmark|676[0n]
Plebejus idas|LYCAE528-15|SS121508||Denmark|676[0n]
Plebejus idas|LYCAE539-15|SS121020||Denmark|676[0n]
Plebejus idas|LYCAE540-15|SS121614||Denmark|676[0n]
Plebejus idas|LYCAE541-15|SS121618||Denmark|676[0n]
Plebejus idas|LYCAE542-15|SS121623||Denmark|676[0n]
Plebejus idas|LYCAE543-15|SS121625||Denmark|676[0n]
Plebejus idas|LYCAE544-15|SS121629||Denmark|676[0n]
Plebejus idas|LYCAE545-15|SS121632||Denmark|676[0n]
Plebejus idas|LYCAE546-15|SS121642||Denmark|676[0n]
Plebejus idas|LYCAE547-15|SS121643||Denmark|676[0n]
Plebejus idas|LYCAE548-15|SS121645||Denmark|676[0n]
Plebejus idas|LYCAE549-15|SS121646||Denmark|676[0n]
Plebejus idas|LYCAE526-15|SS121497||Denmark|676[1n]
Plebejus idas|LYCAE497-15|SS121573||Denmark|674[0n]
Plebejus idas|LYCAE480-15|SS121525||Denmark|674[0n]
Plebejus idas|LYCAE479-15|SS121524||Denmark|676[0n]
Plebejus idas|LYCAE478-15|SS121520||Denmark|676[0n]
Plebejus idas|LYCAE476-15|SS11276||Denmark|676[0n]
Plebejus idas|LYCAE475-15|SS11274||Denmark|676[0n]
Plebejus idas|LYCAE474-15|SS11273||Denmark|676[0n]
Plebejus idas|LYCAE473-15|SS11272||Denmark|676[0n]
Plebejus idas|LYCAE472-15|SS11270||Denmark|676[0n]
Plebejus idas|LYCAE471-15|SS11269||Denmark|676[0n]
Plebejus idas|LYCAE470-15|SS11268||Denmark|676[0n]
Plebejus idas|LYCAE469-15|SS11266||Denmark|676[0n]
Plebejus idas|LYCAE468-15|SS11265||Denmark|676[0n]
Plebejus idas|BIBSA261-15|RVcoll.14-E058||Italy|658[0n]
Plebejus idas|BIBSA422-15|RVcoll.14-I090||Italy|658[0n]
Plebejus idas|BIBSA1174-15|15-M204||Italy|658[0n]
Plebejus idas|EULEP2223-15|RVcoll.14-N401|Genit. examined|Ukraine|658[0n]
Plebejus idas|EULEP1514-15|RVcoll.14-G435||Greece|658[0n]
Plebejus idas|EULEP2363-15|RVcoll.14-V056||Ukraine|658[0n]
Plebejus idas|BIBSA421-15|RVcoll.14-I089||Italy|658[0n]
Plebejus idas|BIBSA423-15|RVcoll.14-I091||Italy|658[0n]
Plebejus idas|BIBSA311-15|RVcoll.14-E113||Italy|658[0n]
Plebejus idas|OXB319-15|RVcoll.14-N033||Italy|658[0n]
Plebejus idas|LYCAE538-15|SS121019||Denmark|676[0n]
Plebejus idas|LYCAE537-15|SS121018||Denmark|676[0n]
Plebejus idas|LYCAE536-15|SS121016||Denmark|676[0n]
Plebejus idas|LYCAE535-15|SS121015||Denmark|676[0n]
Plebejus idas|LYCAE534-15|SS121013||Denmark|676[0n]
Plebejus idas|LYCAE533-15|SS121012||Denmark|676[0n]
Plebejus idas|LYCAE532-15|SS121010||Denmark|676[0n]
Plebejus idas|LYCAE531-15|SS121009||Denmark|676[0n]
Plebejus idas|LYCAE530-15|SS121007||Denmark|676[0n]
Plebejus idas|LYCAE529-15|SS121005||Denmark|676[0n]
Plebejus idas|LYCAE525-15|SS121493||Denmark|676[0n]
Plebejus idas|LYCAE523-15|SS121051||Denmark|676[0n]
Plebejus idas|LYCAE522-15|SS121050||Denmark|676[0n]
Plebejus idas|LYCAE521-15|SS121049||Denmark|676[0n]
Plebejus idas|LYCAE520-15|SS121048||Denmark|676[0n]
Plebejus idas|LYCAE519-15|SS121047||Denmark|676[0n]
Plebejus idas|LYCAE518-15|SS121046||Denmark|676[0n]
Plebejus idas|LYCAE517-15|SS121045||Denmark|676[0n]
Plebejus idas|LYCAE516-15|SS121044||Denmark|676[0n]
Plebejus idas|LYCAE512-15|SS12801||Denmark|676[0n]
Plebejus idas|LYCAE511-15|SS121077||Denmark|676[0n]
Plebejus idas|LYCAE510-15|SS121064||Denmark|676[0n]
Plebejus idas|LYCAE509-15|SS121063||Denmark|676[0n]
Plebejus idas|LYCAE508-15|SS121062||Denmark|676[0n]
Plebejus idas|LYCAE504-15|SS119||Sweden|676[0n]
Plebejus idas|LYCAE503-15|SS1110||Sweden|676[0n]
Plebejus idas|LYCAE489-15|SS121104||Denmark|676[0n]

Plebejus idas|LYCAE504-15|SS119||Sweden|676[0n]
Plebejus idas|LYCAE503-15|SS1110||Sweden|676[0n]
Plebejus idas|LYCAE489-15|SS121104||Denmark|676[0n]
Plebejus idas|LYCAE488-15|SS1138||Sweden|676[0n]
Plebejus idas|EULEP2471-15|RVcoll.14-V298||Switzerland|658[0n]
Plebejus idas|EULEP2391-15|RVcoll.14-V094||Ukraine|658[0n]
Plebejus idas|PHLAH696-12|TLMF Lep 08515||Austria|658[0n]
Plebejus idas|LEATD299-13|TLMF Lep 12946||Italy|658[0n]
Plebejus idas|LEATD138-13|TLMF Lep 12785||Italy|638[0n]
Plebejus idas|LEATG099-14|TLMF Lep 13886||Austria|658[0n]
Neolysandra coelestina|EULEP2584-15|RVcoll.14-V591||Russia|658[0n]
Neolysandra coelestina|EULEP1810-15|RVcoll.14-H785||Greece|658[0n]
Neolysandra coelestina|EULEP1793-15|RVcoll.14-H664||Greece|658[0n]
Neolysandra coelestina|EULEP1792-15|RVcoll.14-H662||Greece|658[0n]
Neolysandra coelestina|EULEP1791-15|RVcoll.14-H659||Greece|658[0n]
Neolysandra coelestina|EULEP1790-15|RVcoll.14-H658||Greece|658[0n]
Neolysandra coelestina|EULEP572-15|RVcoll.14-F791||Greece|658[0n]
Neolysandra coelestina|EULEP4325-16|RVcoll14A938||Russia|658[0n]
Neolysandra coelestina|EULEP4324-16|RVcoll14A937||Russia|658[0n]
Lysandra bellargus|EZSPM711-12|Rvcoll. 12-L597||Portugal|620[0n]
Lysandra bellargus|WMB3305-14|RVcoll.08-P472||Spain|620[0n]
Lysandra bellargus|EZSPC954-10|RVcoll.09-X532||Spain|617[0n]
Lysandra bellargus|EZSPN458-09|RVcoll.08-H665||Spain|658[0n]
Lysandra bellargus|EZSPM922-12|RVcoll.12-M644||Spain|641[0n]
Lysandra bellargus|EZSPC514-09|RVcoll.06-G560||Spain|658[0n]
Lysandra bellargus|EZROM733-08|RV-06-G452||Spain|658[0n]
Lysandra bellargus|EZSPC516-09|RVcoll.08-L439||Spain|658[0n]
Lysandra bellargus|EZSPC515-09|RVcoll.08-R282||Spain|658[0n]
Lysandra bellargus|EZSPM173-09|RVcoll.08-R036||Spain|658[0n]
Lysandra bellargus|EZSPC822-10|RVcoll.08-P722||Spain|658[0n]
Lysandra bellargus|EZSPC730-10|RVcoll.08-H666||Spain|658[0n]
Lysandra bellargus|EZSPC748-10|RVcoll.08-L071||Spain|658[0n]
Lysandra bellargus|EZSPC726-10|RVcoll.08-H581||Spain|634[0n]
Lysandra bellargus|EZSPM269-09|RVcoll.08-R486||Spain|658[0n]
Lysandra bellargus|EZSPN390-09|RVcoll.08-H444||Spain|658[0n]
Lysandra bellargus|EZSPN356-09|RVcoll.08-H331||Spain|658[0n]
Lysandra bellargus|EZSPN689-09|RVcoll.08-L019||Spain|658[0n]
Lysandra bellargus|EZSPN134-09|RVcoll.06-G483||Spain|658[0n]
Lysandra bellargus|WMB1968-13|RVcoll.11-I185||Italy|658[0n]
Lysandra bellargus|EULEP1063-15|RVcoll.14-E966||Serbia|658[0n]
Lysandra bellargus|WMB3458-14|RVcoll.10-A585||France|658[0n]
Lysandra bellargus|WMB6565-18|RVcoll13U058||Italy|658[0n]
Lysandra bellargus|WMB2465-13|RVcoll.11-H365||Italy|658[0n]
Lysandra bellargus|WMB2461-13|RVcoll.11-H348||Italy|658[0n]
Lysandra bellargus|WMB2221-13|RVcoll.11-Y047||Italy|658[0n]
Lysandra bellargus|EULEP2132-15|RVcoll.14-J972||Switzerland|658[0n]
Lysandra bellargus|EULEP627-15|RVcoll.11-J011||Switzerland|658[0n]
Lysandra bellargus|EULEP4189-16|RVcoll15G532||Switzerland|658[0n]
Lysandra bellargus|EULEP4186-16|RVcoll15C253||Italy|658[0n]
Lysandra bellargus|WMB5292-14|RVcoll.14-I589||France|658[0n]
Lysandra bellargus|WMB5271-14|RVcoll.14-A573||Italy|658[0n]
Lysandra bellargus|WMB5237-14|RVcoll.13-U087||Italy|658[0n]
Lysandra bellargus|WMB5198-14|RVcoll.13-T914||Italy|658[0n]
Lysandra bellargus|WMB4911-14|RVcoll.14-I458||Italy|658[0n]
Lysandra bellargus|WMB6585-18|RVcoll11I281||Italy|658[0n]
Lysandra bellargus|WMB2126-13|RVcoll.12-R233||Italy|658[0n]
Lysandra bellargus|WMB1796-13|RVcoll.12-Q315||France|658[0n]
Lysandra bellargus|WMB5171-14|RVcoll.13-S516||Italy|658[0n]
Lysandra bellargus|WMB5135-14|RVcoll.07-E054||Italy|658[0n]
Lysandra bellargus|WMB5017-14|RVcoll. 14-I564||Italy|658[0n]
Lysandra bellargus|WMB4948-14|RVcoll. 14-I495||Italy|658[0n]
Lysandra bellargus|WMB109-11|RVcoll.10-B631||France|658[0n]
Lysandra bellargus|WMB201-11|RVcoll.07-W062||France|658[0n]
Lysandra bellargus|EZROM497-08|RV-06-M938||Romania|658[0n]
Lysandra bellargus|GWORR410-10|BC ZSM Lep 29626||Italy|658[0n]
Lysandra bellargus|GWORR409-10|BC ZSM Lep 29625||Italy|658[0n]
Lysandra bellargus|GWORR408-10|BC ZSM Lep 29624||Italy|658[0n]
Lysandra bellargus|BIBSA1332-15|15-M824||Italy|658[0n]
Lysandra bellargus|BIBSA104-15|RVcoll.14-D527||Italy|658[0n]
Lysandra bellargus|OXB706-15|13-T884||Italy|658[0n]
Lysandra bellargus|BIBSA1877-17|RVcoll16C778||Italy|658[0n]
Lysandra bellargus|BIBSA597-15|LEP-SS-00217||Italy|658[0n]
Lysandra bellargus|BIBSA596-15|LEP-SS-00216||Italy|658[0n]
Lysandra bellargus|BIBSA595-15|LEP-SS-00215||Italy|658[0n]
Lysandra bellargus|BIBSA239-15|RVcoll.14-E031||Italy|658[0n]
Lysandra bellargus|BIBSA1841-17|RVcoll16C718||Italy|658[0n]
Lysandra bellargus|BIBSA1352-15|15-M852||Italy|658[0n]
Lysandra bellargus|BIBSA623-15|LEP-SS-00243||Italy|658[0n]
Lysandra bellargus|BIBSA408-15|RVcoll.14-I076||Italy|658[0n]
Lysandra bellargus|OXB782-15|14-A756||Italy|658[0n]
Lysandra bellargus|OXB717-15|13-U105||Italy|658[0n]
Lysandra bellargus|OXB939-15|15-A583||Italy|658[0n]
Lysandra bellargus|WMB1701-13|RVcoll.12-P619||France|658[0n]
Lysandra bellargus|WMB3429-14|RVcoll.09-X278||France|658[0n]
Lysandra bellargus|EZSPC517-09|RVcoll.08-M925||Spain|658[0n]
Lysandra bellargus|ABOLD062-16|TLMF Lep 21140||Austria|658[0n]
Lysandra bellargus|ODOPE245-11|BC ZSM Lep 53116||Germany|658[0n]
Lysandra bellargus|PHLAW020-13|TLMF Lep 09817||Austria|658[0n]
Lysandra bellargus|LEATD298-13|TLMF Lep 12945||Italy|637[0n]
Lysandra corydonius|EULEP4202-16|RVcoll14V607||Russia|622[0n]
Lysandra corydonius|EULEP4201-16|RVcoll14V606||Russia|658[0n]
Lysandra corydonius|EULEP4200-16|RVcoll14V605||Russia|641[0n]
Lysandra corydonius|EULEP4199-16|RVcoll14V604||Russia|658[0n]
Lysandra bellargus|OXB242-15|OXB-TGS-637||United Kingdom|658[0n]
Lysandra bellargus|OXB241-15|OXB-TGS-636||United Kingdom|658[0n]
Lysandra bellargus|OXB204-15|OXB-TGS-186||United Kingdom|658[0n]
Lysandra bellargus|EULEP2941-15|RVcoll.14-O074||France|658[0n]
Lysandra coridon|WMB4344-14|RVcoll.14-A826||Italy|658[0n]
Lysandra coridon|OXB772-15|14-A712||Italy|614[0n]
Lysandra coridon|WMB2052-13|RVcoll.12-Q802||Italy|658[0n]
Lysandra coridon|GWORU348-10|BC ZSM Lep 31939||Italy|658[0n]
Lysandra coridon|GWORR413-10|BC ZSM Lep 29629||Italy|658[0n]
[illegible]

Lysandra coridon|WMB2052-13|RVcoll.12-Q802||Italy|658[0n]
Lysandra coridon|GWORU348-10|BC ZSM Lep 31939||Italy|658[0n]
Lysandra coridon|GWORR413-10|BC ZSM Lep 29629||Italy|658[0n]
Lysandra coridon|OXB1532-16|OXB-TGS-1250||United Kingdom|658[0n]
Lysandra coridon|EULEP556-15|RVcoll.08-M182|Corsica|France|658[0n]
Lysandra coridon|WMB2593-13|RVcoll.12-R230||Italy|658[0n]
Lysandra coridon|EULEP2943-15|RVcoll.14-O076|Corsica|France|658[0n]
Lysandra coridon|EULEP557-15|RVcoll.08-M183|Corsica|France|658[0n]
Lysandra coridon|WMB2044-13|RVcoll.12-Q776||Italy|658[0n]
Lysandra coridon|OXB238-15|OXB-TGS-516||United Kingdom|658[0n]
Lysandra coridon|OXB1533-16|OXB-TGS-1251||United Kingdom|658[0n]
Lysandra coridon|OXB284-15|OXB-TGS-967||United Kingdom|653[0n]
Lysandra coridon|OXB243-15|OXB-TGS-640||United Kingdom|658[0n]
Lysandra coridon|WMB1703-13|RVcoll.12-P628||France|658[0n]
Lysandra coridon|LEATJ1279-16|TLMF Lep 19602||Czech Republic|600[0n]
Lysandra coridon|EZSPC811-10|RVcoll.08-P558||Spain|658[0n]
Lysandra coridon|EZSPN190-09|RVcoll.07-C273||Spain|658[0n]
Lysandra coridon|EZSPC810-10|RVcoll.08-P557||Spain|658[0n]
Lysandra coridon|EZSPN189-09|RVcoll.07-C272||Spain|658[1n]
Lysandra coridon|EZROM279-08|RV-07-E440|Genit. examined|Romania|658[0n]
Lysandra bellargus|OXB1557-16|OXB-TGS-1275||France|658[0n]
Lysandra bellargus|EZROM636-08|RV-07-D529||Romania|658[0n]
Lysandra bellargus|EZROM496-08|RV-06-M933||Romania|658[0n]
Lysandra bellargus|FBLMU255-09|BC ZSM Lep 25765||Germany|658[0n]
Lysandra bellargus|EULEP2384-15|RVcoll.14-V079||Ukraine|658[0n]
Lysandra bellargus|LOWA764-06|2005-LOWA-764||Ukraine|658[0n]
Lysandra bellargus|LOWA762-06|2005-LOWA-762||Ukraine|658[0n]
Lysandra bellargus|EZROM274-08|RV-07-D149||Romania|658[0n]
Lysandra bellargus|EZRMN069-08|RVcoll.08-M560||Romania|658[0n]
Lysandra bellargus|EULEP2210-15|RVcoll.14-N385||Ukraine|658[0n]
Lysandra bellargus|EULEP4185-16|RVcoll10A979||Bulgaria|658[0n]
Lysandra bellargus|EZRMN068-08|RVcoll.08-M388|Genit. examined|Romania|658[0n]
Lysandra bellargus|EZRMN067-08|RVcoll.07-D497|Genit. examined|Romania|658[0n]
Lysandra bellargus|OXB358-15|RVcoll.14-N072||Italy|658[0n]
Lysandra bellargus|LEASS687-17|TLMF Lep 22335||Austria|658[0n]
Lysandra bellargus|LEASS914-17|KLM Lep 08419||Austria|658[0n]
Lysandra bellargus|GBLAA1392-15|BC ZSM Lep 87088||Germany|658[0n]
Lysandra bellargus|EULEP1666-15|RVcoll.14-G890||Greece|658[0n]
Lysandra bellargus|EULEP1494-15|RVcoll.14-G328||Greece|658[0n]
Lysandra bellargus|EULEP1227-15|RVcoll.14-F530||Greece|658[0n]
Lysandra bellargus|EULEP5078-16|RVcoll16J300||Germany|658[0n]
Lysandra bellargus|EULEP5061-16|RVcoll16J112||Czech Republic|658[0n]
Lysandra bellargus|EULEP4188-16|RVcoll15G458||Switzerland|658[0n]
Lysandra bellargus|LOWA763-06|2005-LOWA-763||Ukraine|658[0n]
Lysandra bellargus|EZROM678-08|RV-07-D948||Romania|658[0n]
Lysandra bellargus|EZROM275-08|RV-07-D435||Romania|658[0n]
Lysandra bellargus|EZRMN070-08|RVcoll.08-M563||Romania|658[0n]
Lysandra bellargus|FBLMT406-09|BC ZSM Lep 24966||Germany|658[0n]
Lysandra bellargus|FBLMV428-09|BC ZSM Lep 28408||Germany|658[0n]
Lysandra bellargus|GWORO801-09|BC ZSM Lep 30493||Germany|658[0n]
Lysandra bellargus|GWORO800-09|BC ZSM Lep 30492||Germany|658[0n]
Lysandra bellargus|LEATD643-13|TLMF Lep 13290||Austria|658[0n]
Lysandra coridon|EULEP2942-15|RVcoll.14-O075||Italy|658[0n]
Lysandra coridon|EULEP684-15|RVcoll.11-J875|Sardinia|Italy|658[0n]
Lysandra coridon|WMB1891-13|RVcoll.11-J882|Sardinia|Italy|658[0n]
Lysandra albicans|WMB3641-14|RVcoll.11-I541||Spain|658[0n]
Lysandra coridon|GBMIN16338-13|JX678094||Spain|657[0n]
Lysandra albicans|EZSPM441-09|Rvcoll.09-V565||Spain|658[0n]
Lysandra bellargus|GBLAA1466-15|BC ZSM Lep 87257||Germany|658[0n]
Lysandra bellargus|GBLAA1455-15|BC ZSM Lep 87246||Germany|658[0n]
Lysandra coridon|EZSPC522-09|RVcoll.08-R210||Spain|658[0n]
Lysandra coridon|EZSPC518-09|RVcoll.06-V740||Spain|658[0n]
Lysandra bellargus|EULEP4187-16|RVcoll15H606||Switzerland|658[0n]
Lysandra bellargus|LEATD467-13|TLMF Lep 13114||Italy|658[0n]
Lysandra bellargus|PHLAI510-13|TLMF Lep 09072||Austria|658[0n]
Lysandra coridon|LEASS529-17|TLMF Lep 22177||Austria|658[0n]
Lysandra coridon|EULEP4197-16|RVcoll15Q073||Russia|635[0n]
Lysandra coridon|EULEP2102-15|RVcoll.14-J591||Ukraine|658[0n]
Lysandra coridon|LEASS532-17|TLMF Lep 22180||Austria|658[0n]
Lysandra coridon|LEASS531-17|TLMF Lep 22179||Austria|658[0n]
Lysandra coridon|EULEP2175-15|RVcoll.14-N210||Greece|658[0n]
Lysandra coridon|EULEP1539-15|RVcoll.14-G504||Greece|658[0n]
Lysandra coridon|LOWA284-06|2005-LOWA-284||Russia|658[0n]
Lysandra coridon|EULEP4995-16|RVcoll16I737||Poland|633[0n]
Lysandra coridon|EULEP5066-16|RVcoll16J138||Czech Republic|658[0n]
Lysandra coridon|EULEP4198-16|RVcoll15Q075||Russia|658[0n]
Lysandra coridon|EZROM282-08|RV-07-C175||Romania|658[0n]
Lysandra coridon|EZRMN073-08|RVcoll.07-E407||Romania|658[0n]
Lysandra coridon|EZRMN072-08|RVcoll.07-E373||Romania|658[0n]
Lysandra coridon|EZROM277-08|RV-07-E402||Romania|658[0n]
Lysandra coridon|EZROM281-08|RV-07-C174|Genit. examined|Romania|658[0n]
Lysandra coridon|EZRMN074-08|RVcoll.07-E563||Romania|658[0n]
Lysandra coridon|LOWA283-06|2005-LOWA-283||Russia|658[0n]
Lysandra coridon|EULEP4196-16|RVcoll15Q183||Ukraine|658[0n]
Lysandra coridon|EULEP2402-15|RVcoll.14-V110||Ukraine|658[0n]
Lysandra coridon|EULEP965-15|RVcoll.14-D032||Bulgaria|658[0n]
Lysandra coridon|EZROM280-08|RV-07-E559||Romania|655[0n]
Lysandra coridon|EZROM278-08|RV-07-E439||Romania|658[0n]
Lysandra coridon|EZROM276-08|RV-07-E305||Romania|658[0n]
Lysandra coridon|EZRMN071-08|RVcoll.06-V713|Genit. examined|Romania|658[0n]
Lysandra coridon|ABOLD031-16|TLMF Lep 21109||Austria|658[0n]
Lysandra coridon|EZSPC521-09|RVcoll.06-A147||Spain|609[0n]
Lysandra coridon|EZSPC519-09|RVcoll.06-V744||Spain|609[0n]
Lysandra coridon|EZSPN106-09|RVcoll.06-A185||Andorra|658[0n]
Lysandra coridon|WMB3119-14|RVcoll.06-A174||Spain|658[0n]
Lysandra coridon|EZSPN507-09|RVcoll.08-H965||Spain|658[0n]
Lysandra coridon|OXB1558-16|OXB-TGS-1276||France|658[0n]
Lysandra coridon|OXB1559-16|OXB-TGS-1277||France|658[0n]
Lysandra coridon|EZSPC520-09|RVcoll.06-A127||Spain|633[0n]
Lysandra coridon|EZSPN105-09|RVcoll.06-A169||Spain|658[0n]
Lysandra coridon|EZSPN104-09|RVcoll.06-A166||Spain|658[0n]
Lysandra coridon|EZSPN103-09|RVcoll.06-A165||Spain|658[0n]
Lysandra coridon|EZSPM881-12|Rvcoll.150511SD05||Spain|658[0n]

Lysandra coridon|EZSPN104-09|RVcoll.06-A166||Spain|658[0n]
Lysandra coridon|EZSPN103-09|RVcoll.06-A165||Spain|658[0n]
Lysandra coridon|EZSPM881-12|Rvcoll.150511SD05||Spain|658[0n]
Lysandra coridon|EZSPC955-10|RVcoll.09-X545||Spain|606[0n]
Lysandra coridon|EZSPM162-09|RVcoll.08-P690||Spain|658[0n]
Lysandra coridon|EZSPM089-09|RVcoll.08-P413||Spain|658[0n]
Lysandra coridon|EZSPM087-09|RVcoll.08-P410||Spain|658[0n]
Lysandra coridon|EZSPM298-09|RVcoll.08-J845||Spain|658[0n]
Lysandra coridon|WMB3445-14|RVcoll.09-X573||Spain|602[0n]
Lysandra coridon|EZSPM148-09|RVcoll.08-P662||Spain|658[0n]
Lysandra coridon|EZSPN787-09|RVcoll.08-L345||Spain|658[0n]
Lysandra coridon|EZSPN786-09|RVcoll.08-L344||Spain|658[0n]
Lysandra hispana|EZSPN780-09|RVcoll.08-L292||Spain|658[0n]
Lysandra hispana|WMB3475-14|RVcoll.10-A858||Spain|658[0n]
Lysandra hispana|EZSPN206-09|RVcoll.07-F050||Spain|658[0n]
Lysandra coridon|EULEP5869-18|RVcoll15O487||Spain|658[0n]
Lysandra albicans|EZSPM440-09|Rvcoll.09-V499||Spain|658[0n]
Lysandra hispana|EZSPN252-09|RVcoll.07-W150||Spain|658[0n]
Lysandra albicans|EZSPM580-12|RVcoll.08-P938||Spain|658[0n]
Lysandra hispana|EZSPN207-09|RVcoll.07-F056||Spain|658[0n]
Lysandra albicans|EZSPM581-12|RVcoll.08-P939||Spain|658[0n]
Lysandra|EZSPN970-09|RVcoll.08-L990|potential hybrid (caerulescens)|Spain|658[0n]
Lysandra albicans|WMB4450-14|RVcoll.14-D314||Spain|658[0n]
Lysandra hispana|EZSPM342-09|RVcoll.08-R006||Spain|658[0n]
Lysandra albicans|WMB4466-14|RVcoll.14-D344||Spain|658[0n]
Lysandra albicans|WMB4451-14|RVcoll.14-D315||Spain|658[0n]
Lysandra albicans|EZSPM859-12|Rvcoll.040611HZ81||Spain|658[0n]
Lysandra|EZSPC1274-10|RVcoll.070207MC50|potential hybrid (caerulescens)|Spain|658[0n]
Lysandra hispana|EZSPN773-09|RVcoll.08-L282||Spain|658[0n]
Lysandra albicans|EZSPN1077-11|Rvcoll.09-V944||Spain|658[0n]
Lysandra albicans|EZSPM109-09|RVcoll.08-P449||Spain|658[0n]
Lysandra albicans|EZSPM030-09|RVcoll.08-J813||Spain|658[0n]
Lysandra albicans|EZSPM126-09|RVcoll.08-P618||Spain|658[0n]
Lysandra albicans|WMB3267-14|RVcoll.08-L869.1||Spain|658[0n]
Lysandra albicans|WMB3271-14|RVcoll.08-L930||Spain|658[0n]
Lysandra albicans|EZSPN177-09|RVcoll.06-K748||Spain|658[0n]
Lysandra hispana|EZSPN766-09|RVcoll.08-L265||Spain|658[0n]
Lysandra hispana|EZSPN553-09|RVcoll.08-J065||Spain|658[0n]
Lysandra albicans|WMB3331-14|RVcoll.08-P766||Spain|658[0n]
Lysandra albicans|EZSPM576-12|RVcoll.08-P933||Spain|658[0n]
Lysandra albicans|EZSPM578-12|RVcoll.08-P936||Spain|658[0n]
Lysandra hispana|EZSPN777-09|RVcoll.08-L287||Spain|658[0n]
Lysandra caelestissima|EZSPN1109-11|Rvcoll.10-B528||Spain|658[0n]
Lysandra caelestissima|EZSPM110-09|RVcoll.08-P450||Spain|658[0n]
Lysandra caelestissima|EZSPM118-09|RVcoll.08-P601||Spain|658[0n]
Lysandra caelestissima|EZSPN285-09|RVcoll.07-W230||Spain|658[0n]
Lysandra caelestissima|EZSPN765-09|RVcoll.08-L254||Spain|658[0n]
Lysandra|EZSPN761-09|RVcoll.08-L242|potential hybrid (caerulescens)|Spain|658[0n]
Lysandra albicans|GBMIN41679-14|KF834395||Spain|1221[0n]
Lysandra albicans|EZSPN679-09|RVcoll.08-J992||Spain|658[0n]
Lysandra hispana|WMB3216-14|RVcoll.08-J678||Spain|658[0n]
Lysandra albicans|EZSPN764-09|RVcoll.08-L249||Spain|658[0n]
Lysandra hispana|EZSPN771-09|RVcoll.08-L280||Spain|658[0n]
Lysandra hispana|WMB264-11|RVcoll.10-A418||France|658[0n]
Lysandra hispana|GBGLL644-14|KF834369||Spain|1495[28n]
Lysandra hispana|EULEP1966-15|RVcoll.14-G830||Spain|658[0n]
Lysandra hispana|EZSPM857-12|Rvcoll.040611HZ30||Spain|658[0n]
Lysandra coridon|BIBSA241-15|RVcoll.14-E034||Italy|658[0n]
Lysandra coridon|EULEP4194-16|RVcoll15I693||Austria|658[0n]
Lysandra coridon|WMB4497-14|RVcoll.14-E173||Italy|658[0n]
Lysandra coridon|BIBSA306-15|RVcoll.14-E108||Italy|658[0n]
Lysandra coridon|BIBSA240-15|RVcoll.14-E033||Italy|658[0n]
Lysandra coridon|WMB1857-13|RVcoll.11-I996||France|658[0n]
Lysandra hispana|WMB3845-14|RVcoll.10-C709||Italy|658[0n]
Lysandra coridon|BIBSA428-15|RVcoll.14-I096||Italy|658[0n]
Lysandra coridon|BIBSA410-15|RVcoll.14-I078||Italy|614[0n]
Lysandra coridon|BIBSA409-15|RVcoll.14-I077||Italy|658[0n]
Lysandra coridon|EULEP4190-16|RVcoll15H263||Switzerland|658[0n]
Lysandra hispana|WMB1663-13|RVcoll.12-P207||France|658[0n]
Lysandra coridon|WMB3667-14|RVcoll.11-I914||France|658[0n]
Lysandra hispana|WMB3649-14|RVcoll.11-I623||France|658[0n]
Lysandra hispana|WMB110-11|RVcoll.10-B632||France|658[0n]
Lysandra coridon|WMB1885-13|RVcoll.11-J748||Italy|658[0n]
Lysandra coridon|WMB3985-14|RVcoll.12-Q272||France|658[0n]
Lysandra coridon|WMB3991-14|RVcoll.12-Q366||France|658[0n]
Lysandra coridon|WMB4989-14|RVcoll. 14-I536||Italy|658[0n]
Lysandra coridon|WMB5264-14|RVcoll.14-A473||Italy|658[0n]
Lysandra coridon|BIBSA943-15|16-A034||Italy|658[0n]
Lysandra coridon|WMB1723-13|RVcoll.12-P811||France|658[0n]
Lysandra coridon|WMB3514-14|RVcoll.10-B861||France|658[0n]
Lysandra coridon|EULEP4195-16|RVcoll15J413||France|638[0n]
Lysandra coridon|LEATD407-13|TLMF Lep 13054||Austria|658[0n]
Lysandra coridon|FBLMW313-10|BC ZSM Lep 37414||Germany|658[0n]
Lysandra coridon|FBLMW314-10|BC ZSM Lep 37415||Germany|658[0n]
Lysandra coridon|GWORO802-09|BC ZSM Lep 30494||Germany|658[0n]
Lysandra coridon|GWORA2453-09|BC ZSM Lep 30665||Germany|658[0n]
Lysandra coridon|GWOSK794-11|BC ZSM Lep 49010||Germany|658[0n]
Lysandra coridon|GWOSK860-11|BC ZSM Lep 49361||Germany|658[0n]
Lysandra coridon|WMB1086-13|RVcoll.10-C741||Italy|658[0n]
Lysandra coridon|WMB5034-14|RVcoll. 14-I581||Italy|658[0n]
Lysandra coridon|EULEP4192-16|RVcoll15H797||Italy|658[0n]
Lysandra coridon|EULEP4193-16|RVcoll15I027||Italy|658[0n]
Lysandra coridon|EULEP1857-15|RVcoll.13-U209||Italy|658[0n]
Lysandra coridon|BIBSA1180-15|15-M215||Italy|658[0n]
Lysandra coridon|BIBSA1081-15|15-K552||Italy|658[0n]
Lysandra hispana|BIBSA102-15|RVcoll.14-D525||Italy|658[0n]
Lysandra coridon|OXB627-15|14-O016||Italy|658[0n]
Lysandra coridon|OXB334-15|RVcoll.14-N048||Italy|658[0n]
Lysandra hispana|BIBSA103-15|RVcoll.14-D526||Italy|634[0n]
Lysandra hispana|WMB2260-13|RVcoll.12-R421||Italy|613[0n]
Lysandra coridon|LEATD148-13|TLMF Lep 12795||Italy|606[0n]
Lysandra coridon|WMB4850-14|RVcoll.14-I397||Italy|658[0n]
Lysandra coridon|WMB3994-14|RVcoll.12-Q401||France|658[0n]

[illegible]
Lysandra coridon|LEATD148-13|TLMF Lep 12795||Italy|606[0n]
Lysandra coridon|WMB4850-14|RVcoll.14-I397||Italy|658[0n]
Lysandra coridon|WMB3994-14|RVcoll.12-Q401||France|658[0n]
Lysandra coridon|WMB3946-14|RVcoll.12-P701||France|658[0n]
Lysandra coridon|PHLAH465-12|TLMF Lep 08284||Austria|658[0n]
Lysandra coridon|EULEP4191-16|RVcoll15H757||Switzerland|658[0n]
Lysandra coridon|EULEP2461-15|RVcoll.14-V286||Switzerland|658[0n]
Lysandra coridon|LEATH671-14|TLMF Lep 15883||Italy|634[0n]
Lysandra hispana|WMB3472-14|RVcoll.10-A657||France|614[0n]
Lysandra coridon|LEATD408-13|TLMF Lep 13055||Austria|647[0n]
Polyommatus amandus|EULEP4724-16|RVcoll16G383||Denmark|658[0n]
Polyommatus amandus|EZSPM664-12|RVcoll.11-I580||Spain|658[0n]
Polyommatus amandus|EZSPM663-12|RVcoll.11-I579||Spain|658[0n]
Polyommatus amandus|EULEP1635-15|RVcoll.14-H981||Romania|658[0n]
Polyommatus amandus|EULEP4502-16|RVcoll15C953||Romania|658[0n]
Polyommatus amandus|EULEP1051-15|RVcoll.14-E923||Serbia|658[0n]
Polyommatus amandus|EULEP549-15|RVcoll.08-L313||Sweden|658[0n]
Polyommatus amandus|EULEP4795-16|RVcoll16H557||Sweden|658[0n]
Polyommatus amandus|EZSPN740-09|RVcoll.08-L162||Spain|658[0n]
Polyommatus amandus|OLEP090-15|RVcoll.14-V664||Spain|658[0n]
Polyommatus amandus|WMB4545-14|RVcoll.030111FB07||Spain|658[0n]
Polyommatus amandus|EZSPN720-09|RVcoll.08-L109||Spain|657[0n]
Polyommatus amandus|LEFIC064-10|MM03348||Finland|658[0n]
Polyommatus amandus|EZSPN255-09|RVcoll.07-W155||Spain|658[0n]
Polyommatus amandus|EZSPC908-10|RVcoll.09-V774||Spain|658[0n]
Polyommatus amandus|EZSPC656-09|RVcoll.07-C664||Spain|658[0n]
Polyommatus amandus|EZSPC660-09|RVcoll.07-W117||Spain|658[0n]
Polyommatus amandus|EZSPC701-10|RVcoll.07-C672||Spain|658[0n]
Polyommatus amandus|WMB5025-14|RVcoll. 14-I572||Italy|658[0n]
Polyommatus amandus|EULEP4504-16|RVcoll15H585||Switzerland|658[0n]
Polyommatus amandus|EULEP4897-16|RVcoll16J600||Russia|658[0n]
Polyommatus amandus|EULEP5870-18|RVcoll15O506||Spain|658[0n]
Polyommatus amandus|EULEP1262-15|RVcoll.14-F652||Greece|658[0n]
Polyommatus amandus|EULEP1918-15|RVcoll.14-B841||Bosnia and Herzegovina|658[0n]
Polyommatus amandus|OLEP083-15|RVcoll.14-V657||Spain|658[0n]
Polyommatus amandus|OLEP084-15|RVcoll.14-V658||Spain|658[0n]
Polyommatus amandus|EZSPN895-09|RVcoll.08-L806||Spain|658[0n]
Polyommatus amandus|EZSPN880-09|RVcoll.08-L785||Spain|622[1n]
Polyommatus amandus|EULEP1145-15|RVcoll.14-F314||Serbia|628[0n]
Polyommatus amandus|EZSPC680-09|RVcoll.08-P371||Spain|658[0n]
Polyommatus amandus|LEFIJ523-10|MM17148||Finland|658[0n]
Polyommatus amandus|GWORA2460-09|BC ZSM Lep 30672||Germany|658[0n]
Polyommatus amandus|LEFIB996-10|MM03194||Finland|658[0n]
Polyommatus amandus|LEATI057-15|TLMF Lep 17442||Austria|658[0n]
Polyommatus amandus|PHLAI503-13|TLMF Lep 09065||Austria|658[0n]
Polyommatus amandus|LEATD457-13|TLMF Lep 13104||Italy|658[0n]
Polyommatus amandus|LEATG233-14|TLMF Lep 14020||Austria|658[0n]
Polyommatus amandus|FBLMW310-10|BC ZSM Lep 37411||Germany|658[4n]
Polyommatus amandus|EULEP1800-15|RVcoll.14-H689||Greece|658[0n]
Polyommatus amandus|EULEP1324-15|RVcoll.14-F798||Greece|658[0n]
Polyommatus amandus|EULEP722-15|RVcoll.12-N795|Lesvos|Greece|658[0n]
Polyommatus amandus|BIBSA067-14|LEP-SS-00067||Italy|658[0n]
Polyommatus amandus|BIBSA066-14|LEP-SS-00066||Italy|658[0n]
Polyommatus amandus|EULEP2370-15|RVcoll.14-V065||Ukraine|613[1n]
Polyommatus amandus|EULEP589-15|RVcoll.10-A712||Estonia|658[0n]
Polyommatus amandus|EULEP368-14|MM23853||Lithuania|658[0n]
Polyommatus amandus|WMB5142-14|RVcoll.09-X953||Italy|658[0n]
Polyommatus amandus|EZROM495-08|RV-07-D035||Romania|658[0n]
Polyommatus amandus|EULEP2169-15|RVcoll.14-K092||Switzerland|625[0n]
Polyommatus amandus|ABOLD648-17|TLMF Lep 21724||Austria|658[0n]
Polyommatus amandus|GWOSI558-10|BC ZSM Lep 44404||Germany|658[0n]
Polyommatus amandus|ABOLD060-16|TLMF Lep 21138||Austria|658[0n]
Polyommatus amandus|EULEP4503-16|RVcoll15H074||Italy|658[0n]
Polyommatus amandus|BIBSA1087-15|15-K576||Italy|658[1n]
Polyommatus amandus|BIBSA1351-15|15-M850||Italy|658[0n]
Polyommatus amandus|BIBSA1000-15|15-C041||Italy|658[0n]
Polyommatus amandus|EULEP4501-16|RVcoll14V314||Italy|658[0n]
Polyommatus amandus|WMB5173-14|RVcoll.13-S519||Italy|658[0n]
Polyommatus amandus|WMB6586-18|RVcoll14A101||Italy|658[0n]
Polyommatus amandus|WMB918-13|RVcoll.09-X927||Italy|658[0n]
Polyommatus amandus|GWORZ058-10|BC ZSM Lep 30414||Italy|658[0n]
Polyommatus amandus|LEATJ239-15|TLMF Lep 18669||Italy|658[0n]
Polyommatus ripartii|OXB1602-16|OXB-TGS-1320||France|658[0n]
Polyommatus ripartii|EULEP278-14|RVcoll.11-I851|Genit. examined|France|658[0n]
Polyommatus ripartii|BIBSA902-15|LD-3514||Italy|658[0n]
Polyommatus ripartii|EULEP3084-15|RVcoll.14-O217||Italy|658[0n]
Polyommatus ripartii|EULEP3083-15|RVcoll.14-O216||Italy|658[0n]
Polyommatus ripartii|EULEP277-14|RVcoll.11-I869|Genit. examined|France|658[0n]
Polyommatus ripartii|EULEP276-14|RVcoll.11-I703|Genit. examined|France|658[0n]
Polyommatus ripartii|EULEP275-14|RVcoll.10-B712|Genit. examined|France|658[0n]
Polyommatus ripartii|EULEP279-14|RVcoll.12-Q453|Genit. examined|Spain|658[0n]
Polyommatus ripartii|EULEP280-14|RVcoll.12-Q455||Spain|658[0n]
Polyommatus ripartii|EZSPM750-12|Rvcoll. 12-L140|Genit. examined|Spain|651[0n]
Polyommatus ripartii|EULEP274-14|RVcoll.12-L137|Genit. examined|Spain|658[0n]
Polyommatus ripartii|EULEP273-14|RVcoll.12-L128|Genit. examined|Spain|658[0n]
Polyommatus ripartii|EULEP5855-18|RVcoll14V718||Spain|658[0n]
Polyommatus ripartii|EZSPN953-09|RVcoll.08-L945|Genit. examined|Spain|658[0n]
Polyommatus ripartii|GBGL10101-12|HM210172||Italy|1498[0n]
Polyommatus ripartii|GBGL10093-12|HM210164||Italy|1498[0n]
Polyommatus ripartii|GBGL10092-12|HM210163||Italy|1498[0n]
Polyommatus ripartii|EULEP5534-17|RVcoll14B596||Spain|658[0n]
Polyommatus ripartii|WMB4448-14|RVcoll.14-D310||Spain|658[0n]
Polyommatus ripartii|EULEP5536-17|RVcoll14B599||Spain|658[0n]
Polyommatus ripartii|EULEP5871-18|RVcoll15O507||Spain|658[0n]
Polyommatus ripartii|EULEP269-14|RVcoll.08-L390|Genit. examined|France|658[0n]
Polyommatus ripartii|EULEP270-14|RVcoll.12-L130|Genit. examined|Spain|658[0n]
Polyommatus ripartii|EZSPC783-10|RVcoll.08-L948|Genit. examined|Spain|658[0n]
Polyommatus ripartii|EZSPC782-10|RVcoll.08-L946|Genit. examined|Spain|658[0n]
Polyommatus ripartii|EZSPN932-09|RVcoll.08-L892|Genit. examined|Spain|658[0n]
Polyommatus ripartii|EULEP272-14|RVcoll.11-I888|Genit. examined|France|658[0n]
Polyommatus ripartii|EULEP5533-17|RVcoll14B595||Spain|658[0n]
Polyommatus ripartii|WMB4437-14|RVcoll.14-B600||Spain|658[0n]
[illegible]

Polyommatus ripartii|EULEP272-14|RVcoll.11-I888|Genit. examined|France|658[0n]
Polyommatus ripartii|EULEP5533-17|RVcoll14B595||Spain|658[0n]
Polyommatus ripartii|WMB4437-14|RVcoll.14-B600||Spain|658[0n]
Polyommatus ripartii|EULEP271-14|RVcoll.12-L138|Genit. examined|Spain|658[0n]
Polyommatus ripartii|EZSPM748-12|Rvcoll. 12-L133|Genit. examined|Spain|646[0n]
Polyommatus ripartii|EZSPM747-12|Rvcoll. 12-L129|Genit. examined|Spain|658[0n]
Polyommatus ripartii|EULEP3068-15|RVcoll.14-O201||Russia|658[0n]
Polyommatus ripartii|EULEP283-14|RVcoll.11-G191||Croatia|658[0n]
Polyommatus ripartii|EULEP282-14|RVcoll.11-G188|Genit. examined|Croatia|658[0n]
Polyommatus ripartii|EULEP285-14|RVcoll.11-G198||Croatia|658[0n]
Polyommatus ripartii|EULEP284-14|RVcoll.11-G193|Genit. examined|Croatia|658[0n]
Polyommatus ripartii|EULEP281-14|RVcoll.09-V363|Genit. examined|Croatia|658[0n]
Polyommatus|EULEP5783-18|RVcoll14G559||Macedonia|658[0n]
Polyommatus|EULEP5782-18|RVcoll14G553||Macedonia|658[0n]
Polyommatus|EULEP5765-18|RVcoll14F722||Greece|658[0n]
Polyommatus|EULEP5794-18|RVcoll14G557||Macedonia|658[0n]
Polyommatus|EULEP5790-18|RVcoll14N526||Macedonia|658[0n]
Polyommatus|EULEP5779-18|RVcoll14G415||Greece|658[0n]
Polyommatus|EULEP5777-18|RVcoll14G366||Greece|658[0n]
Polyommatus|EULEP5791-18|RVcoll14N527||Macedonia|658[0n]
Polyommatus ripartii|EULEP296-14|RVcoll.11-G192|Genit. examined|Croatia|658[0n]
Polyommatus ripartii|EULEP287-14|RVcoll.11-G196|Genit. examined|Croatia|658[0n]
Polyommatus ripartii|EULEP286-14|RVcoll.11-G190|Genit. examined|Croatia|658[0n]
Polyommatus ripartii|EZSPN204-09|RVcoll.07-F035|Genit. examined|Spain|658[1n]
Polyommatus ripartii|EULEP4538-16|RVcoll15Q172||Ukraine|638[0n]
Polyommatus ripartii|GBGL10096-12|HM210167||Italy|1498[0n]
Polyommatus ripartii|EULEP3086-15|RVcoll.14-O219||Italy|658[0n]
Polyommatus ripartii|EULEP3085-15|RVcoll.14-O218||Italy|658[0n]
Polyommatus ripartii|GBGL10097-12|HM210168||Italy|1498[0n]
Polyommatus ripartii|EULEP295-14|RVcoll.11-G197||Croatia|658[0n]
Polyommatus ripartii|EULEP294-14|RVcoll.11-G194|Genit. examined|Croatia|658[0n]
Polyommatus ripartii|EULEP293-14|RVcoll.11-G189||Croatia|658[0n]
Polyommatus|EULEP5786-18|RVcoll14N522||Bosnia and Herzegovina|658[0n]
Polyommatus|EULEP5793-18|RVcoll14O306||Bosnia and Herzegovina|658[0n]
Polyommatus ripartii|EULEP2093-15|RVcoll.14-J582||Ukraine|658[0n]
Polyommatus ripartii|EULEP4537-16|RVcoll15Q168||Ukraine|636[0n]
Polyommatus ripartii|EZSPC1302-10|RVcoll.300107SM82||Spain|658[0n]
Polyommatus ripartii|EZSPC1289-10|RVcoll.110806MU4||Spain|658[0n]
Polyommatus ripartii|EZSPM125-09|RVcoll.08-P615||Spain|658[0n]
Polyommatus ripartii|EZSPN1014-09|RVcoll.08-M935|Genit. examined|Spain|658[0n]
Polyommatus ripartii|EZSPC815-10|RVcoll.08-P614.1|Genit. examined|Spain|658[0n]
Polyommatus ripartii|EZSPC702-10|RVcoll.07-F038|Genit. examined|Spain|658[0n]
Polyommatus ripartii|EULEP288-14|RVcoll.08-M934|Genit. examined|Spain|658[0n]
Polyommatus ripartii|EULEP289-14|RVcoll.09-V723|Genit. examined|Spain|658[0n]
Polyommatus ripartii|EULEP290-14|RVcoll.09-V724|Genit. examined|Spain|658[0n]
Polyommatus ripartii|EULEP291-14|RVcoll.09-X021|Genit. examined|Spain|658[0n]
Polyommatus ripartii|EULEP292-14|RVcoll.11-G195||Croatia|658[0n]
Polyommatus ripartii|EZSPM073-09|RVcoll.08-P316|Genit. examined|Spain|636[0n]
Polyommatus ripartii|EZSPN1006-09|RVcoll.08-M697|Genit. examined|Spain|658[0n]
Polyommatus ripartii|EZSPN187-09|RVcoll.07-C089|Genit. examined|Spain|658[0n]
Polyommatus ripartii pelopi|GBMIN71205-17|KY066717||Greece|654[0n]
Polyommatus ripartii pelopi|GBMIN71201-17|KY066718||Greece|657[0n]
Polyommatus ripartii pelopi|GBMIN71198-17|KY066713||Greece|657[0n]
Polyommatus ripartii pelopi|GBMIN71197-17|KY066715||Greece|657[0n]
Polyommatus|EULEP5773-18|RVcoll14G287||Greece|658[0n]
Polyommatus ripartii pelopi|GBMIN71199-17|KY066714||Greece|654[0n]
Polyommatus ripartii pelopi|GBMIN71204-17|KY066719||Greece|654[0n]
Polyommatus ripartii pelopi|GBMIN71206-17|KY066720||Greece|657[0n]
Polyommatus ripartii pelopi|GBMIN71203-17|KY066696||Bulgaria|639[0n]
Polyommatus ripartii pelopi|GBMIN71202-17|KY066722||Bulgaria|654[0n]
Polyommatus ripartii pelopi|GBMIN71200-17|KY066716||Greece|654[0n]
Polyommatus ripartii pelopi|GBMIN71195-17|KY066721||Greece|657[0n]
Polyommatus ripartii pelopi|GBMIN71194-17|KY066723||Bulgaria|654[0n]
Polyommatus ripartii pelopi|GBMIN71196-17|KY066712||Greece|654[0n]
Polyommatus ripartii|EULEP304-14|RVcoll.12-M017||Bulgaria|658[0n]
Polyommatus ripartii|EULEP303-14|RVcoll.12-M016||Bulgaria|658[0n]
Polyommatus ripartii|EULEP302-14|RVcoll.12-M014||Bulgaria|658[0n]
Polyommatus ripartii|EULEP301-14|RVcoll.12-M012||Bulgaria|658[0n]
Polyommatus ripartii|EULEP300-14|RVcoll.12-M011|Genit. examined|Bulgaria|658[0n]
Polyommatus ripartii|EULEP299-14|RVcoll.11-J903|Genit. examined|Bulgaria|658[0n]
Polyommatus ripartii|EULEP298-14|RVcoll.10-A998|Genit. examined|Bulgaria|658[0n]
Polyommatus|EULEP5774-18|RVcoll14G305||Greece|658[0n]
Polyommatus|EULEP5771-18|RVcoll14F963||Greece|658[0n]
Polyommatus|EULEP5770-18|RVcoll14F938||Greece|658[0n]
Polyommatus|EULEP5755-18|RVcoll14C344||Greece|658[0n]
Polyommatus|EULEP5758-18|RVcoll14C546||Greece|658[0n]
Polyommatus|EULEP5754-18|RVcoll14C218||Greece|658[0n]
Polyommatus|EULEP5761-18|RVcoll14C946||Bulgaria|658[0n]
Polyommatus|EULEP5760-18|RVcoll14C737||Bulgaria|658[0n]
Polyommatus nephohiptamenos|GBMIN81873-17|KY050605||Greece|657[0n]
Polyommatus nephohiptamenos|GBMIN81872-17|KY066694||Greece|654[0n]
Polyommatus|EULEP5757-18|RVcoll14C467||Greece|658[0n]
Polyommatus|EULEP5749-18|RVcoll13Y092||Greece|658[0n]
Polyommatus|EULEP5753-18|RVcoll14C155||Greece|658[0n]
Polyommatus nephohiptamenos|EULEP297-14|RVcoll.09-V964|Genit. examined|Bulgaria|658[0n]
Polyommatus|EULEP5750-18|RVcoll13Y093||Greece|658[0n]
Polyommatus nephohiptamenos|GBMIN81876-17|KY066695||Greece|654[0n]
Polyommatus nephohiptamenos|GBMIN81875-17|KY050604||Greece|654[0n]
Polyommatus nephohiptamenos|GBMIN81874-17|KY050603||Greece|657[0n]
Polyommatus nephohiptamenos|GBGL20654-18|KY050606||Greece|657[0n]
Polyommatus admetus|GBMIN81770-17|KY050595||Greece|654[0n]
Polyommatus admetus|EULEP5213-17|RVcoll14O340||Greece|658[0n]
Polyommatus admetus|GBMIN81771-17|KY050596||Greece|657[0n]
Polyommatus admetus|EULEP5208-17|RVcoll14G676||Greece|653[0n]
Polyommatus admetus|EULEP5215-17|RVcoll15P063||Croatia|658[0n]
Polyommatus admetus|EULEP5216-17|RVcoll15P064||Croatia|658[0n]
Polyommatus admetus|EULEP3375-16|RVcoll15P066||Croatia|655[0n]
Polyommatus admetus|EULEP305-14|RVcoll.09-V962|Genit. examined|Bulgaria|658[0n]
Polyommatus admetus|EULEP5197-17|RVcoll14C330||Greece|658[0n]
Polyommatus admetus|EULEP5210-17|RVcoll14O336||Macedonia|658[0n]
Polyommatus admetus|EULEP5194-17|RVcoll14B690||Albania|655[0n]
Polyommatus admetus|EULEP5211-17|RVcoll14O337||Macedonia|658[0n]
Polyommatus admetus|GBMIN81768-17|KY050597||Bulgaria|632[0n]

Polyommatus admetus|EULEP5194-17|RVcoll14B690||Albania|655[0n]
Polyommatus admetus|EULEP5211-17|RVcoll14O337||Macedonia|658[0n]
Polyommatus admetus|GBMIN81768-17|KY050597||Bulgaria|632[0n]
Polyommatus admetus|EULEP5199-17|RVcoll14F601||Greece|655[0n]
Polyommatus admetus|EULEP3370-16|RVcoll14F242||Serbia|655[0n]
Polyommatus admetus|EULEP3369-16|RVcoll14C468||Greece|655[0n]
Polyommatus admetus|EULEP3372-16|RVcoll14L002||Romania|655[0n]
Polyommatus admetus|EULEP3368-16|RVcoll14L007||Romania|655[0n]
Polyommatus admetus|EULEP3367-16|RVcoll14L006||Romania|655[0n]
Polyommatus admetus|EULEP3366-16|RVcoll14L001||Romania|655[0n]
Polyommatus admetus|EULEP3365-16|RVcoll14L009||Romania|655[0n]
Polyommatus admetus|EULEP3364-16|RVcoll14G780||Romania|655[0n]
Polyommatus admetus|EULEP5218-17|RVcoll15Q046||Montenegro|658[0n]
Polyommatus admetus|EULEP3376-16|RVcoll15Q045||Montenegro|655[0n]
Polyommatus admetus|EULEP3374-16|RVcoll14F364||Bulgaria|655[0n]
Polyommatus admetus|EULEP5198-17|RVcoll14C663||Greece|658[0n]
Polyommatus admetus|EULEP306-14|RVcoll.09-V963|Genit. examined|Bulgaria|658[0n]
Polyommatus admetus|EULEP5195-17|RVcoll14B722||Albania|655[0n]
Polyommatus admetus|EULEP3371-16|RVcoll14O392||Hungary|655[0n]
Polyommatus admetus|EULEP5217-17|RVcoll15Q038||Macedonia|658[0n]
Polyommatus admetus|EULEP5203-17|RVcoll14G186||Greece|658[0n]
Polyommatus admetus|EULEP5209-17|RVcoll14H493||Greece|658[0n]
Polyommatus admetus|EULEP5212-17|RVcoll14O338||Macedonia|658[0n]
Polyommatus admetus|EULEP5207-17|RVcoll14G471||Greece|655[0n]
Polyommatus admetus|EULEP5206-17|RVcoll14G365||Greece|655[0n]
Polyommatus admetus|EULEP5202-17|RVcoll14G106||Greece|655[0n]
Polyommatus admetus|EULEP5196-17|RVcoll14B725||Albania|658[0n]
Polyommatus admetus|EULEP3373-16|RVcoll14C831||Bulgaria|655[0n]
Polyommatus admetus|EULEP5204-17|RVcoll14G197||Greece|655[0n]
Polyommatus admetus|EULEP5205-17|RVcoll14G212||Greece|655[0n]
Polyommatus admetus|EULEP5201-17|RVcoll14F939||Greece|658[0n]
Polyommatus admetus|EULEP5214-17|RVcoll14O341||Greece|658[0n]
Polyommatus admetus|EULEP5200-17|RVcoll14F936||Greece|655[0n]
Polyommatus admetus|GBGL20652-18|KY050594||Greece|657[0n]
Polyommatus nivescens|EZSPC1332-10|RVcoll.130209KL56||Spain|658[1n]
Polyommatus nivescens|EZSPC868-10|RVcoll.09-T102||Spain|658[0n]
Polyommatus nivescens|EZSPC824-10|RVcoll.08-P750||Spain|658[0n]
Polyommatus nivescens|EZSPC867-10|RVcoll.09-T101||Spain|658[0n]
Polyommatus nivescens|EZSPC887-10|RVcoll.09-T157||Spain|658[0n]
Polyommatus nivescens|EZSPC900-10|RVcoll.09-V489||Spain|658[0n]
Polyommatus nivescens|EZSPM429-09|Rvcoll.09-X523||Spain|614[0n]
Polyommatus nivescens|EZSPM459-09|Rvcoll.09-V578||Spain|658[0n]
Polyommatus nivescens|EZSPN728-09|RVcoll.08-L136||Spain|658[0n]
Polyommatus golgus|EZSPM656-12|RVcoll.11-I481||Spain|618[4n]
Polyommatus golgus|EZSPN666-09|RVcoll.08-J970||Spain|658[0n]
Polyommatus golgus|EZSPN670-09|RVcoll.08-J976||Spain|658[0n]
Polyommatus golgus|EZSPN673-09|RVcoll.08-J979||Spain|658[0n]
Polyommatus golgus|EZSPN671-09|RVcoll.08-J977||Spain|658[0n]
Polyommatus golgus|EZSPM655-12|RVcoll.11-I480||Spain|658[0n]
Polyommatus golgus|EZSPM657-12|RVcoll.11-I482||Spain|658[0n]
Polyommatus golgus|EZSPC894-10|RVcoll.09-V421||Spain|658[0n]
Polyommatus golgus|EZSPC902-10|RVcoll.09-V523||Spain|658[0n]
Polyommatus golgus|EZSPN174-09|RVcoll.06-K710||Spain|614[0n]
Polyommatus golgus|EZSPC899-10|RVcoll.09-V487||Spain|658[0n]
Polyommatus golgus|EZSPC898-10|RVcoll.09-V482||Spain|658[0n]
Polyommatus golgus|EZSPC897-10|RVcoll.09-V480||Spain|658[0n]
Polyommatus golgus|EZSPC896-10|RVcoll.09-V476||Spain|658[0n]
Polyommatus golgus|EZSPN168-09|RVcoll.06-K694||Spain|658[0n]
Polyommatus dorylas|EZSPM267-09|RVcoll.08-R483||Spain|658[0n]
Polyommatus dorylas|EZSPC1390-10|RVcoll.09-X579||Spain|658[0n]
Polyommatus dorylas|EZSPC1387-10|RVcoll.09-X550||Spain|658[0n]
Polyommatus dorylas|EZSPC1383-10|RVcoll.09-X522||Spain|658[0n]
Polyommatus dorylas|EZSPM159-09|RVcoll.08-P685||Spain|658[0n]
Polyommatus dorylas|EZSPC1065-10|RVcoll.08-L168||Spain|658[0n]
Polyommatus dorylas|EZSPC1235-10|RVcoll.09-V715||Spain|658[0n]
Polyommatus dorylas|EZSPM117-09|RVcoll.08-P600||Spain|658[0n]
Polyommatus dorylas|EZSPN878-09|RVcoll.08-L779||Spain|658[0n]
Polyommatus dorylas|EZSPC682-09|RVcoll.08-R162||Spain|658[0n]
Polyommatus dorylas|EZSPC681-09|RVcoll.08-R106||Spain|658[0n]
Polyommatus dorylas|EZSPC650-09|RVcoll.07-C627||Spain|658[0n]
Polyommatus dorylas|EZSPC1231-10|RVcoll.09-V696||Spain|658[0n]
Polyommatus dorylas|EZSPC659-09|RVcoll.07-F024||Spain|658[0n]
Polyommatus dorylas|GWORO803-09|BC ZSM Lep 30495||Germany|658[0n]
Polyommatus dorylas|EULEP652-15|RVcoll.14-G385||Greece|658[1n]
Polyommatus dorylas|LEASS734-17|TLMF Lep 22382||Austria|658[0n]
Polyommatus dorylas|EULEP4512-16|RVcoll15H158||Italy|658[0n]
Polyommatus dorylas|EZROM697-08|RV-07-F561||Romania|652[0n]
Polyommatus dorylas|EULEP4511-16|RVcoll15G540||Switzerland|658[1n]
Polyommatus dorylas|EZROM498-08|RV-06-M979||Romania|658[0n]
Polyommatus dorylas|EZRMN202-08|RVcoll.08-M641||Romania|658[0n]
Polyommatus dorylas|EULEP1882-15|RVcoll.14-A616||Italy|658[0n]
Polyommatus dorylas|EULEP2133-15|RVcoll.14-J975||Switzerland|658[0n]
Polyommatus dorylas|EZRMN369-08|RVcoll.08-M736||Romania|658[0n]
Polyommatus dorylas|ABOLD626-17|TLMF Lep 21702||Austria|658[0n]
Polyommatus dorylas|LEASS700-17|TLMF Lep 22348||Austria|658[0n]
Polyommatus dorylas|LEASS534-17|TLMF Lep 22182||Austria|658[0n]
Polyommatus dorylas|EULEP945-15|RVcoll.14-C942||Bulgaria|658[0n]
Polyommatus dorylas|EULEP1342-15|RVcoll.14-F847||Greece|658[0n]
Polyommatus dorylas|EULEP1273-15|RVcoll.14-F672||Greece|658[0n]
Polyommatus dorylas|EULEP5043-16|RVcoll16J039||Slovakia|658[0n]
Polyommatus dorylas|EULEP4509-16|RVcoll14V176||Italy|658[0n]
Polyommatus dorylas|EZRMN201-08|RVcoll.08-M511|Genit. examined|Romania|658[0n]
Polyommatus dorylas|EZRMN200-08|RVcoll.08-M510||Romania|658[0n]
Polyommatus dorylas|EZROM499-08|RV-06-N001|Genit. examined|Romania|658[0n]
Polyommatus dorylas|LEATG489-14|TLMF Lep 14276||Italy|658[0n]
Polyommatus dorylas|LEATG469-14|TLMF Lep 14256||Italy|658[0n]
Polyommatus dorylas|EULEP1535-15|RVcoll.14-G492||Greece|658[0n]
Polyommatus dorylas|EULEP1443-15|RVcoll.14-G143||Greece|658[0n]
Polyommatus dorylas|ABOLD086-16|TLMF Lep 21164||Austria|658[0n]
Polyommatus dorylas|LEATG569-14|TLMF Lep 14356||Austria|658[4n]
Polyommatus dorylas|GWORA2454-09|BC ZSM Lep 30666||Germany|658[0n]
Polyommatus dorylas|LEATG484-14|TLMF Lep 14271||Italy|630[4n]
[illegible]

Polyommatus dorylas|LEATG569-14|TLMF Lep 14[illegible]||Austria|658[4n]
Polyommatus dorylas|GWORA2454-09|BC ZSM Lep 30666||Germany|658[0n]
Polyommatus dorylas|LEATG484-14|TLMF Lep 14271||Italy|630[4n]
Polyommatus damon|LEATG419-14|TLMF Lep 14206||Italy|658[0n]
Polyommatus damon|LEATG420-14|TLMF Lep 14207||Italy|658[0n]
Polyommatus damon|PHLAI494-13|TLMF Lep 09056||Austria|601[0n]
Polyommatus damon|GWOSK862-11|BC ZSM Lep 49363||Germany|658[0n]
Polyommatus damon|LEATJ1155-16|TLMF Lep 19478||Czech Republic|658[0n]
Polyommatus damon|EZSPN935-09|RVcoll.08-L895||Spain|658[0n]
Polyommatus damon|EZSPN934-09|RVcoll.08-L894||Spain|658[0n]
Polyommatus damon|PHLAF637-11|TLMF Lep 05807||Switzerland|658[0n]
Polyommatus damon|EULEP329-14|MM23814||Latvia|614[0n]
Polyommatus damon|ABOLD618-17|TLMF Lep 21694||Austria|658[0n]
Polyommatus damon|LEATG223-14|TLMF Lep 14010||Austria|658[0n]
Polyommatus damon|EULEP630-15|RVcoll.11-J018||Switzerland|658[0n]
Polyommatus damon|EULEP1873-15|RVcoll.14-A230||Italy|658[0n]
Polyommatus damon|WMB5261-14|RVcoll.14-A301||Italy|658[0n]
Polyommatus damon|WMB4752-14|RVcoll.07-E176.1||Italy|658[0n]
Polyommatus damon|BIBSA425-15|RVcoll.14-I093||Italy|610[0n]
Polyommatus damon|WMB2386-13|RVcoll.07-E242||Italy|658[0n]
Polyommatus damon|BIBSA263-15|RVcoll.14-E061||Italy|658[0n]
Polyommatus damon|EULEP4505-16|RVcoll15J417||France|658[0n]
Polyommatus damon|WMB1865-13|RVcoll.11-J196||France|658[0n]
Polyommatus damon|WMB2707-13|RVcoll.10-C130||France|658[0n]
Polyommatus damon|BIBSA312-15|RVcoll.14-E114||Italy|658[0n]
Polyommatus damon|WMB1642-13|RVcoll.12-O905||Italy|658[0n]
Polyommatus damon|WMB1637-13|RVcoll.12-O861||France|658[0n]
Polyommatus damon|PHLAA430-09|TLMF Lep 00470||France|658[0n]
Polyommatus damon|EZSPM015-09|RVcoll.08-P001||Spain|658[0n]
Polyommatus damon|EZSPC1116-10|RVcoll.08-P326||Spain|658[0n]
Polyommatus damon|EZSPM200-09|RVcoll.08-R289||Spain|658[0n]
Polyommatus damon|EZSPN203-09|RVcoll.07-F023||Spain|622[2n]
Polyommatus damon|EZSPC1125-10|RVcoll.08-P629||Spain|658[0n]
Polyommatus damon|EZSPC1379-10|RVcoll.09-X513||Spain|658[0n]
Polyommatus damon|EZSPC1104-10|RVcoll.08-L994||Spain|658[0n]
Polyommatus damon|EZSPN284-09|RVcoll.07-W228||Spain|623[0n]
Polyommatus damon|EZSPN754-09|RVcoll.08-L230||Spain|658[0n]
Polyommatus damon|EZSPN743-09|RVcoll.08-L167||Spain|658[0n]
Polyommatus damon|EULEP5792-18|RVcoll14O305||Macedonia|658[0n]
Polyommatus damon|EULEP5125-17|RVcoll16J880||Macedonia|658[0n]
Polyommatus damon|PHLAF389-11|TLMF Lep 05559||Macedonia|658[0n]
Polyommatus damocles|EULEP2492-15|RVcoll.14-V349||Ukraine|614[0n]
Polyommatus damocles|EULEP2491-15|RVcoll.14-V348||Ukraine|658[0n]
Polyommatus damocles|EULEP2490-15|RVcoll.14-V347||Ukraine|658[0n]
Polyommatus damocles|EULEP2489-15|RVcoll.14-V346||Ukraine|658[0n]
Polyommatus damocles|EULEP2098-15|RVcoll.14-J587||Ukraine|658[0n]
Polyommatus damocles|EULEP2097-15|RVcoll.14-J586||Ukraine|658[0n]
Polyommatus damocles|EULEP2096-15|RVcoll.14-J585||Ukraine|658[0n]
Polyommatus damocles|EULEP2095-15|RVcoll.14-J584||Ukraine|658[0n]
Polyommatus damone|EULEP437-14|KN00799||Russia|658[0n]
Polyommatus damone|EULEP3076-15|RVcoll.14-O209||Russia|658[0n]
Polyommatus damone|EULEP2285-15|RVcoll.14-N471||Russia|658[0n]
Polyommatus damone|EULEP436-14|KN00798||Russia|658[0n]
Polyommatus damone|EULEP2563-15|RVcoll.14-V538||Ukraine|611[0n]
Polyommatus damone|EULEP2091-15|RVcoll.14-J579||Ukraine|658[0n]
Polyommatus damone|EULEP2063-15|RVcoll.14-J548||Ukraine|658[0n]
Polyommatus damone|EULEP435-14|KN00797||Ukraine|658[0n]
Polyommatus damone|EULEP4842-16|RVcoll16H749||Ukraine|658[0n]
Polyommatus damone|EULEP2564-15|RVcoll.14-V539||Ukraine|658[0n]
Polyommatus damone|EULEP4542-16|RVcoll14V614||Russia|642[0n]
Polyommatus damone|EULEP2501-15|RVcoll.14-V363||Ukraine|611[0n]
Polyommatus damone|EULEP2506-15|RVcoll.14-V372||Ukraine|611[0n]
Polyommatus damone|EULEP2507-15|RVcoll.14-V373||Ukraine|658[0n]
Polyommatus damone|EULEP2092-15|RVcoll.14-J581||Ukraine|658[0n]
Polyommatus damone|EULEP434-14|KN00796||Ukraine|658[0n]
Polyommatus damone|WMB6590-18|RVcoll14J580||Ukraine|658[0n]
Polyommatus damone|EULEP2043-15|RVcoll.14-J523||Ukraine|658[0n]
Polyommatus damone|EULEP4826-16|RVcoll16H723||Ukraine|658[0n]
Polyommatus damone|WMB6589-18|RVcoll14J522||Ukraine|658[0n]
Polyommatus iphigenia|EULEP1348-15|RVcoll.14-F894||Greece|658[0n]
Polyommatus iphigenia|EULEP1490-15|RVcoll.14-G322||Greece|658[0n]
Polyommatus iphigenia|EULEP1350-15|RVcoll.14-F898||Greece|658[0n]
Polyommatus iphigenia|EULEP5768-18|RVcoll14F873||Greece|658[0n]
Polyommatus iphigenia|EULEP1492-15|RVcoll.14-G326||Greece|658[0n]
Polyommatus iphigenia|EULEP5766-18|RVcoll14F867||Greece|658[0n]
Polyommatus violetae|EZSPC923-10|RVcoll.09-V910||Spain|658[0n]
Polyommatus violetae|EZSPC922-10|RVcoll.09-V909||Spain|658[0n]
Polyommatus violetae|EZSPC921-10|RVcoll.09-V906||Spain|658[0n]
Polyommatus violetae|EZSPN200-09|RVcoll.07-C687||Spain|658[0n]
Polyommatus violetae|EULEP1973-15|RVcoll.14-H858||Spain|658[0n]
Polyommatus violetae|EULEP1972-15|RVcoll.14-H857||Spain|658[0n]
Polyommatus violetae|WMB4522-14|RVcoll.14-E222||Spain|658[0n]
Polyommatus violetae|EZSPN199-09|RVcoll.07-C686||Spain|658[0n]
Polyommatus|EULEP5769-18|RVcoll14F937||Greece|658[0n]
Polyommatus|EULEP5748-18|RVcoll13Y090||Greece|658[0n]
Polyommatus|EULEP5785-18|RVcoll14N521||Macedonia|658[0n]
Polyommatus|EULEP5751-18|RVcoll14B767||Albania|658[0n]
Polyommatus|EULEP5745-18|RVcoll12M010|Genit. examined|Bulgaria|658[0n]
Polyommatus|EULEP5780-18|RVcoll14G496||Greece|658[0n]
Polyommatus|EULEP5743-18|RVcoll10A996|Genit. examined|Bulgaria|658[0n]
Polyommatus humedasae|OXB1023-15|15-A973||Italy|658[0n]
Polyommatus humedasae|EULEP4527-16|RVcoll15H044||Italy|658[0n]
Polyommatus humedasae|EULEP4526-16|RVcoll15H043||Italy|658[0n]
Polyommatus humedasae|GBGL10098-12|HM210169||Italy|1498[0n]
Polyommatus humedasae|EULEP569-15|RVcoll.09-X294||Italy|658[0n]
Polyommatus humedasae|EULEP4528-16|RVcoll15H058||Italy|658[0n]
Polyommatus humedasae|EULEP4525-16|RVcoll15H031||Italy|658[0n]
Polyommatus|EULEP5788-18|RVcoll14N524||Bosnia and Herzegovina|658[0n]
Polyommatus|EULEP5787-18|RVcoll14N523||Bosnia and Herzegovina|658[0n]
Polyommatus|EULEP5775-18|RVcoll14G312||Greece|658[0n]
Polyommatus|EULEP5776-18|RVcoll14G348||Greece|658[0n]
Polyommatus aroaniensis|GBMIN81773-17|KY050598||Greece|657[0n]
Polyommatus|EULEP5772-18|RVcoll14G282||Greece|658[0n]

Polyommatus|EULEP5776-18|RVcoll14G348||Greece|658[0n]
Polyommatus aroaniensis|GBMIN81773-17|KY050598||Greece|657[0n]
Polyommatus|EULEP5772-18|RVcoll14G282||Greece|658[0n]
Polyommatus|EULEP5767-18|RVcoll14F869||Greece|658[0n]
Polyommatus timfristos|GBMIN81947-17|KY066724||Greece|657[0n]
Polyommatus timfristos|GBMIN81948-17|KY066725||Greece|657[0n]
Polyommatus timfristos|GBMIN81950-17|KY066729||Greece|657[0n]
Polyommatus timfristos|GBMIN81949-17|KY066728||Greece|654[0n]
Polyommatus timfristos|GBMIN81951-17|KY066726||Greece|654[0n]
Polyommatus timfristos|GBGL20651-18|KY066727||Greece|654[0n]
Polyommatus|EULEP5746-18|RVcoll12M013|Genit. examined|Bulgaria|658[0n]
Polyommatus orphicus orphicus|GBMIN71193-17|KY066698||Bulgaria|657[0n]
Polyommatus orphicus orphicus|GBMIN71185-17|KY066707||Bulgaria|639[0n]
Polyommatus orphicus orphicus|GBGL20650-18|KY066704||Bulgaria|634[0n]
Polyommatus|EULEP5752-18|RVcoll14B793||Albania|658[0n]
Polyommatus orphicus orphicus|GBMIN71191-17|KY066697||Bulgaria|657[0n]
Polyommatus orphicus orphicus|GBMIN71188-17|KY066700||Bulgaria|639[1n]
Polyommatus orphicus orphicus|GBMIN71189-17|KY066699||Bulgaria|654[0n]
Polyommatus orphicus orphicus|GBMIN71183-17|KY066703||Bulgaria|633[0n]
Polyommatus orphicus orphicus|GBMIN71182-17|KY066701||Bulgaria|639[1n]
Polyommatus orphicus orphicus|GBMIN71190-17|KY066702||Bulgaria|636[0n]
Polyommatus orphicus orphicus|GBMIN71192-17|KY066710||Bulgaria|639[0n]
Polyommatus orphicus orphicus|GBMIN71187-17|KY066708||Bulgaria|639[0n]
Polyommatus orphicus orphicus|GBMIN71186-17|KY066706||Bulgaria|639[0n]
Polyommatus orphicus orphicus|GBMIN71181-17|KY066709||Bulgaria|639[0n]
Polyommatus orphicus orphicus|GBMIN71184-17|KY066711||Bulgaria|639[0n]
Polyommatus orphicus orphicus|GBMIN71180-17|KY066705||Bulgaria|639[0n]
Polyommatus|EULEP5762-18|RVcoll14D016||Bulgaria|658[0n]
Polyommatus|EULEP5747-18|RVcoll12M015|Genit. examined|Bulgaria|658[0n]
Polyommatus|EULEP5778-18|RVcoll14G375||Greece|658[0n]
Polyommatus|EULEP5744-18|RVcoll10A999|Genit. examined|Bulgaria|658[0n]
Polyommatus orphicus eleniae|GBMIN71179-17|KY050601||Greece|654[0n]
Polyommatus|EULEP5789-18|RVcoll14N525||Macedonia|658[0n]
Polyommatus|EULEP5784-18|RVcoll14N520||Macedonia|658[0n]
Polyommatus|EULEP5781-18|RVcoll14G500||Greece|658[0n]
Polyommatus|EULEP5756-18|RVcoll14C380||Greece|658[0n]
Polyommatus|EULEP5759-18|RVcoll14C649||Greece|658[0n]
Polyommatus|EULEP5763-18|RVcoll14D051||Bulgaria|658[0n]
Polyommatus orphicus eleniae|GBMIN71178-17|KY050599||Greece|657[0n]
Polyommatus orphicus eleniae|GBGL20649-18|KY050602||Greece|654[0n]
Polyommatus orphicus eleniae|GBGL20648-18|KY050600||Greece|654[0n]
Polyommatus fabressei|EZSPC1328-10|RVcoll.130209KL89||Spain|658[0n]
Polyommatus fulgens|EZSPM425-09|Rvcoll.09-X583||Spain|658[0n]
Polyommatus fulgens|EZSPM426-09|Rvcoll.09-X565||Spain|658[0n]
Polyommatus fulgens|EZSPM077-09|RVcoll.08-P327||Spain|658[0n]
Polyommatus fulgens|WMB3298-14|RVcoll.08-P330||Spain|658[0n]
Polyommatus fulgens|EZSPM001-09|RVcoll.08-M964||Spain|658[0n]
Polyommatus fulgens|EZSPN205-09|RVcoll.07-F036||Spain|658[0n]
Polyommatus fabressei|EZSPN919-09|RVcoll.08-L864||Spain|658[0n]
Polyommatus fabressei|WMB3703-14|RVcoll.12-L134||Spain|658[0n]
Polyommatus fabressei|EZSPN920-09|RVcoll.08-L865||Spain|658[0n]
Polyommatus fulgens|EZSPN954-09|RVcoll.08-L947||Spain|658[0n]
Polyommatus fulgens|WMB4449-14|RVcoll.14-D312||Spain|658[0n]
Polyommatus fulgens|EZSPN1114-11|Rvcoll.10-C313||Spain|629[0n]
Polyommatus fabressei|EZSPM749-12|Rvcoll. 12-L135||Spain|621[0n]
Polyommatus fabressei|EZSPM746-12|Rvcoll. 12-L126||Spain|621[0n]
Polyommatus fabressei|EZSPM828-12|Rvcoll. 140611ZG95||Spain|658[0n]
Polyommatus fabressei|EZSPM353-09|RVcoll.08-R080||Spain|658[0n]
Polyommatus fabressei|EZSPM338-09|RVcoll.08-R086||Spain|658[0n]
Polyommatus fabressei|EZSPM334-09|RVcoll.08-R073||Spain|658[0n]
Polyommatus fabressei|EZSPM331-09|RVcoll.08-R069||Spain|658[0n]
Polyommatus fabressei|EZSPM336-09|RVcoll.08-R084||Spain|658[0n]
Polyommatus fabressei|EZSPN286-09|RVcoll.07-W234||Spain|658[0n]
Polyommatus fabressei|EZSPM333-09|RVcoll.08-R071||Spain|658[0n]
Polyommatus fabressei|EZSPM335-09|RVcoll.08-R083||Spain|658[0n]
Polyommatus fabressei|EZSPM827-12|Rvcoll. 140611ZG90||Spain|658[0n]
Polyommatus fabressei|EZSPC755-10|RVcoll.08-L241||Spain|658[0n]
Polyommatus fabressei|EZSPC814-10|RVcoll.08-P613||Spain|658[0n]
Polyommatus fabressei|EZSPC952-10|RVcoll.09-X504||Spain|658[0n]
Polyommatus fabressei|EZSPM866-12|Rvcoll.080611XP76||Spain|658[0n]
Polyommatus fabressei|EZSPM879-12|Rvcoll.150310ZX38||Spain|658[0n]
Polyommatus fabressei|EZSPC1280-10|RVcoll.121109PY21||Spain|658[0n]
Polyommatus fabressei|EZSPC1295-10|RVcoll.290808XZ3||Spain|658[0n]
Polyommatus fabressei|EZSPC1304-10|RVcoll.121109PY22||Spain|658[0n]
Polyommatus fabressei|EZSPC1327-10|RVcoll.121109PY07||Spain|658[0n]
Polyommatus fabressei|WMB4571-14|RVcoll.140410KV18||Spain|658[0n]
Polyommatus fabressei|EZSPM823-12|Rvcoll. 140611ZG01||Spain|639[0n]
Polyommatus fabressei|EZSPN220-09|RVcoll.07-F212||Spain|617[0n]
Polyommatus fabressei|EZSPM337-09|RVcoll.08-R085||Spain|658[0n]
Polyommatus fabressei|EZSPM352-09|RVcoll.08-R090||Spain|658[0n]
Polyommatus fabressei|EZSPC754-10|RVcoll.08-L212||Spain|658[0n]
Polyommatus fabressei|EZSPM332-09|RVcoll.08-R070||Spain|658[0n]
Polyommatus fabressei|EZSPN218-09|RVcoll.07-F079||Spain|658[0n]
Polyommatus fulgens|EZSPN188-09|RVcoll.07-C091||Spain|658[0n]
Polyommatus fulgens|EZSPC780-10|RVcoll.08-L898||Spain|658[0n]
Polyommatus fulgens|EZSPN952-09|RVcoll.08-L944||Spain|658[0n]
Polyommatus fulgens|EZSPN937-09|RVcoll.08-L899||Spain|658[0n]
Polyommatus dolus|WMB1716-13|RVcoll.12-P690||France|658[0n]
Polyommatus dolus|BIBSA894-15|LD-3506||Italy|658[0n]
Polyommatus dolus|BIBSA893-15|LD-3505||Italy|658[0n]
Polyommatus dolus|EULEP751-15|RVcoll.12-P936||France|658[0n]
Polyommatus dolus|WMB1724-13|RVcoll.12-P827||France|658[0n]
Polyommatus dolus|WMB104-11|RVcoll.10-B621||France|658[0n]
Polyommatus dolus|BCLEP116-17|LEP-SS-00567||Italy|658[0n]
Polyommatus dolus|WMB915-13|RVcoll.09-X912||Italy|658[0n]
Polyommatus dolus|WMB2376-13|RVcoll.06-V790.1||Italy|658[0n]
Polyommatus dolus|EULEP1842-15|LD-2653||Italy|658[0n]
Polyommatus dolus|WMB779-13|RVcoll.06-V796||Italy|658[0n]
Polyommatus dolus|WMB778-13|RVcoll.06-V791||Italy|658[0n]
Polyommatus dolus|WMB2382-13|RVcoll.07-E105||Italy|658[0n]
Polyommatus dolus|EULEP513-15|RVcoll.07-E101||Italy|658[0n]
Polyommatus dolus|WMB807-13|RVcoll.07-E086||Italy|636[0n]
Polyommatus dolus|PHLSA459-11|TLMF Lep 05914||Italy|658[0n]

Polyommatus dolus|WMB[illegible]|RVcoll.07-E1[illegible]||Italy|658[0n]
Polyommatus dolus|EULEP513-15|RVcoll.07-E101||Italy|658[0n]
Polyommatus dolus|WMB807-13|RVcoll.07-E086||Italy|636[0n]
Polyommatus dolus|PHLSA459-11|TLMF Lep 05914||Italy|658[0n]
Polyommatus daphnis|EZSPC1126-10|RVcoll.08-P636||Spain|658[0n]
Polyommatus daphnis|EZSPM130-09|RVcoll.08-P635||Spain|658[0n]
Polyommatus daphnis|EZSPN779-09|RVcoll.08-L291||Spain|658[0n]
Polyommatus daphnis|EZSPC535-09|RVcoll.07-F533||Spain|634[0n]
Polyommatus daphnis|EZSPC538-09|RVcoll.08-P064||Spain|632[0n]
Polyommatus daphnis|EZSPC536-09|RVcoll.07-F005||Spain|658[0n]
Polyommatus daphnis|EZSPC537-09|RVcoll.08-M695||Spain|613[0n]
Polyommatus daphnis|EZSPC638-09|RVcoll.08-L376||Spain|658[0n]
Polyommatus daphnis|EULEP4508-16|RVcoll15Q071||Russia|658[0n]
Polyommatus daphnis|LOWA524-06|2005-LOWA-524||Ukraine|658[0n]
Polyommatus daphnis|EULEP149-14|RVcoll.10-C471||Romania|658[0n]
Polyommatus daphnis|EZROM401-08|RV-07-E425|Genit. examined|Romania|658[0n]
Polyommatus daphnis|FBLMV713-09|BC ZSM Lep 28693||Germany|658[0n]
Polyommatus daphnis|GWOSK859-11|BC ZSM Lep 49360||Germany|658[0n]
Polyommatus daphnis|GWORO762-09|BC ZSM Lep 30454||Germany|658[0n]
Polyommatus daphnis|EULEP4850-16|RVcoll16H776||Bulgaria|658[0n]
Polyommatus daphnis|EULEP174-14|RVcoll.11-J335||Romania|658[0n]
Polyommatus daphnis|EZRMN097-08|RVcoll.06-V686||Romania|658[0n]
Polyommatus daphnis|EULEP867-15|RVcoll.14-C322||Greece|658[0n]
Polyommatus daphnis|EZROM399-08|RV-06-M883||Romania|658[0n]
Polyommatus daphnis|EZROM638-08|RV-06-N022|Genit. examined|Romania|658[0n]
Polyommatus daphnis|EULEP1624-15|RVcoll.14-G713||Greece|614[0n]
Polyommatus daphnis|EULEP1126-15|RVcoll.14-F240||Serbia|658[0n]
Polyommatus daphnis|EULEP2224-15|RVcoll.14-N402||Ukraine|614[0n]
Polyommatus daphnis|LOWA523-06|2005-LOWA-523||Ukraine|658[0n]
Polyommatus daphnis|EULEP1200-15|RVcoll.14-F492||Bulgaria|620[0n]
Polyommatus daphnis|GBMIN20564-13|JX093461||Ukraine|1495[0n]
Polyommatus daphnis|EZRMN098-08|RVcoll.07-E413||Romania|658[0n]
Polyommatus daphnis|EULEP4506-16|RVcoll10B323||Bulgaria|658[0n]
Polyommatus daphnis|EULEP4916-16|RVcoll16J619||Slovakia|658[0n]
Polyommatus daphnis|LOWA522-06|2005-LOWA-522||Ukraine|658[0n]
Polyommatus daphnis|EULEP1241-15|RVcoll.14-F584||Greece|658[0n]
Polyommatus daphnis|LEASS530-17|TLMF Lep 22178||Austria|658[0n]
Polyommatus daphnis|EULEP2405-15|RVcoll.14-V116||Ukraine|651[0n]
Polyommatus daphnis|EZROM400-08|RV-06-M884||Romania|632[0n]
Polyommatus daphnis|ABOLD623-17|TLMF Lep 21699||Austria|658[0n]
Polyommatus daphnis|LEATJ1196-16|TLMF Lep 19519||Austria|658[0n]
Polyommatus daphnis|EULEP1463-15|RVcoll.14-G222||Greece|658[0n]
Polyommatus daphnis|EULEP1390-15|RVcoll.14-F997||Greece|658[0n]
Polyommatus daphnis|BIBSA1577-16|11-J689||Italy|618[0n]
Polyommatus daphnis|WMB705-12|RVcoll.11-H303|Sicily|Italy|658[0n]
Polyommatus daphnis|WMB704-12|RVcoll.11-H302|Sicily|Italy|658[0n]
Polyommatus daphnis|WMB703-12|RVcoll.11-H301|Sicily|Italy|658[0n]
Polyommatus daphnis|WMB702-12|RVcoll.11-H300|Sicily|Italy|658[0n]
Polyommatus daphnis|OXB373-15|RVcoll.14-N087||Italy|658[0n]
Polyommatus daphnis|WMB6574-18|RVcoll14A100||Italy|658[0n]
Polyommatus daphnis|WMB678-12|RVcoll.11-E212||France|658[0n]
Polyommatus daphnis|WMB677-12|RVcoll.11-E209||France|658[0n]
Polyommatus daphnis|BIBSA1746-16|12-Q809||Italy|658[0n]
Polyommatus daphnis|BIBSA1160-15|15-L127||Italy|658[0n]
Polyommatus daphnis|WMB798-13|RVcoll.07-E001||Italy|658[0n]
Polyommatus daphnis|WMB2122-13|RVcoll.12-R199||Italy|658[0n]
Polyommatus daphnis|WMB1714-13|RVcoll.12-P677||France|658[0n]
Polyommatus daphnis|WMB5490-14|RVcoll.LD-2652||Italy|658[0n]
Polyommatus daphnis|WMB4954-14|RVcoll. 14-I501||Italy|658[0n]
Polyommatus daphnis|WMB676-12|RVcoll.11-E208||France|658[0n]
Polyommatus daphnis|BIBSA1591-16|12-Q805||Italy|658[0n]
Polyommatus daphnis|WMB2053-13|RVcoll.12-Q807||Italy|658[0n]
Polyommatus daphnis|WMB4953-14|RVcoll. 14-I500||Italy|658[0n]
Polyommatus daphnis|EULEP4507-16|RVcoll14V175||Italy|658[0n]
Polyommatus daphnis|WMB4678-14|RVcoll.LD-2651||Italy|658[0n]
Polyommatus daphnis|WMB795-13|RVcoll.07-D874||Italy|647[0n]
Polyommatus daphnis|WMB2116-13|RVcoll.12-R188||Italy|658[0n]
Polyommatus daphnis|LEATG426-14|TLMF Lep 14213||Italy|639[0n]
Polyommatus daphnis|LEATG425-14|TLMF Lep 14212||Italy|658[1n]
Polyommatus thersites|WMB2755-13|RVcoll.10-C669||Italy|658[0n]
Polyommatus thersites|EULEP2406-15|RVcoll.14-V117||Ukraine|658[0n]
Polyommatus thersites|EULEP1360-15|RVcoll.14-F926||Greece|658[0n]
Polyommatus thersites|EULEP180-14|RVcoll.11-J407||Romania|658[0n]
Polyommatus thersites|EZRMN214-08|RVcoll.08-M657||Romania|658[0n]
Polyommatus thersites|EZROM508-08|RV-07-D204|Genit. examined|Romania|658[0n]
Polyommatus thersites|EULEP2094-15|RVcoll.14-J583||Ukraine|658[0n]
Polyommatus thersites|EULEP1560-15|RVcoll.14-G556||Macedonia|614[0n]
Polyommatus thersites|EZROM509-08|RV-07-D224||Romania|658[0n]
Polyommatus thersites|EZRMN212-08|RVcoll.08-M370||Romania|658[0n]
Polyommatus thersites|EZROM506-08|RV-06-V687|Genit. examined|Romania|658[0n]
Polyommatus thersites|EULEP1191-15|RVcoll.14-F464||Bulgaria|658[0n]
Polyommatus thersites|EULEP1240-15|RVcoll.14-F583||Greece|658[0n]
Polyommatus thersites|EULEP1667-15|RVcoll.14-G894||Greece|622[0n]
Polyommatus thersites|EULEP872-15|RVcoll.14-C367||Greece|658[0n]
Polyommatus thersites|EULEP856-15|RVcoll.14-G021||Greece|658[0n]
Polyommatus thersites|EULEP1113-15|RVcoll.14-F188||Serbia|658[0n]
Polyommatus thersites|EZROM507-08|RV-07-D143||Romania|658[0n]
Polyommatus thersites|EZRMN211-08|RVcoll.07-D142||Romania|658[0n]
Polyommatus thersites|WMB228-11|RVcoll.09-T548||Italy|658[0n]
Polyommatus thersites|WMB227-11|RVcoll.09-T547||Italy|658[0n]
Polyommatus thersites|GBLAB122-13|BC ZSM Lep 75748|Identified by barcode|Germany|658[0n]
Polyommatus thersites|BIBSA655-15|RVcoll. 15-A818||Italy|658[0n]
Polyommatus thersites|WMB2072-13|RVcoll.12-Q931||Italy|658[0n]
Polyommatus thersites|WMB5191-14|RVcoll.13-T783||Italy|658[0n]
Polyommatus thersites|OXB878-15|15-A520||Italy|658[0n]
Polyommatus thersites|WMB105-11|RVcoll.10-B622||France|658[0n]
Polyommatus thersites|ABOLD625-17|TLMF Lep 21701||Austria|658[0n]
Polyommatus thersites|FBLMW304-10|BC ZSM Lep 37405||Germany|658[0n]
Polyommatus thersites|WMB3739-14|RVcoll.12-P863||France|658[0n]
Polyommatus thersites|WMB835-13|RVcoll.07-E224||Italy|658[0n]
Polyommatus thersites|WMB930-13|RVcoll.10-A514||France|658[0n]
Polyommatus thersites|WMB1776-13|RVcoll.12-Q095||France|658[0n]
Polyommatus thersites|WMB1711-13|RVcoll.12-P659||France|658[0n]

Polyommatus thersites|WMB930-13|RVcoll.10-A514||France|658[0n]
Polyommatus thersites|WMB1776-13|RVcoll.12-Q095||France|658[0n]
Polyommatus thersites|WMB1711-13|RVcoll.12-P659||France|658[0n]
Polyommatus thersites|BIBSA1128-15|15-L932||Italy|658[0n]
Polyommatus thersites|BIBSA648-15|RVcoll. 15-A811||Italy|658[0n]
Polyommatus thersites|LEASS574-17|TLMF Lep 22222||Austria|658[0n]
Polyommatus thersites|GBMIN16200-13|JX678093||France|657[0n]
Polyommatus thersites|OXB946-15|15-A590||Italy|658[0n]
Polyommatus thersites|WMB3660-14|RVcoll.11-I778||France|601[0n]
Polyommatus thersites|EZROM505-08|RV-06-M854||Romania|658[0n]
Polyommatus thersites|LEATI061-15|TLMF Lep 17446||Austria|658[0n]
Polyommatus thersites|LEATI060-15|TLMF Lep 17445||Austria|658[0n]
Polyommatus thersites|WMB3205-14|RVcoll.08-J212||Spain|658[0n]
Polyommatus thersites|EULEP244-14|RVcoll.12-N705||Spain|658[0n]
Polyommatus thersites|WMB6570-18|RVcoll06A245|Genit. examined|Spain|658[0n]
Polyommatus thersites|EZSPM454-09|Rvcoll.09-V908||Spain|658[0n]
Polyommatus thersites|EZSPM630-12|RVcoll.11-E038||Spain|658[0n]
Polyommatus thersites|EULEP5157-17|RVcoll10A865||Spain|658[0n]
Polyommatus thersites|EZSPN863-09|RVcoll.08-L733||Spain|658[0n]
Polyommatus thersites|EZSPN936-09|RVcoll.08-L896||Spain|658[0n]
Polyommatus thersites|EZROM752-08|RV-06-G453||Spain|658[0n]
Polyommatus thersites|WMB3341-14|RVcoll.08-P924||Spain|658[0n]
Polyommatus thersites|WMB3446-14|RVcoll.09-X576||Spain|658[0n]
Polyommatus thersites|WMB3254-14|RVcoll.08-L421||Spain|658[0n]
Polyommatus thersites|WMB3316-14|RVcoll.08-P632||Spain|658[0n]
Polyommatus thersites|WMB6568-18|RVcoll06A224|Genit. examined|Spain|658[0n]
Polyommatus thersites|WMB6569-18|RVcoll06A243|Genit. examined|Spain|658[0n]
Polyommatus thersites|WMB6571-18|RVcoll11E037||Spain|658[0n]
Polyommatus thersites|EZSPM588-12|RVcoll.09-V912||Spain|658[0n]
Polyommatus thersites|EZSPC287-09|RVcoll.08-P349||Spain|658[0n]
Polyommatus thersites|EZSPC286-09|RVcoll.06-A221||Spain|658[0n]
Polyommatus thersites|EZSPN917-09|RVcoll.08-L861||Spain|658[0n]
Polyommatus thersites|EZSPN149-09|RVcoll.08-R052||Spain|658[0n]
Polyommatus thersites|EZSPN716-09|RVcoll.08-L094||Spain|658[0n]
Polyommatus thersites|EZSPN750-09|RVcoll.08-L203||Spain|658[0n]
Polyommatus thersites|EULEP2171-15|RVcoll.14-K099||Switzerland|658[0n]
Polyommatus thersites|WMB2702-13|RVcoll.10-C087||France|658[0n]
Polyommatus thersites|PHLAW030-13|TLMF Lep 09827||Austria|658[0n]
Polyommatus thersites|PHLAW028-13|TLMF Lep 09825||Austria|626[0n]
Polyommatus thersites|PHLAW029-13|TLMF Lep 09826||Austria|658[0n]
Polyommatus escheri|EULEP4524-16|RVcoll14O561||Greece|634[0n]
Polyommatus escheri|EULEP1537-15|RVcoll.14-G497||Greece|658[0n]
Polyommatus escheri|EULEP1525-15|RVcoll.14-G468||Greece|617[0n]
Polyommatus escheri|EULEP1899-15|RVcoll.14-B770||Albania|658[0n]
Polyommatus escheri|EULEP1292-15|RVcoll.14-F717||Greece|658[0n]
Polyommatus escheri|EULEP1403-15|RVcoll.14-G057||Greece|658[0n]
Polyommatus escheri|EULEP871-15|RVcoll.14-C366||Greece|658[0n]
Polyommatus escheri|EULEP1246-15|RVcoll.14-F598||Greece|658[0n]
Polyommatus escheri|EULEP687-15|RVcoll.11-J904||Bulgaria|658[0n]
Polyommatus escheri|EULEP4521-16|RVcoll10A981||Bulgaria|658[0n]
Polyommatus escheri|OXB1592-16|OXB-TGS-1310||France|658[0n]
Polyommatus escheri|WMB3662-14|RVcoll.11-I783||France|658[0n]
Polyommatus escheri|WMB5139-14|RVcoll.07-E127||Italy|658[0n]
Polyommatus escheri|BIBSA267-15|RVcoll.14-E066||Italy|658[0n]
Polyommatus escheri|EULEP4523-16|RVcoll15G466||Switzerland|624[0n]
Polyommatus escheri|EULEP4522-16|RVcoll15G271||Switzerland|636[0n]
Polyommatus escheri|WMB2384-13|RVcoll.07-E208||Italy|658[0n]
Polyommatus escheri|WMB1610-13|RVcoll.12-O704||France|632[0n]
Polyommatus escheri|WMB816-13|RVcoll.07-E151||Italy|658[0n]
Polyommatus escheri|WMB3740-14|RVcoll.12-P865||France|658[0n]
Polyommatus escheri|WMB3496-14|RVcoll.10-B705||France|658[0n]
Polyommatus escheri|BIBSA650-15|RVcoll. 15-A813||Italy|658[0n]
Polyommatus escheri|BIBSA1406-15|15-N013||Italy|658[0n]
Polyommatus escheri|BCLEP118-17|LEP-SS-00569||Italy|658[0n]
Polyommatus escheri|WMB2255-13|RVcoll.12-R409||Italy|658[0n]
Polyommatus escheri|WMB199-11|RVcoll.07-W060||France|658[0n]
Polyommatus escheri|EZSPN1016-09|RVcoll.08-M941||Spain|658[0n]
Polyommatus escheri|GBMIN20562-13|JX093465||Spain|1495[60n]
Polyommatus escheri|WMB3415-14|RVcoll.09-X013||Spain|658[0n]
Polyommatus escheri|EZSPC1326-10|RVcoll.130209KL20||Spain|658[0n]
Polyommatus escheri|EZSPN1011-09|RVcoll.08-M924||Spain|658[0n]
Polyommatus escheri|EZSPN748-09|RVcoll.08-L192||Spain|658[0n]
Polyommatus escheri|EZSPN719-09|RVcoll.08-L105||Spain|658[0n]
Polyommatus escheri|EZSPN192-09|RVcoll.07-C477||Spain|658[0n]
Polyommatus escheri|WMB4518-14|RVcoll.14-E216||Spain|658[0n]
Polyommatus escheri|EZSPC926-10|RVcoll.09-V923||Spain|658[0n]
Polyommatus escheri|EZSPC925-10|RVcoll.09-V921||Spain|647[0n]
Polyommatus escheri|EZSPM755-12|Rvcoll. 12-L163||Spain|621[0n]
Polyommatus escheri|EZSPC774-10|RVcoll.08-L787||Spain|658[0n]
Polyommatus escheri|WMB3380-14|RVcoll.09-V351||Spain|658[0n]
Polyommatus escheri|EZSPM075-09|RVcoll.08-P319||Spain|658[0n]
Polyommatus escheri|EZSPN663-09|RVcoll.08-J966||Spain|658[0n]
Polyommatus escheri|EZSPC742-10|RVcoll.08-J995||Spain|658[0n]
Polyommatus escheri|EZSPC901-10|RVcoll.09-V518||Spain|619[1n]
Polyommatus escheri|EZSPN669-09|RVcoll.08-J975||Spain|658[0n]
Polyommatus escheri|EZSPN668-09|RVcoll.08-J973||Spain|658[0n]
Polyommatus escheri|EZSPN667-09|RVcoll.08-J971||Spain|622[0n]
Polyommatus escheri|EZSPC786-10|RVcoll.08-M036||Spain|658[0n]
Polyommatus escheri|EZSPN642-09|RVcoll.08-J776||Spain|658[0n]
Polyommatus escheri|EZSPC775-10|RVcoll.08-L791||Spain|658[0n]
Polyommatus escheri|EZSPC1272-10|RVcoll.280808DF13||Spain|658[0n]
Polyommatus escheri|EZSPC1321-10|RVcoll.130209KL53||Spain|658[0n]
Polyommatus escheri|EZSPM745-12|Rvcoll. 12-L052||Spain|621[0n]
Polyommatus escheri|EZSPN882-09|RVcoll.08-L789||Spain|637[0n]
Polyommatus celina|EZSPN317-09|RVcoll.08-H177|Genit. examined|Spain|658[0n]
Polyommatus celina|GBGL20032-15|KM459295|Menorca|Spain|655[0n]
Polyommatus celina|GBGL19982-15|KM459240|Sardinia|Italy|655[0n]
Polyommatus celina|GBGL20037-15|KM459300|Sardinia|Italy|655[0n]
Polyommatus celina|BIBSA1317-15|12-O457|Sardinia|Italy|658[0n]
Polyommatus celina|GBGL19938-15|KM459187|Sardinia|Italy|655[0n]
Polyommatus celina|BIBSA1284-15|13-T681|Sardinia|Italy|658[0n]
Polyommatus celina|GBGL19984-15|KM459242|Sardinia|Italy|655[0n]
Polyommatus celina|GBGL20035-15|KM459298|Sant Antioco|Italy|655[0n]

[illegible]
Polyommatus celina|BIBSA1284-15|13-T681|Sardinia|Italy|658[0n]
Polyommatus celina|GBGL19984-15|KM459242|Sardinia|Italy|655[0n]
Polyommatus celina|GBGL20035-15|KM459298|Sant Antioco|Italy|655[0n]
Polyommatus celina|GBMIN32585-13|JN084664|Mallorca|Spain|676[0n]
Polyommatus celina|GBGL19939-15|KM459188|Sardinia|Italy|655[0n]
Polyommatus celina|GBGL19985-15|KM459243|Sardinia|Italy|655[0n]
Polyommatus celina|EULEP046-14|RVcoll.08-H191|Genit. examined|Spain|676[0n]
Polyommatus celina|EULEP047-14|RVcoll.08-H195|Genit. examined|Spain|676[0n]
Polyommatus celina|EULEP096-14|RVcoll.09-T570|Genit. examined|Italy|676[0n]
Polyommatus celina|EULEP110-14|RVcoll.09-X105|Genit. examined|Spain|676[0n]
Polyommatus celina|EULEP111-14|RVcoll.09-X106|Genit. examined|Spain|676[0n]
Polyommatus celina|EULEP112-14|RVcoll.09-X114|Genit. examined|Spain|676[0n]
Polyommatus celina|GBMIN32562-13|JN084663|Sardinia|Italy|676[0n]
Polyommatus celina|GBMIN32586-13|JN084662|Menorca|Spain|676[0n]
Polyommatus celina|WMB2996-14|RVcoll.12-O408|San Pietro|Italy|658[0n]
Polyommatus celina|WMB2997-14|RVcoll.12-O409|San Pietro|Italy|658[0n]
Polyommatus celina|EULEP148-14|RVcoll.10-C449||Spain|658[0n]
Polyommatus celina|EULEP153-14|RVcoll.11-D598||Spain|658[0n]
Polyommatus celina|BIBSA1325-15|12-Q480|Sardinia|Italy|658[0n]
Polyommatus celina|GBGL19970-15|KM459226|Ibiza|Spain|655[0n]
Polyommatus celina|GBGL19986-15|KM459244|Sardinia|Italy|655[0n]
Polyommatus celina|GBGL20047-15|KM459310|Formentera|Spain|655[0n]
Polyommatus celina|GBGL20050-15|KM459313|Ibiza|Spain|655[0n]
Polyommatus celina|GBGL20046-15|KM459309|Formentera|Spain|655[0n]
Polyommatus celina|GBGL20048-15|KM459311|Ibiza|Spain|655[0n]
Polyommatus celina|OXB1244-15|Rvcoll_13-T694|Sardinia|Italy|658[0n]
Polyommatus celina|GBGL19983-15|KM459241|Sardinia|Italy|655[0n]
Polyommatus celina|GBGL19987-15|KM459245|Sardinia|Italy|655[0n]
Polyommatus celina|GBMIN32561-13|JN084665|Sardinia|Italy|676[0n]
Polyommatus celina|GBGL20033-15|KM459296|Sardinia|Italy|655[0n]
Polyommatus celina|BIBSA1282-15|13-T668|Sardinia|Italy|612[0n]
Polyommatus celina|WMB6541-18|RVcollLD0414|Sardinia. Genit. examined|Italy|658[0n]
Polyommatus celina|GBGL20049-15|KM459312|Ibiza|Spain|643[0n]
Polyommatus celina|GBGL20034-15|KM459297|Sardinia|Italy|616[0n]
Polyommatus celina|EZSPM884-12|Rvcoll.12-M602||Spain|642[0n]
Polyommatus celina|EZSPM883-12|Rvcoll.12-M601||Spain|658[0n]
Polyommatus celina|EZSPC799-10|RVcoll.08-P310||Spain|658[0n]
Polyommatus celina|EZSPC797-10|RVcoll.08-P297||Spain|658[0n]
Polyommatus celina|EZSPM057-09|RVcoll.08-P282|Genit. examined|Spain|658[0n]
Polyommatus celina|EZSPM039-09|RVcoll.08-P244|Genit. examined|Spain|658[0n]
Polyommatus celina|EZSPN314-09|RVcoll.08-H172|Genit. examined|Spain|658[0n]
Polyommatus celina|GBGL20044-15|KM459307|Malta|Malta|655[0n]
Polyommatus celina|GBGL20043-15|KM459306|Malta|Malta|655[0n]
Polyommatus celina|GBGL20016-15|KM459274|Vulcano|Italy|655[0n]
Polyommatus celina|GBGL20017-15|KM459275|Vulcano|Italy|655[0n]
Polyommatus celina|GBGL20052-15|KM459315|Ustica|Italy|655[0n]
Polyommatus celina|GBGL20041-15|KM459304|Ustica|Italy|655[0n]
Polyommatus celina|GBGL20025-15|KM459283|Sicily|Italy|655[0n]
Polyommatus celina|GBGL19964-15|KM459215|Lipari|Italy|655[0n]
Polyommatus celina|GBMIN32578-13|JN084678|Sicily|Italy|676[0n]
Polyommatus celina|GBGL20042-15|KM459305|Malta|Malta|655[0n]
Polyommatus celina|GBGL20057-15|KM459320|Malta|Malta|655[0n]
Polyommatus celina|GBGL20055-15|KM459318|Malta|Malta|655[0n]
Polyommatus celina|GBGL20021-15|KM459279|Salina|Italy|655[0n]
Polyommatus celina|GBGL19981-15|KM459239|Gozo|Malta|655[0n]
Polyommatus celina|GBGL19979-15|KM459237|Gozo|Malta|655[0n]
Polyommatus celina|GBGL19977-15|KM459235|Gozo|Malta|655[0n]
Polyommatus celina|EULEP5733-17|RVcollLD1572|Lipari|Italy|676[0n]
Polyommatus celina|GBGL20056-15|KM459319|Malta|Malta|655[0n]
Polyommatus celina|GBGL19963-15|KM459214|Lipari|Italy|655[0n]
Polyommatus celina|GBGL19965-15|KM459216|Lipari|Italy|655[0n]
Polyommatus celina|GBGL19961-15|KM459211|Sicily|Italy|655[0n]
Polyommatus celina|GBGL20053-15|KM459316|Ustica|Italy|655[0n]
Polyommatus celina|GBGL19975-15|KM459233|Malta|Malta|655[0n]
Polyommatus celina|GBMIN32553-13|JN084681|Sicily|Italy|658[0n]
Polyommatus celina|GBGL20040-15|KM459303|Ustica|Italy|655[0n]
Polyommatus celina|GBGL19978-15|KM459236|Gozo|Malta|655[0n]
Polyommatus celina|GBGL20036-15|KM459299|Sicily|Italy|655[0n]
Polyommatus celina|GBGL20023-15|KM459281|Salina|Italy|655[0n]
Polyommatus celina|GBGL20018-15|KM459276|Vulcano|Italy|655[0n]
Polyommatus celina|GBGL20015-15|KM459273|Vulcano|Italy|655[0n]
Polyommatus celina|GBGL20012-15|KM459270|Levanzo|Italy|655[0n]
Polyommatus celina|GBGL20011-15|KM459269|Levanzo|Italy|655[0n]
Polyommatus celina|GBGL19997-15|KM459255|Sicily|Italy|655[0n]
Polyommatus celina|GBGL19996-15|KM459254|Sicily|Italy|655[0n]
Polyommatus celina|GBGL19980-15|KM459238|Gozo|Malta|655[0n]
Polyommatus celina|GBGL19972-15|KM459229|Sicily|Italy|655[0n]
Polyommatus celina|OXB690-15|12-M386|Sicily W|Italy|658[0n]
Polyommatus celina|BIBSA1790-16|16-A527||Italy|658[0n]
Polyommatus celina|GBGL19973-15|KM459230|Sicily|Italy|655[0n]
Polyommatus celina|GBGL20024-15|KM459282|Sicily|Italy|655[0n]
Polyommatus celina|GBMIN32579-13|JN084676|Sicily|Italy|676[0n]
Polyommatus celina|GBGL20054-15|KM459317|Malta|Malta|655[0n]
Polyommatus celina|GBGL20039-15|KM459302|Sicily|Italy|655[0n]
Polyommatus celina|GBGL20038-15|KM459301|Sicily|Italy|655[0n]
Polyommatus celina|GBGL20022-15|KM459280|Salina|Italy|655[0n]
Polyommatus celina|GBGL20019-15|KM459277|Salina|Italy|655[0n]
Polyommatus celina|GBGL20014-15|KM459272|Levanzo|Italy|655[0n]
Polyommatus celina|GBGL20013-15|KM459271|Levanzo|Italy|655[0n]
Polyommatus celina|GBGL20010-15|KM459268|Levanzo|Italy|655[0n]
Polyommatus celina|GBGL19976-15|KM459234|Gozo|Malta|655[0n]
Polyommatus celina|GBGL19962-15|KM459212|Sicily|Italy|655[0n]
Polyommatus celina|GBGL19974-15|KM459231|Sicily|Italy|655[0n]
Polyommatus celina|EULEP5732-17|RVcollLD1571|Lipari|Italy|676[0n]
Polyommatus celina|GBGL19960-15|KM459210|Sicily|Italy|655[0n]
Polyommatus celina|GBGL20051-15|KM459314|Sicily|Italy|655[0n]
Polyommatus celina|GBGL20026-15|KM459284|Sicily|Italy|655[0n]
Polyommatus celina|GBGL20020-15|KM459278|Salina|Italy|655[0n]
Polyommatus celina|GBGL19959-15|KM459209|Sicily|Italy|655[0n]
Polyommatus celina|EULEP094-14|RVcoll.09-T513|Genit. examined|Italy|658[0n]
Polyommatus celina|WMB5475-14|RVcoll.14-N694|Alicudi|Italy|658[0n]
Polyommatus celina|EULEP117-14|RVcoll.09-X369||Spain|658[0n]
[illegible]

Polyommatus celina|EULEP094-14|RVcoll.09-1513|Genit. examined|Italy|658[0n]
Polyommatus celina|WMB5475-14|RVcoll.14-N694|Alicudi|Italy|658[0n]
Polyommatus celina|EULEP117-14|RVcoll.09-X369||Spain|658[0n]
Polyommatus celina|WMB6533-18|RVcoll11D664||Spain|658[3n]
Polyommatus celina|WMB4426-14|RVcoll.14-B484||Portugal|658[0n]
Polyommatus celina|GBGL19942-15|KM459191||Spain|655[0n]
Polyommatus celina|GBGL20045-15|KM459308|Malta|Malta|655[0n]
Polyommatus celina|GBGL20006-15|KM459264|Pantelleria|Italy|655[0n]
Polyommatus celina|WMB4390-14|RVcoll.14-B304||Portugal|658[0n]
Polyommatus celina|EZSPM957-12|Rvcoll.12-M708||Spain|658[0n]
Polyommatus celina|GBMIN32582-13|JN084670||Spain|676[0n]
Polyommatus celina|GBGL19950-15|KM459199||Spain|655[0n]
Polyommatus celina|WMB4560-14|RVcoll.090211SD56||Spain|658[0n]
Polyommatus celina|GBGL19999-15|KM459257|Lampedusa|Italy|655[0n]
Polyommatus celina|GBGL20000-15|KM459258|Lampedusa|Italy|655[0n]
Polyommatus celina|GBGL19998-15|KM459256|Lampedusa|Italy|655[0n]
Polyommatus celina|EULEP072-14|RVcoll.08-L130||Spain|658[0n]
Polyommatus celina|WMB3559-14|RVcoll.11-D905||Spain|658[0n]
Polyommatus celina|GBMIN32583-13|JN084668||Spain|676[0n]
Polyommatus celina|GBGL20007-15|KM459265|Pantelleria|Italy|655[0n]
Polyommatus celina|GBGL20003-15|KM459261|Lampedusa|Italy|655[0n]
Polyommatus celina|GBGL20001-15|KM459259|Lampedusa|Italy|655[0n]
Polyommatus celina|GBGL19969-15|KM459225||Spain|655[0n]
Polyommatus celina|GBGL19968-15|KM459223||Spain|655[0n]
Polyommatus celina|GBGL19949-15|KM459198||Spain|655[0n]
Polyommatus celina|GBGL19948-15|KM459197||Spain|655[0n]
Polyommatus celina|GBGL19937-15|KM459186||Spain|655[0n]
Polyommatus celina|GBGL19935-15|KM459184||Spain|655[0n]
Polyommatus celina|WMB4420-14|RVcoll.14-B471||Portugal|658[0n]
Polyommatus celina|WMB3183-14|RVcoll.08-H936||Spain|658[0n]
Polyommatus celina|EZSPC729-10|RVcoll.08-H634||Spain|658[0n]
Polyommatus celina|EZSPM756-12|Rvcoll. 12-L164||Spain|658[0n]
Polyommatus celina|EZSPN711-09|RVcoll.08-L063|Genit. examined|Spain|658[0n]
Polyommatus celina|GBGL20005-15|KM459263|Pantelleria|Italy|655[0n]
Polyommatus celina|GBMIN32556-13|JN084675||Spain|676[0n]
Polyommatus celina|GBGL19952-15|KM459201||Spain|655[0n]
Polyommatus celina|EULEP241-14|RVcoll.12-M686||Spain|658[0n]
Polyommatus celina|EZSPN514-09|RVcoll.08-H977||Spain|658[0n]
Polyommatus celina|EULEP163-14|RVcoll.11-I455||Spain|658[0n]
Polyommatus celina|EZSPN446-09|RVcoll.08-H637|Genit. examined|Spain|658[0n]
Polyommatus celina|GBMIN32581-13|JN084672||Spain|676[0n]
Polyommatus celina|GBMIN32557-13|JN084673||Spain|676[0n]
Polyommatus celina|GBGL19941-15|KM459190||Spain|655[0n]
Polyommatus celina|GBGL19951-15|KM459200||Spain|655[0n]
Polyommatus celina|GBGL20009-15|KM459267|Pantelleria|Italy|655[0n]
Polyommatus celina|GBGL20008-15|KM459266|Pantelleria|Italy|655[0n]
Polyommatus celina|GBGL20004-15|KM459262|Lampedusa|Italy|655[0n]
Polyommatus celina|GBGL20002-15|KM459260|Lampedusa|Italy|655[0n]
Polyommatus celina|GBGL19971-15|KM459228|Pantelleria|Italy|655[0n]
Polyommatus celina|GBGL19967-15|KM459222||Spain|655[0n]
Polyommatus celina|GBGL19947-15|KM459196||Spain|655[0n]
Polyommatus celina|EULEP005-14|RVcoll.06-A042||Spain|658[0n]
Polyommatus celina|WMB3259-14|RVcoll.08-L623||Spain|658[0n]
Polyommatus celina|EZSPC694-10|RVcoll.09-V472||Spain|658[0n]
Polyommatus celina|EZSPM869-12|Rvcoll.090211SD59||Spain|658[0n]
Polyommatus celina|EZSPM466-09|Rvcoll.09-V900|Genit. examined|Spain|658[0n]
Polyommatus celina|EZSPM971-12|Rvcoll.12-M729||Spain|658[0n]
Polyommatus celina|EZSPN817-09|RVcoll.08-L622||Spain|658[0n]
Polyommatus celina|GBGL19930-15|KM459176||Spain|655[0n]
Polyommatus celina|GBGL19946-15|KM459195||Spain|655[0n]
Polyommatus celina|EZSPN169-09|RVcoll.06-K695|Genit. examined|Spain|658[0n]
Polyommatus eros|EZSPN260-09|RVcoll.07-W164||Spain|658[0n]
Polyommatus eros|EULEP1426-15|RVcoll.14-G103||Greece|658[0n]
Polyommatus eros|GBMIN32572-13|JN084690||Greece|658[0n]
Polyommatus eros|EULEP1415-15|RVcoll.14-G085||Greece|658[0n]
Polyommatus eros|EULEP1407-15|RVcoll.14-G072||Greece|658[0n]
Polyommatus eros|EULEP1404-15|RVcoll.14-G066||Greece|658[0n]
Polyommatus eros|EULEP1383-15|RVcoll.14-F984||Greece|658[0n]
Polyommatus eros|EULEP1377-15|RVcoll.14-F975||Greece|658[0n]
Polyommatus eros|EULEP2366-15|RVcoll.14-V059||Ukraine|658[0n]
Polyommatus eros|EULEP2242-15|RVcoll.14-N424||Ukraine|658[0n]
Polyommatus eros|EZSPC1166-10|RVcoll.09-T064||Spain|658[0n]
Polyommatus eros|EZSPN275-09|RVcoll.07-W200||Spain|658[0n]
Polyommatus eros|EZSPN274-09|RVcoll.07-W199||Spain|658[0n]
Polyommatus eros|EZSPM196-09|RVcoll.08-R233||Spain|658[0n]
Polyommatus eros|EZSPM195-09|RVcoll.08-R232||Spain|658[0n]
Polyommatus eros|EZSPN259-09|RVcoll.07-W163||Spain|658[0n]
Polyommatus eros|EZSPC1251-10|RVcoll.09-V839||Spain|658[0n]
Polyommatus eros|EZSPM410-09|Rvcoll.09-V828||Spain|658[0n]
Polyommatus eros|EZSPM185-09|RVcoll.08-R181||Spain|611[0n]
Polyommatus eros|WMB6573-18|RVcoll14A084||Italy|658[0n]
Polyommatus eros|WMB6572-18|RVcoll14A083||Italy|658[0n]
Polyommatus eros|GBMIN32551-13|JN084685||Italy|676[0n]
Polyommatus eros|BIBSA1355-15|15-M856||Italy|618[0n]
Polyommatus eros|BIBSA1344-15|15-M837||Italy|658[0n]
Polyommatus eros|GBMIN32552-13|JN084683||Bulgaria|676[0n]
Polyommatus eros|EULEP1144-15|RVcoll.14-F306||Serbia|658[0n]
Polyommatus eros|GBMIN32576-13|JN084682||Bulgaria|676[0n]
Polyommatus eros|EULEP950-15|RVcoll.14-C958||Bulgaria|658[0n]
Polyommatus eros|EULEP919-15|RVcoll.14-C790||Bulgaria|658[0n]
Polyommatus eros|EULEP1248-15|RVcoll.14-F605||Greece|658[0n]
Polyommatus eros|EULEP4514-16|RVcoll10B401||Bulgaria|658[0n]
Polyommatus eros|EULEP4513-16|RVcoll14O332||Bulgaria|644[0n]
Polyommatus eros|BIBSA315-15|RVcoll.14-E117||Italy|658[0n]
Polyommatus eros|WMB2674-13|RVcoll.10-B865||France|658[0n]
Polyommatus eros|BIBSA1189-15|15-M250||Italy|658[0n]
Polyommatus eros|EULEP4520-16|RVcoll15G314||Switzerland|636[0n]
Polyommatus eros|PHLAA341-09|TLMF Lep 00381||Austria|656[0n]
Polyommatus eros|LEATG570-14|TLMF Lep 14357||Austria|658[0n]
Polyommatus eros|WMB2701-13|RVcoll.10-C085||France|658[0n]
Polyommatus eros|PHLSA398-11|TLMF Lep 05853||Austria|658[0n]
Polyommatus eros|EULEP4516-16|RVcoll15H580||Switzerland|658[0n]
Polyommatus eros|EULEP4515-16|RVcoll15G864||Switzerland|658[0n]

Polyommatus eros|PHLSA398-11|TLMF Lep 05853||Austria|658[0n]
Polyommatus eros|EULEP4516-16|RVcoll15H580||Switzerland|658[0n]
Polyommatus eros|EULEP4515-16|RVcoll15G864||Switzerland|658[0n]
Polyommatus eros|EULEP4518-16|RVcoll15I764||Austria|658[0n]
Polyommatus eros|EULEP4517-16|RVcoll15I269||Austria|658[0n]
Polyommatus eros|GBMIN32575-13|JN084684||Switzerland|676[0n]
Polyommatus eros|LEASS528-17|TLMF Lep 22176||Austria|658[0n]
Polyommatus eros|BIBSA313-15|RVcoll.14-E115||Italy|658[0n]
Polyommatus eros|BIBSA429-15|RVcoll.14-I097||Italy|658[0n]
Polyommatus eros|EULEP2465-15|RVcoll.14-V291||Switzerland|658[0n]
Polyommatus eros|EULEP1523-15|RVcoll.14-G453||Greece|658[0n]
Polyommatus eros|EULEP1140-15|RVcoll.14-F299||Serbia|658[0n]
Polyommatus eros|EULEP4519-16|RVcoll15J298||France|658[0n]
Polyommatus eros|PHLSA397-11|TLMF Lep 05852||Austria|658[0n]
Polyommatus eros|PHLAI502-13|TLMF Lep 09064||Austria|607[0n]
Polyommatus icarus|GBMIN32548-13|JN084691|Crete|Greece|676[1n]
Polyommatus icarus|GBMIN32539-13|JN084709|Crete|Greece|611[0n]
Polyommatus icarus|GBMIN32571-13|JN084692|Crete|Greece|676[0n]
Polyommatus icarus|EULEP004-14|150308PP67|Crete. Genit. examined|Greece|658[1n]
Polyommatus icarus|LON231-08|NHMO-07020||Norway|657[5n]
Polyommatus icarus|EULEP160-14|RVcoll.11-E016||Spain|658[0n]
Polyommatus icarus|EULEP069-14|RVcoll.08-J962|Genit. examined|Spain|676[0n]
Polyommatus icarus|EULEP104-14|RVcoll.09-V446|Genit. examined|Spain|676[0n]
Polyommatus icarus|GBGL20127-15|KM459412||Spain|655[0n]
Polyommatus icarus|GBGL20126-15|KM459411||Spain|655[0n]
Polyommatus icarus|GBGL20125-15|KM459409||Spain|655[0n]
Polyommatus icarus|EULEP164-14|RVcoll.11-I458||Spain|658[0n]
Polyommatus icarus|EULEP162-14|RVcoll.11-I454||Spain|658[0n]
Polyommatus icarus|EZSPN665-09|RVcoll.08-J968||Spain|658[0n]
Polyommatus icarus|EZSPN661-09|RVcoll.08-J960|Genit. examined|Spain|633[0n]
Polyommatus icarus|OXB418-15|OXB-TGS-100||United Kingdom|658[0n]
Polyommatus icarus|OXB553-15|OXB-TGS-928||United Kingdom|658[0n]
Polyommatus icarus|OXB552-15|OXB-TGS-927||United Kingdom|658[0n]
Polyommatus icarus|OXB551-15|OXB-TGS-926||United Kingdom|658[0n]
Polyommatus icarus|OXB423-15|OXB-TGS-105||United Kingdom|658[0n]
Polyommatus icarus|OXB422-15|OXB-TGS-104||United Kingdom|658[0n]
Polyommatus icarus|OXB421-15|OXB-TGS-103||United Kingdom|658[0n]
Polyommatus icarus|OXB420-15|OXB-TGS-102||United Kingdom|658[0n]
Polyommatus icarus|OXB419-15|OXB-TGS-101||United Kingdom|658[0n]
Polyommatus icarus|OXB398-15|OXB-TGS-062||United Kingdom|658[0n]
Polyommatus icarus|OXB397-15|OXB-TGS-061||United Kingdom|658[0n]
Polyommatus icarus|OXB396-15|OXB-TGS-035||United Kingdom|658[0n]
Polyommatus icarus|OXB390-15|OXB-TGS-020||United Kingdom|658[0n]
Polyommatus icarus|OXB388-15|OXB-TGS-018||United Kingdom|658[0n]
Polyommatus icarus|WMB4034-14|RVcoll.12-Z169||Ireland|658[0n]
Polyommatus icarus|OXB544-15|OXB-TGS-919||United Kingdom|658[0n]
Polyommatus icarus|WMB4020-14|RVcoll.12-R455||United Kingdom|658[0n]
Polyommatus icarus|EZSPN772-09|RVcoll.08-L281|Genit. examined|Spain|658[0n]
Polyommatus icarus|GBMIN32573-13|JN084688||France|676[0n]
Polyommatus icarus|GBGL20128-15|KM459414||France|655[0n]
Polyommatus icarus|LEASS697-17|TLMF Lep 22345||Austria|658[0n]
Polyommatus icarus|BIBSA909-15|16-A000||France|658[0n]
Polyommatus icarus|WMB3671-14|RVcoll.11-I974||France|658[0n]
Polyommatus icarus|FBLMT894-09|BC ZSM Lep 25454||Germany|658[0n]
Polyommatus icarus|LON634-09|NHMO-08152||Norway|657[0n]
Polyommatus icarus|GBMIN32568-13|JN084698||France|676[0n]
Polyommatus icarus|GBGL20103-15|KM459386||Italy|655[0n]
Polyommatus icarus|GBGL20087-15|KM459366||France|655[0n]
Polyommatus icarus|BIBSA1175-15|15-M205||Italy|658[0n]
Polyommatus icarus|WMB3998-14|RVcoll.12-Q454||Spain|658[0n]
Polyommatus icarus|WMB120-11|RVcoll.10-C178||France|658[0n]
Polyommatus icarus|EZROM751-08|RV-06-G433||Spain|658[0n]
Polyommatus icarus|GBGL20095-15|KM459378||Croatia|655[0n]
Polyommatus icarus|WMB4986-14|RVcoll. 14-I533||Italy|658[0n]
Polyommatus icarus|WMB6532-18|RVcoll10A994|Genit. examined|Bulgaria|658[0n]
Polyommatus icarus|BIBSA1209-15|15-M327||Italy|612[0n]
Polyommatus icarus|BIBSA268-15|RVcoll.14-E067||Italy|658[0n]
Polyommatus icarus|GBMIN32545-13|JN084697||Bulgaria|676[0n]
Polyommatus icarus|OXB582-15|14-N971||Italy|658[0n]
Polyommatus icarus|OXB364-15|RVcoll.14-N078||Italy|658[0n]
Polyommatus icarus|OXB992-15|15-A942||Italy|658[0n]
Polyommatus icarus|GBGL20141-15|KM459427||France|655[0n]
Polyommatus icarus|GBGL20124-15|KM459408|Argentario|Italy|655[0n]
Polyommatus icarus|OXB339-15|RVcoll.14-N053||Italy|658[0n]
Polyommatus icarus|LEATJ1211-16|TLMF Lep 19534||Croatia|658[0n]
Polyommatus icarus|BIBSA426-15|RVcoll.14-I094||Italy|610[0n]
Polyommatus icarus|BIBSA1139-15|15-L971||Italy|658[0n]
Polyommatus icarus|EULEP1397-15|RVcoll.14-G023||Greece|658[0n]
Polyommatus icarus|EULEP3484-16|RVcoll14O551||Greece|658[0n]
Polyommatus icarus|WMB5089-14|RVcoll. 14-L220||Italy|658[0n]
Polyommatus icarus|WMB5075-14|RVcoll. 14-L206||Italy|658[0n]
Polyommatus icarus|EZROM504-08|RV-07-C964|Genit. examined|Romania|658[0n]
Polyommatus icarus|EZROM501-08|RV-06-V653|Genit. examined|Romania|658[0n]
Polyommatus icarus|EZRMN208-08|RVcoll.08-M630||Romania|658[0n]
Polyommatus icarus|EULEP1957-15|RVcoll.14-G762||Romania|658[0n]
Polyommatus icarus|EULEP2351-15|RVcoll.14-V039||Ukraine|658[0n]
Polyommatus icarus|EZRMN209-08|RVcoll.08-M425|Genit. examined|Romania|658[0n]
Polyommatus icarus|OXB1334-15|Rvcoll_15-M678||France|658[0n]
Polyommatus icarus|EULEP4529-16|RVcoll15G529||Switzerland|658[0n]
Polyommatus icarus|FBLMT902-09|BC ZSM Lep 25462||Germany|658[0n]
Polyommatus icarus|FBLMW315-10|BC ZSM Lep 37416||Germany|658[0n]
Polyommatus icarus|GBMIN32544-13|JN084699||Spain|676[0n]
Polyommatus icarus|EZSPM465-09|Rvcoll.09-V941||Spain|658[0n]
Polyommatus icarus|WMB4447-14|RVcoll.14-D309||Spain|658[0n]
Polyommatus icarus|WMB6530-18|RVcoll09V903|Genit. examined|Spain|658[0n]
Polyommatus icarus|EULEP116-14|RVcoll.09-X366||Spain|658[0n]
Polyommatus icarus|EULEP118-14|RVcoll.09-X372||Spain|658[0n]
Polyommatus icarus|GBGL20088-15|KM459369||Spain|655[0n]
Polyommatus icarus|GBGL20090-15|KM459372||Spain|655[0n]
Polyommatus icarus|GBGL20091-15|KM459373||Spain|655[0n]
Polyommatus icarus|HBOK173-08|OK520-79||Spain|641[0n]
Polyommatus icarus|EULEP5722-17|RVcoll16L194||Italy|658[0n]
Polyommatus icarus|EULEP235-14|RVcoll.12-L152||Spain|658[0n]

[illegible]
Polyommatus icarus|HBOK173-08|OK520-79||Spain|641[0n]
Polyommatus icarus|EULEP5722-17|RVcoll16L194||Italy|658[0n]
Polyommatus icarus|EULEP235-14|RVcoll.12-L152||Spain|658[0n]
Polyommatus icarus|WMB119-11|RVcoll.10-C177||France|658[0n]
Polyommatus icarus|GBGL20111-15|KM459395|Corsica|France|655[0n]
Polyommatus icarus|BIBSA1335-15|15-M827||Italy|658[0n]
Polyommatus icarus|WMB6070-18|RVcollLD0384|Corsica. Genit. examined|France|658[0n]
Polyommatus icarus|GBMIN32565-13|JN084704||Spain|676[0n]
Polyommatus icarus|GBMIN32542-13|JN084703||Italy|676[0n]
Polyommatus icarus|GBGL20093-15|KM459376||France|655[0n]
Polyommatus icarus|WMB3838-14|RVcoll.10-C516||Italy|658[0n]
Polyommatus icarus|GBGL20145-15|KM459431||Italy|655[0n]
Polyommatus icarus|GBGL20138-15|KM459424|Corsica|France|655[0n]
Polyommatus icarus|GBGL20106-15|KM459390|Corsica|France|655[0n]
Polyommatus icarus|GBGL20105-15|KM459389|Corsica|France|655[0n]
Polyommatus icarus|GBGL20107-15|KM459391|Corsica|France|655[0n]
Polyommatus icarus|OXB1122-15|15-A655||Italy|658[0n]
Polyommatus icarus|WMB3476-14|RVcoll.10-A866||Spain|658[0n]
Polyommatus icarus|GBGL20152-15|KM459439|Capraia|Italy|655[0n]
Polyommatus icarus|WMB4984-14|RVcoll. 14-I531||Italy|658[0n]
Polyommatus icarus|WMB5172-14|RVcoll.13-S518||Italy|658[0n]
Polyommatus icarus|GBGL20100-15|KM459383||Italy|655[0n]
Polyommatus icarus|OXB1274-15|Rvcoll_15-M134||France|658[0n]
Polyommatus icarus|OXB1160-15|Rvcoll_16-A047||Italy|658[0n]
Polyommatus icarus|EZSPM751-12|Rvcoll. 12-L141||Spain|621[0n]
Polyommatus icarus|GBGL20084-15|KM459356||Italy|655[0n]
Polyommatus icarus|GBMIN32567-13|JN084700||Italy|676[0n]
Polyommatus icarus|GBMIN32566-13|JN084702||Italy|676[0n]
Polyommatus icarus|EULEP034-14|RVcoll.07-E051|Genit. examined|Italy|676[0n]
Polyommatus icarus|EULEP033-14|RVcoll.07-D899|Genit. examined|Italy|676[0n]
Polyommatus icarus|EULEP048-14|RVcoll.08-H255|Genit. examined|Spain|676[0n]
Polyommatus icarus|EULEP035-14|RVcoll.07-F062|Genit. examined|Spain|676[0n]
Polyommatus icarus|EULEP052-14|RVcoll.08-J199|Genit. examined|Portugal|676[0n]
Polyommatus icarus|EULEP051-14|RVcoll.08-J150|Genit. examined|Portugal|676[0n]
Polyommatus icarus|EULEP095-14|RVcoll.09-T560||Italy|676[0n]
Polyommatus icarus|EULEP071-14|RVcoll.08-L092|Genit. examined|Spain|676[0n]
Polyommatus icarus|EULEP032-14|RVcoll.07-D864|Genit. examined|Italy|676[0n]
Polyommatus icarus|EULEP102-14|RVcoll.09-V243|Genit. examined|France|676[0n]
Polyommatus icarus|EULEP120-14|RVcoll.09-X521|Genit. examined|Spain|676[0n]
Polyommatus icarus|EULEP268-14|RVcoll.LD-292||Italy|676[0n]
Polyommatus icarus|GBMIN32543-13|JN084701|Corsica|France|676[0n]
Polyommatus icarus|GBGL20135-15|KM459421|Argentario|Italy|655[0n]
Polyommatus icarus|GBGL20134-15|KM459420|Argentario|Italy|655[0n]
Polyommatus icarus|WMB2847-13|RVcoll.12-Q795||Italy|658[0n]
Polyommatus icarus|WMB3461-14|RVcoll.10-A606||France|658[0n]
Polyommatus icarus|GBGL20133-15|KM459419|Ischia|Italy|655[0n]
Polyommatus icarus|GBGL20132-15|KM459418|Ischia|Italy|655[0n]
Polyommatus icarus|GBGL20131-15|KM459417|Ischia|Italy|655[0n]
Polyommatus icarus|GBGL20130-15|KM459416|Elba|Italy|655[0n]
Polyommatus icarus|GBGL20129-15|KM459415|Elba|Italy|655[0n]
Polyommatus icarus|GBGL20122-15|KM459406|Argentario|Italy|655[0n]
Polyommatus icarus|GBGL20118-15|KM459402|Pianosa|Italy|655[0n]
Polyommatus icarus|GBGL20117-15|KM459401|Pianosa|Italy|655[0n]
Polyommatus icarus|GBGL20112-15|KM459396|Corsica|France|655[0n]
Polyommatus icarus|GBGL20110-15|KM459394|Corsica|France|655[0n]
Polyommatus icarus|GBGL20109-15|KM459393|Corsica|France|655[0n]
Polyommatus icarus|GBGL20104-15|KM459388|Corsica|France|655[0n]
Polyommatus icarus|GBGL20101-15|KM459384||Italy|655[0n]
Polyommatus icarus|GBGL20099-15|KM459382|Elba|Italy|655[0n]
Polyommatus icarus|GBGL20098-15|KM459381|Elba|Italy|655[0n]
Polyommatus icarus|GBGL20097-15|KM459380||France|655[0n]
Polyommatus icarus|GBMIN16302-13|JX678166|Corsica|France|657[0n]
Polyommatus icarus|OXB1590-16|OXB-TGS-1308||France|658[0n]
Polyommatus icarus|OXB1589-16|OXB-TGS-1307||France|658[0n]
Polyommatus icarus|OXB1553-16|OXB-TGS-1271||Andorra|658[0n]
Polyommatus icarus|OXB1552-16|OXB-TGS-1270||Andorra|658[0n]
Polyommatus icarus|OXB1077-15|15-A610||Italy|658[0n]
Polyommatus icarus|OXB954-15|15-A904||Italy|658[0n]
Polyommatus icarus|OXB904-15|15-A548||Italy|658[0n]
Polyommatus icarus|OXB890-15|15-A532||Italy|658[0n]
Polyommatus icarus|OXB588-15|14-N977||Italy|658[0n]
Polyommatus icarus|OXB877-15|15-A519||Italy|658[0n]
Polyommatus icarus|BIBSA043-14|LEP-SS-00042||Italy|658[0n]
Polyommatus icarus|BIBSA042-14|LEP-SS-00041||Italy|658[0n]
Polyommatus icarus|BIBSA969-15|14-A121||Italy|658[0n]
Polyommatus icarus|BIBSA1389-15|15-M985||Italy|658[0n]
Polyommatus icarus|BIBSA120-15|RVcoll.14-D555||Italy|658[0n]
Polyommatus icarus|BIBSA1044-15|15-C233||Italy|658[0n]
Polyommatus icarus|BIBSA1017-15|15-C144||Italy|658[0n]
Polyommatus icarus|GBGL20144-15|KM459430||Italy|655[0n]
Polyommatus icarus|GBGL20143-15|KM459429||France|655[0n]
Polyommatus icarus|BIBSA978-15|14-W014||Italy|658[0n]
Polyommatus icarus|EULEP237-14|RVcoll.12-L155||Spain|658[0n]
Polyommatus icarus|GBGL20140-15|KM459426|Corsica|France|655[0n]
Polyommatus icarus|GBGL20139-15|KM459425|Corsica|France|655[0n]
Polyommatus icarus|EULEP236-14|RVcoll.12-L154||Spain|658[0n]
Polyommatus icarus|EULEP234-14|RVcoll.12-L149||Spain|658[0n]
Polyommatus icarus|EULEP097-14|RVcoll.09-T572|Genit. examined|Italy|658[0n]
Polyommatus icarus|WMB5153-14|RVcoll.12-P526||France|658[0n]
Polyommatus icarus|WMB5062-14|RVcoll. 14-L193||Italy|658[0n]
Polyommatus icarus|WMB4780-14|RVcoll. 14-I327||Italy|658[0n]
Polyommatus icarus|WMB4480-14|RVcoll.14-D922||Italy|658[0n]
Polyommatus icarus|WMB4300-14|RVcoll.14-A397||Italy|658[0n]
Polyommatus icarus|WMB4293-14|RVcoll.14-A389||Italy|658[0n]
Polyommatus icarus|WMB4142-14|RVcoll.13-S657||Italy|658[0n]
Polyommatus icarus|WMB3223-14|RVcoll.08-J743||Spain|658[0n]
Polyommatus icarus|WMB3040-14|RVcoll.13-S568|San Domino|Italy|658[0n]
Polyommatus icarus|WMB3438-14|RVcoll.09-X551||Spain|658[0n]
Polyommatus icarus|EZSPC759-10|RVcoll.08-L276||Spain|658[0n]
Polyommatus icarus|EZSPM754-12|Rvcoll. 12-L159||Spain|658[0n]
Polyommatus icarus|EZSPC284-09|RVcoll.07-C463|Genit. examined|Spain|658[0n]
Polyommatus icarus|EZSPM753-12|Rvcoll. 12-L153||Spain|658[0n]

Polyommatus icarus|EZSPM754-12|Rvcoll. 12-L159||Spain|658[0n]
Polyommatus icarus|EZSPC284-09|RVcoll.07-C463|Genit. examined|Spain|658[0n]
Polyommatus icarus|EZSPM753-12|Rvcoll. 12-L153||Spain|658[0n]
Polyommatus icarus|EZSPM752-12|Rvcoll. 12-L143||Spain|658[0n]
Polyommatus icarus|EZSPM345-09|RVcoll.08-R025||Spain|658[0n]
Polyommatus icarus|EZSPN578-09|RVcoll.08-J127|Genit. examined|Portugal|658[0n]
Polyommatus icarus|EZSPM948-12|Rvcoll.12-M687||Spain|658[0n]
Polyommatus icarus|EZSPN384-09|RVcoll.08-H434|Genit. examined|Spain|658[0n]
Polyommatus icarus|EZSPN426-09|RVcoll.08-H592|Genit. examined|Spain|658[0n]
Polyommatus icarus|EZSPN142-09|RVcoll.06-G507|Genit. examined|Spain|658[0n]
Polyommatus icarus|EZSPC669-09|RVcoll.08-M674||Spain|658[0n]
Polyommatus icarus|EZSPC662-09|RVcoll.08-H232||Spain|658[0n]
Polyommatus icarus|GWORZ057-10|BC ZSM Lep 30413||Italy|658[0n]
Polyommatus icarus|GWORR418-10|BC ZSM Lep 29634||Italy|658[0n]
Polyommatus icarus|GBGL20147-15|KM459433|Corsica|France|655[0n]
Polyommatus icarus|GBGL20146-15|KM459432|Corsica|France|655[0n]
Polyommatus icarus|GBGL20096-15|KM459379||Spain|655[0n]
Polyommatus icarus|GBGL20092-15|KM459375||Italy|655[0n]
Polyommatus icarus|GBGL20149-15|KM459435|Ischia|Italy|655[0n]
Polyommatus icarus|GBGL20148-15|KM459434|Ischia|Italy|655[0n]
Polyommatus icarus|GBGL20150-15|KM459436||Italy|655[0n]
Polyommatus icarus|GBGL20102-15|KM459385||Italy|655[0n]
Polyommatus icarus|GBGL20113-15|KM459397|Corsica|France|655[0n]
Polyommatus icarus|GBGL20114-15|KM459398||Italy|655[0n]
Polyommatus icarus|GBGL20119-15|KM459403|Pianosa|Italy|655[0n]
Polyommatus icarus|WMB3688-14|RVcoll.11-J235||Spain|658[0n]
Polyommatus icarus|GBGL20116-15|KM459400||Italy|655[0n]
Polyommatus icarus|GBGL20120-15|KM459404|Pianosa|Italy|655[0n]
Polyommatus icarus|GBGL20115-15|KM459399||Italy|655[0n]
Polyommatus icarus|GBGL20121-15|KM459405|Pianosa|Italy|655[0n]
Polyommatus icarus|GBGL20059-15|KM459322|Capraia|Italy|655[0n]
Polyommatus icarus|GBGL20137-15|KM459423||Italy|655[0n]
Polyommatus icarus|GBGL20069-15|KM459337||Spain|673[0n]
Polyommatus icarus|GBGL20058-15|KM459321|Capraia|Italy|655[2n]
Polyommatus icarus|OXB1002-15|15-A952||Italy|632[0n]
Polyommatus icarus|GBGL20151-15|KM459438|Corsica|France|640[0n]
Polyommatus icarus|GBGL20108-15|KM459392|Corsica|France|655[0n]
Polyommatus icarus|BIBSA340-15|RVcoll.14-I008||Italy|632[0n]
Polyommatus icarus|BIBSA1294-15|14-E286|Corsica|France|618[0n]
Polyommatus icarus|WMB4467-14|RVcoll.14-D348||Spain|614[0n]
Polyommatus icarus|EZSPM325-09|RVcoll.08-J899||Spain|658[1n]
Polyommatus icarus|GWORU349-10|BC ZSM Lep 31940||Italy|658[0n]
Polyommatus icarus|GWORR414-10|BC ZSM Lep 29630||Italy|658[0n]
Polyommatus icarus|LEFIJ521-10|MM17146||Finland|658[0n]
Polyommatus icarus|OXB550-15|OXB-TGS-925||United Kingdom|658[0n]
Polyommatus icarus|OXB554-15|OXB-TGS-929||United Kingdom|658[0n]
Polyommatus icarus|WMB3993-14|RVcoll.12-Q394||France|658[0n]
Polyommatus icarus|OXB387-15|OXB-TGS-017||United Kingdom|658[0n]
Polyommatus icarus|OXB436-15|OXB-TGS-160||United Kingdom|622[0n]
Polyommatus icarus|GBMIN32541-13|JN084705||Italy|676[0n]
Polyommatus icarus|BIBSA1054-15|15-M991||Italy|658[0n]
Polyommatus icarus|GBMIN32564-13|JN084706|Capri|Italy|676[0n]
Polyommatus icarus|WMB6539-18|RVcoll14A082||Italy|658[0n]
Polyommatus icarus|OXB435-15|OXB-TGS-159||United Kingdom|658[0n]
Polyommatus icarus|OXB389-15|OXB-TGS-019||United Kingdom|658[0n]
Polyommatus icarus|OXB486-15|OXB-TGS-518||United Kingdom|658[0n]
Polyommatus icarus|OXB437-15|OXB-TGS-161||United Kingdom|658[0n]
Polyommatus icarus|OXB527-15|OXB-TGS-861||United Kingdom|658[0n]
Polyommatus icarus|OXB487-15|OXB-TGS-519||United Kingdom|658[0n]
Polyommatus icarus|OXB529-15|OXB-TGS-875||United Kingdom|658[0n]
Polyommatus icarus|OXB528-15|OXB-TGS-862||United Kingdom|658[0n]
Polyommatus icarus|OXB531-15|OXB-TGS-877||United Kingdom|658[0n]
Polyommatus icarus|OXB558-15|OXB-TGS-933||United Kingdom|658[0n]
Polyommatus icarus|OXB458-15|OXB-TGS-343||United Kingdom|658[0n]
Polyommatus icarus|OXB530-15|OXB-TGS-876||United Kingdom|658[0n]
Polyommatus icarus|OXB543-15|OXB-TGS-918||United Kingdom|658[0n]
Polyommatus icarus|OXB559-15|OXB-TGS-934||United Kingdom|658[0n]
Polyommatus icarus|WMB4026-14|RVcoll.12-Z115||Ireland|658[0n]
Polyommatus icarus|WMB4019-14|RVcoll.12-R452||United Kingdom|658[0n]
Polyommatus icarus|GBGL20068-15|KM459336||Spain|655[0n]
Polyommatus icarus|EZSPM874-12|Rvcoll.130711PX33||Spain|658[0n]
Polyommatus icarus|WMB5151-14|RVcoll.12-P242||France|658[0n]
Polyommatus icarus|WMB4044-14|RVcoll.13-S266||Spain|658[0n]
Polyommatus icarus|WMB3689-14|RVcoll.11-J238||Spain|658[0n]
Polyommatus icarus|WMB3407-14|RVcoll.09-V894||Spain|658[0n]
Polyommatus icarus|WMB6529-18|RVcoll08J116|Genit. examined|Portugal|658[0n]
Polyommatus icarus|EZSPM271-09|RVcoll.08-R488|Genit. examined|Spain|658[0n]
Polyommatus icarus|EULEP103-14|RVcoll.09-V396|Genit. examined|Spain|676[0n]
Polyommatus icarus|EULEP106-14|RVcoll.09-V488|Genit. examined|Spain|676[0n]
Polyommatus icarus|EULEP109-14|RVcoll.09-V905|Genit. examined|Spain|676[0n]
Polyommatus icarus|EULEP113-14|RVcoll.09-X210|Genit. examined|France|676[0n]
Polyommatus icarus|GBMIN32540-13|JN084707||Spain|676[0n]
Polyommatus icarus|WMB3698-14|RVcoll.11-J554||Spain|658[0n]
Polyommatus icarus|WMB3904-14|RVcoll.12-P295||France|658[0n]
Polyommatus icarus|WMB3950-14|RVcoll.12-P711||France|658[0n]
Polyommatus icarus|WMB4468-14|RVcoll.14-D350||Spain|658[0n]
Polyommatus icarus|EULEP027-14|RVcoll.07-D768||Spain|658[0n]
Polyommatus icarus|EULEP028-14|RVcoll.07-D769||Spain|658[0n]
Polyommatus icarus|EULEP029-14|RVcoll.07-D778||Spain|658[0n]
Polyommatus icarus|EULEP238-14|RVcoll.12-L160||Spain|658[0n]
Polyommatus icarus|GBGL20089-15|KM459370||Spain|655[0n]
Polyommatus icarus|GBGL20094-15|KM459377||Spain|655[0n]
Polyommatus icarus|GBGL20142-15|KM459428|Levant|France|655[0n]
Polyommatus icarus|GBGL20079-15|KM459351||France|673[0n]
Polyommatus icarus|WMB5453-14|RVcoll.14-M901||Spain|637[0n]
Polyommatus icarus|WMB3399-14|RVcoll.09-V741||Spain|658[0n]
Polyommatus icarus|EZSPM873-12|Rvcoll.130711PX19||Spain|658[0n]
Polyommatus icarus|EZSPM467-09|Rvcoll.09-V907|Genit. examined|Spain|658[0n]
Polyommatus icarus|EZSPM246-09|RVcoll.08-R458|Genit. examined|Spain|658[0n]
Polyommatus icarus|EZSPM141-09|RVcoll.08-P653|Genit. examined|Spain|658[0n]
Polyommatus icarus|EZSPC285-09|RVcoll.07-C623|Genit. examined|Spain|658[0n]
Polyommatus icarus|EZSPC283-09|RVcoll.08-P076|Genit. examined|Spain|658[0n]
Polyommatus icarus|EZSPN173-09|RVcoll.06-K699||Spain|658[0n]

[illegible]
Polyommatus icarus|EZSPC285-09|RVcoll.07-C623|Genit. examined|Spain|658[0n]
Polyommatus icarus|EZSPC283-09|RVcoll.08-P076|Genit. examined|Spain|658[0n]
Polyommatus icarus|EZSPN173-09|RVcoll.06-K699||Spain|658[0n]
Polyommatus icarus|EZSPN171-09|RVcoll.06-K697|Genit. examined|Spain|658[0n]
Polyommatus icarus|EZSPC666-09|RVcoll.08-L473|Genit. examined|Spain|658[0n]
Polyommatus icarus|WMB106-11|RVcoll.10-B623||France|638[0n]
Polyommatus icarus|HBOK172-08|OK520-78||Spain|658[0n]
Polyommatus icarus|GBLAA056-14|BC ZSM Lep 80717||Germany|658[0n]
Polyommatus icarus|EULEP4945-16|RVcoll16I161||Germany|658[0n]
Polyommatus icarus|EULEP4534-16|RVcoll15I899||Liechtenstein|658[0n]
Polyommatus icarus|OXB820-15|14-U783||France|658[0n]
Polyommatus icarus|OXB822-15|14-U785||France|658[0n]
Polyommatus icarus|OXB1200-15|Rvcoll_15-M616||France|658[0n]
Polyommatus icarus|GBLAA1061-15|BC ZSM Lep 86567||Germany|658[0n]
Polyommatus icarus|BIBSA1259-15|15-F875||France|658[0n]
Polyommatus icarus|BIBSA1250-15|15-F830||France|658[0n]
Polyommatus icarus|EULEP362-14|MM23847||Lithuania|658[0n]
Polyommatus icarus|EULEP4981-16|RVcoll16I578||Poland|658[0n]
Polyommatus icarus|EULEP4960-16|RVcoll16I288||Poland|658[0n]
Polyommatus icarus|WMB5293-14|RVcoll.14-I590||France|658[0n]
Polyommatus icarus|WMB6540-18|RVcoll14B625||Ukraine|658[0n]
Polyommatus icarus|ODOPE242-11|BC ZSM Lep 53113||Germany|658[0n]
Polyommatus icarus|GWORO805-09|BC ZSM Lep 30497||Germany|658[0n]
Polyommatus icarus|ODOPE364-11|BC ZSM Lep 50765||Germany|658[0n]
Polyommatus icarus|EZROM500-08|RV-06-M943|Genit. examined|Romania|650[1n]
Polyommatus icarus|OXB1413-15|Rvcoll_15-M788||France|658[0n]
Polyommatus icarus|OXB1392-15|Rvcoll_15-M757||France|658[0n]
Polyommatus icarus|OXB1367-15|Rvcoll_15-M727||France|658[0n]
Polyommatus icarus|OXB1363-15|Rvcoll_15-M718||France|658[0n]
Polyommatus icarus|OXB1253-15|Rvcoll_15-M107||France|658[0n]
Polyommatus icarus|OXB570-15|OXB-TGS-945||United Kingdom|658[0n]
Polyommatus icarus|OXB569-15|OXB-TGS-944||United Kingdom|658[0n]
Polyommatus icarus|OXB568-15|OXB-TGS-943||United Kingdom|658[0n]
Polyommatus icarus|OXB521-15|OXB-TGS-793||United Kingdom|658[0n]
Polyommatus icarus|OXB516-15|OXB-TGS-769||United Kingdom|658[0n]
Polyommatus icarus|OXB511-15|OXB-TGS-732||United Kingdom|658[0n]
Polyommatus icarus|OXB510-15|OXB-TGS-731||United Kingdom|658[0n]
Polyommatus icarus|OXB504-15|OXB-TGS-675||United Kingdom|658[0n]
Polyommatus icarus|OXB503-15|OXB-TGS-674||United Kingdom|658[0n]
Polyommatus icarus|OXB496-15|OXB-TGS-650||United Kingdom|658[0n]
Polyommatus icarus|OXB480-15|OXB-TGS-468||United Kingdom|658[0n]
Polyommatus icarus|OXB478-15|OXB-TGS-457||United Kingdom|658[0n]
Polyommatus icarus|OXB477-15|OXB-TGS-456||United Kingdom|658[0n]
Polyommatus icarus|OXB268-15|OXB-TGS-951||United Kingdom|658[0n]
Polyommatus icarus|OXB1614-16|OXB-TGS-1332||United Kingdom|658[0n]
Polyommatus icarus|BIBSA916-15|16-A007||France|658[0n]
Polyommatus icarus|EULEP2433-15|RVcoll.14-V231||Belgium|658[0n]
Polyommatus icarus|EULEP4533-16|RVcoll15I714||Austria|658[0n]
Polyommatus icarus|PHLAH454-12|TLMF Lep 08273||Austria|658[0n]
Polyommatus icarus|OXB1216-15|Rvcoll_15-M642||France|658[0n]
Polyommatus icarus|OXB488-15|OXB-TGS-541||United Kingdom|623[0n]
Polyommatus icarus|GBMIN32546-13|JN084695||Denmark|676[0n]
Polyommatus icarus|OXB517-15|OXB-TGS-770||United Kingdom|658[0n]
Polyommatus icarus|OXB497-15|OXB-TGS-651||United Kingdom|658[0n]
Polyommatus icarus|OXB489-15|OXB-TGS-542||United Kingdom|658[0n]
Polyommatus icarus|OXB459-15|OXB-TGS-344||United Kingdom|658[0n]
Polyommatus icarus|EULEP4540-16|RVcoll15Q093||Russia|645[0n]
Polyommatus icarus|OXB522-15|OXB-TGS-794||United Kingdom|658[0n]
Polyommatus icarus|EULEP2023-15|RVcoll.14-I860||Poland|658[0n]
Polyommatus icarus|EULEP4536-16|RVcoll15Q070||Russia|658[0n]
Polyommatus icarus|WMB6538-18|RVcoll11J530|Rhodes|Greece|658[0n]
Polyommatus icarus|WMB6537-18|RVcoll11J519|Karpathos|Greece|658[0n]
Polyommatus icarus|WMB6536-18|RVcoll11J518|Karpathos|Greece|658[0n]
Polyommatus icarus|WMB6535-18|RVcoll11J508|Karpathos|Greece|658[0n]
Polyommatus icarus|WMB6534-18|RVcoll11J497|Nisyros|Greece|658[0n]
Polyommatus icarus|EZROM502-08|RV-07-D104|Genit. examined|Romania|658[0n]
Polyommatus icarus|EULEP4541-16|RVcoll15Q094||Russia|634[0n]
Polyommatus icarus|GBGL20123-15|KM459407|Argentario|Italy|655[2n]
Polyommatus icarus|EZRMN210-08|RVcoll.07-D053|Genit. examined|Romania|658[0n]
Polyommatus icarus|GBMIN32547-13|JN084693||Germany|676[0n]
Polyommatus icarus|LEASS533-17|TLMF Lep 22181||Austria|658[0n]
Polyommatus icarus|LEASS917-17|KLM Lep 08422||Austria|658[0n]
Polyommatus icarus|LEASS536-17|TLMF Lep 22184||Austria|658[0n]
Polyommatus icarus|OXB746-15|13-U411||Italy|658[0n]
Polyommatus icarus|GBGL20136-15|KM459422||Italy|655[0n]
Polyommatus icarus|BIBSA314-15|RVcoll.14-E116||Italy|658[0n]
Polyommatus icarus|BIBSA1137-15|15-L950||Italy|658[0n]
Polyommatus icarus|EULEP5032-16|RVcoll16I980||Slovakia|658[0n]
Polyommatus icarus|EULEP4532-16|RVcoll15I355||Austria|658[0n]
Polyommatus icarus|EULEP4531-16|RVcoll15H602||Switzerland|658[0n]
Polyommatus icarus|EULEP4530-16|RVcoll15G985||Switzerland|658[0n]
Polyommatus icarus|EZROM503-08|RV-07-D439|Genit. examined|Romania|658[0n]
Polyommatus icarus|EZROM593-08|RV-07-C366||Romania|658[0n]
Polyommatus icarus|EZRMN207-08|RVcoll.08-M374|Genit. examined|Romania|658[0n]
Polyommatus icarus|EZRMN206-08|RVcoll.08-M230|Genit. examined|Romania|658[0n]
Polyommatus icarus|GWOSZ099-11|BC ZSM Lep 41855||Italy|658[0n]
Polyommatus icarus|GWOSU026-11|BC ZSM Lep 53277|Identified by barcode|Germany|658[0n]
Polyommatus icarus|ABOLD435-16|TLMF Lep 21587||Austria|658[0n]
Polyommatus icarus|LEATG234-14|TLMF Lep 14021||Austria|658[0n]
Polyommatus icarus|ABOLD070-16|TLMF Lep 21148||Austria|658[0n]
Polyommatus icarus|LEATD021-13|TLMF Lep 12668||Italy|658[0n]
Polyommatus icarus|LEFIE954-10|MM10352||Finland|658[0n]
Polyommatus icarus|GBMIN32570-13|JN084694||Finland|676[0n]
Polyommatus icarus|EULEP1340-15|RVcoll.14-F830||Greece|658[0n]
Polyommatus icarus|EULEP1243-15|RVcoll.14-F591||Greece|658[0n]
Polyommatus icarus|EULEP335-14|MM23820||Estonia|658[0n]
Polyommatus icarus|EULEP4756-16|RVcoll16G983||Sweden|658[0n]
Polyommatus icarus|LON963-12|NHMO Lep2011.023||Norway|658[0n]
Polyommatus icarus|LEFIJ522-10|MM17147||Finland|658[0n]
Polyommatus icarus|PHLAC364-10|TLMF Lep 02399||Italy|624[0n]
Scolitantides orion|EZSPC326-09|RVcoll.08-J359||Spain|658[0n]
Scolitantides orion|EZSPC1188-10|RVcoll.09-T227||Spain|658[0n]
[illegible]

Polyommatus icarus|PHLAC364-10|TLMF Lep 02399||Italy|624[0n]
Scolitantides orion|EZSPC326-09|RVcoll.08-J359||Spain|658[0n]
Scolitantides orion|EZSPC1188-10|RVcoll.09-T227||Spain|658[0n]
Scolitantides orion|EZSPC1043-10|RVcoll.08-J373||Spain|658[0n]
Scolitantides orion|EZSPC1233-10|RVcoll.09-V699||Spain|658[0n]
Scolitantides orion|EZSPN295-09|RVcoll.08-J662||Spain|658[0n]
Scolitantides orion|EZSPC328-09|RVcoll.08-L426||Spain|658[0n]
Scolitantides orion|EZSPC327-09|RVcoll.08-L419||Spain|658[0n]
Scolitantides orion|EZSPC325-09|RVcoll.07-C619||Spain|658[0n]
Scolitantides orion|EZSPC324-09|RVcoll.06-H848||Spain|658[0n]
Scolitantides orion|LEFIL048-10|MM19048||Russia|658[0n]
Scolitantides orion|ABOLD615-17|TLMF Lep 21691||Austria|658[0n]
Scolitantides orion|LEASS909-17|KLM Lep 08414||Austria|658[0n]
Scolitantides orion|EULEP2135-15|RVcoll.14-J979||Switzerland|658[0n]
Scolitantides orion|LON810-11|NHMO Lep09008||Norway|658[0n]
Scolitantides orion|LON809-11|NHMO Lep09007||Norway|658[0n]
Scolitantides orion|LON808-11|NHMO Lep09006||Norway|658[0n]
Scolitantides orion|LEFIJ2526-15|MM23894||Finland|658[0n]
Scolitantides orion|LEFIJ2525-15|MM23893||Finland|658[0n]
Scolitantides orion|LEFIL376-10|MM18686||Finland|658[0n]
Scolitantides orion|FBLMT884-09|BC ZSM Lep 25444||Germany|658[0n]
Scolitantides orion|EZRMN274-08|RVcoll.08-M508||Romania|658[0n]
Scolitantides orion|EZROM687-08|RV-07-D019||Romania|658[0n]
Scolitantides orion|EZROM548-08|RV-07-E346||Romania|658[0n]
Scolitantides orion|EZROM547-08|RV-07-D002||Romania|658[0n]
Scolitantides orion|EZROM698-08|RV-07-F562||Romania|658[0n]
Scolitantides orion|WMB5105-14|RVcoll. 14-L236||Italy|658[0n]
Scolitantides orion|EULEP020-14|RVcoll.07-D018||Romania|658[0n]
Scolitantides orion|EULEP4656-16|RVcoll10A937||Bulgaria|658[0n]
Scolitantides orion|EULEP5023-16|RVcoll16I950||Slovakia|658[0n]
Scolitantides orion|EULEP1680-15|RVcoll.14-G982||Greece|658[0n]
Scolitantides orion|EULEP1704-15|RVcoll.14-H140||Greece|658[0n]
Scolitantides orion|EZRMN273-08|RVcoll.08-M257||Romania|658[1n]
Scolitantides orion|EZRMN272-08|RVcoll.07-D017||Romania|658[0n]
Scolitantides orion|GWOTF687-12|BC ZSM Lep 62393||Germany|658[0n]
Scolitantides orion|LEASS1057-17|TLMF Lep 22610||Austria|658[0n]
Scolitantides orion|LEASS1056-17|TLMF Lep 22609||Austria|658[0n]
Scolitantides orion|ABOLD066-16|TLMF Lep 21144||Austria|658[0n]
Praephilotes anthracias|EULEP4552-16|RVcoll14A952||Russia|658[0n]
Praephilotes anthracias|EULEP4551-16|RVcoll14A951||Russia|658[0n]
Pseudophilotes bavius|EULEP4564-16|RVcoll10B590||Romania|658[0n]
Pseudophilotes bavius|EULEP2065-15|RVcoll.14-J550||Ukraine|658[0n]
Pseudophilotes bavius|EULEP3115-15|RVcoll.14-O248||Macedonia|658[0n]
Pseudophilotes bavius|EULEP3114-15|RVcoll.14-O247||Macedonia|658[0n]
Pseudophilotes bavius|EULEP4566-16|RVcoll15Q003||Macedonia|658[0n]
Pseudophilotes bavius|EULEP4565-16|RVcoll15Q002||Macedonia|658[0n]
Pseudophilotes bavius|EULEP1692-15|RVcoll.14-H039||Greece|658[0n]
Pseudophilotes bavius|EULEP1681-15|RVcoll.14-G983||Greece|658[0n]
Pseudophilotes bavius|EULEP1676-15|RVcoll.14-G949||Greece|658[0n]
Pseudophilotes bavius|EULEP3112-15|RVcoll.14-O245||Greece|658[0n]
Pseudophilotes bavius|EULEP3111-15|RVcoll.14-O244||Greece|658[0n]
Pseudophilotes bavius|EULEP3109-15|RVcoll.14-O242||Greece|658[0n]
Pseudophilotes bavius|EULEP5596-17|RVcoll14O315||Greece|658[0n]
Pseudophilotes bavius|EULEP5595-17|RVcoll14O314||Greece|658[0n]
Pseudophilotes bavius|EULEP577-15|RVcoll.10-A312||Romania|658[0n]
Pseudophilotes bavius|EZROM516-08|RV-07-D117||Romania|658[0n]
Pseudophilotes bavius|EULEP2479-15|RVcoll.14-V336||Ukraine|658[0n]
Pseudophilotes bavius|EULEP2477-15|RVcoll.14-V333||Ukraine|658[0n]
Pseudophilotes bavius|EULEP2066-15|RVcoll.14-J551||Ukraine|658[0n]
Pseudophilotes bavius|EULEP4568-16|RVcoll14A948||Russia|658[0n]
Pseudophilotes bavius|EULEP4567-16|RVcoll14A947||Russia|658[0n]
Pseudophilotes bavius|EZROM515-08|RV-06-K583|Genit. examined|Romania|658[0n]
Pseudophilotes bavius|EZRMN222-08|RVcoll.06-K589|Genit. examined|Romania|658[0n]
Pseudophilotes bavius|EZRMN221-08|RVcoll.06-K587|Genit. examined|Romania|658[0n]
Pseudophilotes bavius|EZRMN220-08|RVcoll.06-K586|Genit. examined|Romania|658[0n]
Pseudophilotes bavius|EZROM565-08|RV-06-K588|Genit. examined|Romania|658[0n]
Pseudophilotes bavius|EZRMN219-08|RVcoll.06-K585|Genit. examined|Romania|658[0n]
Pseudophilotes bavius|EZRMN218-08|RVcoll.06-K584|Genit. examined|Romania|658[0n]
Pseudophilotes barbagiae|WMB317-11|RVcoll.11-E511||Italy|658[0n]
Pseudophilotes barbagiae|EULEP611-15|RVcoll.11-E514|Sardinia|Italy|658[0n]
Pseudophilotes barbagiae|EULEP612-15|RVcoll.11-E515|Sardinia|Italy|658[0n]
Pseudophilotes barbagiae|WMB1159-13|RVcoll.11-E513|Sardinia|Italy|617[0n]
Pseudophilotes barbagiae|WMB1158-13|RVcoll.11-E512|Sardinia|Italy|658[0n]
Pseudophilotes abencerragus|EZSPM760-12|Rvcoll. 12-L555||Portugal|628[0n]
Pseudophilotes abencerragus|EZSPC731-10|RVcoll.08-H667.1||Spain|658[0n]
Pseudophilotes abencerragus|EZSPM759-12|Rvcoll. 12-L554||Portugal|658[0n]
Pseudophilotes abencerragus|EZSPM758-12|Rvcoll. 12-L553||Portugal|621[0n]
Pseudophilotes abencerragus|EZSPN386-09|RVcoll.08-H437||Spain|647[0n]
Pseudophilotes abencerragus|WMB5508-14|RVcoll.14-N310||Spain|658[0n]
Pseudophilotes abencerragus|EZSPC714-10|RVcoll.08-H315||Spain|658[0n]
Pseudophilotes abencerragus|EZSPC720-10|RVcoll.08-H435||Spain|658[0n]
Pseudophilotes abencerragus|EZSPN388-09|RVcoll.08-H439||Spain|658[0n]
Pseudophilotes abencerragus|EULEP5588-17|RVcoll14N312||Spain|658[0n]
Pseudophilotes abencerragus|EULEP5587-17|RVcoll14N311||Spain|658[0n]
Pseudophilotes abencerragus|EZSPC721-10|RVcoll.08-H438.1||Spain|658[0n]
Pseudophilotes abencerragus|EZSPC715-10|RVcoll.08-H316||Spain|658[0n]
Pseudophilotes abencerragus|EZSPN385-09|RVcoll.08-H436||Spain|658[0n]
Pseudophilotes panoptes|WMB4580-14|RVcoll.150308GV90||Spain|658[0n]
Pseudophilotes panoptes|EZSPM285-09|SMcoll.150308GV24||Spain|658[0n]
Pseudophilotes panoptes|EZSPM283-09|SMcoll.150308PP69||Spain|658[0n]
Pseudophilotes panoptes|EZSPN410-09|RVcoll.08-H513||Spain|658[0n]
Pseudophilotes panoptes|EZSPN683-09|RVcoll.08-L007||Spain|658[0n]
Pseudophilotes panoptes|EZSPC689-10|RVcoll.06-A007||Spain|658[0n]
Pseudophilotes panoptes|EZSPN685-09|RVcoll.08-L013||Spain|638[0n]
Pseudophilotes panoptes|EZSPN611-09|RVcoll.08-J335||Spain|658[0n]
Pseudophilotes panoptes|EZSPN1042-11|Rvcoll.08-J369||Spain|658[0n]
Pseudophilotes panoptes|EZSPN1097-11|Rvcoll.10-A206||Spain|658[0n]
Pseudophilotes panoptes|WMB3368-14|RVcoll.09-T238|Genit. examined|Spain|658[0n]
Pseudophilotes panoptes|EZSPN156-09|RVcoll.06-H852|Genit. examined|Spain|640[0n]
Pseudophilotes panoptes|EZSPN140-09|RVcoll.06-G504||Spain|658[0n]
Pseudophilotes panoptes|EZSPC738-10|RVcoll.08-J209||Spain|658[0n]
Pseudophilotes panoptes|EZSPM616-12|RVcoll.11-D837||Spain|658[0n]
Pseudophilotes panoptes|EZSPM612-12|RVcoll.11-D802||Spain|658[0n]

Pseudophilotes panoptes|EZSPC738-10|RVcoll.08-J209||Spain|658[0n]
Pseudophilotes panoptes|EZSPM616-12|RVcoll.11-D837||Spain|658[0n]
Pseudophilotes panoptes|EZSPM612-12|RVcoll.11-D802||Spain|658[0n]
Pseudophilotes panoptes|WMB3218-14|RVcoll.08-J718||Spain|658[0n]
Pseudophilotes panoptes|EZSPC687-10|RVcoll.06-A015||Spain|634[0n]
Pseudophilotes panoptes|EZSPN1029-11|Rvcoll.06-A009.1||Spain|658[0n]
Pseudophilotes panoptes|EZSPN828-09|RVcoll.08-L643||Spain|658[0n]
Pseudophilotes vicrama|EULEP4570-16|RVcoll14O559||Estonia|658[0n]
Pseudophilotes vicrama|EULEP4828-16|RVcoll16H726||Ukraine|658[0n]
Pseudophilotes baton|WMB3420-14|RVcoll.09-X239||France|658[0n]
Pseudophilotes vicrama|EZRMN226-08|RVcoll.08-M369||Romania|646[0n]
Pseudophilotes vicrama|EULEP715-15|RVcoll.12-M254|Crete|Greece|658[0n]
Pseudophilotes vicrama|EULEP709-15|RVcoll.12-M213|Crete|Greece|658[0n]
Pseudophilotes vicrama|LEFIJ2194-14|MM23534||Finland|658[0n]
Pseudophilotes vicrama|LEFIJ2193-14|MM23533||Finland|658[0n]
Pseudophilotes vicrama|EULEP1742-15|RVcoll.14-H347||Greece|658[0n]
Pseudophilotes vicrama|EULEP1562-15|RVcoll.14-G562||Macedonia|658[0n]
Pseudophilotes vicrama|EULEP1406-15|RVcoll.14-G071||Greece|658[0n]
Pseudophilotes vicrama|EULEP1371-15|RVcoll.14-F964||Greece|658[0n]
Pseudophilotes vicrama|EULEP875-15|RVcoll.14-C397||Greece|658[0n]
Pseudophilotes vicrama|EULEP1287-15|RVcoll.14-F705||Greece|658[0n]
Pseudophilotes vicrama|EULEP4569-16|RVcoll10A982||Bulgaria|658[0n]
Pseudophilotes baton|WMB4779-14|RVcoll. 14-I326||Italy|658[0n]
Pseudophilotes baton|WMB352-11|RVcoll.11-E789||France|658[0n]
Pseudophilotes baton|WMB2637-13|RVcoll.11-E828|Corsica|France|658[0n]
Pseudophilotes baton|WMB1518-13|RVcoll.12-O144|Corsica|France|658[0n]
Pseudophilotes baton|WMB1177-13|RVcoll.11-E844|Corsica|France|658[0n]
Pseudophilotes vicrama|EZROM521-08|RV-07-C101||Romania|658[0n]
Pseudophilotes vicrama|EZROM520-08|RV-07-D202||Romania|658[0n]
Pseudophilotes vicrama|EZROM519-08|RV-07-D112||Romania|658[0n]
Pseudophilotes vicrama|EZROM518-08|RV-07-D054||Romania|658[0n]
Pseudophilotes vicrama|EZROM517-08|RV-06-K590||Romania|658[0n]
Pseudophilotes vicrama|EZRMN225-08|RVcoll.08-M258||Romania|658[0n]
Pseudophilotes vicrama|EZRMN224-08|RVcoll.07-D187||Romania|658[0n]
Pseudophilotes vicrama|EZRMN223-08|RVcoll.06-V652||Romania|658[0n]
Pseudophilotes baton|EZSPN232-09|RVcoll.07-W059||France|658[0n]
Pseudophilotes baton|EZSPN231-09|RVcoll.07-W055||France|658[0n]
Pseudophilotes baton|WMB1692-13|RVcoll.12-P562||France|658[0n]
Pseudophilotes baton|WMB3455-14|RVcoll.10-A553||France|658[0n]
Pseudophilotes baton|WMB271-11|RVcoll.10-A486||France|658[0n]
Pseudophilotes baton|LENOA1461-11|LN-BD1455||France|658[0n]
Pseudophilotes panoptes|WMB4547-14|RVcoll.040611HZ48||Spain|658[0n]
Pseudophilotes panoptes|WMB3585-14|RVcoll.11-E070||Spain|658[0n]
Pseudophilotes panoptes|WMB3582-14|RVcoll.11-E061||Spain|658[0n]
Pseudophilotes panoptes|EZSPN654-09|RVcoll.08-J798||Spain|658[0n]
Pseudophilotes baton|BIBSA1161-15|15-L134||Italy|658[0n]
Pseudophilotes baton|EULEP3118-15|RVcoll.14-O251||Italy|658[0n]
Pseudophilotes baton|EULEP3117-15|RVcoll.14-O250||Italy|658[0n]
Pseudophilotes baton|BIBSA732-15|LD-3105||Italy|658[0n]
Pseudophilotes baton|BIBSA1013-15|15-C135||Italy|658[0n]
Pseudophilotes baton|BIBSA1756-16|14-A094||Italy|658[0n]
Pseudophilotes baton|BIBSA612-15|LEP-SS-00232||Italy|658[0n]
Pseudophilotes baton|BIBSA480-15|LEP-SS-00100||Italy|658[0n]
Pseudophilotes baton|BIBSA051-14|LEP-SS-00051||Italy|658[0n]
Pseudophilotes baton|OXB1130-15|15-A663||Italy|658[0n]
Pseudophilotes baton|OXB916-15|15-A560||Italy|658[0n]
Pseudophilotes baton|OXB949-15|15-A593||Italy|658[0n]
Pseudophilotes baton|WMB805-13|RVcoll.07-E056||Italy|641[0n]
Pseudophilotes baton|EULEP5879-18|RVcoll15O516||Spain|609[0n]
Pseudophilotes baton|OXB882-15|15-A524||Italy|630[0n]
Pseudophilotes baton|WMB1297-13|RVcoll.12-M586|Sicily|Italy|650[0n]
Pseudophilotes baton|ABOLD480-16|TLMF Lep 21463||Austria|621[0n]
Pseudophilotes vicrama|ABOLD478-16|TLMF Lep 21461|Genit. examined|Austria|634[0n]
Pseudophilotes baton|EULEP2156-15|RVcoll.14-K067||Switzerland|658[0n]
Pseudophilotes baton|EULEP240-14|RVcoll.12-M018||Spain|658[0n]
Pseudophilotes baton|EULEP4563-16|RVcoll15J169||Switzerland|658[0n]
Pseudophilotes baton|EULEP4562-16|RVcoll15G670||Switzerland|658[0n]
Pseudophilotes baton|WMB4937-14|RVcoll.14-I484||Italy|658[0n]
Pseudophilotes baton|WMB4856-14|RVcoll.14-I403||Italy|658[0n]
Pseudophilotes baton|WMB4785-14|RVcoll. 14-I332||Italy|658[0n]
Pseudophilotes baton|WMB567-11|RVcoll.11-H787|Vulcano|Italy|658[0n]
Pseudophilotes baton|WMB6587-18|RVcoll14A102||Italy|658[0n]
Pseudophilotes baton|WMB3977-14|RVcoll.12-Q211||France|658[0n]
Pseudophilotes baton|WMB4240-14|RVcoll.13-T844||Italy|658[0n]
Pseudophilotes baton|WMB4130-14|RVcoll.13-S616||Italy|658[0n]
Pseudophilotes baton|WMB2380-13|RVcoll.07-E057||Italy|658[0n]
Pseudophilotes baton|WMB2036-13|RVcoll.12-Q747||Italy|658[0n]
Pseudophilotes baton|WMB1924-13|RVcoll.11-H788|Vulcano|Italy|658[0n]
Pseudophilotes baton|WMB1798-13|RVcoll.12-Q317||France|658[0n]
Pseudophilotes baton|WMB799-13|RVcoll.07-E010||Italy|658[0n]
Pseudophilotes baton|WMB789-13|RVcoll.07-D839||Italy|658[0n]
Pseudophilotes baton|WMB2809-13|RVcoll.12-M420|Sicily|Italy|658[0n]
Pseudophilotes baton|WMB3457-14|RVcoll.10-A575||France|658[0n]
Pseudophilotes baton|WMB148-11|RVcoll.11-D210|Sicily|Italy|658[0n]
Pseudophilotes baton|EZSPC881-10|RVcoll.09-T142||Spain|658[0n]
Pseudophilotes baton|EZSPC762-10|RVcoll.08-L431.1||Spain|658[0n]
Pseudophilotes panoptes|EZSPM320-09|RVcoll.08-J888||Spain|658[0n]
Pseudophilotes panoptes|EZSPN524-09|RVcoll.08-H994||Spain|658[0n]
Pseudophilotes baton|EZSPM570-10|RVcoll.09-X022||Spain|658[0n]
Pseudophilotes baton|EZSPM569-10|RVcoll.09-X003||Spain|658[0n]
Pseudophilotes baton|EZSPN800-09|RVcoll.08-L417||Spain|658[0n]
Pseudophilotes baton|EZSPN801-09|RVcoll.08-L430||Spain|658[0n]
Pseudophilotes baton|GWORZ050-10|BC ZSM Lep 30406||Italy|658[0n]
Pseudophilotes baton|GWOTF686-12|BC ZSM Lep 62392||Germany|658[0n]
Pseudophilotes baton|GWOTF685-12|BC ZSM Lep 62391||Germany|658[0n]
Pseudophilotes baton|LEATD300-13|TLMF Lep 12947||Italy|658[0n]
Pseudophilotes baton|ABOLD481-16|TLMF Lep 21464||Austria|658[0n]
Iolana debilitata|EULEP067-14|RVcoll.08-J706||Spain|658[0n]
Iolana debilitata|EULEP066-14|RVcoll.08-J704||Spain|658[0n]
Iolana debilitata|EULEP065-14|RVcoll.08-J701||Spain|658[0n]
Iolana debilitata|EULEP064-14|RVcoll.08-J700||Spain|658[0n]
Iolana debilitata|EULEP037-14|RVcoll.07-W202||Spain|658[0n]
[illegible]

Iolana debilitata|EULEP065-14|RVcoll.08-J701||Spain|658[0n]
Iolana debilitata|EULEP064-14|RVcoll.08-J700||Spain|658[0n]
Iolana debilitata|EULEP037-14|RVcoll.07-W202||Spain|658[0n]
Iolana debilitata|EULEP036-14|RVcoll.07-W087||Spain|658[0n]
Iolana debilitata|EULEP068-14|RVcoll.08-J783||Spain|658[0n]
Iolana debilitata|EULEP074-14|RVcoll.08-L349||Spain|658[0n]
Iolana debilitata|EULEP073-14|RVcoll.08-L348||Spain|658[0n]
Iolana debilitata|EULEP039-14|RVcoll.07-Z063||Spain|658[0n]
Iolana debilitata|EULEP038-14|RVcoll.07-Z062||Spain|658[0n]
Iolana debilitata|EULEP010-14|RVcoll.06-G520||Spain|658[0n]
Iolana debilitata|EULEP009-14|RVcoll.06-G519||Spain|658[0n]
Iolana debilitata|EULEP008-14|RVcoll.06-G518||Spain|658[0n]
Iolana debilitata|EULEP007-14|RVcoll.06-G517||Spain|658[0n]
Iolana iolas|BCLEP117-17|LEP-SS-00568||Italy|658[0n]
Iolana iolas|EZSPC941-10|RVcoll.09-V968||France|658[0n]
Iolana iolas|EULEP5297-17|RVcoll08M159||Switzerland|676[0n]
Iolana iolas|EULEP5377-17|RVcoll10A413||France|676[0n]
Iolana iolas|EULEP5378-17|RVcoll10A414||France|676[0n]
Iolana iolas|EULEP5379-17|RVcoll10A415||France|650[0n]
Iolana iolas|EULEP5230-17|RVcoll07C807||Switzerland|649[0n]
Iolana iolas|EULEP5229-17|RVcoll07C806||Switzerland|676[0n]
Iolana iolas|EULEP5228-17|RVcoll07C805||Switzerland|676[0n]
Iolana iolas|EULEP5163-17|RVcoll16C447||Italy|658[0n]
Iolana iolas|BIBSA862-15|LD-3474||Italy|658[0n]
Iolana iolas|BIBSA1006-15|15-C105||Italy|658[0n]
Iolana iolas|EULEP5292-17|RVcoll08J665||Italy|676[0n]
Iolana iolas|EULEP5578-17|RVcoll14H095||Greece|655[0n]
Iolana iolas|EULEP5580-17|RVcoll14H805||Greece|655[0n]
Iolana iolas|EULEP5584-17|RVcoll14H998||Romania|655[0n]
Iolana iolas|EULEP5585-17|RVcoll14H999||Romania|655[0n]
Iolana iolas|EULEP5592-17|RVcoll14N517||Macedonia|655[0n]
Iolana iolas|EULEP5632-17|RVcoll15C958||Romania|658[0n]
Iolana iolas|EULEP5291-17|RVcoll08J648||Bulgaria|676[0n]
Iolana iolas|EULEP5576-17|RVcoll14H053||Greece|655[0n]
Iolana iolas|EULEP5572-17|RVcoll14G370||Greece|655[0n]
Iolana iolas|EULEP5219-17|RVcoll06H647||Greece|658[2n]
Iolana iolas|LEATG482-14|TLMF Lep 14269||Italy|658[0n]
Glaucopsyche melanops|EZSPN1035-11|Rvcoll.06-G516||Spain|658[0n]
Glaucopsyche melanops|WMB3187-14|RVcoll.08-J014||Spain|658[0n]
Glaucopsyche melanops|EZSPN450-09|RVcoll.08-H645||Spain|658[0n]
Glaucopsyche melanops|EZSPN374-09|RVcoll.08-H400||Spain|658[0n]
Glaucopsyche melanops|OXB948-15|15-A592||Italy|658[0n]
Glaucopsyche melanops|WMB941-13|RVcoll.10-A620||France|658[0n]
Glaucopsyche melanops|WMB934-13|RVcoll.10-A590||France|658[0n]
Glaucopsyche melanops|WMB3536-14|RVcoll.10-C358||Spain|658[0n]
Glaucopsyche melanops|WMB3466-14|RVcoll.10-A621||France|658[0n]
Glaucopsyche melanops|WMB3319-14|RVcoll.08-P701||Spain|658[0n]
Glaucopsyche melanops|WMB3174-14|RVcoll.08-H566||Spain|658[0n]
Glaucopsyche melanops|WMB3125-14|RVcoll.06-G505||Spain|658[0n]
Glaucopsyche melanops|EZSPM253-09|RVcoll.08-R465||Spain|658[0n]
Glaucopsyche melanops|EZSPM251-09|RVcoll.08-R463||Spain|658[0n]
Glaucopsyche melanops|EZSPN1043-11|Rvcoll.08-J399||Spain|658[0n]
Glaucopsyche melanops|EZSPN1032-11|Rvcoll.06-G443||Spain|658[0n]
Glaucopsyche melanops|EZSPM694-12|Rvcoll. 12-L546||Portugal|658[0n]
Glaucopsyche melanops|EZSPN845-09|RVcoll.08-L682||Spain|658[0n]
Glaucopsyche melanops|EZSPN571-09|RVcoll.08-J118||Portugal|658[0n]
Glaucopsyche melanops|EZSPN457-09|RVcoll.08-H663||Spain|658[0n]
Glaucopsyche melanops|EZSPN372-09|RVcoll.08-H397||Spain|658[0n]
Glaucopsyche melanops|WMB3557-14|RVcoll.11-D902||Spain|658[0n]
Glaucopsyche melanops|EZSPN691-09|RVcoll.08-L022||Spain|658[0n]
Glaucopsyche melanops|EZSPN094-09|RVcoll.06-A024||Spain|658[0n]
Glaucopsyche melanops|EZSPN086-09|RVcoll.06-A005||Spain|658[0n]
Glaucopsyche paphos|EULEP4045-16|RVcoll16H950||Cyprus|658[0n]
Glaucopsyche paphos|EULEP4044-16|RVcoll16H949||Cyprus|658[0n]
Glaucopsyche paphos|EULEP4043-16|RVcoll14A923||Cyprus|658[0n]
Glaucopsyche alexis|BIBSA1809-17|RVcoll14I278||Italy|658[0n]
Glaucopsyche alexis|WMB3825-14|RVcoll.09-X828||Italy|658[0n]
Glaucopsyche alexis|EZROM1003-08|RVcoll.07-D001|Genit. examined|Romania|658[0n]
Glaucopsyche alexis|LYCAE258-15|SS121911||Serbia|676[0n]
Glaucopsyche alexis|LYCAE259-15|ZTM008||Serbia|676[0n]
Glaucopsyche alexis|LYCAE260-15|ZTM009||Serbia|676[0n]
Glaucopsyche alexis|LYCAE261-15|ZTM010||Serbia|676[0n]
Glaucopsyche alexis|LYCAE262-15|ZTM011||Serbia|676[0n]
Glaucopsyche alexis|LYCAE263-15|ZTM012||Serbia|676[0n]
Glaucopsyche alexis|EULEP1665-15|RVcoll.14-G884||Greece|658[0n]
Glaucopsyche alexis|EZSPC1035-10|RVcoll.08-J210||Spain|658[0n]
Glaucopsyche alexis|EULEP1815-15|RVcoll.14-H810||Greece|658[0n]
Glaucopsyche alexis|EULEP1799-15|RVcoll.14-H686||Greece|658[0n]
Glaucopsyche alexis|EULEP1794-15|RVcoll.14-H666||Greece|658[0n]
Glaucopsyche alexis|EZSPM248-09|RVcoll.08-R460||Spain|628[0n]
Glaucopsyche alexis|WMB950-13|RVcoll.10-A673||France|658[0n]
Glaucopsyche alexis|EZSPM268-09|RVcoll.08-R485||Spain|658[0n]
Glaucopsyche alexis|EZSPC418-09|RVcoll.08-J372||Spain|658[0n]
Glaucopsyche alexis|WMB3234-14|RVcoll.08-J887||Spain|658[0n]
Glaucopsyche alexis|WMB3366-14|RVcoll.09-T204||Spain|658[0n]
Glaucopsyche alexis|EZSPC1063-10|RVcoll.08-L112||Spain|658[0n]
Glaucopsyche alexis|EZROM721-08|RV-06-G434||Spain|658[0n]
Glaucopsyche alexis|EZSPN712-09|RVcoll.08-L066||Spain|626[0n]
Glaucopsyche alexis|EZSPC1045-10|RVcoll.08-J661||Spain|658[0n]
Glaucopsyche alexis|EZSPN854-09|RVcoll.08-L704||Spain|658[0n]
Glaucopsyche alexis|EZSPN628-09|RVcoll.08-J721||Spain|658[0n]
Glaucopsyche alexis|EZSPN424-09|RVcoll.08-H587||Spain|658[0n]
Glaucopsyche alexis|EZSPC420-09|RVcoll.08-L434||Spain|658[0n]
Glaucopsyche alexis|EZSPC419-09|RVcoll.08-L410||Spain|658[0n]
Glaucopsyche alexis|EZSPC417-09|RVcoll.08-J371||Spain|658[0n]
Glaucopsyche alexis|FBLMT890-09|BC ZSM Lep 25450||Germany|658[0n]
Glaucopsyche alexis|EZROM1001-08|RVcoll.06-K577||Romania|658[0n]
Glaucopsyche alexis|LON957-12|NHMO Lep2011.017||Norway|654[0n]
Glaucopsyche alexis|LYCAE334-15|NK00P598||Russia|649[0n]
Glaucopsyche alexis|LYCAE332-15|AD00P209||Russia|676[0n]
Glaucopsyche alexis|LYCAE335-15|NK00P599||Russia|676[0n]
Glaucopsyche alexis|LYCAE268-15|SS12579||Sweden|676[0n]
Glaucopsyche alexis|LYCAE267-15|SS12573||Sweden|676[0n]

Glaucopsyche alexis|LYCAE335-15|NK00P599||Russia|676[0n]
Glaucopsyche alexis|LYCAE268-15|SS12579||Sweden|676[0n]
Glaucopsyche alexis|LYCAE267-15|SS12573||Sweden|676[0n]
Glaucopsyche alexis|LYCAE266-15|SS12571||Sweden|676[0n]
Glaucopsyche alexis|LYCAE265-15|SS12568||Sweden|676[0n]
Glaucopsyche alexis|LYCAE250-15|SS12687||Sweden|676[0n]
Glaucopsyche alexis|LYCAE249-15|SS12319||Sweden|676[0n]
Glaucopsyche alexis|LYCAE248-15|SS12317||Sweden|676[0n]
Glaucopsyche alexis|LYCAE247-15|SS12316||Sweden|676[0n]
Glaucopsyche alexis|LYCAE246-15|SS12314||Sweden|676[0n]
Glaucopsyche alexis|LYCAE245-15|SS12313||Sweden|676[0n]
Glaucopsyche alexis|LYCAE243-15|NK00P616||Russia|676[0n]
Glaucopsyche alexis|LYCAE331-15|AD00P210||Russia|676[0n]
Glaucopsyche alexis|LYCAE330-15|AD00P208||Russia|676[0n]
Glaucopsyche alexis|LYCAE312-15|SS12397||Sweden|676[0n]
Glaucopsyche alexis|LYCAE311-15|SS12396||Sweden|676[0n]
Glaucopsyche alexis|LYCAE310-15|SS12394||Sweden|676[0n]
Glaucopsyche alexis|LYCAE309-15|SS12393||Sweden|676[0n]
Glaucopsyche alexis|LYCAE308-15|SS12392||Sweden|676[0n]
Glaucopsyche alexis|LYCAE307-15|SS12390||Sweden|676[0n]
Glaucopsyche alexis|LYCAE306-15|SS12386||Sweden|676[0n]
Glaucopsyche alexis|LYCAE305-15|SS12385||Sweden|676[0n]
Glaucopsyche alexis|LYCAE269-15|SS12585||Sweden|676[0n]
Glaucopsyche alexis|LYCAE270-15|SS12591||Sweden|676[0n]
Glaucopsyche alexis|LYCAE271-15|SS12599||Sweden|676[0n]
Glaucopsyche alexis|LYCAE282-15|SS12410||Sweden|676[0n]
Glaucopsyche alexis|LYCAE283-15|SS12411||Sweden|676[0n]
Glaucopsyche alexis|LYCAE284-15|SS12413||Sweden|676[0n]
Glaucopsyche alexis|LYCAE285-15|SS12414||Sweden|676[0n]
Glaucopsyche alexis|LYCAE286-15|SS12417||Sweden|676[0n]
Glaucopsyche alexis|LYCAE287-15|SS12420||Sweden|676[0n]
Glaucopsyche alexis|LYCAE288-15|SS12429||Sweden|676[0n]
Glaucopsyche alexis|LYCAE289-15|SS12434||Sweden|676[0n]
Glaucopsyche alexis|LYCAE244-15|NK00P615||Russia|676[0n]
Glaucopsyche alexis|LEASS916-17|KLM Lep 08421||Austria|658[0n]
Glaucopsyche alexis|LEATJ1182-16|TLMF Lep 19505||Czech Republic|658[0n]
Glaucopsyche alexis|GBLAB129-13|BC ZSM Lep 75755||France|658[0n]
Glaucopsyche alexis|GBLAA1443-15|BC ZSM Lep 87234||Germany|658[0n]
Glaucopsyche alexis|EULEP2349-15|RVcoll.14-V037||Ukraine|658[0n]
Glaucopsyche alexis|EULEP2229-15|RVcoll.14-N408||Ukraine|658[0n]
Glaucopsyche alexis|EULEP4744-16|RVcoll16G687||Sweden|658[0n]
Glaucopsyche alexis|EZROM1007-08|RVcoll.08-M518||Romania|658[0n]
Glaucopsyche alexis|EZROM1005-08|RVcoll.08-M375||Romania|658[0n]
Glaucopsyche alexis|EZROM201-08|RV-07-D228||Romania|658[0n]
Glaucopsyche alexis|EZROM200-08|RV-07-D225||Romania|658[0n]
Glaucopsyche alexis|EZROM199-08|RV-07-D030||Romania|658[0n]
Glaucopsyche alexis|EZROM198-08|RV-06-K595||Romania|658[0n]
Glaucopsyche alexis|EZROM197-08|RV-06-K592||Romania|658[0n]
Glaucopsyche alexis|EZROM1006-08|RVcoll.08-M405||Romania|658[0n]
Glaucopsyche alexis|EZROM1004-08|RVcoll.08-M219||Romania|658[0n]
Glaucopsyche alexis|EZROM1002-08|RVcoll.06-K631||Romania|658[0n]
Glaucopsyche alexis|LEFIC520-10|MM04237||Finland|658[0n]
Glaucopsyche alexis|LEFIJ798-10|MM17423||Finland|658[0n]
Glaucopsyche alexis|LEFIC345-10|MM03860||Finland|658[0n]
Glaucopsyche alexis|LEFIJ513-10|MM17138||Finland|658[0n]
Glaucopsyche alexis|ABOLD416-16|TLMF Lep 21568||Austria|658[0n]
Glaucopsyche alexis|ABOLD082-16|TLMF Lep 21160||Austria|658[0n]
Glaucopsyche alexis|PHLAW021-13|TLMF Lep 09818||Austria|658[0n]
Glaucopsyche alexis|BIBSA047-14|LEP-SS-00046||Italy|631[0n]
Glaucopsyche alexis|BIBSA1798-16|16-A552||Italy|658[0n]
Glaucopsyche alexis|OXB1056-15|15-A601||Italy|658[0n]
Glaucopsyche alexis|EULEP2145-15|RVcoll.14-K021||Switzerland|658[0n]
Glaucopsyche alexis|WMB5331-14|RVcoll.14-J187|Corsica|France|658[0n]
Glaucopsyche alexis|LEATG424-14|TLMF Lep 14211||Italy|658[0n]
Glaucopsyche alexis|LEATG423-14|TLMF Lep 14210||Italy|658[0n]
Glaucopsyche alexis|GWORZ051-10|BC ZSM Lep 30407||Italy|658[0n]
Glaucopsyche alexis|WMB155-11|RVcoll.11-D304|Sicily|Italy|658[0n]
Glaucopsyche alexis|WMB187-11|RVcoll.11-D494||Italy|658[0n]
Glaucopsyche alexis|WMB277-11|RVcoll.10-A543||France|658[0n]
Glaucopsyche alexis|WMB2886-14|RVcoll.11-D058|Sicily|Italy|658[0n]
Glaucopsyche alexis|WMB3423-14|RVcoll.09-X255||France|658[0n]
Glaucopsyche alexis|WMB3059-14|RVcoll.13-T747|Elba|Italy|658[0n]
Glaucopsyche alexis|WMB1123-13|RVcoll.11-D249|Sicily|Italy|658[0n]
Glaucopsyche alexis|WMB945-13|RVcoll.10-A640||France|658[0n]
Glaucopsyche alexis|WMB2209-13|RVcoll.11-Y020|Elba|Italy|658[0n]
Glaucopsyche alexis|WMB2210-13|RVcoll.11-Y021|Elba|Italy|658[0n]
Glaucopsyche alexis|WMB2230-13|RVcoll.11-Y070||Italy|658[0n]
Glaucopsyche alexis|WMB2339-13|RVcoll.12-M281||Italy|658[0n]
Glaucopsyche alexis|WMB2340-13|RVcoll.12-M282||Italy|658[0n]
Glaucopsyche alexis|WMB3820-14|RVcoll.09-T540||Italy|658[0n]
Glaucopsyche alexis|WMB2445-13|RVcoll.11-D389|Sicily|Italy|658[0n]
Glaucopsyche alexis|WMB4124-14|RVcoll.13-S595||Italy|658[0n]
Glaucopsyche alexis|WMB4666-14|RVcoll.LD-2616|Ischia|Italy|658[0n]
Glaucopsyche alexis|WMB4667-14|RVcoll.LD-2617|Ischia|Italy|658[0n]
Glaucopsyche alexis|WMB4712-14|RVcoll.LD-2936||Italy|658[0n]
Glaucopsyche alexis|WMB346-11|RVcoll.11-E736||France|658[0n]
Glaucopsyche alexis|WMB615-11|RVcoll.11-I032|Sicily|Italy|658[0n]
Glaucopsyche alexis|WMB4778-14|RVcoll. 14-I325||Italy|658[0n]
Glaucopsyche alexis|WMB4809-14|RVcoll. 14-I356||Italy|658[0n]
Glaucopsyche alexis|WMB5326-14|RVcoll.14-J033|Corsica|France|658[0n]
Glaucopsyche alexis|WMB5206-14|RVcoll.13-T939||Italy|658[0n]
Glaucopsyche alexis|BIBSA1588-16|12-M569||Italy|658[0n]
Glaucopsyche alexis|BIBSA573-15|LEP-SS-00193||Italy|658[0n]
Glaucopsyche alexis|BIBSA574-15|LEP-SS-00194||Italy|658[0n]
Glaucopsyche alexis|BIBSA1544-16|11-D336||Italy|658[0n]
Glaucopsyche alexis|BIBSA1003-15|15-C091||Italy|658[0n]
Glaucopsyche alexis|BIBSA1649-16|15-C633||Italy|658[0n]
Glaucopsyche alexis|BIBSA1052-15|12-R414|Sicily|Italy|658[0n]
Glaucopsyche alexis|BIBSA048-14|LEP-SS-00047||Italy|658[0n]
Glaucopsyche alexis|OXB869-15|15-A536||Italy|658[0n]
Glaucopsyche alexis|OXB1129-15|15-A662||Italy|658[0n]
Glaucopsyche alexis|OXB1026-15|15-A976||Italy|658[0n]
Glaucopsyche alexis|OXB1095-15|15-A628||Italy|658[0n]

[illegible]
Glaucopsyche alexis|OXB1129-15|15-A662||Italy|658[0n]
Glaucopsyche alexis|OXB1026-15|15-A976||Italy|658[0n]
Glaucopsyche alexis|OXB1095-15|15-A628||Italy|658[0n]
Glaucopsyche alexis|WMB2234-13|RVcoll.11-Y092||Italy|609[0n]
Glaucopsyche alexis|LEATH763-14|TLMF Lep 15975||Italy|632[0n]
Glaucopsyche alexis|LEATH764-14|TLMF Lep 15976||Italy|658[0n]
Turanana taygetica|EULEP1429-15|RVcoll.14-G109||Greece|658[0n]
Turanana taygetica|EULEP1419-15|RVcoll.14-G090||Greece|658[0n]
Turanana taygetica|EULEP1414-15|RVcoll.14-G084||Greece|658[0n]
Turanana taygetica|EULEP1331-15|RVcoll.14-F812||Greece|658[0n]
Turanana taygetica|EULEP1326-15|RVcoll.14-F800||Greece|658[0n]
Turanana taygetica|EULEP1325-15|RVcoll.14-F799||Greece|658[0n]
Phengaris nausithous|EULEP4404-16|RVcoll14A987||Bulgaria|658[0n]
Phengaris nausithous|EULEP4403-16|RVcoll14A986||Bulgaria|658[0n]
Phengaris nausithous|EZRMN085-08|RVcoll.07-C165||Romania|658[0n]
Phengaris nausithous|EZROM384-08|RV-06-M889||Romania|657[0n]
Phengaris nausithous|EZROM1035-09|RVcoll.06-M890||Romania|658[0n]
Phengaris nausithous|EZROM1039-09|RVcoll.07-C167||Romania|658[0n]
Phengaris nausithous|EZROM1038-09|RVcoll.07-C161||Romania|658[0n]
Phengaris nausithous|EZRMN084-08|RVcoll.07-C164||Romania|658[0n]
Phengaris nausithous|EZRMN083-08|RVcoll.06-M917||Romania|658[0n]
Phengaris nausithous|EZROM385-08|RV-07-C166||Romania|658[0n]
Phengaris nausithous|EULEP2946-15|RVcoll.14-O079||Hungary|600[0n]
Phengaris nausithous|EULEP2944-15|RVcoll.14-O077||Hungary|658[0n]
Phengaris nausithous|ABOLD613-17|TLMF Lep 21689||Austria|658[0n]
Phengaris nausithous|ABOLD434-16|TLMF Lep 21586||Austria|658[0n]
Phengaris nausithous|EULEP469-14|KN00831||Russia|658[0n]
Phengaris nausithous|EULEP468-14|KN00830||Russia|658[0n]
Phengaris nausithous|EZROM585-08|RV-07-C331||Romania|657[0n]
Phengaris nausithous|EZRMN086-08|RVcoll.07-C334||Romania|658[0n]
Phengaris nausithous|EZROM586-08|RV-07-C332||Romania|658[0n]
Phengaris nausithous|EULEP2284-15|RVcoll.14-N470||Ukraine|658[0n]
Phengaris nausithous|EULEP4965-16|RVcoll16I353||Poland|658[0n]
Phengaris nausithous|FBLMV483-09|BC ZSM Lep 28463||Germany|658[0n]
Phengaris nausithous|GWORA2458-09|BC ZSM Lep 30670||Germany|658[0n]
Phengaris nausithous|GWORO806-09|BC ZSM Lep 30498||Germany|634[0n]
Phengaris nausithous|ABOLB315-15|TLMF Lep 17320||Austria|658[0n]
Phengaris nausithous|EZSPM294-09|RVcoll.08-J834||Spain|658[0n]
Phengaris nausithous|EZSPC1101-10|RVcoll.08-L971||Spain|658[0n]
Phengaris nausithous|EZSPC1147-10|RVcoll.08-P775||Spain|658[0n]
Phengaris nausithous|EZSPC1185-10|RVcoll.09-T172||Spain|658[0n]
Phengaris nausithous|EZSPN929-09|RVcoll.08-L884||Spain|623[0n]
Phengaris nausithous|EZSPN928-09|RVcoll.08-L883||Spain|658[0n]
Phengaris nausithous|EZSPN512-09|RVcoll.08-H972||Spain|658[0n]
Phengaris nausithous|EZSPN511-09|RVcoll.08-H971||Spain|658[0n]
Phengaris nausithous|GWORA2459-09|BC ZSM Lep 30671||Germany|658[0n]
Phengaris nausithous|PHLAH711-12|TLMF Lep 08530||Austria|658[0n]
Phengaris nausithous|PHLAF629-11|TLMF Lep 05799||Switzerland|658[0n]
Phengaris teleius|GBLAD462-14|BC ZSM Lep 77988||Germany|658[0n]
Phengaris teleius|FBLMV484-09|BC ZSM Lep 28464||Germany|658[0n]
Phengaris teleius|GWORK517-09|BC ZSM Lep 21847||Germany|658[0n]
Phengaris teleius|GWORA2457-09|BC ZSM Lep 30669||Germany|658[0n]
Phengaris teleius|EULEP2216-15|RVcoll.14-N394||Ukraine|658[0n]
Phengaris teleius|EULEP761-15|RVcoll.12-R703||Romania|658[0n]
Phengaris teleius|EULEP5087-16|RVcoll16J783||Poland|658[0n]
Phengaris teleius|EULEP2951-15|RVcoll.14-O084||Serbia|613[0n]
Phengaris teleius|EULEP2232-15|RVcoll.14-N411||Ukraine|658[0n]
Phengaris teleius|EULEP327-14|MM23812||Latvia|658[0n]
Phengaris teleius|EULEP4966-16|RVcoll16I360||Poland|658[0n]
Phengaris teleius|EULEP2394-15|RVcoll.14-V097||Ukraine|658[0n]
Phengaris teleius|EULEP2231-15|RVcoll.14-N410||Ukraine|658[0n]
Phengaris teleius|LEASS680-17|TLMF Lep 22328||Austria|658[0n]
Phengaris teleius|LEASS737-17|TLMF Lep 22385||Austria|658[0n]
Phengaris teleius|EULEP595-15|RVcoll.10-C326||Switzerland|658[0n]
Phengaris teleius|EULEP4407-16|RVcoll15I888||Liechtenstein|658[0n]
Phengaris teleius|EZRMN089-08|RVcoll.06-M885||Romania|658[0n]
Phengaris teleius|EZRMN088-08|RVcoll.07-C168||Romania|658[0n]
Phengaris teleius|EZRMN087-08|RVcoll.07-C163||Romania|658[0n]
Phengaris teleius|EZROM389-08|RV-07-E460|Genit. examined|Romania|657[0n]
Phengaris teleius|EZROM388-08|RV-06-M891|Genit. examined|Romania|658[0n]
Phengaris teleius|EZROM387-08|RV-07-C162||Romania|658[0n]
Phengaris teleius|EZROM679-08|RV-06-M964||Romania|658[0n]
Phengaris teleius|GWORT457-10|BC ZSM Lep 32143||Germany|658[0n]
Phengaris teleius|ABOLD620-17|TLMF Lep 21696||Austria|658[0n]
Phengaris teleius|GWORO808-09|BC ZSM Lep 30500||Germany|658[0n]
Phengaris teleius|ODOPE755-11|BC ZSM Lep 50396||Germany|658[0n]
Phengaris teleius|PHLAW003-13|TLMF Lep 09800||Austria|658[0n]
Phengaris teleius|PHLAF628-11|TLMF Lep 05798||Switzerland|658[0n]
Phengaris teleius|ABOLD433-16|TLMF Lep 21585||Austria|658[0n]
Phengaris arion|EULEP2361-15|RVcoll.14-V053||Ukraine|658[0n]
Phengaris arion|WMB4872-14|RVcoll.14-I419||Italy|658[0n]
Phengaris arion|WMB4287-14|RVcoll.14-A339||Italy|658[0n]
Phengaris arion|WMB4273-14|RVcoll.14-A214||Italy|658[0n]
Phengaris arion|EZRMN082-08|RVcoll.08-M634||Romania|658[0n]
Phengaris arion|EZRMN079-08|RVcoll.08-M307||Romania|658[0n]
Phengaris arion|WMB3655-14|RVcoll.11-I733||France|658[0n]
Phengaris arion|EZSPN529-09|RVcoll.08-H999||Spain|658[0n]
Phengaris arion|EZSPC525-09|RVcoll.08-R116||Spain|658[0n]
Phengaris arion|EZSPC524-09|RVcoll.08-R102||Spain|658[0n]
Phengaris arion|EZSPN033-09|RVcoll.09-T072||Spain|658[0n]
Phengaris arion|EZSPC526-09|RVcoll.08-R119||Spain|658[0n]
Phengaris arion|EZSPC523-09|RVcoll.08-R101||Spain|658[0n]
Phengaris arion|EZRMN081-08|RVcoll.08-M566||Romania|658[0n]
Phengaris arion|EZRMN080-08|RVcoll.08-M337||Romania|658[0n]
Phengaris arion|EZROM637-08|RV-06-M988||Romania|658[0n]
Phengaris arion|FBLMV697-09|BC ZSM Lep 28677||Germany|658[0n]
Phengaris arion|ABOLD612-17|TLMF Lep 21688||Austria|658[0n]
Phengaris arion|WMB5373-14|RVcoll.14-J774||France|658[0n]
Phengaris arion|WMB939-13|RVcoll.10-A604||France|658[0n]
Phengaris arion|EULEP4397-16|RVcoll15G822||Italy|658[0n]
Phengaris arion|EULEP4396-16|RVcoll10B310||Bulgaria|658[0n]
Phengaris arion|EULEP4400-16|RVcoll15H299||Switzerland|658[0n]
[illegible]

Phengaris arion|EULEP4397-16|RVcoll15G822||Italy|658[0n]
Phengaris arion|EULEP4396-16|RVcoll10B310||Bulgaria|658[0n]
Phengaris arion|EULEP4400-16|RVcoll15H299||Switzerland|658[0n]
Phengaris arion|EULEP4399-16|RVcoll15H156||Italy|658[0n]
Phengaris arion|EULEP4402-16|RVcoll15H595||Switzerland|658[0n]
Phengaris arion|EULEP4401-16|RVcoll15H444||Switzerland|658[0n]
Phengaris arion|EULEP185-14|RVcoll.11-J864||Romania|658[0n]
Phengaris arion|EULEP145-14|RVcoll.10-C430||Romania|658[0n]
Phengaris arion|EULEP1519-15|RVcoll.14-G442||Greece|658[0n]
Phengaris arion|EULEP5143-17|RVcoll16J899||Serbia|658[0n]
Phengaris arion|EZROM381-08|RV-06-M887||Romania|658[0n]
Phengaris arion|LEEUA502-11|MM20561||Latvia|658[0n]
Phengaris arion|GWORA2456-09|BC ZSM Lep 30668||Germany|658[0n]
Phengaris arion|GWORO807-09|BC ZSM Lep 30499||Germany|658[0n]
Phengaris arion|LEATD294-13|TLMF Lep 12941||Italy|658[0n]
Phengaris arion|PHLAI499-13|TLMF Lep 09061||Austria|658[0n]
Phengaris arion|ODOPE754-11|BC ZSM Lep 50395||Germany|658[0n]
Phengaris arion|PHLAB359-10|TLMF Lep 01159||Switzerland|658[0n]
Phengaris arion|EZROM382-08|RV-07-D480||Romania|658[0n]
Phengaris arion|EZROM383-08|RV-07-C135||Romania|658[0n]
Phengaris arion|BIBSA1217-15|15-M352||Italy|658[0n]
Phengaris arion|EULEP2459-15|RVcoll.14-V283||Switzerland|658[0n]
Phengaris arion|BIBSA1245-15|15-F824||France|658[0n]
Phengaris arion|BIBSA433-15|RVcoll.14-I101||Italy|658[0n]
Phengaris arion|BIBSA659-15|RVcoll. 15-A822||Italy|658[0n]
Phengaris arion|GBLAB127-13|BC ZSM Lep 75753||Germany|658[0n]
Phengaris arion|LEASS545-17|TLMF Lep 22193||Austria|658[0n]
Phengaris arion|LEASS1051-17|TLMF Lep 22604||Austria|658[0n]
Phengaris arion|LEASS561-17|TLMF Lep 22209||Austria|658[0n]
Phengaris arion|LEASS562-17|TLMF Lep 22210||Austria|658[0n]
Phengaris arion|OXB736-15|13-U240||Italy|658[0n]
Phengaris arion|OXB987-15|15-A937||Italy|658[0n]
Phengaris arion|OXB322-15|RVcoll.14-N036||Italy|658[0n]
Phengaris arion|OXB1263-15|Rvcoll_15-M119||France|658[0n]
Phengaris arion|EULEP1272-15|RVcoll.14-F671||Greece|616[0n]
Phengaris arion|GWOSK800-11|BC ZSM Lep 49016||Germany|638[0n]
Phengaris arion|BIBSA657-15|RVcoll. 15-A820||Italy|634[0n]
Phengaris arion|LEFIL250-10|MM19250||Estonia|632[0n]
Phengaris arion|LEFIJ2530-15|MM23892||Finland|609[0n]
Phengaris arion|LEFIJ2529-15|MM23891||Finland|643[0n]
Phengaris arion|EZROM371-08|07-E620||Spain|609[0n]
Phengaris arion|LEATF472-14|TLMF Lep 13784||Austria|622[0n]
Phengaris arion|PHLAH701-12|TLMF Lep 08520||Austria|658[0n]
Phengaris arion|BIBSA638-15|RVcoll. 15-A801||Italy|658[0n]
Phengaris arion|EULEP4398-16|RVcoll15G901||Switzerland|658[0n]
Phengaris arion|WMB4878-14|RVcoll.14-I425||Italy|658[0n]
Phengaris arion|EZSPN1021-09|RVcoll.08-M953||Spain|658[0n]
Phengaris arion|PHLAB325-10|TLMF Lep 01125||Switzerland|658[0n]
Phengaris arion|LEATD295-13|TLMF Lep 12942||Italy|658[0n]
Phengaris alcon|EULEP4394-16|RVcoll15G139||France|658[0n]
Phengaris alcon|EULEP059-14|RVcoll.08-J489||Spain|658[0n]
Phengaris alcon|EULEP060-14|RVcoll.08-J490||Spain|658[0n]
Phengaris alcon|EULEP061-14|RVcoll.08-J491||Spain|658[0n]
Phengaris alcon|EULEP201-14|RVcoll.12-L013||Spain|658[0n]
Phengaris alcon|EULEP202-14|RVcoll.12-L014||Spain|658[0n]
Phengaris alcon|BIBSA1892-17|LZCollB17A015||Italy|658[0n]
Phengaris alcon|WMB1264-13|RVcoll.12-M088||France|606[0n]
Phengaris alcon|EZSPM791-12|Rvcoll. 070611MH65||Spain|658[0n]
Phengaris alcon|EZROM370-08|RVcoll.07-E619||Spain|658[0n]
Phengaris alcon|EZSPM805-12|Rvcoll. 090611SZ07||Spain|658[0n]
Phengaris alcon|EZSPN516-09|RVcoll.08-H981||Spain|658[0n]
Phengaris alcon|EZSPN515-09|RVcoll.08-H980||Spain|658[0n]
Phengaris alcon|EZSPN223-09|RVcoll.07-F532||Spain|658[0n]
Phengaris alcon|EZSPN741-09|RVcoll.08-L163||Spain|658[0n]
Phengaris alcon|PHLAB363-10|TLMF Lep 01163||Switzerland|658[0n]
Phengaris alcon|LEATG257-14|TLMF Lep 14044||Austria|658[0n]
Phengaris alcon|EULEP4405-16|RVcoll14W539||Italy|658[0n]
Phengaris alcon|BIBSA434-15|RVcoll.14-I102||Italy|658[0n]
Phengaris alcon|OXB617-15|14-O006||Italy|658[0n]
Phengaris alcon|EULEP4406-16|RVcoll14W684||Italy|637[0n]
Phengaris alcon|EULEP4395-16|RVcoll15G232||Switzerland|637[0n]
Phengaris alcon|PHLAB362-10|TLMF Lep 01162||Switzerland|635[0n]
Phengaris alcon|ABOLD436-16|TLMF Lep 21588||Austria|658[0n]
Phengaris alcon|LEATG259-14|TLMF Lep 14046||Italy|627[0n]
Phengaris alcon|LEATG260-14|TLMF Lep 14047||Italy|658[0n]
Phengaris alcon|ODOPE248-11|BC ZSM Lep 53119||Germany|658[0n]
Phengaris alcon|ABOLD621-17|TLMF Lep 21697||Austria|658[0n]
Phengaris alcon|ABOLD617-17|TLMF Lep 21693||Austria|658[0n]
Phengaris alcon|FBLMV714-09|BC ZSM Lep 28694|f. rebeli, from G. cruciata|Germany|658[0n]
Phengaris alcon|EZRMN076-08|RVcoll.07-C158||Romania|658[0n]
Phengaris alcon|EZRMN078-08|RVcoll.08-M513||Romania|658[0n]
Phengaris alcon|EZROM1042-09|RVcoll.07-C928||Romania|658[0n]
Phengaris alcon|EZROM377-08|RV-06-M892||Romania|658[0n]
Phengaris alcon|EZROM379-08|RV-07-C156||Romania|658[0n]
Phengaris alcon|EZROM386-08|RV-06-N013||Romania|658[0n]
Phengaris alcon|EZROM380-08|RV-07-C927||Romania|658[0n]
Phengaris alcon|EZRMN075-08|RVcoll.06-M886||Romania|658[0n]
Phengaris alcon|EZRMN077-08|RVcoll.07-E461||Romania|658[0n]
Phengaris alcon|WMB5236-14|RVcoll.13-U086||Italy|658[0n]
Phengaris alcon|EULEP4391-16|RVcoll15G821||Italy|658[0n]
Phengaris alcon|EULEP4392-16|RVcoll15H231||Switzerland|658[0n]
Phengaris alcon|EULEP4393-16|RVcoll15I161||Austria|658[0n]
Phengaris alcon|EULEP062-14|RVcoll.08-J492||Spain|658[0n]
Phengaris alcon|EULEP063-14|RVcoll.08-J493||Spain|658[0n]
Phengaris alcon|EULEP340-14|MM23825||Latvia|658[0n]
Phengaris alcon|EULEP1112-15|RVcoll.14-F185||Serbia|658[0n]
Phengaris alcon|EULEP946-15|RVcoll.14-C944||Bulgaria|658[0n]
Phengaris alcon|EULEP2397-15|RVcoll.14-V101||Ukraine|658[0n]
Phengaris alcon|EULEP2230-15|RVcoll.14-N409||Ukraine|658[0n]
Phengaris alcon|BIBSA1072-15|15-I095||Italy|658[0n]
Phengaris alcon|BIBSA1893-17|LZCollB17A016||Italy|658[0n]
Phengaris alcon|BIBSA1894-17|LZCollB17A017||Italy|658[0n]
Phengaris alcon|GBLAC370-13|BC ZSM Lep 75236||Germany|658[0n]

Phengaris alcon|BIBSA1893-17|LZCollB17A016||Italy|658[0n]
Phengaris alcon|BIBSA1894-17|LZCollB17A017||Italy|658[0n]
Phengaris alcon|GBLAC370-13|BC ZSM Lep 75236||Germany|658[0n]
Phengaris alcon|LEASS745-17|TLMF Lep 22393||Austria|658[0n]
Phengaris alcon|LEASS544-17|TLMF Lep 22192||Austria|658[0n]
Phengaris alcon|LEASS738-17|TLMF Lep 22386||Austria|658[0n]
Phengaris alcon|OXB727-15|13-U176||Italy|658[0n]
Phengaris alcon|OXB317-15|RVcoll.14-N031||Italy|658[0n]
Phengaris alcon|ABOLD616-17|TLMF Lep 21692||Austria|633[0n]
Phengaris alcon|EZROM378-08|RV-07-E595||Romania|658[0n]
Phengaris alcon|LEATG258-14|TLMF Lep 14045||Austria|658[1n]
Phengaris alcon|LEATJ761-15|TLMF Lep 18336||Italy|658[0n]
Phengaris alcon|LEATJ762-15|TLMF Lep 18337||Italy|658[0n]
Luthrodes galba|EULEP2922-15|RVcoll.14-O055|Cyprus|Cyprus|658[0n]
Luthrodes galba|EULEP2923-15|RVcoll.14-O056|Cyprus|Cyprus|658[0n]
Luthrodes galba|EULEP2921-15|RVcoll.14-U874|Cyprus|Cyprus|658[0n]
Freyeria trochylus|EULEP1743-15|RVcoll.14-H348||Greece|658[0n]
Freyeria trochylus|EULEP1320-15|RVcoll.14-F784||Greece|658[0n]
Freyeria trochylus|EULEP1319-15|RVcoll.14-F782||Greece|658[0n]
Freyeria trochylus|EULEP1318-15|RVcoll.14-F781||Greece|658[0n]
Freyeria trochylus|EULEP4876-16|RVcoll16H969||Cyprus|644[0n]
Tarucus balkanicus|EULEP2557-15|RVcoll.14-V482||Greece|627[0n]
Tarucus balkanicus|EULEP1833-15|RVcoll.14-J955||Macedonia|658[0n]
Tarucus balkanicus|EULEP4662-16|RVcoll14A921||Cyprus|658[0n]
Tarucus balkanicus|EULEP1601-15|RVcoll.14-G654||Greece|658[0n]
Tarucus balkanicus|EULEP1608-15|RVcoll.14-G663||Greece|658[0n]
Tarucus balkanicus|EULEP1830-15|RVcoll.14-J951||Bulgaria|658[0n]
Tarucus balkanicus|EULEP1834-15|RVcoll.14-J956||Macedonia|658[0n]
Tarucus balkanicus|EULEP1576-15|RVcoll.14-G600||Macedonia|658[0n]
Tarucus balkanicus|EULEP1301-15|RVcoll.14-F738||Greece|658[0n]
Tarucus balkanicus|EULEP1597-15|RVcoll.14-G646||Greece|658[0n]
Tarucus balkanicus|EULEP1300-15|RVcoll.14-F737||Greece|658[0n]
Tarucus balkanicus|EULEP1299-15|RVcoll.14-F736||Greece|658[0n]
Tarucus balkanicus|EULEP4663-16|RVcoll14A983||Bulgaria|658[0n]
Tarucus balkanicus|EULEP4877-16|RVcoll16H970||Cyprus|658[0n]
Tarucus balkanicus|EULEP4661-16|RVcoll14A922||Cyprus|658[0n]
Tarucus theophrastus|EZSPC1152-10|RVcoll.08-R029.1||Spain|658[0n]
Tarucus theophrastus|EZSPM170-09|RVcoll.08-R027||Spain|658[0n]
Tarucus theophrastus|EZSPM169-09|RVcoll.08-R022||Spain|658[0n]
Tarucus theophrastus|EZSPM167-09|RVcoll.08-R019||Spain|658[0n]
Celastrina argiolus|WMB1488-13|RVcoll.12-O016|Giglio|Italy|658[0n]
Celastrina argiolus|WMB2597-13|RVcoll.12-R353|Elba|Italy|658[0n]
Celastrina argiolus|BIBSA076-14|LEP-SS-00076||Italy|658[0n]
Celastrina argiolus|WMB2617-13|RVcoll.12-O185|Corsica|France|658[0n]
Celastrina argiolus|WMB2273-13|RVcoll.LD-2733||Malta|658[0n]
Celastrina argiolus|WMB2202-13|RVcoll.10-C583||Malta|658[0n]
Celastrina argiolus|WMB2174-13|RVcoll.12-R342|Elba|Italy|658[0n]
Celastrina argiolus|WMB1564-13|RVcoll.12-O435|Sardinia|Italy|658[0n]
Celastrina argiolus|WMB1810-13|RVcoll.11-I369||Italy|658[0n]
Celastrina argiolus|WMB1531-13|RVcoll.12-O250|Corsica|France|658[0n]
Celastrina argiolus|WMB2728-13|RVcoll.10-C585||Malta|658[0n]
Celastrina argiolus|WMB2833-13|RVcoll.12-O545|Sardinia|Italy|658[0n]
Celastrina argiolus|WMB2812-13|RVcoll.12-M531|Capraia|Italy|658[0n]
Celastrina argiolus|WMB2977-14|RVcoll.12-O087|Corsica|France|658[0n]
Celastrina argiolus|WMB2960-14|RVcoll.12-N639|Menorca|Spain|658[0n]
Celastrina argiolus|WMB2908-14|RVcoll.11-E825|Corsica|France|658[0n]
Celastrina argiolus|EZSPM1001-12|Rvcoll.12-L887||Spain|658[0n]
Celastrina argiolus|BIBSA1412-15|15-N031||Italy|658[0n]
Celastrina argiolus|BIBSA1276-15|12-O352|Sardinia|Italy|658[0n]
Celastrina argiolus|WMB5506-14|RVcoll.14-J441|Montecristo|Italy|658[0n]
Celastrina argiolus|WMB5325-14|RVcoll.14-J016|La Maddalena|Italy|658[0n]
Celastrina argiolus|OXB1246-15|Rvcoll_13-T728|Sardinia|Italy|658[0n]
Celastrina argiolus|OXB1241-15|Rvcoll_13-T685|Sardinia|Italy|658[0n]
Celastrina argiolus|WMB323-11|RVcoll.11-E608||Italy|658[0n]
Celastrina argiolus|WMB316-11|RVcoll.11-E502||Italy|658[0n]
Celastrina argiolus|WMB359-11|RVcoll.11-E946||France|658[0n]
Celastrina argiolus|WMB355-11|RVcoll.11-E813||France|658[0n]
Celastrina argiolus|EZSPM994-12|Rvcoll.12-L880||Spain|658[0n]
Celastrina argiolus|EULEP3633-16|RVcoll15P023||Belarus|658[0n]
Celastrina argiolus|OXB223-15|OXB-TGS-420||United Kingdom|658[0n]
Celastrina argiolus|LEFIJ2395-14|MM23778||Finland|658[0n]
Celastrina argiolus|EULEP2340-15|RVcoll.14-V026||Ukraine|658[0n]
Celastrina argiolus|EULEP839-15|RVcoll.14-C017||Sweden|658[0n]
Celastrina argiolus|EULEP4940-16|RVcoll16I066||Poland|658[0n]
Celastrina argiolus|EULEP4749-16|RVcoll16G788||Sweden|658[0n]
Celastrina argiolus|GWORK518-09|BC ZSM Lep 21848||Germany|658[0n]
Celastrina argiolus|LEFIJ512-10|MM17137||Finland|658[0n]
Celastrina argiolus|LEFIJ511-10|MM17136||Finland|658[0n]
Celastrina argiolus|FBLMU439-09|BC ZSM Lep 27089||Germany|658[0n]
Celastrina argiolus|GWORL416-09|BC ZSM Lep 22318||Germany|658[0n]
Celastrina argiolus|LEFIB124-10|MM00488||Finland|658[0n]
Celastrina argiolus|EZSPC200-09|RVcoll.06-H841||Spain|654[0n]
Celastrina argiolus|GWORO793-09|BC ZSM Lep 30485||Germany|658[0n]
Celastrina argiolus|LEATI009-15|TLMF Lep 17394||Austria|658[0n]
Celastrina argiolus|BIBSA1778-16|16-A484||Italy|613[0n]
Celastrina argiolus|OXB1385-15|Rvcoll_15-M750||France|658[0n]
Celastrina argiolus|WMB5364-14|RVcoll.14-J736||France|658[0n]
Celastrina argiolus|BIBSA1796-16|16-A550||Italy|611[0n]
Celastrina argiolus|GBLAC285-13|BC ZSM Lep 78666||Germany|658[0n]
Celastrina argiolus|WMB1930-13|RVcoll.11-H806|Vulcano|Italy|633[0n]
Celastrina argiolus|WMB1931-13|RVcoll.11-H807|Vulcano|Italy|658[0n]
Celastrina argiolus|ABOLD076-16|TLMF Lep 21154||Austria|658[0n]
Celastrina argiolus|LEATI010-15|TLMF Lep 17395||Austria|658[0n]
Celastrina argiolus|ODOPE367-11|BC ZSM Lep 50768||Germany|658[0n]
Celastrina argiolus|EZROM843-08|RVcoll.07-C418||Romania|658[0n]
Celastrina argiolus|EZROM844-08|RVcoll.07-D573||Romania|658[0n]
Celastrina argiolus|EZROM084-08|RV-06-K579||Romania|658[0n]
Celastrina argiolus|EZROM085-08|RV-06-K581||Romania|658[0n]
Celastrina argiolus|EZROM086-08|RV-07-D959||Romania|658[0n]
Celastrina argiolus|EZSPN819-09|RVcoll.08-L628||Spain|658[0n]
Celastrina argiolus|EZSPN408-09|RVcoll.08-H504||Spain|658[0n]
Celastrina argiolus|EZSPN092-09|RVcoll.06-A021||Spain|658[0n]
[illegible]

Celastrina argiolus|EZSPN819-09|RVcoll.08-L028||Spain|658[0n]
Celastrina argiolus|EZSPN408-09|RVcoll.08-H504||Spain|658[0n]
Celastrina argiolus|EZSPN092-09|RVcoll.06-A021||Spain|658[0n]
Celastrina argiolus|EZSPM055-09|RVcoll.08-P279||Spain|658[0n]
Celastrina argiolus|EZSPN325-09|RVcoll.08-H196||Spain|658[0n]
Celastrina argiolus|EZSPN354-09|RVcoll.08-H327||Spain|658[0n]
Celastrina argiolus|EZSPN486-09|RVcoll.08-H923||Spain|658[0n]
Celastrina argiolus|EZSPM1030-12|Rvcoll.12-M780||Spain|658[0n]
Celastrina argiolus|EZSPM270-09|RVcoll.08-R487||Spain|658[0n]
Celastrina argiolus|EZSPN309-09|RVcoll.08-H116||Spain|658[0n]
Celastrina argiolus|EZSPM080-09|RVcoll.08-J815||Spain|658[0n]
Celastrina argiolus|EZSPM1045-12|Rvcoll.12-N000||Spain|658[0n]
Celastrina argiolus|EZSPM135-09|RVcoll.08-P645||Spain|658[0n]
Celastrina argiolus|EZSPM175-09|RVcoll.08-R040||Spain|658[0n]
Celastrina argiolus|EZSPM249-09|RVcoll.08-R461||Spain|658[0n]
Celastrina argiolus|EZSPM939-12|Rvcoll.12-M673||Spain|658[0n]
Celastrina argiolus|WMB137-11|RVcoll.11-D152|Pantelleria|Italy|658[0n]
Celastrina argiolus|WMB002-11|RVcoll.11-D514||Italy|658[0n]
Celastrina argiolus|WMB122-11|RVcoll.11-D000|Capri|Italy|658[0n]
Celastrina argiolus|WMB2922-14|RVcoll.11-I370|Argentario|Italy|658[0n]
Celastrina argiolus|WMB2923-14|RVcoll.11-I372|Argentario|Italy|658[0n]
Celastrina argiolus|WMB3401-14|RVcoll.09-V775||Spain|658[0n]
Celastrina argiolus|WMB3441-14|RVcoll.09-X556||Spain|658[0n]
Celastrina argiolus|WMB2957-14|RVcoll.12-N009|Ibiza|Spain|658[0n]
Celastrina argiolus|WMB3039-14|RVcoll.13-S567|San Domino|Italy|658[0n]
Celastrina argiolus|WMB3696-14|RVcoll.11-J551||Spain|658[0n]
Celastrina argiolus|WMB3202-14|RVcoll.08-J203||Spain|658[0n]
Celastrina argiolus|WMB3246-14|RVcoll.08-L218||Spain|658[0n]
Celastrina argiolus|WMB3251-14|RVcoll.08-L295||Spain|658[0n]
Celastrina argiolus|WMB2784-13|RVcoll.11-I301|Pianosa|Italy|658[0n]
Celastrina argiolus|WMB2844-13|RVcoll.12-Q704||Italy|658[0n]
Celastrina argiolus|WMB3586-14|RVcoll.11-E089||Spain|658[0n]
Celastrina argiolus|WMB3644-14|RVcoll.11-I588||Spain|658[0n]
Celastrina argiolus|WMB3665-14|RVcoll.11-I866||France|658[0n]
Celastrina argiolus|WMB1133-13|RVcoll.11-E111|Gozo|Malta|658[0n]
Celastrina argiolus|WMB1134-13|RVcoll.11-E112|Gozo|Malta|658[0n]
Celastrina argiolus|WMB2764-13|RVcoll.11-H808.1|Vulcano|Italy|658[0n]
Celastrina argiolus|WMB1487-13|RVcoll.12-O015|Giglio|Italy|658[0n]
Celastrina argiolus|WMB1091-13|RVcoll.10-C756|Capraia|Italy|658[0n]
Celastrina argiolus|WMB1773-13|RVcoll.12-Q065||France|658[0n]
Celastrina argiolus|WMB1568-13|RVcoll.12-O497|Sardinia|Italy|658[0n]
Celastrina argiolus|WMB1596-13|RVcoll.12-O616||France|658[0n]
Celastrina argiolus|WMB1706-13|RVcoll.12-P640||France|658[0n]
Celastrina argiolus|WMB1737-13|RVcoll.12-P949|Levant|France|658[0n]
Celastrina argiolus|WMB1941-13|RVcoll.11-H871|Salina|Italy|658[0n]
Celastrina argiolus|WMB1990-13|RVcoll.11-I302|Pianosa|Italy|658[0n]
Celastrina argiolus|WMB1991-13|RVcoll.11-I303|Pianosa|Italy|658[0n]
Celastrina argiolus|WMB2208-13|RVcoll.11-Y017|Elba|Italy|658[0n]
Celastrina argiolus|WMB2429-13|RVcoll.11-D005|Capri|Italy|658[0n]
Celastrina argiolus|WMB5925-17|RVcoll11D238|Sicily|Italy|658[0n]
Celastrina argiolus|WMB2432-13|RVcoll.11-D113|Pantelleria|Italy|658[0n]
Celastrina argiolus|WMB2574-13|RVcoll.12-Q973|Sicily|Italy|658[0n]
Celastrina argiolus|WMB4151-14|RVcoll.13-S695||Italy|658[0n]
Celastrina argiolus|WMB4241-14|RVcoll.13-T848||Italy|658[0n]
Celastrina argiolus|WMB3879-14|RVcoll.12-M506||Italy|658[0n]
Celastrina argiolus|WMB4484-14|RVcoll.14-D929||Italy|658[0n]
Celastrina argiolus|WMB4494-14|RVcoll.14-E170||Italy|658[0n]
Celastrina argiolus|WMB4561-14|RVcoll.090211SD65||Spain|658[0n]
Celastrina argiolus|WMB4575-14|RVcoll.150308GV11|Genit. examined|Spain|658[0n]
Celastrina argiolus|WMB4012-14|RVcoll.12-R198||Italy|658[0n]
Celastrina argiolus|WMB4296-14|RVcoll.14-A392||Italy|658[0n]
Celastrina argiolus|WMB4328-14|RVcoll.14-A679||Italy|658[0n]
Celastrina argiolus|WMB4377-14|RVcoll.14-B176||Portugal|658[0n]
Celastrina argiolus|WMB4657-14|RVcoll.LD-2558|Lipari|Italy|658[0n]
Celastrina argiolus|WMB4664-14|RVcoll.LD-2574|Stromboli|Italy|658[0n]
Celastrina argiolus|WMB4668-14|RVcoll.LD-2619|Ischia|Italy|658[0n]
Celastrina argiolus|WMB489-11|RVcoll.11-H535|Sicily|Italy|658[0n]
Celastrina argiolus|WMB482-11|RVcoll.11-H511|Capri|Italy|658[0n]
Celastrina argiolus|WMB529-11|RVcoll.11-H657|Pantelleria|Italy|658[0n]
Celastrina argiolus|WMB5083-14|RVcoll. 14-L214||Italy|658[0n]
Celastrina argiolus|WMB582-11|RVcoll.11-H870|Salina|Italy|658[0n]
Celastrina argiolus|WMB631-11|RVcoll.11-I116||Italy|658[0n]
Celastrina argiolus|WMB4754-14|RVcoll. 14-I301||Italy|658[0n]
Celastrina argiolus|WMB4764-14|RVcoll. 14-I311||Italy|658[0n]
Celastrina argiolus|WMB4975-14|RVcoll. 14-I522||Italy|658[0n]
Celastrina argiolus|WMB5044-14|RVcoll. 14-L175||Italy|658[0n]
Celastrina argiolus|WMB5174-14|RVcoll.13-S521||Italy|658[0n]
Celastrina argiolus|WMB5265-14|RVcoll.14-A484||Italy|658[0n]
Celastrina argiolus|WMB5477-14|RVcoll.14-N702|Alicudi|Italy|658[0n]
Celastrina argiolus|WMB5484-14|RVcoll.14-N725|Stromboli|Italy|658[0n]
Celastrina argiolus|EULEP3631-16|RVcoll15H380||Switzerland|658[0n]
Celastrina argiolus|EULEP5006-16|RVcoll16I810||Germany|658[0n]
Celastrina argiolus|EULEP5643-17|RVcoll15D999|Gorgona|Italy|658[0n]
Celastrina argiolus|EULEP5691-17|RVcoll16L034||Italy|658[0n]
Celastrina argiolus|EULEP700-15|RVcoll.12-M149|Crete|Greece|658[0n]
Celastrina argiolus|EULEP1199-15|RVcoll.14-F486||Bulgaria|658[0n]
Celastrina argiolus|EULEP1589-15|RVcoll.14-G622||Greece|658[0n]
Celastrina argiolus|EULEP855-15|RVcoll.14-C165||Greece|658[0n]
Celastrina argiolus|EULEP2421-15|RVcoll.14-V203||Belgium|658[0n]
Celastrina argiolus|EULEP1994-15|RVcoll.14-I760||Poland|658[0n]
Celastrina argiolus|BIBSA1119-15|15-L864||Italy|658[0n]
Celastrina argiolus|BIBSA1219-15|15-L952||Italy|658[0n]
Celastrina argiolus|BIBSA1605-16|14-A651||Italy|658[0n]
Celastrina argiolus|BIBSA987-15|14-W581||Italy|658[0n]
Celastrina argiolus|BIBSA1636-16|15-C491||Italy|658[0n]
Celastrina argiolus|BIBSA1337-15|15-M829||Italy|658[0n]
Celastrina argiolus|BIBSA1550-16|11-D388||Italy|658[0n]
Celastrina argiolus|BIBSA455-15|RVcoll.14-I123||Italy|658[0n]
Celastrina argiolus|BIBSA1635-16|15-C488||Italy|658[0n]
Celastrina argiolus|BIBSA075-14|LEP-SS-00075||Italy|658[0n]
Celastrina argiolus|GBLAA057-14|BC ZSM Lep 80718||Germany|658[0n]
Celastrina argiolus|GBLAF828-14|BC ZSM Lep 82914||Germany|658[0n]
Celastrina argiolus|GBLAC1090-13|BC ZSM Lep 77381||Germany|658[0n]

Celastrina argiolus|GBLAA057-14|BC ZSM Lep 80718||Germany|658[0n]
Celastrina argiolus|GBLAF828-14|BC ZSM Lep 82914||Germany|658[0n]
Celastrina argiolus|GBLAC1090-13|BC ZSM Lep 77381||Germany|658[0n]
Celastrina argiolus|LEASS915-17|KLM Lep 08420||Austria|658[0n]
Celastrina argiolus|OXB1448-16|OXB-TGS-1222||United Kingdom|658[0n]
Celastrina argiolus|OXB576-15|14-N965||Italy|658[0n]
Celastrina argiolus|OXB751-15|13-U472||Italy|658[0n]
Celastrina argiolus|OXB900-15|15-A544||Italy|658[0n]
Celastrina argiolus|OXB945-15|15-A589||Italy|658[0n]
Celastrina argiolus|OXB978-15|15-A928||Italy|658[0n]
Celastrina argiolus|OXB1555-16|OXB-TGS-1273||France|658[0n]
Celastrina argiolus|OXB269-15|OXB-TGS-952||United Kingdom|658[0n]
Celastrina argiolus|OXB1211-15|Rvcoll_15-M636||France|658[0n]
Celastrina argiolus|BIBSA941-15|16-A032||Italy|658[0n]
Celastrina argiolus|EZSPC280-09|RVcoll.08-R285||Spain|652[0n]
Celastrina argiolus|EZSPC212-09|RVcoll.08-J338||Spain|647[0n]
Celastrina argiolus|EZSPC251-09|RVcoll.08-P323||Spain|647[0n]
Celastrina argiolus|EZSPC240-09|RVcoll.08-P034||Spain|641[0n]
Celastrina argiolus|WMB1289-13|RVcoll.12-M530|Capraia|Italy|636[0n]
Celastrina argiolus|EZSPM038-09|RVcoll.08-P243||Spain|639[0n]
Celastrina argiolus|EZROM087-08|RV-07-D977||Romania|609[0n]
Celastrina argiolus|BIBSA734-15|RVcoll. 11-D029||Italy|658[0n]
Celastrina argiolus|EULEP1935-15|RVcoll.14-B906||Bosnia and Herzegovina|658[0n]
Celastrina argiolus|EULEP1336-15|RVcoll.14-F825||Greece|658[0n]
Celastrina argiolus|EULEP3632-16|RVcoll15I500||Austria|658[0n]
Celastrina argiolus|WMB5965-17|RVcoll14N636|Vulcano|Italy|658[0n]
Celastrina argiolus|WMB2727-13|RVcoll.10-C584||Malta|658[0n]
Celastrina argiolus|WMB175-11|RVcoll.11-D436|Sicily|Italy|658[0n]
Celastrina argiolus|WMB167-11|RVcoll.11-D387|Sicily|Italy|658[0n]
Celastrina argiolus|EZROM088-08|RV-07-E384||Romania|658[0n]
Celastrina argiolus|EZROM846-08|RVcoll.08-M444||Romania|658[0n]
Celastrina argiolus|EZROM845-08|RVcoll.07-D932||Romania|658[0n]
Celastrina argiolus|LEATG421-14|TLMF Lep 14208||Italy|658[0n]
Celastrina argiolus|PHLAW027-13|TLMF Lep 09824||Austria|658[0n]
Celastrina argiolus|LEATJ254-15|TLMF Lep 18684||Italy|658[0n]
Celastrina argiolus|LEATJ253-15|TLMF Lep 18683||Italy|658[0n]
Azanus jesous|EULEP030-14|RVcoll.07-D788||Spain|658[0n]
Cacyreus marshalli|EZSPC1190-10|RVcoll.09-V334||Spain|658[0n]
Cacyreus marshalli|WMB2955-14|RVcoll.12-N002|Ibiza|Spain|658[0n]
Cacyreus marshalli|WMB2961-14|RVcoll.12-N669|Menorca|Spain|658[0n]
Cacyreus marshalli|WMB1105-13|RVcoll.10-C783||Italy|658[0n]
Cacyreus marshalli|WMB1117-13|RVcoll.11-D159|Marettimo|Italy|658[0n]
Cacyreus marshalli|WMB2732-13|RVcoll.10-C590||Malta|658[0n]
Cacyreus marshalli|WMB2733-13|RVcoll.10-C591||Malta|658[0n]
Cacyreus marshalli|WMB810-13|RVcoll.07-E108||Italy|658[0n]
Cacyreus marshalli|WMB1485-13|RVcoll.12-O013|Giglio|Italy|658[0n]
Cacyreus marshalli|WMB1266-13|RVcoll.12-M285|Lipari|Italy|658[0n]
Cacyreus marshalli|WMB1561-13|RVcoll.12-O407|San Pietro|Italy|658[0n]
Cacyreus marshalli|WMB1756-13|RVcoll.12-P977|Levant|France|658[0n]
Cacyreus marshalli|WMB1757-13|RVcoll.12-P978|Levant|France|658[0n]
Cacyreus marshalli|WMB1915-13|RVcoll.11-H660|Pantelleria|Italy|658[0n]
Cacyreus marshalli|WMB1952-13|RVcoll.11-H978||Italy|658[0n]
Cacyreus marshalli|WMB2203-13|RVcoll.10-C599||Malta|658[0n]
Cacyreus marshalli|WMB2383-13|RVcoll.07-E114||Italy|658[0n]
Cacyreus marshalli|WMB2424-13|RVcoll.10-C770|Giglio|Italy|658[0n]
Cacyreus marshalli|WMB3805-14|RVcoll.07-D845||Italy|658[0n]
Cacyreus marshalli|WMB2612-13|RVcoll.12-P982|Levant|France|658[0n]
Cacyreus marshalli|WMB4635-14|RVcoll.LD-2398|Ustica|Italy|658[0n]
Cacyreus marshalli|WMB518-11|RVcoll.11-H610|Lampedusa|Italy|658[0n]
Cacyreus marshalli|WMB531-11|RVcoll.11-H659|Pantelleria|Italy|658[0n]
Cacyreus marshalli|WMB570-11|RVcoll.11-H794|Vulcano|Italy|658[0n]
Cacyreus marshalli|BIBSA621-15|LEP-SS-00241||Italy|658[0n]
Cacyreus marshalli|BIBSA464-15|RVcoll.14-I132||Italy|658[0n]
Cacyreus marshalli|BIBSA465-15|RVcoll.14-I133||Italy|658[0n]
Cacyreus marshalli|BIBSA466-15|RVcoll.14-I134||Italy|658[0n]
Cacyreus marshalli|BIBSA467-15|RVcoll.14-I135||Italy|658[0n]
Cacyreus marshalli|BIBSA469-15|RVcoll.14-I137||Italy|658[0n]
Cacyreus marshalli|BIBSA471-15|RVcoll.14-I139||Italy|658[0n]
Cacyreus marshalli|BIBSA473-15|RVcoll.14-I141||Italy|658[0n]
Cacyreus marshalli|BIBSA475-15|RVcoll.14-I143||Italy|658[0n]
Cacyreus marshalli|BIBSA472-15|RVcoll.14-I140||Italy|658[0n]
Cacyreus marshalli|BIBSA068-14|LEP-SS-00068||Italy|658[0n]
Cacyreus marshalli|OXB1169-15|Rvcoll_16-A056||Italy|658[0n]
Cacyreus marshalli|BIBSA470-15|RVcoll.14-I138||Italy|653[0n]
Cacyreus marshalli|WMB1927-13|RVcoll.11-H793|Vulcano|Italy|646[0n]
Cacyreus marshalli|BIBSA468-15|RVcoll.14-I136||Italy|648[0n]
Cacyreus marshalli|BIBSA474-15|RVcoll.14-I142||Italy|632[0n]
Cacyreus marshalli|WMB1560-13|RVcoll.12-O406|San Pietro|Italy|633[0n]
Cacyreus marshalli|WMB2438-13|RVcoll.11-D161|Marettimo|Italy|633[0n]
Cacyreus marshalli|WMB1101-13|RVcoll.10-C771|Giglio|Italy|623[0n]
Cacyreus marshalli|EZSPC1121-10|RVcoll.08-P468||Spain|633[0n]
Cacyreus marshalli|EZSPC1118-10|RVcoll.08-P460||Spain|658[0n]
Cacyreus marshalli|WMB140-11|RVcoll.11-D160|Marettimo|Italy|658[0n]
Cacyreus marshalli|EZSPM219-09|RVcoll.08-R406||Spain|658[0n]
Cacyreus marshalli|EZSPM206-09|RVcoll.08-R324||Spain|658[0n]
Cacyreus marshalli|EZSPM202-09|RVcoll.08-R300||Spain|655[0n]
Cacyreus marshalli|EZSPM113-09|RVcoll.08-P462||Spain|658[0n]
Cacyreus marshalli|EZSPM059-09|RVcoll.08-P285||Spain|658[0n]
Cacyreus marshalli|EZSPM045-09|RVcoll.08-J814||Spain|658[0n]
Cacyreus marshalli|EZSPM058-09|RVcoll.08-P284||Spain|658[0n]
Cacyreus marshalli|EZSPN222-09|RVcoll.07-F308||Spain|658[0n]
Cacyreus marshalli|GWORU378-10|BC ZSM Lep 31969||Italy|658[0n]
Cacyreus marshalli|GWORR417-10|BC ZSM Lep 29633||Italy|658[0n]
Cacyreus marshalli|LEATG236-14|TLMF Lep 14023||Italy|658[0n]
Cacyreus marshalli|LEATG487-14|TLMF Lep 14274||Italy|658[0n]
Cacyreus marshalli|PHLAH796-12|TLMF Lep 08600||Austria|658[0n]
Lampides boeticus|BCLEP104-17|LEP-SS-00555||Italy|658[0n]
Lampides boeticus|WMB1140-13|RVcoll.11-E131|Gozo|Malta|658[0n]
Lampides boeticus|WMB3955-14|RVcoll.12-P760||France|658[0n]
Lampides boeticus|WMB642-11|RVcoll.11-I154||Italy|658[0n]
Lampides boeticus|WMB292-11|RVcoll.11-E300||Italy|658[0n]
Lampides boeticus|EZROM728-08|RV-06-G522||Spain|658[0n]
Lampides boeticus|EZSPN1028-09|RVcoll.08-M963||Spain|658[0n]

Lampides boeticus|WMB[illegible]-11|RVcoll.11-H[illegible]||Italy|658[0n]
Lampides boeticus|WMB292-11|RVcoll.11-E300||Italy|658[0n]
Lampides boeticus|EZROM728-08|RV-06-G522||Spain|658[0n]
Lampides boeticus|EZSPN1028-09|RVcoll.08-M963||Spain|658[0n]
Lampides boeticus|EULEP1766-15|RVcoll.14-H447||Greece|658[0n]
Lampides boeticus|WMB4659-14|RVcoll.LD-2567|Lipari|Italy|658[0n]
Lampides boeticus|WMB2304-13|RVcoll.11-E134|Gozo|Malta|658[0n]
Lampides boeticus|WMB1492-13|RVcoll.12-O021|Giglio|Italy|658[0n]
Lampides boeticus|WMB4433-14|RVcoll.14-B527||Portugal|658[0n]
Lampides boeticus|WMB2628-13|RVcoll.12-P960|Levant|France|658[0n]
Lampides boeticus|BIBSA1531-16|07-E045||Italy|658[0n]
Lampides boeticus|BCLEP105-17|LEP-SS-00556||Italy|658[0n]
Lampides boeticus|EZSPM960-12|Rvcoll.12-M711||Spain|658[0n]
Lampides boeticus|WMB2625-13|RVcoll.12-R445|Corsica|France|658[0n]
Lampides boeticus|WMB3064-14|RVcoll.14-A794|Capri|Italy|658[0n]
Lampides boeticus|BIBSA1664-16|15-C760||Italy|658[0n]
Lampides boeticus|WMB4634-14|RVcoll.LD-2394|Ustica|Italy|658[0n]
Lampides boeticus|WMB2173-13|RVcoll.12-R333|Elba|Italy|658[0n]
Lampides boeticus|EZSPN479-09|RVcoll.08-H914||Spain|658[0n]
Lampides boeticus|EZSPM970-12|Rvcoll.12-M728||Spain|658[0n]
Lampides boeticus|BIBSA077-14|LEP-SS-00077||Italy|658[0n]
Lampides boeticus|WMB2263-13|RVcoll.12-R525|Capraia|Italy|658[0n]
Lampides boeticus|WMB2952-14|RVcoll.12-M862|Mallorca|Spain|658[0n]
Lampides boeticus|EZSPN097-09|RVcoll.06-A033||Spain|658[0n]
Lampides boeticus|WMB3177-14|RVcoll.08-H585||Spain|658[0n]
Lampides boeticus|WMB2824-13|RVcoll.12-O353|Sardinia|Italy|658[0n]
Lampides boeticus|WMB3798-14|RVcoll.12-O614||France|658[0n]
Lampides boeticus|EZROM227-08|RV-07-E443||Romania|658[0n]
Lampides boeticus|WMB1748-13|RVcoll.12-P961|Levant|France|658[0n]
Lampides boeticus|OXB1190-15|Rvcoll_16-A077||Italy|658[0n]
Lampides boeticus|WMB2086-13|RVcoll.12-R029|Sicily|Italy|658[0n]
Lampides boeticus|EZSPM103-09|RVcoll.08-P435||Spain|658[0n]
Lampides boeticus|EZSPN623-09|RVcoll.08-J707||Spain|658[0n]
Lampides boeticus|OXB1166-15|Rvcoll_16-A053||Italy|658[0n]
Lampides boeticus|WMB2460-13|RVcoll.11-H311|Sicily|Italy|658[0n]
Lampides boeticus|WMB2731-13|RVcoll.10-C589||Malta|658[0n]
Lampides boeticus|WMB2758-13|RVcoll.11-H693|Levanzo|Italy|658[0n]
Lampides boeticus|WMB806-13|RVcoll.07-E058||Italy|658[0n]
Lampides boeticus|WMB1493-13|RVcoll.12-O022|Giglio|Italy|658[0n]
Lampides boeticus|WMB1497-13|RVcoll.12-O040||Italy|658[0n]
Lampides boeticus|WMB912-13|RVcoll.09-X901|Capri|Italy|658[0n]
Lampides boeticus|WMB1059-13|RVcoll.10-C679|Lipari|Italy|658[0n]
Lampides boeticus|WMB1554-13|RVcoll.12-O384|Sardinia|Italy|658[0n]
Lampides boeticus|WMB1559-13|RVcoll.12-O403|San Pietro|Italy|658[0n]
Lampides boeticus|WMB1588-13|RVcoll.12-O580|Sardinia|Italy|658[0n]
Lampides boeticus|WMB1672-13|RVcoll.12-P280||France|658[0n]
Lampides boeticus|WMB1732-13|RVcoll.12-P909||France|658[0n]
Lampides boeticus|WMB1749-13|RVcoll.12-P962|Levant|France|658[0n]
Lampides boeticus|WMB1879-13|RVcoll.11-J687|Sicily|Italy|658[0n]
Lampides boeticus|WMB2368-13|RVcoll.12-Q935||Italy|658[0n]
Lampides boeticus|WMB1919-13|RVcoll.11-H692|Levanzo|Italy|657[0n]
Lampides boeticus|WMB2005-13|RVcoll.12-Q393||France|658[0n]
Lampides boeticus|WMB2176-13|RVcoll.12-R344|Elba|Italy|658[0n]
Lampides boeticus|WMB2196-13|RVcoll.09-X944|Ponza|Italy|658[0n]
Lampides boeticus|WMB2197-13|RVcoll.09-X945|Ponza|Italy|658[0n]
Lampides boeticus|WMB2303-13|RVcoll.11-E133|Gozo|Malta|658[0n]
Lampides boeticus|WMB2323-13|RVcoll.11-H662|Pantelleria|Italy|658[0n]
Lampides boeticus|WMB2324-13|RVcoll.11-H663|Pantelleria|Italy|658[0n]
Lampides boeticus|WMB2405-13|RVcoll.09-X946|Ponza|Italy|658[0n]
Lampides boeticus|WMB5943-17|RVcoll11J740|Sicily|Italy|658[0n]
Lampides boeticus|WMB2595-13|RVcoll.12-R332|Elba|Italy|658[0n]
Lampides boeticus|WMB2626-13|RVcoll.12-R446|Corsica|France|658[0n]
Lampides boeticus|WMB2627-13|RVcoll.12-R447|Corsica|France|658[0n]
Lampides boeticus|WMB3894-14|RVcoll.12-O979||France|658[0n]
Lampides boeticus|WMB4507-14|RVcoll.14-E186||Italy|658[0n]
Lampides boeticus|WMB3923-14|RVcoll.12-P504||France|658[0n]
Lampides boeticus|WMB4331-14|RVcoll.14-A711||Italy|658[0n]
Lampides boeticus|WMB4395-14|RVcoll.14-B349||Portugal|658[0n]
Lampides boeticus|WMB4473-14|RVcoll.14-D908||Italy|658[0n]
Lampides boeticus|WMB4475-14|RVcoll.14-D913||Italy|658[0n]
Lampides boeticus|WMB4658-14|RVcoll.LD-2566|Lipari|Italy|658[0n]
Lampides boeticus|WMB4739-14|RVcoll.LD-3407|Montecristo|Italy|658[0n]
Lampides boeticus|WMB520-11|RVcoll.11-H612|Lampedusa|Italy|658[0n]
Lampides boeticus|WMB532-11|RVcoll.11-H665|Pantelleria|Italy|658[0n]
Lampides boeticus|WMB536-11|RVcoll.11-H691|Levanzo|Italy|658[0n]
Lampides boeticus|WMB4825-14|RVcoll. 14-I372||Italy|658[0n]
Lampides boeticus|WMB5340-14|RVcoll.14-J329|Pianosa|Italy|658[0n]
Lampides boeticus|WMB4973-14|RVcoll. 14-I520||Italy|658[0n]
Lampides boeticus|WMB5309-14|RVcoll.14-I679||France|658[0n]
Lampides boeticus|WMB5474-14|RVcoll.14-N693|Alicudi|Italy|658[0n]
Lampides boeticus|EULEP5142-17|RVcoll16J898||Serbia|658[0n]
Lampides boeticus|EULEP698-15|RVcoll.12-M137|Crete|Greece|658[0n]
Lampides boeticus|EULEP1313-15|RVcoll.14-F771||Greece|658[0n]
Lampides boeticus|EULEP878-15|RVcoll.14-C430||Greece|658[0n]
Lampides boeticus|EULEP1405-15|RVcoll.14-G068||Greece|658[0n]
Lampides boeticus|BIBSA920-15|16-A011||France|658[0n]
Lampides boeticus|BIBSA1278-15|12-O465|Sardinia|Italy|658[0n]
Lampides boeticus|BIBSA1304-15|11-E352|Sardinia|Italy|658[0n]
Lampides boeticus|BIBSA100-15|RVcoll.14-D522||Italy|658[0n]
Lampides boeticus|BIBSA1328-15|12-Q594|Sicily|Italy|658[0n]
Lampides boeticus|BIBSA078-14|LEP-SS-00078||Italy|658[0n]
Lampides boeticus|BIBSA1876-17|RVcoll16C777||Italy|658[0n]
Lampides boeticus|OXB860-15|15-A504||Italy|658[0n]
Lampides boeticus|OXB379-15|RVcoll.14-N093|Montecristo|Italy|658[0n]
Lampides boeticus|OXB380-15|RVcoll.14-N094|Montecristo|Italy|658[0n]
Lampides boeticus|OXB1554-16|OXB-TGS-1272||France|658[0n]
Lampides boeticus|OXB1251-15|Rvcoll_14-E294bis|Corsica|France|658[0n]
Lampides boeticus|OXB1167-15|Rvcoll_16-A054||Italy|658[0n]
Lampides boeticus|EULEP1508-15|RVcoll.14-G418||Greece|618[0n]
Lampides boeticus|WMB3930-14|RVcoll.12-P532||France|620[0n]
Lampides boeticus|OXB1574-16|OXB-TGS-1292||France|633[0n]
Lampides boeticus|WMB4431-14|RVcoll.14-B517||Portugal|615[0n]
Lampides boeticus|WMB5463-14|RVcoll.14-N643|Vulcano|Italy|634[0n]

Lampides boeticus|OXB1574-16|OXB-TGS-1292||France|633[0n]
Lampides boeticus|WMB4431-14|RVcoll.14-B517||Portugal|615[0n]
Lampides boeticus|WMB5463-14|RVcoll.14-N643|Vulcano|Italy|634[0n]
Lampides boeticus|WMB4670-14|RVcoll.LD-2623|Ischia|Italy|621[0n]
Lampides boeticus|WMB1916-13|RVcoll.11-H661|Pantelleria|Italy|637[0n]
Lampides boeticus|BIBSA1281-15|12-Q492|Sardinia|Italy|606[0n]
Lampides boeticus|EULEP5723-17|RVcoll16L196||Italy|658[0n]
Lampides boeticus|WMB1062-13|RVcoll.10-C683|Stromboli|Italy|658[0n]
Lampides boeticus|WMB2730-13|RVcoll.10-C588||Malta|658[0n]
Lampides boeticus|WMB1226-13|RVcoll.11-H286|Sicily|Italy|658[0n]
Lampides boeticus|WMB1141-13|RVcoll.11-E132|Gozo|Malta|658[0n]
Lampides boeticus|WMB3583-14|RVcoll.11-E065||Spain|658[0n]
Lampides boeticus|WMB3566-14|RVcoll.11-D969||Spain|658[0n]
Lampides boeticus|WMB3285-14|RVcoll.08-M681||Spain|658[0n]
Lampides boeticus|WMB3261-14|RVcoll.08-L727||Spain|658[0n]
Lampides boeticus|WMB3691-14|RVcoll.11-J246||Spain|658[0n]
Lampides boeticus|WMB3684-14|RVcoll.11-J226||Spain|658[0n]
Lampides boeticus|WMB3063-14|RVcoll.14-A793|Capri|Italy|658[0n]
Lampides boeticus|WMB2995-14|RVcoll.12-O402|San Pietro|Italy|658[0n]
Lampides boeticus|WMB2983-14|RVcoll.12-O229|Corsica|France|658[0n]
Lampides boeticus|WMB2963-14|RVcoll.12-O023|Giglio|Italy|658[0n]
Lampides boeticus|WMB2941-14|RVcoll.12-M369|Linosa|Italy|658[0n]
Lampides boeticus|WMB3381-14|RVcoll.09-V395||Spain|658[0n]
Lampides boeticus|WMB2869-14|RVcoll.10-C558|Argentario|Italy|658[0n]
Lampides boeticus|WMB2866-14|RVcoll.10-C550|Ibiza|Spain|658[0n]
Lampides boeticus|EZSPM900-12|Rvcoll.12-M622||Spain|658[0n]
Lampides boeticus|EZSPM037-09|RVcoll.08-P240||Spain|658[0n]
Lampides boeticus|EZSPM699-12|Rvcoll. 12-L601||Portugal|658[0n]
Lampides boeticus|EZSPM369-09|RVcoll.08-J839||Spain|658[0n]
Lampides boeticus|EZSPN598-09|RVcoll.08-J188||Portugal|658[0n]
Lampides boeticus|EZSPM1006-12|Rvcoll.12-L895||Spain|658[0n]
Lampides boeticus|EZSPN454-09|RVcoll.08-H659||Spain|658[0n]
Lampides boeticus|EZSPN700-09|RVcoll.08-L043||Spain|658[0n]
Lampides boeticus|EZROM673-08|RV-07-E424||Romania|658[0n]
Lampides boeticus|EZROM226-08|RV-07-E423||Romania|658[0n]
Lampides boeticus|EZSPC460-09|RVcoll.08-P005||Spain|658[0n]
Lampides boeticus|EZSPC459-09|RVcoll.08-M688||Spain|658[0n]
Lampides boeticus|GWORO989-09|BC ZSM Lep 32296||Italy|658[0n]
Lampides boeticus|GWORZ045-10|BC ZSM Lep 30401||Italy|658[0n]
Lampides boeticus|LEATG485-14|TLMF Lep 14272||Italy|658[0n]
Lycaena phlaeas|EULEP729-15|RVcoll.12-N818|Lesvos|Greece|658[0n]
Lycaena phlaeas|EZROM262-08|RV-07-D134||Romania|658[0n]
Lycaena phlaeas|WMB3014-14|RVcoll.12-R162|Sicily|Italy|658[0n]
Lycaena phlaeas|WMB2885-14|RVcoll.11-D032|Sicily|Italy|658[0n]
Lycaena phlaeas|WMB2767-13|RVcoll.11-H823|Vulcano|Italy|658[0n]
Lycaena phlaeas|WMB2804-13|RVcoll.12-M299|Lipari|Italy|658[0n]
Lycaena phlaeas|WMB1271-13|RVcoll.12-M297|Lipari|Italy|658[0n]
Lycaena phlaeas|WMB2774-13|RVcoll.11-H887|Salina|Italy|658[0n]
Lycaena phlaeas|WMB1936-13|RVcoll.11-H820|Vulcano|Italy|658[0n]
Lycaena phlaeas|WMB4632-14|RVcoll.LD-2387|Ustica|Italy|658[0n]
Lycaena phlaeas|WMB4633-14|RVcoll.LD-2388|Ustica|Italy|658[0n]
Lycaena phlaeas|WMB495-11|RVcoll.11-H543|Sicily|Italy|658[0n]
Lycaena phlaeas|WMB553-11|RVcoll.11-H746|Sicily|Italy|658[0n]
Lycaena phlaeas|WMB575-11|RVcoll.11-H826|Vulcano|Italy|658[0n]
Lycaena phlaeas|BIBSA1797-16|16-A551||Italy|658[0n]
Lycaena phlaeas|WMB587-11|RVcoll.11-H884|Salina|Italy|658[0n]
Lycaena phlaeas|BIBSA1780-16|16-A498||Italy|658[0n]
Lycaena phlaeas|EULEP2932-15|RVcoll.14-O065||Norway|658[0n]
Lycaena phlaeas|OXB682-15|12-M340|Sicily|Italy|658[0n]
Lycaena phlaeas|OXB1042-15|LD-2983||Italy|658[0n]
Lycaena phlaeas|WMB1946-13|RVcoll.11-H885|Salina|Italy|650[0n]
Lycaena phlaeas|WMB174-11|RVcoll.11-D435|Sicily|Italy|643[0n]
Lycaena phlaeas|WMB4024-14|RVcoll.12-Z113||Ireland|636[0n]
Lycaena phlaeas|WMB1270-13|RVcoll.12-M295|Lipari|Italy|636[0n]
Lycaena phlaeas|WMB162-11|RVcoll.11-D360|Sicily|Italy|641[0n]
Lycaena phlaeas|EULEP5675-17|RVcoll16J799|Ikaria|Greece|658[0n]
Lycaena phlaeas|EULEP1223-15|RVcoll.14-F526||Greece|658[0n]
Lycaena phlaeas|EULEP1583-15|RVcoll.14-G610||Greece|658[0n]
Lycaena phlaeas|EULEP937-15|RVcoll.14-C895||Bulgaria|658[0n]
Lycaena phlaeas|EULEP1421-15|RVcoll.14-G096||Greece|658[0n]
Lycaena phlaeas|EULEP1686-15|RVcoll.14-H004||Greece|658[0n]
Lycaena phlaeas|EULEP1716-15|RVcoll.14-H183||Greece|658[0n]
Lycaena phlaeas|BIBSA1185-15|15-M233||Italy|658[0n]
Lycaena phlaeas|OXB370-15|RVcoll.14-N084||Italy|658[0n]
Lycaena phlaeas|EZROM263-08|RV-07-D960||Romania|658[1n]
Lycaena phlaeas|EZROM261-08|RV-06-K658||Romania|658[0n]
Lycaena phlaeas|EZRMN054-08|RVcoll.08-M459||Romania|658[0n]
Lycaena phlaeas|EZRMN053-08|RVcoll.08-M400||Romania|658[0n]
Lycaena phlaeas|EZRMN051-08|RVcoll.08-M235||Romania|658[0n]
Lycaena phlaeas|EZRMN052-08|RVcoll.08-M351||Romania|658[0n]
Lycaena phlaeas|PHLAW039-13|TLMF Lep 09836||Austria|658[0n]
Lycaena phlaeas|EULEP4160-16|RVcoll15G035||France|658[0n]
Lycaena phlaeas|EZROM732-08|RV-06-H854||Spain|658[0n]
Lycaena phlaeas|WMB5504-14|RVcoll.14-J430|Montecristo|Italy|658[0n]
Lycaena phlaeas|BIBSA1312-15|12-O182|Corsica|France|658[0n]
Lycaena phlaeas|WMB349-11|RVcoll.11-E756||France|639[0n]
Lycaena phlaeas|WMB341-11|RVcoll.11-E693||France|658[0n]
Lycaena phlaeas|WMB2632-13|RVcoll.12-O090|Corsica|France|658[0n]
Lycaena phlaeas|WMB364-11|RVcoll.11-E971||France|658[0n]
Lycaena phlaeas|WMB2909-14|RVcoll.11-E877|Corsica|France|658[0n]
Lycaena phlaeas|OXB975-15|15-A925||Italy|658[0n]
Lycaena phlaeas|WMB5503-14|RVcoll.14-J429|Montecristo|Italy|658[0n]
Lycaena phlaeas|WMB2902-14|RVcoll.11-E737|Corsica|France|658[0n]
Lycaena phlaeas|WMB5076-14|RVcoll. 14-L207||Italy|658[0n]
Lycaena phlaeas|OXB237-15|OXB-TGS-512||United Kingdom|658[0n]
Lycaena phlaeas|OXB219-15|OXB-TGS-362||United Kingdom|658[0n]
Lycaena phlaeas|WMB4512-14|RVcoll.14-E202||Spain|658[0n]
Lycaena phlaeas|EULEP4884-16|RVcoll16H993||Denmark|649[0n]
Lycaena phlaeas|GWOSZ098-11|BC ZSM Lep 41854||Italy|658[0n]
Lycaena phlaeas|LEATG015-14|TLMF Lep 13802||Austria|658[0n]
Lycaena phlaeas|WMB3408-14|RVcoll.09-V920||Spain|658[0n]
Lycaena phlaeas|WMB2921-14|RVcoll.11-I364|Argentario|Italy|658[0n]
Lycaena phlaeas|BIBSA911-15|16-A002||France|658[0n]

[illegible]
Lycaena phlaeas|WMB3408-14|RVcoll.09-V920||Spain|658[0n]
Lycaena phlaeas|WMB2921-14|RVcoll.11-I364|Argentario|Italy|658[0n]
Lycaena phlaeas|BIBSA911-15|16-A002||France|658[0n]
Lycaena phlaeas|BIBSA1266-15|LD-2684||Italy|658[0n]
Lycaena phlaeas|WMB4409-14|RVcoll.14-B445||Portugal|658[0n]
Lycaena phlaeas|WMB4368-14|RVcoll.14-B100||Portugal|658[0n]
Lycaena phlaeas|WMB5052-14|RVcoll. 14-L183||Italy|658[0n]
Lycaena phlaeas|WMB4912-14|RVcoll.14-I459||Italy|658[0n]
Lycaena phlaeas|WMB5374-14|RVcoll.14-J776||France|658[0n]
Lycaena phlaeas|WMB4865-14|RVcoll.14-I412||Italy|658[0n]
Lycaena phlaeas|BIBSA1126-15|15-L926||Italy|658[0n]
Lycaena phlaeas|EULEP2159-15|RVcoll.14-K071||Switzerland|658[0n]
Lycaena phlaeas|EULEP2426-15|RVcoll.14-V211||Belgium|658[0n]
Lycaena phlaeas|EULEP2393-15|RVcoll.14-V096||Ukraine|658[0n]
Lycaena phlaeas|EULEP753-15|RVcoll.12-Q563||Sweden|658[0n]
Lycaena phlaeas|EULEP4987-16|RVcoll16I687||Poland|658[0n]
Lycaena phlaeas|BIBSA1630-16|15-C467||Italy|658[0n]
Lycaena phlaeas|BIBSA1586-16|12-M508||Italy|658[0n]
Lycaena phlaeas|LOWA777-06|2005-LOWA-777||Russia|658[0n]
Lycaena phlaeas|WMB5500-14|RVcoll.14-J410|Giglio|Italy|658[0n]
Lycaena phlaeas|WMB337-11|RVcoll.11-E676||Italy|658[0n]
Lycaena phlaeas|WMB320-11|RVcoll.11-E565||Italy|658[0n]
Lycaena phlaeas|WMB308-11|RVcoll.11-E367||Italy|658[0n]
Lycaena phlaeas|WMB4685-14|RVcoll.LD-2685||Italy|658[0n]
Lycaena phlaeas|WMB4683-14|RVcoll.LD-2675|Giglio|Italy|658[0n]
Lycaena phlaeas|WMB4673-14|RVcoll.LD-2630|Ischia|Italy|658[0n]
Lycaena phlaeas|WMB2645-13|RVcoll.12-R200||Italy|658[0n]
Lycaena phlaeas|WMB2326-13|RVcoll.11-H668|Pantelleria|Italy|658[0n]
Lycaena phlaeas|WMB2325-13|RVcoll.11-H667|Pantelleria|Italy|658[0n]
Lycaena phlaeas|WMB2241-13|RVcoll.12-R373|Elba|Italy|658[0n]
Lycaena phlaeas|WMB1993-13|RVcoll.11-I307|Pianosa|Italy|658[0n]
Lycaena phlaeas|WMB1992-13|RVcoll.11-I306|Pianosa|Italy|658[0n]
Lycaena phlaeas|WMB2366-13|RVcoll.12-Q927||Italy|658[0n]
Lycaena phlaeas|WMB2365-13|RVcoll.12-Q926||Italy|658[0n]
Lycaena phlaeas|WMB634-11|RVcoll.11-I125||Italy|658[0n]
Lycaena phlaeas|WMB5093-14|RVcoll. 14-L224||Italy|658[0n]
Lycaena phlaeas|WMB533-11|RVcoll.11-H666|Pantelleria|Italy|658[0n]
Lycaena phlaeas|WMB522-11|RVcoll.11-H615|Lampedusa|Italy|658[0n]
Lycaena phlaeas|WMB4227-14|RVcoll.13-T771||Italy|658[0n]
Lycaena phlaeas|WMB4139-14|RVcoll.13-S648||Italy|658[0n]
Lycaena phlaeas|WMB4669-14|RVcoll.LD-2620|Ischia|Italy|658[0n]
Lycaena phlaeas|WMB4416-14|RVcoll.14-B461||Portugal|658[0n]
Lycaena phlaeas|WMB4340-14|RVcoll.14-A731||Italy|658[0n]
Lycaena phlaeas|WMB3976-14|RVcoll.12-Q210||France|658[0n]
Lycaena phlaeas|WMB3899-14|RVcoll.12-P193||France|658[0n]
Lycaena phlaeas|WMB4232-14|RVcoll.13-T819||Italy|658[0n]
Lycaena phlaeas|WMB1684-13|RVcoll.12-P502||France|658[0n]
Lycaena phlaeas|WMB1614-13|RVcoll.12-O741||France|658[0n]
Lycaena phlaeas|WMB1557-13|RVcoll.12-O398|San Pietro|Italy|658[0n]
Lycaena phlaeas|WMB1538-13|RVcoll.12-O279|La Maddalena|Italy|658[0n]
Lycaena phlaeas|WMB1496-13|RVcoll.12-O039||Italy|658[0n]
Lycaena phlaeas|WMB3561-14|RVcoll.11-D915||Spain|658[0n]
Lycaena phlaeas|WMB2798-13|RVcoll.11-Y011|Elba|Italy|658[0n]
Lycaena phlaeas|WMB2785-13|RVcoll.11-I304|Pianosa|Italy|658[0n]
Lycaena phlaeas|WMB3489-14|RVcoll.10-B683||France|658[0n]
Lycaena phlaeas|WMB3685-14|RVcoll.11-J227||Spain|658[0n]
Lycaena phlaeas|WMB3102-14|RVcoll.06-A016||Spain|658[0n]
Lycaena phlaeas|WMB3006-14|RVcoll.12-O476|Sardinia|Italy|658[0n]
Lycaena phlaeas|WMB2953-14|RVcoll.12-M967|Ibiza|Spain|658[0n]
Lycaena phlaeas|WMB3424-14|RVcoll.09-X269||France|658[0n]
Lycaena phlaeas|WMB5510-14|RVcoll.14-M307||France|658[0n]
Lycaena phlaeas|WMB240-11|RVcoll.09-T593||Italy|658[0n]
Lycaena phlaeas|WMB138-11|RVcoll.11-D153|Pantelleria|Italy|658[0n]
Lycaena phlaeas|EZSPM930-12|Rvcoll.12-M660||Spain|658[0n]
Lycaena phlaeas|EZSPM071-09|RVcoll.08-P312||Spain|658[0n]
Lycaena phlaeas|EZSPM051-09|RVcoll.08-P269||Spain|658[0n]
Lycaena phlaeas|EZSPM046-09|RVcoll.08-P261||Spain|658[0n]
Lycaena phlaeas|EZSPM1038-12|Rvcoll.12-M789||Spain|658[0n]
Lycaena phlaeas|EZSPM709-12|Rvcoll. 12-L611||Portugal|658[0n]
Lycaena phlaeas|EZSPM708-12|Rvcoll. 12-L596||Portugal|658[0n]
Lycaena phlaeas|EZSPM707-12|Rvcoll. 12-L578||Portugal|658[0n]
Lycaena phlaeas|EZSPM706-12|Rvcoll. 12-L565||Portugal|658[0n]
Lycaena phlaeas|EZSPM379-09|RVcoll.08-J873||Spain|658[0n]
Lycaena phlaeas|EZSPN592-09|RVcoll.08-J175||Portugal|658[0n]
Lycaena phlaeas|EZSPM998-12|Rvcoll.12-L884||Spain|658[0n]
Lycaena phlaeas|EZSPM976-12|Rvcoll.12-M734||Spain|658[0n]
Lycaena phlaeas|EZSPN445-09|RVcoll.08-H631||Spain|658[0n]
Lycaena phlaeas|EZSPN375-09|RVcoll.08-H402||Spain|658[0n]
Lycaena phlaeas|EZSPN348-09|RVcoll.08-H314||Spain|658[0n]
Lycaena phlaeas|EZSPN321-09|RVcoll.08-H182||Spain|658[0n]
Lycaena phlaeas|EZSPN307-09|RVcoll.08-H113||Spain|658[0n]
Lycaena phlaeas|EZSPC503-09|RVcoll.08-M906||Spain|658[0n]
Lycaena phlaeas|EZSPC502-09|RVcoll.08-L437||Spain|658[0n]
Lycaena phlaeas|EZSPN688-09|RVcoll.08-L017||Spain|658[0n]
Lycaena phlaeas|EZSPN087-09|RVcoll.06-A011||Spain|658[0n]
Lycaena phlaeas|EZSPN723-09|RVcoll.08-L114||Spain|658[0n]
Lycaena phlaeas|EZROM260-08|RV-06-K651||Romania|658[0n]
Lycaena phlaeas|EZRMN050-08|RVcoll.06-M916||Romania|658[0n]
Lycaena phlaeas|LEFIJ494-10|MM17119||Finland|658[0n]
Lycaena phlaeas|LEFIC267-10|MM03698||Finland|658[0n]
Lycaena phlaeas|LEFIE915-10|MM10279||Finland|658[0n]
Lycaena phlaeas|FBLMV674-09|BC ZSM Lep 28654||Germany|658[0n]
Lycaena phlaeas|LON964-12|NHMO Lep2011.024||Norway|658[0n]
Lycaena phlaeas|LON387-08|NHMO-08035||Norway|657[0n]
Lycaena phlaeas|GWORO789-09|BC ZSM Lep 30481||Germany|658[0n]
Lycaena phlaeas|FBLMU448-09|BC ZSM Lep 27098||Germany|658[0n]
Lycaena phlaeas|BIBSA1262-15|15-F919||France|658[0n]
Lycaena phlaeas|BIBSA1183-15|15-M223||Italy|658[0n]
Lycaena phlaeas|BIBSA1323-15|12-Q476|Sardinia|Italy|658[0n]
Lycaena phlaeas|BIBSA1027-15|15-C175||Italy|658[0n]
Lycaena phlaeas|BIBSA578-15|LEP-SS-00198||Italy|658[0n]
Lycaena phlaeas|BIBSA577-15|LEP-SS-00197||Italy|658[0n]
[illegible]

Lycaena phlaeas|BIBSA1027-15|15-C175||Italy|658[0n]
Lycaena phlaeas|BIBSA578-15|LEP-SS-00198||Italy|658[0n]
Lycaena phlaeas|BIBSA577-15|LEP-SS-00197||Italy|658[0n]
Lycaena phlaeas|EULEP4163-16|RVcoll15J145||Switzerland|658[0n]
Lycaena phlaeas|EULEP4161-16|RVcoll15I371||Austria|658[0n]
Lycaena phlaeas|BIBSA333-15|RVcoll.14-I001||Italy|658[0n]
Lycaena phlaeas|BIBSA1418-15|15-N088||Italy|658[0n]
Lycaena phlaeas|BIBSA055-14|LEP-SS-00055||Italy|658[0n]
Lycaena phlaeas|BIBSA054-14|LEP-SS-00054||Italy|658[0n]
Lycaena phlaeas|EULEP4950-16|RVcoll16I174||Germany|658[0n]
Lycaena phlaeas|EULEP5065-16|RVcoll16J137||Czech Republic|658[0n]
Lycaena phlaeas|GBLAA019-14|BC ZSM Lep 80680||Germany|658[0n]
Lycaena phlaeas|LEASS525-17|TLMF Lep 22173||Italy|658[0n]
Lycaena phlaeas|OXB1061-15|13-T791||Italy|658[0n]
Lycaena phlaeas|OXB224-15|OXB-TGS-422||United Kingdom|658[0n]
Lycaena phlaeas|WMB4424-14|RVcoll.14-B479||Portugal|658[0n]
Lycaena phlaeas|OXB1328-15|Rvcoll_15-M600||France|658[0n]
Lycaena phlaeas|OXB1148-15|Rvcoll_15-N102||Italy|658[0n]
Lycaena phlaeas|OXB1170-15|Rvcoll_16-A057||Italy|658[0n]
Lycaena phlaeas|OXB1243-15|Rvcoll_13-T690|Sardinia|Italy|658[0n]
Lycaena phlaeas|OXB1412-15|Rvcoll_15-M787||France|658[0n]
Lycaena phlaeas|EZSPC501-09|RVcoll.08-H234||Spain|647[0n]
Lycaena phlaeas|GWORZ041-10|BC ZSM Lep 30397||Italy|615[0n]
Lycaena phlaeas|LEFIJ4753-16|ZMBSU-02130||Belarus|613[0n]
Lycaena phlaeas|OXB1527-16|OXB-TGS-1245||United Kingdom|638[0n]
Lycaena phlaeas|WMB3928-14|RVcoll.12-P527||France|620[0n]
Lycaena phlaeas|EULEP4162-16|RVcoll15I473||Austria|624[0n]
Lycaena phlaeas|EULEP2018-15|RVcoll.14-I848||Poland|617[0n]
Lycaena phlaeas|GBLAA378-14|BC ZSM Lep 80374||Germany|624[0n]
Lycaena phlaeas|WMB2175-13|RVcoll.12-R343|Elba|Italy|605[0n]
Lycaena phlaeas|WMB2936-14|RVcoll.12-L510|Ibiza|Spain|623[0n]
Lycaena phlaeas|EZSPC500-09|RVcoll.07-C406||Spain|627[0n]
Lycaena phlaeas|PHLAI507-13|TLMF Lep 09069||Austria|624[0n]
Lycaena phlaeas|LEATF454-14|TLMF Lep 13766||Italy|658[0n]
Lycaena phlaeas|LEATH467-14|TLMF Lep 15679||Italy|658[0n]
Lycaena phlaeas|LEATG479-14|TLMF Lep 14266||Italy|658[0n]
Lycaena phlaeas|LEATG480-14|TLMF Lep 14267||Italy|658[0n]
Lycaena phlaeas|ABOLD001-16|TLMF Lep 21079||Austria|658[0n]
Lycaena phlaeas|LEATH466-14|TLMF Lep 15678||Italy|658[0n]
Lycaena dispar|EULEP763-15|RVcoll.12-R745||Romania|658[0n]
Lycaena dispar|EULEP4890-16|RVcoll16J584||Moldova|658[0n]
Lycaena dispar|EZRMN044-08|RVcoll.08-M210||Romania|658[0n]
Lycaena dispar|EZRMN041-08|RVcoll.07-D006||Romania|658[0n]
Lycaena dispar|EZROM254-08|RV-07-D099||Romania|656[0n]
Lycaena dispar|LEASS508-17|TLMF Lep 22156||Austria|658[0n]
Lycaena dispar|EULEP1025-15|RVcoll.14-E821||Serbia|614[0n]
Lycaena dispar|LEFIL041-10|MM19041||Russia|658[0n]
Lycaena dispar|LEFIL042-10|MM19042||Russia|658[0n]
Lycaena dispar|ABOLD603-17|TLMF Lep 21679||Austria|658[0n]
Lycaena dispar|LEFIJ310-10|MM15910||Finland|658[0n]
Lycaena dispar|EZROM635-08|RV-07-C935||Romania|658[0n]
Lycaena dispar|EZROM253-08|RV-06-M827||Romania|658[0n]
Lycaena dispar|EZROM255-08|RV-07-D194||Romania|658[0n]
Lycaena dispar|EZRMN042-08|RVcoll.07-D269||Romania|658[0n]
Lycaena dispar|EZRMN043-08|RVcoll.07-D555||Romania|658[0n]
Lycaena dispar|EZRMN045-08|RVcoll.08-M372||Romania|658[0n]
Lycaena dispar|WMB5101-14|RVcoll. 14-L232||Italy|658[0n]
Lycaena dispar|EULEP5054-16|RVcoll16J093||Czech Republic|658[0n]
Lycaena dispar|EULEP4903-16|RVcoll16J606||Russia|658[0n]
Lycaena dispar|EULEP4969-16|RVcoll16I395||Poland|658[0n]
Lycaena dispar|EULEP336-14|MM23821||Estonia|658[0n]
Lycaena dispar|EULEP2415-15|RVcoll.14-V190||Belgium|658[0n]
Lycaena dispar|EULEP2531-15|RVcoll.14-V410||Ukraine|658[0n]
Lycaena dispar|EULEP2373-15|RVcoll.14-V068||Ukraine|658[0n]
Lycaena dispar|EULEP2935-15|RVcoll.14-O068||France|658[0n]
Lycaena dispar|BIBSA457-15|RVcoll.14-I125||Italy|658[0n]
Lycaena dispar|LEASS1036-17|TLMF Lep 22589||Austria|658[0n]
Lycaena dispar|LEFIJ4754-16|ZMBSU-02132||Belarus|658[0n]
Lycaena dispar|LEASS910-17|KLM Lep 08415||Austria|658[0n]
Lycaena dispar|LEASS681-17|TLMF Lep 22329||Austria|658[0n]
Lycaena dispar|LEASS686-17|TLMF Lep 22334||Austria|658[0n]
Lycaena dispar|OXB1111-15|15-A644||Italy|658[0n]
Lycaena dispar|EULEP323-14|MM23808||Latvia|618[0n]
Lycaena dispar|FBLMZ145-12|BC ZSM Lep 51401||Germany|627[0n]
Lycaena dispar|LEFIL372-10|MM18682||Finland|658[0n]
Lycaena dispar|GWOTF697-12|BC ZSM Lep 62403||Croatia|658[0n]
Lycaena dispar|ABOLD052-16|TLMF Lep 21130||Austria|658[0n]
Lycaena helle|EULEP473-14|KN00835||Russia|658[0n]
Lycaena helle|EULEP472-14|KN00834||Russia|658[0n]
Lycaena helle|EULEP471-14|KN00833||Russia|658[0n]
Lycaena helle|EZSPN193-09|RVcoll.07-C487||France|658[0n]
Lycaena helle|EULEP377-14|MM23862||Finland|658[0n]
Lycaena helle|EULEP4750-16|RVcoll16G829||Sweden|658[0n]
Lycaena helle|EZSPN056-09|RVcoll.09-V313||France|658[0n]
Lycaena helle|EZSPN055-09|RVcoll.09-V312||France|658[0n]
Lycaena helle|EZSPN054-09|RVcoll.09-V311||France|658[0n]
Lycaena helle|EZSPN053-09|RVcoll.09-V310||France|658[0n]
Lycaena helle|EZSPN052-09|RVcoll.09-V309||France|658[0n]
Lycaena helle|EULEP138-14|RVcoll.10-C403||Romania|658[0n]
Lycaena helle|EULEP137-14|RVcoll.10-C402||Romania|658[0n]
Lycaena helle|EULEP4863-16|RVcoll16H861||Poland|632[0n]
Lycaena helle|EZROM256-08|RV-07-D251||Romania|658[0n]
Lycaena helle|EULEP4147-16|RVcoll15P068||Serbia|658[0n]
Lycaena helle|EULEP4148-16|RVcoll15P030||Ukraine|658[0n]
Lycaena helle|EULEP4149-16|RVcoll14A981||Bulgaria|658[0n]
Lycaena helle|EULEP378-14|MM23863||Finland|658[0n]
Lycaena helle|EULEP5647-17|RVcoll15F999||Spain|658[0n]
Lycaena helle|EULEP2199-15|RVcoll.14-N359||Ukraine|658[0n]
Lycaena helle|EZRMN048-08|RVcoll.07-D305||Romania|658[1n]
Lycaena helle|EZROM257-08|RV-07-D253||Romania|658[0n]
Lycaena helle|EZRMN047-08|RVcoll.07-D299||Romania|658[0n]
Lycaena helle|EZROM674-08|RV-07-D284||Romania|658[0n]
Lycaena helle|EZRMN046-08|RVcoll.07-D262||Romania|658[0n]

Lycaena helle|EZRMN047-08|RVcoll.07-D299||Romania|658[0n]
Lycaena helle|EZROM674-08|RV-07-D284||Romania|658[0n]
Lycaena helle|EZRMN046-08|RVcoll.07-D262||Romania|658[0n]
Lycaena helle|LEFIF122-10|MM10578||Finland|658[0n]
Lycaena helle|LEFIF121-10|MM10577||Finland|658[0n]
Lycaena helle|GWORO790-09|BC ZSM Lep 30482||Germany|658[0n]
Lycaena helle|GWORO761-09|BC ZSM Lep 30453||Germany|658[0n]
Lycaena helle|ABOLD611-17|TLMF Lep 21687||Austria|658[0n]
Lycaena helle|LEATJ1261-16|TLMF Lep 19584||Austria|658[0n]
Lycaena thersamon|OXB937-15|15-A581||Italy|658[0n]
Lycaena thersamon|BIBSA477-15|LEP-SS-00097||Italy|658[0n]
Lycaena thersamon|BIBSA476-15|LEP-SS-00096||Italy|658[0n]
Lycaena thersamon|WMB5280-14|RVcoll.14-A694||Italy|658[0n]
Lycaena thersamon|WMB1872-13|RVcoll.11-J606||Italy|658[0n]
Lycaena thersamon|WMB1871-13|RVcoll.11-J605||Italy|658[0n]
Lycaena thersamon|EULEP4878-16|RVcoll16H971||Cyprus|658[0n]
Lycaena thersamon|EULEP5140-17|RVcoll16J896||Serbia|658[0n]
Lycaena thersamon|EULEP2530-15|RVcoll.14-V409||Ukraine|658[0n]
Lycaena thersamon|EULEP2195-15|RVcoll.14-N339||Ukraine|658[0n]
Lycaena thersamon|EULEP4829-16|RVcoll16H729||Ukraine|658[0n]
Lycaena thersamon|EZROM264-08|RV-06-K665||Romania|658[1n]
Lycaena thersamon|EULEP768-15|RVcoll.12-R785||Romania|658[0n]
Lycaena thersamon|EZRMN055-08|RVcoll.07-C143||Romania|658[0n]
Lycaena thersamon|EULEP4847-16|RVcoll16H756||Bulgaria|658[0n]
Lycaena thersamon|EZROM266-08|RV-07-D007||Romania|658[0n]
Lycaena thersamon|EZROM265-08|RV-07-E468||Romania|658[0n]
Lycaena thersamon|EZRMN057-08|RVcoll.08-M396||Romania|658[0n]
Lycaena thersamon|EZRMN056-08|RVcoll.07-E445||Romania|658[0n]
Lycaena thersamon|EZROM676-08|RV-07-E444||Romania|658[0n]
Lycaena bleusei|EZSPN857-09|RVcoll.08-L717||Spain|658[1n]
Lycaena bleusei|EZSPC732-10|RVcoll.08-H900||Spain|658[0n]
Lycaena bleusei|EZSPC803-10|RVcoll.08-P432||Spain|658[0n]
Lycaena bleusei|WMB4382-14|RVcoll.14-B236||Portugal|658[0n]
Lycaena bleusei|EZSPC735-10|RVcoll.08-H937||Spain|658[0n]
Lycaena bleusei|EZSPC920-10|RVcoll.09-V898||Spain|658[0n]
Lycaena bleusei|EZSPC770-10|RVcoll.08-L718||Spain|658[0n]
Lycaena bleusei|EZSPM101-09|RVcoll.08-P433||Spain|658[0n]
Lycaena bleusei|EZSPN495-09|RVcoll.08-H938||Spain|658[0n]
Lycaena bleusei|EZSPN351-09|RVcoll.08-H322||Spain|658[0n]
Lycaena bleusei|EZSPN830-09|RVcoll.08-L646||Spain|658[0n]
Lycaena alciphron|EULEP4895-16|RVcoll16J598||Russia|658[0n]
Lycaena alciphron|EULEP4145-16|RVcoll15P009||Belarus|658[0n]
Lycaena alciphron|EZRMN039-08|RVcoll.08-M271||Romania|658[0n]
Lycaena alciphron|EULEP1890-15|RVcoll.14-B703||Albania|658[0n]
Lycaena alciphron|EULEP1524-15|RVcoll.14-G455||Greece|658[0n]
Lycaena alciphron|EULEP1281-15|RVcoll.14-F690||Greece|658[0n]
Lycaena alciphron|EULEP4141-16|RVcoll10B348||Bulgaria|658[0n]
Lycaena alciphron|EULEP1045-15|RVcoll.14-E912||Serbia|658[0n]
Lycaena alciphron|EZROM251-08|RV-07-D918||Romania|658[0n]
Lycaena alciphron|EZRMN040-08|RVcoll.08-M531||Romania|658[0n]
Lycaena alciphron|GWOSI556-10|BC ZSM Lep 44402||Germany|658[0n]
Lycaena alciphron|EULEP582-15|RVcoll.10-A690||Estonia|658[0n]
Lycaena alciphron|LEASS1038-17|TLMF Lep 22591||Austria|658[0n]
Lycaena alciphron|EULEP2365-15|RVcoll.14-V058||Ukraine|658[0n]
Lycaena alciphron|EULEP319-14|MM23804||Latvia|658[0n]
Lycaena alciphron|EULEP4144-16|RVcoll15Q082||Russia|658[0n]
Lycaena alciphron|EZROM634-08|RV-07-D536||Romania|658[0n]
Lycaena alciphron|GWOTF696-12|BC ZSM Lep 62402||Croatia|658[0n]
Lycaena alciphron|LEASS839-17|TLMF Lep 22487||Austria|658[0n]
Lycaena alciphron|EULEP4142-16|RVcoll15G034||France|658[0n]
Lycaena alciphron|FBLMZ173-12|BC ZSM Lep 51429||Germany|658[0n]
Lycaena alciphron|LEASS772-17|TLMF Lep 22420||Austria|658[0n]
Lycaena alciphron|LEASS771-17|TLMF Lep 22419||Austria|658[0n]
Lycaena alciphron|EZRMN038-08|RVcoll.07-D358||Romania|658[0n]
Lycaena alciphron|EZRMN037-08|RVcoll.07-C922||Romania|658[0n]
Lycaena alciphron|EZROM252-08|RV-07-C958||Romania|657[0n]
Lycaena alciphron|EZROM250-08|RV-06-M840||Romania|658[0n]
Lycaena alciphron|ABOLD002-16|TLMF Lep 21080||Austria|658[0n]
Lycaena alciphron|EZSPN731-09|RVcoll.08-L139||Spain|658[0n]
Lycaena alciphron|EZSPN542-09|RVcoll.08-J037||Spain|658[0n]
Lycaena alciphron|EZSPN664-09|RVcoll.08-J967||Spain|658[0n]
Lycaena alciphron|EZSPC493-09|RVcoll.08-R191||Spain|658[0n]
Lycaena alciphron|EZSPC1355-10|RVcoll.09-V901||Spain|658[0n]
Lycaena alciphron|EZSPC492-09|RVcoll.08-R147||Spain|658[0n]
Lycaena alciphron|WMB1608-13|RVcoll.12-O687||France|658[0n]
Lycaena alciphron|WMB932-13|RVcoll.10-A520||France|658[0n]
Lycaena alciphron|EZSPC1084-10|RVcoll.08-L738||Spain|658[0n]
Lycaena alciphron|EZSPC1078-10|RVcoll.08-L638||Spain|658[0n]
Lycaena alciphron|EZROM731-08|RV-07-C073||Spain|658[0n]
Lycaena alciphron|EZSPM430-09|RVcoll.09-X503||Spain|633[0n]
Lycaena alciphron|EZSPN856-09|RVcoll.08-L712||Spain|629[0n]
Lycaena alciphron|EZSPC494-09|RVcoll.08-R190||Spain|658[0n]
Lycaena alciphron|WMB250-11|RVcoll.09-V248||France|658[0n]
Lycaena alciphron|EZSPN1020-09|RVcoll.08-M952||Spain|658[0n]
Lycaena alciphron|WMB3140-14|RVcoll.07-C017||France|658[0n]
Lycaena alciphron|EZSPC495-09|RVcoll.08-P031||Spain|635[0n]
Lycaena alciphron|OXB1041-15|LD-2589||Italy|658[0n]
Lycaena alciphron|WMB619-11|RVcoll.11-I047|Sicily|Italy|658[0n]
Lycaena alciphron|WMB590-11|RVcoll.11-H919|Sicily|Italy|658[0n]
Lycaena alciphron|WMB1954-13|RVcoll.11-I055|Sicily|Italy|658[0n]
Lycaena alciphron|WMB2918-14|RVcoll.11-I050|Sicily|Italy|658[0n]
Lycaena alciphron|EULEP510-15|RVcoll.07-C809||Switzerland|658[0n]
Lycaena alciphron|BIBSA235-15|RVcoll.14-E027||Italy|640[0n]
Lycaena alciphron|EULEP4143-16|RVcoll15G590||Switzerland|658[0n]
Lycaena alciphron|WMB896-13|RVcoll.09-X837||Italy|658[0n]
Lycaena alciphron|WMB4951-14|RVcoll. 14-I498||Italy|658[0n]
Lycaena alciphron|WMB4896-14|RVcoll.14-I443||Italy|658[0n]
Lycaena alciphron|BIBSA1672-16|15-C805||Italy|658[0n]
Lycaena alciphron|WMB4282-14|RVcoll.14-A330||Italy|658[0n]
Lycaena alciphron|WMB5141-14|RVcoll.07-E177||Italy|658[0n]
Lycaena alciphron|BIBSA1350-15|15-M849||Italy|658[0n]
Lycaena alciphron|BIBSA1347-15|15-M843||Italy|658[0n]
[illegible]

[illegible]
Lycaena alciphron|BIBSA1350-15|15-M849||Italy|658[0n]
Lycaena alciphron|BIBSA1347-15|15-M843||Italy|658[0n]
Lycaena alciphron|WMB2282-13|RVcoll.09-T580||Italy|658[0n]
Lycaena alciphron|LEATD155-13|TLMF Lep 12802||Italy|658[0n]
Lycaena alciphron|ABOLA863-15|TLMF Lep 16823||Austria|658[0n]
Lycaena alciphron|ABOLA862-15|TLMF Lep 16822||Austria|658[0n]
Lycaena alciphron|LEATD154-13|TLMF Lep 12801||Italy|658[0n]
Lycaena alciphron|BIBSA1018-15|15-C151||Italy|658[0n]
Lycaena alciphron|BIBSA061-14|LEP-SS-00061||Italy|658[0n]
Lycaena alciphron|BIBSA062-14|LEP-SS-00062||Italy|658[0n]
Lycaena alciphron|LEASS730-17|TLMF Lep 22378||Austria|658[0n]
Lycaena alciphron|LEASS731-17|TLMF Lep 22379||Austria|658[0n]
Lycaena alciphron|BIBSA229-15|RVcoll.14-E020||Italy|633[0n]
Lycaena alciphron|BIBSA1731-16|LD-2553||Italy|615[0n]
Lycaena alciphron|BCLEP110-17|LEP-SS-00561||Italy|658[0n]
Lycaena alciphron|BIBSA1763-16|LD-2554||Italy|658[0n]
Lycaena alciphron|BIBSA1166-15|15-M186||Italy|658[0n]
Lycaena alciphron|WMB4157-14|RVcoll.13-S714||Italy|658[0n]
Lycaena alciphron|WMB2040-13|RVcoll.12-Q761||Italy|658[0n]
Lycaena alciphron|WMB2027-13|RVcoll.12-Q705||Italy|658[0n]
Lycaena alciphron|WMB1963-13|RVcoll.11-I112||Italy|658[0n]
Lycaena alciphron|PHLSA428-11|TLMF Lep 05883||Italy|658[0n]
Lycaena dimorpha|EULEP429-14|KN00791||Russia|658[0n]
Lycaena thetis|EULEP2937-15|RVcoll.14-O070||Greece|658[0n]
Lycaena thetis|EULEP2936-15|RVcoll.14-O069||Greece|658[0n]
Lycaena thetis|EULEP1416-15|RVcoll.14-G086||Greece|658[0n]
Lycaena thetis|EULEP4166-16|RVcoll15Q056||Greece|658[0n]
Lycaena thetis|EULEP2939-15|RVcoll.14-O072||Greece|658[0n]
Lycaena thetis|EULEP2938-15|RVcoll.14-O071||Greece|658[0n]
Lycaena thetis|EULEP4165-16|RVcoll15Q055||Greece|658[0n]
Lycaena hippothoe|EULEP4154-16|RVcoll15I347||Austria|658[0n]
Lycaena hippothoe|BIBSA985-15|14-W533||Italy|620[0n]
Lycaena hippothoe|BIBSA232-15|RVcoll.14-E023||Italy|642[0n]
Lycaena hippothoe|EULEP4152-16|RVcoll15H432||Switzerland|658[0n]
Lycaena hippothoe|EULEP637-15|RVcoll.11-J104||Switzerland|658[0n]
Lycaena hippothoe|EULEP4158-16|RVcoll15G386||Switzerland|658[0n]
Lycaena hippothoe|EULEP4150-16|RVcoll15H002||Switzerland|658[0n]
Lycaena hippothoe|BIBSA400-15|RVcoll.14-I068||Italy|658[0n]
Lycaena hippothoe|BIBSA403-15|RVcoll.14-I071||Italy|658[0n]
Lycaena hippothoe|WMB1638-13|RVcoll.12-O867||France|658[0n]
Lycaena hippothoe|WMB2001-13|RVcoll.12-Q387||France|658[0n]
Lycaena hippothoe|EULEP4155-16|RVcoll15J507||France|658[0n]
Lycaena hippothoe|BIBSA402-15|RVcoll.14-I070||Italy|658[0n]
Lycaena hippothoe|BIBSA406-15|RVcoll.14-I074||Italy|658[0n]
Lycaena hippothoe|BIBSA233-15|RVcoll.14-E024||Italy|658[0n]
Lycaena hippothoe|BIBSA325-15|RVcoll.14-E128||Italy|632[0n]
Lycaena hippothoe|BIBSA231-15|RVcoll.14-E022||Italy|638[0n]
Lycaena hippothoe|WMB2700-13|RVcoll.10-C077||France|627[0n]
Lycaena hippothoe|WMB2675-13|RVcoll.10-B870||France|658[0n]
Lycaena hippothoe|PHLAF620-11|TLMF Lep 05790||France|658[0n]
Lycaena hippothoe|WMB4934-14|RVcoll.14-I481||Italy|658[0n]
Lycaena hippothoe|WMB5255-14|RVcoll.14-A252||Italy|658[0n]
Lycaena hippothoe|EULEP814-15|RVcoll.14-A253||Italy|658[0n]
Lycaena hippothoe|PHLSA439-11|TLMF Lep 05894||Italy|633[0n]
Lycaena hippothoe|LEASS523-17|TLMF Lep 22171||Austria|658[0n]
Lycaena hippothoe|LEATJ1163-16|TLMF Lep 19486||Czech Republic|658[0n]
Lycaena hippothoe|EULEP1997-15|RVcoll.14-I770||Slovakia|658[0n]
Lycaena hippothoe|EULEP5010-16|RVcoll16I889||Slovakia|658[0n]
Lycaena hippothoe|EULEP5052-16|RVcoll16J079||Czech Republic|658[0n]
Lycaena hippothoe|ABOLD605-17|TLMF Lep 21681||Austria|658[0n]
Lycaena hippothoe|GWOSU070-11|BC ZSM Lep 53321||Germany|658[0n]
Lycaena hippothoe|GWOTF695-12|BC ZSM Lep 62401||Croatia|658[0n]
Lycaena hippothoe|LEASS911-17|KLM Lep 08416||Austria|658[0n]
Lycaena hippothoe|EULEP4153-16|RVcoll15I024||Italy|658[0n]
Lycaena hippothoe|GBLAB777-13|BC ZSM Lep 75548||Germany|658[0n]
Lycaena hippothoe|ODOPE750-11|BC ZSM Lep 50391||Germany|658[0n]
Lycaena hippothoe|EZSPM418-09|Rvcoll.09-V841||Spain|658[0n]
Lycaena hippothoe|EZSPC1353-10|RVcoll.09-V882||Spain|658[0n]
Lycaena hippothoe|EZSPC499-09|RVcoll.08-M910||Spain|658[0n]
Lycaena hippothoe|EULEP4151-16|RVcoll15H183||Switzerland|658[0n]
Lycaena hippothoe|EULEP4157-16|RVcoll15G175||France|658[0n]
Lycaena hippothoe|EULEP220-14|RVcoll.12-L051||Spain|658[0n]
Lycaena hippothoe|EULEP2122-15|RVcoll.14-J895|Genit. examined|France|658[0n]
Lycaena hippothoe|EULEP4911-16|RVcoll16J614||Russia|627[0n]
Lycaena hippothoe|EULEP4156-16|RVcoll15G099||France|624[0n]
Lycaena hippothoe|EZSPC498-09|RVcoll.07-W214||Spain|634[0n]
Lycaena hippothoe|EZSPC1352-10|RVcoll.09-V881||Spain|630[0n]
Lycaena hippothoe|EZSPC496-09|RVcoll.07-C633||Spain|622[0n]
Lycaena hippothoe|EZSPC497-09|RVcoll.07-C665||Spain|658[0n]
Lycaena hippothoe|EZSPC655-09|RVcoll.07-C662||France|658[0n]
Lycaena hippothoe|GWOSK799-11|BC ZSM Lep 49015||Germany|658[0n]
Lycaena hippothoe|LON501-08|NHMO-08153||Norway|657[0n]
Lycaena hippothoe|GWORO788-09|BC ZSM Lep 30480||Germany|658[0n]
Lycaena hippothoe|PHLAW031-13|TLMF Lep 09828||Austria|658[0n]
Lycaena candens|EULEP2934-15|RVcoll.14-O067||Albania|658[0n]
Lycaena candens|EULEP906-15|RVcoll.14-C749|Genit. examined|Bulgaria|658[0n]
Lycaena candens|EULEP552-15|RVcoll.08-L368||Greece|658[0n]
Lycaena candens|EULEP1893-15|RVcoll.14-B710||Albania|658[0n]
Lycaena candens|EULEP936-15|RVcoll.14-C894||Bulgaria|658[0n]
Lycaena candens|EULEP4146-16|RVcoll14O335||Bulgaria|658[0n]
Lycaena candens|EULEP581-15|RVcoll.10-A397|Genit. examined|Bulgaria|658[0n]
Lycaena candens|EULEP1146-15|RVcoll.14-F317|Genit. examined|Serbia|658[0n]
Lycaena candens|EULEP1089-15|RVcoll.14-F049|Genit. examined|Serbia|658[0n]
Lycaena hippothoe|EULEP1052-15|RVcoll.14-E925|Genit. examined|Serbia|658[0n]
Lycaena hippothoe|EULEP544-15|RVcoll.08-L302||Denmark|658[0n]
Lycaena hippothoe|EZSPC1122-10|RVcoll.08-P479||Spain|658[0n]
Lycaena hippothoe|EZSPM312-09|RVcoll.08-J866||Spain|658[0n]
Lycaena hippothoe|EZSPN886-09|RVcoll.08-L796||Spain|655[0n]
Lycaena hippothoe|EZSPN885-09|RVcoll.08-L795||Spain|658[0n]
Lycaena hippothoe|EULEP151-14|RVcoll.10-C491||Romania|658[0n]
Lycaena hippothoe|EULEP4861-16|RVcoll16H853||Poland|658[0n]
Lycaena hippothoe|EULEP4720-16|RVcoll16B364||Norway|658[0n]

Lycaena hippothoe|EULEP151-14|RVcoll.10-C491||Romania|658[0n]
Lycaena hippothoe|EULEP4861-16|RVcoll16H853||Poland|658[0n]
Lycaena hippothoe|EULEP4720-16|RVcoll16B364||Norway|658[0n]
Lycaena hippothoe|EULEP4714-16|RVcoll16B315||Sweden|658[0n]
Lycaena hippothoe|EZROM259-08|RV-06-N003||Romania|658[0n]
Lycaena hippothoe|EZROM258-08|RV-06-M977||Romania|658[0n]
Lycaena hippothoe|EZRMN049-08|RVcoll.06-N020||Romania|658[0n]
Lycaena hippothoe|EZROM675-08|RV-06-N011||Romania|658[0n]
Lycaena hippothoe|LEFIJ498-10|MM17123||Finland|658[0n]
Lycaena hippothoe|LEFIJ497-10|MM17122||Finland|658[0n]
Lycaena hippothoe|EZRMN330-08|RVcoll.08-M783||Romania|658[0n]
Lycaena hippothoe|LEFID354-10|MM06257||Finland|658[0n]
Lycaena hippothoe|LEATD456-13|TLMF Lep 13103||Italy|658[0n]
Lycaena hippothoe|LEATH741-14|TLMF Lep 15953||Italy|634[0n]
Lycaena hippothoe|LEATG395-14|TLMF Lep 14182||Italy|610[0n]
Lycaena hippothoe|LEATG097-14|TLMF Lep 13884||Austria|658[0n]
Lycaena hippothoe|PHLAF636-11|TLMF Lep 05806||Switzerland|658[0n]
Lycaena hippothoe|PHLAW032-13|TLMF Lep 09829||Austria|658[0n]
Lycaena hippothoe|WMB5115-14|RVcoll. 14-L246||Italy|658[0n]
Lycaena hippothoe|EULEP2445-15|RVcoll.14-V255||Switzerland|658[0n]
Lycaena hippothoe|PHLAI504-13|TLMF Lep 09066||Austria|658[0n]
Lycaena virgaureae|OXB1299-15|Rvcoll_15-M166||France|658[0n]
Lycaena virgaureae|WMB3669-14|RVcoll.11-I957||France|658[0n]
Lycaena virgaureae|WMB2842-13|RVcoll.12-Q683||Italy|658[0n]
Lycaena virgaureae|WMB809-13|RVcoll.07-E094||Italy|632[0n]
Lycaena virgaureae|WMB2014-13|RVcoll.12-Q655||Italy|658[0n]
Lycaena virgaureae|WMB5249-14|RVcoll.14-A223||Italy|639[0n]
Lycaena virgaureae|WMB2020-13|RVcoll.12-Q681||Italy|658[0n]
Lycaena virgaureae|EZSPC512-09|RVcoll.08-R117||Spain|658[0n]
Lycaena virgaureae|EZSPC513-09|RVcoll.08-P006||Spain|658[0n]
Lycaena virgaureae|EZSPC510-09|RVcoll.08-M921||Spain|658[0n]
Lycaena virgaureae|EZSPC1239-10|RVcoll.09-V750||Spain|658[0n]
Lycaena virgaureae|EZSPC511-09|RVcoll.08-P386||Spain|647[0n]
Lycaena virgaureae|EZSPC509-09|RVcoll.08-R197||Spain|622[0n]
Lycaena virgaureae|GWORO760-09|BC ZSM Lep 30452||Germany|658[0n]
Lycaena virgaureae|EZSPC1145-10|RVcoll.08-P762||Spain|658[0n]
Lycaena virgaureae|EZSPC1100-10|RVcoll.08-L969||Spain|658[0n]
Lycaena virgaureae|EZSPN962-09|RVcoll.08-L968||Spain|658[0n]
Lycaena virgaureae|EZSPN947-09|RVcoll.08-L936||Spain|658[0n]
Lycaena virgaureae|EZSPM301-09|RVcoll.08-J848||Spain|658[0n]
Lycaena virgaureae|EZSPM083-09|RVcoll.08-P403||Spain|658[0n]
Lycaena virgaureae|EZSPM849-12|Rvcoll. 150511SD71||Spain|658[0n]
Lycaena virgaureae|LON882-11|NHMO Lep09080||Norway|658[0n]
Lycaena virgaureae|LEFIJ496-10|MM17121||Finland|658[0n]
Lycaena virgaureae|EZROM272-08|RV-07-D518||Romania|658[0n]
Lycaena virgaureae|WMB3507-14|RVcoll.10-B785||France|658[0n]
Lycaena virgaureae|EZROM677-08|RV-06-N002||Romania|658[0n]
Lycaena virgaureae|EZROM271-08|RV-06-M879||Romania|620[1n]
Lycaena virgaureae|EZRMN063-08|RVcoll.06-M904||Romania|645[0n]
Lycaena virgaureae|LEFIB038-10|MM00305||Finland|642[0n]
Lycaena virgaureae|WMB3333-14|RVcoll.08-P777||Spain|658[0n]
Lycaena virgaureae|PHLAH830-12|TLMF Lep 08634||Spain|635[0n]
Lycaena virgaureae|EULEP5073-16|RVcoll16J198||Czech Republic|658[0n]
Lycaena virgaureae|GBLAA1250-15|BC ZSM Lep 87136||Germany|658[0n]
Lycaena virgaureae|GBLAF378-14|BC ZSM Lep 82084||Germany|658[0n]
Lycaena virgaureae|LEFIG533-10|MM14659||Finland|658[0n]
Lycaena virgaureae|EULEP4177-16|RVcoll15G038||France|658[0n]
Lycaena virgaureae|BIBSA407-15|RVcoll.14-I075||Italy|658[0n]
Lycaena virgaureae|EZRMN065-08|RVcoll.07-C907||Romania|658[3n]
Lycaena virgaureae|EZROM273-08|RV-07-E489||Romania|656[0n]
Lycaena virgaureae|EZRMN064-08|RVcoll.06-V698||Romania|658[5n]
Lycaena virgaureae|EULEP1521-15|RVcoll.14-G449||Greece|658[0n]
Lycaena virgaureae|EULEP891-15|RVcoll.14-C629||Greece|658[0n]
Lycaena virgaureae|EULEP1147-15|RVcoll.14-F322||Serbia|658[0n]
Lycaena virgaureae|EULEP1047-15|RVcoll.14-E915||Serbia|658[0n]
Lycaena virgaureae|PHLAF290-11|TLMF Lep 05460||Macedonia|658[0n]
Lycaena virgaureae|PHLAF291-11|TLMF Lep 05461||Macedonia|658[0n]
Lycaena virgaureae|EULEP2014-15|RVcoll.14-I806||Slovakia|658[0n]
Lycaena virgaureae|LEATA081-13|TLMF Lep 09498||Austria|658[0n]
Lycaena virgaureae|EULEP4962-16|RVcoll16I302||Poland|658[0n]
Lycaena virgaureae|PHLAI506-13|TLMF Lep 09068||Austria|658[0n]
Lycaena virgaureae|LEATD146-13|TLMF Lep 12793||Italy|658[0n]
Lycaena virgaureae|PHLSA405-11|TLMF Lep 05860||Austria|658[0n]
Lycaena virgaureae|GWORA2465-09|BC ZSM Lep 30677||Germany|658[0n]
Lycaena virgaureae|ABOLD604-17|TLMF Lep 21680||Austria|658[0n]
Lycaena virgaureae|EZRMN066-08|RVcoll.08-M590||Romania|658[0n]
Lycaena virgaureae|WMB1866-13|RVcoll.11-J201||France|658[0n]
Lycaena virgaureae|WMB2003-13|RVcoll.12-Q390||France|658[0n]
Lycaena virgaureae|WMB4315-14|RVcoll.14-A513||Italy|658[0n]
Lycaena virgaureae|WMB4988-14|RVcoll. 14-I535||Italy|658[0n]
Lycaena virgaureae|EULEP4799-16|RVcoll16H607||Sweden|658[0n]
Lycaena virgaureae|EULEP4178-16|RVcoll15H670||Switzerland|658[0n]
Lycaena virgaureae|EULEP4179-16|RVcoll15I177||Austria|658[0n]
Lycaena virgaureae|EULEP4180-16|RVcoll15I765||Austria|658[0n]
Lycaena virgaureae|EULEP4183-16|RVcoll15Q174||Ukraine|658[0n]
Lycaena virgaureae|EULEP634-15|RVcoll.11-J046||Switzerland|658[0n]
Lycaena virgaureae|EULEP984-15|RVcoll.14-D145||Austria|658[0n]
Lycaena virgaureae|EULEP1974-15|RVcoll.14-H875||France|658[0n]
Lycaena virgaureae|BIBSA1215-15|15-M342||Italy|658[0n]
Lycaena virgaureae|BIBSA401-15|RVcoll.14-I069||Italy|658[0n]
Lycaena virgaureae|BIBSA305-15|RVcoll.14-E107||Italy|658[0n]
Lycaena virgaureae|BIBSA238-15|RVcoll.14-E030||Italy|658[0n]
Lycaena virgaureae|GWOTL173-13|BC ZSM Lep 67104||Germany|658[0n]
Lycaena virgaureae|GWOTL174-13|BC ZSM Lep 67105||Germany|658[0n]
Lycaena virgaureae|EULEP324-14|MM23809||Latvia|658[0n]
Lycaena virgaureae|PHLSA406-11|TLMF Lep 05861||Austria|625[1n]
Lycaena virgaureae|WMB2705-13|RVcoll.10-C124||France|646[0n]
Lycaena virgaureae|EULEP4181-16|RVcoll15J322||France|637[0n]
Lycaena virgaureae|EULEP4182-16|RVcoll15Q169||Ukraine|634[0n]
Lycaena virgaureae|BIBSA237-15|RVcoll.14-E029||Italy|627[0n]
Lycaena virgaureae|LEATC621-13|TLMF Lep 12603||Austria|632[0n]
Lycaena ottomana|EULEP2547-15|RVcoll.14-V458||Greece|616[0n]
Lycaena ottomana|EULEP2929-15|RVcoll.14-Q062||Albania|658[0n]

[illegible]
Lycaena virgaureae|LEATC621-13|TLMF Lep 12603||Austria|632[0n]
Lycaena ottomana|EULEP2547-15|RVcoll.14-V458||Greece|616[0n]
Lycaena ottomana|EULEP2929-15|RVcoll.14-O062||Albania|658[0n]
Lycaena ottomana|EULEP1474-15|RVcoll.14-G248||Greece|658[0n]
Lycaena ottomana|EULEP1473-15|RVcoll.14-G240||Greece|658[0n]
Lycaena ottomana|EULEP1465-15|RVcoll.14-G226||Greece|658[0n]
Lycaena ottomana|EULEP1366-15|RVcoll.14-F944||Greece|658[0n]
Lycaena ottomana|EULEP1365-15|RVcoll.14-F935||Greece|658[0n]
Lycaena ottomana|EULEP2931-15|RVcoll.14-O064|Thassos|Greece|658[0n]
Lycaena ottomana|EULEP1831-15|RVcoll.14-J952||Bulgaria|658[0n]
Lycaena ottomana|EULEP4159-16|RVcoll14A961||Bulgaria|658[0n]
Lycaena tityrus|EULEP4169-16|RVcoll15H554||Switzerland|658[0n]
Lycaena tityrus|EZSPM143-09|RVcoll.08-P655||Spain|658[0n]
Lycaena tityrus|BIBSA1253-15|15-F838||France|658[0n]
Lycaena tityrus|EULEP1982-15|RVcoll.14-H934||France|658[0n]
Lycaena tityrus|WMB5301-14|RVcoll.14-I619||France|658[0n]
Lycaena tityrus|WMB3321-14|RVcoll.08-P729||Spain|658[0n]
Lycaena tityrus|WMB3365-14|RVcoll.09-T178||Spain|658[0n]
Lycaena tityrus|EZSPM769-12|Rvcoll. 050111LP02||Spain|658[0n]
Lycaena tityrus|EZSPM252-09|RVcoll.08-R464||Spain|658[0n]
Lycaena tityrus|EZSPM142-09|RVcoll.08-P654||Spain|658[0n]
Lycaena tityrus|EZSPM297-09|RVcoll.08-J842||Spain|658[0n]
Lycaena tityrus|EZSPN570-09|RVcoll.08-J114||Portugal|658[0n]
Lycaena tityrus|EZSPN391-09|RVcoll.08-H447||Spain|658[0n]
Lycaena tityrus|EZROM268-08|RV-06-M976||Romania|658[0n]
Lycaena tityrus|EULEP1486-15|RVcoll.14-G298||Greece|658[0n]
Lycaena tityrus|EULEP1394-15|RVcoll.14-G015||Greece|658[0n]
Lycaena tityrus|FBLMU496-09|BC ZSM Lep 27146||Germany|658[0n]
Lycaena tityrus|EULEP325-14|MM23810||Latvia|658[0n]
Lycaena tityrus|EULEP4983-16|RVcoll16I610||Poland|658[0n]
Lycaena tityrus|EULEP4948-16|RVcoll16I167||Germany|658[0n]
Lycaena tityrus|EULEP5063-16|RVcoll16J118||Czech Republic|658[0n]
Lycaena tityrus|EULEP5046-16|RVcoll16J042||Slovakia|658[0n]
Lycaena tityrus|EULEP4184-16|RVcoll15Q118||Russia|658[0n]
Lycaena tityrus|EULEP4175-16|RVcoll15Q077||Russia|658[0n]
Lycaena tityrus|EULEP4172-16|RVcoll15I491||Austria|658[0n]
Lycaena tityrus|EULEP4167-16|RVcoll10B347||Bulgaria|658[0n]
Lycaena tityrus|LOWA289-06|2005-LOWA-289||Russia|658[0n]
Lycaena tityrus|WMB5282-14|RVcoll.14-A743||Italy|658[0n]
Lycaena tityrus|WMB5201-14|RVcoll.13-T929||Italy|658[0n]
Lycaena tityrus|WMB5192-14|RVcoll.13-T801||Italy|658[0n]
Lycaena tityrus|WMB5150-14|RVcoll.12-M542||Italy|658[0n]
Lycaena tityrus|WMB5149-14|RVcoll.11-Y081||Italy|658[0n]
Lycaena tityrus|WMB5148-14|RVcoll.11-J591||Italy|658[0n]
Lycaena tityrus|WMB4983-14|RVcoll. 14-I530||Italy|658[0n]
Lycaena tityrus|WMB5376-14|RVcoll.14-J778||France|658[0n]
Lycaena tityrus|WMB2389-13|RVcoll.08-R617||Italy|658[0n]
Lycaena tityrus|WMB2215-13|RVcoll.11-Y029||Italy|658[0n]
Lycaena tityrus|WMB2035-13|RVcoll.12-Q746||Italy|658[0n]
Lycaena tityrus|WMB1761-13|RVcoll.12-P996|Levant|France|658[0n]
Lycaena tityrus|WMB1760-13|RVcoll.12-P995|Levant|France|658[0n]
Lycaena tityrus|WMB1689-13|RVcoll.12-P546||France|658[0n]
Lycaena tityrus|WMB1829-13|RVcoll.11-I754||France|658[0n]
Lycaena tityrus|WMB3468-14|RVcoll.10-A625||France|658[0n]
Lycaena tityrus|WMB3430-14|RVcoll.09-X279||France|658[0n]
Lycaena tityrus|WMB3428-14|RVcoll.09-X277||France|658[0n]
Lycaena tityrus|WMB3406-14|RVcoll.09-V821||Spain|658[0n]
Lycaena tityrus|EZROM270-08|RV-07-E479||Romania|658[0n]
Lycaena tityrus|EZSPC506-09|RVcoll.08-P003||Spain|658[0n]
Lycaena tityrus|EZSPC505-09|RVcoll.08-R309||Spain|658[0n]
Lycaena tityrus|EZSPC504-09|RVcoll.07-C624||Spain|658[0n]
Lycaena tityrus|EZRMN058-08|RVcoll.07-D317||Romania|658[0n]
Lycaena tityrus|EZRMN061-08|RVcoll.08-M612||Romania|658[0n]
Lycaena tityrus|EZROM267-08|RV-06-K611||Romania|658[0n]
Lycaena tityrus|LEFID122-10|MM05858||Estonia|658[0n]
Lycaena tityrus|GWORA2468-09|BC ZSM Lep 30680||Germany|658[0n]
Lycaena tityrus|GWORZ040-10|BC ZSM Lep 30396||Italy|658[0n]
Lycaena tityrus|LEFIJ1005-11|MM21245||Estonia|658[0n]
Lycaena tityrus|GWORK519-09|BC ZSM Lep 21849||Germany|658[0n]
Lycaena tityrus|ABOLB032-15|TLMF Lep 17037||Italy|658[0n]
Lycaena tityrus|ABOLD094-16|TLMF Lep 21172||Austria|658[0n]
Lycaena tityrus|PHLAW038-13|TLMF Lep 09835||Austria|658[0n]
Lycaena tityrus|EULEP1204-15|RVcoll.14-F499||Greece|658[0n]
Lycaena tityrus|EULEP333-14|MM23818||Estonia|658[0n]
Lycaena tityrus|EULEP2022-15|RVcoll.14-I857||Poland|658[0n]
Lycaena tityrus|EULEP2392-15|RVcoll.14-V095||Ukraine|658[0n]
Lycaena tityrus|BIBSA1202-15|15-M300||Italy|658[0n]
Lycaena tityrus|BIBSA677-15|RVcoll. 12-M505||Italy|658[0n]
Lycaena tityrus|GBLAA381-14|BC ZSM Lep 80472||Germany|658[0n]
Lycaena tityrus|BIBSA050-14|LEP-SS-00049||Italy|658[0n]
Lycaena tityrus|LEASS1037-17|TLMF Lep 22590||Austria|658[0n]
Lycaena tityrus|OXB361-15|RVcoll.14-N075||Italy|658[0n]
Lycaena tityrus|OXB947-15|15-A591||Italy|658[0n]
Lycaena tityrus|OXB1359-15|Rvcoll_15-M712||France|658[0n]
Lycaena tityrus|BIBSA1338-15|15-M830||Italy|613[0n]
Lycaena tityrus|BIBSA704-15|RVcoll. 12-R431||Italy|627[0n]
Lycaena tityrus|EULEP4176-16|RVcoll15Q109||Russia|633[0n]
Lycaena tityrus|EZSPC508-09|RVcoll.08-P033||Spain|634[0n]
Lycaena tityrus|EZSPC507-09|RVcoll.08-P004||Spain|648[0n]
Lycaena tityrus|EULEP1617-15|RVcoll.14-G689||Greece|658[0n]
Lycaena tityrus|EZROM269-08|RV-07-C903||Romania|658[0n]
Lycaena tityrus|EZRMN060-08|RVcoll.08-M559||Romania|658[0n]
Lycaena tityrus|EZRMN059-08|RVcoll.08-M460||Romania|658[0n]
Lycaena tityrus|GBLAB132-13|BC ZSM Lep 75758||Germany|658[0n]
Lycaena tityrus|GWORO791-09|BC ZSM Lep 30483||Germany|658[0n]
Lycaena tityrus|EULEP2217-15|RVcoll.14-N395||Ukraine|658[0n]
Lycaena tityrus|BIBSA1028-15|15-C181||Italy|658[0n]
Lycaena tityrus|BIBSA338-15|RVcoll.14-I006||Italy|658[0n]
Lycaena tityrus|BIBSA049-14|LEP-SS-00048||Italy|658[0n]
Lycaena tityrus|EZRMN062-08|RVcoll.08-M639||Romania|658[0n]
Lycaena tityrus|PHLAB312-10|TLMF Lep 01112||Switzerland|658[0n]
Lycaena tityrus|WMB1632-13|RVcoll.12-O843||France|634[0n]
[illegible]

Lycaena tityrus|EZRMN062-08|RVcoll.08-M639||Romania|658[0n]
Lycaena tityrus|PHLAB312-10|TLMF Lep 01112||Switzerland|658[0n]
Lycaena tityrus|WMB1632-13|RVcoll.12-O843||France|634[0n]
Lycaena tityrus|ODOPE749-11|BC ZSM Lep 50390||Germany|612[0n]
Lycaena tityrus|BIBSA137-15|RVcoll.14-I151||Italy|627[0n]
Lycaena tityrus|BIBSA405-15|RVcoll.14-I073||Italy|633[0n]
Lycaena tityrus|EULEP2449-15|RVcoll.14-V269||Switzerland|658[0n]
Lycaena tityrus|EULEP4168-16|RVcoll15H189||Switzerland|658[0n]
Lycaena tityrus|WMB5116-14|RVcoll. 14-L247||Italy|658[0n]
Lycaena tityrus|PHLAB361-10|TLMF Lep 01161||Switzerland|658[0n]
Lycaena tityrus|LEATH781-14|TLMF Lep 15993||Italy|658[0n]
Lycaena tityrus|PHLAH697-12|TLMF Lep 08516||Austria|658[0n]
Lycaena tityrus|LEASS547-17|TLMF Lep 22195||Austria|658[0n]
Lycaena tityrus|LEASS522-17|TLMF Lep 22170||Austria|658[0n]
Lycaena tityrus|BIBSA304-15|RVcoll.14-E106||Italy|658[0n]
Lycaena tityrus|BIBSA303-15|RVcoll.14-E105||Italy|658[0n]
Lycaena tityrus|BIBSA236-15|RVcoll.14-E028||Italy|658[0n]
Lycaena tityrus|BIBSA234-15|RVcoll.14-E026||Italy|658[0n]
Lycaena tityrus|BIBSA404-15|RVcoll.14-I072||Italy|658[0n]
Lycaena tityrus|EULEP4174-16|RVcoll15G336||Switzerland|658[0n]
Lycaena tityrus|EULEP4173-16|RVcoll15I623||Austria|658[0n]
Lycaena tityrus|EULEP4171-16|RVcoll15I362||Austria|658[0n]
Lycaena tityrus|EULEP4170-16|RVcoll15H987||Italy|658[0n]
Lycaena tityrus|GWOTF674-12|BC ZSM Lep 62380||Germany|658[0n]
Lycaena tityrus|PHLAA644-09|TLMF Lep 00684||France|658[0n]
Lycaena tityrus|LEATD296-13|TLMF Lep 12943||Italy|658[0n]
Lycaena tityrus|LEATG007-14|TLMF Lep 13794||Austria|658[0n]
Lycaena tityrus|LEATG427-14|TLMF Lep 14214||Italy|658[0n]
Tomares nogelii|EULEP3155-15|RVcoll.14-O288||Ukraine|658[0n]
Tomares nogelii|EULEP2253-15|RVcoll.14-N436||Ukraine|658[0n]
Tomares nogelii|EULEP2254-15|RVcoll.14-N437||Ukraine|624[0n]
Tomares nogelii|EULEP2485-15|RVcoll.14-V342||Ukraine|620[0n]
Tomares nogelii|EULEP2484-15|RVcoll.14-V341||Ukraine|658[0n]
Tomares nogelii|EULEP2483-15|RVcoll.14-V340||Ukraine|658[0n]
Tomares nogelii|EULEP2482-15|RVcoll.14-V339||Ukraine|658[0n]
Tomares nogelii|EULEP3156-15|RVcoll.14-O289||Ukraine|658[0n]
Tomares nogelii|EULEP427-14|KN00789||Ukraine|658[0n]
Tomares nogelii|EULEP426-14|KN00788||Ukraine|658[0n]
Tomares callimachus|EULEP2251-15|RVcoll.14-N434||Ukraine|658[0n]
Tomares callimachus|EULEP2502-15|RVcoll.14-V365||Ukraine|658[0n]
Tomares callimachus|EULEP2496-15|RVcoll.14-V354||Ukraine|658[0n]
Tomares callimachus|EULEP2079-15|RVcoll.14-J567||Ukraine|658[0n]
Tomares callimachus|EULEP2078-15|RVcoll.14-J566||Ukraine|658[0n]
Tomares callimachus|EULEP409-14|KN00771||Russia|658[0n]
Tomares callimachus|EULEP2498-15|RVcoll.14-V358||Ukraine|658[0n]
Tomares callimachus|EULEP2221-15|RVcoll.14-N399||Ukraine|658[0n]
Tomares callimachus|EULEP2520-15|RVcoll.14-V392||Ukraine|658[0n]
Tomares callimachus|EULEP2521-15|RVcoll.14-V393||Ukraine|658[0n]
Tomares callimachus|EULEP2252-15|RVcoll.14-N435||Ukraine|614[0n]
Tomares callimachus|EULEP2250-15|RVcoll.14-N433||Ukraine|623[0n]
Tomares callimachus|EULEP2497-15|RVcoll.14-V357||Ukraine|612[0n]
Tomares callimachus|EULEP408-14|KN00770||Russia|658[0n]
Tomares ballus|EULEP3154-15|RVcoll.14-O287||France|658[0n]
Tomares ballus|EZSPC1277-10|RVcoll.070207MC2||Spain|658[0n]
Tomares ballus|EZSPC1011-10|RVcoll.08-H540||Spain|658[0n]
Tomares ballus|EZSPC1275-10|RVcoll.130606MU13||Spain|658[0n]
Tomares ballus|EZSPN414-09|RVcoll.08-H532||Spain|658[0n]
Tomares ballus|EZSPN379-09|RVcoll.08-H425||Spain|658[0n]
Tomares ballus|EZSPN341-09|RVcoll.08-H300||Spain|658[0n]
Tomares ballus|EZSPC1323-10|RVcoll.130209KL35||Spain|658[0n]
Tomares ballus|EZSPN613-09|RVcoll.08-J357||Spain|658[0n]
Tomares ballus|EZSPC1010-10|RVcoll.08-H531||Spain|658[0n]
Tomares ballus|EZSPC1006-10|RVcoll.08-H413||Spain|658[0n]
Tomares ballus|EZSPN430-09|RVcoll.08-H607||Spain|658[0n]
Tomares ballus|EZSPN129-09|RVcoll.06-G460||Spain|658[0n]
Zizeeria karsandra|EULEP5902-18|RVcoll17A035|Malta|Malta|658[0n]
Zizeeria karsandra|EULEP5901-18|RVcoll17A033|Malta|Malta|624[0n]
Zizeeria karsandra|EULEP5900-18|RVcoll17A032|Malta|Malta|619[0n]
Zizeeria karsandra|EULEP4874-16|RVcoll16H962||Cyprus|658[0n]
Zizeeria karsandra|EULEP4711-16|RVcoll16H961||Cyprus|658[0n]
Zizeeria knysna|EZSPN289-09|RVcoll.07-W243||Spain|658[0n]
Zizeeria knysna|EZSPN288-09|RVcoll.07-W242||Spain|658[0n]
Zizeeria knysna|EZSPM618-12|RVcoll.11-D865||Spain|658[0n]
Zizeeria knysna|EZSPM347-09|RVcoll.08-R030||Spain|658[0n]
Zizeeria knysna|EZSPM168-09|RVcoll.08-R021||Spain|658[0n]
Zizeeria knysna|EZSPN821-09|RVcoll.08-L630||Spain|658[0n]
Zizeeria knysna|EZSPC996-10|RVcoll.07-W244||Spain|658[0n]
Zizeeria knysna|EZSPC997-10|RVcoll.07-W245||Spain|658[0n]
Zizeeria knysna|EZSPC1153-10|RVcoll.08-R032||Spain|658[0n]
Zizeeria knysna|EZSPM171-09|RVcoll.08-R028||Spain|658[1n]
Zizeeria knysna|EZSPN820-09|RVcoll.08-L629||Spain|658[0n]
Leptotes pirithous|EULEP5699-17|RVcoll16L042||Italy|658[0n]
Leptotes pirithous|EULEP5687-17|RVcoll16L023|Corsica|France|658[0n]
Leptotes pirithous|EULEP5686-17|RVcoll16L022|Corsica|France|658[0n]
Leptotes pirithous|WMB1119-13|RVcoll.11-D170|Marettimo|Italy|629[0n]
Leptotes pirithous|WMB1907-13|RVcoll.11-H616|Lampedusa|Italy|654[0n]
Leptotes pirithous|WMB2954-14|RVcoll.12-N001|Ibiza|Spain|658[0n]
Leptotes pirithous|WMB1671-13|RVcoll.12-P279||France|658[0n]
Leptotes pirithous|WMB1752-13|RVcoll.12-P967|Levant|France|658[0n]
Leptotes pirithous|WMB1753-13|RVcoll.12-P968|Levant|France|658[0n]
Leptotes pirithous|WMB2258-13|RVcoll.12-R417||Malta|658[0n]
Leptotes pirithous|WMB2290-13|RVcoll.10-C603||Malta|658[0n]
Leptotes pirithous|WMB2291-13|RVcoll.10-C604||Malta|658[0n]
Leptotes pirithous|WMB5966-17|RVcoll14N669|Salina|Italy|658[0n]
Leptotes pirithous|WMB5974-17|RVcoll14W001|Ustica|Italy|658[0n]
Leptotes pirithous|WMB2440-13|RVcoll.11-D180|Marettimo|Italy|658[0n]
Leptotes pirithous|WMB4581-14|RVcoll.150308GV92||Spain|658[0n]
Leptotes pirithous|WMB6068-18|RVcoll16L026|Corsica|France|658[0n]
Leptotes pirithous|WMB6069-18|RVcoll16L195||Italy|658[0n]
Leptotes pirithous|WMB519-11|RVcoll.11-H611|Lampedusa|Italy|658[0n]
Leptotes pirithous|WMB5359-14|RVcoll.14-J705||France|658[0n]
Leptotes pirithous|WMB5002-14|RVcoll. 14-I549||Italy|658[0n]
Leptotes pirithous|WMB5459-14|RVcoll.14-N602|Levanzo|Italy|658[0n]

Leptotes pirithous|WMB5359-14|RVcoll.14-J705||France|658[0n]
Leptotes pirithous|WMB5002-14|RVcoll. 14-I549||Italy|658[0n]
Leptotes pirithous|WMB5459-14|RVcoll.14-N602|Levanzo|Italy|658[0n]
Leptotes pirithous|WMB5476-14|RVcoll.14-N697|Alicudi|Italy|658[0n]
Leptotes pirithous|WMB5483-14|RVcoll.14-N723|Stromboli|Italy|658[0n]
Leptotes pirithous|EULEP5681-17|RVcoll16L003|Sardinia|Italy|658[0n]
Leptotes pirithous|EULEP5682-17|RVcoll16L004|Sardinia|Italy|658[0n]
Leptotes pirithous|EULEP5684-17|RVcoll16L007|Sardinia|Italy|658[0n]
Leptotes pirithous|EULEP5696-17|RVcoll16L039||Italy|658[0n]
Leptotes pirithous|EULEP5715-17|RVcoll16L071|Elba|Italy|658[0n]
Leptotes pirithous|EULEP5716-17|RVcoll16L072|Giglio|Italy|658[0n]
Leptotes pirithous|BIBSA1781-16|16-A499||Italy|658[0n]
Leptotes pirithous|BIBSA343-15|RVcoll.14-I011||Italy|658[0n]
Leptotes pirithous|BIBSA346-15|RVcoll.14-I014||Italy|658[0n]
Leptotes pirithous|OXB933-15|15-A577||Italy|658[0n]
Leptotes pirithous|WMB1778-13|RVcoll.12-Q104||France|635[0n]
Leptotes pirithous|WMB1772-13|RVcoll.12-Q059||France|658[0n]
Leptotes pirithous|WMB2741-13|RVcoll.10-C605||Malta|658[0n]
Leptotes pirithous|WMB1114-13|RVcoll.11-D140|Pantelleria|Italy|658[0n]
Leptotes pirithous|WMB3682-14|RVcoll.11-J223||Spain|658[0n]
Leptotes pirithous|WMB3060-14|RVcoll.13-T752|Elba|Italy|658[0n]
Leptotes pirithous|WMB3410-14|RVcoll.09-V928||Spain|658[0n]
Leptotes pirithous|WMB3385-14|RVcoll.09-V458||Spain|658[0n]
Leptotes pirithous|WMB135-11|RVcoll.11-D139|Pantelleria|Italy|658[0n]
Leptotes pirithous|EZSPC480-09|RVcoll.08-M698||Spain|658[0n]
Leptotes pirithous|EZSPC479-09|RVcoll.08-R164||Spain|658[0n]
Leptotes pirithous|EZSPC477-09|RVcoll.08-R158||Spain|658[0n]
Leptotes pirithous|EZSPC476-09|RVcoll.08-M715||Spain|658[0n]
Leptotes pirithous|EZSPC478-09|RVcoll.07-C072||Spain|658[0n]
Leptotes pirithous|WMB143-11|RVcoll.11-D169|Marettimo|Italy|658[0n]
Leptotes pirithous|EZSPM212-09|RVcoll.08-R333||Spain|658[0n]
Leptotes pirithous|EZSPM056-09|RVcoll.08-P281||Spain|658[0n]
Leptotes pirithous|EZSPM061-09|RVcoll.08-P287||Spain|658[0n]
Leptotes pirithous|EZSPN322-09|RVcoll.08-H183||Spain|658[0n]
Leptotes pirithous|EZSPN214-09|RVcoll.07-F072||Spain|658[0n]
Leptotes pirithous|EZSPN093-09|RVcoll.06-A022||Spain|658[0n]
Leptotes pirithous|WMB4422-14|RVcoll.14-B476||Portugal|658[0n]
Leptotes pirithous|OXB934-15|15-A578||Italy|658[0n]
Leptotes pirithous|OXB935-15|15-A579||Italy|658[0n]
Leptotes pirithous|OXB928-15|15-A572|Montecristo|Italy|649[0n]
Leptotes pirithous|OXB599-15|14-N988||Italy|658[0n]
Leptotes pirithous|BIBSA768-15|LEP-SS-00279||Italy|658[0n]
Leptotes pirithous|BIBSA479-15|LEP-SS-00099||Italy|658[0n]
Leptotes pirithous|BIBSA478-15|LEP-SS-00098||Italy|658[0n]
Leptotes pirithous|BIBSA714-15|RVcoll. 14-A710||Italy|658[0n]
Leptotes pirithous|BIBSA713-15|RVcoll. 14-A708||Italy|658[0n]
Leptotes pirithous|BIBSA1374-15|15-M933||Italy|658[0n]
Leptotes pirithous|BIBSA1779-16|16-A485||Italy|658[0n]
Leptotes pirithous|EULEP5693-17|RVcoll16L036||Italy|658[0n]
Leptotes pirithous|EULEP5692-17|RVcoll16L035||Italy|658[0n]
Leptotes pirithous|EULEP5513-17|RVcoll13T842||Italy|658[0n]
Leptotes pirithous|EULEP5512-17|RVcoll13T828||Italy|658[0n]
Leptotes pirithous|WMB5462-14|RVcoll.14-N639|Vulcano|Italy|658[0n]
Leptotes pirithous|WMB5341-14|RVcoll.14-J341|Pianosa|Italy|658[0n]
Leptotes pirithous|WMB4789-14|RVcoll. 14-I336||Italy|658[0n]
Leptotes pirithous|WMB5086-14|RVcoll. 14-L217||Italy|658[0n]
Leptotes pirithous|WMB4272-14|RVcoll.14-A205||Italy|658[0n]
Leptotes pirithous|WMB4233-14|RVcoll.13-T822||Italy|658[0n]
Leptotes pirithous|WMB5957-17|RVcoll14A709||Italy|658[0n]
Leptotes pirithous|WMB2426-13|RVcoll.10-C786||Italy|658[0n]
Leptotes pirithous|WMB1097-13|RVcoll.10-C765|Giglio|Italy|658[0n]
Leptotes pirithous|WMB1093-13|RVcoll.10-C759|Capraia|Italy|658[0n]
Leptotes pirithous|WMB1092-13|RVcoll.10-C758|Capraia|Italy|658[0n]
Leptotes pirithous|WMB2743-13|RVcoll.10-C615||Italy|658[0n]
Leptotes pirithous|WMB1106-13|RVcoll.10-C785||Italy|658[0n]
Leptotes pirithous|EZSPM364-09|RVcoll.08-J825||Spain|658[0n]
Leptotes pirithous|EZSPN846-09|RVcoll.08-L684||Spain|658[0n]
Leptotes pirithous|EZSPN818-09|RVcoll.08-L626||Spain|658[0n]
Leptotes pirithous|GWORZ044-10|BC ZSM Lep 30400||Italy|658[0n]
Leptotes pirithous|EZSPN587-09|RVcoll.08-J149||Portugal|658[0n]
Leptotes pirithous|EZSPN872-09|RVcoll.08-L762||Spain|658[0n]
Leptotes pirithous|EZSPM882-12|Rvcoll.12-M600||Spain|658[0n]
Leptotes pirithous|WMB3248-14|RVcoll.08-L261||Spain|658[0n]
Leptotes pirithous|WMB3262-14|RVcoll.08-L743||Spain|658[0n]
Leptotes pirithous|WMB4378-14|RVcoll.14-B193||Portugal|658[0n]
Leptotes pirithous|OXB1165-15|Rvcoll_16-A052||Italy|658[0n]
Leptotes pirithous|EULEP1578-15|RVcoll.14-G603||Greece|658[0n]
Leptotes pirithous|EULEP4846-16|RVcoll16H755||Bulgaria|658[0n]
Leptotes pirithous|EULEP4131-16|RVcoll10B339||Bulgaria|658[0n]
Leptotes pirithous|WMB5348-14|RVcoll.14-J380|Giannutri|Italy|658[0n]
Leptotes pirithous|WMB4758-14|RVcoll. 14-I305||Italy|658[0n]
Leptotes pirithous|WMB4482-14|RVcoll.14-D927||Italy|658[0n]
Leptotes pirithous|WMB4306-14|RVcoll.14-A413||Italy|658[0n]
Leptotes pirithous|WMB804-13|RVcoll.07-E046||Italy|658[0n]
Leptotes pirithous|EZRMN023-08|RVcoll.08-M581||Romania|658[0n]
Leptotes pirithous|EZROM243-08|RV-07-E448||Romania|658[0n]
Leptotes pirithous|PHLAF293-11|TLMF Lep 05463||Macedonia|658[0n]
Callophrys avis|EZSPN615-09|RVcoll.08-J376||Spain|658[0n]
Callophrys avis|EZSPN616-09|RVcoll.08-J382||Spain|658[0n]
Callophrys avis|EZSPC1287-10|RVcoll.100607MP29||Spain|658[0n]
Callophrys avis|WMB5188-14|RVcoll.13-T168||Spain|658[0n]
Callophrys avis|WMB5189-14|RVcoll.13-T169||Spain|658[0n]
Callophrys avis|EZSPN614-09|RVcoll.08-J375||Spain|630[0n]
Callophrys avis|EZSPN809-09|RVcoll.08-L501||Spain|658[0n]
Callophrys avis|EZSPN808-09|RVcoll.08-L500||Spain|658[0n]
Callophrys rubi|EZSPM247-09|RVcoll.08-R459|Genit. examined|Spain|656[0n]
Callophrys rubi|GBLAB130-13|BC ZSM Lep 75756||Germany|658[0n]
Callophrys rubi|OXB897-15|15-A541||Italy|658[0n]
Callophrys rubi|EZROM071-08|RV-07-D230||Romania|658[1n]
Callophrys rubi|EZROM825-08|RVcoll.08-M215||Romania|658[0n]
Callophrys rubi|EZROM823-08|RVcoll.06-M944||Romania|658[0n]
Callophrys rubi|LENOA1412-11|LN-BD1406||France|658[0n]

Callophrys rubi|EZROM825-08|RVcoll.08-M215||Romania|658[0n]
Callophrys rubi|EZROM823-08|RVcoll.06-M944||Romania|658[0n]
Callophrys rubi|LENOA1412-11|LN-BD1406||France|658[0n]
Callophrys rubi|GWORO785-09|BC ZSM Lep 30477||Germany|658[0n]
Callophrys chalybeitincta|EULEP2203-15|RVcoll.14-N363||Russia|658[0n]
Callophrys suaveola|EULEP416-14|KN00778||Russia|658[0n]
Callophrys suaveola|EULEP415-14|KN00777||Russia|658[0n]
Callophrys suaveola|EULEP3587-16|RVcoll15Q148||Russia|658[0n]
Callophrys rubi|EZROM710-08|RV-06-G432||Spain|658[0n]
Callophrys rubi|EULEP3607-16|RVcoll15G856||Italy|658[0n]
Callophrys rubi|OXB943-15|15-A587||Italy|658[0n]
Callophrys rubi|BIBSA878-15|LD-3490||Italy|658[0n]
Callophrys rubi|WMB347-11|RVcoll.11-E749||France|658[0n]
Callophrys rubi|WMB306-11|RVcoll.11-E359||Italy|658[0n]
Callophrys rubi|WMB2614-13|RVcoll.11-E753|Corsica|France|658[0n]
Callophrys rubi|WMB2613-13|RVcoll.11-E752|Corsica|France|658[0n]
Callophrys rubi|WMB2204-13|RVcoll.11-Y009|Elba|Italy|658[0n]
Callophrys rubi|WMB1171-13|RVcoll.11-E772|Corsica|France|658[0n]
Callophrys rubi|WMB1156-13|RVcoll.11-E504|Sardinia|Italy|658[0n]
Callophrys rubi|WMB1153-13|RVcoll.11-E456|Sardinia|Italy|658[0n]
Callophrys rubi|WMB3093-14|RVcoll.LD-1775|Giglio|Italy|658[0n]
Callophrys rubi|WMB3092-14|RVcoll.LD-1774|Giglio|Italy|658[0n]
Callophrys rubi|WMB3091-14|RVcoll.LD-1773|Giglio|Italy|658[0n]
Callophrys rubi|WMB3053-14|RVcoll.13-T650|Sardinia|Italy|658[0n]
Callophrys rubi|OXB1159-15|Rvcoll_16-A046|Elba|Italy|658[0n]
Callophrys rubi|OXB1158-15|Rvcoll_LD-2694|Giglio|Italy|658[0n]
Callophrys rubi|WMB3052-14|RVcoll.13-T648|Sardinia|Italy|658[0n]
Callophrys rubi|LEATD068-13|TLMF Lep 12715||Italy|658[0n]
Callophrys rubi|WMB2447-13|RVcoll.11-E357|Sardinia|Italy|658[0n]
Callophrys rubi|WMB2205-13|RVcoll.11-Y010|Elba|Italy|658[0n]
Callophrys rubi|LEATD067-13|TLMF Lep 12714||Italy|658[0n]
Callophrys rubi|EZSPM989-12|Rvcoll.12-L874||Spain|658[0n]
Callophrys rubi|OXB1444-16|OXB-TGS-1218||United Kingdom|658[0n]
Callophrys rubi|OXB1443-16|OXB-TGS-1217||United Kingdom|658[0n]
Callophrys rubi|EULEP4818-16|RVcoll16H713||Belgium|658[0n]
Callophrys rubi|WMB4037-14|RVcoll.12-Z343||Spain|658[0n]
Callophrys rubi|WMB2934-14|RVcoll.12-L227|Mallorca|Spain|658[0n]
Callophrys rubi|WMB2933-14|RVcoll.12-L226|Mallorca|Spain|658[0n]
Callophrys rubi|EZSPM991-12|Rvcoll.12-L876||Spain|658[0n]
Callophrys rubi|EZSPM990-12|Rvcoll.12-L875||Spain|658[0n]
Callophrys rubi|EZSPM988-12|Rvcoll.12-L873||Spain|658[0n]
Callophrys rubi|EZSPC219-09|RVcoll.08-L428||Spain|655[0n]
Callophrys rubi|GBLAA1274-15|BC ZSM Lep 87160||Germany|658[0n]
Callophrys rubi|EZSPN407-09|RVcoll.08-H501||Spain|658[0n]
Callophrys rubi|EZSPC213-09|RVcoll.08-J374||Spain|655[0n]
Callophrys rubi|EZSPC214-09|RVcoll.08-J385||Spain|658[0n]
Callophrys rubi|EZSPN337-09|RVcoll.08-H269||Spain|658[0n]
Callophrys rubi|EZSPN635-09|RVcoll.08-J746||Spain|658[0n]
Callophrys rubi|EZSPM281-09|SMcoll.150308PP64||Spain|658[0n]
Callophrys rubi|EZSPM359-09|RVcoll.08-R039||Spain|658[0n]
Callophrys rubi|WMB278-11|RVcoll.10-A555||France|658[0n]
Callophrys rubi|WMB3535-14|RVcoll.10-C353||Spain|658[0n]
Callophrys rubi|WMB875-13|RVcoll.09-X253||France|658[0n]
Callophrys rubi|WMB943-13|RVcoll.10-A637||France|658[0n]
Callophrys rubi|WMB4576-14|RVcoll.150308GV19||Spain|658[0n]
Callophrys rubi|WMB4028-14|RVcoll.12-Z133||Ireland|658[0n]
Callophrys rubi|EULEP3608-16|RVcoll15G873||Switzerland|658[0n]
Callophrys rubi|EZSPC1373-10|RVcoll.09-X216||France|658[0n]
Callophrys rubi|GBLAA1357-15|BC ZSM Lep 87053||Germany|658[0n]
Callophrys rubi|LEASS458-17|TLMF Lep 21916||Austria|658[0n]
Callophrys rubi|OXB950-15|15-A594||Italy|658[0n]
Callophrys rubi|EZSPC201-09|RVcoll.06-H868||Spain|651[0n]
Callophrys rubi|EZSPN690-09|RVcoll.08-L020||Spain|633[0n]
Callophrys rubi|OXB1247-15|Rvcoll_13-T731|Sardinia|Italy|612[0n]
Callophrys rubi|WMB2228-13|RVcoll.11-Y066||Italy|612[0n]
Callophrys rubi|EULEP1550-15|RVcoll.14-G533||Greece|614[0n]
Callophrys rubi|EULEP2371-15|RVcoll.14-V066||Ukraine|613[0n]
Callophrys rubi|EZROM069-08|RV-06-K553||Romania|609[0n]
Callophrys rubi|ODOPE752-11|BC ZSM Lep 50393||Germany|658[0n]
Callophrys rubi|ODOPE370-11|BC ZSM Lep 50771||Germany|658[0n]
Callophrys rubi|PHLAB297-10|TLMF Lep 01097||Switzerland|658[0n]
Callophrys rubi|LEATD092-13|TLMF Lep 12739||Austria|658[0n]
Callophrys rubi|PHLAB296-10|TLMF Lep 01096||Switzerland|658[0n]
Callophrys rubi|GWORZ043-10|BC ZSM Lep 30399||Italy|658[0n]
Callophrys rubi|BIBSA1585-16|12-M484||Italy|658[0n]
Callophrys rubi|BIBSA057-14|LEP-SS-00057||Italy|632[0n]
Callophrys rubi|WMB4787-14|RVcoll. 14-I334||Italy|658[0n]
Callophrys rubi|WMB4505-14|RVcoll.14-E183||Italy|658[0n]
Callophrys rubi|WMB4841-14|RVcoll. 14-I388||Italy|658[0n]
Callophrys rubi|WMB4795-14|RVcoll. 14-I342||Italy|658[0n]
Callophrys rubi|BIBSA571-15|LEP-SS-00191||Italy|658[0n]
Callophrys rubi|BIBSA572-15|LEP-SS-00192||Italy|658[0n]
Callophrys rubi|BIBSA1370-15|15-M914||Italy|658[0n]
Callophrys rubi|BIBSA1710-16|15-N150||Italy|658[0n]
Callophrys rubi|BIBSA1631-16|15-C470||Italy|658[0n]
Callophrys rubi|BIBSA998-15|15-C035||Italy|658[0n]
Callophrys rubi|BIBSA1648-16|15-C631||Italy|658[0n]
Callophrys rubi|BIBSA058-14|LEP-SS-00058||Italy|658[0n]
Callophrys rubi|OXB883-15|15-A525||Italy|658[0n]
Callophrys rubi|OXB708-15|13-T887||Italy|658[0n]
Callophrys rubi|OXB921-15|15-A565||Italy|658[0n]
Callophrys rubi|OXB929-15|15-A573||Italy|658[0n]
Callophrys rubi|OXB930-15|15-A574||Italy|658[0n]
Callophrys rubi|OXB936-15|15-A580||Italy|658[0n]
Callophrys rubi|OXB1027-15|15-A977||Italy|658[0n]
Callophrys rubi|WMB4811-14|RVcoll. 14-I358||Italy|614[0n]
Callophrys rubi|WMB4502-14|RVcoll.14-E179||Italy|658[0n]
Callophrys rubi|WMB4253-14|RVcoll.13-U075||Italy|658[0n]
Callophrys rubi|WMB4174-14|RVcoll.13-S765||Italy|658[0n]
Callophrys rubi|WMB4126-14|RVcoll.13-S607||Italy|658[0n]
Callophrys rubi|WMB2390-13|RVcoll.09-T534||Italy|658[0n]
Callophrys rubi|WMB2337-13|RVcoll.12-M278||Italy|658[0n]
Callophrys rubi|WMB890-13|RVcoll.09-X813||Italy|658[0n]

Callophrys rubi|WMB2390-13|RVcoll.09-T534||Italy|658[0n]
Callophrys rubi|WMB2337-13|RVcoll.12-M278||Italy|658[0n]
Callophrys rubi|WMB890-13|RVcoll.09-X813||Italy|658[0n]
Callophrys rubi|WMB185-11|RVcoll.11-D490||Italy|658[0n]
Callophrys rubi|EULEP1355-15|RVcoll.14-F919||Greece|658[0n]
Callophrys rubi|EULEP841-15|RVcoll.14-C021||Sweden|658[0n]
Callophrys rubi|EZROM072-08|RV-07-D469||Romania|658[0n]
Callophrys rubi|WMB2943-14|RVcoll.12-M399|Sicily|Italy|658[0n]
Callophrys rubi|EULEP3609-16|RVcoll15Q066||Russia|652[0n]
Callophrys chalybeitincta|EULEP3592-16|RVcoll15Q115||Russia|645[0n]
Callophrys rubi|EULEP3610-16|RVcoll15P026||Belarus|658[0n]
Callophrys rubi|EULEP3614-16|RVcoll15Q139||Russia|658[0n]
Callophrys rubi|EULEP4761-16|RVcoll16H049||Sweden|658[0n]
Callophrys rubi|EULEP1108-15|RVcoll.14-F177||Serbia|658[0n]
Callophrys rubi|EULEP2140-15|RVcoll.14-J992||Switzerland|658[0n]
Callophrys rubi|EULEP1705-15|RVcoll.14-H141||Greece|658[0n]
Callophrys chalybeitincta|EULEP2505-15|RVcoll.14-V599||Russia|658[0n]
Callophrys rubi|EULEP2196-15|RVcoll.14-N354||Ukraine|658[0n]
Callophrys rubi|BIBSA1581-16|12-M370||Italy|658[0n]
Callophrys rubi|LEASS524-17|TLMF Lep 22172||Austria|658[0n]
Callophrys chalybeitincta|EULEP2518-15|RVcoll.14-V600||Russia|616[0n]
Callophrys rubi|EULEP3605-16|RVcoll10A805||Estonia|615[0n]
Callophrys chalybeitincta|EULEP3591-16|RVcoll15Q114||Russia|658[0n]
Callophrys chalybeitincta|EULEP3590-16|RVcoll15Q113||Russia|658[0n]
Callophrys chalybeitincta|EULEP3589-16|RVcoll14V601||Russia|658[0n]
Callophrys rubi|WMB618-11|RVcoll.11-I039|Sicily|Italy|658[0n]
Callophrys rubi|WMB504-11|RVcoll.11-H562|Sicily|Italy|658[0n]
Callophrys rubi|WMB2815-13|RVcoll.12-M566|Sicily|Italy|658[0n]
Callophrys rubi|WMB179-11|RVcoll.11-D454|Sicily|Italy|658[0n]
Callophrys rubi|EZROM070-08|RV-07-D207||Romania|658[0n]
Callophrys rubi|EZROM068-08|RV-06-K552||Romania|658[0n]
Callophrys rubi|EZROM1049-09|RVcoll.07-D298||Romania|658[0n]
Callophrys rubi|EZROM824-08|RVcoll.07-D292||Romania|658[0n]
Callophrys rubi|LEFIJ493-10|MM17118||Finland|658[0n]
Callophrys rubi|LEFID008-10|MM05430||Finland|658[0n]
Callophrys rubi|GWORO786-09|BC ZSM Lep 30478||Germany|658[0n]
Callophrys rubi|LEFIB084-10|MM00386||Finland|658[0n]
Callophrys rubi|LEATA400-13|TLMF Lep 10007||Austria|658[0n]
Callophrys rubi|ABOLD074-16|TLMF Lep 21152||Austria|658[0n]
Callophrys rubi|LEATA077-13|TLMF Lep 09494||Austria|658[0n]
Neolycaena rhymnus|EULEP2358-15|RVcoll.14-V050||Ukraine|647[0n]
Neolycaena rhymnus|EULEP4892-16|RVcoll16J591||Ukraine|658[0n]
Neolycaena rhymnus|EULEP2032-15|RVcoll.14-J511||Ukraine|658[0n]
Neolycaena rhymnus|EULEP2240-15|RVcoll.14-N420||Ukraine|658[0n]
Neolycaena rhymnus|EULEP2241-15|RVcoll.14-N421||Ukraine|658[0n]
Neolycaena rhymnus|EULEP2266-15|RVcoll.14-N451||Ukraine|658[0n]
Neolycaena rhymnus|EULEP3007-15|RVcoll.14-O140||Russia|658[0n]
Neolycaena rhymnus|EULEP412-14|KN00774||Russia|658[1n]
Neolycaena rhymnus|EULEP4323-16|RVcoll14V610||Russia|624[0n]
Neolycaena rhymnus|EULEP4322-16|RVcoll14V609||Russia|658[0n]
Satyrium pruni|EZROM542-08|RV-06-M829||Romania|658[0n]
Satyrium pruni|EZROM543-08|RV-07-D307||Romania|658[0n]
Satyrium pruni|EZRMN270-08|RVcoll.08-M303||Romania|658[0n]
Satyrium pruni|EZRMN271-08|RVcoll.08-M364||Romania|658[0n]
Satyrium pruni|EZRMN269-08|RVcoll.08-M218||Romania|658[0n]
Satyrium pruni|EZRMN268-08|RVcoll.08-M207||Romania|658[0n]
Satyrium pruni|LEFIJ492-10|MM17117||Finland|658[0n]
Satyrium pruni|GBLAA1447-15|BC ZSM Lep 87238||Germany|658[0n]
Satyrium pruni|GBLAA1433-15|BC ZSM Lep 87224||Germany|658[0n]
Satyrium pruni|EULEP2215-15|RVcoll.14-N393||Ukraine|658[0n]
Satyrium pruni|EULEP2206-15|RVcoll.14-N366||Ukraine|658[0n]
Satyrium pruni|EULEP2379-15|RVcoll.14-V074||Ukraine|658[0n]
Satyrium pruni|EULEP1099-15|RVcoll.14-F138||Serbia|658[0n]
Satyrium pruni|EULEP5883-18|RVcoll15O582||Spain|658[0n]
Satyrium pruni|EULEP5138-17|RVcoll16J894||Serbia|658[0n]
Satyrium pruni|LEASS907-17|KLM Lep 08412||Austria|658[0n]
Satyrium pruni|LEASS1033-17|TLMF Lep 22586||Austria|658[0n]
Satyrium pruni|EULEP360-14|MM23845||Lithuania|658[0n]
Satyrium pruni|EULEP212-14|RVcoll.12-L033||Spain|658[0n]
Satyrium pruni|EULEP4857-16|RVcoll16H830||Poland|658[0n]
Satyrium pruni|EULEP4645-16|RVcoll10A793||Estonia|658[0n]
Satyrium pruni|WMB5337-14|RVcoll.14-J228||Spain|658[0n]
Satyrium pruni|WMB5336-14|RVcoll.14-J227||Spain|658[0n]
Satyrium pruni|EZSPC1143-10|RVcoll.08-P737||Spain|658[0n]
Satyrium pruni|EZSPC1142-10|RVcoll.08-P736||Spain|658[0n]
Satyrium pruni|EZSPC1141-10|RVcoll.08-P735||Spain|658[0n]
Satyrium pruni|EZSPC1140-10|RVcoll.08-P734||Spain|658[0n]
Satyrium pruni|EZROM686-08|RV-07-D594||Romania|658[0n]
Satyrium pruni|EZROM544-08|RV-07-C923||Romania|658[0n]
Satyrium pruni|LEFIJ797-10|MM17422||Finland|658[0n]
Satyrium pruni|LEFIJ796-10|MM17421||Finland|658[0n]
Satyrium pruni|FBLMU259-09|BC ZSM Lep 25769||Germany|658[0n]
Satyrium pruni|FBLMU254-09|BC ZSM Lep 25764||Germany|658[0n]
Satyrium pruni|LEASS746-17|TLMF Lep 22394||Austria|658[0n]
Satyrium pruni|LEASS696-17|TLMF Lep 22344||Austria|658[0n]
Satyrium pruni|ABOLD491-16|TLMF Lep 21474||Austria|658[0n]
Satyrium ledereri|EULEP5114-17|RVcoll16J869|Samos|Greece|658[0n]
Satyrium ledereri|EULEP5113-17|RVcoll16J868|Samos|Greece|658[0n]
Satyrium ledereri|EULEP5112-17|RVcoll16J867|Samos|Greece|658[0n]
Satyrium w-album|EULEP203-14|RVcoll.12-L015||Spain|658[2n]
Satyrium w-album|WMB2584-13|RVcoll.12-R106|Sicily|Italy|658[0n]
Satyrium w-album|WMB2583-13|RVcoll.12-R105|Sicily|Italy|658[0n]
Satyrium w-album|WMB2104-13|RVcoll.12-R117|Sicily|Italy|658[0n]
Satyrium w-album|WMB2098-13|RVcoll.12-R111|Sicily|Italy|658[0n]
Satyrium w-album|EZSPC322-09|RVcoll.07-C479||Spain|658[0n]
Satyrium w-album|EZSPC637-09|RVcoll.08-L382||Spain|658[0n]
Satyrium w-album|EZSPM863-12|Rvcoll.070611MH52||Spain|658[0n]
Satyrium w-album|EZSPC323-09|RVcoll.07-C685||Spain|658[0n]
Satyrium w-album|EULEP4914-16|RVcoll16J617||Russia|658[0n]
Satyrium w-album|EULEP4655-16|RVcoll15I759||Austria|658[0n]
Satyrium w-album|EULEP083-14|RVcoll.08-P969||Romania|658[0n]
Satyrium w-album|EULEP077-14|RVcoll.08-P961||Spain|658[0n]
Satyrium w-album|WMB5392-14|RVcoll.14-J873||France|658[0n]

Satyrium w-album|EULEP[illegible]|RVcoll15I739||Austria|658[0n]
Satyrium w-album|EULEP083-14|RVcoll.08-P969||Romania|658[0n]
Satyrium w-album|EULEP077-14|RVcoll.08-P961||Spain|658[0n]
Satyrium w-album|WMB5392-14|RVcoll.14-J873||France|658[0n]
Satyrium w-album|EZSPM865-12|Rvcoll.070611MH66||Spain|658[0n]
Satyrium w-album|EULEP084-14|RVcoll.08-P970||Romania|658[0n]
Satyrium w-album|EULEP5660-17|RVcoll15L326||Italy|658[0n]
Satyrium w-album|EULEP1131-15|RVcoll.14-F260||Serbia|658[0n]
Satyrium w-album|EULEP830-15|RVcoll.14-B932||Sweden|658[0n]
Satyrium w-album|EULEP3135-15|RVcoll.14-O268||United Kingdom|658[0n]
Satyrium w-album|BIBSA904-15|LD-3516||Italy|658[0n]
Satyrium w-album|BIBSA905-15|LD-3517||Italy|658[0n]
Satyrium w-album|BCLEP115-17|LEP-SS-00566||Italy|658[0n]
Satyrium w-album|EULEP078-14|RVcoll.08-P962||Spain|658[0n]
Satyrium w-album|LEASS1053-17|TLMF Lep 22606||Austria|658[0n]
Satyrium w-album|OXB1289-15|Rvcoll_15-M152||France|658[0n]
Satyrium w-album|EULEP2398-15|RVcoll.14-V104||Ukraine|614[0n]
Satyrium w-album|EULEP4654-16|RVcoll15I487||Austria|623[0n]
Satyrium w-album|EULEP4652-16|RVcoll10B341||Bulgaria|633[0n]
Satyrium w-album|LEFIC996-10|MM05411||Finland|643[0n]
Satyrium w-album|ABOLD608-17|TLMF Lep 21684||Austria|658[0n]
Satyrium w-album|ABOLD645-17|TLMF Lep 21721||Austria|658[0n]
Satyrium w-album|FBLMU441-09|BC ZSM Lep 27091||Germany|658[0n]
Satyrium w-album|LEFIL363-10|MM18673||Finland|658[0n]
Satyrium w-album|EZRMN259-08|RVcoll.08-M543||Romania|658[0n]
Satyrium w-album|EZRMN258-08|RVcoll.08-M538||Romania|658[0n]
Satyrium w-album|EZRMN257-08|RVcoll.08-M537||Romania|658[0n]
Satyrium w-album|EZRMN256-08|RVcoll.08-M536||Romania|658[0n]
Satyrium w-album|EZRMN255-08|RVcoll.08-M497||Romania|658[0n]
Satyrium w-album|GWOSK865-11|BC ZSM Lep 49366||Germany|658[0n]
Satyrium w-album|LEFIL362-10|MM18672||Finland|658[0n]
Satyrium w-album|LASTS335-14|TLMF Lep 14787||Austria|658[0n]
Satyrium acaciae|BIBSA1377-15|15-M949||Italy|658[0n]
Satyrium acaciae|WMB4884-14|RVcoll.14-I431||Italy|658[0n]
Satyrium acaciae|OXB979-15|15-A929||Italy|658[0n]
Satyrium acaciae|WMB794-13|RVcoll.07-D872||Italy|619[0n]
Satyrium acaciae|WMB791-13|RVcoll.07-D851||Italy|618[0n]
Satyrium acaciae|WMB790-13|RVcoll.07-D848||Italy|658[0n]
Satyrium acaciae|EULEP1739-15|RVcoll.14-H308||Greece|658[0n]
Satyrium acaciae|BIBSA1840-17|RVcoll16C717||Italy|658[0n]
Satyrium acaciae|EULEP1422-15|RVcoll.14-G097||Greece|658[0n]
Satyrium acaciae|EULEP1332-15|RVcoll.14-F813||Greece|658[0n]
Satyrium acaciae|EZSPC1300-10|RVcoll.090806MA6||Spain|658[0n]
Satyrium acaciae|EZSPC308-09|RVcoll.08-M701||Spain|658[0n]
Satyrium acaciae|EZSPC311-09|RVcoll.08-P356||Spain|658[0n]
Satyrium acaciae|EZSPC309-09|RVcoll.08-R280||Spain|658[0n]
Satyrium acaciae|EZSPN876-09|RVcoll.08-L771||Spain|658[0n]
Satyrium acaciae|EZSPC310-09|RVcoll.08-M927||Spain|658[0n]
Satyrium acaciae|EZSPN1001-09|RVcoll.08-M086||Spain|658[0n]
Satyrium acaciae|EZSPN926-09|RVcoll.08-L877||Spain|658[0n]
Satyrium acaciae|EZSPC1354-10|RVcoll.09-V888||Spain|658[0n]
Satyrium acaciae|EZSPC1294-10|RVcoll.210207TM50||Spain|658[0n]
Satyrium acaciae|EZSPC1297-10|RVcoll.030908SF61||Spain|658[0n]
Satyrium acaciae|EZSPC307-09|RVcoll.07-C480||Spain|658[0n]
Satyrium acaciae|EZSPN152-09|RVcoll.08-R057||Spain|658[0n]
Satyrium acaciae|EZROM537-08|RV-06-M913||Romania|658[0n]
Satyrium acaciae|WMB1823-13|RVcoll.11-I644||France|658[0n]
Satyrium acaciae|EZROM538-08|RV-07-D965||Romania|658[0n]
Satyrium acaciae|EULEP4642-16|RVcoll10A958||Bulgaria|658[0n]
Satyrium acaciae|EULEP4891-16|RVcoll16J587||Moldova|658[0n]
Satyrium acaciae|EULEP127-14|RVcoll.10-A336||Romania|658[0n]
Satyrium acaciae|EULEP1163-15|RVcoll.14-F367||Bulgaria|658[0n]
Satyrium acaciae|EULEP1193-15|RVcoll.14-F466||Bulgaria|658[0n]
Satyrium acaciae|EULEP2372-15|RVcoll.14-V067||Ukraine|658[0n]
Satyrium acaciae|GBLAB131-13|BC ZSM Lep 75757||Germany|658[0n]
Satyrium acaciae|LEASS906-17|KLM Lep 08411||Austria|658[0n]
Satyrium acaciae|LEASS1028-17|TLMF Lep 22581||Austria|658[0n]
Satyrium acaciae|EULEP1105-15|RVcoll.14-F168||Serbia|623[0n]
Satyrium acaciae|EULEP999-15|RVcoll.14-G420||Greece|614[0n]
Satyrium acaciae|EZROM540-08|RV-07-C972||Romania|641[0n]
Satyrium acaciae|EZROM536-08|RV-06-M877||Romania|658[0n]
Satyrium acaciae|EZRMN262-08|RVcoll.08-M384||Romania|658[0n]
Satyrium acaciae|EZRMN261-08|RVcoll.08-M378||Romania|658[0n]
Satyrium acaciae|EZRMN260-08|RVcoll.08-M208||Romania|658[0n]
Satyrium acaciae|EZRMN254-08|RVcoll.07-D928||Romania|658[0n]
Satyrium acaciae|EZRMN253-08|RVcoll.07-D571||Romania|658[0n]
Satyrium acaciae|EZRMN252-08|RVcoll.07-D371||Romania|658[0n]
Satyrium acaciae|EZRMN251-08|RVcoll.07-C422||Romania|658[0n]
Satyrium acaciae|ABOLD609-17|TLMF Lep 21685||Austria|658[0n]
Satyrium acaciae|FBLMU262-09|BC ZSM Lep 25772||Germany|658[0n]
Satyrium acaciae|ABOLD004-16|TLMF Lep 21082||Austria|658[0n]
Satyrium esculi|EZSPC1074-10|RVcoll.08-L454||Spain|658[0n]
Satyrium esculi|EZSPM1032-12|Rvcoll.12-M782||Spain|658[0n]
Satyrium esculi|WMB5379-14|RVcoll.14-J786||France|658[0n]
Satyrium esculi|EZSPM916-12|Rvcoll.12-M638||Spain|658[0n]
Satyrium esculi|EZSPC1358-10|RVcoll.09-V940||Spain|658[0n]
Satyrium esculi|EZSPC1281-10|RVcoll.210907WR75||Spain|658[0n]
Satyrium esculi|EZSPC1296-10|RVcoll.270808DT2||Spain|658[0n]
Satyrium esculi|WMB3193-14|RVcoll.08-J055||Spain|658[0n]
Satyrium esculi|WMB3194-14|RVcoll.08-J060||Spain|658[0n]
Satyrium esculi|WMB3245-14|RVcoll.08-L194||Spain|658[0n]
Satyrium esculi|WMB3588-14|RVcoll.11-E101||Spain|658[0n]
Satyrium esculi|WMB3738-14|RVcoll.12-M657||Spain|658[0n]
Satyrium esculi|WMB4573-14|RVcoll.140410KV82||Spain|658[0n]
Satyrium esculi|BIBSA906-15|LD-3518||France|658[0n]
Satyrium esculi|BIBSA907-15|LD-3519||France|658[0n]
Satyrium esculi|EZSPM932-12|Rvcoll.12-M664||Spain|631[0n]
Satyrium esculi|EZSPM909-12|Rvcoll.12-M631||Spain|639[0n]
Satyrium esculi|EZSPM908-12|Rvcoll.12-M630||Spain|640[0n]
Satyrium esculi|EZSPC929-10|RVcoll.08-R160.1||Spain|658[0n]
Satyrium esculi|EZSPM201-09|RVcoll.08-R295||Spain|658[0n]
Satyrium esculi|EZSPM072-09|RVcoll.08-P314||Spain|658[0n]
Satyrium esculi|EZSPN862-09|RVcoll.08-L730||Spain|658[0n]

Satyrium esculi|EZSPM201-09|RVcoll.08-R295||Spain|658[0n]
Satyrium esculi|EZSPM072-09|RVcoll.08-P314||Spain|658[0n]
Satyrium esculi|EZSPN862-09|RVcoll.08-L730||Spain|658[0n]
Satyrium esculi|EZSPN1018-09|RVcoll.08-M949|Genit. examined|Spain|658[0n]
Satyrium esculi|EZSPN988-09|RVcoll.08-M031||Spain|658[0n]
Satyrium esculi|EZSPN903-09|RVcoll.08-L822||Spain|658[0n]
Satyrium esculi|EZSPM361-09|RVcoll.08-J818||Spain|658[0n]
Satyrium esculi|EZSPN522-09|RVcoll.08-H989||Spain|658[0n]
Satyrium esculi|EZSPM954-12|Rvcoll.12-M705||Spain|658[0n]
Satyrium esculi|EZSPM952-12|Rvcoll.12-M702||Spain|658[0n]
Satyrium esculi|EZSPN804-09|RVcoll.08-L457||Spain|658[0n]
Satyrium ilicis|EULEP4643-16|RVcoll14O310|Lesvos|Greece|658[0n]
Satyrium ilicis|EZROM539-08|RV-07-D974||Romania|658[0n]
Satyrium ilicis|EULEP4644-16|RVcoll15G201||France|634[0n]
Satyrium ilicis|WMB2795-13|RVcoll.11-J716|Sicily|Italy|658[0n]
Satyrium ilicis|BIBSA1002-15|15-C080||Italy|658[0n]
Satyrium ilicis|EZSPC313-09|RVcoll.08-R288||Spain|658[0n]
Satyrium ilicis|WMB1073-13|RVcoll.10-C697|Ischia|Italy|658[0n]
Satyrium ilicis|WMB1072-13|RVcoll.10-C696|Ischia|Italy|658[0n]
Satyrium ilicis|WMB1498-13|RVcoll.12-O044||Italy|658[0n]
Satyrium ilicis|WMB812-13|RVcoll.07-E111||Italy|658[0n]
Satyrium ilicis|WMB2752-13|RVcoll.10-C662||Italy|658[0n]
Satyrium ilicis|WMB2789-13|RVcoll.11-I357||Italy|658[0n]
Satyrium ilicis|WMB2873-14|RVcoll.10-C565|Argentario|Italy|658[0n]
Satyrium ilicis|WMB2856-14|RVcoll.09-X793|Elba|Italy|658[0n]
Satyrium ilicis|WMB2855-14|RVcoll.09-X784|Elba|Italy|658[0n]
Satyrium ilicis|EZSPM229-09|RVcoll.08-R424||Spain|658[0n]
Satyrium ilicis|WMB4286-14|RVcoll.14-A338||Italy|658[0n]
Satyrium ilicis|WMB4499-14|RVcoll.14-E175||Italy|658[0n]
Satyrium ilicis|EZSPM090-09|RVcoll.08-P414||Spain|658[0n]
Satyrium ilicis|EZSPN1023-09|RVcoll.08-M955.1||Spain|658[0n]
Satyrium ilicis|EULEP1252-15|RVcoll.14-F621||Greece|658[0n]
Satyrium ilicis|EULEP5886-18|RVcoll15O590||Spain|658[0n]
Satyrium ilicis|EULEP5144-17|RVcoll16J900||Serbia|658[0n]
Satyrium ilicis|WMB4870-14|RVcoll.14-I417||Italy|658[0n]
Satyrium ilicis|WMB4121-14|RVcoll.13-S581||Italy|658[0n]
Satyrium ilicis|WMB2468-13|RVcoll.11-H516|Capri|Italy|658[0n]
Satyrium ilicis|EZSPN959-09|RVcoll.08-L958|Genit. examined|Spain|658[0n]
Satyrium ilicis|EZSPN927-09|RVcoll.08-L882||Spain|658[0n]
Satyrium ilicis|EZSPC316-09|RVcoll.07-C474||Spain|658[0n]
Satyrium ilicis|EZSPC312-09|RVcoll.08-M700||Spain|658[0n]
Satyrium ilicis|EZSPC223-09|RVcoll.08-L453||Spain|658[0n]
Satyrium ilicis|EZRMN267-08|RVcoll.08-M448||Romania|658[0n]
Satyrium ilicis|WMB4836-14|RVcoll. 14-I383||Italy|658[0n]
Satyrium ilicis|WMB483-11|RVcoll.11-H514|Capri|Italy|658[0n]
Satyrium ilicis|WMB3822-14|RVcoll.09-T581||Italy|658[0n]
Satyrium ilicis|WMB3806-14|RVcoll.07-D871||Italy|658[0n]
Satyrium ilicis|WMB2421-13|RVcoll.10-C698|Ischia|Italy|658[0n]
Satyrium ilicis|WMB2288-13|RVcoll.10-C544||Italy|658[0n]
Satyrium ilicis|WMB2287-13|RVcoll.10-C543||Italy|658[0n]
Satyrium ilicis|WMB1902-13|RVcoll.11-H515|Capri|Italy|658[0n]
Satyrium ilicis|EZRMN266-08|RVcoll.08-M431||Romania|658[0n]
Satyrium ilicis|GWORZ042-10|BC ZSM Lep 30398||Italy|658[0n]
Satyrium ilicis|EULEP1652-15|RVcoll.14-G837||Greece|658[0n]
Satyrium ilicis|EULEP1359-15|RVcoll.14-F925||Greece|658[0n]
Satyrium ilicis|EULEP1719-15|RVcoll.14-H203||Greece|658[0n]
Satyrium ilicis|EULEP1769-15|RVcoll.14-H488||Greece|658[0n]
Satyrium ilicis|BIBSA1394-15|15-M998||Italy|658[0n]
Satyrium ilicis|BIBSA1411-15|15-N021||Italy|658[0n]
Satyrium ilicis|EZROM541-08|RV-06-M907||Romania|658[0n]
Satyrium ilicis|BIBSA651-15|RVcoll. 15-A814||Italy|658[0n]
Satyrium ilicis|BIBSA763-15|LEP-SS-00274||Italy|658[0n]
Satyrium ilicis|OXB972-15|15-A922||Italy|658[0n]
Satyrium ilicis|OXB973-15|15-A923||Italy|658[0n]
Satyrium ilicis|OXB1094-15|15-A627||Italy|658[1n]
Satyrium ilicis|EZRMN265-08|RVcoll.08-M429||Romania|658[0n]
Satyrium ilicis|GWOTF698-12|BC ZSM Lep 62404||Croatia|658[0n]
Satyrium ilicis|BIBSA1252-15|15-F837||France|658[0n]
Satyrium ilicis|OXB1038-15|LD-2822||Italy|658[0n]
Satyrium ilicis|EULEP2237-15|RVcoll.14-N417||Ukraine|658[0n]
Satyrium ilicis|EULEP4898-16|RVcoll16J601||Russia|658[0n]
Satyrium ilicis|WMB616-11|RVcoll.11-I033|Sicily|Italy|658[0n]
Satyrium ilicis|WMB4718-14|RVcoll.LD-3009|Sicily|Italy|658[0n]
Satyrium ilicis|WMB1877-13|RVcoll.11-J650|Sicily|Italy|658[0n]
Satyrium ilicis|WMB2107-13|RVcoll.12-R131|Sicily|Italy|658[0n]
Satyrium ilicis|EZSPC315-09|RVcoll.08-M930|Genit. examined|Spain|658[0n]
Satyrium ilicis|EZSPC314-09|RVcoll.08-M929|Genit. examined|Spain|658[0n]
Satyrium ilicis|EZRMN264-08|RVcoll.08-M390||Romania|658[0n]
Satyrium ilicis|EZRMN263-08|RVcoll.08-M383||Romania|658[0n]
Satyrium ilicis|ABOLD058-16|TLMF Lep 21136||Austria|658[0n]
Satyrium spini|EZSPM602-12|RVcoll.11-I540||Spain|658[0n]
Satyrium spini|EZSPC319-09|RVcoll.07-C691||Spain|658[0n]
Satyrium spini|WMB3578-14|RVcoll.11-E007||Spain|658[0n]
Satyrium spini|WMB3197-14|RVcoll.08-J066.1||Spain|658[0n]
Satyrium spini|EZSPN964-09|RVcoll.08-L974||Spain|658[0n]
Satyrium spini|WMB282-11|RVcoll.10-A676||France|658[0n]
Satyrium spini|EZSPM867-12|Rvcoll.090111XZ15||Spain|658[0n]
Satyrium spini|WMB3240-14|RVcoll.08-L108||Spain|658[0n]
Satyrium spini|EZSPM953-12|Rvcoll.12-M703||Spain|658[0n]
Satyrium spini|EZSPN916-09|RVcoll.08-L860||Spain|658[0n]
Satyrium spini|EZSPN944-09|RVcoll.08-L922||Spain|658[0n]
Satyrium spini|WMB3247-14|RVcoll.08-L223||Spain|658[0n]
Satyrium spini|EZSPC318-09|RVcoll.07-F092||Spain|642[0n]
Satyrium spini|EZSPC317-09|RVcoll.08-R159||Spain|658[0n]
Satyrium spini|EZSPM646-12|RVcoll.11-D629||Spain|658[0n]
Satyrium spini|EZSPM856-12|Rvcoll.040611HZ11||Spain|658[0n]
Satyrium spini|EZSPN705-09|RVcoll.08-L051||Spain|658[0n]
Satyrium spini|EZSPN701-09|RVcoll.08-L044||Spain|658[0n]
Satyrium spini|EZSPN584-09|RVcoll.08-J144||Portugal|658[0n]
Satyrium spini|EZSPM860-12|Rvcoll.050111LP84||Spain|628[0n]
Satyrium spini|WMB3351-14|RVcoll.08-R401||Spain|658[0n]
Satyrium spini|EZSPM150-09|RVcoll.08-P665|Genit. examined|Spain|658[0n]
Satyrium spini|EZSPM002-09|RVcoll.08-M965||Spain|658[0n]

Satyrium spini|WMB3351-14|RVcoll.08-R401||Spain|658[0n]
Satyrium spini|EZSPM150-09|RVcoll.08-P665|Genit. examined|Spain|658[0n]
Satyrium spini|EZSPM002-09|RVcoll.08-M965||Spain|658[0n]
Satyrium spini|EZSPC321-09|RVcoll.08-P035||Spain|658[0n]
Satyrium spini|EZSPC320-09|RVcoll.08-P029||Spain|658[0n]
Satyrium spini|EZSPN137-09|RVcoll.06-G496||Spain|658[0n]
Satyrium spini|EULEP2504-15|RVcoll.14-V370||Ukraine|658[0n]
Satyrium spini|EZROM545-08|RV-06-M929||Romania|658[0n]
Satyrium spini|EULEP2395-15|RVcoll.14-V099||Ukraine|613[0n]
Satyrium spini|EZROM546-08|RV-06-M946||Romania|658[0n]
Satyrium spini|EZROM324-08|RV-08-A002||Romania|658[1n]
Satyrium spini|EZROM309-08|RV-07-E671||Romania|656[0n]
Satyrium spini|EZROM342-08|RV-08-A020||Romania|658[0n]
Satyrium spini|EZROM308-08|RV-07-E670||Romania|632[0n]
Satyrium spini|EZROM350-08|RV-08-A028|Genit. examined|Romania|658[0n]
Satyrium spini|EZROM349-08|RV-08-A027|Genit. examined|Romania|658[0n]
Satyrium spini|WMB5250-14|RVcoll.14-A226||Italy|658[0n]
Satyrium spini|WMB5182-14|RVcoll.13-S669||Italy|658[0n]
Satyrium spini|BCLEP146-17|LP-SS-00546||Italy|658[0n]
Satyrium spini|EULEP4648-16|RVcoll15C262||Italy|658[0n]
Satyrium spini|BIBSA1375-15|15-M937||Italy|658[0n]
Satyrium spini|WMB2030-13|RVcoll.12-Q726||Italy|658[0n]
Satyrium spini|WMB2845-13|RVcoll.12-Q730||Italy|658[0n]
Satyrium spini|GBLAA880-14|BC ZSM Lep 83441|subm. as S. ilicis, corrected 2014-08-20 (AHS ...
Satyrium spini|LENOA1459-11|LN-BD1453||France|658[0n]
Satyrium spini|GBLAA1373-15|BC ZSM Lep 87069||Germany|658[0n]
Satyrium spini|WMB1665-13|RVcoll.12-P214||France|658[0n]
Satyrium spini|EULEP4651-16|RVcoll15I554||Italy|658[0n]
Satyrium spini|LEASS549-17|TLMF Lep 22197||Austria|658[0n]
Satyrium spini|LEASS548-17|TLMF Lep 22196||Austria|658[0n]
Satyrium spini|EULEP2212-15|RVcoll.14-N389||Ukraine|658[0n]
Satyrium spini|EULEP4650-16|RVcoll15H927||Italy|658[0n]
Satyrium spini|WMB5391-14|RVcoll.14-J872||France|658[0n]
Satyrium spini|WMB5028-14|RVcoll. 14-I575||Italy|658[0n]
Satyrium spini|FBLMV667-09|BC ZSM Lep 28647||Germany|658[0n]
Satyrium spini|ABOLD607-17|TLMF Lep 21683||Austria|658[0n]
Satyrium spini|GWORO787-09|BC ZSM Lep 30479||Germany|658[0n]
Satyrium spini|LEATJ1311-16|TLMF Lep 19634||Austria|658[0n]
Satyrium spini|ABOLD055-16|TLMF Lep 21133||Austria|658[0n]
Satyrium spini|LEATI007-15|TLMF Lep 17392||Austria|658[0n]
Satyrium spini|LEATI062-15|TLMF Lep 17447||Austria|658[0n]
Satyrium spini|EULEP1529-15|RVcoll.14-G479||Greece|658[0n]
Satyrium spini|EULEP1744-15|RVcoll.14-H352||Greece|658[0n]
Satyrium spini|EULEP1255-15|RVcoll.14-F626||Greece|658[0n]
Satyrium spini|EULEP1563-15|RVcoll.14-G563||Macedonia|658[0n]
Satyrium spini|EULEP1484-15|RVcoll.14-G295||Greece|658[0n]
Satyrium spini|EULEP1455-15|RVcoll.14-G184||Greece|658[0n]
Satyrium spini|EULEP1384-15|RVcoll.14-F986||Greece|658[0n]
Satyrium spini|EULEP4646-16|RVcoll10B305||Bulgaria|658[0n]
Satyrium spini|PHLAF330-11|TLMF Lep 05500||Macedonia|658[0n]
Satyrium spini|BIBSA276-15|RVcoll.14-E076||Italy|658[0n]
Satyrium spini|EULEP4649-16|RVcoll15H159||Italy|658[0n]
Satyrium spini|EULEP4647-16|RVcoll14V318||Italy|658[0n]
Satyrium spini|LEATG393-14|TLMF Lep 14180||Italy|658[0n]
Satyrium spini|WMB1789-13|RVcoll.12-Q216||France|658[0n]
Satyrium spini|BIBSA445-15|RVcoll.14-I113||Italy|658[0n]
Satyrium spini|OXB720-15|13-U118||Italy|658[0n]
Satyrium spini|OXB1186-15|Rvcoll_16-A073||Italy|658[0n]
Satyrium spini|LEATI063-15|TLMF Lep 17448||Italy|613[0n]
Satyrium spini|LEATJ223-15|TLMF Lep 18653||Italy|658[0n]
Cigaritis acamas|EULEP2614-15|RVcoll.14-N554|Cyprus|Cyprus|658[0n]
Cigaritis acamas|EULEP2613-15|RVcoll.14-N553|Cyprus|Cyprus|658[0n]
Cigaritis acamas|EULEP2612-15|RVcoll.14-N552|Cyprus|Cyprus|658[0n]
Cigaritis acamas|EULEP2611-15|RVcoll.14-N551|Cyprus|Cyprus|658[0n]
Favonius quercus|WMB6282-18|RVcoll15F489||Portugal|658[0n]
Favonius quercus|WMB6273-18|RVcoll14B114||Portugal|658[0n]
Favonius quercus|EZSPM446-09|Rvcoll.09-V502||Spain|651[0n]
Favonius quercus|WMB6285-18|RVcoll15P436||Spain|658[0n]
Favonius quercus|WMB6271-18|RVcoll14A075||Portugal|658[0n]
Favonius quercus|EZSPC1094-10|RVcoll.08-L923||Spain|658[0n]
Favonius quercus|WMB6274-18|RVcoll14B115||Portugal|658[0n]
Favonius quercus|EZSPN555-09|RVcoll.08-J068|Genit. examined|Spain|639[0n]
Favonius quercus|EZRMN352-08|RVcoll.07-D699||Romania|623[0n]
Favonius quercus|EULEP4040-16|RVcoll15Q181||Ukraine|658[0n]
Favonius quercus|LOWA765-06|2005-LOWA-765||Ukraine|658[0n]
Favonius quercus|LOWA531-06|2005-LOWA-531||Ukraine|612[0n]
Favonius quercus|WMB2094-13|RVcoll.12-R095|Sicily|Italy|658[0n]
Favonius quercus|LEASS683-17|TLMF Lep 22331||Austria|658[0n]
Favonius quercus|EULEP1459-15|RVcoll.14-G209||Greece|658[0n]
Favonius quercus|EULEP1452-15|RVcoll.14-G173||Greece|658[0n]
Favonius quercus|EULEP375-14|MM23860||Finland|658[0n]
Favonius quercus|EZRMN327-08|RVcoll.08-H033||Romania|658[0n]
Favonius quercus|EZRMN307-08|RVcoll.08-M791||Romania|658[0n]
Favonius quercus|LEASS908-17|KLM Lep 08413||Austria|658[0n]
Favonius quercus|EULEP1373-15|RVcoll.14-F967||Greece|658[0n]
Favonius quercus|LEASS682-17|TLMF Lep 22330||Austria|658[0n]
Favonius quercus|WMB6275-18|RVcoll14C723|Genit. examined|Greece|658[0n]
Favonius quercus|LEFIA1145-10|MM04799||Finland|658[0n]
Favonius quercus|LEASS1027-17|TLMF Lep 22580||Austria|658[0n]
Favonius quercus|EULEP1412-15|RVcoll.14-G079||Greece|658[0n]
Favonius quercus|EZRMN134-08|RVcoll.08-M650||Romania|658[0n]
Favonius quercus|ABOLD610-17|TLMF Lep 21686||Austria|658[0n]
Favonius quercus|BIBSA071-14|LEP-SS-00071||Italy|658[0n]
Favonius quercus|BIBSA706-15|RVcoll. 14-A093||Italy|658[0n]
Favonius quercus|BIBSA702-15|RVcoll. 12-Q959||Italy|658[0n]
Favonius quercus|WMB709-12|RVcoll.11-H337|Sicily. Genit. examined|Italy|658[0n]
Favonius quercus|WMB6278-18|RVcoll15F391||Portugal|658[0n]
Favonius quercus|WMB6267-18|RVcoll10C644|Sicily|Italy|658[0n]
Favonius quercus|OXB1057-15|15-A602||Italy|658[0n]
Favonius quercus|WMB6292-18|RVcollLD2532|Sardinia|Italy|658[0n]
Favonius quercus|WMB6291-18|RVcollLD2495|Sardinia|Italy|658[0n]
Favonius quercus|WMB6061-18|RVcoll14E271|Corsica|France|658[0n]
[illegible]

Favonius quercus|WMB6292-18|RVcollLD2552|Sardinia|Italy|658[0n]
Favonius quercus|WMB6291-18|RVcollLD2495|Sardinia|Italy|658[0n]
Favonius quercus|WMB6061-18|RVcoll14E271|Corsica|France|658[0n]
Favonius quercus|WMB2598-13|RVcoll.12-R395|Elba|Italy|658[0n]
Favonius quercus|WMB2179-13|RVcoll.12-R350|Elba|Italy|658[0n]
Favonius quercus|WMB2178-13|RVcoll.12-R349|Elba. Genit. examined|Italy|658[0n]
Favonius quercus|WMB1898-13|RVcoll.11-J970|Sardinia. Genit. examined|Italy|658[0n]
Favonius quercus|WMB6293-18|RVcollLD2743||Italy|658[0n]
Favonius quercus|GWORA2471-09|BC ZSM Lep 30683||Germany|658[0n]
Favonius quercus|LENOA1410-11|LN-BD1404||France|609[2n]
Favonius quercus|WMB6060-18|RVcoll14D507||Italy|658[0n]
Favonius quercus|EZROM741-08|RV-06-G541|Genit. examined|Spain|650[0n]
Favonius quercus|BIBSA708-15|RVcoll. 14-A096||Italy|658[0n]
Favonius quercus|WMB6284-18|RVcoll15P435||Spain|658[0n]
Favonius quercus|WMB6283-18|RVcoll15K779||Spain|658[0n]
Favonius quercus|EZSPC1253-10|RVcoll.09-V847||Spain|658[0n]
Favonius quercus|WMB735-12|RVcoll.11-I857||France|635[0n]
Favonius quercus|CGUKA603-09|UKLB7D05||United Kingdom|658[0n]
Favonius quercus|WMB6277-18|RVcoll15D511||Spain|658[0n]
Favonius quercus|WMB6276-18|RVcoll15D510||Spain|658[0n]
Favonius quercus|WMB6272-18|RVcoll14A099||Italy|658[0n]
Favonius quercus|WMB6266-18|RVcoll08L925||Spain|658[0n]
Favonius quercus|WMB6063-18|RVcoll14E273|Corsica|France|658[0n]
Favonius quercus|WMB6062-18|RVcoll14E272|Corsica|France|658[0n]
Favonius quercus|WMB4005-14|RVcoll.12-Q552||Spain|658[0n]
Favonius quercus|WMB2416-13|RVcoll.10-C645|Sicily|Italy|658[0n]
Favonius quercus|WMB2130-13|RVcoll.12-R243|Genit. examined|Italy|658[0n]
Favonius quercus|WMB1783-13|RVcoll.12-Q147|Genit. examined|France|658[0n]
Favonius quercus|WMB832-13|RVcoll.07-E219||Italy|658[0n]
Favonius quercus|WMB6289-18|RVcoll15P717||Spain|658[0n]
Favonius quercus|WMB6287-18|RVcoll15P496||Spain|658[0n]
Favonius quercus|WMB6286-18|RVcoll15P495||Spain|658[0n]
Favonius quercus|WMB6281-18|RVcoll15F441||Portugal|658[0n]
Favonius quercus|WMB6280-18|RVcoll15F440||Portugal|658[0n]
Favonius quercus|WMB6279-18|RVcoll15F392||Portugal|658[0n]
Favonius quercus|WMB2749-13|RVcoll.10-C643|Sicily|Italy|658[0n]
Favonius quercus|WMB3493-14|RVcoll.10-B691||France|658[0n]
Favonius quercus|EZSPC1095-10|RVcoll.08-L926|Genit. examined|Spain|658[0n]
Favonius quercus|EZSPC1227-10|RVcoll.09-V594||Spain|658[0n]
Favonius quercus|EZSPN945-09|RVcoll.08-L924||Spain|658[0n]
Favonius quercus|EZSPM445-09|Rvcoll.09-V593||Spain|658[0n]
Favonius quercus|EZSPM290-09|RVcoll.08-J821||Spain|658[0n]
Favonius quercus|EZSPC565-09|RVcoll.07-F332||Spain|658[0n]
Favonius quercus|EZSPC564-09|RVcoll.08-R320||Spain|658[0n]
Favonius quercus|EZSPC566-09|RVcoll.08-P328||Spain|658[0n]
Favonius quercus|EZSPC563-09|RVcoll.08-R294||Spain|658[0n]
Favonius quercus|GWORA2472-09|BC ZSM Lep 30684||Germany|658[0n]
Favonius quercus|GWORA2470-09|BC ZSM Lep 30682||Germany|658[0n]
Favonius quercus|GWOSI557-10|BC ZSM Lep 44403||Germany|658[0n]
Favonius quercus|LEFIB330-10|MM00871||Finland|658[0n]
Favonius quercus|LEFIB001-10|MM00001||Finland|658[0n]
Favonius quercus|WMB6290-18|RVcoll16F779||Italy|658[0n]
Favonius quercus|WMB6294-18|RVcollLD2744||Italy|658[0n]
Favonius quercus|WMB734-12|RVcoll.11-I856||France|658[0n]
Favonius quercus|WMB5390-14|RVcoll.14-J871||France|658[0n]
Favonius quercus|EULEP376-14|MM23861||Finland|658[0n]
Favonius quercus|EULEP5697-17|RVcoll16L040||Italy|658[0n]
Favonius quercus|WMB6288-18|RVcoll15P499||Spain|658[0n]
Favonius quercus|WMB5316-14|RVcoll.14-I750||France|658[0n]
Favonius quercus|EULEP5658-17|RVcoll15K653||Italy|658[0n]
Favonius quercus|BIBSA747-15|LEP-SS-00248||Italy|658[0n]
Favonius quercus|EZSPC1030-10|RVcoll.08-J086||Spain|658[0n]
Favonius quercus|LEATI016-15|TLMF Lep 17401||Austria|658[0n]
Laeosopis roboris|EZSPM1033-12|Rvcoll.12-M783||Spain|658[0n]
Laeosopis roboris|EULEP247-14|RVcoll.12-N763||Spain|658[0n]
Laeosopis roboris|EZSPC1088-10|RVcoll.08-L850||Spain|635[0n]
Laeosopis roboris|EZSPN864-09|RVcoll.08-L735||Spain|627[0n]
Laeosopis roboris|EZSPN968-09|RVcoll.08-L985||Spain|658[0n]
Laeosopis roboris|EZSPN912-09|RVcoll.08-L849||Spain|658[0n]
Laeosopis roboris|EZSPM1034-12|Rvcoll.12-M784||Spain|658[0n]
Laeosopis roboris|EZSPM931-12|Rvcoll.12-M663||Spain|634[0n]
Laeosopis roboris|EZSPM980-12|Rvcoll.12-M742||Spain|658[0n]
Laeosopis roboris|EZSPM951-12|Rvcoll.12-M696||Spain|655[0n]
Laeosopis roboris|EZSPM843-12|Rvcoll. 140711ZG98||Spain|658[0n]
Laeosopis roboris|EZSPM834-12|Rvcoll. 140711ZG21||Spain|658[0n]
Laeosopis roboris|EZSPM793-12|Rvcoll. 070611MH72||Spain|658[0n]
Laeosopis roboris|WMB3309-14|RVcoll.08-P492||Spain|658[0n]
Laeosopis roboris|EZSPC1364-10|RVcoll.09-X023||Spain|658[0n]
Laeosopis roboris|EZSPM079-09|RVcoll.08-P362||Spain|658[0n]
Laeosopis roboris|EZSPM313-09|RVcoll.08-J868||Spain|658[0n]
Laeosopis roboris|EULEP2909-15|RVcoll.14-U862||France|621[0n]
Laeosopis roboris|EULEP2116-15|RVcoll.14-J781||France|624[0n]
Laeosopis roboris|EZSPC1191-10|RVcoll.09-V339||Spain|658[0n]
Laeosopis roboris|EZSPM024-09|RVcoll.08-P091||Spain|658[0n]
Laeosopis roboris|EZSPN478-09|RVcoll.08-H913||Spain|658[0n]
Thecla betulae|GWORO784-09|BC ZSM Lep 30476||Germany|658[0n]
Thecla betulae|EZRMN280-08|RVcoll.08-M651||Romania|658[0n]
Thecla betulae|EULEP474-14|KN00836||Russia|658[1n]
Thecla betulae|LEFIJ524-10|MM17149||Finland|658[0n]
Thecla betulae|LEFID253-10|MM06118||Finland|658[0n]
Thecla betulae|EZRMN308-08|RVcoll.08-M792||Romania|658[0n]
Thecla betulae|EULEP4665-16|RVcoll15P045||Ukraine|658[0n]
Thecla betulae|EULEP752-15|RVcoll.12-Q562||Sweden|658[0n]
Thecla betulae|EULEP620-15|RVcoll.11-H234||Germany|658[0n]
Thecla betulae|LON189-08|NHMO-06190||Norway|657[1n]
Thecla betulae|EZRMN281-08|RVcoll.08-M660||Romania|609[0n]
Thecla betulae|LOWA780-06|2005-LOWA-780||Russia|656[0n]
Thecla betulae|LEFIG409-10|MM14435||Finland|658[0n]
Thecla betulae|EULEP5613-17|RVcoll14V562||Spain|658[0n]
Thecla betulae|GBLAB133-13|BC ZSM Lep 75759||Germany|658[0n]
Thecla betulae|ABOLD606-17|TLMF Lep 21682||Austria|658[0n]
Thecla betulae|ODOPE751-11|BC ZSM Lep 50392||Germany|615[0n]
Thecla betulae|EZSPC330-09|RVcoll.08-L512||Spain|658[0n]

Thecla betulae|ABOLD606-17|TLMF Lep 21682||Austria|658[0n]
Thecla betulae|ODOPE751-11|BC ZSM Lep 50392||Germany|615[0n]
Thecla betulae|EZSPC330-09|RVcoll.08-L512||Spain|658[0n]
Thecla betulae|EZSPC331-09|RVcoll.08-P081||Spain|658[0n]
Thecla betulae|EZSPC1211-10|RVcoll.09-V501||Spain|658[0n]
Thecla betulae|WMB1833-13|RVcoll.11-I817||France|658[0n]
Thecla betulae|EULEP5077-16|RVcoll16J277||Germany|658[0n]
Thecla betulae|EULEP057-14|RVcoll.08-J487||Spain|658[0n]
Thecla betulae|EULEP058-14|RVcoll.08-J488||Spain|658[0n]
Thecla betulae|EULEP4963-16|RVcoll16I341||Poland|658[0n]
Thecla betulae|EULEP4991-16|RVcoll16I709||Poland|658[0n]
Thecla betulae|EULEP131-14|RVcoll.10-B440||Romania|658[0n]
Thecla betulae|EULEP206-14|RVcoll.12-L019||Portugal|658[0n]
Thecla betulae|EULEP211-14|RVcoll.12-L030||Spain|658[0n]
Thecla betulae|EULEP5614-17|RVcoll14V563||Spain|658[0n]
Thecla betulae|EULEP322-14|MM23807||Latvia|658[0n]
Thecla betulae|EULEP968-15|RVcoll.14-D041||Bulgaria|658[0n]
Thecla betulae|EULEP3147-15|RVcoll.14-O280||United Kingdom|658[0n]
Thecla betulae|BIBSA871-15|LD-3483||Italy|658[0n]
Thecla betulae|BIBSA872-15|LD-3484||Italy|658[0n]
Thecla betulae|EULEP5616-17|RVcoll14V565||Spain|658[0n]
Thecla betulae|EULEP5615-17|RVcoll14V564||Spain|658[0n]
Thecla betulae|EULEP213-14|RVcoll.12-L034||Spain|658[0n]
Thecla betulae|BIBSA874-15|LD-3486||Italy|658[0n]
Thecla betulae|LEASS904-17|KLM Lep 08409||Austria|658[0n]
Thecla betulae|EZROM552-08|RV-06-V715||Romania|652[0n]
Thecla betulae|EZSPC329-09|RVcoll.06-V724||Spain|653[0n]
Thecla betulae|EZRMN309-08|RVcoll.08-M793||Romania|611[0n]
Thecla betulae|EZRMN345-08|RVcoll.08-H042||Romania|649[0n]
Thecla betulae|EZRMN344-08|RVcoll.08-H041||Romania|649[0n]
Thecla betulae|LASTS333-14|TLMF Lep 14785||Austria|658[0n]
Thecla betulae|LEATG477-14|TLMF Lep 14264||Italy|658[0n]
Thecla betulae|LEATI006-15|TLMF Lep 17391||Austria|658[0n]
Thecla betulae|LEATG478-14|TLMF Lep 14265||Italy|658[0n]
Tongeia fischeri|EULEP428-14|KN00790||Russia|658[0n]
Cupido osiris|EULEP1664-15|RVcoll.14-G881||Greece|658[0n]
Cupido osiris|EULEP1803-15|RVcoll.14-H721||Greece|658[0n]
Cupido osiris|EULEP1346-15|RVcoll.14-F884||Greece|658[0n]
Cupido osiris|EULEP1500-15|RVcoll.14-G378||Greece|658[0n]
Cupido osiris|EZSPC1111-10|RVcoll.08-M939||Spain|658[0n]
Cupido osiris|EZSPN1015-09|RVcoll.08-M938||Spain|658[0n]
Cupido osiris|EZSPN1064-11|Rvcoll.08-P738||Spain|658[0n]
Cupido osiris|EZSPM801-12|Rvcoll. 090211SD04||Spain|658[0n]
Cupido osiris|EZSPM802-12|Rvcoll. 090211SD10||Spain|658[0n]
Cupido osiris|EULEP3746-16|RVcoll15H136||Italy|658[0n]
Cupido osiris|EULEP1851-15|RVcoll.13-T918||Italy|658[0n]
Cupido osiris|WMB673-12|RVcoll.09-V276||France|658[0n]
Cupido osiris|WMB670-12|RVcoll.07-E126||Italy|658[0n]
Cupido osiris|EULEP2155-15|RVcoll.14-K063||Switzerland|658[0n]
Cupido osiris|WMB280-11|RVcoll.10-A563||France|658[0n]
Cupido osiris|EZROM719-08|RV-07-C482||Spain|658[0n]
Cupido osiris|EZSPN065-09|RVcoll.09-V719||Spain|658[0n]
Cupido osiris|WMB3433-14|RVcoll.09-X316||Spain|658[0n]
Cupido osiris|EULEP1228-15|RVcoll.14-F531||Greece|658[0n]
Cupido osiris|EULEP1172-15|RVcoll.14-F393||Bulgaria|658[0n]
Cupido osiris|EULEP3743-16|RVcoll10A976||Bulgaria|658[0n]
Cupido osiris|EULEP2248-15|RVcoll.14-N431||Ukraine|658[0n]
Cupido osiris|EULEP089-14|RVcoll.08-P975||Romania|658[0n]
Cupido osiris|EULEP3747-16|RVcoll15Q197||Ukraine|658[0n]
Cupido osiris|EZROM312-08|RV-07-E674||Romania|658[0n]
Cupido osiris|EZROM901-08|RVcoll.08-M672||Romania|658[0n]
Cupido osiris|EZRMN383-09|RVcoll.08-L360||Romania|658[0n]
Cupido osiris|EZSPM778-12|Rvcoll. 050211XV70||Spain|658[0n]
Cupido osiris|EZSPN534-09|RVcoll.08-J010||Spain|658[0n]
Cupido osiris|EZSPN525-09|RVcoll.08-H995||Spain|658[0n]
Cupido osiris|EZSPC1112-10|RVcoll.08-M940||Spain|658[0n]
Cupido osiris|EZSPC371-09|RVcoll.08-H293||Spain|658[0n]
Cupido osiris|EZSPN057-09|RVcoll.09-V352||Spain|658[0n]
Cupido osiris|EZSPC370-09|RVcoll.07-C481||Spain|658[0n]
Cupido osiris|LOWA535-06|2005-LOWA-535||Ukraine|658[0n]
Cupido osiris|OXB766-15|14-A189||Italy|658[0n]
Cupido osiris|BIBSA637-15|RVcoll. 15-A800||Italy|633[0n]
Cupido osiris|BIBSA634-15|RVcoll. 15-A797||Italy|636[0n]
Cupido osiris|BIBSA633-15|RVcoll. 15-A796||Italy|658[0n]
Cupido osiris|EULEP3745-16|RVcoll15C492||Italy|658[0n]
Cupido osiris|WMB2041-13|RVcoll.12-Q764||Italy|658[0n]
Cupido osiris|EZSPC1413-10|RVcoll.09-V124||Spain|658[0n]
Cupido osiris|EZSPC1412-10|RVcoll.09-V123||Spain|658[0n]
Cupido osiris|EZSPC1411-10|RVcoll.09-V122||Spain|658[0n]
Cupido osiris|EZSPC1410-10|RVcoll.09-V121||Spain|658[0n]
Cupido osiris|EZSPN639-09|RVcoll.08-J767||Spain|658[0n]
Cupido osiris|EZSPN540-09|RVcoll.08-J032||Spain|658[0n]
Cupido osiris|EZSPN148-09|RVcoll.08-R049||Spain|658[0n]
Cupido osiris|EZSPN713-09|RVcoll.08-L074||Spain|658[0n]
Cupido osiris|EZRMN425-09|RVcoll.09-V674|Genit. examined|Romania|658[0n]
Cupido osiris|EZSPN1055-11|Rvcoll.08-M804||Spain|658[0n]
Cupido osiris|EZROM900-08|RVcoll.08-M567||Romania|658[0n]
Cupido osiris|EZROM137-08|RV-06-K599||Romania|658[0n]
Cupido osiris|EZROM899-08|RVcoll.08-M304||Romania|649[0n]
Cupido osiris|ABOLD647-17|TLMF Lep 21723||Austria|635[0n]
Cupido lorquinii|EZSPC728-10|RVcoll.08-H623||Spain|658[0n]
Cupido minimus|EULEP099-14|RVcoll.09-V160||Spain|658[0n]
Cupido minimus|EULEP098-14|RVcoll.09-V159||Spain|658[0n]
Cupido lorquinii|EZSPN795-09|RVcoll.08-L354||Spain|658[0n]
Cupido lorquinii|EZSPC745-10|RVcoll.08-L037||Spain|658[0n]
Cupido lorquinii|EZSPC821-10|RVcoll.08-P712||Spain|658[0n]
Cupido minimus|EZSPC831-10|RVcoll.08-R095||Spain|658[0n]
Cupido minimus|EZSPC832-10|RVcoll.08-R096||Spain|658[0n]
Cupido minimus|EZSPC833-10|RVcoll.08-R097||Spain|658[0n]
Cupido minimus|EZSPC1407-10|RVcoll.09-V118||Spain|658[0n]
Cupido lorquinii|EZSPC727-10|RVcoll.08-H621.1||Spain|658[0n]
Cupido minimus|EULEP101-14|RVcoll.09-V162||Spain|658[0n]
Cupido minimus|EZSPC830-10|RVcoll.08-R094||Spain|658[0n]

Cupido minimus|EZSPC1407-10|RVcoll.09-V118||Spain|658[0n]
Cupido lorquinii|EZSPC727-10|RVcoll.08-H621.1||Spain|658[0n]
Cupido minimus|EULEP101-14|RVcoll.09-V162||Spain|658[0n]
Cupido minimus|EZSPC830-10|RVcoll.08-R094||Spain|658[2n]
Cupido lorquinii|EZSPM606-12|RVcoll.11-D612||Spain|658[0n]
Cupido lorquinii|EZSPN793-09|RVcoll.08-L352||Spain|658[0n]
Cupido lorquinii|WMB3544-14|RVcoll.11-D816||Spain|658[0n]
Cupido lorquinii|EZSPN440-09|RVcoll.08-H622||Spain|636[0n]
Cupido lorquinii|EZSPM610-12|RVcoll.11-D682||Spain|658[0n]
Cupido lorquinii|EZSPC744-10|RVcoll.08-L036||Spain|635[0n]
Cupido lorquinii|EZSPC743-10|RVcoll.08-L035||Spain|658[0n]
Cupido lorquinii|EZSPN684-09|RVcoll.08-L009||Spain|658[0n]
Cupido lorquinii|EZSPN692-09|RVcoll.08-L023||Spain|636[0n]
Cupido lorquinii|WMB006-11|RVcoll.11-D831||Spain|658[0n]
Cupido lorquinii|EZSPC737-10|RVcoll.08-J206||Spain|658[0n]
Cupido lorquinii|EZSPN794-09|RVcoll.08-L353||Spain|658[0n]
Cupido lorquinii|EZSPC766-10|RVcoll.08-L674||Spain|658[0n]
Cupido minimus|EZSPC1446-10|RVcoll.09-V158||Spain|606[0n]
Cupido minimus|EULEP100-14|RVcoll.09-V161||Spain|658[0n]
Cupido lorquinii|EZSPN697-09|RVcoll.08-L034||Spain|640[0n]
Cupido lorquinii|EZSPN842-09|RVcoll.08-L673||Spain|658[0n]
Cupido minimus|WMB2939-14|RVcoll.12-M338|Sicily|Italy|658[0n]
Cupido minimus|WMB486-11|RVcoll.11-H531|Sicily|Italy|658[0n]
Cupido minimus|BIBSA1590-16|12-M588||Italy|658[0n]
Cupido minimus|BIBSA1573-16|11-I094||Italy|658[0n]
Cupido minimus|WMB610-11|RVcoll.11-I013|Sicily|Italy|658[0n]
Cupido minimus|WMB559-11|RVcoll.11-H752|Sicily|Italy|658[0n]
Cupido minimus|WMB150-11|RVcoll.11-D246|Sicily|Italy|658[0n]
Cupido minimus|EULEP3738-16|RVcoll15I051||Italy|658[0n]
Cupido minimus|EULEP1650-15|RVcoll.14-F683||Greece|658[0n]
Cupido minimus|EULEP1861-15|RVcoll.13-U261||Italy|658[0n]
Cupido minimus|EULEP2012-15|RVcoll.14-I802||Slovakia|658[0n]
Cupido minimus|BIBSA1356-15|15-M859||Italy|658[0n]
Cupido minimus|BIBSA1651-16|15-C634||Italy|658[0n]
Cupido minimus|OXB705-15|13-T882||Italy|658[0n]
Cupido minimus|EULEP1559-15|RVcoll.14-G555||Macedonia|614[0n]
Cupido minimus|EULEP900-15|RVcoll.14-C731||Bulgaria|658[0n]
Cupido minimus|WMB4177-14|RVcoll.13-S778||Italy|658[0n]
Cupido minimus|ABOLD431-16|TLMF Lep 21583||Austria|658[0n]
Cupido minimus|EULEP3737-16|RVcoll15H539||Switzerland|658[0n]
Cupido minimus|EULEP4781-16|RVcoll16H341||Norway|658[0n]
Cupido minimus|EZROM133-08|RV-06-N004|Genit. examined|Romania|658[0n]
Cupido minimus|LENOA1375-11|LN-BD1375||France|658[0n]
Cupido minimus|WMB4861-14|RVcoll.14-I408||Italy|658[0n]
Cupido minimus|WMB2344-13|RVcoll.12-M488||Italy|658[0n]
Cupido minimus|EZSPN425-09|RVcoll.08-H591||Spain|658[0n]
Cupido minimus|EZSPM810-12|Rvcoll. 130211WX60||Spain|658[0n]
Cupido minimus|GBLAA1364-15|BC ZSM Lep 87060||Germany|658[0n]
Cupido minimus|GBLAD265-14|BC ZSM Lep 78836||Germany|658[0n]
Cupido minimus|ABOLD088-16|TLMF Lep 21166||Austria|658[0n]
Cupido minimus|WMB2345-13|RVcoll.12-M489||Italy|658[0n]
Cupido minimus|OXB625-15|14-O014||Italy|658[0n]
Cupido minimus|LEATF113-14|TLMF Lep 13425||Italy|658[0n]
Cupido minimus|EULEP5079-16|RVcoll16J314||Germany|658[0n]
Cupido minimus|FBLMU452-09|BC ZSM Lep 27102||Germany|658[0n]
Cupido minimus|GBMIN32765-13|GQ128947||Russia|1493[63n]
Cupido minimus|EULEP1671-15|RVcoll.14-G909||Greece|658[0n]
Cupido minimus|BIBSA056-14|LEP-SS-00056||Italy|658[0n]
Cupido minimus|EZROM135-08|RV-07-C941||Romania|658[0n]
Cupido minimus|EZROM136-08|RV-07-E525||Romania|658[0n]
Cupido minimus|GWORZ048-10|BC ZSM Lep 30404||Italy|658[0n]
Cupido minimus|LON084-08|NHMO-06084||Norway|657[0n]
Cupido minimus|LEFIF123-10|MM10579||Finland|658[0n]
Cupido minimus|LON962-12|NHMO Lep2011.022||Norway|658[0n]
Cupido minimus|EZROM320-08|RV-07-E682||Romania|658[0n]
Cupido minimus|EZROM321-08|RV-07-E683||Romania|658[0n]
Cupido minimus|EZSPC369-09|RVcoll.08-H291||Spain|658[0n]
Cupido minimus|WMB004-11|RVcoll.11-D667||Spain|658[0n]
Cupido minimus|WMB005-11|RVcoll.11-D668||Spain|658[0n]
Cupido minimus|EZSPM765-12|Rvcoll. 040611HZ26||Spain|658[0n]
Cupido minimus|EZSPM599-12|RVcoll.11-D670||Spain|658[0n]
Cupido minimus|EZSPM600-12|RVcoll.11-D671||Spain|658[0n]
Cupido minimus|EZSPM608-12|RVcoll.11-D675||Spain|658[0n]
Cupido minimus|EZSPN536-09|RVcoll.08-J018||Spain|658[0n]
Cupido minimus|EZSPN636-09|RVcoll.08-J758||Spain|658[0n]
Cupido minimus|EZSPN648-09|RVcoll.08-J789||Spain|658[0n]
Cupido minimus|EZSPM272-09|RVcoll.08-R489||Spain|658[0n]
Cupido minimus|EZSPM809-12|Rvcoll. 130211WX11||Spain|658[0n]
Cupido minimus|EZSPM782-12|Rvcoll. 070111GN93||Spain|658[0n]
Cupido minimus|EZSPM785-12|Rvcoll. 070211PL52||Spain|658[0n]
Cupido minimus|EZSPC819-10|RVcoll.08-P706||Spain|658[0n]
Cupido minimus|EZSPC820-10|RVcoll.08-P707||Spain|658[0n]
Cupido minimus|EZSPC858-10|RVcoll.08-R490||Spain|658[0n]
Cupido minimus|EZSPC859-10|RVcoll.08-R491||Spain|658[0n]
Cupido minimus|WMB254-11|RVcoll.09-V281||France|658[0n]
Cupido minimus|WMB3454-14|RVcoll.10-A513||France|658[0n]
Cupido minimus|WMB3462-14|RVcoll.10-A607||France|658[0n]
Cupido minimus|WMB1652-13|RVcoll.12-P019||France|658[0n]
Cupido minimus|WMB1709-13|RVcoll.12-P655||France|658[0n]
Cupido minimus|WMB4550-14|RVcoll.070111GN07||Spain|658[0n]
Cupido minimus|WMB4029-14|RVcoll.12-Z144||Ireland|658[0n]
Cupido minimus|WMB5375-14|RVcoll.14-J777||France|658[0n]
Cupido minimus|EULEP3736-16|RVcoll10A753|Genit. examined|Estonia|658[0n]
Cupido minimus|EULEP1239-15|RVcoll.14-F565||Greece|658[0n]
Cupido minimus|EULEP1795-15|RVcoll.14-H667||Greece|658[0n]
Cupido minimus|EULEP2400-15|RVcoll.14-V107||Ukraine|658[0n]
Cupido minimus|EULEP1934-15|RVcoll.14-B905||Bosnia and Herzegovina|658[0n]
Cupido minimus|EULEP2179-15|RVcoll.14-N215||Greece|658[0n]
Cupido minimus|BIBSA622-15|LEP-SS-00242||Italy|658[0n]
Cupido minimus|BIBSA664-15|RVcoll. 15-A827||Italy|658[0n]
Cupido minimus|BIBSA1748-16|11-I215||Italy|658[0n]
Cupido minimus|GBLAA1684-15|BC ZSM Lep 80065|LF Schumacher|Germany|658[0n]
Cupido minimus|GBLAA1685-15|BC ZSM Lep 80066|LF Schumacher|Germany|658[0n]

Cupido minimus|BIBSA1748-16|11-I215||Italy|658[0n]
Cupido minimus|GBLAA1684-15|BC ZSM Lep 80065|LF Schumacher|Germany|658[0n]
Cupido minimus|GBLAA1685-15|BC ZSM Lep 80066|LF Schumacher|Germany|658[0n]
Cupido minimus|GBLAB120-13|BC ZSM Lep 75746||Germany|658[0n]
Cupido minimus|EZROM898-08|RVcoll.08-M349||Romania|658[0n]
Cupido minimus|GBLAC745-13|BC ZSM Lep 77796||Germany|658[0n]
Cupido minimus|OXB285-15|OXB-TGS-968||United Kingdom|658[0n]
Cupido minimus|OXB1373-15|Rvcoll_15-M737||France|658[0n]
Cupido minimus|EZROM622-08|RV-07-D521||Romania|652[0n]
Cupido minimus|EULEP4727-16|RVcoll16G402||Denmark|637[0n]
Cupido minimus|EZSPN626-09|RVcoll.08-J716||Spain|640[0n]
Cupido minimus|EZROM347-08|RV-08-A025||Romania|609[0n]
Cupido minimus|OXB1293-15|Rvcoll_15-M158||France|612[0n]
Cupido minimus|EZROM345-08|RV-08-A023||Romania|609[0n]
Cupido minimus|EULEP2213-15|RVcoll.14-N390||Ukraine|601[0n]
Cupido minimus|LEFIF124-10|MM10580||Finland|622[0n]
Cupido minimus|LENOA1376-11|LN-BD1376||France|621[0n]
Cupido minimus|OXB270-15|OXB-TGS-953||United Kingdom|658[0n]
Cupido minimus|LEFIL356-10|MM18666||Finland|658[0n]
Cupido minimus|GBLAD950-14|BC ZSM Lep 84556||Germany|658[0n]
Cupido minimus|PHLAB299-10|TLMF Lep 01099||Switzerland|658[0n]
Cupido minimus|BIBSA380-15|RVcoll.14-I048||Italy|639[0n]
Cupido minimus|EULEP2170-15|RVcoll.14-K098||Switzerland|623[0n]
Cupido minimus|EULEP3742-16|RVcoll15G258||Switzerland|634[0n]
Cupido minimus|WMB3141-14|RVcoll.07-C022||France|658[0n]
Cupido minimus|EZSPN257-09|RVcoll.07-W160||Spain|627[0n]
Cupido minimus|EZSPC368-09|RVcoll.08-R113||Spain|658[0n]
Cupido minimus|EZSPC367-09|RVcoll.08-M911||Spain|658[0n]
Cupido minimus|GBLAA1686-15|BC ZSM Lep 80067|07.06.2014 LF Schumacher|Germany|658[0n]
Cupido minimus|BIBSA688-15|RVcoll. 11-I214||Italy|658[0n]
Cupido minimus|EZROM134-08|RV-07-D920|Genit. examined|Romania|658[0n]
Cupido minimus|FBLMT889-09|BC ZSM Lep 25449||Germany|658[0n]
Cupido minimus|LEATG417-14|TLMF Lep 14204||Italy|658[0n]
Cupido minimus|OXB1004-15|15-A954||Italy|658[0n]
Cupido minimus|OXB338-15|RVcoll.14-N052||Italy|658[0n]
Cupido minimus|OXB920-15|15-A564||Italy|658[0n]
Cupido minimus|OXB919-15|15-A563||Italy|658[0n]
Cupido minimus|OXB580-15|14-N969||Italy|658[0n]
Cupido minimus|BIBSA379-15|RVcoll.14-I047||Italy|658[0n]
Cupido minimus|BIBSA1847-17|RVcoll16C725||Italy|658[0n]
Cupido minimus|BIBSA964-15|13-U337||Italy|658[0n]
Cupido minimus|EULEP3741-16|RVcoll15G126||France|658[0n]
Cupido minimus|EULEP3740-16|RVcoll15I811||Austria|658[0n]
Cupido minimus|EULEP3739-16|RVcoll15I218||Austria|658[0n]
Cupido minimus|WMB5224-14|RVcoll.13-T980||Italy|658[0n]
Cupido minimus|WMB4941-14|RVcoll.14-I488||Italy|658[0n]
Cupido minimus|WMB4127-14|RVcoll.13-S610||Italy|658[0n]
Cupido minimus|WMB2220-13|RVcoll.11-Y045||Italy|658[0n]
Cupido minimus|WMB1974-13|RVcoll.11-I212||Italy|658[0n]
Cupido minimus|WMB2689-13|RVcoll.10-B997||France|658[0n]
Cupido minimus|WMB3369-14|RVcoll.09-T241||Spain|658[0n]
Cupido minimus|EZSPC278-09|RVcoll.06-G465||Spain|658[0n]
Cupido minimus|EZSPC366-09|RVcoll.07-C630||Spain|658[0n]
Cupido minimus|EZROM718-08|RV-06-G445||Spain|658[0n]
Cupido minimus|GWORO792-09|BC ZSM Lep 30484||Germany|658[0n]
Cupido minimus|GWOSZ100-11|BC ZSM Lep 41856||Italy|658[0n]
Cupido minimus|GWOSF851-10|BC ZSM Lep 45837||Germany|658[0n]
Cupido minimus|GWORL438-09|BC ZSM Lep 22340||Germany|658[0n]
Cupido minimus|GWORA2462-09|BC ZSM Lep 30674||Austria|658[0n]
Cupido minimus|LEATG470-14|TLMF Lep 14257||Italy|658[0n]
Cupido minimus|PHLAH453-12|TLMF Lep 08272||Austria|658[0n]
Cupido minimus|LEATH471-14|TLMF Lep 15683||Italy|658[0n]
Cupido minimus|LEATF114-14|TLMF Lep 13426||Italy|658[0n]
Cupido minimus|LEATD095-13|TLMF Lep 12742||Austria|658[0n]
Cupido argiades|GBLAC380-13|BC ZSM Lep 75246||Germany|658[0n]
Cupido argiades|GWOSF853-10|BC ZSM Lep 45839||Germany|658[0n]
Cupido argiades|BIBSA1814-17|RVcoll14I283||Italy|658[0n]
Cupido argiades|LEASS521-17|TLMF Lep 22169||Austria|658[0n]
Cupido argiades|OXB1594-16|OXB-TGS-1312||France|658[0n]
Cupido argiades|EZROM998-08|RVcoll.07-D902||Romania|658[0n]
Cupido argiades|EZROM582-08|RV-07-C335||Romania|658[0n]
Cupido argiades|EZROM999-08|RVcoll.08-M447||Romania|658[0n]
Cupido argiades|EZROM997-08|RVcoll.07-D580||Romania|658[0n]
Cupido argiades|EZSPC416-09|RVcoll.08-R291||Spain|658[0n]
Cupido argiades|EZROM188-08|RV-06-K543||Romania|657[0n]
Cupido argiades|EZROM189-08|RV-06-K545|Genit. examined|Romania|658[0n]
Cupido argiades|EZROM190-08|RV-06-M930|Genit. examined|Romania|658[0n]
Cupido argiades|EZROM192-08|RV-07-C951||Romania|658[0n]
Cupido argiades|EZSPC412-09|RVcoll.08-M707||Spain|658[0n]
Cupido argiades|EZSPC413-09|RVcoll.08-R270||Spain|658[0n]
Cupido argiades|EZSPC414-09|RVcoll.08-R271||Spain|658[0n]
Cupido argiades|EZSPM402-09|Rvcoll.09-V803||Spain|658[0n]
Cupido argiades|EZSPN1088-11|Rvcoll.09-X569||Spain|658[0n]
Cupido argiades|EZSPM104-09|RVcoll.08-P438||Spain|658[0n]
Cupido argiades|EZSPM134-09|RVcoll.08-P644||Spain|658[0n]
Cupido argiades|EZSPM250-09|RVcoll.08-R462||Spain|658[0n]
Cupido argiades|EZSPM409-09|Rvcoll.09-V826||Spain|658[0n]
Cupido argiades|EZSPM132-09|RVcoll.08-P641||Spain|658[0n]
Cupido argiades|WMB3705-14|RVcoll.12-L296||Spain|658[0n]
Cupido argiades|WMB2708-13|RVcoll.10-C166||France|658[0n]
Cupido argiades|WMB1657-13|RVcoll.12-P083||France|658[0n]
Cupido argiades|WMB1837-13|RVcoll.11-I867||France|658[0n]
Cupido argiades|WMB4002-14|RVcoll.12-Q519||Spain|658[0n]
Cupido argiades|WMB5094-14|RVcoll. 14-L225||Italy|658[0n]
Cupido argiades|EULEP4039-16|RVcoll15P029||Belarus|658[0n]
Cupido argiades|EULEP3730-16|RVcoll15J246||Switzerland|658[0n]
Cupido argiades|EULEP3731-16|RVcoll15G052||France|658[0n]
Cupido argiades|EULEP3732-16|RVcoll15G127||France|658[0n]
Cupido argiades|EULEP3733-16|RVcoll15Q108||Russia|658[0n]
Cupido argiades|EULEP5026-16|RVcoll16I956||Slovakia|658[0n]
Cupido argiades|EULEP5033-16|RVcoll16I982||Czech Republic|658[0n]
Cupido argiades|EULEP4899-16|RVcoll16J602||Russia|658[0n]
Cupido argiades|EULEP4964-16|RVcoll16J342||Poland|658[0n]

[illegible]
Cupido argiades|EULEP5033-16|RVcoll16I982||Czech Republic|658[0n]
Cupido argiades|EULEP4899-16|RVcoll16J602||Russia|658[0n]
Cupido argiades|EULEP4964-16|RVcoll16I342||Poland|658[0n]
Cupido argiades|EULEP366-14|MM23851||Lithuania|658[0n]
Cupido argiades|EULEP367-14|MM23852||Lithuania|658[0n]
Cupido argiades|EULEP2193-15|RVcoll.14-N333||Ukraine|658[0n]
Cupido argiades|EULEP2336-15|RVcoll.14-V021||Ukraine|658[0n]
Cupido argiades|BIBSA1130-15|15-L939||Italy|658[0n]
Cupido argiades|BIBSA1182-15|15-M222||Italy|658[0n]
Cupido argiades|BIBSA914-15|16-A005||France|658[0n]
Cupido argiades|BIBSA1378-15|15-M953||Italy|658[0n]
Cupido argiades|BIBSA447-15|RVcoll.14-I115||Italy|658[0n]
Cupido argiades|BIBSA1886-17|LZCollB17A009||Italy|658[0n]
Cupido argiades|BIBSA767-15|LEP-SS-00278||Italy|658[0n]
Cupido argiades|BIBSA461-15|RVcoll.14-I129||Italy|658[0n]
Cupido argiades|GBLAC378-13|BC ZSM Lep 75244||Germany|658[0n]
Cupido argiades|GBLAC379-13|BC ZSM Lep 75245||Germany|658[0n]
Cupido argiades|BIBSA460-15|RVcoll.14-I128||Italy|658[0n]
Cupido argiades|BIBSA069-14|LEP-SS-00069||Italy|658[0n]
Cupido argiades|BIBSA070-14|LEP-SS-00070||Italy|658[0n]
Cupido argiades|BIBSA1880-17|LZCollB17A003||Italy|658[0n]
Cupido argiades|BIBSA1882-17|LZCollB17A005||Italy|658[0n]
Cupido argiades|BIBSA1883-17|LZCollB17A006||Italy|658[0n]
Cupido argiades|BIBSA1885-17|LZCollB17A008||Italy|658[0n]
Cupido argiades|GBLAF779-14|BC ZSM Lep 82865||Germany|658[0n]
Cupido argiades|GBLAA164-14|BC ZSM Lep 80635||Germany|658[0n]
Cupido argiades|GBLAC300-13|BC ZSM Lep 75166||Germany|658[0n]
Cupido argiades|LEASS1058-17|TLMF Lep 22611||Austria|658[0n]
Cupido argiades|EZSPN526-09|RVcoll.08-H996||Spain|658[0n]
Cupido argiades|OXB589-15|14-N978||Italy|658[0n]
Cupido argiades|OXB981-15|15-A931||Italy|658[0n]
Cupido argiades|OXB1556-16|OXB-TGS-1274||France|658[0n]
Cupido argiades|OXB1370-15|Rvcoll_15-M732||France|658[0n]
Cupido argiades|OXB1402-15|Rvcoll_15-M771||France|658[0n]
Cupido argiades|BIBSA1887-17|LZCollB17A010||Italy|630[0n]
Cupido argiades|BIBSA766-15|LEP-SS-00277||Italy|633[0n]
Cupido argiades|EULEP2414-15|RVcoll.14-V189||Belgium|617[0n]
Cupido argiades|EULEP4852-16|RVcoll16H778||Bulgaria|623[0n]
Cupido argiades|EZSPC415-09|RVcoll.08-R272||Spain|632[0n]
Cupido argiades|EZROM191-08|RV-07-D957||Romania|658[0n]
Cupido argiades|LEFIF125-10|MM10581||Finland|658[0n]
Cupido argiades|LEFIF126-10|MM10582||Finland|658[0n]
Cupido argiades|ABOLD635-17|TLMF Lep 21711||Austria|658[0n]
Cupido argiades|GWOSI555-10|BC ZSM Lep 44401||Germany|658[0n]
Cupido argiades|GWOSF855-10|BC ZSM Lep 45841||Austria|658[0n]
Cupido argiades|GWOSF852-10|BC ZSM Lep 45838||Germany|658[0n]
Cupido argiades|GWOTD805-12|BC ZSM Lep 64696||Croatia|658[0n]
Cupido argiades|GWORR415-10|BC ZSM Lep 29631||Italy|658[0n]
Cupido argiades|ABOLD430-16|TLMF Lep 21582||Austria|658[0n]
Cupido argiades|ABOLD429-16|TLMF Lep 21581||Austria|658[0n]
Cupido argiades|ABOLD036-16|TLMF Lep 21114||Austria|658[0n]
Cupido alcetas|EZROM1063-09|RVcoll.07-F559|Genit. examined|Romania|658[0n]
Cupido alcetas|EULEP5136-17|RVcoll16J892||Serbia|658[0n]
Cupido alcetas|EULEP2529-15|RVcoll.14-V404||Ukraine|658[0n]
Cupido alcetas|EZRMN407-09|RVcoll.07-D210||Romania|639[0n]
Cupido alcetas|EZRMN409-09|RVcoll.07-E458|Genit. examined|Romania|639[0n]
Cupido alcetas|EZRMN406-09|RVcoll.07-E526||Romania|646[0n]
Cupido decoloratus|EZROM194-08|RV-07-D308|Genit. examined|Romania|652[0n]
Cupido alcetas|OXB1106-15|15-A639||Italy|658[0n]
Cupido alcetas|EZSPC1249-10|RVcoll.09-V824||Spain|658[0n]
Cupido alcetas|WMB5298-14|RVcoll.14-I605||France|658[0n]
Cupido alcetas|EULEP1985-15|RVcoll.14-H964||France|658[0n]
Cupido alcetas|EZSPC1210-10|RVcoll.09-V500||Spain|633[0n]
Cupido alcetas|EZSPM407-09|Rvcoll.09-V823||Spain|658[0n]
Cupido alcetas|EZSPC411-09|RVcoll.08-P058||Spain|658[0n]
Cupido alcetas|EZSPC408-09|RVcoll.08-R286||Spain|658[0n]
Cupido alcetas|EZSPC407-09|RVcoll.07-E644||Spain|658[0n]
Cupido alcetas|EZSPC410-09|RVcoll.07-E639||Spain|658[0n]
Cupido alcetas|EZSPC409-09|RVcoll.08-R305||Spain|658[0n]
Cupido alcetas|EZSPN178-09|RVcoll.06-V729||Spain|658[0n]
Cupido alcetas|WMB4985-14|RVcoll. 14-I532||Italy|658[0n]
Cupido alcetas|WMB6555-18|RVcoll14A090||Italy|658[0n]
Cupido decoloratus|EZROM1072-09|RVcoll.08-L364|Genit. examined|Romania|658[0n]
Cupido decoloratus|EZROM343-08|RV-08-A021||Romania|658[0n]
Cupido alcetas|WMB5193-14|RVcoll.13-T803||Italy|658[0n]
Cupido alcetas|WMB5102-14|RVcoll. 14-L233||Italy|658[0n]
Cupido decoloratus|EZROM1000-08|RVcoll.08-M565|Genit. examined|Romania|658[0n]
Cupido decoloratus|EZROM1045-09|RVcoll.07-D109.1||Romania|658[0n]
Cupido decoloratus|EULEP2194-15|RVcoll.14-N334||Ukraine|658[0n]
Cupido decoloratus|EULEP2475-15|RVcoll.14-V329|Genit. examined|Ukraine|658[0n]
Cupido decoloratus|EULEP2401-15|RVcoll.14-V109||Ukraine|658[0n]
Cupido alcetas|EULEP2143-15|RVcoll.14-J999||Switzerland|658[0n]
Cupido alcetas|EULEP818-15|RVcoll.14-A317||Italy|658[0n]
Cupido alcetas|EULEP614-15|RVcoll.11-H125||Switzerland|658[0n]
Cupido alcetas|EULEP5049-16|RVcoll16J050||Czech Republic|658[0n]
Cupido decoloratus|EULEP3734-16|RVcoll15Q040||Serbia|658[0n]
Cupido alcetas|EULEP3728-16|RVcoll15J655||France|658[0n]
Cupido alcetas|EULEP3727-16|RVcoll15J138||Switzerland|658[0n]
Cupido decoloratus|EZROM193-08|RV-06-K610|Genit. examined|Romania|658[0n]
Cupido decoloratus|EZROM996-08|RVcoll.08-M655||Romania|658[0n]
Cupido alcetas|WMB2186-13|RVcoll.07-E092||Italy|658[0n]
Cupido alcetas|WMB2011-13|RVcoll.12-Q638||Italy|658[0n]
Cupido alcetas|WMB5493-14|RVcoll.LD-3367||Italy|658[0n]
Cupido alcetas|WMB5248-14|RVcoll.14-A213||Italy|658[0n]
Cupido alcetas|EZSPM363-09|RVcoll.08-J824||Spain|658[0n]
Cupido alcetas|EZSPM292-09|RVcoll.08-J823||Spain|658[0n]
Cupido alcetas|WMB2703-13|RVcoll.10-C112||France|658[0n]
Cupido alcetas|WMB3374-14|RVcoll.09-V205||France|658[0n]
Cupido alcetas|WMB940-13|RVcoll.10-A618||France|658[0n]
Cupido alcetas|WMB815-13|RVcoll.07-E150||Italy|658[0n]
Cupido alcetas|WMB4692-14|RVcoll.LD-2755||Italy|658[0n]
Cupido alcetas|WMB4224-14|RVcoll.13-T761||Italy|658[0n]
[illegible]

Cupido alcetas|WMB815-13|RVcoll.07-E150||Italy|658[0n]
Cupido alcetas|WMB4692-14|RVcoll.LD-2755||Italy|658[0n]
Cupido alcetas|WMB4224-14|RVcoll.13-T761||Italy|658[0n]
Cupido decoloratus|EULEP2569-15|RVcoll.14-V544||Ukraine|658[0n]
Cupido decoloratus|EULEP2341-15|RVcoll.14-V027||Ukraine|658[0n]
Cupido alcetas|BIBSA1413-15|15-N027||Italy|658[0n]
Cupido alcetas|BIBSA1053-15|15-M987||Italy|658[0n]
Cupido alcetas|BIBSA1226-15|15-L964||Italy|658[0n]
Cupido decoloratus|EULEP2570-15|RVcoll.14-V546||Ukraine|658[0n]
Cupido alcetas|GWORZ047-10|BC ZSM Lep 30403||Italy|658[0n]
Cupido alcetas|GWORZ046-10|BC ZSM Lep 30402||Italy|658[0n]
Cupido decoloratus|ABOLD634-17|TLMF Lep 21710||Austria|658[0n]
Cupido decoloratus|ABOLD633-17|TLMF Lep 21709||Austria|658[0n]
Cupido alcetas|BIBSA751-15|LEP-SS-00252||Italy|658[0n]
Cupido alcetas|BIBSA752-15|LEP-SS-00253||Italy|658[0n]
Cupido alcetas|OXB771-15|14-A406||Italy|658[0n]
Cupido alcetas|OXB871-15|15-A513||Italy|658[0n]
Cupido alcetas|OXB884-15|15-A526||Italy|658[0n]
Cupido alcetas|OXB1136-15|15-A669||Italy|658[0n]
Cupido decoloratus|EZROM195-08|RV-07-C123|Genit. examined|Romania|658[0n]
Cupido alcetas|OXB963-15|15-A913||Italy|658[0n]
Cupido alcetas|OXB1593-16|OXB-TGS-1311||France|658[0n]
Cupido alcetas|OXB1279-15|Rvcoll_15-M139||France|658[0n]
Cupido alcetas|OXB980-15|15-A930||Italy|658[0n]
Cupido decoloratus|EZROM329-08|RV-08-A007||Romania|609[0n]
Cupido alcetas|OXB1257-15|Rvcoll_15-M113||France|621[0n]
Cupido alcetas|GWORR419-10|BC ZSM Lep 29635||Italy|636[0n]
Cupido alcetas|LEATJ208-15|TLMF Lep 18638||Italy|658[0n]
Libythea celtis|EULEP5589-17|RVcoll14N440||Ukraine|655[0n]
Libythea celtis|EZRMN028-08|RVcoll.08-M278||Romania|658[0n]
Libythea celtis|EZRMN027-08|RVcoll.08-M264||Romania|658[0n]
Libythea celtis|EZRMN024-08|RVcoll.08-M259||Romania|658[0n]
Libythea celtis|EULEP5577-17|RVcoll14H058||Greece|655[0n]
Libythea celtis|EULEP5543-17|RVcoll14C158||Greece|655[0n]
Libythea celtis|EULEP5471-17|RVcoll12N836|Lesvos|Greece|655[0n]
Libythea celtis|EULEP5570-17|RVcoll14G116||Greece|655[0n]
Libythea celtis|EZRMN025-08|RVcoll.08-M262||Romania|658[0n]
Libythea celtis|EZRMN026-08|RVcoll.08-M263||Romania|658[0n]
Libythea celtis|EULEP5599-17|RVcoll14U821||Hungary|658[0n]
Libythea celtis|EULEP5600-17|RVcoll14U823||Hungary|658[0n]
Libythea celtis|EULEP5608-17|RVcoll14U885||Romania|658[0n]
Libythea celtis|EULEP5609-17|RVcoll14U886||Romania|658[0n]
Libythea celtis|EULEP2917-15|RVcoll.14-U870||Albania|658[0n]
Libythea celtis|ABOLD571-17|2015_DR_LC6||Austria|658[0n]
Libythea celtis|WMB506-11|RVcoll.11-H572|Sicily|Italy|633[0n]
Libythea celtis|EZSPC482-09|RVcoll.08-J379||Spain|617[0n]
Libythea celtis|WMB4584-14|RVcoll.150308PP77||Spain|658[0n]
Libythea celtis|EZSPN552-09|RVcoll.08-J059||Spain|658[0n]
Libythea celtis|EZSPC1015-10|RVcoll.08-H606||Spain|658[0n]
Libythea celtis|EZSPM617-12|RVcoll.11-D839||Spain|658[0n]
Libythea celtis|WMB4798-14|RVcoll. 14-I345||Italy|658[0n]
Libythea celtis|WMB635-11|RVcoll.11-I132||Italy|658[0n]
Libythea celtis|WMB1962-13|RVcoll.11-I101|Sicily|Italy|658[0n]
Libythea celtis|WMB2096-13|RVcoll.12-R103|Sicily|Italy|658[0n]
Libythea celtis|BIBSA588-15|LEP-SS-00208||Italy|658[0n]
Libythea celtis|BIBSA587-15|LEP-SS-00207||Italy|658[0n]
Libythea celtis|WMB1591-13|RVcoll.12-O594|Sardinia|Italy|658[0n]
Libythea celtis|WMB1576-13|RVcoll.12-O523|Sardinia|Italy|658[0n]
Libythea celtis|WMB1569-13|RVcoll.12-O499|Sardinia|Italy|658[0n]
Libythea celtis|WMB1515-13|RVcoll.12-O122|Corsica|France|658[0n]
Libythea celtis|BIBSA1536-16|10-C641||Italy|658[0n]
Libythea celtis|BIBSA635-15|RVcoll. 15-A798||Italy|658[0n]
Libythea celtis|BIBSA1645-16|15-C582||Italy|658[0n]
Libythea celtis|BIBSA1001-15|15-C062||Italy|658[0n]
Libythea celtis|EULEP5607-17|RVcoll14U884||Romania|658[0n]
Libythea celtis|EULEP5606-17|RVcoll14U883||Romania|658[0n]
Libythea celtis|WMB5495-14|RVcoll.LD-3414||Italy|658[0n]
Libythea celtis|WMB4810-14|RVcoll. 14-I357||Italy|658[0n]
Libythea celtis|WMB4585-14|RVcoll.170410YM74||Spain|658[0n]
Libythea celtis|WMB4556-14|RVcoll.14-E402|Corsica|France|658[0n]
Libythea celtis|WMB2292-13|RVcoll.10-C658||Italy|658[0n]
Libythea celtis|WMB1965-13|RVcoll.11-I130||Italy|658[0n]
Libythea celtis|WMB2831-13|RVcoll.12-O524|Sardinia|Italy|658[0n]
Libythea celtis|WMB2780-13|RVcoll.11-I100|Sicily|Italy|658[0n]
Libythea celtis|EZSPC481-09|RVcoll.08-J378||Spain|658[0n]
Libythea celtis|EZSPN428-09|RVcoll.08-H603||Spain|658[0n]
Libythea celtis|EZSPC484-09|RVcoll.08-J349||Spain|658[0n]
Libythea celtis|EZSPC483-09|RVcoll.08-L446||Spain|658[0n]
Libythea celtis|EZSPC639-09|RVcoll.08-L374||Spain|658[0n]
Libythea celtis|GWORU087-10|BC ZSM Lep 30348||Italy|658[0n]
Libythea celtis|GWORO992-09|BC ZSM Lep 32299||Italy|658[0n]
Libythea celtis|LEATJ284-15|TLMF Lep 18714||Italy|658[0n]
Libythea celtis|ABOLD572-17|2015_DR_LC7||Austria|658[0n]
Libythea celtis|LEATJ285-15|TLMF Lep 18715||Italy|658[0n]
Iphiclides podalirius|EULEP701-15|RVcoll.12-M170|Crete|Greece|658[0n]
Iphiclides feisthamelii|EZSPM099-09|RVcoll.08-P429||Spain|658[0n]
Iphiclides feisthamelii|EZSPM450-09|Rvcoll.09-V588||Spain|658[0n]
Iphiclides podalirius|BIBSA1766-16|LD-2971|Sicily|Italy|658[0n]
Iphiclides podalirius|WMB2442-13|RVcoll.11-D226|Sicily|Italy|658[0n]
Iphiclides podalirius|BIBSA1329-15|12-R000|Sicily|Italy|658[0n]
Iphiclides podalirius|BIBSA1600-16|12-R126|Sicily|Italy|658[0n]
Iphiclides podalirius|WMB1955-13|RVcoll.11-I057|Sicily|Italy|639[0n]
Iphiclides podalirius|WMB1294-13|RVcoll.12-M562|Sicily|Italy|636[0n]
Iphiclides podalirius|WMB127-11|RVcoll.11-D093|Sicily|Italy|658[0n]
Iphiclides feisthamelii|WMB3315-14|RVcoll.08-P631||Spain|658[0n]
Iphiclides feisthamelii|WMB3126-14|RVcoll.06-G529||Spain|658[0n]
Iphiclides podalirius|WMB1173-13|RVcoll.11-E823|Corsica|France|658[0n]
Iphiclides feisthamelii|WMB3481-14|RVcoll.10-B529||Spain|658[0n]
Iphiclides podalirius|WMB3945-14|RVcoll.12-P698||France|658[0n]
Iphiclides feisthamelii|WMB3777-14|RVcoll.12-N728||Spain|658[0n]
Iphiclides feisthamelii|EZSPN403-09|RVcoll.08-H477||Spain|658[0n]
Iphiclides feisthamelii|EZSPC449-09|RVcoll.08-J383||Spain|658[0n]
Iphiclides feisthamelii|WMB4046-14|RVcoll.13-S279||Spain|658[0n]

Iphiclides feisthamelii|EZSPN403-09|RVcoll.08-H477||Spain|658[0n]
Iphiclides feisthamelii|EZSPC449-09|RVcoll.08-J383||Spain|658[0n]
Iphiclides feisthamelii|WMB4046-14|RVcoll.13-S279||Spain|658[0n]
Iphiclides feisthamelii|WMB3995-14|RVcoll.12-Q420||Spain|658[0n]
Iphiclides podalirius|WMB4637-14|RVcoll.LD-2403|Corsica|France|658[0n]
Iphiclides feisthamelii|WMB4400-14|RVcoll.14-B411||Portugal|658[0n]
Iphiclides podalirius|WMB5327-14|RVcoll.14-J074|Corsica|France|658[0n]
Iphiclides podalirius|WMB354-11|RVcoll.11-E811|Corsica|France|658[0n]
Iphiclides feisthamelii|EZSPM449-09|Rvcoll.09-X581||Spain|655[0n]
Iphiclides feisthamelii|EZSPN330-09|RVcoll.08-H248||Spain|658[0n]
Iphiclides feisthamelii|EZSPN477-09|RVcoll.08-H912||Spain|658[0n]
Iphiclides feisthamelii|EZSPC451-09|RVcoll.06-G512||Spain|658[0n]
Iphiclides feisthamelii|EZSPM611-12|RVcoll.11-D685||Spain|658[0n]
Iphiclides feisthamelii|EZSPM629-12|RVcoll.11-E018||Spain|658[0n]
Iphiclides feisthamelii|EZROM725-08|RV-06-G438||Spain|658[0n]
Iphiclides feisthamelii|EZSPM275-09|SMcoll.08-J611||Spain|658[0n]
Iphiclides feisthamelii|EZSPM362-09|RVcoll.08-J820||Spain|658[0n]
Iphiclides podalirius|BIBSA922-15|16-A013||France|658[0n]
Iphiclides podalirius|OXB1065-15|14-E296|Corsica|France|658[0n]
Iphiclides podalirius|WMB4642-14|RVcoll.LD-2419|Corsica|France|608[0n]
Iphiclides feisthamelii|EZSPM803-12|Rvcoll. 090211SD11||Spain|633[0n]
Iphiclides feisthamelii|EZSPN561-09|RVcoll.08-J100||Portugal|623[0n]
Iphiclides feisthamelii|WMB4417-14|RVcoll.14-B465||Portugal|636[0n]
Iphiclides podalirius|LEFIL147-10|MM19147||Russia|658[0n]
Iphiclides podalirius|EULEP4835-16|RVcoll16H739||Ukraine|658[0n]
Iphiclides podalirius|EZROM1030-08|RVcoll.08-M475||Romania|658[0n]
Iphiclides podalirius|ABOLC133-16|TLMF Lep 20166||Austria|658[0n]
Iphiclides podalirius|LEATG462-14|TLMF Lep 14249||Italy|658[0n]
Iphiclides podalirius|EULEP001-14|RVcoll.10-C413||Romania|658[0n]
Iphiclides feisthamelii|EZSPM419-09|Rvcoll.09-V842||Spain|658[0n]
Iphiclides podalirius|EZROM1028-08|RVcoll.08-M270||Romania|658[0n]
Iphiclides podalirius|EULEP4099-16|RVcoll15J679||France|658[0n]
Iphiclides podalirius|EULEP1367-15|RVcoll.14-F947||Greece|658[0n]
Iphiclides podalirius|EULEP897-15|RVcoll.14-C726||Bulgaria|658[0n]
Iphiclides podalirius|EULEP1197-15|RVcoll.14-F475||Bulgaria|658[0n]
Iphiclides podalirius|EZROM220-08|RV-07-D235||Romania|658[0n]
Iphiclides feisthamelii|EZSPC450-09|RVcoll.07-C075||Spain|658[0n]
Iphiclides podalirius|EULEP5022-16|RVcoll16I948||Slovakia|658[0n]
Iphiclides feisthamelii|EULEP003-14|RVcoll.12-P223||France|658[0n]
Iphiclides feisthamelii|EULEP002-14|RVcoll.12-P222||France|658[0n]
Iphiclides podalirius|EULEP4098-16|RVcoll15J192||Switzerland|658[0n]
Iphiclides podalirius|WMB5497-14|RVcoll.14-J285|Elba|Italy|658[0n]
Iphiclides podalirius|WMB5040-14|RVcoll. 14-L171||Italy|658[0n]
Iphiclides podalirius|WMB4968-14|RVcoll. 14-I515||Italy|658[0n]
Iphiclides podalirius|WMB4855-14|RVcoll.14-I402||Italy|658[0n]
Iphiclides podalirius|WMB4804-14|RVcoll. 14-I351||Italy|658[0n]
Iphiclides podalirius|WMB641-11|RVcoll.11-I153||Italy|658[0n]
Iphiclides podalirius|WMB5084-14|RVcoll. 14-L215||Italy|658[0n]
Iphiclides podalirius|WMB6563-18|RVcoll14A108||Italy|658[0n]
Iphiclides podalirius|WMB4342-14|RVcoll.14-A746||Italy|658[0n]
Iphiclides podalirius|WMB4288-14|RVcoll.14-A340||Italy|658[0n]
Iphiclides podalirius|WMB4271-14|RVcoll.14-A202||Italy|658[0n]
Iphiclides podalirius|BIBSA701-15|RVcoll. 12-Q860||Italy|658[0n]
Iphiclides podalirius|BIBSA1423-15|15-N093||Italy|658[0n]
Iphiclides podalirius|WMB3971-14|RVcoll.12-Q140||France|658[0n]
Iphiclides podalirius|WMB3916-14|RVcoll.12-P380||France|658[0n]
Iphiclides podalirius|WMB3912-14|RVcoll.12-P373||France|658[0n]
Iphiclides podalirius|WMB3893-14|RVcoll.12-O922||France|658[0n]
Iphiclides podalirius|WMB2360-13|RVcoll.12-Q917||Italy|658[0n]
Iphiclides podalirius|WMB3853-14|RVcoll.11-H367||Italy|658[0n]
Iphiclides podalirius|WMB4131-14|RVcoll.13-S618||Italy|658[0n]
Iphiclides podalirius|WMB2361-13|RVcoll.12-Q918||Italy|658[0n]
Iphiclides podalirius|WMB3834-14|RVcoll.09-X916||Italy|658[0n]
Iphiclides podalirius|WMB1895-13|RVcoll.11-J963|Sardinia|Italy|658[0n]
Iphiclides podalirius|WMB1730-13|RVcoll.12-P898||France|658[0n]
Iphiclides podalirius|WMB2120-13|RVcoll.12-R192||Italy|658[0n]
Iphiclides podalirius|WMB1800-13|RVcoll.12-Q336||France|658[0n]
Iphiclides podalirius|WMB3465-14|RVcoll.10-A613||France|658[0n]
Iphiclides podalirius|WMB259-11|RVcoll.10-A211||France|658[0n]
Iphiclides podalirius|EZROM219-08|RV-06-K576||Romania|658[0n]
Iphiclides podalirius|EZROM1029-08|RVcoll.08-M306||Romania|658[0n]
Iphiclides podalirius|EZROM351-08|RV-08-A029||Romania|658[0n]
Iphiclides podalirius|GWORA2430-09|BC ZSM Lep 30642||Germany|658[0n]
Iphiclides podalirius|ABOLD084-16|TLMF Lep 21162||Austria|658[0n]
Iphiclides podalirius|BIBSA580-15|LEP-SS-00200||Italy|658[0n]
Iphiclides podalirius|BIBSA1031-15|15-C187||Italy|658[0n]
Iphiclides podalirius|BIBSA1667-16|15-C772||Italy|658[0n]
Iphiclides podalirius|BIBSA1171-15|15-M197||Italy|658[0n]
Iphiclides podalirius|BIBSA1140-15|15-L969||Italy|658[0n]
Iphiclides podalirius|EULEP2164-15|RVcoll.14-K084||Switzerland|658[0n]
Iphiclides podalirius|BIBSA349-15|RVcoll.14-I017||Italy|658[0n]
Iphiclides podalirius|BIBSA970-15|14-A128||Italy|658[0n]
Iphiclides podalirius|EULEP5662-17|RVcoll15M429||Italy|658[0n]
Iphiclides podalirius|EZRMN332-08|RVcoll.08-H001||Romania|658[0n]
Iphiclides podalirius|EULEP1600-15|RVcoll.14-G653||Greece|658[0n]
Iphiclides podalirius|EULEP1306-15|RVcoll.14-F754||Greece|658[0n]
Iphiclides podalirius|EZSPM448-09|Rvcoll.09-X218||France|658[0n]
Iphiclides podalirius|BIBSA1618-16|15-C421||Italy|658[0n]
Iphiclides podalirius|EULEP1767-15|RVcoll.14-H456||Greece|658[0n]
Iphiclides podalirius|EULEP1442-15|RVcoll.14-G142||Greece|658[0n]
Iphiclides podalirius|LEASS703-17|TLMF Lep 22351||Austria|658[0n]
Iphiclides podalirius|OXB587-15|14-N976||Italy|658[0n]
Iphiclides podalirius|OXB888-15|15-A530||Italy|658[0n]
Iphiclides podalirius|OXB910-15|15-A554||Italy|658[0n]
Iphiclides podalirius|OXB974-15|15-A924||Italy|658[0n]
Iphiclides podalirius|OXB1028-15|15-A978||Italy|658[0n]
Iphiclides podalirius|BIBSA1613-16|15-C379||Italy|658[0n]
Iphiclides podalirius|OXB1259-15|Rvcoll_15-M115||France|658[0n]
Iphiclides podalirius|WMB2216-13|RVcoll.11-Y033||Italy|658[0n]
Iphiclides podalirius|OXB1366-15|Rvcoll_15-M724||France|658[0n]
Iphiclides podalirius|WMB4765-14|RVcoll. 14-I312||Italy|658[0n]
Iphiclides podalirius|OXB1388-15|Rvcoll_15-M753||France|658[0n]
[illegible]

Iphiclides podalirius|OXB[illegible]-15|Rvcoll_15-M724||France|658[0n]
Iphiclides podalirius|WMB4765-14|RVcoll. 14-I312||Italy|658[0n]
Iphiclides podalirius|OXB1388-15|Rvcoll_15-M753||France|658[0n]
Iphiclides podalirius|LEASS856-17|KLM Lep 08361||Austria|658[1n]
Iphiclides podalirius|WMB4783-14|RVcoll. 14-I330||Italy|626[0n]
Iphiclides podalirius|WMB4728-14|RVcoll.LD-3110||Italy|622[0n]
Iphiclides podalirius|WMB4672-14|RVcoll.LD-2625|Ischia|Italy|622[0n]
Iphiclides podalirius|EZSPC452-09|RVcoll.08-R262||Spain|636[0n]
Iphiclides podalirius|EULEP1702-15|RVcoll.14-H121|Genit. examined|Greece|614[0n]
Iphiclides podalirius|WMB3846-14|RVcoll.10-C716||Italy|614[0n]
Iphiclides podalirius|LEATI005-15|TLMF Lep 17390||Austria|622[0n]
Iphiclides podalirius|LEATD016-13|TLMF Lep 12663||Italy|658[0n]
Archon apollinus|EULEP2620-15|RVcoll.14-N560||Greece|658[0n]
Archon apollinus|EULEP2619-15|RVcoll.14-N559||Greece|658[0n]
Archon apollinus|EULEP2618-15|RVcoll.14-N558||Greece|658[0n]
Archon apollinus|EULEP568-15|RVcoll.09-X199|Samos|Greece|658[0n]
Archon apollinus|EULEP2108-15|RVcoll.14-J599|Lesvos|Greece|658[0n]
Archon apollinus|EULEP561-15|RVcoll.08-R594|Samos|Greece|658[0n]
Pieris brassicae|EULEP1990-15|RVcoll.14-I682||France|658[0n]
Pieris brassicae|BIBSA1240-15|15-F810||France|658[0n]
Pieris brassicae|WMB4971-14|RVcoll. 14-I518||Italy|658[0n]
Pieris brassicae|WMB493-11|RVcoll.11-H540|Sicily|Italy|658[0n]
Pieris brassicae|WMB3056-14|RVcoll.13-T726|Sardinia|Italy|658[0n]
Pieris brassicae|WMB1099-13|RVcoll.10-C768|Giglio|Italy|658[0n]
Pieris brassicae|EULEP696-15|RVcoll.12-M112|Crete|Greece|658[0n]
Pieris brassicae|BIBSA1853-17|RVcoll16C734||Italy|658[0n]
Pieris brassicae|WMB4491-14|RVcoll.14-E167||Italy|658[0n]
Pieris brassicae|BCLEP111-17|LEP-SS-00562||Italy|658[0n]
Pieris brassicae|EULEP2430-15|RVcoll.14-V222||Belgium|658[0n]
Pieris brassicae|WMB4129-14|RVcoll.13-S614||Italy|658[0n]
Pieris brassicae|EZSPN1024-09|RVcoll.08-M956||Spain|658[0n]
Pieris brassicae|EZSPC598-09|RVcoll.08-P071||Spain|658[0n]
Pieris brassicae|WMB5043-14|RVcoll. 14-L174||Italy|658[0n]
Pieris brassicae|WMB5170-14|RVcoll.13-S515||Italy|658[0n]
Pieris brassicae|WMB2320-13|RVcoll.11-H633|Lampedusa|Italy|658[0n]
Pieris brassicae|WMB527-11|RVcoll.11-H631|Lampedusa|Italy|658[0n]
Pieris brassicae|WMB1913-13|RVcoll.11-H635|Lampedusa|Italy|654[0n]
Pieris brassicae|WMB2935-14|RVcoll.12-L500|Mallorca|Spain|658[0n]
Pieris brassicae|WMB2889-14|RVcoll.11-D141|Pantelleria|Italy|658[0n]
Pieris brassicae|WMB263-11|RVcoll.10-A417||France|658[0n]
Pieris brassicae|WMB136-11|RVcoll.11-D144|Pantelleria|Italy|658[0n]
Pieris brassicae|WMB1066-13|RVcoll.10-C688|Stromboli|Italy|658[0n]
Pieris brassicae|WMB1113-13|RVcoll.11-D118|Pantelleria|Italy|658[0n]
Pieris brassicae|GWORA2448-09|BC ZSM Lep 30660||Germany|658[1n]
Pieris brassicae|OXB707-15|13-T886||Italy|658[0n]
Pieris brassicae|BIBSA1035-15|15-C195||Italy|658[0n]
Pieris brassicae|WMB2726-13|RVcoll.10-C582||Malta|658[0n]
Pieris brassicae|EULEP022-14|RVcoll.07-D470||Romania|658[0n]
Pieris brassicae|WMB5344-14|RVcoll.14-J363|Pianosa|Italy|611[0n]
Pieris brassicae|EULEP1381-15|RVcoll.14-F981||Greece|658[0n]
Pieris brassicae|EULEP987-15|RVcoll.14-D161||Austria|658[0n]
Pieris brassicae|EULEP1967-15|RVcoll.14-G831|Lesvos|Greece|658[0n]
Pieris brassicae|EULEP1741-15|RVcoll.14-H330||Greece|658[0n]
Pieris brassicae|EULEP738-15|RVcoll.12-N849|Lesvos|Greece|658[0n]
Pieris brassicae|EZRMN164-08|RVcoll.08-M393||Romania|658[0n]
Pieris brassicae|WMB3958-14|RVcoll.12-P769||France|658[0n]
Pieris brassicae|EZRMN165-08|RVcoll.08-M422||Romania|658[0n]
Pieris brassicae|EZSPN300-09|RVcoll.08-H104||Spain|658[0n]
Pieris brassicae|EZSPM999-12|Rvcoll.12-L885||Spain|658[0n]
Pieris brassicae|EZSPM1003-12|Rvcoll.12-L889||Spain|658[0n]
Pieris brassicae|EZSPM1007-12|Rvcoll.12-L896||Spain|658[0n]
Pieris brassicae|EZSPC595-09|RVcoll.08-L513||Spain|658[0n]
Pieris brassicae|EZSPC596-09|RVcoll.08-M703||Spain|658[0n]
Pieris brassicae|WMB142-11|RVcoll.11-D164|Marettimo|Italy|658[0n]
Pieris brassicae|WMB165-11|RVcoll.11-D372|Sicily|Italy|658[0n]
Pieris brassicae|EZROM374-08|07-E602||Spain|658[0n]
Pieris brassicae|WMB118-11|RVcoll.10-C176||France|658[0n]
Pieris brassicae|WMB2890-14|RVcoll.11-D212|Sicily|Italy|658[0n]
Pieris brassicae|WMB3045-14|RVcoll.13-S583|San Domino|Italy|658[0n]
Pieris brassicae|WMB3158-14|RVcoll.08-H247||Spain|658[0n]
Pieris brassicae|WMB765-13|RVcoll.11-Y004|Elba|Italy|658[0n]
Pieris brassicae|WMB1118-13|RVcoll.11-D163|Marettimo|Italy|658[0n]
Pieris brassicae|WMB1154-13|RVcoll.11-E484|Sardinia|Italy|658[0n]
Pieris brassicae|WMB1155-13|RVcoll.11-E485|Sardinia|Italy|658[0n]
Pieris brassicae|WMB1182-13|RVcoll.11-E939|Corsica|France|658[0n]
Pieris brassicae|WMB2663-13|RVcoll.10-B769||France|658[0n]
Pieris brassicae|WMB2772-13|RVcoll.11-H877|Salina|Italy|658[0n]
Pieris brassicae|WMB811-13|RVcoll.07-E109||Italy|658[0n]
Pieris brassicae|WMB1060-13|RVcoll.10-C680|Lipari|Italy|658[0n]
Pieris brassicae|WMB1104-13|RVcoll.10-C780||Italy|658[0n]
Pieris brassicae|WMB2015-13|RVcoll.12-Q659||Italy|658[0n]
Pieris brassicae|WMB2206-13|RVcoll.11-Y015|Elba|Italy|658[0n]
Pieris brassicae|WMB2271-13|RVcoll.LD-2731||Malta|658[0n]
Pieris brassicae|WMB2319-13|RVcoll.11-H632|Lampedusa|Italy|658[0n]
Pieris brassicae|WMB2353-13|RVcoll.12-Q889||Italy|658[0n]
Pieris brassicae|WMB2359-13|RVcoll.12-Q914||Italy|658[0n]
Pieris brassicae|WMB2431-13|RVcoll.11-D027|Capri|Italy|658[0n]
Pieris brassicae|WMB2439-13|RVcoll.11-D168|Marettimo|Italy|658[0n]
Pieris brassicae|WMB2448-13|RVcoll.11-E360|Sardinia|Italy|658[0n]
Pieris brassicae|WMB2576-13|RVcoll.12-R017|Sicily|Italy|658[0n]
Pieris brassicae|WMB4137-14|RVcoll.13-S645||Italy|658[0n]
Pieris brassicae|WMB3895-14|RVcoll.12-O996||France|658[0n]
Pieris brassicae|WMB4504-14|RVcoll.14-E181||Italy|658[0n]
Pieris brassicae|WMB3964-14|RVcoll.12-P929||France|658[0n]
Pieris brassicae|WMB4289-14|RVcoll.14-A342||Italy|658[0n]
Pieris brassicae|WMB4337-14|RVcoll.14-A723||Italy|658[0n]
Pieris brassicae|WMB4630-14|RVcoll.LD-2382|Ustica|Italy|658[0n]
Pieris brassicae|WMB4681-14|RVcoll.LD-2665||Italy|658[0n]
Pieris brassicae|WMB4709-14|RVcoll.LD-2915|Asinara|Italy|658[0n]
Pieris brassicae|WMB4710-14|RVcoll.LD-2916|Asinara|Italy|658[0n]
Pieris brassicae|WMB307-11|RVcoll.11-E362||Italy|658[0n]
Pieris brassicae|WMB353-11|RVcoll.11-E809||France|655[0n]
Pieris brassicae|WMB480-11|RVcoll.11-H505|Capri|Italy|658[0n]

Pieris brassicae|WMB307-11|RVcoll.11-E362||Italy|658[0n]
Pieris brassicae|WMB353-11|RVcoll.11-E809||France|655[0n]
Pieris brassicae|WMB480-11|RVcoll.11-H505|Capri|Italy|658[0n]
Pieris brassicae|WMB572-11|RVcoll.11-H798|Vulcano|Italy|658[0n]
Pieris brassicae|WMB585-11|RVcoll.11-H878|Salina|Italy|658[0n]
Pieris brassicae|WMB638-11|RVcoll.11-I140||Italy|658[0n]
Pieris brassicae|WMB4762-14|RVcoll. 14-I309||Italy|658[0n]
Pieris brassicae|WMB4797-14|RVcoll. 14-I344||Italy|658[0n]
Pieris brassicae|WMB4869-14|RVcoll.14-I416||Italy|658[0n]
Pieris brassicae|WMB4897-14|RVcoll.14-I444||Italy|658[0n]
Pieris brassicae|WMB5472-14|RVcoll.14-N690|Alicudi|Italy|658[0n]
Pieris brassicae|EULEP4409-16|RVcoll10A953||Bulgaria|658[0n]
Pieris brassicae|EULEP5031-16|RVcoll16I978||Slovakia|658[0n]
Pieris brassicae|EULEP5703-17|RVcoll16L046||Italy|658[0n]
Pieris brassicae|EULEP5704-17|RVcoll16L047||Italy|658[0n]
Pieris brassicae|EULEP1211-15|RVcoll.14-F507||Greece|658[0n]
Pieris brassicae|EULEP1334-15|RVcoll.14-F818||Greece|658[0n]
Pieris brassicae|BIBSA1212-15|15-M337||Italy|658[0n]
Pieris brassicae|BIBSA913-15|16-A004||France|658[0n]
Pieris brassicae|BIBSA1593-16|12-Q944||Italy|658[0n]
Pieris brassicae|BIBSA1310-15|12-M358|Sicily|Italy|658[0n]
Pieris brassicae|BIBSA1786-16|16-A520||Italy|658[0n]
Pieris brassicae|BIBSA1392-15|15-M990||Italy|658[0n]
Pieris brassicae|BIBSA698-15|RVcoll. 11-I190||Italy|658[0n]
Pieris brassicae|BIBSA021-14|LEP-SS-00021||Italy|658[0n]
Pieris brassicae|BIBSA022-14|LEP-SS-00022||Italy|658[0n]
Pieris brassicae|BIBSA1831-17|RVcoll16C706||Italy|658[0n]
Pieris brassicae|OXB726-15|13-U160||Italy|658[0n]
Pieris brassicae|OXB1039-15|LD-2836||Italy|658[0n]
Pieris brassicae|OXB1344-15|Rvcoll_15-M692||France|658[0n]
Pieris brassicae|OXB1229-15|Rvcoll_15-M664||France|658[0n]
Pieris brassicae|OXB1236-15|Rvcoll_13-T628|Sardinia|Italy|658[0n]
Pieris brassicae|OXB1240-15|Rvcoll_13-T676|Sardinia|Italy|658[0n]
Pieris brassicae|WMB1928-13|RVcoll.11-H796|Vulcano|Italy|654[0n]
Pieris brassicae|WMB1100-13|RVcoll.10-C769|Giglio|Italy|623[0n]
Pieris brassicae|WMB4826-14|RVcoll. 14-I373||Italy|658[0n]
Pieris brassicae|BIBSA439-15|RVcoll.14-I107||Italy|658[0n]
Pieris brassicae|BIBSA1549-16|11-D375||Italy|619[0n]
Pieris brassicae|BIBSA252-15|RVcoll.14-E048||Italy|622[1n]
Pieris brassicae|EULEP4411-16|RVcoll15G141||France|624[0n]
Pieris brassicae|EULEP1703-15|RVcoll.14-H135||Greece|614[0n]
Pieris brassicae|WMB1944-13|RVcoll.11-H876|Salina|Italy|637[0n]
Pieris brassicae|EZROM293-08|RV-07-E655||Romania|609[0n]
Pieris brassicae|EZROM307-08|RV-07-E669||Romania|658[0n]
Pieris brassicae|EZROM459-08|RV-06-M876||Romania|658[0n]
Pieris brassicae|GWORU083-10|BC ZSM Lep 30344||Italy|658[0n]
Pieris brassicae|PHLAW011-13|TLMF Lep 09808||Austria|658[0n]
Pieris brassicae|ABOLD049-16|TLMF Lep 21127||Austria|658[0n]
Pieris brassicae|WMB4414-14|RVcoll.14-B458||Portugal|658[0n]
Pieris brassicae|WMB4429-14|RVcoll.14-B495||Portugal|658[0n]
Pieris brassicae|EZSPM739-12|Rvcoll. 12-L588||Portugal|658[0n]
Pieris brassicae|EZSPN577-09|RVcoll.08-J124||Portugal|658[0n]
Pieris brassicae|WMB3167-14|RVcoll.08-H505||Spain|658[0n]
Pieris brassicae|EZSPC597-09|RVcoll.08-J345||Spain|658[0n]
Pieris brassicae|EZSPN373-09|RVcoll.08-H399||Spain|658[0n]
Pieris brassicae|EZSPM619-12|RVcoll.11-D877||Spain|658[0n]
Pieris brassicae|EZSPM279-09|SMcoll.150308PP65||Spain|658[0n]
Pieris brassicae|WMB3553-14|RVcoll.11-D895||Spain|658[0n]
Pieris brassicae|EZSPM740-12|Rvcoll. 12-L589||Portugal|621[0n]
Pieris brassicae|EZSPN839-09|RVcoll.08-L667||Spain|618[0n]
Pieris brassicae|GBLAC960-13|BC ZSM Lep 76491||Germany|658[0n]
Pieris brassicae|EZROM460-08|RV-07-C969||Romania|658[0n]
Pieris brassicae|GBLAA348-14|BC ZSM Lep 80344||Germany|658[0n]
Pieris brassicae|EULEP5081-16|RVcoll16J322||Germany|658[0n]
Pieris brassicae|WMB4394-14|RVcoll.14-B347||Portugal|658[0n]
Pieris brassicae|GBLAF826-14|BC ZSM Lep 82912||Germany|602[1n]
Pieris brassicae|EULEP4938-16|RVcoll16I050||Poland|658[0n]
Pieris brassicae|WMB126-11|RVcoll.11-D022|Capri|Italy|658[0n]
Pieris brassicae|OXB1549-16|OXB-TGS-1267||France|658[0n]
Pieris brassicae|LEATG069-14|TLMF Lep 13856||Austria|658[0n]
Pieris brassicae|LENOA1340-11|LN-BD1340||France|658[0n]
Pieris brassicae|FBLMU454-09|BC ZSM Lep 27104||Germany|658[0n]
Pieris brassicae|GWORK291-09|BC ZSM Lep 21526||Germany|658[0n]
Pieris brassicae|EZROM647-08|RV-07-D357||Romania|658[0n]
Pieris brassicae|EZSPM232-09|RVcoll.08-R428||Spain|658[0n]
Pieris brassicae|WMB4544-14|RVcoll.020808CB25||Spain|658[0n]
Pieris brassicae|WMB4688-14|RVcoll.LD-2688|Giglio|Italy|658[0n]
Pieris brassicae|WMB544-11|RVcoll.11-H732|Sicily|Italy|658[0n]
Pieris brassicae|WMB5077-14|RVcoll. 14-L208||Italy|658[0n]
Pieris brassicae|WMB5491-14|RVcoll.LD-2758||Italy|658[0n]
Pieris brassicae|EULEP4412-16|RVcoll15P015||Belarus|658[0n]
Pieris brassicae|EULEP4726-16|RVcoll16G388||Denmark|658[0n]
Pieris brassicae|OXB1446-16|OXB-TGS-1220||United Kingdom|658[0n]
Pieris brassicae|OXB898-15|15-A542||Italy|658[0n]
Pieris brassicae|OXB193-15|OXB-TGS-036||United Kingdom|658[0n]
Pieris brassicae|OXB196-15|OXB-TGS-081||United Kingdom|658[0n]
Pieris brassicae|OXB1313-15|Rvcoll_15-M578||France|658[0n]
Pieris brassicae|WMB1068-13|RVcoll.10-C692|Ischia|Italy|607[0n]
Pieris brassicae|EULEP4410-16|RVcoll15G045||France|649[0n]
Pieris brassicae|WMB1170-13|RVcoll.11-E761|Corsica|France|615[0n]
Pieris brassicae|LEASS693-17|TLMF Lep 22341||Austria|614[0n]
Pieris brassicae|GWORA2449-09|BC ZSM Lep 30661||Germany|623[0n]
Pieris brassicae|LEATG070-14|TLMF Lep 13857||Austria|611[0n]
Pieris brassicae|LEFIJ4544-16|ZMBSU-02110||Belarus|658[0n]
Pieris brassicae|OXB1322-15|Rvcoll_15-M593||France|658[0n]
Pieris brassicae|LEFIJ4745-16|ZMBSU-02156||Belarus|658[0n]
Pieris brassicae|GBLAF252-14|BC ZSM Lep 81958||Germany|658[0n]
Pieris brassicae|WMB2970-14|RVcoll.12-O058|Argentario|Italy|658[0n]
Pieris brassicae|LEFIJ502-10|MM17127||Finland|658[0n]
Pieris brassicae|LEFIC266-10|MM03697||Finland|658[0n]
Pieris brassicae|LEFIC265-10|MM03696||Finland|658[0n]
Pieris brassicae|LEATG533-14|TLMF Lep 14320||Italy|658[0n]
Pieris brassicae|LEATG532-14|TLMF Lep 14319||Italy|658[0n]

[illegible]
Pieris brassicae|LEFIC265-10|MM03696||Finland|658[0n]
Pieris brassicae|LEATG533-14|TLMF Lep 14320||Italy|658[0n]
Pieris brassicae|LEATG532-14|TLMF Lep 14319||Italy|658[0n]
Gonepteryx farinosa|EULEP2544-15|RVcoll.14-V446||Greece|658[0n]
Gonepteryx farinosa|EULEP1740-15|RVcoll.14-H314||Greece|658[0n]
Gonepteryx farinosa|EULEP1723-15|RVcoll.14-H225||Greece|658[0n]
Gonepteryx farinosa|EULEP1458-15|RVcoll.14-G204||Greece|658[0n]
Gonepteryx farinosa|EULEP1437-15|RVcoll.14-G127||Greece|658[0n]
Gonepteryx farinosa|EULEP1781-15|RVcoll.14-H585||Greece|658[0n]
Gonepteryx farinosa|EULEP1387-15|RVcoll.14-F992|Genit. examined|Greece|658[0n]
Gonepteryx farinosa|EULEP1708-15|RVcoll.14-H147||Greece|614[0n]
Gonepteryx farinosa|EULEP1214-15|RVcoll.14-F512||Greece|658[0n]
Gonepteryx farinosa|EULEP1203-15|RVcoll.14-F497||Greece|658[0n]
Gonepteryx cleopatra|BIBSA1303-15|10-B463|Sardinia|Italy|658[0n]
Gonepteryx cleopatra|WMB5351-14|RVcoll.14-J383|Giannutri|Italy|655[0n]
Gonepteryx cleopatra|WMB1917-13|RVcoll.11-H686|Levanzo|Italy|651[0n]
Gonepteryx cleopatra|EZSPN338-09|RVcoll.08-H270||Spain|658[0n]
Gonepteryx cleopatra|EZSPN331-09|RVcoll.08-H250||Spain|658[0n]
Gonepteryx cleopatra|EZSPN310-09|RVcoll.08-H117||Spain|658[0n]
Gonepteryx cleopatra|EZSPN409-09|RVcoll.08-H510||Spain|658[0n]
Gonepteryx cleopatra|EZSPM348-09|RVcoll.08-R043||Spain|658[0n]
Gonepteryx cleopatra|EZSPM628-12|RVcoll.11-D973||Spain|658[0n]
Gonepteryx cleopatra|WMB2408-13|RVcoll.10-C518||Italy|658[0n]
Gonepteryx cleopatra|WMB2402-13|RVcoll.09-X931|Elba|Italy|658[0n]
Gonepteryx cleopatra|WMB2397-13|RVcoll.09-X868|Sardinia|Italy|658[0n]
Gonepteryx cleopatra|WMB2272-13|RVcoll.LD-2732||Malta|658[0n]
Gonepteryx cleopatra|WMB2863-14|RVcoll.09-X885|Corsica|France|658[0n]
Gonepteryx cleopatra|WMB284-11|RVcoll.10-B600||France|658[0n]
Gonepteryx cleopatra|EZSPN1045-11|Rvcoll.08-L041||Spain|658[0n]
Gonepteryx cleopatra|EZSPN1004-09|RVcoll.08-M680||Spain|658[0n]
Gonepteryx cleopatra|WMB1486-13|RVcoll.12-O014|Giglio|Italy|658[0n]
Gonepteryx cleopatra|WMB1167-13|RVcoll.11-E658|Sardinia|Italy|658[0n]
Gonepteryx cleopatra|WMB1132-13|RVcoll.11-E110|Gozo|Malta|658[0n]
Gonepteryx cleopatra|WMB3650-14|RVcoll.11-I624||France|658[0n]
Gonepteryx cleopatra|WMB909-13|RVcoll.09-X898|Capri|Italy|658[0n]
Gonepteryx cleopatra|WMB1539-13|RVcoll.12-O281|La Maddalena|Italy|658[0n]
Gonepteryx cleopatra|WMB1524-13|RVcoll.12-O202|Corsica|France|658[0n]
Gonepteryx cleopatra|WMB1499-13|RVcoll.12-O045||Italy|658[0n]
Gonepteryx cleopatra|WMB338-11|RVcoll.11-E677||France|658[0n]
Gonepteryx cleopatra|WMB330-11|RVcoll.11-E634|Spargi|Italy|658[0n]
Gonepteryx cleopatra|WMB1067-13|RVcoll.10-C689|Ischia|Italy|658[0n]
Gonepteryx cleopatra|WMB928-13|RVcoll.10-A473||France|658[0n]
Gonepteryx cleopatra|WMB4805-14|RVcoll. 14-I352||Italy|658[0n]
Gonepteryx cleopatra|WMB4768-14|RVcoll. 14-I315||Italy|658[0n]
Gonepteryx cleopatra|WMB3920-14|RVcoll.12-P459||France|658[0n]
Gonepteryx cleopatra|WMB4495-14|RVcoll.14-E171||Italy|658[0n]
Gonepteryx cleopatra|WMB1742-13|RVcoll.12-P954|Levant|France|658[0n]
Gonepteryx cleopatra|WMB1780-13|RVcoll.12-Q114||France|658[0n]
Gonepteryx cleopatra|WMB1291-13|RVcoll.12-M538|Capraia|Italy|658[0n]
Gonepteryx cleopatra|WMB1096-13|RVcoll.10-C764|Giglio|Italy|658[0n]
Gonepteryx cleopatra|WMB1981-13|RVcoll.11-I284|Pianosa|Italy|658[0n]
Gonepteryx cleopatra|WMB1980-13|RVcoll.11-I283|Pianosa|Italy|658[0n]
Gonepteryx cleopatra|WMB1964-13|RVcoll.11-I115||Italy|658[0n]
Gonepteryx cleopatra|WMB1743-13|RVcoll.12-P955|Levant|France|658[0n]
Gonepteryx cleopatra|WMB300-11|RVcoll.11-E331||Italy|658[0n]
Gonepteryx cleopatra|WMB4684-14|RVcoll.LD-2680|Giglio|Italy|658[0n]
Gonepteryx cleopatra|WMB4421-14|RVcoll.14-B473||Portugal|658[0n]
Gonepteryx cleopatra|WMB3927-14|RVcoll.12-P513||France|658[0n]
Gonepteryx cleopatra|WMB614-11|RVcoll.11-I029|Sicily|Italy|658[0n]
Gonepteryx cleopatra|WMB534-11|RVcoll.11-H687|Levanzo|Italy|658[0n]
Gonepteryx cleopatra|WMB477-11|RVcoll.11-H502|Capri|Italy|658[0n]
Gonepteryx cleopatra|WMB514-11|RVcoll.11-H601|Sicily|Italy|658[0n]
Gonepteryx cleopatra|WMB5009-14|RVcoll. 14-I556||Italy|658[0n]
Gonepteryx cleopatra|WMB4981-14|RVcoll. 14-I528||Italy|658[0n]
Gonepteryx cleopatra|WMB5387-14|RVcoll.14-J862||France|658[0n]
Gonepteryx cleopatra|WMB4816-14|RVcoll. 14-I363||Italy|658[0n]
Gonepteryx cleopatra|EULEP726-15|RVcoll.12-N810|Lesvos|Greece|658[0n]
Gonepteryx cleopatra|EULEP702-15|RVcoll.12-M171|Crete|Greece|658[0n]
Gonepteryx cleopatra|EULEP5706-17|RVcoll16L049||Italy|658[0n]
Gonepteryx cleopatra|WMB5220-14|RVcoll.13-T970||Italy|658[0n]
Gonepteryx cleopatra|WMB501-11|RVcoll.11-H559|Sicily|Italy|658[0n]
Gonepteryx cleopatra|WMB368-11|RVcoll.11-E978||France|658[0n]
Gonepteryx cleopatra|WMB2236-13|RVcoll.11-Y112||Italy|658[0n]
Gonepteryx cleopatra|WMB2182-13|RVcoll.12-R355|Elba|Italy|658[0n]
Gonepteryx cleopatra|WMB4483-14|RVcoll.14-D928||Italy|658[0n]
Gonepteryx cleopatra|WMB4148-14|RVcoll.13-S690||Italy|658[0n]
Gonepteryx cleopatra|WMB2622-13|RVcoll.11-E909|Corsica. Genit. examined|France|658[0n]
Gonepteryx cleopatra|WMB2603-13|RVcoll.12-R527|Capraia|Italy|658[0n]
Gonepteryx cleopatra|WMB327-11|RVcoll.11-E622|Santa Maria|Italy|658[0n]
Gonepteryx cleopatra|WMB302-11|RVcoll.11-E346||Italy|655[0n]
Gonepteryx cleopatra|WMB3564-14|RVcoll.11-D939||Spain|658[0n]
Gonepteryx cleopatra|WMB3288-14|RVcoll.08-M691||Spain|658[0n]
Gonepteryx cleopatra|WMB3044-14|RVcoll.13-S576|San Domino|Italy|658[0n]
Gonepteryx cleopatra|WMB3027-14|RVcoll.13-S392|Ibiza|Spain|658[0n]
Gonepteryx cleopatra|WMB2986-14|RVcoll.12-O282|La Maddalena|Italy|658[0n]
Gonepteryx cleopatra|WMB3416-14|RVcoll.09-X018||Spain|658[0n]
Gonepteryx cleopatra|WMB3397-14|RVcoll.09-V586||Spain|658[0n]
Gonepteryx cleopatra|WMB3356-14|RVcoll.09-T097||Spain|658[0n]
Gonepteryx cleopatra|WMB2920-14|RVcoll.11-I362|Argentario|Italy|658[0n]
Gonepteryx cleopatra|WMB2911-14|RVcoll.11-E950|Corsica|France|658[0n]
Gonepteryx cleopatra|EZSPC1148-10|RVcoll.08-P900||Spain|658[0n]
Gonepteryx cleopatra|WMB2868-14|RVcoll.10-C553|Ibiza|Spain|658[0n]
Gonepteryx cleopatra|WMB154-11|RVcoll.11-D298|Sicily|Italy|658[0n]
Gonepteryx cleopatra|EZSPM914-12|Rvcoll.12-M636||Spain|658[0n]
Gonepteryx cleopatra|EZSPM048-09|RVcoll.08-P266||Spain|658[0n]
Gonepteryx cleopatra|EZSPM034-09|RVcoll.08-P235||Spain|658[0n]
Gonepteryx cleopatra|EULEP1392-15|RVcoll.14-G006||Greece|658[0n]
Gonepteryx cleopatra|EULEP1307-15|RVcoll.14-F755||Greece|658[0n]
Gonepteryx cleopatra|BIBSA727-15|LD-2834||Italy|658[0n]
Gonepteryx cleopatra|EULEP2862-15|RVcoll.14-N946|Rhodes|Greece|658[0n]
Gonepteryx cleopatra|BIBSA1621-16|15-C423||Italy|658[0n]
Gonepteryx cleopatra|BIBSA1295-15|14-E290|Corsica|France|658[0n]
[illegible]

Gonepteryx cleopatra|EULEP2862-15|RVcoll.14-N946|Rhodes|Greece|658[0n]
Gonepteryx cleopatra|BIBSA1621-16|15-C423||Italy|658[0n]
Gonepteryx cleopatra|BIBSA1295-15|14-E290|Corsica|France|658[0n]
Gonepteryx cleopatra|BIBSA1420-15|15-N090||Italy|658[0n]
Gonepteryx cleopatra|BIBSA649-15|RVcoll. 15-A812||Italy|658[0n]
Gonepteryx cleopatra|BIBSA656-15|RVcoll. 15-A819||Italy|658[0n]
Gonepteryx cleopatra|BIBSA1537-16|10-C647||Italy|658[0n]
Gonepteryx cleopatra|BIBSA344-15|RVcoll.14-I012||Italy|658[0n]
Gonepteryx cleopatra|BIBSA1662-16|15-C744||Italy|658[0n]
Gonepteryx cleopatra|BIBSA1793-16|16-A534||Italy|658[0n]
Gonepteryx cleopatra|OXB1079-15|15-A612||Italy|658[0n]
Gonepteryx cleopatra|EZSPM434-09|Rvcoll.09-X101||Spain|658[0n]
Gonepteryx cleopatra|WMB1565-13|RVcoll.12-O456|Sardinia|Italy|622[0n]
Gonepteryx cleopatra|WMB2240-13|RVcoll.12-R371|Elba|Italy|630[0n]
Gonepteryx cleopatra|WMB4503-14|RVcoll.14-E180||Italy|610[0n]
Gonepteryx cleopatra|WMB281-11|RVcoll.10-A674||France|630[0n]
Gonepteryx cleopatra|EZSPM370-09|RVcoll.08-J841||Spain|624[0n]
Gonepteryx cleopatra|EZSPN313-09|RVcoll.08-H171||Spain|658[0n]
Gonepteryx cleopatra|GWORU085-10|BC ZSM Lep 30346||Italy|658[0n]
Gonepteryx rhamni|EZSPM695-12|Rvcoll. 12-L598||Portugal|658[0n]
Gonepteryx rhamni|EZSPN852-09|RVcoll.08-L700||Spain|658[0n]
Gonepteryx rhamni|EZSPM813-12|Rvcoll. 130711PX13||Spain|658[0n]
Gonepteryx rhamni|WMB3233-14|RVcoll.08-J886||Spain|658[0n]
Gonepteryx rhamni|EZSPC1184-10|RVcoll.09-T163||Spain|658[0n]
Gonepteryx rhamni|EZSPM239-09|RVcoll.08-R442||Spain|658[0n]
Gonepteryx rhamni|EZSPN580-09|RVcoll.08-J135||Portugal|658[0n]
Gonepteryx rhamni|EZSPN418-09|RVcoll.08-H552||Spain|658[0n]
Gonepteryx rhamni|EZSPN464-09|RVcoll.08-H678||Spain|658[1n]
Gonepteryx rhamni|OXB309-15|RVcoll.14-N023|Argentario|Italy|658[0n]
Gonepteryx rhamni|BIBSA1149-15|15-L066||Italy|658[0n]
Gonepteryx rhamni|WMB3300-14|RVcoll.08-P407||Spain|658[0n]
Gonepteryx rhamni|EZSPC1192-10|RVcoll.09-V340||Spain|658[0n]
Gonepteryx rhamni|EZSPC1230-10|RVcoll.09-V694||Spain|658[0n]
Gonepteryx rhamni|EZSPC425-09|RVcoll.08-P070||Spain|658[0n]
Gonepteryx rhamni|EZSPC423-09|RVcoll.08-M706||Spain|658[0n]
Gonepteryx rhamni|WMB1611-13|RVcoll.12-O714||France|658[0n]
Gonepteryx rhamni|WMB4242-14|RVcoll.13-T863||Italy|658[0n]
Gonepteryx rhamni|WMB4033-14|RVcoll.12-Z165||Ireland|658[0n]
Gonepteryx rhamni|EULEP1358-15|RVcoll.14-F923||Greece|658[0n]
Gonepteryx rhamni|OXB1252-15|Rvcoll_14-E313bis|Corsica|France|658[0n]
Gonepteryx rhamni|EULEP889-15|RVcoll.14-C539||Greece|621[0n]
Gonepteryx rhamni|WMB1176-13|RVcoll.11-E841|Corsica|France|612[0n]
Gonepteryx rhamni|EZROM204-08|RV-07-D300||Romania|658[0n]
Gonepteryx rhamni|BIBSA1348-15|15-M844||Italy|658[0n]
Gonepteryx rhamni|WMB4961-14|RVcoll. 14-I508||Italy|658[0n]
Gonepteryx rhamni|WMB4899-14|RVcoll.14-I446||Italy|658[0n]
Gonepteryx rhamni|WMB4788-14|RVcoll. 14-I335||Italy|658[0n]
Gonepteryx rhamni|WMB4654-14|RVcoll.LD-2534||Italy|658[0n]
Gonepteryx rhamni|WMB899-13|RVcoll.09-X852||Italy|658[0n]
Gonepteryx rhamni|GWORU086-10|BC ZSM Lep 30347||Italy|658[0n]
Gonepteryx rhamni|WMB356-11|RVcoll.11-E840||France|658[0n]
Gonepteryx rhamni|EULEP5623-17|RVcoll14W017||Italy|658[0n]
Gonepteryx rhamni|EULEP1362-15|RVcoll.14-F929||Greece|658[0n]
Gonepteryx rhamni|WMB4800-14|RVcoll. 14-I347||Italy|658[0n]
Gonepteryx rhamni|BIBSA590-15|LEP-SS-00210||Italy|658[0n]
Gonepteryx rhamni|BIBSA589-15|LEP-SS-00209||Italy|658[0n]
Gonepteryx rhamni|BIBSA444-15|RVcoll.14-I112||Italy|658[0n]
Gonepteryx rhamni|BIBSA1391-15|15-M988||Italy|658[0n]
Gonepteryx rhamni|BIBSA1229-15|15-N035||Italy|658[0n]
Gonepteryx rhamni|EULEP1818-15|RVcoll.14-H824||Greece|658[0n]
Gonepteryx rhamni|EULEP1391-15|RVcoll.14-G004||Greece|658[0n]
Gonepteryx rhamni|EULEP1279-15|RVcoll.14-F686||Greece|658[0n]
Gonepteryx rhamni|EULEP4049-16|RVcoll15I071||Italy|658[0n]
Gonepteryx rhamni|WMB645-11|RVcoll.11-I186||Italy|658[0n]
Gonepteryx rhamni|GWORA2452-09|BC ZSM Lep 30664||Germany|658[0n]
Gonepteryx rhamni|OXB1283-15|Rvcoll_15-M144||France|658[0n]
Gonepteryx rhamni|WMB3032-14|RVcoll.13-S549|San Domino|Italy|658[0n]
Gonepteryx rhamni|EULEP1777-15|RVcoll.14-H553||Greece|658[0n]
Gonepteryx rhamni|EZROM290-08|RV-07-E652||Romania|609[0n]
Gonepteryx rhamni|LEFIJ499-10|MM17124||Finland|658[0n]
Gonepteryx rhamni|EULEP4051-16|RVcoll15I457||Austria|658[0n]
Gonepteryx rhamni|EULEP4046-16|RVcoll10A763||Estonia|658[0n]
Gonepteryx rhamni|LOWA166-06|2005-LOWA-166||Russia|658[0n]
Gonepteryx rhamni|LEATH786-14|TLMF Lep 15998||Italy|658[0n]
Gonepteryx rhamni|FBLMX237-11|BC ZSM Lep 50448||Germany|658[0n]
Gonepteryx rhamni|EULEP2423-15|RVcoll.14-V206||Belgium|614[0n]
Gonepteryx rhamni|LEFIJ4549-16|ZMBSU-02104||Belarus|613[0n]
Gonepteryx rhamni|WMB2232-13|RVcoll.11-Y086||Italy|630[0n]
Gonepteryx rhamni|LEATG037-14|TLMF Lep 13824||Austria|658[0n]
Gonepteryx rhamni|LEATG038-14|TLMF Lep 13825||Austria|658[0n]
Gonepteryx rhamni|EZSPC422-09|RVcoll.08-H231||Spain|658[0n]
Gonepteryx rhamni|EZSPC421-09|RVcoll.08-H221||Spain|658[0n]
Gonepteryx rhamni|EZROM1008-08|RVcoll.07-D304||Romania|658[0n]
Gonepteryx rhamni|EZROM583-08|RV-07-C368||Romania|658[0n]
Gonepteryx rhamni|EZROM202-08|RV-06-K609||Romania|658[0n]
Gonepteryx rhamni|FBLMT874-09|BC ZSM Lep 25434||Germany|658[0n]
Gonepteryx rhamni|LEFID863-10|MM06996||Finland|658[0n]
Gonepteryx rhamni|LEFIB372-10|MM00941||Finland|658[0n]
Gonepteryx rhamni|EULEP2127-15|RVcoll.14-J934||France|658[0n]
Gonepteryx rhamni|EULEP981-15|RVcoll.14-D134||Austria|658[0n]
Gonepteryx rhamni|EULEP1343-15|RVcoll.14-F851|Genit. examined|Greece|658[0n]
Gonepteryx rhamni|EULEP1258-15|RVcoll.14-F640||Greece|658[0n]
Gonepteryx rhamni|EULEP1111-15|RVcoll.14-F183||Serbia|658[0n]
Gonepteryx rhamni|EULEP755-15|RVcoll.12-Q567||Sweden|658[0n]
Gonepteryx rhamni|EULEP5018-16|RVcoll16I929||Slovakia|658[0n]
Gonepteryx rhamni|EULEP4953-16|RVcoll16I197||Germany|658[0n]
Gonepteryx rhamni|EULEP4736-16|RVcoll16G546||Sweden|658[0n]
Gonepteryx rhamni|EULEP4055-16|RVcoll15P037||Ukraine|658[0n]
Gonepteryx rhamni|EULEP188-14|RVcoll.11-J886||Romania|658[0n]
Gonepteryx rhamni|EULEP091-14|RVcoll.08-P978||Romania|658[0n]
Gonepteryx rhamni|EULEP2005-15|RVcoll.14-I790||Slovakia|658[0n]
Gonepteryx rhamni|EULEP1941-15|RVcoll.14-B913||Bosnia and Herzegovina|658[0n]
Gonepteryx rhamni|EULEP2233-15|RVcoll.14-N412||Russia|658[0n]

Gonepteryx rhamni|EULEP2005-15|RVcoll.14-I790||Slovakia|658[0n]
Gonepteryx rhamni|EULEP1941-15|RVcoll.14-B913||Bosnia and Herzegovina|658[0n]
Gonepteryx rhamni|EULEP2233-15|RVcoll.14-N412||Russia|658[0n]
Gonepteryx rhamni|EULEP2006-15|RVcoll.14-I795||Poland|658[0n]
Gonepteryx rhamni|BIBSA1097-15|15-L353||Italy|658[0n]
Gonepteryx rhamni|BIBSA1176-15|15-M207||Italy|658[0n]
Gonepteryx rhamni|GBLAA1091-15|BC ZSM Lep 86597||Germany|658[0n]
Gonepteryx rhamni|GBLAA1359-15|BC ZSM Lep 87055||Germany|658[0n]
Gonepteryx rhamni|GBLAC1014-13|BC ZSM Lep 76545||Germany|658[0n]
Gonepteryx rhamni|GBLAA2130-15|BC ZSM Lep 79846||Germany|658[0n]
Gonepteryx rhamni|GBLAC280-13|BC ZSM Lep 78661||Germany|658[0n]
Gonepteryx rhamni|GBLAB316-13|BC ZSM Lep 72617||Germany|658[0n]
Gonepteryx rhamni|LEFIJ4750-16|ZMBSU-02159||Belarus|658[0n]
Gonepteryx rhamni|OXB1447-16|OXB-TGS-1221||United Kingdom|658[0n]
Gonepteryx rhamni|OXB754-15|13-U484||Italy|658[0n]
Gonepteryx rhamni|OXB912-15|15-A556||Italy|658[0n]
Gonepteryx rhamni|OXB203-15|OXB-TGS-185||United Kingdom|658[0n]
Gonepteryx rhamni|OXB222-15|OXB-TGS-418||United Kingdom|658[0n]
Gonepteryx rhamni|OXB265-15|OXB-TGS-948||United Kingdom|658[0n]
Gonepteryx rhamni|OXB1400-15|Rvcoll_15-M767||France|658[0n]
Gonepteryx rhamni|BIBSA223-15|RVcoll.14-E011||Italy|636[0n]
Gonepteryx rhamni|EULEP4961-16|RVcoll16I300||Poland|640[0n]
Gonepteryx rhamni|EULEP4053-16|RVcoll15G163||France|638[0n]
Gonepteryx rhamni|EULEP4054-16|RVcoll15P019||Belarus|658[0n]
Gonepteryx rhamni|EULEP4052-16|RVcoll15I605||Austria|658[0n]
Gonepteryx rhamni|EULEP4050-16|RVcoll15I370||Austria|658[0n]
Gonepteryx rhamni|EULEP4048-16|RVcoll15H748||Switzerland|658[0n]
Gonepteryx rhamni|EULEP4047-16|RVcoll15H072||Italy|658[0n]
Gonepteryx rhamni|LOWA165-06|2005-LOWA-165||Russia|658[0n]
Gonepteryx rhamni|WMB5307-14|RVcoll.14-I672||France|658[0n]
Gonepteryx rhamni|WMB4929-14|RVcoll.14-I476||Italy|658[0n]
Gonepteryx rhamni|WMB5385-14|RVcoll.14-J846||France|658[0n]
Gonepteryx rhamni|WMB4808-14|RVcoll. 14-I355||Italy|658[0n]
Gonepteryx rhamni|WMB2190-13|RVcoll.09-V203||France|658[0n]
Gonepteryx rhamni|WMB3471-14|RVcoll.10-A643||France|658[0n]
Gonepteryx rhamni|EZSPC1167-10|RVcoll.09-T071||Spain|658[0n]
Gonepteryx rhamni|EZROM203-08|RV-06-K634||Romania|658[0n]
Gonepteryx rhamni|LON353-08|NHMO-08001||Norway|657[0n]
Gonepteryx rhamni|GWORA2451-09|BC ZSM Lep 30663||Germany|658[0n]
Gonepteryx rhamni|LEATI379-15|TLMF Lep 17859||Austria|658[0n]
Gonepteryx rhamni|LEATG521-14|TLMF Lep 14308||Italy|658[0n]
Gonepteryx rhamni|PHLAW013-13|TLMF Lep 09810||Austria|658[0n]
Gonepteryx rhamni|LASTS178-14|TLMF Lep 14630||Austria|658[0n]
Gonepteryx rhamni|LEATA049-13|TLMF Lep 09466||Italy|658[0n]
Gonepteryx rhamni|LEATJ1315-16|TLMF Lep 19638||Austria|658[0n]
Danaus plexippus|EZSPC1347-10|RVcoll.130209KL61||Spain|658[0n]
Danaus plexippus|EZSPM686-12|Rvcoll. 07-C577||Spain|658[0n]
Danaus plexippus|EZSPM685-12|Rvcoll. 07-C576||Spain|658[0n]
Danaus plexippus|EZSPM277-09|SMcoll.08-J600||Spain|658[0n]
Danaus chrysippus|WMB1906-13|RVcoll.11-H614|Lampedusa|Italy|634[0n]
Danaus chrysippus|EZSPN783-09|RVcoll.08-L326||Spain|658[0n]
Danaus chrysippus|EZSPM286-09|SMcoll.140907FY95||Spain|658[0n]
Danaus chrysippus|EZSPC1198-10|RVcoll.09-V389||Spain|658[0n]
Danaus chrysippus|EZSPC1197-10|RVcoll.09-V388||Spain|658[0n]
Danaus chrysippus|EZSPC1196-10|RVcoll.09-V387||Spain|658[0n]
Danaus chrysippus|EZSPC1195-10|RVcoll.09-V386||Spain|658[0n]
Danaus chrysippus|EZSPC1394-10|RVcoll.09-X596||Spain|658[0n]
Danaus chrysippus|EZSPC1393-10|RVcoll.09-X595||Spain|658[0n]
Danaus chrysippus|EZSPC1392-10|RVcoll.09-X594||Spain|658[0n]
Danaus chrysippus|EZSPM284-09|SMcoll.140907FY83||Spain|658[0n]
Danaus chrysippus|BIBSA610-15|LEP-SS-00230||Italy|658[0n]
Danaus chrysippus|WMB521-11|RVcoll.11-H613|Lampedusa|Italy|658[0n]
Danaus chrysippus|EZSPN784-09|RVcoll.08-L327||Spain|658[0n]
Euphydryas desfontainii|WMB3702-14|RVcoll.12-L049||Spain|658[0n]
Euphydryas desfontainii|EULEP2849-15|RVcoll.14-N933||France|658[0n]
Euphydryas desfontainii|EZSPC1310-10|RVcoll.130209KL99||Spain|658[0n]
Euphydryas desfontainii|EZSPC1319-10|RVcoll.130209KL32||Spain|658[0n]
Euphydryas desfontainii|EZSPC1106-10|RVcoll.08-M004||Spain|658[0n]
Euphydryas desfontainii|EZSPC1107-10|RVcoll.08-M005||Spain|658[0n]
Euphydryas desfontainii|WMB4460-14|RVcoll.14-D328||Spain|658[0n]
Euphydryas desfontainii|EZSPM851-12|Rvcoll. 201210GK02||Spain|633[0n]
Euphydryas desfontainii|EZSPC1105-10|RVcoll.08-M002||Spain|639[0n]
Euphydryas desfontainii|EZSPN976-09|RVcoll.08-M003||Spain|621[0n]
Euphydryas desfontainii|EZSPN640-09|RVcoll.08-J772||Spain|658[0n]
Euphydryas desfontainii|EZSPN547-09|RVcoll.08-J049||Spain|658[0n]
Euphydryas desfontainii|EZSPC1072-10|RVcoll.08-L402||Spain|658[0n]
Euphydryas desfontainii|EZSPN798-09|RVcoll.08-L401||Spain|658[0n]
Euphydryas desfontainii|EZSPC1073-10|RVcoll.08-L403||Spain|658[0n]
Euphydryas desfontainii|EZSPN143-09|RVcoll.06-G511||Spain|658[0n]
Euphydryas aurinia|EZSPN568-09|RVcoll.08-J109||Portugal|658[0n]
Euphydryas aurinia|EZSPN569-09|RVcoll.08-J110||Portugal|658[0n]
Euphydryas aurinia|EZSPN629-09|RVcoll.08-J726||Spain|658[0n]
Euphydryas aurinia|EZSPN855-09|RVcoll.08-L706||Spain|658[0n]
Euphydryas aurinia|WMB3178-14|RVcoll.08-H594||Spain|658[0n]
Euphydryas aurinia|WMB3190-14|RVcoll.08-J036||Spain|658[0n]
Euphydryas aurinia|WMB3214-14|RVcoll.08-J658||Spain|658[0n]
Euphydryas aurinia|EZSPN482-09|RVcoll.08-H919||Spain|658[1n]
Euphydryas aurinia|EZSPN474-09|RVcoll.08-H907||Spain|658[0n]
Euphydryas aurinia|EZSPN357-09|RVcoll.08-H334||Spain|658[0n]
Euphydryas aurinia|EZSPM242-09|RVcoll.08-R449||Spain|658[0n]
Euphydryas aurinia|EZSPM322-09|RVcoll.08-J894||Spain|631[0n]
Euphydryas aurinia|EZSPN1111-11|Rvcoll.10-B599||Spain|658[0n]
Euphydryas aurinia|EZSPC403-09|RVcoll.06-H892||Spain|658[0n]
Euphydryas aurinia|EZSPN058-09|RVcoll.09-V353||Spain|658[0n]
Euphydryas aurinia|WMB3560-14|RVcoll.11-D912||Spain|658[0n]
Euphydryas aurinia|EZSPC1053-10|RVcoll.08-J797||Spain|658[0n]
Euphydryas aurinia|EZSPC1299-10|RVcoll.210306RV7||Spain|658[0n]
Euphydryas aurinia|EZSPC1206-10|RVcoll.09-V474.1||Spain|658[0n]
Euphydryas aurinia|EZSPM424-09|Rvcoll.09-V599||Spain|658[0n]
Euphydryas aurinia|EZSPC406-09|RVcoll.08-L406||Spain|658[0n]
Euphydryas aurinia|EZSPC405-09|RVcoll.08-L405||Spain|658[0n]
Euphydryas aurinia|EZSPC404-09|RVcoll.08-L404||Spain|658[0n]
[illegible]

Euphydryas aurinia|EZSPC406-09|RVcoll.08-L406||Spain|658[0n]
Euphydryas aurinia|EZSPC405-09|RVcoll.08-L405||Spain|658[0n]
Euphydryas aurinia|EZSPC404-09|RVcoll.08-L404||Spain|658[0n]
Euphydryas aurinia|EZSPN709-09|RVcoll.08-L060||Spain|658[0n]
Euphydryas aurinia|OXB1024-15|15-A974||Italy|658[0n]
Euphydryas aurinia|BIBSA631-15|RVcoll. 15-A794||Italy|636[0n]
Euphydryas aurinia|EZSPC1376-10|RVcoll.09-X242||France|658[0n]
Euphydryas aurinia|EZSPC1375-10|RVcoll.09-X241||France|658[0n]
Euphydryas aurinia|WMB944-13|RVcoll.10-A639||France|658[0n]
Euphydryas aurinia|WMB3453-14|RVcoll.10-A507||France|658[0n]
Euphydryas aurinia|WMB260-11|RVcoll.10-A213||France|658[0n]
Euphydryas aurinia|ABOLD595-17|TLMF Lep 21671||Austria|658[0n]
Euphydryas aurinia|LEATJ1256-16|TLMF Lep 19579||Austria|658[0n]
Euphydryas aurinia|EULEP5141-17|RVcoll16J897||Serbia|658[0n]
Euphydryas aurinia|LEATJ1291-16|TLMF Lep 19614||Austria|658[0n]
Euphydryas aurinia|LEATJ1292-16|TLMF Lep 19615||Austria|658[0n]
Euphydryas aurinia|GWORR676-10|BC ZSM Lep 32172|Neotype|Germany|658[0n]
Euphydryas aurinia|GWOSU077-11|BC ZSM Lep 53328||Germany|658[0n]
Euphydryas aurinia|LENOA1371-11|LN-BD1371||France|658[0n]
Euphydryas aurinia|LEFIL393-10|MM18691||Finland|658[0n]
Euphydryas aurinia|FBLMU477-09|BC ZSM Lep 27127||Germany|658[0n]
Euphydryas aurinia|GWOSK894-11|BC ZSM Lep 49395||Germany|658[0n]
Euphydryas aurinia|GWOSK895-11|BC ZSM Lep 49396||Germany|658[0n]
Euphydryas aurinia|LEFID126-10|MM05864||Estonia|658[0n]
Euphydryas aurinia|EULEP4021-16|RVcoll15G121||France|658[0n]
Euphydryas aurinia|EULEP4739-16|RVcoll16G574||Sweden|658[0n]
Euphydryas aurinia|EULEP4013-16|RVcoll10A771||Estonia|658[0n]
Euphydryas aurinia|EULEP1061-15|RVcoll.14-E962||Serbia|658[0n]
Euphydryas aurinia|EULEP2274-15|RVcoll.14-N460||Ukraine|658[0n]
Euphydryas aurinia|EULEP2853-15|RVcoll.14-N937||United Kingdom|658[0n]
Euphydryas aurinia|LEASS735-17|TLMF Lep 22383||Austria|658[0n]
Euphydryas aurinia|OXB206-15|OXB-TGS-188||United Kingdom|658[0n]
Euphydryas aurinia|GBLAB323-13|BC ZSM Lep 72624||Germany|646[0n]
Euphydryas aurinia|LEATJ1293-16|TLMF Lep 19616||Austria|639[0n]
Euphydryas aurinia|GWOSA775-10|BC ZSM Lep 36641||Germany|634[0n]
Euphydryas aurinia|EULEP2559-15|RVcoll.14-V526||Ukraine|617[0n]
Euphydryas aurinia|EULEP4022-16|RVcoll15G239||Switzerland|627[0n]
Euphydryas aurinia|WMB758-12|RVcoll.11-J957||Italy|638[0n]
Euphydryas aurinia|PHLAB295-10|TLMF Lep 01095||Switzerland|636[0n]
Euphydryas aurinia|LEATJ1294-16|TLMF Lep 19617||Austria|658[0n]
Euphydryas aurinia|EULEP1847-15|RVcoll.13-S502||Italy|658[0n]
Euphydryas aurinia|PHLAH457-12|TLMF Lep 08276||Austria|658[0n]
Euphydryas aurinia|EZSPM006-09|RVcoll.08-M980||Andorra|639[0n]
Euphydryas aurinia|EZSPM004-09|RVcoll.08-M978||Andorra|634[0n]
Euphydryas aurinia|EZSPM005-09|RVcoll.08-M979||Andorra|634[0n]
Euphydryas aurinia|EZSPN051-09|RVcoll.09-V305||France|658[2n]
Euphydryas aurinia|EZSPC657-09|RVcoll.07-C678||France|658[0n]
Euphydryas aurinia|PHLAH296-12|TLMF Lep 07735||Italy|658[0n]
Euphydryas aurinia|BIBSA892-15|LD-3504||Italy|658[0n]
Euphydryas aurinia|EZROM182-08|RV-07-D348||Romania|658[0n]
Euphydryas aurinia|PHLAH295-12|TLMF Lep 07734||Italy|658[0n]
Euphydryas aurinia|EZROM183-08|RV-07-C115||Romania|658[0n]
Euphydryas aurinia|EZROM982-08|RVcoll.07-C116||Romania|658[0n]
Euphydryas aurinia|EULEP506-15|RVcoll.07-C531||Bulgaria|658[0n]
Euphydryas aurinia|EULEP504-15|RVcoll.07-C528||Bulgaria|658[0n]
Euphydryas aurinia|WMB759-12|RVcoll.11-J959||Italy|658[0n]
Euphydryas aurinia|WMB5232-14|RVcoll.13-U059||Italy|658[0n]
Euphydryas aurinia|EULEP4014-16|RVcoll15G677||Italy|658[0n]
Euphydryas aurinia|EULEP4015-16|RVcoll15G866||Switzerland|658[0n]
Euphydryas aurinia|EULEP4016-16|RVcoll15H575||Switzerland|658[0n]
Euphydryas aurinia|EULEP4017-16|RVcoll15H873||Italy|658[0n]
Euphydryas aurinia|EULEP4018-16|RVcoll15I025||Italy|658[0n]
Euphydryas aurinia|EULEP4019-16|RVcoll15I173||Austria|658[0n]
Euphydryas aurinia|EULEP4020-16|RVcoll15J035||Switzerland|658[0n]
Euphydryas aurinia|EULEP1058-15|RVcoll.14-E950||Serbia|658[0n]
Euphydryas aurinia|EULEP2076-15|RVcoll.14-J564||Ukraine|658[0n]
Euphydryas aurinia|EULEP1852-15|RVcoll.13-T930||Italy|658[0n]
Euphydryas aurinia|EULEP1869-15|RVcoll.13-U360||Italy|658[0n]
Euphydryas aurinia|EULEP2463-15|RVcoll.14-V289||Switzerland|658[0n]
Euphydryas aurinia|EULEP2558-15|RVcoll.14-V525||Ukraine|658[0n]
Euphydryas aurinia|EULEP2854-15|RVcoll.14-N938||United Kingdom|658[0n]
Euphydryas aurinia|BIBSA1038-15|15-C209||Italy|658[0n]
Euphydryas aurinia|BIBSA395-15|RVcoll.14-I063||Italy|658[0n]
Euphydryas aurinia|BIBSA165-15|RVcoll.14-I177||Italy|658[0n]
Euphydryas aurinia|BIBSA300-15|RVcoll.14-E102||Italy|658[0n]
Euphydryas aurinia|LEASS1023-17|TLMF Lep 22576||Austria|658[0n]
Euphydryas aurinia|LEASS1024-17|TLMF Lep 22577||Austria|658[0n]
Euphydryas aurinia|LEFIJ2527-15|MM23901||Finland|658[0n]
Euphydryas aurinia|LEFIJ2528-15|MM23902||Finland|658[0n]
Euphydryas aurinia|OXB829-15|14-U792||Italy|658[0n]
Euphydryas aurinia|OXB711-15|13-T893||Italy|658[0n]
Euphydryas aurinia|OXB915-15|15-A559||Italy|658[0n]
Euphydryas aurinia|OXB282-15|OXB-TGS-965||United Kingdom|658[0n]
Euphydryas aurinia|BIBSA1441-16|RVcoll.14-I195||Italy|658[0n]
Euphydryas aurinia|BIBSA1442-16|RVcoll.14-I196||Italy|658[0n]
Euphydryas aurinia|BIBSA891-15|LD-3503||Italy|658[0n]
Euphydryas aurinia|BIBSA161-15|RVcoll.14-I173||Italy|658[0n]
Euphydryas aurinia|BIBSA162-15|RVcoll.14-I174||Italy|658[0n]
Euphydryas aurinia|BIBSA1815-17|RVcoll14I284||Italy|658[0n]
Euphydryas aurinia|BIBSA1816-17|RVcoll14I285||Italy|658[0n]
Euphydryas aurinia|BIBSA1819-17|RVcoll14I288||Italy|658[0n]
Euphydryas aurinia|BIBSA1820-17|RVcoll14I289||Italy|658[0n]
Euphydryas aurinia|BIBSA1443-16|RVcoll.14-I197||Italy|644[0n]
Euphydryas aurinia|WMB2219-13|RVcoll.11-Y041||Italy|630[0n]
Euphydryas aurinia|WMB2801-13|RVcoll.11-Y042||Italy|658[0n]
Euphydryas aurinia|EZROM181-08|RV-07-D246||Romania|658[0n]
Euphydryas aurinia|EZROM986-08|RVcoll.07-D327||Romania|658[0n]
Euphydryas aurinia|EZROM984-08|RVcoll.07-D254||Romania|658[0n]
Euphydryas aurinia|EZROM985-08|RVcoll.07-D270||Romania|658[0n]
Euphydryas aurinia|EZROM983-08|RVcoll.07-D248||Romania|658[0n]
Euphydryas aurinia|FBLMX203-11|BC ZSM Lep 50414|ssp. glaciegenita (debilis)|Germany|658[0n]
Euphydryas aurinia|PHLAI490-13|TLMF Lep 09052||Austria|658[0n]
Euphydryas aurinia|PHLAH294-12|TLMF Lep 07733||Austria|658[0n]

Euphydryas aurinia|FBLMX203-11|BC ZSM Lep 50414|ssp. glaciegenita (debilis)|Germany|658[0n]
Euphydryas aurinia|PHLAI490-13|TLMF Lep 09052||Austria|658[0n]
Euphydryas aurinia|PHLAH294-12|TLMF Lep 07733||Austria|658[0n]
Euphydryas aurinia|PHLSA408-11|TLMF Lep 05863||Austria|658[0n]
Euphydryas aurinia|PHLAB346-10|TLMF Lep 01146||Switzerland|658[0n]
Euphydryas aurinia|PHLAB373-10|TLMF Lep 01173||Switzerland|658[0n]
Euphydryas aurinia|PHLSA698-11|TLMF Lep 06153||Austria|658[0n]
Euphydryas aurinia|PHLAB294-10|TLMF Lep 01094||Switzerland|658[0n]
Euphydryas aurinia|PHLAH293-12|TLMF Lep 07732||Austria|658[0n]
Euphydryas aurinia|LEATD137-13|TLMF Lep 12784||Italy|658[0n]
Euphydryas aurinia|LEATC110-13|TLMF Lep 11237||Italy|658[0n]
Euphydryas aurinia|PHLAB366-10|TLMF Lep 01166||Switzerland|658[0n]
Euphydryas cynthia|LEASS1022-17|TLMF Lep 22575||Austria|658[0n]
Euphydryas cynthia|LEASS898-17|KLM Lep 08403||Austria|658[0n]
Euphydryas cynthia|EULEP4026-16|RVcoll15I157||Austria|658[0n]
Euphydryas cynthia|EULEP921-15|RVcoll.14-C801||Bulgaria|658[0n]
Euphydryas cynthia|EULEP914-15|RVcoll.14-C773||Bulgaria|658[0n]
Euphydryas cynthia|EULEP4025-16|RVcoll15H534||Switzerland|658[0n]
Euphydryas cynthia|PHLAW022-13|TLMF Lep 09819||Austria|658[0n]
Euphydryas cynthia|LEATG227-14|TLMF Lep 14014||Austria|658[0n]
Euphydryas cynthia|LEATC088-13|TLMF Lep 11215||Italy|658[0n]
Euphydryas cynthia|BIBSA166-15|RVcoll.14-I178||Italy|658[0n]
Euphydryas cynthia|EULEP4028-16|RVcoll15I692||Austria|658[0n]
Euphydryas cynthia|EULEP4023-16|RVcoll14V911||Italy|658[0n]
Euphydryas cynthia|ABOLD593-17|TLMF Lep 21669||Austria|658[0n]
Euphydryas cynthia|EULEP4024-16|RVcoll15G737||Italy|658[0n]
Euphydryas cynthia|EULEP4027-16|RVcoll15I647||Austria|658[0n]
Euphydryas cynthia|EULEP4029-16|RVcoll14A992||Italy|658[0n]
Euphydryas cynthia|EULEP2855-15|RVcoll.14-N939||Italy|658[0n]
Euphydryas cynthia|BIBSA167-15|RVcoll.14-I179||Italy|658[0n]
Euphydryas cynthia|GBLAC369-13|BC ZSM Lep 75235||Germany|658[0n]
Euphydryas cynthia|OXB842-15|14-U805||Italy|658[0n]
Euphydryas cynthia|BIBSA307-15|RVcoll.14-E109||Italy|640[0n]
Euphydryas cynthia|GWORA2492-09|BC ZSM Lep 30704||Germany|647[0n]
Euphydryas cynthia|GWOTF678-12|BC ZSM Lep 62384||Germany|658[0n]
Euphydryas cynthia|LEATC089-13|TLMF Lep 11216||Italy|658[0n]
Euphydryas cynthia|LEATG226-14|TLMF Lep 14013||Austria|658[0n]
Euphydryas maturna|GWOSU1040-11|BC Back 0090||Italy|658[0n]
Euphydryas iduna|LEFID416-10|MM06340||Finland|658[0n]
Euphydryas iduna|EULEP4785-16|RVcoll16H435||Sweden|658[0n]
Euphydryas iduna|EULEP4718-16|RVcoll16B355||Norway|658[0n]
Euphydryas iduna|WMB6629-18|RVcoll16B368||Norway|658[0n]
Euphydryas iduna|LON211-08|NHMO-06223||Norway|657[0n]
Euphydryas iduna|LEFIA736-10|MM04090||Finland|658[0n]
Euphydryas intermedia|EULEP2860-15|RVcoll.14-N944||Russia|658[0n]
Euphydryas intermedia|EULEP2859-15|RVcoll.14-N943||Russia|658[0n]
Euphydryas iduna|LEFIA735-10|MM04089||Finland|658[0n]
Euphydryas maturna|EULEP4038-16|RVcoll14A977||Bulgaria|658[0n]
Euphydryas maturna|WMB6625-18|RVcoll14E799||Romania|658[0n]
Euphydryas maturna|EZROM991-08|RVcoll.08-M250||Romania|658[0n]
Euphydryas maturna|EZROM990-08|RVcoll.08-M249||Romania|658[0n]
Euphydryas intermedia|PHLAW061-13|TLMF Lep 09858||Austria|658[0n]
Euphydryas intermedia|BIBSA1190-15|15-M251||Italy|658[0n]
Euphydryas intermedia|LEATG434-14|TLMF Lep 14221||Italy|658[0n]
Euphydryas intermedia|LEATG433-14|TLMF Lep 14220||Italy|658[0n]
Euphydryas intermedia|EULEP806-15|RVcoll.13-U314||Italy|658[0n]
Euphydryas intermedia|WMB6624-18|RVcoll13U315||Italy|658[0n]
Euphydryas maturna|EULEP604-15|RVcoll.10-C406||Romania|658[0n]
Euphydryas maturna|EULEP996-15|RVcoll.14-E159||Romania|658[0n]
Euphydryas maturna|EULEP765-15|RVcoll.12-R760||Romania|658[0n]
Euphydryas maturna|EZROM992-08|RVcoll.08-M363||Romania|658[0n]
Euphydryas maturna|FBLMX202-11|BC ZSM Lep 50413||Germany|658[0n]
Euphydryas maturna|GWOSU1039-11|BC Back 0089||Germany|658[0n]
Euphydryas maturna|LEFIL394-10|MM18692||Finland|658[0n]
Euphydryas maturna|EULEP4712-16|RVcoll16B309||Sweden|658[0n]
Euphydryas maturna|EULEP4713-16|RVcoll16B311||Sweden|658[0n]
Euphydryas maturna|EULEP4836-16|RVcoll16H741||Ukraine|658[0n]
Euphydryas maturna|EULEP4858-16|RVcoll16H831||Poland|658[0n]
Euphydryas maturna|EULEP2273-15|RVcoll.14-N459||Ukraine|658[0n]
Euphydryas maturna|EULEP2577-15|RVcoll.14-V555||Ukraine|658[0n]
Euphydryas maturna|LEASS1020-17|TLMF Lep 22573||Austria|658[0n]
Euphydryas maturna|LEASS845-17|TLMF Lep 22493||Austria|658[0n]
Euphydryas maturna|LEFIJ2521-15|MM23275||Finland|643[0n]
Euphydryas maturna|LEFIJ2522-15|MM23276||Finland|643[0n]
Euphydryas maturna|FBLMZ174-12|BC ZSM Lep 51430||Germany|632[0n]
Euphydryas maturna|PHLAJ643-15|KLM Lep 03778||Austria|658[0n]
Euphydryas intermedia|LEASS1021-17|TLMF Lep 22574||Austria|658[0n]
Euphydryas intermedia|LEATJ1251-16|TLMF Lep 19574||Austria|658[0n]
Euphydryas maturna|EZROM184-08|RV-06-K677||Romania|658[0n]
Euphydryas maturna|EZROM988-08|RVcoll.06-K680||Romania|658[0n]
Euphydryas maturna|EZROM987-08|RVcoll.06-K678||Romania|658[0n]
Euphydryas maturna|EZROM989-08|RVcoll.06-K681||Romania|658[0n]
Euphydryas maturna|WMB6627-18|RVcoll15P071||Macedonia|658[0n]
Euphydryas maturna|WMB6628-18|RVcoll15P074||Macedonia|658[0n]
Euphydryas intermedia|EULEP4030-16|RVcoll15H370||Switzerland|658[0n]
Euphydryas intermedia|EULEP4031-16|RVcoll15H371||Switzerland|658[0n]
Euphydryas intermedia|EULEP4032-16|RVcoll15H433||Switzerland|658[0n]
Euphydryas intermedia|EULEP4033-16|RVcoll15H702||Switzerland|658[0n]
Euphydryas intermedia|EULEP4034-16|RVcoll15H703||Switzerland|658[0n]
Euphydryas maturna|EULEP4035-16|RVcoll15P069||Macedonia|658[0n]
Euphydryas maturna|EULEP4036-16|RVcoll15P070||Macedonia|658[0n]
Euphydryas maturna|EULEP4037-16|RVcoll15P072||Macedonia|658[0n]
Euphydryas intermedia|EULEP2454-15|RVcoll.14-V276||Switzerland|658[0n]
Euphydryas maturna|EULEP2576-15|RVcoll.14-V554||Ukraine|658[0n]
Euphydryas maturna|EULEP2850-15|RVcoll.14-N934||Serbia|658[0n]
Euphydryas maturna|EULEP2851-15|RVcoll.14-N935||Serbia|658[0n]
Euphydryas intermedia|OXB615-15|14-O004||Italy|658[0n]
Euphydryas maturna|ABOLD590-17|TLMF Lep 21666||Austria|658[0n]
Euphydryas maturna|ABOLD591-17|TLMF Lep 21667||Austria|658[0n]
Euphydryas maturna|EZROM185-08|RV-06-M841||Romania|643[2n]
Euphydryas maturna|LEFIJ800-10|MM17425||Finland|658[0n]
Euphydryas maturna|LEFIJ799-10|MM17424||Finland|658[0n]
Euphydryas maturna|[illegible]|MM17175||Finland|658[0n]

Euphydryas maturna|EZROM[illegible]||Romania|[illegible]
Euphydryas maturna|LEFIJ800-10|MM17425||Finland|658[0n]
Euphydryas maturna|LEFIJ799-10|MM17424||Finland|658[0n]
Euphydryas maturna|LEFIJ550-10|MM17175||Finland|658[0n]
Euphydryas intermedia|LEATG222-14|TLMF Lep 14009||Austria|658[0n]
Euphydryas intermedia|LEATG221-14|TLMF Lep 14008||Austria|658[0n]
Melitaea trivia|EZSPN834-09|RVcoll.08-L655||Spain|658[0n]
Melitaea trivia|WMB4401-14|RVcoll.14-B412||Portugal|658[0n]
Melitaea trivia|EZSPM100-09|RVcoll.08-P430||Spain|658[0n]
Melitaea trivia|EZSPN868-09|RVcoll.08-L746|Genit. examined|Spain|658[1n]
Melitaea trivia|WMB3207-14|RVcoll.08-J216||Spain|658[0n]
Melitaea trivia|WMB3418-14|RVcoll.09-X048||Spain|658[0n]
Melitaea trivia|WMB3357-14|RVcoll.09-T130||Spain|658[0n]
Melitaea trivia|EZSPC1285-10|RVcoll.150707PK18||Spain|658[0n]
Melitaea trivia|EZSPC1283-10|RVcoll.270808DT38||Spain|658[0n]
Melitaea trivia|EZROM739-08|RV-06-G474||Spain|658[0n]
Melitaea trivia|EZSPN493-09|RVcoll.08-H931||Spain|658[0n]
Melitaea trivia|EZSPC558-09|RVcoll.06-G476||Spain|658[0n]
Melitaea trivia|EZSPC557-09|RVcoll.06-G475||Spain|658[0n]
Melitaea trivia|EZSPN176-09|RVcoll.06-K712||Spain|658[0n]
Melitaea trivia|EZSPN175-09|RVcoll.06-K711||Spain|658[0n]
Melitaea trivia|EZSPC559-09|RVcoll.06-G477||Spain|658[0n]
Melitaea trivia|EZSPC643-09|RVcoll.08-L373||Spain|658[0n]
Melitaea trivia|EULEP2983-15|RVcoll.14-O116|Samos|Greece|658[0n]
Melitaea trivia|EULEP2982-15|RVcoll.14-O115|Samos|Greece|658[0n]
Melitaea trivia|EULEP2985-15|RVcoll.14-O118|Symi|Greece|658[0n]
Melitaea trivia|EULEP2986-15|RVcoll.14-O119|Symi|Greece|631[0n]
Melitaea trivia|EULEP2984-15|RVcoll.14-O117|Symi|Greece|631[0n]
Melitaea trivia|EULEP4298-16|RVcoll14V857||Bulgaria|658[0n]
Melitaea trivia|EULEP4297-16|RVcoll10B326||Bulgaria|658[0n]
Melitaea trivia|EULEP2980-15|RVcoll.14-O113||Russia|658[0n]
Melitaea trivia|EULEP2976-15|RVcoll.14-O109||Russia|658[0n]
Melitaea trivia|EULEP2975-15|RVcoll.14-O108||Russia|658[0n]
Melitaea trivia|EULEP419-14|KN00781||Russia|658[1n]
Melitaea trivia|OXB848-15|14-U811||Italy|658[0n]
Melitaea trivia|WMB5016-14|RVcoll. 14-I563||Italy|658[0n]
Melitaea trivia|EZROM642-08|RV-07-D481||Romania|658[0n]
Melitaea trivia|EULEP1236-15|RVcoll.14-F551||Greece|658[0n]
Melitaea trivia|EULEP2339-15|RVcoll.14-V025||Ukraine|658[0n]
Melitaea trivia|EZRMN122-08|RVcoll.07-D591||Romania|658[0n]
Melitaea trivia|EZROM426-08|RV-06-M925||Romania|658[0n]
Melitaea trivia|EZROM428-08|RV-07-E353||Romania|658[0n]
Melitaea trivia|EULEP2979-15|RVcoll.14-O112||Macedonia|658[0n]
Melitaea trivia|EULEP1643-15|RVcoll.10-B325||Bulgaria|658[0n]
Melitaea trivia|EULEP1417-15|RVcoll.14-G087||Greece|658[0n]
Melitaea trivia|EULEP564-15|RVcoll.14-G039||Greece|658[0n]
Melitaea trivia|EULEP1749-15|RVcoll.14-H381||Greece|658[0n]
Melitaea trivia|EULEP1748-15|RVcoll.14-H380||Greece|658[0n]
Melitaea trivia|EULEP1732-15|RVcoll.14-H266||Greece|658[0n]
Melitaea trivia|EULEP1570-15|RVcoll.14-G573||Macedonia|658[0n]
Melitaea trivia|LEATJ1323-16|TLMF Lep 19646||Greece|658[0n]
Melitaea trivia|LEATJ1322-16|TLMF Lep 19645||Greece|658[0n]
Melitaea trivia|EULEP1685-15|RVcoll.14-G997||Greece|658[0n]
Melitaea trivia|EULEP4918-16|RVcoll16J624||Greece|658[0n]
Melitaea trivia|EULEP1515-15|RVcoll.14-G436||Greece|658[0n]
Melitaea trivia|EULEP1078-15|RVcoll.14-F020||Serbia|658[0n]
Melitaea trivia|EULEP5034-16|RVcoll16I986||Slovakia|658[0n]
Melitaea trivia|EZROM427-08|RV-07-D218||Romania|658[0n]
Melitaea trivia|ABOLD597-17|TLMF Lep 21673||Austria|658[0n]
Melitaea trivia|EZRMN124-08|RVcoll.08-M407||Romania|658[0n]
Melitaea trivia|EULEP2981-15|RVcoll.14-O114||Russia|625[0n]
Melitaea trivia|LEASS1008-17|TLMF Lep 22561||Austria|658[0n]
Melitaea trivia|EULEP2192-15|RVcoll.14-N329||Ukraine|658[0n]
Melitaea trivia|EULEP021-14|RVcoll.07-D058||Romania|658[0n]
Melitaea trivia|EZRMN123-08|RVcoll.07-E301||Romania|658[0n]
Melitaea trivia|EZRMN125-08|RVcoll.08-M420||Romania|658[0n]
Melitaea trivia|WMB2642-13|RVcoll.12-Q754||Italy|658[0n]
Melitaea trivia|WMB836-13|RVcoll.07-E231||Italy|658[0n]
Melitaea trivia|BIBSA991-15|15-C014||Italy|658[0n]
Melitaea trivia|BIBSA603-15|LEP-SS-00223||Italy|632[0n]
Melitaea trivia|BIBSA741-15|LEP-SS-00268||Italy|658[0n]
Melitaea trivia|WMB2466-13|RVcoll.11-H369||Italy|658[0n]
Melitaea trivia|WMB2065-13|RVcoll.12-Q875||Italy|658[0n]
Melitaea trivia|EULEP5700-17|RVcoll16L043||Italy|658[0n]
Melitaea trivia|BIBSA085-14|LEP-SS-00085||Italy|658[0n]
Melitaea trivia|WMB2646-13|RVcoll.12-M546||Italy|658[0n]
Melitaea trivia|WMB2064-13|RVcoll.12-Q874||Italy|658[0n]
Melitaea trivia|GWORU090-10|BC ZSM Lep 30351||Italy|658[0n]
Melitaea cinxia|LEASS766-17|TLMF Lep 22414||Austria|658[0n]
Melitaea cinxia|LEASS756-17|TLMF Lep 22404||Austria|658[0n]
Melitaea cinxia|LEFIB318-10|MM00849||Finland|658[0n]
Melitaea cinxia|EZSPN530-09|RVcoll.08-J000||Spain|658[0n]
Melitaea cinxia|WMB3213-14|RVcoll.08-J654||Spain|658[0n]
Melitaea cinxia|EULEP4883-16|RVcoll16H989||Denmark|658[0n]
Melitaea cinxia|WMB3358-14|RVcoll.09-T140||Spain|658[0n]
Melitaea cinxia|EZSPC1308-10|RVcoll.130209KL31||Spain|658[0n]
Melitaea cinxia|EZSPC541-09|RVcoll.06-H880||Spain|616[0n]
Melitaea cinxia|WMB3188-14|RVcoll.08-J019||Spain|658[0n]
Melitaea cinxia|EZROM735-08|RV-06-G454||Spain|658[0n]
Melitaea cinxia|EZSPC1344-10|RVcoll.130209KL22||Spain|658[0n]
Melitaea cinxia|EZSPN978-09|RVcoll.08-M012||Spain|658[0n]
Melitaea cinxia|EZSPM804-12|Rvcoll. 090211SD72||Spain|658[0n]
Melitaea cinxia|EZSPN884-09|RVcoll.08-L793||Spain|658[0n]
Melitaea cinxia|EZSPN870-09|RVcoll.08-L753||Spain|658[0n]
Melitaea cinxia|EZSPC544-09|RVcoll.08-L497||Spain|658[0n]
Melitaea cinxia|EZSPN501-09|RVcoll.08-H951||Spain|658[0n]
Melitaea cinxia|EZSPN833-09|RVcoll.08-L654||Spain|658[0n]
Melitaea cinxia|BIBSA1261-15|15-F892||France|658[0n]
Melitaea cinxia|WMB3907-14|RVcoll.12-P321||France|658[0n]
Melitaea cinxia|EULEP2977-15|RVcoll.14-O110|Isle of Wight|United Kingdom|658[0n]
Melitaea cinxia|WMB1695-13|RVcoll.12-P570||France|658[0n]
Melitaea cinxia|WMB5287-14|RVcoll.14-E424||France|658[0n]
Melitaea cinxia|WMB5314-14|RVcoll.14-I747||France|658[0n]

Melitaea cinxia|WMB1695-13|RVcoll.12-P570||France|658[0n]
Melitaea cinxia|WMB5287-14|RVcoll.14-E424||France|658[0n]
Melitaea cinxia|WMB5314-14|RVcoll.14-I747||France|658[0n]
Melitaea cinxia|EZSPC542-09|RVcoll.07-C629||Spain|634[0n]
Melitaea cinxia|EZSPC543-09|RVcoll.08-J388||Spain|658[0n]
Melitaea cinxia|WMB1774-13|RVcoll.12-Q066||France|658[0n]
Melitaea cinxia|WMB3464-14|RVcoll.10-A610||France|658[0n]
Melitaea cinxia|PHLAB357-10|TLMF Lep 01157||Switzerland|658[0n]
Melitaea cinxia|WMB2814-13|RVcoll.12-M564|Sicily|Italy|658[0n]
Melitaea cinxia|BIBSA1584-16|12-M457||Italy|658[0n]
Melitaea cinxia|WMB1286-13|RVcoll.12-M456|Sicily|Italy|658[0n]
Melitaea cinxia|LEATG452-14|TLMF Lep 14239||Italy|658[0n]
Melitaea cinxia|BIBSA1739-16|11-I211||Italy|658[0n]
Melitaea cinxia|EULEP2134-15|RVcoll.14-J977||Switzerland|658[0n]
Melitaea cinxia|OXB944-15|15-A588||Italy|658[0n]
Melitaea cinxia|OXB1137-15|15-A670||Italy|658[0n]
Melitaea cinxia|OXB1107-15|15-A640||Italy|658[0n]
Melitaea cinxia|BIBSA1340-15|15-M832||Italy|658[0n]
Melitaea cinxia|EULEP4269-16|RVcoll15C270||Italy|658[0n]
Melitaea cinxia|WMB4943-14|RVcoll. 14-I490||Italy|658[0n]
Melitaea cinxia|WMB4853-14|RVcoll.14-I400||Italy|658[0n]
Melitaea cinxia|WMB4790-14|RVcoll. 14-I337||Italy|658[0n]
Melitaea cinxia|WMB4167-14|RVcoll.13-S728||Italy|658[0n]
Melitaea cinxia|WMB2233-13|RVcoll.11-Y089||Italy|658[0n]
Melitaea cinxia|GWORU092-10|BC ZSM Lep 30353||Italy|658[0n]
Melitaea cinxia|WMB629-11|RVcoll.11-I107|Sicily|Italy|658[0n]
Melitaea cinxia|WMB1282-13|RVcoll.12-M413|Sicily|Italy|632[0n]
Melitaea cinxia|BIBSA089-14|LEP-SS-00089||Italy|658[0n]
Melitaea cinxia|BIBSA683-15|RVcoll. 11-I210||Italy|658[0n]
Melitaea cinxia|WMB3856-14|RVcoll.11-I169||Italy|658[0n]
Melitaea cinxia|BIBSA243-15|RVcoll.14-E036||Italy|635[0n]
Melitaea cinxia|LEASS886-17|KLM Lep 08391||Austria|658[0n]
Melitaea cinxia|WMB5169-14|RVcoll.13-S513||Italy|658[0n]
Melitaea cinxia|OXB874-15|15-A516||Italy|658[0n]
Melitaea cinxia|WMB5199-14|RVcoll.13-T915||Italy|658[0n]
Melitaea cinxia|WMB2600-13|RVcoll.12-R405||Italy|658[0n]
Melitaea cinxia|LEATI048-15|TLMF Lep 17433||Austria|658[0n]
Melitaea cinxia|LEATG451-14|TLMF Lep 14238||Italy|658[0n]
Melitaea cinxia|WMB3855-14|RVcoll.11-I166||Italy|658[0n]
Melitaea cinxia|WMB2222-13|RVcoll.11-Y048||Italy|658[0n]
Melitaea cinxia|WMB2348-13|RVcoll.12-M499||Italy|658[0n]
Melitaea cinxia|WMB2349-13|RVcoll.12-M500||Italy|658[0n]
Melitaea cinxia|BIBSA1330-15|12-R413|Sicily|Italy|658[0n]
Melitaea cinxia|BIBSA1557-16|LEP-SS-00383||Italy|658[0n]
Melitaea cinxia|BIBSA493-15|LEP-SS-00113||Italy|658[0n]
Melitaea cinxia|WMB1973-13|RVcoll.11-I208||Italy|619[0n]
Melitaea cinxia|WMB3529-14|RVcoll.10-C095||France|658[0n]
Melitaea cinxia|WMB3485-14|RVcoll.10-B641||France|658[0n]
Melitaea cinxia|LEATI049-15|TLMF Lep 17434||Austria|658[0n]
Melitaea cinxia|ABOLB058-15|TLMF Lep 17063||Italy|658[0n]
Melitaea cinxia|ABOLB059-15|TLMF Lep 17064||Italy|658[0n]
Melitaea cinxia|LEASS884-17|KLM Lep 08389||Austria|658[0n]
Melitaea cinxia|EULEP1714-15|RVcoll.14-H173||Greece|658[0n]
Melitaea cinxia|EULEP1772-15|RVcoll.14-H530||Greece|658[0n]
Melitaea cinxia|EULEP1762-15|RVcoll.14-H431||Greece|658[0n]
Melitaea cinxia|EULEP1489-15|RVcoll.14-G321||Greece|658[0n]
Melitaea cinxia|EULEP1756-15|RVcoll.14-H417||Greece|658[0n]
Melitaea cinxia|EZRMN106-08|RVcoll.07-D040||Romania|658[0n]
Melitaea cinxia|EZRMN108-08|RVcoll.08-M308||Romania|658[0n]
Melitaea cinxia|EULEP1168-15|RVcoll.14-F376||Bulgaria|658[0n]
Melitaea cinxia|EZROM412-08|RV-07-D164||Romania|658[0n]
Melitaea cinxia|EZROM163-08|RV-07-C981||Romania|654[0n]
Melitaea cinxia|LEASS1007-17|TLMF Lep 22560||Austria|658[0n]
Melitaea cinxia|EZRMN109-08|RVcoll.08-M359||Romania|658[0n]
Melitaea cinxia|GWORK317-09|BC ZSM Lep 21552||Germany|658[0n]
Melitaea cinxia|GWOSK891-11|BC ZSM Lep 49392||Germany|658[0n]
Melitaea cinxia|GWOSK892-11|BC ZSM Lep 49393||Germany|658[0n]
Melitaea cinxia|EZROM413-08|RV-07-D473||Romania|658[0n]
Melitaea cinxia|EZROM639-08|RV-07-D319||Romania|658[0n]
Melitaea cinxia|EZRMN107-08|RVcoll.07-D288||Romania|658[0n]
Melitaea cinxia|EULEP040-14|RVcoll.08-H003||Romania|658[0n]
Melitaea cinxia|GWORR677-10|BC ZSM Lep 32173||Germany|632[0n]
Melitaea cinxia|GWOTF694-12|BC ZSM Lep 62400||Croatia|658[0n]
Melitaea cinxia|BIBSA994-15|15-C021||Italy|658[0n]
Melitaea cinxia|WMB5494-14|RVcoll.LD-3377||Italy|658[0n]
Melitaea cinxia|LEASS755-17|TLMF Lep 22403||Austria|658[0n]
Melitaea cinxia|BIBSA1238-15|15-D903||France|658[0n]
Melitaea cinxia|EULEP1642-15|RVcoll.14-F552||Greece|658[0n]
Melitaea cinxia|WMB3427-14|RVcoll.09-X275||France|658[0n]
Melitaea cinxia|WMB255-11|RVcoll.09-V282||France|658[0n]
Melitaea cinxia|FBLMT871-09|BC ZSM Lep 25431||Germany|658[0n]
Melitaea cinxia|LON932-12|NHMO Lep2010.005||Norway|658[0n]
Melitaea cinxia|ABOLD415-16|TLMF Lep 21567||Austria|658[0n]
Melitaea didyma|OXB1173-15|Rvcoll_16-A060||Italy|658[0n]
Melitaea didyma|EULEP5861-18|RVcoll15K607||Italy|658[0n]
Melitaea arduinna|EULEP2959-15|RVcoll.14-O092||Russia|658[0n]
Melitaea arduinna|EULEP5612-17|RVcoll14V529||Ukraine|655[0n]
Melitaea arduinna|EULEP5611-17|RVcoll14V528||Ukraine|655[0n]
Melitaea arduinna|EULEP5598-17|RVcoll14U600||Russia|655[0n]
Melitaea arduinna|EULEP5574-17|RVcoll14G534||Greece|655[0n]
Melitaea arduinna|EULEP5552-17|RVcoll14E591||Romania|655[0n]
Melitaea arduinna|EULEP5551-17|RVcoll14E556||Romania|655[0n]
Melitaea arduinna|EULEP5554-17|RVcoll14E672||Romania|655[0n]
Melitaea arduinna|EULEP5553-17|RVcoll14E622||Romania|655[0n]
Melitaea arduinna|EULEP5556-17|RVcoll14E863||Serbia|655[0n]
Melitaea arduinna|EULEP5555-17|RVcoll14E804||Romania|655[0n]
Melitaea arduinna|EULEP5557-17|RVcoll14F154||Serbia|655[0n]
Melitaea arduinna|EULEP5558-17|RVcoll14F155||Serbia|655[0n]
Melitaea arduinna|EULEP5559-17|RVcoll14F226||Serbia|655[0n]
Melitaea arduinna|EULEP5560-17|RVcoll14F227||Serbia|655[0n]
Melitaea arduinna|EULEP5561-17|RVcoll14F320||Serbia|655[0n]
Melitaea arduinna|EULEP5586-17|RVcoll14J524||Ukraine|655[0n]
Melitaea arduinna|EULEP5593-17|RVcoll14N518||Macedonia|655[0n]

Melitaea arduinna|EULEP5561-17|RVcoll14F320||Serbia|655[0n]
Melitaea arduinna|EULEP5586-17|RVcoll14J524||Ukraine|655[0n]
Melitaea arduinna|EULEP5593-17|RVcoll14N518||Macedonia|655[0n]
Melitaea arduinna|EULEP5594-17|RVcoll14N519||Macedonia|655[0n]
Melitaea arduinna|EULEP2960-15|RVcoll.14-O093||Russia|658[0n]
Melitaea arduinna|EULEP5532-17|RVcoll14A976||Bulgaria|658[0n]
Melitaea arduinna|EULEP5531-17|RVcoll14A975||Bulgaria|658[0n]
Melitaea diamina|EULEP5894-18|RVcoll16L860||Spain|658[0n]
Melitaea diamina|EULEP2965-15|RVcoll.14-O098||Spain|658[0n]
Melitaea diamina|EZSPC1258-10|RVcoll.280808DF35||Spain|658[0n]
Melitaea diamina|BIBSA742-15|LEP-SS-00269||Italy|658[0n]
Melitaea diamina|OXB316-15|RVcoll.14-N030||Italy|658[0n]
Melitaea diamina|EZSPC1189-10|RVcoll.09-V317||Spain|658[0n]
Melitaea diamina|EZSPC546-09|RVcoll.07-C681|Genit. examined|Spain|658[0n]
Melitaea diamina|WMB5178-14|RVcoll.13-S526||Italy|658[0n]
Melitaea diamina|PHLAI520-13|TLMF Lep 09082||Austria|620[0n]
Melitaea diamina|WMB830-13|RVcoll.07-E212||Italy|633[0n]
Melitaea diamina|EULEP640-15|RVcoll.11-J120||Switzerland|658[0n]
Melitaea diamina|EULEP2466-15|RVcoll.14-V292||Switzerland|658[0n]
Melitaea diamina|EULEP1980-15|RVcoll.14-H916||France|658[0n]
Melitaea diamina|BIBSA244-15|RVcoll.14-E038||Italy|658[0n]
Melitaea diamina|LEASS1050-17|TLMF Lep 22603||Austria|658[0n]
Melitaea diamina|LEASS584-17|TLMF Lep 22232||Austria|658[0n]
Melitaea diamina|OXB375-15|RVcoll.14-N089||Italy|658[0n]
Melitaea diamina|EULEP4277-16|RVcoll15G137||France|636[0n]
Melitaea diamina|EULEP4278-16|RVcoll15G236||Switzerland|643[0n]
Melitaea diamina|EULEP4276-16|RVcoll15G080||France|642[0n]
Melitaea diamina|EULEP4275-16|RVcoll15I677||Austria|658[0n]
Melitaea diamina|EULEP4274-16|RVcoll15H600||Switzerland|658[0n]
Melitaea diamina|EULEP4273-16|RVcoll15H311||Switzerland|658[0n]
Melitaea diamina|EULEP4272-16|RVcoll15H192||Switzerland|658[0n]
Melitaea diamina|EULEP4271-16|RVcoll15G994||Switzerland|658[0n]
Melitaea diamina|EULEP4741-16|RVcoll16G658||Sweden|658[0n]
Melitaea diamina|WMB1859-13|RVcoll.11-J159||France|658[0n]
Melitaea diamina|EZSPC1174-10|RVcoll.09-T133||Spain|658[0n]
Melitaea diamina|EZSPC1173-10|RVcoll.09-T132||Spain|658[0n]
Melitaea diamina|EZSPC986-10|RVcoll.07-C671||Spain|658[0n]
Melitaea diamina|EZSPC1243-10|RVcoll.09-V756||Spain|658[0n]
Melitaea diamina|EZSPM787-12|Rvcoll. 070611MH11||Spain|658[0n]
Melitaea diamina|EZROM736-08|RV-07-C676||Spain|658[0n]
Melitaea diamina|EZSPC548-09|RVcoll.08-R115||Spain|658[0n]
Melitaea diamina|EZSPC547-09|RVcoll.07-W140||Spain|658[0n]
Melitaea diamina|EZSPC545-09|RVcoll.08-P219|Genit. examined|Spain|658[0n]
Melitaea diamina|LEFIL380-10|MM18690||Finland|658[0n]
Melitaea diamina|LEFIL379-10|MM18689||Finland|658[0n]
Melitaea diamina|PHLAA683-09|TLMF Lep 00723||Austria|658[0n]
Melitaea diamina|LEATD292-13|TLMF Lep 12939||Italy|658[0n]
Melitaea diamina|PHLAI547-13|TLMF Lep 09109||Austria|658[0n]
Melitaea diamina|PHLSA656-11|TLMF Lep 06111||Austria|658[0n]
Melitaea diamina|LEATG437-14|TLMF Lep 14224||Italy|658[0n]
Melitaea diamina|EZRMN111-08|RVcoll.06-M880||Romania|658[0n]
Melitaea diamina|EULEP1021-15|RVcoll.14-E761||Romania|658[0n]
Melitaea diamina|EULEP4279-16|RVcoll14E348||Romania|658[0n]
Melitaea diamina|EULEP1143-15|RVcoll.14-F305||Serbia|658[0n]
Melitaea diamina|EULEP1059-15|RVcoll.14-E960||Serbia|658[0n]
Melitaea diamina|EZRMN110-08|RVcoll.06-M864||Romania|658[0n]
Melitaea diamina|EZRMN112-08|RVcoll.08-M592||Romania|658[0n]
Melitaea diamina|EZROM415-08|RV-07-D542||Romania|658[0n]
Melitaea diamina|EZROM414-08|RV-06-M863||Romania|658[0n]
Melitaea diamina|EZROM681-08|RV-07-D528||Romania|658[0n]
Melitaea diamina|EZROM680-08|RV-06-M866||Romania|658[0n]
Melitaea diamina|EULEP318-14|MM23803||Latvia|658[0n]
Melitaea diamina|EULEP1919-15|RVcoll.14-B842||Bosnia and Herzegovina|658[0n]
Melitaea diamina|EULEP2218-15|RVcoll.14-N396||Ukraine|658[0n]
Melitaea diamina|EULEP2235-15|RVcoll.14-N415||Ukraine|658[0n]
Melitaea diamina|LEASS688-17|TLMF Lep 22336||Austria|658[0n]
Melitaea diamina|LEASS1062-17|TLMF Lep 22615||Austria|658[0n]
Melitaea diamina|LEASS879-17|KLM Lep 08384||Austria|658[0n]
Melitaea diamina|OXB1303-15|Rvcoll_15-M173||France|658[0n]
Melitaea diamina|EULEP4915-16|RVcoll16J618||Russia|636[0n]
Melitaea diamina|EULEP4867-16|RVcoll16H896||Poland|658[0n]
Melitaea diamina|EULEP4823-16|RVcoll16H719||Belgium|658[0n]
Melitaea diamina|GWORK313-09|BC ZSM Lep 21548||Germany|658[0n]
Melitaea diamina|ODOPE246-11|BC ZSM Lep 53117||Germany|658[0n]
Melitaea diamina|GWORA2500-09|BC ZSM Lep 30712||Germany|658[0n]
Melitaea diamina|GWORR678-10|BC ZSM Lep 32174||Germany|658[0n]
Melitaea diamina|ABOLD089-16|TLMF Lep 21167||Austria|658[0n]
Melitaea deione|BIBSA1515-16|RVcoll.14-I269||Italy|618[0n]
Melitaea deione|BIBSA890-15|LD-3502||Italy|658[0n]
Melitaea deione|BIBSA885-15|LD-3497||Italy|658[0n]
Melitaea deione|WMB842-13|RVcoll.07-E285||France|658[0n]
Melitaea deione|EULEP2961-15|RVcoll.14-O094||Switzerland|658[0n]
Melitaea deione|WMB786-13|RVcoll.07-C502|ex. ovo|Italy|632[0n]
Melitaea deione|WMB785-13|RVcoll.07-C501|ex. ovo|Italy|658[0n]
Melitaea deione|EZSPN101-09|RVcoll.06-A041|Genit. examined|Spain|651[0n]
Melitaea deione|WMB4567-14|RVcoll.130711PX37||Spain|658[0n]
Melitaea deione|EZSPM261-09|RVcoll.08-R475||Spain|658[0n]
Melitaea deione|EZSPC1081-10|RVcoll.08-L681||Spain|658[0n]
Melitaea deione|EZSPC1427-10|RVcoll.09-V138||Spain|658[0n]
Melitaea deione|EZSPM614-12|RVcoll.11-D821||Spain|658[0n]
Melitaea deione|EZSPM613-12|RVcoll.11-D820||Spain|658[0n]
Melitaea deione|EZSPN1007-09|RVcoll.08-M699||Spain|658[0n]
Melitaea deione|EZSPC1080-10|RVcoll.08-L680||Spain|658[0n]
Melitaea deione|EZSPC1291-10|RVcoll.140602MU8||Spain|658[0n]
Melitaea deione|EZSPM120-09|RVcoll.08-P604||Spain|658[0n]
Melitaea deione|EZSPC1271-10|RVcoll.290208WQ20||Spain|658[0n]
Melitaea deione|EZSPC1270-10|RVcoll.290208WQ35||Spain|658[0n]
Melitaea deione|EZSPN311-09|RVcoll.08-J663||Spain|658[0n]
Melitaea deione|EZSPM330-09|RVcoll.08-R055||Spain|658[0n]
Melitaea deione|EULEP5919-18|RVcoll15A753||Portugal|658[0n]
Melitaea deione|EZSPN1012-09|RVcoll.08-M932||Spain|658[0n]
Melitaea deione|EZSPN081-09|RVcoll.08-J650||Spain|658[0n]
[illegible]

Melitaea deione|EULEP5919-18|RVcoll15A755||Portugal|658[0n]
Melitaea deione|EZSPN1012-09|RVcoll.08-M932||Spain|658[0n]
Melitaea deione|EZSPN081-09|RVcoll.08-J650||Spain|658[0n]
Melitaea deione|WMB1687-13|RVcoll.12-P540||France|658[0n]
Melitaea deione|EZSPN131-09|RVcoll.06-G472|Genit. examined|Spain|658[0n]
Melitaea deione|EZSPN844-09|RVcoll.08-L679||Spain|639[0n]
Melitaea varia|LEASS1017-17|TLMF Lep 22570||Austria|658[0n]
Melitaea varia|LEASS1016-17|TLMF Lep 22569||Austria|658[1n]
Melitaea varia|LEATG453-14|TLMF Lep 14240||Italy|658[0n]
Melitaea varia|WMB1615-13|RVcoll.12-O744||France|658[0n]
Melitaea varia|LEATJ1330-16|TLMF Lep 19653||Italy|658[0n]
Melitaea varia|PHLAA415-09|TLMF Lep 00455||France|658[0n]
Melitaea varia|PHLAF621-11|TLMF Lep 05791||France|658[0n]
Melitaea varia|EULEP4304-16|RVcoll15G325||Switzerland|658[0n]
Melitaea varia|EULEP4303-16|RVcoll15G234||Switzerland|658[0n]
Melitaea varia|LEASS673-17|TLMF Lep 22321||Austria|658[0n]
Melitaea varia|OXB846-15|14-U809||Italy|658[0n]
Melitaea varia|BIBSA417-15|RVcoll.14-I085||Italy|658[0n]
Melitaea varia|LEASS672-17|TLMF Lep 22320||Austria|658[0n]
Melitaea varia|LEASS671-17|TLMF Lep 22319||Austria|658[0n]
Melitaea varia|EULEP4302-16|RVcoll15J081||Switzerland|658[0n]
Melitaea varia|EULEP4301-16|RVcoll15J051||Switzerland|658[0n]
Melitaea varia|LEATC090-13|TLMF Lep 11217||Italy|658[0n]
Melitaea varia|PHLAB1221-10|TLMF Lep 02021||Italy|658[0n]
Melitaea varia|EULEP816-15|RVcoll.14-A274||Italy|658[0n]
Melitaea varia|EULEP817-15|RVcoll.14-A292||Italy|658[0n]
Melitaea varia|OXB769-15|14-A236||Italy|658[0n]
Melitaea varia|PHLAB1220-10|TLMF Lep 02020||Italy|644[0n]
Melitaea varia|EULEP4300-16|RVcoll15H657||Switzerland|658[0n]
Melitaea varia|BIBSA416-15|RVcoll.14-I084||Italy|658[0n]
Melitaea varia|BIBSA415-15|RVcoll.14-I083||Italy|658[0n]
Melitaea varia|BIBSA414-15|RVcoll.14-I082||Italy|658[0n]
Melitaea varia|WMB2696-13|RVcoll.10-C057||France|658[0n]
Melitaea varia|EULEP4299-16|RVcoll15H543||Switzerland|658[0n]
Melitaea varia|PHLAF640-11|TLMF Lep 05810||Switzerland|658[0n]
Melitaea varia|PHLAB377-10|TLMF Lep 01177||Switzerland|658[0n]
Melitaea varia|PHLAB380-10|TLMF Lep 01180||Switzerland|658[0n]
Melitaea parthenoides|WMB3349-14|RVcoll.08-R277||Spain|658[0n]
Melitaea parthenoides|EZSPC985-10|RVcoll.07-C483||Spain|658[0n]
Melitaea parthenoides|EZSPC999-10|RVcoll.07-Z074||Spain|658[0n]
Melitaea parthenoides|EZSPN296-09|RVcoll.07-Z073||Spain|658[0n]
Melitaea parthenoides|WMB3363-14|RVcoll.09-T174||Spain|658[0n]
Melitaea parthenoides|EZSPC1032-10|RVcoll.08-J115||Portugal|658[0n]
Melitaea parthenoides|EZSPM149-09|RVcoll.08-P664||Spain|658[0n]
Melitaea parthenoides|EZSPN567-09|RVcoll.08-J108||Portugal|658[0n]
Melitaea parthenoides|EULEP5885-18|RVcoll15O585||Spain|658[2n]
Melitaea parthenoides|WMB3282-14|RVcoll.08-M089||Spain|658[0n]
Melitaea parthenoides|WMB3322-14|RVcoll.08-P733||Spain|658[0n]
Melitaea parthenoides|EULEP5884-18|RVcoll15O584||Spain|658[0n]
Melitaea parthenoides|EZSPC1175-10|RVcoll.09-T134||Spain|642[0n]
Melitaea parthenoides|EZSPN1000-09|RVcoll.08-M084||Spain|658[0n]
Melitaea parthenoides|EZSPN890-09|RVcoll.08-L801|Genit. examined|Spain|658[0n]
Melitaea parthenoides|EZSPN832-09|RVcoll.08-L650||Spain|658[0n]
Melitaea parthenoides|EZSPC1031-10|RVcoll.08-J112||Portugal|658[0n]
Melitaea parthenoides|EZSPN657-09|RVcoll.08-J955||Spain|658[0n]
Melitaea parthenoides|WMB3354-14|RVcoll.08-R422||Spain|658[0n]
Melitaea parthenoides|EZSPM427-09|Rvcoll.09-X546||Spain|653[0n]
Melitaea parthenoides|WMB5296-14|RVcoll.14-I596||France|658[0n]
Melitaea parthenoides|EZSPM647-12|RVcoll.11-I423||Spain|658[0n]
Melitaea parthenoides|EZSPN742-09|RVcoll.08-L166||Spain|658[0n]
Melitaea parthenoides|EULEP4268-16|RVcoll15J648||France|658[0n]
Melitaea parthenoides|EULEP2119-15|RVcoll.14-J880||France|658[0n]
Melitaea parthenoides|BIBSA1264-15|15-F932||France|658[0n]
Melitaea parthenoides|OXB1537-16|OXB-TGS-1255||France|658[0n]
Melitaea parthenoides|EULEP4249-16|RVcoll15J591||France|642[0n]
Melitaea parthenoides|WMB4564-14|RVcoll.090611SZ34||Spain|658[0n]
Melitaea parthenoides|WMB4546-14|RVcoll.030111FB49||Spain|658[0n]
Melitaea parthenoides|EZSPC1066-10|RVcoll.08-L182||Spain|658[0n]
Melitaea parthenoides|EZSPC1303-10|RVcoll.140602MU13||Spain|658[0n]
Melitaea parthenoides|EZSPC1288-10|100908AW5||Spain|658[0n]
Melitaea parthenoides|EZSPC1257-10|RVcoll.280808DF25||Spain|658[0n]
Melitaea parthenoides|EZSPM475-09|Rvcoll.09-V443||Spain|658[0n]
Melitaea parthenoides|EZSPM474-09|Rvcoll.09-V435||Spain|657[0n]
Melitaea parthenoides|EZSPN656-09|RVcoll.08-J952||Spain|658[0n]
Melitaea parthenoides|LENOA1365-11|LN-BD1365||France|658[0n]
Melitaea parthenoides|LENOA1364-11|LN-BD1364||France|658[0n]
Melitaea aurelia|EZROM409-08|RV-06-M961|Genit. examined|Romania|658[0n]
Melitaea aurelia|EULEP4232-16|RVcoll10B304|Genit. examined|Bulgaria|658[0n]
Melitaea aurelia|ABOLD640-17|TLMF Lep 21716||Austria|658[0n]
Melitaea aurelia|ABOLD592-17|TLMF Lep 21668||Austria|658[0n]
Melitaea aurelia|EULEP1023-15|RVcoll.14-E787||Romania|614[0n]
Melitaea aurelia|EULEP4262-16|RVcoll15P083||Macedonia|658[0n]
Melitaea aurelia|ABOLD638-17|TLMF Lep 21714||Austria|658[0n]
Melitaea aurelia|ABOLD637-17|TLMF Lep 21713||Austria|658[0n]
Melitaea aurelia|LEASS882-17|KLM Lep 08387||Austria|658[0n]
Melitaea aurelia|EULEP1648-15|RVcoll.14-F129||Serbia|658[0n]
Melitaea aurelia|EULEP1107-15|RVcoll.14-F174||Serbia|658[0n]
Melitaea aurelia|EULEP1103-15|RVcoll.14-F163||Serbia|658[0n]
Melitaea aurelia|EULEP4261-16|RVcoll15P082||Macedonia|658[0n]
Melitaea aurelia|EULEP4231-16|RVcoll10A933||Bulgaria|658[0n]
Melitaea aurelia|EULEP1174-15|RVcoll.14-F398||Bulgaria|658[0n]
Melitaea aurelia|EULEP1171-15|RVcoll.14-F382||Bulgaria|658[0n]
Melitaea aurelia|LEASS1018-17|TLMF Lep 22571||Austria|658[0n]
Melitaea aurelia|EZROM410-08|RV-07-D421|Genit. examined|Romania|658[0n]
Melitaea aurelia|EULEP519-15|RVcoll.14-E949||Serbia|658[0n]
Melitaea aurelia|EULEP1118-15|RVcoll.14-F167||Serbia|658[0n]
Melitaea aurelia|EULEP1012-15|RVcoll.14-E601||Romania|658[0n]
Melitaea aurelia|EZRMN104-08|RVcoll.07-D411|Genit. examined|Romania|658[0n]
Melitaea aurelia|EZROM407-08|RV-06-K676|Genit. examined|Romania|658[0n]
Melitaea aurelia|EZRMN105-08|RVcoll.08-M280|Genit. examined|Romania|658[0n]
Melitaea aurelia|EZRMN103-08|RVcoll.07-D387|Genit. examined|Romania|658[0n]
Melitaea aurelia|EZRMN102-08|RVcoll.06-M825|Genit. examined|Romania|658[0n]
Melitaea aurelia|EZRMN333-08|RVcoll.08-H005|Genit. examined|Romania|658[0n]

Melitaea aurelia|EZRMN103-08|RVcoll.07-D387|Genit. examined|Romania|658[0n]
Melitaea aurelia|EZRMN102-08|RVcoll.06-M825|Genit. examined|Romania|658[0n]
Melitaea aurelia|EZRMN333-08|RVcoll.08-H005|Genit. examined|Romania|658[0n]
Melitaea aurelia|EZRMN334-08|RVcoll.08-H006|Genit. examined|Romania|658[0n]
Melitaea aurelia|BIBSA887-15|LD-3499||Italy|658[0n]
Melitaea aurelia|BIBSA886-15|LD-3498||Italy|658[0n]
Melitaea aurelia|EULEP766-15|RVcoll.12-R765||Romania|658[0n]
Melitaea aurelia|EULEP1005-15|RVcoll.14-E620||Romania|613[0n]
Melitaea aurelia|EULEP2259-15|RVcoll.14-N443||Ukraine|658[0n]
Melitaea aurelia|EULEP1121-15|RVcoll.14-F215||Serbia|658[0n]
Melitaea aurelia|EULEP1006-15|RVcoll.14-E838||Serbia|658[0n]
Melitaea aurelia|EULEP1003-15|RVcoll.14-E778||Romania|658[0n]
Melitaea aurelia|EZROM411-08|RV-07-C980|Genit. examined|Romania|658[0n]
Melitaea aurelia|EZROM408-08|RV-06-M931|Genit. examined|Romania|658[0n]
Melitaea aurelia|GBLAB320-13|BC ZSM Lep 72621||Germany|658[0n]
Melitaea aurelia|ABOLD639-17|TLMF Lep 21715||Austria|658[0n]
Melitaea aurelia|EULEP2989-15|RVcoll.14-O122||France|658[0n]
Melitaea aurelia|LEEUA530-11|MM20589||Latvia|658[0n]
Melitaea aurelia|FBLMU476-09|BC ZSM Lep 27126|ID verfied by dissection|Germany|630[0n]
Melitaea aurelia|GBLAB778-13|BC ZSM Lep 75549||Germany|658[0n]
Melitaea aurelia|GBLAB779-13|BC ZSM Lep 75550||Germany|658[0n]
Melitaea aurelia|EULEP2168-15|RVcoll.14-K091||Switzerland|625[0n]
Melitaea aurelia|PHLAI550-13|TLMF Lep 09112||Austria|658[0n]
Melitaea asteria|EULEP4229-16|RVcoll15I195||Austria|658[0n]
Melitaea asteria|EULEP4228-16|RVcoll15I194||Austria|658[0n]
Melitaea asteria|PHLSA404-11|TLMF Lep 05859||Austria|658[0n]
Melitaea asteria|LEASS1013-17|TLMF Lep 22566||Austria|658[0n]
Melitaea asteria|LEASS1012-17|TLMF Lep 22565||Austria|658[0n]
Melitaea asteria|EULEP4227-16|RVcoll15H459||Switzerland|658[0n]
Melitaea asteria|EULEP4226-16|RVcoll15H458||Switzerland|658[0n]
Melitaea asteria|PHLSA409-11|TLMF Lep 05864||Austria|658[0n]
Melitaea asteria|PHLSA410-11|TLMF Lep 05865||Austria|658[0n]
Melitaea asteria|LEATC285-13|TLMF Lep 11412||Austria|658[0n]
Melitaea aurelia|LEATG454-14|TLMF Lep 14241||Italy|658[0n]
Melitaea aurelia|EULEP4265-16|RVcoll15H164||Italy|658[0n]
Melitaea aurelia|EULEP4264-16|RVcoll15H145||Italy|658[0n]
Melitaea aurelia|EULEP4263-16|RVcoll15H095||Italy|658[0n]
Melitaea aurelia|EULEP4237-16|RVcoll15H019||Italy|658[0n]
Melitaea aurelia|WMB2231-13|RVcoll.11-Y077||Italy|658[0n]
Melitaea aurelia|LEATG455-14|TLMF Lep 14242||Italy|648[2n]
Melitaea aurelia|ABOLA860-15|TLMF Lep 16820||Austria|658[0n]
Melitaea aurelia|ABOLA859-15|TLMF Lep 16819||Austria|658[0n]
Melitaea athalia|LEATJ1297-16|TLMF Lep 19620||Austria|658[0n]
Melitaea athalia|EULEP2387-15|RVcoll.14-V084||Ukraine|658[0n]
Melitaea athalia|EULEP2380-15|RVcoll.14-V075||Ukraine|658[0n]
Melitaea athalia|EZRMN372-08|RVcoll.08-M103|Genit. examined|Romania|658[0n]
Melitaea athalia|EULEP2234-15|RVcoll.14-N413||Ukraine|658[0n]
Melitaea athalia|EULEP4251-16|RVcoll15Q078||Russia|658[0n]
Melitaea athalia|EULEP4252-16|RVcoll15P033||Ukraine|658[0n]
Melitaea athalia|EULEP317-14|MM23802||Latvia|658[0n]
Melitaea athalia|EULEP4267-16|RVcoll15Q076||Russia|658[0n]
Melitaea athalia|EULEP5036-16|RVcoll16J000||Slovakia|658[0n]
Melitaea athalia|EULEP2015-15|RVcoll.14-I815||Slovakia|658[0n]
Melitaea athalia|EULEP348-14|MM23833||Lithuania|658[0n]
Melitaea athalia|LOWA291-06|2005-LOWA-291||Russia|658[0n]
Melitaea athalia|LOWA290-06|2005-LOWA-290||Russia|658[0n]
Melitaea athalia|LEFIJ549-10|MM17174||Finland|658[0n]
Melitaea athalia|EZROM693-08|RV-07-C312|Genit. examined|Romania|658[0n]
Melitaea athalia|EZRMN373-08|RVcoll.08-M104|Genit. examined|Romania|658[0n]
Melitaea athalia|EULEP4939-16|RVcoll16I052||Poland|658[0n]
Melitaea athalia|EULEP785-15|RVcoll.12-Z197||Sweden|658[0n]
Melitaea athalia|EULEP4909-16|RVcoll16J612||Russia|658[0n]
Melitaea athalia|EULEP347-14|MM23832||Lithuania|658[0n]
Melitaea athalia|EULEP4784-16|RVcoll16H415||Sweden|658[0n]
Melitaea athalia|LEFIE848-10|MM10073||Finland|658[0n]
Melitaea athalia|LEFID245-10|MM06109||Finland|658[0n]
Melitaea athalia|LON203-08|NHMO-06212||Sweden|657[0n]
Melitaea athalia|LON023-08|NHMO-06023||Norway|657[0n]
Melitaea athalia|EULEP970-15|RVcoll.14-D059||Bulgaria|658[0n]
Melitaea athalia|EULEP869-15|RVcoll.14-C353||Greece|658[0n]
Melitaea athalia|EULEP515-15|RVcoll.14-F538||Greece|658[0n]
Melitaea athalia|EULEP1234-15|RVcoll.14-F546||Greece|658[0n]
Melitaea athalia|EULEP1142-15|RVcoll.14-F303||Serbia|658[0n]
Melitaea athalia|EULEP1096-15|RVcoll.14-F120||Serbia|658[0n]
Melitaea athalia|EULEP1513-15|RVcoll.14-G434||Greece|658[0n]
Melitaea athalia|EULEP2988-15|RVcoll.14-O121||Greece|658[0n]
Melitaea athalia|EULEP1149-15|RVcoll.14-F327|Genit. examined|Serbia|658[0n]
Melitaea athalia|EULEP5829-18|RVcoll14U528||Albania|658[0n]
Melitaea athalia|EZRMN101-08|RVcoll.08-M597|Genit. examined|Romania|658[0n]
Melitaea athalia|EZRMN100-08|RVcoll.08-M346|Genit. examined|Romania|658[0n]
Melitaea athalia|EULEP4230-16|RVcoll10A789||Estonia|658[0n]
Melitaea athalia|EZROM694-08|RV-07-C314|Genit. examined|Romania|652[0n]
Melitaea athalia|EZRMN374-08|RVcoll.08-M105|Genit. examined|Romania|658[0n]
Melitaea athalia|OXB740-15|13-U296||Italy|658[0n]
Melitaea athalia|OXB747-15|13-U438||Italy|658[0n]
Melitaea athalia|EULEP4242-16|RVcoll15I087|Genit. examined|Italy|658[0n]
Melitaea athalia|EULEP4241-16|RVcoll15I041|Genit. examined|Italy|658[0n]
Melitaea athalia|EULEP1122-15|RVcoll.14-F216||Serbia|658[0n]
Melitaea athalia|EZROM406-08|RV-07-E394|Genit. examined|Romania|658[0n]
Melitaea athalia|EZROM404-08|RV-07-D295|Genit. examined|Romania|658[0n]
Melitaea athalia|GWOSN694-11|BC ZSM Lep 52900||Germany|658[0n]
Melitaea athalia|GWORR680-10|BC ZSM Lep 32176||Germany|658[0n]
Melitaea athalia|LEATF462-14|TLMF Lep 13774||Austria|658[0n]
Melitaea athalia|EZRMN099-08|RVcoll.08-M222|Genit. examined|Romania|658[0n]
Melitaea athalia|EZROM405-08|RV-07-D976|Genit. examined|Romania|658[0n]
Melitaea athalia|EULEP514-15|RVcoll.14-F060||Serbia|658[0n]
Melitaea athalia|EZROM403-08|RV-06-M839|Genit. examined|Romania|658[0n]
Melitaea athalia|EZROM402-08|RV-06-K602|Genit. examined|Romania|658[0n]
Melitaea athalia|EULEP1042-15|RVcoll.14-E908||Serbia|658[0n]
Melitaea athalia|EULEP1040-15|RVcoll.14-E904||Serbia|658[0n]
Melitaea athalia|EULEP1070-15|RVcoll.14-E989||Serbia|658[0n]
Melitaea athalia|GWOTD804-12|BC ZSM Lep 64695||Croatia|658[0n]
Melitaea athalia|EULEP4250-16|RVcoll15G067||France|636[0n]

Melitaea athalia|EULEP1040-15|RVcoll.14-E904||Serbia|658[0n]
Melitaea athalia|EULEP1070-15|RVcoll.14-E989||Serbia|658[0n]
Melitaea athalia|GWOTD804-12|BC ZSM Lep 64695||Croatia|658[0n]
Melitaea athalia|EULEP4250-16|RVcoll15G067||France|636[0n]
Melitaea athalia|GWOSN693-11|BC ZSM Lep 52899|(det. H Kolbeck) previously aurelia (MS ...
Melitaea athalia|GWORR679-10|BC ZSM Lep 32175|(det. H Kolbeck) previously aurelia (MS ...
Melitaea athalia|GWOSA739-10|BC ZSM Lep 35750||Germany|658[0n]
Melitaea athalia|FBLMU112-09|BC ZSM Lep 25622||Germany|658[0n]
Melitaea athalia|FBLMT898-09|BC ZSM Lep 25458||Germany|658[0n]
Melitaea athalia|FBLMU119-09|BC ZSM Lep 25629||Germany|650[0n]
Melitaea athalia|GWOSK889-11|BC ZSM Lep 49390||Germany|658[0n]
Melitaea athalia|LEATJ1299-16|TLMF Lep 19622||Austria|658[0n]
Melitaea athalia|GWORA2498-09|BC ZSM Lep 30710|(det. H Kolbeck) previously aurelia (MS ...
Melitaea athalia|LEASS514-17|TLMF Lep 22162||Austria|658[0n]
Melitaea athalia|BIBSA1116-15|15-L852|Genit. examined|Italy|658[0n]
Melitaea athalia|BIBSA1115-15|15-L848|Genit. examined|Italy|658[0n]
Melitaea athalia|EULEP4820-16|RVcoll16H716||Belgium|658[0n]
Melitaea athalia|GWOSA740-10|BC ZSM Lep 35751||Germany|658[0n]
Melitaea athalia|GWORA2497-09|BC ZSM Lep 30709||Germany|658[0n]
Melitaea athalia|FBLMX265-11|BC ZSM Lep 50476||Germany|658[0n]
Melitaea athalia|FBLMZ162-12|BC ZSM Lep 51418||Germany|658[0n]
Melitaea athalia|ABOLD023-16|TLMF Lep 21101||Austria|658[0n]
Melitaea athalia|EULEP4246-16|RVcoll15I725||Austria|658[0n]
Melitaea athalia|LEASS497-17|TLMF Lep 22145||Austria|658[0n]
Melitaea athalia|LEASS489-17|TLMF Lep 22137||Austria|658[0n]
Melitaea athalia|LEASS880-17|KLM Lep 08385||Austria|658[0n]
Melitaea athalia|EULEP4247-16|RVcoll15I809||Austria|658[0n]
Melitaea athalia|EULEP4245-16|RVcoll15I661||Austria|658[0n]
Melitaea athalia|EULEP4243-16|RVcoll15I360||Austria|658[0n]
Melitaea athalia|LEATJ1298-16|TLMF Lep 19621||Austria|658[0n]
Melitaea athalia|LEATJ1296-16|TLMF Lep 19619||Austria|658[0n]
Melitaea athalia|EULEP1900-15|RVcoll.14-B773||Albania|658[0n]
Melitaea athalia|EULEP1177-15|RVcoll.14-F407|Genit. examined|Bulgaria|658[0n]
Melitaea athalia|EULEP1030-15|RVcoll.14-E853||Serbia|658[0n]
Melitaea athalia|EULEP1261-15|RVcoll.14-F650|Genit. examined|Greece|658[0n]
Melitaea athalia|EULEP1270-15|RVcoll.14-F666|Genit. examined|Greece|658[0n]
Melitaea athalia|EULEP1031-15|RVcoll.14-E859|Genit. examined|Serbia|613[0n]
Melitaea athalia|EULEP5918-18|RVcoll17G017||Albania|658[0n]
Melitaea athalia|EULEP5916-18|RVcoll17G015||Albania|658[0n]
Melitaea britomartis|EULEP1018-15|RVcoll.14-E727||Romania|658[0n]
Melitaea britomartis|EULEP1020-15|RVcoll.14-E733|Genit. examined|Romania|658[0n]
Melitaea britomartis|EULEP1007-15|RVcoll.14-E766||Romania|614[0n]
Melitaea britomartis|EULEP2369-15|RVcoll.14-V063||Ukraine|658[0n]
Melitaea britomartis|EULEP2381-15|RVcoll.14-V076||Ukraine|612[0n]
Melitaea britomartis|EULEP2207-15|RVcoll.14-N367|Genit. examined|Ukraine|658[0n]
Melitaea britomartis|LEASS1014-17|TLMF Lep 22567||Austria|658[0n]
Melitaea britomartis|EULEP4266-16|RVcoll15Q069||Russia|658[0n]
Melitaea britomartis|EZROM588-08|RV-07-C313|Genit. examined|Romania|658[0n]
Melitaea britomartis|LEASS675-17|TLMF Lep 22323||Austria|658[0n]
Melitaea britomartis|LEASS674-17|TLMF Lep 22322||Austria|658[0n]
Melitaea britomartis|LEASS585-17|TLMF Lep 22233||Austria|658[0n]
Melitaea britomartis|LEASS581-17|TLMF Lep 22229||Austria|658[0n]
Melitaea britomartis|EULEP088-14|RVcoll.08-P974||Romania|658[0n]
Melitaea britomartis|EULEP2386-15|RVcoll.14-V083||Ukraine|658[0n]
Melitaea britomartis|EULEP2197-15|RVcoll.14-N356|Genit. examined|Ukraine|658[0n]
Melitaea britomartis|EULEP2236-15|RVcoll.14-N416|Genit. examined|Ukraine|658[0n]
Melitaea britomartis|EULEP2208-15|RVcoll.14-N369|Genit. examined|Ukraine|614[0n]
Melitaea britomartis|EZRMN370-08|RVcoll.08-M100|Genit. examined|Romania|658[0n]
Melitaea britomartis|GWOSA741-10|BC ZSM Lep 35752||Germany|658[0n]
Melitaea britomartis|EULEP2992-15|RVcoll.14-O125||Sweden|658[0n]
Melitaea britomartis|GBLAD165-14|BC ZSM Lep 78736||Germany|658[0n]
Melitaea britomartis|GBLAB772-13|BC ZSM Lep 75543||Germany|658[0n]
Melitaea britomartis|EULEP2991-15|RVcoll.14-O124||Sweden|631[0n]
Melitaea britomartis|FBLMU265-09|BC ZSM Lep 25775||Germany|658[0n]
Melitaea britomartis|FBLMU260-09|BC ZSM Lep 25770|ID verfied by dissection|Germany|658[0n]
Melitaea britomartis|FBLMU256-09|BC ZSM Lep 25766|ID verfied by dissection|Germany|658[0n]
Melitaea britomartis|GWOSK890-11|BC ZSM Lep 49391||Germany|658[0n]
Melitaea celadussa|EZSPN494-09|RVcoll.08-H935||Spain|658[0n]
Melitaea celadussa|EZSPN473-09|RVcoll.08-H904||Spain|658[0n]
Melitaea celadussa|EZSPC1426-10|RVcoll.09-V137||Spain|658[0n]
Melitaea celadussa|EZSPC1425-10|RVcoll.09-V136||Spain|658[0n]
Melitaea celadussa|EZSPC1424-10|RVcoll.09-V135||Spain|658[0n]
Melitaea celadussa|WMB4520-14|RVcoll.14-E220||Spain|658[0n]
Melitaea celadussa|EZSPM601-12|RVcoll.11-I433||Spain|658[0n]
Melitaea celadussa|EZSPC1056-10|RVcoll.08-J954||Spain|638[0n]
Melitaea celadussa|EZSPC1423-10|RVcoll.09-V134||Spain|658[0n]
Melitaea celadussa|EZSPM660-12|RVcoll.11-I507||Spain|658[0n]
Melitaea celadussa|EULEP4239-16|RVcoll15H589||Switzerland|658[0n]
Melitaea celadussa|OXB1588-16|OXB-TGS-1306||France|658[0n]
Melitaea celadussa|WMB5381-14|RVcoll.14-J820||France|658[0n]
Melitaea celadussa|WMB1834-13|RVcoll.11-I830||France|658[0n]
Melitaea celadussa|WMB3968-14|RVcoll.12-Q105||France|658[0n]
Melitaea celadussa|WMB1775-13|RVcoll.12-Q078||France|658[0n]
Melitaea celadussa|EZSPN1010-09|RVcoll.08-M915||Spain|658[0n]
Melitaea celadussa|EZSPC257-09|RVcoll.08-P395|Genit. examined|Spain|655[0n]
Melitaea celadussa|WMB4040-14|RVcoll.12-Z356||Spain|658[0n]
Melitaea celadussa|EZSPC207-09|RVcoll.08-P394|Genit. examined|Spain|654[0n]
Melitaea celadussa|EZSPC1298-10|RVcoll.100908AW26||Spain|658[0n]
Melitaea celadussa|EZSPC1273-10|RVcoll.220602AM3||Spain|658[0n]
Melitaea celadussa|EZSPC1269-10|RVcoll.210907WR65||Spain|658[0n]
Melitaea celadussa|EZROM734-08|RV-07-C071||Spain|658[0n]
Melitaea celadussa|WMB4553-14|RVcoll.070611MH09||Spain|658[0n]
Melitaea celadussa|WMB4180-14|RVcoll.13-S845||Portugal|658[0n]
Melitaea celadussa|WMB3297-14|RVcoll.08-P221||Spain|658[0n]
Melitaea celadussa|EZSPN997-09|RVcoll.08-M074||Spain|658[0n]
Melitaea celadussa|EZSPN913-09|RVcoll.08-L852|Genit. examined|Spain|658[0n]
Melitaea celadussa|EZSPM304-09|RVcoll.08-J851||Spain|658[0n]
Melitaea celadussa|EZSPN583-09|RVcoll.08-J143||Portugal|658[0n]
Melitaea celadussa|EZSPM027-09|RVcoll.08-P214||France|658[0n]
Melitaea celadussa|EZSPC560-09|RVcoll.07-C680|Genit. examined|Spain|658[0n]
Melitaea celadussa|EZSPC539-09|RVcoll.07-C635||Spain|658[0n]
Melitaea celadussa|OXB966-15|15-A916||Italy|658[0n]
Melitaea celadussa|OXB965-15|15-A915||Italy|658[0n]

Melitaea celadussa|EZSPC539-09|RVcoll.07-C635||Spain|658[0n]
Melitaea celadussa|OXB966-15|15-A916||Italy|658[0n]
Melitaea celadussa|OXB965-15|15-A915||Italy|658[0n]
Melitaea celadussa|OXB964-15|15-A914||Italy|658[0n]
Melitaea celadussa|EULEP2994-15|RVcoll.14-O127||Italy|658[0n]
Melitaea celadussa|BIBSA1862-17|RVcoll16C754||Italy|658[0n]
Melitaea celadussa|WMB5095-14|RVcoll. 14-L226||Italy|658[0n]
Melitaea celadussa|WMB5240-14|RVcoll.13-U092||Italy|658[0n]
Melitaea celadussa|WMB4947-14|RVcoll. 14-I494||Italy|658[0n]
Melitaea celadussa|BIBSA1346-15|15-M840||Italy|658[0n]
Melitaea celadussa|WMB4879-14|RVcoll.14-I426||Italy|658[0n]
Melitaea celadussa|WMB1082-13|RVcoll.10-C728||Italy|658[0n]
Melitaea celadussa|BIBSA957-15|12-R404||Italy|658[0n]
Melitaea celadussa|WMB4686-14|RVcoll.LD-2686||Italy|658[0n]
Melitaea celadussa|BIBSA1747-16|11-H980||Italy|658[0n]
Melitaea celadussa|WMB551-11|RVcoll.11-H741|Sicily|Italy|658[0n]
Melitaea celadussa|WMB2575-13|RVcoll.12-R009|Sicily|Italy|658[0n]
Melitaea celadussa|OXB1144-15|Rvcoll_15-N098||Italy|658[0n]
Melitaea celadussa|EULEP4236-16|RVcoll15G991|Genit. examined|Switzerland|658[0n]
Melitaea celadussa|EULEP635-15|RVcoll.11-J063|Genit. examined|Switzerland|658[0n]
Melitaea celadussa|OXB1146-15|Rvcoll_15-N100||Italy|658[0n]
Melitaea celadussa|BIBSA951-15|09-X863||Italy|658[0n]
Melitaea celadussa|WMB4317-14|RVcoll.14-A525||Italy|658[0n]
Melitaea celadussa|OXB977-15|15-A927||Italy|658[0n]
Melitaea celadussa|OXB1029-15|LD-0251||Italy|658[0n]
Melitaea celadussa|WMB556-11|RVcoll.11-H749|Sicily|Italy|658[0n]
Melitaea celadussa|WMB503-11|RVcoll.11-H561|Sicily|Italy|658[0n]
Melitaea celadussa|WMB2877-14|RVcoll.10-C638|Sicily|Italy|658[0n]
Melitaea celadussa|BIBSA090-14|LEP-SS-00090||Italy|658[0n]
Melitaea celadussa|BIBSA1744-16|11-I247||Italy|658[0n]
Melitaea celadussa|WMB651-11|RVcoll.11-I204||Italy|658[0n]
Melitaea celadussa|WMB2028-13|RVcoll.12-Q707||Italy|618[0n]
Melitaea celadussa|BIBSA679-15|RVcoll. 11-I207||Italy|658[0n]
Melitaea celadussa|BIBSA494-15|LEP-SS-00114||Italy|658[0n]
Melitaea celadussa|BIBSA1407-15|15-N014||Italy|658[0n]
Melitaea celadussa|BIBSA1343-15|15-M836||Italy|658[0n]
Melitaea celadussa|WMB4887-14|RVcoll.14-I434||Italy|658[0n]
Melitaea celadussa|WMB4863-14|RVcoll.14-I410||Italy|658[0n]
Melitaea celadussa|WMB4170-14|RVcoll.13-S742||Italy|658[0n]
Melitaea celadussa|WMB4158-14|RVcoll.13-S715||Italy|658[0n]
Melitaea celadussa|WMB3099-14|RVcoll.LD-2632|Ischia|Italy|658[0n]
Melitaea celadussa|WMB3098-14|RVcoll.LD-2631|Ischia|Italy|658[0n]
Melitaea celadussa|GWORU094-10|BC ZSM Lep 30355||Italy|658[0n]
Melitaea celadussa|BIBSA1842-17|RVcoll16C719||Italy|658[0n]
Melitaea celadussa|GWORA2499-09|BC ZSM Lep 30711|Changed to athalia (AS)|Germany|658[0n]
Melitaea celadussa|OXB1003-15|15-A953||Italy|658[0n]
Melitaea celadussa|OXB998-15|15-A948||Italy|658[0n]
Melitaea celadussa|OXB612-15|14-O001||Italy|658[0n]
Melitaea celadussa|WMB5109-14|RVcoll. 14-L240||Italy|658[0n]
Melitaea athalia|LEATD293-13|TLMF Lep 12940|Genit. examined|Italy|658[0n]
Melitaea celadussa|OXB1595-16|OXB-TGS-1313||France|658[0n]
Melitaea athalia|OXB844-15|14-U807|Genit. examined|Italy|658[0n]
Melitaea celadussa|OXB1273-15|Rvcoll_15-M133||France|658[0n]
Melitaea celadussa|EULEP4289-16|RVcoll15G145|Genit. examined|France|658[0n]
Melitaea celadussa|EULEP4290-16|RVcoll15G187||France|658[0n]
Melitaea celadussa|EULEP4291-16|RVcoll14V894||France|658[0n]
Melitaea celadussa|EULEP4292-16|RVcoll15G160||France|658[0n]
Melitaea celadussa|OXB1280-15|Rvcoll_15-M140||France|612[0n]
Melitaea celadussa|EULEP4259-16|RVcoll15G136||France|636[0n]
Melitaea celadussa|EULEP4258-16|RVcoll15G134||France|642[0n]
Melitaea celadussa|EULEP4257-16|RVcoll15H191||Switzerland|658[0n]
Melitaea celadussa|EULEP4256-16|RVcoll15H149||Italy|658[0n]
Melitaea celadussa|EULEP4238-16|RVcoll15H332|Genit. examined|Switzerland|658[0n]
Melitaea celadussa|BIBSA450-15|RVcoll.14-I118||Italy|658[0n]
Melitaea celadussa|OXB1121-15|15-A654||Italy|658[0n]
Melitaea celadussa|EULEP4235-16|RVcoll15G841||Italy|658[0n]
Melitaea celadussa|EULEP4234-16|RVcoll15G804||Italy|658[0n]
Melitaea celadussa|OXB1145-15|Rvcoll_15-N099||Italy|658[0n]
Melitaea celadussa|OXB1143-15|Rvcoll_15-N097||Italy|658[0n]
Melitaea celadussa|BIBSA958-15|12-R426||Italy|658[0n]
Melitaea celadussa|BIBSA990-15|15-C002||Italy|658[0n]
Melitaea celadussa|BIBSA1379-15|15-M960||Italy|658[0n]
Melitaea celadussa|WMB2286-13|RVcoll.10-C542||Italy|658[0n]
Melitaea celadussa|WMB2295-13|RVcoll.10-C670||Italy|658[0n]
Melitaea celadussa|WMB3857-14|RVcoll.11-I170||Italy|658[0n]
Melitaea celadussa|WMB3854-14|RVcoll.11-I120||Italy|658[0n]
Melitaea celadussa|OXB340-15|RVcoll.14-N054||Italy|658[0n]
Melitaea athalia|OXB725-15|13-U151|Genit. examined|Italy|658[0n]
Melitaea celadussa|BIBSA124-15|RVcoll.14-D573||Italy|658[0n]
Melitaea celadussa|EULEP4253-16|RVcoll14V142||Italy|658[0n]
Melitaea celadussa|EULEP4248-16|RVcoll15J251||Switzerland|658[0n]
Melitaea celadussa|WMB4991-14|RVcoll. 14-I538||Italy|658[0n]
Melitaea celadussa|PHLAB360-10|TLMF Lep 01160||Switzerland|658[0n]
Melitaea celadussa|PHLAB326-10|TLMF Lep 01126||Austria|658[0n]
Melitaea celadussa|PHLSA657-11|TLMF Lep 06112||Austria|658[0n]
Melitaea celadussa|WMB1727-13|RVcoll.12-P876||France|658[0n]
Melitaea athalia|OXB721-15|13-U124|Genit. examined|Italy|658[0n]
Melitaea athalia|PHLAI519-13|TLMF Lep 09081|genitalia examined|Austria|658[0n]
Melitaea celadussa|EULEP4255-16|RVcoll15G573||Switzerland|658[0n]
Melitaea celadussa|EULEP2157-15|RVcoll.14-K068||Switzerland|658[0n]
Melitaea celadussa|OXB349-15|RVcoll.14-N063||Italy|658[0n]
Melitaea celadussa|OXB359-15|RVcoll.14-N073||Italy|658[0n]
Melitaea celadussa|EULEP647-15|RVcoll.11-J148||Switzerland|658[0n]
Melitaea celadussa|EULEP4260-16|RVcoll15G464||Switzerland|634[0n]
Melitaea celadussa|EULEP4254-16|RVcoll15G530||Switzerland|636[0n]
Melitaea celadussa|EULEP4233-16|RVcoll15G608||Switzerland|658[0n]
Melitaea celadussa|WMB5033-14|RVcoll. 14-I580||Italy|658[0n]
Melitaea celadussa|PHLAH288-12|TLMF Lep 07727||Switzerland|658[0n]
Melitaea celadussa|OXB1568-16|OXB-TGS-1286||France|658[0n]
Melitaea celadussa|OXB1572-16|OXB-TGS-1290||France|631[0n]
Melitaea celadussa|WMB1850-13|RVcoll.11-I949||France|658[0n]
Melitaea celadussa|OXB1571-16|OXB-TGS-1289||France|658[0n]
Melitaea celadussa|OXB1421-15|Rvcoll_15-M147||France|658[0n]

[illegible]
Melitaea celadussa|WMB1850-13|RVcoll.11-I949||France|658[0n]
Melitaea celadussa|OXB1571-16|OXB-TGS-1289||France|658[0n]
Melitaea celadussa|OXB1421-15|Rvcoll_15-M147||France|658[0n]
Melitaea celadussa|BIBSA247-15|RVcoll.14-E043||Italy|658[0n]
Melitaea celadussa|WMB1599-13|RVcoll.12-O623||France|658[0n]
Melitaea celadussa|WMB3799-14|RVcoll.12-O624||France|658[0n]
Melitaea celadussa|WMB5156-14|RVcoll.12-P926||France|658[0n]
Melitaea celadussa|WMB1779-13|RVcoll.12-Q106||France|658[0n]
Melitaea celadussa|WMB3486-14|RVcoll.10-B642||France|658[0n]
Melitaea celadussa|WMB111-11|RVcoll.10-B644||France|658[0n]
Melitaea athalia|LEATC245-13|TLMF Lep 11372|Genit. examined|Italy|658[0n]
Melitaea celadussa|OXB845-15|14-U808||Italy|658[0n]
Melitaea celadussa|GBLAA1948-15|BC ZSM Lep 89069||Italy|658[0n]
Melitaea celadussa|EULEP4244-16|RVcoll15I495|Genit. examined|Austria|658[0n]
Melitaea celadussa|EULEP4240-16|RVcoll15H751||Switzerland|658[0n]
Melitaea celadussa|WMB5111-14|RVcoll. 14-L242||Italy|658[0n]
Melitaea celadussa|GWOSZ215-11|BC ZSM Lep 41971||Italy|658[0n]
Melitaea celadussa|PHLAH287-12|TLMF Lep 07726||Austria|658[0n]
Melitaea athalia|PHLAC444-10|TLMF Lep 02479|Genit. examined|Italy|658[0n]
Melitaea aetherie|WMB1284-13|RVcoll.12-M424|Sicily|Italy|658[0n]
Melitaea aetherie|WMB1283-13|RVcoll.12-M423|Sicily|Italy|658[0n]
Melitaea aetherie|OLEP056-15|RVcoll.14-V873||Italy|658[0n]
Melitaea aetherie|BIBSA1508-16|RVcoll.14-I262||Italy|658[0n]
Melitaea aetherie|WMB1278-13|RVcoll.12-M377|Sicily|Italy|658[0n]
Melitaea aetherie|WMB1277-13|RVcoll.12-M376|Sicily|Italy|658[0n]
Melitaea aetherie|EZSPM719-12|Rvcoll. 12-L023||Spain|658[0n]
Melitaea aetherie|EZSPM718-12|Rvcoll. 12-L022||Spain|658[0n]
Melitaea aetherie|EZSPM721-12|Rvcoll. 12-L048||Spain|658[0n]
Melitaea aetherie|EZSPM720-12|Rvcoll. 12-L024||Spain|658[0n]
Melitaea aetherie|EZSPN452-09|RVcoll.08-H654||Spain|658[0n]
Melitaea aetherie|EZSPN451-09|RVcoll.08-H653||Spain|658[0n]
Melitaea ornata|EULEP2971-15|RVcoll.14-O104|Chios|Greece|658[0n]
Melitaea ornata|EULEP2970-15|RVcoll.14-O103||Greece|658[0n]
Melitaea ornata|EULEP1747-15|RVcoll.14-H378||Greece|658[0n]
Melitaea ornata|EULEP1711-15|RVcoll.14-H164||Greece|658[0n]
Melitaea ornata|EULEP1530-15|RVcoll.14-G482||Greece|658[0n]
Melitaea ornata|EULEP499-15|RVcoll.07-C512||Greece|658[0n]
Melitaea ornata|EULEP1832-15|RVcoll.14-J953||Bulgaria|658[0n]
Melitaea ornata|EULEP1701-15|RVcoll.14-H119||Greece|614[0n]
Melitaea ornata|EULEP1745-15|RVcoll.14-H355||Greece|658[0n]
Melitaea ornata|EULEP1253-15|RVcoll.14-F623||Greece|658[0n]
Melitaea ornata|EULEP2551-15|RVcoll.14-V470||Greece|658[0n]
Melitaea ornata|EULEP1812-15|RVcoll.14-H790||Greece|658[0n]
Melitaea ornata|EULEP1796-15|RVcoll.14-H669||Greece|658[0n]
Melitaea ornata|EULEP1786-15|RVcoll.14-H630||Greece|658[0n]
Melitaea ornata|EULEP2974-15|RVcoll.14-O107||Albania|658[0n]
Melitaea ornata|EULEP2968-15|RVcoll.14-O101||Greece|658[0n]
Melitaea ornata|EULEP1761-15|RVcoll.14-H428||Greece|658[0n]
Melitaea ornata|EULEP1289-15|RVcoll.14-F712||Greece|658[0n]
Melitaea ornata|EULEP5914-18|RVcoll17G011||Albania|658[0n]
Melitaea ornata|EULEP4286-16|RVcoll14O321||Macedonia|658[0n]
Melitaea|WMB4511-14|RVcoll.14-E201|phoebe/ornata|Spain|658[0n]
Melitaea|EZSPM623-12|RVcoll.11-D910|phoebe/ornata|Spain|658[0n]
Melitaea|EZSPN084-09|RVcoll.06-A003|phoebe/ornata|Spain|658[0n]
Melitaea|EZSPM895-12|Rvcoll.12-M617|phoebe/ornata|Spain|658[0n]
Melitaea|EZSPM631-12|RVcoll.11-E050|phoebe/ornata|Spain|658[0n]
Melitaea|EZSPN404-09|RVcoll.08-H479|phoebe/ornata|Spain|658[0n]
Melitaea|EZSPC554-09|RVcoll.07-C628|phoebe/ornata|Spain|658[0n]
Melitaea phoebe|EULEP5845-18|RVcoll14U561||Albania|658[0n]
Melitaea phoebe|EULEP5843-18|RVcoll14U557||Albania|658[0n]
Melitaea phoebe|EULEP5841-18|RVcoll14U555||Albania|658[0n]
Melitaea phoebe|EULEP1577-15|RVcoll.14-G602||Macedonia|658[0n]
Melitaea ornata|EULEP2972-15|RVcoll.14-O105|red larva head|Slovenia|658[0n]
Melitaea|OXB703-15|13-T879|phoebe/ornata|Italy|658[0n]
Melitaea|WMB4251-14|RVcoll.13-U066|phoebe/ornata|Italy|622[0n]
Melitaea phoebe|EZRMN117-08|RVcoll.08-M371||Romania|658[0n]
Melitaea phoebe|EZROM1047-09|RVcoll.07-D182||Romania|658[0n]
Melitaea phoebe|EZRMN121-08|RVcoll.08-M572||Romania|658[0n]
Melitaea phoebe|EZRMN119-08|RVcoll.08-M418||Romania|658[0n]
Melitaea phoebe|EZRMN116-08|RVcoll.08-M224||Romania|658[0n]
Melitaea phoebe|EZROM640-08|RV-07-D123||Romania|658[0n]
Melitaea phoebe|EZROM422-08|RV-07-D116||Romania|658[0n]
Melitaea phoebe|EZROM421-08|RV-07-D111||Romania|658[0n]
Melitaea phoebe|EZROM419-08|RV-07-D004||Romania|658[0n]
Melitaea phoebe|EZRMN120-08|RVcoll.08-M551||Romania|658[0n]
Melitaea phoebe|EZROM1058-09|RVcoll.07-E393||Romania|658[0n]
Melitaea phoebe|EULEP1173-15|RVcoll.14-F394||Bulgaria|658[0n]
Melitaea phoebe|EULEP1128-15|RVcoll.14-F247||Serbia|658[0n]
Melitaea phoebe|EULEP5917-18|RVcoll17G016||Albania|658[0n]
Melitaea phoebe|EULEP5915-18|RVcoll17G014||Albania|658[0n]
Melitaea phoebe|EULEP5913-18|RVcoll17G010||Albania|658[0n]
Melitaea phoebe|EULEP5912-18|RVcoll17G009||Albania|658[0n]
Melitaea phoebe|EULEP5911-18|RVcoll17G008||Albania|658[0n]
Melitaea phoebe|EULEP5910-18|RVcoll17G002||Albania|658[0n]
Melitaea phoebe|EULEP5909-18|RVcoll17G001||Albania|658[0n]
Melitaea phoebe|EULEP5847-18|RVcoll14U563||Albania|658[0n]
Melitaea phoebe|EULEP5846-18|RVcoll14U562||Albania|658[0n]
Melitaea phoebe|EULEP5844-18|RVcoll14U560||Albania|658[0n]
Melitaea phoebe|EULEP5842-18|RVcoll14U556||Albania|658[0n]
Melitaea phoebe|EULEP5835-18|RVcoll14U541||Albania|658[0n]
Melitaea phoebe|EULEP4886-16|RVcoll16J564||Moldova|658[0n]
Melitaea|EULEP5029-16|RVcoll16I968|phoebe/ornata|Slovakia|658[0n]
Melitaea phoebe|EULEP4293-16|RVcoll10B308||Bulgaria|658[0n]
Melitaea ornata|EULEP4288-16|RVcoll15O027|Sicily|Italy|658[0n]
Melitaea phoebe|EULEP4285-16|RVcoll14O307||Albania|658[0n]
Melitaea|WMB5229-14|RVcoll.13-T991|phoebe/ornata|Italy|658[0n]
Melitaea ornata|WMB603-11|RVcoll.11-I002|Sicily|Italy|658[0n]
Melitaea ornata|WMB595-11|RVcoll.11-H939|Sicily|Italy|658[0n]
Melitaea|WMB4168-14|RVcoll.13-S730|phoebe/ornata|Italy|658[0n]
Melitaea phoebe|WMB3526-14|RVcoll.10-C074||France|658[0n]
Melitaea ornata|WMB2778-13|RVcoll.11-I046|Sicily|Italy|658[0n]
Melitaea ornata|WMB2948-14|RVcoll.12-M438|Sicily|Italy|658[0n]
[illegible]

Melitaea phoebe|WMB3526-14|RVcoll.10-C074||France|658[0n]
Melitaea ornata|WMB2778-13|RVcoll.11-I046|Sicily|Italy|658[0n]
Melitaea ornata|WMB2948-14|RVcoll.12-M438|Sicily|Italy|658[0n]
Melitaea ornata|WMB2947-14|RVcoll.12-M433|Sicily|Italy|658[0n]
Melitaea|EZSPN502-09|RVcoll.08-H956|phoebe/ornata|Spain|658[0n]
Melitaea|EZSPN487-09|RVcoll.08-H924|phoebe/ornata|Spain|658[0n]
Melitaea|EZSPN328-09|RVcoll.08-H236|phoebe/ornata|Spain|658[0n]
Melitaea|EZSPN695-09|RVcoll.08-L027|phoebe/ornata|Spain|658[0n]
Melitaea phoebe|EZRMN118-08|RVcoll.08-M406||Romania|658[0n]
Melitaea phoebe|EZRMN115-08|RVcoll.08-M214||Romania|658[0n]
Melitaea phoebe|EZROM1050-09|RVcoll.07-D325||Romania|658[0n]
Melitaea phoebe|EZROM1051-09|RVcoll.07-D477||Romania|658[0n]
Melitaea phoebe|EZROM641-08|RV-07-D316||Romania|658[0n]
Melitaea phoebe|EZROM425-08|RV-07-E422||Romania|658[0n]
Melitaea phoebe|EZROM424-08|RV-06-V689||Romania|658[0n]
Melitaea phoebe|EZROM423-08|RV-07-C127||Romania|658[0n]
Melitaea phoebe|EZROM420-08|RV-07-D048||Romania|658[0n]
Melitaea phoebe|EZROM589-08|RV-07-C300||Romania|658[0n]
Melitaea phoebe|PHLAI539-13|TLMF Lep 09101||Austria|658[0n]
Melitaea|PHLAI480-13|TLMF Lep 09042|Melitaea phoebe/ornata|Italy|658[0n]
Melitaea|ABOLD024-16|TLMF Lep 21102|Melitaea phoebe/ornata|Austria|658[0n]
Melitaea phoebe|EULEP1595-15|RVcoll.14-G640||Greece|658[0n]
Melitaea phoebe|EULEP632-15|RVcoll.11-J032||Switzerland|658[0n]
Melitaea phoebe|EULEP5830-18|RVcoll14U529||Albania|658[0n]
Melitaea phoebe|EULEP4896-16|RVcoll16J599||Russia|658[0n]
Melitaea phoebe|EULEP2082-15|RVcoll.14-J570||Ukraine|658[0n]
Melitaea phoebe|EULEP1520-15|RVcoll.14-G446||Greece|658[0n]
Melitaea phoebe|EULEP5832-18|RVcoll14U531||Albania|658[0n]
Melitaea phoebe|EULEP5831-18|RVcoll14U530||Albania|658[0n]
Melitaea phoebe|EULEP5834-18|RVcoll14U540||Albania|658[0n]
Melitaea phoebe|EULEP5833-18|RVcoll14U532||Albania|658[0n]
Melitaea phoebe|EULEP2462-15|RVcoll.14-V287||Switzerland|658[0n]
Melitaea phoebe|EULEP2494-15|RVcoll.14-V351||Ukraine|658[0n]
Melitaea phoebe|EULEP1894-15|RVcoll.14-B720||Albania|658[0n]
Melitaea|EULEP1916-15|RVcoll.14-B829|phoebe/ornata|Bosnia and Herzegovina|658[0n]
Melitaea ornata|EULEP2967-15|RVcoll.14-O100|Sicily|Italy|658[0n]
Melitaea ornata|EULEP2969-15|RVcoll.14-O102|red larva head|Italy|658[0n]
Melitaea ornata|BIBSA1770-16|LD-1507||Italy|658[0n]
Melitaea ornata|BIBSA720-15|LD-1501||Italy|658[0n]
Melitaea|BIBSA954-15|15-C288|phoebe/ornata|Italy|658[0n]
Melitaea|LEASS1009-17|TLMF Lep 22562|Melitaea phoebe/ornata|Austria|658[0n]
Melitaea phoebe|LEFIJ6627-17|MM26009||Bulgaria|658[0n]
Melitaea|BIBSA599-15|LEP-SS-00219|phoebe/ornata|Italy|647[0n]
Melitaea|BIBSA086-14|LEP-SS-00086|phoebe/ornata|Italy|658[0n]
Melitaea|WMB1972-13|RVcoll.11-I201|phoebe/ornata|Italy|658[0n]
Melitaea phoebe|EULEP344-14|MM23829||Lithuania|658[0n]
Melitaea phoebe|EULEP591-15|RVcoll.10-A732||Estonia|658[0n]
Melitaea phoebe|LEEUA531-11|MM20590||Latvia|609[0n]
Melitaea phoebe|LEFIJ1426-12|MM22093||Latvia|622[0n]
Melitaea ornata|WMB1957-13|RVcoll.11-I071|Sicily|Italy|645[0n]
Melitaea|EULEP2966-15|RVcoll.14-O099|phoebe/ornata|France|613[0n]
Melitaea|WMB3291-14|RVcoll.08-M950|phoebe/ornata|Spain|620[0n]
Melitaea phoebe|EULEP4295-16|RVcoll15G462||Switzerland|637[0n]
Melitaea phoebe|EULEP535-15|RVcoll.08-H877||Serbia|613[0n]
Melitaea|EZSPC553-09|RVcoll.08-L412|phoebe/ornata|Spain|634[0n]
Melitaea|PHLAI478-13|TLMF Lep 09040|Melitaea phoebe/ornata|Austria|647[0n]
Melitaea|WMB3275-14|RVcoll.08-M013|phoebe/ornata|Spain|658[0n]
Melitaea|WMB3328-14|RVcoll.08-P757|phoebe/ornata|Spain|658[0n]
Melitaea|EZSPM263-09|RVcoll.08-R478|phoebe/ornata|Spain|658[0n]
Melitaea phoebe|PHLAI481-13|TLMF Lep 09043||Austria|658[0n]
Melitaea|WMB253-11|RVcoll.09-V279|phoebe/ornata|France|658[0n]
Melitaea|BIBSA1398-15|15-N002|phoebe/ornata|Italy|658[0n]
Melitaea|WMB4304-14|RVcoll.14-A403|phoebe/ornata|Italy|658[0n]
Melitaea|WMB2463-13|RVcoll.11-H350|phoebe/ornata|Italy|658[0n]
Melitaea|WMB4155-14|RVcoll.13-S712|phoebe/ornata|Italy|658[0n]
Melitaea|WMB4246-14|RVcoll.13-U029|phoebe/ornata|Italy|658[0n]
Melitaea|WMB3877-14|RVcoll.11-Y110|phoebe/ornata|Italy|658[0n]
Melitaea|WMB3961-14|RVcoll.12-P791|phoebe/ornata|France|658[0n]
Melitaea|WMB4830-14|RVcoll. 14-I377|phoebe/ornata|Italy|658[0n]
Melitaea|WMB4867-14|RVcoll.14-I414|phoebe/ornata|Italy|658[0n]
Melitaea|WMB4892-14|RVcoll.14-I439|phoebe/ornata|Italy|658[0n]
Melitaea|WMB4979-14|RVcoll. 14-I526|phoebe/ornata|Italy|658[0n]
Melitaea|WMB5041-14|RVcoll. 14-L172|phoebe/ornata|Italy|658[0n]
Melitaea|EULEP4294-16|RVcoll15J546|phoebe/ornata|France|658[0n]
Melitaea|BIBSA1207-15|15-M322|phoebe/ornata|Italy|658[0n]
Melitaea phoebe|EULEP2973-15|RVcoll.14-O106|black larva head|Croatia|658[0n]
Melitaea|BIBSA681-15|RVcoll. 12-M510|phoebe/ornata|Italy|658[0n]
Melitaea|BIBSA717-15|RVcoll. 14-A780|phoebe/ornata|Italy|658[0n]
Melitaea|BIBSA1715-16|15-N187|phoebe/ornata|Italy|658[0n]
Melitaea|BIBSA459-15|RVcoll.14-I127|phoebe/ornata|Italy|658[0n]
Melitaea|BIBSA1007-15|15-C109|phoebe/ornata|Italy|658[0n]
Melitaea|OXB781-15|14-A750|phoebe/ornata|Italy|658[0n]
Melitaea|OXB1116-15|15-A649|phoebe/ornata|Italy|658[0n]
Melitaea|OXB891-15|15-A533|phoebe/ornata|Italy|658[0n]
Melitaea|OXB1008-15|15-A958|phoebe/ornata|Italy|658[0n]
Melitaea|OXB1573-16|OXB-TGS-1291|phoebe/ornata|France|658[0n]
Melitaea|OXB1587-16|OXB-TGS-1305|phoebe/ornata|France|658[0n]
Melitaea|BIBSA248-15|RVcoll.14-E044|phoebe/ornata|Italy|633[0n]
Melitaea|BIBSA246-15|RVcoll.14-E041|phoebe/ornata|Italy|658[0n]
Melitaea|BIBSA1566-16|LEP-SS-00386|phoebe/ornata|Italy|614[0n]
Melitaea|WMB2213-13|RVcoll.11-Y026|phoebe/ornata|Italy|632[0n]
Melitaea|WMB3850-14|RVcoll.10-C738|phoebe/ornata|Italy|614[0n]
Melitaea|WMB1712-13|RVcoll.12-P661|phoebe/ornata|France|658[0n]
Melitaea|WMB1602-13|RVcoll.12-O658|phoebe/ornata|France|658[0n]
Melitaea|WMB893-13|RVcoll.09-X830|phoebe/ornata|Italy|658[0n]
Melitaea|WMB1235-13|RVcoll.11-H370|phoebe/ornata|Italy|658[0n]
Melitaea|WMB1234-13|RVcoll.11-H366|phoebe/ornata|Italy|658[0n]
Melitaea|WMB3656-14|RVcoll.11-I765|phoebe/ornata|France|658[0n]
Melitaea|WMB3463-14|RVcoll.10-A609|phoebe/ornata|France|658[0n]
Melitaea|WMB3038-14|RVcoll.13-S565|San Domino. phoebe/ornata|Italy|658[0n]
Melitaea|EZROM738-08|RV-06-H893|phoebe/ornata|Spain|658[0n]
Melitaea|EZSPC555-09|RVcoll.08-L464|phoebe/ornata|Spain|658[0n]
Melitaea|EZSPC556-09|RVcoll.08-M684|phoebe/ornata|Spain|658[0n]

Melitaea|EZROM738-08|RV-06-H893|phoebe/ornata|Spain|658[0n]
Melitaea|EZSPC555-09|RVcoll.08-L464|phoebe/ornata|Spain|658[0n]
Melitaea|EZSPC556-09|RVcoll.08-M684|phoebe/ornata|Spain|658[0n]
Melitaea|PHLAI479-13|TLMF Lep 09041|Melitaea phoebe/ornata|Italy|658[0n]
Melitaea didyma|EZSPC1226-10|RVcoll.09-V584||Spain|658[0n]
Melitaea didyma|EZSPM604-12|RVcoll.11-D602||Spain|658[0n]
Melitaea didyma|EZSPC1225-10|RVcoll.09-V583||Spain|658[0n]
Melitaea didyma|EZSPC1224-10|RVcoll.09-V572||Spain|658[0n]
Melitaea didyma|EZSPM605-12|RVcoll.11-D609||Spain|658[0n]
Melitaea didyma|EZSPN680-09|RVcoll.08-J993||Spain|658[0n]
Melitaea didyma|WMB3986-14|RVcoll.12-Q313||France|658[0n]
Melitaea didyma|WMB720-12|RVcoll.11-I119||Italy|658[0n]
Melitaea didyma|WMB2285-13|RVcoll.10-C541||Italy|658[0n]
Melitaea didyma|WMB2363-13|RVcoll.12-Q922||Italy|658[0n]
Melitaea didyma|BIBSA1560-16|LEP-SS-00384||Italy|658[0n]
Melitaea didyma|BIBSA1563-16|LEP-SS-00385||Italy|614[0n]
Melitaea didyma|BIBSA092-14|LEP-SS-00092||Italy|658[0n]
Melitaea didyma|OXB780-15|14-A748||Italy|658[0n]
Melitaea didyma|WMB2643-13|RVcoll.12-R429||Italy|658[0n]
Melitaea didyma|BIBSA993-15|15-C019||Italy|658[0n]
Melitaea didyma|BIBSA675-15|RVcoll. 11-I203||Italy|658[0n]
Melitaea didyma|BIBSA1042-15|15-C220||Italy|658[0n]
Melitaea didyma|WMB4900-14|RVcoll.14-I447||Italy|658[0n]
Melitaea didyma|WMB4140-14|RVcoll.13-S653||Italy|658[0n]
Melitaea didyma|GWORU091-10|BC ZSM Lep 30352||Italy|658[0n]
Melitaea didyma|GWORO995-09|BC ZSM Lep 32302||Italy|658[0n]
Melitaea didyma|EULEP5863-18|RVcoll15L572||Italy|658[0n]
Melitaea didyma|BIBSA1807-17|RVcoll14I276||Italy|658[0n]
Melitaea didyma|OXB371-15|RVcoll.14-N085||Italy|658[0n]
Melitaea didyma|OXB1138-15|15-A671||Italy|658[0n]
Melitaea didyma|OXB1124-15|15-A657||Italy|658[0n]
Melitaea didyma|OXB1114-15|15-A647||Italy|658[0n]
Melitaea didyma|OXB579-15|14-N968||Italy|658[0n]
Melitaea didyma|OXB843-15|14-U806||Italy|658[0n]
Melitaea didyma|BIBSA1231-15|15-N038||Italy|658[0n]
Melitaea didyma|EULEP5857-18|RVcoll14W669||Italy|658[0n]
Melitaea didyma|WMB3890-14|RVcoll.12-O903||Italy|658[0n]
Melitaea didyma|EULEP748-15|RVcoll.12-O756||France|658[0n]
Melitaea didyma|LEASS1011-17|TLMF Lep 22564||Austria|658[0n]
Melitaea didyma|EULEP5865-18|RVcoll15M518||Italy|658[0n]
Melitaea didyma|EULEP5858-18|RVcoll14W858||Italy|658[0n]
Melitaea didyma|EULEP5823-18|RVcoll12P738||France|658[0n]
Melitaea didyma|EULEP5819-18|RVcoll12O695||France|658[0n]
Melitaea didyma|EULEP5800-18|RVcoll10A578||France|658[0n]
Melitaea didyma|WMB4960-14|RVcoll. 14-I507||Italy|658[0n]
Melitaea didyma|WMB730-12|RVcoll.11-I689||France|658[0n]
Melitaea didyma|WMB3962-14|RVcoll.12-P796||France|658[0n]
Melitaea didyma|WMB112-11|RVcoll.10-B647||France|658[0n]
Melitaea didyma|WMB2227-13|RVcoll.11-Y064||Italy|658[0n]
Melitaea didyma|WMB4166-14|RVcoll.13-S727||Italy|658[0n]
Melitaea didyma|BIBSA1752-16|12-Q783||Italy|658[0n]
Melitaea didyma|EULEP5610-17|RVcoll14V137||Italy|658[0n]
Melitaea didyma|BCLEP283-17|LEP-SS-00709||Italy|658[0n]
Melitaea didyma|WMB5230-14|RVcoll.13-T997||Italy|658[0n]
Melitaea didyma|BIBSA1383-15|15-M979||Italy|658[0n]
Melitaea didyma|WMB4873-14|RVcoll.14-I420||Italy|658[0n]
Melitaea didyma|WMB4680-14|RVcoll.LD-2663||Italy|658[0n]
Melitaea didyma|WMB3836-14|RVcoll.09-X918||Italy|658[0n]
Melitaea didyma|EULEP5663-17|RVcoll15M430||Italy|658[0n]
Melitaea didyma|WMB5163-14|RVcoll.12-R443||Italy|658[0n]
Melitaea didyma|EULEP5438-17|RVcoll11H277||Italy|658[0n]
Melitaea didyma|EULEP5714-17|RVcoll16L069||Italy|658[0n]
Melitaea didyma|BIBSA1158-15|15-L123||Italy|658[0n]
Melitaea didyma|OXB1080-15|15-A613||Italy|658[0n]
Melitaea didyma|OXB1089-15|15-A622||Italy|658[0n]
Melitaea didyma|BIBSA1509-16|RVcoll.14-I263||Italy|646[0n]
Melitaea didyma|WMB739-12|RVcoll.11-J622||Italy|616[0n]
Melitaea didyma|WMB2599-13|RVcoll.12-R399|Elba|Italy|658[0n]
Melitaea didyma|WMB2184-13|RVcoll.12-R360|Elba|Italy|658[0n]
Melitaea didyma|WMB2183-13|RVcoll.12-R359|Elba|Italy|658[0n]
Melitaea didyma|WMB3873-14|RVcoll.11-Y078||Italy|658[0n]
Melitaea didyma|WMB5099-14|RVcoll. 14-L230||Italy|658[0n]
Melitaea didyma|OXB879-15|15-A521||Italy|658[0n]
Melitaea didyma|EULEP5717-17|RVcoll16L073||Italy|658[0n]
Melitaea didyma|EULEP5826-18|RVcoll14A356||Italy|658[0n]
Melitaea didyma|WMB5243-14|RVcoll.13-U095||Italy|658[0n]
Melitaea didyma|WMB4910-14|RVcoll.14-I457||Italy|658[0n]
Melitaea didyma|WMB4858-14|RVcoll.14-I405||Italy|658[0n]
Melitaea didyma|WMB3849-14|RVcoll.10-C737||Italy|658[0n]
Melitaea didyma|WMB2965-14|RVcoll.12-O043|Argentario|Italy|658[0n]
Melitaea didyma|BIBSA1866-17|RVcoll16C759||Italy|658[0n]
Melitaea didyma|BIBSA1865-17|RVcoll16C758||Italy|658[0n]
Melitaea didyma|BIBSA1341-15|15-M834||Italy|658[0n]
Melitaea didyma|WMB722-12|RVcoll.11-I202||Italy|658[0n]
Melitaea didyma|EULEP4282-16|RVcoll15I122||Italy|658[0n]
Melitaea didyma|LEASS887-17|KLM Lep 08392||Austria|658[0n]
Melitaea didyma|LEASS684-17|TLMF Lep 22332||Austria|658[0n]
Melitaea didyma|OXB741-15|13-U302||Italy|658[0n]
Melitaea didyma|BIBSA091-14|LEP-SS-00091||Italy|632[0n]
Melitaea didyma|EULEP4281-16|RVcoll15I088||Italy|658[0n]
Melitaea didyma|GWORO994-09|BC ZSM Lep 32301||Italy|658[0n]
Melitaea didyma|BIBSA1589-16|12-M585||Italy|658[0n]
Melitaea didyma|WMB546-11|RVcoll.11-H734|Sicily|Italy|658[0n]
Melitaea didyma|WMB555-11|RVcoll.11-H748|Sicily|Italy|658[0n]
Melitaea didyma|WMB2916-14|RVcoll.11-H995|Sicily|Italy|658[0n]
Melitaea didyma|WMB627-11|RVcoll.11-I076|Sicily|Italy|658[0n]
Melitaea didyma|BIBSA1575-16|11-I104||Italy|658[0n]
Melitaea didyma|BIBSA1559-16|11-H586||Italy|658[0n]
Melitaea didyma|WMB507-11|RVcoll.11-H582|Sicily|Italy|658[0n]
Melitaea didyma|WMB2913-14|RVcoll.11-H583|Sicily|Italy|658[0n]
Melitaea didyma|WMB220-11|RVcoll.08-R519|Sicily|Italy|651[0n]
Melitaea didyma|EZSPC1048-10|RVcoll.08-J737||Spain|658[0n]
[illegible]

Melitaea didyma|WMB2913-14|RVcoll.11-H583|Sicily|Italy|658[0n]
Melitaea didyma|WMB220-11|RVcoll.08-R519|Sicily|Italy|651[0n]
Melitaea didyma|EZSPC1048-10|RVcoll.08-J737||Spain|658[0n]
Melitaea didyma|EZSPC1046-10|RVcoll.08-J709||Spain|658[0n]
Melitaea didyma|EZSPC1067-10|RVcoll.08-L204||Spain|658[0n]
Melitaea didyma|EZSPC990-10|RVcoll.07-F075||Spain|658[0n]
Melitaea didyma|EULEP5798-18|RVcoll08J742||Spain|658[0n]
Melitaea didyma|EZSPM355-09|RVcoll.08-R068||Spain|658[0n]
Melitaea didyma|EZSPC1092-10|RVcoll.08-L915.1||Spain|658[0n]
Melitaea didyma|EZSPC1091-10|RVcoll.08-L914||Spain|642[0n]
Melitaea didyma|EZSPC1082-10|RVcoll.08-L728||Spain|658[0n]
Melitaea didyma|EZSPC1017-10|RVcoll.08-H692||Spain|658[0n]
Melitaea didyma|EZSPN757-09|RVcoll.08-L234||Spain|658[0n]
Melitaea didyma|EULEP5881-18|RVcoll15O556||Spain|658[0n]
Melitaea didyma|EULEP5880-18|RVcoll15O555||Spain|658[0n]
Melitaea didyma|EZSPN908-09|RVcoll.08-L832||Spain|658[0n]
Melitaea didyma|WMB731-12|RVcoll.11-I690||France|658[0n]
Melitaea didyma|EULEP5860-18|RVcoll15J468||France|658[0n]
Melitaea didyma|EULEP5820-18|RVcoll12O852||France|658[0n]
Melitaea didyma|EULEP5806-18|RVcoll11I691||France|658[0n]
Melitaea didyma|EULEP5801-18|RVcoll10B993||France|658[0n]
Melitaea didyma|EULEP4283-16|RVcoll15J455||France|658[0n]
Melitaea didyma|WMB3522-14|RVcoll.10-B979||France|658[0n]
Melitaea didyma|EZSPC1099-10|RVcoll.08-L951||Spain|658[0n]
Melitaea didyma|EZSPN900-09|RVcoll.08-L812|Genit. examined|Spain|658[0n]
Melitaea didyma|EZSPC552-09|RVcoll.08-P048||Spain|658[0n]
Melitaea didyma|EZSPC549-09|RVcoll.06-H891||Spain|658[0n]
Melitaea didyma|EZROM737-08|RV-06-G478||Spain|658[0n]
Melitaea didyma|EZSPC550-09|RVcoll.08-L440||Spain|658[0n]
Melitaea didyma|EULEP1216-15|RVcoll.14-F516||Greece|658[0n]
Melitaea didyma|EULEP5828-18|RVcoll14F502||Greece|658[0n]
Melitaea didyma|LEASS758-17|TLMF Lep 22406||Austria|658[0n]
Melitaea didyma|EULEP1138-15|RVcoll.14-E683||Romania|658[0n]
Melitaea didyma|EULEP143-14|RVcoll.10-C421||Romania|658[0n]
Melitaea didyma|LEASS757-17|TLMF Lep 22405||Austria|658[0n]
Melitaea didyma|EZRMN113-08|RVcoll.08-M265||Romania|658[0n]
Melitaea didyma|EULEP2528-15|RVcoll.14-V402||Ukraine|658[0n]
Melitaea didyma|EULEP2513-15|RVcoll.14-V382||Ukraine|658[0n]
Melitaea didyma|EULEP531-15|RVcoll.14-F524||Greece|658[0n]
Melitaea didyma|EULEP1905-15|RVcoll.14-B797||Albania|658[0n]
Melitaea didyma|EULEP1370-15|RVcoll.14-F960||Greece|658[0n]
Melitaea didyma|EULEP187-14|RVcoll.11-J885||Romania|658[0n]
Melitaea didyma|EZRMN114-08|RVcoll.08-M300||Romania|658[0n]
Melitaea didyma|EZROM418-08|RV-06-M995||Romania|658[0n]
Melitaea didyma|EZROM417-08|RV-06-M973||Romania|658[0n]
Melitaea didyma|EZROM416-08|RV-06-M962||Romania|658[0n]
Melitaea didyma|EULEP1184-15|RVcoll.14-F449||Bulgaria|658[0n]
Melitaea didyma|EULEP1264-15|RVcoll.14-F656||Greece|658[0n]
Melitaea didyma|EULEP1453-15|RVcoll.14-G179||Greece|658[0n]
Melitaea didyma|EULEP546-15|RVcoll.14-G522||Greece|658[0n]
Melitaea didyma|EULEP931-15|RVcoll.14-C867||Bulgaria|658[0n]
Melitaea didyma|EULEP930-15|RVcoll.14-C850||Bulgaria|658[0n]
Melitaea didyma|EULEP1057-15|RVcoll.14-E947||Serbia|658[0n]
Melitaea didyma|EULEP4849-16|RVcoll16H772||Bulgaria|658[0n]
Melitaea didyma|EULEP1432-15|RVcoll.14-G115||Greece|658[0n]
Melitaea didyma|EULEP1787-15|RVcoll.14-H637||Greece|658[0n]
Melitaea didyma|EULEP498-15|RVcoll.07-C504||Greece|618[0n]
Melitaea didyma|EULEP1722-15|RVcoll.14-H222||Greece|658[0n]
Melitaea didyma|LEASS767-17|TLMF Lep 22415||Austria|658[0n]
Melitaea didyma|EULEP2198-15|RVcoll.14-N358||Ukraine|658[0n]
Melitaea didyma|EULEP2390-15|RVcoll.14-V093||Ukraine|658[0n]
Melitaea didyma|EULEP1593-15|RVcoll.14-G632||Greece|658[0n]
Melitaea didyma|EULEP533-15|RVcoll.14-E985||Serbia|658[0n]
Melitaea didyma|EULEP5827-18|RVcoll14E926||Serbia|658[0n]
Melitaea didyma|ABOLD071-16|TLMF Lep 21149||Austria|658[0n]
Melitaea didyma|BIBSA956-15|12-R197||Italy|658[0n]
Melitaea didyma|WMB2724-13|RVcoll.10-C562||Italy|658[0n]
Melitaea didyma|WMB1804-13|RVcoll.12-Q342||France|658[0n]
Melitaea didyma|WMB3692-14|RVcoll.11-J247||Spain|658[0n]
Melitaea didyma|EULEP5799-18|RVcoll10A449||France|658[0n]
Melitaea didyma|EULEP5807-18|RVcoll11I766||France|658[0n]
Melitaea didyma|EULEP5808-18|RVcoll11J199||France|658[0n]
Melitaea didyma|EULEP5822-18|RVcoll12P518||France|658[0n]
Melitaea didyma|EZSPC1374-10|RVcoll.09-X232||France|658[0n]
Melitaea didyma|WMB3944-14|RVcoll.12-P697||France|614[0n]
Melitaea didyma|WMB3938-14|RVcoll.12-P593||France|658[0n]
Melitaea didyma|WMB3918-14|RVcoll.12-P416||France|658[0n]
Melitaea didyma|EULEP5825-18|RVcoll12Q108||France|658[0n]
Melitaea didyma|EULEP5824-18|RVcoll12Q067||France|658[0n]
Melitaea didyma|WMB1763-13|RVcoll.12-Q005|Levant|France|658[0n]
Melitaea didyma|WMB1762-13|RVcoll.12-Q004|Levant|France|658[0n]
Melitaea didyma|EULEP1979-15|RVcoll.14-H910||France|658[0n]
Melitaea didyma|EULEP5821-18|RVcoll12O967||France|658[0n]
Melitaea didyma|EZSPC1047-10|RVcoll.08-J735||Spain|658[0n]
Melitaea didyma|OXB325-15|RVcoll.14-N039||Italy|658[0n]
Melitaea didyma|EULEP5864-18|RVcoll15M034||Italy|658[0n]
Melitaea didyma|EULEP5862-18|RVcoll15L153||Italy|658[0n]
Melitaea didyma|EULEP5859-18|RVcoll15H077||Italy|658[0n]
Melitaea didyma|EULEP646-15|RVcoll.11-J146||Switzerland|658[0n]
Melitaea didyma|EULEP4284-16|RVcoll15G479||Switzerland|658[0n]
Melitaea didyma|EULEP4280-16|RVcoll15H301||Switzerland|658[0n]
Melitaea didyma|EULEP5856-18|RVcoll14V970||Spain|658[0n]
Melitaea didyma|EZSPC1149-10|RVcoll.08-P929||Spain|658[0n]
Melitaea didyma|EZSPC975-10|RVcoll.06-G491||Spain|658[0n]
Melitaea didyma|EZSPC974-10|RVcoll.06-G485||Spain|658[0n]
Melitaea didyma|EZSPN1026-09|RVcoll.08-M960||Spain|658[0n]
Melitaea didyma|EZSPM797-12|Rvcoll. 090111XZ00||Spain|658[0n]
Melitaea didyma|EZSPC551-09|RVcoll.08-P370||Spain|658[0n]
Melitaea didyma|EZSPN539-09|RVcoll.08-J030||Spain|658[0n]
Melitaea didyma|LEATG045-14|TLMF Lep 13832||Austria|658[0n]
Melitaea didyma|LEATG046-14|TLMF Lep 13833||Austria|658[0n]
Melitaea didyma|OXB1364-15|Rvcoll_15-M720||France|658[0n]
Melitaea didyma|OXB1355-15|Rvcoll_15-M707||France|658[0n]

Melitaea didyma|LEATG046-14|TLMF Lep 13833||Austria|658[0n]
Melitaea didyma|OXB1364-15|Rvcoll_15-M720||France|658[0n]
Melitaea didyma|OXB1355-15|Rvcoll_15-M707||France|658[0n]
Melitaea didyma|GWORK312-09|BC ZSM Lep 21547||Germany|658[0n]
Melitaea didyma|BIBSA308-15|RVcoll.14-E110||Italy|658[0n]
Melitaea didyma|PHLAH471-12|TLMF Lep 08290||Austria|615[0n]
Melitaea didyma|BIBSA245-15|RVcoll.14-E039||Italy|630[0n]
Melitaea didyma|LASTS423-14|TLMF Lep 14875||Italy|643[0n]
Borbo borbonica|EULEP229-14|RVcoll.12-L081||Spain|658[0n]
Borbo borbonica|EULEP223-14|RVcoll.12-L075||Spain|658[0n]
Borbo borbonica|EULEP228-14|RVcoll.12-L080||Spain|658[0n]
Borbo borbonica|EULEP227-14|RVcoll.12-L079||Spain|658[0n]
Borbo borbonica|EULEP226-14|RVcoll.12-L078||Spain|658[0n]
Borbo borbonica|EULEP225-14|RVcoll.12-L077||Spain|658[0n]
Borbo borbonica|EULEP224-14|RVcoll.12-L076||Spain|658[0n]
Borbo borbonica|EULEP222-14|RVcoll.12-L074||Spain|658[0n]
Borbo borbonica|EULEP031-14|RVcoll.07-D799||Spain|658[0n]
Borbo borbonica|EULEP025-14|RVcoll.07-D710||Spain|658[0n]
Gegenes nostrodamus|EULEP4041-16|RVcoll14E442||Macedonia|658[0n]
Gegenes nostrodamus|WMB5048-14|RVcoll. 14-L179||Italy|655[0n]
Gegenes nostrodamus|EULEP1588-15|RVcoll.14-G620||Greece|614[0n]
Gegenes nostrodamus|EULEP1645-15|LR-08-D096.1||Greece|615[0n]
Gegenes nostrodamus|WMB2314-13|RVcoll.11-H622|Lampedusa|Italy|658[0n]
Gegenes nostrodamus|WMB5955-17|RVcoll14A097||Italy|658[0n]
Gegenes nostrodamus|WMB5971-17|RVcoll14V915||Italy|658[0n]
Gegenes nostrodamus|WMB4403-14|RVcoll.14-B420||Portugal|658[0n]
Gegenes nostrodamus|WMB4660-14|RVcoll.LD-2569|Lipari|Italy|658[0n]
Gegenes nostrodamus|WMB4676-14|RVcoll.LD-2640|Ischia|Italy|658[0n]
Gegenes nostrodamus|WMB524-11|RVcoll.11-H620|Lampedusa|Italy|658[0n]
Gegenes nostrodamus|WMB613-11|RVcoll.11-I026|Sicily|Italy|658[0n]
Gegenes nostrodamus|EULEP230-14|RVcoll.12-L082||Spain|658[0n]
Gegenes nostrodamus|BIBSA653-15|RVcoll. 15-A816||Italy|658[0n]
Gegenes nostrodamus|EULEP231-14|RVcoll.12-L083||Spain|658[0n]
Gegenes nostrodamus|EULEP232-14|RVcoll.12-L084||Spain|658[0n]
Gegenes nostrodamus|EULEP233-14|RVcoll.12-L085||Spain|658[0n]
Gegenes nostrodamus|BIBSA879-15|LD-3491||Italy|658[0n]
Gegenes nostrodamus|BIBSA619-15|LEP-SS-00239||Italy|658[0n]
Gegenes nostrodamus|WMB1909-13|RVcoll.11-H621|Lampedusa|Italy|635[0n]
Gegenes nostrodamus|WMB2127-13|RVcoll.12-R236||Italy|658[0n]
Gegenes nostrodamus|WMB2777-13|RVcoll.11-I025|Sicily|Italy|658[0n]
Gegenes nostrodamus|WMB3449-14|RVcoll.10-A274||Spain|658[0n]
Gegenes nostrodamus|EZSPC1110-10|RVcoll.08-M712.1||Spain|658[0n]
Gegenes nostrodamus|EZSPC1341-10|RVcoll.130209KL14||Spain|658[0n]
Gegenes nostrodamus|EZSPM1040-12|Rvcoll.12-L450||Spain|658[0n]
Gegenes nostrodamus|EZSPN982-09|RVcoll.08-M022||Spain|658[0n]
Gegenes nostrodamus|EZSPN981-09|RVcoll.08-M021||Spain|658[0n]
Gegenes nostrodamus|EZSPM590-12|RVcoll.10-A016||Spain|658[0n]
Gegenes nostrodamus|EZSPN125-09|RVcoll.06-A312||Spain|658[0n]
Gegenes nostrodamus|EZSPN124-09|RVcoll.06-A311||Spain|658[0n]
Gegenes nostrodamus|EZSPN123-09|RVcoll.06-A310||Spain|658[0n]
Gegenes nostrodamus|EZSPN109-09|RVcoll.06-A259||Spain|658[0n]
Gegenes nostrodamus|EZSPN803-09|RVcoll.08-L456||Spain|658[0n]
Gegenes pumilio|WMB854-13|RVcoll.08-R544|Sicily|Italy|658[0n]
Gegenes pumilio|WMB2716-13|RVcoll.10-C524||Italy|627[0n]
Gegenes pumilio|EULEP1625-15|RVcoll.14-G723||Greece|627[0n]
Gegenes pumilio|EULEP1585-15|RVcoll.14-G614||Greece|658[0n]
Gegenes pumilio|EULEP1782-15|RVcoll.14-H588||Greece|658[0n]
Gegenes pumilio|EULEP1774-15|RVcoll.14-H536||Greece|658[0n]
Gegenes pumilio|EULEP1288-15|RVcoll.14-F708||Greece|658[0n]
Gegenes pumilio|EULEP4917-16|RVcoll16J623||Greece|658[0n]
Gegenes pumilio|WMB530-11|RVcoll.11-H658|Pantelleria|Italy|658[0n]
Gegenes pumilio|WMB134-11|RVcoll.11-D138|Pantelleria|Italy|658[0n]
Gegenes pumilio|BIBSA489-15|LEP-SS-00109||Italy|658[0n]
Gegenes pumilio|WMB2270-13|RVcoll.LD-2713||Malta|658[0n]
Gegenes pumilio|EULEP2861-15|RVcoll.14-N945|Cyprus|Cyprus|658[0n]
Gegenes pumilio|BCLEP103-17|LEP-SS-00554||Italy|658[0n]
Gegenes pumilio|BIBSA490-15|LEP-SS-00110||Italy|658[0n]
Gegenes pumilio|BCLEP102-17|LEP-SS-00553||Italy|622[0n]
Gegenes pumilio|EULEP5683-17|RVcoll16L005|Sardinia|Italy|626[0n]
Gegenes pumilio|EULEP5671-17|RVcoll16J792|Fournoi|Greece|658[0n]
Gegenes pumilio|EULEP4042-16|RVcoll16H959||Cyprus|658[0n]
Gegenes pumilio|WMB5482-14|RVcoll.14-N707|Alicudi|Italy|658[0n]
Gegenes pumilio|WMB5461-14|RVcoll.14-N608|Levanzo|Italy|658[0n]
Gegenes pumilio|WMB4781-14|RVcoll. 14-I328||Italy|658[0n]
Gegenes pumilio|WMB4777-14|RVcoll. 14-I324||Italy|658[0n]
Gegenes pumilio|WMB579-11|RVcoll.11-H867|Salina|Italy|658[0n]
Gegenes pumilio|WMB478-11|RVcoll.11-H503|Capri|Italy|658[0n]
Gegenes pumilio|WMB286-11|RVcoll.08-H705||Italy|658[0n]
Gegenes pumilio|WMB4671-14|RVcoll.LD-2624|Ischia|Italy|658[0n]
Gegenes pumilio|WMB4636-14|RVcoll.LD-2401|Ustica|Italy|658[0n]
Gegenes pumilio|WMB2409-13|RVcoll.10-C523||Italy|658[0n]
Gegenes pumilio|WMB2375-13|RVcoll.LD-2721||Malta|658[0n]
Gegenes pumilio|WMB2373-13|RVcoll.LD-2714||Malta|658[0n]
Gegenes pumilio|WMB1279-13|RVcoll.12-M382|Sicily|Italy|658[0n]
Gegenes pumilio|WMB2745-13|RVcoll.10-C619||Italy|658[0n]
Gegenes pumilio|WMB2744-13|RVcoll.10-C618||Italy|658[0n]
Gegenes pumilio|WMB2729-13|RVcoll.10-C587||Malta|658[0n]
Gegenes pumilio|WMB3087-14|RVcoll.14-D954|Giglio|Italy|658[0n]
Gegenes pumilio|WMB3086-14|RVcoll.14-D953|Giglio|Italy|658[0n]
Gegenes pumilio|WMB3072-14|RVcoll.14-D939|Elba|Italy|658[0n]
Gegenes pumilio|WMB3071-14|RVcoll.14-D938|Elba|Italy|658[0n]
Gegenes pumilio|WMB2930-14|RVcoll.11-J733|Sicily|Italy|658[0n]
Gegenes pumilio|WMB2929-14|RVcoll.11-J732|Sicily|Italy|658[0n]
Gegenes pumilio|WMB225-11|RVcoll.08-R547|Sicily|Italy|658[0n]
Gegenes pumilio|WMB224-11|RVcoll.08-R543|Sicily|Italy|658[0n]
Gegenes pumilio|EZSPM1042-12|Rvcoll.12-M906||Spain|658[0n]
Gegenes pumilio|EZSPM1041-12|Rvcoll.12-M791||Spain|658[0n]
Gegenes pumilio|GWORZ074-10|BC ZSM Lep 30430||Italy|658[0n]
Pelopidas thrax|EULEP3035-15|RVcoll.14-O168|Cyprus|Cyprus|658[0n]
Pelopidas thrax|EULEP3037-15|RVcoll.14-O170|Cyprus|Cyprus|658[0n]
Pelopidas thrax|EULEP3036-15|RVcoll.14-O169|Cyprus|Cyprus|658[0n]
Pelopidas thrax|EULEP2290-15|RVcoll.14-N488|Samos|Greece|658[0n]
Pelopidas thrax|EULEP2289-15|RVcoll.14-N487|Samos|Greece|658[0n]

[illegible]
Pelopidas thrax|EULEP3036-15|RVcoll.14-O169|Cyprus|Cyprus|658[0n]
Pelopidas thrax|EULEP2290-15|RVcoll.14-N488|Samos|Greece|658[0n]
Pelopidas thrax|EULEP2289-15|RVcoll.14-N487|Samos|Greece|658[0n]
Pelopidas thrax|EULEP1987-15|RVcoll.14-H994|Chios|Greece|658[0n]
Pelopidas thrax|EULEP4881-16|RVcoll16H974||Cyprus|658[0n]
Hesperia comma|WMB4372-14|RVcoll.14-B155||Portugal|658[0n]
Hesperia comma|WMB3249-14|RVcoll.08-L271||Spain|658[0n]
Hesperia comma|WMB3393-14|RVcoll.09-V573||Spain|658[0n]
Hesperia comma|EZSPC430-09|RVcoll.08-P366||Spain|658[0n]
Hesperia comma|EZSPM462-09|Rvcoll.09-V541||Spain|658[0n]
Hesperia comma|EZSPM107-09|RVcoll.08-P446||Spain|658[0n]
Hesperia comma|EZSPN969-09|RVcoll.08-L989||Spain|656[0n]
Hesperia comma|EZSPM461-09|Rvcoll.09-V932||Spain|658[0n]
Hesperia comma|EZROM723-08|RV-06-G555||Spain|658[0n]
Hesperia comma|EZSPC431-09|RVcoll.08-R100||Spain|658[0n]
Hesperia comma|EULEP4805-16|RVcoll16H691||Norway|658[0n]
Hesperia comma|GBLAB139-13|BC ZSM Lep 75765||Germany|658[0n]
Hesperia comma|OXB234-15|OXB-TGS-509||United Kingdom|658[0n]
Hesperia comma|OXB235-15|OXB-TGS-510||United Kingdom|658[0n]
Hesperia comma|WMB1229-13|RVcoll.11-H299|Sicily|Italy|658[0n]
Hesperia comma|WMB2092-13|RVcoll.12-R076|Sicily|Italy|658[0n]
Hesperia comma|WMB2794-13|RVcoll.11-J701|Sicily|Italy|632[0n]
Hesperia comma|WMB3139-14|RVcoll.06-V732||Spain|658[0n]
Hesperia comma|EZSPC429-09|RVcoll.08-R170||Spain|658[0n]
Hesperia comma|EZSPC428-09|RVcoll.08-R153||Spain|658[0n]
Hesperia comma|EZSPN119-09|RVcoll.06-A292||Spain|658[0n]
Hesperia comma|EZSPN112-09|RVcoll.06-A269||Spain|658[0n]
Hesperia comma|LEFIJ490-10|MM17115||Finland|658[0n]
Hesperia comma|LEFIJ489-10|MM17114||Finland|658[0n]
Hesperia comma|LON254-08|NHMO-07043||Norway|657[0n]
Hesperia comma|LEFID810-10|MM06896||Finland|658[0n]
Hesperia comma|LEASS551-17|TLMF Lep 22199||Austria|658[0n]
Hesperia comma|ABOLD027-16|TLMF Lep 21105||Austria|658[0n]
Hesperia comma|EULEP4993-16|RVcoll16I721||Poland|658[0n]
Hesperia comma|EULEP4058-16|RVcoll15I818||Austria|658[0n]
Hesperia comma|ABOLB031-15|TLMF Lep 17036||Italy|658[0n]
Hesperia comma|PHLAI513-13|TLMF Lep 09075||Austria|658[0n]
Hesperia comma|LEATG024-14|TLMF Lep 13811||Austria|658[0n]
Hesperia comma|GWORR701-10|BC ZSM Lep 32197||Germany|658[0n]
Hesperia comma|GWORR702-10|BC ZSM Lep 32198||Germany|658[0n]
Hesperia comma|GWORZ073-10|BC ZSM Lep 30429||Italy|658[0n]
Hesperia comma|GWORA2488-09|BC ZSM Lep 30700||Germany|658[0n]
Hesperia comma|EZROM207-08|RV-06-V656||Romania|658[0n]
Hesperia comma|WMB285-11|RVcoll.10-B601||France|658[0n]
Hesperia comma|WMB3674-14|RVcoll.11-I988||France|658[0n]
Hesperia comma|WMB3502-14|RVcoll.10-B750||France|658[0n]
Hesperia comma|WMB3661-14|RVcoll.11-I782||France|658[0n]
Hesperia comma|WMB1696-13|RVcoll.12-P571||France|658[0n]
Hesperia comma|WMB1887-13|RVcoll.11-J755||Italy|658[0n]
Hesperia comma|WMB2019-13|RVcoll.12-Q677||Italy|658[0n]
Hesperia comma|WMB2055-13|RVcoll.12-Q823||Italy|658[0n]
Hesperia comma|WMB2566-13|RVcoll.12-Q822||Italy|658[0n]
Hesperia comma|WMB3980-14|RVcoll.12-Q231||France|658[0n]
Hesperia comma|WMB3989-14|RVcoll.12-Q333||France|658[0n]
Hesperia comma|WMB5046-14|RVcoll. 14-L177||Italy|658[0n]
Hesperia comma|EULEP4057-16|RVcoll15I092||Italy|658[0n]
Hesperia comma|EULEP4059-16|RVcoll15J015||Switzerland|658[0n]
Hesperia comma|EULEP4061-16|RVcoll15J301||France|658[0n]
Hesperia comma|EULEP5037-16|RVcoll16J003||Slovakia|658[0n]
Hesperia comma|EULEP5080-16|RVcoll16J319||Germany|658[0n]
Hesperia comma|EULEP016-14|RVcoll.07-C396||Romania|658[0n]
Hesperia comma|EULEP191-14|RVcoll.11-J950||Romania|658[0n]
Hesperia comma|EULEP192-14|RVcoll.11-J951||Romania|658[0n]
Hesperia comma|EULEP5711-17|RVcoll16L056||Italy|658[0n]
Hesperia comma|EULEP633-15|RVcoll.11-J045||Switzerland|658[0n]
Hesperia comma|EULEP885-15|RVcoll.14-C501||Greece|658[0n]
Hesperia comma|EULEP964-15|RVcoll.14-D029||Bulgaria|658[0n]
Hesperia comma|EULEP982-15|RVcoll.14-D137||Austria|658[0n]
Hesperia comma|EULEP2438-15|RVcoll.14-V241||Switzerland|658[0n]
Hesperia comma|EULEP190-14|RVcoll.11-J949||Romania|658[0n]
Hesperia comma|EULEP136-14|RVcoll.10-C382||Romania|658[0n]
Hesperia comma|EULEP1955-15|RVcoll.14-G742||Romania|658[0n]
Hesperia comma|BIBSA1191-15|15-M266||Italy|658[0n]
Hesperia comma|BIBSA110-15|RVcoll.14-D538||Italy|658[0n]
Hesperia comma|BIBSA491-15|LEP-SS-00111||Italy|658[0n]
Hesperia comma|BIBSA492-15|LEP-SS-00112||Italy|658[0n]
Hesperia comma|BIBSA224-15|RVcoll.14-E014||Italy|658[0n]
Hesperia comma|BIBSA301-15|RVcoll.14-E103||Italy|658[0n]
Hesperia comma|OXB336-15|RVcoll.14-N050||Italy|658[0n]
Hesperia comma|OXB307-15|RVcoll.14-N021||Italy|658[0n]
Hesperia comma|EULEP4060-16|RVcoll15J281||Switzerland|658[0n]
Hesperia comma|BIBSA309-15|RVcoll.14-E111||Italy|658[0n]
Hesperia comma|LEASS920-17|KLM Lep 08425||Austria|658[0n]
Hesperia comma|OXB1291-15|Rvcoll_15-M154||France|658[0n]
Hesperia comma|LON208-08|NHMO-06220||Norway|657[0n]
Hesperia comma|EULEP4719-16|RVcoll16B363||Norway|642[0n]
Hesperia comma|FBLMU513-09|BC ZSM Lep 27163||Germany|613[0n]
Hesperia comma|WMB3952-14|RVcoll.12-P720||France|620[0n]
Hesperia comma|EULEP5055-16|RVcoll16J101||Czech Republic|637[0n]
Hesperia comma|BIBSA109-15|RVcoll.14-D537||Italy|637[0n]
Hesperia comma|EZROM296-08|RV-07-E658||Romania|633[0n]
Hesperia comma|PHLAI540-13|TLMF Lep 09102||Austria|625[0n]
Hesperia comma|LEATG025-14|TLMF Lep 13812||Austria|658[0n]
Hesperia comma|LEATG399-14|TLMF Lep 14186||Italy|658[0n]
Ochlodes sylvanus|BIBSA1838-17|RVcoll16C714||Italy|658[0n]
Ochlodes sylvanus|LON951-12|NHMO Lep2011.011||Norway|658[0n]
Ochlodes sylvanus|WMB3686-14|RVcoll.11-J233||Spain|658[0n]
Ochlodes sylvanus|OXB1405-15|Rvcoll_15-M774||France|658[0n]
Ochlodes sylvanus|OXB1208-15|Rvcoll_15-M633||France|658[0n]
Ochlodes sylvanus|BIBSA1402-15|15-N008||Italy|658[0n]
Ochlodes sylvanus|WMB5365-14|RVcoll.14-J737||France|658[0n]
Ochlodes sylvanus|WMB4146-14|RVcoll.13-S685||Italy|658[0n]
[illegible]

Ochlodes sylvanus|BIBSA1402-15|15-N008||Italy|658[0n]
Ochlodes sylvanus|WMB5365-14|RVcoll.14-J737||France|658[0n]
Ochlodes sylvanus|WMB4146-14|RVcoll.13-S685||Italy|658[0n]
Ochlodes sylvanus|WMB3841-14|RVcoll.10-C521||Italy|658[0n]
Ochlodes sylvanus|GWORR404-10|BC ZSM Lep 29620||Italy|658[0n]
Ochlodes sylvanus|EZSPM915-12|Rvcoll.12-M637||Spain|658[0n]
Ochlodes sylvanus|OXB1266-15|Rvcoll_15-M123||France|658[0n]
Ochlodes sylvanus|LEFIB870-10|MM02929||Finland|632[0n]
Ochlodes sylvanus|OXB236-15|OXB-TGS-511||United Kingdom|658[0n]
Ochlodes sylvanus|OXB993-15|15-A943||Italy|658[0n]
Ochlodes sylvanus|OXB1119-15|15-A652||Italy|658[0n]
Ochlodes sylvanus|BIBSA342-15|RVcoll.14-I010||Italy|658[0n]
Ochlodes sylvanus|WMB4226-14|RVcoll.13-T769||Italy|658[0n]
Ochlodes sylvanus|BIBSA1090-15|15-L338||Italy|658[0n]
Ochlodes sylvanus|WMB2083-13|RVcoll.12-R016|Sicily|Italy|658[0n]
Ochlodes sylvanus|OXB634-15|14-O023||Italy|658[0n]
Ochlodes sylvanus|OXB586-15|14-N975||Italy|658[0n]
Ochlodes sylvanus|OXB1424-15|Rvcoll_15-M571||France|658[0n]
Ochlodes sylvanus|OXB1422-15|Rvcoll_15-M157||France|658[0n]
Ochlodes sylvanus|GBLAA1371-15|BC ZSM Lep 87067||Germany|658[0n]
Ochlodes sylvanus|GBLAF829-14|BC ZSM Lep 82915||Germany|658[0n]
Ochlodes sylvanus|GBLAB045-13|BC ZSM Lep 72536||Germany|658[0n]
Ochlodes sylvanus|BIBSA1833-17|RVcoll16C708||Italy|658[0n]
Ochlodes sylvanus|GBLAA406-14|BC ZSM Lep 80497||Germany|658[0n]
Ochlodes sylvanus|BIBSA446-15|RVcoll.14-I114||Italy|658[0n]
Ochlodes sylvanus|BIBSA692-15|RVcoll. 11-I255||Italy|658[0n]
Ochlodes sylvanus|BIBSA098-15|RVcoll.14-D520||Italy|658[0n]
Ochlodes sylvanus|EULEP2447-15|RVcoll.14-V263||Switzerland|658[0n]
Ochlodes sylvanus|EULEP2428-15|RVcoll.14-V217||Belgium|658[0n]
Ochlodes sylvanus|WMB1233-13|RVcoll.11-H362||Italy|658[0n]
Ochlodes sylvanus|WMB3700-14|RVcoll.12-P807||France|658[0n]
Ochlodes sylvanus|WMB3506-14|RVcoll.10-B781||France|658[0n]
Ochlodes sylvanus|WMB3443-14|RVcoll.09-X558||Spain|658[0n]
Ochlodes sylvanus|EZSPN266-09|RVcoll.07-W180||Spain|658[0n]
Ochlodes sylvanus|EZSPN1002-09|RVcoll.08-M088||Spain|658[0n]
Ochlodes sylvanus|WMB2883-14|RVcoll.10-C777|Argentario|Italy|658[0n]
Ochlodes sylvanus|WMB113-11|RVcoll.10-B656||France|658[0n]
Ochlodes sylvanus|EZSPN987-09|RVcoll.08-M030||Spain|658[0n]
Ochlodes sylvanus|EZSPM375-09|RVcoll.08-J862||Spain|658[0n]
Ochlodes sylvanus|EZSPC578-09|RVcoll.07-C666||Spain|658[0n]
Ochlodes sylvanus|EZSPN590-09|RVcoll.08-J160||Portugal|658[0n]
Ochlodes sylvanus|EZSPN544-09|RVcoll.08-J044||Spain|658[0n]
Ochlodes sylvanus|EZSPN265-09|RVcoll.07-W177||Spain|658[0n]
Ochlodes sylvanus|EZSPN241-09|RVcoll.07-W096||Spain|658[0n]
Ochlodes sylvanus|EZSPC577-09|RVcoll.08-P063||Spain|658[0n]
Ochlodes sylvanus|EZRMN151-08|RVcoll.08-M458||Romania|658[0n]
Ochlodes sylvanus|EZRMN149-08|RVcoll.08-M377||Romania|658[0n]
Ochlodes sylvanus|EULEP2383-15|RVcoll.14-V078||Ukraine|658[0n]
Ochlodes sylvanus|EULEP1616-15|RVcoll.14-G688||Greece|658[0n]
Ochlodes sylvanus|EULEP957-15|RVcoll.14-D004||Bulgaria|658[0n]
Ochlodes sylvanus|EULEP790-15|RVcoll.12-Z224||Sweden|658[0n]
Ochlodes sylvanus|EZSPN113-09|RVcoll.06-A272||Spain|658[0n]
Ochlodes sylvanus|EZSPC576-09|RVcoll.07-W104||Spain|658[0n]
Ochlodes sylvanus|EZSPC575-09|RVcoll.08-M713||Spain|658[0n]
Ochlodes sylvanus|EZSPC574-09|RVcoll.08-L509||Spain|658[0n]
Ochlodes sylvanus|EZRMN148-08|RVcoll.08-M227||Romania|658[0n]
Ochlodes sylvanus|EZROM446-08|RV-06-M958||Romania|658[0n]
Ochlodes sylvanus|EULEP584-15|RVcoll.10-A693||Estonia|658[0n]
Ochlodes sylvanus|EULEP543-15|RVcoll.08-L301||Denmark|658[0n]
Ochlodes sylvanus|EULEP1364-15|RVcoll.14-F932||Greece|658[0n]
Ochlodes sylvanus|EULEP1055-15|RVcoll.14-E939||Serbia|658[0n]
Ochlodes sylvanus|EZROM445-08|RV-06-M847||Romania|658[0n]
Ochlodes sylvanus|EZROM444-08|RV-07-C934||Romania|658[0n]
Ochlodes sylvanus|EULEP353-14|MM23838||Lithuania|658[0n]
Ochlodes sylvanus|EULEP352-14|MM23837||Lithuania|658[0n]
Ochlodes sylvanus|EULEP5040-16|RVcoll16J025||Slovakia|658[0n]
Ochlodes sylvanus|EULEP4751-16|RVcoll16G846||Sweden|658[0n]
Ochlodes sylvanus|EZROM443-08|RV-07-D115||Romania|658[0n]
Ochlodes sylvanus|EZROM339-08|RV-08-A017||Romania|658[0n]
Ochlodes sylvanus|BIBSA1067-15|14-W658||Italy|658[0n]
Ochlodes sylvanus|BIBSA729-15|LD-3020||Italy|658[0n]
Ochlodes sylvanus|BIBSA1110-15|15-L813||Italy|658[0n]
Ochlodes sylvanus|EULEP2000-15|RVcoll.14-I776||Slovakia|658[0n]
Ochlodes sylvanus|EULEP4337-16|RVcoll15G164||France|658[0n]
Ochlodes sylvanus|EULEP4336-16|RVcoll15G087||France|658[0n]
Ochlodes sylvanus|EZRMN150-08|RVcoll.08-M399||Romania|658[0n]
Ochlodes sylvanus|FBLMT906-09|BC ZSM Lep 25466||Germany|658[0n]
Ochlodes sylvanus|EZRMN336-08|RVcoll.08-H008||Romania|658[0n]
Ochlodes sylvanus|FBLMU257-09|BC ZSM Lep 25767||Germany|658[0n]
Ochlodes sylvanus|LEFIJ487-10|MM17112||Finland|658[0n]
Ochlodes sylvanus|FBLMU507-09|BC ZSM Lep 27157||Germany|658[0n]
Ochlodes sylvanus|EULEP4335-16|RVcoll15I142||Austria|658[0n]
Ochlodes sylvanus|EULEP4334-16|RVcoll15H184||Switzerland|658[0n]
Ochlodes sylvanus|EULEP4333-16|RVcoll15G792||Italy|658[0n]
Ochlodes sylvanus|EULEP4332-16|RVcoll15G037||France|658[0n]
Ochlodes sylvanus|GWORZ071-10|BC ZSM Lep 30427||Italy|658[0n]
Ochlodes sylvanus|GWORR703-10|BC ZSM Lep 32199||Germany|658[0n]
Ochlodes sylvanus|WMB4980-14|RVcoll. 14-I527||Italy|658[0n]
Ochlodes sylvanus|WMB5384-14|RVcoll.14-J845||France|658[0n]
Ochlodes sylvanus|WMB4883-14|RVcoll.14-I430||Italy|658[0n]
Ochlodes sylvanus|WMB4820-14|RVcoll. 14-I367||Italy|658[0n]
Ochlodes sylvanus|LEATG023-14|TLMF Lep 13810||Austria|658[0n]
Ochlodes sylvanus|LEATH761-14|TLMF Lep 15973||Italy|658[0n]
Ochlodes sylvanus|WMB648-11|RVcoll.11-I193||Italy|658[0n]
Ochlodes sylvanus|WMB5092-14|RVcoll. 14-L223||Italy|658[0n]
Ochlodes sylvanus|WMB4316-14|RVcoll.14-A517||Italy|658[0n]
Ochlodes sylvanus|WMB3975-14|RVcoll.12-Q206||France|658[0n]
Ochlodes sylvanus|LEATG022-14|TLMF Lep 13809||Austria|658[0n]
Ochlodes sylvanus|ABOLD426-16|TLMF Lep 21578||Austria|658[0n]
Ochlodes sylvanus|WMB5302-14|RVcoll.14-I624||France|658[0n]
Ochlodes sylvanus|WMB5242-14|RVcoll.13-U094||Italy|658[0n]
Ochlodes sylvanus|WMB3883-14|RVcoll.12-M520||Italy|658[0n]
Ochlodes sylvanus|WMB2586-13|RVcoll.12-R129|Sicily|Italy|658[0n]

Ochlodes sylvanus|WMB5242-14|RVcoll.13-U094||Italy|658[0n]
Ochlodes sylvanus|WMB3883-14|RVcoll.12-M520||Italy|658[0n]
Ochlodes sylvanus|WMB2586-13|RVcoll.12-R129|Sicily|Italy|658[0n]
Ochlodes sylvanus|LEATG404-14|TLMF Lep 14191||Italy|658[0n]
Ochlodes sylvanus|ABOLD065-16|TLMF Lep 21143||Austria|658[0n]
Ochlodes sylvanus|WMB3803-14|RVcoll.07-D840||Italy|658[0n]
Ochlodes sylvanus|WMB2293-13|RVcoll.10-C666||Italy|658[0n]
Ochlodes sylvanus|WMB1878-13|RVcoll.11-J681|Sicily|Italy|658[0n]
Ochlodes sylvanus|WMB1598-13|RVcoll.12-O620||France|658[0n]
Ochlodes sylvanus|WMB2017-13|RVcoll.12-Q668||Italy|658[0n]
Ochlodes sylvanus|WMB2371-13|RVcoll.12-R441||Italy|658[0n]
Ochlodes sylvanus|WMB2088-13|RVcoll.12-R032|Sicily|Italy|658[0n]
Ochlodes sylvanus|WMB1821-13|RVcoll.11-I637||France|658[0n]
Ochlodes sylvanus|BIBSA1249-15|15-F828||France|658[0n]
Ochlodes sylvanus|BIBSA1237-15|15-C340||Italy|658[0n]
Ochlodes sylvanus|BIBSA1751-16|11-I256||Italy|658[0n]
Ochlodes sylvanus|BIBSA1085-15|15-K574||Italy|658[0n]
Ochlodes sylvanus|BIBSA017-14|LEP-SS-00017||Italy|658[0n]
Ochlodes sylvanus|BIBSA016-14|LEP-SS-00016||Italy|658[0n]
Ochlodes sylvanus|GBLAA1284-15|BC ZSM Lep 87170||Germany|658[0n]
Ochlodes sylvanus|GBLAD256-14|BC ZSM Lep 78827||Germany|658[0n]
Ochlodes sylvanus|OXB756-15|13-U491||Italy|658[0n]
Ochlodes sylvanus|OXB719-15|13-U110||Italy|658[0n]
Ochlodes sylvanus|LEASS552-17|TLMF Lep 22200||Austria|658[0n]
Ochlodes sylvanus|LEATJ1224-16|TLMF Lep 19547||Hungary|658[0n]
Ochlodes sylvanus|OXB908-15|15-A552||Italy|658[0n]
Ochlodes sylvanus|OXB347-15|RVcoll.14-N061||Italy|658[0n]
Ochlodes sylvanus|OXB958-15|15-A908||Italy|658[0n]
Ochlodes sylvanus|OXB1040-15|LD-2855||Italy|658[0n]
Ochlodes sylvanus|OXB226-15|OXB-TGS-447||United Kingdom|658[0n]
Ochlodes sylvanus|OXB1319-15|Rvcoll_15-M590||France|658[0n]
Ochlodes sylvanus|OXB1331-15|Rvcoll_15-M672||France|658[0n]
Ochlodes sylvanus|OXB1191-15|Rvcoll_15-M603||France|658[0n]
Ochlodes sylvanus|OXB1199-15|Rvcoll_15-M615||France|658[0n]
Ochlodes sylvanus|OXB1275-15|Rvcoll_15-M135||France|658[0n]
Ochlodes sylvanus|OXB1389-15|Rvcoll_15-M754||France|658[0n]
Ochlodes sylvanus|BIBSA1568-16|LEP-SS-00387||Italy|638[0n]
Ochlodes sylvanus|LEFIB869-10|MM02928||Finland|648[0n]
Ochlodes sylvanus|BIBSA139-15|RVcoll.14-I153||Italy|644[0n]
Ochlodes sylvanus|BIBSA996-15|15-C028||Italy|622[0n]
Ochlodes sylvanus|BIBSA984-15|14-W528||Italy|622[0n]
Ochlodes sylvanus|PHLAH467-12|TLMF Lep 08286||Austria|615[0n]
Ochlodes sylvanus|LEATG403-14|TLMF Lep 14190||Italy|658[0n]
Thymelicus sylvestris|EULEP1901-15|RVcoll.14-B776|Genit. examined|Albania|658[0n]
Thymelicus sylvestris|EULEP1507-15|RVcoll.14-G416|Genit. examined|Greece|658[0n]
Thymelicus sylvestris|EULEP1278-15|RVcoll.14-F685|Genit. examined|Greece|658[0n]
Thymelicus sylvestris|EULEP1267-15|RVcoll.14-F661||Greece|658[0n]
Thymelicus sylvestris|EULEP4689-16|RVcoll10B380||Bulgaria|658[0n]
Thymelicus sylvestris|OXB330-15|RVcoll.14-N044||Italy|658[0n]
Thymelicus sylvestris|GBLAA1402-15|BC ZSM Lep 87098||Germany|658[0n]
Thymelicus sylvestris|EULEP4703-16|RVcoll15Q170||Ukraine|658[0n]
Thymelicus sylvestris|EZSPM1028-12|Rvcoll.12-M778||Spain|658[0n]
Thymelicus sylvestris|OXB1270-15|Rvcoll_15-M129||France|658[0n]
Thymelicus sylvestris|EZSPC345-09|RVcoll.08-P355||Spain|658[0n]
Thymelicus sylvestris|EZSPC346-09|RVcoll.08-L486||Spain|658[0n]
Thymelicus sylvestris|EZSPN291-09|RVcoll.07-W252||Spain|658[0n]
Thymelicus sylvestris|EZSPM961-12|Rvcoll.12-M712||Spain|658[0n]
Thymelicus sylvestris|EZSPM973-12|Rvcoll.12-M731||Spain|658[0n]
Thymelicus sylvestris|EZSPM632-12|RVcoll.11-E182||Spain|658[0n]
Thymelicus sylvestris|EZSPN597-09|RVcoll.08-J187||Portugal|658[0n]
Thymelicus sylvestris|EZSPN979-09|RVcoll.08-M017||Spain|658[0n]
Thymelicus sylvestris|EZSPC934-10|RVcoll.06-A265||Spain|658[0n]
Thymelicus sylvestris|EZSPC936-10|RVcoll.08-L506||Spain|658[0n]
Thymelicus sylvestris|EZSPM928-12|Rvcoll.12-M652||Spain|658[0n]
Thymelicus sylvestris|EZSPC719-10|RVcoll.08-H340||Spain|658[0n]
Thymelicus sylvestris|EZSPC1050-10|RVcoll.08-J775||Spain|658[0n]
Thymelicus sylvestris|EZSPC1058-10|RVcoll.08-L005|Genit. examined|Spain|658[0n]
Thymelicus sylvestris|WMB3121-14|RVcoll.06-A301||Spain|658[0n]
Thymelicus sylvestris|WMB3478-14|RVcoll.10-A876||Spain|658[0n]
Thymelicus sylvestris|WMB1600-13|RVcoll.12-O632||France|658[0n]
Thymelicus sylvestris|WMB1851-13|RVcoll.11-I958||France|658[0n]
Thymelicus sylvestris|WMB4463-14|RVcoll.14-D334||Spain|658[0n]
Thymelicus sylvestris|WMB5370-14|RVcoll.14-J760||France|658[0n]
Thymelicus sylvestris|WMB5217-14|RVcoll.13-T967||Italy|658[0n]
Thymelicus sylvestris|WMB5228-14|RVcoll.13-T988||Italy|658[0n]
Thymelicus sylvestris|EULEP4692-16|RVcoll15G033||Belgium|658[0n]
Thymelicus sylvestris|EULEP4700-16|RVcoll15G054||France|658[0n]
Thymelicus sylvestris|EULEP4702-16|RVcoll15G133||France|658[0n]
Thymelicus sylvestris|EULEP508-15|RVcoll.07-C554||Germany|658[0n]
Thymelicus sylvestris|EULEP1992-15|RVcoll.14-I709||France|658[0n]
Thymelicus sylvestris|BIBSA1046-15|15-M628||France|658[0n]
Thymelicus sylvestris|BIBSA1365-15|15-M895||Italy|658[0n]
Thymelicus sylvestris|LEATJ1170-16|TLMF Lep 19493||Czech Republic|658[0n]
Thymelicus sylvestris|OXB1420-15|Rvcoll_15-M130||France|658[0n]
Thymelicus sylvestris|OXB1423-15|Rvcoll_15-M169||France|658[0n]
Thymelicus sylvestris|OXB1206-15|Rvcoll_15-M625||France|658[0n]
Thymelicus sylvestris|OXB1231-15|Rvcoll_15-M666||France|658[0n]
Thymelicus sylvestris|OXB1376-15|Rvcoll_15-M740||France|658[0n]
Thymelicus sylvestris|OXB1393-15|Rvcoll_15-M759||France|658[0n]
Thymelicus sylvestris|WMB4891-14|RVcoll.14-I438||Italy|658[0n]
Thymelicus sylvestris|OXB1404-15|Rvcoll_15-M773||France|658[0n]
Thymelicus sylvestris|EULEP4701-16|RVcoll15G079||France|643[0n]
Thymelicus sylvestris|WMB3307-14|RVcoll.08-P489||Spain|609[0n]
Thymelicus sylvestris|EZSPM924-12|Rvcoll.12-M646||Spain|620[0n]
Thymelicus sylvestris|EZSPM1024-12|Rvcoll.12-M774||Spain|633[0n]
Thymelicus sylvestris|EZSPC344-09|RVcoll.07-C634|Genit. examined|Spain|653[0n]
Thymelicus sylvestris|EZSPC343-09|RVcoll.08-R284||Spain|658[0n]
Thymelicus sylvestris|EZSPC342-09|RVcoll.08-L451||Spain|658[0n]
Thymelicus sylvestris|EZSPN122-09|RVcoll.06-A300||Spain|658[0n]
Thymelicus sylvestris|FBLMZ508-12|BC ZSM Lep 61264||Germany|658[0n]
Thymelicus sylvestris|WMB2254-13|RVcoll.12-R407||Italy|658[0n]
Thymelicus sylvestris|OXB760-15|13-U501||Italy|658[0n]
Thymelicus sylvestris|OXB[illegible]-15|15-A62[illegible]||Italy|658[0n]

Thymelicus sylvestris|FBLMZ308-12|BC ZSM Lep 61264||Germany|658[0n]
Thymelicus sylvestris|WMB2254-13|RVcoll.12-R407||Italy|658[0n]
Thymelicus sylvestris|OXB760-15|13-U501||Italy|658[0n]
Thymelicus sylvestris|OXB1090-15|15-A623||Italy|658[0n]
Thymelicus sylvestris|BIBSA007-14|LEP-SS-00007||Italy|658[0n]
Thymelicus sylvestris|BIBSA1644-16|LEP-SS-00396||Italy|658[0n]
Thymelicus sylvestris|BIBSA006-14|LEP-SS-00006||Italy|658[0n]
Thymelicus sylvestris|BIBSA1530-16|07-E030||Italy|658[0n]
Thymelicus sylvestris|BIBSA1641-16|LEP-SS-00395||Italy|658[0n]
Thymelicus sylvestris|EULEP1920-15|RVcoll.14-B843||Bosnia and Herzegovina|658[0n]
Thymelicus sylvestris|EULEP594-15|RVcoll.10-C133||France|658[0n]
Thymelicus sylvestris|EULEP1117-15|RVcoll.14-F196||Serbia|658[0n]
Thymelicus sylvestris|EULEP1032-15|RVcoll.14-E875||Serbia|658[0n]
Thymelicus sylvestris|EULEP1009-15|RVcoll.14-E536||Romania|658[0n]
Thymelicus sylvestris|EULEP4691-16|RVcoll15C300||Italy|658[0n]
Thymelicus sylvestris|EULEP4690-16|RVcoll10B975||France|658[0n]
Thymelicus sylvestris|WMB4923-14|RVcoll.14-I470||Italy|658[0n]
Thymelicus sylvestris|WMB4920-14|RVcoll.14-I467||Italy|658[0n]
Thymelicus sylvestris|WMB4720-14|RVcoll.LD-3012|Sicily|Italy|658[0n]
Thymelicus sylvestris|WMB4283-14|RVcoll.14-A332||Italy|658[0n]
Thymelicus sylvestris|WMB3861-14|RVcoll.11-I262||Italy|658[0n]
Thymelicus sylvestris|GWORZ070-10|BC ZSM Lep 30426||Italy|658[0n]
Thymelicus sylvestris|GWORZ069-10|BC ZSM Lep 30425||Italy|658[0n]
Thymelicus sylvestris|PHLAF639-11|TLMF Lep 05809||Switzerland|658[0n]
Thymelicus sylvestris|PHLAH466-12|TLMF Lep 08285||Austria|658[0n]
Thymelicus sylvestris|LEATI072-15|TLMF Lep 17457||Austria|658[0n]
Thymelicus sylvestris|ABOLD005-16|TLMF Lep 21083||Austria|658[0n]
Thymelicus sylvestris|EULEP4698-16|RVcoll15I830||Austria|658[0n]
Thymelicus sylvestris|EZROM559-08|RV-07-D942|Genit. examined|Romania|658[0n]
Thymelicus sylvestris|EULEP1238-15|RVcoll.14-F555||Greece|658[0n]
Thymelicus sylvestris|EULEP5007-16|RVcoll16I837||Poland|658[0n]
Thymelicus sylvestris|EZROM557-08|RV-06-M948||Romania|658[0n]
Thymelicus sylvestris|EULEP1356-15|RVcoll.14-F921||Greece|658[0n]
Thymelicus sylvestris|EULEP1363-15|RVcoll.14-F930||Greece|658[0n]
Thymelicus sylvestris|EULEP1382-15|RVcoll.14-F983||Greece|658[0n]
Thymelicus sylvestris|EULEP1480-15|RVcoll.14-G273||Greece|658[0n]
Thymelicus sylvestris|EULEP1628-15|RVcoll.14-G730||Greece|658[0n]
Thymelicus sylvestris|EULEP1721-15|RVcoll.14-H212||Greece|658[0n]
Thymelicus sylvestris|EULEP1754-15|RVcoll.14-H409||Greece|658[0n]
Thymelicus sylvestris|EULEP1814-15|RVcoll.14-H807||Greece|658[0n]
Thymelicus sylvestris|EULEP1819-15|RVcoll.14-H826||Greece|658[0n]
Thymelicus sylvestris|EZROM556-08|RV-06-M940|Genit. examined|Romania|652[0n]
Thymelicus sylvestris|EULEP4959-16|RVcoll16I280||Poland|658[0n]
Thymelicus sylvestris|EULEP4704-16|RVcoll15P001||Belarus|658[0n]
Thymelicus sylvestris|LEEUA528-11|MM20587||Latvia|658[0n]
Thymelicus sylvestris|LEEUA527-11|MM20586||Latvia|658[0n]
Thymelicus sylvestris|OXB221-15|OXB-TGS-403||United Kingdom|658[0n]
Thymelicus sylvestris|EULEP769-15|RVcoll.12-R917|Chios|Greece|658[0n]
Thymelicus sylvestris|EULEP770-15|RVcoll.12-R919|Chios|Greece|658[0n]
Thymelicus sylvestris|EULEP721-15|RVcoll.12-N790|Lesvos|Greece|658[0n]
Thymelicus sylvestris|EULEP720-15|RVcoll.12-N789|Lesvos|Greece|658[0n]
Thymelicus sylvestris|BIBSA1737-16|LD-2933||Italy|658[0n]
Thymelicus sylvestris|EULEP4688-16|RVcoll10B350||Bulgaria|658[0n]
Thymelicus sylvestris|EULEP4728-16|RVcoll16G412||Denmark|658[0n]
Thymelicus sylvestris|WMB5263-14|RVcoll.14-A462||Italy|658[0n]
Thymelicus sylvestris|WMB4901-14|RVcoll.14-I448||Italy|658[0n]
Thymelicus sylvestris|WMB4898-14|RVcoll.14-I445||Italy|658[0n]
Thymelicus sylvestris|WMB4866-14|RVcoll.14-I413||Italy|658[0n]
Thymelicus sylvestris|WMB4821-14|RVcoll. 14-I368||Italy|658[0n]
Thymelicus sylvestris|WMB625-11|RVcoll.11-I066|Sicily|Italy|658[0n]
Thymelicus sylvestris|WMB565-11|RVcoll.11-H773|Sicily|Italy|658[0n]
Thymelicus sylvestris|WMB4540-14|RVcoll.LD-2810||Italy|658[0n]
Thymelicus sylvestris|EULEP5644-17|RVcoll15F148||Portugal|658[0n]
Thymelicus sylvestris|EULEP5625-17|RVcoll14W456||United Kingdom|658[0n]
Thymelicus sylvestris|EULEP5624-17|RVcoll14W437||United Kingdom|658[0n]
Thymelicus sylvestris|EULEP5445-17|RVcoll11H977|Sicily|Italy|658[0n]
Thymelicus sylvestris|EULEP4695-16|RVcoll15H998||Italy|658[0n]
Thymelicus sylvestris|EULEP4693-16|RVcoll15H342||Switzerland|658[0n]
Thymelicus sylvestris|EULEP1190-15|RVcoll.14-F463||Bulgaria|658[0n]
Thymelicus sylvestris|EULEP1019-15|RVcoll.14-E730||Romania|658[0n]
Thymelicus sylvestris|EULEP645-15|RVcoll.11-J141||Switzerland|658[0n]
Thymelicus sylvestris|EULEP1215-15|RVcoll.14-F515||Greece|658[0n]
Thymelicus sylvestris|EULEP958-15|RVcoll.14-D009||Bulgaria|658[0n]
Thymelicus sylvestris|EULEP932-15|RVcoll.14-C868||Bulgaria|658[0n]
Thymelicus sylvestris|EULEP975-15|RVcoll.14-D089||Bulgaria|658[0n]
Thymelicus sylvestris|EULEP1978-15|RVcoll.14-H908||France|658[0n]
Thymelicus sylvestris|EULEP1983-15|RVcoll.14-H943||France|658[0n]
Thymelicus sylvestris|BIBSA1236-15|15-C334||Italy|658[0n]
Thymelicus sylvestris|BIBSA725-15|LD-2809||Italy|658[0n]
Thymelicus sylvestris|BIBSA1408-15|15-N015||Italy|658[0n]
Thymelicus sylvestris|BIBSA1562-16|11-H772||Italy|658[0n]
Thymelicus sylvestris|BIBSA988-15|14-W628||Italy|658[0n]
Thymelicus sylvestris|BIBSA1614-16|15-C380||Italy|658[0n]
Thymelicus sylvestris|BIBSA1810-17|RVcoll14I279||Italy|658[0n]
Thymelicus sylvestris|OXB1435-16|OXB-TGS-1209||United Kingdom|658[0n]
Thymelicus sylvestris|OXB213-15|OXB-TGS-265||United Kingdom|658[0n]
Thymelicus sylvestris|OXB218-15|OXB-TGS-358||United Kingdom|658[0n]
Thymelicus sylvestris|OXB229-15|OXB-TGS-465||United Kingdom|658[0n]
Thymelicus sylvestris|EULEP4699-16|RVcoll15J233||Switzerland|658[2n]
Thymelicus sylvestris|EULEP1002-15|RVcoll.14-G456||Greece|614[0n]
Thymelicus sylvestris|WMB4822-14|RVcoll. 14-I369||Italy|632[0n]
Thymelicus sylvestris|BIBSA1750-16|11-I263||Italy|614[0n]
Thymelicus sylvestris|WMB4719-14|RVcoll.LD-3011|Sicily|Italy|629[0n]
Thymelicus sylvestris|WMB4161-14|RVcoll.13-S718||Italy|617[0n]
Thymelicus sylvestris|WMB4153-14|RVcoll.13-S707||Italy|658[0n]
Thymelicus sylvestris|WMB1978-13|RVcoll.11-I260||Italy|658[0n]
Thymelicus sylvestris|WMB1951-13|RVcoll.11-H964|Sicily|Italy|658[0n]
Thymelicus sylvestris|WMB1822-13|RVcoll.11-I639||France|658[0n]
Thymelicus sylvestris|WMB792-13|RVcoll.07-D852||Italy|658[0n]
Thymelicus sylvestris|WMB3510-14|RVcoll.10-B804||France|658[0n]
Thymelicus sylvestris|WMB2779-13|RVcoll.11-I099|Sicily|Italy|658[0n]
Thymelicus sylvestris|EZSPC998-10|RVcoll.09-V464||Spain|658[0n]
Thymelicus sylvestris|EZSPC1204-10|RVcoll.09-V461||Spain|658[0n]

Thymelicus sylvestris|WMB2779-13|RVcoll.11-I099|Sicily|Italy|658[0n]
Thymelicus sylvestris|EZSPC998-10|RVcoll.09-V464||Spain|658[0n]
Thymelicus sylvestris|EZSPC1204-10|RVcoll.09-V461||Spain|658[0n]
Thymelicus sylvestris|EZSPC1203-10|RVcoll.09-V451||Spain|658[0n]
Thymelicus sylvestris|EZSPM645-12|RVcoll.11-I407||Spain|658[0n]
Thymelicus sylvestris|EZSPM633-12|RVcoll.11-G143||Spain|658[0n]
Thymelicus sylvestris|EZSPM621-12|RVcoll.11-D880.1||Spain|658[0n]
Thymelicus sylvestris|EZSPM620-12|RVcoll.11-D879.1||Spain|658[0n]
Thymelicus sylvestris|EZSPN1073-11|Rvcoll.09-V460||Spain|658[0n]
Thymelicus sylvestris|EZSPM665-12|RVcoll.11-I581||Spain|658[0n]
Thymelicus sylvestris|EZSPM659-12|RVcoll.11-I501|Genit. examined|Spain|658[0n]
Thymelicus sylvestris|EZROM692-08|RV-07-E469||Romania|658[0n]
Thymelicus sylvestris|EZROM690-08|RV-07-D567||Romania|658[0n]
Thymelicus sylvestris|EZROM337-08|RV-08-A015||Romania|658[0n]
Thymelicus sylvestris|EZRMN295-08|RVcoll.08-M517||Romania|658[0n]
Thymelicus sylvestris|EZRMN290-08|RVcoll.06-V658||Romania|658[0n]
Thymelicus sylvestris|EZRMN286-08|RVcoll.07-D561||Romania|658[0n]
Thymelicus sylvestris|GWORA2487-09|BC ZSM Lep 30699||Germany|658[0n]
Thymelicus sylvestris|LENOA1329-11|LN-BD1329||France|658[0n]
Thymelicus sylvestris|LENOA1328-11|LN-BD1328||France|658[0n]
Thymelicus sylvestris|GWORR704-10|BC ZSM Lep 32200||Germany|658[0n]
Thymelicus sylvestris|LEATH784-14|TLMF Lep 15996||Italy|658[0n]
Thymelicus sylvestris|LEATH760-14|TLMF Lep 15972||Italy|658[0n]
Thymelicus sylvestris|LEATG413-14|TLMF Lep 14200||Italy|658[0n]
Thymelicus sylvestris|LEATG412-14|TLMF Lep 14199||Italy|658[0n]
Thymelicus sylvestris|LEATI054-15|TLMF Lep 17439||Austria|658[0n]
Thymelicus sylvestris|EZRMN293-08|RVcoll.08-M474||Romania|658[0n]
Thymelicus sylvestris|EZRMN292-08|RVcoll.08-M409||Romania|658[0n]
Thymelicus sylvestris|EZRMN291-08|RVcoll.08-M397||Romania|658[0n]
Thymelicus sylvestris|EULEP4686-16|RVcoll10A939||Bulgaria|658[0n]
Thymelicus sylvestris|EULEP4687-16|RVcoll10A963||Bulgaria|658[0n]
Thymelicus sylvestris|EULEP4697-16|RVcoll15I724||Austria|658[0n]
Thymelicus sylvestris|EULEP686-15|RVcoll.11-J902||Bulgaria|658[0n]
Thymelicus sylvestris|EULEP2368-15|RVcoll.14-V062||Ukraine|658[0n]
Thymelicus sylvestris|GBLAA1055-15|BC ZSM Lep 86561||Germany|658[0n]
Thymelicus sylvestris|EULEP4696-16|RVcoll15I358||Austria|632[0n]
Thymelicus sylvestris|EZROM555-08|RV-07-D570|Genit. examined|Romania|652[0n]
Thymelicus sylvestris|EZRMN296-08|RVcoll.08-M596||Romania|658[0n]
Thymelicus sylvestris|EZRMN294-08|RVcoll.08-M502||Romania|658[0n]
Thymelicus sylvestris|EZROM691-08|RV-07-D904||Romania|658[0n]
Thymelicus sylvestris|EZROM558-08|RV-06-V657|Genit. examined|Romania|658[0n]
Thymelicus sylvestris|FBLMU266-09|BC ZSM Lep 25776||Germany|658[0n]
Thymelicus sylvestris|LEATG033-14|TLMF Lep 13820||Austria|658[0n]
Thymelicus acteon|WMB513-11|RVcoll.11-H600|Sicily|Italy|658[0n]
Thymelicus acteon|WMB497-11|RVcoll.11-H547|Sicily|Italy|658[0n]
Thymelicus acteon|WMB2940-14|RVcoll.12-M341|Sicily|Italy|658[0n]
Thymelicus acteon|WMB3097-14|RVcoll.LD-2588|Sicily|Italy|658[0n]
Thymelicus acteon|WMB2417-13|RVcoll.10-C648|Sicily|Italy|658[0n]
Thymelicus acteon|BIBSA1741-16|11-H346||Italy|658[0n]
Thymelicus acteon|BIBSA1306-15|11-Y097|Sicily|Italy|620[0n]
Thymelicus acteon|WMB1280-13|RVcoll.12-M388|Sicily|Italy|631[0n]
Thymelicus acteon|WMB3013-14|RVcoll.12-R091|Sicily|Italy|658[0n]
Thymelicus acteon|WMB2915-14|RVcoll.11-H983|Sicily|Italy|658[0n]
Thymelicus acteon|EZSPM1023-12|Rvcoll.12-M773||Spain|658[0n]
Thymelicus acteon|WMB3708-14|RVcoll.12-L609||Portugal|658[0n]
Thymelicus acteon|EZSPM950-12|Rvcoll.12-M691||Spain|641[0n]
Thymelicus acteon|EZSPM962-12|Rvcoll.12-M713||Spain|653[0n]
Thymelicus acteon|OXB969-15|15-A919||Italy|658[0n]
Thymelicus acteon|BIBSA1421-15|15-N091||Italy|658[0n]
Thymelicus acteon|OXB971-15|15-A921||Italy|658[0n]
Thymelicus acteon|OXB970-15|15-A920||Italy|658[0n]
Thymelicus acteon|WMB2249-13|RVcoll.12-R392|Elba|Italy|658[0n]
Thymelicus acteon|EULEP1532-15|RVcoll.14-G487||Greece|658[0n]
Thymelicus acteon|EULEP719-15|RVcoll.12-N785|Lesvos|Greece|658[0n]
Thymelicus acteon|EULEP718-15|RVcoll.12-N784|Lesvos|Greece|658[0n]
Thymelicus acteon|EULEP1001-15|RVcoll.14-G722||Greece|658[0n]
Thymelicus acteon|EULEP1656-15|RVcoll.14-G866||Greece|658[0n]
Thymelicus acteon|EULEP1755-15|RVcoll.14-H412||Greece|658[0n]
Thymelicus acteon|EULEP1771-15|RVcoll.14-H495||Greece|658[0n]
Thymelicus acteon|WMB4818-14|RVcoll. 14-I365||Italy|613[0n]
Thymelicus acteon|PHLAF632-11|TLMF Lep 05802||Greece|658[0n]
Thymelicus acteon|LEATJ1173-16|TLMF Lep 19496||Czech Republic|658[0n]
Thymelicus acteon|WMB3278-14|RVcoll.08-M056||Spain|658[0n]
Thymelicus acteon|EZRMN282-08|RVcoll.08-M549||Romania|658[0n]
Thymelicus acteon|OXB1399-15|Rvcoll_15-M766||France|658[0n]
Thymelicus acteon|OXB1214-15|Rvcoll_15-M640||France|658[0n]
Thymelicus acteon|WMB3774-14|RVcoll.12-N717||Spain|658[0n]
Thymelicus acteon|FBLMW320-10|BC ZSM Lep 37421||Germany|658[0n]
Thymelicus acteon|PHLAA439-09|TLMF Lep 00479||France|658[0n]
Thymelicus acteon|EZRMN283-08|RVcoll.08-M561||Romania|658[0n]
Thymelicus acteon|GWOSK782-11|BC ZSM Lep 48998||Germany|658[0n]
Thymelicus acteon|FBLMU510-09|BC ZSM Lep 27160||Germany|658[0n]
Thymelicus acteon|GWORZ072-10|BC ZSM Lep 30428||Italy|658[0n]
Thymelicus acteon|EZSPC332-09|RVcoll.07-C616||Spain|658[0n]
Thymelicus acteon|EZSPN120-09|RVcoll.06-A293||Spain|658[0n]
Thymelicus acteon|EZSPN267-09|RVcoll.07-W181||Spain|658[0n]
Thymelicus acteon|EZSPN096-09|RVcoll.06-A031||Spain|658[0n]
Thymelicus acteon|EZSPC336-09|RVcoll.08-M693||Spain|658[0n]
Thymelicus acteon|EZSPC333-09|RVcoll.08-P332||Spain|658[0n]
Thymelicus acteon|EZSPM376-09|RVcoll.08-J865||Spain|658[0n]
Thymelicus acteon|EZSPN574-09|RVcoll.08-J121||Portugal|658[0n]
Thymelicus acteon|EZSPM648-12|RVcoll.11-I432||Spain|658[0n]
Thymelicus acteon|EZSPM622-12|RVcoll.11-D897||Spain|658[0n]
Thymelicus acteon|EZSPM589-12|RVcoll.09-V937||Spain|658[0n]
Thymelicus acteon|EZSPM1029-12|Rvcoll.12-M779||Spain|658[0n]
Thymelicus acteon|WMB3339-14|RVcoll.08-P916||Spain|658[0n]
Thymelicus acteon|WMB2870-14|RVcoll.10-C559|Argentario|Italy|658[0n]
Thymelicus acteon|EZSPM923-12|Rvcoll.12-M645||Spain|658[0n]
Thymelicus acteon|EZSPN983-09|RVcoll.08-M023||Spain|658[0n]
Thymelicus acteon|WMB2974-14|RVcoll.12-O068|Argentario|Italy|658[0n]
Thymelicus acteon|WMB3352-14|RVcoll.08-R403||Spain|658[0n]
Thymelicus acteon|WMB3524-14|RVcoll.10-C050||France|658[0n]
Thymelicus acteon|WMB3244-14|RVcoll.08-L187||Spain|658[0n]

Thymelicus acteon|WMB29[illegible]-14|RVcoll.12-[illegible]|Argentario|Italy|658[0n]
Thymelicus acteon|WMB3352-14|RVcoll.08-R403||Spain|658[0n]
Thymelicus acteon|WMB3524-14|RVcoll.10-C050||France|658[0n]
Thymelicus acteon|WMB3244-14|RVcoll.08-L187||Spain|658[0n]
Thymelicus acteon|WMB3221-14|RVcoll.08-J739||Spain|658[0n]
Thymelicus acteon|WMB3122-14|RVcoll.06-A303||Spain|658[0n]
Thymelicus acteon|WMB1070-13|RVcoll.10-C694|Ischia|Italy|658[0n]
Thymelicus acteon|WMB3543-14|RVcoll.11-D679||Spain|658[0n]
Thymelicus acteon|WMB1790-13|RVcoll.12-Q220||France|658[0n]
Thymelicus acteon|WMB1071-13|RVcoll.10-C695|Ischia|Italy|658[0n]
Thymelicus acteon|WMB1820-13|RVcoll.11-I636||France|658[0n]
Thymelicus acteon|WMB1819-13|RVcoll.11-I626||France|658[0n]
Thymelicus acteon|WMB3842-14|RVcoll.10-C525||Italy|658[0n]
Thymelicus acteon|WMB2012-13|RVcoll.12-Q644||Italy|658[0n]
Thymelicus acteon|WMB2201-13|RVcoll.09-X950|Ponza|Italy|658[0n]
Thymelicus acteon|WMB2200-13|RVcoll.09-X949|Ponza|Italy|658[0n]
Thymelicus acteon|WMB2410-13|RVcoll.10-C531|Ischia|Italy|658[0n]
Thymelicus acteon|WMB2406-13|RVcoll.09-X952|Ponza|Italy|658[0n]
Thymelicus acteon|WMB2412-13|RVcoll.10-C547||Italy|658[0n]
Thymelicus acteon|WMB2411-13|RVcoll.10-C546||Italy|658[0n]
Thymelicus acteon|WMB2462-13|RVcoll.11-H349||Italy|658[0n]
Thymelicus acteon|WMB3807-14|RVcoll.07-D880||Italy|658[0n]
Thymelicus acteon|WMB4498-14|RVcoll.14-E174||Italy|658[0n]
Thymelicus acteon|WMB4138-14|RVcoll.13-S646||Italy|658[0n]
Thymelicus acteon|WMB4017-14|RVcoll.12-R437||Italy|658[0n]
Thymelicus acteon|WMB4006-14|RVcoll.12-Q657||Italy|658[0n]
Thymelicus acteon|WMB661-11|RVcoll.11-I355|Argentario|Italy|658[0n]
Thymelicus acteon|WMB4434-14|RVcoll.14-B530||Portugal|658[0n]
Thymelicus acteon|BIBSA640-15|RVcoll. 15-A803||Italy|658[0n]
Thymelicus acteon|BIBSA1848-17|RVcoll16C726||Italy|658[0n]
Thymelicus acteon|BIBSA963-15|15-C306||Italy|658[0n]
Thymelicus acteon|BIBSA652-15|RVcoll. 15-A815||Italy|658[0n]
Thymelicus acteon|BIBSA1400-15|15-N005||Italy|658[0n]
Thymelicus acteon|BIBSA1382-15|15-M975||Italy|658[0n]
Thymelicus acteon|BIBSA1235-15|15-N078||Italy|658[0n]
Thymelicus acteon|EULEP1192-15|RVcoll.14-F465||Bulgaria|658[0n]
Thymelicus acteon|EULEP082-14|RVcoll.08-P968||Romania|658[0n]
Thymelicus acteon|EULEP081-14|RVcoll.08-P967||Romania|658[0n]
Thymelicus acteon|EULEP080-14|RVcoll.08-P966||Romania|658[0n]
Thymelicus acteon|EULEP076-14|RVcoll.08-M569.1||Romania|658[0n]
Thymelicus acteon|EULEP075-14|RVcoll.08-M562.1||Romania|658[0n]
Thymelicus acteon|EULEP5075-16|RVcoll16J230||Germany|658[0n]
Thymelicus acteon|WMB5273-14|RVcoll.14-A589||Italy|658[0n]
Thymelicus acteon|WMB5161-14|RVcoll.12-R425||Italy|658[0n]
Thymelicus acteon|WMB4970-14|RVcoll. 14-I517||Italy|658[0n]
Thymelicus acteon|WMB4925-14|RVcoll.14-I472||Italy|658[0n]
Thymelicus acteon|WMB5382-14|RVcoll.14-J841||France|658[0n]
Thymelicus acteon|WMB5355-14|RVcoll.14-J698||France|658[0n]
Thymelicus acteon|WMB4881-14|RVcoll.14-I428||Italy|658[0n]
Thymelicus acteon|WMB4871-14|RVcoll.14-I418||Italy|658[0n]
Thymelicus acteon|WMB4846-14|RVcoll. 14-I393||Italy|658[0n]
Thymelicus acteon|WMB4823-14|RVcoll. 14-I370||Italy|658[0n]
Thymelicus acteon|WMB4773-14|RVcoll. 14-I320||Italy|658[0n]
Thymelicus acteon|WMB4772-14|RVcoll. 14-I319||Italy|658[0n]
Thymelicus acteon|BIBSA1668-16|15-C787||Italy|658[0n]
Thymelicus acteon|BIBSA945-15|16-A036||Italy|658[0n]
Thymelicus acteon|GWORA2883-15|BC ZSM Lep 86151||Germany|658[0n]
Thymelicus acteon|GBLAB142-13|BC ZSM Lep 75768||Germany|658[0n]
Thymelicus acteon|OXB983-15|15-A933||Italy|658[0n]
Thymelicus acteon|OXB1021-15|15-A971||Italy|658[0n]
Thymelicus acteon|OXB1052-15|15-A597||Italy|658[0n]
Thymelicus acteon|OXB1096-15|15-A629||Italy|658[0n]
Thymelicus acteon|OXB244-15|OXB-TGS-737||United Kingdom|658[0n]
Thymelicus acteon|OXB245-15|OXB-TGS-738||United Kingdom|658[0n]
Thymelicus acteon|OXB246-15|OXB-TGS-739||United Kingdom|658[0n]
Thymelicus acteon|OXB1149-15|Rvcoll_15-N103||Italy|658[0n]
Thymelicus acteon|GBLAA1341-15|BC ZSM Lep 87037||Germany|658[0n]
Thymelicus acteon|OXB1215-15|Rvcoll_15-M641||France|658[0n]
Thymelicus acteon|ABOLD653-17|TLMF Lep 21729||Austria|658[0n]
Thymelicus acteon|EZSPC335-09|RVcoll.08-M683||Spain|641[0n]
Thymelicus acteon|EZSPC334-09|RVcoll.08-P364|Genit. examined|Spain|658[0n]
Thymelicus acteon|BIBSA754-15|LEP-SS-00255||Italy|618[0n]
Thymelicus acteon|WMB2742-13|RVcoll.10-C612||Italy|614[0n]
Thymelicus acteon|BIBSA1612-16|15-C374||Italy|620[0n]
Thymelicus acteon|BIBSA1659-16|15-C734||Italy|634[0n]
Thymelicus acteon|PHLAH861-12|TLMF Lep 08665||France|634[0n]
Thymelicus acteon|ABOLD044-16|TLMF Lep 21122||Austria|658[0n]
Thymelicus hyrax|EULEP1314-15|RVcoll.14-F772||Greece|658[0n]
Thymelicus hyrax|EULEP1315-15|RVcoll.14-F773||Greece|658[0n]
Thymelicus hyrax|EULEP1316-15|RVcoll.14-F774||Greece|658[0n]
Thymelicus hyrax|EULEP3151-15|RVcoll.14-O284|Samos|Greece|611[0n]
Thymelicus hyrax|EULEP1311-15|RVcoll.14-F768||Greece|658[0n]
Thymelicus hyrax|EULEP1308-15|RVcoll.14-F761||Greece|658[0n]
Thymelicus hyrax|EULEP3150-15|RVcoll.14-O283|Samos|Greece|658[0n]
Thymelicus hyrax|EULEP678-15|RVcoll.11-J529|Symi|Greece|658[0n]
Thymelicus hyrax|EULEP677-15|RVcoll.11-J528|Symi|Greece|658[0n]
Thymelicus lineola|BIBSA1565-16|11-H986|Sicily|Italy|658[0n]
Thymelicus lineola|BIBSA1755-16|11-H768|Sicily|Italy|617[0n]
Thymelicus lineola|BIBSA1738-16|LD-2934|Sicily|Italy|658[0n]
Thymelicus lineola|WMB1281-13|RVcoll.12-M389|Sicily|Italy|658[0n]
Thymelicus lineola|WMB564-11|RVcoll.11-H764|Sicily|Italy|658[0n]
Thymelicus lineola|WMB1228-13|RVcoll.11-H290|Sicily|Italy|658[0n]
Thymelicus lineola|WMB2808-13|RVcoll.12-M390|Sicily|Italy|658[0n]
Thymelicus lineola|OXB228-15|OXB-TGS-464||United Kingdom|658[0n]
Thymelicus lineola|EULEP650-15|RVcoll.11-J198||France|658[0n]
Thymelicus lineola|EULEP4668-16|RVcoll10B803||France|658[0n]
Thymelicus lineola|BIBSA628-15|RVcoll. 15-A791||Italy|658[0n]
Thymelicus lineola|WMB653-11|RVcoll.11-I244||Italy|658[0n]
Thymelicus lineola|WMB3889-14|RVcoll.12-O854||France|658[0n]
Thymelicus lineola|WMB1998-13|RVcoll.12-Q352||France|658[0n]
Thymelicus lineola|OXB1329-15|Rvcoll_15-M601||France|658[0n]
Thymelicus lineola|BIBSA1850-17|RVcoll16C728||Italy|658[0n]
Thymelicus lineola|WMB5234-14|RVcoll.13-U080||Italy|658[0n]

Thymelicus lineola|OXB1329-15|Rvcoll_15-M601||France|658[0n]
Thymelicus lineola|BIBSA1850-17|RVcoll16C728||Italy|658[0n]
Thymelicus lineola|WMB5234-14|RVcoll.13-U080||Italy|658[0n]
Thymelicus lineola|EULEP4680-16|RVcoll15J573||France|658[0n]
Thymelicus lineola|EZSPC339-09|RVcoll.08-P388||Spain|658[0n]
Thymelicus lineola|BIBSA019-14|LEP-SS-00019||Italy|658[0n]
Thymelicus lineola|WMB2601-13|RVcoll.12-R424||Italy|621[0n]
Thymelicus lineola|WMB870-13|RVcoll.09-T589||Italy|658[0n]
Thymelicus lineola|EZSPC341-09|RVcoll.07-C668||Spain|658[0n]
Thymelicus lineola|OXB353-15|RVcoll.14-N067||Italy|658[0n]
Thymelicus lineola|OXB718-15|13-U108||Italy|658[0n]
Thymelicus lineola|BIBSA961-15|13-U211||Italy|658[0n]
Thymelicus lineola|BIBSA018-14|LEP-SS-00018||Italy|632[0n]
Thymelicus lineola|EULEP4683-16|RVcoll15G152||France|636[0n]
Thymelicus lineola|WMB2034-13|RVcoll.12-Q744||Italy|658[0n]
Thymelicus lineola|WMB1836-13|RVcoll.11-I842||France|658[0n]
Thymelicus lineola|EULEP2121-15|RVcoll.14-J892||France|658[0n]
Thymelicus lineola|EULEP4670-16|RVcoll15C331||Italy|637[0n]
Thymelicus lineola|OXB1409-15|Rvcoll_15-M783||France|658[0n]
Thymelicus lineola|OXB1383-15|Rvcoll_15-M748||France|658[0n]
Thymelicus lineola|OXB1382-15|Rvcoll_15-M746||France|658[0n]
Thymelicus lineola|OXB1287-15|Rvcoll_15-M150||France|658[0n]
Thymelicus lineola|OXB1284-15|Rvcoll_15-M146||France|658[0n]
Thymelicus lineola|OXB1193-15|Rvcoll_15-M606||France|658[0n]
Thymelicus lineola|OXB231-15|OXB-TGS-495||United Kingdom|658[0n]
Thymelicus lineola|OXB227-15|OXB-TGS-462||United Kingdom|658[0n]
Thymelicus lineola|OXB212-15|OXB-TGS-264||United Kingdom|658[0n]
Thymelicus lineola|OXB211-15|OXB-TGS-260||United Kingdom|658[0n]
Thymelicus lineola|BIBSA1643-16|15-C581||Italy|658[0n]
Thymelicus lineola|BIBSA614-15|LEP-SS-00234||Italy|658[0n]
Thymelicus lineola|BIBSA613-15|LEP-SS-00233||Italy|658[0n]
Thymelicus lineola|BIBSA1376-15|15-M938||Italy|658[0n]
Thymelicus lineola|BIBSA1014-15|15-C136||Italy|658[0n]
Thymelicus lineola|EULEP2332-15|RVcoll.14-V017||Denmark|658[0n]
Thymelicus lineola|EULEP636-15|RVcoll.11-J082||Switzerland|658[0n]
Thymelicus lineola|EULEP4682-16|RVcoll15G116||France|658[0n]
Thymelicus lineola|EULEP4681-16|RVcoll15G055||France|658[0n]
Thymelicus lineola|EULEP4679-16|RVcoll15J271||Switzerland|658[0n]
Thymelicus lineola|EULEP4673-16|RVcoll15H323||Switzerland|658[0n]
Thymelicus lineola|EULEP4672-16|RVcoll15H210||Switzerland|658[0n]
Thymelicus lineola|EULEP4669-16|RVcoll14W470||Italy|658[0n]
Thymelicus lineola|WMB5380-14|RVcoll.14-J792||France|658[0n]
Thymelicus lineola|WMB4824-14|RVcoll. 14-I371||Italy|658[0n]
Thymelicus lineola|WMB4313-14|RVcoll.14-A471||Italy|658[0n]
Thymelicus lineola|WMB4277-14|RVcoll.14-A227||Italy|658[0n]
Thymelicus lineola|WMB3812-14|RVcoll.07-E029||Italy|658[0n]
Thymelicus lineola|WMB1853-13|RVcoll.11-I980||France|658[0n]
Thymelicus lineola|WMB1581-13|RVcoll.12-O544|Sardinia|Italy|658[0n]
Thymelicus lineola|WMB2835-13|RVcoll.12-O577|Sardinia|Italy|658[0n]
Thymelicus lineola|WMB2820-13|RVcoll.12-O071||Italy|658[0n]
Thymelicus lineola|WMB2976-14|RVcoll.12-O072|Argentario|Italy|658[0n]
Thymelicus lineola|WMB2975-14|RVcoll.12-O069|Argentario|Italy|658[0n]
Thymelicus lineola|WMB238-11|RVcoll.09-T586||Italy|658[0n]
Thymelicus lineola|EZSPC340-09|RVcoll.07-C667||Spain|658[0n]
Thymelicus lineola|EZSPC338-09|RVcoll.08-L488||Spain|658[0n]
Thymelicus lineola|FBLMW323-10|BC ZSM Lep 37424||Germany|658[0n]
Thymelicus lineola|PHLAJ195-14|KLM Lep 01240||Italy|658[0n]
Thymelicus lineola|PHLAA645-09|TLMF Lep 00685||France|658[0n]
Thymelicus lineola|PHLAW033-13|TLMF Lep 09830||Austria|658[0n]
Thymelicus lineola|WMB4509-14|RVcoll.14-E197||Spain|658[0n]
Thymelicus lineola|EZSPC736-10|RVcoll.08-J031||Spain|658[0n]
Thymelicus lineola|EZSPN992-09|RVcoll.08-M052||Spain|658[0n]
Thymelicus lineola|EZSPN991-09|RVcoll.08-M051||Spain|658[0n]
Thymelicus lineola|EZSPC1359-10|RVcoll.09-V942||Spain|658[0n]
Thymelicus lineola|EZSPM896-12|Rvcoll.12-M618||Spain|615[1n]
Thymelicus lineola|EZSPC789-10|RVcoll.08-M050||Spain|658[0n]
Thymelicus lineola|EZSPN239-09|RVcoll.07-W093||Spain|658[0n]
Thymelicus lineola|EZSPN238-09|RVcoll.07-W088||Spain|658[0n]
Thymelicus lineola|EZSPC823-10|RVcoll.08-P725||Spain|658[0n]
Thymelicus lineola|EZSPC718-10|RVcoll.08-H333||Spain|658[0n]
Thymelicus lineola|EZSPC704-10|RVcoll.07-W098||Spain|658[0n]
Thymelicus lineola|EZSPM893-12|Rvcoll.12-M615||Spain|634[0n]
Thymelicus lineola|EZSPN236-09|RVcoll.07-W086||Spain|658[0n]
Thymelicus lineola|GBLAA1110-15|BC ZSM Lep 86616||Germany|658[0n]
Thymelicus lineola|EZSPC337-09|RVcoll.08-L487|Genit. examined|Spain|658[0n]
Thymelicus lineola|EULEP4678-16|RVcoll15J219||Switzerland|658[0n]
Thymelicus lineola|LEATJ1169-16|TLMF Lep 19492||Czech Republic|658[0n]
Thymelicus lineola|GBLAA1382-15|BC ZSM Lep 87078||Germany|658[0n]
Thymelicus lineola|EULEP1008-15|RVcoll.14-E767||Romania|658[0n]
Thymelicus lineola|EULEP4984-16|RVcoll16I662||Poland|658[0n]
Thymelicus lineola|EULEP1257-15|RVcoll.14-F635||Greece|658[0n]
Thymelicus lineola|EULEP788-15|RVcoll.12-Z219||Sweden|658[0n]
Thymelicus lineola|EZROM595-08|RV-07-C308||Romania|658[1n]
Thymelicus lineola|EZRMN289-08|RVcoll.08-M432||Romania|658[0n]
Thymelicus lineola|EZRMN288-08|RVcoll.08-M428||Romania|658[0n]
Thymelicus lineola|EZRMN287-08|RVcoll.08-M426||Romania|658[0n]
Thymelicus lineola|EULEP4894-16|RVcoll16J597||Russia|658[0n]
Thymelicus lineola|LEFIJ743-10|MM17368||Finland|658[0n]
Thymelicus lineola|EULEP1176-15|RVcoll.14-F403||Bulgaria|658[0n]
Thymelicus lineola|EULEP042-14|RVcoll.08-H019||Romania|658[0n]
Thymelicus lineola|EULEP4671-16|RVcoll15G996||Switzerland|658[0n]
Thymelicus lineola|EULEP551-15|RVcoll.08-L318||Denmark|658[0n]
Thymelicus lineola|EULEP4694-16|RVcoll15H726||Switzerland|638[0n]
Thymelicus lineola|EULEP4674-16|RVcoll15H728||Switzerland|658[0n]
Thymelicus lineola|LEATH672-14|TLMF Lep 15884||Italy|658[0n]
Thymelicus lineola|PHLAF638-11|TLMF Lep 05808||Switzerland|658[0n]
Thymelicus lineola|LEATG034-14|TLMF Lep 13821||Austria|658[0n]
Thymelicus lineola|ABOLD072-16|TLMF Lep 21150||Austria|658[0n]
Thymelicus lineola|LEATG027-14|TLMF Lep 13814||Austria|658[0n]
Thymelicus lineola|LEATG410-14|TLMF Lep 14197||Italy|658[0n]
Thymelicus lineola|LEATG028-14|TLMF Lep 13815||Austria|658[0n]
Thymelicus lineola|GWORL458-09|BC ZSM Lep 22360||Germany|658[0n]
Thymelicus lineola|LEFIG536-10|MM14671||Finland|658[0n]

[illegible]
Thymelicus lineola|LEATG028-14|TLMF Lep 13815||Austria|658[0n]
Thymelicus lineola|GWORL458-09|BC ZSM Lep 22360||Germany|658[0n]
Thymelicus lineola|LEFIG536-10|MM14671||Finland|658[0n]
Thymelicus lineola|FBLMU440-09|BC ZSM Lep 27090||Germany|658[0n]
Thymelicus lineola|LEFIA1394-10|MM03333||Finland|658[0n]
Thymelicus lineola|EZROM553-08|RV-06-M868||Romania|658[0n]
Thymelicus lineola|EULEP4666-16|RVcoll10A949||Bulgaria|658[0n]
Thymelicus lineola|EULEP4667-16|RVcoll10A968||Bulgaria|658[0n]
Thymelicus lineola|EULEP4675-16|RVcoll15H907||Italy|658[0n]
Thymelicus lineola|EULEP4676-16|RVcoll15I120||Italy|658[0n]
Thymelicus lineola|EULEP4677-16|RVcoll15I483||Austria|658[0n]
Thymelicus lineola|EULEP4685-16|RVcoll15Q092||Russia|658[0n]
Thymelicus lineola|EULEP762-15|RVcoll.12-R720||Romania|658[0n]
Thymelicus lineola|EULEP1033-15|RVcoll.14-E878||Serbia|658[0n]
Thymelicus lineola|EULEP1119-15|RVcoll.14-F208||Serbia|658[0n]
Thymelicus lineola|EULEP868-15|RVcoll.14-C352||Greece|658[0n]
Thymelicus lineola|EULEP972-15|RVcoll.14-D063||Bulgaria|658[0n]
Thymelicus lineola|EULEP988-15|RVcoll.14-D232||Austria|658[0n]
Thymelicus lineola|EULEP1930-15|RVcoll.14-B896||Bosnia and Herzegovina|658[0n]
Thymelicus lineola|EULEP2009-15|RVcoll.14-I798||Poland|658[0n]
Thymelicus lineola|EULEP2176-15|RVcoll.14-N211||Greece|658[0n]
Thymelicus lineola|BIBSA1168-15|15-M191||Italy|658[0n]
Thymelicus lineola|BIBSA1198-15|15-M290||Italy|658[0n]
Thymelicus lineola|BIBSA936-15|16-A027||Italy|658[0n]
Thymelicus lineola|BIBSA432-15|RVcoll.14-I100||Italy|658[0n]
Thymelicus lineola|BIBSA282-15|RVcoll.14-E082||Italy|658[0n]
Thymelicus lineola|GBLAD447-14|BC ZSM Lep 77973||Germany|658[0n]
Thymelicus lineola|LEFIJ4738-16|ZMBSU-02121||Belarus|658[0n]
Thymelicus lineola|LEASS513-17|TLMF Lep 22161||Austria|658[0n]
Thymelicus lineola|OXB620-15|14-O009||Italy|658[0n]
Thymelicus lineola|OXB759-15|13-U495||Italy|658[0n]
Thymelicus lineola|EULEP1551-15|RVcoll.14-G538||Greece|615[0n]
Thymelicus lineola|EULEP1271-15|RVcoll.14-F668||Greece|658[0n]
Thymelicus lineola|EZROM554-08|RV-06-M920||Romania|652[0n]
Thymelicus lineola|BIBSA280-15|RVcoll.14-E080||Italy|640[1n]
Thymelicus lineola|EULEP5021-16|RVcoll16I941||Slovakia|658[0n]
Thymelicus lineola|EULEP4684-16|RVcoll15Q091||Russia|635[0n]
Thymelicus lineola|BIBSA279-15|RVcoll.14-E079||Italy|638[0n]
Thymelicus lineola|BIBSA431-15|RVcoll.14-I099||Italy|634[0n]
Thymelicus lineola|PHLAF294-11|TLMF Lep 05464||Macedonia|658[0n]
Thymelicus lineola|LEATG411-14|TLMF Lep 14198||Italy|610[1n]
Erynnis marloyi|EULEP1374-15|RVcoll.14-F969||Greece|658[0n]
Erynnis marloyi|EULEP1768-15|RVcoll.14-H485||Greece|658[0n]
Erynnis marloyi|EULEP1813-15|RVcoll.14-H801||Greece|658[0n]
Erynnis marloyi|EULEP1333-15|RVcoll.14-F817||Greece|658[0n]
Erynnis marloyi|EULEP725-15|RVcoll.12-N807|Lesvos|Greece|658[0n]
Erynnis marloyi|EULEP724-15|RVcoll.12-N806|Lesvos|Greece|658[0n]
Erynnis marloyi|EULEP4000-16|RVcoll14A982||Bulgaria|658[0n]
Erynnis tages|WMB4030-14|RVcoll.12-Z150||Ireland|658[0n]
Erynnis tages|OXB248-15|OXB-TGS-789||United Kingdom|658[0n]
Erynnis tages|OXB1445-16|OXB-TGS-1219||United Kingdom|658[0n]
Erynnis tages|EZSPC1139-10|RVcoll.08-P728||Spain|658[0n]
Erynnis tages|EZROM720-08|RV-06-G448||Spain|658[0n]
Erynnis tages|BIBSA753-15|LEP-SS-00254||Italy|658[0n]
Erynnis tages|OXB1371-15|Rvcoll_15-M734||France|658[0n]
Erynnis tages|OXB1269-15|Rvcoll_15-M128||France|658[0n]
Erynnis tages|OXB1356-15|Rvcoll_15-M708||France|658[0n]
Erynnis tages|EULEP1195-15|RVcoll.14-F472||Bulgaria|658[0n]
Erynnis tages|EULEP2460-15|RVcoll.14-V285||Switzerland|658[0n]
Erynnis tages|BIBSA1360-15|15-M864||Italy|658[0n]
Erynnis tages|LON959-12|NHMO Lep2011.019||Norway|658[1n]
Erynnis tages|WMB1840-13|RVcoll.11-I886||France|658[0n]
Erynnis tages|BIBSA769-15|LEP-SS-00280||Italy|658[0n]
Erynnis tages|GBLAB141-13|BC ZSM Lep 75767||Germany|614[0n]
Erynnis tages|LEATJ1230-16|TLMF Lep 19553||Hungary|658[0n]
Erynnis tages|WMB3581-14|RVcoll.11-E030||Spain|658[0n]
Erynnis tages|WMB3467-14|RVcoll.10-A624||France|614[0n]
Erynnis tages|WMB1694-13|RVcoll.12-P569||France|624[0n]
Erynnis tages|EULEP1621-15|RVcoll.14-G709||Greece|614[0n]
Erynnis tages|GBLAA1342-15|BC ZSM Lep 87038||Germany|633[0n]
Erynnis tages|WMB5197-14|RVcoll.13-T912||Italy|658[0n]
Erynnis tages|WMB5190-14|RVcoll.13-T776||Italy|658[0n]
Erynnis tages|WMB5160-14|RVcoll.12-R194||Italy|658[0n]
Erynnis tages|WMB5137-14|RVcoll.07-E072||Italy|658[0n]
Erynnis tages|WMB5064-14|RVcoll. 14-L195||Italy|658[0n]
Erynnis tages|WMB633-11|RVcoll.11-I122||Italy|658[0n]
Erynnis tages|WMB2214-13|RVcoll.11-Y028||Italy|658[0n]
Erynnis tages|WMB2051-13|RVcoll.12-Q793||Italy|658[0n]
Erynnis tages|EZSPC1135-10|RVcoll.08-P718||Spain|658[0n]
Erynnis tages|WMB197-11|RVcoll.07-W041||France|658[0n]
Erynnis tages|EZSPC1284-10|RVcoll.030908SF85||Spain|658[0n]
Erynnis tages|EZSPM771-12|Rvcoll. 050111LP29||Spain|658[0n]
Erynnis tages|EZSPN334-09|RVcoll.08-H261||Spain|658[0n]
Erynnis tages|EZSPC398-09|RVcoll.08-J365||Spain|658[0n]
Erynnis tages|EZSPM354-09|RVcoll.08-R067||Spain|658[0n]
Erynnis tages|EZSPN641-09|RVcoll.08-J773||Spain|658[0n]
Erynnis tages|EZSPN538-09|RVcoll.08-J024||Spain|658[0n]
Erynnis tages|EZSPC401-09|RVcoll.08-P315||Spain|658[0n]
Erynnis tages|EZSPN230-09|RVcoll.07-W034||Spain|658[0n]
Erynnis tages|EZSPN421-09|RVcoll.08-H576||Spain|658[0n]
Erynnis tages|EZSPN111-09|RVcoll.06-A263||Spain|658[0n]
Erynnis tages|EZROM976-08|RVcoll.08-M350||Romania|658[0n]
Erynnis tages|EZROM974-08|RVcoll.08-M242||Romania|658[0n]
Erynnis tages|EZROM973-08|RVcoll.07-D303||Romania|658[0n]
Erynnis tages|EZROM975-08|RVcoll.08-M309||Romania|658[0n]
Erynnis tages|GWOSK781-11|BC ZSM Lep 48997||Germany|658[0n]
Erynnis tages|GWOSK780-11|BC ZSM Lep 48996||Germany|658[0n]
Erynnis tages|FBLMU509-09|BC ZSM Lep 27159||Germany|658[0n]
Erynnis tages|FBLMX129-11|BC ZSM Lep 37610||Germany|658[0n]
Erynnis tages|FBLMX127-11|BC ZSM Lep 37608||Germany|658[0n]
Erynnis tages|WMB5267-14|RVcoll.14-A510||Italy|658[0n]
Erynnis tages|WMB5213-14|RVcoll.13-T953||Italy|658[0n]
[illegible]

Erynnis tages|FBLMX127-11|BC ZSM Lep 37608||Germany|658[0n]
Erynnis tages|WMB5267-14|RVcoll.14-A510||Italy|658[0n]
Erynnis tages|WMB5213-14|RVcoll.13-T953||Italy|658[0n]
Erynnis tages|WMB5291-14|RVcoll.14-I586||France|658[0n]
Erynnis tages|WMB5284-14|RVcoll.14-A839||Italy|658[0n]
Erynnis tages|EULEP4734-16|RVcoll16G504||Sweden|658[0n]
Erynnis tages|EULEP4002-16|RVcoll15G922||Switzerland|658[0n]
Erynnis tages|EULEP4996-16|RVcoll16I742||Poland|658[0n]
Erynnis tages|EULEP5048-16|RVcoll16J047||Czech Republic|658[0n]
Erynnis tages|EULEP1428-15|RVcoll.14-G108||Greece|658[0n]
Erynnis tages|EULEP836-15|RVcoll.14-C010||Sweden|658[0n]
Erynnis tages|EULEP1942-15|RVcoll.14-B916||Bosnia and Herzegovina|658[0n]
Erynnis tages|EULEP1805-15|RVcoll.14-H744||Greece|658[0n]
Erynnis tages|EULEP2353-15|RVcoll.14-V042||Ukraine|658[0n]
Erynnis tages|EULEP1981-15|RVcoll.14-H931||France|658[0n]
Erynnis tages|BIBSA1157-15|15-L116||Italy|658[0n]
Erynnis tages|BIBSA1127-15|15-L927||Italy|658[0n]
Erynnis tages|BIBSA1860-17|RVcoll16C748||Italy|658[0n]
Erynnis tages|BIBSA1222-15|15-L956||Italy|658[0n]
Erynnis tages|BIBSA010-14|LEP-SS-00010||Italy|658[0n]
Erynnis tages|BIBSA1861-17|RVcoll16C749||Italy|658[0n]
Erynnis tages|OXB777-15|14-A738||Italy|658[0n]
Erynnis tages|OXB593-15|14-N982||Italy|658[0n]
Erynnis tages|OXB1127-15|15-A660||Italy|658[0n]
Erynnis tages|OXB729-15|13-U192||Italy|658[0n]
Erynnis tages|OXB922-15|15-A566||Italy|658[0n]
Erynnis tages|OXB348-15|RVcoll.14-N062||Italy|658[0n]
Erynnis tages|OXB1010-15|15-A960||Italy|658[0n]
Erynnis tages|OXB320-15|RVcoll.14-N034||Italy|658[0n]
Erynnis tages|EZSPC399-09|RVcoll.06-G586||Spain|612[0n]
Erynnis tages|OXB1108-15|15-A641||Italy|658[2n]
Erynnis tages|BIBSA219-15|RVcoll.14-E007||Italy|605[0n]
Erynnis tages|EULEP4001-16|RVcoll10A754||Estonia|622[0n]
Erynnis tages|WMB3293-14|RVcoll.08-M967||France|614[0n]
Erynnis tages|EZSPC400-09|RVcoll.08-M905||Spain|622[0n]
Erynnis tages|EZROM179-08|RV-07-D240||Romania|646[0n]
Erynnis tages|EZROM178-08|RV-07-D061||Romania|646[0n]
Erynnis tages|EZROM177-08|RV-06-K549||Romania|646[0n]
Erynnis tages|EZROM176-08|RV-06-K547||Romania|646[0n]
Erynnis tages|GWORZ061-10|BC ZSM Lep 30417||Italy|645[0n]
Erynnis tages|FBLMW329-10|BC ZSM Lep 37430||Germany|658[0n]
Erynnis tages|ODOPE302-11|BC ZSM Lep 50703||Germany|658[0n]
Erynnis tages|LEATD093-13|TLMF Lep 12740||Austria|658[0n]
Erynnis tages|LEATG003-14|TLMF Lep 13790||Austria|658[0n]
Erynnis tages|PHLAH455-12|TLMF Lep 08274||Austria|658[0n]
Erynnis tages|ABOLD081-16|TLMF Lep 21159||Austria|658[0n]
Erynnis tages|LEATD017-13|TLMF Lep 12664||Italy|658[0n]
Erynnis tages|LEATD463-13|TLMF Lep 13110||Italy|658[0n]
Pyrgus malvoides|EULEP2162-15|RVcoll.14-K075||Switzerland|658[0n]
Pyrgus malvoides|BIBSA1570-16|11-I079||Italy|658[0n]
Pyrgus malvoides|WMB1958-13|RVcoll.11-I081|Sicily|Italy|650[0n]
Pyrgus malvoides|WMB1276-13|RVcoll.12-M329|Sicily|Italy|658[0n]
Pyrgus malvoides|WMB2816-13|RVcoll.12-M578|Sicily|Italy|658[0n]
Pyrgus malvoides|WMB2946-14|RVcoll.12-M421|Sicily|Italy|658[0n]
Pyrgus malvoides|WMB2944-14|RVcoll.12-M404|Sicily|Italy|658[0n]
Pyrgus malvoides|WMB076-11|RVcoll.LD-3043|Sicily|Italy|658[0n]
Pyrgus malvoides|EZSPN159-09|RVcoll.06-J009||Spain|658[0n]
Pyrgus malvoides|EZSPN1063-11|Rvcoll.08-P732||Spain|658[0n]
Pyrgus malvoides|WMB3154-14|RVcoll.07-W077||Spain|658[0n]
Pyrgus malvoides|EZSPN420-09|RVcoll.08-H567||Spain|658[0n]
Pyrgus malvoides|EZSPN121-09|RVcoll.06-A295||Spain|658[0n]
Pyrgus malvoides|WMB5378-14|RVcoll.14-J785||France|658[0n]
Pyrgus malvoides|WMB5311-14|RVcoll.14-I715||France|658[0n]
Pyrgus malvoides|WMB5315-14|RVcoll.14-I749||France|658[0n]
Pyrgus malvoides|WMB5308-14|RVcoll.14-I677||France|643[0n]
Pyrgus malvoides|WMB3921-14|RVcoll.12-P484||France|620[0n]
Pyrgus malvoides|WMB3915-14|RVcoll.12-P378||France|658[0n]
Pyrgus malvoides|WMB1656-13|RVcoll.12-P081||France|658[0n]
Pyrgus malvoides|WMB3779-14|RVcoll.12-N766||Spain|658[0n]
Pyrgus malvoides|WMB3120-14|RVcoll.06-A291||Spain|658[0n]
Pyrgus malvoides|WMB3337-14|RVcoll.08-P906||Spain|658[0n]
Pyrgus malvoides|WMB3336-14|RVcoll.08-P903||Spain|658[0n]
Pyrgus malvoides|EZSPN1049-11|Rvcoll.08-L423||Spain|658[0n]
Pyrgus malvoides|EZSPN1033-11|Rvcoll.06-G457|Genit. examined|Spain|658[0n]
Pyrgus malvoides|EZSPN242-09|RVcoll.07-W113||Spain|658[0n]
Pyrgus malvoides|EZSPN213-09|RVcoll.07-F068||Spain|658[0n]
Pyrgus malvoides|EZSPN118-09|RVcoll.06-A290||Spain|658[0n]
Pyrgus malvoides|WMB2377-13|RVcoll.07-D800||Italy|658[0n]
Pyrgus malvoides|WMB4163-14|RVcoll.13-S721||Italy|658[0n]
Pyrgus malvoides|BCLEP284-17|LEP-SS-00729||Italy|658[0n]
Pyrgus malvoides|OXB770-15|14-A385||Italy|658[0n]
Pyrgus malvoides|WMB4927-14|RVcoll.14-I474||Italy|658[0n]
Pyrgus malvoides|WMB5147-14|RVcoll.11-J589||Italy|658[0n]
Pyrgus malvoides|BIBSA1037-15|15-C206||Italy|658[0n]
Pyrgus malvoides|WMB4859-14|RVcoll.14-I406||Italy|638[0n]
Pyrgus malvoides|WMB4230-14|RVcoll.13-T779||Italy|658[0n]
Pyrgus malvoides|WMB1809-13|RVcoll.11-I359||Italy|658[0n]
Pyrgus malvoides|BIBSA327-15|RVcoll.14-E132||Italy|633[0n]
Pyrgus malvoides|WMB1839-13|RVcoll.11-I883||France|658[0n]
Pyrgus malvoides|WMB2851-13|RVcoll.10-C147||France|658[0n]
Pyrgus malvoides|FBLMX214-11|BC ZSM Lep 50425||Germany|658[0n]
Pyrgus malvoides|WMB2350-13|RVcoll.12-M555||Italy|658[0n]
Pyrgus malvoides|PHLAF635-11|TLMF Lep 05805||Switzerland|658[0n]
Pyrgus malvoides|LEATD466-13|TLMF Lep 13113||Italy|658[0n]
Pyrgus malvoides|BIBSA1859-17|RVcoll16C746||Italy|658[0n]
Pyrgus malvoides|BIBSA697-15|RVcoll. 11-I235||Italy|658[0n]
Pyrgus malvoides|BIBSA669-15|RVcoll. 11-I236||Italy|658[0n]
Pyrgus malvoides|WMB3484-14|RVcoll.10-B620.1||France|601[0n]
Pyrgus malvoides|OXB855-15|14-U816||Italy|658[0n]
Pyrgus malvoides|EULEP4617-16|RVcoll15G711||Italy|658[0n]
Pyrgus malvoides|LEATJ1342-16|TLMF Lep 19665||Austria|658[0n]
Pyrgus malvoides|ABOLB021-15|TLMF Lep 17026||Austria|658[0n]
Pyrgus malvoides|WMB4249-14|RVcoll.13-U053||Italy|658[0n]

Pyrgus malvoides|LEATJ1342-16|TLMF Lep 19665||Austria|658[0n]
Pyrgus malvoides|ABOLB021-15|TLMF Lep 17026||Austria|658[0n]
Pyrgus malvoides|WMB4249-14|RVcoll.13-U053||Italy|658[0n]
Pyrgus malvoides|WMB4225-14|RVcoll.13-T763||Italy|658[0n]
Pyrgus malvoides|WMB2226-13|RVcoll.11-Y062||Italy|658[0n]
Pyrgus malvoides|WMB2225-13|RVcoll.11-Y061||Italy|658[0n]
Pyrgus malvoides|WMB1977-13|RVcoll.11-I232||Italy|658[0n]
Pyrgus malvoides|WMB1768-13|RVcoll.12-Q026||France|658[0n]
Pyrgus malvoides|WMB892-13|RVcoll.09-X827||Italy|658[0n]
Pyrgus malvoides|WMB858-13|RVcoll.09-T271||France|658[0n]
Pyrgus malvoides|WMB249-11|RVcoll.09-V227||France|658[0n]
Pyrgus malvoides|LEATD380-13|TLMF Lep 13027||Italy|658[0n]
Pyrgus malvoides|LEATD094-13|TLMF Lep 12741||Austria|658[0n]
Pyrgus malvoides|PHLAW036-13|TLMF Lep 09833||Austria|658[0n]
Pyrgus malvoides|WMB4817-14|RVcoll. 14-I364||Italy|658[0n]
Pyrgus malvoides|WMB5082-14|RVcoll. 14-L213||Italy|658[0n]
Pyrgus malvoides|EULEP4618-16|RVcoll15G958||Switzerland|658[0n]
Pyrgus malvoides|WMB5214-14|RVcoll.13-T961||Italy|658[0n]
Pyrgus malvoides|BIBSA1233-15|15-N045||Italy|658[0n]
Pyrgus malvoides|BIBSA1129-15|15-L938||Italy|658[0n]
Pyrgus malvoides|BIBSA1673-16|15-C824||Italy|658[0n]
Pyrgus malvoides|BIBSA1064-15|14-V320||Italy|658[0n]
Pyrgus malvoides|BIBSA322-15|RVcoll.14-E124||Italy|658[0n]
Pyrgus malvoides|BIBSA341-15|RVcoll.14-I009||Italy|658[0n]
Pyrgus malvoides|BIBSA1634-16|LEP-SS-00393||Italy|658[0n]
Pyrgus malvoides|BIBSA011-14|LEP-SS-00011||Italy|658[0n]
Pyrgus malvoides|BIBSA012-14|LEP-SS-00012||Italy|658[0n]
Pyrgus malvoides|LEASS569-17|TLMF Lep 22217||Austria|658[0n]
Pyrgus malvoides|LEASS567-17|TLMF Lep 22215||Austria|658[0n]
Pyrgus malvoides|LEASS568-17|TLMF Lep 22216||Austria|658[0n]
Pyrgus malvoides|OXB600-15|14-N989||Italy|658[0n]
Pyrgus malvoides|OXB1120-15|15-A653||Italy|658[0n]
Pyrgus malvoides|OXB887-15|15-A529||Italy|658[0n]
Pyrgus malvoides|OXB955-15|15-A905||Italy|658[0n]
Pyrgus malvoides|BIBSA147-15|RVcoll.14-I159||Italy|651[0n]
Pyrgus malvoides|EULEP4619-16|RVcoll15G290||Switzerland|638[0n]
Pyrgus malvoides|WMB813-13|RVcoll.07-E128||Italy|630[0n]
Pyrgus malvoides|PHLAI514-13|TLMF Lep 09076||Austria|627[0n]
Pyrgus malvoides|LEATJ1341-16|TLMF Lep 19664||Austria|658[0n]
Pyrgus malvae|EULEP4615-16|RVcoll10B419||Bulgaria|658[0n]
Pyrgus malvae|EULEP1968-15|RVcoll.14-H832||Serbia|658[0n]
Pyrgus malvae|EULEP1056-15|RVcoll.14-E941||Serbia|614[0n]
Pyrgus malvae|EZROM529-08|RV-07-D133||Romania|652[0n]
Pyrgus malvae|EULEP4809-16|RVcoll16H701||Belgium|658[0n]
Pyrgus malvae|EULEP4616-16|RVcoll15P028||Belarus|658[0n]
Pyrgus malvae|FBLMU505-09|BC ZSM Lep 27155||Germany|658[0n]
Pyrgus malvae|EZRMN239-08|RVcoll.08-M229||Romania|658[0n]
Pyrgus malvae|EZRMN242-08|RVcoll.08-M376||Romania|658[0n]
Pyrgus malvae|EZRMN243-08|RVcoll.08-M414||Romania|658[0n]
Pyrgus malvae|EZROM528-08|RV-06-K575||Romania|658[0n]
Pyrgus malvae|EZROM530-08|RV-07-D217||Romania|658[0n]
Pyrgus malvae|EZROM656-08|RV-07-D522||Romania|658[0n]
Pyrgus malvae|EULEP1864-15|RVcoll.13-U280|Genit. examined|Italy|658[0n]
Pyrgus malvae|EULEP1865-15|RVcoll.13-U283||Italy|658[0n]
Pyrgus malvae|LEASS461-17|TLMF Lep 21919||Austria|645[0n]
Pyrgus malvae|EZRMN241-08|RVcoll.08-M352||Romania|644[0n]
Pyrgus malvae|LEATC581-13|TLMF Lep 12563||Austria|616[0n]
Pyrgus malvae|ABOLD414-16|TLMF Lep 21566||Austria|658[0n]
Pyrgus malvae|LEATC580-13|TLMF Lep 12562||Austria|658[0n]
Pyrgus malvae|EULEP1344-15|RVcoll.14-F858||Greece|658[0n]
Pyrgus malvae|LEFIB013-10|MM00029||Finland|658[0n]
Pyrgus malvae|FBLMT879-09|BC ZSM Lep 25439||Germany|658[0n]
Pyrgus malvae|FBLMT877-09|BC ZSM Lep 25437||Germany|658[0n]
Pyrgus malvae|GBLAA1391-15|BC ZSM Lep 87087||Germany|658[0n]
Pyrgus malvae|GBLAA1378-15|BC ZSM Lep 87074||Germany|636[0n]
Pyrgus malvae|GBLAA1322-15|BC ZSM Lep 87208||Germany|658[0n]
Pyrgus malvae|GBLAA1321-15|BC ZSM Lep 87207||Germany|658[0n]
Pyrgus malvae|GBLAA1278-15|BC ZSM Lep 87164||Germany|658[0n]
Pyrgus malvae|GBLAB160-13|BC ZSM Lep 75786||France|658[0n]
Pyrgus malvae|GBLAD976-14|BC ZSM Lep 84392||Germany|658[0n]
Pyrgus malvae|EULEP2354-15|RVcoll.14-V043||Ukraine|658[0n]
Pyrgus malvae|EULEP1662-15|RVcoll.14-G878||Greece|658[0n]
Pyrgus malvae|EULEP1581-15|RVcoll.14-G608||Greece|658[0n]
Pyrgus malvae|EULEP838-15|RVcoll.14-C013||Sweden|658[0n]
Pyrgus malvae|EULEP4740-16|RVcoll16G578||Sweden|658[0n]
Pyrgus malvae|EZRMN240-08|RVcoll.08-M244||Romania|658[0n]
Pyrgus malvae|LEFIA1335-10|MM00034||Finland|658[0n]
Pyrgus malvae|GWORA2490-09|BC ZSM Lep 30702||Germany|658[0n]
Pyrgus malvae|FBLMX254-11|BC ZSM Lep 50465||Germany|658[0n]
Pyrgus malvae|GWOR3938-09|BC ZSM Lep 21222||Germany|658[0n]
Pyrgus malvae|GWOSR002-11|BC ZSM Lep 44038||Germany|658[0n]
Pyrgus malvae|GWOSR001-11|BC ZSM Lep 44037||Germany|658[0n]
Pyrgus malvae|LEFIB319-10|MM00850||Finland|658[0n]
Pyrgus malvae|FBLMW325-10|BC ZSM Lep 37426||Germany|658[0n]
Pyrgus malvae|GWORR699-10|BC ZSM Lep 32195||Germany|658[0n]
Pyrgus malvae|GWORR698-10|BC ZSM Lep 32194||Germany|658[0n]
Pyrgus malvae|PHLAW035-13|TLMF Lep 09832||Austria|658[0n]
Pyrgus malvae|LEATI071-15|TLMF Lep 17456||Austria|658[0n]
Pyrgus malvae|ABOLA895-15|TLMF Lep 16855||Austria|658[0n]
Pyrgus malvae|PHLAH459-12|TLMF Lep 08278||Austria|658[0n]
Pyrgus cinarae|EZSPC067-09|RVcoll.08-R496||Russia|658[0n]
Pyrgus cinarae|EZSPM482-10|RVcoll.09-X606||Russia|601[0n]
Pyrgus cinarae|EZSPM485-10|RVcoll.09-X609||Russia|658[0n]
Pyrgus cinarae|EZSPM488-10|RVcoll.09-X612||Russia|658[0n]
Pyrgus cinarae|EZSPM489-10|RVcoll.09-X613||Russia|658[0n]
Pyrgus cinarae|EZSPM490-10|RVcoll.09-X614||Russia|658[0n]
Pyrgus cinarae|EZSPM492-10|RVcoll.09-X616||Russia|658[0n]
Pyrgus cinarae|EZSPM493-10|RVcoll.09-X617||Russia|658[0n]
Pyrgus cinarae|EZSPM491-10|RVcoll.09-X615||Russia|650[0n]
Pyrgus cinarae|EZSPM486-10|RVcoll.09-X610||Russia|627[0n]
Pyrgus cinarae|EZSPM483-10|RVcoll.09-X607||Russia|646[0n]
Pyrgus cinarae|EZSPM481-10|RVcoll.09-X605||Russia|658[0n]
Pyrgus cinarae|EZSPC167-09|RVcoll.07-W023||Russia|658[0n]

Pyrgus cinarae|EZSPM480-10|RVcoll.09-X610||Russia|627[0n]
Pyrgus cinarae|EZSPM483-10|RVcoll.09-X607||Russia|646[0n]
Pyrgus cinarae|EZSPM481-10|RVcoll.09-X605||Russia|658[0n]
Pyrgus cinarae|EZSPC167-09|RVcoll.07-W023||Russia|658[0n]
Pyrgus cinarae|EZSPC068-09|RVcoll.08-R497||Russia|658[0n]
Pyrgus cinarae|EZSPC066-09|RVcoll.08-R495||Russia|658[0n]
Pyrgus cinarae|EZSPM494-10|RVcoll.09-X618||Russia|658[0n]
Pyrgus cinarae|EZSPM479-10|RVcoll.09-X603||Ukraine|658[0n]
Pyrgus cinarae|EZSPN008-09|RVcoll.08-D412.1||Greece|658[0n]
Pyrgus cinarae|EZSPC612-09|RVcoll.07-C539|ex. ovo|Greece|658[2n]
Pyrgus cinarae|EZSPM478-10|RVcoll.09-X602||Ukraine|605[0n]
Pyrgus cinarae|EULEP3127-15|RVcoll.14-O260||Macedonia|658[0n]
Pyrgus cinarae|EULEP1622-15|RVcoll.14-G710||Greece|658[0n]
Pyrgus cinarae|EULEP1516-15|RVcoll.14-G438||Greece|658[0n]
Pyrgus cinarae|EULEP1502-15|RVcoll.14-G387||Greece|658[0n]
Pyrgus cinarae|EULEP883-15|RVcoll.14-C473||Greece|658[0n]
Pyrgus cinarae|EULEP1185-15|RVcoll.14-F450||Bulgaria|658[0n]
Pyrgus cinarae|EULEP4612-16|RVcoll10B329||Bulgaria|658[0n]
Pyrgus cinarae|EZSPC614-09|RVcoll.07-C541|ex. ovo|Greece|658[0n]
Pyrgus cinarae|EZSPC613-09|RVcoll.07-C540|ex. ovo|Greece|658[0n]
Pyrgus cinarae|EZSPC611-09|RVcoll.07-C538|ex. ovo|Greece|658[0n]
Pyrgus cinarae|EZSPC069-09|RVcoll.08-R498||Russia|658[0n]
Pyrgus cinarae|EZSPC027-09|RVcoll.08-D411||Greece|658[0n]
Pyrgus cinarae|EZSPM389-09|Rvcoll.09-T116||Spain|658[0n]
Pyrgus cinarae|EZSPM390-09|Rvcoll.09-T117||Spain|658[0n]
Pyrgus cinarae|EZSPM381-09|Rvcoll.09-T107||Spain|658[0n]
Pyrgus cinarae|EZSPM383-09|Rvcoll.09-T109||Spain|658[0n]
Pyrgus cinarae|EZSPM476-10|RVcoll.09-X600||Spain|632[0n]
Pyrgus cinarae|EZSPM387-09|Rvcoll.09-T113||Spain|628[0n]
Pyrgus cinarae|EZSPM386-09|Rvcoll.09-T112||Spain|658[0n]
Pyrgus cinarae|EZSPM385-09|Rvcoll.09-T111||Spain|658[0n]
Pyrgus cinarae|EZSPM384-09|Rvcoll.09-T110||Spain|658[0n]
Pyrgus cinarae|EZSPC010-09|RVcoll.07-W241||Spain|658[0n]
Pyrgus cinarae|EZSPC009-09|RVcoll.07-W220||Spain|658[0n]
Pyrgus cinarae|EZSPN040-09|RVcoll.09-T100||Spain|658[0n]
Pyrgus cinarae|EZSPM591-12|RVcoll.10-A037||Spain|658[0n]
Pyrgus armoricanus|BIBSA1574-16|11-I098|Sicily|Italy|658[0n]
Pyrgus armoricanus|BIBSA689-15|RVcoll. 11-H990|Sicily|Italy|658[0n]
Pyrgus armoricanus|WMB5159-14|RVcoll.12-R110|Sicily|Italy|658[0n]
Pyrgus armoricanus|WMB5145-14|RVcoll.11-H991|Sicily|Italy|658[0n]
Pyrgus armoricanus|WMB550-11|RVcoll.11-H740|Sicily|Italy|658[0n]
Pyrgus armoricanus|WMB549-11|RVcoll.11-H739|Sicily|Italy|658[0n]
Pyrgus armoricanus|WMB1950-13|RVcoll.11-H930|Sicily|Italy|658[0n]
Pyrgus armoricanus|EULEP2410-15|RVcoll.14-V128||Ukraine|658[0n]
Pyrgus armoricanus|WMB3281-14|RVcoll.08-M087||Spain|658[0n]
Pyrgus armoricanus|WMB3494-14|RVcoll.10-B693|Genit. examined|France|658[0n]
Pyrgus armoricanus|WMB3276-14|RVcoll.08-M018||Spain|658[0n]
Pyrgus armoricanus|EZSPC291-09|RVcoll.07-E638||Spain|658[0n]
Pyrgus armoricanus|LEASS742-17|TLMF Lep 22390||Austria|658[0n]
Pyrgus armoricanus|WMB4893-14|RVcoll.14-I440||Italy|658[0n]
Pyrgus armoricanus|BIBSA014-14|LEP-SS-00014||Italy|658[0n]
Pyrgus armoricanus|EZROM654-08|RV-07-D247|Genit. examined|Romania|658[0n]
Pyrgus armoricanus|EZSPC138-09|RVcoll.07-E360|Genit. examined|Romania|658[0n]
Pyrgus armoricanus|EZSPC131-09|RVcoll.07-D265|Genit. examined|Romania|658[0n]
Pyrgus armoricanus|BIBSA1628-16|15-C457||Italy|658[0n]
Pyrgus armoricanus|EZSPC174-09|RVcoll.07-W038||France|658[0n]
Pyrgus armoricanus|EZSPC179-09|RVcoll.07-W069||France|658[0n]
Pyrgus armoricanus|LEASS830-17|TLMF Lep 22478||Austria|658[0n]
Pyrgus armoricanus|LEASS831-17|TLMF Lep 22479||Austria|658[0n]
Pyrgus armoricanus|BIBSA755-15|LEP-SS-00256||Italy|658[0n]
Pyrgus armoricanus|EZROM524-08|RV-07-C111||Romania|658[0n]
Pyrgus armoricanus|EZROM523-08|RV-07-C110||Romania|658[0n]
Pyrgus armoricanus|EZROM522-08|RV-07-D144||Romania|658[0n]
Pyrgus armoricanus|EZRMN426-09|RVcoll.09-V676|Genit. examined|Romania|658[0n]
Pyrgus armoricanus|EZRMN424-09|RVcoll.09-V673||Romania|658[0n]
Pyrgus armoricanus|EZRMN417-09|RVcoll.07-D147|Genit. examined|Romania|658[0n]
Pyrgus armoricanus|WMB3265-14|RVcoll.08-L788||Spain|658[0n]
Pyrgus armoricanus|WMB3255-14|RVcoll.08-L443||Spain|658[0n]
Pyrgus armoricanus|WMB3075-14|RVcoll.14-D942|Elba|Italy|658[0n]
Pyrgus armoricanus|WMB3074-14|RVcoll.14-D941|Elba|Italy|658[0n]
Pyrgus armoricanus|EZRMN416-09|RVcoll.07-D140|Genit. examined|Romania|658[0n]
Pyrgus armoricanus|EZRMN414-09|RVcoll.07-D128|Genit. examined|Romania|658[0n]
Pyrgus armoricanus|EZROM567-08|RV-07-D921||Romania|658[0n]
Pyrgus armoricanus|EZRMN415-09|RVcoll.07-D131|Genit. examined|Romania|658[0n]
Pyrgus armoricanus|WMB5328-14|RVcoll.14-J136|Corsica|France|658[0n]
Pyrgus armoricanus|WMB662-11|RVcoll.11-I356|Argentario|Italy|658[0n]
Pyrgus armoricanus|WMB4842-14|RVcoll. 14-I389||Italy|658[0n]
Pyrgus armoricanus|WMB288-11|RVcoll.08-H787||France|658[0n]
Pyrgus armoricanus|WMB4332-14|RVcoll.14-A716||Italy|658[0n]
Pyrgus armoricanus|WMB4485-14|RVcoll.14-D932||Italy|658[0n]
Pyrgus armoricanus|WMB3848-14|RVcoll.10-C720||Italy|658[0n]
Pyrgus armoricanus|WMB1590-13|RVcoll.12-O592|Sardinia|Italy|658[0n]
Pyrgus armoricanus|WMB1575-13|RVcoll.12-O522|Sardinia|Italy|658[0n]
Pyrgus armoricanus|WMB1574-13|RVcoll.12-O521|Sardinia|Italy|658[0n]
Pyrgus armoricanus|WMB3073-14|RVcoll.14-D940|Elba|Italy|658[0n]
Pyrgus armoricanus|WMB3447-14|RVcoll.10-A223||Spain|658[0n]
Pyrgus armoricanus|WMB3376-14|RVcoll.09-V229||France|658[0n]
Pyrgus armoricanus|WMB3375-14|RVcoll.09-V216||France|658[0n]
Pyrgus armoricanus|WMB3338-14|RVcoll.08-P913||Spain|658[0n]
Pyrgus armoricanus|EZSPC293-09|RVcoll.08-J387||Spain|658[0n]
Pyrgus armoricanus|EZSPC171-09|RVcoll.07-W033||Spain|658[0n]
Pyrgus armoricanus|EZSPC109-09|RVcoll.06-G571||Spain|658[0n]
Pyrgus armoricanus|EZRMN418-09|RVcoll.07-D174|Genit. examined|Romania|658[0n]
Pyrgus armoricanus|EZRMN413-09|RVcoll.07-D126|Genit. examined|Romania|658[0n]
Pyrgus armoricanus|EZRMN233-08|RVcoll.08-M256||Romania|658[0n]
Pyrgus armoricanus|EZRMN232-08|RVcoll.07-D534||Romania|658[0n]
Pyrgus armoricanus|EZSPM329-09|RVcoll.08-R062||Spain|658[0n]
Pyrgus armoricanus|EZSPC292-09|RVcoll.06-A103||Spain|658[0n]
Pyrgus armoricanus|EZSPC147-09|RVcoll.07-F300|Genit. examined|Spain|658[0n]
Pyrgus armoricanus|EZSPC126-09|RVcoll.07-D120||Romania|658[0n]
Pyrgus armoricanus|EZSPC106-09|RVcoll.06-A298||Spain|658[0n]
Pyrgus armoricanus|EZSPC1161-10|RVcoll.09-T031||Spain|658[0n]
Pyrgus armoricanus|EZSPC1160-10|RVcoll.09-T030||Spain|658[0n]

Pyrgus armoricanus|EZSPC106-09|RVcoll.06-A298||Spain|658[0n]
Pyrgus armoricanus|EZSPC1161-10|RVcoll.09-T031||Spain|658[0n]
Pyrgus armoricanus|EZSPC1160-10|RVcoll.09-T030||Spain|658[0n]
Pyrgus armoricanus|WMB202-11|RVcoll.07-W070||France|658[0n]
Pyrgus armoricanus|EZRMN231-08|RVcoll.07-D455||Romania|658[0n]
Pyrgus armoricanus|GWOSK770-11|BC ZSM Lep 48986||Germany|658[0n]
Pyrgus armoricanus|GWORO990-09|BC ZSM Lep 32297||Italy|658[0n]
Pyrgus armoricanus|GWORZ066-10|BC ZSM Lep 30422||Italy|658[0n]
Pyrgus armoricanus|GWORL256-09|BC ZSM Lep 18662||Germany|658[0n]
Pyrgus armoricanus|GWORL248-09|BC ZSM Lep 18654||Germany|658[0n]
Pyrgus armoricanus|EULEP1037-15|RVcoll.14-E898||Serbia|658[0n]
Pyrgus armoricanus|EULEP4592-16|RVcoll15J142||Switzerland|658[0n]
Pyrgus armoricanus|BIBSA1629-16|15-C458||Italy|658[0n]
Pyrgus armoricanus|EULEP1963-15|RVcoll.14-G808||Romania|658[0n]
Pyrgus armoricanus|EULEP1962-15|RVcoll.14-G807||Romania|658[0n]
Pyrgus armoricanus|EULEP823-15|RVcoll.14-E910||Serbia|658[0n]
Pyrgus armoricanus|BIBSA707-15|RVcoll. 14-A095||Italy|658[0n]
Pyrgus armoricanus|BIBSA114-15|RVcoll.14-D544||Italy|658[0n]
Pyrgus armoricanus|BIBSA1627-16|15-C456||Italy|658[0n]
Pyrgus armoricanus|BIBSA950-15|16-A041||Italy|658[0n]
Pyrgus armoricanus|BIBSA015-14|LEP-SS-00015||Italy|658[0n]
Pyrgus armoricanus|BIBSA013-14|LEP-SS-00013||Italy|658[0n]
Pyrgus armoricanus|EZSPC023-09|RVcoll.07-W288||Italy|658[0n]
Pyrgus armoricanus|EZSPC022-09|RVcoll.07-W287||Italy|658[0n]
Pyrgus armoricanus|EZSPC136-09|RVcoll.07-E129||Italy|658[0n]
Pyrgus armoricanus|EZSPC135-09|RVcoll.07-E085|Genit. examined|Italy|658[0n]
Pyrgus armoricanus|EZSPC615-09|RVcoll.07-C558|ex. ovo|Germany|658[0n]
Pyrgus armoricanus|EZSPM396-09|Rvcoll.09-V368|Genit. examined|Bulgaria|658[0n]
Pyrgus armoricanus|LEASS744-17|TLMF Lep 22392||Austria|658[0n]
Pyrgus armoricanus|OXB885-15|15-A527||Italy|658[0n]
Pyrgus armoricanus|OXB931-15|15-A575||Italy|658[0n]
Pyrgus armoricanus|BCLEP101-17|LEP-SS-00552||Italy|658[0n]
Pyrgus armoricanus|OXB994-15|15-A944||Italy|658[0n]
Pyrgus armoricanus|OXB1081-15|15-A614||Italy|658[0n]
Pyrgus armoricanus|WMB5241-14|RVcoll.13-U093||Italy|658[0n]
Pyrgus armoricanus|ABOLD652-17|TLMF Lep 21728||Austria|658[0n]
Pyrgus armoricanus|EZSPC112-09|RVcoll.06-J012||Spain|658[0n]
Pyrgus armoricanus|ABOLD629-17|TLMF Lep 21705||Austria|634[0n]
Pyrgus armoricanus|GBLAB136-13|BC ZSM Lep 75762||Germany|647[0n]
Pyrgus armoricanus|BIBSA107-15|RVcoll.14-D535||Italy|644[0n]
Pyrgus armoricanus|EULEP1044-15|RVcoll.14-E911||Serbia|614[0n]
Pyrgus armoricanus|WMB1685-13|RVcoll.12-P519||France|631[0n]
Pyrgus armoricanus|EULEP2396-15|RVcoll.14-V100||Ukraine|621[0n]
Pyrgus armoricanus|LEATH756-14|TLMF Lep 15968||Italy|623[1n]
Pyrgus armoricanus|ABOLD029-16|TLMF Lep 21107||Austria|658[0n]
Pyrgus armoricanus|EULEP2476-15|RVcoll.14-V331|Genit. examined|Ukraine|658[0n]
Pyrgus armoricanus|EULEP4840-16|RVcoll16H746||Ukraine|658[0n]
Pyrgus armoricanus|EULEP1194-15|RVcoll.14-F469||Bulgaria|658[0n]
Pyrgus armoricanus|EULEP1189-15|RVcoll.14-F461||Bulgaria|658[0n]
Pyrgus armoricanus|EULEP1188-15|RVcoll.14-F456||Bulgaria|658[0n]
Pyrgus armoricanus|EZSPC127-09|RVcoll.07-D127|Genit. examined|Romania|658[0n]
Pyrgus armoricanus|EZRMN412-09|RVcoll.07-D034|Genit. examined|Romania|658[0n]
Pyrgus armoricanus|EZSPC130-09|RVcoll.07-D195|Genit. examined|Romania|658[0n]
Pyrgus armoricanus|EULEP894-15|RVcoll.14-C690||Greece|658[0n]
Pyrgus armoricanus|EULEP882-15|RVcoll.14-C472||Greece|658[0n]
Pyrgus armoricanus|EULEP1467-15|RVcoll.14-G230||Greece|658[0n]
Pyrgus armoricanus|EULEP928-15|RVcoll.14-C825||Bulgaria|658[0n]
Pyrgus armoricanus|EZSPM393-09|Rvcoll.09-V365|Genit. examined|Bulgaria|658[0n]
Pyrgus armoricanus|EZSPM394-09|Rvcoll.09-V366|Genit. examined|Greece|658[0n]
Pyrgus armoricanus|EZSPM533-10|RVcoll.09-V952||Bulgaria|622[0n]
Pyrgus armoricanus|EULEP1961-15|RVcoll.14-G795||Romania|658[0n]
Pyrgus armoricanus|EULEP1960-15|RVcoll.14-G791||Romania|658[0n]
Pyrgus armoricanus|EULEP1959-15|RVcoll.14-G782||Romania|658[0n]
Pyrgus armoricanus|EULEP1958-15|RVcoll.14-G763||Romania|658[0n]
Pyrgus armoricanus|EULEP1956-15|RVcoll.14-G754||Romania|658[0n]
Pyrgus armoricanus|EULEP1804-15|RVcoll.14-H742||Greece|658[0n]
Pyrgus armoricanus|EULEP1801-15|RVcoll.14-H700||Greece|658[0n]
Pyrgus armoricanus|EULEP1731-15|RVcoll.14-H263||Greece|658[0n]
Pyrgus armoricanus|EULEP1689-15|RVcoll.14-H026||Greece|658[0n]
Pyrgus armoricanus|EULEP1675-15|RVcoll.14-G941||Greece|658[0n]
Pyrgus armoricanus|EULEP1620-15|RVcoll.14-G699||Greece|658[0n]
Pyrgus armoricanus|EULEP1477-15|RVcoll.14-G258||Greece|658[0n]
Pyrgus armoricanus|EULEP1468-15|RVcoll.14-G231||Greece|658[0n]
Pyrgus armoricanus|EZRMN234-08|RVcoll.08-M419||Romania|658[0n]
Pyrgus carlinae|WMB1629-13|RVcoll.12-O836||France|658[0n]
Pyrgus carlinae|WMB1631-13|RVcoll.12-O840||France|658[0n]
Pyrgus carlinae|WMB1844-13|RVcoll.11-I903||France|658[0n]
Pyrgus carlinae|WMB1847-13|RVcoll.11-I919||France|658[0n]
Pyrgus carlinae|EULEP4604-16|RVcoll15J333||France|658[0n]
Pyrgus carlinae|EULEP4613-16|RVcoll10C067|Genit. examined|France|658[0n]
Pyrgus carlinae|EULEP575-15|RVcoll.09-X690|Genit. examined|Switzerland|658[0n]
Pyrgus carlinae|BIBSA319-15|RVcoll.14-E121||Italy|658[0n]
Pyrgus carlinae|WMB1630-13|RVcoll.12-O837||France|636[0n]
Pyrgus carlinae|EULEP4603-16|RVcoll15J288||Switzerland|637[0n]
Pyrgus carlinae|WMB1622-13|RVcoll.12-O770||France|632[0n]
Pyrgus carlinae|WMB1618-13|RVcoll.12-O751||France|658[0n]
Pyrgus carlinae|WMB1609-13|RVcoll.12-O692|Genit. examined|France|658[0n]
Pyrgus carlinae|WMB1594-13|RVcoll.12-O611||France|658[0n]
Pyrgus carlinae|WMB2672-13|RVcoll.10-B853|Genit. examined|France|658[0n]
Pyrgus carlinae|WMB3523-14|RVcoll.10-C005||France|658[0n]
Pyrgus carlinae|WMB3516-14|RVcoll.10-B907||France|658[0n]
Pyrgus cirsii|EZSPM341-09|RVcoll.08-R018||Spain|658[0n]
Pyrgus cirsii|WMB1828-13|RVcoll.11-I728||France|658[0n]
Pyrgus cirsii|WMB1792-13|RVcoll.12-Q283||France|658[0n]
Pyrgus cirsii|WMB3152-14|RVcoll.07-F091||Spain|658[0n]
Pyrgus cirsii|EZSPC111-09|RVcoll.06-G650||Spain|658[0n]
Pyrgus cirsii|EZSPM181-09|RVcoll.08-R161||Spain|658[0n]
Pyrgus cirsii|EZSPM106-09|RVcoll.08-P443||Spain|658[0n]
Pyrgus cirsii|EZSPM388-09|Rvcoll.09-T115||Spain|658[0n]
Pyrgus cirsii|EZSPM340-09|RVcoll.08-R012||Spain|658[0n]
Pyrgus cirsii|EZSPM339-09|RVcoll.08-R010||Spain|658[0n]
Pyrgus cirsii|EZSPN813-09|RVcoll.08-L605||Spain|658[0n]
Pyrgus cirsii|EZSPN221-09|RVcoll.07-F304|Genit. examined|Spain|658[0n]

[illegible]
Pyrgus cirsii|EZSPM339-09|RVcoll.08-R010||Spain|658[0n]
Pyrgus cirsii|EZSPN813-09|RVcoll.08-L605||Spain|658[0n]
Pyrgus cirsii|EZSPN221-09|RVcoll.07-F304|Genit. examined|Spain|658[0n]
Pyrgus cirsii|EZSPN212-09|RVcoll.07-F067||Spain|658[0n]
Pyrgus cirsii|EZSPN157-09|RVcoll.06-J002||Spain|658[0n]
Pyrgus onopordi|EZSPC1222-10|RVcoll.09-V546||Spain|658[0n]
Pyrgus onopordi|EZSPC1159-10|RVcoll.09-T028||Spain|658[0n]
Pyrgus onopordi|EZSPN336-09|RVcoll.08-H268||Spain|658[0n]
Pyrgus onopordi|EZSPC1208-10|RVcoll.09-V496||Spain|658[0n]
Pyrgus onopordi|EZSPC1209-10|RVcoll.09-V497||Spain|658[0n]
Pyrgus onopordi|EZSPC1004-10|RVcoll.09-V490||Spain|658[0n]
Pyrgus onopordi|EZSPC1024-10|RVcoll.08-J020||Spain|658[0n]
Pyrgus onopordi|EZSPC1025-10|RVcoll.08-J040||Spain|658[0n]
Pyrgus onopordi|EZSPC1026-10|RVcoll.08-J047||Spain|658[0n]
Pyrgus onopordi|WMB203-11|RVcoll.07-W071||France|658[0n]
Pyrgus onopordi|EZSPC1150-10|RVcoll.08-P931||Spain|658[0n]
Pyrgus onopordi|WMB3155-14|RVcoll.07-W081||Spain|658[0n]
Pyrgus onopordi|WMB3184-14|RVcoll.08-H978||Spain|658[0n]
Pyrgus onopordi|WMB3483-14|RVcoll.10-B616||France|658[0n]
Pyrgus onopordi|WMB3541-14|RVcoll.11-D665||Spain|658[0n]
Pyrgus onopordi|WMB3570-14|RVcoll.11-D987||Spain|658[0n]
Pyrgus onopordi|WMB833-13|RVcoll.07-E222||Italy|658[0n]
Pyrgus onopordi|WMB946-13|RVcoll.10-A646||France|658[0n]
Pyrgus onopordi|WMB1055-13|RVcoll.09-X676||Italy|658[0n]
Pyrgus onopordi|WMB1056-13|RVcoll.09-X677||Italy|658[0n]
Pyrgus onopordi|WMB1799-13|RVcoll.12-Q322||France|658[0n]
Pyrgus onopordi|WMB2123-13|RVcoll.12-R209||Italy|658[0n]
Pyrgus onopordi|WMB1726-13|RVcoll.12-P861||France|658[0n]
Pyrgus onopordi|WMB4574-14|RVcoll.140611ZG29||Spain|658[0n]
Pyrgus onopordi|WMB6575-18|RVcoll14A008|Genit. examined|Italy|658[0n]
Pyrgus onopordi|WMB6576-18|RVcoll14A009|Genit. examined|Italy|658[0n]
Pyrgus onopordi|WMB6577-18|RVcoll14A085||Italy|658[0n]
Pyrgus onopordi|WMB6578-18|RVcoll14A086||Italy|658[0n]
Pyrgus onopordi|EULEP4614-16|RVcoll14V896||Italy|658[0n]
Pyrgus onopordi|EULEP259-14|RVcoll.12-Q531||Spain|658[0n]
Pyrgus onopordi|EULEP260-14|RVcoll.12-Q551||Spain|658[0n]
Pyrgus onopordi|EULEP5670-17|RVcoll16A563||Italy|658[0n]
Pyrgus onopordi|EULEP1854-15|RVcoll.13-U047||Italy|658[0n]
Pyrgus onopordi|BIBSA955-15|12-R187||Italy|658[0n]
Pyrgus onopordi|OXB710-15|13-T890||Italy|658[0n]
Pyrgus onopordi|OXB1025-15|15-A975||Italy|658[0n]
Pyrgus onopordi|OXB1147-15|Rvcoll_15-N101||Italy|658[0n]
Pyrgus onopordi|BIBSA658-15|RVcoll. 15-A821||Italy|637[0n]
Pyrgus onopordi|EZSPN292-09|RVcoll.07-W264||Spain|615[3n]
Pyrgus onopordi|EZSPN234-09|RVcoll.07-W078||Spain|658[0n]
Pyrgus onopordi|EZSPN211-09|RVcoll.07-F066||Spain|658[0n]
Pyrgus onopordi|EZSPN102-09|RVcoll.06-A043||Spain|658[0n]
Pyrgus onopordi|GWORZ063-10|BC ZSM Lep 30419||Italy|658[0n]
Pyrgus alveus|EZSPC061-09|RVcoll.08-P545|Genit. examined|Spain|658[0n]
Pyrgus foulquieri|EZSPC036-09|RVcoll.08-H207||Italy|658[0n]
Pyrgus foulquieri|EZSPC037-09|RVcoll.08-H208||Italy|658[0n]
Pyrgus foulquieri|EZSPN009-09|RVcoll.08-H206.1|Genit. examined|Italy|658[0n]
Pyrgus|OXB696-15|12-Q713|alveus/foulquieri|Italy|658[0n]
Pyrgus|EULEP3124-15|RVcoll.14-O257|alveus/foulquieri|Italy|617[0n]
Pyrgus|WMB3148-14|RVcoll.07-E642|alveus/foulquieri|Spain|658[0n]
Pyrgus foulquieri|EZSPC143-09|RVcoll.07-F115|Genit. examined|Spain|658[0n]
Pyrgus foulquieri|EZSPC142-09|RVcoll.07-F114|Genit. examined|Spain|658[0n]
Pyrgus foulquieri|EZSPC141-09|RVcoll.07-F113|Genit. examined|Spain|658[0n]
Pyrgus foulquieri|EZSPC139-09|RVcoll.07-F109|Genit. examined|Spain|658[0n]
Pyrgus foulquieri|EZSPC108-09|RVcoll.06-G535||Spain|658[0n]
Pyrgus foulquieri|EZSPC105-09|RVcoll.06-A287|Genit. examined|Spain|658[0n]
Pyrgus foulquieri|EZSPC104-09|RVcoll.06-A285||Spain|658[0n]
Pyrgus foulquieri|EZSPC103-09|RVcoll.06-A284|Genit. examined|Spain|658[0n]
Pyrgus foulquieri|EZSPC101-09|RVcoll.06-A257|Genit. examined|Spain|658[0n]
Pyrgus foulquieri|EZSPC063-09|RVcoll.08-P547|Genit. examined|Spain|658[0n]
Pyrgus foulquieri|EZSPC062-09|RVcoll.08-P546|Genit. examined|Spain|658[0n]
Pyrgus foulquieri|EZSPC047-09|RVcoll.08-M678|Genit. examined|Spain|658[0n]
Pyrgus foulquieri|EZSPC008-09|RVcoll.07-W207|Genit. examined|Spain|658[0n]
Pyrgus foulquieri|EZSPC007-09|RVcoll.07-W206|Genit. examined|Spain|658[0n]
Pyrgus foulquieri|EZSPC006-09|RVcoll.07-W205|Genit. examined|Spain|658[0n]
Pyrgus alveus|OXB1297-15|Rvcoll_15-M162||France|658[0n]
Pyrgus alveus|EULEP4593-16|RVcoll15G144||France|658[0n]
Pyrgus alveus|EZSPN021-09|RVcoll.09-T025|Genit. examined|Spain|658[0n]
Pyrgus alveus|EZSPN020-09|RVcoll.09-T024|Genit. examined|Spain|658[0n]
Pyrgus alveus|EZSPN019-09|RVcoll.09-T023|Genit. examined|Spain|658[0n]
Pyrgus alveus|EZSPN018-09|RVcoll.09-T022|Genit. examined|Spain|658[0n]
Pyrgus alveus|EZSPN017-09|RVcoll.09-T021|Genit. examined|Spain|652[0n]
Pyrgus alveus|EZSPN073-09|RVcoll.09-V927||Spain|658[0n]
Pyrgus alveus|EZSPN072-09|RVcoll.09-V926||Spain|658[0n]
Pyrgus alveus|EZSPC003-09|RVcoll.07-W190|Genit. examined|Spain|658[0n]
Pyrgus alveus|EZSPC188-09|RVcoll.07-W143||Spain|658[0n]
Pyrgus alveus|EZSPM177-09|RVcoll.08-R120||Spain|658[0n]
Pyrgus alveus|EZSPC005-09|RVcoll.07-W192|Genit. examined|Spain|658[0n]
Pyrgus alveus|EZSPC001-09|RVcoll.07-W187||Spain|658[0n]
Pyrgus alveus|EZSPM382-09|RVcoll.09-T108||Spain|658[0n]
Pyrgus alveus|EZSPN071-09|RVcoll.09-V484|Genit. examined|Spain|658[0n]
Pyrgus alveus|EZSPC117-09|RVcoll.07-C048|Genit. examined|Spain|658[0n]
Pyrgus|WMB3350-14|RVcoll.08-R283|alveus/foulquieri|Spain|658[0n]
Pyrgus alveus|WMB3144-14|RVcoll.07-C416|Genit. examined|Spain|658[0n]
Pyrgus alveus|EZSPC181-09|RVcoll.07-W090|Genit. examined|Spain|658[1n]
Pyrgus alveus|EZSPC180-09|RVcoll.07-W089|Genit. examined|Spain|658[0n]
Pyrgus alveus|EZSPC118-09|RVcoll.07-C049||Spain|658[0n]
Pyrgus alveus|EZSPC115-09|RVcoll.06-V723|Genit. examined|Spain|658[0n]
Pyrgus alveus|EZSPC107-09|RVcoll.06-A314|Genit. examined|Spain|658[0n]
Pyrgus alveus|EZSPC043-09|RVcoll.08-L207||Spain|658[0n]
Pyrgus alveus|EZSPC042-09|RVcoll.08-L184||Spain|658[0n]
Pyrgus alveus|EZSPC116-09|RVcoll.06-V749||Spain|658[0n]
Pyrgus alveus|GWORL218-09|BC ZSM Lep 18624||Germany|658[0n]
Pyrgus alveus|ODOPE256-11|BC ZSM Lep 53127||Germany|658[0n]
Pyrgus alveus|ODOPE258-11|BC ZSM Lep 53129||Germany|658[0n]
Pyrgus alveus|ODOPE257-11|BC ZSM Lep 53128||Germany|658[0n]
Pyrgus alveus|FBLMW333-10|BC ZSM Lep 37434|ID verified by dissection|Germany|658[0n]
[illegible]

Pyrgus alveus|ODOPE258-11|BC ZSM Lep 53129||Germany|658[0n]
Pyrgus alveus|ODOPE257-11|BC ZSM Lep 53128||Germany|658[0n]
Pyrgus alveus|FBLMW333-10|BC ZSM Lep 37434|ID verified by dissection|Germany|658[0n]
Pyrgus alveus|GWORL226-09|BC ZSM Lep 18632||Germany|658[0n]
Pyrgus alveus|GWORL240-09|BC ZSM Lep 18646||Germany|658[0n]
Pyrgus alveus|GWORL238-09|BC ZSM Lep 18644||Germany|658[0n]
Pyrgus alveus|GWORL246-09|BC ZSM Lep 18652||Germany|658[0n]
Pyrgus alveus|GWORL242-09|BC ZSM Lep 18648||Germany|658[0n]
Pyrgus alveus|GWORT475-10|BC ZSM Lep 32161||Germany|658[0n]
Pyrgus alveus|GWOSK772-11|BC ZSM Lep 48988||Germany|658[0n]
Pyrgus alveus|GWOSN700-11|BC ZSM Lep 52906||Germany|658[0n]
Pyrgus alveus|EZRMN227-08|RVcoll.08-M584||Romania|658[0n]
Pyrgus alveus|EZRMN228-08|RVcoll.08-M585||Romania|658[0n]
Pyrgus alveus|EZRMN229-08|RVcoll.08-M586|Genit. examined|Romania|658[0n]
Pyrgus alveus|EZRMN230-08|RVcoll.08-M587|Genit. examined|Romania|658[0n]
Pyrgus alveus|EZROM323-08|RV-08-A001||Romania|658[0n]
Pyrgus alveus|EULEP4582-16|RVcoll15I784||Austria|658[0n]
Pyrgus alveus|EULEP092-14|RVcoll.08-R371||Romania|658[0n]
Pyrgus alveus|EULEP1133-15|RVcoll.14-F265||Serbia|658[0n]
Pyrgus alveus|GWOSN698-11|BC ZSM Lep 52904||Germany|631[0n]
Pyrgus alveus|GWORT473-10|BC ZSM Lep 32159||Germany|614[0n]
Pyrgus alveus|GWORL236-09|BC ZSM Lep 18642||Germany|658[0n]
Pyrgus alveus|GWORL228-09|BC ZSM Lep 18634||Germany|658[0n]
Pyrgus alveus|GWORL224-09|BC ZSM Lep 18630||Germany|658[0n]
Pyrgus alveus|GWORL222-09|BC ZSM Lep 18628||Germany|658[0n]
Pyrgus alveus|GWORL220-09|BC ZSM Lep 18626||Germany|658[0n]
Pyrgus alveus|GWORL216-09|BC ZSM Lep 18622||Germany|658[0n]
Pyrgus alveus|EZSPC041-09|RVcoll.08-H870|Genit. examined|Serbia|658[0n]
Pyrgus alveus|EULEP5129-17|RVcoll16J884||Greece|658[0n]
Pyrgus alveus|EULEP5128-17|RVcoll16J883||Greece|658[0n]
Pyrgus alveus|ABOLD630-17|TLMF Lep 21706||Austria|658[0n]
Pyrgus alveus|EULEP5126-17|RVcoll16J881||Macedonia|658[0n]
Pyrgus alveus|EZSPM534-10|RVcoll.09-V953||Bulgaria|658[0n]
Pyrgus alveus|EZSPM535-10|RVcoll.09-V954||Bulgaria|658[0n]
Pyrgus alveus|EZSPC029-09|RVcoll.08-D512|Genit. examined|Macedonia|658[0n]
Pyrgus alveus|EZSPC040-09|RVcoll.08-H865|Genit. examined|Serbia|658[0n]
Pyrgus alveus|EZSPC149-09|RVcoll.07-F522||Estonia|658[0n]
Pyrgus alveus|EZSPC148-09|RVcoll.07-F521||Estonia|658[0n]
Pyrgus alveus|EZSPM536-10|RVcoll.09-V955||Bulgaria|629[0n]
Pyrgus alveus|EULEP4855-16|RVcoll16H828||Poland|635[0n]
Pyrgus alveus|EULEP4578-16|RVcoll14O334||Bulgaria|658[0n]
Pyrgus alveus|EULEP5086-16|RVcoll16J391||Germany|658[0n]
Pyrgus alveus|GWOSM336-11|BC ZSM Lep 43422||Germany|658[0n]
Pyrgus alveus|FBLMW352-10|BC ZSM Lep 37453||Germany|658[0n]
Pyrgus alveus|FBLMW342-10|BC ZSM Lep 37443||Germany|658[0n]
Pyrgus alveus|FBLMW340-10|BC ZSM Lep 37441||Germany|658[0n]
Pyrgus alveus|FBLMW296-10|BC ZSM Lep 37397||Germany|658[0n]
Pyrgus alveus|FBLMW350-10|BC ZSM Lep 37451||Germany|658[4n]
Pyrgus foulquieri|EZSPC020-09|RVcoll.07-W285|Genit. examined|Italy|607[1n]
Pyrgus alveus|GWORL230-09|BC ZSM Lep 18636||Germany|658[0n]
Pyrgus alveus|FBLMW298-10|BC ZSM Lep 37399||Germany|658[0n]
Pyrgus alveus|FBLMW319-10|BC ZSM Lep 37420||Germany|658[0n]
Pyrgus alveus|FBLMW334-10|BC ZSM Lep 37435|ID verified by dissection|Germany|658[0n]
Pyrgus alveus|FBLMW346-10|BC ZSM Lep 37447||Germany|658[0n]
Pyrgus alveus|FBLMW348-10|BC ZSM Lep 37449||Germany|658[0n]
Pyrgus alveus|FBLMW355-10|BC ZSM Lep 37456||Germany|658[0n]
Pyrgus alveus|GWORL212-09|BC ZSM Lep 18618||Germany|658[0n]
Pyrgus alveus|FBLMX120-11|BC ZSM Lep 37601||Germany|658[0n]
Pyrgus alveus|FBLMX126-11|BC ZSM Lep 37607|ID verfied by dissection|Germany|658[0n]
Pyrgus alveus|FBLMX128-11|BC ZSM Lep 37609||Germany|658[0n]
Pyrgus alveus|FBLMX131-11|BC ZSM Lep 37612||Austria|658[0n]
Pyrgus alveus|ODOPE252-11|BC ZSM Lep 53123||Germany|658[0n]
Pyrgus alveus|ODOPE253-11|BC ZSM Lep 53124||Germany|658[0n]
Pyrgus alveus|ODOPE254-11|BC ZSM Lep 53125||Germany|658[0n]
Pyrgus alveus|ODOPE259-11|BC ZSM Lep 53130||Germany|658[0n]
Pyrgus alveus|ODOPE260-11|BC ZSM Lep 53131||Germany|658[0n]
Pyrgus alveus|GWOSK775-11|BC ZSM Lep 48991||Germany|658[0n]
Pyrgus alveus|GWOSA771-10|BC ZSM Lep 36637||Germany|658[0n]
Pyrgus alveus|FBLMT896-09|BC ZSM Lep 25456||Germany|658[0n]
Pyrgus foulquieri|WMB1713-13|RVcoll.12-P663|Genit. examined|France|658[0n]
Pyrgus foulquieri|WMB1725-13|RVcoll.12-P855|Genit. examined|France|658[0n]
Pyrgus|WMB1733-13|RVcoll.12-P913|alveus/foulquieri|France|658[0n]
Pyrgus foulquieri|BIBSA938-15|16-A029|Genit. examined|Italy|658[0n]
Pyrgus foulquieri|BIBSA939-15|16-A030|Genit. examined|Italy|658[0n]
Pyrgus foulquieri|EZSPC019-09|RVcoll.07-W284|Genit. examined|Italy|658[0n]
Pyrgus foulquieri|EZSPC033-09|RVcoll.08-H204|Genit. examined|Italy|658[0n]
Pyrgus foulquieri|EZSPC034-09|RVcoll.08-H205|Genit. examined|Italy|658[0n]
Pyrgus foulquieri|EZSPC154-09|RVcoll.07-W010|Genit. examined|Italy|658[0n]
Pyrgus foulquieri|EZSPC155-09|RVcoll.07-W011||Italy|658[0n]
Pyrgus foulquieri|EZSPC156-09|RVcoll.07-W012|Genit. examined|Italy|658[0n]
Pyrgus alveus|ABOLD624-17|TLMF Lep 21700||Austria|658[0n]
Pyrgus foulquieri|EZSPC157-09|RVcoll.07-W013|Genit. examined|Italy|658[0n]
Pyrgus foulquieri|OXB1187-15|Rvcoll_16-A074|Genit. examined|Italy|658[0n]
Pyrgus alveus|GWORL214-09|BC ZSM Lep 18620||Germany|622[0n]
Pyrgus alveus|GWORL210-09|BC ZSM Lep 18616||Germany|658[0n]
Pyrgus alveus|GWORL208-09|BC ZSM Lep 18614||Germany|658[0n]
Pyrgus alveus|GWORL206-09|BC ZSM Lep 18612||Germany|658[0n]
Pyrgus alveus|GWORL204-09|BC ZSM Lep 18610||Germany|658[0n]
Pyrgus alveus|GWORL202-09|BC ZSM Lep 18608||Germany|658[0n]
Pyrgus alveus|GWORL200-09|BC ZSM Lep 18606||Germany|658[0n]
Pyrgus alveus|GWORL196-09|BC ZSM Lep 18602||Germany|658[0n]
Pyrgus alveus|EZSPC039-09|RVcoll.08-H830|Genit. examined|Sweden|658[0n]
Pyrgus alveus|EZSPC038-09|RVcoll.08-H826|Genit. examined|Sweden|658[0n]
Pyrgus alveus|LEFID733-10|MM06791||Finland|658[0n]
Pyrgus alveus|LEFIL371-10|MM18681||Finland|658[0n]
Pyrgus warrenensis|EULEP4626-16|RVcoll15J087|Genit. examined|Switzerland|658[0n]
Pyrgus warrenensis|LEASS702-17|TLMF Lep 22350||Austria|658[0n]
Pyrgus alveus|BIBSA1070-15|15-H940||Italy|658[0n]
Pyrgus alveus|EULEP4640-16|RVcoll15H995||Italy|658[0n]
Pyrgus warrenensis|EULEP4639-16|RVcoll15H876||Italy|658[0n]
Pyrgus warrenensis|EULEP4634-16|RVcoll15J071||Switzerland|658[0n]
Pyrgus warrenensis|EULEP4622-16|RVcoll15H808|Genit. examined|Italy|658[0n]
Pyrgus warrenensis|EULEP4590-16|RVcoll14V885||Italy|658[0n]

Pyrgus warrenensis|EULEP4634-16|RVcoll15J071||Switzerland|658[0n]
Pyrgus warrenensis|EULEP4622-16|RVcoll15H808|Genit. examined|Italy|658[0n]
Pyrgus warrenensis|EULEP4590-16|RVcoll14V885||Italy|658[0n]
Pyrgus warrenensis|EULEP4575-16|RVcoll15J060||Switzerland|658[0n]
Pyrgus alveus|LEATD149-13|TLMF Lep 12796||Italy|658[0n]
Pyrgus alveus|GWORT474-10|BC ZSM Lep 32160||Germany|658[0n]
Pyrgus alveus|GWOSZ214-11|BC ZSM Lep 41970||Italy|658[0n]
Pyrgus alveus|GWOSN699-11|BC ZSM Lep 52905||Germany|658[0n]
Pyrgus alveus|OXB674-15|08-L396||Italy|658[0n]
Pyrgus alveus|OXB767-15|14-A225|Genit. examined|Italy|658[0n]
Pyrgus alveus|GWOSN697-11|BC ZSM Lep 52903||Germany|658[0n]
Pyrgus alveus|GWOSN695-11|BC ZSM Lep 52901||Germany|658[0n]
Pyrgus alveus|GWORT472-10|BC ZSM Lep 32158||Germany|658[0n]
Pyrgus alveus|GWORR700-10|BC ZSM Lep 32196||Germany|658[0n]
Pyrgus alveus|LEATJ1339-16|TLMF Lep 19662||Austria|658[0n]
Pyrgus alveus|EULEP5127-17|RVcoll16J882||Macedonia|658[0n]
Pyrgus warrenensis|GWORL193-09|BC ZSM Lep 18599||Germany|658[0n]
Pyrgus alveus|LEATJ1335-16|TLMF Lep 19658||Austria|658[0n]
Pyrgus alveus|LEATG029-14|TLMF Lep 13816||Austria|658[0n]
Pyrgus alveus|LEATJ1333-16|TLMF Lep 19656||Austria|658[0n]
Pyrgus alveus|ABOLA429-14|TLMF Lep 16116||Austria|658[0n]
Pyrgus warrenensis|EZSPC024-09|RVcoll.07-W289||Italy|658[0n]
Pyrgus alveus|EZSPC032-09|RVcoll.08-H203|Genit. examined|Italy|658[0n]
Pyrgus alveus|EZSPM527-10|RVcoll.09-T187|Genit. examined|France|658[0n]
Pyrgus alveus|EZSPC137-09|RVcoll.07-E214|Genit. examined|Italy|658[0n]
Pyrgus alveus|EZSPC158-09|RVcoll.07-W014|Genit. examined|Italy|658[0n]
Pyrgus alveus|EZSPC159-09|RVcoll.07-W015|Genit. examined|Italy|658[0n]
Pyrgus alveus|EZSPC160-09|RVcoll.07-W016|Genit. examined|Italy|658[0n]
Pyrgus alveus|EZSPM529-10|RVcoll.09-T189||France|658[0n]
Pyrgus alveus|LEASS555-17|TLMF Lep 22203||Austria|658[0n]
Pyrgus alveus|LEASS566-17|TLMF Lep 22214||Austria|658[0n]
Pyrgus alveus|LEASS570-17|TLMF Lep 22218||Austria|658[0n]
Pyrgus warrenensis|EULEP4580-16|RVcoll15I345||Austria|658[0n]
Pyrgus warrenensis|OXB315-15|RVcoll.14-N029||Italy|658[0n]
Pyrgus alveus|EZSPM528-10|RVcoll.09-T188|Genit. examined|France|639[0n]
Pyrgus warrenensis|EULEP4636-16|RVcoll15G281||Switzerland|636[0n]
Pyrgus warrenensis|EULEP4635-16|RVcoll15G280||Switzerland|634[0n]
Pyrgus alveus|EULEP4606-16|RVcoll15J570||France|643[0n]
Pyrgus alveus|EZSPM530-10|RVcoll.09-T190||France|632[0n]
Pyrgus alveus|EULEP4591-16|RVcoll15I515||Austria|634[0n]
Pyrgus alveus|WMB1861-13|RVcoll.11-J167|Genit. examined|France|627[0n]
Pyrgus alveus|BIBSA326-15|RVcoll.14-E130||Italy|633[0n]
Pyrgus alveus|EULEP1859-15|RVcoll.13-U226|Genit. examined|Italy|622[0n]
Pyrgus warrenensis|GWOSK777-11|BC ZSM Lep 48993||Germany|633[0n]
Pyrgus alveus|LEATJ1338-16|TLMF Lep 19661||Austria|633[0n]
Pyrgus alveus|ABOLA479-14|TLMF Lep 16166||Austria|658[0n]
Pyrgus alveus|EZSPC031-09|RVcoll.08-H202|Genit. examined|Italy|658[0n]
Pyrgus warrenensis|EZSPC030-09|RVcoll.08-H201||Italy|658[0n]
Pyrgus alveus|EZSPC021-09|RVcoll.07-W286|Genit. examined|Italy|658[0n]
Pyrgus alveus|BIBSA316-15|RVcoll.14-E118||Italy|658[0n]
Pyrgus alveus|EULEP642-15|RVcoll.11-J127|Genit. examined|Switzerland|658[0n]
Pyrgus warrenensis|EULEP4638-16|RVcoll15H646||Switzerland|658[0n]
Pyrgus warrenensis|EULEP4633-16|RVcoll15I189||Austria|658[0n]
Pyrgus warrenensis|EULEP4632-16|RVcoll15I156||Austria|658[0n]
Pyrgus alveus|EULEP4628-16|RVcoll15H270||Switzerland|658[0n]
Pyrgus alveus|EULEP4624-16|RVcoll15I452||Austria|658[0n]
Pyrgus warrenensis|EULEP4623-16|RVcoll15I158||Austria|658[0n]
Pyrgus alveus|EULEP4621-16|RVcoll15H752||Switzerland|658[0n]
Pyrgus warrenensis|EULEP4607-16|RVcoll15H269||Switzerland|658[0n]
Pyrgus warrenensis|EULEP4605-16|RVcoll15J457||France|658[0n]
Pyrgus warrenensis|EULEP4581-16|RVcoll15I363||Austria|658[0n]
Pyrgus alveus|EULEP4579-16|RVcoll15H235||Switzerland|658[0n]
Pyrgus warrenensis|EULEP4577-16|RVcoll15G282||Switzerland|658[0n]
Pyrgus warrenensis|EULEP4576-16|RVcoll15J332||France|658[0n]
Pyrgus warrenensis|EULEP4574-16|RVcoll15I998||Switzerland|658[0n]
Pyrgus warrenensis|EULEP4573-16|RVcoll15H344||Switzerland|658[0n]
Pyrgus alveus|EULEP4572-16|RVcoll15G959||Switzerland|658[0n]
Pyrgus alveus|EULEP4571-16|RVcoll14V897||Italy|658[0n]
Pyrgus alveus|WMB2843-13|RVcoll.12-Q690|Genit. examined|Italy|658[0n]
Pyrgus alveus|GWOSN696-11|BC ZSM Lep 52902||Germany|658[0n]
Pyrgus warrenensis|GWOSK771-11|BC ZSM Lep 48987||Germany|658[0n]
Pyrgus alveus|ODOPE200-11|BC ZSM Lep 53071||Austria|658[0n]
Pyrgus alveus|FBLMX235-11|BC ZSM Lep 50446|ID verfied by dissection|Germany|658[0n]
Pyrgus alveus|FBLMX233-11|BC ZSM Lep 50444||Germany|658[0n]
Pyrgus alveus|FBLMX231-11|BC ZSM Lep 50442|ID verified by barcode|Germany|658[0n]
Pyrgus alveus|FBLMX229-11|BC ZSM Lep 50440||Germany|658[0n]
Pyrgus alveus|FBLMX226-11|BC ZSM Lep 50437||Germany|658[0n]
Pyrgus alveus|FBLMX224-11|BC ZSM Lep 50435||Germany|658[0n]
Pyrgus alveus|FBLMX222-11|BC ZSM Lep 50433||Germany|658[0n]
Pyrgus alveus|FBLMX220-11|BC ZSM Lep 50431||Germany|658[0n]
Pyrgus alveus|FBLMX216-11|BC ZSM Lep 50427||Germany|658[0n]
Pyrgus alveus|FBLMX124-11|BC ZSM Lep 37605||Germany|658[0n]
Pyrgus alveus|FBLMX218-11|BC ZSM Lep 50429|Provisional ID, to be verified|Germany|658[0n]
Pyrgus alveus|FBLMW353-10|BC ZSM Lep 37454||Austria|658[0n]
Pyrgus warrenensis|FBLMW321-10|BC ZSM Lep 37422||Austria|658[0n]
Pyrgus alveus|FBLMW318-10|BC ZSM Lep 37419||Austria|658[0n]
Pyrgus alveus|FBLMW317-10|BC ZSM Lep 37418||Austria|658[0n]
Pyrgus alveus|FBLMW294-10|BC ZSM Lep 37395||Austria|658[0n]
Pyrgus alveus|FBLMW288-10|BC ZSM Lep 37389||Austria|658[0n]
Pyrgus alveus|GWOSU033-11|BC ZSM Lep 53284||Switzerland|658[0n]
Pyrgus alveus|GWORT471-10|BC ZSM Lep 32157||Germany|658[0n]
Pyrgus warrenensis|GWORL194-09|BC ZSM Lep 18600||Germany|658[0n]
Pyrgus warrenensis|GWORL190-09|BC ZSM Lep 18596||Germany|658[0n]
Pyrgus alveus|LEATH742-14|TLMF Lep 15954||Italy|658[0n]
Pyrgus alveus|PHLAW045-13|TLMF Lep 09842||Austria|658[0n]
Pyrgus alveus|PHLAB354-10|TLMF Lep 01154||Switzerland|658[0n]
Pyrgus alveus|LEATJ1334-16|TLMF Lep 19657||Austria|658[0n]
Pyrgus alveus|PHLAF641-11|TLMF Lep 05811||Switzerland|658[0n]
Pyrgus alveus|PHLAB1181-10|TLMF Lep 01981||Italy|658[0n]
Pyrgus alveus|PHLAB355-10|TLMF Lep 01155||Switzerland|658[0n]
Pyrgus serratulae|EULEP1435-15|RVcoll.14-G123||Greece|658[0n]
Pyrgus serratulae|EULEP991-15|RVcoll.14-G049||Greece|658[0n]
[illegible]

Pyrgus alveus|PHLAB555-10|TLMF Lep 01155||Switzerland|658[0n]
Pyrgus serratulae|EULEP1435-15|RVcoll.14-G123||Greece|658[0n]
Pyrgus serratulae|EULEP991-15|RVcoll.14-G049||Greece|658[0n]
Pyrgus serratulae|EULEP1338-15|RVcoll.14-F827||Greece|658[0n]
Pyrgus serratulae|EULEP2523-15|RVcoll.14-V395||Ukraine|658[0n]
Pyrgus serratulae|EULEP2522-15|RVcoll.14-V394||Ukraine|658[0n]
Pyrgus serratulae|EULEP1694-15|RVcoll.14-H050||Greece|658[0n]
Pyrgus serratulae|EULEP1661-15|RVcoll.14-G876||Greece|658[0n]
Pyrgus serratulae|EULEP1230-15|RVcoll.14-F533||Greece|658[0n]
Pyrgus serratulae|EULEP4919-16|RVcoll16J625||Greece|658[0n]
Pyrgus serratulae|GBLAB143-13|BC ZSM Lep 75769|Identified by barcode|Germany|658[0n]
Pyrgus serratulae|EZSPC791-10|RVcoll.08-R134|Genit. examined|Spain|658[0n]
Pyrgus serratulae|EZSPC785-10|RVcoll.08-R133|Genit. examined|Spain|658[0n]
Pyrgus serratulae|EZSPN262-09|RVcoll.07-W171||Spain|658[0n]
Pyrgus serratulae|EZSPC1061-10|RVcoll.08-L083||Spain|658[0n]
Pyrgus serratulae|EZSPN233-09|RVcoll.07-W074||Spain|658[0n]
Pyrgus serratulae|WMB3325-14|RVcoll.08-P742||Spain|658[0n]
Pyrgus serratulae|EZSPC1181-10|RVcoll.09-T154||Spain|658[0n]
Pyrgus serratulae|EZSPC1163-10|RVcoll.09-T038||Spain|658[0n]
Pyrgus serratulae|EZSPM123-09|RVcoll.08-P612||Spain|658[0n]
Pyrgus serratulae|EZSPC971-10|RVcoll.09-T155||Spain|658[0n]
Pyrgus serratulae|EZSPC970-10|RVcoll.08-P743||Spain|658[0n]
Pyrgus serratulae|EZSPN116-09|RVcoll.06-A282||Spain|658[0n]
Pyrgus serratulae|EZSPC1162-10|RVcoll.09-T035||Spain|641[1n]
Pyrgus serratulae|EZSPN847-09|RVcoll.08-L691||Spain|658[0n]
Pyrgus serratulae|EZSPN110-09|RVcoll.06-A260||Spain|658[0n]
Pyrgus serratulae|EZSPC1252-10|RVcoll.09-V840|Genit. examined|Spain|658[0n]
Pyrgus serratulae|EZSPN196-09|RVcoll.07-C660||France|658[0n]
Pyrgus serratulae|EZSPN799-09|RVcoll.08-L414||Spain|658[0n]
Pyrgus serratulae|EULEP2163-15|RVcoll.14-K079||Switzerland|658[0n]
Pyrgus serratulae|OXB750-15|13-U458||Italy|658[0n]
Pyrgus serratulae|OXB749-15|13-U455||Italy|658[0n]
Pyrgus serratulae|BIBSA1218-15|15-M356||Italy|658[0n]
Pyrgus serratulae|EULEP1871-15|RVcoll.13-U454||Italy|658[0n]
Pyrgus serratulae|EULEP4625-16|RVcoll15I656||Austria|658[0n]
Pyrgus serratulae|PHLAI870-13|KLM Lep 01060||Austria|658[0n]
Pyrgus serratulae|LEATD379-13|TLMF Lep 13026||Italy|658[0n]
Pyrgus serratulae|LEATD378-13|TLMF Lep 13025||Italy|658[0n]
Pyrgus serratulae|EULEP4620-16|RVcoll15G876||Switzerland|658[0n]
Pyrgus serratulae|ODOPE201-11|BC ZSM Lep 53072|Identified by barcode|Austria|658[0n]
Pyrgus serratulae|GWOSK773-11|BC ZSM Lep 48989||Germany|658[0n]
Pyrgus serratulae|GWOSK778-11|BC ZSM Lep 48994||Germany|658[0n]
Pyrgus serratulae|WMB787-13|RVcoll.07-D816||Italy|658[0n]
Pyrgus serratulae|WMB942-13|RVcoll.10-A634||France|658[0n]
Pyrgus serratulae|WMB6579-18|RVcoll14A091||Italy|658[0n]
Pyrgus serratulae|WMB6580-18|RVcoll14A092||Italy|658[0n]
Pyrgus serratulae|WMB4930-14|RVcoll.14-I477||Italy|658[0n]
Pyrgus serratulae|WMB4938-14|RVcoll.14-I485||Italy|658[0n]
Pyrgus serratulae|EULEP4608-16|RVcoll15J518||France|658[0n]
Pyrgus serratulae|EULEP4627-16|RVcoll15J337||France|658[0n]
Pyrgus serratulae|BIBSA321-15|RVcoll.14-E123||Italy|658[0n]
Pyrgus serratulae|EZSPN041-09|RVcoll.09-V211|Genit. examined|France|658[0n]
Pyrgus serratulae|EZSPN042-09|RVcoll.09-V212|Genit. examined|France|658[0n]
Pyrgus serratulae|EZSPN044-09|RVcoll.09-V214|Genit. examined|France|658[0n]
Pyrgus serratulae|EZSPN045-09|RVcoll.09-V215|Genit. examined|France|658[0n]
Pyrgus serratulae|EZSPN043-09|RVcoll.09-V213|Genit. examined|France|652[0n]
Pyrgus serratulae|EULEP1073-15|RVcoll.14-E992||Serbia|627[0n]
Pyrgus serratulae|EZSPM526-10|RVcoll.09-X675||Spain|629[0n]
Pyrgus serratulae|LEFID123-10|MM05859||Estonia|641[0n]
Pyrgus serratulae|FBLMX130-11|BC ZSM Lep 37611|ID verfied by dissection|Germany|658[0n]
Pyrgus serratulae|GWOSR003-11|BC ZSM Lep 44039||Germany|658[0n]
Pyrgus serratulae|PHLAF649-11|TLMF Lep 05819||Austria|658[0n]
Pyrgus serratulae|LEATJ1336-16|TLMF Lep 19659||Austria|658[0n]
Pyrgus serratulae|LEATG026-14|TLMF Lep 13813||Austria|658[0n]
Pyrgus serratulae|LEATJ1337-16|TLMF Lep 19660||Austria|658[0n]
Pyrgus andromedae|EZROM696-08|RV-07-F557|Genit. examined|Romania|658[0n]
Pyrgus andromedae|EULEP4777-16|RVcoll16H301||Norway|658[0n]
Pyrgus andromedae|EULEP152-14|RVcoll.11-D597||Romania|658[0n]
Pyrgus andromedae|EULEP565-15|RVcoll.09-V611||Norway|658[0n]
Pyrgus andromedae|EULEP566-15|RVcoll.09-V614||Norway|658[0n]
Pyrgus andromedae|EULEP825-15|RVcoll.14-B629||Sweden|658[0n]
Pyrgus andromedae|LEFIJ483-10|MM17108||Finland|658[1n]
Pyrgus andromedae|LEFIJ484-10|MM17109||Finland|658[0n]
Pyrgus andromedae|LEFIF116-10|MM10572||Finland|631[0n]
Pyrgus andromedae|LEFIA748-10|MM04102||Finland|630[0n]
Pyrgus andromedae|LON175-08|NHMO-06176||Norway|657[0n]
Pyrgus andromedae|WMB5113-14|RVcoll. 14-L244||Italy|658[0n]
Pyrgus andromedae|EULEP5848-18|RVcoll14U564||Albania|658[0n]
Pyrgus andromedae|EULEP5840-18|RVcoll14U550||Albania|658[0n]
Pyrgus andromedae|EULEP5839-18|RVcoll14U549||Albania|658[0n]
Pyrgus andromedae|EZSPN1092-11|Rvcoll.10-A005||Spain|658[0n]
Pyrgus andromedae|EZSPN1093-11|Rvcoll.10-A006||Spain|658[0n]
Pyrgus andromedae|EZSPC290-09|RVcoll.07-W125||Spain|658[0n]
Pyrgus andromedae|EZSPN1091-11|Rvcoll.10-A004||Spain|638[0n]
Pyrgus andromedae|EZSPC289-09|RVcoll.07-W110|Genit. examined|Spain|658[0n]
Pyrgus andromedae|EZSPC288-09|RVcoll.07-W109|Genit. examined|Spain|658[0n]
Pyrgus andromedae|LEASS923-17|KLM Lep 08428||Austria|658[0n]
Pyrgus andromedae|BIBSA317-15|RVcoll.14-E119||Italy|658[0n]
Pyrgus andromedae|WMB925-13|RVcoll.10-A083||Italy|658[0n]
Pyrgus andromedae|WMB924-13|RVcoll.10-A082||Italy|658[0n]
Pyrgus andromedae|LEASS573-17|TLMF Lep 22221||Austria|658[0n]
Pyrgus andromedae|LEASS572-17|TLMF Lep 22220||Austria|658[0n]
Pyrgus andromedae|WMB921-13|RVcoll.10-A078||Italy|658[0n]
Pyrgus andromedae|ABOLB048-15|TLMF Lep 17053||Italy|658[0n]
Pyrgus andromedae|GWORL284-09|BC ZSM Lep 21996||Germany|658[0n]
Pyrgus andromedae|EULEP4583-16|RVcoll14V900||Switzerland|658[0n]
Pyrgus andromedae|EULEP4584-16|RVcoll15G861||Switzerland|658[0n]
Pyrgus andromedae|EULEP4585-16|RVcoll15H475||Switzerland|658[0n]
Pyrgus andromedae|EULEP4586-16|RVcoll15I683||Austria|658[0n]
Pyrgus andromedae|EULEP4587-16|RVcoll15I769||Austria|658[0n]
Pyrgus andromedae|EULEP4588-16|RVcoll15J033||Switzerland|658[0n]
Pyrgus andromedae|LEASS704-17|TLMF Lep 22352||Austria|658[0n]
Pyrgus andromedae|EULEP574-15|RVcoll.09-X688||Austria|653[0n]

Pyrgus andromedae|EULEP4588-16|RVcoll15J033||Switzerland|658[0n]
Pyrgus andromedae|LEASS704-17|TLMF Lep 22352||Austria|658[0n]
Pyrgus andromedae|EULEP574-15|RVcoll.09-X688||Austria|653[0n]
Pyrgus andromedae|ABOLB057-15|TLMF Lep 17062||Italy|622[0n]
Pyrgus andromedae|LEATJ1248-16|TLMF Lep 19571||Austria|658[0n]
Pyrgus andromedae|LEATI017-15|TLMF Lep 17402||Austria|658[0n]
Pyrgus andromedae|LEATJ1249-16|TLMF Lep 19572||Austria|658[0n]
Pyrgus andromedae|ABOLB023-15|TLMF Lep 17028||Italy|658[0n]
Pyrgus andromedae|LEATJ267-15|TLMF Lep 18697||Austria|658[0n]
Pyrgus andromedae|PHLAB308-10|TLMF Lep 01108||Switzerland|658[0n]
Pyrgus andromedae|LEATC130-13|TLMF Lep 11257||Italy|658[0n]
Pyrgus andromedae|PHLAC706-10|TLMF Lep 02741||Switzerland|658[0n]
Pyrgus andromedae|PHLAW005-13|TLMF Lep 09802||Austria|658[0n]
Pyrgus centaureae|LEFIJ485-10|MM17110||Finland|658[0n]
Pyrgus centaureae|EULEP4763-16|RVcoll16H069||Sweden|658[0n]
Pyrgus centaureae|EULEP5169-17|RVcoll16G763||Sweden|658[0n]
Pyrgus centaureae|EULEP526-15|RVcoll.08-H812||Sweden|658[0n]
Pyrgus centaureae|LEFIF117-10|MM10573||Finland|633[0n]
Pyrgus centaureae|LEFIJ431-10|MM18621||Finland|658[0n]
Pyrgus centaureae|LON035-08|NHMO-06035||Norway|657[0n]
Pyrgus cacaliae|EULEP165-14|RVcoll.11-I596||Romania|658[0n]
Pyrgus cacaliae|EZROM336-08|RV-08-A014|Genit. examined|Romania|658[0n]
Pyrgus cacaliae|EZROM306-08|RV-07-E668||Romania|658[0n]
Pyrgus cacaliae|EZRMN235-08|RVcoll.08-M576||Romania|658[0n]
Pyrgus cacaliae|EULEP4631-16|RVcoll15G675||Italy|658[0n]
Pyrgus cacaliae|BIBSA318-15|RVcoll.14-E120||Italy|633[0n]
Pyrgus cacaliae|EULEP4602-16|RVcoll15G213||Switzerland|641[0n]
Pyrgus cacaliae|EULEP4597-16|RVcoll15G942||Switzerland|658[0n]
Pyrgus cacaliae|EULEP4596-16|RVcoll15G674||Italy|658[0n]
Pyrgus cacaliae|EULEP4589-16|RVcoll15G658||Switzerland|658[0n]
Pyrgus cacaliae|WMB1048-13|RVcoll.08-P525||France|658[0n]
Pyrgus cacaliae|WMB1049-13|RVcoll.08-P526||France|658[0n]
Pyrgus cacaliae|WMB1047-13|RVcoll.08-P524||France|658[0n]
Pyrgus cacaliae|WMB923-13|RVcoll.10-A080||Italy|658[0n]
Pyrgus cacaliae|WMB922-13|RVcoll.10-A079||Italy|658[0n]
Pyrgus cacaliae|FBLMX125-11|BC ZSM Lep 37606|Identified by barcode|Germany|658[0n]
Pyrgus cacaliae|ABOLB049-15|TLMF Lep 17054||Italy|658[0n]
Pyrgus cacaliae|EZSPC295-09|RVcoll.07-W247||Spain|658[0n]
Pyrgus cacaliae|EZSPC296-09|RVcoll.07-W249||Spain|658[0n]
Pyrgus cacaliae|EZSPC297-09|RVcoll.07-W291||Spain|658[0n]
Pyrgus cacaliae|EZSPC294-09|RVcoll.07-W246||Spain|641[0n]
Pyrgus cacaliae|EZSPM594-12|RVcoll.10-A056||Spain|658[0n]
Pyrgus cacaliae|EZSPM593-12|RVcoll.10-A055||Spain|658[0n]
Pyrgus cacaliae|EZSPM592-12|RVcoll.10-A053||Spain|658[0n]
Pyrgus cacaliae|EZSPN290-09|RVcoll.07-W248||Spain|658[0n]
Pyrgus cacaliae|EZSPC298-09|RVcoll.07-W292||Spain|658[0n]
Pyrgus cacaliae|ABOLB020-15|TLMF Lep 17025||Italy|658[0n]
Pyrgus cacaliae|ABOLB050-15|TLMF Lep 17055||Austria|658[0n]
Pyrgus cacaliae|LEATC125-13|TLMF Lep 11252||Italy|658[0n]
Pyrgus cacaliae|LEATD301-13|TLMF Lep 12948||Italy|658[0n]
Pyrgus cacaliae|FBLMX122-11|BC ZSM Lep 37603|ID verified by barcode|Germany|658[0n]
Pyrgus cacaliae|EULEP4599-16|RVcoll15H478||Switzerland|658[0n]
Pyrgus cacaliae|EULEP4600-16|RVcoll15H807||Italy|658[0n]
Pyrgus cacaliae|EULEP4637-16|RVcoll15I927||Switzerland|658[0n]
Pyrgus cacaliae|EULEP571-15|RVcoll.09-X684||Austria|658[0n]
Pyrgus cacaliae|EULEP1986-15|RVcoll.14-H988||Switzerland|658[0n]
Pyrgus cacaliae|LEATC619-13|TLMF Lep 12601||Austria|621[0n]
Pyrgus cacaliae|ABOLB022-15|TLMF Lep 17027||Italy|658[1n]
Pyrgus cacaliae|PHLAF634-11|TLMF Lep 05804||Switzerland|658[0n]
Pyrgus cacaliae|ABOLD016-16|TLMF Lep 21094||Austria|609[8n]
Pyrgus cacaliae|LEASS554-17|TLMF Lep 22202||Austria|658[0n]
Pyrgus cacaliae|EULEP912-15|RVcoll.14-C771||Bulgaria|658[0n]
Pyrgus cacaliae|EULEP4598-16|RVcoll15H281||Switzerland|658[0n]
Pyrgus cacaliae|EULEP4595-16|RVcoll10B435||Bulgaria|658[0n]
Pyrgus cacaliae|EULEP4594-16|RVcoll10B432||Bulgaria|658[0n]
Pyrgus cacaliae|LEATJ268-15|TLMF Lep 18698||Austria|658[0n]
Pyrgus cacaliae|EULEP909-15|RVcoll.14-C767||Bulgaria|658[0n]
Pyrgus cacaliae|EULEP4601-16|RVcoll15I251||Austria|658[0n]
Pyrgus cacaliae|LEATJ1250-16|TLMF Lep 19573||Austria|658[0n]
Pyrgus sidae|EULEP1504-15|RVcoll.14-G395||Greece|658[0n]
Pyrgus sidae|EZSPC610-09|RVcoll.07-C514|ex. ovo|Greece|658[0n]
Pyrgus sidae|EZSPC183-09|RVcoll.07-W092||Spain|658[0n]
Pyrgus sidae|EZSPC164-09|RVcoll.07-W020||Russia|658[0n]
Pyrgus sidae|EZSPC173-09|RVcoll.07-W037||France|658[0n]
Pyrgus sidae|EZSPN046-09|RVcoll.09-V217||France|658[0n]
Pyrgus sidae|EZSPN047-09|RVcoll.09-V218||France|658[0n]
Pyrgus sidae|EZSPN048-09|RVcoll.09-V219||France|658[0n]
Pyrgus sidae|EZSPN049-09|RVcoll.09-V220||France|658[0n]
Pyrgus sidae|EZSPN050-09|RVcoll.09-V221||France|658[0n]
Pyrgus sidae|EZSPC102-09|RVcoll.06-A275||Italy|658[0n]
Pyrgus sidae|EZSPC172-09|RVcoll.07-W036||France|658[0n]
Pyrgus sidae|EZSPC175-09|RVcoll.07-W049||France|658[0n]
Pyrgus sidae|EZSPC176-09|RVcoll.07-W050||France|658[0n]
Pyrgus sidae|EZSPC177-09|RVcoll.07-W051||France|658[0n]
Pyrgus sidae|EZSPC178-09|RVcoll.07-W052||France|658[0n]
Pyrgus sidae|BIBSA618-15|LEP-SS-00238||Italy|658[0n]
Pyrgus sidae|EZSPM531-10|RVcoll.09-T191||Italy|623[0n]
Pyrgus sidae|WMB5223-14|RVcoll.13-T978||Italy|638[0n]
Pyrgus sidae|WMB5195-14|RVcoll.13-T861||Italy|658[0n]
Pyrgus sidae|WMB5185-14|RVcoll.13-S731||Italy|658[0n]
Pyrgus sidae|WMB5184-14|RVcoll.13-S720||Italy|658[0n]
Pyrgus sidae|WMB4857-14|RVcoll.14-I404||Italy|658[0n]
Pyrgus sidae|WMB644-11|RVcoll.11-I184||Italy|658[0n]
Pyrgus sidae|EZSPC185-09|RVcoll.07-W100||Spain|658[0n]
Pyrgus sidae|EZSPN023-09|RVcoll.09-T040||Spain|658[2n]
Pyrgus sidae|EZSPC187-09|RVcoll.07-W102||Spain|658[0n]
Pyrgus sidae|EZSPC182-09|RVcoll.07-W091||Spain|658[0n]
Pyrgus sidae|EZSPN026-09|RVcoll.09-T043||Spain|658[0n]
Pyrgus sidae|EZSPN025-09|RVcoll.09-T042||Spain|658[0n]
Pyrgus sidae|EZSPN024-09|RVcoll.09-T041||Spain|658[0n]
Pyrgus sidae|EZSPN006-09|RVcoll.07-W101.1||Spain|658[0n]
Pyrgus sidae|EULEP2566-15|RVcoll.14-V541||Ukraine|658[0n]
Pyrgus sidae|EULEP2565-15|RVcoll.14-V540||Ukraine|658[0n]

[illegible]
Pyrgus sidae|EZSPN006-09|RVcoll.07-W101.1||Spain|658[0n]
Pyrgus sidae|EULEP2566-15|RVcoll.14-V541||Ukraine|658[0n]
Pyrgus sidae|EULEP2565-15|RVcoll.14-V540||Ukraine|658[0n]
Pyrgus sidae|EZSPC165-09|RVcoll.07-W021||Russia|658[0n]
Pyrgus sidae|EZSPC618-09|RVcoll.08-J440||Russia|658[0n]
Pyrgus sidae|EZSPC065-09|RVcoll.08-R494||Russia|658[0n]
Pyrgus sidae|EZSPC163-09|RVcoll.07-W019||Russia|658[0n]
Pyrgus sidae|EZSPC166-09|RVcoll.07-W022||Russia|658[0n]
Pyrgus sidae|EZSPC064-09|RVcoll.08-R493||Russia|658[0n]
Pyrgus sidae|EZSPC044-09|RVcoll.08-L624||Russia|658[0n]
Pyrgus sidae|EZROM657-08|RV-06-N024||Romania|658[0n]
Pyrgus sidae|EZROM531-08|RV-06-M918||Romania|658[0n]
Pyrgus sidae|EZRMN246-08|RVcoll.07-D412||Romania|658[0n]
Pyrgus sidae|OXB1134-15|15-A667||Italy|658[0n]
Pyrgus sidae|EZSPC184-09|RVcoll.07-W099||Spain|658[0n]
Pyrgus sidae|EZSPN022-09|RVcoll.09-T039||Spain|658[3n]
Pyrgus sidae|EULEP1049-15|RVcoll.14-E919||Serbia|658[0n]
Pyrgus sidae|EZSPM400-09|Rvcoll.09-V372||Bulgaria|658[0n]
Pyrgus sidae|EZSPC616-09|RVcoll.07-C561|ex. ovo|Greece|658[0n]
Pyrgus sidae|EULEP4629-16|RVcoll10B370||Bulgaria|658[0n]
Pyrgus sidae|EULEP1127-15|RVcoll.14-F243||Serbia|658[0n]
Pyrgus sidae|EULEP4630-16|RVcoll14V860||Bulgaria|658[0n]
Pyrgus sidae|EULEP1232-15|RVcoll.14-F539||Greece|658[0n]
Pyrgus sidae|EULEP1669-15|RVcoll.14-G907||Greece|658[0n]
Pyrgus sidae|EZSPM399-09|Rvcoll.09-V371||Bulgaria|618[0n]
Pyrgus sidae|EZSPC025-09|RVcoll.07-W297||Greece|658[0n]
Pyrgus sidae|EZROM533-08|RV-07-D039||Romania|658[0n]
Pyrgus sidae|EZROM532-08|RV-07-D024||Romania|658[0n]
Pyrgus sidae|EZRMN245-08|RVcoll.07-D189||Romania|658[0n]
Pyrgus sidae|EZRMN244-08|RVcoll.07-D166||Romania|658[0n]
Pyrgus carthami|EZROM655-08|RV-06-M903||Romania|658[0n]
Pyrgus carthami|EULEP2364-15|RVcoll.14-V057||Ukraine|658[0n]
Pyrgus carthami|LEASS750-17|TLMF Lep 22398||Austria|658[0n]
Pyrgus carthami|EULEP1167-15|RVcoll.14-F375||Bulgaria|658[0n]
Pyrgus carthami|EULEP1161-15|RVcoll.14-F363||Bulgaria|658[0n]
Pyrgus carthami|LEASS557-17|TLMF Lep 22205||Austria|658[0n]
Pyrgus carthami|ABOLD631-17|TLMF Lep 21707||Austria|634[0n]
Pyrgus carthami|LEASS754-17|TLMF Lep 22402||Austria|658[0n]
Pyrgus carthami|EULEP012-14|RVcoll.06-K671||Romania|658[0n]
Pyrgus carthami|EZROM527-08|RV-07-D422||Romania|658[0n]
Pyrgus carthami|EZROM526-08|RV-07-D390||Romania|658[0n]
Pyrgus carthami|EZROM525-08|RV-06-K667||Romania|658[0n]
Pyrgus carthami|EZRMN238-08|RVcoll.08-M501||Romania|658[0n]
Pyrgus carthami|EZRMN237-08|RVcoll.07-D400||Romania|658[0n]
Pyrgus carthami|EZRMN236-08|RVcoll.06-M912||Romania|658[0n]
Pyrgus carthami|WMB829-13|RVcoll.07-E203||Italy|658[0n]
Pyrgus carthami|WMB828-13|RVcoll.07-E201||Italy|658[0n]
Pyrgus carthami|BIBSA320-15|RVcoll.14-E122||Italy|658[0n]
Pyrgus carthami|BIBSA273-15|RVcoll.14-E073||Italy|658[0n]
Pyrgus carthami|EULEP2144-15|RVcoll.14-K006||Switzerland|658[0n]
Pyrgus carthami|EULEP4610-16|RVcoll15G523||Switzerland|658[0n]
Pyrgus carthami|WMB1838-13|RVcoll.11-I872||France|658[0n]
Pyrgus carthami|WMB1620-13|RVcoll.12-O759||France|658[0n]
Pyrgus carthami|WMB2679-13|RVcoll.10-B900||France|658[0n]
Pyrgus carthami|EZSPC674-09|RVcoll.08-M959||Spain|658[0n]
Pyrgus carthami|EZSPC301-09|RVcoll.07-E641||Spain|658[0n]
Pyrgus carthami|EZSPN253-09|RVcoll.07-W152||Spain|658[0n]
Pyrgus carthami|EZSPC300-09|RVcoll.08-P342||Spain|658[0n]
Pyrgus carthami|EZSPC299-09|RVcoll.08-L433||Spain|658[0n]
Pyrgus carthami|EZSPC641-09|RVcoll.08-L378||Spain|658[0n]
Pyrgus carthami|WMB3437-14|RVcoll.09-X549||Spain|658[0n]
Pyrgus carthami|WMB3273-14|RVcoll.08-L961||Spain|658[0n]
Pyrgus carthami|EZSPN270-09|RVcoll.07-W189||Spain|658[0n]
Pyrgus carthami|EZSPM356-09|RVcoll.08-R061||Spain|658[0n]
Pyrgus carthami|EZSPM350-09|RVcoll.08-R063||Spain|658[1n]
Pyrgus carthami|EZSPN064-09|RVcoll.09-V717||Spain|658[0n]
Pyrgus carthami|GBLAA1441-15|BC ZSM Lep 87232||Germany|658[0n]
Pyrgus carthami|EZSPN074-09|RVcoll.09-V526||Spain|658[0n]
Pyrgus carthami|WMB3390-14|RVcoll.09-V481||Spain|658[0n]
Pyrgus carthami|EZSPM122-09|RVcoll.08-P611||Spain|658[0n]
Pyrgus carthami|EZSPN235-09|RVcoll.07-W084||Spain|658[0n]
Pyrgus carthami|EZSPN114-09|RVcoll.06-A278||Spain|658[0n]
Pyrgus carthami|FBLMW326-10|BC ZSM Lep 37427||Germany|658[0n]
Pyrgus carthami|WMB803-13|RVcoll.07-E044||Italy|658[0n]
Pyrgus carthami|BIBSA641-15|RVcoll. 15-A804||Italy|658[0n]
Pyrgus carthami|WMB652-11|RVcoll.11-I229||Italy|658[0n]
Pyrgus carthami|GWORZ064-10|BC ZSM Lep 30420||Italy|658[0n]
Pyrgus carthami|BIBSA274-15|RVcoll.14-E074||Italy|658[0n]
Pyrgus carthami|EULEP4611-16|RVcoll15J463||France|621[0n]
Pyrgus carthami|WMB2385-13|RVcoll.07-E225||Italy|658[0n]
Pyrgus carthami|GWORL254-09|BC ZSM Lep 18660||France|658[0n]
Pyrgus carthami|LASTS021-14|TLMF Lep 14473||Austria|658[0n]
Pyrgus carthami|LASTS020-14|TLMF Lep 14472||Austria|658[0n]
Pyrgus carthami|ABOLB051-15|TLMF Lep 17056||Italy|658[0n]
Pyrgus carthami|EULEP4609-16|RVcoll14V313||Italy|658[0n]
Pyrgus carthami|GBLAA1951-15|BC ZSM Lep 89072||Italy|658[0n]
Heteropterus morpheus|OXB332-15|RVcoll.14-N046||Italy|658[0n]
Heteropterus morpheus|EULEP2868-15|RVcoll.14-N952||France|658[0n]
Heteropterus morpheus|WMB3537-14|RVcoll.10-C492||Spain|658[0n]
Heteropterus morpheus|OXB850-15|14-U813||Italy|658[0n]
Heteropterus morpheus|BIBSA1123-15|15-L896||Italy|658[0n]
Heteropterus morpheus|BIBSA1381-15|15-M964||Italy|658[0n]
Heteropterus morpheus|WMB5335-14|RVcoll.14-J225||Spain|658[0n]
Heteropterus morpheus|WMB5334-14|RVcoll.14-J224||Spain|658[0n]
Heteropterus morpheus|EULEP204-14|RVcoll.12-L017||Spain|658[0n]
Heteropterus morpheus|WMB869-13|RVcoll.09-T577||Italy|658[0n]
Heteropterus morpheus|WMB895-13|RVcoll.09-X834||Italy|658[0n]
Heteropterus morpheus|WMB2392-13|RVcoll.09-T578||Italy|658[0n]
Heteropterus morpheus|WMB4928-14|RVcoll.14-I475||Italy|658[0n]
Heteropterus morpheus|WMB5097-14|RVcoll. 14-L228||Italy|658[0n]
Heteropterus morpheus|EULEP4900-16|RVcoll16J603||Russia|658[0n]
Heteropterus morpheus|EULEP205-14|RVcoll.12-L018||Spain|658[0n]
[illegible]

Heteropterus morpheus|WMB5097-14|RVcoll.14-L228||Italy|658[0n]
Heteropterus morpheus|EULEP4900-16|RVcoll16J603||Russia|658[0n]
Heteropterus morpheus|EULEP205-14|RVcoll.12-L018||Spain|658[0n]
Heteropterus morpheus|EULEP218-14|RVcoll.12-L043||Spain|658[0n]
Heteropterus morpheus|EULEP219-14|RVcoll.12-L044||Spain|658[0n]
Heteropterus morpheus|EULEP1022-15|RVcoll.14-E772||Romania|658[0n]
Heteropterus morpheus|EULEP1028-15|RVcoll.14-E841||Serbia|658[0n]
Heteropterus morpheus|EULEP1034-15|RVcoll.14-E884||Serbia|658[0n]
Heteropterus morpheus|BIBSA740-15|LEP-SS-00267||Italy|658[0n]
Heteropterus morpheus|BIBSA1867-17|RVcoll16C761||Italy|658[0n]
Heteropterus morpheus|BIBSA1845-17|RVcoll16C723||Italy|658[0n]
Heteropterus morpheus|GBLAC284-13|BC ZSM Lep 78665|Change from Sp. sertorius (2010-10-07 AHS ...
Heteropterus morpheus|LEASS519-17|TLMF Lep 22167||Austria|658[0n]
Heteropterus morpheus|GBLAA374-14|BC ZSM Lep 80370||Germany|603[0n]
Heteropterus morpheus|WMB793-13|RVcoll.07-D853||Italy|630[0n]
Heteropterus morpheus|EZSPN263-09|RVcoll.07-W175||Spain|658[0n]
Heteropterus morpheus|EZSPN271-09|RVcoll.07-W193||Spain|658[0n]
Heteropterus morpheus|EZSPN264-09|RVcoll.07-W176||Spain|658[0n]
Heteropterus morpheus|EZROM209-08|RV-07-C140||Romania|658[0n]
Heteropterus morpheus|EZROM208-08|RV-07-C139||Romania|658[0n]
Heteropterus morpheus|EZROM1012-08|RVcoll.08-M520||Romania|658[0n]
Heteropterus morpheus|LEFIL059-10|MM19059||Russia|658[0n]
Heteropterus morpheus|LEFIL058-10|MM19058||Russia|658[0n]
Heteropterus morpheus|LEATJ1193-16|TLMF Lep 19516||Austria|658[0n]
Heteropterus morpheus|ABOLD053-16|TLMF Lep 21131||Austria|658[0n]
Heteropterus morpheus|LEATJ1194-16|TLMF Lep 19517||Austria|658[0n]
Carterocephalus silvicola|EULEP320-14|MM23805||Latvia|658[0n]
Carterocephalus silvicola|LON805-11|NHMO Lep09003||Norway|658[0n]
Carterocephalus silvicola|EULEP4716-16|RVcoll16B328||Sweden|658[0n]
Carterocephalus silvicola|LON870-11|NHMO Lep09068||Norway|658[0n]
Carterocephalus silvicola|EULEP784-15|RVcoll.12-Z194||Sweden|658[0n]
Carterocephalus silvicola|EULEP528-15|RVcoll.08-H834||Sweden|658[0n]
Carterocephalus silvicola|EULEP5089-16|RVcoll16B308||Sweden|658[0n]
Carterocephalus silvicola|EULEP3630-16|RVcoll14O556||Estonia|658[0n]
Carterocephalus silvicola|EULEP3629-16|RVcoll10A795||Estonia|658[0n]
Carterocephalus silvicola|LEFIJ481-10|MM17106||Finland|658[0n]
Carterocephalus silvicola|LEFIJ480-10|MM17105||Finland|658[0n]
Carterocephalus silvicola|LEFIA1040-10|MM00616||Finland|658[0n]
Carterocephalus silvicola|LEFIG184-10|MM14052||Finland|658[0n]
Carterocephalus palaemon|EULEP4871-16|RVcoll16H935||Poland|658[0n]
Carterocephalus palaemon|EULEP354-14|MM23839||Lithuania|658[0n]
Carterocephalus palaemon|EULEP3622-16|RVcoll10A788||Estonia|658[0n]
Carterocephalus palaemon|LEFIJ479-10|MM17104||Finland|658[0n]
Carterocephalus palaemon|LEFIB139-10|MM00511||Finland|658[0n]
Carterocephalus palaemon|EZROM840-08|RVcoll.07-D290||Romania|658[0n]
Carterocephalus palaemon|EULEP3628-16|RVcoll15P022||Belarus|658[0n]
Carterocephalus palaemon|EULEP756-15|RVcoll.12-R592||Romania|658[0n]
Carterocephalus palaemon|EZROM082-08|RV-07-D244||Romania|658[0n]
Carterocephalus palaemon|LEATJ1174-16|TLMF Lep 19497||Czech Republic|658[0n]
Carterocephalus palaemon|EZROM081-08|RV-07-D556||Romania|658[0n]
Carterocephalus palaemon|PHLAB368-10|TLMF Lep 01168||Switzerland|658[0n]
Carterocephalus palaemon|PHLAI563-13|TLMF Lep 09125||Austria|658[0n]
Carterocephalus palaemon|LEATG057-14|TLMF Lep 13844||Austria|658[0n]
Carterocephalus palaemon|EZROM083-08|RV-07-D589||Romania|658[1n]
Carterocephalus palaemon|EULEP3627-16|RVcoll15I825||Austria|658[0n]
Carterocephalus palaemon|WMB5338-14|RVcoll.14-J229||Spain|658[0n]
Carterocephalus palaemon|EULEP3623-16|RVcoll14W726||Italy|658[0n]
Carterocephalus palaemon|EULEP3625-16|RVcoll15H351||Switzerland|658[0n]
Carterocephalus palaemon|EULEP3626-16|RVcoll15I200||Austria|658[0n]
Carterocephalus palaemon|EULEP4755-16|RVcoll16G964||Sweden|658[0n]
Carterocephalus palaemon|EULEP023-14|RVcoll.07-D557||Romania|658[0n]
Carterocephalus palaemon|EULEP5146-17|RVcoll16J902||Serbia|658[0n]
Carterocephalus palaemon|EULEP5170-17|RVcoll16G803||Sweden|658[0n]
Carterocephalus palaemon|EULEP502-15|RVcoll.07-C523||Bulgaria|658[0n]
Carterocephalus palaemon|EULEP1862-15|RVcoll.13-U264||Italy|658[0n]
Carterocephalus palaemon|EULEP2160-15|RVcoll.14-K072||Switzerland|658[0n]
Carterocephalus palaemon|EULEP2693-15|RVcoll.14-N777||United Kingdom|658[0n]
Carterocephalus palaemon|BIBSA148-15|RVcoll.14-I160||Italy|658[0n]
Carterocephalus palaemon|GBLAA1360-15|BC ZSM Lep 87056||Germany|658[0n]
Carterocephalus palaemon|GBLAA408-14|BC ZSM Lep 80499||Germany|658[0n]
Carterocephalus palaemon|GBLAB158-13|BC ZSM Lep 75784||Germany|658[0n]
Carterocephalus palaemon|LEATJ1225-16|TLMF Lep 19548||Hungary|658[0n]
Carterocephalus palaemon|OXB614-15|14-O003||Italy|658[0n]
Carterocephalus palaemon|OXB321-15|RVcoll.14-N035||Italy|658[0n]
Carterocephalus palaemon|BIBSA149-15|RVcoll.14-I161||Italy|650[0n]
Carterocephalus palaemon|BIBSA1102-15|15-L765||Italy|612[0n]
Carterocephalus palaemon|EZSPC235-09|RVcoll.08-M918||Spain|653[0n]
Carterocephalus palaemon|EZSPC209-09|RVcoll.07-W142||Spain|657[0n]
Carterocephalus palaemon|EZSPN228-09|RVcoll.07-W030||Spain|658[0n]
Carterocephalus palaemon|EZSPN227-09|RVcoll.07-W029||Spain|658[0n]
Carterocephalus palaemon|EZSPN226-09|RVcoll.07-W028||Spain|658[0n]
Carterocephalus palaemon|EZSPN225-09|RVcoll.07-W027||Spain|658[0n]
Carterocephalus palaemon|EZROM666-08|RV-06-K682||Romania|658[0n]
Carterocephalus palaemon|EZROM842-08|RVcoll.08-M340||Romania|658[0n]
Carterocephalus palaemon|EZROM841-08|RVcoll.08-M339||Romania|658[0n]
Carterocephalus palaemon|LEFIJ478-10|MM17103||Finland|658[0n]
Carterocephalus palaemon|FBLMT881-09|BC ZSM Lep 25441||Germany|658[0n]
Carterocephalus palaemon|LEFIE936-10|MM10323||Finland|658[0n]
Carterocephalus palaemon|LEFIE935-10|MM10322||Finland|658[0n]
Carterocephalus palaemon|LON473-08|NHMO-08124||Norway|657[0n]
Carterocephalus palaemon|GWORA2489-09|BC ZSM Lep 30701||Germany|658[0n]
Carterocephalus palaemon|FBLMW324-10|BC ZSM Lep 37425||Germany|658[0n]
Carterocephalus palaemon|PHLAW019-13|TLMF Lep 09816||Austria|658[0n]
Carterocephalus palaemon|ABOLD075-16|TLMF Lep 21153||Austria|658[0n]
Carterocephalus palaemon|LEATC579-13|TLMF Lep 12561||Austria|658[0n]
Carterocephalus palaemon|PHLAC400-10|TLMF Lep 02435||Italy|658[0n]
Hamearis lucina|EZROM316-08|RV-07-E678||Romania|609[0n]
Hamearis lucina|EZROM315-08|RV-07-E677||Romania|646[0n]
Hamearis lucina|EZROM318-08|RV-07-E680||Romania|658[0n]
Hamearis lucina|LEATG391-14|TLMF Lep 14178||Italy|658[0n]
Hamearis lucina|LEATG392-14|TLMF Lep 14179||Italy|658[1n]
Hamearis lucina|ODOPE747-11|BC ZSM Lep 50388||Germany|658[0n]
Hamearis lucina|GWORZ060-10|BC ZSM Lep 30416||Italy|658[0n]

Hamearis lucina|LEATG392-14|TLMF Lep 14179||Italy|658[1n]
Hamearis lucina|ODOPE747-11|BC ZSM Lep 50388||Germany|658[0n]
Hamearis lucina|GWORZ060-10|BC ZSM Lep 30416||Italy|658[0n]
Hamearis lucina|GWORO794-09|BC ZSM Lep 30486||Germany|658[0n]
Hamearis lucina|GWOSK795-11|BC ZSM Lep 49011||Germany|658[0n]
Hamearis lucina|LENOA1358-11|LN-BD1358||France|658[0n]
Hamearis lucina|GWOSA774-10|BC ZSM Lep 36640||Germany|658[0n]
Hamearis lucina|GWOSI559-10|BC ZSM Lep 44405||Germany|658[0n]
Hamearis lucina|EZROM1011-08|RVcoll.08-M209||Romania|658[0n]
Hamearis lucina|EZROM1009-08|RVcoll.08-M225||Romania|658[0n]
Hamearis lucina|EZROM1010-08|RVcoll.08-M356||Romania|658[0n]
Hamearis lucina|EZSPC427-09|RVcoll.07-W137||Spain|658[0n]
Hamearis lucina|EZSPN873-09|RVcoll.08-L763||Spain|658[0n]
Hamearis lucina|EZROM205-08|RV-07-D106||Romania|658[0n]
Hamearis lucina|EZROM206-08|RV-07-D205||Romania|658[0n]
Hamearis lucina|EZSPN037-09|RVcoll.09-T083||Spain|658[0n]
Hamearis lucina|EZSPC426-09|RVcoll.07-W105||Spain|658[0n]
Hamearis lucina|EZSPN627-09|RVcoll.08-J719||Spain|658[0n]
Hamearis lucina|EZROM722-08|RV-06-G509||Spain|658[0n]
Hamearis lucina|EZSPC1164-10|RVcoll.09-T048||Spain|658[0n]
Hamearis lucina|EZSPC1176-10|RVcoll.09-T136||Spain|658[0n]
Hamearis lucina|WMB3323-14|RVcoll.08-P740||Spain|658[0n]
Hamearis lucina|WMB4558-14|RVcoll.090211SD17||Spain|658[0n]
Hamearis lucina|EULEP5025-16|RVcoll16I955||Slovakia|658[0n]
Hamearis lucina|EULEP4813-16|RVcoll16H708||Belgium|658[0n]
Hamearis lucina|EULEP141-14|RVcoll.10-C415||Romania|658[0n]
Hamearis lucina|EULEP1989-15|RVcoll.14-I607||France|658[0n]
Hamearis lucina|BCLEP114-17|LEP-SS-00565||Italy|658[0n]
Hamearis lucina|EZROM365-08|07-E616||Spain|646[0n]
Hamearis lucina|LENOA1357-11|LN-BD1357||France|637[0n]
Hamearis lucina|PHLAI508-13|TLMF Lep 09070||Austria|630[0n]
Hamearis lucina|PHLAW017-13|TLMF Lep 09814||Austria|658[0n]
Hamearis lucina|LEATG081-14|TLMF Lep 13868||Austria|658[0n]
Hamearis lucina|ABOLD083-16|TLMF Lep 21161||Austria|658[0n]
Hamearis lucina|LEATJ1295-16|TLMF Lep 19618||Austria|658[0n]
Hamearis lucina|LEASS498-17|TLMF Lep 22146||Austria|658[0n]
Hamearis lucina|EULEP4056-16|RVcoll14V315||Italy|658[0n]
Hamearis lucina|WMB5202-14|RVcoll.13-T931||Italy|658[0n]
Hamearis lucina|LEATA078-13|TLMF Lep 09495||Italy|658[0n]
Spialia phlomidis|EULEP1183-15|RVcoll.14-F442||Bulgaria|658[0n]
Spialia phlomidis|EULEP1182-15|RVcoll.14-F441||Bulgaria|658[0n]
Spialia phlomidis|EULEP1181-15|RVcoll.14-F440||Bulgaria|658[0n]
Spialia phlomidis|EULEP3145-15|RVcoll.14-O278||Greece|658[0n]
Spialia phlomidis|EULEP3144-15|RVcoll.14-O277||Macedonia|658[0n]
Spialia phlomidis|EULEP3146-15|RVcoll.14-O279||Greece|658[0n]
Spialia phlomidis|EULEP1536-15|RVcoll.14-G494||Greece|658[0n]
Spialia phlomidis|EULEP1528-15|RVcoll.14-G478||Greece|658[0n]
Spialia phlomidis|EULEP1526-15|RVcoll.14-G473||Greece|658[0n]
Spialia phlomidis|EULEP3170-15|RVcoll.07-C524|Genit. examined|Greece|658[0n]
Spialia phlomidis|EULEP3169-15|RVcoll.07-C520|Genit. examined|Greece|658[0n]
Spialia phlomidis|EULEP4659-16|RVcoll14O317||Greece|658[0n]
Spialia therapne|EULEP3190-15|RVcoll.09-T179|Corsica. Genit. examined|France|658[0n]
Spialia therapne|EULEP3279-15|RVcoll.12-O151|Corsica|France|658[0n]
Spialia therapne|BIBSA1271-15|11-E770|Corsica|France|658[0n]
Spialia therapne|EULEP3194-15|RVcoll.09-V197|Corsica. Genit. examined|France|658[0n]
Spialia therapne|WMB291-11|RVcoll.09-V195|Genit. examined|France|658[0n]
Spialia therapne|EULEP3191-15|RVcoll.09-T181|Sardinia|Italy|658[0n]
Spialia therapne|EULEP3193-15|RVcoll.09-T185|Sardinia|Italy|658[0n]
Spialia therapne|EULEP3192-15|RVcoll.09-T182|Sardinia. Genit. examined|Italy|658[0n]
Spialia therapne|EULEP3183-15|RVcoll.08-H737|Sardinia. Genit. examined|Italy|658[0n]
Spialia therapne|EULEP3182-15|RVcoll.08-H727|Sardinia. Genit. examined|Italy|658[0n]
Spialia therapne|WMB672-12|RVcoll.08-H730|Genit. examined|Italy|658[0n]
Spialia therapne|WMB671-12|RVcoll.08-H725|Genit. examined|Italy|658[0n]
Spialia therapne|WMB287-11|RVcoll.08-H723|Genit. examined|Italy|658[0n]
Spialia orbifer|EULEP1160-15|RVcoll.14-F362||Bulgaria|658[0n]
Spialia orbifer|EULEP1303-15|RVcoll.14-F748||Greece|658[0n]
Spialia orbifer|EULEP1446-15|RVcoll.14-G147||Greece|658[0n]
Spialia orbifer|EULEP1483-15|RVcoll.14-G291||Greece|658[0n]
Spialia orbifer|EULEP1505-15|RVcoll.14-G413||Greece|658[0n]
Spialia orbifer|EULEP1657-15|RVcoll.14-G867||Greece|658[0n]
Spialia orbifer|EULEP1759-15|RVcoll.14-H422||Greece|658[0n]
Spialia orbifer|EULEP1043-15|RVcoll.14-E909||Serbia|609[0n]
Spialia orbifer|EULEP3327-15|RVcoll.14-B593||Greece|658[0n]
Spialia orbifer|EULEP3204-15|RVcoll.10-B368||Bulgaria|658[0n]
Spialia orbifer|EULEP3168-15|RVcoll.07-C509||Greece|658[0n]
Spialia orbifer|EULEP3167-15|RVcoll.07-C508||Greece|658[0n]
Spialia orbifer|EULEP1000-15|RVcoll.14-G477||Greece|614[0n]
Spialia orbifer|EULEP3326-15|RVcoll.14-B592||Greece|658[0n]
Spialia rosae|EULEP3260-15|RVcoll.12-L086|Paratype|Spain|658[0n]
Spialia rosae|EULEP3282-15|RVcoll.12-Q460||Spain|658[0n]
Spialia rosae|EULEP3283-15|RVcoll.12-Q461||Spain|658[0n]
Spialia rosae|EULEP3299-15|RVcoll.13-S881|Paratype|Spain|658[0n]
Spialia rosae|EULEP3300-15|RVcoll.13-S883|Paratype|Spain|658[0n]
Spialia rosae|EULEP3306-15|RVcoll.13-S893|Paratype|Spain|658[0n]
Spialia rosae|EULEP3307-15|RVcoll.13-S894|Paratype|Spain|658[0n]
Spialia rosae|EULEP3198-15|RVcoll.09-V936|Paratype. Genit. examined|Spain|658[0n]
Spialia rosae|EULEP3233-15|RVcoll.11-G137|Holotype|Spain|658[0n]
Spialia rosae|EULEP3316-15|RVcoll.14-A013|Paratype|Spain|658[0n]
Spialia rosae|EULEP3317-15|RVcoll.14-A018|Paratype|Spain|658[0n]
Spialia rosae|EULEP3318-15|RVcoll.14-A019|Paratype|Spain|658[0n]
Spialia rosae|EULEP3319-15|RVcoll.14-A020|Paratype|Spain|658[0n]
Spialia rosae|EULEP3320-15|RVcoll.14-A024|Paratype|Spain|658[0n]
Spialia rosae|EULEP3321-15|RVcoll.14-A025|Paratype|Spain|658[0n]
Spialia rosae|EULEP3322-15|RVcoll.14-A027|Paratype|Spain|658[0n]
Spialia rosae|EULEP3323-15|RVcoll.14-A034|Paratype|Spain|658[0n]
Spialia rosae|EULEP3328-15|RVcoll.14-B686|Paratype|Spain|658[0n]
Spialia rosae|EULEP3329-15|RVcoll.14-B687|Paratype|Spain|658[0n]
Spialia rosae|EULEP3330-15|RVcoll.14-D744|Paratype|Spain|658[0n]
Spialia rosae|EULEP3340-15|RVcoll.14-N293|Paratype|Spain|658[0n]
Spialia rosae|EULEP3344-15|RVcoll.14-N313|Paratype|Spain|658[0n]
Spialia rosae|EULEP3345-15|RVcoll.14-N314|Paratype|Spain|658[0n]
Spialia rosae|EULEP3346-15|RVcoll.14-N316|Paratype|Spain|658[0n]
[illegible]

Spialia rosae|EULEP3344-15|RVcoll.14-N313|Paratype|Spain|658[0n]
Spialia rosae|EULEP3345-15|RVcoll.14-N314|Paratype|Spain|658[0n]
Spialia rosae|EULEP3346-15|RVcoll.14-N316|Paratype|Spain|658[0n]
Spialia rosae|EULEP3347-15|RVcoll.14-N317|Paratype|Spain|658[0n]
Spialia rosae|EULEP3348-15|RVcoll.14-N318|Paratype|Spain|658[0n]
Spialia rosae|EULEP3349-15|RVcoll.14-N319|Paratype|Spain|658[0n]
Spialia rosae|EULEP3350-15|RVcoll.14-N321|Paratype|Spain|658[0n]
Spialia rosae|EULEP3351-15|RVcoll.14-N322|Paratype|Spain|658[0n]
Spialia rosae|EULEP3352-15|RVcoll.14-N323|Paratype|Spain|658[0n]
Spialia rosae|EULEP3355-15|RVcoll.14-V493|Paratype|Spain|658[0n]
Spialia rosae|EULEP3357-15|RVcoll.14-V495|Paratype|Spain|658[0n]
Spialia rosae|EULEP3358-15|RVcoll.14-V497|Paratype|Spain|658[0n]
Spialia rosae|EULEP3359-15|RVcoll.14-V918|Paratype|Spain|658[0n]
Spialia rosae|EULEP3159-15|RVcoll.06-A280|Paratype. Genit. examined|Spain|616[0n]
Spialia rosae|EULEP3264-15|RVcoll.12-L091|Paratype|Spain|658[0n]
Spialia rosae|EULEP3257-15|RVcoll.12-L068|Paratype|Spain|618[0n]
Spialia rosae|EULEP3238-15|RVcoll.11-G144|Paratype|Spain|658[0n]
Spialia rosae|WMB4452-14|RVcoll.14-D318|Paratype|Spain|658[0n]
Spialia rosae|EULEP3235-15|RVcoll.11-G139|Paratype. Genit. examined|Spain|658[0n]
Spialia rosae|EULEP3234-15|RVcoll.11-G138|Paratype. Genit. examined|Spain|658[0n]
Spialia rosae|EULEP3232-15|RVcoll.11-G136|Paratype|Spain|658[0n]
Spialia rosae|EULEP3231-15|RVcoll.11-G135|Paratype. Genit. examined|Spain|658[0n]
Spialia rosae|EULEP3230-15|RVcoll.11-G134|Paratype|Spain|658[0n]
Spialia rosae|EULEP3229-15|RVcoll.11-G133|Paratype. Genit. examined|Spain|658[0n]
Spialia rosae|EULEP3228-15|RVcoll.11-G132|Paratype. Genit. examined|Spain|658[0n]
Spialia rosae|EULEP3227-15|RVcoll.11-G131|Paratype. Genit. examined|Spain|658[0n]
Spialia rosae|EULEP3226-15|RVcoll.11-G129|Paratype. Genit. examined|Spain|658[0n]
Spialia rosae|EULEP3225-15|RVcoll.11-G128|Paratype. Genit. examined|Spain|658[0n]
Spialia rosae|EULEP3224-15|RVcoll.11-G127|Paratype. Genit. examined|Spain|658[0n]
Spialia rosae|EULEP3223-15|RVcoll.11-G126|Paratype. Genit. examined|Spain|658[0n]
Spialia rosae|EULEP3222-15|RVcoll.11-G125|Paratype. Genit. examined|Spain|658[0n]
Spialia rosae|EULEP3221-15|RVcoll.11-G124|Paratype. Genit. examined|Spain|658[0n]
Spialia rosae|EULEP3220-15|RVcoll.11-G123|Paratype. Genit. examined|Spain|658[0n]
Spialia rosae|EULEP3219-15|RVcoll.11-G122|Paratype. Genit. examined|Spain|658[0n]
Spialia rosae|EULEP3218-15|RVcoll.11-G121|Paratype. Genit. examined|Spain|658[0n]
Spialia rosae|EULEP3217-15|RVcoll.11-G120|Paratype. Genit. examined|Spain|658[0n]
Spialia rosae|EULEP3216-15|RVcoll.11-G119|Paratype. Genit. examined|Spain|658[0n]
Spialia rosae|EULEP3215-15|RVcoll.11-G118|Paratype. Genit. examined|Spain|658[0n]
Spialia rosae|EULEP3214-15|RVcoll.11-G117|Paratype. Genit. examined|Spain|658[0n]
Spialia rosae|EULEP3203-15|RVcoll.10-A099|Paratype. Genit. examined|Spain|658[0n]
Spialia rosae|EULEP3202-15|RVcoll.10-A098|Paratype|Spain|658[0n]
Spialia rosae|EULEP3200-15|RVcoll.09-X678|Paratype|Spain|658[0n]
Spialia rosae|EULEP3197-15|RVcoll.09-V471|Paratype. Genit. examined|Spain|658[0n]
Spialia rosae|EULEP3196-15|RVcoll.09-V468|Paratype|Spain|658[0n]
Spialia rosae|EULEP3261-15|RVcoll.12-L088|Paratype|Spain|658[0n]
Spialia rosae|EULEP3258-15|RVcoll.12-L069|Paratype|Spain|658[0n]
Spialia rosae|EULEP3255-15|RVcoll.12-L065|Paratype|Spain|658[0n]
Spialia rosae|EULEP3240-15|RVcoll.11-G146|Paratype|Spain|658[0n]
Spialia rosae|EULEP3239-15|RVcoll.11-G145|Paratype|Spain|658[0n]
Spialia rosae|EULEP3237-15|RVcoll.11-G141|Paratype. Genit. examined|Spain|658[0n]
Spialia rosae|EULEP3236-15|RVcoll.11-G140|Paratype. Genit. examined|Spain|658[0n]
Spialia rosae|EULEP221-14|RVcoll.12-L073|Paratype|Spain|658[0n]
Spialia rosae|EULEP161-14|RVcoll.11-G130|Paratype. Genit. examined|Spain|658[0n]
Spialia rosae|EULEP121-14|RVcoll.10-A097|Paratype|Spain|658[0n]
Spialia rosae|EULEP105-14|RVcoll.09-V459|Paratype|Spain|658[0n]
Spialia rosae|WMB4454-14|RVcoll.14-D320|Paratype|Spain|658[0n]
Spialia rosae|EZSPM468-09|Rvcoll.09-V519|Paratype|Spain|658[0n]
Spialia orbifer|WMB562-11|RVcoll.11-H758|Sicily|Italy|658[0n]
Spialia orbifer|EULEP3249-15|RVcoll.11-H992|Sicily. Genit. examined|Italy|658[0n]
Spialia orbifer|EULEP3290-15|RVcoll.12-Q588|Sicily|Italy|658[0n]
Spialia orbifer|WMB740-12|RVcoll.11-J653|Sicily. Genit. examined|Italy|642[0n]
Spialia orbifer|WMB716-12|RVcoll.11-H963|Sicily. Genit. examined|Italy|658[0n]
Spialia orbifer|WMB712-12|RVcoll.11-H757|Sicily|Italy|658[0n]
Spialia orbifer|WMB058-11|RVcoll.LD-2583|Sicily|Italy|658[0n]
Spialia orbifer|EZRMN411-09|RVcoll.07-E361||Romania|648[0n]
Spialia orbifer|EZROM550-08|RV-07-D138|Genit. examined|Romania|658[0n]
Spialia orbifer|EZROM549-08|RV-07-D046|Genit. examined|Romania|658[0n]
Spialia orbifer|EZRMN427-09|RVcoll.09-V680|Genit. examined|Romania|658[0n]
Spialia orbifer|EZRMN279-08|RVcoll.08-M558||Romania|658[0n]
Spialia orbifer|EZRMN278-08|RVcoll.08-M439|Genit. examined|Romania|658[0n]
Spialia orbifer|EZRMN277-08|RVcoll.08-M387||Romania|658[0n]
Spialia orbifer|EZRMN276-08|RVcoll.07-D056|Genit. examined|Romania|658[0n]
Spialia orbifer|EZROM688-08|RV-07-D036|Genit. examined|Romania|658[0n]
Spialia orbifer|EZRMN275-08|RVcoll.07-D038||Romania|658[0n]
Spialia sertorius|EULEP4660-16|RVcoll15G461||Switzerland|632[0n]
Spialia sertorius|LEATG409-14|TLMF Lep 14196||Italy|658[0n]
Spialia sertorius|LEATD018-13|TLMF Lep 12665||Italy|658[0n]
Spialia sertorius|ABOLB054-15|TLMF Lep 17059||Italy|658[0n]
Spialia sertorius|BIBSA005-14|LEP-SS-00005||Italy|658[0n]
Spialia sertorius|EULEP3289-15|RVcoll.12-Q587||Italy|658[0n]
Spialia sertorius|EULEP3276-15|RVcoll.12-M483||Italy|658[0n]
Spialia sertorius|WMB2811-13|RVcoll.12-M482||Italy|658[0n]
Spialia sertorius|EULEP3250-15|RVcoll.11-I198|Genit. examined|Italy|658[0n]
Spialia sertorius|WMB649-11|RVcoll.11-I197||Italy|658[0n]
Spialia sertorius|BIBSA1837-17|RVcoll16C712||Italy|658[0n]
Spialia sertorius|BIBSA605-15|LEP-SS-00225||Italy|658[0n]
Spialia sertorius|EULEP3363-15|RVcoll.15-A626||Italy|658[0n]
Spialia sertorius|EULEP3362-15|RVcoll.15-A596||Italy|658[0n]
Spialia sertorius|EULEP3361-15|RVcoll.15-A512||Italy|658[0n]
Spialia sertorius|EULEP3360-15|RVcoll.15-A508||Italy|658[0n]
Spialia sertorius|EULEP3171-15|RVcoll.07-E110|Genit. examined|Italy|658[0n]
Spialia sertorius|EULEP3291-15|RVcoll.13-S769||Italy|658[0n]
Spialia sertorius|EULEP5460-17|RVcoll11Y087||Italy|658[0n]
Spialia sertorius|WMB5283-14|RVcoll.14-A821||Italy|658[0n]
Spialia sertorius|WMB5200-14|RVcoll.13-T922||Italy|658[0n]
Spialia sertorius|WMB5047-14|RVcoll. 14-L178||Italy|658[0n]
Spialia sertorius|WMB723-12|RVcoll.11-I253|Genit. examined|Italy|658[0n]
Spialia sertorius|WMB711-12|RVcoll.11-H363|Genit. examined|Italy|658[0n]
Spialia sertorius|WMB4244-14|RVcoll.13-U013||Italy|658[0n]
Spialia sertorius|WMB2567-13|RVcoll.12-Q894||Italy|658[0n]
Spialia sertorius|WMB093-11|RVcoll.LD-3155||Italy|658[0n]
Spialia sertorius|WMB092-11|RVcoll.LD-3154||Italy|658[0n]
Spialia sertorius|WMB089-11|RVcoll.LD-3150||Italy|658[0n]

Spialia sertorius|WMB093-11|RVcoll.LD-3155||Italy|658[0n]
Spialia sertorius|WMB092-11|RVcoll.LD-3154||Italy|658[0n]
Spialia sertorius|WMB089-11|RVcoll.LD-3150||Italy|658[0n]
Spialia sertorius|WMB088-11|RVcoll.LD-3149|Elba|Italy|658[0n]
Spialia sertorius|GWORZ067-10|BC ZSM Lep 30423||Italy|658[0n]
Spialia sertorius|EULEP3195-15|RVcoll.09-V462|Genit. examined|Spain|658[0n]
Spialia sertorius|EULEP3251-15|RVcoll.11-I936|Genit. examined|France|658[0n]
Spialia sertorius|EULEP3325-15|RVcoll.14-B579||Spain|658[0n]
Spialia sertorius|EULEP3184-15|RVcoll.08-J022||Spain|658[0n]
Spialia sertorius|EULEP3310-15|RVcoll.13-S897||Spain|658[0n]
Spialia sertorius|EULEP3287-15|RVcoll.12-Q467||Spain|658[0n]
Spialia sertorius|EZSPM029-09|RVcoll.08-J812|Genit. examined|Spain|658[0n]
Spialia sertorius|EZSPM273-09|RVcoll.08-R492||Spain|658[0n]
Spialia sertorius|EULEP3188-15|RVcoll.08-L432||Spain|658[0n]
Spialia sertorius|EULEP249-14|RVcoll.12-Q418||Spain|658[0n]
Spialia sertorius|WMB3996-14|RVcoll.12-Q429||Spain|658[0n]
Spialia sertorius|EZSPN335-09|RVcoll.08-H263|Genit. examined|Spain|658[0n]
Spialia sertorius|EULEP3187-15|RVcoll.08-L076||Spain|658[0n]
Spialia sertorius|EZSPM878-12|Rvcoll.140410KV16||Spain|658[0n]
Spialia sertorius|EZSPN208-09|RVcoll.07-F059|Genit. examined|Spain|658[0n]
Spialia sertorius|EULEP3177-15|RVcoll.07-W066|Genit. examined|France|658[0n]
Spialia sertorius|WMB3960-14|RVcoll.12-P785||France|658[0n]
Spialia sertorius|WMB3906-14|RVcoll.12-P315||France|658[0n]
Spialia sertorius|WMB198-11|RVcoll.07-W044||France|658[0n]
Spialia sertorius|BIBSA278-15|RVcoll.14-E078||Italy|658[0n]
Spialia sertorius|EULEP3205-15|RVcoll.11-D601||Spain|658[0n]
Spialia sertorius|EULEP3314-15|RVcoll.14-A011||Spain|658[0n]
Spialia sertorius|EULEP3311-15|RVcoll.13-S898||Spain|658[0n]
Spialia sertorius|EULEP070-14|RVcoll.08-L065||Spain|610[0n]
Spialia sertorius|EULEP3262-15|RVcoll.12-L089||Spain|658[0n]
Spialia sertorius|EULEP3308-15|RVcoll.13-S895||Spain|658[0n]
Spialia sertorius|EULEP3207-15|RVcoll.11-D616|Genit. examined|Spain|658[0n]
Spialia sertorius|EULEP3206-15|RVcoll.11-D615|Genit. examined|Spain|658[0n]
Spialia sertorius|EULEP3309-15|RVcoll.13-S896||Spain|658[0n]
Spialia sertorius|EULEP3301-15|RVcoll.13-S884||Spain|658[0n]
Spialia sertorius|EULEP239-14|RVcoll.12-L610||Portugal|658[0n]
Spialia sertorius|WMB3196-14|RVcoll.08-J064||Spain|658[0n]
Spialia sertorius|EULEP255-14|RVcoll.12-Q459||Spain|658[0n]
Spialia sertorius|EULEP256-14|RVcoll.12-Q469||Spain|658[0n]
Spialia sertorius|EULEP3263-15|RVcoll.12-L090||Spain|658[0n]
Spialia sertorius|EULEP3266-15|RVcoll.12-L480||Spain|658[0n]
Spialia sertorius|EULEP3286-15|RVcoll.12-Q466||Spain|658[0n]
Spialia sertorius|EULEP3288-15|RVcoll.12-Q468||Spain|658[0n]
Spialia sertorius|EULEP3302-15|RVcoll.13-S885||Spain|658[0n]
Spialia sertorius|EULEP3303-15|RVcoll.13-S888||Spain|658[0n]
Spialia sertorius|EULEP3304-15|RVcoll.13-S891||Spain|658[0n]
Spialia sertorius|EULEP3305-15|RVcoll.13-S892||Spain|658[0n]
Spialia sertorius|EULEP3185-15|RVcoll.08-J042||Spain|658[0n]
Spialia sertorius|EULEP3201-15|RVcoll.09-X683||Spain|658[0n]
Spialia sertorius|EULEP3312-15|RVcoll.13-S899||Spain|658[0n]
Spialia sertorius|EULEP3315-15|RVcoll.14-A012||Spain|658[0n]
Spialia sertorius|EULEP3324-15|RVcoll.14-B576||Spain|658[0n]
Spialia sertorius|EULEP3331-15|RVcoll.14-N284||Spain|658[0n]
Spialia sertorius|EULEP3332-15|RVcoll.14-N285||Spain|658[0n]
Spialia sertorius|EULEP3333-15|RVcoll.14-N286||Spain|658[0n]
Spialia sertorius|EULEP3334-15|RVcoll.14-N287||Spain|658[0n]
Spialia sertorius|EULEP3335-15|RVcoll.14-N288||Spain|658[0n]
Spialia sertorius|EULEP3336-15|RVcoll.14-N289||Spain|658[0n]
Spialia sertorius|EULEP3337-15|RVcoll.14-N290||Spain|658[0n]
Spialia sertorius|EULEP3338-15|RVcoll.14-N291||Spain|658[0n]
Spialia sertorius|EULEP3339-15|RVcoll.14-N292||Spain|658[0n]
Spialia sertorius|EULEP3341-15|RVcoll.14-N294||Spain|658[0n]
Spialia sertorius|EULEP3342-15|RVcoll.14-N295||Spain|658[0n]
Spialia sertorius|EULEP3343-15|RVcoll.14-N296||Spain|658[0n]
Spialia sertorius|EULEP3353-15|RVcoll.14-V491||Spain|658[0n]
Spialia sertorius|EULEP3354-15|RVcoll.14-V492||Spain|658[0n]
Spialia sertorius|EULEP3356-15|RVcoll.14-V494||Spain|658[0n]
Spialia sertorius|EULEP006-14|RVcoll.06-A264||Spain|647[0n]
Spialia sertorius|WMB4453-14|RVcoll.14-D319||Spain|658[0n]
Spialia sertorius|WMB3258-14|RVcoll.08-L496||Spain|658[0n]
Spialia sertorius|EZSPC1259-10|RVcoll.030908SF81||Spain|658[0n]
Spialia sertorius|EZSPM880-12|Rvcoll.150310ZX95||Spain|658[0n]
Spialia sertorius|EZSPM868-12|Rvcoll.090211SD27||Spain|658[0n]
Spialia sertorius|EULEP3166-15|RVcoll.07-C503||Germany|658[0n]
Spialia sertorius|EULEP3172-15|RVcoll.07-E202||Italy|658[0n]
Spialia sertorius|EULEP3176-15|RVcoll.07-W043||France|658[0n]
Spialia sertorius|EULEP3181-15|RVcoll.08-H548||Spain|658[0n]
Spialia sertorius|EULEP3189-15|RVcoll.08-P721||Spain|658[0n]
Spialia sertorius|EULEP3199-15|RVcoll.09-X575||Spain|658[0n]
Spialia sertorius|EULEP2153-15|RVcoll.14-K060||Switzerland|658[0n]
Spialia sertorius|BIBSA927-15|16-A018||Italy|658[0n]
Spialia sertorius|GBLAB159-13|BC ZSM Lep 75785||Germany|658[0n]
Spialia sertorius|GBLAA1409-15|BC ZSM Lep 87105||Germany|658[0n]
Spialia sertorius|LEASS924-17|KLM Lep 08429||Austria|658[0n]
Spialia sertorius|LEASS752-17|TLMF Lep 22400||Austria|658[0n]
Spialia sertorius|LEASS753-17|TLMF Lep 22401||Austria|658[0n]
Spialia sertorius|LEASS770-17|TLMF Lep 22418||Austria|658[0n]
Spialia sertorius|GWORA2882-15|BC ZSM Lep 86150||Germany|638[0n]
Spialia sertorius|GWORA2990-15|BC ZSM Lep 86068||Germany|632[0n]
Spialia sertorius|EULEP3158-15|RVcoll.06-A029|Genit. examined|Spain|638[0n]
Spialia sertorius|EULEP3285-15|RVcoll.12-Q464||Spain|658[0n]
Spialia sertorius|EULEP3284-15|RVcoll.12-Q463||Spain|658[0n]
Spialia sertorius|EULEP3281-15|RVcoll.12-Q217||France|658[0n]
Spialia sertorius|EULEP3280-15|RVcoll.12-P219||France|658[0n]
Spialia sertorius|EULEP3274-15|RVcoll.12-L493||Spain|658[0n]
Spialia sertorius|EULEP3273-15|RVcoll.12-L492||Spain|658[0n]
Spialia sertorius|EULEP3272-15|RVcoll.12-L491||Spain|658[0n]
Spialia sertorius|EULEP3271-15|RVcoll.12-L490||Spain|658[0n]
Spialia sertorius|EULEP3270-15|RVcoll.12-L489||Spain|658[0n]
Spialia sertorius|EULEP3269-15|RVcoll.12-L485||Spain|658[0n]
Spialia sertorius|EULEP3268-15|RVcoll.12-L484||Spain|658[0n]
Spialia sertorius|EULEP3267-15|RVcoll.12-L483||Spain|658[0n]
Spialia sertorius|EULEP3265-15|RVcoll.12-L477||Spain|658[0n]

Spialia sertorius|EULEP3269-15|RVcoll.12-L48[illegible]||Spain|658[0n]
Spialia sertorius|EULEP3268-15|RVcoll.12-L484||Spain|658[0n]
Spialia sertorius|EULEP3267-15|RVcoll.12-L483||Spain|658[0n]
Spialia sertorius|EULEP3265-15|RVcoll.12-L477||Spain|658[0n]
Spialia sertorius|EULEP3259-15|RVcoll.12-L071||Spain|658[0n]
Spialia sertorius|EULEP011-14|RVcoll.06-J013||Spain|658[0n]
Spialia sertorius|WMB5146-14|RVcoll.11-I601||France|658[0n]
Spialia sertorius|WMB4944-14|RVcoll. 14-I491||Italy|658[0n]
Spialia sertorius|WMB732-12|RVcoll.11-I701|Genit. examined|France|658[0n]
Spialia sertorius|WMB3936-14|RVcoll.12-P568||France|658[0n]
Spialia sertorius|WMB3931-14|RVcoll.12-P533||France|658[0n]
Spialia sertorius|WMB3326-14|RVcoll.08-P745||Spain|658[0n]
Spialia sertorius|EZSPM074-09|RVcoll.08-P318|Genit. examined|Spain|658[0n]
Spialia sertorius|EZSPN194-09|RVcoll.07-C499|Genit. examined|Spain|658[0n]
Spialia sertorius|EZSPN422-09|RVcoll.08-H578|Genit. examined|Spain|658[0n]
Spialia sertorius|EZSPN160-09|RVcoll.06-J014|Genit. examined|Spain|658[0n]
Spialia sertorius|EZSPN115-09|RVcoll.06-A281|Genit. examined|Spain|658[0n]
Spialia sertorius|GWOSK779-11|BC ZSM Lep 48995||Germany|658[0n]
Spialia sertorius|GWOSK776-11|BC ZSM Lep 48992||Germany|658[0n]
Spialia sertorius|GWOSK774-11|BC ZSM Lep 48990||Germany|658[0n]
Spialia sertorius|FBLMU508-09|BC ZSM Lep 27158||Germany|658[0n]
Spialia sertorius|FBLMW327-10|BC ZSM Lep 37428||Germany|658[0n]
Spialia sertorius|LEATG030-14|TLMF Lep 13817||Austria|658[0n]
Spialia sertorius|PHLAW040-13|TLMF Lep 09837||Austria|658[0n]
Spialia sertorius|FBLMX121-11|BC ZSM Lep 37602||Germany|658[0n]
Spialia sertorius|FBLMW328-10|BC ZSM Lep 37429||Germany|658[0n]
Spialia sertorius|GWOTF688-12|BC ZSM Lep 62394||Germany|658[0n]
Spialia sertorius|ABOLD092-16|TLMF Lep 21170||Austria|658[0n]
Carcharodus tripolinus|EZSPN1096-11|Rvcoll.10-A015||Spain|658[0n]
Carcharodus tripolinus|EULEP158-14|RVcoll.11-D638||Portugal|658[0n]
Carcharodus tripolinus|EZSPM1012-12|Rvcoll.12-L058||Portugal|658[0n]
Carcharodus tripolinus|EULEP2692-15|RVcoll.14-N776||Spain|658[0n]
Carcharodus tripolinus|EULEP2691-15|RVcoll.14-N775||Spain|658[0n]
Carcharodus tripolinus|EULEP159-14|RVcoll.11-D639||Portugal|658[0n]
Carcharodus tripolinus|EULEP157-14|RVcoll.11-D637||Portugal|658[0n]
Carcharodus tripolinus|EZSPN518-09|RVcoll.08-H983||Spain|658[0n]
Carcharodus tripolinus|EZSPN517-09|RVcoll.08-H982||Spain|658[0n]
Carcharodus tripolinus|EZSPM673-12|Rvcoll. 12-L544||Portugal|658[0n]
Carcharodus tripolinus|EZSPM671-12|Rvcoll. 12-L054||Spain|658[0n]
Carcharodus alceae|EULEP2378-15|RVcoll.14-V073||Ukraine|611[0n]
Carcharodus alceae|EULEP4942-16|RVcoll16I112||Poland|658[0n]
Carcharodus alceae|EULEP1187-15|RVcoll.14-F455||Bulgaria|658[0n]
Carcharodus alceae|EULEP1208-15|RVcoll.14-F504||Greece|658[0n]
Carcharodus alceae|EULEP942-15|RVcoll.14-C932||Bulgaria|658[0n]
Carcharodus alceae|EULEP4851-16|RVcoll16H777||Bulgaria|637[0n]
Carcharodus alceae|EZROM077-08|RV-07-E531|Genit. examined|Romania|658[0n]
Carcharodus alceae|EZROM611-08|RV-07-E523||Romania|658[0n]
Carcharodus alceae|EZROM829-08|RVcoll.08-M220|Genit. examined|Romania|658[0n]
Carcharodus alceae|OXB686-15|12-M380|Sicily W|Italy|658[0n]
Carcharodus alceae|WMB508-11|RVcoll.11-H592|Sicily|Italy|658[0n]
Carcharodus alceae|WMB075-11|RVcoll.LD-2990|Sicily|Italy|658[0n]
Carcharodus alceae|BIBSA1540-16|11-D216|Sicily|Italy|658[0n]
Carcharodus alceae|WMB070-11|RVcoll.LD-2786|Sicily|Italy|658[0n]
Carcharodus alceae|WMB5460-14|RVcoll.14-N605|Levanzo|Italy|632[0n]
Carcharodus alceae|WMB600-11|RVcoll.11-H970|Sicily|Italy|658[0n]
Carcharodus alceae|WMB071-11|RVcoll.LD-2854|Sicily|Italy|658[0n]
Carcharodus alceae|WMB060-11|RVcoll.LD-2596|Sicily|Italy|658[0n]
Carcharodus alceae|WMB059-11|RVcoll.LD-2587|Sicily|Italy|658[0n]
Carcharodus alceae|WMB057-11|RVcoll.LD-2568|Lipari|Italy|658[0n]
Carcharodus alceae|WMB166-11|RVcoll.11-D380|Sicily|Italy|658[0n]
Carcharodus alceae|OXB701-15|13-T874||Italy|658[0n]
Carcharodus alceae|EULEP1785-15|RVcoll.14-H620||Greece|658[0n]
Carcharodus alceae|EULEP1060-15|RVcoll.14-E961||Serbia|658[0n]
Carcharodus alceae|WMB3881-14|RVcoll.12-M515||Italy|658[0n]
Carcharodus alceae|WMB3905-14|RVcoll.12-P301||France|658[0n]
Carcharodus alceae|WMB1667-13|RVcoll.12-P227||France|624[0n]
Carcharodus alceae|WMB3690-14|RVcoll.11-J243||Spain|658[0n]
Carcharodus alceae|EZSPN537-09|RVcoll.08-J023||Spain|658[0n]
Carcharodus alceae|EZSPM936-12|Rvcoll.12-M670||Spain|658[0n]
Carcharodus alceae|EZSPN136-09|RVcoll.06-G495|Genit. examined|Spain|658[0n]
Carcharodus alceae|EULEP704-15|RVcoll.12-M178|Crete|Greece|658[1n]
Carcharodus alceae|EULEP713-15|RVcoll.12-M247||Greece|658[0n]
Carcharodus alceae|WMB2615-13|RVcoll.12-O180|Corsica|France|658[0n]
Carcharodus alceae|WMB657-11|RVcoll.11-I289|Pianosa|Italy|658[0n]
Carcharodus alceae|BIBSA1293-15|14-E284|Corsica|France|612[0n]
Carcharodus alceae|WMB1985-13|RVcoll.11-I290|Pianosa|Italy|639[0n]
Carcharodus alceae|WMB1509-13|RVcoll.12-O095|Corsica|France|658[0n]
Carcharodus alceae|WMB2782-13|RVcoll.11-I291|Pianosa|Italy|658[0n]
Carcharodus alceae|WMB021-11|RVcoll.LD-691||France|658[0n]
Carcharodus alceae|WMB017-11|RVcoll.LD-396||France|658[0n]
Carcharodus alceae|EULEP1760-15|RVcoll.14-H424||Greece|658[0n]
Carcharodus alceae|EULEP1569-15|RVcoll.14-G572||Macedonia|658[0n]
Carcharodus alceae|EULEP1004-15|RVcoll.14-G495||Greece|658[0n]
Carcharodus alceae|FBLMZ166-12|BC ZSM Lep 51422||Germany|658[0n]
Carcharodus alceae|EZSPN984-09|RVcoll.08-M024||Spain|658[0n]
Carcharodus alceae|EZSPN107-09|RVcoll.06-A254||Spain|658[0n]
Carcharodus alceae|EZSPN126-09|RVcoll.06-A315||Spain|658[0n]
Carcharodus alceae|EZSPN413-09|RVcoll.08-H530||Spain|658[0n]
Carcharodus alceae|EZSPN433-09|RVcoll.08-H611||Spain|658[0n]
Carcharodus alceae|EZSPC203-09|RVcoll.07-C498||Spain|655[0n]
Carcharodus alceae|EZROM711-08|RV-06-H853||Spain|658[0n]
Carcharodus alceae|EZSPN229-09|RVcoll.07-W032||Spain|658[0n]
Carcharodus alceae|EZSPN281-09|RVcoll.07-W216||Spain|658[0n]
Carcharodus alceae|EZSPN545-09|RVcoll.08-J046||Spain|658[0n]
Carcharodus alceae|EZSPM360-09|RVcoll.08-R026||Spain|658[0n]
Carcharodus alceae|EZSPM368-09|RVcoll.08-J837||Spain|658[0n]
Carcharodus alceae|EZSPN332-09|RVcoll.08-H257||Spain|658[0n]
Carcharodus alceae|EZSPC1071-10|RVcoll.08-L283||Spain|658[0n]
Carcharodus alceae|WMB016-11|RVcoll.LD-360||Italy|658[0n]
Carcharodus alceae|WMB2981-14|RVcoll.12-O174|Corsica|France|658[0n]
Carcharodus alceae|WMB2993-14|RVcoll.12-O354|Sardinia|Italy|658[0n]
Carcharodus alceae|WMB3054-14|RVcoll.13-T683|Sardinia|Italy|658[0n]
Carcharodus alceae|WMB3269-14|RVcoll.08-L910||Spain|658[0n]

Carcharodus alceae|WMB2993-14|RVcoll.12-O354|Sardinia|Italy|658[0n]
Carcharodus alceae|WMB3054-14|RVcoll.13-T683|Sardinia|Italy|658[0n]
Carcharodus alceae|WMB3269-14|RVcoll.08-L910||Spain|658[0n]
Carcharodus alceae|WMB3314-14|RVcoll.08-P497||Spain|658[0n]
Carcharodus alceae|WMB1570-13|RVcoll.12-O506|Sardinia|Italy|658[0n]
Carcharodus alceae|WMB4393-14|RVcoll.14-B338||Portugal|658[0n]
Carcharodus alceae|WMB312-11|RVcoll.11-E447||Italy|658[0n]
Carcharodus alceae|WMB336-11|RVcoll.11-E674||Italy|658[0n]
Carcharodus alceae|EULEP026-14|RVcoll.07-D759||Spain|658[0n]
Carcharodus alceae|EULEP1602-15|RVcoll.14-G655||Greece|658[0n]
Carcharodus alceae|EULEP1493-15|RVcoll.14-G327||Greece|658[0n]
Carcharodus alceae|EULEP1674-15|RVcoll.14-G926||Greece|658[0n]
Carcharodus alceae|BIBSA1322-15|12-O560|Sardinia|Italy|658[0n]
Carcharodus alceae|ABOLD628-17|TLMF Lep 21704||Austria|658[0n]
Carcharodus alceae|EZSPC195-09|RVcoll.06-G462|Genit. examined|Spain|645[0n]
Carcharodus alceae|OXB953-15|15-A903||Italy|613[0n]
Carcharodus alceae|WMB1536-13|RVcoll.12-O276|La Maddalena|Italy|632[0n]
Carcharodus alceae|EZSPC194-09|RVcoll.06-G440|Genit. examined|Spain|633[0n]
Carcharodus alceae|EZROM073-08|RV-07-D139||Romania|658[0n]
Carcharodus alceae|EULEP1457-15|RVcoll.14-G190||Greece|658[0n]
Carcharodus alceae|EULEP850-15|RVcoll.14-C134||Greece|624[0n]
Carcharodus alceae|EZROM305-08|RV-07-E667||Romania|602[0n]
Carcharodus alceae|EZROM837-08|RVcoll.08-M402|Genit. examined|Romania|658[0n]
Carcharodus alceae|FBLMT405-09|BC ZSM Lep 24965||Germany|658[0n]
Carcharodus alceae|WMB2874-14|RVcoll.10-C570|Argentario|Italy|658[0n]
Carcharodus alceae|WMB029-11|RVcoll.LD-995|Elba|Italy|658[0n]
Carcharodus alceae|WMB3697-14|RVcoll.11-J552||Spain|658[0n]
Carcharodus alceae|WMB3831-14|RVcoll.09-X913||Italy|658[0n]
Carcharodus alceae|WMB4018-14|RVcoll.12-R444||Italy|658[0n]
Carcharodus alceae|WMB6056-18|RVcoll16L070||Italy|658[0n]
Carcharodus alceae|WMB5073-14|RVcoll. 14-L204||Italy|658[0n]
Carcharodus alceae|WMB4760-14|RVcoll. 14-I307||Italy|658[0n]
Carcharodus alceae|WMB5042-14|RVcoll. 14-L173||Italy|658[0n]
Carcharodus alceae|EULEP3615-16|RVcoll15J200||Switzerland|658[0n]
Carcharodus alceae|EULEP5062-16|RVcoll16J115||Czech Republic|658[0n]
Carcharodus alceae|EULEP5702-17|RVcoll16L045||Italy|658[0n]
Carcharodus alceae|EULEP1277-15|RVcoll.14-F682||Greece|658[0n]
Carcharodus alceae|EULEP1302-15|RVcoll.14-F740||Greece|658[0n]
Carcharodus alceae|EULEP1655-15|RVcoll.14-G854||Greece|658[0n]
Carcharodus alceae|BIBSA1384-15|15-M980||Italy|658[0n]
Carcharodus alceae|LEASS1042-17|TLMF Lep 22595||Austria|658[0n]
Carcharodus alceae|EZSPC232-09|RVcoll.08-M714||Spain|654[0n]
Carcharodus alceae|EZROM074-08|RV-07-E428||Romania|658[0n]
Carcharodus alceae|EZROM300-08|RV-07-E662||Romania|658[0n]
Carcharodus alceae|EZROM828-08|RVcoll.08-M430||Romania|658[0n]
Carcharodus alceae|EZROM827-08|RVcoll.08-M413||Romania|658[0n]
Carcharodus alceae|EZROM826-08|RVcoll.08-M261||Romania|658[0n]
Carcharodus alceae|WMB3082-14|RVcoll.14-D949|Giglio|Italy|658[0n]
Carcharodus alceae|BIBSA992-15|15-C018||Italy|658[0n]
Carcharodus alceae|OXB666-15|07-D802||Italy|658[0n]
Carcharodus alceae|OXB856-15|15-A500||Italy|630[0n]
Carcharodus alceae|LEASS669-17|TLMF Lep 22317||Austria|658[0n]
Carcharodus alceae|LEASS1055-17|TLMF Lep 22608||Austria|658[0n]
Carcharodus alceae|LEATJ1218-16|TLMF Lep 19541||Croatia|658[0n]
Carcharodus alceae|LEATJ1217-16|TLMF Lep 19540||Croatia|658[0n]
Carcharodus alceae|BIBSA009-14|LEP-SS-00009||Italy|658[0n]
Carcharodus alceae|BIBSA008-14|LEP-SS-00008||Italy|658[0n]
Carcharodus alceae|BIBSA1622-16|15-C424||Italy|658[0n]
Carcharodus alceae|BIBSA127-15|RVcoll.14-D582||Italy|658[0n]
Carcharodus alceae|BIBSA953-15|15-C283||Italy|658[0n]
Carcharodus alceae|BIBSA1208-15|15-M325||Italy|658[0n]
Carcharodus alceae|EULEP2167-15|RVcoll.14-K089||Switzerland|658[0n]
Carcharodus alceae|EULEP1976-15|RVcoll.14-H896||France|658[0n]
Carcharodus alceae|EULEP5688-17|RVcoll16L031||Italy|658[0n]
Carcharodus alceae|EULEP5641-17|RVcoll15D997|Gorgona|Italy|658[0n]
Carcharodus alceae|EULEP3616-16|RVcoll15J673||France|658[0n]
Carcharodus alceae|WMB5246-14|RVcoll.14-A162||Italy|658[0n]
Carcharodus alceae|WMB5361-14|RVcoll.14-J719||France|658[0n]
Carcharodus alceae|WMB5090-14|RVcoll. 14-L221||Italy|658[0n]
Carcharodus alceae|WMB4476-14|RVcoll.14-D915||Italy|658[0n]
Carcharodus alceae|WMB4327-14|RVcoll.14-A678||Italy|658[0n]
Carcharodus alceae|WMB4326-14|RVcoll.14-A664||Italy|658[0n]
Carcharodus alceae|WMB3947-14|RVcoll.12-P703||France|658[0n]
Carcharodus alceae|WMB3909-14|RVcoll.12-P331||France|658[0n]
Carcharodus alceae|WMB2616-13|RVcoll.12-P974|Levant|France|658[0n]
Carcharodus alceae|WMB3818-14|RVcoll.07-E100||Italy|658[0n]
Carcharodus alceae|WMB2343-13|RVcoll.12-M481||Italy|658[0n]
Carcharodus alceae|WMB2342-13|RVcoll.12-M480||Italy|658[0n]
Carcharodus alceae|WMB1754-13|RVcoll.12-P972|Levant|France|658[0n]
Carcharodus alceae|WMB1719-13|RVcoll.12-P715||France|658[0n]
Carcharodus alceae|WMB1502-13|RVcoll.12-O054||Italy|658[0n]
Carcharodus alceae|WMB3156-14|RVcoll.07-W153||Spain|658[0n]
Carcharodus alceae|WMB3030-14|RVcoll.13-S531|San Domino|Italy|658[0n]
Carcharodus alceae|WMB3017-14|RVcoll.12-R357|Elba|Italy|658[0n]
Carcharodus alceae|WMB2968-14|RVcoll.12-O055|Argentario|Italy|658[0n]
Carcharodus alceae|WMB091-11|RVcoll.LD-3153||Italy|658[0n]
Carcharodus alceae|WMB086-11|RVcoll.LD-3147|Elba|Italy|658[0n]
Carcharodus alceae|WMB085-11|RVcoll.LD-3146||Italy|658[0n]
Carcharodus alceae|WMB067-11|RVcoll.LD-2637|Ischia|Italy|658[0n]
Carcharodus alceae|WMB066-11|RVcoll.LD-2614|Elba|Italy|658[0n]
Carcharodus alceae|WMB196-11|RVcoll.07-W039||France|658[0n]
Carcharodus alceae|EZSPN209-09|RVcoll.07-F061||Spain|658[0n]
Carcharodus alceae|LEATG397-14|TLMF Lep 14184||Italy|658[0n]
Muschampia cribrellum|EULEP4310-16|RVcoll14A940||Russia|658[0n]
Muschampia cribrellum|EZRMN131-08|RVcoll.08-M298||Romania|658[0n]
Muschampia cribrellum|EULEP4309-16|RVcoll14A973||Bulgaria|658[0n]
Muschampia cribrellum|EULEP2996-15|RVcoll.14-O129||Macedonia|658[0n]
Muschampia cribrellum|EULEP2995-15|RVcoll.14-O128||Macedonia|658[0n]
Muschampia cribrellum|EULEP5111-17|RVcoll16J866||Macedonia|658[0n]
Muschampia cribrellum|EULEP5110-17|RVcoll16J865||Greece|658[0n]
Muschampia cribrellum|EULEP5109-17|RVcoll16J864||Greece|658[0n]
Muschampia cribrellum|EULEP4308-16|RVcoll15Q006||Macedonia|658[0n]
Muschampia cribrellum|EZRMN130-08|RVcoll.08-M297|Genit. examined|Romania|658[0n]

[illegible]
Muschampia cribrellum|EULEP5109-17|RVcoll16J864||Greece|658[0n]
Muschampia cribrellum|EULEP4308-16|RVcoll15Q006||Macedonia|658[0n]
Muschampia cribrellum|EZRMN130-08|RVcoll.08-M297|Genit. examined|Romania|658[0n]
Muschampia cribrellum|EZRMN129-08|RVcoll.08-M296|Genit. examined|Romania|658[0n]
Muschampia cribrellum|EZROM433-08|RV-07-D402||Romania|658[0n]
Muschampia cribrellum|EZROM432-08|RV-07-D401||Romania|658[0n]
Muschampia cribrellum|EZRMN132-08|RVcoll.08-M299||Romania|658[0n]
Muschampia proto|EULEP4312-16|RVcoll15Q178||Ukraine|658[0n]
Muschampia proto|EULEP4314-16|RVcoll15Q182||Ukraine|658[0n]
Muschampia proto|EULEP4831-16|RVcoll16H732||Ukraine|658[0n]
Muschampia proto|EULEP4313-16|RVcoll15Q180||Ukraine|637[0n]
Muschampia proto|EULEP3003-15|RVcoll.14-O136|Symi|Greece|658[0n]
Muschampia proto|EULEP3002-15|RVcoll.14-O135|Symi|Greece|658[0n]
Muschampia proto|EULEP4311-16|RVcoll14O308|Tilos|Greece|658[0n]
Muschampia proto|BIBSA736-15|RVcoll. 14-U895||Italy|658[0n]
Muschampia proto|BIBSA735-15|RVcoll. 14-D742||Italy|658[0n]
Muschampia proto|EULEP1456-15|RVcoll.14-G189||Greece|658[0n]
Muschampia proto|EULEP1470-15|RVcoll.14-G237||Greece|658[0n]
Muschampia proto|EULEP1464-15|RVcoll.14-G225||Greece|658[0n]
Muschampia proto|EULEP1454-15|RVcoll.14-G182||Greece|658[0n]
Muschampia proto|EULEP1598-15|RVcoll.14-G647||Greece|658[0n]
Muschampia proto|EULEP3004-15|RVcoll.14-O137||Albania|658[0n]
Muschampia proto|EULEP1596-15|RVcoll.14-G645||Greece|658[0n]
Muschampia proto|WMB2115-13|RVcoll.12-R185|Sicily|Italy|658[0n]
Muschampia proto|WMB2114-13|RVcoll.12-R184|Sicily|Italy|658[0n]
Muschampia proto|EZSPC988-10|RVcoll.07-C699||Spain|658[0n]
Muschampia proto|EZSPN215-09|RVcoll.07-F074||Spain|658[0n]
Muschampia proto|WMB4423-14|RVcoll.14-B477||Portugal|658[0n]
Muschampia proto|WMB4406-14|RVcoll.14-B438||Portugal|658[0n]
Muschampia proto|WMB4461-14|RVcoll.14-D331||Spain|658[0n]
Muschampia proto|EZSPC1059-10|RVcoll.08-L046||Spain|658[0n]
Muschampia proto|EULEP155-14|RVcoll.11-D635||Spain|658[0n]
Muschampia proto|WMB3344-14|RVcoll.08-R033||Spain|658[0n]
Muschampia proto|EZSPC1151-10|RVcoll.08-R007||Spain|658[0n]
Muschampia proto|WMB3157-14|RVcoll.07-W253||Spain|658[0n]
Muschampia proto|WMB926-13|RVcoll.10-A234||France|658[0n]
Muschampia proto|EULEP154-14|RVcoll.11-D634||Portugal|658[0n]
Muschampia proto|EZSPN543-09|RVcoll.08-J039||Spain|623[0n]
Muschampia proto|EZSPM965-12|Rvcoll.12-M717||Spain|658[0n]
Muschampia proto|EZSPN293-09|RVcoll.07-W265||Spain|658[0n]
Muschampia proto|EZSPM111-09|RVcoll.08-P453|Genit. examined|Spain|658[0n]
Muschampia proto|EULEP156-14|RVcoll.11-D636||Portugal|658[0n]
Muschampia proto|EZSPM940-12|Rvcoll.12-M674||Spain|658[0n]
Muschampia proto|EZSPC973-10|RVcoll.06-A320||Spain|658[0n]
Muschampia proto|EZSPM966-12|Rvcoll.12-M718||Spain|658[0n]
Muschampia proto|EZSPN240-09|RVcoll.07-W094||Spain|658[0n]
Muschampia proto|WMB115-11|RVcoll.10-B664||France|658[0n]
Muschampia proto|WMB5152-14|RVcoll.12-P284||France|658[0n]
Muschampia proto|WMB1722-13|RVcoll.12-P783||France|658[0n]
Muschampia proto|WMB3287-14|RVcoll.08-M689||Spain|658[0n]
Muschampia proto|EZSPC1137-10|RVcoll.08-P726||Spain|658[0n]
Muschampia proto|EZSPC1018-10|RVcoll.09-V505||Spain|658[0n]
Muschampia proto|EZSPC1214-10|RVcoll.09-V510||Spain|658[0n]
Muschampia proto|EZSPM731-12|Rvcoll. 12-L587||Portugal|658[0n]
Muschampia proto|EZSPN185-09|RVcoll.07-C087||Spain|658[0n]
Muschampia tessellum|EULEP2552-15|RVcoll.14-V471||Greece|658[0n]
Muschampia tessellum|EULEP3001-15|RVcoll.14-O134||Macedonia|658[0n]
Muschampia tessellum|EULEP3000-15|RVcoll.14-O133||Macedonia|658[0n]
Muschampia tessellum|EULEP4316-16|RVcoll15G029||Greece|658[0n]
Muschampia tessellum|EULEP2553-15|RVcoll.14-V472||Greece|658[0n]
Muschampia tessellum|EULEP4320-16|RVcoll14A978||Bulgaria|658[0n]
Muschampia tessellum|EULEP4321-16|RVcoll14A979||Bulgaria|658[0n]
Muschampia tessellum|EULEP2333-15|RVcoll.14-V598||Russia|658[0n]
Muschampia tessellum|EULEP4319-16|RVcoll15Q068||Russia|658[0n]
Muschampia tessellum|EULEP2567-15|RVcoll.14-V542||Ukraine|658[0n]
Muschampia tessellum|EULEP2568-15|RVcoll.14-V543||Ukraine|658[0n]
Muschampia tessellum|EULEP2587-15|RVcoll.14-V597||Russia|658[0n]
Muschampia tessellum|EULEP4315-16|RVcoll15G028||Greece|632[0n]
Muschampia tessellum|EULEP4318-16|RVcoll15Q176||Ukraine|619[0n]
Muschampia tessellum|EULEP4317-16|RVcoll15Q173||Ukraine|623[0n]
Muschampia tessellum|EZROM435-08|RV-07-C126||Romania|658[0n]
Muschampia tessellum|EZROM434-08|RV-07-C125||Romania|658[0n]
Muschampia tessellum|EZRMN133-08|RVcoll.08-M668||Romania|658[0n]
Muschampia tessellum|EZRMN335-08|RVcoll.08-H007||Romania|658[0n]
Carcharodus lavatherae|EZROM713-08|RV-06-G459||Spain|658[0n]
Carcharodus lavatherae|EZSPC259-09|RVcoll.08-P365||Spain|655[0n]
Carcharodus lavatherae|EZSPC263-09|RVcoll.08-R111||Spain|658[0n]
Carcharodus lavatherae|EZSPN940-09|RVcoll.08-L912||Spain|658[0n]
Carcharodus lavatherae|EZSPN996-09|RVcoll.08-M071||Spain|658[0n]
Carcharodus lavatherae|EZSPC933-10|RVcoll.06-A255.1||Spain|658[0n]
Carcharodus lavatherae|EZSPC1207-10|RVcoll.09-V475||Spain|658[0n]
Carcharodus lavatherae|EZSPC1003-10|RVcoll.08-H286||Spain|658[0n]
Carcharodus lavatherae|EZSPC1023-10|RVcoll.08-J017||Spain|658[0n]
Carcharodus lavatherae|EZSPC1186-10|RVcoll.09-T176||Spain|658[0n]
Carcharodus lavatherae|EZSPC220-09|RVcoll.08-L435|Genit. examined|Spain|627[0n]
Carcharodus lavatherae|EZSPC210-09|RVcoll.08-H285||Spain|655[0n]
Carcharodus lavatherae|EZSPN117-09|RVcoll.06-A289||Spain|658[0n]
Carcharodus lavatherae|EULEP3619-16|RVcoll15Q165||Ukraine|623[0n]
Carcharodus lavatherae|EULEP1512-15|RVcoll.14-G431||Greece|658[0n]
Carcharodus lavatherae|EZROM311-08|RV-07-E673||Romania|658[0n]
Carcharodus lavatherae|EULEP3621-16|RVcoll15Q041||Serbia|658[0n]
Carcharodus lavatherae|EULEP500-15|RVcoll.07-C513||Greece|658[0n]
Carcharodus lavatherae|EULEP1499-15|RVcoll.14-G371||Greece|658[0n]
Carcharodus lavatherae|EZROM304-08|RV-07-E666||Romania|646[0n]
Carcharodus lavatherae|EZROM834-08|Rvcoll.08-M711||Romania|658[0n]
Carcharodus lavatherae|EZROM833-08|RVcoll.08-M521||Romania|658[0n]
Carcharodus lavatherae|EULEP3617-16|RVcoll10A967||Bulgaria|658[0n]
Carcharodus lavatherae|ABOLD627-17|TLMF Lep 21703||Austria|658[0n]
Carcharodus lavatherae|EULEP1615-15|RVcoll.14-G684||Greece|614[0n]
Carcharodus lavatherae|EULEP3620-16|RVcoll15Q167||Ukraine|622[0n]
Carcharodus lavatherae|EULEP3618-16|RVcoll15G603||Switzerland|636[0n]
Carcharodus lavatherae|WMB831-13|RVcoll.07-E213||Italy|636[0n]
[illegible]

Carcharodus lavatherae|EULEP3620-16|RVcoll15Q167||Ukraine|622[0n]
Carcharodus lavatherae|EULEP3618-16|RVcoll15G603||Switzerland|636[0n]
Carcharodus lavatherae|WMB831-13|RVcoll.07-E213||Italy|636[0n]
Carcharodus lavatherae|WMB796-13|RVcoll.07-D881||Italy|658[0n]
Carcharodus lavatherae|LEATG402-14|TLMF Lep 14189||Italy|658[0n]
Carcharodus stauderi|EULEP2688-15|RVcoll.14-N772|Symi|Greece|614[2n]
Carcharodus stauderi|EULEP2687-15|RVcoll.14-N771|Symi|Greece|658[0n]
Carcharodus stauderi|EULEP673-15|RVcoll.11-J507|Tilos|Greece|658[0n]
Carcharodus stauderi|EULEP672-15|RVcoll.11-J506|Tilos|Greece|658[0n]
Carcharodus stauderi|EULEP671-15|RVcoll.11-J505|Tilos|Greece|658[0n]
Carcharodus stauderi|EULEP670-15|RVcoll.11-J504|Tilos|Greece|658[0n]
Carcharodus baeticus|WMB3242-14|RVcoll.08-L148||Spain|620[0n]
Carcharodus baeticus|EULEP257-14|RVcoll.12-Q527||Spain|658[0n]
Carcharodus baeticus|EULEP5527-17|RVcoll14A901||Italy|628[0n]
Carcharodus baeticus|WMB3192-14|RVcoll.08-J051||Spain|658[0n]
Carcharodus baeticus|WMB3189-14|RVcoll.08-J027||Spain|658[0n]
Carcharodus baeticus|WMB3392-14|RVcoll.09-V563||Spain|658[0n]
Carcharodus baeticus|EZSPC989-10|RVcoll.09-V456||Spain|658[0n]
Carcharodus baeticus|EZSPC969-10|RVcoll.08-L911||Spain|658[0n]
Carcharodus baeticus|EZSPN1046-11|Rvcoll.08-L174||Spain|658[0n]
Carcharodus baeticus|EZSPN1031-11|Rvcoll.06-A270||Spain|658[0n]
Carcharodus baeticus|EZSPM955-12|Rvcoll.12-M706||Spain|658[0n]
Carcharodus baeticus|EZSPN806-09|RVcoll.08-L470|Genit. examined|Spain|658[0n]
Carcharodus baeticus|EZSPN158-09|RVcoll.06-J003||Spain|658[0n]
Carcharodus baeticus|WMB4465-14|RVcoll.14-D338||Spain|658[0n]
Carcharodus baeticus|WMB3149-14|RVcoll.07-F018||Spain|658[0n]
Carcharodus baeticus|EZSPC976-10|RVcoll.09-V453||Spain|658[0n]
Carcharodus baeticus|EZSPN141-09|RVcoll.06-G506||Spain|658[0n]
Carcharodus floccifera|EZSPN283-09|RVcoll.07-W219||Spain|658[0n]
Carcharodus floccifera|BIBSA1638-16|15-C503||Italy|658[0n]
Carcharodus floccifera|EZROM325-08|RV-08-A003|Genit. examined|Romania|658[0n]
Carcharodus floccifera|WMB079-11|RVcoll.LD-3106|Sicily|Italy|658[0n]
Carcharodus floccifera|WMB4267-14|RVcoll.14-A168||Italy|658[0n]
Carcharodus floccifera|WMB502-11|RVcoll.11-H560|Sicily|Italy|658[0n]
Carcharodus floccifera|WMB6602-18|RVcoll14W002|Sicily|Italy|658[0n]
Carcharodus floccifera|WMB596-11|RVcoll.11-H940|Sicily|Italy|658[0n]
Carcharodus floccifera|WMB606-11|RVcoll.11-I005|Sicily|Italy|658[0n]
Carcharodus floccifera|EULEP621-15|RVcoll.11-H292|Sicily. Genit. examined|Italy|658[0n]
Carcharodus floccifera|EULEP2473-15|RVcoll.14-V326|Genit. examined|Ukraine|658[0n]
Carcharodus floccifera|OXB1155-15|Rvcoll_16-A043|Sicily|Italy|658[0n]
Carcharodus floccifera|WMB2917-14|RVcoll.11-I024|Sicily|Italy|625[0n]
Carcharodus floccifera|EZRMN310-08|RVcoll.08-M794||Romania|604[0n]
Carcharodus floccifera|EZROM075-08|RV-07-D306|Genit. examined|Romania|658[0n]
Carcharodus orientalis|EULEP1534-15|RVcoll.14-G491|Genit. examined|Greece|658[0n]
Carcharodus orientalis|EULEP1312-15|RVcoll.14-F770|Genit. examined|Greece|658[0n]
Carcharodus orientalis|EZROM836-08|RVcoll.08-M391|Genit. examined|Romania|658[0n]
Carcharodus orientalis|EULEP1420-15|RVcoll.14-G092|Genit. examined|Greece|658[0n]
Carcharodus orientalis|EULEP896-15|RVcoll.14-C712|Genit. examined|Greece|658[0n]
Carcharodus orientalis|EULEP1789-15|RVcoll.14-H645|Genit. examined|Greece|658[0n]
Carcharodus orientalis|EULEP1604-15|RVcoll.14-G657|Genit. examined|Greece|658[0n]
Carcharodus orientalis|EULEP1328-15|RVcoll.14-F807|Genit. examined|Greece|658[0n]
Carcharodus orientalis|EULEP1250-15|RVcoll.14-F607|Genit. examined|Greece|658[0n]
Carcharodus orientalis|EULEP2690-15|RVcoll.14-N774|Lesvos|Greece|614[0n]
Carcharodus orientalis|EULEP1783-15|RVcoll.14-H595|Genit. examined|Greece|658[0n]
Carcharodus orientalis|EULEP1471-15|RVcoll.14-G238|Genit. examined|Greece|658[0n]
Carcharodus orientalis|EULEP864-15|RVcoll.14-C237|Genit. examined|Greece|658[1n]
Carcharodus orientalis|EULEP1659-15|RVcoll.14-G871|Genit. examined|Greece|614[0n]
Carcharodus orientalis|EULEP1603-15|RVcoll.14-G656|Genit. examined|Greece|614[0n]
Carcharodus orientalis|EZROM665-08|RV-07-D119|Genit. examined|Romania|658[0n]
Carcharodus orientalis|EZROM078-08|RV-07-D055|Genit. examined|Romania|658[0n]
Carcharodus orientalis|EZROM079-08|RV-07-D074|Genit. examined|Romania|658[0n]
Carcharodus orientalis|EZROM838-08|RVcoll.08-M437|Genit. examined|Romania|658[0n]
Carcharodus orientalis|EZROM835-08|RVcoll.08-M386|Genit. examined|Romania|658[0n]
Carcharodus orientalis|EZROM080-08|RV-07-D132|Genit. examined|Romania|658[0n]
Carcharodus orientalis|EZROM664-08|RV-07-D059|Genit. examined|Romania|658[0n]
Carcharodus orientalis|EZRMN331-08|RVcoll.08-M784|Genit. examined|Romania|658[0n]
Carcharodus floccifera|OXB1156-15|Rvcoll_16-A044|Sicily|Italy|658[0n]
Carcharodus floccifera|BIBSA1534-16|08-R541||Italy|658[0n]
Carcharodus floccifera|WMB222-11|RVcoll.08-R540|Sicily|Italy|658[0n]
Carcharodus floccifera|EZSPN280-09|RVcoll.07-W215||Spain|658[0n]
Carcharodus floccifera|FBLMX123-11|BC ZSM Lep 37604||Germany|658[0n]
Carcharodus floccifera|EZSPN282-09|RVcoll.07-W218||Spain|658[0n]
Carcharodus floccifera|BIBSA440-15|RVcoll.14-I108||Italy|658[0n]
Carcharodus floccifera|EZROM831-08|RVcoll.08-M588|Genit. examined|Romania|658[0n]
Carcharodus floccifera|EZROM830-08|RVcoll.08-M583|Genit. examined|Romania|658[0n]
Carcharodus floccifera|EZSPC221-09|RVcoll.08-L436|Genit. examined|Spain|656[0n]
Carcharodus floccifera|EZROM832-08|RVcoll.08-M605|Genit. examined|Romania|658[0n]
Carcharodus floccifera|EZROM712-08|RV-07-F301|Genit. examined|Spain|658[0n]
Carcharodus floccifera|EZSPC265-09|RVcoll.08-R118||Spain|658[0n]
Carcharodus floccifera|EZSPC204-09|RVcoll.07-C626|Genit. examined|Spain|658[0n]
Carcharodus floccifera|EZSPC935-10|RVcoll.07-W293||Spain|658[0n]
Carcharodus floccifera|WMB2699-13|RVcoll.10-C076||France|658[0n]
Carcharodus floccifera|WMB2673-13|RVcoll.10-B862||France|658[0n]
Carcharodus floccifera|GWOSA770-10|BC ZSM Lep 36636||Germany|658[0n]
Carcharodus floccifera|WMB1846-13|RVcoll.11-I915||France|658[0n]
Carcharodus floccifera|WMB5389-14|RVcoll.14-J869||France|658[0n]
Carcharodus floccifera|EULEP1102-15|RVcoll.14-F151|Genit. examined|Serbia|658[0n]
Carcharodus floccifera|EULEP1151-15|RVcoll.14-F330|Genit. examined|Serbia|658[0n]
Carcharodus floccifera|EULEP1517-15|RVcoll.14-G440|Genit. examined|Greece|658[0n]
Carcharodus floccifera|EULEP974-15|RVcoll.14-D087|Genit. examined|Bulgaria|658[0n]
Carcharodus floccifera|BIBSA191-15|RVcoll.14-D979||Italy|658[0n]
Carcharodus floccifera|LEASS850-17|TLMF Lep 22498||Austria|658[0n]
Carcharodus floccifera|EZROM839-08|RVcoll.08-M454|Genit. examined|Romania|658[0n]
Carcharodus floccifera|EZROM613-08|RV-07-D016|Genit. examined|Romania|658[0n]
Carcharodus floccifera|EZRMN347-08|RVcoll.07-D614|Genit. examined|Romania|649[1n]
Carcharodus floccifera|OXB886-15|15-A528||Italy|658[0n]
Carcharodus floccifera|BIBSA662-15|RVcoll. 15-A825||Italy|658[0n]
Carcharodus floccifera|BIBSA1637-16|15-C502||Italy|658[0n]
Carcharodus floccifera|WMB5167-14|RVcoll.13-S500||Italy|658[0n]
Carcharodus floccifera|WMB090-11|RVcoll.LD-3152||Italy|658[0n]
Carcharodus floccifera|WMB2949-14|RVcoll.12-M528|Capraia|Italy|658[0n]
Carcharodus floccifera|WMB1287-13|RVcoll.12-M527||Italy|658[0n]
Carcharodus floccifera|WMB4828-14|RVcoll. 14-I375||Italy|658[0n]

Carcharodus floccifera|WMB2949-14|RVcoll.12-M528|Capraia|Italy|658[0n]
Carcharodus floccifera|WMB1287-13|RVcoll.12-M527||Italy|658[0n]
Carcharodus floccifera|WMB4828-14|RVcoll. 14-I375||Italy|658[0n]
Carcharodus floccifera|EULEP1856-15|RVcoll.13-U145|Genit. examined|Italy|658[0n]
Carcharodus floccifera|BIBSA1023-15|15-C168||Italy|658[0n]
Carcharodus floccifera|BIBSA1039-15|15-C217||Italy|658[0n]
Carcharodus floccifera|BIBSA1063-15|14-V319||Italy|658[0n]
Carcharodus floccifera|BIBSA645-15|RVcoll. 15-A808||Italy|658[0n]
Carcharodus floccifera|WMB4827-14|RVcoll. 14-I374||Italy|614[0n]
Carcharodus floccifera|GWORZ062-10|BC ZSM Lep 30418||Italy|647[0n]
Carcharodus floccifera|PHLAH464-12|TLMF Lep 08283||Austria|658[0n]
Colias palaeno|BIBSA1194-15|15-M277||Italy|658[0n]
Colias palaeno|EULEP2112-15|RVcoll.14-J605||Switzerland|658[0n]
Colias palaeno|EULEP3713-16|RVcoll15H405||Switzerland|658[0n]
Colias palaeno|EULEP3710-16|RVcoll14V890||Austria|658[0n]
Colias palaeno|LON055-08|NHMO-06055||Norway|657[0n]
Colias palaeno|ABOLB060-15|TLMF Lep 17065||Austria|658[0n]
Colias crocea|WMB5001-14|RVcoll. 14-I548||Italy|658[0n]
Colias crocea|WMB3545-14|RVcoll.11-D861||Spain|614[0n]
Colias crocea|WMB3533-14|RVcoll.10-C157||France|614[0n]
Colias erate|EULEP2508-15|RVcoll.14-V376||Ukraine|658[0n]
Colias erate|EULEP2573-15|RVcoll.14-V550||Ukraine|658[0n]
Colias erate|LEASS966-17|TLMF Lep 22519||Austria|658[0n]
Colias erate|LEASS479-17|TLMF Lep 22127||Austria|658[0n]
Colias erate|ABOLD657-17|TLMF Lep 21733||Austria|658[0n]
Colias erate|ABOLD576-17|TLMF Lep 21652||Austria|658[0n]
Colias erate|EULEP2245-15|RVcoll.14-N427||Ukraine|617[0n]
Colias erate|EZRMN396-09|RVcoll.07-F556|Genit. examined|Romania|658[0n]
Colias erate|EULEP764-15|RVcoll.12-R746||Romania|658[0n]
Colias crocea|EULEP732-15|RVcoll.12-N832|Lesvos|Greece|658[0n]
Colias crocea|EULEP693-15|RVcoll.12-M104|Crete|Greece|658[0n]
Colias crocea|EZSPM035-09|RVcoll.08-P236||Spain|658[0n]
Colias erate|EZROM576-08|RV-07-C375|Genit. examined|Romania|658[0n]
Colias|EZROM575-08|RV-07-C376|Possible hybrid. Genit. examined|Romania|658[0n]
Colias erate|EZROM187-08|RV-06-V720|Genit. examined|Romania|655[0n]
Colias erate|EZROM161-08|RV-06-V721|Genit. examined|Romania|658[0n]
Colias erate|EZROM127-08|RV-06-V722|Genit. examined|Romania|658[0n]
Colias erate|EZROM126-08|RV-06-V718|Genit. examined|Romania|658[0n]
Colias|EZROM124-08|RV-06-V783|Possible hybrid. Genit. examined|Romania|658[0n]
Colias crocea|EZROM122-08|RV-07-D199||Romania|658[0n]
Colias erate|EZRMN397-09|RVcoll.08-M114|Genit. examined|Romania|658[0n]
Colias erate|EZROM617-08|RV-06-V785|Genit. examined|Romania|658[0n]
Colias|EZROM894-08|RVcoll.08-M453|Possible hybrid. Genit. examined|Romania|658[0n]
Colias erate|EZROM893-08|RVcoll.08-M659|Genit. examined|Romania|658[0n]
Colias erate|EZROM892-08|RVcoll.08-M451|Genit. examined|Romania|658[0n]
Colias|EZROM891-08|RVcoll.08-M411|Possible hybrid. Genit. examined|Romania|658[0n]
Colias crocea|EZSPM683-12|Rvcoll. 12-L545||Portugal|621[0n]
Colias crocea|EZSPN449-09|RVcoll.08-H641||Spain|658[0n]
Colias crocea|BIBSA1092-15|15-L341||Italy|612[0n]
Colias crocea|BIBSA667-15|RVcoll. 12-Q787||Italy|624[0n]
Colias crocea|WMB4965-14|RVcoll. 14-I512||Italy|629[0n]
Colias crocea|GBLAB767-13|BC ZSM Lep 75538||Germany|621[0n]
Colias crocea|GWORR426-10|BC ZSM Lep 29642||Italy|633[0n]
Colias crocea|WMB4814-14|RVcoll. 14-I361||Italy|633[0n]
Colias crocea|EULEP5053-16|RVcoll16J085||Czech Republic|658[0n]
Colias crocea|EZSPN579-09|RVcoll.08-J129||Portugal|637[0n]
Colias crocea|BIBSA198-15|RVcoll.14-D986||Italy|638[0n]
Colias crocea|WMB3926-14|RVcoll.12-P512||France|620[0n]
Colias crocea|EZROM889-08|RVcoll.07-C950|Genit. examined|Romania|658[0n]
Colias crocea|EZROM125-08|RV-06-V784|Genit. examined|Romania|656[0n]
Colias crocea|WMB1606-13|RVcoll.12-O678||France|658[0n]
Colias crocea|WMB1680-13|RVcoll.12-P457||France|658[0n]
Colias crocea|WMB1738-13|RVcoll.12-P950|Levant|France|658[0n]
Colias crocea|WMB1910-13|RVcoll.11-H624|Lampedusa|Italy|658[0n]
Colias crocea|WMB1920-13|RVcoll.11-H695|Levanzo|Italy|658[0n]
Colias crocea|WMB1925-13|RVcoll.11-H789|Vulcano|Italy|658[0n]
Colias crocea|WMB1979-13|RVcoll.11-I282|Pianosa|Italy|658[0n]
Colias crocea|WMB3839-14|RVcoll.10-C519||Italy|658[0n]
Colias crocea|WMB2181-13|RVcoll.12-R354|Elba|Italy|658[0n]
Colias crocea|WMB2265-13|RVcoll.12-Z336|Ponza|Italy|658[0n]
Colias crocea|WMB2284-13|RVcoll.10-C539||Italy|658[0n]
Colias crocea|WMB2309-13|RVcoll.11-E153|Gozo|Malta|658[0n]
Colias crocea|WMB2310-13|RVcoll.11-E154|Gozo|Malta|658[0n]
Colias crocea|WMB2315-13|RVcoll.11-H623|Lampedusa|Italy|658[0n]
Colias crocea|WMB2316-13|RVcoll.11-H625.1|Lampedusa|Italy|658[0n]
Colias crocea|WMB2352-13|RVcoll.12-Q886||Italy|658[0n]
Colias crocea|WMB2449-13|RVcoll.11-E371|Sardinia|Italy|658[0n]
Colias crocea|WMB4147-14|RVcoll.13-S689||Italy|658[0n]
Colias crocea|WMB4489-14|RVcoll.14-E164||Italy|658[0n]
Colias crocea|WMB4492-14|RVcoll.14-E168||Italy|658[0n]
Colias crocea|WMB4292-14|RVcoll.14-A373||Italy|658[0n]
Colias crocea|WMB4373-14|RVcoll.14-B156||Portugal|658[0n]
Colias crocea|WMB4703-14|RVcoll.LD-2897|Asinara|Italy|658[0n]
Colias crocea|WMB293-11|RVcoll.11-E301||Italy|658[0n]
Colias crocea|WMB309-11|RVcoll.11-E423||Italy|658[0n]
Colias crocea|WMB324-11|RVcoll.11-E609||Italy|658[0n]
Colias crocea|WMB342-11|RVcoll.11-E698||France|658[0n]
Colias crocea|WMB360-11|RVcoll.11-E958||France|658[0n]
Colias crocea|WMB491-11|RVcoll.11-H538|Sicily|Italy|658[0n]
Colias crocea|WMB4844-14|RVcoll. 14-I391||Italy|658[0n]
Colias crocea|WMB537-11|RVcoll.11-H696|Levanzo|Italy|658[0n]
Colias crocea|WMB568-11|RVcoll.11-H790|Vulcano|Italy|658[0n]
Colias crocea|WMB5071-14|RVcoll. 14-L202||Italy|658[0n]
Colias crocea|WMB581-11|RVcoll.11-H869|Salina|Italy|658[0n]
Colias crocea|WMB591-11|RVcoll.11-H920|Sicily|Italy|658[0n]
Colias crocea|WMB654-11|RVcoll.11-I251||Italy|658[0n]
Colias crocea|WMB4753-14|RVcoll. 14-I300||Italy|658[0n]
Colias crocea|WMB4837-14|RVcoll. 14-I384||Italy|658[0n]
Colias crocea|WMB5357-14|RVcoll.14-J700||France|658[0n]
Colias crocea|WMB4903-14|RVcoll.14-I450||Italy|658[0n]
Colias crocea|WMB5057-14|RVcoll. 14-L188||Italy|658[0n]
Colias crocea|WMB5205-14|RVcoll.13-T938||Italy|658[0n]
Colias crocea|WMB5222-14|RVcoll.13-T972||Italy|658[0n]

Colias crocea|[illegible]
Colias crocea|WMB5057-14|RVcoll. 14-L188||Italy|658[0n]
Colias crocea|WMB5205-14|RVcoll.13-T938||Italy|658[0n]
Colias crocea|WMB5222-14|RVcoll.13-T972||Italy|658[0n]
Colias crocea|LOWA288-06|2005-LOWA-288||Russia|658[0n]
Colias crocea|LOWA529-06|2005-LOWA-529||Ukraine|658[0n]
Colias crocea|LOWA530-06|2005-LOWA-530||Ukraine|658[0n]
Colias crocea|EULEP3705-16|RVcoll15G845||Italy|658[0n]
Colias crocea|EULEP3706-16|RVcoll15I359||Austria|658[0n]
Colias crocea|EULEP3725-16|RVcoll14A985||Bulgaria|658[0n]
Colias crocea|EULEP5657-17|RVcoll15K000|Gorgona|Italy|658[0n]
Colias crocea|EULEP5720-17|RVcoll16L191||Italy|658[0n]
Colias crocea|EULEP1210-15|RVcoll.14-F506||Greece|658[0n]
Colias crocea|EULEP1317-15|RVcoll.14-F776||Greece|658[0n]
Colias crocea|EULEP559-15|RVcoll.08-P955|Rhodes. Genit. examined|Greece|658[0n]
Colias crocea|EULEP911-15|RVcoll.14-C770||Bulgaria|658[0n]
Colias crocea|EULEP1433-15|RVcoll.14-G119||Greece|658[0n]
Colias crocea|EULEP2222-15|RVcoll.14-N400||Ukraine|658[0n]
Colias crocea|BIBSA1164-15|15-M183||Italy|658[0n]
Colias crocea|BIBSA1246-15|15-F825||France|658[0n]
Colias crocea|BIBSA912-15|16-A003||France|658[0n]
Colias crocea|BIBSA918-15|16-A009||France|658[0n]
Colias crocea|BIBSA731-15|LD-3040||Italy|657[0n]
Colias crocea|BIBSA1607-16|14-A718||Italy|658[0n]
Colias crocea|BIBSA1020-15|15-C162||Italy|658[0n]
Colias crocea|BIBSA1287-15|13-T688|Sardinia|Italy|658[0n]
Colias crocea|BIBSA1311-15|12-M360|Sicily|Italy|658[0n]
Colias crocea|BIBSA1314-15|12-O374|Sardinia|Italy|658[0n]
Colias crocea|BIBSA1792-16|16-A529||Italy|658[0n]
Colias crocea|BIBSA1324-15|12-Q479|Sardinia|Italy|658[0n]
Colias crocea|BIBSA1404-15|15-N010||Italy|658[0n]
Colias crocea|BIBSA1419-15|15-N089||Italy|658[0n]
Colias crocea|BIBSA687-15|RVcoll. 11-I252||Italy|657[0n]
Colias crocea|BIBSA1546-16|11-D339||Italy|658[0n]
Colias crocea|BIBSA152-15|RVcoll.14-I164||Italy|658[0n]
Colias crocea|BIBSA153-15|RVcoll.14-I165||Italy|658[0n]
Colias crocea|BIBSA293-15|RVcoll.14-E095||Italy|658[0n]
Colias crocea|BIBSA332-15|RVcoll.14-I000||Italy|658[0n]
Colias crocea|BIBSA1657-16|15-C690||Italy|658[0n]
Colias crocea|BIBSA038-14|LEP-SS-00038||Italy|658[0n]
Colias crocea|BIBSA1834-17|RVcoll16C709||Italy|658[0n]
Colias crocea|LEASS964-17|TLMF Lep 22517||Austria|658[0n]
Colias crocea|LEASS863-17|KLM Lep 08368||Austria|658[0n]
Colias crocea|OXB857-15|15-A501||Italy|658[0n]
Colias crocea|OXB858-15|15-A502||Italy|658[0n]
Colias crocea|OXB571-15|14-N960||Italy|658[0n]
Colias crocea|OXB669-15|07-D808||Italy|658[0n]
Colias crocea|OXB752-15|13-U474||Italy|658[0n]
Colias crocea|OXB755-15|13-U490||Italy|658[0n]
Colias crocea|OXB889-15|15-A531||Italy|658[0n]
Colias crocea|OXB892-15|15-A534||Italy|658[0n]
Colias crocea|OXB942-15|15-A586||Italy|658[0n]
Colias crocea|OXB985-15|15-A935||Italy|658[0n]
Colias crocea|OXB1044-15|LD-3039||Italy|658[0n]
Colias crocea|OXB1066-15|14j287||Italy|658[0n]
Colias crocea|OXB1067-15|14j370||Italy|658[0n]
Colias crocea|OXB1073-15|15-A606||Italy|658[0n]
Colias crocea|OXB1550-16|OXB-TGS-1268||Andorra|658[0n]
Colias crocea|OXB1551-16|OXB-TGS-1269||France|658[0n]
Colias crocea|OXB264-15|OXB-TGS-947||United Kingdom|658[0n]
Colias crocea|OXB324-15|RVcoll.14-N038||Italy|658[0n]
Colias crocea|OXB1352-15|Rvcoll_15-M704||France|658[0n]
Colias crocea|OXB1209-15|Rvcoll_15-M634||France|658[0n]
Colias crocea|OXB1264-15|Rvcoll_15-M120||France|658[0n]
Colias crocea|ABOLD658-17|TLMF Lep 21734||Austria|658[0n]
Colias crocea|BIBSA1761-16|LD-2804||Italy|617[0n]
Colias crocea|WMB1911-13|RVcoll.11-H626|Lampedusa|Italy|653[0n]
Colias crocea|BIBSA039-14|LEP-SS-00039||Italy|632[0n]
Colias crocea|WMB1693-13|RVcoll.12-P566||France|640[0n]
Colias crocea|WMB1558-13|RVcoll.12-O400|San Pietro|Italy|617[0n]
Colias crocea|WMB1288-13|RVcoll.12-M529|Capraia|Italy|658[0n]
Colias crocea|WMB1098-13|RVcoll.10-C766|Giglio|Italy|658[0n]
Colias crocea|WMB907-13|RVcoll.09-X896|Capri|Italy|658[0n]
Colias crocea|WMB900-13|RVcoll.09-X856||Italy|658[0n]
Colias crocea|WMB1500-13|RVcoll.12-O047||Italy|658[0n]
Colias crocea|WMB1484-13|RVcoll.12-O012|Giglio|Italy|658[0n]
Colias crocea|WMB2759-13|RVcoll.11-H694|Levanzo|Italy|658[0n]
Colias crocea|WMB2735-13|RVcoll.10-C593||Malta|658[0n]
Colias crocea|WMB2734-13|RVcoll.10-C592||Malta|658[0n]
Colias crocea|WMB1178-13|RVcoll.11-E847|Corsica|France|658[0n]
Colias crocea|WMB1147-13|RVcoll.11-E152|Gozo|Malta|658[0n]
Colias crocea|WMB1146-13|RVcoll.11-E151|Gozo|Malta|658[0n]
Colias crocea|WMB1116-13|RVcoll.11-D158|Marettimo|Italy|658[0n]
Colias crocea|WMB1111-13|RVcoll.11-D095|Pantelleria|Italy|658[0n]
Colias crocea|WMB3709-14|RVcoll.12-L615||Portugal|658[0n]
Colias crocea|WMB3636-14|RVcoll.11-I449||Spain|658[0n]
Colias crocea|WMB3574-14|RVcoll.11-D994||Spain|658[0n]
Colias crocea|WMB3556-14|RVcoll.11-D900||Spain|658[0n]
Colias crocea|WMB3310-14|RVcoll.08-P493||Spain|658[0n]
Colias crocea|WMB3231-14|RVcoll.08-J879||Spain|658[0n]
Colias crocea|WMB3226-14|RVcoll.08-J765||Spain|658[0n]
Colias crocea|WMB3172-14|RVcoll.08-H547||Spain|658[0n]
Colias crocea|WMB3162-14|RVcoll.08-H411||Spain|658[0n]
Colias crocea|WMB3159-14|RVcoll.08-H252||Spain|658[0n]
Colias crocea|WMB3695-14|RVcoll.11-J550||Spain|658[0n]
Colias crocea|WMB3687-14|RVcoll.11-J234||Spain|658[0n]
Colias crocea|WMB3035-14|RVcoll.13-S558|San Domino|Italy|658[0n]
Colias crocea|WMB3028-14|RVcoll.13-S399|Ibiza|Spain|658[0n]
Colias crocea|WMB2966-14|RVcoll.12-O046|Argentario|Italy|658[0n]
Colias crocea|WMB2937-14|RVcoll.12-L513|Ibiza|Spain|658[0n]
Colias crocea|WMB2910-14|RVcoll.11-E944|Corsica|France|658[0n]
Colias crocea|WMB2904-14|RVcoll.11-E773|Corsica|France|658[0n]
Colias crocea|WMB2901-14|RVcoll.11-E735|Corsica|France|658[0n]

Colias crocea|WMB2910-14|RVcoll.11-E944|Corsica|France|658[0n]
Colias crocea|WMB2904-14|RVcoll.11-E773|Corsica|France|658[0n]
Colias crocea|WMB2901-14|RVcoll.11-E735|Corsica|France|658[0n]
Colias crocea|WMB2888-14|RVcoll.11-D119|Pantelleria|Italy|658[0n]
Colias crocea|WMB2881-14|RVcoll.10-C685|Stromboli|Italy|658[0n]
Colias crocea|WMB2880-14|RVcoll.10-C678|Lipari|Italy|658[0n]
Colias crocea|WMB128-11|RVcoll.11-D121|Pantelleria|Italy|658[0n]
Colias crocea|WMB107-11|RVcoll.10-B626||France|658[0n]
Colias crocea|EZROM717-08|RV-06-H846||Spain|658[0n]
Colias crocea|EZSPC363-09|RVcoll.08-L490||Spain|658[0n]
Colias crocea|WMB2854-14|RVcoll.09-X100|Menorca|Spain|658[0n]
Colias crocea|WMB169-11|RVcoll.11-D407|Sicily|Italy|658[0n]
Colias crocea|WMB168-11|RVcoll.11-D397|Sicily|Italy|658[0n]
Colias crocea|WMB139-11|RVcoll.11-D157|Marettimo|Italy|658[0n]
Colias crocea|EZSPM935-12|Rvcoll.12-M669||Spain|658[0n]
Colias crocea|EZSPM238-09|RVcoll.08-R439||Spain|658[0n]
Colias crocea|EZSPM052-09|RVcoll.08-P270||Spain|658[0n]
Colias crocea|EZSPM684-12|Rvcoll. 12-L567||Portugal|658[0n]
Colias crocea|EZSPM278-09|SMcoll.08-J601||Spain|658[0n]
Colias crocea|EZSPN649-09|RVcoll.08-J790||Spain|658[0n]
Colias crocea|EZSPM987-12|Rvcoll.12-M756||Spain|658[0n]
Colias crocea|EZSPM986-12|Rvcoll.12-M755||Spain|658[0n]
Colias crocea|EZSPM981-12|Rvcoll.12-M744||Spain|658[0n]
Colias crocea|EZSPM959-12|Rvcoll.12-M710||Spain|658[0n]
Colias crocea|EZSPN489-09|RVcoll.08-H926||Spain|658[0n]
Colias crocea|EZSPN488-09|RVcoll.08-H925||Spain|658[0n]
Colias crocea|EZSPN320-09|RVcoll.08-H181||Spain|658[0n]
Colias crocea|EZSPN306-09|RVcoll.08-H111||Spain|658[0n]
Colias crocea|EZSPC364-09|RVcoll.08-M687||Spain|658[0n]
Colias crocea|EZSPC365-09|RVcoll.08-P073||Spain|658[0n]
Colias crocea|EZROM123-08|RV-07-D588|Genit. examined|Romania|658[0n]
Colias crocea|EZROM121-08|RV-07-D169||Romania|658[0n]
Colias crocea|EZROM317-08|RV-07-E679||Romania|658[0n]
Colias erate|EZRMN398-09|RVcoll.08-M115|Genit. examined|Romania|658[0n]
Colias crocea|EZROM890-08|RVcoll.07-E364|Genit. examined|Romania|658[0n]
Colias crocea|EZRMN395-09|RVcoll.08-H036|Genit. examined|Romania|658[0n]
Colias crocea|GWORA2439-09|BC ZSM Lep 30651||Germany|658[0n]
Colias crocea|GWORO783-09|BC ZSM Lep 30475||Germany|658[0n]
Colias crocea|GWOSI552-10|BC ZSM Lep 44398||Germany|658[0n]
Colias crocea|ABOLB043-15|TLMF Lep 17048||Austria|658[0n]
Colias crocea|LEFIE435-10|MM09186||Italy|658[0n]
Colias crocea|LEATG522-14|TLMF Lep 14309||Italy|658[0n]
Colias crocea|LEATG008-14|TLMF Lep 13795||Austria|658[0n]
Colias crocea|ABOLD404-16|TLMF Lep 21556||Austria|658[0n]
Colias crocea|ABOLD043-16|TLMF Lep 21121||Austria|658[0n]
Colias crocea|LEATG523-14|TLMF Lep 14310||Italy|658[0n]
Colias crocea|LEFIL144-10|MM19144||Spain|658[0n]
Colias crocea|PHLAH699-12|TLMF Lep 08518||Austria|658[0n]
Colias chrysotheme|LEASS960-17|TLMF Lep 22513||Austria|658[0n]
Colias chrysotheme|LEATJ1197-16|TLMF Lep 19520||Hungary|658[0n]
Colias chrysotheme|EULEP2050-15|RVcoll.14-J533||Ukraine|658[0n]
Colias chrysotheme|EULEP2049-15|RVcoll.14-J532||Ukraine|658[0n]
Colias chrysotheme|EULEP4825-16|RVcoll16H721||Ukraine|658[0n]
Colias chrysotheme|EZROM120-08|RV-07-D438|Genit. examined|Romania|658[0n]
Colias chrysotheme|EZROM119-08|RV-06-V681|Genit. examined|Romania|658[0n]
Colias chrysotheme|EZROM888-08|RVcoll.07-D424|Genit. examined|Romania|658[0n]
Colias chrysotheme|EZROM669-08|RV-06-V682||Romania|658[0n]
Colias palaeno|EULEP3711-16|RVcoll14V893||Switzerland|658[0n]
Colias myrmidone|EULEP2053-15|RVcoll.14-J538||Ukraine|658[0n]
Colias caucasica|EULEP3702-16|RVcoll15Q011||Macedonia|640[0n]
Colias myrmidone|EULEP2773-15|RVcoll.14-N857||Russia|658[0n]
Colias myrmidone|EULEP2772-15|RVcoll.14-N856||Czech Republic|658[0n]
Colias caucasica|EULEP2760-15|RVcoll.14-N844||Serbia|658[0n]
Colias caucasica|EULEP2759-15|RVcoll.14-N843||Serbia|658[0n]
Colias caucasica|EULEP2758-15|RVcoll.14-N842||Serbia|658[0n]
Colias caucasica|EULEP2757-15|RVcoll.14-N841||Serbia|658[0n]
Colias caucasica|EULEP5145-17|RVcoll16J901||Serbia|658[0n]
Colias myrmidone|EULEP3724-16|RVcoll15Q102||Russia|658[0n]
Colias caucasica|EULEP3704-16|RVcoll15Q013||Macedonia|658[0n]
Colias caucasica|EULEP3703-16|RVcoll15Q012||Macedonia|658[0n]
Colias caucasica|EULEP3699-16|RVcoll15Q060||Serbia|658[0n]
Colias caucasica|EULEP3698-16|RVcoll15Q059||Serbia|658[0n]
Colias caucasica|EULEP3696-16|RVcoll15Q057||Serbia|658[0n]
Colias caucasica|EULEP3695-16|RVcoll15P055||Serbia|658[0n]
Colias caucasica|EULEP3693-16|RVcoll15P053||Serbia|658[0n]
Colias myrmidone|EZROM132-08|RV-06-V651|Genit. examined|Romania|658[0n]
Colias myrmidone|EZROM131-08|RV-06-V650|Genit. examined|Romania|658[0n]
Colias myrmidone|EZROM670-08|RV-06-M835||Romania|658[0n]
Colias myrmidone|EULEP3723-16|RVcoll15Q101||Russia|658[0n]
Colias caucasica|EULEP2292-15|RVcoll.14-N492||Greece|658[0n]
Colias myrmidone|EULEP995-15|RVcoll.14-E158||Romania|608[0n]
Colias caucasica|EULEP3701-16|RVcoll15Q007||Macedonia|658[0n]
Colias caucasica|EULEP3697-16|RVcoll15Q058||Serbia|658[0n]
Colias caucasica|EULEP3694-16|RVcoll15P054||Serbia|658[0n]
Colias myrmidone|EZROM897-08|RVcoll.07-C136||Romania|658[0n]
Colias hecla|EULEP369-14|MM23854||Finland|658[0n]
Colias hecla|LEFIC096-10|MM03410||Finland|658[0n]
Colias hecla|EULEP372-14|MM23857||Finland|658[0n]
Colias hecla|EULEP4774-16|RVcoll16H290||Norway|658[0n]
Colias hecla|LEFIF120-10|MM10576||Finland|658[0n]
Colias hecla|LEFID428-10|MM06358||Finland|658[0n]
Colias palaeno|EULEP2775-15|RVcoll.14-N859||France|658[0n]
Colias palaeno|LEASS1046-17|TLMF Lep 22599||Austria|658[0n]
Colias palaeno|EULEP5162-17|RVcoll15M097||Italy|658[0n]
Colias palaeno|EULEP3716-16|RVcoll15O013||Italy|658[0n]
Colias palaeno|EULEP3712-16|RVcoll15G526||Switzerland|658[0n]
Colias palaeno|EULEP3624-16|RVcoll15G676||Italy|658[0n]
Colias palaeno|GWOSA728-10|BC ZSM Lep 35739||Germany|658[0n]
Colias palaeno|GWOSA727-10|BC ZSM Lep 35738||Germany|658[0n]
Colias palaeno|GWORO782-09|BC ZSM Lep 30474||Germany|658[0n]
Colias palaeno|FBLMU459-09|BC ZSM Lep 27109||Germany|658[0n]
Colias palaeno|PHLAI549-13|TLMF Lep 09111||Austria|658[0n]
Colias|LEFIJ2403-14|MM23786|hybrid|Norway|658[0n]

[illegible]
Colias palaeno|FBLMU459-09|BC ZSM Lep 27109||Germany|658[0n]
Colias palaeno|PHLAI549-13|TLMF Lep 09111||Austria|658[0n]
Colias|LEFIJ2403-14|MM23786|hybrid|Norway|658[0n]
Colias|LEFIL357-10|MM18667|hybrid|Finland|658[0n]
Colias tyche|LEFIA738-10|MM04092||Finland|658[0n]
Colias|LEFIJ2400-14|MM23783|hybrid|Sweden|618[0n]
Colias|LEFIJ2401-14|MM23784|hybrid|Norway|658[0n]
Colias tyche|EULEP373-14|MM23858||Finland|658[0n]
Colias tyche|EULEP4717-16|RVcoll16B350||Sweden|658[0n]
Colias tyche|LEFIC098-10|MM03412||Finland|658[0n]
Colias tyche|LON935-12|NHMO Lep2010.008|ssp werdandi|Norway|658[0n]
Colias tyche|LEFIA022-10|MM00093||Finland|658[0n]
Colias|LEFIJ2399-14|MM23782|hybrid|Finland|658[0n]
Colias palaeno|EULEP2279-15|RVcoll.14-N465||Ukraine|638[0n]
Colias palaeno|EULEP2774-15|RVcoll.14-N858||Estonia|658[0n]
Colias palaeno|EULEP5179-17|RVcoll16H176||Norway|658[0n]
Colias palaeno|EULEP349-14|MM23834||Lithuania|658[0n]
Colias palaeno|EULEP4742-16|RVcoll16G675||Sweden|658[0n]
Colias palaeno|EULEP3715-16|RVcoll15O011||France|658[0n]
Colias palaeno|EULEP3714-16|RVcoll15G090||France|658[0n]
Colias palaeno|LEFIC097-10|MM03411||Finland|658[0n]
Colias palaeno|LEFIA1057-10|MM03233||Finland|658[0n]
Colias palaeno|LEFIJ510-10|MM17135||Finland|658[0n]
Colias palaeno|LEFIJ509-10|MM17134||Finland|658[0n]
Colias palaeno|PHLSA726-11|TLMF Lep 06181||Austria|658[0n]
Colias palaeno|LEATF466-14|TLMF Lep 13778||Austria|658[0n]
Colias palaeno|LEATG526-14|TLMF Lep 14313||Italy|658[0n]
Colias caucasica|EULEP2291-15|RVcoll.14-N491||Greece|658[0n]
Colias myrmidone|EULEP3722-16|RVcoll15Q100||Russia|658[0n]
Colias hecla|LEFIJ2424-14|MM23473||Finland|658[0n]
Colias hecla|LEFIJ2423-14|MM23472||Finland|658[0n]
Colias hecla|LON185-08|NHMO-06186||Norway|657[0n]
Colias aurorina|EULEP1823-15|RVcoll.14-J943||Albania|658[0n]
Colias aurorina|EULEP1822-15|RVcoll.14-J942||Albania|658[0n]
Colias aurorina|EULEP1821-15|RVcoll.14-J941||Albania|658[0n]
Colias aurorina|EULEP1322-15|RVcoll.14-F789||Greece|658[0n]
Colias aurorina|EULEP1285-15|RVcoll.14-F698||Greece|658[0n]
Colias aurorina|EULEP1282-15|RVcoll.14-F691||Greece|658[0n]
Colias aurorina|EULEP3692-16|RVcoll14O325||Greece|658[0n]
Colias palaeno|LEATJ1192-16|TLMF Lep 19515||Lithuania|658[0n]
Colias palaeno|ABOLD013-16|TLMF Lep 21091||Austria|658[0n]
Colias phicomone|EZSPC983-10|RVcoll.07-C020||France|640[0n]
Colias phicomone|EZSPC984-10|RVcoll.07-C028||France|658[0n]
Colias phicomone|EZSPC982-10|RVcoll.07-C019||France|658[0n]
Colias phicomone|EZSPC709-10|RVcoll.07-W195||Spain|658[0n]
Colias phicomone|EZSPM010-09|RVcoll.08-M990||Andorra|658[0n]
Colias phicomone|EZSPM832-12|Rvcoll. 140711ZG16||Spain|658[0n]
Colias phicomone|EZSPC862-10|RVcoll.09-T065||Spain|658[0n]
Colias phicomone|EZSPC861-10|RVcoll.09-T063||Spain|658[0n]
Colias phicomone|EZSPC847-10|RVcoll.08-R222||Spain|658[0n]
Colias phicomone|EZSPC846-10|RVcoll.08-R219||Spain|658[0n]
Colias phicomone|EZSPM192-09|RVcoll.08-R220||Spain|658[0n]
Colias phicomone|EZSPM191-09|RVcoll.08-R218||Spain|658[0n]
Colias phicomone|EZSPN273-09|RVcoll.07-W196||Spain|658[0n]
Colias phicomone|EZSPN272-09|RVcoll.07-W194||Spain|658[0n]
Colias phicomone|WMB1860-13|RVcoll.11-J162||France|658[0n]
Colias phicomone|EZSPC878-10|RVcoll.09-T129||Spain|658[0n]
Colias phicomone|EZSPC877-10|RVcoll.09-T128||Spain|658[0n]
Colias phicomone|LEATJ1325-16|TLMF Lep 19648||Italy|629[0n]
Colias phicomone|ABOLD577-17|TLMF Lep 21653||Austria|658[0n]
Colias phicomone|GBLAB769-13|BC ZSM Lep 75540||Germany|658[0n]
Colias phicomone|OXB626-15|14-O015||Italy|658[0n]
Colias phicomone|BIBSA378-15|RVcoll.14-I046||Italy|658[0n]
Colias phicomone|BIBSA377-15|RVcoll.14-I045||Italy|658[0n]
Colias phicomone|BIBSA292-15|RVcoll.14-E092||Italy|658[0n]
Colias phicomone|EULEP2457-15|RVcoll.14-V280||Switzerland|658[0n]
Colias phicomone|EULEP992-15|RVcoll.14-D242||Austria|658[0n]
Colias phicomone|EULEP3721-16|RVcoll15G379||Switzerland|658[0n]
Colias phicomone|EULEP3720-16|RVcoll15I668||Austria|658[0n]
Colias phicomone|EULEP3719-16|RVcoll15I180||Austria|658[0n]
Colias phicomone|EULEP3718-16|RVcoll15H982||Italy|658[0n]
Colias phicomone|EULEP3717-16|RVcoll15H656||Switzerland|658[0n]
Colias phicomone|WMB1893-13|RVcoll.11-J956||Italy|658[0n]
Colias phicomone|WMB1625-13|RVcoll.12-O788||France|658[0n]
Colias phicomone|WMB2677-13|RVcoll.10-B879||France|658[0n]
Colias phicomone|GWORA2442-09|BC ZSM Lep 30654||Germany|658[0n]
Colias phicomone|GWOTF671-12|BC ZSM Lep 62377||Germany|658[0n]
Colias phicomone|FBLMZ172-12|BC ZSM Lep 51428||Germany|658[0n]
Colias phicomone|PHLAB371-10|TLMF Lep 01171||Switzerland|658[0n]
Colias phicomone|PHLAI545-13|TLMF Lep 09107||Austria|658[0n]
Colias phicomone|PHLAA141-09|TLMF Lep 00181||Italy|658[0n]
Colias phicomone|LEATC097-13|TLMF Lep 11224||Italy|658[0n]
Colias phicomone|LEATC133-13|TLMF Lep 11260||Italy|658[0n]
Colias phicomone|PHLAA420-09|TLMF Lep 00460||France|658[0n]
Colias phicomone|PHLSA727-11|TLMF Lep 06182||Austria|658[0n]
Colias hyale|EULEP2411-15|RVcoll.14-V129||Ukraine|631[1n]
Colias hyale|EULEP2389-15|RVcoll.14-V089||Ukraine|658[0n]
Colias hyale|EULEP4830-16|RVcoll16H731||Ukraine|658[0n]
Colias hyale|LOWA782-06|2005-LOWA-782||Russia|658[0n]
Colias hyale|LEFID236-10|MM06097||Finland|658[0n]
Colias hyale|EULEP606-15|RVcoll.10-C418||Romania|627[0n]
Colias hyale|EZROM885-08|RVcoll.07-E541|Genit. examined|Romania|658[0n]
Colias hyale|EZROM895-08|RVcoll.08-M334|Genit. examined|Romania|658[0n]
Colias hyale|EZROM896-08|RVcoll.08-M335|Genit. examined|Romania|658[0n]
Colias hyale|EZROM130-08|RV-07-C104|Genit. examined|Romania|658[0n]
Colias hyale|WMB1854-13|RVcoll.11-I983||France|658[0n]
Colias hyale|WMB1855-13|RVcoll.11-I984||France|658[0n]
Colias hyale|EULEP3708-16|RVcoll15I891||Liechtenstein|658[0n]
Colias hyale|EULEP4910-16|RVcoll16J613||Russia|658[0n]
Colias hyale|EULEP4943-16|RVcoll16I126||Poland|658[0n]
Colias hyale|EULEP757-15|RVcoll.12-R655||Romania|658[0n]
Colias hyale|EULEP985-15|RVcoll.14-D146||Austria|658[0n]
[illegible]

Colias hyale|EULEP4943-16|RVcoll16I126||Poland|658[0n]
Colias hyale|EULEP757-15|RVcoll.12-R655||Romania|658[0n]
Colias hyale|EULEP985-15|RVcoll.14-D146||Austria|658[0n]
Colias hyale|BIBSA865-15|LD-3477||Italy|658[0n]
Colias hyale|BIBSA866-15|LD-3478||Italy|658[0n]
Colias hyale|GBLAC301-13|BC ZSM Lep 75167|GBOL-MNC-0001|Germany|658[0n]
Colias hyale|GBLAA1090-15|BC ZSM Lep 86596||Germany|658[0n]
Colias hyale|GBLAB317-13|BC ZSM Lep 72618||Germany|658[0n]
Colias hyale|LEFIJ4547-16|ZMBSU-02111||Belarus|658[0n]
Colias hyale|LEFIJ4748-16|ZMBSU-02129||Belarus|658[0n]
Colias hyale|LEASS861-17|KLM Lep 08366||Austria|658[0n]
Colias hyale|LEASS862-17|KLM Lep 08367||Austria|658[0n]
Colias hyale|OXB1221-15|Rvcoll_15-M648||France|658[0n]
Colias hyale|ABOLD659-17|TLMF Lep 21735||Austria|658[0n]
Colias hyale|ABOLD575-17|TLMF Lep 21651||Austria|658[0n]
Colias hyale|EZRMN354-08|RVcoll.08-M721|Genit. examined|Romania|649[0n]
Colias hyale|EZRMN353-08|RVcoll.08-M720|Genit. examined|Romania|649[0n]
Colias hyale|LEFIJ508-10|MM17133||Finland|658[0n]
Colias hyale|PHLAW014-13|TLMF Lep 09811||Austria|658[0n]
Colias hyale|ABOLB045-15|TLMF Lep 17050||Italy|658[0n]
Colias hyale|LEATG529-14|TLMF Lep 14316||Italy|658[0n]
Colias hyale|ABOLD403-16|TLMF Lep 21555||Austria|658[0n]
Colias hyale|ABOLB009-15|TLMF Lep 17014||Austria|658[0n]
Colias hyale|ABOLB044-15|TLMF Lep 17049||Austria|658[0n]
Colias alfacariensis|FBLMZ482-12|BC ZSM Lep 61238||Germany|658[3n]
Colias alfacariensis|GWORA2881-15|BC ZSM Lep 86149||Germany|626[0n]
Colias alfacariensis|GBLAD168-14|BC ZSM Lep 78739||Germany|658[0n]
Colias alfacariensis|EULEP989-15|RVcoll.14-D233||Austria|658[0n]
Colias alfacariensis|GWORT456-10|BC ZSM Lep 32142|changed to alfac. (det. Segerer)|Germany|...
Colias alfacariensis|GWORA2441-09|BC ZSM Lep 30653||Germany|658[0n]
Colias alfacariensis|GWORT455-10|BC ZSM Lep 32141|changed to alfac. (det. Segerer)|Germany|...
Colias alfacariensis|GWORT454-10|BC ZSM Lep 32140||Germany|658[0n]
Colias alfacariensis|GWORT453-10|BC ZSM Lep 32139||Germany|658[0n]
Colias alfacariensis|LEATG092-14|TLMF Lep 13879||Austria|658[0n]
Colias alfacariensis|LEATG094-14|TLMF Lep 13881||Austria|658[0n]
Colias alfacariensis|ABOLD574-17|TLMF Lep 21650||Austria|658[0n]
Colias alfacariensis|LEASS848-17|TLMF Lep 22496||Austria|658[0n]
Colias alfacariensis|EULEP619-15|RVcoll.11-H198||Germany|658[0n]
Colias alfacariensis|EULEP537-15|RVcoll.08-H882||Serbia|658[0n]
Colias alfacariensis|EULEP1116-15|RVcoll.14-F194||Serbia|658[0n]
Colias alfacariensis|EZROM128-08|RV-07-D093|Genit. examined|Romania|655[0n]
Colias alfacariensis|EZROM117-08|RV-06-K644|Genit. examined|Romania|658[0n]
Colias alfacariensis|EZROM115-08|RV-07-D012|Genit. examined|Romania|657[0n]
Colias alfacariensis|EZRMN399-09|RVcoll.08-L359|Genit. examined|Romania|658[0n]
Colias alfacariensis|EZROM621-08|RV-07-C974|Genit. examined|Romania|658[0n]
Colias alfacariensis|EZROM620-08|RV-07-D363|Genit. examined|Romania|658[0n]
Colias alfacariensis|EZROM886-08|RVcoll.08-M500|Genit. examined|Romania|658[0n]
Colias alfacariensis|EZROM569-08|RV-07-C996|Genit. examined|Romania|658[0n]
Colias alfacariensis|EZRMN375-08|RVcoll.08-M116|Genit. examined|Romania|658[0n]
Colias alfacariensis|FBLMV298-09|BC ZSM Lep 28278||Germany|658[0n]
Colias alfacariensis|ABOLD402-16|TLMF Lep 21554||Austria|658[0n]
Colias alfacariensis|ABOLD405-16|TLMF Lep 21557||Austria|658[0n]
Colias alfacariensis|ABOLD406-16|TLMF Lep 21558||Austria|658[0n]
Colias alfacariensis|WMB3311-14|RVcoll.08-P494||Spain|610[0n]
Colias alfacariensis|EZSPM161-09|RVcoll.08-P689||Spain|614[0n]
Colias alfacariensis|EZROM716-08|RV-06-G437||Spain|658[0n]
Colias alfacariensis|EZSPN411-09|RVcoll.08-H521||Spain|658[0n]
Colias alfacariensis|EZSPN758-09|RVcoll.08-L236||Spain|658[0n]
Colias alfacariensis|EZSPN139-09|RVcoll.06-G498||Spain|658[0n]
Colias alfacariensis|EZSPN724-09|RVcoll.08-L119||Spain|658[0n]
Colias alfacariensis|EZSPC361-09|RVcoll.08-H290||Spain|658[0n]
Colias alfacariensis|EZSPN344-09|RVcoll.08-H307||Spain|658[0n]
Colias alfacariensis|EZSPN326-09|RVcoll.08-H200||Spain|658[0n]
Colias alfacariensis|EZSPM326-09|RVcoll.08-R051||Spain|658[0n]
Colias alfacariensis|EZSPM307-09|RVcoll.08-J855||Spain|658[0n]
Colias alfacariensis|EZSPC360-09|RVcoll.08-L335||Spain|658[0n]
Colias alfacariensis|EZSPC359-09|RVcoll.07-Z078||Spain|658[0n]
Colias alfacariensis|EZSPM025-09|RVcoll.08-P097||Spain|658[0n]
Colias alfacariensis|EZSPM327-09|RVcoll.08-R064||Spain|658[0n]
Colias alfacariensis|EZSPM438-09|Rvcoll.09-V947||Spain|658[0n]
Colias alfacariensis|EZSPC760-10|RVcoll.08-L424||Spain|658[0n]
Colias alfacariensis|EZSPC761-10|RVcoll.08-L425||Spain|658[0n]
Colias alfacariensis|EZSPC693-10|RVcoll.06-G523||Spain|658[0n]
Colias alfacariensis|EZSPC700-10|RVcoll.06-V779||Spain|658[0n]
Colias alfacariensis|WMB3417-14|RVcoll.09-X019||Spain|658[0n]
Colias alfacariensis|WMB3444-14|RVcoll.09-X572||Spain|658[0n]
Colias alfacariensis|WMB3204-14|RVcoll.08-J205||Spain|658[0n]
Colias alfacariensis|WMB3220-14|RVcoll.08-J731||Spain|658[0n]
Colias alfacariensis|WMB3572-14|RVcoll.11-D989||Spain|658[0n]
Colias alfacariensis|EZSPM439-09|Rvcoll.09-V934||Spain|648[0n]
Colias alfacariensis|EZSPC358-09|RVcoll.08-L336||Spain|636[0n]
Colias alfacariensis|LENOA1351-11|LN-BD1351||France|658[0n]
Colias alfacariensis|BIBSA1162-15|15-M178||Italy|618[0n]
Colias alfacariensis|EULEP1927-15|RVcoll.14-B872||Albania|658[0n]
Colias alfacariensis|EULEP1902-15|RVcoll.14-B785||Albania|658[0n]
Colias alfacariensis|EULEP1660-15|RVcoll.14-G873||Greece|658[0n]
Colias alfacariensis|EULEP1498-15|RVcoll.14-G369||Greece|658[0n]
Colias alfacariensis|EULEP4887-16|RVcoll16J565||Moldova|658[0n]
Colias alfacariensis|WMB4854-14|RVcoll.14-I401||Italy|658[0n]
Colias alfacariensis|WMB1676-13|RVcoll.12-P376||France|658[0n]
Colias alfacariensis|EZROM116-08|RV-07-D232|Genit. examined|Romania|656[0n]
Colias alfacariensis|EZROM887-08|RVcoll.08-M236|Genit. examined|Romania|658[0n]
Colias alfacariensis|EULEP2211-15|RVcoll.14-N386||Ukraine|617[0n]
Colias alfacariensis|EULEP3709-16|RVcoll15J406||France|634[0n]
Colias alfacariensis|WMB3469-14|RVcoll.10-A627||France|658[0n]
Colias alfacariensis|EZROM129-08|RV-07-C105|Genit. examined|Romania|658[0n]
Colias alfacariensis|EZROM118-08|RV-06-K654||Romania|646[0n]
Colias alfacariensis|EZROM619-08|RV-07-C119||Romania|658[0n]
Colias alfacariensis|EZROM618-08|RV-07-C114||Romania|658[0n]
Colias alfacariensis|EZRMN324-08|RVcoll.08-H004|Genit. examined|Romania|658[0n]
Colias alfacariensis|ABOLD010-16|TLMF Lep 21088||Austria|658[0n]
Colias alfacariensis|GBLAA1950-15|BC ZSM Lep 89071||Italy|658[0n]
Colias alfacariensis|OXB873-15|15-A515||Italy|658[0n]

Colias alfacariensis|ABOLD010-16|TLMF Lep 21088||Austria|658[0n]
Colias alfacariensis|GBLAA1950-15|BC ZSM Lep 89071||Italy|658[0n]
Colias alfacariensis|OXB873-15|15-A515||Italy|658[0n]
Colias alfacariensis|OXB1365-15|Rvcoll_15-M721||France|658[0n]
Colias alfacariensis|BIBSA197-15|RVcoll.14-D985||Italy|636[0n]
Colias alfacariensis|BIBSA196-15|RVcoll.14-D984||Italy|658[0n]
Colias alfacariensis|BIBSA591-15|LEP-SS-00211||Italy|658[0n]
Colias alfacariensis|BIBSA1353-15|15-M853||Italy|658[0n]
Colias alfacariensis|BIBSA106-15|RVcoll.14-D534||Italy|658[0n]
Colias alfacariensis|EULEP2151-15|RVcoll.14-K045||Switzerland|658[0n]
Colias alfacariensis|EULEP2130-15|RVcoll.14-J970||Switzerland|658[0n]
Colias alfacariensis|EULEP3707-16|RVcoll15H740||Switzerland|658[0n]
Colias alfacariensis|EULEP3691-16|RVcoll15J458||France|658[0n]
Colias alfacariensis|EULEP3690-16|RVcoll15J123||Switzerland|658[0n]
Colias alfacariensis|WMB5211-14|RVcoll.13-T951||Italy|658[0n]
Colias alfacariensis|WMB5186-14|RVcoll.13-S766||Italy|658[0n]
Colias alfacariensis|WMB5138-14|RVcoll.07-E076||Italy|658[0n]
Colias alfacariensis|WMB5050-14|RVcoll. 14-L181||Italy|658[0n]
Colias alfacariensis|WMB646-11|RVcoll.11-I187||Italy|658[0n]
Colias alfacariensis|WMB2217-13|RVcoll.11-Y035||Italy|658[0n]
Colias alfacariensis|WMB2016-13|RVcoll.12-Q660||Italy|658[0n]
Colias alfacariensis|WMB1720-13|RVcoll.12-P721||France|658[0n]
Colias alfacariensis|WMB827-13|RVcoll.07-E197||Italy|658[0n]
Colias alfacariensis|WMB2687-13|RVcoll.10-B988||France|658[0n]
Colias alfacariensis|WMB3654-14|RVcoll.11-I707||France|658[0n]
Colias alfacariensis|WMB2839-13|RVcoll.12-Q641||Italy|658[0n]
Colias alfacariensis|WMB3426-14|RVcoll.09-X273||France|658[0n]
Colias alfacariensis|WMB108-11|RVcoll.10-B628||France|658[0n]
Colias alfacariensis|EZRMN329-08|RVcoll.08-H035|Genit. examined|Romania|658[0n]
Colias alfacariensis|EZRMN328-08|RVcoll.08-H034|Genit. examined|Romania|658[0n]
Colias alfacariensis|LENOA1343-11|LN-BD1343||France|658[0n]
Colias alfacariensis|GWORR424-10|BC ZSM Lep 29640||Italy|658[0n]
Colias alfacariensis|PHLAH460-12|TLMF Lep 08279||Austria|658[0n]
Colias alfacariensis|LEATG524-14|TLMF Lep 14311||Italy|658[0n]
Colias alfacariensis|PHLAH698-12|TLMF Lep 08517||Austria|658[0n]
Colias alfacariensis|PHLSA631-11|TLMF Lep 06086||Italy|658[0n]
Colias alfacariensis|LEATJ216-15|TLMF Lep 18646||Italy|658[0n]
Leptidea duponcheli|EULEP5735-17|RVcollLR08D261|Genit. examined|Greece|676[0n]
Leptidea duponcheli|EULEP1558-15|RVcoll.14-G554||Macedonia|614[0n]
Leptidea duponcheli|EULEP5425-17|RVcoll11E273|Genit. examined|France|658[0n]
Leptidea duponcheli|EULEP5442-17|RVcoll11H419|Genit. examined|France|658[0n]
Leptidea duponcheli|EULEP1280-15|RVcoll.14-F687||Greece|658[0n]
Leptidea duponcheli|EULEP1518-15|RVcoll.14-C476||Greece|658[0n]
Leptidea duponcheli|EULEP1693-15|RVcoll.14-H040||Greece|658[0n]
Leptidea duponcheli|EULEP2913-15|RVcoll.14-U866||Albania|658[0n]
Leptidea duponcheli|OXB1603-16|OXB-TGS-1321||France|658[0n]
Leptidea duponcheli|GBGLP113-13|KC866120||Macedonia|658[0n]
Leptidea duponcheli|EULEP5332-17|RVcoll09V207||France|676[0n]
Leptidea duponcheli|EULEP5333-17|RVcoll09V208||France|676[0n]
Leptidea duponcheli|EULEP5385-17|RVcoll10B373|Genit. examined|Bulgaria|676[0n]
Leptidea duponcheli|GBMIN34113-13|JF512570||France|676[0n]
Leptidea duponcheli|EULEP2256-15|RVcoll.14-N439||Ukraine|607[1n]
Leptidea duponcheli|EULEP5487-17|RVcoll12R257|Genit. examined|Greece|655[0n]
Leptidea morsei|EULEP2914-15|RVcoll.14-U867||Slovenia|658[0n]
Leptidea morsei|EULEP2916-15|RVcoll.14-U869||Slovenia|613[0n]
Leptidea morsei|EULEP2915-15|RVcoll.14-U868||Slovenia|610[0n]
Leptidea morsei|EZROM237-08|RV-07-C176|Genit. examined|Romania|658[0n]
Leptidea morsei|EZROM348-08|RV-08-A026|Genit. examined|Romania|658[0n]
Leptidea morsei|EULEP4128-16|RVcoll14O558||Romania|658[0n]
Leptidea morsei|EZRMN009-08|RVcoll.08-M499||Romania|658[0n]
Leptidea morsei|EZRMN008-08|RVcoll.08-M498||Romania|658[0n]
Leptidea juvernica|GBGLP241-13|KC865992||Ireland|658[0n]
Leptidea juvernica|GBGLP242-13|KC865991||Ireland|658[0n]
Leptidea juvernica|GBGLP243-13|KC865990||Ireland|658[0n]
Leptidea juvernica|GBGLP244-13|KC865989||Ireland|658[0n]
Leptidea juvernica|GBMIN34040-13|JF512716||Ireland|658[0n]
Leptidea juvernica|GBMIN34041-13|JF512714||Ireland|658[0n]
Leptidea juvernica|GBMIN34160-13|JF512715||Ireland|658[0n]
Leptidea juvernica|GBMIN34161-13|JF512713||Ireland|658[0n]
Leptidea juvernica|GBGLP102-13|KC866131||Ireland|643[0n]
Leptidea juvernica|EULEP5301-17|RVcoll08P980||Ireland|658[0n]
Leptidea juvernica|EULEP5500-17|RVcoll12Z138|Genit. examined|Ireland|658[0n]
Leptidea juvernica|EULEP5499-17|RVcoll12Z039|Genit. examined|United Kingdom|658[0n]
Leptidea juvernica|EULEP5498-17|RVcoll12Z036|Genit. examined|United Kingdom|658[0n]
Leptidea juvernica|EULEP5495-17|RVcoll12Z021|Genit. examined|Ireland|658[0n]
Leptidea juvernica|EULEP5494-17|RVcoll12Z010|Genit. examined|Ireland|658[0n]
Leptidea juvernica|GBGLP248-13|KC865985||Belgium|658[0n]
Leptidea juvernica|GBGLP247-13|KC865986||Belgium|658[0n]
Leptidea juvernica|GBLAB315-13|BC ZSM Lep 72616|Identified by barcode|Germany|658[0n]
Leptidea juvernica|EULEP5444-17|RVcoll11H425|Genit. examined|France|658[0n]
Leptidea juvernica|EULEP5422-17|RVcoll11E258|Genit. examined|France|658[0n]
Leptidea juvernica|EULEP5421-17|RVcoll11E257|Genit. examined|France|658[0n]
Leptidea juvernica|EULEP5420-17|RVcoll11E254|Genit. examined|France|658[0n]
Leptidea juvernica|EULEP5373-17|RVcoll10A264|Genit. examined|France|658[0n]
Leptidea juvernica|EULEP5372-17|RVcoll10A263|Genit. examined|France|658[0n]
Leptidea juvernica|EULEP5371-17|RVcoll10A262|Genit. examined|France|658[0n]
Leptidea juvernica|EULEP5370-17|RVcoll10A259|Genit. examined|France|658[0n]
Leptidea juvernica|EULEP5367-17|RVcoll09X268|Genit. examined|France|658[0n]
Leptidea juvernica|GBMIN34190-13|JF512655||France|658[0n]
Leptidea juvernica|GBGLP249-13|KC865984||Belgium|658[0n]
Leptidea juvernica|PHLAI560-13|TLMF Lep 09122||Austria|658[0n]
Leptidea juvernica|FBLMT876-09|BC ZSM Lep 25436|ID verified by dissection|Germany|658[0n]
Leptidea juvernica|FBLMZ481-12|BC ZSM Lep 61237||Germany|658[0n]
Leptidea juvernica|EULEP5724-17|RVcollKK0901||Russia|658[0n]
Leptidea juvernica|EULEP5730-17|RVcollKK0907||Russia|658[0n]
Leptidea juvernica|EULEP357-14|MM23842||Lithuania|658[0n]
Leptidea juvernica|LEFIJ2181-14|MM23256||Finland|658[0n]
Leptidea juvernica|EULEP5316-17|RVcoll08Y008|Genit. examined|Russia|673[0n]
Leptidea juvernica|EULEP5312-17|RVcoll08Y004|Genit. examined|Russia|658[0n]
Leptidea juvernica|EULEP5356-17|RVcoll09V653|Genit. examined|Romania|658[0n]
Leptidea juvernica|EULEP5350-17|RVcoll09V638|Genit. examined|Romania|658[0n]
Leptidea juvernica|EZRMN014-08|RVcoll.08-M322|Genit. examined|Romania|658[0n]
[illegible]

Leptidea juvernica|EULEP5356-17|RVcoll09V653|Genit. examined|Romania|658[0n]
Leptidea juvernica|EULEP5350-17|RVcoll09V638|Genit. examined|Romania|658[0n]
Leptidea juvernica|EZRMN014-08|RVcoll.08-M322|Genit. examined|Romania|658[0n]
Leptidea juvernica|GBLAD166-14|BC ZSM Lep 78737||Germany|658[0n]
Leptidea juvernica|FBLMU118-09|BC ZSM Lep 25628|Identified by barcode|Germany|653[0n]
Leptidea juvernica|EULEP5299-17|RVcoll08M325|Genit. examined|Romania|676[0n]
Leptidea juvernica|EULEP5311-17|RVcoll08Y003|Genit. examined|Russia|676[0n]
Leptidea juvernica|EULEP5315-17|RVcoll08Y007|Genit. examined|Russia|676[0n]
Leptidea juvernica|EULEP5318-17|RVcoll08Y010|Genit. examined|Russia|676[0n]
Leptidea juvernica|EULEP5320-17|RVcoll08Y012|Genit. examined|Russia|676[0n]
Leptidea juvernica|EULEP358-14|MM23843||Lithuania|658[0n]
Leptidea juvernica|EULEP5740-17|RVcollLR08D680|Genit. examined|Slovenia|658[0n]
Leptidea juvernica|EULEP5731-17|RVcollKK0908||Russia|658[0n]
Leptidea juvernica|EULEP5729-17|RVcollKK0906|Genit. examined|Russia|658[0n]
Leptidea juvernica|EULEP5728-17|RVcollKK0905||Russia|658[0n]
Leptidea juvernica|EULEP5727-17|RVcollKK0904|Genit. examined|Russia|658[0n]
Leptidea juvernica|EULEP5726-17|RVcollKK0903||Russia|658[0n]
Leptidea juvernica|EULEP5725-17|RVcollKK0902|Genit. examined|Russia|658[0n]
Leptidea juvernica|LEASS874-17|KLM Lep 08379||Austria|658[0n]
Leptidea juvernica|GBLAB770-13|BC ZSM Lep 75541||Germany|658[0n]
Leptidea juvernica|LEFIJ2213-14|MM23366||Finland|658[0n]
Leptidea juvernica|LEASS871-17|KLM Lep 08376||Austria|658[0n]
Leptidea juvernica|COLFH1415-16|MM25524||Finland|658[0n]
Leptidea juvernica|LEFIJ2643-15|MM24186||Finland|658[0n]
Leptidea juvernica|LEASS851-17|TLMF Lep 22499||Austria|658[0n]
Leptidea juvernica|LEASS849-17|TLMF Lep 22497||Austria|658[0n]
Leptidea juvernica|LEASS480-17|TLMF Lep 22128||Austria|658[0n]
Leptidea juvernica|LEFIJ2180-14|MM23255||Finland|658[0n]
Leptidea juvernica|EULEP5408-17|RVcoll11D557|Genit. examined|Russia|658[0n]
Leptidea juvernica|EULEP5407-17|RVcoll11D556|Genit. examined|Russia|658[0n]
Leptidea juvernica|EULEP5406-17|RVcoll11D555|Genit. examined|Russia|658[0n]
Leptidea juvernica|EULEP5405-17|RVcoll11D554|Genit. examined|Russia|658[0n]
Leptidea juvernica|EULEP5395-17|RVcoll10C244|Genit. examined|Czech Republic|658[0n]
Leptidea juvernica|EULEP5392-17|RVcoll10B480|Genit. examined|Sweden|658[0n]
Leptidea juvernica|EULEP5391-17|RVcoll10B479|Genit. examined|Sweden|658[0n]
Leptidea juvernica|EULEP5390-17|RVcoll10B471|Genit. examined|Sweden|658[0n]
Leptidea juvernica|EULEP5389-17|RVcoll10B468|Genit. examined|Sweden|658[0n]
Leptidea juvernica|EULEP5358-17|RVcoll09V664|Genit. examined|Romania|658[0n]
Leptidea juvernica|EULEP5354-17|RVcoll09V648|Genit. examined|Romania|658[0n]
Leptidea juvernica|EULEP5353-17|RVcoll09V644|Genit. examined|Romania|658[0n]
Leptidea juvernica|EULEP5314-17|RVcoll08Y006|Genit. examined|Russia|658[0n]
Leptidea juvernica|EULEP5313-17|RVcoll08Y005|Genit. examined|Russia|658[0n]
Leptidea juvernica|GBGLP254-13|KC865979||Czech Republic|658[0n]
Leptidea juvernica|OXB376-15|RVcoll.14-N090||Italy|658[0n]
Leptidea juvernica|EULEP5310-17|RVcoll08Y002|Genit. examined|Russia|658[0n]
Leptidea juvernica|EULEP5309-17|RVcoll08Y001|Genit. examined|Russia|658[0n]
Leptidea juvernica|EULEP5302-17|RVcoll08R369|Genit. examined|Italy|658[0n]
Leptidea juvernica|EULEP5298-17|RVcoll08M313|Genit. examined|Romania|658[0n]
Leptidea juvernica|EULEP5547-17|RVcoll14E324|Genit. examined|Sweden|658[0n]
Leptidea juvernica|EULEP5463-17|RVcoll12M089|Genit. examined|Finland|658[0n]
Leptidea juvernica|EZRMN017-08|RVcoll.08-M331|Genit. examined|Romania|658[0n]
Leptidea juvernica|EZRMN016-08|RVcoll.08-M324|Genit. examined|Romania|658[0n]
Leptidea juvernica|EZRMN013-08|RVcoll.08-M312|Genit. examined|Romania|658[0n]
Leptidea juvernica|EZRMN012-08|RVcoll.08-M311|Genit. examined|Romania|658[0n]
Leptidea juvernica|EZRMN011-08|RVcoll.08-M310|Genit. examined|Romania|658[0n]
Leptidea juvernica|EZRMN015-08|RVcoll.08-M323|Genit. examined|Romania|658[0n]
Leptidea juvernica|EZROM703-08|RV-07-E553|Genit. examined|Romania|658[0n]
Leptidea juvernica|LEFIJ2040-13|MM22892||Finland|658[0n]
Leptidea juvernica|FBLMZ163-12|BC ZSM Lep 51419|Identified by barcode|Germany|658[0n]
Leptidea juvernica|PHLAW041-13|TLMF Lep 09838||Austria|658[0n]
Leptidea juvernica|EULEP5357-17|RVcoll09V655|Genit. examined|Romania|658[0n]
Leptidea juvernica|EULEP5355-17|RVcoll09V652|Genit. examined|Romania|658[0n]
Leptidea juvernica|GBGLP256-13|KC865977||Sweden|658[0n]
Leptidea juvernica|GBGLP255-13|KC865978||Sweden|658[0n]
Leptidea juvernica|EULEP5319-17|RVcoll08Y011|Genit. examined|Russia|658[0n]
Leptidea juvernica|EULEP5317-17|RVcoll08Y009|Genit. examined|Russia|658[0n]
Leptidea juvernica|GBGLP258-13|KC865975||Sweden|658[0n]
Leptidea juvernica|GBGLP257-13|KC865976||Sweden|658[0n]
Leptidea juvernica|GBGLP260-13|KC865973||Germany|658[0n]
Leptidea juvernica|GBGLP259-13|KC865974||Sweden|658[0n]
Leptidea juvernica|EULEP5349-17|RVcoll09V637|Genit. examined|Romania|658[0n]
Leptidea juvernica|EULEP5348-17|RVcoll09V633|Genit. examined|Romania|658[0n]
Leptidea juvernica|EULEP5352-17|RVcoll09V641|Genit. examined|Romania|658[0n]
Leptidea juvernica|EULEP5351-17|RVcoll09V639|Genit. examined|Romania|658[0n]
Leptidea juvernica|GBGLP262-13|KC865971||Czech Republic|658[0n]
Leptidea juvernica|GBGLP261-13|KC865972||Czech Republic|658[0n]
Leptidea juvernica|GBGLP263-13|KC865970||Czech Republic|658[0n]
Leptidea juvernica|GBGLP264-13|KC865969||Czech Republic|658[0n]
Leptidea juvernica|GBGLP265-13|KC865968||Czech Republic|658[0n]
Leptidea juvernica|GBGLP272-13|KC865961||Germany|658[0n]
Leptidea juvernica|GBGLP279-13|KC865954||Sweden|658[0n]
Leptidea juvernica|GBGLP284-13|KC865949||Italy|658[0n]
Leptidea juvernica|EULEP5488-17|RVcoll12R269|Genit. examined|Greece|655[0n]
Leptidea juvernica|EULEP5489-17|RVcoll12R270|Genit. examined|Greece|655[0n]
Leptidea juvernica|EULEP5490-17|RVcoll12R271|Genit. examined|Greece|655[0n]
Leptidea juvernica|GBMIN87363-17|KU355287||Bulgaria|655[0n]
Leptidea juvernica|GBMIN87364-17|KU355284||Ukraine|655[0n]
Leptidea juvernica|GBGL20659-18|KU355288|||655[0n]
Leptidea juvernica|GBMIN87365-17|KU355283||Ukraine|655[0n]
Leptidea juvernica|GBMIN87366-17|KU355285||Bulgaria|655[0n]
Leptidea juvernica|GBMIN87367-17|KU355286||Bulgaria|655[0n]
Leptidea juvernica|GBGLP107-13|KC866126||Sweden|645[0n]
Leptidea juvernica|GBGLP111-13|KC866122||Sweden|645[0n]
Leptidea juvernica|GBGLP112-13|KC866121||Sweden|645[0n]
Leptidea juvernica|GBGLP108-13|KC866125||Sweden|645[0n]
Leptidea juvernica|GBGLP109-13|KC866124||Sweden|645[0n]
Leptidea juvernica|GBGLP110-13|KC866123||Sweden|645[0n]
Leptidea juvernica|GBGLP103-13|KC866130||Germany|643[0n]
Leptidea juvernica|LEFID125-10|MM05862||Estonia|622[0n]
Leptidea juvernica|FBLMU383-09|BC ZSM Lep 27033|Identified by barcode|Germany|644[0n]
Leptidea juvernica|PHLAH475-12|TLMF Lep 08294||Austria|615[0n]
Leptidea reali|EZSPN400-09|RVcoll.08-H468|Genit. examined|Spain|658[0n]
Leptidea reali|GBMIN34210-13|JF512615||Italy|676[0n]

Leptidea juvernica|PHLAH475-12|TLMF Lep 08294||Austria|615[0n]
Leptidea reali|EZSPN400-09|RVcoll.08-H468|Genit. examined|Spain|658[0n]
Leptidea reali|GBMIN34210-13|JF512615||Italy|676[0n]
Leptidea reali|GBMIN34046-13|JF512704||Italy|658[0n]
Leptidea reali|GBGLP115-13|KC866118||Spain|658[0n]
Leptidea reali|EULEP5426-17|RVcoll11E280|Genit. examined|France|658[0n]
Leptidea reali|GBMIN34209-13|JF512617||Spain|676[0n]
Leptidea reali|GBMIN34090-13|JF512616||Italy|676[0n]
Leptidea reali|EZSPM703-12|Rvcoll. 12-L116|Genit. examined|Spain|658[0n]
Leptidea reali|EULEP5247-17|RVcoll07E083|Genit. examined|Italy|658[0n]
Leptidea reali|EZSPN254-09|RVcoll.07-W154|Genit. examined|Spain|658[1n]
Leptidea reali|EULEP5325-17|RVcoll09T247|Genit. examined|France|676[0n]
Leptidea reali|EULEP5324-17|RVcoll09T246|Genit. examined|France|676[0n]
Leptidea reali|EULEP5375-17|RVcoll10A411|Genit. examined|France|676[0n]
Leptidea reali|EULEP5326-17|RVcoll09T249|Genit. examined|France|676[0n]
Leptidea reali|GBMIN34091-13|JF512614||Italy|676[0n]
Leptidea reali|EULEP5376-17|RVcoll10A412|Genit. examined|France|676[0n]
Leptidea reali|GBMIN34093-13|JF512610||Italy|676[0n]
Leptidea reali|GBMIN34092-13|JF512612||Italy|676[0n]
Leptidea reali|EULEP5290-17|RVcoll08J396|Genit. examined|Spain|676[0n]
Leptidea reali|GBMIN34211-13|JF512613||Italy|676[0n]
Leptidea reali|GBMIN34212-13|JF512611||Italy|676[0n]
Leptidea reali|GBMIN34213-13|JF512609||Italy|676[0n]
Leptidea reali|EULEP5362-17|RVcoll09V733|Genit. examined|Spain|658[0n]
Leptidea reali|EULEP5361-17|RVcoll09V714|Genit. examined|Spain|658[0n]
Leptidea reali|EULEP5480-17|RVcoll12P124|Genit. examined|France|658[0n]
Leptidea reali|EULEP5479-17|RVcoll12P122|Genit. examined|France|658[0n]
Leptidea reali|EZSPC474-09|RVcoll.07-C636|Genit. examined|Spain|658[0n]
Leptidea reali|EZSPM870-12|Rvcoll.120711LF04||Spain|658[0n]
Leptidea reali|EULEP5364-17|RVcoll09V743|Genit. examined|Spain|658[0n]
Leptidea reali|EULEP5363-17|RVcoll09V734|Genit. examined|Spain|658[0n]
Leptidea reali|EULEP093-14|RVcoll.09-T221|Genit. examined|Spain|658[0n]
Leptidea reali|EULEP5244-17|RVcoll07E080|Genit. examined|Italy|658[0n]
Leptidea reali|EULEP5424-17|RVcoll11E271|Genit. examined|France|658[0n]
Leptidea reali|EULEP5366-17|RVcoll09V815|Genit. examined|Spain|658[0n]
Leptidea reali|EULEP5482-17|RVcoll12Q377|Genit. examined|France|658[0n]
Leptidea reali|EULEP5481-17|RVcoll12P130|Genit. examined|France|658[0n]
Leptidea reali|EULEP5428-17|RVcoll11E284|Genit. examined|France|658[0n]
Leptidea reali|EULEP5427-17|RVcoll11E283|Genit. examined|France|658[0n]
Leptidea reali|EULEP5484-17|RVcoll12Q381||France|658[0n]
Leptidea reali|EULEP5483-17|RVcoll12Q378|Genit. examined|France|658[0n]
Leptidea reali|EULEP5908-18|RVcoll17D396||France|658[0n]
Leptidea reali|EULEP5443-17|RVcoll11H420|Genit. examined|France|658[0n]
Leptidea reali|EULEP5441-17|RVcoll11H418|Genit. examined|France|658[0n]
Leptidea reali|EULEP5440-17|RVcoll11H414|Genit. examined|France|658[0n]
Leptidea reali|EULEP5439-17|RVcoll11H408|Genit. examined|France|658[0n]
Leptidea reali|EULEP5431-17|RVcoll11E288|Genit. examined|France|658[0n]
Leptidea reali|EULEP5430-17|RVcoll11E286|Genit. examined|France|658[0n]
Leptidea reali|EULEP5429-17|RVcoll11E285|Genit. examined|France|658[0n]
Leptidea reali|EULEP5246-17|RVcoll07E082|Genit. examined|Italy|658[0n]
Leptidea reali|EULEP5245-17|RVcoll07E081|Genit. examined|Italy|658[0n]
Leptidea reali|EULEP5269-17|RVcoll08H280|Genit. examined|Spain|658[0n]
Leptidea reali|EULEP5265-17|RVcoll07W134|Genit. examined|Spain|658[0n]
Leptidea reali|EULEP5294-17|RVcoll08L090|Genit. examined|Spain|658[0n]
Leptidea reali|EULEP5293-17|RVcoll08L078|Genit. examined|Spain|658[0n]
Leptidea reali|EULEP5327-17|RVcoll09T250|Genit. examined|France|658[0n]
Leptidea reali|EULEP5323-17|RVcoll09T245|Genit. examined|France|658[0n]
Leptidea reali|EULEP5334-17|RVcoll09V231|Genit. examined|France|658[0n]
Leptidea reali|EULEP5328-17|RVcoll09T251|Genit. examined|France|658[0n]
Leptidea reali|EULEP5336-17|RVcoll09V321|Genit. examined|Spain|658[0n]
Leptidea reali|EULEP5335-17|RVcoll09V320|Genit. examined|Spain|658[0n]
Leptidea reali|EULEP5345-17|RVcoll09V344|Genit. examined|Spain|658[0n]
Leptidea reali|EULEP5337-17|RVcoll09V325|Genit. examined|Spain|658[0n]
Leptidea reali|EULEP5360-17|RVcoll09V713|Genit. examined|Spain|658[0n]
Leptidea reali|EULEP5347-17|RVcoll09V347|Genit. examined|Spain|658[0n]
Leptidea reali|BIBSA940-15|16-A031||Italy|658[0n]
Leptidea reali|EULEP307-14|RVcoll.07-W133|Genit. examined|Spain|658[0n]
Leptidea reali|EULEP5227-17|RVcoll07C613|Genit. examined|Spain|658[0n]
Leptidea reali|EULEP5224-17|RVcoll07C279|Genit. examined|Spain|658[0n]
Leptidea reali|OXB1577-16|OXB-TGS-1295||France|658[0n]
Leptidea reali|BIBSA228-15|RVcoll.14-E019||Italy|658[0n]
Leptidea reali|OXB1581-16|OXB-TGS-1299||France|658[0n]
Leptidea reali|OXB1578-16|OXB-TGS-1296||France|658[0n]
Leptidea reali|OXB1604-16|OXB-TGS-1322||France|658[0n]
Leptidea reali|OXB1582-16|OXB-TGS-1300||France|658[0n]
Leptidea reali|OXB1607-16|OXB-TGS-1325||France|658[0n]
Leptidea reali|GBGLP117-13|KC866116||Spain|658[0n]
Leptidea reali|GBGLP118-13|KC866115||Spain|658[0n]
Leptidea reali|GBGLP119-13|KC866114||Spain|658[0n]
Leptidea reali|GBGLP120-13|KC866113||Spain|658[0n]
Leptidea reali|GBGLP121-13|KC866112||Spain|658[0n]
Leptidea reali|GBGLP116-13|KC866117||Spain|658[0n]
Leptidea reali|GBGLP122-13|KC866111||Spain|658[0n]
Leptidea reali|GBMIN34182-13|JF512671||France|658[0n]
Leptidea reali|EULEP5322-17|RVcoll09T203|Genit. examined|Spain|643[0n]
Leptidea reali|EULEP5478-17|RVcoll12P040|Genit. examined|France|645[0n]
Leptidea reali|EZSPC468-09|RVcoll.08-J391|Genit. examined|Spain|633[0n]
Leptidea reali|EZSPN154-09|RVcoll.08-R059|Genit. examined|Spain|658[0n]
Leptidea sinapis|GWORO852-09|BC ZSM Lep 30544||Germany|658[0n]
Leptidea sinapis|EULEP5816-18|RVcoll12L209||Spain|658[0n]
Leptidea sinapis|EULEP5383-17|RVcoll10B320|Genit. examined|Bulgaria|658[0n]
Leptidea sinapis|EULEP5251-17|RVcoll07E174|Genit. examined|Italy|676[0n]
Leptidea sinapis|EULEP5254-17|RVcoll07E217|Genit. examined|Italy|676[0n]
Leptidea sinapis|EULEP5255-17|RVcoll07E237|Genit. examined|Italy|676[0n]
Leptidea sinapis|EULEP5253-17|RVcoll07E216|Genit. examined|Italy|658[0n]
Leptidea sinapis|ABOLB055-15|TLMF Lep 17060||Italy|658[0n]
Leptidea sinapis|EULEP5402-17|RVcoll10C255|Genit. examined|Italy|658[0n]
Leptidea sinapis|EULEP5400-17|RVcoll10C253|Genit. examined|Italy|658[0n]
Leptidea sinapis|ABOLB056-15|TLMF Lep 17061||Italy|658[0n]
Leptidea sinapis|PHLAI531-13|TLMF Lep 09093||Italy|658[0n]
Leptidea sinapis|EULEP5736-17|RVcollLR08D355|Genit. examined|Greece|658[0n]
Leptidea sinapis|EULEP5741-17|RVcollLR08D800|Genit. examined|Italy|658[0n]
Leptidea sinapis|EULEP5742-17|RVcollLR08D801|Genit. examined|Italy|658[0n]

Leptidea sinapis|PHLAI531-13|TLMF Lep 09093||Italy|658[0n]
Leptidea sinapis|EULEP5736-17|RVcollLR08D355|Genit. examined|Greece|658[0n]
Leptidea sinapis|EULEP5741-17|RVcollLR08D800|Genit. examined|Italy|658[0n]
Leptidea sinapis|EULEP5742-17|RVcollLR08D801|Genit. examined|Italy|658[0n]
Leptidea sinapis|BIBSA1178-15|15-M209||Italy|658[0n]
Leptidea sinapis|OXB633-15|14-O022||Italy|658[0n]
Leptidea sinapis|OXB344-15|RVcoll.14-N058||Italy|658[0n]
Leptidea sinapis|GBGLP140-13|KC866093||Italy|658[0n]
Leptidea sinapis|GBGLP141-13|KC866092||Croatia|658[0n]
Leptidea sinapis|GBMIN34144-13|JF513033||Italy|658[0n]
Leptidea sinapis|GBMIN87369-17|KU355293||Greece|655[0n]
Leptidea sinapis|PHLAI530-13|TLMF Lep 09092||Italy|635[0n]
Leptidea sinapis|PHLAI532-13|TLMF Lep 09094||Italy|631[0n]
Leptidea sinapis|GWOSZ097-11|BC ZSM Lep 41853||Italy|637[0n]
Leptidea sinapis|LEATH717-14|TLMF Lep 15929||Italy|634[0n]
Leptidea sinapis|LEATA122-13|TLMF Lep 09539||Italy|658[0n]
Leptidea sinapis|GBMIN87370-17|KU355294||Greece|655[0n]
Leptidea sinapis|EULEP5738-17|RVcollLR08D573|Genit. examined|Bulgaria|658[0n]
Leptidea sinapis|EZSPM701-12|Rvcoll. 12-L113|Genit. examined|Spain|658[0n]
Leptidea sinapis|GBLAA1318-15|BC ZSM Lep 87204||Germany|658[1n]
Leptidea sinapis|EULEP5260-17|RVcoll07E254|Genit. examined|France|676[0n]
Leptidea sinapis|GBMIN34164-13|JF512707||Switzerland|658[0n]
Leptidea sinapis|GBGLP136-13|KC866097||France|658[0n]
Leptidea sinapis|GBGLP152-13|KC866081||Spain|658[0n]
Leptidea sinapis|OXB1580-16|OXB-TGS-1298||France|658[0n]
Leptidea sinapis|GBLAA1331-15|BC ZSM Lep 87027||Germany|658[0n]
Leptidea sinapis|GBLAC279-13|BC ZSM Lep 78660||Germany|658[0n]
Leptidea sinapis|EULEP5906-18|RVcoll17D378||France|658[0n]
Leptidea sinapis|EULEP5867-18|RVcoll15O481||Spain|658[0n]
Leptidea sinapis|EULEP5812-18|RVcoll12L196||Spain|658[0n]
Leptidea sinapis|EULEP5368-17|RVcoll09X562|Genit. examined|Spain|658[0n]
Leptidea sinapis|WMB3734-14|RVcoll.12-M087||France|658[0n]
Leptidea sinapis|EZSPM237-09|RVcoll.08-R438|Genit. examined|Spain|658[0n]
Leptidea sinapis|EZSPM236-09|RVcoll.08-R436||Spain|658[0n]
Leptidea sinapis|EULEP5486-17|RVcoll12Q589|Sicily|Italy|658[0n]
Leptidea sinapis|EULEP5262-17|RVcoll07E256|Genit. examined|France|676[0n]
Leptidea sinapis|EULEP5261-17|RVcoll07E255|Genit. examined|France|676[0n]
Leptidea sinapis|EULEP5257-17|RVcoll07E250|Genit. examined|France|676[0n]
Leptidea sinapis|GBGLP143-13|KC866090||Italy|658[0n]
Leptidea sinapis|BIBSA1873-17|RVcoll16C772||Italy|658[0n]
Leptidea sinapis|BIBSA1872-17|RVcoll16C770||Italy|658[0n]
Leptidea sinapis|WMB3874-14|RVcoll.11-Y080|Genit. examined|Italy|658[0n]
Leptidea sinapis|GBGLP142-13|KC866091||Italy|658[0n]
Leptidea sinapis|GBGLP144-13|KC866089||Italy|658[0n]
Leptidea sinapis|WMB4179-14|RVcoll.13-S791|Genit. examined|Italy|658[0n]
Leptidea sinapis|EULEP5226-17|RVcoll07C470|Genit. examined|Spain|670[0n]
Leptidea sinapis|EULEP5248-17|RVcoll07E138|Genit. examined|Italy|676[0n]
Leptidea sinapis|EULEP5241-17|RVcoll07D939|Genit. examined|Romania|676[0n]
Leptidea sinapis|EULEP5258-17|RVcoll07E252|Genit. examined|France|676[0n]
Leptidea sinapis|EULEP5256-17|RVcoll07E249|Genit. examined|France|676[0n]
Leptidea sinapis|EULEP5266-17|RVcoll08H275|Genit. examined|Spain|676[0n]
Leptidea sinapis|EULEP5259-17|RVcoll07E253|Genit. examined|France|676[0n]
Leptidea sinapis|EULEP5232-17|RVcoll07D089|Genit. examined|Romania|676[0n]
Leptidea sinapis|EULEP5223-17|RVcoll06N005|Genit. examined|Romania|676[0n]
Leptidea sinapis|EULEP5342-17|RVcoll09V331|Genit. examined|Spain|676[0n]
Leptidea sinapis|EULEP5338-17|RVcoll09V326|Genit. examined|Spain|676[0n]
Leptidea sinapis|EULEP5344-17|RVcoll09V342|Genit. examined|Spain|676[0n]
Leptidea sinapis|EULEP5343-17|RVcoll09V341|Genit. examined|Spain|676[0n]
Leptidea sinapis|EULEP5346-17|RVcoll09V345|Genit. examined|Spain|676[0n]
Leptidea sinapis|GBMIN34106-13|JF512584||Romania|676[0n]
Leptidea sinapis|GBMIN34222-13|JF512591||Italy|676[0n]
Leptidea sinapis|GBGLP175-13|KC866058||United Kingdom|658[0n]
Leptidea sinapis|GBGLP174-13|KC866059||United Kingdom|658[0n]
Leptidea sinapis|GBGLP171-13|KC866062||United Kingdom|658[0n]
Leptidea sinapis|GBGLP170-13|KC866063||United Kingdom|658[0n]
Leptidea sinapis|GBGLP160-13|KC866073||Italy|658[0n]
Leptidea sinapis|GBGLP159-13|KC866074||Ireland|658[0n]
Leptidea sinapis|GBGLP154-13|KC866079||Italy|658[0n]
Leptidea sinapis|OXB1613-16|OXB-TGS-1331||France|658[0n]
Leptidea sinapis|OXB1575-16|OXB-TGS-1293||France|658[0n]
Leptidea sinapis|OXB986-15|15-A936||Italy|658[0n]
Leptidea sinapis|BIBSA523-15|LEP-SS-00143||Italy|658[0n]
Leptidea sinapis|BIBSA699-15|RVcoll. 12-Q854|Genit. examined|Italy|658[0n]
Leptidea sinapis|BIBSA1410-15|15-N019||Italy|658[0n]
Leptidea sinapis|BCLEP281-17|LEP-SS-00696||Italy|658[0n]
Leptidea sinapis|EULEP5907-18|RVcoll17D388||France|658[0n]
Leptidea sinapis|EULEP5868-18|RVcoll15O482||Spain|658[0n]
Leptidea sinapis|EULEP5852-18|RVcoll14V699||Spain|658[0n]
Leptidea sinapis|EULEP5850-18|RVcoll14V697||Spain|658[0n]
Leptidea sinapis|EULEP5849-18|RVcoll14V696||Spain|658[0n]
Leptidea sinapis|EULEP5737-17|RVcollLR08D389|Genit. examined|Greece|658[0n]
Leptidea sinapis|EULEP5734-17|RVcollLR08D204|Genit. examined|Greece|658[0n]
Leptidea sinapis|EULEP5701-17|RVcoll16L044||Italy|658[0n]
Leptidea sinapis|EULEP5818-18|RVcoll12L216|Genit. examined|Spain|658[0n]
Leptidea sinapis|EULEP5817-18|RVcoll12L213||Spain|658[0n]
Leptidea sinapis|EULEP5814-18|RVcoll12L198||Spain|658[0n]
Leptidea sinapis|EULEP5813-18|RVcoll12L197||Spain|658[0n]
Leptidea sinapis|EULEP5811-18|RVcoll12L189||Spain|658[0n]
Leptidea sinapis|EULEP5810-18|RVcoll12L188||Spain|658[0n]
Leptidea sinapis|EULEP5809-18|RVcoll12L187||Spain|658[0n]
Leptidea sinapis|EULEP5459-17|RVcoll11J573||Italy|658[0n]
Leptidea sinapis|EULEP5446-17|RVcoll11I182|Genit. examined|Italy|658[0n]
Leptidea sinapis|EULEP5401-17|RVcoll10C254|Genit. examined|Italy|658[0n]
Leptidea sinapis|EULEP5399-17|RVcoll10C252|Genit. examined|Italy|658[0n]
Leptidea sinapis|EULEP5398-17|RVcoll10C251|Genit. examined|Italy|658[0n]
Leptidea sinapis|GBGLP164-13|KC866069||France|658[0n]
Leptidea sinapis|GBGLP163-13|KC866070||Italy|658[0n]
Leptidea sinapis|GBGLP158-13|KC866075||Ireland|658[0n]
Leptidea sinapis|GBGLP157-13|KC866076||Spain|658[0n]
Leptidea sinapis|OXB1609-16|OXB-TGS-1327||France|658[0n]
Leptidea sinapis|OXB1576-16|OXB-TGS-1294||France|658[0n]
Leptidea sinapis|EULEP5331-17|RVcoll09V199|Corsica. Genit. examined|France|658[0n]
Leptidea sinapis|EULEP5305-17|RVcoll08R552|Genit. examined|Italy|658[0n]

Leptidea sinapis|OXB1576-16|OXB-TGS-1294||France|658[0n]
Leptidea sinapis|EULEP5331-17|RVcoll09V199|Corsica. Genit. examined|France|658[0n]
Leptidea sinapis|EULEP5305-17|RVcoll08R552|Genit. examined|Italy|658[0n]
Leptidea sinapis|OXB596-15|14-N985||Italy|658[0n]
Leptidea sinapis|OXB876-15|15-A518||Italy|658[0n]
Leptidea sinapis|EULEP5304-17|RVcoll08R551||Italy|658[0n]
Leptidea sinapis|EULEP5296-17|RVcoll08L578|Genit. examined|Ireland|658[0n]
Leptidea sinapis|OXB834-15|14-U797|Genit. examined|Italy|658[0n]
Leptidea sinapis|BIBSA037-14|LEP-SS-00037||Italy|658[0n]
Leptidea sinapis|EULEP5496-17|RVcoll12Z034|Genit. examined|Ireland|658[0n]
Leptidea sinapis|EULEP5468-17|RVcoll12M497|Genit. examined|Italy|658[0n]
Leptidea sinapis|BIBSA036-14|LEP-SS-00036||Italy|658[0n]
Leptidea sinapis|BIBSA035-14|LEP-SS-00035||Italy|658[0n]
Leptidea sinapis|EULEP5467-17|RVcoll12M471|Sicily. Genit. examined|Italy|658[0n]
Leptidea sinapis|EULEP5466-17|RVcoll12M470|Sicily. Genit. examined|Italy|658[0n]
Leptidea sinapis|WMB4183-14|RVcoll.13-S867|Genit. examined|Portugal|658[0n]
Leptidea sinapis|WMB4182-14|RVcoll.13-S848||Portugal|658[0n]
Leptidea sinapis|WMB4181-14|RVcoll.13-S846||Portugal|658[0n]
Leptidea sinapis|WMB3830-14|RVcoll.09-X889|Genit. examined|Italy|658[0n]
Leptidea sinapis|WMB3733-14|RVcoll.12-M078||France|658[0n]
Leptidea sinapis|WMB3451-14|RVcoll.10-A410|Genit. examined|France|658[0n]
Leptidea sinapis|WMB3370-14|RVcoll.09-T243|Genit. examined|France|658[0n]
Leptidea sinapis|WMB261-11|RVcoll.10-A271|Genit. examined|France|658[0n]
Leptidea sinapis|EZSPM401-09|Rvcoll.09-V802|Genit. examined|Spain|658[0n]
Leptidea sinapis|EZSPM705-12|Rvcoll. 12-L215|Genit. examined|Spain|658[0n]
Leptidea sinapis|EZSPM702-12|Rvcoll. 12-L115|Genit. examined|Spain|658[0n]
Leptidea sinapis|EZSPM328-09|RVcoll.08-R034||Spain|658[0n]
Leptidea sinapis|EZSPM1010-12|Rvcoll.12-L056||Spain|658[0n]
Leptidea sinapis|EZSPN472-09|RVcoll.08-H902||Spain|658[0n]
Leptidea sinapis|EZSPN371-09|RVcoll.08-H396||Spain|658[0n]
Leptidea sinapis|EZSPN347-09|RVcoll.08-H312|Genit. examined|Spain|658[0n]
Leptidea sinapis|GBGLP177-13|KC866056||United Kingdom|658[0n]
Leptidea sinapis|GBGLP176-13|KC866057||United Kingdom|658[0n]
Leptidea sinapis|GBGLP185-13|KC866048||Spain|658[0n]
Leptidea sinapis|GBGLP182-13|KC866051||Italy|658[0n]
Leptidea sinapis|GBGLP181-13|KC866052||Croatia|658[0n]
Leptidea sinapis|GBGLP180-13|KC866053||Macedonia|658[0n]
Leptidea sinapis|GBGLP179-13|KC866054||United Kingdom|658[0n]
Leptidea sinapis|GBGLP178-13|KC866055||United Kingdom|658[0n]
Leptidea sinapis|GBGLP187-13|KC866046||Croatia|658[0n]
Leptidea sinapis|GBGLP186-13|KC866047||Italy|658[0n]
Leptidea sinapis|GBGLP193-13|KC866040|Corsica|France|658[0n]
Leptidea sinapis|GBGLP189-13|KC866044||Spain|658[0n]
Leptidea sinapis|GBMIN34020-13|JF513042||Spain|658[0n]
Leptidea sinapis|GBGLP169-13|KC866064||United Kingdom|658[0n]
Leptidea sinapis|GBMIN34045-13|JF512706||Italy|658[0n]
Leptidea sinapis|GBMIN34044-13|JF512708||Greece|658[0n]
Leptidea sinapis|GBMIN34054-13|JF512688||Ireland|658[0n]
Leptidea sinapis|GBMIN34053-13|JF512690||Ireland|658[0n]
Leptidea sinapis|EULEP5341-17|RVcoll09V330|Genit. examined|Spain|658[0n]
Leptidea sinapis|EULEP5339-17|RVcoll09V328|Genit. examined|Spain|658[0n]
Leptidea sinapis|EULEP5465-17|RVcoll12M445|Sicily|Italy|658[0n]
Leptidea sinapis|EULEP5464-17|RVcoll12M263||Italy|658[0n]
Leptidea sinapis|EULEP5242-17|RVcoll07D940|Genit. examined|Romania|658[0n]
Leptidea sinapis|EULEP5221-17|RVcoll06K558|Genit. examined|Romania|658[0n]
Leptidea sinapis|EULEP4130-16|RVcoll15Q098||Russia|658[0n]
Leptidea sinapis|WMB4949-14|RVcoll. 14-I496|Genit. examined|Italy|658[0n]
Leptidea sinapis|WMB4868-14|RVcoll.14-I415|Genit. examined|Italy|658[0n]
Leptidea sinapis|WMB4319-14|RVcoll.14-A549||Italy|658[0n]
Leptidea sinapis|EZSPC473-09|RVcoll.06-V761|Genit. examined|Spain|658[0n]
Leptidea sinapis|EZSPN147-09|RVcoll.08-R046|Genit. examined|Spain|658[0n]
Leptidea sinapis|EZRMN021-08|RVcoll.08-M326|Genit. examined|Romania|658[0n]
Leptidea sinapis|EZROM239-08|RV-06-K554|Genit. examined|Romania|658[0n]
Leptidea sinapis|EZSPC475-09|RVcoll.08-J352||Spain|658[0n]
Leptidea sinapis|EZROM287-08|RV-07-E649|Genit. examined|Romania|658[0n]
Leptidea sinapis|EZROM242-08|RV-07-C990|Genit. examined|Romania|658[0n]
Leptidea sinapis|LEFIC521-10|MM04238||Finland|658[0n]
Leptidea sinapis|EULEP5387-17|RVcoll10B453|Genit. examined|Croatia|658[0n]
Leptidea sinapis|EULEP5386-17|RVcoll10B385|Genit. examined|Bulgaria|658[0n]
Leptidea sinapis|GWOSZ096-11|BC ZSM Lep 41852||Italy|658[0n]
Leptidea sinapis|LEFIG106-10|MM13893||Finland|658[0n]
Leptidea sinapis|EULEP5384-17|RVcoll10B359|Genit. examined|Bulgaria|658[0n]
Leptidea sinapis|EULEP5365-17|RVcoll09V806|Genit. examined|Spain|658[0n]
Leptidea sinapis|GBGLP162-13|KC866071||Italy|658[0n]
Leptidea sinapis|GBGLP161-13|KC866072||Italy|658[0n]
Leptidea sinapis|GBGLP173-13|KC866060||United Kingdom|658[0n]
Leptidea sinapis|GBGLP172-13|KC866061||United Kingdom|658[0n]
Leptidea sinapis|GBMIN34146-13|JF513029||Romania|658[0n]
Leptidea sinapis|GBMIN34145-13|JF513031||Romania|658[0n]
Leptidea sinapis|EULEP5516-17|RVcoll14A044|Genit. examined|United Kingdom|658[0n]
Leptidea sinapis|EULEP5497-17|RVcoll12Z035|Genit. examined|Ireland|658[0n]
Leptidea sinapis|EULEP5518-17|RVcoll14A055|Genit. examined|United Kingdom|658[0n]
Leptidea sinapis|EULEP5517-17|RVcoll14A054|Genit. examined|United Kingdom|658[0n]
Leptidea sinapis|EULEP5289-17|RVcoll08H903|Genit. examined|Spain|658[0n]
Leptidea sinapis|EULEP5267-17|RVcoll08H277|Genit. examined|Spain|658[0n]
Leptidea sinapis|GBGLP156-13|KC866077||Germany|658[0n]
Leptidea sinapis|GBGLP155-13|KC866078||Italy|658[0n]
Leptidea sinapis|GBMIN34163-13|JF512709||Greece|658[0n]
Leptidea sinapis|GBMIN34165-13|JF512705||Italy|658[0n]
Leptidea sinapis|GBMIN34172-13|JF512691||Ireland|658[0n]
Leptidea sinapis|GBMIN34173-13|JF512689||Ireland|658[0n]
Leptidea sinapis|GBMIN34174-13|JF512687||Ireland|658[0n]
Leptidea sinapis|GWORU076-10|BC ZSM Lep 30337||Italy|658[0n]
Leptidea sinapis|EZSPM323-09|RVcoll.08-J897||Spain|658[1n]
Leptidea sinapis|GBMIN87368-17|KU355292||Greece|655[0n]
Leptidea sinapis|EULEP1644-15|LR-08-D095|Genit. examined|Greece|614[0n]
Leptidea sinapis|EULEP5284-17|RVcoll08H744|Sardinia|Italy|658[0n]
Leptidea sinapis|EULEP5283-17|RVcoll08H741|Sardinia. Genit. examined|Italy|658[0n]
Leptidea sinapis|EULEP5282-17|RVcoll08H734|Sardinia|Italy|658[0n]
Leptidea sinapis|EULEP5281-17|RVcoll08H733|Sardinia. Genit. examined|Italy|658[0n]
Leptidea sinapis|EULEP5280-17|RVcoll08H732|Sardinia. Genit. examined|Italy|658[0n]
Leptidea sinapis|EULEP5279-17|RVcoll08H731|Sardinia. Genit. examined|Italy|658[0n]
Leptidea sinapis|EULEP5278-17|RVcoll08H721|Sardinia. Genit. examined|Italy|658[0n]

Leptidea sinapis|EULEP5280-17|RVcoll08H732|Sardinia. Genit. examined|Italy|658[0n]
Leptidea sinapis|EULEP5279-17|RVcoll08H731|Sardinia. Genit. examined|Italy|658[0n]
Leptidea sinapis|EULEP5278-17|RVcoll08H721|Sardinia. Genit. examined|Italy|658[0n]
Leptidea sinapis|EULEP5277-17|RVcoll08H720|Sardinia|Italy|658[0n]
Leptidea sinapis|EULEP5276-17|RVcoll08H717|Sardinia|Italy|658[0n]
Leptidea sinapis|EULEP5225-17|RVcoll07C467|Genit. examined|Spain|640[0n]
Leptidea sinapis|EZSPC469-09|RVcoll.08-J393|Genit. examined|Spain|652[0n]
Leptidea sinapis|OXB1585-16|OXB-TGS-1303||France|638[0n]
Leptidea sinapis|GBGLP094-13|KC866139||Ireland|642[0n]
Leptidea sinapis|GBGLP095-13|KC866138||Ireland|642[0n]
Leptidea sinapis|GBGLP096-13|KC866137||Ireland|642[0n]
Leptidea sinapis|GBGLP097-13|KC866136||Ireland|642[0n]
Leptidea sinapis|GBGLP098-13|KC866135||France|642[0n]
Leptidea sinapis|GBGLP101-13|KC866132||Spain|643[0n]
Leptidea sinapis|GBGLP104-13|KC866129||Germany|644[0n]
Leptidea sinapis|EZSPN294-09|RVcoll.08-J660||Spain|646[0n]
Leptidea sinapis|EZSPN814-09|RVcoll.08-L617|Genit. examined|Spain|634[0n]
Leptidea sinapis|GWORU372-10|BC ZSM Lep 31963||Italy|646[0n]
Leptidea sinapis|GWORR402-10|BC ZSM Lep 29618||Italy|658[0n]
Leptidea sinapis|EULEP5511-17|RVcoll13S302|Ibiza|Spain|658[0n]
Leptidea sinapis|EULEP5510-17|RVcoll13S284|Ibiza|Spain|658[0n]
Leptidea sinapis|EULEP5249-17|RVcoll07E139|Genit. examined|Italy|676[0n]
Leptidea sinapis|EULEP5270-17|RVcoll08H281|Genit. examined|Spain|676[0n]
Leptidea sinapis|GBMIN34221-13|JF512593||Italy|676[0n]
Leptidea sinapis|WMB3990-14|RVcoll.12-Q334|Genit. examined|France|658[0n]
Leptidea sinapis|WMB4515-14|RVcoll.14-E205||Spain|658[0n]
Leptidea sinapis|WMB4990-14|RVcoll. 14-I537||Italy|658[0n]
Leptidea sinapis|WMB4972-14|RVcoll. 14-I519||Italy|658[0n]
Leptidea sinapis|EULEP5905-18|RVcoll17D367||France|658[0n]
Leptidea sinapis|EULEP5904-18|RVcoll17D351||France|658[0n]
Leptidea sinapis|EULEP5285-17|RVcoll08H769|Corsica. Genit. examined|France|658[0n]
Leptidea sinapis|EULEP5271-17|RVcoll08H525|Genit. examined|Spain|658[0n]
Leptidea sinapis|EULEP5903-18|RVcoll17D350||France|658[0n]
Leptidea sinapis|EULEP5866-18|RVcoll15O480||Spain|658[0n]
Leptidea sinapis|EULEP5268-17|RVcoll08H278|Genit. examined|Spain|658[0n]
Leptidea sinapis|EULEP5252-17|RVcoll07E215|Genit. examined|Italy|658[0n]
Leptidea sinapis|EULEP5250-17|RVcoll07E142|Genit. examined|Italy|658[0n]
Leptidea sinapis|EULEP5485-17|RVcoll12Q385|Genit. examined|France|658[0n]
Leptidea sinapis|EULEP5287-17|RVcoll08H779|Corsica. Genit. examined|France|658[0n]
Leptidea sinapis|EULEP5286-17|RVcoll08H778|Corsica. Genit. examined|France|658[0n]
Leptidea sinapis|EULEP5815-18|RVcoll12L208||Spain|658[0n]
Leptidea sinapis|EULEP5340-17|RVcoll09V329|Genit. examined|Spain|658[0n]
Leptidea sinapis|EULEP5303-17|RVcoll08R538|Corsica|France|658[0n]
Leptidea sinapis|EULEP5288-17|RVcoll08H786|Corsica. Genit. examined|France|658[0n]
Leptidea sinapis|EULEP5851-18|RVcoll14V698||Spain|658[0n]
Leptidea sinapis|EULEP5655-17|RVcoll15J990||Italy|658[0n]
Leptidea sinapis|EULEP5854-18|RVcoll14V704||Spain|658[0n]
Leptidea sinapis|EULEP5853-18|RVcoll14V702||Spain|658[0n]
Leptidea sinapis|EULEP5330-17|RVcoll09V196|Corsica. Genit. examined|France|658[0n]
Leptidea sinapis|EULEP5329-17|RVcoll09T520|Corsica. Genit. examined|France|658[0n]
Leptidea sinapis|OXB1109-15|15-A642||Italy|658[0n]
Leptidea sinapis|OXB1005-15|15-A955||Italy|658[0n]
Leptidea sinapis|GBGLP222-13|KC866011||France|658[0n]
Leptidea sinapis|GBGLP229-13|KC866004|Corsica|France|658[0n]
Leptidea sinapis|GBGLP230-13|KC866003|Corsica|France|658[0n]
Leptidea sinapis|GBMIN34156-13|JF513009||Italy|658[0n]
Leptidea sinapis|GBMIN34186-13|JF512663||Spain|658[0n]
Leptidea sinapis|EULEP5492-17|RVcoll12R805||Russia|655[0n]
Leptidea sinapis|EULEP5493-17|RVcoll12R807||Russia|655[0n]
Leptidea sinapis|EULEP5359-17|RVcoll09V669|Genit. examined|Romania|658[1n]
Leptidea sinapis|OXB1135-15|15-A668||Italy|632[0n]
Leptidea sinapis|WMB3800-14|RVcoll.12-O633|Genit. examined|France|646[0n]
Leptidea sinapis|WMB3867-14|RVcoll.11-J582||Italy|658[0n]
Leptidea sinapis|WMB3450-14|RVcoll.10-A400|Genit. examined|France|658[0n]
Leptidea sinapis|WMB3448-14|RVcoll.10-A248|Genit. examined|France|658[0n]
Leptidea sinapis|EZSPM815-12|Rvcoll. 130711PX77||Spain|658[0n]
Leptidea sinapis|EZSPM814-12|Rvcoll. 130711PX23||Spain|658[0n]
Leptidea sinapis|EZSPM140-09|RVcoll.08-P652|Genit. examined|Spain|658[0n]
Leptidea sinapis|EZSPM704-12|Rvcoll. 12-L123|Genit. examined|Spain|658[0n]
Leptidea sinapis|EZSPM700-12|Rvcoll. 12-L108|Genit. examined|Spain|658[0n]
Leptidea sinapis|EZSPN412-09|RVcoll.08-H527|Genit. examined|Spain|658[0n]
Leptidea sinapis|EZSPN146-09|RVcoll.08-R045||Spain|658[0n]
Leptidea sinapis|GBGLP129-13|KC866104||Macedonia|658[0n]
Leptidea sinapis|GBLAB314-13|BC ZSM Lep 72615||Germany|658[0n]
Leptidea sinapis|EULEP5542-17|RVcoll14B991||Sweden|658[0n]
Leptidea sinapis|ABOLD632-17|TLMF Lep 21708||Austria|658[0n]
Leptidea sinapis|LEASS868-17|KLM Lep 08373||Austria|658[0n]
Leptidea sinapis|EULEP4998-16|RVcoll16I778||Poland|658[0n]
Leptidea sinapis|GWORO781-09|BC ZSM Lep 30473||Germany|636[0n]
Leptidea sinapis|GBGLP131-13|KC866102||Sweden|658[0n]
Leptidea sinapis|GWORA2450-09|BC ZSM Lep 30662||Austria|658[0n]
Leptidea sinapis|GBGLP135-13|KC866098||Macedonia|658[0n]
Leptidea sinapis|GBGLP134-13|KC866099||Hungary|658[0n]
Leptidea sinapis|LEFIJ4742-16|ZMBSU-02154||Belarus|658[1n]
Leptidea sinapis|GBGLP132-13|KC866101||Czech Republic|658[0n]
Leptidea sinapis|EULEP5231-17|RVcoll07D086|Genit. examined|Romania|676[0n]
Leptidea sinapis|EULEP5222-17|RVcoll06K560|Genit. examined|Romania|676[0n]
Leptidea sinapis|EULEP5264-17|RVcoll07F511|Genit. examined|Romania|676[0n]
Leptidea sinapis|EULEP5263-17|RVcoll07E366|Genit. examined|Romania|676[0n]
Leptidea sinapis|EULEP5220-17|RVcoll06K557|Genit. examined|Romania|676[0n]
Leptidea sinapis|GBMIN34107-13|JF512582||Romania|676[0n]
Leptidea sinapis|GBMIN34108-13|JF512580||Romania|676[0n]
Leptidea sinapis|GBMIN34227-13|JF512581||Romania|676[0n]
Leptidea sinapis|EULEP5388-17|RVcoll10B457|Genit. examined|Croatia|658[0n]
Leptidea sinapis|EULEP5382-17|RVcoll10B303|Genit. examined|Bulgaria|658[0n]
Leptidea sinapis|EULEP5548-17|RVcoll14E325|Genit. examined|Sweden|658[0n]
Leptidea sinapis|EULEP5477-17|RVcoll12P001|Genit. examined|France|658[0n]
Leptidea sinapis|GBGLP201-13|KC866032||Czech Republic|658[0n]
Leptidea sinapis|GBGLP200-13|KC866033||Czech Republic|658[0n]
Leptidea sinapis|GBLAC425-13|BC ZSM Lep 77666||Germany|658[0n]
Leptidea sinapis|EULEP359-14|MM23844||Lithuania|658[0n]
Leptidea sinapis|EULEP5233-17|RVcoll07D151|Genit. examined|Romania|658[0n]
[illegible]

Leptidea sinapis|GBLAC425-13|BC ZSM Lep 77666||Germany|658[0n]
Leptidea sinapis|EULEP359-14|MM23844||Lithuania|658[0n]
Leptidea sinapis|EULEP5233-17|RVcoll07D151|Genit. examined|Romania|658[0n]
Leptidea sinapis|EULEP5042-16|RVcoll16J037||Slovakia|658[0n]
Leptidea sinapis|EULEP4759-16|RVcoll16H010||Sweden|658[0n]
Leptidea sinapis|EULEP4129-16|RVcoll15P017||Belarus|658[0n]
Leptidea sinapis|WMB3590-14|RVcoll.11-E239|Genit. examined|France|658[0n]
Leptidea sinapis|EZRMN019-08|RVcoll.08-M315|Genit. examined|Romania|658[0n]
Leptidea sinapis|EZROM241-08|RV-07-D081|Genit. examined|Romania|658[0n]
Leptidea sinapis|EZROM240-08|RV-07-D080|Genit. examined|Romania|658[0n]
Leptidea sinapis|EZROM238-08|RV-06-K556|Genit. examined|Romania|658[0n]
Leptidea sinapis|EZRMN022-08|RVcoll.08-M329|Genit. examined|Romania|658[0n]
Leptidea sinapis|EZRMN020-08|RVcoll.08-M316|Genit. examined|Romania|658[0n]
Leptidea sinapis|EZRMN010-08|RVcoll.08-M528||Romania|658[0n]
Leptidea sinapis|EZROM584-08|RV-07-C307|Genit. examined|Romania|658[0n]
Leptidea sinapis|EZROM1079-09|RVcoll.08-M555|Genit. examined|Romania|658[0n]
Leptidea sinapis|LEFID238-10|MM06100||Finland|658[0n]
Leptidea sinapis|LEFIL475-10|MM18773||Finland|658[0n]
Leptidea sinapis|GWOTD801-12|BC ZSM Lep 64692||Germany|658[0n]
Leptidea sinapis|ODOPE740-11|BC ZSM Lep 50381|ID verfied by dissection|Germany|658[0n]
Leptidea sinapis|WMB5386-14|RVcoll.14-J848||France|658[0n]
Leptidea sinapis|WMB3591-14|RVcoll.11-E260|Genit. examined|France|658[0n]
Leptidea sinapis|LEFID110-10|MM05733||Finland|658[0n]
Leptidea sinapis|ABOLC190-16|TLMF Lep 20223||Austria|658[0n]
Leptidea sinapis|LEASS478-17|TLMF Lep 22126||Austria|658[0n]
Leptidea sinapis|LEASS462-17|TLMF Lep 21920||Austria|658[0n]
Leptidea sinapis|EULEP5423-17|RVcoll11E261|Genit. examined|France|658[0n]
Leptidea sinapis|EULEP5404-17|RVcoll11D553|Genit. examined|Russia|658[0n]
Leptidea sinapis|ABOLD409-16|TLMF Lep 21561||Austria|658[0n]
Leptidea sinapis|LEATG068-14|TLMF Lep 13855||Austria|658[0n]
Leptidea sinapis|PHLAJ646-15|KLM Lep 03781||Austria|658[0n]
Leptidea sinapis|ABOLD413-16|TLMF Lep 21565||Austria|658[0n]
Leptidea sinapis|ABOLA904-15|TLMF Lep 16864||Austria|658[0n]
Leptidea sinapis|ABOLD410-16|TLMF Lep 21562||Austria|658[0n]
Leptidea sinapis|PHLAJ645-15|KLM Lep 03780||Austria|658[0n]
Leptidea sinapis|PHLAJ648-15|KLM Lep 03783||Austria|658[0n]
Leptidea sinapis|ABOLD411-16|TLMF Lep 21563||Austria|658[0n]
Leptidea sinapis|ABOLD095-16|TLMF Lep 21173||Austria|658[0n]
Leptidea sinapis|LEASS870-17|KLM Lep 08375||Austria|658[0n]
Leptidea sinapis|GBLAA1376-15|BC ZSM Lep 87072||Germany|658[0n]
Leptidea sinapis|EULEP5397-17|RVcoll10C248|Genit. examined|Czech Republic|658[0n]
Leptidea sinapis|EULEP5396-17|RVcoll10C245|Genit. examined|Czech Republic|658[0n]
Leptidea sinapis|LEASS774-17|TLMF Lep 22422||Austria|658[0n]
Leptidea sinapis|LEASS773-17|TLMF Lep 22421||Austria|658[0n]
Leptidea sinapis|GBGLP203-13|KC866030||Czech Republic|658[0n]
Leptidea sinapis|GBGLP202-13|KC866031||Hungary|658[0n]
Leptidea sinapis|GBLAB765-13|BC ZSM Lep 75536||Germany|658[0n]
Leptidea sinapis|GBLAB764-13|BC ZSM Lep 75535||Germany|658[0n]
Leptidea sinapis|OXB1419-15|Rvcoll_15-M125||France|658[0n]
Leptidea sinapis|LEASS869-17|KLM Lep 08374||Austria|658[0n]
Leptidea sinapis|OXB1583-16|OXB-TGS-1301||France|658[0n]
Leptidea sinapis|OXB1579-16|OXB-TGS-1297||France|658[0n]
Leptidea sinapis|OXB764-15|13-U509||Italy|658[0n]
Leptidea sinapis|OXB763-15|13-U508|Genit. examined|Italy|658[0n]
Leptidea sinapis|GBGLP197-13|KC866036||Sweden|658[0n]
Leptidea sinapis|GBGLP196-13|KC866037||Sweden|658[0n]
Leptidea sinapis|GBGLP195-13|KC866038||Sweden|658[0n]
Leptidea sinapis|GBGLP194-13|KC866039||Sweden|658[0n]
Leptidea sinapis|GBMIN34162-13|JF512711||Slovenia|658[0n]
Leptidea sinapis|LEFIJ5635-17|MM25693||Finland|658[0n]
Leptidea sinapis|ABOLD650-17|TLMF Lep 21726||Austria|658[0n]
Leptidea sinapis|ABOLD641-17|TLMF Lep 21717||Austria|658[0n]
Leptidea sinapis|ABOLD636-17|TLMF Lep 21712||Austria|658[0n]
Leptidea sinapis|OXB1414-15|Rvcoll_15-M791||France|658[0n]
Leptidea sinapis|OXB1223-15|Rvcoll_15-M652||France|658[0n]
Leptidea sinapis|OXB1207-15|Rvcoll_15-M631||France|658[0n]
Leptidea sinapis|OXB1347-15|Rvcoll_15-M696||France|658[0n]
Leptidea sinapis|OXB1339-15|Rvcoll_15-M686||France|658[0n]
Leptidea sinapis|OXB1304-15|Rvcoll_15-M174||France|658[0n]
Leptidea sinapis|OXB1610-16|OXB-TGS-1328||France|658[0n]
Leptidea sinapis|OXB1605-16|OXB-TGS-1323||France|658[0n]
Leptidea sinapis|OXB1584-16|OXB-TGS-1302||France|658[0n]
Leptidea sinapis|PHLAI523-13|TLMF Lep 09085||Austria|658[0n]
Leptidea sinapis|ABOLD019-16|TLMF Lep 21097||Austria|658[0n]
Leptidea sinapis|LEASS873-17|KLM Lep 08378||Austria|658[0n]
Leptidea sinapis|LEASS872-17|KLM Lep 08377||Austria|658[0n]
Leptidea sinapis|PHLSA683-11|TLMF Lep 06138||Austria|658[0n]
Leptidea sinapis|PHLAF324-11|TLMF Lep 05494||Macedonia|658[0n]
Leptidea sinapis|GBGLP199-13|KC866034||Czech Republic|658[0n]
Leptidea sinapis|GBGLP198-13|KC866035||Czech Republic|658[0n]
Leptidea sinapis|EULEP5437-17|RVcoll11H240|Genit. examined|Germany|658[0n]
Leptidea sinapis|EULEP5436-17|RVcoll11H237|Genit. examined|Germany|658[0n]
Leptidea sinapis|WMB5288-14|RVcoll.14-H965||France|658[0n]
Leptidea sinapis|WMB5015-14|RVcoll. 14-I562||Italy|658[0n]
Leptidea sinapis|GBGLP205-13|KC866028||Macedonia|658[0n]
Leptidea sinapis|GBGLP204-13|KC866029||Macedonia|658[0n]
Leptidea sinapis|GBGLP207-13|KC866026||Croatia|658[0n]
Leptidea sinapis|GBGLP206-13|KC866027||Macedonia|658[0n]
Leptidea sinapis|EULEP337-14|MM23822||Estonia|658[0n]
Leptidea sinapis|EULEP5739-17|RVcollLR08D677|Genit. examined|Slovenia|658[0n]
Leptidea sinapis|GBGLP209-13|KC866024||Croatia|658[0n]
Leptidea sinapis|GBGLP208-13|KC866025||Croatia|658[0n]
Leptidea sinapis|GBGLP221-13|KC866012||Romania|658[0n]
Leptidea sinapis|GBGLP219-13|KC866014||Sweden|658[0n]
Leptidea sinapis|GBGLP211-13|KC866022||Luxembourg|658[0n]
Leptidea sinapis|GBGLP210-13|KC866023||Belgium|658[0n]
Leptidea sinapis|GBGLP217-13|KC866016||Bulgaria|658[0n]
Leptidea sinapis|GBGLP212-13|KC866021||Belgium|658[0n]
Leptidea sinapis|GBMIN34043-13|JF512710||Slovenia|658[0n]
Leptidea sinapis|GBMIN34050-13|JF512696||Ukraine|658[0n]
Leptidea sinapis|GBMIN34052-13|JF512692||Estonia|658[0n]
Leptidea sinapis|GBGLP214-13|KC866019||Germany|658[0n]
Leptidea sinapis|GBMIN34141-13|JF513039||Romania|658[0n]

Leptidea sinapis|GBMIN34052-13|JF512692||Estonia|658[0n]
Leptidea sinapis|GBGLP214-13|KC866019||Germany|658[0n]
Leptidea sinapis|GBMIN34141-13|JF513039||Romania|658[0n]
Leptidea sinapis|GBMIN34102-13|JF512592||Romania|676[0n]
Leptidea sinapis|GBMIN87371-17|KU355291||Serbia|655[0n]
Leptidea sinapis|GBMIN87372-17|KU355289||Bosnia and Herzegovina|655[0n]
Leptidea sinapis|GBMIN87373-17|KU355290||Serbia|655[0n]
Leptidea sinapis|EZRMN018-08|RVcoll.08-M201|Genit. examined|Romania|658[1n]
Leptidea sinapis|LEFIB130-10|MM00498||Finland|638[0n]
Leptidea sinapis|GWORA2880-15|BC ZSM Lep 86148||Germany|638[0n]
Leptidea sinapis|OXB1611-16|OXB-TGS-1329||France|638[0n]
Leptidea sinapis|GBMIN34157-13|JF512721||Bulgaria|621[0n]
Leptidea sinapis|ABOLD412-16|TLMF Lep 21564||Austria|622[0n]
Leptidea sinapis|GBGLP099-13|KC866134||Germany|642[0n]
Leptidea sinapis|GBGLP100-13|KC866133||Czech Republic|642[0n]
Leptidea sinapis|EULEP5240-17|RVcoll07D938|Genit. examined|Romania|676[0n]
Leptidea sinapis|GBGLP105-13|KC866128||Finland|625[0n]
Leptidea sinapis|LEATG005-14|TLMF Lep 13792||Austria|605[1n]
Leptidea sinapis|PHLAJ647-15|KLM Lep 03782||Austria|658[0n]
Aporia crataegi|BIBSA1760-16|LD-3058||Italy|658[0n]
Aporia crataegi|WMB620-11|RVcoll.11-I056|Sicily|Italy|658[0n]
Aporia crataegi|WMB2951-14|RVcoll.12-M597|Sicily|Italy|658[0n]
Aporia crataegi|EULEP2615-15|RVcoll.14-N555|Cyprus|Cyprus|645[0n]
Aporia crataegi|WMB566-11|RVcoll.11-H779|Sicily|Italy|658[0n]
Aporia crataegi|WMB500-11|RVcoll.11-H556|Sicily|Italy|658[0n]
Aporia crataegi|WMB219-11|RVcoll.08-R518|Sicily|Italy|658[0n]
Aporia crataegi|WMB902-13|RVcoll.09-X860||Italy|658[0n]
Aporia crataegi|EZSPN836-09|RVcoll.08-L660||Spain|658[0n]
Aporia crataegi|EULEP1817-15|RVcoll.14-H819||Greece|658[0n]
Aporia crataegi|EULEP1758-15|RVcoll.14-H420||Greece|658[0n]
Aporia crataegi|EULEP1449-15|RVcoll.14-G167||Greece|658[0n]
Aporia crataegi|EULEP2616-15|RVcoll.14-N556|Samos|Greece|658[0n]
Aporia crataegi|WMB4874-14|RVcoll.14-I421||Italy|658[0n]
Aporia crataegi|WMB4173-14|RVcoll.13-S757||Italy|658[0n]
Aporia crataegi|EZROM706-08|RV-06-H886||Spain|658[0n]
Aporia crataegi|EZSPN681-09|RVcoll.08-L003||Spain|658[0n]
Aporia crataegi|EZROM017-08|RV-06-M826||Romania|658[1n]
Aporia crataegi|GWORA2447-09|BC ZSM Lep 30659||Germany|658[0n]
Aporia crataegi|EULEP596-15|RVcoll.10-C327||Switzerland|658[0n]
Aporia crataegi|EULEP3439-16|RVcoll15G943||Switzerland|658[0n]
Aporia crataegi|EULEP3441-16|RVcoll15I113||Italy|658[0n]
Aporia crataegi|EZSPN500-09|RVcoll.08-H949||Spain|658[0n]
Aporia crataegi|EULEP1914-15|RVcoll.14-B815||Bosnia and Herzegovina|658[0n]
Aporia crataegi|BIBSA1714-16|15-N185||Italy|617[0n]
Aporia crataegi|BIBSA1165-15|15-M184||Italy|612[0n]
Aporia crataegi|BIBSA1828-17|RVcoll16C703||Italy|658[0n]
Aporia crataegi|BIBSA1874-17|RVcoll16C773||Italy|658[0n]
Aporia crataegi|BIBSA1380-15|15-M963||Italy|658[0n]
Aporia crataegi|BIBSA1150-15|15-L079||Italy|658[0n]
Aporia crataegi|WMB4176-14|RVcoll.13-S770||Italy|658[0n]
Aporia crataegi|EULEP1630-15|RVcoll.14-G732||Greece|658[0n]
Aporia crataegi|EULEP1658-15|RVcoll.14-G868||Greece|658[0n]
Aporia crataegi|BIBSA1011-15|15-C121||Italy|658[0n]
Aporia crataegi|BIBSA1521-16|LEP-SS-00366||Italy|658[0n]
Aporia crataegi|BIBSA1735-16|LD-2794||Italy|658[0n]
Aporia crataegi|BIBSA040-14|LEP-SS-00040||Italy|658[0n]
Aporia crataegi|BIBSA1800-16|15-C369||Italy|658[0n]
Aporia crataegi|WMB4885-14|RVcoll.14-I432||Italy|658[0n]
Aporia crataegi|WMB3859-14|RVcoll.11-I248||Italy|658[0n]
Aporia crataegi|WMB4156-14|RVcoll.13-S713||Italy|658[0n]
Aporia crataegi|WMB2370-13|RVcoll.12-R440||Italy|658[0n]
Aporia crataegi|WMB894-13|RVcoll.09-X832||Italy|658[0n]
Aporia crataegi|WMB2754-13|RVcoll.10-C667||Italy|658[0n]
Aporia crataegi|GWORK512-09|BC ZSM Lep 21842||Germany|658[0n]
Aporia crataegi|GWORU084-10|BC ZSM Lep 30345||Italy|658[0n]
Aporia crataegi|GWORO779-09|BC ZSM Lep 30471||Germany|658[0n]
Aporia crataegi|EZSPN461-09|RVcoll.08-H670||Spain|658[0n]
Aporia crataegi|EZROM770-08|RVcoll.08-M385||Romania|658[0n]
Aporia crataegi|LEFIA024-10|MM00095||Finland|658[0n]
Aporia crataegi|EULEP4817-16|RVcoll16H712||Belgium|653[0n]
Aporia crataegi|EULEP3440-16|RVcoll15H076||Italy|658[0n]
Aporia crataegi|EULEP4723-16|RVcoll16G381||Denmark|658[0n]
Aporia crataegi|ABOLD067-16|TLMF Lep 21145||Austria|658[0n]
Aporia crataegi|LEATG531-14|TLMF Lep 14318||Italy|658[0n]
Aporia crataegi|EZROM767-08|RVcoll.06-K660||Romania|658[0n]
Aporia crataegi|EZROM768-08|RVcoll.07-D450||Romania|658[0n]
Aporia crataegi|EZROM769-08|RVcoll.08-M206||Romania|658[0n]
Aporia crataegi|EZROM018-08|RV-07-D461||Romania|658[0n]
Aporia crataegi|EZSPN392-09|RVcoll.08-H448|ex. pupa|Spain|658[0n]
Aporia crataegi|EZSPM254-09|RVcoll.08-R466||Spain|658[0n]
Aporia crataegi|EZSPC1005-10|RVcoll.08-H342||Spain|658[0n]
Aporia crataegi|WMB274-11|RVcoll.10-A506||France|658[0n]
Aporia crataegi|EZSPC094-09|RVcoll.07-C400||Spain|658[0n]
Aporia crataegi|EZSPC211-09|RVcoll.08-H294||Spain|658[0n]
Aporia crataegi|EZSPC215-09|RVcoll.08-J386||Spain|658[0n]
Aporia crataegi|WMB6002-17|RVcoll12L486||Spain|658[0n]
Aporia crataegi|WMB6003-17|RVcoll12L487||Spain|658[0n]
Aporia crataegi|WMB3346-14|RVcoll.08-R056||Spain|658[0n]
Aporia crataegi|WMB3367-14|RVcoll.09-T208||Spain|658[0n]
Aporia crataegi|WMB3165-14|RVcoll.08-H460||Spain|658[0n]
Aporia crataegi|WMB3253-14|RVcoll.08-L409||Spain|658[0n]
Aporia crataegi|WMB3277-14|RVcoll.08-M049||Spain|658[0n]
Aporia crataegi|WMB873-13|RVcoll.09-X233||France|658[0n]
Aporia crataegi|WMB929-13|RVcoll.10-A479||France|658[0n]
Aporia crataegi|EULEP3438-16|RVcoll10A800||Estonia|658[0n]
Aporia crataegi|EULEP691-15|RVcoll.12-M007||Sweden|658[0n]
Aporia crataegi|EULEP1053-15|RVcoll.14-E927||Serbia|658[0n]
Aporia crataegi|EULEP962-15|RVcoll.14-D024||Bulgaria|658[0n]
Aporia crataegi|EULEP2124-15|RVcoll.14-J906||France|658[0n]
Aporia crataegi|BIBSA356-15|RVcoll.14-I024||Italy|658[0n]
Aporia crataegi|BIBSA357-15|RVcoll.14-I025||Italy|658[0n]
Aporia crataegi|GBLAF496-14|BC ZSM Lep 82202||Germany|658[0n]
Aporia crataegi|GBLAF770-14|BC ZSM Lep 82856||Germany|658[0n]

Aporia crataegi|BIBSA356-15|RVcoll.14-I024||Italy|658[0n]
Aporia crataegi|BIBSA357-15|RVcoll.14-I025||Italy|658[0n]
Aporia crataegi|GBLAF496-14|BC ZSM Lep 82202||Germany|658[0n]
Aporia crataegi|GBLAF770-14|BC ZSM Lep 82856||Germany|658[0n]
Aporia crataegi|LEFIJ4744-16|ZMBSU-02155||Belarus|658[0n]
Aporia crataegi|EULEP341-14|MM23826||Lithuania|658[0n]
Aporia crataegi|LEFIJ506-10|MM17131||Finland|658[0n]
Aporia crataegi|LEFIJ505-10|MM17130||Finland|658[0n]
Aporia crataegi|OXB742-15|13-U305||Italy|658[0n]
Aporia crataegi|OXB333-15|RVcoll.14-N047||Italy|658[0n]
Aporia crataegi|BIBSA041-14|LEP-SS-00050||Italy|630[0n]
Aporia crataegi|EZSPC224-09|RVcoll.08-L482||Spain|654[0n]
Aporia crataegi|LEATJ1179-16|TLMF Lep 19502||Czech Republic|638[0n]
Aporia crataegi|EULEP4834-16|RVcoll16H737||Ukraine|601[0n]
Aporia crataegi|EULEP1175-15|RVcoll.14-F402||Bulgaria|647[0n]
Aporia crataegi|EULEP3442-16|RVcoll15G119||France|644[0n]
Aporia crataegi|EZSPN564-09|RVcoll.08-J105||Portugal|629[0n]
Aporia crataegi|WMB5227-14|RVcoll.13-T986||Italy|628[0n]
Aporia crataegi|PHLAI536-13|TLMF Lep 09098||Austria|658[0n]
Aporia crataegi|LEATJ1316-16|TLMF Lep 19639||Austria|658[0n]
Aporia crataegi|BIBSA975-15|14-V304||Italy|658[0n]
Aporia crataegi|EULEP2149-15|RVcoll.14-K037||Switzerland|658[0n]
Aporia crataegi|EZSPN618-09|RVcoll.08-J892||Spain|658[0n]
Aporia crataegi|PHLAI534-13|TLMF Lep 09096||Austria|658[0n]
Aporia crataegi|PHLAI535-13|TLMF Lep 09097||Austria|658[0n]
Aporia crataegi|LEATD286-13|TLMF Lep 12933||Italy|658[0n]
Pontia daplidice|WMB357-11|RVcoll.11-E863|Corsica|France|658[0n]
Pontia daplidice|WMB4707-14|RVcoll.LD-2913|Asinara|Italy|658[0n]
Pontia daplidice|WMB528-11|RVcoll.11-H642|Lampedusa|Italy|658[0n]
Pontia daplidice|WMB1582-13|RVcoll.12-O547|Sardinia|Italy|658[0n]
Pontia daplidice|WMB4391-14|RVcoll.14-B308||Portugal|658[0n]
Pontia daplidice|WMB3897-14|RVcoll.12-P127||France|658[0n]
Pontia daplidice|WMB2900-14|RVcoll.11-E659|Sardinia|Italy|658[0n]
Pontia daplidice|EZSPN1056-11|Rvcoll.08-P079||Spain|658[0n]
Pontia daplidice|EZSPN520-09|RVcoll.08-H985||Spain|658[0n]
Pontia daplidice|EZSPM603-12|RVcoll.11-D599||Spain|658[0n]
Pontia daplidice|WMB3456-14|RVcoll.10-A557||France|658[0n]
Pontia daplidice|EZSPN308-09|RVcoll.08-H114||Spain|643[0n]
Pontia daplidice|EZSPN135-09|RVcoll.06-G487||Spain|658[0n]
Pontia daplidice|WMB3241-14|RVcoll.08-L132||Spain|658[0n]
Pontia daplidice|WMB2322-13|RVcoll.11-H637|Lampedusa|Italy|658[0n]
Pontia daplidice|EZSPM373-09|RVcoll.08-J860||Spain|658[1n]
Pontia daplidice|EZSPM913-12|Rvcoll.12-M635||Spain|658[0n]
Pontia daplidice|EZSPM890-12|Rvcoll.12-M608||Spain|658[0n]
Pontia daplidice|WMB2636-13|RVcoll.11-E876|Corsica|France|658[0n]
Pontia daplidice|WMB3179-14|RVcoll.08-H595||Spain|658[0n]
Pontia daplidice|EZSPN1052-11|Rvcoll.08-L476||Spain|658[0n]
Pontia daplidice|EZSPM978-12|Rvcoll.12-M738||Spain|658[0n]
Pontia daplidice|EZSPN660-09|RVcoll.08-J959||Spain|658[0n]
Pontia daplidice|EZSPN1057-11|Rvcoll.08-P337||Spain|658[0n]
Pontia daplidice|WMB1521-13|RVcoll.12-O162|Corsica|France|658[0n]
Pontia daplidice|WMB3683-14|RVcoll.11-J224||Spain|658[0n]
Pontia daplidice|WMB3925-14|RVcoll.12-P510||France|658[0n]
Pontia daplidice|WMB295-11|RVcoll.11-E308||Italy|658[0n]
Pontia daplidice|EZSPM033-09|RVcoll.08-P234||Spain|646[0n]
Pontia daplidice|EZSPN217-09|RVcoll.07-F078||Spain|658[0n]
Pontia daplidice|WMB3161-14|RVcoll.08-H346||Spain|658[0n]
Pontia daplidice|OXB1189-15|Rvcoll_16-A076||Italy|658[0n]
Pontia daplidice|WMB1777-13|RVcoll.12-Q096||France|658[0n]
Pontia daplidice|WMB2958-14|RVcoll.12-N011|Ibiza|Spain|658[0n]
Pontia daplidice|BIBSA269-15|RVcoll.14-E068||Italy|630[0n]
Pontia daplidice|EZSPN396-09|RVcoll.08-H456||Spain|658[0n]
Pontia daplidice|EZSPN1065-11|Rvcoll.08-R105||Spain|658[0n]
Pontia daplidice|EZSPM995-12|Rvcoll.12-L881||Spain|658[0n]
Pontia daplidice|EZSPM757-12|Rvcoll. 12-L541||Spain|658[0n]
Pontia daplidice|EZSPM889-12|Rvcoll.12-M607||Spain|658[0n]
Pontia daplidice|EZSPM934-12|Rvcoll.12-M668||Spain|658[0n]
Pontia daplidice|WMB267-11|RVcoll.10-A438||France|658[0n]
Pontia daplidice|WMB3024-14|RVcoll.13-S371|Ibiza|Spain|658[0n]
Pontia daplidice|WMB3693-14|RVcoll.11-J250||Spain|658[0n]
Pontia daplidice|WMB2821-13|RVcoll.12-O277|La Maddalena|Italy|658[0n]
Pontia daplidice|WMB1514-13|RVcoll.12-O121|Corsica|France|658[0n]
Pontia daplidice|WMB1537-13|RVcoll.12-O278|La Maddalena|Italy|658[0n]
Pontia daplidice|WMB1668-13|RVcoll.12-P235||France|658[0n]
Pontia daplidice|WMB2321-13|RVcoll.11-H636|Lampedusa|Italy|658[0n]
Pontia daplidice|WMB3913-14|RVcoll.12-P374||France|658[0n]
Pontia daplidice|WMB333-11|RVcoll.11-E670||Italy|658[0n]
Pontia daplidice|WMB1914-13|RVcoll.11-H638|Lampedusa|Italy|654[0n]
Pontia daplidice|WMB3123-14|RVcoll.06-G435||Spain|646[0n]
Pontia daplidice|EZSPM050-09|RVcoll.08-P268||Spain|646[0n]
Pontia daplidice|WMB3914-14|RVcoll.12-P377||France|620[0n]
Pontia daplidice|EZSPN1050-11|Rvcoll.08-L442||Spain|623[0n]
Pontia daplidice|EZSPM176-09|RVcoll.08-R041||Spain|646[0n]
Pontia daplidice|EZSPM115-09|RVcoll.08-P474||Spain|645[0n]
Pontia daplidice|EZSPN327-09|RVcoll.08-H214||Spain|645[0n]
Pontia daplidice|EZSPN324-09|RVcoll.08-H188||Spain|645[0n]
Pontia daplidice|EZSPN095-09|RVcoll.06-A030||Spain|658[0n]
Pontia daplidice|EZSPN778-09|RVcoll.08-L288||Spain|658[0n]
Pontia daplidice|GWOSU1021-11|BC Back 0071||Spain|658[0n]
Pontia edusa|PHLAH473-12|TLMF Lep 08292||Austria|615[0n]
Pontia edusa|BIBSA1395-15|15-M999||Italy|658[0n]
Pontia edusa|WMB663-11|RVcoll.11-I358|Argentario|Italy|658[0n]
Pontia edusa|WMB5066-14|RVcoll. 14-L197||Italy|658[0n]
Pontia edusa|BIBSA030-14|LEP-SS-00030||Italy|658[0n]
Pontia edusa|BIBSA1774-16|12-Q939||Italy|658[0n]
Pontia edusa|BIBSA1718-16|15-N216||Italy|658[0n]
Pontia edusa|WMB3036-14|RVcoll.13-S561|San Domino|Italy|658[0n]
Pontia edusa|GWORU078-10|BC ZSM Lep 30339||Italy|658[0n]
Pontia edusa|WMB2757-13|RVcoll.11-H689|Levanzo|Italy|658[0n]
Pontia edusa|WMB3081-14|RVcoll.14-D948|Giglio|Italy|658[0n]
Pontia edusa|WMB2351-13|RVcoll.12-Q884||Italy|658[0n]
Pontia edusa|WMB1918-13|RVcoll.11-H688|Levanzo|Italy|658[0n]
Pontia edusa|WMB4675-14|RVcoll.LD-2634|Ischia|Italy|658[0n]

Pontia edusa|WMB2351-13|RVcoll.12-Q884||Italy|658[0n]
Pontia edusa|WMB1918-13|RVcoll.11-H688|Levanzo|Italy|658[0n]
Pontia edusa|WMB4675-14|RVcoll.LD-2634|Ischia|Italy|658[0n]
Pontia edusa|WMB4614-14|RVcoll.LD-1879||Italy|658[0n]
Pontia edusa|WMB5085-14|RVcoll. 14-L216||Italy|658[0n]
Pontia edusa|WMB4677-14|RVcoll.LD-2643||Italy|658[0n]
Pontia edusa|WMB5212-14|RVcoll.13-T952||Italy|658[0n]
Pontia edusa|WMB5489-14|RVcoll.LD-2644||Italy|658[0n]
Pontia edusa|EULEP5897-18|RVcoll17A023|Malta|Malta|657[0n]
Pontia edusa|EULEP5899-18|RVcoll17A025|Malta|Malta|656[0n]
Pontia edusa|BIBSA1788-16|16-A524||Italy|658[0n]
Pontia edusa|BIBSA1367-15|15-M909||Italy|658[0n]
Pontia edusa|WMB4144-14|RVcoll.13-S661||Italy|658[0n]
Pontia edusa|BIBSA644-15|RVcoll. 15-A807||Italy|658[0n]
Pontia edusa|OXB1132-15|15-A665||Italy|658[0n]
Pontia edusa|BIBSA029-14|LEP-SS-00029||Italy|632[0n]
Pontia edusa|WMB1932-13|RVcoll.11-H810|Vulcano|Italy|658[0n]
Pontia edusa|PHLAI568-13|TLMF Lep 09130||Italy|658[0n]
Pontia edusa|WMB181-11|RVcoll.11-D461|Sicily|Italy|658[0n]
Pontia edusa|EULEP4944-16|RVcoll16I145||Poland|633[0n]
Pontia edusa|EZROM514-08|RV-07-C942||Romania|658[0n]
Pontia edusa|EULEP2571-15|RVcoll.14-V548||Ukraine|658[0n]
Pontia edusa|LEFIJ507-10|MM17132||Finland|642[0n]
Pontia edusa|LEASS957-17|TLMF Lep 22510||Austria|658[0n]
Pontia edusa|EULEP1706-15|RVcoll.14-H142||Greece|614[0n]
Pontia edusa|EULEP1579-15|RVcoll.14-G604||Greece|636[0n]
Pontia edusa|EULEP1212-15|RVcoll.14-F508||Greece|658[0n]
Pontia edusa|EULEP706-15|RVcoll.12-M182|Crete|Greece|658[0n]
Pontia edusa|EZROM512-08|RV-07-D955||Romania|658[0n]
Pontia edusa|EZROM513-08|RV-07-D970||Romania|658[0n]
Pontia edusa|EZROM511-08|RV-06-V684||Romania|658[0n]
Pontia edusa|EZROM510-08|RV-06-M919||Romania|658[0n]
Pontia edusa|EZRMN217-08|RVcoll.08-M360||Romania|658[0n]
Pontia edusa|LEFIF119-10|MM10575||Finland|658[0n]
Pontia edusa|EULEP938-15|RVcoll.14-C905||Bulgaria|658[0n]
Pontia edusa|EULEP1304-15|RVcoll.14-F750||Greece|658[0n]
Pontia edusa|GWOSI553-10|BC ZSM Lep 44399||Germany|658[0n]
Pontia edusa|BIBSA1626-16|LEP-SS-00392||Italy|658[0n]
Pontia edusa|OXB685-15|12-M379|Sicily W|Italy|658[0n]
Pontia edusa|OXB684-15|12-M354|Sicily E|Italy|658[0n]
Pontia edusa|WMB4691-14|RVcoll.LD-2745||Italy|658[0n]
Pontia edusa|WMB4589-14|RVcoll.LD-170|Elba|Italy|658[0n]
Pontia edusa|WMB4294-14|RVcoll.14-A390||Italy|653[0n]
Pontia edusa|EULEP5896-18|RVcoll17A022|Malta|Malta|651[0n]
Pontia edusa|EULEP5898-18|RVcoll17A024|Malta|Malta|651[0n]
Pontia edusa|WMB4909-14|RVcoll.14-I456||Italy|658[0n]
Pontia edusa|WMB4819-14|RVcoll. 14-I366||Italy|658[0n]
Pontia edusa|WMB4766-14|RVcoll. 14-I313||Italy|658[0n]
Pontia edusa|WMB535-11|RVcoll.11-H690|Levanzo|Italy|658[0n]
Pontia edusa|EZROM594-08|RV-07-C378||Romania|658[0n]
Pontia edusa|EZRMN216-08|RVcoll.08-M234||Romania|658[0n]
Pontia edusa|GWOSU1022-11|BC Back 0072||Germany|658[0n]
Pontia edusa|ODOPE744-11|BC ZSM Lep 50385||Germany|658[0n]
Pontia edusa|PHLAI567-13|TLMF Lep 09129||Italy|658[0n]
Pontia edusa|ABOLD057-16|TLMF Lep 21135||Austria|658[0n]
Pontia edusa|WMB4674-14|RVcoll.LD-2633|Ischia|Italy|658[0n]
Pontia edusa|WMB4663-14|RVcoll.LD-2573|Stromboli|Italy|658[0n]
Pontia edusa|GWOSN899-11|BC ZSM Lep 54530|e.l.|Germany|658[0n]
Pontia edusa|GWORA2445-09|BC ZSM Lep 30657||Germany|658[0n]
Pontia edusa|WMB2736-13|RVcoll.10-C594||Malta|658[0n]
Pontia edusa|WMB3076-14|RVcoll.14-D943|Elba|Italy|658[0n]
Pontia edusa|WMB2964-14|RVcoll.12-O038|Argentario|Italy|658[0n]
Pontia edusa|WMB164-11|RVcoll.11-D369|Sicily|Italy|658[0n]
Pontia edusa|WMB476-11|RVcoll.11-H501|Capri|Italy|658[0n]
Pontia edusa|WMB511-11|RVcoll.11-H598|Sicily|Italy|658[0n]
Pontia edusa|EZRMN215-08|RVcoll.07-D901||Romania|658[0n]
Pontia edusa|LEFIF118-10|MM10574||Finland|658[0n]
Pontia edusa|WMB2413-13|RVcoll.10-C616||Italy|658[0n]
Pontia edusa|WMB2283-13|RVcoll.10-C453||Malta|658[0n]
Pontia edusa|WMB2374-13|RVcoll.LD-2719||Malta|658[0n]
Pontia edusa|WMB2112-13|RVcoll.12-R182|Sicily|Italy|658[0n]
Pontia edusa|WMB2067-13|RVcoll.12-Q897||Italy|658[0n]
Pontia edusa|WMB834-13|RVcoll.07-E223||Italy|658[0n]
Pontia edusa|WMB2765-13|RVcoll.11-H813|Vulcano|Italy|658[0n]
Pontia edusa|WMB2737-13|RVcoll.10-C595||Malta|658[0n]
Pontia edusa|WMB4662-14|RVcoll.LD-2572|Lipari|Italy|658[0n]
Pontia edusa|WMB4661-14|RVcoll.LD-2571|Lipari|Italy|658[0n]
Pontia edusa|WMB4330-14|RVcoll.14-A703||Italy|658[0n]
Pontia edusa|WMB4270-14|RVcoll.14-A172||Italy|658[0n]
Pontia edusa|WMB4493-14|RVcoll.14-E169||Italy|658[0n]
Pontia edusa|WMB4486-14|RVcoll.14-D933||Italy|658[0n]
Pontia edusa|WMB2644-13|RVcoll.10-C563||Italy|658[0n]
Pontia edusa|WMB5973-17|RVcoll14W000|Ustica|Italy|658[0n]
Pontia edusa|EULEP4548-16|RVcoll15J126||Switzerland|658[0n]
Pontia edusa|WMB4950-14|RVcoll. 14-I497||Italy|658[0n]
Pontia edusa|EULEP5694-17|RVcoll16L037|Elba|Italy|658[0n]
Pontia edusa|EULEP4970-16|RVcoll16I404||Poland|658[0n]
Pontia edusa|EULEP735-15|RVcoll.12-N846|Lesvos|Greece|658[0n]
Pontia edusa|EULEP5719-17|RVcoll16L190||Italy|658[0n]
Pontia edusa|BIBSA1232-15|15-N043||Italy|658[0n]
Pontia edusa|EULEP1488-15|RVcoll.14-G319||Greece|658[0n]
Pontia edusa|BIBSA1305-15|11-Y096|Sicily|Italy|658[0n]
Pontia edusa|BIBSA1015-15|15-C138||Italy|658[0n]
Pontia edusa|BIBSA1749-16|12-R164||Italy|658[0n]
Pontia edusa|BIBSA1772-16|LD-2597||Italy|658[0n]
Pontia edusa|BIBSA454-15|RVcoll.14-I122||Italy|658[0n]
Pontia edusa|BIBSA1832-17|RVcoll16C707||Italy|658[0n]
Pontia edusa|LEATJ1164-16|TLMF Lep 19487||Czech Republic|658[0n]
Pontia edusa|OXB1101-15|15-A634||Italy|658[0n]
Pontia edusa|OXB605-15|14-N994||Italy|658[0n]
Pontia edusa|OXB1175-15|Rvcoll_16-A062||Italy|658[0n]
Pontia edusa|OXB1176-15|Rvcoll_16-A063||Italy|658[0n]
Pontia edusa|EULEP5895-18|RVcoll17A021|Malta|Malta|653[0n]

[illegible]
Pontia edusa|OXB1175-15|Rvcoll_16-A062||Italy|658[0n]
Pontia edusa|OXB1176-15|Rvcoll_16-A063||Italy|658[0n]
Pontia edusa|EULEP5895-18|RVcoll17A021|Malta|Malta|653[0n]
Pontia edusa|EULEP4549-16|RVcoll15C275||Italy|647[0n]
Pontia edusa|WMB1058-13|RVcoll.10-C675|Lipari|Italy|622[0n]
Pontia edusa|ABOLB141-15|TLMF Lep 17146||Austria|658[0n]
Pontia chloridice|EULEP3089-15|RVcoll.14-O222|Cyprus|Cyprus|658[0n]
Pontia chloridice|EULEP1574-15|RVcoll.14-G595||Macedonia|614[0n]
Pontia chloridice|EULEP1572-15|RVcoll.14-G576||Macedonia|658[0n]
Pontia chloridice|EULEP1571-15|RVcoll.14-G575||Macedonia|658[0n]
Pontia chloridice|EULEP1221-15|RVcoll.14-F521||Greece|658[0n]
Pontia chloridice|EULEP1220-15|RVcoll.14-F520||Greece|658[0n]
Pontia chloridice|EULEP1217-15|RVcoll.14-F517||Greece|658[0n]
Pontia callidice|LEATG067-14|TLMF Lep 13854||Austria|658[1n]
Pontia callidice|EZSPM023-09|RVcoll.08-P088||Andorra|646[0n]
Pontia callidice|EZSPM639-12|RVcoll.11-H033||Spain|658[0n]
Pontia callidice|EZSPM638-12|RVcoll.11-H031||Spain|658[0n]
Pontia callidice|EZSPM637-12|RVcoll.11-H030||Spain|658[0n]
Pontia callidice|EZSPN1069-11|Rvcoll.09-T054||Spain|658[0n]
Pontia callidice|EZSPN248-09|RVcoll.07-W124||Spain|658[0n]
Pontia callidice|EZSPN030-09|RVcoll.09-T061||Spain|658[0n]
Pontia callidice|EZSPN029-09|RVcoll.09-T056||Spain|658[0n]
Pontia callidice|OXB372-15|RVcoll.14-N086||Italy|658[0n]
Pontia callidice|LEASS959-17|TLMF Lep 22512||Austria|658[0n]
Pontia callidice|LEASS859-17|KLM Lep 08364||Austria|658[0n]
Pontia callidice|EULEP4546-16|RVcoll15H661||Switzerland|658[0n]
Pontia callidice|EULEP4544-16|RVcoll15G731||Italy|658[0n]
Pontia callidice|GWOSN576-11|BC ZSM Lep 52687||Germany|658[0n]
Pontia callidice|ODOPE743-11|BC ZSM Lep 50384||Germany|658[0n]
Pontia callidice|LEATC618-13|TLMF Lep 12600||Austria|658[0n]
Pontia callidice|LEATC114-13|TLMF Lep 11241||Italy|658[0n]
Pontia callidice|PHLAH290-12|TLMF Lep 07729||Austria|658[0n]
Pontia callidice|EULEP4545-16|RVcoll15H572||Switzerland|658[0n]
Pontia callidice|LEATC115-13|TLMF Lep 11242||Italy|658[0n]
Pontia callidice|EULEP4547-16|RVcoll15J377||France|658[0n]
Pontia callidice|BIBSA331-15|RVcoll.14-E137||Italy|658[0n]
Pontia callidice|EULEP4543-16|RVcoll14V912||Switzerland|653[0n]
Pontia callidice|LEATG066-14|TLMF Lep 13853||Austria|658[0n]
Pieris krueperi|EULEP3040-15|RVcoll.14-O173|Corfu|Greece|658[0n]
Pieris krueperi|EULEP3041-15|RVcoll.14-O174|Samos|Greece|658[0n]
Pieris krueperi|EULEP1690-15|RVcoll.14-H031||Greece|658[0n]
Pieris krueperi|EULEP3039-15|RVcoll.14-O172||Macedonia|658[0n]
Pieris krueperi|EULEP1811-15|RVcoll.14-H789||Greece|658[0n]
Pieris krueperi|EULEP1776-15|RVcoll.14-H548||Greece|658[0n]
Pieris krueperi|EULEP1753-15|RVcoll.14-H405||Greece|658[0n]
Pieris krueperi|EULEP1735-15|RVcoll.14-H288||Greece|658[0n]
Pieris krueperi|EULEP1700-15|RVcoll.14-H111||Greece|658[0n]
Pieris krueperi|EULEP1691-15|RVcoll.14-H034||Greece|658[0n]
Pieris krueperi|EULEP1607-15|RVcoll.14-G662||Greece|658[0n]
Pieris krueperi|EULEP4432-16|RVcoll14A970||Bulgaria|658[0n]
Pieris ergane|EULEP1159-15|RVcoll.14-F361||Bulgaria|658[0n]
Pieris ergane|EULEP1369-15|RVcoll.14-F957||Greece|658[0n]
Pieris ergane|EULEP1541-15|RVcoll.14-G510||Greece|658[0n]
Pieris ergane|EULEP3038-15|RVcoll.14-O171||Hungary|658[0n]
Pieris ergane|EULEP4430-16|RVcoll14G180||Greece|658[0n]
Pieris ergane|EULEP4429-16|RVcoll10A986||Bulgaria|658[0n]
Pieris ergane|EULEP1738-15|RVcoll.14-H303||Greece|658[0n]
Pieris ergane|EULEP1424-15|RVcoll.14-G101||Greece|658[0n]
Pieris ergane|EULEP1296-15|RVcoll.14-F728||Greece|658[0n]
Pieris ergane|WMB5136-14|RVcoll.07-E063||Italy|658[0n]
Pieris ergane|EZSPC1260-10|RVcoll.220602AT3||Spain|658[0n]
Pieris ergane|EZSPM821-12|Rvcoll. 140410KV39||Spain|658[0n]
Pieris ergane|EZSPN082-09|RVcoll.08-J653||Spain|658[0n]
Pieris ergane|EZSPC601-09|RVcoll.08-L530||Spain|658[0n]
Pieris ergane|EZSPC599-09|RVcoll.08-L528||Spain|658[0n]
Pieris ergane|EULEP3042-15|RVcoll.14-O175||France|658[0n]
Pieris ergane|EZSPC600-09|RVcoll.08-L529||Spain|658[0n]
Pieris ergane|EZSPN039-09|RVcoll.09-T096||Spain|658[0n]
Pieris ergane|EZSPN038-09|RVcoll.09-T095||Spain|658[0n]
Pieris ergane|EULEP4431-16|RVcoll15C555||Italy|614[0n]
Pieris ergane|WMB5221-14|RVcoll.13-T971||Italy|658[0n]
Pieris ergane|WMB2024-13|RVcoll.12-Q699||Italy|658[0n]
Pieris ergane|WMB797-13|RVcoll.07-D893||Italy|658[0n]
Pieris ergane|WMB2125-13|RVcoll.12-R222||Italy|658[0n]
Pieris ergane|BIBSA756-15|LEP-SS-00257||Italy|658[0n]
Pieris ergane|EULEP1849-15|RVcoll.13-S663||Italy|658[0n]
Pieris ergane|WMB5183-14|RVcoll.13-S688||Italy|658[0n]
Pieris ergane|WMB4013-14|RVcoll.12-R201||Italy|658[0n]
Pieris ergane|GWORU080-10|BC ZSM Lep 30341||Italy|658[0n]
Pieris napi|GWORO778-09|BC ZSM Lep 30470||Germany|658[0n]
Pieris napi|OXB456-15|OXB-TGS-330||United Kingdom|614[0n]
Pieris napi|WMB351-11|RVcoll.11-E778||France|658[0n]
Pieris napi|EZSPM234-09|RVcoll.08-R432||Spain|658[0n]
Pieris napi|OXB383-15|OXB-TGS-003||United Kingdom|658[0n]
Pieris napi|OXB1197-15|Rvcoll_15-M613||France|658[0n]
Pieris napi|EZSPN562-09|RVcoll.08-J101||Portugal|650[0n]
Pieris|EULEP736-15|RVcoll.12-N847|napi/balcana|Greece|658[0n]
Pieris|EZROM1071-09|RVcoll.08-L363|napi/balcana|Romania|658[0n]
Pieris napi|WMB4027-14|RVcoll.12-Z121||Ireland|621[0n]
Pieris napi|LEASS512-17|TLMF Lep 22160||Austria|658[0n]
Pieris napi|GBLAA1079-15|BC ZSM Lep 86585||Germany|658[0n]
Pieris|EZRMN179-08|RVcoll.08-M212|napi/balcana|Romania|658[0n]
Pieris|EZROM465-08|RV-07-C989|napi/balcana. Genit. examined|Romania|658[0n]
Pieris napi|EZROM1041-09|RVcoll.07-C327||Romania|658[0n]
Pieris napi|LEFIB138-10|MM00509||Finland|658[0n]
Pieris napi|EZSPN861-09|RVcoll.08-L729||Spain|633[0n]
Pieris napi|EULEP978-15|RVcoll.14-D103||Austria|658[0n]
Pieris napi|EZSPM233-09|RVcoll.08-R430||Spain|658[0n]
Pieris napi|OXB460-15|OXB-TGS-392||United Kingdom|658[0n]
Pieris napi|OXB394-15|OXB-TGS-033||United Kingdom|658[0n]
Pieris napi|OXB429-15|OXB-TGS-141||United Kingdom|658[0n]
Pieris napi|EULEP4437-16|RVcoll15G039||France|616[0n]
[illegible]

Pieris napi|OXB394-15|OXB-TGS-033||United Kingdom|658[0n]
Pieris napi|OXB429-15|OXB-TGS-141||United Kingdom|658[0n]
Pieris napi|EULEP4437-16|RVcoll15G039||France|616[0n]
Pieris napi|EZSPM374-09|RVcoll.08-J861||Spain|658[0n]
Pieris napi|EULEP4449-16|RVcoll15G161||France|624[0n]
Pieris napi|EULEP4949-16|RVcoll16I169||Germany|651[0n]
Pieris napi|EULEP4452-16|RVcoll15P008||Belarus|658[0n]
Pieris napi|OXB457-15|OXB-TGS-331||United Kingdom|658[0n]
Pieris napi|OXB1450-16|OXB-TGS-1224||United Kingdom|658[0n]
Pieris napi|EZSPM146-09|RVcoll.08-P659||Spain|658[0n]
Pieris napi|OXB439-15|OXB-TGS-164||United Kingdom|658[0n]
Pieris napi|OXB440-15|OXB-TGS-165||United Kingdom|658[0n]
Pieris napi|OXB441-15|OXB-TGS-166||United Kingdom|658[0n]
Pieris napi|OXB1315-15|Rvcoll_15-M581||France|658[0n]
Pieris napi|OXB447-15|OXB-TGS-206||United Kingdom|658[0n]
Pieris napi|OXB448-15|OXB-TGS-207||United Kingdom|658[0n]
Pieris napi|OXB461-15|OXB-TGS-393||United Kingdom|658[0n]
Pieris napi|OXB484-15|OXB-TGS-507||United Kingdom|658[0n]
Pieris napi|OXB485-15|OXB-TGS-508||United Kingdom|658[0n]
Pieris napi|OXB523-15|OXB-TGS-831||United Kingdom|658[0n]
Pieris napi|OXB535-15|OXB-TGS-890||United Kingdom|658[0n]
Pieris napi|OXB536-15|OXB-TGS-891||United Kingdom|658[0n]
Pieris napi|OXB540-15|OXB-TGS-915||United Kingdom|658[0n]
Pieris napi|OXB541-15|OXB-TGS-916||United Kingdom|658[0n]
Pieris napi|OXB542-15|OXB-TGS-917||United Kingdom|658[0n]
Pieris napi|OXB548-15|OXB-TGS-923||United Kingdom|658[0n]
Pieris napi|OXB549-15|OXB-TGS-924||United Kingdom|658[0n]
Pieris napi|OXB1386-15|Rvcoll_15-M751||France|658[0n]
Pieris napi|OXB1406-15|Rvcoll_15-M776||France|658[0n]
Pieris napi|OXB438-15|OXB-TGS-163||United Kingdom|658[0n]
Pieris napi|OXB433-15|OXB-TGS-145||United Kingdom|658[0n]
Pieris napi|OXB431-15|OXB-TGS-143||United Kingdom|658[0n]
Pieris napi|OXB430-15|OXB-TGS-142||United Kingdom|658[0n]
Pieris napi|OXB412-15|OXB-TGS-079||United Kingdom|658[0n]
Pieris napi|OXB411-15|OXB-TGS-078||United Kingdom|658[0n]
Pieris napi|OXB410-15|OXB-TGS-077||United Kingdom|658[0n]
Pieris napi|OXB409-15|OXB-TGS-076||United Kingdom|658[0n]
Pieris napi|OXB408-15|OXB-TGS-075||United Kingdom|658[0n]
Pieris napi|OXB403-15|OXB-TGS-067||United Kingdom|658[0n]
Pieris napi|OXB402-15|OXB-TGS-066||United Kingdom|658[0n]
Pieris napi|OXB401-15|OXB-TGS-065||United Kingdom|658[0n]
Pieris napi|OXB400-15|OXB-TGS-064||United Kingdom|658[0n]
Pieris napi|OXB399-15|OXB-TGS-063||United Kingdom|658[0n]
Pieris napi|OXB1035-15|LD-2417||France|658[0n]
Pieris napi|OXB385-15|OXB-TGS-005||United Kingdom|658[0n]
Pieris napi|OXB384-15|OXB-TGS-004||United Kingdom|658[0n]
Pieris napi|OXB382-15|OXB-TGS-002||United Kingdom|658[0n]
Pieris napi|OXB381-15|OXB-TGS-001||United Kingdom|658[0n]
Pieris napi|OXB1526-16|OXB-TGS-1244||United Kingdom|658[0n]
Pieris napi|OXB1453-16|OXB-TGS-1227||United Kingdom|658[0n]
Pieris napi|OXB1451-16|OXB-TGS-1225||United Kingdom|658[0n]
Pieris napi|OXB1449-16|OXB-TGS-1223||United Kingdom|658[0n]
Pieris napi|LEFIJ4746-16|ZMBSU-02125||Belarus|658[0n]
Pieris napi|BIBSA1132-15|15-L942||Italy|658[0n]
Pieris napi|EULEP2020-15|RVcoll.14-I853||Poland|658[0n]
Pieris napi|EULEP833-15|RVcoll.14-B988||Sweden|658[0n]
Pieris napi|EULEP5887-18|RVcoll15O591||France|658[0n]
Pieris napi|EULEP4435-16|RVcoll10A765||Estonia|658[0n]
Pieris bryoniae|EULEP4425-16|RVcoll15I549||Italy|658[0n]
Pieris napi|WMB3828-14|RVcoll.09-X850||Italy|658[0n]
Pieris napi|WMB1690-13|RVcoll.12-P557||France|658[0n]
Pieris napi|WMB3773-14|RVcoll.12-N707||Spain|658[0n]
Pieris napi|WMB1181-13|RVcoll.11-E875|Corsica|France|658[0n]
Pieris napi|WMB3164-14|RVcoll.08-H441||Spain|658[0n]
Pieris napi|WMB2907-14|RVcoll.11-E819|Corsica|France|658[0n]
Pieris napi|EZSPM877-12|Rvcoll.130711PX87||Spain|658[0n]
Pieris napi|EZSPM875-12|Rvcoll.130711PX61||Spain|658[0n]
Pieris napi|EZSPM861-12|Rvcoll.050111LP92||Spain|658[0n]
Pieris napi|EZSPM442-09|Rvcoll.09-V598||Spain|658[0n]
Pieris napi|EZSPM174-09|RVcoll.08-R037||Spain|658[0n]
Pieris napi|EZSPM343-09|RVcoll.08-R004||Spain|658[0n]
Pieris napi|EZSPN485-09|RVcoll.08-H922||Spain|658[0n]
Pieris napi|EZSPN484-09|RVcoll.08-H921||Spain|658[0n]
Pieris napi|EZSPN429-09|RVcoll.08-H605||Spain|658[0n]
Pieris napi|EZSPC607-09|RVcoll.08-P007||Spain|658[0n]
Pieris napi|EZROM468-08|RV-07-D261||Romania|658[0n]
Pieris|EZROM1044-09|RVcoll.07-D065|napi/balcana|Romania|658[0n]
Pieris napi|EZROM467-08|RV-06-K569|Genit. examined|Romania|658[0n]
Pieris napi|EZROM590-08|RV-07-C315||Romania|658[0n]
Pieris bryoniae|GWOSF849-10|BC ZSM Lep 45835||Germany|658[0n]
Pieris napi|LENOA1352-11|LN-BD1352||France|658[0n]
Pieris napi|OXB1300-15|Rvcoll_15-M167||France|658[0n]
Pieris napi|OXB1452-16|OXB-TGS-1226||United Kingdom|658[0n]
Pieris napi|LON869-11|NHMO Lep09067||Norway|658[0n]
Pieris napi|LEFIB141-10|MM00514||Finland|658[0n]
Pieris napi|EULEP2334-15|RVcoll.14-V019||Denmark|658[0n]
Pieris napi|EULEP1995-15|RVcoll.14-I765||Poland|658[0n]
Pieris napi|EULEP4443-16|RVcoll15I390||Austria|658[0n]
Pieris napi|WMB3264-14|RVcoll.08-L766||Spain|658[0n]
Pieris napi|ABOLD063-16|TLMF Lep 21141||Austria|658[0n]
Pieris napi|GWORU079-10|BC ZSM Lep 30340||Italy|630[0n]
Pieris napi|BIBSA1572-16|LEP-SS-00389||Italy|658[0n]
Pieris napi|GBLAA1949-15|BC ZSM Lep 89070||Italy|658[0n]
Pieris napi|WMB4997-14|RVcoll. 14-I544||Italy|658[0n]
Pieris napi|EULEP5690-17|RVcoll16L033||Italy|630[0n]
Pieris napi|WMB2336-13|RVcoll.12-M277||Italy|658[0n]
Pieris napi|OXB1225-15|Rvcoll_15-M657||France|613[0n]
Pieris napi|OXB1055-15|15-A600||Italy|658[0n]
Pieris|EULEP1065-15|RVcoll.14-E970|napi/balcana|Serbia|614[0n]
Pieris napi|GBLAA1498-15|BC ZSM Lep 87289||Germany|658[0n]
Pieris napi|BIBSA026-14|LEP-SS-00026||Italy|632[0n]
Pieris|EULEP1697-15|RVcoll.14-H092|napi/balcana|Greece|624[0n]
Pieris napi|WMB2857-14|RVcoll.09-X818|Argentario|Italy|658[0n]

[illegible]
Pieris napi|BIBSA026-14|LEP-SS-00026||Italy|632[0n]
Pieris|EULEP1697-15|RVcoll.14-H092|napi/balcana|Greece|624[0n]
Pieris napi|WMB2857-14|RVcoll.09-X818|Argentario|Italy|658[0n]
Pieris|EZROM470-08|RV-07-D027|napi/balcana. Genit. examined|Romania|658[0n]
Pieris napi|WMB5369-14|RVcoll.14-J757||France|658[0n]
Pieris napi|WMB4851-14|RVcoll.14-I398||Italy|658[0n]
Pieris napi|GWORU373-10|BC ZSM Lep 31964||Italy|658[0n]
Pieris napi|GWORR431-10|BC ZSM Lep 29647||Italy|658[0n]
Pieris napi|EZROM649-08|RV-07-D544||Romania|658[0n]
Pieris|EZROM469-08|RV-07-D091|napi/balcana|Romania|658[0n]
Pieris|EZROM1059-09|RVcoll.07-E442|napi/balcana|Romania|658[0n]
Pieris|EZROM650-08|RV-07-D929|napi/balcana|Romania|658[0n]
Pieris napi|WMB4557-14|RVcoll.080611XP57||Spain|658[0n]
Pieris napi|WMB3896-14|RVcoll.12-P016||France|658[0n]
Pieris|EZROM472-08|RV-07-E530|napi/balcana|Romania|658[0n]
Pieris|EZROM1054-09|RVcoll.07-D979|napi/balcana|Romania|658[0n]
Pieris napi|WMB1285-13|RVcoll.12-M449|Sicily|Italy|658[0n]
Pieris napi|WMB3208-14|RVcoll.08-J305||Spain|658[0n]
Pieris napi|WMB1296-13|RVcoll.12-M568|Sicily|Italy|658[0n]
Pieris napi|WMB1771-13|RVcoll.12-Q049||France|658[0n]
Pieris napi|WMB2099-13|RVcoll.12-R112|Sicily|Italy|658[0n]
Pieris napi|WMB2066-13|RVcoll.12-Q888||Italy|658[0n]
Pieris napi|WMB2042-13|RVcoll.12-Q771||Italy|658[0n]
Pieris napi|WMB1601-13|RVcoll.12-O649||France|658[0n]
Pieris napi|WMB4014-14|RVcoll.12-R202||Italy|658[0n]
Pieris napi|WMB4001-14|RVcoll.12-Q513||Spain|658[0n]
Pieris napi|WMB3957-14|RVcoll.12-P768||France|658[0n]
Pieris napi|WMB3940-14|RVcoll.12-P629||France|658[0n]
Pieris napi|WMB3869-14|RVcoll.11-J598||Italy|658[0n]
Pieris napi|WMB3860-14|RVcoll.11-I249||Italy|658[0n]
Pieris napi|WMB4235-14|RVcoll.13-T835||Italy|658[0n]
Pieris napi|WMB4152-14|RVcoll.13-S701||Italy|658[0n]
Pieris napi|WMB2634-13|RVcoll.12-O104|Corsica|France|658[0n]
Pieris napi|WMB2582-13|RVcoll.12-R102|Sicily|Italy|658[0n]
Pieris napi|WMB3804-14|RVcoll.07-D842||Italy|658[0n]
Pieris napi|WMB2218-13|RVcoll.11-Y038||Italy|658[0n]
Pieris napi|WMB2207-13|RVcoll.11-Y016|Elba|Italy|658[0n]
Pieris napi|WMB2043-13|RVcoll.12-Q773||Italy|658[0n]
Pieris napi|WMB3175-14|RVcoll.08-H572||Spain|658[0n]
Pieris napi|WMB3089-14|RVcoll.14-E185|Elba|Italy|658[0n]
Pieris napi|WMB2871-14|RVcoll.10-C560|Argentario|Italy|658[0n]
Pieris napi|WMB2858-14|RVcoll.09-X819|Argentario|Italy|658[0n]
Pieris napi|WMB149-11|RVcoll.11-D211|Sicily|Italy|658[0n]
Pieris napi|EZSPM166-09|RVcoll.08-R001||Spain|658[0n]
Pieris napi|EZSPC606-09|RVcoll.07-C673||Spain|658[0n]
Pieris napi|EZSPC605-09|RVcoll.08-P376||Spain|658[0n]
Pieris napi|EZSPN883-09|RVcoll.08-L792||Spain|658[0n]
Pieris napi|EZSPC604-09|RVcoll.06-H857||Spain|658[0n]
Pieris napi|BIBSA1836-17|RVcoll16C711||Italy|658[0n]
Pieris napi|BIBSA151-15|RVcoll.14-I163||Italy|658[0n]
Pieris napi|WMB4833-14|RVcoll. 14-I380||Italy|658[0n]
Pieris napi|WMB4799-14|RVcoll. 14-I346||Italy|658[0n]
Pieris napi|BIBSA1740-16|14-A791||Italy|658[0n]
Pieris napi|BIBSA1734-16|LD-2783||Italy|658[0n]
Pieris napi|WMB639-11|RVcoll.11-I143||Italy|658[0n]
Pieris napi|WMB5091-14|RVcoll. 14-L222||Italy|658[0n]
Pieris napi|WMB5060-14|RVcoll. 14-L191||Italy|658[0n]
Pieris napi|WMB4978-14|RVcoll. 14-I525||Italy|658[0n]
Pieris napi|WMB4721-14|RVcoll.LD-3013|Sicily|Italy|658[0n]
Pieris napi|WMB4687-14|RVcoll.LD-2687||Italy|658[0n]
Pieris napi|WMB4933-14|RVcoll.14-I480||Italy|658[0n]
Pieris napi|WMB4926-14|RVcoll.14-I473||Italy|658[0n]
Pieris napi|WMB4308-14|RVcoll.14-A421||Italy|658[0n]
Pieris napi|WMB4295-14|RVcoll.14-A391||Italy|658[0n]
Pieris napi|WMB5226-14|RVcoll.13-T985||Italy|658[0n]
Pieris napi|WMB5129-14|RVcoll. 14-L260||Italy|658[0n]
Pieris napi|EULEP4440-16|RVcoll15H204||Switzerland|658[0n]
Pieris napi|WMB5498-14|RVcoll.14-J402|Giannutri|Italy|658[0n]
Pieris napi|EULEP4445-16|RVcoll15I733||Austria|658[0n]
Pieris napi|EULEP4444-16|RVcoll15I477||Austria|658[0n]
Pieris napi|EULEP4448-16|RVcoll15G084||France|658[0n]
Pieris napi|EULEP4446-16|RVcoll15I824||Austria|658[0n]
Pieris napi|EULEP4979-16|RVcoll16I521||Poland|658[0n]
Pieris napi|EULEP4453-16|RVcoll15P034||Ukraine|658[0n]
Pieris napi|EULEP2431-15|RVcoll.14-V227||Belgium|658[0n]
Pieris napi|EULEP2407-15|RVcoll.14-V118||Ukraine|658[0n]
Pieris|EULEP934-15|RVcoll.14-C882|napi/balcana|Bulgaria|658[0n]
Pieris napi|EULEP625-15|RVcoll.11-J008||Switzerland|658[0n]
Pieris napi|OXB913-15|15-A557||Italy|658[0n]
Pieris napi|OXB906-15|15-A550||Italy|658[0n]
Pieris napi|OXB671-15|07-E084||Italy|658[0n]
Pieris napi|OXB1125-15|15-A658||Italy|658[0n]
Pieris napi|OXB598-15|14-N987||Italy|658[0n]
Pieris napi|OXB591-15|14-N980||Italy|658[0n]
Pieris napi|OXB581-15|14-N970||Italy|658[0n]
Pieris|LEATJ1240-16|TLMF Lep 19563|napi/balcana|Croatia|658[0n]
Pieris napi|BIBSA1830-17|RVcoll16C705||Italy|658[0n]
Pieris napi|BIBSA972-15|15-C310||Italy|658[0n]
Pieris napi|BIBSA257-15|RVcoll.14-E053||Italy|658[0n]
Pieris napi|BIBSA255-15|RVcoll.14-E051||Italy|658[0n]
Pieris napi|BIBSA1716-16|15-N209||Italy|658[0n]
Pieris napi|BIBSA680-15|RVcoll. 12-Q810||Italy|658[0n]
Pieris napi|BIBSA1414-15|15-N033||Italy|658[0n]
Pieris napi|BIBSA1336-15|15-M828||Italy|658[0n]
Pieris napi|BIBSA116-15|RVcoll.14-D549||Italy|658[0n]
Pieris napi|BIBSA1768-16|LD-3095||Italy|658[0n]
Pieris napi|BIBSA1302-15|09-X880|Corsica|France|658[0n]
Pieris napi|BIBSA1677-16|15-C880||Italy|658[0n]
Pieris napi|BIBSA1010-15|15-C120||Italy|658[0n]
Pieris napi|EULEP1999-15|RVcoll.14-I775||Slovakia|658[0n]
Pieris napi|WMB5074-14|RVcoll. 14-L205||Italy|658[0n]
Pieris napi|WMB6567-18|RVcoll14A080||Italy|658[0n]
[illegible]

Pieris napi|EULEP1999-15|RVcoll.14-I775||Slovakia|658[0n]
Pieris napi|WMB5074-14|RVcoll. 14-L205||Italy|658[0n]
Pieris napi|WMB6567-18|RVcoll14A080||Italy|658[0n]
Pieris napi|OXB758-15|13-U493||Italy|658[0n]
Pieris napi|OXB709-15|13-T889||Italy|658[0n]
Pieris napi|OXB925-15|15-A569||Italy|658[0n]
Pieris napi|OXB343-15|RVcoll.14-N057||Italy|658[0n]
Pieris napi|OXB369-15|RVcoll.14-N083||Italy|658[0n]
Pieris napi|OXB956-15|15-A906||Italy|658[0n]
Pieris napi|OXB967-15|15-A917||Italy|658[0n]
Pieris napi|OXB968-15|15-A918||Italy|658[0n]
Pieris napi|OXB996-15|15-A946||Italy|658[0n]
Pieris napi|OXB1336-15|Rvcoll_15-M680||France|658[0n]
Pieris napi|OXB1195-15|Rvcoll_15-M610||France|658[0n]
Pieris napi|OXB1007-15|15-A957||Italy|658[0n]
Pieris napi|OXB1261-15|Rvcoll_15-M117||France|658[0n]
Pieris napi|OXB1368-15|Rvcoll_15-M729||France|658[0n]
Pieris napi|OXB1098-15|15-A631||Italy|658[1n]
Pieris napi|WMB1174-13|RVcoll.11-E833|Corsica|France|614[0n]
Pieris napi|EULEP4438-16|RVcoll15G544||Switzerland|639[0n]
Pieris napi|BIBSA1640-16|15-C554||Italy|623[0n]
Pieris napi|EULEP4450-16|RVcoll15Q121||Russia|645[0n]
Pieris napi|BIBSA025-14|LEP-SS-00025||Italy|630[0n]
Pieris napi|LEATH753-14|TLMF Lep 15965||Italy|634[0n]
Pieris napi|LEATF467-14|TLMF Lep 13779||Austria|658[0n]
Pieris napi|EULEP4447-16|RVcoll15I887||Liechtenstein|658[0n]
Pieris napi|GBLAA2129-15|BC ZSM Lep 79845||Germany|658[0n]
Pieris napi|LEATG538-14|TLMF Lep 14325||Italy|652[0n]
Pieris napi|GBLAA1317-15|BC ZSM Lep 87203||Germany|658[1n]
Pieris napi|BIBSA256-15|RVcoll.14-E052||Italy|637[0n]
Pieris napi|LEATF469-14|TLMF Lep 13781||Austria|658[0n]
Pieris napi|LEATG537-14|TLMF Lep 14324||Italy|658[0n]
Pieris napi|LEATH788-14|TLMF Lep 16000||Italy|658[0n]
Pieris napi|LON172-08|NHMO-06173||Norway|657[0n]
Pieris|EZROM471-08|RV-07-D101|napi/balcana|Romania|658[0n]
Pieris napi|WMB2978-14|RVcoll.12-O138|Corsica|France|658[0n]
Pieris napi|WMB3505-14|RVcoll.10-B779||France|658[0n]
Pieris napi|WMB4907-14|RVcoll.14-I454||Italy|658[0n]
Pieris napi|EULEP4439-16|RVcoll15G742||Switzerland|658[0n]
Pieris napi|EULEP4780-16|RVcoll16H339||Norway|658[0n]
Pieris napi|EULEP5175-17|RVcoll16H096||Sweden|658[0n]
Pieris napi|EULEP2141-15|RVcoll.14-J995||Switzerland|658[0n]
Pieris napi|BIBSA1084-15|15-K573||Italy|658[0n]
Pieris napi|OXB432-15|OXB-TGS-144||United Kingdom|658[0n]
Pieris napi|LEATH719-14|TLMF Lep 15931||Italy|634[0n]
Pieris napi|EZROM466-08|RV-06-K568||Romania|658[0n]
Pieris napi|EULEP5035-16|RVcoll16I994||Slovakia|658[0n]
Pieris napi|ABOLD421-16|TLMF Lep 21573||Austria|658[0n]
Pieris napi|OXB534-15|OXB-TGS-889||United Kingdom|658[0n]
Pieris napi|OXB1228-15|Rvcoll_15-M661||France|658[0n]
Pieris|EULEP1219-15|RVcoll.14-F519|napi/balcana|Greece|658[0n]
Pieris napi|EULEP4441-16|RVcoll15H388||Switzerland|658[0n]
Pieris|EULEP4436-16|RVcoll10B449|napi/balcana|Croatia|658[0n]
Pieris napi|WMB4392-14|RVcoll.14-B314||Portugal|658[0n]
Pieris napi|EZROM747-08|RV-07-F503||Spain|658[0n]
Pieris bryoniae|EZRMN169-08|RVcoll.06-N014||Romania|658[0n]
Pieris bryoniae|EZRMN168-08|RVcoll.06-M991||Romania|658[0n]
Pieris bryoniae|EZRMN167-08|RVcoll.06-M969||Romania|658[0n]
Pieris|EZROM700-08|RV-07-F564|napi/balcana|Romania|658[0n]
Pieris bryoniae|EZROM462-08|RV-06-M997||Romania|658[0n]
Pieris bryoniae|EZROM461-08|RV-06-M972|Genit. examined|Romania|658[0n]
Pieris bryoniae|EZRMN166-08|RVcoll.06-M968||Romania|658[0n]
Pieris napi|LEFIJ500-10|MM17125||Finland|658[0n]
Pieris napi|FBLMU449-09|BC ZSM Lep 27099|trans. bryoniae?|Germany|658[0n]
Pieris|EZRMN180-08|RVcoll.08-M243|napi/balcana. Genit. examined|Romania|658[0n]
Pieris|PHLAF292-11|TLMF Lep 05462|napi/balcana|Macedonia|658[0n]
Pieris bryoniae|EULEP2172-15|RVcoll.14-M520||Romania|658[0n]
Pieris bryoniae|EZRMN170-08|RVcoll.08-M496||Romania|658[0n]
Pieris bryoniae|ODOPE742-11|BC ZSM Lep 50383||Germany|658[0n]
Pieris bryoniae|PHLAB375-10|TLMF Lep 01175||Switzerland|634[0n]
Pieris|EULEP1345-15|RVcoll.14-F860|napi/balcana|Greece|658[1n]
Pieris|EULEP1931-15|RVcoll.14-B897|napi/balcana|Bosnia and Herzegovina|658[0n]
Pieris|EULEP4461-16|RVcoll14O312|napi/balcana|Albania|658[0n]
Pieris bryoniae|EZRMN171-08|RVcoll.08-M609||Romania|658[0n]
Pieris bryoniae|EZRMN172-08|RVcoll.08-M616|Genit. examined|Romania|658[0n]
Pieris bryoniae|BIBSA419-15|RVcoll.14-I087||Italy|632[0n]
Pieris bryoniae|BIBSA420-15|RVcoll.14-I088||Italy|632[0n]
Pieris bryoniae|EULEP4428-16|RVcoll15G411||Switzerland|634[0n]
Pieris bryoniae|PHLAH700-12|TLMF Lep 08519||Austria|658[0n]
Pieris bryoniae|EULEP4420-16|RVcoll15H698||Switzerland|658[0n]
Pieris bryoniae|EULEP4419-16|RVcoll15H593||Switzerland|658[0n]
Pieris bryoniae|EULEP4418-16|RVcoll15H424||Switzerland|658[0n]
Pieris bryoniae|EULEP4417-16|RVcoll15H326||Switzerland|658[0n]
Pieris bryoniae|EULEP4416-16|RVcoll15H241||Switzerland|658[0n]
Pieris bryoniae|EULEP4415-16|RVcoll15G871||Switzerland|658[0n]
Pieris bryoniae|EULEP4414-16|RVcoll15G816||Italy|658[0n]
Pieris bryoniae|EULEP4413-16|RVcoll15G687||Italy|658[0n]
Pieris bryoniae|WMB880-13|RVcoll.09-X287||Italy|658[0n]
Pieris bryoniae|EZROM683-08|RV-06-M978||Romania|658[0n]
Pieris bryoniae|GWORL288-09|BC ZSM Lep 22000||Germany|658[0n]
Pieris bryoniae|LEATD287-13|TLMF Lep 12934||Italy|658[0n]
Pieris bryoniae|PHLAB289-10|TLMF Lep 01089||Switzerland|658[0n]
Pieris bryoniae|LEATG035-14|TLMF Lep 13822||Austria|658[0n]
Pieris bryoniae|PHLAH456-12|TLMF Lep 08275||Austria|658[0n]
Pieris bryoniae|LEATG036-14|TLMF Lep 13823||Austria|658[0n]
Pieris bryoniae|EULEP4424-16|RVcoll15I210||Austria|658[0n]
Pieris bryoniae|EULEP4423-16|RVcoll15I098||Italy|658[0n]
Pieris bryoniae|EULEP4422-16|RVcoll15H997||Italy|658[0n]
Pieris bryoniae|EULEP4421-16|RVcoll15H895||Italy|658[0n]
Pieris bryoniae|EULEP4426-16|RVcoll15I612||Austria|658[0n]
Pieris bryoniae|EULEP4427-16|RVcoll15I808||Austria|658[0n]
Pieris bryoniae|EULEP4442-16|RVcoll15I001||Italy|658[0n]
Pieris bryoniae|EULEP567-15|RVcoll.09-X079||Switzerland|658[0n]
Pieris bryoniae|EULEP804-15|RVcoll.13-U263||Italy|658[0n]

Pieris bryoniae|EULEP4427-16|RVcoll15I808||Austria|658[0n]
Pieris bryoniae|EULEP4442-16|RVcoll15I001||Italy|658[0n]
Pieris bryoniae|EULEP567-15|RVcoll.09-X079||Switzerland|658[0n]
Pieris bryoniae|EULEP804-15|RVcoll.13-U263||Italy|658[0n]
Pieris bryoniae|EULEP810-15|RVcoll.13-U450||Italy|658[0n]
Pieris|EULEP1411-15|RVcoll.14-G078|napi/balcana|Greece|658[0n]
Pieris bryoniae|EULEP1868-15|RVcoll.13-U338||Italy|658[0n]
Pieris bryoniae|EULEP2452-15|RVcoll.14-V274||Switzerland|658[0n]
Pieris bryoniae|EULEP2283-15|RVcoll.14-N469||Ukraine|658[0n]
Pieris bryoniae|BIBSA254-15|RVcoll.14-E050||Italy|658[0n]
Pieris bryoniae|OXB624-15|14-O013||Italy|658[0n]
Pieris bryoniae|OXB735-15|13-U237||Italy|658[0n]
Pieris bryoniae|ABOLD578-17|TLMF Lep 21654||Austria|658[0n]
Pieris bryoniae|BIBSA142-15|RVcoll.14-I155||Italy|629[0n]
Pieris bryoniae|PHLAB288-10|TLMF Lep 01088||Switzerland|630[0n]
Pieris bryoniae|LEATD452-13|TLMF Lep 13099||Italy|658[0n]
Pieris mannii|EZSPC1263-10|RVcoll.060604EM1||Spain|658[0n]
Pieris mannii|EZSPM852-12|Rvcoll. 201210GK94||Spain|658[0n]
Pieris mannii|EZSPC768-10|RVcoll.08-L678|Genit. examined|Spain|658[0n]
Pieris mannii|EZSPM762-12|Rvcoll. 030111FB42||Spain|658[0n]
Pieris mannii|EZSPC963-10|RVcoll.150308PP15||Spain|658[0n]
Pieris mannii|EZSPC962-10|RVcoll.150308PP11||Spain|658[0n]
Pieris mannii|EZSPM357-09|RVcoll.08-R053|Genit. examined|Spain|658[0n]
Pieris mannii|EZSPN531-09|RVcoll.08-J006|Genit. examined|Spain|658[0n]
Pieris mannii|EZSPN781-09|RVcoll.08-L294|Genit. examined|Spain|658[0n]
Pieris mannii|EZSPC1324-10|RVcoll.130209KL97||Spain|658[0n]
Pieris mannii|EZSPC767-10|RVcoll.08-L677|Genit. examined|Spain|658[0n]
Pieris mannii|EZSPN843-09|RVcoll.08-L676|Genit. examined|Spain|658[0n]
Pieris mannii|EULEP1611-15|RVcoll.14-G670||Greece|614[0n]
Pieris mannii|LEASS762-17|TLMF Lep 22410||Austria|658[0n]
Pieris mannii|LEASS761-17|TLMF Lep 22409||Austria|658[0n]
Pieris mannii|LEASS760-17|TLMF Lep 22408||Austria|658[0n]
Pieris mannii|EULEP4433-16|RVcoll10B343||Bulgaria|658[0n]
Pieris mannii|OXB1091-15|15-A624||Italy|658[0n]
Pieris mannii|OXB363-15|RVcoll.14-N077||Italy|658[0n]
Pieris mannii|BIBSA642-15|RVcoll. 15-A805||Italy|658[0n]
Pieris mannii|EULEP1939-15|RVcoll.14-B911||Bosnia and Herzegovina|658[0n]
Pieris mannii|EULEP1752-15|RVcoll.14-H404||Greece|658[0n]
Pieris mannii|EULEP1746-15|RVcoll.14-H365||Greece|658[0n]
Pieris mannii|EULEP1698-15|RVcoll.14-H099||Greece|658[0n]
Pieris mannii|WMB4791-14|RVcoll. 14-I338||Italy|658[0n]
Pieris mannii|WMB604-11|RVcoll.11-I003|Sicily|Italy|658[0n]
Pieris mannii|WMB2296-13|RVcoll.10-C671||Italy|658[0n]
Pieris mannii|EULEP1231-15|RVcoll.14-F535||Greece|658[0n]
Pieris mannii|WMB6607-18|RVcoll15D785||Spain|658[1n]
Pieris mannii|GWORU082-10|BC ZSM Lep 30343||Italy|658[0n]
Pieris mannii|GWOTF704-12|BC ZSM Lep 62410||Germany|658[0n]
Pieris mannii|BIBSA721-15|LD-1752||Italy|658[0n]
Pieris mannii|WMB5473-14|RVcoll.14-N691|Alicudi|Italy|658[0n]
Pieris mannii|WMB2297-13|RVcoll.10-C672||Italy|658[0n]
Pieris mannii|WMB3504-14|RVcoll.10-B770||France|658[0n]
Pieris mannii|EZSPC910-10|RVcoll.09-V801||Spain|658[0n]
Pieris mannii|ABOLD643-17|TLMF Lep 21719||Austria|658[0n]
Pieris mannii|OXB1174-15|Rvcoll_16-A061||Italy|658[0n]
Pieris mannii|EULEP2137-15|RVcoll.14-J984||Switzerland|658[0n]
Pieris mannii|EULEP4434-16|RVcoll15J150||Switzerland|658[0n]
Pieris mannii|WMB1883-13|RVcoll.11-J743||Italy|658[0n]
Pieris mannii|EZSPN067-09|RVcoll.09-V745|Genit. examined|Spain|658[0n]
Pieris mannii|WMB6605-18|RVcoll15D783||Spain|658[0n]
Pieris mannii|EZSPC602-09|RVcoll.07-Z067|Genit. examined|Spain|658[0n]
Pieris mannii|EZROM746-08|RV-06-V759|Genit. examined|Spain|658[0n]
Pieris mannii|PHLAA440-09|TLMF Lep 00480||France|658[0n]
Pieris mannii|BIBSA719-15|LD-1494||Italy|658[0n]
Pieris mannii|WMB2928-14|RVcoll.11-J726|Sicily|Italy|658[0n]
Pieris mannii|OXB775-15|14-A724||Italy|658[0n]
Pieris mannii|EULEP870-15|RVcoll.14-C364||Greece|658[0n]
Pieris mannii|WMB1272-13|RVcoll.12-M300|Lipari|Italy|614[0n]
Pieris mannii|WMB6606-18|RVcoll15D784||Spain|658[4n]
Pieris mannii|EULEP5892-18|RVcoll15O611||Spain|658[0n]
Pieris mannii|WMB4552-14|RVcoll.070611MH02||Spain|658[0n]
Pieris mannii|EZSPC939-10|RVcoll.09-X050|Genit. examined|Spain|658[0n]
Pieris mannii|EZSPN066-09|RVcoll.09-V744|Genit. examined|Spain|658[0n]
Pieris mannii|WMB860-13|RVcoll.09-T294||France|658[0n]
Pieris mannii|WMB2195-13|RVcoll.09-X940|Ponza|Italy|658[0n]
Pieris mannii|WMB2428-13|RVcoll.10-C794|Ischia|Italy|658[0n]
Pieris mannii|WMB4237-14|RVcoll.13-T838||Italy|658[0n]
Pieris mannii|WMB3871-14|RVcoll.11-Y030||Italy|658[0n]
Pieris mannii|WMB3970-14|RVcoll.12-Q139||France|658[0n]
Pieris mannii|WMB4336-14|RVcoll.14-A722||Italy|658[0n]
Pieris mannii|WMB6609-18|RVcoll15D787||Spain|658[0n]
Pieris mannii|WMB481-11|RVcoll.11-H506|Capri|Italy|658[0n]
Pieris mannii|WMB538-11|RVcoll.11-H697|Levanzo|Italy|658[0n]
Pieris mannii|WMB571-11|RVcoll.11-H795|Vulcano|Italy|658[0n]
Pieris mannii|WMB584-11|RVcoll.11-H874|Salina|Italy|658[0n]
Pieris mannii|WMB4792-14|RVcoll. 14-I339||Italy|658[0n]
Pieris mannii|WMB5098-14|RVcoll. 14-L229||Italy|658[0n]
Pieris mannii|WMB5127-14|RVcoll. 14-L258||Italy|658[0n]
Pieris mannii|EULEP4872-16|RVcoll16H947||France|658[0n]
Pieris mannii|EULEP5654-17|RVcoll15J980||Italy|658[0n]
Pieris mannii|EULEP5713-17|RVcoll16L058||Italy|658[0n]
Pieris mannii|BIBSA932-15|16-A023||Italy|658[0n]
Pieris mannii|BIBSA749-15|LEP-SS-00250||Italy|658[0n]
Pieris mannii|OXB1256-15|Rvcoll_15-M112||France|658[0n]
Pieris mannii|ABOLD642-17|TLMF Lep 21718||Austria|658[0n]
Pieris mannii|BIBSA684-15|RVcoll. 12-Q811||Italy|618[0n]
Pieris mannii|WMB2194-13|RVcoll.09-X939|Ponza|Italy|634[0n]
Pieris mannii|WMB1943-13|RVcoll.11-H875|Salina|Italy|658[0n]
Pieris mannii|WMB1661-13|RVcoll.12-P162||France|658[0n]
Pieris mannii|WMB1273-13|RVcoll.12-M301|Lipari|Italy|658[0n]
Pieris mannii|WMB1069-13|RVcoll.10-C693|Ischia|Italy|658[0n]
Pieris mannii|WMB2719-13|RVcoll.10-C534|Ischia|Italy|658[0n]
Pieris mannii|WMB2805-13|RVcoll.12-M302|Lipari|Italy|658[0n]
Pieris mannii|WMB3065-14|RVcoll.14-A797|Capri|Italy|658[0n]

[illegible]
Pieris mannii|WMB2719-13|RVcoll.10-C534|Ischia|Italy|658[0n]
Pieris mannii|WMB2805-13|RVcoll.12-M302|Lipari|Italy|658[0n]
Pieris mannii|WMB3065-14|RVcoll.14-A797|Capri|Italy|658[0n]
Pieris mannii|WMB3020-14|RVcoll.12-Z334|Ponza|Italy|658[0n]
Pieris mannii|WMB3378-14|RVcoll.09-V298||France|658[0n]
Pieris mannii|WMB124-11|RVcoll.11-D019|Capri|Italy|658[0n]
Pieris mannii|EZSPN068-09|RVcoll.09-V748|Genit. examined|Spain|658[0n]
Pieris mannii|EZRMN178-08|RVcoll.08-M647||Romania|658[0n]
Pieris mannii|EZRMN176-08|RVcoll.08-M544|Genit. examined|Romania|658[0n]
Pieris mannii|EZRMN175-08|RVcoll.08-M529||Romania|658[0n]
Pieris mannii|EZRMN174-08|RVcoll.08-M272||Romania|658[0n]
Pieris mannii|EZROM699-08|RV-07-F563||Romania|658[0n]
Pieris mannii|EZROM648-08|RV-07-E356||Romania|658[0n]
Pieris mannii|EZROM463-08|RV-07-C956|Genit. examined|Romania|658[0n]
Pieris mannii|EZROM464-08|RV-07-C963|Genit. examined|Romania|658[0n]
Pieris mannii|EZRMN177-08|RVcoll.08-M547||Romania|658[0n]
Pieris mannii|EZRMN173-08|RVcoll.08-M248||Romania|658[0n]
Pieris mannii|PHLAF626-11|TLMF Lep 05796||Liechtenstein|658[0n]
Pieris mannii|PHLAF627-11|TLMF Lep 05797||Liechtenstein|658[0n]
Pieris rapae|WMB1274-13|RVcoll.12-M305|Lipari|Italy|658[0n]
Pieris rapae|WMB2982-14|RVcoll.12-O184|Corsica|France|658[0n]
Pieris rapae|EULEP846-15|RVcoll.14-C068||Sweden|658[0n]
Pieris rapae|WMB296-11|RVcoll.11-E310||Italy|658[0n]
Pieris rapae|EULEP4458-16|RVcoll15I472||Austria|658[0n]
Pieris rapae|WMB3046-14|RVcoll.13-S623|Capraia|Italy|647[0n]
Pieris rapae|EZSPM743-12|Rvcoll. 12-L550||Portugal|621[0n]
Pieris rapae|GBLAD097-14|BC ZSM Lep 78668||Germany|658[1n]
Pieris rapae|EULEP4459-16|RVcoll15I742||Austria|658[0n]
Pieris rapae|OXB1327-15|Rvcoll_15-M599||France|658[0n]
Pieris rapae|WMB4913-14|RVcoll.14-I460||Italy|658[0n]
Pieris rapae|EULEP5721-17|RVcoll16L192||Italy|658[0n]
Pieris rapae|WMB141-11|RVcoll.11-D162|Marettimo|Italy|658[0n]
Pieris rapae|EZRMN183-08|RVcoll.08-M412||Romania|658[0n]
Pieris rapae|WMB573-11|RVcoll.11-H799|Vulcano|Italy|658[0n]
Pieris rapae|OXB341-15|RVcoll.14-N055||Italy|658[0n]
Pieris rapae|EZSPM063-09|RVcoll.08-P292||Spain|646[0n]
Pieris rapae|OXB1237-15|Rvcoll_13-T633|Sardinia|Italy|658[0n]
Pieris rapae|WMB5262-14|RVcoll.14-A378||Italy|658[0n]
Pieris rapae|WMB1986-13|RVcoll.11-I295|Pianosa|Italy|658[0n]
Pieris rapae|WMB1179-13|RVcoll.11-E848|Corsica|France|658[0n]
Pieris rapae|LEFIA1301-10|MM06585||Finland|658[0n]
Pieris rapae|LEATD147-13|TLMF Lep 12794||Italy|658[0n]
Pieris rapae|OXB572-15|14-N961||Italy|658[0n]
Pieris rapae|WMB2864-14|RVcoll.09-X905|Capri|Italy|658[0n]
Pieris rapae|GBLAA1261-15|BC ZSM Lep 87147||Germany|658[0n]
Pieris rapae|EULEP4937-16|RVcoll16I043||Poland|658[0n]
Pieris rapae|BIBSA1005-15|15-C094||Italy|658[0n]
Pieris rapae|WMB3037-14|RVcoll.13-S564|San Domino|Italy|658[0n]
Pieris rapae|ABOLC189-16|TLMF Lep 20222||Austria|658[0n]
Pieris rapae|OXB1070-15|15-A603||Italy|658[0n]
Pieris rapae|EULEP1309-15|RVcoll.14-F765||Greece|658[0n]
Pieris rapae|WMB4459-14|RVcoll.14-D327||Spain|658[0n]
Pieris rapae|EZSPC747-10|RVcoll.08-L052||Spain|658[0n]
Pieris rapae|EZSPN443-09|RVcoll.08-H629||Spain|658[0n]
Pieris rapae|LOWA161-06|2005-LOWA-161||Russia|658[0n]
Pieris rapae|BIBSA1319-15|12-O470|Sardinia|Italy|658[0n]
Pieris rapae|WMB1573-13|RVcoll.12-O518|Sardinia|Italy|658[0n]
Pieris rapae|WMB2467-13|RVcoll.11-H510|Capri|Italy|658[0n]
Pieris rapae|BIBSA1358-15|15-M862||Italy|658[0n]
Pieris rapae|OXB1547-16|OXB-TGS-1265||France|658[1n]
Pieris rapae|OXB1360-15|Rvcoll_15-M714||France|658[0n]
Pieris rapae|OXB1340-15|Rvcoll_15-M688||France|658[0n]
Pieris rapae|WMB3646-14|RVcoll.11-I602||France|658[0n]
Pieris rapae|WMB3230-14|RVcoll.08-J875||Spain|658[0n]
Pieris rapae|GBLAC982-13|BC ZSM Lep 76513||Germany|658[0n]
Pieris rapae|WMB2783-13|RVcoll.11-I299|Pianosa|Italy|658[0n]
Pieris rapae|OXB1456-16|OXB-TGS-1230||United Kingdom|658[0n]
Pieris rapae|BIBSA150-15|RVcoll.14-I162||Italy|658[0n]
Pieris rapae|WMB2300-13|RVcoll.11-E108||Malta|658[0n]
Pieris rapae|WMB1491-13|RVcoll.12-O019|Giglio|Italy|658[0n]
Pieris rapae|WMB2762-13|RVcoll.11-H800|Vulcano|Italy|658[0n]
Pieris rapae|WMB1143-13|RVcoll.11-E139|Gozo|Malta|658[0n]
Pieris rapae|WMB1115-13|RVcoll.11-D154|Pantelleria|Italy|658[0n]
Pieris rapae|WMB133-11|RVcoll.11-D137|Pantelleria|Italy|658[0n]
Pieris rapae|WMB4405-14|RVcoll.14-B436||Portugal|658[0n]
Pieris rapae|EULEP4460-16|RVcoll15G130||France|658[0n]
Pieris rapae|OXB1286-15|Rvcoll_15-M149||France|658[0n]
Pieris rapae|EZSPM344-09|RVcoll.08-R005||Spain|658[1n]
Pieris rapae|GWORK293-09|BC ZSM Lep 21528||Germany|658[2n]
Pieris rapae|OXB365-15|RVcoll.14-N079||Italy|658[0n]
Pieris rapae|OXB1529-16|OXB-TGS-1247||United Kingdom|658[0n]
Pieris rapae|OXB1524-16|OXB-TGS-1242||United Kingdom|658[0n]
Pieris rapae|LEFIJ4747-16|ZMBSU-02157||Belarus|658[0n]
Pieris rapae|EULEP5011-16|RVcoll16I892||Slovakia|658[0n]
Pieris rapae|WMB4415-14|RVcoll.14-B459||Portugal|629[0n]
Pieris rapae|WMB3400-14|RVcoll.09-V747||Spain|658[0n]
Pieris rapae|WMB3439-14|RVcoll.09-X554||Spain|658[0n]
Pieris rapae|WMB3294-14|RVcoll.08-M971||France|658[0n]
Pieris rapae|WMB2817-13|RVcoll.12-O020|Giglio|Italy|658[0n]
Pieris rapae|WMB1987-13|RVcoll.11-I296|Pianosa|Italy|658[0n]
Pieris rapae|WMB2334-13|RVcoll.12-M266||Italy|658[0n]
Pieris rapae|WMB2427-13|RVcoll.10-C790|Ischia|Italy|658[0n]
Pieris rapae|EULEP1218-15|RVcoll.14-F518||Greece|658[0n]
Pieris rapae|BIBSA1787-16|16-A522||Italy|658[0n]
Pieris rapae|OXB1112-15|15-A645||Italy|658[0n]
Pieris rapae|OXB748-15|13-U444||Italy|658[0n]
Pieris rapae|OXB1378-15|Rvcoll_15-M742||France|658[0n]
Pieris rapae|WMB2896-14|RVcoll.11-E364|Sardinia|Italy|646[0n]
Pieris rapae|EZSPN377-09|RVcoll.08-H414||Spain|658[1n]
Pieris rapae|LEFIJ503-10|MM17128||Finland|658[0n]
Pieris rapae|LON569-08|NHMO-08221||Norway|657[0n]
Pieris rapae|GWORA2446-09|BC ZSM Lep 30658||Germany|658[0n]
[illegible]

Pieris rapae|LEFIJ503-10|MM17128||Finland|658[0n]
Pieris rapae|LON569-08|NHMO-08221||Norway|657[0n]
Pieris rapae|GWORA2446-09|BC ZSM Lep 30658||Germany|658[0n]
Pieris rapae|WMB4145-14|RVcoll.13-S662||Italy|614[0n]
Pieris rapae|WMB3473-14|RVcoll.10-A667||France|658[0n]
Pieris rapae|EZSPN760-09|RVcoll.08-L239||Spain|658[0n]
Pieris rapae|EZSPM982-12|Rvcoll.12-M745||Spain|658[0n]
Pieris rapae|EZSPM992-12|Rvcoll.12-L878||Spain|658[0n]
Pieris rapae|EZSPN581-09|RVcoll.08-J138||Portugal|658[0n]
Pieris rapae|EZSPN603-09|RVcoll.08-J306||Spain|658[0n]
Pieris rapae|EZSPM235-09|RVcoll.08-R435||Spain|658[0n]
Pieris rapae|EZSPC884-10|RVcoll.09-T147||Spain|658[0n]
Pieris rapae|EZSPC890-10|RVcoll.09-V394||Spain|658[0n]
Pieris rapae|EZSPM885-12|Rvcoll.12-M603||Spain|658[0n]
Pieris rapae|EZSPM897-12|Rvcoll.12-M619||Spain|658[0n]
Pieris rapae|EZSPM919-12|Rvcoll.12-M641||Spain|658[0n]
Pieris rapae|WMB176-11|RVcoll.11-D439|Sicily|Italy|658[0n]
Pieris rapae|WMB3029-14|RVcoll.13-S404|Ibiza|Spain|658[0n]
Pieris rapae|WMB3681-14|RVcoll.11-J222||Spain|658[0n]
Pieris rapae|WMB3170-14|RVcoll.08-H518||Spain|658[0n]
Pieris rapae|WMB3575-14|RVcoll.11-D995||Spain|658[0n]
Pieris rapae|WMB3672-14|RVcoll.11-I975||France|658[0n]
Pieris rapae|WMB2242-13|RVcoll.12-R377|Elba|Italy|658[0n]
Pieris rapae|WMB4516-14|RVcoll.14-E207||Spain|658[0n]
Pieris rapae|WMB4385-14|RVcoll.14-B280||Portugal|658[0n]
Pieris rapae|WMB494-11|RVcoll.11-H541|Sicily|Italy|658[0n]
Pieris rapae|EULEP5067-16|RVcoll16J147||Czech Republic|658[0n]
Pieris rapae|EULEP643-15|RVcoll.14-F669||Greece|658[0n]
Pieris rapae|OXB602-15|14-N991||Italy|658[0n]
Pieris rapae|OXB1139-15|15-A672||Italy|658[0n]
Pieris rapae|OXB688-15|12-M383|Sicily W|Italy|658[0n]
Pieris rapae|EZSPM894-12|Rvcoll.12-M616||Spain|637[0n]
Pieris rapae|EZSPM047-09|RVcoll.08-P264||Spain|646[0n]
Pieris rapae|EZSPM136-09|RVcoll.08-P646||Spain|646[0n]
Pieris rapae|EZSPM742-12|Rvcoll. 12-L549||Portugal|621[0n]
Pieris rapae|EZSPN315-09|RVcoll.08-H173||Spain|646[0n]
Pieris rapae|EZSPN693-09|RVcoll.08-L025||Spain|646[0n]
Pieris rapae|EZSPN686-09|RVcoll.08-L014||Spain|646[0n]
Pieris rapae|EZROM476-08|RV-07-D209|Genit. examined|Romania|658[0n]
Pieris rapae|EZSPM977-12|Rvcoll.12-M737||Spain|658[0n]
Pieris rapae|EZSPC664-09|RVcoll.08-J377||Spain|658[0n]
Pieris rapae|BIBSA1827-17|RVcoll16C701||Italy|658[0n]
Pieris rapae|BIBSA452-15|RVcoll.14-I120||Italy|658[0n]
Pieris rapae|EULEP4454-16|RVcoll10B450||Croatia|658[0n]
Pieris rapae|OXB896-15|15-A540||Italy|658[0n]
Pieris rapae|WMB4603-14|RVcoll.LD-1398||Italy|628[0n]
Pieris rapae|WMB4767-14|RVcoll. 14-I314||Italy|630[0n]
Pieris rapae|EZROM477-08|RV-07-C973|Genit. examined|Romania|658[0n]
Pieris rapae|GBLAD930-14|BC ZSM Lep 84536||Germany|658[0n]
Pieris rapae|BIBSA335-15|RVcoll.14-I003||Italy|658[0n]
Pieris rapae|WMB5968-17|RVcoll14N712|Stromboli|Italy|658[0n]
Pieris rapae|WMB1065-13|RVcoll.10-C687|Stromboli|Italy|658[0n]
Pieris rapae|WMB1555-13|RVcoll.12-O396|San Pietro|Italy|658[0n]
Pieris rapae|EZROM474-08|RV-06-K650||Romania|658[0n]
Pieris rapae|EZSPN749-09|RVcoll.08-L201||Spain|658[0n]
Pieris rapae|EZSPC667-09|RVcoll.08-L474||Spain|658[0n]
Pieris rapae|EZRMN181-08|RVcoll.08-M241||Romania|658[0n]
Pieris rapae|EZRMN182-08|RVcoll.08-M336||Romania|658[0n]
Pieris rapae|EZRMN184-08|RVcoll.08-M636||Romania|658[0n]
Pieris rapae|EZSPN088-09|RVcoll.06-A017||Spain|658[0n]
Pieris rapae|EZSPM213-09|RVcoll.08-R334||Spain|658[0n]
Pieris rapae|WMB2906-14|RVcoll.11-E816|Corsica|France|658[0n]
Pieris rapae|WMB2914-14|RVcoll.11-H698|Levanzo|Italy|658[0n]
Pieris rapae|WMB3023-14|RVcoll.13-S368|Ibiza|Spain|658[0n]
Pieris rapae|WMB3031-14|RVcoll.13-S537|San Domino|Italy|658[0n]
Pieris rapae|WMB3048-14|RVcoll.13-S632|Capraia|Italy|658[0n]
Pieris rapae|WMB3222-14|RVcoll.08-J741||Spain|658[0n]
Pieris rapae|WMB3562-14|RVcoll.11-D926||Spain|658[0n]
Pieris rapae|WMB3587-14|RVcoll.11-E097||Spain|658[0n]
Pieris rapae|WMB1130-13|RVcoll.11-E105||Malta|658[0n]
Pieris rapae|WMB1142-13|RVcoll.11-E138|Gozo|Malta|658[0n]
Pieris rapae|WMB2721-13|RVcoll.10-C536|Ischia|Italy|658[0n]
Pieris rapae|WMB2773-13|RVcoll.11-H880|Salina|Italy|658[0n]
Pieris rapae|WMB1489-13|RVcoll.12-O017|Giglio|Italy|658[0n]
Pieris rapae|WMB1490-13|RVcoll.12-O018|Giglio|Italy|658[0n]
Pieris rapae|WMB1503-13|RVcoll.12-O062||Italy|658[0n]
Pieris rapae|WMB1275-13|RVcoll.12-M306|Lipari|Italy|658[0n]
Pieris rapae|WMB1803-13|RVcoll.12-Q339||France|658[0n]
Pieris rapae|WMB2084-13|RVcoll.12-R019|Sicily|Italy|658[0n]
Pieris rapae|WMB1750-13|RVcoll.12-P965|Levant|France|658[0n]
Pieris rapae|WMB1751-13|RVcoll.12-P966|Levant|France|658[0n]
Pieris rapae|WMB1889-13|RVcoll.11-J759||Italy|658[0n]
Pieris rapae|WMB1945-13|RVcoll.11-H882|Salina|Italy|658[0n]
Pieris rapae|WMB1988-13|RVcoll.11-I297|Pianosa|Italy|658[0n]
Pieris rapae|WMB1989-13|RVcoll.11-I298|Pianosa|Italy|658[0n]
Pieris rapae|WMB2177-13|RVcoll.12-R347|Elba|Italy|658[0n]
Pieris rapae|WMB2289-13|RVcoll.10-C600||Malta|658[0n]
Pieris rapae|WMB2305-13|RVcoll.11-E140|Gozo|Malta|658[0n]
Pieris rapae|WMB2306-13|RVcoll.11-E141|Gozo|Malta|658[0n]
Pieris rapae|WMB2313-13|RVcoll.11-H619|Lampedusa|Italy|658[0n]
Pieris rapae|WMB2357-13|RVcoll.12-Q902||Italy|658[0n]
Pieris rapae|WMB5967-17|RVcoll14N711|Stromboli|Italy|658[0n]
Pieris rapae|WMB2635-13|RVcoll.12-O102|Corsica|France|658[0n]
Pieris rapae|WMB3911-14|RVcoll.12-P341||France|658[0n]
Pieris rapae|WMB3935-14|RVcoll.12-P549||France|658[0n]
Pieris rapae|WMB3956-14|RVcoll.12-P766||France|658[0n]
Pieris rapae|WMB4010-14|RVcoll.12-Q813||Italy|658[0n]
Pieris rapae|WMB4302-14|RVcoll.14-A401||Italy|658[0n]
Pieris rapae|WMB321-11|RVcoll.11-E574||Italy|658[0n]
Pieris rapae|WMB340-11|RVcoll.11-E683||France|658[0n]
Pieris rapae|WMB365-11|RVcoll.11-E972||France|658[0n]
Pieris rapae|WMB6608-18|RVcoll15D786||Spain|658[0n]
Pieris rapae|WMB479-11|RVcoll.11-H504|Capri|Italy|658[0n]

Pieris rapae|WMB365-11|RVcoll.11-E972||France|658[0n]
Pieris rapae|WMB6608-18|RVcoll15D786||Spain|658[0n]
Pieris rapae|WMB479-11|RVcoll.11-H504|Capri|Italy|658[0n]
Pieris rapae|WMB540-11|RVcoll.11-H703|Levanzo|Italy|658[0n]
Pieris rapae|WMB5080-14|RVcoll. 14-L211||Italy|658[0n]
Pieris rapae|WMB5087-14|RVcoll. 14-L218||Italy|658[0n]
Pieris rapae|WMB586-11|RVcoll.11-H883|Salina|Italy|658[0n]
Pieris rapae|WMB612-11|RVcoll.11-I023|Sicily|Italy|658[0n]
Pieris rapae|WMB4982-14|RVcoll. 14-I529||Italy|658[0n]
Pieris rapae|WMB5207-14|RVcoll.13-T940||Italy|658[0n]
Pieris rapae|WMB5499-14|RVcoll.14-J405|Giannutri|Italy|658[0n]
Pieris rapae|EULEP5447-17|RVcoll11I294|Pianosa|Italy|658[0n]
Pieris rapae|EULEP5891-18|RVcoll15O597||Spain|658[0n]
Pieris rapae|EULEP1202-15|RVcoll.14-F496||Greece|658[0n]
Pieris rapae|EULEP1461-15|RVcoll.14-G213||Greece|658[0n]
Pieris rapae|EULEP1527-15|RVcoll.14-G474||Greece|658[0n]
Pieris rapae|EULEP2246-15|RVcoll.14-N428||Ukraine|658[0n]
Pieris rapae|EULEP2355-15|RVcoll.14-V044||Ukraine|658[0n]
Pieris rapae|BIBSA1254-15|15-F848||France|658[0n]
Pieris rapae|BIBSA910-15|16-A001||France|658[0n]
Pieris rapae|BIBSA959-15|15-C304||Italy|658[0n]
Pieris rapae|BIBSA1615-16|15-C388||Italy|658[0n]
Pieris rapae|BIBSA1068-15|15-H920||Italy|658[0n]
Pieris rapae|BIBSA1309-15|12-M357|Sicily|Italy|658[0n]
Pieris rapae|BIBSA115-15|RVcoll.14-D548||Italy|658[0n]
Pieris rapae|BIBSA1385-15|15-M981||Italy|658[0n]
Pieris rapae|BIBSA1401-15|15-N006||Italy|658[0n]
Pieris rapae|BIBSA686-15|RVcoll. 12-Q812||Italy|658[0n]
Pieris rapae|BIBSA1736-16|LD-2805||Italy|658[0n]
Pieris rapae|BIBSA1852-17|RVcoll16C733||Italy|658[0n]
Pieris rapae|GBLAF377-14|BC ZSM Lep 82083||Germany|658[0n]
Pieris rapae|BIBSA024-14|LEP-SS-00024||Italy|658[0n]
Pieris rapae|BIBSA1616-16|LEPSS-00390||Italy|658[0n]
Pieris rapae|BIBSA1829-17|RVcoll16C704||Italy|658[0n]
Pieris rapae|GBLAB312-13|BC ZSM Lep 72613||Germany|658[0n]
Pieris rapae|LEFIJ4546-16|ZMBSU-02127||Belarus|658[0n]
Pieris rapae|LEASS860-17|KLM Lep 08365||Austria|658[0n]
Pieris rapae|OXB1454-16|OXB-TGS-1228||United Kingdom|658[0n]
Pieris rapae|OXB1523-16|OXB-TGS-1241||United Kingdom|658[0n]
Pieris rapae|OXB995-15|15-A945||Italy|658[0n]
Pieris rapae|OXB1548-16|OXB-TGS-1266||Andorra|658[0n]
Pieris rapae|OXB1308-15|Rvcoll_15-M572||France|658[0n]
Pieris rapae|OXB1141-15|Rvcoll_15-N095||Italy|658[0n]
Pieris rapae|WMB2437-13|RVcoll.11-D146|Pantelleria|Italy|658[0n]
Pieris rapae|OXB1177-15|Rvcoll_16-A064||Italy|658[0n]
Pieris rapae|OXB1192-15|Rvcoll_15-M605||France|658[0n]
Pieris rapae|OXB1198-15|Rvcoll_15-M614||France|658[0n]
Pieris rapae|OXB1205-15|Rvcoll_15-M621||France|658[0n]
Pieris rapae|EULEP5642-17|RVcoll15D998|Gorgona|Italy|658[0n]
Pieris rapae|OXB1220-15|Rvcoll_15-M646||France|658[0n]
Pieris rapae|ABOLD663-17|TLMF Lep 21739||Austria|658[0n]
Pieris rapae|EULEP1590-15|RVcoll.14-G624||Greece|618[0n]
Pieris rapae|OXB859-15|15-A503||Italy|658[0n]
Pieris rapae|EZSPM744-12|Rvcoll. 12-L612||Portugal|621[0n]
Pieris rapae|EZSPM741-12|Rvcoll. 12-L595||Portugal|621[0n]
Pieris rapae|BIBSA921-15|16-A012||France|628[0n]
Pieris rapae|EZSPM028-09|RVcoll.08-P225||Spain|646[0n]
Pieris rapae|WMB3047-14|RVcoll.13-S624|Capraia|Italy|646[0n]
Pieris rapae|OXB267-15|OXB-TGS-950||United Kingdom|634[0n]
Pieris rapae|BIBSA1535-16|10-C538||Italy|618[0n]
Pieris rapae|EZSPM886-12|Rvcoll.12-M604||Spain|630[0n]
Pieris rapae|EZSPN299-09|RVcoll.08-H103||Spain|608[0n]
Pieris rapae|EZSPN702-09|RVcoll.08-L045||Spain|646[0n]
Pieris rapae|EZSPC603-09|RVcoll.08-L455||Spain|658[1n]
Pieris rapae|LEFIJ504-10|MM17129||Finland|634[0n]
Pieris rapae|GWORU081-10|BC ZSM Lep 30342||Italy|658[0n]
Pieris rapae|LENOA1341-11|LN-BD1341||France|658[0n]
Pieris rapae|LEATJ1308-16|TLMF Lep 19631||Austria|658[0n]
Pieris rapae|LEATG536-14|TLMF Lep 14323||Italy|658[0n]
Pieris rapae|ABOLD085-16|TLMF Lep 21163||Austria|658[0n]
Pieris rapae|PHLAI544-13|TLMF Lep 09106||Austria|658[0n]
Pieris rapae|LEATJ1307-16|TLMF Lep 19630||Austria|658[0n]
Pieris rapae|OXB1390-15|Rvcoll_15-M755||France|658[0n]
Pieris rapae|WMB1929-13|RVcoll.11-H804|Vulcano|Italy|654[0n]
Pieris rapae|WMB554-11|RVcoll.11-H747|Sicily|Italy|658[0n]
Pieris rapae|WMB5471-14|RVcoll.14-N683|Alicudi|Italy|658[0n]
Pieris rapae|BIBSA1134-15|15-L945||Italy|658[0n]
Pieris rapae|BIBSA1708-16|15-N142||Italy|658[0n]
Pieris rapae|OXB668-15|07-D807||Italy|658[0n]
Pieris rapae|WMB4631-14|RVcoll.LD-2386|Ustica|Italy|646[0n]
Pieris rapae|WMB4228-14|RVcoll.13-T772||Italy|658[0n]
Pieris rapae|WMB3847-14|RVcoll.10-C718||Italy|658[0n]
Pieris rapae|WMB2763-13|RVcoll.11-H803|Vulcano|Italy|658[0n]
Pieris rapae|WMB2720-13|RVcoll.10-C535|Ischia|Italy|658[0n]
Pieris rapae|WMB125-11|RVcoll.11-D020|Capri|Italy|658[0n]
Pieris rapae|WMB190-11|RVcoll.11-D505||Italy|658[0n]
Pieris rapae|EULEP2432-15|RVcoll.14-V229||Belgium|658[0n]
Pieris rapae|OXB1528-16|OXB-TGS-1246||United Kingdom|658[0n]
Pieris rapae|WMB2740-13|RVcoll.10-C602||Malta|630[0n]
Pieris rapae|EULEP733-15|RVcoll.12-N833|Lesvos|Greece|658[0n]
Pieris rapae|BIBSA023-14|LEP-SS-00023||Italy|658[0n]
Pieris rapae|WMB1552-13|RVcoll.12-O373|Sardinia|Italy|658[0n]
Pieris rapae|WMB2661-13|RVcoll.10-A687||France|658[0n]
Pieris rapae|EZROM591-08|RV-07-C356||Romania|658[0n]
Pieris rapae|OXB1455-16|OXB-TGS-1229||United Kingdom|658[0n]
Pieris rapae|OXB1230-15|Rvcoll_15-M665||France|658[0n]
Pieris rapae|EULEP592-15|RVcoll.10-A741||Estonia|658[0n]
Pieris rapae|WMB251-11|RVcoll.09-V253||France|658[0n]
Pieris rapae|OXB761-15|13-U502||Italy|658[0n]
Pieris rapae|EZROM748-08|RV-06-H864||Spain|658[0n]
Pieris rapae|EULEP692-15|RVcoll.12-M100|Crete|Greece|658[0n]
Pieris rapae|EZROM475-08|RV-07-D108|Genit. examined|Romania|658[0n]
[illegible]

Pieris rapae|EZROM748-08|RV-06-H864||Spain|658[0n]
Pieris rapae|EULEP692-15|RVcoll.12-M100|Crete|Greece|658[0n]
Pieris rapae|EZROM475-08|RV-07-D108|Genit. examined|Romania|658[0n]
Pieris rapae|WMB3452-14|RVcoll.10-A455||France|658[0n]
Pieris rapae|WMB2806-13|RVcoll.12-M307|Lipari|Italy|658[0n]
Pieris rapae|WMB2825-13|RVcoll.12-O377|Sardinia|Italy|658[0n]
Pieris rapae|WMB2596-13|RVcoll.12-R348|Elba|Italy|658[0n]
Pieris rapae|WMB4591-14|RVcoll.LD-376|Corsica|France|658[0n]
Pieris rapae|WMB4690-14|RVcoll.LD-2706|Ponza|Italy|658[0n]
Pieris rapae|WMB510-11|RVcoll.11-H597|Sicily|Italy|658[0n]
Pieris rapae|WMB5321-14|RVcoll.14-I981|La Maddalena|Italy|658[0n]
Pieris rapae|WMB5063-14|RVcoll. 14-L194||Italy|658[0n]
Pieris rapae|EULEP4456-16|RVcoll15G732||Italy|658[0n]
Pieris rapae|EULEP4457-16|RVcoll15G892||Switzerland|658[0n]
Pieris rapae|EULEP4725-16|RVcoll16G385||Denmark|658[0n]
Pieris rapae|EULEP2165-15|RVcoll.14-K085||Switzerland|658[0n]
Pieris rapae|BIBSA1854-17|RVcoll16C736||Italy|658[0n]
Pieris rapae|BIBSA418-15|RVcoll.14-I086||Italy|658[0n]
Pieris rapae|GBLAA1293-15|BC ZSM Lep 87179||Germany|658[0n]
Pieris rapae|OXB839-15|14-U802||Italy|658[0n]
Pieris rapae|OXB679-15|11-Y106|Sicily W|Italy|658[0n]
Pieris rapae|OXB1142-15|Rvcoll_15-N096||Italy|658[0n]
Pieris rapae|ABOLD580-17|TLMF Lep 21656||Austria|658[0n]
Pieris rapae|EZSPC608-09|RVcoll.06-G428|Genit. examined|Spain|658[1n]
Pieris rapae|OXB677-15|11-D376|Sicily E|Italy|620[0n]
Pieris rapae|EZSPC663-09|RVcoll.08-J362||Spain|647[0n]
Pieris rapae|WMB3898-14|RVcoll.12-P176||France|658[0n]
Pieris rapae|WMB539-11|RVcoll.11-H699|Levanzo|Italy|658[0n]
Pieris rapae|GBLAA1396-15|BC ZSM Lep 87092||Germany|658[0n]
Pieris rapae|OXB261-15|OXB-TGS-868||United Kingdom|658[0n]
Pieris rapae|WMB523-11|RVcoll.11-H617|Lampedusa|Italy|658[0n]
Pieris rapae|WMB325-11|RVcoll.11-E612||Italy|658[0n]
Pieris rapae|WMB4711-14|RVcoll.LD-2918|Asinara|Italy|658[0n]
Pieris rapae|WMB1908-13|RVcoll.11-H618|Lampedusa|Italy|658[0n]
Pieris rapae|WMB099-11|RVcoll.10-B606||France|658[0n]
Pieris rapae|EULEP916-15|RVcoll.14-C782||Bulgaria|658[0n]
Pieris rapae|EULEP4455-16|RVcoll15G036||France|658[0n]
Pieris rapae|EZSPC885-10|RVcoll.09-T148||Spain|658[0n]
Pieris rapae|BIBSA1579-16|12-M337||Italy|630[0n]
Pieris rapae|WMB1089-13|RVcoll.10-C754|Capraia|Italy|631[0n]
Pieris rapae|EZROM473-08|RV-06-K542|Genit. examined|Romania|652[0n]
Pieris rapae|CGUKB956-09|UKLB21G05||United Kingdom|627[0n]
Pieris rapae|LEATA402-13|TLMF Lep 10009||Austria|658[0n]
Pieris rapae|EULEP2007-15|RVcoll.14-I796||Poland|658[0n]
Pieris rapae|LEATF470-14|TLMF Lep 13782||Austria|658[0n]
Euchloe tagis|BIBSA884-15|LD-3496||Italy|658[0n]
Euchloe tagis|BIBSA883-15|LD-3495||Italy|658[0n]
Euchloe tagis|OXB938-15|15-A582||Italy|658[0n]
Euchloe tagis|EULEP801-15|RVcoll.13-T850||Italy|658[0n]
Euchloe tagis|WMB5194-14|RVcoll.13-T855||Italy|658[0n]
Euchloe tagis|WMB4239-14|RVcoll.13-T843||Italy|658[0n]
Euchloe tagis|GWOSU979-11|BC Back 0029||Italy|658[0n]
Euchloe tagis|EULEP2847-15|RVcoll.14-N931||France|658[0n]
Euchloe tagis|EULEP2846-15|RVcoll.14-N930||France|653[0n]
Euchloe tagis|EZSPC1007-10|RVcoll.08-H429||Spain|658[0n]
Euchloe tagis|GWOSU975-11|BC Back 0025||Spain|658[0n]
Euchloe tagis|EZSPN532-09|RVcoll.08-J008||Spain|658[0n]
Euchloe tagis|GWOSU974-11|BC Back 0024||Spain|658[0n]
Euchloe tagis|GWOSU976-11|BC Back 0026||Spain|658[0n]
Euchloe tagis|GWOSU977-11|BC Back 0027||Spain|658[0n]
Euchloe tagis|EZSPN343-09|RVcoll.08-H306||Spain|658[0n]
Euchloe tagis|GWOSU982-11|BC Back 0032||Spain|658[0n]
Euchloe tagis|EZSPC1039-10|RVcoll.08-J316||Spain|658[0n]
Euchloe tagis|EZSPC1038-10|RVcoll.08-J315||Spain|658[0n]
Euchloe tagis|EZSPM692-12|Rvcoll. 12-L047||Spain|658[0n]
Euchloe tagis|GWOSU980-11|BC Back 0030||Spain|658[0n]
Euchloe tagis|EZSPC1022-10|RVcoll.08-J003||Spain|658[0n]
Euchloe tagis|EZSPM691-12|Rvcoll. 12-L046||Spain|658[0n]
Euchloe tagis|EZSPC1012-10|RVcoll.08-H542||Spain|658[0n]
Euchloe tagis|EZSPN1106-11|Rvcoll.10-B498||Spain|658[0n]
Euchloe tagis|WMB4045-14|RVcoll.13-S274||Spain|658[0n]
Euchloe tagis|EZSPN607-09|RVcoll.08-J317||Spain|658[0n]
Euchloe tagis|EZSPN380-09|RVcoll.08-H427||Spain|658[0n]
Euchloe tagis|EZSPN416-09|RVcoll.08-H541||Spain|658[0n]
Euchloe tagis|PHLAH282-12|TLMF Lep 07721||Spain|658[0n]
Zegris eupheme|EZSPC1014-10|RVcoll.08-H560||Spain|658[0n]
Zegris eupheme|EZSPC1013-10|RVcoll.08-H544||Spain|658[0n]
Zegris eupheme|EZSPN535-09|RVcoll.08-J012||Spain|658[0n]
Zegris eupheme|EZSPN533-09|RVcoll.08-J009||Spain|658[0n]
Zegris eupheme|EZSPN349-09|RVcoll.08-H318||Spain|658[0n]
Zegris eupheme|EZSPN419-09|RVcoll.08-H559||Spain|658[0n]
Zegris eupheme|EZSPN417-09|RVcoll.08-H543||Spain|658[0n]
Zegris eupheme|EZSPM319-09|RVcoll.08-J885||Spain|658[0n]
Zegris eupheme|EZSPC1001-10|RVcoll.08-H242||Spain|658[0n]
Zegris eupheme|EZSPC1000-10|RVcoll.08-H238||Spain|658[0n]
Zegris eupheme|EZSPC1002-10|RVcoll.08-H243||Spain|658[0n]
Zegris eupheme|EZSPN329-09|RVcoll.08-H240||Spain|658[0n]
Zegris eupheme|GWOSU952-11|BC Back 0002||Spain|658[0n]
Zegris eupheme|EULEP2042-15|RVcoll.14-J521||Ukraine|658[0n]
Zegris eupheme|EULEP2041-15|RVcoll.14-J520||Ukraine|658[0n]
Zegris eupheme|EULEP466-14|KN00828||Russia|658[0n]
Zegris eupheme|EULEP465-14|KN00827||Russia|658[0n]
Zegris eupheme|GWOSU955-11|BC Back 0005||Russia|658[0n]
Euchloe belemia|EZSPC1311-10|RVcoll.130209KL19||Spain|658[0n]
Euchloe belemia|EZSPM607-12|RVcoll.11-D641||Spain|658[0n]
Euchloe belemia|EZSPM624-12|RVcoll.11-D924||Spain|658[0n]
Euchloe belemia|EZSPM627-12|RVcoll.11-D960||Spain|658[0n]
Euchloe belemia|EZSPM315-09|RVcoll.08-J871||Spain|658[0n]
Euchloe belemia|EZSPM316-09|RVcoll.08-J876||Spain|658[0n]
Euchloe belemia|EZSPM688-12|Rvcoll. 12-L593||Portugal|621[0n]
Euchloe belemia|EZSPM687-12|Rvcoll. 12-L592||Portugal|626[0n]
Euchloe belemia|EZSPM626-12|RVcoll.11-D959||Spain|632[0n]
Euchloe belemia|EZSPM625-12|RVcoll.11-D925||Spain|658[0n]

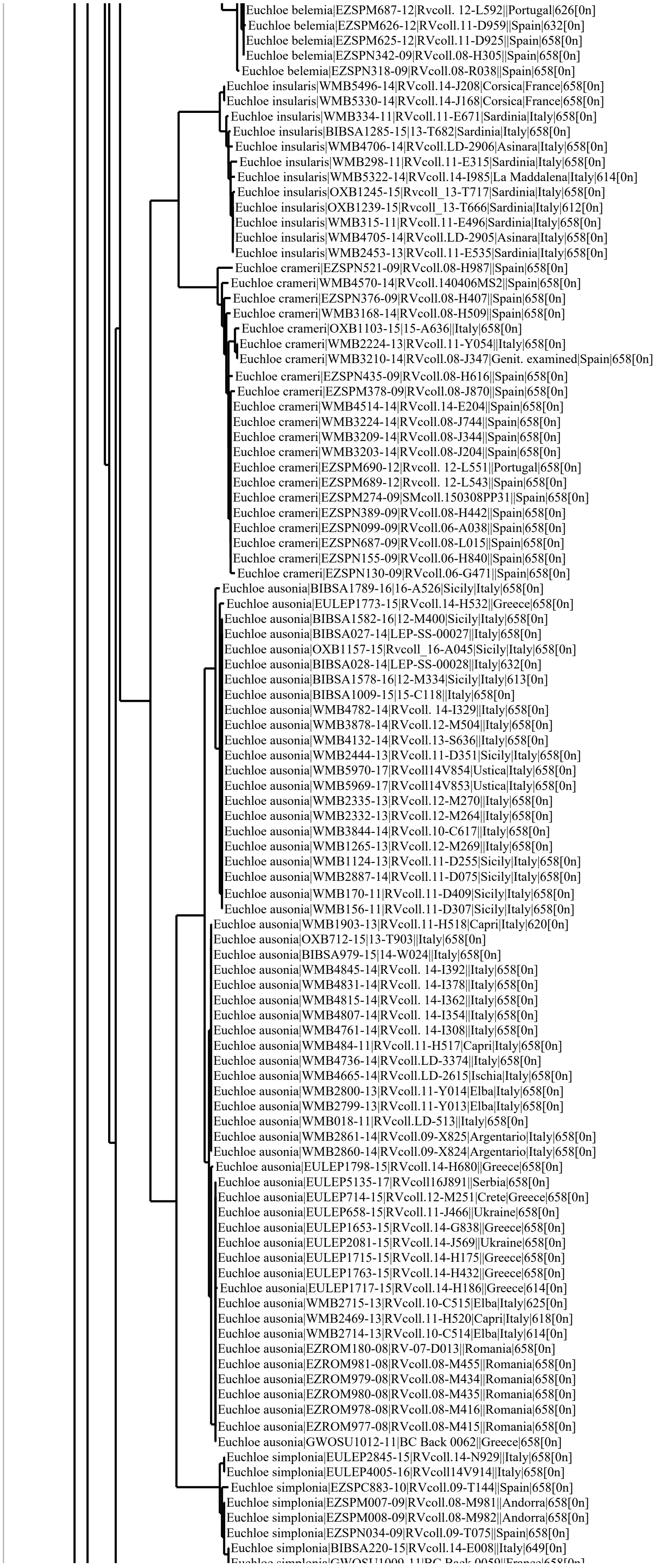
Euchloe belemia|EZSPM687-12|Rvcoll. 12-L592||Portugal|626[0n]
Euchloe belemia|EZSPM626-12|RVcoll.11-D959||Spain|632[0n]
Euchloe belemia|EZSPM625-12|RVcoll.11-D925||Spain|658[0n]
Euchloe belemia|EZSPN342-09|RVcoll.08-H305||Spain|658[0n]
Euchloe belemia|EZSPN318-09|RVcoll.08-R038||Spain|658[0n]
Euchloe insularis|WMB5496-14|RVcoll.14-J208|Corsica|France|658[0n]
Euchloe insularis|WMB5330-14|RVcoll.14-J168|Corsica|France|658[0n]
Euchloe insularis|WMB334-11|RVcoll.11-E671|Sardinia|Italy|658[0n]
Euchloe insularis|BIBSA1285-15|13-T682|Sardinia|Italy|658[0n]
Euchloe insularis|WMB4706-14|RVcoll.LD-2906|Asinara|Italy|658[0n]
Euchloe insularis|WMB298-11|RVcoll.11-E315|Sardinia|Italy|658[0n]
Euchloe insularis|WMB5322-14|RVcoll.14-I985|La Maddalena|Italy|614[0n]
Euchloe insularis|OXB1245-15|Rvcoll_13-T717|Sardinia|Italy|658[0n]
Euchloe insularis|OXB1239-15|Rvcoll_13-T666|Sardinia|Italy|612[0n]
Euchloe insularis|WMB315-11|RVcoll.11-E496|Sardinia|Italy|658[0n]
Euchloe insularis|WMB4705-14|RVcoll.LD-2905|Asinara|Italy|658[0n]
Euchloe insularis|WMB2453-13|RVcoll.11-E535|Sardinia|Italy|658[0n]
Euchloe crameri|EZSPN521-09|RVcoll.08-H987||Spain|658[0n]
Euchloe crameri|WMB4570-14|RVcoll.140406MS2||Spain|658[0n]
Euchloe crameri|EZSPN376-09|RVcoll.08-H407||Spain|658[0n]
Euchloe crameri|WMB3168-14|RVcoll.08-H509||Spain|658[0n]
Euchloe crameri|OXB1103-15|15-A636||Italy|658[0n]
Euchloe crameri|WMB2224-13|RVcoll.11-Y054||Italy|658[0n]
Euchloe crameri|WMB3210-14|RVcoll.08-J347|Genit. examined|Spain|658[0n]
Euchloe crameri|EZSPN435-09|RVcoll.08-H616||Spain|658[0n]
Euchloe crameri|EZSPM378-09|RVcoll.08-J870||Spain|658[0n]
Euchloe crameri|WMB4514-14|RVcoll.14-E204||Spain|658[0n]
Euchloe crameri|WMB3224-14|RVcoll.08-J744||Spain|658[0n]
Euchloe crameri|WMB3209-14|RVcoll.08-J344||Spain|658[0n]
Euchloe crameri|WMB3203-14|RVcoll.08-J204||Spain|658[0n]
Euchloe crameri|EZSPM690-12|Rvcoll. 12-L551||Portugal|658[0n]
Euchloe crameri|EZSPM689-12|Rvcoll. 12-L543||Spain|658[0n]
Euchloe crameri|EZSPM274-09|SMcoll.150308PP31||Spain|658[0n]
Euchloe crameri|EZSPN389-09|RVcoll.08-H442||Spain|658[0n]
Euchloe crameri|EZSPN099-09|RVcoll.06-A038||Spain|658[0n]
Euchloe crameri|EZSPN687-09|RVcoll.08-L015||Spain|658[0n]
Euchloe crameri|EZSPN155-09|RVcoll.06-H840||Spain|658[0n]
Euchloe crameri|EZSPN130-09|RVcoll.06-G471||Spain|658[0n]
Euchloe ausonia|BIBSA1789-16|16-A526|Sicily|Italy|658[0n]
Euchloe ausonia|EULEP1773-15|RVcoll.14-H532||Greece|658[0n]
Euchloe ausonia|BIBSA1582-16|12-M400|Sicily|Italy|658[0n]
Euchloe ausonia|BIBSA027-14|LEP-SS-00027||Italy|658[0n]
Euchloe ausonia|OXB1157-15|Rvcoll_16-A045|Sicily|Italy|658[0n]
Euchloe ausonia|BIBSA028-14|LEP-SS-00028||Italy|632[0n]
Euchloe ausonia|BIBSA1578-16|12-M334|Sicily|Italy|613[0n]
Euchloe ausonia|BIBSA1009-15|15-C118||Italy|658[0n]
Euchloe ausonia|WMB4782-14|RVcoll. 14-I329||Italy|658[0n]
Euchloe ausonia|WMB3878-14|RVcoll.12-M504||Italy|658[0n]
Euchloe ausonia|WMB4132-14|RVcoll.13-S636||Italy|658[0n]
Euchloe ausonia|WMB2444-13|RVcoll.11-D351|Sicily|Italy|658[0n]
Euchloe ausonia|WMB5970-17|RVcoll14V854|Ustica|Italy|658[0n]
Euchloe ausonia|WMB5969-17|RVcoll14V853|Ustica|Italy|658[0n]
Euchloe ausonia|WMB2335-13|RVcoll.12-M270||Italy|658[0n]
Euchloe ausonia|WMB2332-13|RVcoll.12-M264||Italy|658[0n]
Euchloe ausonia|WMB3844-14|RVcoll.10-C617||Italy|658[0n]
Euchloe ausonia|WMB1265-13|RVcoll.12-M269||Italy|658[0n]
Euchloe ausonia|WMB1124-13|RVcoll.11-D255|Sicily|Italy|658[0n]
Euchloe ausonia|WMB2887-14|RVcoll.11-D075|Sicily|Italy|658[0n]
Euchloe ausonia|WMB170-11|RVcoll.11-D409|Sicily|Italy|658[0n]
Euchloe ausonia|WMB156-11|RVcoll.11-D307|Sicily|Italy|658[0n]
Euchloe ausonia|WMB1903-13|RVcoll.11-H518|Capri|Italy|620[0n]
Euchloe ausonia|OXB712-15|13-T903||Italy|658[0n]
Euchloe ausonia|BIBSA979-15|14-W024||Italy|658[0n]
Euchloe ausonia|WMB4845-14|RVcoll. 14-I392||Italy|658[0n]
Euchloe ausonia|WMB4831-14|RVcoll. 14-I378||Italy|658[0n]
Euchloe ausonia|WMB4815-14|RVcoll. 14-I362||Italy|658[0n]
Euchloe ausonia|WMB4807-14|RVcoll. 14-I354||Italy|658[0n]
Euchloe ausonia|WMB4761-14|RVcoll. 14-I308||Italy|658[0n]
Euchloe ausonia|WMB484-11|RVcoll.11-H517|Capri|Italy|658[0n]
Euchloe ausonia|WMB4736-14|RVcoll.LD-3374||Italy|658[0n]
Euchloe ausonia|WMB4665-14|RVcoll.LD-2615|Ischia|Italy|658[0n]
Euchloe ausonia|WMB2800-13|RVcoll.11-Y014|Elba|Italy|658[0n]
Euchloe ausonia|WMB2799-13|RVcoll.11-Y013|Elba|Italy|658[0n]
Euchloe ausonia|WMB018-11|RVcoll.LD-513||Italy|658[0n]
Euchloe ausonia|WMB2861-14|RVcoll.09-X825|Argentario|Italy|658[0n]
Euchloe ausonia|WMB2860-14|RVcoll.09-X824|Argentario|Italy|658[0n]
Euchloe ausonia|EULEP1798-15|RVcoll.14-H680||Greece|658[0n]
Euchloe ausonia|EULEP5135-17|RVcoll16J891||Serbia|658[0n]
Euchloe ausonia|EULEP714-15|RVcoll.12-M251|Crete|Greece|658[0n]
Euchloe ausonia|EULEP658-15|RVcoll.11-J466||Ukraine|658[0n]
Euchloe ausonia|EULEP1653-15|RVcoll.14-G838||Greece|658[0n]
Euchloe ausonia|EULEP2081-15|RVcoll.14-J569||Ukraine|658[0n]
Euchloe ausonia|EULEP1715-15|RVcoll.14-H175||Greece|658[0n]
Euchloe ausonia|EULEP1763-15|RVcoll.14-H432||Greece|658[0n]
Euchloe ausonia|EULEP1717-15|RVcoll.14-H186||Greece|614[0n]
Euchloe ausonia|WMB2715-13|RVcoll.10-C515|Elba|Italy|625[0n]
Euchloe ausonia|WMB2469-13|RVcoll.11-H520|Capri|Italy|618[0n]
Euchloe ausonia|WMB2714-13|RVcoll.10-C514|Elba|Italy|614[0n]
Euchloe ausonia|EZROM180-08|RV-07-D013||Romania|658[0n]
Euchloe ausonia|EZROM981-08|RVcoll.08-M455||Romania|658[0n]
Euchloe ausonia|EZROM979-08|RVcoll.08-M434||Romania|658[0n]
Euchloe ausonia|EZROM980-08|RVcoll.08-M435||Romania|658[0n]
Euchloe ausonia|EZROM978-08|RVcoll.08-M416||Romania|658[0n]
Euchloe ausonia|EZROM977-08|RVcoll.08-M415||Romania|658[0n]
Euchloe ausonia|GWOSU1012-11|BC Back 0062||Greece|658[0n]
Euchloe simplonia|EULEP2845-15|RVcoll.14-N929||Italy|658[0n]
Euchloe simplonia|EULEP4005-16|RVcoll14V914||Italy|658[0n]
Euchloe simplonia|EZSPC883-10|RVcoll.09-T144||Spain|658[0n]
Euchloe simplonia|EZSPM007-09|RVcoll.08-M981||Andorra|658[0n]
Euchloe simplonia|EZSPM008-09|RVcoll.08-M982||Andorra|658[0n]
Euchloe simplonia|EZSPN034-09|RVcoll.09-T075||Spain|658[0n]
Euchloe simplonia|BIBSA220-15|RVcoll.14-E008||Italy|649[0n]

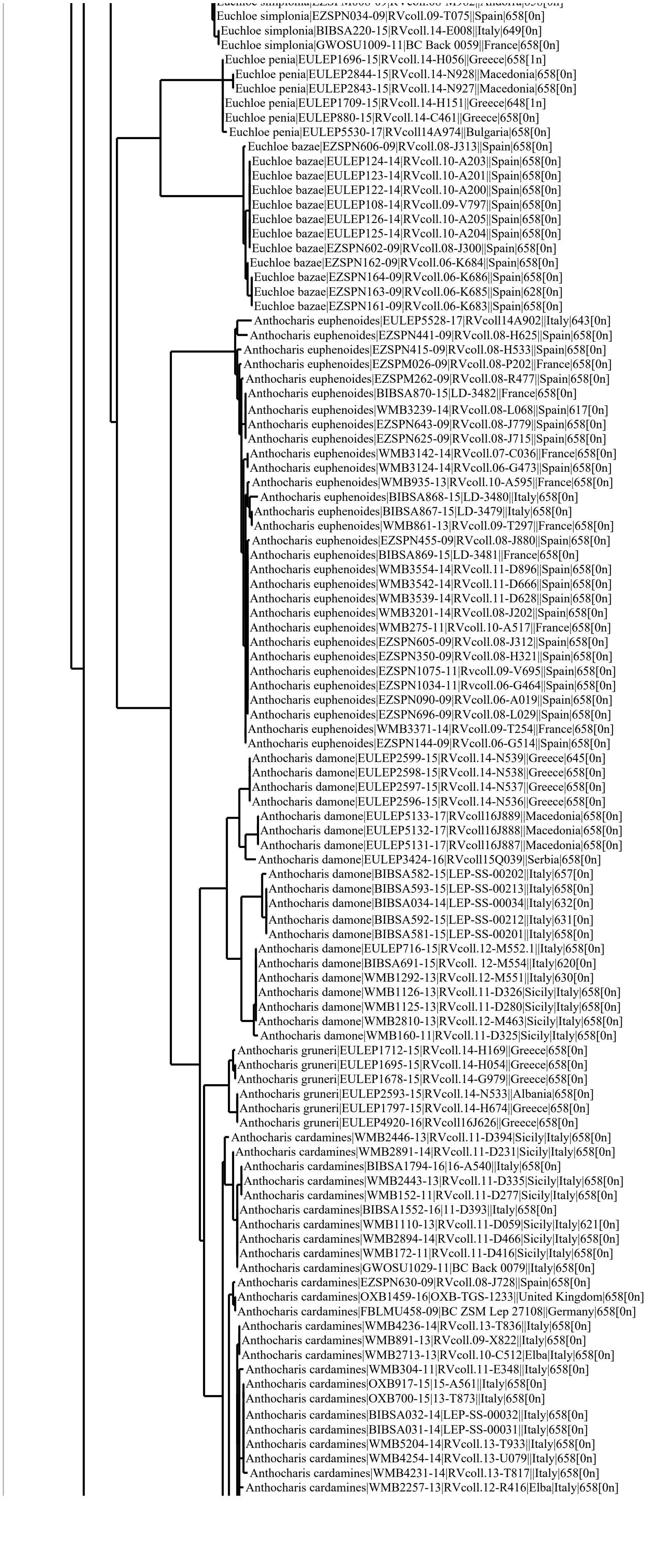
Euchloe simplonia|EZSPN034-09|RVcoll.09-T075||Spain|658[0n]
Euchloe simplonia|BIBSA220-15|RVcoll.14-E008||Italy|649[0n]
Euchloe simplonia|GWOSU1009-11|BC Back 0059||France|658[0n]
Euchloe penia|EULEP1696-15|RVcoll.14-H056||Greece|658[1n]
Euchloe penia|EULEP2844-15|RVcoll.14-N928||Macedonia|658[0n]
Euchloe penia|EULEP2843-15|RVcoll.14-N927||Macedonia|658[0n]
Euchloe penia|EULEP1709-15|RVcoll.14-H151||Greece|648[1n]
Euchloe penia|EULEP880-15|RVcoll.14-C461||Greece|658[0n]
Euchloe penia|EULEP5530-17|RVcoll14A974||Bulgaria|658[0n]
Euchloe bazae|EZSPN606-09|RVcoll.08-J313||Spain|658[0n]
Euchloe bazae|EULEP124-14|RVcoll.10-A203||Spain|658[0n]
Euchloe bazae|EULEP123-14|RVcoll.10-A201||Spain|658[0n]
Euchloe bazae|EULEP122-14|RVcoll.10-A200||Spain|658[0n]
Euchloe bazae|EULEP108-14|RVcoll.09-V797||Spain|658[0n]
Euchloe bazae|EULEP126-14|RVcoll.10-A205||Spain|658[0n]
Euchloe bazae|EULEP125-14|RVcoll.10-A204||Spain|658[0n]
Euchloe bazae|EZSPN602-09|RVcoll.08-J300||Spain|658[0n]
Euchloe bazae|EZSPN162-09|RVcoll.06-K684||Spain|658[0n]
Euchloe bazae|EZSPN164-09|RVcoll.06-K686||Spain|658[0n]
Euchloe bazae|EZSPN163-09|RVcoll.06-K685||Spain|628[0n]
Euchloe bazae|EZSPN161-09|RVcoll.06-K683||Spain|658[0n]
Anthocharis euphenoides|EULEP5528-17|RVcoll14A902||Italy|643[0n]
Anthocharis euphenoides|EZSPN441-09|RVcoll.08-H625||Spain|658[0n]
Anthocharis euphenoides|EZSPN415-09|RVcoll.08-H533||Spain|658[0n]
Anthocharis euphenoides|EZSPM026-09|RVcoll.08-P202||France|658[0n]
Anthocharis euphenoides|EZSPM262-09|RVcoll.08-R477||Spain|658[0n]
Anthocharis euphenoides|BIBSA870-15|LD-3482||France|658[0n]
Anthocharis euphenoides|WMB3239-14|RVcoll.08-L068||Spain|617[0n]
Anthocharis euphenoides|EZSPN643-09|RVcoll.08-J779||Spain|658[0n]
Anthocharis euphenoides|EZSPN625-09|RVcoll.08-J715||Spain|658[0n]
Anthocharis euphenoides|WMB3142-14|RVcoll.07-C036||France|658[0n]
Anthocharis euphenoides|WMB3124-14|RVcoll.06-G473||Spain|658[0n]
Anthocharis euphenoides|WMB935-13|RVcoll.10-A595||France|658[0n]
Anthocharis euphenoides|BIBSA868-15|LD-3480||Italy|658[0n]
Anthocharis euphenoides|BIBSA867-15|LD-3479||Italy|658[0n]
Anthocharis euphenoides|WMB861-13|RVcoll.09-T297||France|658[0n]
Anthocharis euphenoides|EZSPN455-09|RVcoll.08-J880||Spain|658[0n]
Anthocharis euphenoides|BIBSA869-15|LD-3481||France|658[0n]
Anthocharis euphenoides|WMB3554-14|RVcoll.11-D896||Spain|658[0n]
Anthocharis euphenoides|WMB3542-14|RVcoll.11-D666||Spain|658[0n]
Anthocharis euphenoides|WMB3539-14|RVcoll.11-D628||Spain|658[0n]
Anthocharis euphenoides|WMB3201-14|RVcoll.08-J202||Spain|658[0n]
Anthocharis euphenoides|WMB275-11|RVcoll.10-A517||France|658[0n]
Anthocharis euphenoides|EZSPN605-09|RVcoll.08-J312||Spain|658[0n]
Anthocharis euphenoides|EZSPN350-09|RVcoll.08-H321||Spain|658[0n]
Anthocharis euphenoides|EZSPN1075-11|Rvcoll.09-V695||Spain|658[0n]
Anthocharis euphenoides|EZSPN1034-11|Rvcoll.06-G464||Spain|658[0n]
Anthocharis euphenoides|EZSPN090-09|RVcoll.06-A019||Spain|658[0n]
Anthocharis euphenoides|EZSPN696-09|RVcoll.08-L029||Spain|658[0n]
Anthocharis euphenoides|WMB3371-14|RVcoll.09-T254||France|658[0n]
Anthocharis euphenoides|EZSPN144-09|RVcoll.06-G514||Spain|658[0n]
Anthocharis damone|EULEP2599-15|RVcoll.14-N539||Greece|645[0n]
Anthocharis damone|EULEP2598-15|RVcoll.14-N538||Greece|658[0n]
Anthocharis damone|EULEP2597-15|RVcoll.14-N537||Greece|658[0n]
Anthocharis damone|EULEP2596-15|RVcoll.14-N536||Greece|658[0n]
Anthocharis damone|EULEP5133-17|RVcoll16J889||Macedonia|658[0n]
Anthocharis damone|EULEP5132-17|RVcoll16J888||Macedonia|658[0n]
Anthocharis damone|EULEP5131-17|RVcoll16J887||Macedonia|658[0n]
Anthocharis damone|EULEP3424-16|RVcoll15Q039||Serbia|658[0n]
Anthocharis damone|BIBSA582-15|LEP-SS-00202||Italy|657[0n]
Anthocharis damone|BIBSA593-15|LEP-SS-00213||Italy|658[0n]
Anthocharis damone|BIBSA034-14|LEP-SS-00034||Italy|632[0n]
Anthocharis damone|BIBSA592-15|LEP-SS-00212||Italy|631[0n]
Anthocharis damone|BIBSA581-15|LEP-SS-00201||Italy|658[0n]
Anthocharis damone|EULEP716-15|RVcoll.12-M552.1||Italy|658[0n]
Anthocharis damone|BIBSA691-15|RVcoll. 12-M554||Italy|620[0n]
Anthocharis damone|WMB1292-13|RVcoll.12-M551||Italy|630[0n]
Anthocharis damone|WMB1126-13|RVcoll.11-D326|Sicily|Italy|658[0n]
Anthocharis damone|WMB1125-13|RVcoll.11-D280|Sicily|Italy|658[0n]
Anthocharis damone|WMB2810-13|RVcoll.12-M463|Sicily|Italy|658[0n]
Anthocharis damone|WMB160-11|RVcoll.11-D325|Sicily|Italy|658[0n]
Anthocharis gruneri|EULEP1712-15|RVcoll.14-H169||Greece|658[0n]
Anthocharis gruneri|EULEP1695-15|RVcoll.14-H054||Greece|658[0n]
Anthocharis gruneri|EULEP1678-15|RVcoll.14-G979||Greece|658[0n]
Anthocharis gruneri|EULEP2593-15|RVcoll.14-N533||Albania|658[0n]
Anthocharis gruneri|EULEP1797-15|RVcoll.14-H674||Greece|658[0n]
Anthocharis gruneri|EULEP4920-16|RVcoll16J626||Greece|658[0n]
Anthocharis cardamines|WMB2446-13|RVcoll.11-D394|Sicily|Italy|658[0n]
Anthocharis cardamines|WMB2891-14|RVcoll.11-D231|Sicily|Italy|658[0n]
Anthocharis cardamines|BIBSA1794-16|16-A540||Italy|658[0n]
Anthocharis cardamines|WMB2443-13|RVcoll.11-D335|Sicily|Italy|658[0n]
Anthocharis cardamines|WMB152-11|RVcoll.11-D277|Sicily|Italy|658[0n]
Anthocharis cardamines|BIBSA1552-16|11-D393||Italy|658[0n]
Anthocharis cardamines|WMB1110-13|RVcoll.11-D059|Sicily|Italy|621[0n]
Anthocharis cardamines|WMB2894-14|RVcoll.11-D466|Sicily|Italy|658[0n]
Anthocharis cardamines|WMB172-11|RVcoll.11-D416|Sicily|Italy|658[0n]
Anthocharis cardamines|GWOSU1029-11|BC Back 0079||Italy|658[0n]
Anthocharis cardamines|EZSPN630-09|RVcoll.08-J728||Spain|658[0n]
Anthocharis cardamines|OXB1459-16|OXB-TGS-1233||United Kingdom|658[0n]
Anthocharis cardamines|FBLMU458-09|BC ZSM Lep 27108||Germany|658[0n]
Anthocharis cardamines|WMB4236-14|RVcoll.13-T836||Italy|658[0n]
Anthocharis cardamines|WMB891-13|RVcoll.09-X822||Italy|658[0n]
Anthocharis cardamines|WMB2713-13|RVcoll.10-C512|Elba|Italy|658[0n]
Anthocharis cardamines|WMB304-11|RVcoll.11-E348||Italy|658[0n]
Anthocharis cardamines|OXB917-15|15-A561||Italy|658[0n]
Anthocharis cardamines|OXB700-15|13-T873||Italy|658[0n]
Anthocharis cardamines|BIBSA032-14|LEP-SS-00032||Italy|658[0n]
Anthocharis cardamines|BIBSA031-14|LEP-SS-00031||Italy|658[0n]
Anthocharis cardamines|WMB5204-14|RVcoll.13-T933||Italy|658[0n]
Anthocharis cardamines|WMB4254-14|RVcoll.13-U079||Italy|658[0n]
Anthocharis cardamines|WMB4231-14|RVcoll.13-T817||Italy|658[0n]
Anthocharis cardamines|WMB2257-13|RVcoll.12-R416|Elba|Italy|658[0n]

Anthocharis cardamines|WMB4254-14|RVcoll.13-U079||Italy|658[0n]
Anthocharis cardamines|WMB4231-14|RVcoll.13-T817||Italy|658[0n]
Anthocharis cardamines|WMB2257-13|RVcoll.12-R416|Elba|Italy|658[0n]
Anthocharis cardamines|WMB4490-14|RVcoll.14-E165||Italy|658[0n]
Anthocharis cardamines|WMB1172-13|RVcoll.11-E775|Corsica|France|658[0n]
Anthocharis cardamines|WMB1175-13|RVcoll.11-E836|Corsica|France|657[0n]
Anthocharis cardamines|WMB931-13|RVcoll.10-A518||France|658[0n]
Anthocharis cardamines|WMB936-13|RVcoll.10-A599||France|658[0n]
Anthocharis cardamines|WMB2211-13|RVcoll.11-Y022|Elba|Italy|658[0n]
Anthocharis cardamines|WMB2451-13|RVcoll.11-E478|Sardinia|Italy|658[0n]
Anthocharis cardamines|WMB2610-13|RVcoll.11-E839|Corsica|France|658[0n]
Anthocharis cardamines|WMB4713-14|RVcoll.LD-2943||Italy|658[0n]
Anthocharis cardamines|WMB314-11|RVcoll.11-E495||Italy|658[0n]
Anthocharis cardamines|WMB647-11|RVcoll.11-I189||Italy|658[0n]
Anthocharis cardamines|WMB4786-14|RVcoll. 14-I333||Italy|658[0n]
Anthocharis cardamines|WMB4812-14|RVcoll. 14-I359||Italy|658[0n]
Anthocharis cardamines|EULEP2139-15|RVcoll.14-J990||Switzerland|658[0n]
Anthocharis cardamines|BIBSA579-15|LEP-SS-00199||Italy|658[0n]
Anthocharis cardamines|BIBSA705-15|RVcoll. 12-R433||Italy|658[0n]
Anthocharis cardamines|GBLAB313-13|BC ZSM Lep 72614||Germany|658[0n]
Anthocharis cardamines|OXB1133-15|15-A666||Italy|658[0n]
Anthocharis cardamines|OXB1054-15|15-A599||Italy|658[0n]
Anthocharis cardamines|EULEP3421-16|RVcoll15G794||Italy|627[0n]
Anthocharis cardamines|BIBSA1008-15|15-C117||Italy|623[0n]
Anthocharis cardamines|WMB4796-14|RVcoll. 14-I343||Italy|610[0n]
Anthocharis cardamines|BIBSA129-15|RVcoll.14-I144||Italy|610[0n]
Anthocharis cardamines|WMB348-11|RVcoll.11-E754||France|638[0n]
Anthocharis cardamines|WMB1157-13|RVcoll.11-E510|Sardinia|Italy|614[0n]
Anthocharis cardamines|WMB3372-14|RVcoll.09-T267||France|658[0n]
Anthocharis cardamines|WMB2859-14|RVcoll.09-X823|Argentario|Italy|658[0n]
Anthocharis cardamines|WMB4750-14|RVcoll.12-R410.1||Italy|658[0n]
Anthocharis cardamines|EZSPN381-09|RVcoll.08-H428||Spain|658[0n]
Anthocharis cardamines|EZSPN369-09|RVcoll.08-J895||Spain|658[0n]
Anthocharis cardamines|EZSPN346-09|RVcoll.08-H311||Spain|658[0n]
Anthocharis cardamines|WMB4747-14|RVcoll.12-M280.1||Italy|658[0n]
Anthocharis cardamines|WMB189-11|RVcoll.11-D499||Italy|658[0n]
Anthocharis cardamines|EZSPC082-09|RVcoll.08-H229||Spain|658[0n]
Anthocharis cardamines|EZSPC083-09|RVcoll.08-J351||Spain|658[0n]
Anthocharis cardamines|EZSPC081-09|RVcoll.06-H861||Spain|658[0n]
Anthocharis cardamines|OXB360-15|RVcoll.14-N074||Italy|658[0n]
Anthocharis cardamines|OXB926-15|15-A570||Italy|658[0n]
Anthocharis cardamines|WMB4125-14|RVcoll.13-S597||Italy|658[0n]
Anthocharis cardamines|LEATG530-14|TLMF Lep 14317||Italy|658[0n]
Anthocharis cardamines|EZSPN694-09|RVcoll.08-L026||Spain|658[0n]
Anthocharis cardamines|GWOSU1024-11|BC Back 0074||Spain|658[0n]
Anthocharis cardamines|OXB275-15|OXB-TGS-958||United Kingdom|658[0n]
Anthocharis cardamines|LEFIJ491-10|MM17116||Finland|658[0n]
Anthocharis cardamines|LEFIB131-10|MM00500||Finland|658[0n]
Anthocharis cardamines|EZROM756-08|RVcoll.07-D315||Romania|658[0n]
Anthocharis cardamines|EZROM008-08|RV-07-D924||Romania|658[0n]
Anthocharis cardamines|WMB252-11|RVcoll.09-V268||France|658[0n]
Anthocharis cardamines|EULEP3423-16|RVcoll15I655||Austria|658[0n]
Anthocharis cardamines|ABOLD073-16|TLMF Lep 21151||Austria|658[0n]
Anthocharis cardamines|EULEP3422-16|RVcoll15H177||Switzerland|658[0n]
Anthocharis cardamines|GWORA2443-09|BC ZSM Lep 30655||Germany|658[0n]
Anthocharis cardamines|PHLAW009-13|TLMF Lep 09806||Austria|658[0n]
Anthocharis cardamines|EZSPC085-09|RVcoll.08-M900||Spain|658[0n]
Anthocharis cardamines|EZSPC084-09|RVcoll.08-L420||Spain|641[0n]
Anthocharis cardamines|GWORA2444-09|BC ZSM Lep 30656||Germany|658[0n]
Anthocharis cardamines|EZROM757-08|RVcoll.08-M213||Romania|658[0n]
Anthocharis cardamines|EZROM005-08|RV-06-K531||Romania|658[0n]
Anthocharis cardamines|LEATG006-14|TLMF Lep 13793||Austria|658[0n]
Anthocharis cardamines|LEFIB129-10|MM00495||Finland|658[0n]
Anthocharis cardamines|LON868-11|NHMO Lep09066||Norway|658[0n]
Anthocharis cardamines|EZROM004-08|RV-06-K525||Romania|658[0n]
Anthocharis cardamines|EZROM755-08|RVcoll.07-D157||Romania|658[0n]
Anthocharis cardamines|EZROM758-08|RVcoll.08-M355||Romania|658[0n]
Anthocharis cardamines|EZROM006-08|RV-07-D096||Romania|658[0n]
Anthocharis cardamines|EZROM007-08|RV-07-D203||Romania|658[0n]
Anthocharis cardamines|EZSPN1040-11|RVcoll.08-H549||Spain|658[0n]
Anthocharis cardamines|EZSPN492-09|RVcoll.08-H929||Spain|658[0n]
Anthocharis cardamines|EZSPM230-09|RVcoll.08-R425||Spain|658[0n]
Anthocharis cardamines|EZSPM231-09|RVcoll.08-R426||Spain|658[0n]
Anthocharis cardamines|WMB3185-14|RVcoll.08-J004||Spain|658[0n]
Anthocharis cardamines|WMB4025-14|RVcoll.12-Z114||Ireland|658[0n]
Anthocharis cardamines|EULEP4752-16|RVcoll16G888||Sweden|658[0n]
Anthocharis cardamines|EULEP837-15|RVcoll.14-C012||Sweden|658[0n]
Anthocharis cardamines|EULEP1673-15|RVcoll.14-G924||Greece|658[0n]
Anthocharis cardamines|EULEP2418-15|RVcoll.14-V195||Belgium|658[0n]
Anthocharis cardamines|BIBSA1646-16|15-C625||Italy|658[0n]
Anthocharis cardamines|GBLAA1297-15|BC ZSM Lep 87183||Germany|658[0n]
Anthocharis cardamines|LEFIJ4743-16|ZMBSU-02103||Belarus|658[0n]
Anthocharis cardamines|LEASS457-17|TLMF Lep 21915||Austria|658[0n]
Anthocharis cardamines|OXB1457-16|OXB-TGS-1231||United Kingdom|658[0n]
Anthocharis cardamines|OXB1458-16|OXB-TGS-1232||United Kingdom|658[0n]
Anthocharis cardamines|OXB1460-16|OXB-TGS-1234||United Kingdom|658[0n]
Anthocharis cardamines|OXB1461-16|OXB-TGS-1235||United Kingdom|658[0n]
Anthocharis cardamines|OXB1462-16|OXB-TGS-1236||United Kingdom|658[0n]
Anthocharis cardamines|OXB1463-16|OXB-TGS-1237||United Kingdom|658[0n]
Anthocharis cardamines|OXB1464-16|OXB-TGS-1238||United Kingdom|658[0n]
Anthocharis cardamines|OXB276-15|OXB-TGS-959||United Kingdom|658[0n]
Anthocharis cardamines|GWORO780-09|BC ZSM Lep 30472||Germany|639[0n]
Anthocharis cardamines|EULEP4865-16|RVcoll16H878||Poland|627[0n]
Anthocharis cardamines|EULEP4885-16|RVcoll16H997||Denmark|634[0n]
Anthocharis cardamines|EULEP2357-15|RVcoll.14-V048||Ukraine|630[0n]
Anthocharis cardamines|LEATF471-14|TLMF Lep 13783||Austria|623[0n]
Anthocharis cardamines|LEATD019-13|TLMF Lep 12666||Italy|658[0n]
Colotis evagore|EULEP5680-17|RVcoll16J926||Spain|658[0n]
Colotis evagore|CLT197-09|08-R020||Spain|658[0n]
Colotis evagore|CLT199-09|08-R024||Spain|658[0n]
Colotis evagore|CLT198-09|08-R023||Spain|658[0n]
