# Supplementary material for: High resolution DNA barcode library for European butterflies reveals continental patterns of mitochondrial genetic diversity
Source: Commun Biol. 2021 Mar 9;4:315. doi: 10.1038/s42003-021-01834-7 (PMC7943782; doi:10.1038/s42003-021-01834-7)
Supplement: Supplementary file 20 — Reporting Summary [file 42003_2021_1834_MOESM20_ESM.pdf]

nature research

Corresponding author(s): Vlad Dinca

Last updated by author(s): Feb 2, 2021

Reporting Summary

Nature Research wishes to improve the reproducibility of the work that we publish. This form provides structure for consistency and transparency in reporting. For further information on Nature Research policies, see our Editorial Policies and the Editorial Policy Checklist.

Statistics

For all statistical analyses, confirm that the following items are present in the figure legend, table legend, main text, or Methods section.

n/a	Confirmed	
☐	☒	The exact sample size (*n*) for each experimental group/condition, given as a discrete number and unit of measurement
☐	☒	A statement on whether measurements were taken from distinct samples or whether the same sample was measured repeatedly
☐	☒	The statistical test(s) used AND whether they are one- or two-sided<br>*Only common tests should be described solely by name; describe more complex techniques in the Methods section.*
☐	☒	A description of all covariates tested
☒	☐	A description of any assumptions or corrections, such as tests of normality and adjustment for multiple comparisons
☐	☒	A full description of the statistical parameters including central tendency (e.g. means) or other basic estimates (e.g. regression coefficient) AND variation (e.g. standard deviation) or associated estimates of uncertainty (e.g. confidence intervals)
☐	☒	For null hypothesis testing, the test statistic (e.g. *F*, *t*, *r*) with confidence intervals, effect sizes, degrees of freedom and *P* value noted<br>*Give P values as exact values whenever suitable.*
☐	☒	For Bayesian analysis, information on the choice of priors and Markov chain Monte Carlo settings
☒	☐	For hierarchical and complex designs, identification of the appropriate level for tests and full reporting of outcomes
☐	☒	Estimates of effect sizes (e.g. Cohen's *d*, Pearson's *r*), indicating how they were calculated

Our web collection on statistics for biologists contains articles on many of the points above.

Software and code

Policy information about availability of computer code

Data collection	Data have been retrieved from the Barcode of Life Data System v4 (BOLD - http://v4.boldsystems.org/index.php)
Data analysis	Several R packages were used: iNEXT (for assessments of haplotype sampling and genetic diversity); ggplot2 (for Loess regression); mgcv (for Generalized Additive Mixed Models). The software PROTAX was used to assess identification success using DNA barcodes.

For manuscripts utilizing custom algorithms or software that are central to the research but not yet described in published literature, software must be made available to editors and reviewers. We strongly encourage code deposition in a community repository (e.g. GitHub). See the Nature Research guidelines for submitting code & software for further information.

Data

Policy information about availability of data

All manuscripts must include a data availability statement. This statement should provide the following information, where applicable:

- Accession codes, unique identifiers, or web links for publicly available datasets
- A list of figures that have associated raw data
- A description of any restrictions on data availability

All sequences in this study have been submitted to GenBank (accession codes MW498979 - MW503694) and, together with associated information, are publicly available in the dataset DS-EUGENMAP (dx.doi.org/10.5883/DS-EUGENMAP) on BOLD at www.boldsystems.org.

Field-specific reporting

Please select the one below that is the best fit for your research. If you are not sure, read the appropriate sections before making your selection.

☐ Life sciences ☐ Behavioural & social sciences ☒ Ecological, evolutionary & environmental sciences

For a reference copy of the document with all sections, see nature.com/documents/nr-reporting-summary-flat.pdf

Ecological, evolutionary & environmental sciences study design

All studies must disclose on these points even when the disclosure is negative.

Study description	We assembled a comprehensive DNA barcode library for European butterflies (22,306 COI sequences, 459 species). We estimated how much of the total COI haplotype diversity has been captured by our dataset. Based on this DNA barcode library, we inferred continental-scale patterns of genetic diversity. The data were also used to assess the performance of DNA barcodes in identifying specimens and to highlight cases needing further taxonomic research.
Research sample	The dataset consists of 22,306 COI sequences representative of 459 species of European Butterflies. Each sequence is at least 600 base pairs.
Sampling strategy	Sampling design aimed to cover as well as possible the European distribution of each species, in order to obtain a good representation of intraspecific genetic diversity. The average number of specimens per species was 48, which should provide a good overview of genetic diversity.
Data collection	DNA data was obtained through standard DNA extraction, amplification and sequencing procedures. Collateral sample data (e.g. GPS coordinates) were obtained by the authors or provided by the collaborators who collected samples.
Timing and spatial scale	The majority of the samples were obtained between 2006-2017. Sampling covered numerous areas across Europe.
Data exclusions	No data exclusions.
Reproducibility	DNA sequences and collateral data (e.g. specimen photos, locality data) are publicly available in the dataset DS-EUGENMAP on BOLD at www.barcodinglife.org. This allows the verification and reproducibility of the analyses.
Randomization	Randomization was not necessary.
Blinding	The type of data and associated analyses did not require blinding.

Did the study involve field work? ☒ Yes ☐ No

Field work, collection and transport

Field conditions	Field work was done by the authors or collaborators in numerous regions of Europe, in order to increase the taxon and geographic coverage. Most species of European butterflies are on wing between March and October, depending on region.
Location	Field work was done in various locations across Europe.
Access & import/export	Austria, Land Kärnten, 26.05.2015, no. SP3-NS-2513/2015 (006/2015)<br>Austria, Land Salzburg, 28.04.2015, no. 205-05RI/547/224-2015<br>Austria, National Park Hohe Tauern, 30.03.2015, no. 31/02/15<br>Czech Republic, Správa Národního Parku Podyjí, 3.08.2016, no. SZ NPP 0422/2016/3<br>Germany, Landkreis Ludwigsglust-Parchim, 27.07.2016, no. 27.07.2016<br>Germany, Landkreis Mecklenburgische Seenplatte, 26.07.2016, no. 661.18.5.2.0003/16<br>Germany, Regierung von Oberbayern, 27.07.2016, no. 55.1-8646-43-2016<br>Germany, Landkreis Rostock, 27.07.2016, 27.07.2016<br>Germany, Landkreis Vorpommern-Greifswald, 9.08.2016, no. 70.1/EUGENMAP/2016/AvS<br>Germany, Thüringer Landesverwaltungsamt, 15.07.2016, no. 4r 0 30-8646.01 -03THLr 6 014<br>Greece, Ministry of Environment and Energy, 1.06.2017, no. 155909/1157<br>Greece, Hellenic Ministry of Environment and Energy, 30.04.2015, no. 124255/1345<br>Greece, Hellenic Ministry of Environment and Energy, 13.08.2014, no. 112441/1860<br>Greece, Hellenic Ministry of Environment and Energy, 5.05.2014, no. 110097/1299<br>Greece, Hellenic Ministry of Environment and Energy, 31.01.2014, no. 140292/3495<br>Italy, Ministero dell'Ambiente e della Tutela del Territorio e del Mare, 24.06.2015, no. 0012493/PNM<br>Netherlands, Rijksdienst voor Ondernemend Nederland, 2.09.2015, no. FF/75A/2015/040<br>Poland, Minister Srodowiska, 25.07.2016, no. DLP-III.286.65.2016.MGr<br>Slovakia, Ministerstvo Zivotného Prostredia Slovenskej Republiky, 13.07.2016, 5579/2016-2.3<br>Spain, Gobierno de Aragón, 3.04.2014, no. 500201/24/2014/1652<br>Spain, Govern de les Illes Balears, 11.03.2014, no. CAP 14/2014

	Spain, Generalitat Valenciana, 6.11.2012, no. 630/12 (FAU12_052)<br>Spain, Generalitat de Catalunya, 17.02.2016, no. SF/115<br>Spain, Gobierno de Aragón, 23.03.2016, no. 500201/24/2016/808<br>Spain, Generalitat de Catalunya, 5.04.2017, no. SF/634<br>Spain, Generalitat de Catalunya, 5.04.2017, no. SF/628<br>Spain, Junta de Andalucia, 11.04.2017, no. 64oxu727PFIRMALdl6j4NW2t4Ig67t<br>Spain, Junta de Andalucia, 21.04.2017, no. 64oxu988PFIRMAftx/tVYpapvhbAIV<br>Spain, Gobierno del Principado de Asturias, 4.04.2017, no. 2017/007323<br>Spain, Castilla-La Mancha, 14.03.2017, no. DGPFEN/SEN/avp_17_158<br>Spain, Xunta de Galicia, 27.03.2017, no. EB-064/2017<br>Spain, Spain, Govern de les Illes Balears, 16.03.2017, no. CAP 10/2017<br>Spain, Conselh Generau d'Aran, 15.03.2017, no. 35/CS/17<br>Spain, Gobierno de Cantabria, 11.05.2017, 417/2017 SEP<br>Spain, Junta de Andalucia, 6.03.2018, no. 64oxu821PFIRMATE1mOM5rrvd9fWro<br>Spain, Gobierno del Principado de Asturias, 21.05.2018, no. 2018/004269<br>Spain, Castilla-La Mancha, 14.02.2018, no. DGPFEN/SEN/avp_18_098<br>Spain, Junta de Castilla y León, 16.03.2018, no. EP/CYL/238/2018<br>Spain, Generalitat de Catalunya, 5.03.2018, no. SF/0205<br>Spain, Generalitat de Catalunya, 5.03.2018, no. SF/0205<br>Spain, Generalitat de Catalunya, 5.03.2018, no. SF/0208<br>Spain, Junta de Andalucia, 21.03.2018, no. 2018107300000590/IRM/MDCG/mes<br>Spain, Generalitat Valenciana, 5.02.2018, no. 055/2018-VS (FAU 18_005)<br>Spain, Conselh Generau d'Aran, 18.04.2018, no. 27/CS/18<br>Spain, Conselh Generau d'Aran, 18.04.2018, no. 29/CS/18<br>Spain, Conselh Generau d'Aran, 18.04.2018, no. 24/CS/18
Disturbance	We estimate that no disturbance has been caused by field work and collecting. A minimum number of samples/species has been sampled locally, always preferring males and specimens at the end of their flight period.

Reporting for specific materials, systems and methods

We require information from authors about some types of materials, experimental systems and methods used in many studies. Here, indicate whether each material, system or method listed is relevant to your study. If you are not sure if a list item applies to your research, read the appropriate section before selecting a response.

Materials & experimental systems

n/a	Involved in the study	
☒	☐	Antibodies
☒	☐	Eukaryotic cell lines
☒	☐	Palaeontology and archaeology
☐	☒	Animals and other organisms
☒	☐	Human research participants
☒	☐	Clinical data
☒	☐	Dual use research of concern

Methods

n/a	Involved in the study	
☒	☐	ChIP-seq
☒	☐	Flow cytometry
☒	☐	MRI-based neuroimaging

Animals and other organisms

Policy information about studies involving animals; ARRIVE guidelines recommended for reporting animal research

Laboratory animals	This study did not include laboratory animals.
Wild animals	Butterflies collected in the field were killed (by pressing the thorax for a few seconds), identified (preliminary identifications), individually placed in glassine envelopes, labeled with unique sample IDs, and dried at room temperature.
Field-collected samples	Butterflies collected in the field were killed and dried at room temperature. Once brought to the laboratory, wings were removed and placed in glassine envelopes, while the bodies were placed in tubes with 99% ethanol and stored at -20ºC.
Ethics oversight	No ethical approval or guidance was necessary because this research involved dead invertebrates (insects).

Note that full information on the approval of the study protocol must also be provided in the manuscript.
